# Mineralocorticoid receptor antagonists and glucocorticoids differentially affect skeletal muscle inflammation and pathology in muscular dystrophy

**DOI:** 10.1172/jci.insight.159875

**Published:** 2022-10-10

**Authors:** Zachary M. Howard, Chetan K. Gomatam, Charles P. Rabolli, Jeovanna Lowe, Arden B. Piepho, Shyam S. Bansal, Federica Accornero, Jill A. Rafael-Fortney

**Affiliations:** 1 Department of Physiology and Cell Biology and; 2 Dorothy M. Davis Heart and Lung Research Institute, College of Medicine, The Ohio State University, Columbus, Ohio, USA.

**Keywords:** Muscle Biology, Cytokines, Innate immunity, Skeletal muscle

## Abstract

Mineralocorticoid receptor antagonists (MRAs) slow cardiomyopathy in patients with Duchenne muscular dystrophy (DMD) and improve skeletal muscle pathology and function in dystrophic mice. However, glucocorticoids, known antiinflammatory drugs, remain a standard of care for DMD, despite substantial side effects. Exact mechanisms underlying mineralocorticoid receptor (MR) signaling contribution to dystrophy are unknown. Whether MRAs affect inflammation in dystrophic muscles and how they compare with glucocorticoids is unclear. The MRA spironolactone and glucocorticoid prednisolone were each administered for 1 week to dystrophic *mdx* mice during peak skeletal muscle necrosis to compare effects on inflammation. Both drugs reduced cytokine levels in *mdx* quadriceps, but prednisolone elevated diaphragm cytokines. Spironolactone did not alter myeloid populations in *mdx* quadriceps or diaphragms, but prednisolone increased F4/80^hi^ macrophages. Both spironolactone and prednisolone reduced inflammatory gene expression in myeloid cells sorted from *mdx* quadriceps, while prednisolone additionally perturbed cell cycle genes. Spironolactone also repressed myeloid expression of the gene encoding fibronectin, while prednisolone increased its expression. Overall, spironolactone exhibits antiinflammatory properties without altering leukocyte distribution within skeletal muscles, while prednisolone suppresses quadriceps cytokines but increases diaphragm cytokines and pathology. Antiinflammatory properties of MRAs and different limb and respiratory muscle responses to glucocorticoids should be considered when optimizing treatments for patients with DMD.

## Graphical abstract

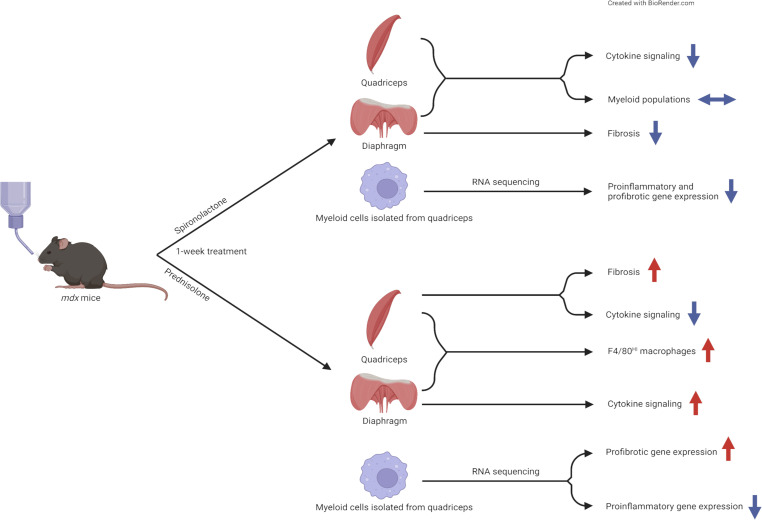

## Introduction

Chronic inflammation exacerbates skeletal muscle pathology in Duchenne muscular dystrophy (DMD) and will likely persist even if emerging genetic therapies are successful. Inflammation is required for efficient wound healing following muscle injury (1). However, continuous cycles of skeletal myofiber necrosis and regeneration resulting from dystrophin protein absence in DMD lead to continual inflammation, dysregulate regeneration, promote fibrosis, and result in loss of ambulation in an individual’s late teenage years (1–4). Glucocorticoids are a standard of care for DMD, delaying loss of ambulation for an average of 3 years, but they have numerous side effects — including osteoporosis, cataracts, and behavioral changes — that are only partially mitigated by intermittent treatment regimens (5–8). Emerging genetic medicines, including gene therapy with microdystrophins, may improve DMD to a less severe Becker muscular dystrophy, but they fail to target muscle stem cells. Therefore, inflammation will likely persist, even if these therapies are successful, requiring antiinflammatory alternatives to glucocorticoids (9).

Glucocorticoids induce antiinflammatory signaling by binding and activating glucocorticoid receptors (GR), which are steroid hormone receptor transcription factors in immune and nonimmune cells (10). Adaptive immune cell functions, specifically subsets of T cells and B cells, are inhibited by glucocorticoids in numerous in vivo and in vitro models of human disease (11–15). Glucocorticoids also affect innate myeloid immune cells, including DCs, monocytes, and macrophages, although many of these studies used in vitro treatments and never in the context of skeletal muscles or DMD (16–18).

Mineralocorticoid receptor antagonists (MRAs) are beneficial for numerous diseases and injuries (19). The endogenous mineralocorticoid, aldosterone, is the primary agonist for mineralocorticoid receptor (MR) and is normally produced by the adrenal glands. Aldosterone is also produced by aldosterone synthase (CYP11B2) in infiltrating myeloid immune cells in dystrophic muscles (20, 21). However, endogenous glucocorticoids (cortisol, corticosterone) have a high affinity for MR and are present at higher serum concentrations than aldosterone (22). Expression of 11 β-hydroxysteroid dehydrogenase type 2 (11β-HSD2), the intracellular enzyme that inactivates cortisol to promote aldosterone selectivity for MR, is also found within dystrophic skeletal muscles, indicating a role for MR in dystrophic muscles (20). Although MRAs were originally used in renal and cardiovascular diseases as a diuretic, they also dampen proinflammatory cytokine signaling and enhance M2 macrophage functions that synergize to arrest fibrosis (23, 24). Myeloid cell MR KO in various renal and cardiovascular preclinical disease models recapitulates improvements and antiinflammatory properties observed with MRA usage, indicating a role of myeloid MR signaling in pathological states (25–28).

Clinical trial and preclinical data support MRA use to slow cardiomyopathy progression in patients with DMD and in mice (29–31). MRAs also improve function and pathology of dystrophic mouse limb and respiratory skeletal muscles (30, 32–36). Glucocorticoids dampen this efficacy, since both drug classes compete for binding to the same receptors (34). Treatment of healthy human myotubes in vitro with MRAs or glucocorticoids in parallel results in different but overlapping gene expression profiles that indicate potential antiinflammatory functions of MRAs in muscle tissue (37). Since MRAs are safe and effective for DMD cardiomyopathy, comparing how MRAs and glucocorticoids affect myeloid inflammation is crucial for improving skeletal muscle treatments for DMD and for expanding the possible use of MRAs for other inflammatory diseases.

To determine how MRAs and glucocorticoids differentially affect inflammation in dystrophic tissues, we treated *mdx* mice short-term with the MRA spironolactone or the glucocorticoid prednisolone during peak dystrophic skeletal muscle necrosis and inflammation (38). From the treated mice, we analyzed skeletal muscle cytokine and chemokine levels, quantified myeloid cell populations in the limb and respiratory muscles, sorted myeloid cells from limb muscles for RNA-Seq, and assessed histological indicators of myofiber damage and fibrosis.

## Results

### Spironolactone and prednisolone reduced chemokine and cytokine levels in dystrophic quadriceps, but prednisolone increased chemokine and cytokine levels in dystrophic diaphragms.

**Figure 1:**
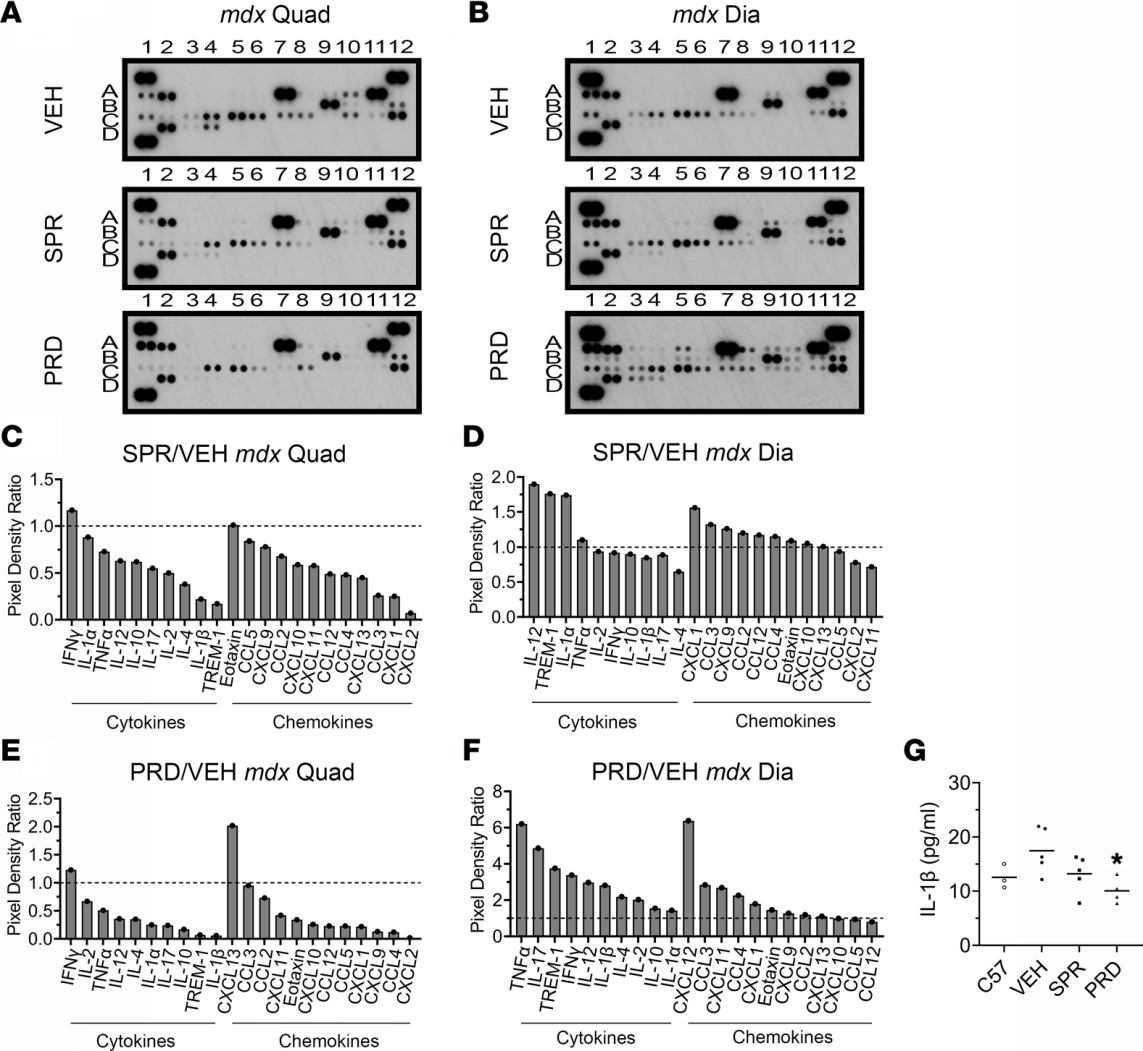
Cytokine and chemokine levels in spironolactone- and prednisolone-treated *mdx* skeletal muscles. (**A**) Proteome profiler cytokine array immunoblots incubated with lysates from spironolactone-treated (SPR-treated) and prednisolone-treated (PRD-treated) 5.5-week-old *mdx* quadriceps compared with vehicle-treated (VEH-treated) controls (5 mg of protein pooled from *n* = 5 SPR, *n* = 4 PRD, *n* = 5 VEH). (**B**) Immunoblots incubated with lysates from SPR- and PRD-treated 5.5-week-old *mdx* diaphragms compared with VEH-treated controls (5 mg of protein). (**C** and **D**) Immunoblot pixel densitometry bar graph displayed as a ratio comparing cytokine and chemokine levels between SPR- and VEH-treated *mdx* quadriceps and *mdx* diaphragms. (**E** and **F**) Immunoblot pixel densitometry bar graph displayed as a ratio comparing cytokine and chemokine levels between PRD- and VEH-treated *mdx* quadriceps and *mdx* diaphragms. A trend-line (dashes) is placed at *y* = 1 on each bar graph to visualize upregulated and downregulated cytokines and chemokines. (**G**) ELISA for IL-1β on soluble protein extracts from quadriceps muscles isolated from *mdx* mice treated for 2 weeks with VEH (*n* = 5), SPR (*n* = 5), or PRD (*n* = 4) compared with untreated WT control (C57) (*n* = 3). Statistics used were ANOVA with Dunnett’s test comparing each group with the VEH. *P ≤ 0.05.

To compare the antiinflammatory effects of GR agonism and MR antagonism on dystrophic muscles, we first performed unbiased assessments of cytokine and chemokine levels in muscles from dystrophin-deficient *mdx* mice after short-term treatment during the peak inflammatory phase (Figure 1). Proteome profiler arrays showed that short-term treatment with prednisolone, the active metabolite of the GR agonist prednisone, decreased numerous chemokines 20% or more; these chemokines included eotaxin (66%), CCL2 (27%), CCL4 (88%), CCL5 (77%), CCL12 (77%), CXCL1 (78%), CXCL2 (98%), CXCL9 (87%), CXCL10 (74%), and CXCL11 (58%) (Figure 1, A and E, and Table 1). Prednisolone also decreased numerous cytokines at least 20%, such as TNF-α (49%), IL-1α (75%), IL-1β (94%), IL-2 (33%), IL-4 (65%), IL-10 (83%), IL-12 (64%), IL-17 (76%), and TREM-1 (93%) (Figure 1, A and E, and Table 2).

Treatment with the MRA spironolactone resulted in reductions of a highly overlapping set of chemokines and cytokines, as observed with GR agonism. Spironolactone reduced, by at least 20% in *mdx* quadriceps, most of the same chemokines as prednisolone, such as CCL2 (32%), CCL4 (52%), CCL12 (51%), CXCL1 (75%), CXCL2 (93%), CXCL9 (22%), and CXCL10 (41%) — and it additionally reduced CCL3 (74%) (Figure 1, A and C, and Table 1). Spironolactone treatment also reduced most of the same cytokines as prednisolone in quadriceps — TNF-α (27%), IL-1β (78%), IL-2 (50%), IL-4 (62%), IL-10 (38%), IL-12 (37%), IL-17 (45%), and TREM-1 (83%) — supporting highly conserved antiinflammatory effects of spironolactone and prednisolone in dystrophic quadriceps muscles (Figure 1, A and C, and Table 2). Both spironolactone and prednisolone increased the cytokine IFN-γ 1.2-fold compared with vehicle in *mdx* quadriceps (Figure 1, A, C, and E, and Table 2). Spironolactone did not increase any quadriceps chemokines, and prednisolone only increased CXCL13 (2.0-fold) compared with vehicle (Figure 1, A, C, and E, and Table 1).

Since quadriceps muscles continue to regenerate during the *mdx* mouse lifespan, we additionally conducted global cytokine and chemokine analyses on the more fibrotic diaphragm respiratory muscle from prednisolone or spironolactone-treated *mdx* mice. In contrast to the reductions observed in quadriceps, prednisolone-treatment substantially elevated *mdx* diaphragm chemokine levels (Figure 1, B and F, and Table 1). Only CCL12 was reduced by prednisolone. Prednisolone increased chemokines in the diaphragm that were reduced in quadriceps, such as eotaxin (1.5-fold), CCL2 (1.2-fold), CCL4 (2.3-fold), CXCL1 (1.8-fold), CXCL2 (6.4-fold), CXCL9 (1.3-fold), and CXCL11 (2.7-fold); it additionally increased CCL3 (2.8-fold) and CXCL13 (1.1-fold). Cytokines that were reduced in quadriceps were also markedly elevated in prednisolone-treated *mdx* diaphragms; these cytokines included TNF-α (6.2-fold), IL-1α (1.4-fold), IL-1β (2.8-fold), IL-2 (2.0-fold), IL-4 (2.2-fold), IL-10 (1.5-fold), IL-12 (3.0-fold), IL-17 (4.9-fold), and TREM-1 (3.8-fold) (Figure 1, B and F, and Table 2). Prednisolone increased IFN-γ (3.4-fold) in the diaphragm, as well as in the quadriceps.

Spironolactone only reduced CXCL2 (22%) and CXCL11 (28%) chemokines and IL-4 (35%) cytokine in *mdx* diaphragms (Figure 1, B and D, and Table 1). Spironolactone elevated most of the same chemokines and cytokines in the diaphragm as prednisolone, although to a much lesser extent. Chemokines slightly increased by spironolactone included eotaxin (1.1-fold), CCL2 (1.2-fold), CCL3 (1.3-fold), CCL4 (1.2-fold), CCL12 (1.2-fold), CXCL1 (1.6-fold), CXCL9 (1.3-fold), and CXCL10 (1.1-fold). Cytokines that increased modestly in spironolactone-treated *mdx* diaphragms were TNF-α (1.1-fold), IL-1α (1.7-fold), IL-12 (1.9-fold), and TREM-1 (1.8-fold).

An ELISA for IL-1β was used to further validate cytokine array results. IL-1β levels in quadriceps soluble protein homogenates were significantly reduced with prednisolone treatment in comparison with vehicle treatment of *mdx* mice (10.1 ± 1.2 pg/mL versus 17.5 ± 1.9 pg/mL; *P* = 0.0418). IL-1β also trended lower with spironolactone treatment (13.2 ± 1.5 pg/mL) to levels similar to WT C57BL/10 (C57) mice (12.6 ± 1.3 pg/mL).

### Prednisolone, but not spironolactone, altered the density and percentage of F4/80^hi^ macrophages in mdx quadriceps.

**Figure 2:**
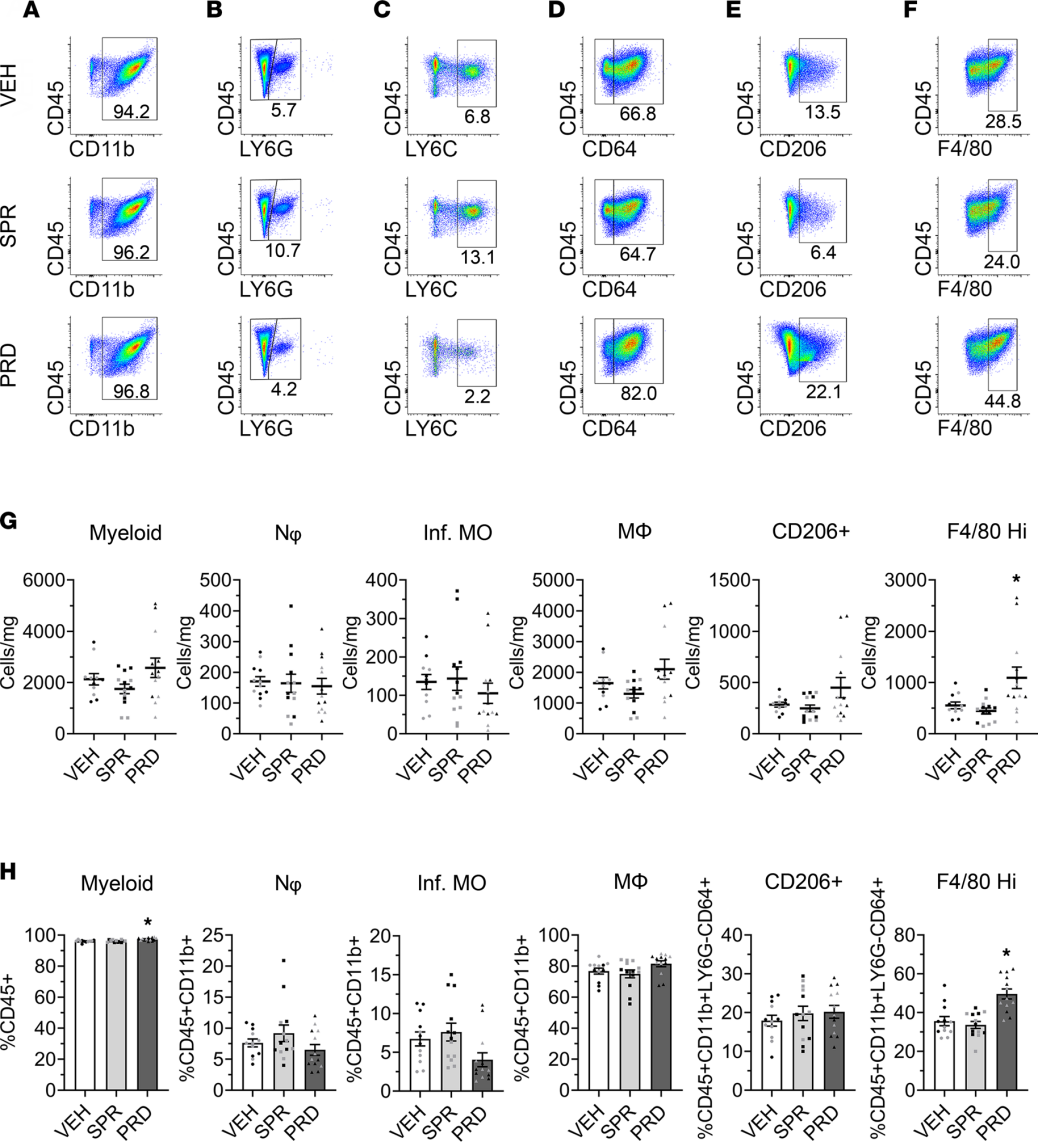
Muscle leukocyte analysis in spironolactone- and prednisolone-treated *mdx* quadriceps. (**A**–**F**) Representative flow cytometry gating dot plots displaying CD45^+^CD11b^+^ myeloid cells (**A**), CD45^+^CD11b^+^LY6G^+^ neutrophils (**B**), CD45^+^CD11b^+^LY6G^–^CD64^–^LY6C^hi^ infiltrating monocytes (**C**), CD45^+^CD11b^+^LY6G^–^CD64^+^ macrophages (**D**), CD45^+^CD11b^+^LY6G^–^CD64^+^CD206^+^ macrophages (**E**), and CD45^+^CD11b^+^LY6G^–^CD64^+^F4/80^hi^ macrophages (**F**) from spironolactone-treated (SPR-treated) (*n* = 13) and prednisolone-treated (PRD-treated) (*n* = 13) 4.5-week-old *mdx* quadriceps compared with vehicle (VEH) (*n* = 12) controls. (**G**) Quantification of myeloid cells, neutrophils (Nφ), infiltrating monocytes (Inf. MO), macrophages (MΦ), CD206^+^ macrophages (CD206^+^), and F4/80^hi^ macrophages represented as dot plots for cells per milligram of muscle (Cells/mg). (**H**) Bar graphs with individual data points as percentages of total CD45^+^ leukocytes (%CD45^+^), CD45^+^ CD11b^+^ myeloid cells (%CD45^+^CD11b^+^) or macrophages (%CD45^+^CD11b^+^LY6G^–^CD64^+^) comparing SPR- and PRD-treated mice with VEH controls. Experimental replicates are denoted by black and gray dots within the graphs. Statistics used were ANOVA with the BKY test *P ≤ 0.05.

We next determined whether the overall reductions in proinflammatory cytokines and chemokines within quadriceps muscles due to short-term prednisolone or spironolactone treatment changed inflammation at the cellular level. Myeloid populations from quadriceps of *mdx* mice following spironolactone or prednisolone treatment from 3.5 to 4.5 weeks of age were quantified using flow cytometry and compared with vehicle-treated *mdx* controls. The skeletal muscle gating strategy is shown in Supplemental Figure 1A (supplemental material available online with this article; https://doi.org/10.1172/jci.insight.159875DS1). Isotype controls are displayed in Supplemental Figure 1B. Representative flow cytometry gating dot plots are displayed for myeloid cell (Figure 2A), neutrophil (Figure 2B), infiltrating monocyte (Figure 2C), macrophage (Figure 2D), CD206^+^ macrophage (Figure 2E), and F4/80^hi^ macrophage (Figure 2F) populations in treated and vehicle control *mdx* quadriceps. The densities of myeloid cells (1,753 ± 185 cells/mg versus 2,131 ± 222 cells/mg; *P* = 0.356), neutrophils (164 ± 30 cells/mg versus 171 ± 16 cells/mg; *P* = 0.859), infiltrating monocytes (144 ± 31 cells/mg versus 135 ± 20 cells/mg; *P* = 0.823), macrophages (1,301 ± 136 cells/mg versus 1,647 ± 187 cells/mg; *P* = 0.313), CD206^+^ macrophages (248 ± 32 cells/mg versus 284 ± 24 cells/mg; *P* = 0.698), and F4/80^hi^ macrophages (445 ± 58 cells/mg versus 554 ± 66 cells/mg; *P* = 0.229) were not different in spironolactone-treated *mdx* quadriceps relative to vehicle controls (Figure 2G). Prednisolone significantly increased the density of F4/80^hi^ macrophages (1,094 ± 210 cells/mg versus 554 ± 66 cells/mg; *P* = 0.027) but did not significantly change the density of myeloid cells (2,578 ± 380 cells/mg versus 2,131 ± 222 cells/mg; *P* = 0.277), neutrophils (155 ± 25 cells/mg versus 171 ± 16 cells/mg; *P* = 0.655), infiltrating monocytes (106 ± 26 cells/mg versus 135 ± 20 cells/mg; *P* = 0.443), macrophages (2,103 ± 324 cells/mg versus 1,647 ± 187 cells/mg; *P* = 0.185), or CD206^+^ macrophages (451 ± 98 cells/mg versus 284 ± 24 cells/mg; *P* = 0.079) compared with vehicle controls.

Spironolactone treatment did not affect the percentage of myeloid cells (96.2% ± 0.2% versus 96.1% ± 0.2% CD45^+^; *P* = 0.870), neutrophils (9.2% ± 1.3% versus 7.6% ± 0.6% CD11b^+^; *P* = 0.281), infiltrating monocytes (7.6% ± 1.1% versus 6.7% ± 0.9% CD11b^+^; *P* = 0.534), macrophages (75.0% ± 2.5 versus 76.8% ± 1.9% CD11b^+^; *P* = 0.559) as a percentage of total myeloid cells, CD206^+^ macrophages (19.8% ± 1.9% versus 17.9% ± 1.4% CD64^+^; *P* = 0.436), or F4/80^hi^ macrophages (33.7% ± 1.8% versus 35.6% ± 2.4% CD64^+^; *P* = 0.553) within *mdx* quadriceps relative to vehicle controls (Figure 2H). Prednisolone significantly and substantially increased the percentage F4/80^hi^ macrophages (49.6% ± 2.6% versus 35.6% ± 2.4% CD64^+^; *P* < 0.001) compared with vehicle controls. Prednisolone also mildly, but significantly, increased the percentage of myeloid cells (97.4% ± 0.3% versus 96.1% ± 0.2% CD45^+^; *P* < 0.001). Prednisolone treatment did not significantly change the percentages of neutrophils (6.5% ± 0.9% versus 7.6% ± 0.6% CD11b^+^; *P* = 0.440), infiltrating monocytes (4.0% ± 0.9% versus 6.7% ± 0.9% CD11b^+^; *P* = 0.067), macrophages (81.6% ± 1.9% versus 76.8% ± 1.9% CD11b^+^; *P* = 0.128), or CD206^+^ macrophages (20.2%± 1.7% versus 17.9% ± 1.4% CD64^+^; *P* = 0.345) in quadriceps muscles relative to vehicle-treated *mdx* controls. All myeloid populations represented as the percentage of total CD45^+^ cells were also calculated (Supplemental Figure 2B). An association between CD206 and F4/80^hi^ macrophage surface markers was found and represented in a t-distributed stochastic neighbor embedding (tSNE) plot; 69.2% ± 1.5% of vehicle-treated *mdx* quadriceps CD206^+^ macrophages were F4/80^hi^ and 38.6% ± 2.8% of F4/80^hi^ macrophages were CD206^+^ (Supplemental Figure 2A).

### Prednisolone, but not spironolactone, altered the percentage of F4/80^hi^ macrophages in mdx diaphragms.

**Figure 3:**
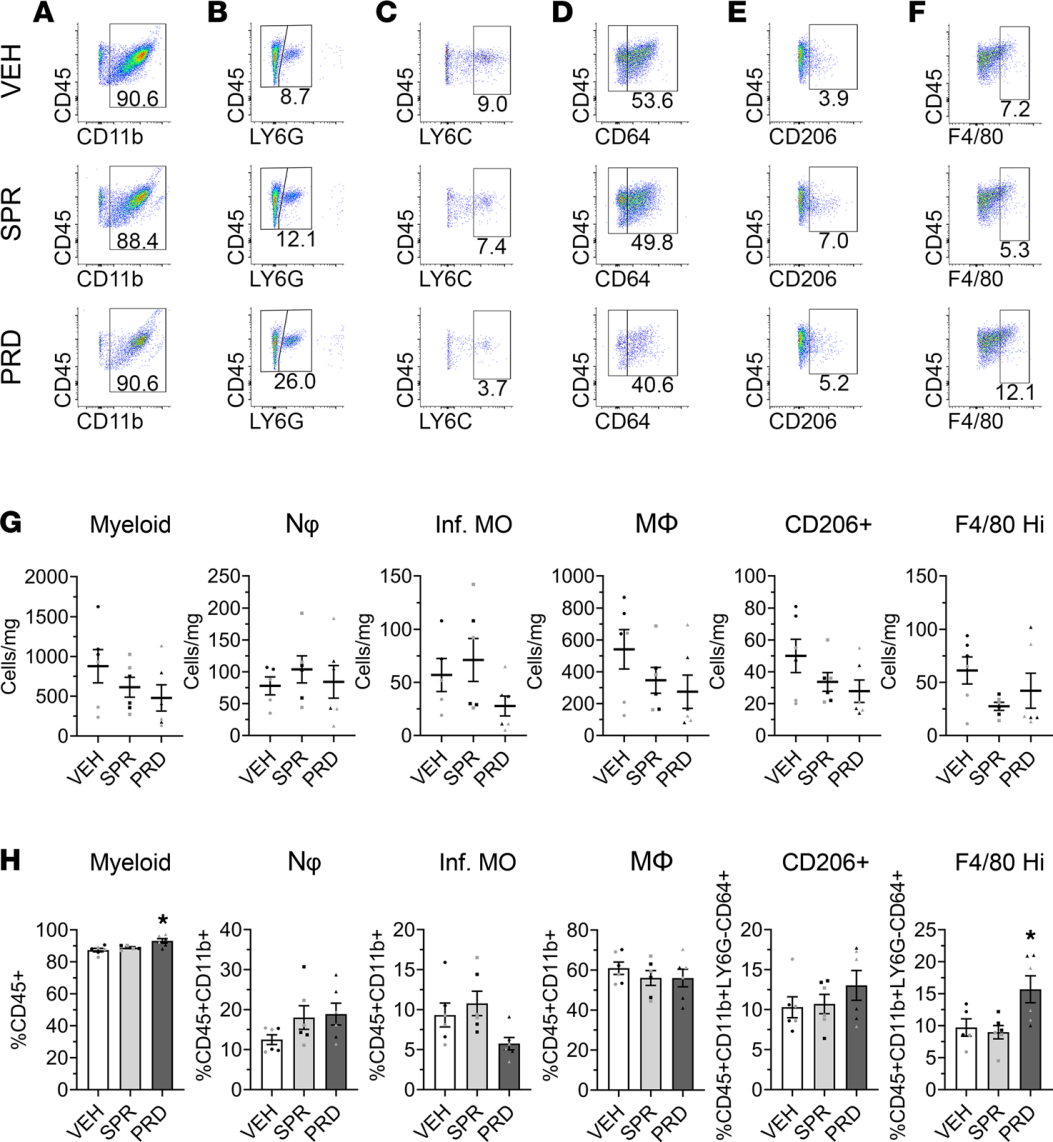
Muscle leukocyte analysis in spironolactone- and prednisolone-treated *mdx* diaphragms. (**A**–**F**) Representative flow cytometry gating dot plots displaying CD45^+^CD11b^+^ myeloid cells (**A**), CD45^+^CD11b^+^LY6G^+^ neutrophils (**B**), CD45^+^CD11b^+^LY6G^–^CD64^–^LY6C^hi^ infiltrating monocytes (**C**), CD45^+^CD11b^+^LY6G^–^CD64^+^ macrophages (**D**), CD45^+^CD11b^+^LY6G^–^CD64^+^CD206^+^ macrophages (**E**), and CD45^+^CD11b^+^LY6G^–^CD64^+^F4/80^hi^ macrophages (**F**) from spironolactone-treated (SPR-treated) and prednisolone-treated (PRD-treated) 4.5-week-old *mdx* diaphragms compared with vehicle (VEH) controls. *n* = 6 replicates per group pooled from 2 mice each were used. (**G**) Quantification of myeloid cells, neutrophils (Nφ), infiltrating monocytes (Inf. MO), macrophages (MΦ), CD206^+^ macrophages (CD206^+^), and F4/80^hi^ macrophages represented as dot plots for cells per milligram of muscle (Cells/mg). (**H**) Bar graphs with individual data points as percentages of total CD45^+^ leukocytes (%CD45^+^), CD45^+^CD11b^+^ myeloid cells (%CD45^+^CD11b^+^), or macrophages (%CD45^+^CD11b^+^LY6G^–^CD64^+^) comparing SPR- and PRD-treated mice with VEH controls. Experimental replicates are denoted by black and gray dots within the graphs. Statistics used were ANOVA with the BKY test *P ≤ 0.05.

We next quantified myeloid cells from diaphragms of spironolactone or prednisolone-treated *mdx* mice as done for quadriceps-derived cells. The representative flow cytometry gating dot plots are shown for myeloid cell (Figure 3A), neutrophil (Figure 3B), infiltrating monocyte (Figure 3C), macrophage (Figure 3D), CD206^+^ macrophage (Figure 3E), and F4/80^hi^ macrophage (Figure 3F) populations in treated and vehicle control *mdx* diaphragms. The density of myeloid cells (614 ± 126 cells/mg versus 879 ± 209 cells/mg; *P* = 0.287), neutrophils (104 ± 21 cells/mg versus 78 ± 14 cells/mg; *P* = 0.421), infiltrating monocytes (71 ± 20 cells/mg versus 57 ± 16 cells/mg; *P* = 0.543), macrophages (347 ± 81 cells/mg versus 542 ± 124 cells/mg; *P* = 0.208), CD206^+^ macrophages (34 cells/mg ± 6 versus 50 ± 10 cells/mg; *P* = 0.172), and F4/80^hi^ macrophages (28 ± 4 cells/mg versus 61 ± 13 cells/mg; *P* = 0.070) were not significantly different in spironolactone-treated *mdx* diaphragms compared with vehicle controls (Figure 3G). Prednisolone treatment did not significantly shift the diaphragm densities of myeloid cells (479 ± 165 cells/mg versus 879 ± 209 cells/mg; *P* = 0.116), neutrophils (84 ± 26 cells/mg versus 78 ± 14 cells/mg; *P* = 0.842), infiltrating monocytes (28 ± 9 cells/mg versus 57 ± 16 cells/mg; *P* = 0.221), macrophages (275 ± 105 cells/mg versus 542 ± 124 cells/mg; *P* = 0.092), CD206^+^ macrophages (28 ± 7 cells/mg versus 50 ± 10 cells/mg; *P* = 0.070), or F4/80^hi^ macrophages (42 ± 17 cells/mg versus 61 ± 13 cells/mg; *P* = 0.382) compared with vehicle-treated *mdx* mice.

Similar to quadriceps, treatment with spironolactone did not significantly change the percentage of diaphragm myeloid cells (88.9% ± 0.6% versus 87.4% ± 1.1% CD45^+^; *P* = 0.369), neutrophils (18.0% ± 3.0% versus 12.5% ± 1.2% CD11b^+^; *P* = 0.128), infiltrating monocytes (10.8% ± 1.5% versus 9.3% ± 1.5% CD11b^+^; *P* = 0.447), macrophages (56.1% ± 3.7% versus 61.0% ± 3.1% CD11b^+^; *P* = 0.374) as a percentage of total myeloid cells, CD206^+^ macrophages (10.7% ± 1.2% versus 10.3% ± 1.3% CD64^+^; *P* = 0.851), or F4/80^hi^ macrophages (9.0% ± 1.0% versus 9.7% ± 1.4% CD64^+^; *P* = 0.679) in *mdx* diaphragms relative to vehicle-treated controls (Figure 3H).

Prednisolone significantly increased percentages of myeloid cells (93.1% ± 1.4% versus 87.4% ± 1.1% CD45^+^; *P* = 0.002) and F4/80^hi^ macrophages (15.7% ± 2.1% versus 9.7% ± 1.4% CD64^+^; *P* = 0.043) in *mdx* diaphragms relative to vehicle controls. Neutrophil (18.9% ± 2.7% versus 12.5% ± 1.2% CD11b^+^; *P* = 0.082), infiltrating monocyte (5.8% ± 0.8% versus 9.3% ± 1.5% CD11b^+^; *P* = 0.073), macrophage (56.0% ± 4.4% versus 61.0% ± 3.1% CD11b^+^; *P* = 0.371), and CD206^+^ macrophage (13.0% ± 1.9% versus 10.3% ± 1.3% CD64^+^; *P* = 0.211) percentages were not significantly affected by prednisolone treatment in *mdx* diaphragms. All diaphragm myeloid populations represented as the percentage of total CD45^+^ cells were also calculated (Supplemental Figure 2C). Similar to the observation in quadriceps, the macrophage association between CD206 and F4/80^hi^ was also found in the diaphragm. Macrophages positive for CD206 were 35.4% ± 1.8% F4/80^hi^ while 47% ± 7.2% of macrophages displaying F4/80^hi^ were CD206^+^ (Supplemental Figure 2A).

### Prednisolone and spironolactone have contrasting effects on molecular signatures of myeloid cells in dystrophic muscles.

**Figure 4:**
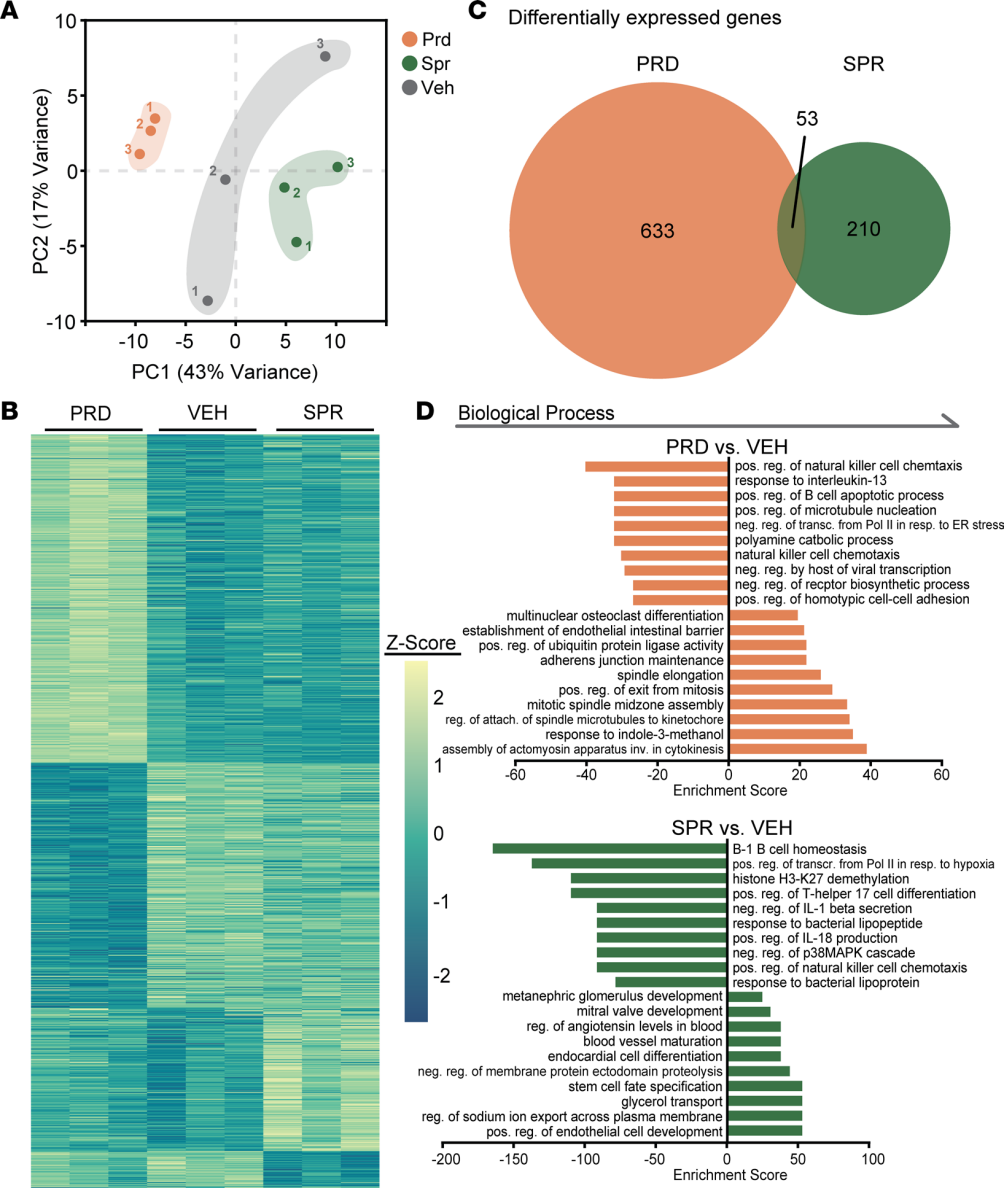
Principal component analysis (PCA), overview of differentially expressed genes, and biological process GO analysis of sequenced RNA from spironolactone- and prednisolone-treated *mdx* quadriceps myeloid cells. (**A**) Principal component analysis revealed 3 distinct groupings based on treatment condition. *n* = 3 replicates pooled from 3 mice each were used for each group. (**B**) Heatmap of all 896 genes that are differentially expressed between either prednisolone treatment versus vehicle, or spironolactone treatment versus vehicle. (**C**) Overlap analysis reveals 53 genes that are differentially expressed in both prednisolone versus vehicle and spironolactone versus vehicle. Prednisolone and spironolactone treatments yield 633 and 210 gene, respectively, that are uniquely differentially expressed in those treatment conditions versus vehicle. (**D**) GO analysis for biological processes.

Since dystrophic quadriceps muscle cytokine levels were reduced similarly by both prednisolone and spironolactone, but myeloid cell populations were changed only by prednisolone, we next determined how each drug affects the molecular signatures of skeletal muscle myeloid cells. RNA was isolated from myeloid cells after FACS from spironolactone-, prednisolone-, or vehicle-treated *mdx* quadriceps muscles using the same 1-week treatment regimen as for the flow cytometry experiments. Myeloid cell gene expression signatures from prednisolone- and spironolactone-treated mice were different from each other and from vehicle-treated *mdx* mice (Figure 4, A–C). Indeed, principal component analysis (PCA) revealed clear separation between vehicle and each of the treatments (Figure 4A). The differential impact that each of the treatments had on gene expression in myeloid cells is further highlighted by global visualization of all differentially regulated transcripts (Figure 4B and Supplemental Tables 1 and 2). In total, 686 and 263 genes were differentially expressed due to prednisolone and spironolactone, respectively, with only 53 genes shared between treatments (Figure 4C and Supplemental Figure 3, A–C). Gene ontology (GO) analysis of *mdx* quadriceps myeloid cell biological processes affected by spironolactone and prednisolone also indicate that the treatment effects differ substantially (Figure 4D). Prednisolone affected numerous signaling pathways involved in mitosis, including assembly of the actomyosin contractile ring and the mitotic spindle. Additionally, prednisolone affected genes associated with the biological categories of cell-to-cell adhesion, NK cell chemotaxis, apoptotic signaling in B cells, and responses to IL-13. Spironolactone predominantly changed pathways associated with IL-18 expression, NK cell chemotaxis, Th17 cell differentiation, histone H3-K27 demethylation, p38 MAPK, transcriptional hypoxic response (primarily via *Vegfa* and *Hif1a*), and B-1 B cell homeostasis (Figure 4D). GO analysis of molecular functions changed from prednisolone or spironolactone treatment, as well (Supplemental Figure 3D).

**Figure 5:**
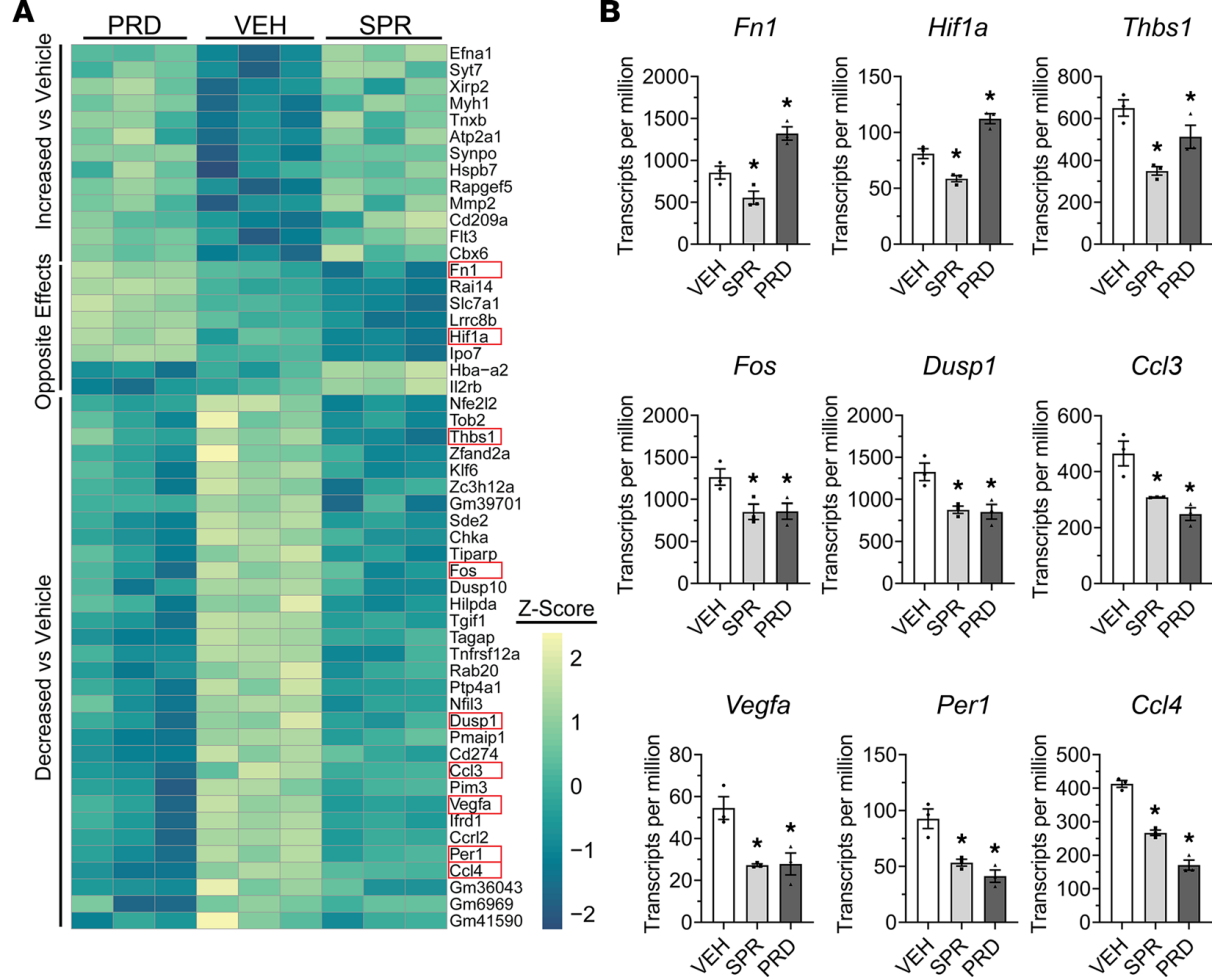
Analysis of overlapped, differentially expressed genes in sequenced RNA from spironolactone- and prednisolone-treated *mdx* quadriceps myeloid cells. (**A**) Heatmap of the 53 genes that are differentially expressed in both prednisolone treatment and spironolactone treatment conditions. Of the commonly differentially expressed genes, 45 genes were regulated in the same direction versus vehicle, while 8 were regulated in the opposite direction versus vehicle. Genes of interest are outlined in red boxes. (**B**) Transcripts per million of differentially expressed genes implicated in DMD pathology: *Fn1*, *Hif1a*, *Thbs1*, *Fos*, *Dusp1*, *Ccl3*, *Vegfa*, *Per1*, and *Ccl4*. Significant differences are from Benjamini-Hochberg adjusted *P* -values following DESeq2 analysis. *P ≤ 0.05.

Despite the clear distinction in gene signature affected by spironolactone and prednisolone on dystrophic myeloid cells, a significant overlap is observed with the 53 genes regulated by both treatments (Figure 4C; Figure 5, A and B; and Supplemental Figure 3C). Spironolactone and prednisolone treatment reduced myeloid cell expression of thrombospondin-1 (THBS1), an adhesive glycoprotein involved in angiogenesis. Additionally, decreases in the expression of *Fos*, a subunit of transcription factor AP-1, were observed with both treatments. Chemokine expression — including expression of *Ccl3 —* was also reduced by spironolactone and prednisolone treatment. Reduction of *Ccl3* expression in both treatment groups validates that at least some of the reduced levels observed in the cytokine/chemokine assays of treated quadriceps muscles are due to transcriptional regulation in myeloid cells, although discrepancies between myeloid cell expression and whole-muscle protein levels are observed. Both treatments also reduced the expression of dual specificity phosphatase 1 (Dusp1), a known regulator of cytokine expression and macrophage activity (39). Dusp1 is known to regulate Vegfa, which is also reduced by both treatments (40). *Mdx* quadriceps myeloid cell expression of *Atp2a1*, or SERCA1, was upregulated in both spironolactone- and prednisolone-treated *mdx* mice, although this is likely a more relevant change if it also occurs in muscle fibers. Additionally, Fms related receptor tyrosine kinase 3 (*Flt3*) expression was upregulated by both treatments. *Mdx* quadriceps myeloid cell expression of *Hif1a* was reduced by spironolactone yet increased by prednisolone treatment. Most relevant for pathology, expression of *Fn1* encoding the extracellular matrix (ECM) component fibronectin was reduced by spironolactone but was increased by prednisolone.

### Spironolactone, but not prednisolone leads to a reduced amount of fibronectin in the diaphragm after 1 week in dystrophic mice.

**Figure 6:**
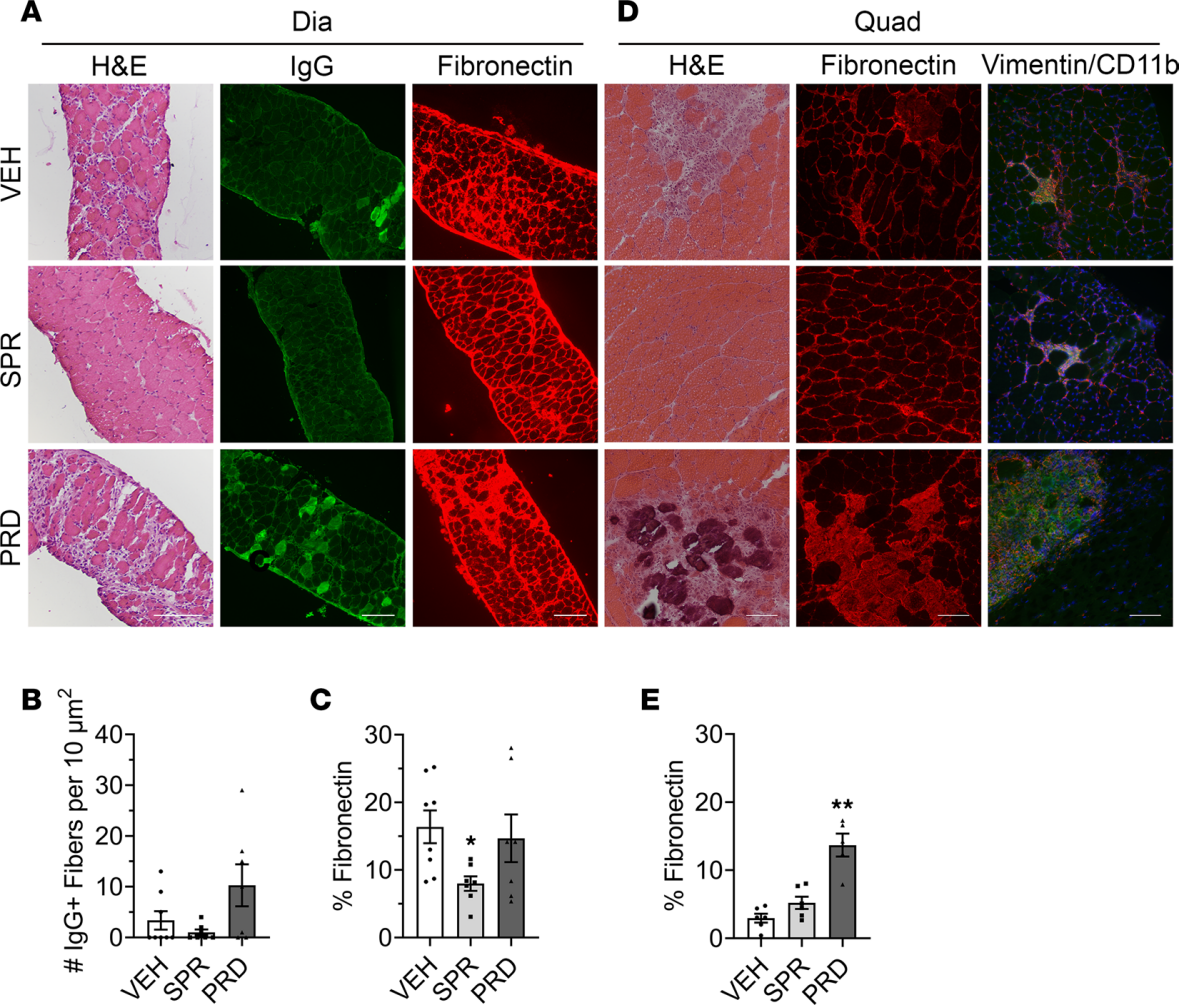
Histology, myofiber degeneration, and fibrosis in spironolactone- and prednisolone-treated *mdx* diaphragms and quadriceps. (**A**) Overall pathology (H&E) and staining for ongoing degenerating myofibers (IgG, green) and fibronectin (red) of diaphragm muscle sections from 4.5-week-old *mdx* mice treated with vehicle (VEH), spironolactone (SPR), or prednisolone (PRD) for 1 week shows active degeneration, inflammation, and fibrosis in dystrophic muscles. (**B**) Quantification of IgG^+^ fibers per 10 μm^2^. (**C**) Percent area of fibronectin staining in diaphragm shows improved dystrophic pathology with SPR but not PRD after 1 week of treatment (*n* = 7 SPR, *n* = 7 PRD, *n* = 8 VEH). (**D**) Staining for overall pathology (H&E), fibrosis (fibronectin, red) and colocalization of fibroblasts (vimentin, red), and myeloid immune cells (CD11b, green) at sites of injury in quadriceps muscle sections from 5.5-week-old *mdx* mice treated for 2 weeks with vehicle, SPR, or PRD. (**E**) Quantification of fibronectin staining in quadriceps muscle sections shows increased fibrosis with PRD treatment (*n* = 6 SPR, *n* = 6 PRD, *n* = 6 VEH). Scale bar: 100 μm. Statistics used were ANOVA with Dunnett’s test comparing each group with the vehicle (VEH). *P ≤ 0.05 and **P ≤ 0.01.

The presence of high levels of *Fn1* gene expression in myeloid cells was a surprise, as was its reduction by spironolactone and its increase by prednisolone treatment. To determine whether these differences in myeloid *Fn1* translated to changes in dystrophic fibrosis after such a short treatment, localization of the *Fn1* gene product, ECM protein fibronectin, was assessed in the dystrophic diaphragm. Since the diaphragm develops more severe fibrosis than quadriceps in dystrophic mice and at a much earlier time point (41) — and RNA-Seq of myeloid cells isolated from the small diaphragm tissue would be technically challenging, requiring pooling large numbers of mice — the effects on diaphragm pathology are critical to investigate. After 1 week of treatment, overall *mdx* diaphragm morphology appeared qualitatively improved by spironolactone treatment but was worsened by prednisolone (Figure 6A). The number of actively degenerating myofibers (IgG^+^) per 10 μm^2^ was not significantly different in *mdx* diaphragms following 1 week of either spironolactone (1.0 ± 0.6 IgG^+^/10 μm^2^ versus 3.4 ± 1.8 IgG^+^/10 μm^2^; *P* = 0.419) or prednisolone (10.3 ± 4.1 IgG^+^/10 μm^2^ versus 3.4 ± 1.8 IgG^+^/10 μm^2^; *P* = 0.289) treatment relative to vehicle controls (Figure 6B). However, spironolactone treatment significantly reduces fibrosis (8.0% ± 1.1% versus 16.4% ± 2.4% fibronectin; *P* = 0.019), whereas prednisolone-treated diaphragms are not different (14.7% ± 3.5% versus 16.4% ± 2.4% fibronectin; *P* = 0.905) from *mdx* vehicle controls (Figure 6C).

Despite the small amount of replacement fibrosis present in *mdx* quadriceps compared with diaphragm, 2 weeks of prednisolone treatment was sufficient to worsen pathology (Figure 6D) and significantly increase fibrosis compared with vehicle controls (13.7% ± 1.7% versus 3.0% ± 0.7% fibronectin; *P* = 0.004) (Figure 6E). Spironolactone-treated quadriceps were not different relative to vehicle controls (5.2% ± 0.9% versus 3.0% ± 0.7% fibronectin; *P* = 0.136). Large areas of injury containing fibrotic replacement of muscle in prednisolone-treated quadriceps contained infiltration of a mixture of fibroblasts and myeloid inflammatory cells (Figure 6D).

## Discussion

In this study, dystrophic (*mdx*) mice were treated with spironolactone or prednisolone during peak skeletal muscle necrosis to delineate the systemic antiinflammatory properties of MRAs and glucocorticoids in dystrophic muscles. Determining the impact of MRAs on dystrophic skeletal muscle inflammation is critical for optimizing therapies for DMD and other muscular dystrophies, as well as for identifying new indications for MRA use. Despite its use as standard-of-care therapy, minimal information exists about how prednisone dampens myeloid inflammation in dystrophic muscles. Since glucocorticoids bind MR and dampen MRA efficacy, comparing how these drugs affect *mdx* limb and respiratory muscle inflammation is necessary for ultimately improving patient outcomes with corticosteroids.

Chemokine and cytokine protein levels were similarly reduced in *mdx* quadriceps muscles by both spironolactone and prednisolone treatment. These data were consistent with an ELISA for IL-1β and with reduced transcription of genes encoding TNF-α, CCL3, and CCL4 in isolated myeloid cells from spironolactone-treated *mdx* mice. However, there was a dramatic increase of almost all diaphragm chemokines and cytokines in prednisolone-treated *mdx* mice. Upregulation of both proinflammatory and antiinflammatory cytokines supports crosstalk to regulate skeletal muscle healing. Prednisolone is likely a more potent immunosuppressant than spironolactone, which may inhibit necessary diaphragm inflammation and paradoxically manifest in the production of more chemokines and cytokines. While inhibiting exogenous inflammation may be beneficial in dystrophic limb skeletal muscle, impeding monocyte recruitment to dystrophic diaphragms could hinder the immune-mediated regenerative response. Splenectomies to reduce muscle infiltrating monocytes in *mdx* mice performed before disease onset reduces myofiber degeneration and inflammation, but when it is performed after disease onset, this procedure increases pathology because monocytes are essential for efficient limb muscle regeneration (42). The *mdx* diaphragm may be even more susceptible to potent immunosuppression because of inherent immunological differences relative to limb muscle (43, 44). However, results from the proteome profiler will need to be validated in the future for each individual cytokine or chemokine to test more specific hypotheses relevant to the function of these inflammatory molecules.

Spironolactone treatment did not significantly change the density or percentages of numerous myeloid populations quantified in *mdx* skeletal muscle; however, treatment reduced fibrosis in *mdx* diaphragms. *Fn1* encoding fibronectin is one of the highest-expressing genes in the CD45^+^CD11b^+^ population that is repressed by spironolactone, supporting that the antifibrotic activity of MR antagonism may be acting directly through macrophages rather than only fibroblasts. In contrast, *Fn1* is surprisingly increased by prednisolone. *Thbs1* encoding the fibronectin-binding protein THBS1 was reduced by both spironolactone and prednisolone. Expression of Thbs1 is positively correlated with disease severity, leading to reduced length of ambulation, in patients with DMD (45). Fibronectin is a soluble, ECM protein elevated in muscular dystrophy, facilitating satellite cell expansion, immune cell invasion and adhesion, and fibroblast proinflammatory activity (46–48). Although fibrocytes are known to express numerous ECM genes, they express low levels of fibronectin (49). Therefore, these data support the observation that myeloid cells may contribute a major source of fibronectin, which is a major component of fibrotic tissue in dystrophic muscles, and that MR antagonism represses this fibrotic pathway. This change in quadriceps muscles suggests that fibronectin activity may represent an early pathogenic step in fibrosis, since these muscles do not become nearly as fibrotic as diaphragm muscles. One week of spironolactone treatment, but not prednisolone treatment, significantly reduced fibronectin localization in *mdx* diaphragms, further supporting the antifibrotic effect of spironolactone on multiple muscle types. Although it was known that daily treatment of dystrophic mice with prednisolone exacerbates, while spironolactone improves, muscle pathology over time, the short-term effects of both treatments had not been previously investigated.

We have previously observed the myofiber membrane–stabilizing effects of spironolactone (50, 51). Numerous pathways involved in membrane stabilization, including basal lamina and ECM components conferring compression resistance and tensile strength, were increased from spironolactone treatment. Basal lamina genes include *Lamb1*, *Col4a1*, and *Col4a2*. Other gene-encoding proteins known to interact in ECM environments include *Dcn*, *Sparc*, *Sparcl1*, *Fbln2*, *Thbs3*, and *Pxdn*. Investigation of whether spironolactone also causes myofibers to upregulate these genes will be needed. It is possible that myeloid cells may have the capacity to reinforce the microenvironment surrounding individual myofibers with ECM in a paracrine manner, while simultaneously inducing fibroblasts to function similarly (52).

Prednisolone treatment increased the percentages of myeloid cells and F4/80^hi^ macrophages in both quadriceps and diaphragms. These results align with the well-documented, potent immunosuppressive effects of leukocyte glucocorticoid signaling. Increases in F4/80^hi^ macrophages via intramuscular injection enhance regeneration following ischemia-reperfusion skeletal muscle injury (53). Diaphragms appear unable to expand the significantly smaller F4/80^hi^ macrophage population to the extent of the quadriceps, and this inability may be detrimental to pathology, since mouse diaphragm–resident macrophages express more stress-response elements while resident macrophages in quadriceps are more M2-like (43). Other groups have observed decreases in *mdx* skeletal muscle F4/80 macrophages by IHC from glucocorticoid treatment, which may be explained by study differences in the route of drug administration, treatment time course, and method of measurement (8). Limitations of interpreting these data are that markers for every immune cell population are not included in the flow cytometry panels and that small amounts of these cells may be included in some of the gates. For example, muscle-infiltrated eosinophils were not analyzed but express F4/80 and possibly CD64. Additionally, circulating monocytes are known to increase cell-surface levels of CD64 when stimulated with inflammatory cytokines (54). Fibrocytes are also CD11b^+^ and LY6C^+^ (49).

Expression of *Fos* (AP-1), *Dusp1*, and *Vegfa* were downregulated by both spironolactone and prednisolone treatment in *mdx* quadriceps myeloid cells. *Dusp1* and *Fos* are 2 of the 4 highest expressing genes in muscle myeloid cells that are repressed by spironolactone. Expression of both genes is known to confer resistance to tyrosine kinase inhibitors in myeloid leukemia, promoting cancerous cell growth (55). Activation of AP-1 signaling is also important for myeloid cell differentiation and survival in the presence of inflammatory cytokines, such as IL-6 (56). In addition, myogenesis is regulated by AP-1 signaling, and pharmacological inhibition of AP-1 prevents muscle wasting in models of cancer cachexia (57, 58). Overall, increasing VEGFA is broadly discussed as a therapeutic option for patients with DMD, with the intention of increasing angiogenesis and satellite cell activity; however, its efficacy is unknown (59, 60). In contrast, another study demonstrated that chronic *Vegfa* expression dysregulates angiogenesis, induces fibrosis, and promotes macrophage accumulation in ischemic rat hindlimb muscles (61).

Other genes significantly changed by spironolactone and prednisolone are less likely to be biologically relevant in myeloid cells, but similar increases in other cell types should be investigated. Expression of *Atp2a1* (SERCA1) was upregulated by both spironolactone and prednisolone in *mdx* quadriceps myeloid cells. Overexpression of SERCA1 in dystrophic mice reduces pathology by improving intracellular Ca^2+^ control, enabling more efficient sarcoplasmic reticulum uptake and, thereby, lowering total cytosolic Ca^2+^ (62, 63). *Hif1a* is another of the 8 genes differentially affected by spironolactone and prednisolone. Chronic hypoxic signaling induces overactivation of fibroblasts in dystrophic skeletal muscle, and inhibitors of hypoxia-inducible factors (HIFs) accelerate recovery following limb injury in mice (64, 65).

Numerous biological processes were affected by prednisolone treatment, including mitosis, cell adhesion, NK cell chemotaxis, and IL-13 immune responses. GR has been found to colocalize with mitotic spindles, and knockdown of GR renders mitosis dysfunctional, interfering with chromosome separation and causing cell death (66). Glucocorticoids are also known to induce induction of apoptosis in healthy and malignant human B cells, suggesting that they may also influence myeloid cell survival (67).

Spironolactone repressed *Dusp1*, *Dusp5*, and *Dusp10*, which encode phosphatases that negatively regulate mitogen-activated protein kinases that are involved in a plethora of inflammatory responses in macrophages, including production of proinflammatory mediators and apoptosis (68). If p38 MAPK signaling activates apoptosis in *mdx* quadriceps myeloid cells, downregulation of the phosphatase DUSP1 may decrease detrimental inflammation. Indeed, muscle-specific KO of *Mapk14*, the gene encoding p38α, in the *mdx* mouse results in amelioration of pathology by preventing B cell leukemia 2–mediated (Bcl-2–mediated) myofiber death (69).

The experiment to identify gene expression changes in dystrophic myeloid cells downstream from glucocorticoid or MRA treatment was specifically designed to assess initial differences before the entire microenvironment had changed due to treatment effects on other cell types and should be interpreted under these limitations. Previous studies of gene expression within entire muscles after 2 weeks or 16 weeks of treatment can be used to compare with these data, to identify acute versus chronic effects and begin to dissect gene expression effects on other cell types (21, 50).

Short-term treatment with spironolactone and prednisolone changed numerous parameters of inflammation and pathology in *mdx* mice. While prednisolone appears to be the more potent immunosuppressant, spironolactone has moderate antiinflammatory properties that may temper excessive inflammation without interfering with efficient regeneration in both limb and respiratory skeletal muscles. Long-term MRA treatment leads to improved diaphragm and limb muscle function and reduced pathology in dystrophic mice (30, 33, 36). However, a complete KO of myeloid MR on an *mdx* background leads to higher cytokine and chemokine levels in quadriceps and increased diaphragm fibrosis, suggesting crosstalk between inflammatory cells and other cell types within the dystrophic skeletal muscle microenvironment (70). Inflammatory cells play a known role in promoting regeneration and repair; therefore, complete abrogation of their injury response can lead to long-term deleterious consequences that may underlie the observed side effects by prednisolone. Moreover, glucocorticoids have recently been demonstrated to induce proadipogenic effects under conditions of upregulated cAMP signaling that occur spatially and temporally in dystrophic skeletal muscle (71, 72).

Histological analysis after short-term treatment was used to validate tissue-level effects due to gene expression changes in fibronectin. Several previous studies demonstrate that *mdx* mice treated daily with prednisolone exhibit more severe cardiac and skeletal muscle pathology (34, 73, 74). However, more recent studies support beneficial effects from weekly dosing in dystrophic mice and patients (8, 75). Another study in which *mdx* mice were treated with prednisolone earlier from 2 to 4 weeks of age identified decreased expression of cellular adhesion molecules, and also showed an improvement in myofiber degeneration (76). Since prednisolone and spironolactone compete for GR and MR binding and reduce the efficacy of each other, and since GR agonism and MR antagonism lead to similar overall antiinflammatory changes, alternating administration of the 2 drugs could be investigated for improved efficacy in future studies.

## Methods

### Mouse treatments.

*Mdx* mice were treated using water bottles containing 250 mg/L spironolactone (Sigma-Aldrich, S3378) or 6.7 mg/L prednisolone (Sigma-Aldrich, P6004), the active metabolite of prednisone dissolved in MediDrop containing sucralose (ClearH2O, 75-01-1001). Mice were treated during the peak of inflammation for 10 days for cytokine- and chemokine-level analysis to allow detection of differences in protein expression (4–5.5 weeks of age), for 7 days (3.5–4.5 weeks of age) for flow cytometry, RNA-Seq, and diaphragm IHC or for 14 days (3.5–5.5 weeks of age) for quadriceps IHC and histology. Approximate dosages for spironolactone and prednisolone treatment were 37.5 and 1 mg/kg × day, respectively (8, 33). The *mdx* mice given MediDrop vehicle were used as controls for all experiments. Mice were housed 5 per cage and euthanized via cervical dislocation.

### Skeletal muscle protein isolation, cytokine proteome arrays, and ELISA.

Isolation of protein from *mdx* skeletal muscle was performed as previously described (44). In brief, 5.5-week-old *mdx* quadriceps and diaphragms dissected from treated and control mice (*n* = vehicle: 2 male [M], 3 female [F]; spironolactone: 2M, 3F; prednisolone: 2M, 2F) for cytokine array analysis and half of each quadriceps from a second cohort of 5.5-week-old *mdx* mice dissected from 2 week-treated or control mice (*n* = vehicle: 4M, 1F; spironolactone: 4M, 1F; prednisolone: 4M; C57: 2M, 1F) for ELISA were flash-frozen in liquid nitrogen and homogenized in manufacturer-recommended lysis buffer containing protease inhibitors. Following protein isolation, sample concentrations were measured using the DC protein assay (Bio-Rad, 5000166). Array membranes from Proteome Profiler Mouse Cytokine Array Kit A (R&D Systems, ARY006) were incubated with 5 mg of protein pooled from all samples from each treatment group and muscle. The assay was completed according to the manufacturer’s instructions in duplicate, and pixel densitometry was performed on the blot films using HL Image++ Quick Spots Tool version 25.0.0r (Western Vision Software).

Soluble protein homogenates isolated from quadriceps from *mdx* mice treated for 2 weeks or from controls were used to perform a standardized ELISA in duplicate for the detection of inflammatory cytokine IL-1β/IL-1F2 according to the manufacturer’s instructions (R&D Systems, MLB00C). ELISA results were quantified by absorbance at 450 nm on a microplate reader (SpectraMax M4, Molecular Devices).

### Generation of single-cell suspensions from skeletal muscles.

Single-cell suspensions were generated from treated 4.5-week-old *mdx* quadriceps and diaphragms for flow cytometric analysis of immune cells as previously described (44). Briefly, quadriceps and diaphragms (*n* = vehicle: 5M, 7F; spironolactone: 5M, 8F; prednisolone: 6M, 7F) were dissected, rinsed in cold DPBS, and finely minced with razor blades. Diaphragms were pooled from 2 mice for each replicate. Pairs of quadriceps from each individual mouse were analyzed independently. Following dissociation, muscles were digested with 10 mL/g of digestion buffer composed of DMEM (Thermo Fisher Scientific, 11995-065), 0.02% Collagenase P (Roche, 11213857001), and 0.1% RQ1 DNase (Promega, M6101) in a 37°C water bath for 30 minutes. After muscle digestion, the resulting suspension was passed through a 70 μm filter then a 40 μm filter. Cells were fixed in 1% paraformaldehyde on ice for 10 minutes. All single-cell suspensions were kept at 4°C following fixation for up to 3 days before staining for flow cytometry. Cells for flow sorting were left unfixed, stained, and sorted immediately.

### Flow cytometric analysis of skeletal muscle immune cells.

Flow cytometry antibodies utilized for staining were as follows: CD45 (phycoerythrin-Cy7; Thermo Fisher Scientific, 25045182), CD11b (allophycocyanin; BioLegend, 101212), LY6G (allophycocyanin/FIRE750; BioLegend, 127652), LY6C (eFluor450; Thermo Fisher Scientific, 48593282), CD64 (BV605; BioLegend, 139323), MHC II (BV650; BioLegend; 107639), CD206 (peridinin-chlorophyll-protein [PerCP] eFluor710; Thermo Fisher Scientific, 46206182), and F4/80 (fluorescein isothiocyanate; BioLegend; 123108). The markers were used to identify leukocytes (CD45^+^), myeloid cells (CD45^+^CD11b^+^), neutrophils (CD45^+^CD11b^+^LY6G^+^), infiltrating monocytes (CD45^+^CD11b^+^LY6G^–^CD64^–^LY6C^hi^), macrophages (CD45^+^CD11b^+^LY6G^–^CD64^+^), CD206^+^ macrophages (CD45^+^CD11b^+^LY6G^–^CD64^+^CD206^+^), and F4/80^hi^ macrophages (CD45^+^CD11b^+^LY6G^–^CD64^+^F4/80^hi^) in the *mdx* quadriceps and diaphragm single-cell suspensions. Corresponding isotype controls were included in all experiments. Cells were permeabilized prior to CD206 staining using DPBS-solubilized 0.5% Tween-20 (Sigma-Aldrich, P1379). Experiments were performed using a Becton Dickinson LSRFortessa Flow Cytometer, and data were analyzed with FlowJo software version 10.7.1 (Becton Dickinson).

### FACS and RNA isolation.

Single-cell suspensions were prepared from quadriceps as described above from 4.5-week-old *mdx* mice treated with spironolactone, prednisolone, or vehicle for 7 days (*n* = vehicle: 4M, 5F; spironolactone: 4M, 5F; prednisolone: 3M, 6F). Cells from 3 pairs of *mdx* quadriceps were pooled for each biological replicate. Freshly isolated unfixed cells were extracellularly stained with CD45 (PE-Cy7; Thermo Fisher Scientific, 25045182) and CD11b (APC; BioLegend, 101212) as described above, and the CD45^+^CD11b^+^ myeloid cells were isolated using a Becton Dickinson FACSAria III. UltraComp eBeads (Thermo Fisher Scientific, 01222242) were used as single-color controls for compensation in real time. Directly following FACS, RNA was isolated from the myeloid cells using a NucleoSpin RNA XS kit (Takara, 740902) according to the manufacturer’s instructions. Live versus dead cells were not distinguished.

### RNA-Seq.

RNA-Seq was performed as previously described (77). RNA was shipped for library preparation (DNBseq-G400 sequencing, transcriptome library, BGI Americas) and RNA-Seq (Complete Genomics, BGI Americas). Fastq files were checked for quality using FASTQC (https://www.bioinformatics.babraham.ac.uk/projects/fastqc/). Processed fastq files were aligned using Hisat2 (78) against the University of California, Santa Cruz (UCSC; Santa Cruz, California, USA) mm39 mouse genome (https://hgdownload.soe.ucsc.edu/goldenPath/mm39/bigZips/). Aligned files were sorted using Samtools (79) and were then used as an input to generate count matrices using HTSeq (80). GTF files matched to UCSC mm39 were used as the HTSeq index. HTSeq count matrices were combined and analyzed for differential expression using DESeq2 (81) with a Benjamini-Hochberg adjusted *P* value less than 0.05. All sequencing data are publicly available through NIH Gene Expression Omnibus (accession no. GSE197553). The PCA plot was generated from DESeq2, and heatmaps were generated using the pheatmap package in R. Heatmaps were produced using normalized read counts to calculate *Z* scores. Plotted transcripts are those that are significantly enriched in their respective categories by DESeq2 (adjusted *P* < 0.05). GO analysis was performed on differentially expressed transcripts using the WEB-based GEne SeT AnaLysis Toolkit (WebGestalt) (82). Overrepresentation analysis was performed to analyze transcripts enriched in prednisolone- or spironolactone-treated animals. The background was set to protein coding transcripts. Categories were selected with an FDR < 0.05, with the minimum number of genes per category set to 10 and the maximum number of genes per category set to 2,000.

### Overlapping gene significance.

Significance of the overlapping 53 genes was determined by randomly selecting 686 genes (total number of significantly altered genes in prednisolone treatment) from the total 50,559 genes that were sequenced. This randomly selected pool of 686 genes was then compared with the significantly altered 263 genes found in spironolactone, and the overlap was calculated. This process was simulated 1,000 times to generate a Poisson distribution to fit the simulated data (Supplemental Figure 2, A–C). A goodness-of-fit test was performed to confirm that the simulated data could be sufficiently modeled by the Poisson distribution. A test mean at the hypothesized value of 53 was then compared with the fitted Poisson distribution to test significance. Simulation was performed using R version 4.1.2. Statistical tests were performed in JMP Pro 15.2.0 (SAS Institute).

### Histology and immunofluorescence analysis.

Diaphragms and quadriceps were isolated from 4.5-week-old *mdx* mice (*n* = vehicle: 3M, 5F; spironolactone: 3M, 4F; prednisolone: 3M, 4F) and 5.5-week-old *mdx* mice (*n* = vehicle: 4M, 2F; spironolactone: 4M, 2F; prednisolone: 4M, 2F), respectively; treated with spironolactone, prednisolone, or vehicle as described above; and embedded in optimal cutting compound, frozen on liquid nitrogen-cooled isopentane, and cut into 8 μm sections on a cryostat (Bright Instruments). Quality control of sections was performed by H&E staining for overall histology. Immunofluorescence was performed with 1:100 Alexa Fluor 488–conjugated goat anti–mouse IgG antibody (Invitrogen, A11029) to detect endogenous IgG and with 1:40 rabbit anti–mouse fibronectin primary antibody (Abcam, 23750) and 1:200 Alexa Fluor 555–conjugated goat anti-rabbit secondary antibody (Invitrogen, A-21429). For fibroblast and myeloid immune cell immunofluorescence colocalization, the sections were incubated with 1:50 rat anti–mouse monoclonal CD11b antibody (BD Pharmigen, 550282) and 1:600 rabbit anti–mouse polyclonal vimentin antibody (Abcam, ab45939); they were then incubated with 1:200 Alexa Fluor 488–conjugated chicken anti-rat (Invitrogen, A-21470) and 1:200 Alexa Fluor 555–conjugated goat anti-rabbit (Invitrogen, A-21429) secondary antibodies. Composite images were taken on a Nikon Eclipse 800 microscope under a 10× objective using a Nikon DS-Ri2 camera driven by Nikon Br Elements software. The number of IgG^+^ myofibers per 10 μm^2^ and percent areas of replacement fibrosis identified by fibronectin staining larger than a myofiber were quantified by an individual blinded to treatment using Adobe Photoshop CS6 as previously described (32, 51, 83).

### Statistics.

Data are displayed as dot plots, with all biological replicates for a given experiment (black dots) or compilation of experiments (black and gray dots) displaying mean ± SEM, or as bar graphs showing mean ± SEM with biological replicates. Grubbs’ test was used to exclude outliers from the compiled skeletal muscle flow cytometry data and immunofluorescence data prior to analysis. In total, 11 outliers across all treatments and skeletal muscle gates, including densities and percentages, were removed from flow cytometric analysis. One outlier was removed from only the quadriceps fibronectin immunofluorescence data. The Brown-Forsythe 1-way ANOVA was used to determine if the SDs were significantly different (*F* ≤ 0.05). For reported *P* values from the flow cytometry data, the ordinary 1-way ANOVA was used with the Benjamini, Krieger, and Yekutieli (BKY) test correcting for the FDR if SDs were not different. The Welch’s ANOVA with the BKY test were utilized if SDs were significantly different. For reported *P* values from the immunofluorescence and ELISA data, the ANOVA tests were applied identically as the flow cytometry statistics, but the Dunnett’s test correcting for multiple comparisons was used instead of BKY. Testing for significance was performed in GraphPad Prism software version 9.3.1 (GraphPad Prism Software). *P* ≤ 0.05 was considered significant.

### Study approval.

Protocols were approved by the IACUC of the Ohio State University and abide by the *Guide for the Care and Use of Laboratory Animals* (National Academies Press, 2011).

## Author contributions

ZMH contributed to overall study design, performed all cell and protein isolations and flow cytometry experiments, analyzed all data, drafted the manuscript, and prepared all figures. CKG performed treatment, dissections, staining, and blinded quantification for diaphragm and quadriceps pathology. CPR performed all bioinformatics analysis, wrote the methods portion for the RNA-Seq and analysis, and prepared figures and tables for the RNA-Seq data. JL maintained the *mdx* mouse colony, assisted with dissections, and performed the cytokine array and ELISA. ABP homogenized and quantified samples and assisted with the ELISA. SSB edited the manuscript and contributed to study design, oversight, and analysis of flow cytometry and sorting experiments. FA edited the manuscript and contributed to the study design, oversight, and analysis of RNA-Seq. JARF edited the manuscript and contributed to design and oversight of overall study implementation and analysis.

## Supplementary Material

Supplemental data

## Figures and Tables

**Table 1:**
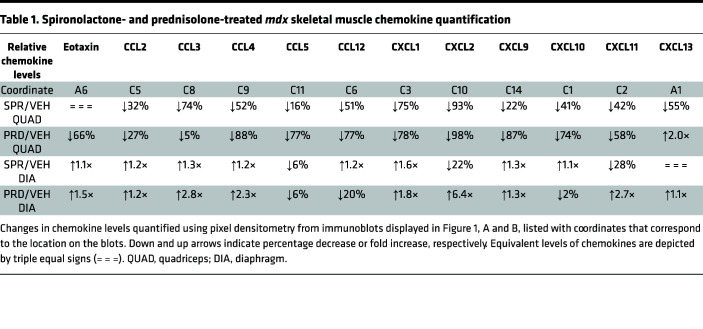
Spironolactone- and prednisolone-treated *mdx* skeletal muscle chemokine quantification

**Table 2:**
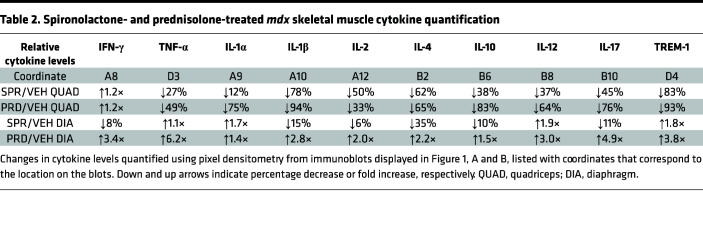
Spironolactone- and prednisolone-treated *mdx* skeletal muscle cytokine quantification

## Version 1 08/30/2022

In-Press Preview

## Version 2 10/10/2022

Electronic publication
