# Supplementary material for: Mineralocorticoid receptor antagonists and glucocorticoids differentially affect skeletal muscle inflammation and pathology in muscular dystrophy
Source: JCI Insight. 2022 Oct 10;7(19):e159875. doi: 10.1172/jci.insight.159875 (PMC9675571; doi:10.1172/jci.insight.159875)
Supplement: Supplemental data [file jciinsight-7-159875-s207.pdf]

Supplemental Fig 1

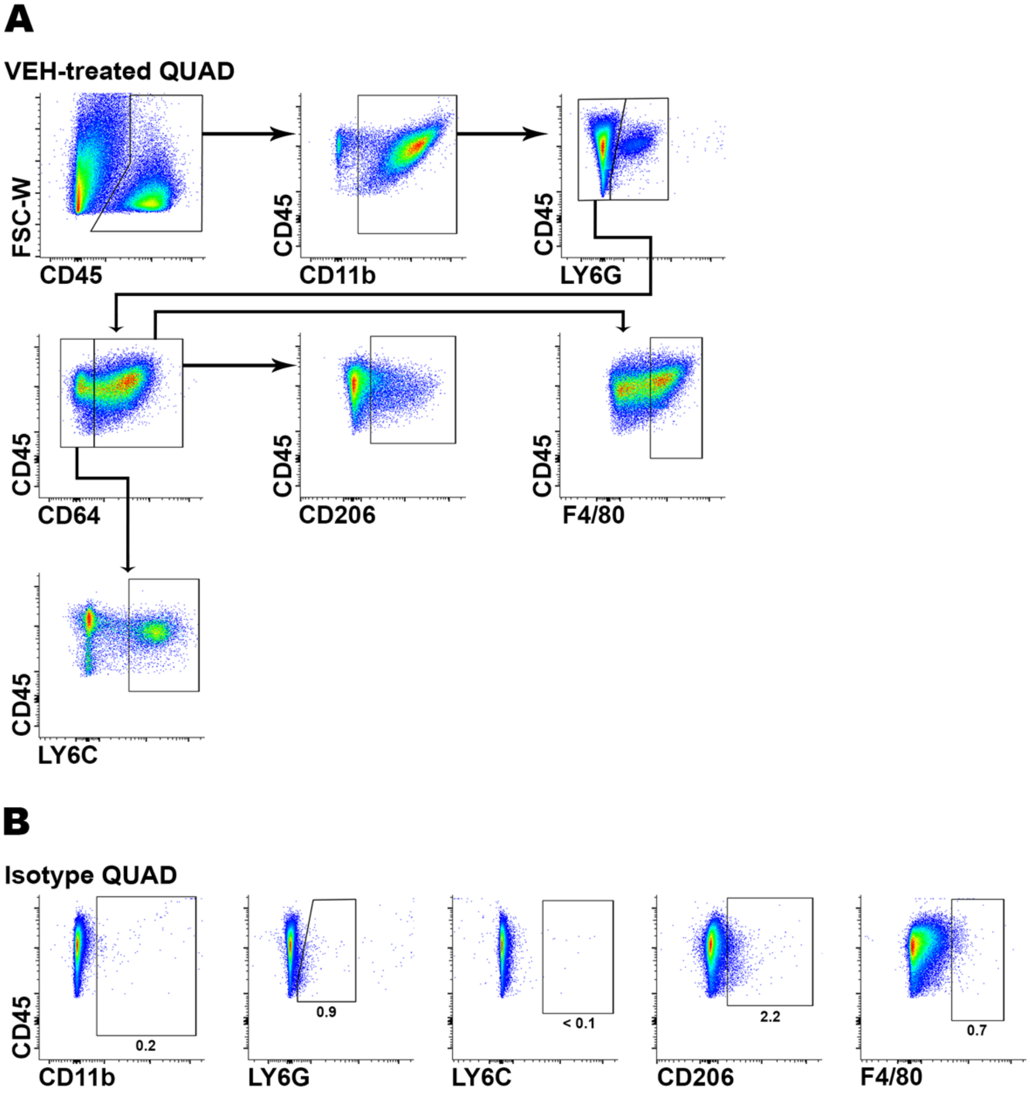

Supplemental Figure 1: Flow cytometry gating strategies for quantifying leukocytes in *mdx* skeletal muscle. (**A**) Representative flow cytometry dot-plots showing the muscle gating strategy, using a quadriceps-derived sample to illustrate. Enabled quantification of leukocytes (CD45^{+}), myeloid cells (CD45^{+} CD11b^{+}), neutrophils (CD45^{+} CD11b^{+} LY6G^{+}), infiltrating monocytes (CD45^{+} CD11b^{+} LY6G^{-} CD64^{-} LY6C^{Hi}), macrophages (CD45^{+} CD11b^{+} LY6G^{-} CD64^{+}), CD206^{+} macrophages (CD45^{+} CD11b^{+} LY6G^{-} CD64^{+} CD206^{+}), and F4/80^{Hi} macrophages (CD45^{+} CD11b^{+} LY6G^{-} CD64^{+} F4/80^{Hi}) in *mdx* quadriceps and diaphragms. (**B**) Isotype controls for CD11b^{+}, LY6G^{+}, LY6C^{+}, CD206^{+}, and F4/80^{Hi} cells using *mdx* quadriceps-derived cells. The anti-mouse CD45 antibody was included in the isotype stain to accurately verify minimal staining specifically on the leukocyte population.

Supplemental Fig 2

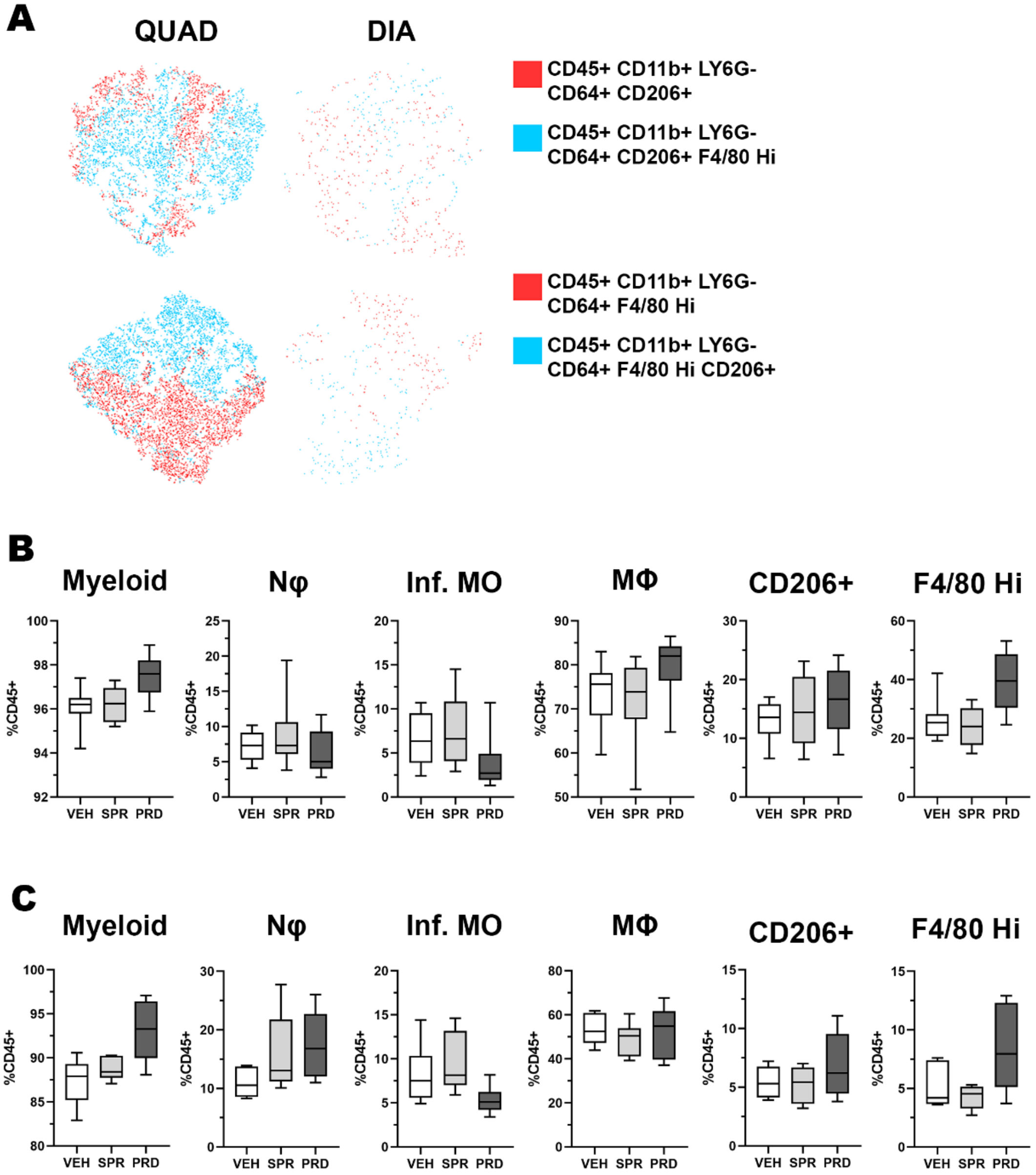

Supplemental Figure 2: tSNE plot of *mdx* muscle CD206$^+$ and F4/80^{Hi} macrophages and *mdx* muscle leukocytes represented as %CD45$^+$. (**A**) Representative tSNE plot visualizing the relationship between *mdx* quadriceps (QUAD) and diaphragm (DIA) macrophages (CD45$^+$ CD11b$^+$ LY6G$^-$ CD64$^+$) displaying CD206 and F4/80^{Hi}. Many of the CD206$^+$ macrophages (top, red) are F4/80^{Hi} (top, blue) in *mdx* quadriceps and diaphragms. Similarly, many of the F4/80^{Hi} macrophages (bottom, red) are CD206$^+$ (bottom, blue) in both *mdx* muscles. (**B**) Box and whisker plots of quantified myeloid populations in *mdx* quadriceps and (**C**) diaphragms comparing spironolactone (SPR)- and prednisolone (PRD)-treated mice to vehicle (VEH) controls as the percentage of total CD45$^+$ leukocytes (%CD45+).

Supplemental Fig 3

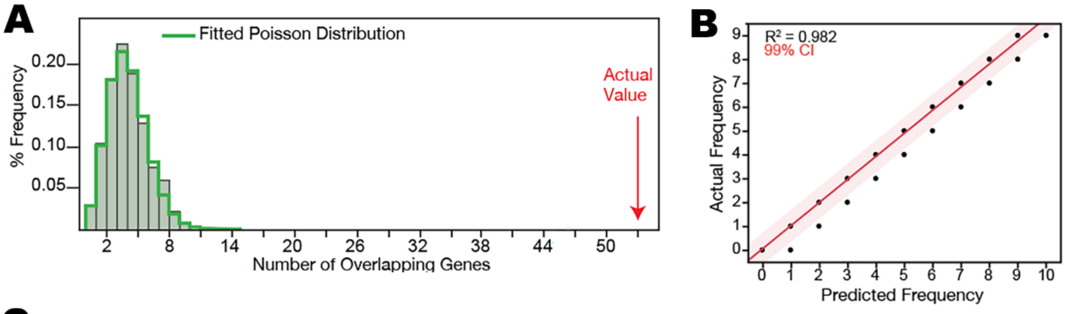

C

Actual Value	53		
95% CI Data		95% CI Poisson	
Mean	3.57	Mean	3.57
lower 95%	3.45	lower 95%	3.45
upper 95%	3.68	upper 95%	3.69
T test statistic	-832	P value	<0.0001

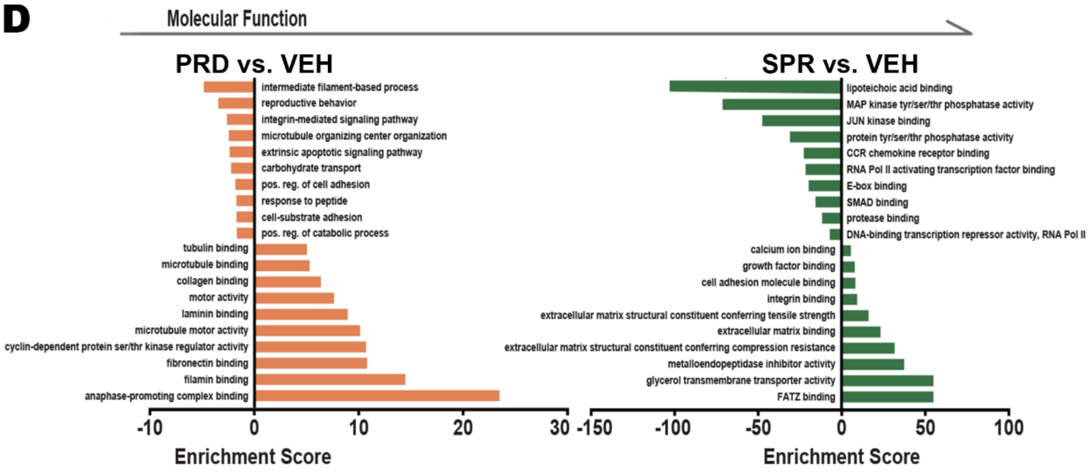

Supplemental Figure 3: Fitted Poisson distribution, quantile-quantile plot, values from simulated overlap analysis, and molecular function gene ontology analysis of myeloid RNA sequencing data. (**A**) Fitted Poisson distribution to a simulated overlap analysis. (**B**) Quantile-Quantile plot of actual vs. predicted values of the simulated overlap analysis. (**C**) Table with numeric values calculated from the simulated overlap analysis. (**D**) Gene ontology analysis for molecular functions.

Supplemental Table 1. Differentially Expressed Genes

RNA Sequencing was performed on Myeloid cells isolated from quadricep muscles of 4.5 week old mdx mice 7 days after treatment with either prednisolone (Prd), Spironolactone (Spr), or Vehicle (Veh).

A. Prednisolone vs Vehicle
B. Spironolactone vs. Vehicle

Differentially expressed genes are those with an adjusted p value < 0.05.
lfcSE is the standard error of the log2FoldChange
stat is the Wald statistic, which is the log2FoldChange divided by the lfcSE

A.

Differentially expressed genes, Prednisolone vs Vehicle. Adjusted p value < 0.05

	baseMean	log2FoldChange	lfcSE	stat	pvalue	padj
Pcsk5	30.2243893	2.522147659	0.463076	5.44650913	5.14E-08	9.78E-06
Dpep1	56.956362	2.27792588	0.550412	4.13858698	3.49E-05	0.00203548
Serpina3n	146.027184	2.236230432	0.282311	7.92115558	2.35E-15	2.47E-12
Mt3	310.840924	2.183313779	0.263272	8.29298944	1.10E-16	1.51E-13
Tg	56.1926818	2.052080643	0.4294	4.77894659	1.76E-06	0.00018335
Prg4	659.351543	1.864575417	0.183738	10.1480187	3.38E-24	2.30E-20
Chst3	79.0953107	1.719591434	0.342993	5.01348296	5.35E-07	7.14E-05
Slc9b2	134.652956	1.716095602	0.32827	5.22770278	1.72E-07	2.75E-05
Sfrp5	47.7426992	1.598657481	0.404839	3.94887324	7.85E-05	0.00377915
Zfp827	29.3236221	1.581719492	0.47655	3.31910768	0.00090306	0.02399348
Ms4a4d	31.7731563	1.56127974	0.500067	3.12214368	0.00179539	0.03934277
Cspg4	1169.8636	1.561194573	0.165672	9.42341451	4.37E-21	1.19E-17
Efna1	34.4576976	1.524946873	0.456578	3.33995098	0.00083793	0.02266073
Ndrg4	41.6002346	1.496911682	0.474199	3.15671298	0.00159558	0.03630684
Syt7	37.9419408	1.479317969	0.456723	3.23898186	0.00119957	0.02983607
Ctsk	2341.17437	1.473435641	0.185457	7.94488306	1.94E-15	2.21E-12
Slc6a12	73.3197886	1.46029939	0.473455	3.08434721	0.00203999	0.04337771
Spink2	44.7965237	1.449186654	0.419236	3.45673007	0.00054677	0.01663506
Lyve1	567.542819	1.437447443	0.390087	3.68493961	0.00022876	0.00898025
Tmem178	74.2399691	1.390279689	0.399588	3.47928176	0.00050276	0.01564524
Zbtb32	99.6425123	1.34734807	0.288997	4.66214888	3.13E-06	0.00029015
Htr7	105.113961	1.332837091	0.286984	4.64428524	3.41E-06	0.00030754
Mybl1	61.8389025	1.318103943	0.361343	3.64779192	0.0002645	0.00977015
Gm36774	64.1555956	1.31568215	0.435053	3.02418636	0.00249303	0.04953349
Serpinb9b	90.8154527	1.308093163	0.3217	4.06618723	4.78E-05	0.00262644
Fah	38.8834348	1.287368372	0.419959	3.0654588	0.00217336	0.04555286
Dusp9	56.4079073	1.28493118	0.355471	3.61472373	0.00030067	0.01053498
Mamdc2	943.34362	1.271393998	0.162994	7.80024061	6.18E-15	6.02E-12
Slc4a11	119.572102	1.250061918	0.267464	4.67376188	2.96E-06	0.00027992
Xirp2	270.473409	1.244090688	0.248575	5.00488232	5.59E-07	7.40E-05
Fancd2	114.181632	1.21410647	0.340264	3.56813477	0.00035953	0.01212974
Cyp2s1	103.186363	1.193690002	0.267461	4.46304215	8.08E-06	0.00062807
4933404O12	83.4417727	1.183263518	0.325253	3.63797914	0.00027479	0.00998754
Fam20a	98.3372653	1.17498043	0.269583	4.3585055	1.31E-05	0.0009345
Angptl2	1307.20461	1.138765665	0.169325	6.72532624	1.75E-11	8.53E-09
E230016K23I	161.608527	1.120971703	0.259938	4.31246136	1.61E-05	0.00107869
Stac2	739.765974	1.104579991	0.184442	5.98876284	2.11E-09	5.76E-07
Ssc5d	309.002037	1.099449918	0.17907	6.13978507	8.26E-10	2.50E-07
Kif14	182.303043	1.090536537	0.244391	4.46225661	8.11E-06	0.00062807
Myh1	170.869684	1.074139924	0.230286	4.66437013	3.10E-06	0.000289
Mastl	141.916543	1.064229662	0.287159	3.70606878	0.0002105	0.00846351
Myo1d	718.272802	1.051950852	0.124528	8.44753077	2.98E-17	5.07E-14
Gprc5b	391.690509	1.050213442	0.211019	4.97687008	6.46E-07	8.21E-05
Eme1	127.629655	1.042565757	0.249887	4.17214471	3.02E-05	0.00181982
Cacna1s	94.2698943	1.03581272	0.337278	3.07109514	0.00213275	0.04494891
Kif20a	172.090509	1.029873533	0.253074	4.06945388	4.71E-05	0.00261095
Col18a1	780.242745	1.028785703	0.181319	5.67389259	1.40E-08	3.06E-06
Gen1	158.816123	1.01933817	0.219814	4.6372753	3.53E-06	0.00031246
Sel1l3	685.162702	1.013900563	0.153096	6.62265398	3.53E-11	1.50E-08
Anln	816.166109	1.00791453	0.176657	5.70550088	1.16E-08	2.68E-06
Fbln1	64.951489	1.007321059	0.296632	3.39585689	0.00068414	0.01971426
Gm13669	63.4100813	1.007018474	0.325003	3.09848964	0.0019451	0.04181653
C77080	413.565001	1.000159029	0.201014	4.97558058	6.51E-07	8.21E-05
Myo18b	205.597546	1.000053953	0.272315	3.67241235	0.00024027	0.00922507
Adamtsl5	393.940856	0.997614695	0.193625	5.15230849	2.57E-07	3.90E-05
Ust	155.89375	0.985113186	0.269659	3.65318304	0.00025901	0.00975394
Npy	600.408288	0.985009323	0.214287	4.59667615	4.29E-06	0.000368
Ccnb1	474.895282	0.98418349	0.185625	5.3020064	1.15E-07	1.95E-05
Klrb1b	579.577151	0.976815107	0.206111	4.73926458	2.14E-06	0.00021656
D17H6S56E-!	3063.89005	0.974061533	0.134103	7.26352468	3.77E-13	2.71E-10
Mapk8ip1	97.7676913	0.973917272	0.265845	3.66348056	0.00024881	0.00949943
Ceacam19	259.479707	0.960386447	0.191057	5.02670187	4.99E-07	6.73E-05
Src	144.262212	0.955731811	0.270069	3.53884462	0.00040188	0.01319002
Ccne1	256.719615	0.950776719	0.218807	4.34526914	1.39E-05	0.00097732
Tnxb	596.267036	0.950563579	0.209451	4.5383649	5.67E-06	0.00046831
Ppm1j	87.5287766	0.948537836	0.310766	3.05225295	0.00227131	0.04669367
Abcb4	208.411725	0.947084129	0.217763	4.34915752	1.37E-05	0.00096904
Nek2	211.812147	0.947074544	0.292029	3.24307892	0.00118245	0.02951805
Aspm	488.339849	0.944122723	0.259509	3.63811089	0.00027465	0.00998754
Mmp12	25282.4442	0.943446463	0.135563	6.95946235	3.42E-12	2.12E-09
Gpr68	461.404135	0.941324404	0.183443	5.1314244	2.88E-07	4.25E-05
Mical2	770.002588	0.939376724	0.169506	5.54184706	2.99E-08	6.09E-06
Cep170b	240.906552	0.934517886	0.195342	4.78401526	1.72E-06	0.00018155

B.

Differentially expressed genes, Spironolactone vs Vehicle. Adjusted p value < 0.05

	baseMean	log2FoldChange	lfcSE	stat	pvalue	padj
Gja5	21.6803135	4.092623144	0.707857	5.78171147	7.39E-09	1.21E-05
Pde9a	13.2430787	3.417933603	0.833799	4.09923137	4.15E-05	0.00723301
Btnl9	14.531344	3.07384788	0.880882	3.48951127	0.0004839	0.03591405
Ngf	9.28162056	2.999875428	0.887946	3.37844395	0.00072897	0.04729308
Stmn2	18.786133	2.896506012	0.753917	3.84194492	0.00012206	0.01540062
Avpr1a	15.6701606	2.769102482	0.669663	4.13507125	3.55E-05	0.00646684
Dkk3	20.6723188	2.587080368	0.591593	4.37307297	1.23E-05	0.00301253
Tmeff2	18.5045849	2.547071805	0.719894	3.53812036	0.00040299	0.0317778
Sema3g	87.579052	2.364535036	0.443637	5.32988955	9.83E-08	7.68E-05
Efna1	34.4576976	2.330218353	0.447514	5.20702609	1.92E-07	0.0001259
Has3	17.9053515	2.271959726	0.623017	3.64670669	0.00026562	0.02466817
Alpl	18.8080661	2.188689334	0.599846	3.64875506	0.00026351	0.02466817
Mall	72.0451635	2.184329619	0.487029	4.48501124	7.29E-06	0.00225636
Tpsb2	173.305347	2.131970072	0.545971	3.90491213	9.43E-05	0.01303156
Kbtbd6	19.7225116	2.081670757	0.58913	3.53346302	0.00041015	0.03218822
Sox17	70.8970386	2.052021636	0.366626	5.59704771	2.18E-08	2.73E-05
Mcpt4	147.65164	2.001418118	0.35456	5.64479836	1.65E-08	2.26E-05
Tmem44	27.2013455	1.980419939	0.485831	4.07635442	4.57E-05	0.00773553
Aqp7	39.7798555	1.975009702	0.475261	4.15563444	3.24E-05	0.00636064
Cma1	90.2340313	1.948708891	0.443513	4.39380658	1.11E-05	0.00290491
Ccdc85a	34.8890233	1.899925112	0.501972	3.78492275	0.00015376	0.01732418
Syt7	37.9419408	1.877576211	0.453034	4.14445	3.41E-05	0.00642186
LOC1185684	131.931707	1.877127332	0.27762	6.76150347	1.37E-11	2.24E-07
Efhd1	26.4680236	1.862713056	0.466757	3.99075877	6.59E-05	0.01010102
Dll4	28.0130036	1.854345859	0.534481	3.46943368	0.00052156	0.0376388
Timp4	74.6092456	1.852455505	0.414548	4.46861138	7.87E-06	0.00232835
Gja4	55.4464634	1.787772742	0.399939	4.47011523	7.82E-06	0.00232835
Apln	33.1812543	1.723080913	0.474057	3.63475671	0.00027824	0.02535413
Bcl6b	36.3335252	1.651250515	0.420698	3.92502322	8.67E-05	0.01239847
Galnt18	35.7100059	1.642659008	0.487367	3.37047533	0.00075039	0.04826464
Gzma	64.5243407	1.631380389	0.380546	4.28694251	1.81E-05	0.00396161
Gzmb	83.6274851	1.622616561	0.392272	4.13646071	3.53E-05	0.00646684
C1qtnf9	132.121868	1.599386259	0.293976	5.44053071	5.31E-08	5.13E-05
Myct1	75.0940727	1.532646398	0.424462	3.61079745	0.00030526	0.02706392
Egfl7	212.161934	1.529797492	0.2887	5.2989246	1.16E-07	8.68E-05
Ptprf	46.2167376	1.524479869	0.402855	3.78419209	0.00015421	0.01732418
Spns2	134.491201	1.520343877	0.305126	4.98266907	6.27E-07	0.0003117
Podxl	522.013107	1.516582938	0.391137	3.87737341	0.00010559	0.01390228
Cpa3	85.2722068	1.506674527	0.439666	3.42686526	0.00061059	0.04155572
Cldn5	45.745901	1.499306102	0.421813	3.55443073	0.0003788	0.03075767
Kif26a	51.4034914	1.490745289	0.423959	3.516248	0.00043769	0.03368404
Hbb-bt	79.3082241	1.463067205	0.416345	3.51407699	0.00044128	0.03368404
Ebf3	74.9018318	1.457733821	0.364763	3.99638741	6.43E-05	0.01010102
Clic5	138.477211	1.448374692	0.308801	4.69031838	2.73E-06	0.00106621
Palmd	64.5764013	1.44492259	0.379198	3.81046969	0.0001387	0.01636696
Grrp1	67.601454	1.439461925	0.392134	3.67084133	0.00024175	0.02376219
Dennd5b	38.0958568	1.385657133	0.397609	3.48497722	0.00049218	0.03620053
Sparcl1	715.471945	1.384242869	0.387227	3.57475773	0.00035055	0.02950037
Hba-a2	573.882644	1.347855193	0.226372	5.9541723	2.61E-09	7.15E-06
Ushbp1	102.838738	1.326234163	0.263469	5.03374793	4.81E-07	0.0002701
C130074G19	105.118216	1.323525549	0.313934	4.21593455	2.49E-05	0.00523068
Esam	189.104948	1.310183957	0.253529	5.16778725	2.37E-07	0.0001439
Fmo1	93.7778241	1.308358487	0.353007	3.70632321	0.00021029	0.02186281
Aplnr	259.508928	1.305104165	0.201851	6.46568963	1.01E-10	5.51E-07
Entpd2	60.2769161	1.30286384	0.357208	3.64735762	0.00026495	0.02466817
Cyyr1	118.471708	1.279985802	0.32785	3.90417658	9.45E-05	0.01303156
Ltbp4	821.957465	1.265582648	0.202351	6.25438421	3.99E-10	1.64E-06
Cpe	96.9596578	1.258925788	0.343501	3.66498896	0.00024735	0.02386484
Pcsk6	74.8003454	1.25887353	0.368223	3.41877692	0.00062903	0.0423026
Tinagl1	384.110914	1.258720122	0.21653	5.81315555	6.13E-09	1.12E-05
Robo4	145.10484	1.24464035	0.299631	4.15391507	3.27E-05	0.00636064
Rgs5	659.240823	1.242644327	0.235279	5.28157994	1.28E-07	8.75E-05
Tie1	344.972746	1.240113073	0.210958	5.87848783	4.14E-09	9.53E-06
Xirp1	65.2108671	1.222656397	0.329219	3.71380965	0.00020416	0.02154185
Galnt16	66.7042851	1.217380875	0.318445	3.82289226	0.0001319	0.0160248
Hspa12b	113.502711	1.215319467	0.318486	3.81592893	0.00013567	0.01617169
Hjv	119.540408	1.211736107	0.341439	3.54890677	0.00038683	0.03125543
Tcap	87.5196706	1.211228529	0.350234	3.45834439	0.00054351	0.03842495
Timp3	358.562892	1.210878268	0.216799	5.58524617	2.33E-08	2.73E-05
Nos3	127.993784	1.210438165	0.318877	3.79593669	0.00014709	0.01699949
Lama5	294.194514	1.209065896	0.293954	4.11311845	3.90E-05	0.00695926
Ablim3	186.568473	1.208081922	0.255434	4.72953202	2.25E-06	0.00097133
Ccm2l	66.3897859	1.208023354	0.322286	3.74829383	0.00017804	0.01971026

Cdh1	106.181002	0.930706922	0.260464	3.57326403	0.00035256	0.0119444
Gm40960	60.8735794	0.927495925	0.290719	3.19034943	0.00142101	0.03368408
Gm30042	68.9042094	0.923692004	0.286877	3.21981748	0.00128272	0.03122055
Pdgfb	1277.87797	0.922564598	0.140478	6.56733564	5.12E-11	2.12E-08
Bub1	404.612559	0.922455758	0.178287	5.17397895	2.29E-07	3.55E-05
Plk1	775.708949	0.920285236	0.149383	6.16057821	7.25E-10	2.25E-07
Dcbld2	168.377099	0.914977744	0.211106	4.33421007	1.46E-05	0.001007
Serpinb6b	101.931183	0.914815423	0.271834	3.36534978	0.00076447	0.02104513
Cd276	862.777708	0.913616079	0.144739	6.31217492	2.75E-10	9.87E-08
Pkd1l2	144.529234	0.910816414	0.214799	4.24031734	2.23E-05	0.00140197
Fanci	114.393109	0.909914804	0.26826	3.39190816	0.00069408	0.01987452
Cdca5	197.286883	0.908364379	0.227411	3.99436645	6.49E-05	0.00321507
Dyrk3	69.9847685	0.902488581	0.283084	3.18806443	0.00143229	0.03377519
Raet1d	183.596046	0.894503657	0.282985	3.16096035	0.0015725	0.03614361
Atp2b4	1546.54904	0.893849018	0.107576	8.30896172	9.65E-17	1.46E-13
Mmp19	6634.46475	0.890672306	0.14026	6.35014802	2.15E-10	7.92E-08
Cenpi	186.362392	0.877129946	0.24128	3.63531881	0.00027764	0.01001109
Efr3b	177.744812	0.874487336	0.26656	3.28063576	0.00103573	0.02673685
Myrf	181.449488	0.872820759	0.212046	4.11617669	3.85E-05	0.00221536
Pimreg	247.604873	0.861771815	0.21496	4.00898545	6.10E-05	0.00311296
Cd163	583.223103	0.859763048	0.220756	3.89463689	9.83E-05	0.00451332
Cenpf	614.63874	0.858474787	0.197709	4.34211296	1.41E-05	0.00098639
Mrgpre	153.743479	0.858415634	0.253513	3.38608622	0.00070897	0.02015827
Polq	148.724538	0.858415229	0.281708	3.04718333	0.00230997	0.04706257
Tdrkh	122.870923	0.85807604	0.25587	3.35356506	0.00079778	0.02179097
Agmo	119.225451	0.856356441	0.27141	3.15521467	0.0016038	0.03637238
Ocstamp	306.791858	0.852528938	0.207805	4.10254524	4.09E-05	0.00234018
Atp2a1	1315.18631	0.849679351	0.210991	4.02709125	5.65E-05	0.00292662
Acp5	6632.42492	0.846171236	0.183797	4.60382563	4.15E-06	0.00035783
4930579G24	99.8726692	0.843915587	0.277855	3.03724939	0.00238748	0.04806696
Gja1	275.373339	0.842818805	0.210943	3.9954838	6.46E-05	0.00321162
Depdc1a	145.214293	0.841435145	0.261201	3.2214105	0.00127561	0.03110305
Slc13a3	657.191024	0.834152926	0.179147	4.65625039	3.22E-06	0.00029457
Kifc1	381.547402	0.831020301	0.230669	3.60264872	0.00031499	0.0108489
Hmmr	364.570636	0.826050028	0.204392	4.04149612	5.31E-05	0.00279229
Spc25	274.549421	0.8230673	0.174638	4.71299994	2.44E-06	0.00024285
Etv1	285.960538	0.821097971	0.175906	4.66781425	3.04E-06	0.00028616
Synpo	381.293278	0.817435777	0.159348	5.12989348	2.90E-07	4.25E-05
Sdc1	1256.73097	0.814695566	0.166186	4.90230842	9.47E-07	0.00011129
Agap1	2723.70811	0.814296251	0.117013	6.95901178	3.43E-12	2.12E-09
Dlg3	351.71901	0.812345627	0.211446	3.84185359	0.00012211	0.00545686
Rad54l	181.746157	0.807686814	0.238088	3.39238676	0.00069287	0.01987452
Kntc1	274.482349	0.807247044	0.218453	3.69529508	0.00021963	0.00872763
Pole	482.394548	0.806775293	0.194286	4.15251733	3.29E-05	0.00194029
Syt11	732.72378	0.803215625	0.141682	5.66913301	1.44E-08	3.06E-06
Itgax	13413.8491	0.799307156	0.149362	5.35145929	8.72E-08	1.54E-05
Flnb	1679.25069	0.797499119	0.12654	6.30232615	2.93E-10	1.01E-07
Ccnb2	709.919414	0.793957393	0.141615	5.6064687	2.06E-08	4.26E-06
Ccna2	1212.64633	0.793917355	0.170983	4.64325599	3.43E-06	0.00030754
Ankrd13b	536.305001	0.792395002	0.133491	5.93595771	2.92E-09	7.66E-07
Cenpn	256.480104	0.789404508	0.232142	3.40052101	0.00067258	0.01958805
Ecm1	17942.1514	0.788076122	0.114208	6.90035982	5.19E-12	2.83E-09
Ect2	326.696592	0.787234541	0.178943	4.39935272	1.09E-05	0.00081311
Slc12a2	136.816588	0.786755868	0.251917	3.12307234	0.00178974	0.039282
Tpx2	1215.96658	0.786609176	0.157868	4.98270834	6.27E-07	8.08E-05
Rad51	184.726007	0.78643431	0.217113	3.62224278	0.00029206	0.01035623
Hr	431.397992	0.78478347	0.221424	3.54426167	0.00039371	0.01302506
LOC1185680	696.577728	0.781864549	0.160374	4.87525177	1.09E-06	0.00012447
Hspb7	273.380753	0.78177013	0.21237	3.68117416	0.00023216	0.00904107
Thbs3	150.950874	0.781345831	0.241108	3.24064274	0.00119261	0.02971702
Ube2c	1091.37932	0.779967131	0.142478	5.47430415	4.39E-08	8.68E-06
Ckap2l	717.022991	0.772958971	0.165868	4.66007584	3.16E-06	0.0002911
Clspn	348.053052	0.769736404	0.202252	3.80581981	0.00014134	0.00615463
Diaph3	371.909113	0.768780416	0.17131	4.4876479	7.20E-06	0.00057067
Lrfn4	111.416897	0.762308212	0.232644	3.27671668	0.00105022	0.02700841
Marveld1	1234.20054	0.760346102	0.125531	6.05701862	1.39E-09	3.97E-07
Ctsf	123.585661	0.759450183	0.241583	3.1436408	0.0016686	0.03740631
Prr11	581.580433	0.758183592	0.151669	4.99893235	5.76E-07	7.56E-05
Ndc1	819.714718	0.755678239	0.124786	6.05580657	1.40E-09	3.97E-07
Rrm2	778.527334	0.753523224	0.174519	4.31770282	1.58E-05	0.00106382
Knstrn	467.079977	0.752898848	0.173517	4.33906189	1.43E-05	0.00099508
Cav1	2163.49318	0.750565412	0.162055	4.63154534	3.63E-06	0.00031916
Iqgap3	673.627928	0.746436935	0.177856	4.196863	2.71E-05	0.00164678
Ahi1	253.51477	0.745894322	0.172743	4.31795121	1.57E-05	0.00106382
Kif22	466.056017	0.743952136	0.186877	3.98096504	6.86E-05	0.00337729
Mki67	7290.11976	0.741468339	0.179008	4.14208495	3.44E-05	0.00201328
Pclaf	554.607631	0.738081439	0.154359	4.78158867	1.74E-06	0.00018234
Brca1	352.367691	0.735322268	0.201479	3.6496181	0.00026263	0.00977015
Rad51ap1	160.992426	0.733906893	0.230108	3.18939735	0.0014257	0.03372822
Rapgef5	145.231767	0.733308476	0.209088	3.50718298	0.00045288	0.01438863
Lama2	251.506522	0.73160592	0.225034	3.25108884	0.00114964	0.02880438
Pdpn	5765.01971	0.731583723	0.133478	5.48092797	4.23E-08	8.48E-06
Dcstamp	210.365466	0.730718994	0.223533	3.26894683	0.00107949	0.02739924
Mamld1	310.671805	0.730604712	0.205016	3.56364223	0.00036574	0.01227856
Fzd1	142.74485	0.729934824	0.239816	3.04372429	0.00233669	0.04749655
Chpf	149.274192	0.729457244	0.218067	3.34511113	0.0008225	0.02233192
Asb1	216.969549	0.729223038	0.171744	4.2459996	2.18E-05	0.00137962
Fblim1	260.863388	0.72780652	0.227816	3.194712	0.0013997	0.03341151
Tlcd3a	154.780147	0.72160617	0.204389	3.53055536	0.00041469	0.01339384

Actn2	85.3794662	1.195050315	0.32816	3.64167112	0.00027087	0.02495997
Cdh5	1077.09909	1.18943511	0.198803	5.98296912	2.19E-09	7.15E-06
Ramp2	194.846068	1.168168588	0.272387	4.28864089	1.80E-05	0.00396161
Pcdh1	134.198722	1.160589808	0.278135	4.17276013	3.01E-05	0.00601937
Gimap4	129.464633	1.157076665	0.2736	4.22908873	2.35E-05	0.00499813
Meox2	119.13671	1.155406457	0.25054	4.61165867	3.99E-06	0.00142437
Clu	172.541759	1.151179145	0.228925	5.02861975	4.94E-07	0.0002701
Cdh13	199.940339	1.14288606	0.271943	4.20266535	2.64E-05	0.00540836
Casq1	113.743795	1.137257871	0.318147	3.57462979	0.00035072	0.02950037
Ptprg	127.253974	1.135752332	0.323771	3.50788898	0.00045168	0.03414016
Sox18	223.839024	1.13005914	0.294791	3.83342193	0.00012637	0.01570279
Arhgef15	186.354717	1.11980324	0.323968	3.45652029	0.0005472	0.03851992
Tek	148.125909	1.104187971	0.317275	3.48021887	0.000501	0.03663078
Mmp15	173.768426	1.101707528	0.241471	4.56248273	5.06E-06	0.00176417
Fbln5	123.433863	1.097800795	0.265269	4.13844835	3.50E-05	0.00646684
Fxyd1	109.718179	1.095623934	0.288642	3.79579223	0.00014717	0.01699949
Aqp1	1526.0852	1.086199406	0.201174	5.39930895	6.69E-08	5.49E-05
Dysf	146.204752	1.08306384	0.27	4.01134172	6.04E-05	0.0098046
Col4a2	2073.71679	1.081105969	0.196483	5.50228352	3.75E-08	3.96E-05
Cd34	294.033114	1.078240557	0.188187	5.72961783	1.01E-08	1.50E-05
Jam2	252.519222	1.075401214	0.267298	4.02323659	5.74E-05	0.00941537
Hspb8	115.092462	1.069007908	0.31889	3.35227538	0.0008015	0.04998572
Lama4	301.84347	1.062702388	0.238001	4.46511997	8.00E-06	0.00232835
Prelp	303.025007	1.059032504	0.258137	4.10260327	4.09E-05	0.00720502
Tspan9	153.06502	1.051747562	0.235673	4.46274961	8.09E-06	0.00232835
Sema3f	103.437225	1.048412389	0.288825	3.6299214	0.00028351	0.02564253
Tspan7	221.796596	1.041056338	0.260036	4.00350946	6.24E-05	0.01003573
Krba1	73.9730621	1.030016084	0.306394	3.36173485	0.00077454	0.04886185
Adgrg1	338.453044	1.020003646	0.216025	4.72169666	2.34E-06	0.00098364
Bcam	343.241244	1.015568919	0.184749	5.49702615	3.86E-08	3.96E-05
Myh1	170.869684	1.006561733	0.230906	4.35917769	1.31E-05	0.00314899
Tnxb	596.267036	1.002986021	0.209456	4.78852498	1.68E-06	0.00074479
Atp1b2	96.0723615	0.991355234	0.28581	3.46858485	0.00052321	0.0376388
Afap1l1	145.079659	0.989748052	0.275783	3.58886562	0.00033212	0.02852059
Col4a1	3105.82809	0.989401705	0.18314	5.40242974	6.57E-08	5.49E-05
Npr1	210.315759	0.988883046	0.258623	3.82365168	0.00013149	0.0160248
Itgb4	96.3938709	0.982087598	0.283375	3.46568386	0.00052888	0.03771638
Tnnt3	1810.59571	0.96900885	0.196934	4.92048694	8.63E-07	0.00040456
Pxdn	431.51777	0.968800947	0.214303	4.520713	6.16E-06	0.00206303
Pecam1	823.517269	0.9498053	0.183257	5.18290482	2.18E-07	0.00013781
Adamts2	192.910737	0.946594343	0.229971	4.11614132	3.85E-05	0.00694414
Igfbp7	916.822153	0.945937521	0.215167	4.39629473	1.10E-05	0.00290491
Myl1	2544.13605	0.939804136	0.188031	4.99812861	5.79E-07	0.00029672
Tnni2	549.922252	0.937387307	0.213721	4.38602271	1.15E-05	0.00295856
Slc9a3r2	484.830978	0.931286807	0.171739	5.42268053	5.87E-08	5.35E-05
Shank3	308.034064	0.930111853	0.27646	3.36436253	0.00076721	0.04858582
Tnnc2	992.751373	0.927398665	0.215329	4.30690115	1.66E-05	0.00374143
Lamb2	409.91925	0.925256282	0.213087	4.34215936	1.41E-05	0.00330592
Itih5	207.383738	0.922883112	0.23978	3.84887057	0.00011866	0.01532537
Plekha4	101.43519	0.916335975	0.249814	3.6680772	0.00024438	0.02376219
Islr	253.238299	0.908320622	0.255438	3.5559284	0.00037665	0.03073512
Gimap6	142.037723	0.907711696	0.228964	3.96442959	7.36E-05	0.01107085
Xirp2	270.473409	0.897107469	0.249764	3.59182015	0.00032838	0.02834753
Mcam	222.599042	0.896974866	0.258787	3.46607154	0.00052812	0.03771638
Cavin2	380.976991	0.8953832	0.234687	3.81521885	0.00013606	0.01617169
Il2rb	225.875593	0.893471833	0.240425	3.71621673	0.00020223	0.02154185
Lynx1	141.309466	0.884915079	0.247452	3.57610498	0.00034875	0.02950037
Pear1	100.223477	0.875574226	0.253775	3.45019755	0.00056018	0.03909793
Cd209a	1316.16331	0.869790311	0.196482	4.42682942	9.56E-06	0.0027043
H2-Oa	113.118287	0.866382685	0.23619	3.66816396	0.0002443	0.02376219
Atp2a1	1315.18631	0.858649649	0.211025	4.06894448	4.72E-05	0.00790419
Nid1	907.747776	0.855279034	0.181626	4.70902409	2.49E-06	0.00102064
Gm10222	465.175965	0.848166519	0.250639	3.38401985	0.00071433	0.04667894
Mylk	261.260096	0.845682389	0.233748	3.61792184	0.00029698	0.02661767
Acta1	6282.84714	0.837981013	0.191448	4.37707432	1.20E-05	0.00301253
C1s1	191.64576	0.833077869	0.243702	3.41843237	0.00062983	0.0423026
Pgam2	345.083487	0.827134044	0.223937	3.69360899	0.00022109	0.02252847
Dchs1	152.851477	0.823373937	0.210574	3.91013489	9.22E-05	0.01293159
Pdzd2	136.633674	0.81529421	0.237015	3.43983603	0.00058207	0.04028295
Ndrg2	183.250136	0.814733445	0.222143	3.66760124	0.00024484	0.02376219
Hspb7	273.380753	0.812661814	0.212458	3.82504856	0.00013075	0.0160248
Dcn	1059.55553	0.803784322	0.21239	3.78446788	0.00015404	0.01732418
Nrep	307.924426	0.793085769	0.170054	4.66374149	3.11E-06	0.00113178
Heg1	570.771722	0.786253056	0.178708	4.39964124	1.08E-05	0.00290491
Cd7	222.071274	0.785911997	0.224857	3.4951705	0.00047376	0.03545032
Ehd2	962.835477	0.784918246	0.222827	3.52254695	0.00042742	0.0330687
Adamts1	235.511012	0.774351157	0.219526	3.52737747	0.0004197	0.03262505
Pitpnm2	205.126849	0.769076266	0.222738	3.45283641	0.00055473	0.03888293
Sparc	4512.2931	0.750870392	0.174393	4.30561941	1.67E-05	0.00374143
Mmp2	489.274332	0.748010159	0.19203	3.89527532	9.81E-05	0.01322144
Col15a1	1087.70289	0.733370424	0.125163	5.85932621	4.65E-09	9.53E-06
Rapgef5	145.231767	0.731954959	0.209477	3.49419413	0.0004755	0.03545032
Thy1	327.180196	0.731624704	0.203178	3.60090644	0.00031711	0.02751975
Epas1	933.879657	0.727839217	0.169031	4.3059591	1.66E-05	0.00374143
Tns2	347.025172	0.724157539	0.198215	3.65338573	0.0002588	0.02460101
Hspg2	2549.87518	0.716322865	0.174968	4.09401537	4.24E-05	0.00731989
Mgll	425.791218	0.711673965	0.182731	3.89464501	9.83E-05	0.01322144
Synpo	381.293278	0.702893329	0.159925	4.39513504	1.11E-05	0.00290491
Fbln2	965.761447	0.697686112	0.17673	3.94775382	7.89E-05	0.0117629

Mcm10	381.867869	0.721068403	0.190812	3.77895338	0.00015749	0.00666638
Kif18b	399.05026	0.720977653	0.194399	3.70875407	0.00020828	0.00839905
Tnfrsf23	381.054474	0.720451523	0.230041	3.13184479	0.00173712	0.03843653
Cxcl14	1986.72092	0.71637636	0.166899	4.29228259	1.77E-05	0.00115885
Nkain1	226.169883	0.713548899	0.195708	3.64599428	0.00026636	0.00981212
Kif2c	342.662012	0.711859041	0.16691	4.26492556	2.00E-05	0.00129789
Tm4sf19	556.377917	0.711812168	0.187697	3.79235527	0.00014923	0.00638906
Aurka	439.150169	0.70686911	0.176523	4.00439889	6.22E-05	0.00312257
Gm11223	133.698284	0.706221015	0.228656	3.08856856	0.00201123	0.04283297
Top2a	2858.40705	0.702747971	0.144521	4.8625946	1.16E-06	0.00013159
Clmp	455.34311	0.694912244	0.157742	4.40537706	1.06E-05	0.00079521
Car5b	314.155484	0.692942737	0.168216	4.11935297	3.80E-05	0.0021943
Foxm1	1062.57746	0.6926396	0.159291	4.34826931	1.37E-05	0.00096904
Cmtm4	254.530657	0.690136364	0.21651	3.18754778	0.00143485	0.03377715
Cd81	832.553337	0.689281744	0.157012	4.38998224	1.13E-05	0.00083519
Prnp	509.588094	0.687344087	0.151431	4.5389975	5.65E-06	0.00046831
Layn	272.992295	0.685617459	0.212537	3.22587598	0.00125588	0.0308981
Ttk	176.82898	0.684354593	0.211887	3.22980994	0.00123873	0.03058664
Cenpe	838.962602	0.683651538	0.197398	3.4633084	0.00053358	0.01637984
Cdk18	1694.77912	0.679632136	0.137561	4.94060306	7.79E-07	9.48E-05
Micall2	357.721827	0.67878223	0.165906	4.09135444	4.29E-05	0.00243224
Pbk	217.32691	0.678688678	0.215703	3.14640603	0.0016529	0.03711545
Spag5	439.854656	0.676148917	0.19416	3.48243261	0.00049688	0.01553321
Syn1	435.069343	0.674806628	0.168645	4.00135387	6.30E-05	0.00314444
Cep55	378.746741	0.67150632	0.184517	3.6392724	0.00027341	0.00998754
B3gnt3	698.978577	0.670621847	0.124558	5.38401983	7.28E-08	1.32E-05
Kif15	392.016418	0.670363391	0.182984	3.66351796	0.00024877	0.00949943
Prc1	825.202673	0.668688882	0.148305	4.50887696	6.52E-06	0.00052874
Endod1	1559.46702	0.667397896	0.124147	5.37587106	7.62E-08	1.37E-05
Mmp2	489.274332	0.666126415	0.192088	3.46782406	0.00052469	0.01617994
Ptgir	746.78684	0.664825574	0.146058	4.55180109	5.32E-06	0.00044751
Mybl2	291.383093	0.66438883	0.19361	3.4315902	0.00060005	0.01801482
Il27ra	182.459362	0.663991067	0.216698	3.0641254	0.00218307	0.04556707
Srgap3	282.297164	0.663843947	0.212076	3.13021927	0.00174676	0.03858723
Prune2	1098.54854	0.661941691	0.149685	4.42223372	9.77E-06	0.00073989
Flt1	694.282961	0.659453327	0.199393	3.30729723	0.00094201	0.02493122
Cd200	610.68457	0.658623792	0.19453	3.38572595	0.0007099	0.02015827
Phyhd1	223.898427	0.656892894	0.194294	3.38091397	0.00072245	0.02047197
Spn	1428.09025	0.653530614	0.149255	4.37860788	1.19E-05	0.00087525
Bend4	327.04035	0.651355632	0.198096	3.28808445	0.00100872	0.02631069
Pls3	372.263832	0.650637388	0.200082	3.25186094	0.00114652	0.02877915
Gtse1	412.22964	0.649125188	0.156964	4.13551363	3.54E-05	0.00205414
Creg2	227.879082	0.643637015	0.183234	3.51265439	0.00044365	0.01416161
Kif4	243.311923	0.642213083	0.202193	3.17623086	0.00149202	0.03476283
Melk	287.36362	0.637392693	0.175926	3.62307924	0.00029112	0.01035623
Ppp1r12b	799.940105	0.63604314	0.135103	4.70785152	2.50E-06	0.00024726
Cd209a	1316.16331	0.63384504	0.196626	3.22361225	0.00126585	0.03092024
Ccnf	410.560884	0.632572048	0.177734	3.55908853	0.00037214	0.01237152
Mthfd2	645.139455	0.631111858	0.18756	3.36485498	0.00076584	0.02104513
Racgap1	1327.36819	0.630891672	0.139379	4.52646095	6.00E-06	0.00048953
Cip2a	532.509298	0.62802245	0.154713	4.05926439	4.92E-05	0.00269466
Cdca3	845.11542	0.623438648	0.167648	3.71872819	0.00020023	0.0081466
St18	234.554176	0.619368564	0.199723	3.10114504	0.00192774	0.04150881
Cd109	3073.61929	0.618431131	0.163095	3.79184678	0.00014953	0.00638906
Arhgef40	481.515324	0.617810801	0.153943	4.0132416	5.99E-05	0.00308041
Paqr4	355.010328	0.617112051	0.158768	3.88686782	0.00010155	0.00464454
Cdc20	530.720394	0.614331283	0.176104	3.48845253	0.00048583	0.01525759
Slc43a3	548.966423	0.611652355	0.165209	3.70230094	0.00021365	0.00853985
Slc39a14	1501.61027	0.611103039	0.179784	3.39909222	0.0006761	0.01960688
Igf2r	7740.19303	0.610842373	0.144053	4.2404004	2.23E-05	0.00140197
2900026A02	407.813775	0.608173657	0.134142	4.53381512	5.79E-06	0.00047564
Bub1b	856.011266	0.606774932	0.140623	4.31490196	1.60E-05	0.0010721
Carmil1	490.27005	0.60344103	0.178634	3.37807976	0.00072994	0.02059849
Pcdh7	576.606083	0.600355381	0.168034	3.57281701	0.00035316	0.0119444
Hivep3	1193.35206	0.5995933	0.169241	3.54282736	0.00039586	0.0130644
Ccnd1	2217.68528	0.587029238	0.152783	3.84223517	0.00012192	0.00545686
Dennd11	333.140046	0.586115631	0.185291	3.16322426	0.00156032	0.03592428
Hells	351.085842	0.584112307	0.184591	3.16435616	0.00155426	0.03584539
Rrm1	1727.70874	0.580464142	0.135221	4.29272132	1.76E-05	0.00115885
Birc5	691.246804	0.57370068	0.167604	3.42295063	0.00061945	0.01838548
Ppp2r3a	282.18155	0.573642442	0.1671	3.43292241	0.00059711	0.01796611
Fn1	123993.871	0.571705481	0.152131	3.75797672	0.00017129	0.00707491
Mad2l1	672.498447	0.569795537	0.149808	3.8034936	0.00014267	0.00619295
Capn5	499.257285	0.569579727	0.15051	3.78433421	0.00015412	0.00654412
Arhgef12	672.196382	0.567912207	0.183363	3.09720021	0.00195358	0.04186679
Phldb1	1271.91896	0.56613884	0.11912	4.75268663	2.01E-06	0.00020418
Tk1	514.152373	0.565797884	0.133503	4.23810359	2.25E-05	0.00140293
Tlr5	521.545971	0.563497052	0.158311	3.55942669	0.00037167	0.01237152
Chst11	2153.28314	0.562057051	0.132604	4.23860336	2.25E-05	0.00140293
Mis18bp1	420.341972	0.555359869	0.16458	3.37440752	0.00073975	0.02074642
Twsg1	718.861722	0.553296803	0.1365	4.05344999	5.05E-05	0.00273406
Ercc6l	255.360143	0.550935659	0.181812	3.03024399	0.00244356	0.04871223
Tfdp2	292.284169	0.548442347	0.180504	3.03839879	0.00237839	0.0480469
Smc2	1206.06413	0.545982939	0.150019	3.63942932	0.00027324	0.00998754
Gm42226	3195.50639	0.545780202	0.107379	5.08273297	3.72E-07	5.23E-05
Fgfr1	1438.55526	0.545671251	0.166757	3.27225309	0.00106694	0.02723295
Dlgap5	484.532158	0.544870525	0.179899	3.02875961	0.0024556	0.04886106
L1cam	1617.61021	0.544622897	0.101843	5.34765978	8.91E-08	1.56E-05
Gask1b	1036.09616	0.543790166	0.147794	3.6793814	0.0002338	0.00905312

Ldb3	296.860534	0.696455413	0.189195	3.68115528	0.00023218	0.02322079
Hspb6	718.215792	0.695633677	0.188337	3.69355963	0.00022114	0.02252847
Lamb1	435.587191	0.674714675	0.187224	3.60378205	0.00031362	0.02736168
Pcolce	512.640691	0.657453537	0.184273	3.5678219	0.00035996	0.02996995
Pkn3	332.04514	0.62847226	0.176229	3.56623316	0.00036215	0.02999985
Fstl1	907.393322	0.612185822	0.155426	3.9387577	8.19E-05	0.01210269
Thbs4	1194.42461	0.609176898	0.173361	3.51392606	0.00044154	0.03368404
Lbp	330.634841	0.605299887	0.166796	3.62898764	0.00028453	0.02564253
Caskin2	510.851582	0.594151768	0.152376	3.89923857	9.65E-05	0.01318935
H2-Ab1	34454.6869	0.580113812	0.145369	3.99064105	6.59E-05	0.01010102
H2-DMb1	4141.4647	0.570685031	0.145515	3.92181923	8.79E-05	0.01242634
Cbx6	331.771727	0.570384912	0.164256	3.47254656	0.00051555	0.03741583
Grap2	420.446282	0.554900358	0.162824	3.40798456	0.00065445	0.04296891
Col3a1	10433.0917	0.554706504	0.153503	3.61364332	0.00030192	0.02691393
LOC1185680	313.927923	0.549270449	0.163161	3.36643279	0.00076147	0.04844719
Flt3	664.015232	0.543756809	0.145123	3.74687237	0.00017905	0.01971026
H2-Aa	30334.7462	0.539294736	0.157447	3.42524335	0.00061425	0.04163187
Ciita	2835.34849	0.534281999	0.138913	3.84615921	0.00011998	0.0153748
Clec10a	1365.29379	0.450774925	0.112856	3.99425308	6.49E-05	0.01010102
Mcl1	28715.3121	-0.319965858	0.095439	-3.3525587	0.00080068	0.04998572
Sh2b2	3334.65268	-0.322059566	0.094396	-3.4117838	0.00064539	0.04293635
Ipo7	3809.96398	-0.322377085	0.088257	-3.6527181	0.00025948	0.02460101
Nfe2l2	8749.0473	-0.325507737	0.082944	-3.9244451	8.69E-05	0.01239847
Exoc5	1951.71423	-0.325820435	0.094877	-3.4341312	0.00059446	0.04079612
Rai14	2315.99357	-0.327603714	0.092862	-3.5278721	0.00041891	0.03262505
Txnrd1	3361.74032	-0.348799762	0.10352	-3.3694047	0.00075331	0.04826464
Emp1	28924.5757	-0.351526158	0.103154	-3.4077812	0.00065493	0.04296891
Foxn2	1683.93672	-0.365791873	0.108856	-3.3603291	0.0007785	0.04892301
Kdm7a	3142.89006	-0.375544459	0.106127	-3.5386371	0.0004022	0.0317778
Tagap	822.462193	-0.37668362	0.110459	-3.4101792	0.0006492	0.04293635
Sgms1	1912.37875	-0.400926967	0.112052	-3.578028	0.0003462	0.02950037
Pag1	1647.97545	-0.405941445	0.120239	-3.3761342	0.00073512	0.04747026
Mdm2	3306.72657	-0.407836298	0.102176	-3.991527	6.56E-05	0.01010102
Slc38a2	4854.80314	-0.410358937	0.115339	-3.557837	0.00037392	0.03066527
Hif1a	6252.22704	-0.416414485	0.118584	-3.5115595	0.00044549	0.03382805
Snx18	2929.00846	-0.424681894	0.114087	-3.722435	0.00019731	0.02129138
Gna13	5822.51613	-0.424830421	0.117748	-3.6079655	0.00030861	0.02708447
Zfand2a	2287.62268	-0.431230608	0.123316	-3.4969685	0.00047058	0.03540555
Fem1c	1861.37339	-0.433895627	0.109241	-3.9719217	7.13E-05	0.01082762
Lrrc8b	722.129181	-0.441663569	0.129234	-3.4175467	0.00063188	0.0423026
Got1	1918.37569	-0.446062545	0.120633	-3.697687	0.00021757	0.02244422
Cflar	2739.18756	-0.447026759	0.118972	-3.7574087	0.00017168	0.01915597
Tob2	3647.91922	-0.468187203	0.128651	-3.6391985	0.00027349	0.02506005
Pvr	1393.87813	-0.476048564	0.130562	-3.6461455	0.0002662	0.02466817
Etv5	775.71836	-0.479036735	0.125542	-3.8157554	0.00013577	0.01617169
Skil	3738.60828	-0.479358447	0.106547	-4.4990245	6.83E-06	0.0022394
Bhlhe40	6894.25639	-0.484667922	0.115825	-4.1844714	2.86E-05	0.00578789
Pmaip1	1107.49399	-0.488981685	0.135534	-3.607811	0.00030879	0.02708447
Chka	681.616482	-0.495672206	0.129273	-3.8343011	0.00012592	0.01570279
Rab20	871.055612	-0.496233219	0.139287	-3.5626788	0.00036709	0.03025631
Tgif1	4715.85137	-0.498820078	0.10689	-4.6666661	3.06E-06	0.00113178
Fos	38309.0125	-0.508574092	0.146166	-3.4794228	0.0005025	0.03663078
Klf6	16560.0913	-0.511192836	0.116354	-4.3934272	1.12E-05	0.00290491
Lin54	709.138247	-0.515584988	0.137977	-3.7367342	0.00018643	0.02025004
Cd274	1635.7048	-0.526696327	0.129051	-4.0813107	4.48E-05	0.00765127
Jdp2	1312.23577	-0.527178294	0.148801	-3.5428323	0.00039585	0.03167221
Sde2	1060.15088	-0.529949602	0.118075	-4.4882361	7.18E-06	0.00225636
Dusp1	36375.2985	-0.537740564	0.146286	-3.6759497	0.00023697	0.02355588
Ccl3	4963.57361	-0.545366847	0.153778	-3.5464509	0.00039046	0.03139355
Slc7a1	1063.68932	-0.546000128	0.135126	-4.0406667	5.33E-05	0.00883048
Tlr2	7927.17424	-0.55037712	0.140016	-3.93081	8.47E-05	0.01228846
Zc3h12a	2635.02794	-0.552040132	0.151086	-3.6538214	0.00025837	0.02460101
Malt1	2764.34929	-0.552623185	0.118287	-4.6718983	2.98E-06	0.00113178
Fn1	123993.871	-0.560963867	0.152147	-3.6869752	0.00022694	0.02296985
Klf9	627.31858	-0.562289104	0.144465	-3.8922208	9.93E-05	0.01324572
Pim3	1178.95538	-0.564458921	0.167683	-3.3662182	0.00076206	0.04844719
Mir22hg	390.872301	-0.571527392	0.165117	-3.4613568	0.00053746	0.03816199
Ccl4	3450.63313	-0.576485799	0.122914	-4.6901388	2.73E-06	0.00106621
Kdm6b	9537.86459	-0.576911827	0.167931	-3.4354049	0.00059167	0.04077545
Nfkbie	2543.01798	-0.579442862	0.147313	-3.933407	8.38E-05	0.01226493
Ptp4a1	1518.69464	-0.584240964	0.134493	-4.3440166	1.40E-05	0.00330592
Olr1	832.198201	-0.607640473	0.176325	-3.4461314	0.00056867	0.03952283
Dusp10	261.896416	-0.610704079	0.180775	-3.3782477	0.00072949	0.04729308
Ppp1r3b	1014.12365	-0.616798526	0.174328	-3.5381421	0.00040295	0.0317778
Osgin2	355.336529	-0.629954238	0.162504	-3.8765465	0.00010595	0.01390228
Tnf	4242.0673	-0.647071232	0.174604	-3.7059452	0.0002106	0.02186281
Cd14	26114.3924	-0.655529005	0.172902	-3.7913433	0.00014983	0.01718593
Odc1	2758.901	-0.664873818	0.156735	-4.2420217	2.22E-05	0.00478065
Tnfrsf12a	426.603035	-0.666429041	0.160741	-4.145972	3.38E-05	0.00642186
Ets2	5364.8704	-0.666921621	0.173551	-3.8428045	0.00012164	0.01540062
Nfil3	3415.87786	-0.678220666	0.163349	-4.1519689	3.30E-05	0.00636064
Fkbp5	1715.16312	-0.679931822	0.151592	-4.4852623	7.28E-06	0.00225636
Nfkbiz	10648.9914	-0.706253266	0.167898	-4.2064441	2.59E-05	0.00538609
Tnfaip3	13274.0989	-0.713014125	0.192036	-3.7129155	0.00020489	0.02154185
Dusp5	1519.16393	-0.728255105	0.188376	-3.8659729	0.00011065	0.01440346
Per1	5339.04002	-0.729825629	0.161409	-4.5215866	6.14E-06	0.00206303
Tiparp	2327.1678	-0.731265393	0.151832	-4.816285	1.46E-06	0.00066636
Hilpda	690.428397	-0.793960429	0.181597	-4.3720985	1.23E-05	0.00301253
Ccrl2	9875.52451	-0.816094745	0.16309	-5.0039615	5.62E-07	0.00029672

Uhrf1	940.582322	0.54362973	0.165967	3.27551923	0.00105468	0.0270212
Ank	2245.47629	0.543315986	0.102845	5.28287762	1.27E-07	2.11E-05
Pkmyt1	501.738366	0.5422361	0.172614	3.1413268	0.00168184	0.03757953
Nectin1	299.948608	0.541196546	0.160478	3.37239711	0.00074517	0.02085556
Nes	1062.56158	0.539386971	0.146179	3.68990233	0.00022434	0.00888883
Rassf8	800.329752	0.539078421	0.137319	3.92572809	8.65E-05	0.00406399
Aurkb	631.596384	0.538810532	0.164628	3.2728933	0.00106453	0.02722232
Rai14	2315.99357	0.537643016	0.091809	5.85612606	4.74E-09	1.17E-06
Tacc2	479.879007	0.537215443	0.176868	3.03738324	0.00238642	0.04806696
Afdn	1109.58075	0.53635434	0.133286	4.02408526	5.72E-05	0.00295302
Dst	706.770137	0.533626489	0.174619	3.05595551	0.00224345	0.04638156
Gmpr	286.020997	0.533235321	0.151759	3.51370706	0.0004419	0.01413872
Myadm	12946.905	0.531213289	0.108599	4.89149346	1.00E-06	0.00011559
Nusap1	632.440927	0.529114076	0.143526	3.68653287	0.00022733	0.00898025
Tjp2	1038.56695	0.528898836	0.140841	3.75529784	0.00017314	0.00712941
Asf1b	698.25243	0.527611197	0.156702	3.36697864	0.00075997	0.02101893
Kif23	901.379852	0.527526035	0.138199	3.81715772	0.000135	0.00591647
Actn1	4763.35446	0.525702023	0.112289	4.6816845	2.85E-06	0.00027311
Stxbp1	360.826352	0.525660284	0.165527	3.17567417	0.00149489	0.03477017
Flt3	664.015232	0.523732199	0.145043	3.61087022	0.00030517	0.01063807
Kcnn4	3638.14436	0.522320725	0.105026	4.97327497	6.58E-07	8.23E-05
Gm6169	686.628088	0.521826124	0.150787	3.4606929	0.00053879	0.01646562
Rcan1	1957.2992	0.521499871	0.116152	4.48981039	7.13E-06	0.00057067
Slc7a1	1063.68932	0.519032947	0.133109	3.89931848	9.65E-05	0.0044419
Dagla	724.787782	0.517172942	0.126675	4.08266282	4.45E-05	0.00248706
Igsf9	420.272947	0.516473266	0.148607	3.47543935	0.00051002	0.01580669
Ago2	2569.07801	0.515011875	0.163075	3.15812797	0.00158786	0.03630684
Tyms	341.258181	0.51489667	0.167341	3.07693898	0.00209138	0.04433208
Nrp2	4920.90774	0.514427586	0.137264	3.74771531	0.00017845	0.00730423
Rhoc	3769.21906	0.514210351	0.109591	4.69209327	2.70E-06	0.00026141
Pde4dip	983.579528	0.514005537	0.120838	4.25367204	2.10E-05	0.00134568
Nemp1	348.694529	0.510513722	0.144584	3.53091396	0.00041413	0.01339384
Slc39a10	1399.4183	0.510203763	0.120576	4.23138245	2.32E-05	0.00142599
Fasn	1717.76945	0.508745138	0.138366	3.67679912	0.00023618	0.00909355
Xpr1	3340.41882	0.506898815	0.11926	4.25035178	2.13E-05	0.0013594
Cks1b	429.950849	0.504816119	0.159263	3.16970896	0.00152592	0.03531112
Cbx6	331.771727	0.503522419	0.164299	3.06467711	0.00217905	0.04555286
Ahnak2	2234.44441	0.497143907	0.162808	3.05355043	0.00226151	0.04656245
Dpep2	7871.67714	0.490273076	0.134059	3.65715127	0.00025503	0.00970883
Lclat1	631.86888	0.489505024	0.134173	3.64830325	0.00026398	0.00977015
Plp2	5533.00479	0.488502904	0.102497	4.76604355	1.88E-06	0.000194
F13a1	17341.232	0.487646594	0.137009	3.55921934	0.00037196	0.01237152
Tmem254a	303.482776	0.482731411	0.157344	3.06799361	0.00215501	0.04525858
Slc7a5	714.74977	0.480357784	0.156719	3.06508618	0.00217607	0.04555286
Alox5	1073.3991	0.480002235	0.15839	3.03051356	0.00244138	0.04871223
Slc1a5	2281.49648	0.478738099	0.120308	3.97926247	6.91E-05	0.00338933
Espl1	958.896017	0.47195946	0.137671	3.42817873	0.00060765	0.01818364
Pald1	738.586156	0.468793357	0.150438	3.11618705	0.00183206	0.03982613
Zadh2	535.46555	0.467862977	0.138797	3.37083296	0.00074941	0.02093135
Gusb	19462.7499	0.465842004	0.099124	4.69958865	2.61E-06	0.0002538
Ptgfrn	882.992722	0.465282695	0.144124	3.22835156	0.00124506	0.03068743
Fyn	1825.81132	0.464716774	0.108094	4.29918726	1.71E-05	0.00113424
Lamc1	2813.12756	0.464277543	0.114694	4.04796703	5.17E-05	0.00275104
Ppfibp1	761.670085	0.462094076	0.138022	3.34797218	0.00081405	0.02214676
St3gal1	6031.56689	0.460332626	0.106245	4.33275272	1.47E-05	0.0010086
Pam	981.247652	0.458518126	0.123558	3.71094822	0.00020648	0.00835128
Arhgap5	836.496552	0.457320143	0.138636	3.29871663	0.00097128	0.02555701
Shtn1	2308.25658	0.455711123	0.137182	3.32195612	0.00089389	0.02384283
Anpep	19550.0877	0.455651509	0.114977	3.96296898	7.40E-05	0.00360335
Stmn1	1693.42684	0.454150473	0.146875	3.09208498	0.00198756	0.0424615
Dhrs9	2376.04727	0.453042563	0.132882	3.40936185	0.00065115	0.01912755
Creld1	937.49811	0.451685208	0.111511	4.05059169	5.11E-05	0.00275104
Atp6v0d2	10262.1374	0.446846074	0.11769	3.79680494	0.00014657	0.00632212
Srxn1	1436.22237	0.443480762	0.130736	3.39218349	0.00069338	0.01987452
Dock4	1386.62948	0.437765067	0.122331	3.57852667	0.00034554	0.0117448
Slc44a1	1586.33845	0.437031153	0.124787	3.50221693	0.0004614	0.01459149
Mcm6	2619.16258	0.437007171	0.132231	3.304874	0.00095019	0.02509904
Plec	21070.2699	0.434246269	0.126001	3.44637488	0.00056816	0.01721055
Aig1	602.920475	0.43342084	0.133243	3.25285266	0.00114253	0.02873182
Fads1	1891.20678	0.428994907	0.124478	3.44635031	0.00056821	0.01721055
Il2rg	4979.99546	0.428210263	0.108228	3.95656033	7.60E-05	0.00368819
Incenp	2091.36229	0.425742641	0.139248	3.05743455	0.0022324	0.04631304
Mcm4	1234.95474	0.425575556	0.133392	3.19041201	0.0014207	0.03368408
Septin11	4050.76757	0.425261207	0.099116	4.29053257	1.78E-05	0.00116243
Ptprs	2692.81935	0.424917313	0.14016	3.03166052	0.00243213	0.0486842
Agpat4	1263.18009	0.421238388	0.135039	3.11938641	0.00181228	0.03952223
Mcm5	1753.34843	0.420983914	0.12025	3.5009162	0.00046366	0.01462895
Pde8a	1669.31727	0.420301419	0.108165	3.88576001	0.00010201	0.00465017
Prkar2a	1835.09328	0.419167756	0.108902	3.84903209	0.00011859	0.00537502
Micall1	1138.9026	0.417244394	0.122633	3.40237897	0.00066802	0.01953885
Vopp1	619.175142	0.414433787	0.130341	3.17960167	0.00147478	0.0344789
Slc12a4	1717.66727	0.410793296	0.113074	3.63297099	0.00028018	0.01007601
Lmna	21916.4372	0.409451202	0.108582	3.77090752	0.00016265	0.00684255
Alg8	831.516784	0.409417234	0.112585	3.63650339	0.00027636	0.01001109
Lig1	1249.91549	0.409385127	0.131221	3.11980882	0.00180968	0.03952223
Serpinb8	1392.78557	0.409381308	0.103737	3.94635484	7.93E-05	0.00378161
Abcb1b	981.620782	0.407565894	0.107984	3.7743296	0.00016044	0.00677021
Smpd1	1117.4934	0.405555223	0.116416	3.48368334	0.00049456	0.01549635
E2f8	779.418183	0.403637349	0.120773	3.34211868	0.00083142	0.0225292

Ck-ps3	119.774587	-0.822974002	0.239947	-3.429822	0.00060398	0.04127681
Thbs1	53731.0359	-0.835868764	0.128728	-6.4932816	8.40E-11	5.51E-07
Ifrd1	5448.15684	-0.883525565	0.167106	-5.2872244	1.24E-07	8.75E-05
Gm39701	893.59768	-0.912552803	0.180638	-5.051826	4.38E-07	0.00025634
Vegfa	1763.10632	-0.935330406	0.188452	-4.9632277	6.93E-07	0.00033446
Gm36043	166.795754	-1.020703812	0.274769	-3.7147688	0.00020339	0.02154185
Slco4a1	166.927003	-1.040191081	0.30501	-3.4103556	0.00064878	0.04293635
Gm6969	146.547425	-1.346031823	0.365225	-3.6854822	0.00022827	0.02296985
Gm41590	105.126674	-1.537455917	0.410723	-3.7432938	0.00018162	0.01985994
LOC1185684	41.0213392	-1.650438563	0.434329	-3.7999738	0.00014471	0.01695397
LOC1185679	38.1982889	-1.664674201	0.477626	-3.4853055	0.00049158	0.03620053
Gm46226	22.8490525	-2.161401401	0.604974	-3.5727169	0.0003533	0.02956516

Lrrc8b	722.129181	0.403567015	0.12679	3.18294421	0.00145786	0.03414191
Hif1a	6252.22704	0.402367744	0.118289	3.4015534	0.00067004	0.019556
Hspa4l	944.682619	0.402053364	0.125639	3.20007674	0.00137391	0.0330855
Pdlim1	2035.59059	0.400749662	0.110073	3.64076691	0.00027183	0.00998653
Pdgfa	1067.86257	0.40023268	0.128827	3.10674892	0.00189157	0.04098902
Rnf141	1319.92785	0.399741165	0.122038	3.27555392	0.00105455	0.0270212
Alcam	5975.83864	0.395660523	0.117614	3.36405964	0.00076805	0.02106341
Dag1	3183.15436	0.395511074	0.11644	3.39670178	0.00068203	0.01969513
Impdh1	1057.42148	0.394371256	0.120615	3.26967602	0.00107671	0.0273797
Gnptab	6103.20569	0.392384819	0.126833	3.09370145	0.00197676	0.04229714
Gpr137b	4354.50911	0.392302764	0.108898	3.60247701	0.0003152	0.0108489
Kifc3	1467.7367	0.390697748	0.101653	3.84344139	0.00012132	0.00545686
Galnt2	3117.14299	0.384990641	0.101387	3.79723313	0.00014632	0.00632212
Rbpj	6956.20858	0.381514824	0.103523	3.68529882	0.00022843	0.00898025
Tmbim1	3846.76067	0.380490147	0.095979	3.96431288	7.36E-05	0.00359596
Arsb	2462.91502	0.379845195	0.118728	3.19929975	0.00137762	0.0331163
Sbk1	806.398473	0.375272472	0.119657	3.13623594	0.00171131	0.03805093
Mfsd6	2300.12194	0.374445318	0.095508	3.92057008	8.83E-05	0.00411805
Gnl3l	622.624977	0.373859512	0.119657	3.12442932	0.0017815	0.03916434
Akt3	1730.32478	0.372486467	0.107011	3.4808314	0.00049986	0.0155906
Galnt7	1834.57836	0.371599862	0.09655	3.84879548	0.0001187	0.00537502
Atp1a1	23817.1661	0.368554107	0.0899	4.09961159	4.14E-05	0.00236012
Ipo8	931.036087	0.367949003	0.114512	3.21318128	0.00131273	0.0318373
Pfkp	3712.15389	0.364213407	0.089975	4.04794001	5.17E-05	0.00275104
Ywhah	7597.29851	0.359718705	0.082955	4.33630974	1.45E-05	0.00100249
Cyb5r3	5933.89187	0.351054787	0.112085	3.13203652	0.00173598	0.03843653
Spred1	2560.6689	0.347151099	0.097454	3.56221725	0.00036774	0.01231509
Abcc1	3518.34991	0.345473125	0.108486	3.18448607	0.00145011	0.034019
Jup	1810.0609	0.345251072	0.107977	3.19744246	0.00138652	0.03324474
Cnih1	1835.31004	0.343134013	0.112045	3.06247649	0.00219514	0.04567896
Tmem109	3280.09155	0.343130051	0.101335	3.38610433	0.00070892	0.02015827
Dock5	1567.86683	0.341591533	0.111298	3.06916638	0.00214657	0.04515085
Hsph1	2902.0004	0.339175657	0.098002	3.46090277	0.00053837	0.01646562
Fam102b	2298.37059	0.337027131	0.097864	3.44382231	0.00057355	0.01733375
Sun1	1117.7629	0.334793452	0.098311	3.40544006	0.00066058	0.01936266
Brip1os	2092.23094	0.333051302	0.109004	3.05541255	0.00224751	0.04638156
Csf2rb	11100.4183	0.33296721	0.109111	3.05162984	0.00227603	0.04672023
Tmem43	4166.66811	0.331764618	0.093792	3.53724095	0.00040433	0.01321589
Inf2	4949.39882	0.329604598	0.08705	3.78636233	0.00015287	0.00651125
Praf2	1008.59817	0.327036989	0.099343	3.29199029	0.00099481	0.02603525
Dpagt1	1951.88757	0.313056537	0.094284	3.32035909	0.00089902	0.02393282
Ipo7	3809.96398	0.312774297	0.087751	3.56432038	0.0003648	0.0122771
Otud6b	1203.92677	0.311594021	0.098727	3.1561185	0.00159884	0.03632029
Cyb5b	1586.28504	0.309759906	0.096946	3.19517678	0.00139745	0.03341151
Ptpn7	3123.50069	0.299813909	0.094608	3.16901687	0.00152956	0.03533532
Gpalpp1	1660.58821	0.298962581	0.093357	3.20236962	0.00136302	0.03288135
Capn2	9209.21884	0.296252578	0.083725	3.53839239	0.00040257	0.01319002
Cers5	1799.63731	0.2951662	0.091964	3.20957205	0.00132933	0.03218247
Ddb1	6731.09076	0.262456292	0.083379	3.14774626	0.00164534	0.03706784
Itgb1	14522.8266	0.259211886	0.076801	3.37509458	0.0007379	0.02073736
Nfe2l2	8749.0473	-0.258475328	0.0829	-3.1179113	0.00182138	0.03965712
Stx4a	3343.60767	-0.273580631	0.084822	-3.2253609	0.00125814	0.0308981
Cln3	4069.04319	-0.276417138	0.087163	-3.1712489	0.00151785	0.03518418
Sin3b	2664.70463	-0.281861958	0.085623	-3.2918845	0.00099518	0.02603525
Arl11	3508.87949	-0.291608209	0.09392	-3.1048738	0.0019036	0.04118429
Rbm39	7303.31769	-0.291890645	0.09145	-3.1917951	0.00141392	0.03368408
Hmox2	2395.21275	-0.297223108	0.095228	-3.1211857	0.00180124	0.03940763
Aldh2	13485.0917	-0.298350223	0.097654	-3.0551715	0.00224932	0.04638156
Sdcbp	23797.9378	-0.300558357	0.094249	-3.1889674	0.00142782	0.03372822
Ccnl2	3684.94326	-0.305490472	0.095725	-3.1913332	0.00141618	0.03368408
Irf8	8264.14461	-0.312826875	0.093817	-3.3344495	0.00085468	0.02302242
Eif4a2	3914.99595	-0.316700887	0.095155	-3.3282733	0.00087386	0.02340025
Wsb1	3758.20122	-0.318889228	0.100988	-3.1576879	0.00159026	0.03630684
Hlx	1595.39348	-0.325960058	0.099949	-3.2612631	0.00110917	0.02794454
Cited2	3197.75772	-0.329003314	0.101855	-3.2301227	0.00123737	0.03058664
Stard5	925.632352	-0.332016988	0.109572	-3.0301224	0.00244455	0.04871223
Ticam1	2162.5257	-0.332444004	0.106245	-3.1290247	0.00175388	0.03868176
Plekho1	13105.8262	-0.333427	0.08703	-3.8311888	0.00012753	0.0056618
Irak2	2882.23542	-0.333619698	0.104702	-3.1863864	0.00144062	0.03385458
Klhl9	2564.25858	-0.336135111	0.110243	-3.0490336	0.00229579	0.04691393
Cd48	5084.79929	-0.336369259	0.102307	-3.287845	0.00100957	0.02631069
Stard3	3249.28662	-0.337016763	0.100133	-3.3656963	0.00076351	0.02104513
Zfp36l2	20895.9806	-0.339980855	0.086549	-3.9281748	8.56E-05	0.00403679
Tfeb	3170.94847	-0.340141097	0.091969	-3.6984326	0.00021693	0.00864568
Pbx2	2730.67026	-0.346863082	0.103879	-3.3391062	0.00084048	0.02268476
Serf2	4917.30948	-0.34789941	0.104476	-3.3299352	0.00086866	0.02335279
Csnk1e	2488.41902	-0.350140042	0.099169	-3.5307321	0.00041441	0.01339384
Psen2	2608.47353	-0.350765287	0.096899	-3.6198985	0.00029472	0.01040677
Lmo2	2262.95246	-0.353593999	0.09678	-3.6535668	0.00025862	0.00975394
Fth1	131122.773	-0.356207068	0.085738	-4.1546079	3.26E-05	0.00193099
Ccdc88b	3612.6347	-0.359288792	0.097561	-3.6827119	0.00023077	0.00901243
Themis2	9396.43568	-0.361001236	0.096619	-3.736357	0.00018671	0.00761915
Nampt	5121.93825	-0.362202944	0.106532	-3.3999314	0.00067403	0.01958848
Snx5	17383.1899	-0.364641553	0.091059	-4.0044673	6.22E-05	0.00312257
Lgals1	32829.8584	-0.36472185	0.099747	-3.6564617	0.00025572	0.00970883
Marcks	8050.40035	-0.365261818	0.10842	-3.3689483	0.00075456	0.02096686
Resf1	4369.54198	-0.367465848	0.120737	-3.0435253	0.00233824	0.04749655
Maf	8645.99763	-0.370472807	0.119883	-3.0902879	0.00199963	0.04265243
Bri3	2013.01927	-0.372511654	0.103376	-3.6034615	0.00031401	0.0108489

Ptafr	7112.04298	-0.376173622	0.110718	-3.3975915	0.00067982	0.01967288
Paxbp1	1942.02622	-0.388066338	0.128006	-3.0316235	0.00243242	0.0486842
Pla2g15	4756.15052	-0.388461882	0.10649	-3.6478753	0.00026442	0.00977015
Arid5b	2185.23997	-0.389922445	0.123715	-3.1517923	0.00162272	0.03674024
Pde1b	2057.21261	-0.390115879	0.118852	-3.2823533	0.00102945	0.02667555
Tob2	3647.91922	-0.391013674	0.128576	-3.041118	0.00235701	0.04774454
Thbs1	53731.0359	-0.399250462	0.128708	-3.1019865	0.00192227	0.04145649
Rilpl2	2248.50046	-0.400860587	0.122154	-3.2815962	0.00103221	0.02669652
Psmb8	4366.44063	-0.401709377	0.127208	-3.1578958	0.00158912	0.03630684
Sertad1	1116.32274	-0.402040228	0.13084	-3.0727557	0.00212092	0.04488813
Otulin	1598.11502	-0.403702248	0.098688	-4.0907077	4.30E-05	0.00243224
Aatk	779.77476	-0.40377559	0.131425	-3.0722946	0.0021242	0.04488813
Ada	1226.36095	-0.404255534	0.10729	-3.767887	0.00016464	0.00686232
Hspa1b	11917.8055	-0.40425626	0.097485	-4.1468356	3.37E-05	0.00198047
Pex13	703.45568	-0.404357845	0.120099	-3.3668722	0.00076026	0.02101893
H3f3b	16284.4772	-0.412962387	0.117155	-3.5249276	0.0004236	0.01364929
Prr5l	546.076908	-0.413586819	0.127858	-3.2347404	0.00121753	0.03022766
Rsrp1	4724.92217	-0.414836596	0.111992	-3.70417	0.00021208	0.00850207
Zfand2a	2287.62268	-0.415181913	0.123248	-3.3686769	0.0007553	0.02096686
Sys1	1174.57541	-0.417805832	0.114527	-3.6481095	0.00026418	0.00977015
Uba7	2531.5423	-0.419056068	0.135279	-3.0977279	0.0019501	0.04185814
Rasgrp2	823.028012	-0.420803398	0.136647	-3.0794965	0.00207351	0.04402168
Tmem251	580.190319	-0.421848865	0.126894	-3.3244074	0.00088607	0.02368057
Arhgef18	661.381625	-0.422149536	0.128525	-3.2845654	0.0010214	0.02651753
Plgrkt	1029.25636	-0.422595273	0.138599	-3.0490391	0.00229575	0.04691393
Suco	921.395386	-0.423171618	0.11252	-3.7608719	0.00016932	0.00703616
Arhgap17	5681.10387	-0.424705533	0.111562	-3.8068923	0.00014072	0.00614765
Zfp703	4655.62686	-0.425585873	0.137134	-3.1034336	0.00191289	0.04131967
Jund	16495.3896	-0.428456816	0.134011	-3.1971682	0.00138784	0.03324474
Clk4	977.739055	-0.430483753	0.109876	-3.9179136	8.93E-05	0.00412683
Gpr34	1080.67983	-0.434325504	0.137845	-3.1508161	0.00162815	0.03677508
Sh2d1b1	761.366178	-0.436841619	0.139427	-3.1331278	0.00172954	0.03839355
Pnrc1	6243.51571	-0.448025605	0.12274	-3.6501917	0.00026204	0.00977015
Pkib	775.635689	-0.449297916	0.124392	-3.6119457	0.00030391	0.0106212
Bcl2a1b	2286.515	-0.451049393	0.110301	-4.0892555	4.33E-05	0.0024374
Sik1	5032.75838	-0.454222066	0.148944	-3.0496097	0.00229139	0.04691393
Cebpa	14644.9926	-0.456316773	0.08042	-5.6741993	1.39E-08	3.06E-06
Ubc	3728.93283	-0.459545093	0.140845	-3.2627822	0.00110324	0.02784666
Ms4a7	9399.34398	-0.45966209	0.10089	-4.5560713	5.21E-06	0.00044123
Epsti1	3621.94644	-0.463938224	0.140571	-3.3003923	0.0009655	0.02545403
Abtb1	1045.1585	-0.464195848	0.118086	-3.9309973	8.46E-05	0.00400354
Syngr1	2875.85535	-0.465399407	0.126475	-3.6797714	0.00023344	0.00905312
Rraga	2014.33527	-0.467123224	0.102823	-4.5429887	5.55E-06	0.00046377
Lilr4b	13955.9144	-0.467151751	0.123979	-3.7680048	0.00016456	0.00686232
Sft2d1	1306.02631	-0.468015953	0.133083	-3.516721	0.00043691	0.01403175
Gpr65	2809.5783	-0.468632717	0.115624	-4.0530739	5.05E-05	0.00273406
Klf6	16560.0913	-0.470546169	0.11634	-4.0445628	5.24E-05	0.00276937
Ddit3	586.774912	-0.474006035	0.131451	-3.6059548	0.00031101	0.01078634
Ccnl1	7372.53722	-0.476307232	0.108862	-4.3753471	1.21E-05	0.00088369
Sgk1	9454.92389	-0.476342129	0.155902	-3.0553915	0.00224767	0.04638156
Slfn2	9189.21218	-0.480776405	0.140666	-3.4178639	0.00063115	0.01865775
Irf1	2586.70364	-0.483687455	0.098073	-4.9319376	8.14E-07	9.73E-05
Trafd1	5328.06213	-0.484440023	0.113791	-4.2572651	2.07E-05	0.00133206
Rnf144b	1277.16539	-0.485599058	0.15382	-3.1569243	0.00159443	0.03630684
Rasa4	6033.64149	-0.486637205	0.098819	-4.9245396	8.46E-07	0.00010022
Cd24a	5551.29163	-0.488206482	0.134967	-3.617222	0.00029778	0.01048777
Ly86	7928.97405	-0.488375141	0.14742	-3.3128053	0.00092365	0.02449297
Stoml1	435.257326	-0.494913716	0.143138	-3.4576048	0.000545	0.01661824
Hspa1a	10764.7791	-0.501166025	0.09566	-5.2390183	1.61E-07	2.62E-05
Rn18s-rs5	10390.7663	-0.503322994	0.139461	-3.6090717	0.00030729	0.01068476
Sowahc	2437.87169	-0.505247993	0.140825	-3.5877767	0.00033351	0.01142145
Clk1	3802.7436	-0.506200224	0.100442	-5.0397174	4.66E-07	6.42E-05
AU022793	351.274968	-0.508746408	0.158712	-3.2054747	0.0013484	0.03258631
Ptger4	1780.89857	-0.509711782	0.122557	-4.1589907	3.20E-05	0.00190824
Cyp27a1	2457.45182	-0.513379338	0.153218	-3.350639	0.00080625	0.02197847
Padi4	1135.00809	-0.515695453	0.156438	-3.2964762	0.00097906	0.02571209
Oas2	5867.71285	-0.52017106	0.128729	-4.0408204	5.33E-05	0.00279229
Zfp622	1285.75672	-0.52108883	0.108241	-4.8141651	1.48E-06	0.00015864
Trip6	454.433597	-0.521632628	0.153935	-3.3886654	0.00070234	0.02006886
Mafk	1588.10733	-0.523920927	0.145882	-3.5914098	0.00032889	0.01129175
Sem1	3698.30559	-0.52560458	0.131263	-4.0041995	6.22E-05	0.00312257
Basp1	8035.17496	-0.525861491	0.115412	-4.5563833	5.20E-06	0.00044123
Id2	3776.53762	-0.527981602	0.12017	-4.3936333	1.11E-05	0.00082574
Amdhd2	3531.1817	-0.531664635	0.111033	-4.7883552	1.68E-06	0.00017906
Pim1	14125.9639	-0.53545888	0.147286	-3.6354952	0.00027745	0.01001109
Hpgds	1920.30667	-0.535710646	0.163963	-3.2672704	0.0010859	0.02745975
Fcrls	4921.02589	-0.539814057	0.17341	-3.1129395	0.00185234	0.04020285
Cxcr4	9255.31988	-0.542234649	0.102503	-5.2899483	1.22E-07	2.06E-05
Zc3h12a	2635.02794	-0.547080365	0.151044	-3.6220033	0.00029233	0.01035623
Cpeb2	2356.03134	-0.547979107	0.138792	-3.9481912	7.87E-05	0.00377915
Gas6	2823.32228	-0.547984897	0.15635	-3.5048509	0.00045686	0.01448149
Aoah	1057.78647	-0.548553872	0.180548	-3.0382668	0.00237943	0.0480469
Snrpd2	1385.73448	-0.549567952	0.155397	-3.5365404	0.0004054	0.01321929
Nod1	1375.76869	-0.552043482	0.157232	-3.5110091	0.00044641	0.01421625
Hmgb1-ps5	277.404019	-0.554310001	0.170568	-3.2497832	0.00115493	0.02888385
Gm39701	893.59768	-0.562366201	0.179897	-3.1260412	0.00177177	0.03901323
F10	4107.10479	-0.56362365	0.167301	-3.3689199	0.00075463	0.02096686
Pfkfb3	3320.29448	-0.569202441	0.161873	-3.516348	0.00043753	0.01403175
H2-Q4	2995.92976	-0.570406172	0.115062	-4.957401	7.14E-07	8.85E-05

Rabgef1	1747.25284	-0.572912984	0.164852	-3.4753079	0.00051027	0.01580669
Flcn	2500.7302	-0.579658789	0.112456	-5.1545495	2.54E-07	3.89E-05
Paox	687.70115	-0.580001638	0.180419	-3.214742	0.00130562	0.03172116
Chaserr	937.687824	-0.580883518	0.14344	-4.0496495	5.13E-05	0.00275104
Otud1	454.317724	-0.580886225	0.176853	-3.2845644	0.0010214	0.02651753
Ppp1r15a	5152.5138	-0.581021232	0.16373	-3.5486632	0.00038719	0.01284045
Sde2	1060.15088	-0.582988892	0.118069	-4.9376956	7.91E-07	9.54E-05
Pparg	1677.04655	-0.583976646	0.158502	-3.6843545	0.00022928	0.00898025
Irf2bp2	7364.29605	-0.590048462	0.094285	-6.2581623	3.90E-10	1.26E-07
Zup1	929.47054	-0.590234166	0.133034	-4.436724	9.13E-06	0.0006994
Rgs2	9462.35582	-0.59135725	0.092331	-6.4047603	1.51E-10	5.87E-08
Zfp991	394.72868	-0.593920925	0.181636	-3.2698385	0.00107609	0.0273797
Sat1	10348.9753	-0.593969591	0.096298	-6.1680049	6.92E-10	2.19E-07
Chka	681.616482	-0.59728436	0.129416	-4.6152248	3.93E-06	0.0003409
Gm35853	287.14021	-0.599051881	0.190142	-3.1505468	0.00162965	0.03677508
Map3k8	1402.96129	-0.606949803	0.135225	-4.4884502	7.17E-06	0.00057067
Tiparp	2327.1678	-0.60757221	0.151686	-4.005449	6.19E-05	0.00312257
Gbp9	1694.55021	-0.609276065	0.177156	-3.4392141	0.00058341	0.01759252
Fos	38309.0125	-0.609581977	0.146168	-4.1704072	3.04E-05	0.00182567
Adrb2	495.829854	-0.613386657	0.152249	-4.02883	5.61E-05	0.00291615
Lst1	3393.95347	-0.616589838	0.152975	-4.0306612	5.56E-05	0.00290461
Dusp10	261.896416	-0.618169317	0.180488	-3.4249912	0.00061482	0.01829691
Hilpda	690.428397	-0.618494108	0.181062	-3.4159242	0.00063566	0.01871283
Tgif1	4715.85137	-0.626701921	0.10693	-5.8608841	4.60E-09	1.16E-06
Tagap	822.462193	-0.629816694	0.111049	-5.6715066	1.42E-08	3.06E-06
Zfand5	4459.94415	-0.632554344	0.100879	-6.2704142	3.60E-10	1.20E-07
Tsc22d3	4050.76447	-0.633901119	0.155881	-4.0665836	4.77E-05	0.00262644
Card11	1408.43896	-0.63469365	0.174997	-3.6268738	0.00028687	0.01026269
Fabp5	15724.4955	-0.637507329	0.137419	-4.6391538	3.50E-06	0.00031165
Ramp1	598.756702	-0.638774647	0.169964	-3.7582933	0.00017108	0.00707491
Polr2k	170.829979	-0.642016021	0.204443	-3.1403164	0.00168765	0.03764768
Siglecf	639.814086	-0.644201235	0.160895	-4.0038725	6.23E-05	0.00312257
Cd300lf	3648.52737	-0.644465889	0.126293	-5.1029342	3.34E-07	4.75E-05
Gab3	539.500269	-0.645914569	0.164806	-3.9192452	8.88E-05	0.00411805
Tnfrsf12a	426.603035	-0.653185557	0.160454	-4.0708604	4.68E-05	0.00260582
Zfp36	79129.1868	-0.659635195	0.142598	-4.6258499	3.73E-06	0.00032595
Rhou	528.504827	-0.662840118	0.181444	-3.6531375	0.00025906	0.00975394
Gm13391	224.413013	-0.66569968	0.21088	-3.1567731	0.00159525	0.03630684
Cd83	6452.91038	-0.669832936	0.175172	-3.8238608	0.00013138	0.00579509
Slc2a3	899.649562	-0.670231067	0.220394	-3.0410621	0.00235745	0.04774454
Gm26947	284.738094	-0.672566534	0.213755	-3.1464373	0.00165273	0.03711545
Rab20	871.055612	-0.673038021	0.139564	-4.822424	1.42E-06	0.00015716
Junb	68761.5571	-0.673557727	0.126702	-5.31608	1.06E-07	1.83E-05
Zbtb16	225.628505	-0.681920951	0.201965	-3.3764343	0.00073432	0.02067927
Ankrd55	243.459323	-0.682286645	0.204974	-3.3286572	0.00087266	0.02340025
Ptp4a1	1518.69464	-0.682445797	0.134552	-5.0719907	3.94E-07	5.48E-05
Fabp4	2881.28127	-0.685448889	0.173733	-3.9454059	7.97E-05	0.00378339
Bambi	512.446147	-0.685487928	0.178154	-3.8477182	0.00011922	0.00538083
Abhd15	152.677822	-0.688222719	0.213493	-3.2236364	0.00126574	0.03092024
Nfil3	3415.87786	-0.689002512	0.163326	-4.2185725	2.46E-05	0.00150269
Tusc1	727.060394	-0.693965214	0.139283	-4.9824021	6.28E-07	8.08E-05
Dedd2	888.32128	-0.694696036	0.185128	-3.7525133	0.00017507	0.00718738
Dusp1	36375.2985	-0.695620307	0.146292	-4.755027	1.98E-06	0.00020334
Rps4l	272.328079	-0.699777966	0.195238	-3.5842235	0.00033808	0.01153697
Hba-a2	573.882644	-0.708614881	0.230747	-3.0709664	0.00213367	0.04494891
Rabepk	601.405238	-0.70910274	0.176813	-4.0104779	6.06E-05	0.00310497
Rhob	12355.414	-0.71131535	0.119068	-5.974038	2.31E-09	6.19E-07
Retnla	472.807092	-0.712159294	0.164626	-4.3259251	1.52E-05	0.00103515
Cela1	138.230608	-0.717551426	0.225792	-3.1779246	0.00148333	0.03461957
Icam2	303.990398	-0.726779234	0.212675	-3.4173154	0.00063242	0.01865775
Tent5c	1594.70396	-0.734228279	0.164418	-4.4656226	7.98E-06	0.00062538
Slc26a11	1146.2355	-0.73724019	0.176373	-4.1800024	2.92E-05	0.00176588
Ptpn18	2676.97496	-0.742992697	0.121094	-6.1356507	8.48E-10	2.51E-07
Maff	708.605791	-0.750940742	0.205728	-3.6501558	0.00026208	0.00977015
Aif1	1985.47849	-0.752638047	0.146359	-5.1423965	2.71E-07	4.06E-05
Arl5c	1012.48906	-0.75456559	0.131088	-5.7561921	8.60E-09	2.02E-06
Gadd45g	1521.47703	-0.760206219	0.135045	-5.6292811	1.81E-08	3.79E-06
Siglecg	418.288529	-0.760606348	0.206828	-3.6774792	0.00023555	0.00909355
Chst15	375.562036	-0.763930408	0.162396	-4.7041239	2.55E-06	0.00025001
0610042G04	201.848123	-0.767687978	0.237575	-3.2313532	0.00123206	0.03053258
Gm10382	419.178799	-0.787512055	0.194685	-4.0450589	5.23E-05	0.00276937
Nr1d2	401.05284	-0.793996358	0.178213	-4.455317	8.38E-06	0.00064507
Pkd1l3	262.785797	-0.798741679	0.263133	-3.0355094	0.0024013	0.04827386
Mmp27	289.99646	-0.806708484	0.232483	-3.4699611	0.00052053	0.01608815
F5	1698.71924	-0.814414834	0.174147	-4.6765868	2.92E-06	0.00027802
Osgin1	730.653716	-0.843459343	0.215025	-3.922608	8.76E-05	0.00410285
H2-K2	633.238194	-0.85354789	0.157755	-5.4105948	6.28E-08	1.17E-05
Cebpb	29941.9705	-0.85979521	0.145084	-5.9262032	3.10E-09	7.97E-07
4930430E12I	3038.63232	-0.880742319	0.127166	-6.9259165	4.33E-12	2.57E-09
Gm26944	107.401372	-0.88445626	0.289062	-3.059751	0.00221521	0.04602641
Dusp2	1161.76476	-0.886456465	0.162785	-5.4455702	5.16E-08	9.78E-06
Cd69	334.795466	-0.887317984	0.209695	-4.2314601	2.32E-05	0.00142599
Cd28	328.592377	-0.893670274	0.185574	-4.815696	1.47E-06	0.00015864
Taf7	418.089767	-0.893720619	0.204606	-4.3680025	1.25E-05	0.00090906
Ier5	10174.6693	-0.910307364	0.111816	-8.1411384	3.92E-16	4.85E-13
Gm36587	165.392856	-0.91202571	0.215302	-4.2360292	2.28E-05	0.00140951
Il2rb	225.875593	-0.928156003	0.24967	-3.7175351	0.00020118	0.0081608
Cd55	110.658206	-0.941386898	0.291976	-3.2241966	0.00126327	0.03092024
Pmaip1	1107.49399	-0.941763741	0.136306	-6.9091902	4.87E-12	2.77E-09

Cd274	1635.7048	-0.964042881	0.129583	-7.4395638	1.01E-13	7.65E-11
Ikzf2	80.9900865	-0.969721927	0.283089	-3.4255074	0.00061365	0.01829691
Ccl3	4963.57361	-0.9697783	0.153921	-6.3004869	2.97E-10	1.01E-07
Gadd45b	7975.52626	-0.970253355	0.152757	-6.351609	2.13E-10	7.92E-08
Pim3	1178.95538	-0.971169313	0.16821	-5.773566	7.76E-09	1.86E-06
Id3	364.209062	-0.971643629	0.186084	-5.2215349	1.77E-07	2.81E-05
Fcrl1	234.568255	-0.974831685	0.255285	-3.818604	0.00013421	0.00590087
LOC1185679	714.429819	-0.977146864	0.14626	-6.6808721	2.38E-11	1.04E-08
Ifi27l2a	8369.08111	-0.983567792	0.195349	-5.0349222	4.78E-07	6.52E-05
Cd5l	798.071268	-0.988009915	0.201796	-4.8960749	9.78E-07	0.0001139
Plk2	5319.62271	-0.98918429	0.279766	-3.5357596	0.0004066	0.01322678
Ccl5	253.402854	-0.997334895	0.25995	-3.8366405	0.00012473	0.00555573
H1f4	169.599866	-0.999371027	0.225991	-4.4221788	9.77E-06	0.00073989
Rgs1	14915.9498	-1.002187209	0.165933	-6.0396941	1.54E-09	4.30E-07
Wfdc17	3774.20142	-1.004791051	0.106275	-9.4546147	3.24E-21	1.10E-17
Snhg15	143.5109	-1.0126782	0.247703	-4.0882693	4.35E-05	0.00243771
LOC1185683	91.1366043	-1.013183723	0.254483	-3.9813451	6.85E-05	0.00337729
Hip1r	267.613058	-1.014489784	0.280104	-3.6218289	0.00029253	0.01035623
Vegfa	1763.10632	-1.019152368	0.188479	-5.4072567	6.40E-08	1.18E-05
LOC1185679	84.8537389	-1.035846988	0.292718	-3.5387254	0.00040206	0.01319002
Rflnb	605.810458	-1.053069586	0.182181	-5.7803371	7.46E-09	1.81E-06
Jun	32586.5374	-1.058859111	0.157572	-6.7198244	1.82E-11	8.55E-09
Gbp4	269.134667	-1.061303578	0.261586	-4.057195	4.97E-05	0.00270777
Depp1	62.8978101	-1.06969414	0.31657	-3.3790173	0.00072745	0.02057096
Atf3	16042.3373	-1.07112259	0.153441	-6.9806967	2.94E-12	2.00E-09
S1pr4	119.419915	-1.074939837	0.327997	-3.2772873	0.0010481	0.02700484
Ifrd1	5448.15684	-1.075083404	0.167153	-6.4317359	1.26E-10	5.06E-08
Gm8818	79.7946887	-1.084530875	0.302617	-3.5838429	0.00033858	0.01153697
H2-Q5	196.175595	-1.095653133	0.277586	-3.947074	7.91E-05	0.00378161
Sema7a	723.305527	-1.104202485	0.160651	-6.8732932	6.27E-12	3.29E-09
Ccrl2	9875.52451	-1.106856622	0.163136	-6.7848825	1.16E-11	5.86E-09
Clec2g	95.4410829	-1.124770597	0.270487	-4.1583134	3.21E-05	0.00190824
Nr1d1	211.912946	-1.139786313	0.267744	-4.2570011	2.07E-05	0.00133206
Vnn3	66.1793568	-1.167620362	0.371946	-3.1392188	0.00169399	0.03772724
Ccr3	363.380205	-1.196903297	0.219534	-5.4520288	4.98E-08	9.70E-06
Peg10	74.4790804	-1.199991895	0.330529	-3.6305166	0.00028285	0.01014555
Per1	5339.04002	-1.21900448	0.16157	-7.544768	4.53E-14	4.12E-11
Prkcq	84.6837264	-1.228285375	0.284998	-4.3098039	1.63E-05	0.00108641
Serpina3g	129.650187	-1.238558797	0.241831	-5.1215846	3.03E-07	4.35E-05
Igk	100.02462	-1.24631477	0.328681	-3.7918682	0.00014952	0.00638906
Rnf125	132.219633	-1.283150332	0.259536	-4.9440099	7.65E-07	9.40E-05
Ikzf3	134.716013	-1.326838055	0.275568	-4.8149252	1.47E-06	0.00015864
Gbp8	123.653414	-1.336291446	0.303938	-4.3965866	1.10E-05	0.00081904
Ccl4	3450.63313	-1.340187954	0.123452	-10.855974	1.87E-27	2.55E-23
Gm36043	166.795754	-1.342498394	0.276741	-4.8510954	1.23E-06	0.00013717
Ccl8	277.761423	-1.348042004	0.179223	-7.5216027	5.41E-14	4.61E-11
Nkg7	60.4531011	-1.360410804	0.433027	-3.1416274	0.00168012	0.03757953
Gm6969	146.547425	-1.375809824	0.365048	-3.7688441	0.00016401	0.00686232
Slc36a3	31.2249784	-1.37841148	0.450003	-3.063113	0.00219047	0.04565161
Tstd1	33.9550601	-1.382928618	0.436057	-3.1714408	0.00151685	0.03518418
Gm8091	58.4018204	-1.389269831	0.414044	-3.3553677	0.0007926	0.02169293
Rn7sk	122.703427	-1.390701794	0.299077	-4.6499746	3.32E-06	0.00030166
Calhm6	300.596483	-1.424348515	0.250381	-5.6887278	1.28E-08	2.91E-06
Il10	495.739616	-1.434053787	0.438874	-3.267574	0.00108474	0.02745975
Septin1	179.226334	-1.528412138	0.298108	-5.127049	2.94E-07	4.27E-05
Gdf15	2851.53021	-1.548632418	0.298868	-5.1816674	2.20E-07	3.45E-05
Cd300ld5	76.1681074	-1.556200703	0.510509	-3.0483296	0.00230117	0.04695359
Tesc	36.0980425	-1.694074487	0.558774	-3.0317724	0.00243122	0.0486842
Alox15	1715.32306	-1.723844489	0.192763	-8.9427986	3.79E-19	7.39E-16
P2rx1	159.74922	-1.795180628	0.268353	-6.6896303	2.24E-11	1.02E-08
Il5ra	66.953906	-1.8074212	0.382868	-4.7207373	2.35E-06	0.00023551
Ccl16-ps	29.3123576	-1.844339292	0.532457	-3.4638253	0.00053255	0.01637984
Gm41590	105.126674	-1.861551655	0.413318	-4.5039174	6.67E-06	0.00053804
9830107B12	92.7905219	-1.865375073	0.416194	-4.4819862	7.40E-06	0.00058264
Eomes	34.8582119	-1.902337519	0.526089	-3.6159979	0.00029919	0.0105103
Fam71a	563.016432	-1.956480584	0.211142	-9.2661709	1.93E-20	4.38E-17
Rab37	58.1317647	-1.989265788	0.412786	-4.8191163	1.44E-06	0.0001585
Gm40543	31.8820994	-2.148885835	0.492643	-4.3619502	1.29E-05	0.00092964
Inpp5j	379.073234	-2.251671368	0.223003	-10.097055	5.69E-24	2.59E-20
Col22a1	54.4333921	-2.38734685	0.455056	-5.2462736	1.55E-07	2.55E-05
Sytl1	107.996529	-2.40310244	0.321139	-7.4830486	7.26E-14	5.82E-11
Gata1	30.404393	-2.491240362	0.630353	-3.9521375	7.75E-05	0.00374372
F730016J06R	27.0191641	-2.553878054	0.585862	-4.3591828	1.31E-05	0.0009345
Pla2g3	39.5705806	-2.6352167	0.542511	-4.8574408	1.19E-06	0.00013395
Fcnb	141.146508	-3.494595976	1.019467	-3.4278664	0.00060834	0.01818364
Itgb2l	61.025822	-5.177918938	1.512908	-3.4224946	0.00062049	0.01838548
Ltf	3218.12916	-5.322767774	1.358053	-3.9194122	8.88E-05	0.00411805
Prg2	119.418301	-5.421875332	1.553753	-3.489535	0.00048386	0.01523103
Mpo	282.704093	-6.377051774	1.66584	-3.8281307	0.00012912	0.00571399

Supplemental Table 2. Transcripts per million

RNA Sequencing was performed on Myeloid cells (CD45+,CD11b+) isolated from quadriceps muscles of 4.5 week old mdx mice 7 days after treatment with either prednisolone (Prd), Spironolactone (Spr), or Vehicle (Veh).
Table is sorted by Largest to smallest TPM for Veh3

	Prd1	Prd2	Prd3	Spr1	Spr2	Spr3	Veh1	Veh2	Veh3
COX1	20774.24	33745.47	20715.04	33398.25	29777.56	32870.82	19377	26296.53	24290.87
Ftl1	16660.68	14362.87	16312	18070.69	18179.54	18379.46	19218.16	16392.93	16928.61
Lyz2	17615.51	20931.81	19727.12	14667.42	16111.67	14569.78	14624.06	17721.43	16596.69
Ctsb	15284.93	13732.88	15879.95	16988.66	16081.2	15252.48	19366.03	14017.2	13917.41
Spp1	14073.26	13870.16	14573.9	10219.48	7613.522	6367.048	9512.558	9335.806	12713.24
CYTB	11351.96	18596.59	11345.62	20454.34	16829.42	19397.22	8922.111	12838.14	12296.57
ND1	10674.64	16302.31	8890.645	17989.14	14403.7	17039.68	8241.573	11259.71	10928.78
Tmsb4x	6852.145	6753.772	7162.685	7903.148	9038.648	8741.625	7164.218	8641.514	8898.607
Psap	9424.773	8369.474	9384.355	9785.211	9739.44	9245.864	11208.24	8241.673	8566.039
Fth1	6974.544	6381.471	7233.448	7898.627	7564.157	6981.018	8424.631	8394.15	8410.626
Apoe	9119.259	6085.799	9065.386	7878.245	9799.341	12400.48	8928.211	9783.605	6767.04
Ctsd	6000.758	5640.58	6342.655	7221.044	6870.787	6976.911	7526.363	5955.388	6209.869
ND2	5875.313	9887.939	4835.797	11334.76	8832.576	10342.68	4304.526	7052.409	6138.657
Vim	7637.606	6826.564	6516.332	5493.695	5063.829	4968.352	5900.226	5674.806	5976.013
Ctss	4745.037	5386.64	6259.936	5707.001	5797.716	5629.43	5227.604	6064.446	5796.632
B2m	4874.608	4522.541	5979.864	5168.109	5583.422	5941.579	4874.337	6292.741	5725.406
Cd68	6436.407	5325.567	5276.026	6793.637	5924.354	5955.214	7111.577	5993.375	5655.291
ND6	4478.306	7689.211	3995.102	8456.499	6817.022	7117.77	4508.437	6043.644	5596.59
Eef1a1	5783.769	5752.155	6087.9	5240.718	5341.98	4921.497	5919.204	5523.671	5595.569
ND4	4827.255	8233.345	5037.403	8826.045	7570.354	9045.382	4091.568	5817.256	5548.119
Lgals3	4762.809	3914.557	3990.235	5606.018	4463.492	5083.204	5106.669	4387.222	4911.962
Actb	5227.607	4121.933	4873.428	4164.339	4275.834	4073.487	4697.84	4192.248	4798.902
Gpnmb	6066.235	6869.035	7086.707	5977.464	4496.295	4158.095	6285.378	4332.09	4579.574
Tpt1	3494.954	3786.841	3573.256	3707.419	3854.298	4069.736	3540.94	4190.775	4307.666
ND5	3679.154	6151.611	3469.598	6502.298	5017.662	5394.346	3515.032	4481.562	4255.525
Fcer1g	3358.047	2959.973	3439.329	3434.411	3930.527	3464.756	3417.33	4078.769	3634.619
Tmsb10	3041.636	2674.689	3493.298	3773.943	3452.935	4137.686	3175.222	3889.554	3506.065
S100a9	634.3987	668.7918	464.6271	800.0369	935.3764	2328.189	272.8529	478.1828	3436.966
Rps26	3446.812	3214.499	3206.004	3060.07	3742.337	3735.882	3191.294	3646.35	3271.201
Tyrobp	2212.798	2350.374	2584.678	2666.159	2918.523	2870.974	2199.247	2918.733	2935.095
Zfp36	2556.684	1947.446	1604.278	2154.721	2309.139	2138.649	3502.158	2926.661	2866.541
Cfl1	3227.769	2788.993	2999.295	2827.195	3062.943	2992.734	3087.366	2935.624	2830.014
Rpl41	2517.802	2554.444	2751.974	2653.242	3010.226	3326.214	2486.402	2903.036	2827.095
S100a8	433.0392	454.877	292.7809	693.0317	698.504	1887.346	189.2881	415.3926	2787.982
S100a11	2805.122	2718.229	2887.845	2135.973	2609.341	2565.912	2188.011	3039.181	2765.786
Ctsc	2609.279	2455.504	3267.812	2283.068	2886.968	2168.926	2324.43	2892.936	2703.675
Junb	1872.481	1663.702	1362.971	1811.329	2036.821	2039.16	2530.018	2377.735	2588.516
Hspa8	2747.041	2843.178	2800.828	2286.378	2473.771	2596.404	2466.333	2617.051	2527.342
Cd74	3255.932	2809.823	2947.807	2955.746	4809.767	4527.166	3583.116	3356.758	2480.204
Rplp1	2340.007	2435.734	2380.797	2440.1	2630.228	2683.76	2307.224	2532.656	2387.054
Cxcl2	1621.115	1315.586	841.9067	800.7489	1166.542	1093.42	1624.713	1856.647	2371.731
Lgals1	2029.934	1839.376	1807.214	2576.109	2220.181	2588.682	2381.52	2257.77	2367.38
C1qb	2027.306	2269.313	2716.279	2975.388	3452.904	3383.229	3118.278	2844.229	2302.064
Rps27	1571.813	1733.021	1566.708	1835.938	2003.6	2047.287	1366.044	2338.599	2216.117
mt-Rnr2	1577.742	2433.794	1378.718	3211.718	2607.824	3377.782	1510.683	2154.672	2212.257
Lilrb4a	2059.178	1854.535	1887.481	1782.284	1673.276	1614.228	2087.08	2042.132	2180.203

H2-D1	1828.147	1934.498	2287.757	2124.188	2144.517	2205.797	1996.123	2407.927	2121.014
Rpl35	1851.533	1764.28	1691.425	1833.6	2039.638	2300.227	1743.152	2094.366	2064.516
Rps15	1829.456	1856.256	1941.089	1980.683	2132.882	2307.523	1647.702	2032.631	2054.383
Cst3	1943.048	2028.313	2203.571	2022.543	2344.762	2350.571	1999.384	2189.942	2011.538
Ngp	3.056017	39.00336	0.429802	358.685	273.5432	1535.99	117.0636	16.23027	1998.992
Fxyd5	2265.389	2070.561	2598.265	1874.174	1920.942	1891.041	1796.865	2184.216	1990.088
Grn	2239.176	2090.639	2507.986	2265.284	2176.222	2097.285	2724.99	2124.421	1975.474
S100a4	1857.675	1983.698	1631.891	1425.803	1441.434	1616.104	1315.443	1841.04	1891.346
Lgmn	1835.813	1592.684	2070.112	2242.334	2136.077	1996.475	2622.119	1888.065	1867.373
Ahnak	2997.446	2650.635	2715.517	2167.826	1747.669	1204.564	2546.632	1748.137	1852.269
Ucp2	2409.621	2416.327	2350.305	1687.616	2108.561	1918.493	2085.669	2220.112	1841.598
Pfn1	2106.455	1718.39	1830.433	1665.675	1964.351	1903.847	1873.118	1977.947	1823.983
Gpx1	1938.859	2084.233	1911.038	1658.512	1708.969	1715.087	1737.086	1783.273	1781.086
Crip1	2248.404	2513.799	2228.571	1770.001	1751.921	2096.343	1717.915	1989.439	1767.275
Ccl6	2002.256	2077.089	1910.017	1259.142	1701.799	1454.837	1482.167	2104.977	1725.641
Cyba	1440.979	1485.716	1493.223	1614.869	1777.709	1764.813	1545.773	1658.542	1723.282
Rpl10	1642.431	1504.735	1500.07	1480.677	1760.817	1685.246	1532.127	1708.027	1703.624
Ifitm3	1287.823	944.5456	1330.497	1177.33	1381.889	1283.583	891.656	1694.415	1653.044
Cd52	1118.24	1197.924	1357.233	1272.536	1519.403	1472.893	1071.006	1770.259	1630.081
Rpl19	1340.354	1392.301	1363.123	1536.325	1544.61	1589.914	1416.068	1697.478	1625.502
mt-Rnr1	1105.278	1689.515	1175.365	2471.041	1965.822	2599.713	1022.124	1611.938	1613.781
Pkm	1625.706	1375.209	1480.887	1206.767	1379.935	1381.025	1401.123	1428.21	1609.894
Gnai2	1665.509	1504.703	1644.755	1437.405	1651.774	1619.71	1553.895	1626.511	1584.614
Cebpb	915.7506	758.9874	722.0204	910.621	1023.695	1140.649	1239.607	1331.071	1582.894
Sh3bgrl3	1888.606	1657.587	1972.678	1585.217	1731.441	1830.552	1043.374	1742.974	1540.129
Myl6	1131.126	1174.621	1166.062	1345.215	1409.69	1541.056	1206.114	1424.387	1536.153
Itm2b	1375.611	1503.402	1809.294	1653.795	1863.12	1626.455	1742.453	1760.513	1532.461
Rpl26	1086.913	1206.717	1007.701	1348.543	1430.132	1578.431	970.0563	1558.006	1524.305
Actg1	2102.114	1650.339	1836.207	1206.969	1397.226	1335.908	1466.384	1446.898	1523.755
Dusp1	992.6896	875.7077	694.9692	894.332	797.4982	944.3547	1296.316	1162.003	1519.905
Ly6e	1411.229	1088.262	1764.169	1358.818	1515.627	1303.951	1154.787	1905.798	1513.093
Fau	1305.935	1378.158	1350.377	1469.17	1613.76	1788.451	1323.002	1682.338	1501.841
Rplp0	1571.221	1483.37	1500.385	1309.522	1473.896	1564.56	1359.943	1500.419	1501.165
Ifi27l2a	647.2042	614.8351	808.363	1126.613	1199.857	1117.582	827.0497	1551.902	1490.796
Selenop	1275.075	1482.855	1736.39	1969.106	2461.594	1875.038	2504.42	1824.309	1481.624
Rps16	1423.992	1373.991	1352.485	1285.95	1622.076	1615.548	1343.196	1551.032	1475.753
Ctsl	1724.987	1516.912	1519.943	1563.377	1077.439	1222.466	1496.233	1285.838	1467.456
Rpl38	1138.841	1165.112	1094.548	1402.709	1513.838	1624.384	1157.167	1506.382	1464.613
Uba52	1467.886	1421.697	1390.699	1402.712	1549.503	1678.724	1377.891	1520.989	1454.68
Rps24	1174.252	1286.867	1211.795	1319.679	1411.271	1500.152	1114.484	1485.299	1454.649
Anxa2	1545.77	1355.22	1309.079	1270.621	1237.731	1266.536	1316.144	1307.768	1440.377
Rpl18a	1270.695	1307.999	1271.358	1376.523	1509.484	1587.581	1229.888	1561.56	1411.385
Calr	1604.537	1353.236	1349.273	1449.857	1413.035	1443.772	1494.231	1341.81	1406.84
Lamp1	1561.543	1489.717	1657.937	1623.405	1529.525	1518.586	1861.941	1561.357	1395.533
C1qc	1324.465	1453.447	1761.547	1830.14	2063.554	2012.464	2121.711	1689.54	1376.83
Rps14	953.9618	1172.677	966.6104	1127.021	1256.465	1220.713	991.2459	1305.592	1374.746
Rpl32	1277.385	1238.112	1274.234	1320.107	1443.057	1541.582	1255.44	1501.996	1364.232
Fabp5	920.2718	702.5434	771.034	1436.92	1129.146	1308.764	1108.643	1092.462	1357.244
C1qa	1095.952	1345.521	1607.737	1406.669	1911.729	1818.76	1720.052	1484.495	1345.889
Capg	1293.63	1234.715	1216.393	1377.515	1224.816	1336.208	1310.389	1302.326	1342.104
Arhgdia	1624.525	1390.177	1600.397	1348.828	1443.19	1367.322	1505.97	1369.13	1313.512
Ppia	1252.187	1227.582	1206.55	1180.734	1250.888	1249.797	1156.713	1185.766	1307.407
Plin2	1188.796	1035.202	1117.658	1279.191	1017.469	1093.5	1312.867	1087.685	1300.527
Rpsa	1232.238	1329.705	1299.982	1186.619	1270.685	1320.473	1234.355	1322.199	1292.969

Rpl12	995.2783	1074.091	987.5727	1144.544	1170.176	1278.872	1064.773	1201.008	1291.038
Srgn	1113.164	987.4938	998.1012	624.8615	1120.541	940.0254	779.3666	1162.596	1285.742
H2-Ab1	1329.956	1171.667	1220.012	1541.861	2309.861	2229.895	1517.268	1468.353	1271.375
Mpeg1	1203.066	1166.469	1438.195	1344.814	1289.552	1124.92	1497.144	1299.696	1271.076
Ctsz	1354.113	1191.202	1354.386	1310.963	1231.821	1334.234	1430.421	1292.217	1262.887
Akr1a1	1260.922	1130.483	1212.721	1430.002	1274	1271.823	1421.59	1197.199	1254.683
Cotl1	1375.365	1189.234	1350.255	1269.951	1373.827	1377.119	1578.929	1316.341	1252.978
Rps19	1062.006	1048.92	1102.744	1056.057	1156.353	1334.311	1040.338	1219.084	1239.96
Ms4a6c	1085.423	945.2605	1144.579	1031.186	1065.834	967.0089	940.8892	1176.003	1229.381
Fos	1022.358	860.5808	697.5913	1031.191	735.4503	793.8301	1454.076	1124.675	1216.037
Rps9	1190.065	1214.66	1205.267	1082.868	1306.296	1311.074	1128.192	1286.751	1205.79
Il1b	1363.908	1254.905	822.5777	534.2217	1091.106	740.5627	642.3497	1024.207	1205.343
Aldoa	1312.822	1260.049	1370.035	1063.409	1174.366	1222.911	1183.67	1154.724	1196.934
H2-K1	948.2086	1010.748	1322.91	1273.617	1295.942	1220.306	1241.707	1432.642	1196.495
Rps18	1086.344	1101.479	1046.578	1086.785	1164.166	1222.853	986.4859	1214.285	1194.375
Tpm3	1452.151	1299.123	1340.617	1138.473	1225.615	1149.562	1250.335	1284.004	1193.027
Arhgdib	1216.636	1092.82	1188.8	969.5462	1182.063	1156.58	1114.121	1152.911	1180.449
Cd14	1104.856	955.5831	725.3183	516.0282	708.4422	651.1976	884.0313	1009.636	1172.729
Cstb	982.5061	915.8747	873.3891	1028.65	928.7994	1038.974	920.2897	1066.083	1163.388
Rps29	996.0372	938.9456	1008.237	1245.741	1256.065	1314.417	969.5116	1204.794	1163.371
Ctsa	1182.695	1152.69	1317.039	1272.675	1278.52	1241.697	1422.023	1202.958	1147.88
H2-Aa	1342.8	1184.481	1092.375	1368.658	2155.896	1988.747	1452.413	1377.211	1144.061
Rps17	958.9381	972.0158	887.9309	1103.248	1150.208	1195.59	882.4376	1086.049	1141.801
Laptm5	1387.65	1228.977	1439.216	1089.088	1368.568	1178.131	1302.202	1343.397	1135.326
Rack1	986.1698	1005.061	1003.837	983.5471	1054.288	1104.293	968.4916	1106.033	1129.262
Rpl18	1132.52	1018.192	1084.067	1022.823	1194.15	1211.692	1056.258	1177.617	1127.561
Pld4	1142.779	1219.971	1456.286	1227.51	1434.777	1302.676	1310.936	1474.143	1121.359
Eef2	1382.965	1320.675	1433.777	1049.699	1132.377	1157.535	1217.694	1163.377	1115.807
Gabarap	1008.854	990.2514	908.015	1072.432	1193.757	1106.224	988.314	1134.823	1099.178
Rpl13	1149.487	1080.645	1082.803	1119.23	1238.533	1294.436	1058.764	1242.892	1097.324
Rpl8	1113.649	982.6566	960.9523	970.3596	1062.71	1086.904	1022.856	1137.334	1093.464
Arpc2	1167.962	988.921	1086.832	961.4877	1003.402	952.0557	1052.215	1051.953	1065.332
Ctsh	926.5996	931.2256	1147.701	1109.622	1301.032	1290.747	1211.245	1216.823	1062.138
Ccl2	1065.222	728.1055	797.2727	858.7172	845.6948	657.1257	747.1489	1354.925	1043.047
H3f3a	820.0341	811.9961	849.8428	864.5783	939.9221	938.1554	827.1278	1026.342	1041.422
S100a6	774.8221	796.7518	795.3472	749.2436	770.9037	840.1719	650.7587	939.1462	1034.775
Rpl23a	843.9455	823.9268	856.9146	979.5964	972.9086	1040.636	913.2893	1002.49	1030.329
Rps8	980.0551	914.609	958.3658	908.3377	998.9639	1160.86	888.7385	1131.756	1025.76
Lcp1	1213.858	997.5434	1167.114	928.3752	891.0046	820.0591	1060.054	907.8555	1019.998
Tgfbi	1189.744	932.8103	1215.899	672.8604	963.8322	791.576	800.0616	946.9739	1011.578
Ubb	861.6791	857.5807	919.5437	980.3473	1006.134	1055.379	1004.178	998.5383	1011.295
Ifi30	988.2781	960.3972	1101.712	1106.428	1229.815	1261.878	1161.497	1093.313	1003.318
Rpl4	1094.609	1064.75	1098.09	980.1224	987.4676	1009.232	986.114	1051.641	1001.272
Cdc42	1088.284	991.672	1027.157	869.6842	970.472	884.6344	968.7846	988.5162	994.0298
Rps5	1046.731	950.3237	974.0251	908.3377	1021.154	1133.148	918.9361	1012.924	990.9868
Anxa1	931.2402	1041.68	892.4379	856.5118	700.4236	869.5977	766.4789	803.3151	981.7151
S100a10	1074.56	1002.374	1011.99	777.2133	836.4923	873.5962	747.9593	929.9459	978.7925
Emp3	1093.329	968.2397	1083.07	865.5312	965.6294	942.5624	883.4324	1003.703	973.3792
Tagln2	1354.702	1216.75	1123.132	934.974	929.668	873.7713	1011.422	1001.798	968.5455
Ifitm2	984.0919	924.4179	1049.643	732.0443	930.1649	854.7017	690.3176	947.8957	967.8429
Nfkbia	862.7483	784.8516	511.4659	469.6488	689.8594	606.085	884.1819	836.5971	950.0024
Rpl7	916.4494	936.5638	912.8754	862.7989	970.1602	976.9895	817.3248	982.0139	943.3229
Rps25	841.7832	828.7912	778.7359	844.4022	886.4608	999.789	769.921	912.5811	935.0829
Arpc1b	945.3065	784.4759	843.2856	834.6968	895.6019	900.5012	897.2993	937.1866	929.9178

Ptma	950.625	885.2915	897.7017	851.0098	834.0376	886.7108	840.8304	901.8508	927.2799
Rgs1	469.2228	478.7822	356.3456	677.3639	410.2412	589.9604	823.1957	757.3689	924.138
Hsp90ab1	1135.12	1120.432	1062.823	878.5441	935.9906	992.4032	972.5576	974.8229	914.5696
Rpl17	724.8438	806.0259	769.2484	787.1473	883.62	907.7919	714.8565	906.7995	913.9692
Anxa5	1002.355	900.4906	905.1219	926.6157	840.6126	854.506	1001.153	863.2261	904.488
Rps23	728.1864	826.468	735.0643	914.223	984.3372	1045.116	836.3444	1005.91	895.0941
Cox6b1	778.3804	766.4455	833.1661	840.0243	850.4856	885.8173	761.1343	839.8119	889.0963
Alox5ap	925.1043	847.9436	946.7311	781.022	886.6022	858.8238	830.2438	941.8564	882.1548
Gnas	858.0198	852.1961	883.1518	1003.811	993.9231	1007.694	996.2285	909.1816	882.1068
Arpc3	767.8879	738.72	733.8859	774.1104	800.5347	839.1691	780.6138	869.8856	881.933
Rpl34	604.7481	683.3767	597.6061	782.0937	852.397	873.9422	594.7579	898.9112	878.4754
Lsp1	955.0037	815.1559	1021.927	802.2667	934.5861	1017.796	847.3828	1020.292	874.9654
Rps3a1	860.9067	839.6178	841.9569	765.2963	869.6372	910.5805	795.9136	907.2092	867.6428
Cd36	1063.702	982.8029	837.9083	1027.521	667.5627	717.6649	1093.4	690.2743	864.8989
Rpl29	858.1469	824.4408	878.6811	926.6539	1020.864	1062.675	980.0469	1036.604	859.3567
Chil3	1493.045	1173.926	1415.294	381.3435	418.9238	602.3501	355.6274	804.4954	852.6082
Rpl13a	688.1128	752.7264	703.9183	723.8577	821.7748	906.2235	644.6305	880.3766	851.9902
Coro1a	644.3046	585.1325	654.4056	674.2571	760.1535	763.6028	580.5783	818.3351	849.8572
Rps4x	961.1546	927.2531	909.3583	784.6031	908.9291	960.3141	834.7706	898.7139	848.2794
Hspa5	763.3344	695.1893	689.3148	750.7699	729.7209	768.4038	868.266	749.0361	847.5878
Rps2	937.4527	922.8362	965.1451	857.8152	896.0436	923.3261	977.3353	939.9929	843.653
Rps7	623.26	690.2966	591.6004	681.8948	741.9825	775.2525	545.0167	775.9737	842.7043
Lpl	911.3804	886.6003	755.1216	1053.25	701.782	733.1527	1019.279	748.3926	831.84
Rpl31	618.8274	654.0225	646.7282	740.246	802.6911	897.0806	620.6917	809.5004	821.0119
Itgb2	945.4839	969.0092	1068.72	684.4101	697.2751	665.656	692.937	788.8246	815.5677
Rps21	594.9098	688.6046	589.9122	718.6729	773.6443	835.0025	562.6574	810.4796	815.0479
Spi1	858.8486	779.9654	905.2552	772.9265	869.0331	853.0142	811.0679	863.8086	810.1875
Rps10	780.3973	770.4359	803.2692	788.5408	870.7312	941.6655	756.9142	902.3525	805.2499
Cap1	869.2648	707.1981	816.9091	720.3263	681.9326	675.9693	792.163	761.0793	796.6313
Lcn2	156.5654	125.943	103.3491	175.3999	124.4141	611.0615	47.89388	65.52947	795.5962
Gusb	1151.891	1274.347	1262.208	909.0273	778.0948	809.7821	1008.111	769.2601	794.8206
Eno1	858.8951	699.7474	737.1434	603.1045	742.7305	739.7986	750.9793	788.7295	782.4752
Rplp2	757.7801	671.0597	664.5746	753.4095	867.0784	893.0825	710.5999	867.416	768.3137
Trem2	662.2587	766.5661	798.9396	905.5365	830.84	840.9543	889.3533	827.7317	766.0696
Lmna	1095.596	1044.44	890.2144	619.3507	586.6244	620.0118	718.1822	708.9747	758.9403
Rpl27a	689.7851	697.4455	768.4948	745.461	817.0014	905.6623	742.9676	828.0487	756.9718
Ybx1	710.8015	743.9105	709.9478	725.4893	714.8656	768.7534	746.8354	736.5696	751.2026
Rpl10a	753.6905	773.5337	739.6164	779.822	800.1215	860.7979	768.2075	812.5957	750.9913
Clic1	685.8248	680.5165	749.7207	625.0481	688.2041	738.0026	614.7547	735.3113	749.8443
Lilr4b	658.1244	536.8765	525.8196	658.3065	582.2402	484.3205	866.5545	673.1639	749.6554
Cd44	1013.848	875.7104	914.4163	599.8041	615.1709	506.9721	658.9541	752.287	748.6128
Hexa	748.4139	737.3868	819.8642	827.2823	830.2717	820.0403	883.7774	740.9135	744.4898
Rpl30	750.2193	750.87	721.8407	781.7125	809.5786	975.4553	670.8916	906.5035	738.5089
Rpl3	733.648	697.0418	718.2121	691.4636	716.9515	755.4123	732.8756	728.367	733.2014
Fcgr3	814.2905	853.5183	968.9351	772.6486	911.3173	806.2093	896.7287	882.0695	730.5824
Fn1	1472.536	1203.556	1287.828	481.8492	711.1246	471.6299	974.3555	878.1032	713.923
Rps28	659.5998	659.6774	654.3017	712.7201	828.0761	832.8334	669.1588	801.7011	708.0481
Ube2d3	694.4794	684.024	671.3493	580.3342	631.5908	585.5848	648.9945	707.5216	703.7094
Rpl35a	597.6246	607.0691	559.1878	595.9611	695.5221	733.5099	571.1129	749.3821	698.6159
Rab7b	732.1768	634.1054	633.7841	760.5984	566.37	586.1964	866.962	699.0917	693.3527
Rpl37a	623.0182	638.8494	585.4676	668.3744	728.3154	783.4842	603.7729	706.4592	693.2662
Rhog	688.3706	677.1333	774.5562	599.0321	744.5689	688.7432	697.5035	737.0754	691.777
Cd9	744.8245	844.2305	797.1132	564.4009	497.3743	513.2306	578.3891	600.2297	689.3581
Atp6v0a1	932.7901	787.0866	792.5583	827.6835	761.0947	708.7578	925.332	668.3509	687.3407

Ccl9	738.2404	877.9017	780.5731	477.8941	596.705	502.8396	485.9854	732.9534	684.5851
Msrb1	532.0275	447.705	536.8926	520.3213	600.3794	618.3888	479.1584	601.2121	675.245
Rpl24	624.5967	614.2209	588.7406	593.3313	668.4194	692.374	552.0524	694.3343	673.813
Sirpa	773.2384	717.5074	828.8281	800.7883	708.4003	631.8829	854.8397	698.4067	668.9457
Rpl36	579.9884	546.9274	532.24	655.2997	712.5866	780.9774	606.486	704.9767	664.9463
Capzb	725.7529	664.4545	719.137	693.6741	728.6207	708.1095	716.8656	725.3683	664.6019
Ly6c2	396.6526	171.1308	411.9121	564.5865	466.9457	439.7741	274.681	707.9288	660.1453
Thbs1	622.6894	461.0629	454.3319	376.9044	362.6528	309.7708	712.995	577.9251	659.0528
Rpl11	602.8745	573.2202	613.9987	681.2105	694.0426	710.6259	668.6761	692.6184	646.7342
Sdcbp	578.8139	485.6642	540.238	595.3478	570.4555	538.7188	628.1077	621.6053	642.3605
Rpl7a	595.895	541.8338	526.787	615.7183	607.4284	677.9874	589.9572	653.4885	638.8188
Ldha	618.6593	585.5647	592.3905	513.7666	559.595	605.8068	591.0641	569.0687	637.9107
Cox6a1	705.7533	616.7251	660.2843	655.8946	697.4715	723.4609	658.7141	633.7821	637.4862
Rpl39	462.5374	499.5252	461.8246	542.0026	633.4616	642.4961	389.8641	639.7138	635.388
Rpl5	584.0963	614.9707	561.7581	540.0847	579.2535	605.8088	521.9532	609.245	632.5165
Pabpc1	850.605	800.3114	817.2915	538.2225	612.0279	578.9656	684.0285	687.5783	631.4317
Pf4	749.8281	642.2346	605.6464	703.8135	632.1466	674.9334	885.8778	568.3092	628.2978
Lamp2	666.466	706.5306	768.1607	548.1612	592.5078	533.6338	643.6412	649.6258	621.0758
Hsp90b1	683.8031	655.9706	647.1674	618.0117	591.9414	593.1148	647.6191	552.9055	619.8579
Csf1r	721.1097	722.115	875.8658	645.7674	776.7594	641.7618	835.3679	723.6442	619.4316
Samhd1	565.0931	464.591	610.7914	561.1387	592.7047	508.5378	546.8474	628.2041	617.5822
Rpl9	504.1423	527.738	540.4501	548.0817	575.4684	579.9932	483.3581	596.6239	616.4849
Mcl1	554.1899	547.5221	481.6634	467.4556	500.615	454.4932	634.0822	600.707	613.3646
Cox8a	581.3368	593.0791	670.6242	614.0903	647.8243	667.4167	601.1559	676.9715	612.3321
Capns1	676.9336	620.6651	702.3091	615.6919	671.1548	639.8954	664.6271	632.0897	611.5167
Rab7	654.7501	624.2516	639.2138	584.3194	540.6815	532.6837	643.382	587.9998	610.8645
Rps13	549.5227	565.9198	525.4844	527.9739	621.5176	617.7632	527.2007	646.0864	610.2629
Csf2ra	639.7115	641.1183	669.7121	588.1263	621.1617	603.7573	642.8285	683.6886	607.9301
Prdx1	544.7954	613.4238	532.4942	560.4178	506.1139	500.3364	486.2041	523.0195	606.6419
Shisa5	700.0322	595.4914	766.3068	571.4263	627.5865	557.148	634.2051	690.5821	605.0089
Jun	436.5291	353.8798	284.4771	688.5665	537.7439	542.2246	912.4532	652.3032	601.9849
Actr3	545.6444	494.5149	551.5109	482.4901	505.5089	487.5727	500.9252	570.5721	601.0539
Lgals3bp	565.841	450.287	734.18	685.6251	668.0067	616.6528	633.4801	647.4426	598.1117
Rnh1	526.1582	448.1139	452.7185	534.9906	489.3779	554.8174	513.7397	530.3807	597.3313
Pld3	729.0536	734.5174	772.6333	710.3994	690.8305	681.4129	781.184	674.9836	595.4341
Cd63	573.4125	723.9807	663.3079	548.8333	551.0233	591.704	535.3525	514.7898	594.0258
Rpl23	562.4197	561.2868	569.6897	624.9863	644.9185	661.7934	543.6297	670.5163	590.6146
Eif4a1	622.0987	533.4392	536.6459	514.2286	485.6681	509.1902	562.2194	548.4689	589.2967
Rhoa	656.9468	628.0401	671.0568	522.7679	548.0594	516.7954	577.9431	599.9626	589.1863
Nr4a1	638.5047	484.0212	402.0187	407.4354	488.4967	382.0996	630.5874	559.1917	584.2175
Emp1	793.0231	680.2089	662.6851	504.9203	430.9655	404.7128	620.3809	574.7877	582.124
Rps12	478.7152	470.8229	476.0949	507.5351	567.8398	571.76	454.0701	587.9437	581.5174
Pdia3	611.5597	564.2013	603.0927	561.5165	565.4425	574.668	580.5894	520.3956	579.872
Ier3	469.3417	364.1055	272.7463	273.1594	430.1875	299.1852	522.358	599.1052	575.7337
Rpl6	594.4734	564.0513	589.9739	568.5632	580.32	602.2482	619.231	605.9427	573.8564
Rps3	477.2866	519.0948	482.2108	509.8196	549.3415	589.9705	475.979	579.0622	572.8035
Clta	590.598	530.5667	628.8477	610.5097	606.1502	642.9723	684.8398	594.017	571.8248
Slfn4	276.6808	130.1562	232.6427	320.2869	281.5408	193.3836	188.5146	310.097	570.7799
Efhd2	602.0653	519.4889	612.8702	547.0151	549.141	503.5443	631.2166	583.622	565.5665
Rps6	576.1343	536.4842	539.1322	499.7464	568.6644	566.8834	495.6647	596.2071	564.8371
Unc93b1	587.9162	600.7288	707.6133	634.354	780.0517	661.2772	743.4934	702.3089	564.262
Rps27a	617.913	429.6054	547.7245	603.821	753.6528	514.9336	377.1724	522.4447	563.9628
Arpc5	568.379	535.6793	572.7415	524.5978	525.8429	501.5393	555.5822	539.5108	563.079
Irf7	334.5663	199.3942	410.8613	452.2037	433.3419	400.4144	296.0123	483.9653	562.8621

Atf3	373.4568	291.0511	230.2475	500.0237	375.6793	393.8538	678.9683	569.6342	562.3161
Elob	468.1679	452.337	463.42	529.463	570.0794	581.1986	452.4061	530.9418	562.2814
Ftl1-ps1	521.9771	466.2492	505.0881	556.7939	534.1809	547.2752	643.9087	514.9274	560.9637
Ms4a6d	471.3246	426.9989	530.0996	419.9503	463.9037	440.0544	382.2792	459.296	558.0107
Msr1	557.366	490.8678	519.1072	403.3291	388.5574	374.2834	406.1245	528.4723	557.1878
Gng5	452.5197	455.3455	493.6116	506.658	512.5906	522.544	436.9642	531.2778	555.9284
Serinc3	685.3822	772.6727	885.7143	572.3766	704.0405	591.0397	772.9212	748.757	552.8168
Dab2	872.6996	739.1106	803.0893	530.3896	573.9915	449.9596	742.1974	629.0642	551.7597
Csf3r	423.2267	400.7324	356.1534	340.2252	465.0596	400.0158	362.5641	442.1121	551.3273
Ost4	489.4866	496.1349	456.3509	502.7852	580.4315	589.1463	490.0033	559.9532	548.5193
Sat1	351.6615	386.9386	398.5074	513.3279	532.4161	504.7292	530.4481	567.1404	544.1166
Plekho1	468.2499	446.0568	475.7496	575.1377	561.2179	605.5936	571.1747	566.2731	540.7807
H3f3b	449.2347	342.4236	390.6709	464.9743	440.2709	410.6278	477.1863	490.0586	537.5735
Mmp12	949.8679	1398.09	1046.991	623.0527	622.5068	698.3956	611.0391	552.2863	533.7825
Calm2	497.6794	481.3304	471.7228	493.0773	494.5692	522.3649	481.4967	544.038	532.5207
Cox4i1	459.0006	496.044	481.7622	500.6799	517.5684	539.1122	476.9695	540.2905	523.7142
Msn	624.4289	551.6664	636.0091	456.654	467.2676	416.1737	534.662	525.8866	520.8749
Pim1	492.4447	382.4811	300.4591	382.2995	415.6871	354.5921	591.9811	526.2554	520.6704
Ptms	591.4353	570.4253	630.4832	698.3297	676.7364	684.6304	649.427	701.9383	518.4927
Tgfb1	673.6171	569.6349	713.0667	540.3251	631.0322	574.1747	688.415	580.736	512.4119
Calm1	508.3137	473.3858	463.3112	459.63	434.0559	444.6758	476.6679	467.0161	512.1468
Gm3788	510.5648	449.0467	560.3731	706.1408	578.0174	764.7116	557.2441	610.0414	511.8692
Npc2	475.9142	464.5789	588.8302	481.3434	498.6247	457.0215	464.8129	527.6305	511.4796
Rpl14	522.7187	489.8677	456.1867	505.1096	518.3298	544.9696	460.2838	520.0343	508.3127
Wfdc17	273.0531	243.4431	265.8735	539.521	560.1831	528.7427	468.8907	523.4355	507.8699
Mt1	523.8999	602.9926	512.2991	462.7864	577.6674	581.6294	642.964	549.0545	507.512
Atp5b	576.0366	525.6273	527.5343	460.1784	447.8187	476.0582	492.9946	461.2216	497.7795
Selplg	490.7215	475.9869	538.5284	495.7467	572.7481	533.5882	565.9581	541.8252	497.0618
Cfp	496.3333	497.4791	491.8836	455.038	527.1792	453.6349	456.5278	508.983	494.8823
Hnrnpa2b1	492.9923	488.7962	494.1392	430.9781	458.3462	442.3023	474.495	509.0337	493.9478
Zyx	629.5627	521.2493	602.301	470.0585	593.6599	539.407	530.3172	568.1683	491.9862
Lst1	320.6346	273.3954	386.5122	487.004	586.8768	488.519	405.769	533.2806	491.5402
Sem1	324.1602	308.2078	337.071	451.0128	427.6579	493.9837	381.9478	458.3797	489.6337
Nme2	387.0133	391.0844	379.0839	423.5918	439.5108	440.3291	366.1786	463.5117	488.3696
Camp	0.555306	6.276715	0.370247	80.34446	62.84709	365.8899	22.19222	3.877176	485.7689
Adam8	597.695	501.8933	514.2594	432.7174	373.9138	394.614	519.3069	392.2582	482.2388
Ccl3	283.9973	256.1559	206.7589	308.9933	308.7716	306.6309	382.2285	532.5016	480.2184
Itgam	566.8687	495.4045	593.0512	366.5114	417.7172	338.3932	454	456.8803	478.9017
Arrb2	451.7356	478.4969	550.7613	499.5862	542.1969	506.6608	569.7558	490.6801	478.5815
Ccl7	488.8378	333.7446	353.8481	417.1385	442.9546	315.2848	385.0626	637.5411	478.3731
Ncf1	449.6477	476.8942	491.4044	343.1679	392.9578	408.4175	431.332	447.8888	476.885
Abhd12	461.9257	449.9425	469.0518	514.9432	551.3505	505.2461	634.3837	488.3833	476.3271
Dazap2	506.0016	464.9923	517.5936	458.9624	502.3112	459.268	542.8844	505.4023	475.0726
Ccrl2	266.5304	232.3266	154.7603	224.2937	266.3589	246.5952	468.8491	415.0145	469.1529
Vat1	580.1719	502.9571	477.5899	539.0673	458.2192	474.1107	621.805	433.9291	466.0406
Gadd45b	290.1521	242.5182	181.4866	306.2931	379.1824	333.7239	455.8948	425.8453	461.4717
Eif5a	510.7505	440.4595	419.983	387.0714	411.3168	427.6779	409.0493	432.3301	460.763
Ecm1	885.6631	959.5291	1036.747	409.5859	504.1505	446.7058	592.8313	549.9559	460.6631
Ppp2r5c	519.5635	531.3053	561.9068	448.4392	442.1266	422.5966	560.6637	460.4407	459.7347
Zbp1	275.2028	154.6859	315.1321	408.339	362.289	327.9537	258.9782	372.6537	456.4583
Ccr2	434.357	402.6981	491.1589	409.3081	521.7153	410.4877	389.1641	562.686	454.2196
Anxa4	508.9204	471.7408	497.7349	455.9274	411.9589	397.8061	517.644	442.001	452.7131
Dynlrb1	451.2268	466.1587	492.0903	437.5444	466.749	506.3594	451.9989	487.8122	451.9844
Retnlg	69.6623	81.11694	90.46209	206.0029	219.531	401.0324	136.3655	107.8221	450.856

Itgax	707.4736	972.2211	892.7204	385.1673	550.1736	530.0862	518.1323	452.2537	449.7262
Rnasek	527.9094	527.8373	572.8968	496.5022	543.2654	506.4921	511.1682	517.3021	447.0791
Prelid1	455.7223	433.8957	432.6606	403.003	462.0969	485.9338	462.9089	459.5322	446.9103
Atp6v0e	406.2212	423.593	384.355	388.9361	395.6914	381.7716	362.1205	425.3062	445.3722
Zeb2	502.0225	464.3331	468.5463	457.3987	371.4777	306.5641	576.357	443.6532	445.0594
Myl12b	407.8447	357.1211	397.2047	402.6247	447.6917	458.4036	382.2918	431.366	441.8872
Arf1	469.051	408.6086	445.0662	398.2113	392.8052	396.2322	442.4272	419.1497	441.5844
Cd84	467.4361	504.2142	500.1661	500.8245	416.3638	375.0371	522.8658	430.2643	440.153
Btf3	430.8936	409.1993	400.4117	379.8709	447.2422	421.8013	401.086	468.8011	436.1465
Taldo1	363.9969	317.4436	369.4707	356.1687	385.7277	383.9831	335.3909	373.3243	432.0989
H2-Eb1	483.6631	422.7452	431.3642	495.1568	782.457	759.1198	554.7504	499.2671	431.5635
Tubb5	553.647	551.6087	542.2871	473.7632	448.9778	468.0741	550.7974	445.069	430.3102
Aprt	402.5341	366.5388	391.3127	420.2041	401.1109	407.2383	420.4721	437.8629	428.8615
Tpp1	564.2319	558.114	602.649	502.5579	480.4453	429.0625	610.632	459.4624	428.465
Rpl27	371.148	357.2789	358.0476	406.9974	438.4298	461.1969	305.8496	439.7341	428.0981
P4hb	485.3204	438.4587	470.3965	403.35	397.6966	389.1017	454.4587	414.7141	423.7149
Tmbim6	442.111	403.6413	445.9253	421.8514	454.4838	404.1038	446.502	427.81	422.1679
App	483.8086	408.694	485.7944	442.0059	415.4771	382.5575	502.9126	418.2151	422.0919
Cflar	448.8124	393.6505	404.6269	355.9831	342.4888	271.4717	542.0452	416.9254	420.4508
Ppp1ca	415.7651	408.5329	408.41	386.9038	426.7661	419.1663	390.9128	437.0915	420.257
Ywhae	479.2004	448.8692	445.9307	393.0555	399.1656	404.6373	428.2736	424.5204	419.5971
Nptn	456.9955	419.6462	472.5963	433.3796	389.7567	363.0441	432.1128	419.4526	418.6544
Ccl4	200.669	154.9809	155.9167	281.4845	266.633	252.9968	393.4463	427.538	418.136
Oaz1	402.5489	407.8967	381.2069	384.2409	424.9596	432.5566	420.1854	410.0031	416.515
Rap1b	416.916	389.4596	420.7714	331.4767	349.4739	314.5388	374.9662	429.0722	414.3521
Atp5j2	331.8512	358.613	314.9196	392.7192	413.5068	447.7511	337.3621	371.8204	412.2058
Slfn2	286.3189	244.2217	261.0956	265.5994	304.6708	286.2224	296.4759	345.243	410.76
Rab5c	421.0946	352.6303	421.0262	381.0216	408.1695	378.1309	416.5171	399.6108	406.2329
Atp5d	431.5716	426.3937	410.3639	381.0721	415.4043	422.0938	400.4437	414.0939	405.995
COX2	367.7229	397.4609	296.5957	454.0767	379.7794	866.9734	225.925	350.4169	403.4645
Plac8	222.9351	79.59311	178.0849	277.6765	259.2614	267.6405	143.9378	363.5458	402.857
Cd53	350.3163	346.5619	371.213	353.6661	367.6494	322.4612	320.4353	422.0149	402.1827
Ubl5	299.4634	310.1393	297.1728	322.0032	410.3839	396.6715	308.2456	349.2032	398.205
Rac1	457.3823	449.4879	481.187	385.2367	415.3614	371.118	456.9699	421.8178	397.014
Rpl38-ps2	295.6077	310.0348	278.5249	356.479	374.7283	419.0851	308.9862	402.7763	395.2166
Hmox1	367.3827	317.8851	314.0565	336.6767	352.8059	376.7958	413.2291	369.8792	392.9614
Slc25a5	414.0945	401.0374	415.2986	349.2403	375.502	371.936	373.0786	407.6682	392.8328
Ms4a7	297.6057	294.8249	350.8306	446.1493	513.0423	444.1303	447.3635	406.165	390.848
Gnb2	374.8765	360.59	382.018	353.1357	383.4913	385.3103	356.0711	382.1629	388.6229
Atp6v1f	330.1839	373.1018	400.9467	370.8737	389.6288	389.5514	359.3984	391.754	388.5005
Cox6c	242.4726	299.6458	245.2565	288.8989	330.2275	339.2005	234.8876	322.4663	388.1395
Selenow	383.7415	366.1719	347.1377	398.7167	412.5031	463.0063	384.4249	370.226	387.774
Grina	311.7368	287.0663	318.7486	273.3908	333.0086	305.5451	330.7242	309.8047	387.4014
Ly86	302.7085	321.296	408.4505	437.3635	560.0897	476.7609	461.7816	536.5776	387.0671
Il1rn	492.1998	415.5851	357.2477	267.88	263.7958	201.9199	225.2199	303.9867	386.575
Npm1	382.471	393.9017	385.7009	369.9707	374.4684	384.9432	362.6056	396.5242	386.3119
Gpi1	413.9516	399.6542	418.3976	336.5319	385.9583	385.3489	417.2216	383.3174	386.2236
Snx5	337.189	322.7601	364.7786	446.4669	410.537	375.7206	483.2313	399.5452	386.0254
Gm9794	366.0235	358.7835	366.1762	411.8673	375.2585	400.4564	334.1055	401.9023	385.8286
Eif1	362.9193	360.4185	332.1394	315.2892	378.4668	360.6606	340.1529	402.1681	385.7842
Tcirg1	379.6724	385.758	373.1985	461.5495	394.261	379.4893	564.1125	376.9391	385.7051
Man2b1	393.3029	423.452	515.515	410.2999	499.9464	447.8504	519.8329	480.5572	384.9813
Cox5b	357.1809	338.642	332.5696	367.5814	376.2712	416.7164	348.2188	402.9649	384.9051
Sh3glb1	386.0012	373.9992	424.8056	349.1517	363.4744	348.3377	397.7175	400.0469	384.5935

Eif3h	331.3192	346.1508	349.0114	333.5934	359.0563	384.3801	316.9121	395.6966	384.5771
Plaur	364.2736	318.4293	326.3058	211.2571	269.6767	292.1006	264.9018	348.4316	383.4079
Rpl36a	337.0498	375.5003	319.7413	369.6588	365.3793	409.8246	307.2298	405.2053	383.3683
Card19	385.9478	356.5355	358.0232	296.4948	303.676	332.0951	339.4816	377.5459	381.035
Cndp2	356.7194	304.3839	317.6051	353.8907	333.3223	362.5647	408.4236	354.9149	379.7875
LOC118568312	226.0977	285.0185	330.0527	318.4096	434.6104	270.5273	203.8588	425.7829	379.2127
Tmed5	369.8455	364.7045	372.2149	287.5883	301.5613	284.6595	314.3377	359.8805	378.1754
Naca	341.8019	336.9356	344.1725	368.9304	339.7434	394.4104	327.0291	399.9287	377.329
Tnfrsf1b	398.0832	389.7722	451.6376	374.1835	370.0983	304.6079	458.4657	396.7714	377.2244
Fcgr2b	376.7627	446.8415	543.14	367.2138	550.3087	440.423	457.9116	497.2393	376.4368
AB124611	311.3579	334.0466	367.5124	362.2529	438.7967	377.8299	395.217	440.9535	374.6318
Trir	343.5777	330.7083	350.5338	352.1973	346.0998	393.2986	340.6908	365.856	374.2558
Eef1g	413.0756	363.5039	368.3074	346.4404	358.4232	377.3476	367.293	374.0304	373.1367
Eif3k	366.5278	353.9624	373.2326	388.0271	402.3691	394.6699	347.6895	409.575	371.5548
Hint1	350.6176	374.7849	345.8146	343.4935	345.1266	377.747	323.9438	348.9736	370.6037
Gdi2	375.9284	415.9881	422.9376	324.8021	398.1657	372.6972	393.8196	378.5533	370.2757
Slc25a3	411.6648	405.6773	417.824	350.2337	373.1127	352.1798	418.2048	378.4721	370.0917
Coro1b	343.4587	333.4436	374.1981	353.3457	416.8326	395.8628	383.7699	387.6791	368.8923
Aldh2	307.0124	286.6823	354.1067	382.115	380.0569	404.8944	401.1982	349.4287	367.1979
Adgre1	408.5359	399.6225	467.6104	392.0933	400.6196	341.765	457.5613	395.0835	365.978
Erp29	320.3187	316.0853	326.1546	378.3531	379.4024	405.4784	363.9783	368.1609	365.6535
Tle5	397.2494	395.928	482.1442	426.0264	440.1683	456.2343	435.6523	403.4063	363.991
Ddx5	359.5284	334.4602	330.5564	325.8162	315.8916	290.9715	365.0957	347.0897	363.6174
Gna13	376.6028	369.0064	370.3378	329.197	279.9366	237.2098	473.4295	349.3807	362.2888
H13	399.7403	346.8129	390.0827	308.2518	374.0663	322.6994	353.5031	375.0587	358.6393
Snx3	449.9058	395.7866	417.3035	406.6706	415.142	407.0861	499.6203	390.0613	355.6644
Zfp36l2	309.4626	272.9352	307.4607	335.8618	309.3279	285.7175	385.5344	340.1686	354.998
Ndufa3	349.5283	298.1905	335.3524	336.4193	370.7936	387.7918	315.7104	355.1914	353.9052
Ptpn6	324.4588	289.0354	335.8403	334.9885	319.861	308.0433	295.9924	345.1436	353.6224
Atp6v0c	397.0668	322.1606	349.5102	354.0461	352.4465	341.1528	367.3008	344.9786	353.2317
Atp5md	244.966	272.0352	215.0575	266.2219	294.974	285.4308	199.2013	319.8938	352.9771
Cd48	267.3237	264.9514	282.8965	320.1566	328.244	320.1503	310.1358	320.6285	352.8175
Bst2	264.0855	200.428	277.9773	318.2796	322.8033	331.6518	267.2016	310.8156	350.8979
Prdx5	217.2348	211.0574	263.2298	252.4352	278.2787	274.0209	208.9292	312.6025	349.6715
Lipa	399.894	401.8482	416.1795	397.1523	341.5627	324.0195	443.2243	335.9434	349.6493
Slc3a2	368.4722	297.4999	307.8902	319.4041	321.02	297.9147	400.5354	341.0148	348.7607
Irf5	372.8163	341.1865	413.6773	340.181	340.0956	340.8096	359.1299	369.9831	348.2086
Pcbp1	354.7388	301.1552	340.1245	336.697	331.0874	335.4566	350.7241	362.8409	347.9544
Sf3b1	338.1922	326.4264	310.5759	321.4558	315.6176	279.9988	402.3945	350.2231	347.6345
Cybb	385.1018	412.2693	460.1992	289.2739	326.5984	290.2519	341.9344	364.2385	344.6059
Cdkn1a	374.0403	332.9567	328.4065	233.7949	281.2982	254.193	312.6979	382.9143	344.4512
Rrbp1	382.6895	291.3691	374.2041	406.3171	338.1975	353.8802	409.3049	359.0816	344.3954
Slc11a1	379.9095	378.7301	425.5318	347.2747	345.1159	288.1343	411.3072	328.1952	342.1818
Chmp4b	293.1383	253.0073	295.5544	305.8788	322.2819	335.9833	254.1662	326.5546	341.3704
Litaf	343.6807	289.9919	313.3107	331.3423	358.7758	334.1831	422.889	345.957	340.978
Atp1a1	448.8956	435.2892	451.2991	283.0982	299.3062	268.9114	323.435	325.777	340.6742
Pirb	368.3949	331.0346	365.8864	302.1292	327.0358	286.3969	320.5209	338.8544	340.5355
Egr1	352.8221	322.2938	255.6909	194.9203	323.1336	255.8029	246.6873	328.9798	340.3815
Tpi1	339.8742	302.1216	299.9816	305.8471	314.7429	349.3092	350.4188	327.3663	340.3318
Plek	409.1671	365.3845	307.7392	238.8011	263.0568	206.4767	301.4652	352.3874	338.7266
Gsn	391.2548	374.6816	367.3958	357.1642	299.316	346.3156	341.1652	321.7542	337.7376
Atp6ap1	397.2712	363.5728	381.6334	322.7594	338.1708	302.4873	368.9948	359.3652	337.0946
Sqstm1	327.1887	328.6551	335.0689	303.4316	300.4781	311.9925	388.7054	338.8436	336.3834
Fcgr1	247.7261	164.5046	281.6127	304.5322	300.2322	269.6241	250.7037	300.4054	335.9634

Jund	310.141	286.907	219.504	279.7573	324.3893	330.4314	371.272	348.856	335.9408
Manf	344.7634	322.0606	331.5173	335.592	313.6894	360.5845	342.7318	320.5866	335.2862
Tppp3	374.0986	417.0543	396.6123	237.2529	300.4467	307.7044	261.4781	351.591	335.084
Ap2s1	331.8642	303.3354	315.7141	313.5637	330.4213	350.6484	331.6535	298.8361	332.4519
Rn18s-rs5	187.3321	254.8027	268.0467	338.6064	366.8204	363.308	289.7128	338.7327	331.9412
Reep3	424.6265	377.662	386.4029	338.4949	337.7809	257.0479	360.5625	361.3444	331.4346
BC005537	377.3743	330.4207	351.0978	350.1711	269.7468	237.6065	340.5312	314.605	331.2124
Fabp4	229.7897	194.4416	150.875	474.6295	299.0285	371.1644	304.7018	253.5128	329.6936
Clec4e	278.8167	246.8634	186.7501	119.5406	188.8411	119.4942	151.6934	306.3359	329.5589
Chmp2a	279.9802	262.4502	283.073	307.0496	328.277	344.3903	288.6294	334.4318	329.2044
Myl12a	326.8106	284.1264	302.5986	299.8236	297.3452	305.5911	306.1195	316.0158	326.0035
Fyb	354.1968	316.4487	392.7576	364.0915	344.0148	284.3707	435.5285	336.1469	325.6989
Prr13	334.1254	353.7893	431.9282	327.2918	353.0045	337.045	327.3877	337.3137	325.68
Csk	333.1311	308.4506	358.3095	345.1719	361.7553	333.5127	383.6085	360.2088	325.17
Clec4d	312.0419	292.7118	241.1607	173.6538	190.0829	181.4165	152.1642	230.4135	324.9916
Gpx4	257.2805	295.6777	273.1416	319.5717	328.3832	349.8934	309.0311	302.3605	324.6684
Ifi204	203.2568	137.5278	219.991	283.585	226.3027	195.602	190.3362	268.2113	324.6186
Psmb6	289.4495	258.6847	251.7954	344.5771	281.8801	309.9499	319.7578	293.6959	324.2766
Hnrnpul1	431.3748	372.593	426.5553	367.4177	373.0884	310.1321	462.0921	357.5626	323.6189
Atf4	306.0212	291.7853	272.9664	238.0853	263.3723	277.8933	297.6643	308.5548	321.2996
Osm	277.5682	204.1427	191.8607	156.6235	250.7864	208.9139	262.8137	298.7039	320.2384
Psmd8	297.3429	305.2487	285.2845	322.9314	303.3564	280.1239	312.0147	322.1719	318.2117
Mafb	327.6391	266.0925	329.166	309.5698	291.5063	277.6819	434.6973	341.9725	317.2894
Edf1	271.7234	253.1094	265.4331	316.7497	310.446	351.1302	296.4664	321.1679	317.0244
Pdia6	340.3267	276.2978	306.8825	318.7172	307.7376	328.1256	342.8001	283.5889	316.6058
Iqgap1	424.6915	354.5916	386.4913	312.5721	262.7248	228.1854	376.417	285.2159	316.2322
Txn1	294.4389	322.2209	298.8517	275.3229	284.59	285.5071	251.4352	274.1575	316.1195
Flna	437.2414	348.8394	376.1352	292.3073	266.675	246.221	330.2375	284.6535	314.3911
Atp5l	212.8908	249.2921	216.0498	246.6231	266.7719	260.5343	206.7419	263.8095	314.2435
Sp100	259.2233	183.8587	271.0734	288.7826	281.0721	252.4516	252.5278	274.9697	314.0944
Atp6v1b2	342.1713	299.2582	310.0201	339.3499	281.8236	265.7789	383.345	298.0997	313.8834
Rhob	242.7569	235.5671	190.8103	322.5277	263.2513	268.6245	397.6955	344.2208	313.223
Ewsr1	343.8606	301.4031	316.4549	332.7959	301.5558	273.1375	368.2815	298.8306	313.0504
Vsir	344.8898	350.6366	409.6733	318.0523	338.2653	328.2671	370.9505	327.7465	312.7874
Atp5a1	344.0488	319.4662	339.7116	295.7891	300.0315	290.3395	331.8431	299.3105	311.6581
Mif	294.9381	238.9576	267.4887	261.6897	333.8762	345.8122	318.4428	311.789	311.295
Niban2	414.6671	325.859	336.03	297.3572	244.4777	256.0315	324.7508	285.1654	310.7577
Atp5c1	294.3861	309.8055	295.1062	289.7998	290.317	301.3605	275.2049	311.9238	310.6397
Bsg	322.1646	329.3012	336.7345	337.0868	336.4595	333.5301	341.8699	309.9278	309.8364
Cxcr4	224.0826	227.3392	257.9335	247.1591	274.1556	287.4149	364.0388	318.2713	309.5722
Tkt	312.202	280.2903	294.9538	259.084	278.0148	288.9212	269.4267	294.8098	309.2961
Nfe2l2	299.9333	282.1144	295.0086	256.797	266.9896	247.3865	360.7547	337.2412	309.2015
Cebpa	255.5282	245.6399	261.0056	321.7515	311.8443	321.1991	359.6776	336.2379	307.8841
Nckap1l	383.516	353.3113	398.1236	332.0536	324.8422	282.3415	423.071	307.2369	307.6742
Plekho2	333.8607	259.7507	287.859	296.9735	255.5684	239.4768	312.7313	285.2889	305.5366
Selenof	237.6861	243.5344	255.8892	248.9866	266.6504	261.7692	245.281	287.1926	304.8823
Taf10	311.1999	283.4952	282.5803	300.0132	321.5072	316.2364	270.7184	308.5031	304.0278
Lyz1	360.7637	528.6468	346.6487	294.2023	336.7841	250.7036	155.8596	367.2241	303.9508
Vamp8	259.4163	262.5946	269.3926	274.3752	307.7903	290.9718	265.6411	329.7897	303.7814
Esd	294.9779	299.8104	263.139	241.0478	236.7937	234.2216	235.4223	248.5763	303.556
Pla2g7	257.0913	341.8757	367.1581	234.3155	288.8447	225.2905	255.2158	301.6442	301.9881
Ndufb8	233.0512	245.8075	245.5167	287.6008	269.6445	287.6085	229.6961	283.1834	301.9169
Mapre1	326.598	308.0957	297.5088	276.5235	265.4941	267.1734	319.6365	289.8142	301.4148
Eif4g3	353.936	311.0624	361.3189	322.8018	286.0568	260.0839	386.2073	319.3271	301.3049

Tln1	414.8302	319.9858	390.5813	306.1564	279.7369	255.024	371.5589	289.7607	301.2401
Ninj1	285.4337	257.6536	271.5817	243.7116	240.027	236.3204	285.0451	297.28	300.2543
Aup1	302.6056	284.7533	316.4648	317.0202	327.3372	312.8273	320.1699	333.9588	300.0258
Cyth4	348.5135	333.1541	386.3311	323.2302	333.2898	311.509	363.1783	333.4705	297.795
Esyt1	354.7314	298.1966	343.5995	332.7687	290.7575	307.3401	382.8103	297.1823	296.3022
Gns	356.9042	341.6553	401.8714	335.0954	342.2264	297.1823	430.0341	317.8243	295.473
Glmp	321.4831	297.7484	336.3826	326.2459	325.6887	301.2001	331.7469	306.1864	294.9888
Rpl36al	273.1156	247.0425	265.6403	312.105	302.8005	322.7763	243.0455	312.4069	294.9492
Tspo	230.4954	223.2288	219.2519	231.5871	272.7517	267.4768	209.044	279.8211	294.6179
Tuba1b	315.9332	303.1784	267.0483	267.4601	256.4648	268.826	304.8444	259.9638	293.6124
Clec7a	232.5047	324.0022	306.1052	197.0745	285.8737	220.1875	224.2084	350.1564	290.9989
Tpm4	337.9107	304.6112	332.6142	238.6321	235.9713	240.5586	247.0721	272.2861	290.9959
Tmem106a	295.88	249.8965	283.1699	325.8172	298.0902	299.1645	339.2724	296.4045	289.9348
Mapk3	326.1523	339.1066	391.3969	311.3834	320.5161	300.7185	343.691	313.9842	289.2042
Pxn	299.1715	281.6497	306.6913	250.72	285.7988	248.3905	322.2153	287.5325	288.9942
Mdh2	335.396	279.8595	268.3998	266.6934	268.6881	262.2555	279.6518	272.3938	288.7098
Atp5g3	295.2374	293.5892	292.4617	286.8136	283.5481	283.9194	303.8023	257.8418	287.6703
Slc15a3	309.7912	214.1121	278.2865	280.6449	239.0685	219.9578	325.1692	259.7708	286.5612
Ncf2	302.4132	300.7308	333.6656	260.201	302.1656	276.7603	313.37	297.8633	285.9836
Stab1	402.6188	330.4324	355.5471	424.7158	371.1781	319.469	601.2924	288.0802	285.5808
Atp6v0b	251.9204	272.0669	263.7511	265.7302	283.4479	278.7278	265.895	257.0761	284.3989
Ptgs2	321.9385	296.1635	187.2433	129.2901	233.1425	132.0423	208.1228	242.5035	284.0543
Gm11808	305.5459	282.8009	257.5449	245.9447	316.1303	343.4594	262.8053	272.838	283.6012
Apobec1	283.5537	274.5738	310.6216	287.3313	268.7444	245.7445	330.5027	295.1989	283.4976
Cxcl10	160.0705	80.4499	139.8954	188.8155	138.3985	71.98853	104.1877	169.7093	283.2392
ATP6	207.7503	344.4085	155.0167	292.5564	316.7141	316.7888	138.6595	215.7803	282.1098
Smpdl3a	287.1574	286.7634	339.5705	294.3828	268.2676	277.6339	294.2641	319.9071	281.5634
Myo1f	278.0147	262.893	300.6125	297.0673	271.4314	257.0458	334.206	280.8501	281.0183
Syngr2	357.076	345.6924	374.2798	287.6371	329.9633	287.4234	312.6487	290.9191	280.0195
Gapdh	322.4894	279.4035	270.9556	250.1509	259.2271	289.0933	282.0825	277.5844	279.2834
Atp5h	239.3302	236.9592	263.0233	261.5196	286.2531	302.5133	222.3043	266.1193	279.0072
H2-T23	203.315	195.8886	248.1742	256.299	263.9529	262.4096	204.0888	266.9983	278.951
Rgs19	249.0313	246.343	265.7139	245.5497	275.1458	265.9143	244.0937	271.2594	278.8318
Sdf4	240.2876	252.8282	284.3911	256.5936	284.2592	292.5348	263.2236	284.4389	278.7573
Uqcrb	228.5303	241.8454	226.938	237.3099	263.2222	278.9769	206.0846	281.9239	278.4569
Eif3f	288.1161	281.6989	307.3546	264.4073	297.0316	285.2535	307.2876	311.371	278.1765
Rac2	274.013	268.1843	301.3471	223.2883	286.5316	269.5759	249.0651	285.0358	278.1447
Surf4	327.6935	291.6205	326.9523	262.1786	263.7457	265.304	310.0535	272.54	277.472
Ifnar2	267.8005	261.8375	282.9614	295.774	297.4607	259.2753	316.0948	292.4483	276.9331
Lgals9	263.2658	194.8313	250.7815	268.8436	262.9675	245.3486	268.2222	260.6245	276.2904
Rab35	307.2452	271.6377	311.6417	251.9452	269.7771	258.209	286.0072	270.5102	274.5764
Pfdn5	225.0998	250.5579	251.0926	237.8846	285.7656	294.089	244.8034	280.8587	274.5366
Tpt1-ps3	213.3995	232.8515	220.1612	226.0697	224.6667	246.3077	208.1665	264.0406	274.0739
Arhgap45	252.1874	266.7814	267.1707	262.3704	297.0743	273.3551	343.9343	279.8499	273.8337
Dpep2	349.8591	448.8123	444.2264	227.4108	231.2849	222.1344	248.8347	321.7253	273.5068
Dad1	312.2666	303.0267	274.8361	278.2477	294.3693	285.8322	272.9259	293.2948	273.2838
Atp5e	233.5362	214.0836	200.2163	256.8623	283.8881	305.8706	222.4104	266.2072	272.2275
Ncf4	257.5491	270.7136	311.1975	198.3931	237.6991	220.6237	221.701	269.5488	271.8859
Sdc3	303.6691	231.403	327.6591	309.85	273.0144	253.8402	334.928	281.8397	270.081
Vmp1	216.4516	249.2436	222.3617	211.1771	218.6511	179.7057	232.9616	292.7112	270.0269
Il10rb	273.4445	243.9356	292.1476	278.5877	261.1961	266.0916	305.5068	265.7383	269.7563
Dhrs3	303.0981	319.0546	328.8413	354.2224	340.3343	322.5584	393.5382	335.7053	269.6558
Rpl37	280.0038	284.4876	267.5661	272.0133	289.6311	374.8373	247.4983	297.5787	269.3062
Hcls1	270.1301	230.6418	284.1462	243.7576	261.6121	250.57	255.0585	288.4727	268.8214

Ms4a6b	207.9174	202.7754	249.3524	224.5622	227.7507	202.9423	198.8278	252.9616	268.6971
Sec14l1	286.2584	280.3144	333.5985	312.2431	344.1479	316.8968	412.5355	290.0002	268.4383
Tpd52	254.2973	211.9468	237.8341	247.3504	274.5943	239.0011	255.5852	272.1322	268.1856
Pitpna	327.1185	298.4331	325.3815	228.9051	247.148	233.886	258.3036	292.0816	267.732
Fes	262.2897	235.147	277.4775	236.5103	260.8095	271.2954	233.5545	273.0016	267.4809
Psme1	213.9784	179.2271	220.5295	242.3467	260.102	271.1347	212.1844	266.8297	266.2962
Prkcd	279.0815	297.9201	321.9777	269.024	272.9378	245.4507	330.4134	286.3889	265.1186
Basp1	225.5776	164.2917	184.4763	269.2185	243.329	240.1138	277.5633	251.8762	263.7882
Creg1	238.0333	296.21	303.2701	280.6585	272.482	268.725	299.1904	268.7687	263.4504
D8Ertd738e	212.1911	206.1055	228.5723	218.4251	249.3756	259.4393	226.3315	239.4775	262.4284
Itgb5	276.9969	264.7095	325.6307	295.6403	333.0019	300.9863	381.4703	270.9101	261.5601
Atox1	222.9387	250.7434	249.5003	238.3139	300.9726	274.091	227.9658	235.6727	261.5143
Pgd	268.1046	264.7543	291.8537	216.9881	238.4737	233.0055	237.4153	243.1415	261.4962
Ltf	1.334395	5.020907	1.132346	59.49028	33.38905	172.3123	15.8186	3.522681	261.258
Klf6	227.0634	200.3673	170.6066	199.8814	169.852	167.3697	290.145	245.5755	261.1674
Smdt1	226.0167	220.5948	222.2519	187.5278	249.6426	250.29	194.8132	247.5814	260.2129
Cox7c	212.1216	227.1958	223.2598	242.8558	269.4022	276.8895	185.4209	280.2526	259.8329
Cxcl1	212.5223	198.4667	75.76448	122.1464	132.6876	133.0805	355.5476	241.4729	259.7055
Max	230.4786	212.1388	240.7087	253.3697	237.2271	236.4589	255.0557	266.356	259.4169
Capza2	240.3052	226.9553	240.8191	205.9266	222.4483	201.7402	208.7611	236.5958	258.9454
Nrros	263.7632	233.4865	271.9876	257.5786	254.608	224.8736	287.3368	258.0404	258.4608
Gpsm3	227.6552	244.7108	286.4702	234.7538	292.3777	273.4006	263.494	265.3665	258.1282
Atp5j	216.5372	219.692	208.5122	249.4831	254.1675	271.9583	201.7733	245.5626	256.5153
Picalm	303.3848	276.0584	296.6889	211.5081	255.3262	222.6165	298.6387	259.9563	256.0709
Tmem50a	262.5215	266.4637	282.0284	238.0644	310.3363	275.3751	266.8853	293.3858	255.7504
Lrp1	432.8353	351.2905	423.6449	323.2616	273.0942	215.8002	435.8027	256.3558	255.3169
Arf5	230.4403	249.6324	259.3272	241.44	252.9605	237.3508	245.8683	262.9768	255.315
G0s2	92.65982	61.52131	98.52416	94.62578	183.5738	183.2976	100.9342	184.8326	254.4093
Gm9844	230.1378	206.7585	247.0542	274.5953	260.4435	306.7427	238.3404	274.5575	254.1967
C3ar1	280.7624	252.6976	321.0339	305.0031	273.3025	234.573	314.226	277.9582	253.8506
Asxl1	290.4239	293.8124	286.8806	262.3596	247.756	228.2226	347.9776	234.1725	252.3696
Rpl21	201.0227	224.0217	217.5958	234.1435	260.5509	302.4941	187.0521	250.5177	252.1164
Bola2	214.3495	226.412	198.0866	221.328	237.9854	234.4304	188.0463	247.78	252.1144
Ms4a4c	151.891	104.0665	177.4755	196.8369	192.7528	156.5311	109.0444	238.7523	251.6729
Ndufa13	191.3775	197.9862	199.3678	197.2377	227.7928	256.3369	208.4344	241.3139	251.294
1810058I24Rik	225.3547	255.1945	271.2177	265.3883	273.9047	275.2152	256.9102	259.0533	250.4531
Atp5pb	232.1072	242.4499	237.8985	219.053	232.1743	229.7321	214.8044	250.3796	250.2677
Cd37	307.7689	307.2255	327.3624	259.9983	299.6167	276.8951	315.6157	301.0621	249.9886
Ier2	212.5369	194.8971	168.7014	170.5983	216.3633	226.0211	207.4323	231.2788	249.6758
Mob1a	282.94	261.9395	287.9778	208.8987	225.425	182.1272	249.4407	222.8556	249.5738
Folr2	266.4836	267.0576	270.1697	314.6927	252.2763	290.42	290.4269	231.6156	249.2238
Sec61b	196.8453	188.7088	175.6747	213.3196	241.611	247.328	200.9582	265.3467	248.9675
Trem1	258.2389	203.1353	162.7586	69.94892	177.6654	133.9064	83.43761	177.6753	248.9529
Ifrd1	159.4598	127.9245	87.49886	134.0479	142.7406	118.8218	281.9639	230.3757	248.5808
Atp5k	159.7921	154.363	146.7649	182.5943	190.4094	247.0578	163.0789	188.2017	248.1684
Il2rg	353.3627	308.376	354.7788	246.2691	249.5886	203.6454	233.2279	242.4956	246.6794
Lyn	279.9407	239.1206	298.3258	218.3947	249.8111	210.5142	247.6251	243.3926	246.4516
Nucb1	269.6643	273.704	307.145	263.4561	262.8021	257.836	295.6089	259.7453	246.2876
Plau	322.1354	200.157	218.5548	298.9656	155.493	175.4966	257.5957	231.7766	245.5665
Por	286.7379	192.1294	192.8872	260.3453	207.8243	218.1385	276.2081	222.5297	245.3513
Plbd1	209.0586	148.8684	181.8394	197.8449	241.2558	229.8892	177.0971	232.8016	245.3186
Plp2	340.0073	362.9483	339.9031	194.3996	211.3313	203.1199	227.2606	238.6388	245.2349
Hexb	270.2933	261.3019	314.0691	288.8046	268.0395	249.8242	343.6677	253.7438	244.8549
Cerk	278.0402	284.0483	308.9331	287.0467	270.624	252.3468	368.0789	252.3662	244.591

Tmem256	157.0531	165.3583	166.5908	175.2313	209.4794	191.8661	172.8246	223.2539	244.4496
Tma7	188.5939	210.2253	214.2055	243.0977	239.3758	257.3027	194.7413	243.4457	244.3626
Nedd8	235.325	240.9902	257.1478	244.0819	255.8001	243.0781	216.6457	260.1812	244.2363
Sema4d	181.7783	184.8506	240.0593	224.3918	195.3584	159.1476	219.1057	231.8926	243.4742
Vasp	216.7069	179.2595	212.7564	160.6391	214.1358	204.4757	185.2941	213.5754	243.3493
Gltp	265.8359	277.7144	270.1081	265.7072	288.0635	266.2046	330.2147	264.01	242.3154
Clec4a2	252.3072	288.9824	297.7169	176.5415	231.582	196.6096	164.5821	251.6603	242.0406
Il1r2	202.8364	176.2716	125.975	60.7705	157.5181	142.9028	91.6367	172.2011	241.5358
Ppp2ca	257.6003	254.1356	238.4688	204.0575	206.8447	202.6523	223.8779	234.1299	241.4094
Atp6v0d1	280.8862	237.0453	240.1923	229.8985	246.3935	241.4228	262.2512	242.1916	239.6576
Lasp1	283.0709	226.8629	264.6223	231.7806	208.0656	204.4467	257.0855	228.6771	239.4356
Csf1	245.8215	226.1342	225.7145	163.0816	143.3105	105.6893	135.528	197.2302	238.6322
Ssbp4	232.6989	221.6995	242.1809	245.2495	238.8377	255.6851	245.4343	243.8179	238.4204
Atpif1	228.0047	270.7882	219.0683	234.6547	264.4892	290.0655	164.0317	228.2445	236.6261
Cyrib	222.3253	205.6697	221.597	191.7766	221.1911	213.4096	229.2544	250.9321	236.3309
Mrpl52	198.4736	187.3434	241.0221	235.4962	227.0853	266.0429	230.1655	255.7131	235.7164
Mlf2	257.1336	255.7593	273.5092	241.6852	254.4941	248.7293	249.884	242.8063	235.6347
M6pr	284.7731	270.4946	277.8619	226.0557	209.8718	198.2815	252.0201	226.1776	235.1527
Psmb2	239.1799	199.3604	238.9072	210.2764	217.3471	228.7581	243.4001	238.5939	234.4749
Slc7a8	274.3256	200.7679	233.9893	225.2449	212.3234	179.1801	304.7862	227.0812	234.2659
Lat2	216.4336	204.1643	194.0558	244.2906	210.4919	235.5499	232.5224	225.9518	233.5502
P2ry6	293.8538	270.932	339.2225	240.9041	303.5018	269.7801	344.9924	261.4993	233.2009
Epn1	260.8206	241.6694	280.6411	244.2765	274.7766	247.1256	281.1908	266.0226	232.7543
Ndufb7	198.2457	178.2158	231.1401	254.4347	263.4977	276.8246	244.2422	213.0114	231.8699
Snx2	256.1893	222.2689	259.8413	212.1161	217.3422	194.076	231.6071	235.0183	231.8109
Gm10076	196.0031	210.637	240.4941	201.0616	218.1628	232.2647	187.0184	231.6266	231.4654
Ifi211	148.9517	93.16343	160.393	205.2642	170.7735	152.9101	123.2537	176.2316	231.333
Lamtor1	249.3778	248.574	252.3987	241.3805	244.0489	238.5273	263.8317	232.0025	231.3084
Rnpep	223.5037	205.6684	241.7827	213.2153	239.5036	221.877	240.4995	221.4254	230.8733
Dnm2	262.0792	241.2947	269.2623	235.5365	231.914	219.1718	283.9148	239.5309	230.831
Gm10925	271.2476	369.7398	179.6044	330.7651	351.448	312.7235	152.1216	267.0126	230.8242
Rps10-ps2	250.6723	229.4028	239.5549	250.5102	258.8687	282.7259	232.5683	268.7606	230.3967
Olfm1	323.3245	277.221	318.5719	213.9955	290.8904	274.8685	219.007	249.236	230.3962
Acp5	304.1513	458.8741	436.3438	155.9706	229.5744	205.4485	173.2915	231.5329	230.1727
Clec4a3	179.6516	172.2147	223.2021	234.528	275.7009	216.1307	223.2562	268.6573	229.8065
Pcbp2	268.9666	243.5011	267.6992	231.4854	244.069	239.0921	267.254	255.6705	229.1225
Hnrnpa1	265.6756	219.8256	215.0778	201.815	197.9948	195.8151	251.0466	229.1891	228.9089
Lrrc25	228.7637	197.5777	223.8251	226.0099	256.3615	226.2128	246.836	234.1004	228.6992
Sgk1	206.2296	209.0441	172.1783	229.1008	163.1697	171.9166	349.5178	213.703	228.6979
Vdac2	256.9097	228.8134	244.2294	204.7878	220.6295	216.9456	228.2612	236.092	228.3898
Scpep1	303.0917	268.7213	287.7821	252.1406	246.7838	259.4515	288.0095	225.967	228.2912
Akt1	247.2546	230.8205	248.5102	203.4968	213.5925	218.9014	228.5692	227.1736	228.1484
Isg15	119.0158	53.24268	109.4222	178.9457	157.4181	118.9458	89.13023	128.6049	227.8438
Sbno2	288.5556	230.6249	262.5899	198.6739	257.9442	223.4982	283.9121	234.7057	227.7449
Ndufa4	198.6624	214.8207	204.8212	228.8291	227.6608	221.0584	192.2999	231.5207	227.6154
Hba-a1	116.3307	138.6713	69.72717	343.3883	328.4092	488.8286	203.9768	125.2049	227.6073
Oasl2	162.8818	101.7843	180.8344	191.1156	181.437	160.3621	151.1033	183.4414	227.4288
Psmb1	202.06	187.4859	201.7088	203.3227	220.1131	219.5194	189.2604	216.9889	227.415
Fosb	249.7762	210.4262	140.1361	208.3202	178.4349	156.2696	348.0113	228.7385	227.2037
Cd300lf	161.2277	117.6524	146.653	189.4466	197.1996	203.2165	208.2164	201.2485	226.7748
Tgif1	177.9642	161.344	141.3031	161.7474	170.3329	152.2418	256.3048	230.5217	226.3585
Atp5g2	221.5809	254.8318	236.8603	217.954	270.3582	254.356	254.52	251.92	225.9677
Fkbp1a	267.2804	287.3386	248.4948	215.4401	229.5235	220.4763	237.7273	242.2729	225.7909
Adam15	302.874	289.7143	314.0369	235.3521	256.1471	238.9849	296.0464	244.1651	225.6859

Oasl1	138.3434	74.25996	146.5279	199.5383	146.0886	116.3475	126.044	160.2648	225.532
Ssr2	232.5303	206.1127	212.9887	207.6488	220.5645	217.848	220.4516	211.8666	225.2569
Psmb8	165.3169	140.8257	178.3926	216.0927	217.9723	214.5433	180.1397	204.8719	225.0882
Asah1	258.6945	253.8246	284.2024	236.5236	268.479	229.1296	286.1587	251.8438	225.076
Hspa1b	188.6275	190.9194	188.2548	249.5452	249.0741	284.7985	267.8529	229.5568	224.982
Vps25	237.0362	228.0168	251.9876	217.6323	219.8937	233.2541	240.2217	233.7755	224.8822
Mbd2	219.719	213.8367	241.5322	224.7189	199.8522	204.6858	206.4615	222.7847	224.8753
Tcf25	244.0776	234.7411	235.6204	219.4399	209.8357	213.0316	275.9099	215.7528	224.7796
Snrnp70	241.2173	253.6418	234.0574	249.6931	244.3495	221.6163	294.9629	233.6186	224.7641
Ifi207	218.8881	170.5213	211.3862	247.5201	193.6151	171.0335	288.3813	219.9472	224.5741
Smap2	265.2554	254.0545	241.687	220.6913	245.0775	224.4896	295.2942	252.5925	224.2442
Ndufa7	189.21	184.1857	200.7393	211.38	218.6106	237.0982	201.4439	232.0437	223.8666
Uqcr11	203.8239	226.488	205.2464	230.3724	225.4839	273.3869	237.8656	227.5941	223.8318
Marcksl1	192.2153	153.8878	126.1614	84.1547	163.6724	110.7324	105.8277	201.6679	223.7916
Rtp4	133.0706	89.93086	165.5648	209.8094	186.2592	166.5079	140.6135	169.0503	223.7119
Galns	238.3786	204.6816	232.2854	264.9874	247.624	250.8187	312.9515	223.9977	223.4531
Ehbp1l1	233.2164	217.0424	225.0097	209.4455	212.2648	208.7334	241.8249	221.8843	223.3739
Wdr1	268.6214	239.3639	249.8249	206.991	205.9128	198.7747	234.0419	204.084	223.2818
Dbi	236.2965	239.6234	214.4106	239.1051	218.66	264.8087	216.3073	246.558	222.7812
Lamtor2	196.2148	201.3239	204.965	222.5519	208.0167	242.3333	203.0631	216.7502	222.7243
H2az1	211.4962	228.8473	220.78	190.8515	209.7161	237.1117	179.2855	209.7047	222.604
Nisch	278.8455	263.7634	254.1871	280.6745	233.1495	194.5394	350.0125	218.9143	222.5823
Hspa1a	170.0589	169.0134	163.4206	220.983	225.064	248.9162	232.5914	226.8439	222.3967
Rnf13	235.2052	246.915	252.2848	218.256	236.4908	202.9277	246.1013	221.5925	222.058
Rpl15	195.5539	196.565	192.565	185.5651	203.0495	217.3782	187.6424	227.2427	221.9687
Ccnl1	177.7647	173.7482	144.8868	174.5324	175.7519	158.4113	245.0421	197.697	221.6053
Atp1b3	255.8134	265.4827	262.0825	181.0241	195.9039	176.2504	195.0577	224.0229	221.128
Nfkbiz	191.1167	181.511	114.6833	114.6407	145.1773	113.1771	220.5164	192.2113	221.0673
Cd300c2	178.7257	202.4243	221.1939	207.8777	242.9014	224.6943	224.7148	244.3564	220.6275
Adgre5	310.0792	290.4195	332.2182	209.8564	232.8481	228.4814	309.2353	249.9379	220.4558
Derl2	243.7735	225.3087	244.1098	243.633	240.3803	219.0257	214.39	225.3518	220.1558
Brk1	217.2463	228.6349	229.5251	178.4722	223.8275	222.2055	209.7787	225.398	219.8904
Atp6v1e1	214.0538	220.4804	212.4841	188.2127	196.357	199.7195	193.5985	210.5661	219.5118
Swi5	191.5451	201.2225	175.9381	200.8899	177.5197	195.3005	181.549	190.0169	219.088
Gm10275	170.5114	186.5399	159.5123	207.2934	176.8837	213.2367	156.8917	200.8897	218.9335
Uqcr10	179.2623	182.2956	187.3295	203.1343	216.582	243.4329	195.8007	198.802	218.8978
Ptprc	228.5694	214.3515	253.2456	198.212	213.8979	181.5074	221.0975	213.7891	218.5749
Rab1b	234.0785	230.9846	226.0686	198.2488	215.9995	221.2739	215.7652	216.9403	218.0299
Rab3il1	244.2256	194.2307	254.1036	239.719	281.4163	280.2004	293.9203	226.0993	218.0065
Grk2	201.7197	199.9555	206.8626	195.2222	220.8041	214.0061	234.6522	223.1735	217.964
Napa	223.7464	197.4944	220.3022	221.0653	212.8278	203.5205	236.1585	206.801	217.9258
Tgm2	246.5424	196.6559	282.7529	183.7584	188.745	174.8494	268.8114	188.8806	217.7961
Emilin2	296.0407	209.321	263.1224	174.7904	187.52	163.3034	200.9928	220.9255	217.7554
Dok2	239.7042	225.6057	227.6228	184.1009	208.3961	187.9605	195.8544	233.4479	217.7504
Colgalt1	286.4251	306.8519	291.3686	216.5288	226.6137	202.1778	272.1127	244.185	217.728
Myh9	271.5384	217.4139	265.6638	202.2479	198.341	176.2122	235.3418	196.5765	217.2836
Cope	194.2158	188.0812	191.3696	204.7964	206.3765	218.3325	204.4509	215.2512	217.0658
Hnrnpk	242.0202	213.0094	220.0801	181.4519	190.9732	183.8842	219.0972	205.1033	216.883
Atp5mpl	170.0656	189.8445	171.7529	193.2917	198.7556	209.0527	155.2359	152.5268	216.6466
Fmnl1	249.5888	203.4496	234.8679	179.9459	201.6812	196.2397	210.5633	228.2485	216.5857
Anxa6	256.0721	223.3812	252.6994	212.9995	235.7088	243.3964	235.8926	217.4043	216.2234
Clcn4	275.5538	239.014	250.9324	200.2696	212.5582	197.597	244.838	218.5311	215.8339
Gm2a	243.865	246.0601	285.9618	215.7964	274.5106	229.5772	251.266	261.7673	214.9987
Cnot2	227.8848	202.8133	237.6018	207.338	219.2544	184.7667	254.5451	212.9983	214.8565

Stat1	160.092	117.0055	194.6903	184.5996	177.0333	157.9767	154.9215	169.1269	214.7217
TrnP	172.1062	400.237	203.5724	526.5913	341.2208	328.2643	327.5393	230.8248	214.5041
Adap2	188.5269	197.1388	217.2319	291.9475	232.2603	209.5332	336.765	222.5966	214.1634
Ostf1	199.8552	180.4198	207.1244	206.3262	193.944	211.0517	202.8831	212.608	213.6308
Themis2	179.6612	156.0401	190.0207	218.9703	224.7991	194.6628	221.6409	211.5036	213.5529
Stk4	215.2754	212.0823	232.423	192.541	193.7243	171.3939	224.2767	206.0414	213.0763
Anpep	354.1209	334.9282	354.499	285.3694	248.8957	241.4878	308.5522	213.3896	212.9135
Drap1	181.242	182.4752	210.456	215.5437	221.659	256.6962	204.9307	233.532	212.8834
Fis1	186.4863	169.3183	183.6864	186.9847	214.0829	229.203	202.5113	206.1605	211.9546
Atp5o	208.9699	200.034	186.5147	203.5148	218.7859	249.2976	209.3752	196.9771	211.6918
Tmem189	254.3303	209.6485	231.8233	214.8028	186.876	183.4166	250.8979	211.1609	211.4118
Gngt2	145.2784	143.7989	194.2749	210.0511	235.5068	236.0731	172.347	214.2593	211.2452
Tubb6	263.8394	218.9427	228.3589	180.176	168.9919	183.4338	244.4036	201.5358	211.1678
Socs3	245.0273	217.6202	175.9328	131.4424	226.1721	170.2783	304.7574	221.698	211.0982
Cd24a	149.6767	132.076	148.0148	145.1044	170.5488	173.9873	167.0666	196.5391	211.0877
Pnrc1	162.2535	161.4195	150.4346	140.8974	189.9104	164.7481	206.2431	202.7121	210.6908
Rps11	186.666	192.7819	179.9644	178.2945	217.4215	227.7226	170.4663	214.1986	210.6474
C3	210.0862	138.7489	180.9906	117.6092	121.6623	122.6935	87.75262	174.7957	210.2951
Gas5	168.8698	204.4963	172.4285	198.2245	181.9867	196.525	177.7864	187.1719	210.038
Hnrnpl	235.8824	215.4724	206.71	202.7646	207.8231	199.1447	247.3641	205.4478	209.9555
Oas2	154.0847	122.4356	165.1791	215.1432	197.5862	156.9757	213.747	183.3277	209.8926
Tnfaip3	204.1762	177.6638	98.73136	130.9612	133.0739	114.9586	256.9757	182.3837	209.8267
Degs1	257.8779	243.7895	246.6437	212.3443	178.489	196.0347	221.8117	208.4722	209.7719
Rnf149	204.1229	213.5318	222.813	138.1128	179.0929	165.9354	190.5732	185.7943	209.1656
Brd2	217.5981	207.3393	220.9396	210.1552	201.9689	185.0346	245.2878	201.5879	208.4556
Clec4n	164.5196	178.9322	190.83	94.58012	122.0419	92.80159	79.70589	168.9686	208.1599
Ier5	131.5255	113.8274	104.4798	202.8255	176.6896	160.4183	214.3506	208.3697	207.8594
Myadm	337.8108	348.6349	332.9053	193.9928	223.6944	180.152	224.3693	243.5329	207.8107
F13a1	370.7043	312.7627	339.8458	169.2303	191.1381	147.2973	214.2208	275.485	207.552
Uqcrh	156.973	190.2762	179.8392	186.0806	199.687	226.5718	148.702	196.3392	207.4787
Gm15427	198.6605	190.1562	175.8588	203.7127	218.4681	228.4182	179.1375	218.0592	207.4103
Ifitm6	133.4203	65.10172	128.7214	85.39162	95.93536	131.1535	48.18963	112.0042	207.3453
Actr2	219.1534	198.6328	223.6821	172.2641	165.3176	154.5567	197.4275	194.4133	206.9717
Ywhaz	240.4012	222.4742	208.1625	167.7552	189.6907	162.5793	194.6138	196.6147	205.6478
Ptbp1	269.988	230.2805	236.3044	204.0574	201.6484	200.3147	267.9628	224.9017	204.7711
Jpt1	205.029	203.8065	200.9813	193.3234	212.8289	205.6232	213.104	197.4118	204.3503
Tnfaip2	202.2437	191.4593	198.9061	160.7094	158.2917	141.1938	200.9243	168.4409	203.381
Mapkapk2	203.9101	186.1504	192.7136	159.3341	173.3839	158.2485	179.6974	195.9073	203.2519
Syk	234.6869	240.0793	264.7561	207.6338	222.9576	194.0028	285.9753	208.8538	203.1853
Atp6v1g1	176.8134	170.7277	169.2757	178.5466	177.2835	205.0695	181.3825	193.1655	203.1799
Serp1	166.9927	163.0516	176.4452	171.1855	200.1651	187.3529	185.1958	211.3452	203.1362
Ranbp1	238.5398	220.0587	187.418	211.9045	182.3163	187.6194	187.2273	195.8899	203.0395
Ssr4	173.1236	166.3516	153.8138	191.51	204.7747	202.398	155.188	196.8537	202.9585
Mndal	155.883	127.369	206.9907	237.5718	202.993	168.1366	197.218	199.3111	202.6776
Pomp	169.6105	150.6292	161.3885	173.2792	196.0683	212.4825	160.2021	198.7902	202.6386
Ywhah	280.5216	277.7688	300.2615	208.8533	208.0217	207.3946	240.788	200.2083	202.124
St13	213.8228	184.8243	181.8525	183.6678	186.1948	186.99	201.1436	202.0833	201.9353
Ehd4	212.5608	185.8011	214.5942	227.6359	209.1356	195.2316	240.0816	208.3263	201.8755
Rbis	147.5123	166.8366	167.3014	190.1281	201.1877	178.9884	131.4568	219.4224	200.8085
Rsu1	212.8482	185.5349	211.3181	198.164	202.9499	213.2383	224.335	182.6361	200.2702
Snx1	204.4995	172.1744	199.5095	186.6752	179.5508	202.0364	189.3122	192.4186	200.0146
Tcn2	203.2729	212.9991	241.9137	199.8656	226.757	204.3047	234.409	205.4281	199.9069
Slc29a3	248.0265	217.4794	253.6212	242.0609	217.412	169.7861	264.9203	216.0973	199.7213
Psmb7	195.7081	186.835	192.3807	192.2801	167.6475	188.9614	192.1474	198.3728	199.3774

Inpp5d	247.6442	215.0359	254.6688	208.9344	219.729	193.5641	292.9284	218.1874	199.2265
Chchd2	152.1196	158.8234	154.6963	173.682	181.8941	199.8865	168.4939	192.801	198.9822
Wdr26	207.508	207.3704	197.82	197.6609	188.0034	168.6478	245.6765	199.6468	198.9231
Ppp2r1a	237.2091	226.2106	223.4984	194.8279	205.3903	203.3424	245.9293	201.5373	198.8897
Ube2m	205.8893	180.7068	189.3348	186.2874	182.129	184.7656	198.0469	182.805	198.7942
Rbm3	153.9119	140.7206	138.4666	159.5039	158.2383	185.1896	156.5975	175.5873	198.7805
Ubxn1	166.8402	176.9925	180.484	189.4943	186.7488	204.5345	171.3504	188.4444	198.3157
Csrnp1	211.0691	169.1249	125.561	112.0762	151.2918	127.4804	197.3488	171.7701	197.3675
Ywhab	230.1629	203.6881	210.9489	190.9896	202.7276	199.2315	213.1899	212.3852	197.2406
Snrpd2	117.2379	137.0932	118.274	157.0231	147.901	186.9999	148.9983	174.1389	197.0575
Sfpq	212.1865	201.5031	195.1067	179.4347	185.6578	176.2526	234.2103	197.7445	197.0524
Nfkbid	158.9723	153.7965	111.554	113.9192	158.1847	133.0749	188.0153	169.8746	196.985
Dnase1l1	196.1137	207.3088	198.5232	202.9861	183.2216	197.8265	219.2629	198.1964	196.1865
Fkbp8	177.5256	178.1582	189.1828	202.6204	196.6678	200.6993	198.2019	188.5709	195.5698
Bax	167.5263	147.2292	163.3986	175.2154	166.8412	188.5507	161.8718	204.2056	195.5409
Laptm4a	186.6935	201.927	211.1056	194.758	210.3764	183.0222	215.8494	213.7814	194.779
Hp	137.076	104.8279	127.0512	97.38174	107.1489	144.5586	56.29677	163.6718	194.7304
Rexo2	207.1481	188.5817	196.3139	182.389	184.6579	190.7644	170.6031	221.7413	194.5918
Eif4g2	240.6183	218.4935	226.9991	171.922	174.9273	164.3493	206.5981	183.3028	193.9836
Glul	211.0478	227.1499	238.3106	197.0804	222.1416	193.9467	270.3233	180.4043	193.9379
Syngr1	160.0113	132.4808	159.691	242.2781	191.8306	233.0848	235.3561	171.7043	193.8448
Fgr	206.7897	173.7632	228.3249	141.8485	178.5378	152.3386	137.3467	193.6651	193.3867
Septin9	229.7	217.8738	250.8643	207.0258	204.0923	192.5657	241.8686	198.9852	193.3568
Dars	189.9934	176.8019	167.3736	154.0866	142.2439	159.9424	145.1053	161.1655	193.2797
Stxbp2	187.9134	172.4851	191.0808	189.0087	184.6327	185.3405	212.9387	186.1277	193.1111
Ms4a4a	173.3831	164.4281	149.2994	149.6635	201.8115	173.1561	188.1745	182.9898	192.4639
Rpn2	247.2787	222.3819	248.3	196.9857	207.2158	201.3771	243.7908	197.1761	191.926
Cuta	206.9238	201.8354	205.8525	204.3405	235.2667	221.9769	225.7959	212.5738	191.5958
Pltp	302.8834	338.9679	340.1579	258.9712	385.8157	337.4393	337.6014	282.2956	191.104
Dap	215.9876	219.5566	228.0675	203.6108	197.968	188.8689	222.3828	175.7752	190.9781
Gnb1	212.8922	192.2019	207.3922	170.305	178.159	166.9549	202.4987	196.1711	190.9736
Copg1	240.9691	207.7963	212.2317	191.0291	197.6486	197.7039	242.1935	189.6437	190.6243
Rasa4	148.341	140.2814	163.6682	224.2695	183.0391	184.6657	224.548	194.1946	189.8779
Cnn2	168.3052	139.7155	168.7915	165.661	173.6547	187.7215	158.0357	160.4208	189.3785
Acod1	94.55317	54.67091	56.3443	49.05668	94.76411	69.39322	56.34599	120.0047	189.1162
Snrpb	186.2442	176.8703	184.881	173.3493	180.6739	176.4403	174.3986	179.5828	188.7252
Tlr2	169.2514	165.7938	147.8326	97.5467	135.8128	117.4473	163.0882	182.9237	187.8972
Cyp4f18	142.4166	156.209	191.7411	168.808	236.7223	204.7377	203.1576	233.2336	187.7865
Slc43a2	209.5305	179.8683	215.2003	211.6784	197.8036	169.3428	283.3053	192.5414	187.7813
Tnf	178.818	149.4048	96.62198	112.7624	121.4532	98.92529	175.107	178.5725	187.6798
Psenen	168.4602	190.3062	176.5282	172.9404	190.7562	178.0301	163.1145	206.4476	187.4023
Cox5a	199.113	172.9722	193.5357	178.0309	174.3954	191.0135	175.6973	180.74	186.8751
Ddx17	219.1909	218.6939	240.3174	218.8481	204.9512	171.7076	235.7878	194.2866	186.648
Rgs10	171.8263	200.3727	212.9069	199.9712	230.4433	221.5337	233.6449	241.6532	186.5915
Cct5	190.4637	166.809	160.5452	166.6972	157.4725	178.2812	169.313	173.9276	186.4569
Ccr5	177.5117	148.2601	202.1091	183.413	175.3578	137.5774	167.4097	180.0304	186.4478
Tnfrsf1a	213.1085	181.0603	199.6774	165.1268	185.7384	164.1508	176.6799	181.2974	186.3162
Fam120a	243.4847	237.7682	235.3311	210.1737	193.4299	167.4557	262.8745	188.1658	186.3069
Xdh	204.0253	189.2614	207.8114	208.3021	166.3305	163.6449	240.1357	182.1965	186.2514
Hmga1	229.778	211.6778	192.2683	152.5731	149.4676	139.8315	169.6634	175.6526	186.1843
Calm3	197.6487	194.7437	202.945	184.6073	189.3624	189.4664	186.8154	183.7824	185.9838
Grb2	184.9102	170.0826	183.4876	184.0702	193.5463	192.4247	204.8392	179.2604	185.8484
Cox7b	142.1673	156.9785	140.5776	172.8724	167.2571	174.9032	143.7888	159.1911	185.7491
Ap2m1	201.0221	180.5665	185.9122	189.3821	181.0392	182.3132	200.0445	184.0273	185.2283

Atp13a2	200.4595	197.6854	227.6194	215.2766	226.4304	203.645	266.3735	206.5315	184.8315
Psmb3	165.8592	156.1143	161.7068	159.8019	173.1303	186.905	163.8989	196.0389	184.8225
Srrm2	247.1706	233.9586	235.2874	239.5065	205.8853	155.7194	297.2368	191.4712	184.7847
Mpp1	181.9466	163.7474	171.867	177.4779	165.4426	163.5074	201.394	171.266	184.6484
Gstm1	227.7232	220.5664	257.2405	217.2216	191.5098	174.1052	204.8867	201.4655	184.4712
Tbca	113.4968	130.385	122.1795	138.7209	154.1719	169.8349	108.8662	155.6603	184.4506
Rpl23a-ps3	137.7111	151.7635	156.4513	152.0403	172.4622	177.3678	172.109	163.5959	184.3857
Actn4	229.3904	194.4205	204.4139	212.7573	171.7667	176.8552	221.6841	187.1354	184.3825
Map7d1	228.9163	214.5759	234.6362	202.5871	190.9925	179.2172	224.6299	190.3171	184.1379
Tpm1	196.4664	229.4888	201.4038	243.7092	170.0733	270.8965	150.888	173.3827	184.0215
Cers2	226.4744	192.4523	212.7063	179.8144	160.4031	157.9223	195.5798	188.1931	183.9647
Gla	174.9455	167.6917	167.8166	182.1704	151.1532	149.3716	178.2605	164.1035	183.8799
Sh3bgrl	183.536	191.3136	181.8173	151.7951	167.2763	147.457	161.085	205.6953	183.6454
Slc6a6	215.9261	196.1451	209.0512	192.5345	183.325	160.6784	247.5877	193.0369	183.4597
Rpl14-ps1	204.4179	194.5524	189.0226	168.4011	189.3598	201.6714	183.8655	186.3358	183.2559
H2-DMa	199.5503	206.2221	229.2687	211.965	326.0903	270.2515	233.2008	252.0379	183.2419
Pcna	212.2486	189.699	194.9798	199.3341	168.7041	200.6345	197.9055	158.8501	182.8761
Mknk2	174.1405	181.6162	171.8981	169.3655	182.385	172.0278	226.1608	181.1645	182.5989
Zfp207	184.3127	190.5249	200.8921	175.6517	169.1991	157.2281	211.7756	187.4698	182.1968
Atp6v0d2	263.1259	319.0854	269.6247	223.3513	185.6283	164.9036	223.4233	196.3263	181.4795
Ndufb9	156.1376	162.1016	152.3755	144.8543	165.0418	175.5818	142.4436	183.0777	181.1436
Itgb1	226.6034	217.5629	231.693	170.3643	163.4314	156.9926	191.8814	169.0323	180.8616
Srsf2	167.1008	172.3505	172.1606	196.0894	180.5918	173.9266	201.1	194.6822	180.7244
Caprin1	209.1296	198.7199	194.6584	162.8123	153.1099	148.3616	199.281	174.2456	180.6688
Get3	180.2614	158.2602	166.1581	170.8779	168.5044	178.1785	183.0907	172.2112	180.6671
Sdf2l1	175.4705	163.576	160.1786	188.4685	176.7896	216.1744	195.8888	166.5887	180.4006
Cdk2ap2	146.78	138.8634	149.475	133.0685	169.5815	169.3906	137.2385	173.7013	180.1459
Ppp1r18	184.316	168.1493	177.3809	160.2002	183.8064	170.9105	184.5339	184.2247	180.1221
Arl8a	213.2183	180.295	178.1261	192.5253	186.129	167.2064	217.8805	194.5384	179.8086
Ptp4a2	187.4206	192.0492	191.9415	169.2842	193.5573	167.1429	194.7223	183.3378	179.789
Dgkz	199.8129	164.8279	170.1538	220.378	165.0391	167.3332	255.6954	179.726	179.7706
Ilk	184.1607	185.9111	201.8724	180.4545	182.5048	194.0962	197.7304	166.2708	179.5326
Wbp2	197.0289	187.7472	199.0837	192.7751	188.5336	175.6784	224.3164	177.4933	179.1221
Cd93	241.1113	212.6409	238.9677	213.7334	178.5751	153.1688	256.9865	184.5001	178.9849
Psma4	146.9412	137.6208	136.9419	139.2272	155.2163	163.6721	127.143	166.6789	178.8083
Malat1	224.2292	244.2504	208.2881	312.8761	185.8348	149.2062	365.8112	174.8859	178.7225
Ypel3	139.774	171.1774	187.556	168.3884	242.1711	213.8471	207.1976	202.3232	178.6791
Vcan	300.0613	180.5024	235.4282	88.76341	118.9907	76.59306	70.21109	221.2635	178.6736
Mtch1	186.6885	183.8782	184.2995	184.8287	189.408	195.5647	177.2055	166.7909	178.5809
Tmed10	223.1201	200.4476	213.6956	178.4687	173.1004	158.0332	206.4418	178.9604	178.5772
Psma7	130.1513	128.3529	133.1367	151.2533	163.8864	162.5262	126.814	183.1862	178.5482
Rsrp1	136.7342	163.3316	147.6822	214.3545	206.7541	191.519	230.3258	166.8896	178.3776
Cd180	209.717	140.4499	185.3134	242.934	162.8118	154.3411	221.7548	177.1638	178.3141
Hnrnpdl	162.0157	159.8215	143.6334	173.4919	158.4723	146.0006	193.8167	172.5757	178.2688
Rnf130	177.4328	156.9075	185.2025	193.3894	199.3876	178.8447	198.8413	194.2365	178.1554
Rgs2	136.0987	137.4085	133.1479	200.4854	179.4696	164.8428	207.8076	202.517	177.7928
Nono	197.5079	193.9092	206.1456	185.5103	182.4497	174.3715	189.4902	183.1434	177.4393
Hsp90aa1	189.1897	190.432	156.762	151.3782	142.4266	160.5659	171.4316	158.7549	177.4224
Hck	141.9077	113.8053	155.8648	143.8533	149.1838	151.229	143.6011	161.804	177.3286
Acta1	227.4046	307.1776	220.0559	267.4113	218.5384	347.8982	128.0597	178.1275	177.0998
Plk3	131.4048	101.4187	108.2387	79.93315	101.9292	91.8173	106.1236	133.5541	176.9329
Ndufv3	174.38	167.3443	174.4002	186.5073	202.4307	214.1044	204.8873	199.773	176.9187
Macroh2a1	201.1691	167.4876	192.8331	187.1791	193.27	185.9034	203.5989	173.8573	175.9892
Cpt1a	206.4015	208.6997	227.0935	180.0851	174.5235	149.9068	218.1473	168.9524	175.9181

Cyth1	197.2423	191.2438	204.2423	160.434	157.6871	142.0175	202.4658	164.3253	175.8883
Btg2	147.4332	133.3212	117.1654	113.3127	156.0153	127.4451	153.2345	173.5333	175.6162
Rtn4	199.7194	186.9328	194.132	188.8931	171.8416	166.5132	205.2989	169.3525	175.457
Gm10123	154.9269	164.6012	174.5753	171.4168	150.9041	169.7366	134.418	165.4802	175.4344
Psme2	138.6299	121.6113	141.2896	150.5113	164.5568	158.8839	138.4642	172.7185	175.2849
Cenpb	176.3884	163.663	171.0221	165.9975	164.8365	168.5976	181.3165	170.3892	174.9585
Sdhb	176.5441	168.1387	177.9081	175.0415	184.9181	172.5317	175.365	172.1702	174.8608
Smox	155.2986	102.3156	150.2716	105.5459	130.3052	129.8583	92.46419	136.1954	174.8491
Timp2	230.844	236.8392	257.7817	211.8803	212.7833	202.4085	257.3506	190.626	174.7777
Glud1	203.3022	199.0317	204.5548	155.1705	164.4648	153.0391	186.2276	182.5157	174.5575
Tecr	212.1279	209.6532	219.9119	174.953	206.7517	200.5286	208.9423	189.9047	174.1903
Slc25a39	214.1854	209.2129	215.3664	191.3526	196.5309	190.3454	209.4228	182.5123	174.088
Cdc37	208.4318	181.4457	193.3253	175.2045	180.6547	210.7945	189.6873	195.2686	173.8927
Pgk1	185.5125	159.691	179.0022	140.915	154.3166	153.2965	163.9508	163.8136	173.8857
Pgls	170.6463	180.962	171.1056	166.3191	199.953	193.5155	170.8558	186.0238	173.7112
Cmtm3	197.8517	196.6197	202.8251	180.9223	187.0175	174.5641	205.0742	184.2337	173.4738
Psma6	154.1131	151.2218	157.152	149.3626	139.8937	147.6128	138.6538	154.4	173.1328
Igsf6	169.3291	174.3218	177.0689	150.806	167.3565	161.878	168.8048	184.9242	173.1026
Lrrfip1	199.0084	171.3847	193.4766	142.7662	138.1388	126.148	158.6442	172.7586	173.0387
Cenpx	168.8742	181.9831	171.1893	184.7825	203.7959	208.8248	186.9484	172.8038	172.9836
Id2	159.8203	125.4033	122.9194	163.2156	150.3017	167.7475	209.5521	183.5768	172.8846
Arl1	165.0632	169.877	178.0546	149.4728	148.112	159.4647	158.8965	164.4907	172.757
Ociad1	167.8812	171.6581	169.2561	164.8351	161.905	154.9771	164.3214	159.9603	172.5591
Blvrb	144.8933	131.7319	170.1769	208.4797	205.5011	186.3132	196.5344	164.5133	172.2811
Cmtm7	190.2946	179.3478	198.7563	163.7893	181.5397	169.5577	199.8254	188.1965	172.256
Rabac1	148.2572	155.8243	159.1733	135.7712	160.5078	160.4816	137.8426	156.2361	172.099
Dynll1	170.5386	167.595	168.6654	149.5072	176.7878	186.3231	148.249	181.461	171.898
Ifngr2	160.0725	180.922	201.9181	147.548	162.3839	153.2176	164.1091	175.1475	171.6651
Gm4332	153.1147	161.4371	141.182	152.3951	177.3186	198.3881	148.1994	167.3429	171.6222
Pdpn	305.5869	264.7492	226.7988	107.8356	117.7116	106.8925	156.3917	132.484	171.6041
Uba1	214.2569	180.8765	208.1213	157.7937	164.457	153.4619	186.8242	179.0556	171.5397
Hpcal1	184.7582	166.2943	204.816	180.6314	187.9078	171.5121	197.5667	189.8352	171.5111
Ube2l3	235.5406	215.4429	235.7431	188.5174	168.5678	160.9063	224.8036	191.188	171.466
Bin2	178.821	156.2739	190.2806	200.6951	200.1163	204.4703	202.6025	202.9549	171.2969
Apbb1ip	191.3752	153.2154	168.2936	153.2511	164.9286	154.0514	179.9709	191.5143	171.1437
Uqcrc1	181.0776	168.4089	173.3645	181.1285	169.9694	167.7645	178.3989	171.7343	170.9877
Rbm39	139.8488	152.3427	138.3219	151.4462	157.0284	140.5642	173.4887	161.061	170.9097
Dok3	144.8346	156.2153	163.6079	143.7737	149.1065	155.626	156.5874	165.7561	170.7475
Glrx	147.6813	101.8113	121.9863	102.1775	134.11	118.9914	102.695	150.0952	170.684
Gm10036	107.8117	116.7861	205.8041	126.6917	205.2689	249.4669	57.20203	260.7295	170.6374
Slc38a10	198.5787	184.961	196.4197	186.7827	168.4707	173.2452	194.41	165.0514	170.5479
Raly	170.6682	159.9568	168.5858	161.8741	164.5443	172.4764	159.9068	172.6419	170.4605
Cd99	136.1731	162.3822	143.1868	183.3124	172.7488	225.4769	178.5695	158.3474	170.0543
Ubtf	193.198	169.0472	169.5704	165.4345	175.6473	161.0916	193.3673	177.4326	169.8558
Znhit1	166.7643	166.5448	177.4374	163.443	180.044	188.4892	167.7847	193.2024	169.809
Dop1b	202.7527	164.5179	190.0179	176.7859	154.1306	131.0236	211.5187	149.6903	169.7652
Hacd4	172.9153	202.9842	248.8868	178.3367	206.0759	160.663	209.3279	186.7687	169.3692
Bcl2a1b	135.3298	137.1406	136.8696	156.8681	171.6646	189.0988	200.318	168.9177	168.7477
Zfp36l1	182.1616	164.2038	170.254	172.9386	199.1449	161.6194	235.3366	202.4625	168.7468
Aif1	96.15611	81.21929	114.1849	170.2618	176.1116	170.3946	133.3674	165.5989	168.5632
Atp1a3	249.5791	258.529	268.49	178.5366	203.0986	181.3156	234.7786	213.5784	168.5366
Bak1	201.7691	188.2581	199.495	169.7311	163.4569	167.726	191.3748	171.7868	168.2989
Rab1a	169.6829	157.5788	163.0228	159.7285	149.0675	148.9401	167.6676	166.0414	167.9773
Cd83	158.5466	155.8963	107.3295	147.4287	131.2048	138.8592	300.2372	184.1203	167.7476

Cbr2	292.3098	247.6167	283.523	207.0579	238.1621	245.3598	302.6651	207.6573	167.487
Rnase4	194.1061	192.6085	232.7066	198.1621	198.2549	196.0759	284.1936	212.8985	167.4341
Ncl	190.4177	170.4037	164.7981	160.253	151.2458	154.2139	176.0513	173.6077	167.277
Ssr3	197.079	195.0403	194.2576	141.1532	155.2663	137.5068	180.2752	165.3546	167.1567
Ckb	197.8591	218.6929	213.9378	189.3657	177.0432	204.6186	177.8254	159.1688	167.1278
Ddost	209.5469	186.6152	193.9899	168.4758	180.5898	174.4505	208.5397	180.7605	167.0295
Ccr1	179.454	154.993	170.0938	100.0708	132.9383	106.8448	112.734	149.4726	166.9483
Mrpl42	178.2564	177.0169	179.1537	164.0752	164.6565	174.3699	176.3103	180.0445	166.6571
Ndufa6	136.7142	165.1803	165.4424	141.7565	172.6326	187.4921	129.4321	172.5798	166.4378
Adipor1	148.2786	157.214	168.819	142.8761	168.2015	150.1906	166.8853	158.4488	166.1595
Clec4a1	166.3854	148.3403	189.6074	152.8214	198.4523	137.3163	160.7139	196.1218	166.041
Srsf5	164.1359	144.2187	132.2753	141.9525	146.1557	143.73	151.1812	162.9793	165.9862
Ppib	148.9898	136.3832	140.5172	149.9928	158.2308	146.9665	137.5749	151.8596	165.9425
Epsti1	125.4348	102.3776	141.7372	160.0252	161.9993	136.8778	141.5448	178.1213	165.842
Atxn2l	200.2829	187.3325	207.0876	175.6672	183.1534	155.1469	218.4011	178.5861	165.7534
Pstpip1	161.0118	176.7243	179.0767	134.9267	141.8515	141.1042	125.9656	153.6004	165.527
Pdia4	201.7087	168.7017	185.1949	162.7162	177.2084	170.54	194.6192	164.3043	165.455
Rhoc	226.3196	282.8401	248.7652	177.4939	158.2958	160.3562	168.3129	174.692	165.367
C5ar1	210.7585	135.3625	147.8744	115.8545	107.2146	106.2956	114.8731	146.4974	165.2207
Hcfc1r1	180.4397	165.9893	190.6865	180.8982	183.0502	171.6747	176.4754	181.8688	165.2154
Gba	178.9865	150.4865	182.6044	174.6869	156.9507	150.2163	204.2748	167.4432	165.0466
Sun2	168.3675	157.6241	164.4207	136.6581	141.5999	159.0043	165.1497	128.8364	164.7315
Ybx3	234.5817	199.0791	191.5264	146.1629	135.2027	157.1477	155.6567	163.1951	164.4504
Tmem179b	165.3638	148.8104	183.8645	161.9174	194.2989	156.927	169.6021	178.3545	164.1482
Uap1l1	167.716	140.3732	143.6684	167.1748	158.1733	173.9572	186.8653	147.7337	163.8246
Necap2	160.7587	156.7337	180.0056	168.593	176.1246	160.3921	182.085	168.7599	163.5298
Cdk4	181.5526	168.9017	166.614	165.5685	166.4536	182.5914	185.2764	165.2456	163.4128
AI413582	142.0902	136.303	157.5347	158.3119	160.3471	175.4236	146.9818	157.3147	163.3365
Anp32b	181.4515	194.3009	158.3052	153.523	157.8029	160.256	157.0616	163.401	163.3213
Ndufb4	120.8892	139.0787	119.682	157.7327	143.975	164.9816	107.0236	137.0717	163.2839
Ifitm1	100.0253	83.23085	76.75414	48.3592	83.80134	83.46914	45.67528	76.82573	163.213
Sf1	215.8185	181.2206	181.2431	157.7306	172.2744	158.6885	206.4375	175.4558	163.1485
Itgal	153.9127	131.055	190.6129	111.7207	158.2955	124.6734	118.7742	146.0789	162.8668
Cyfip1	202.2725	177.5595	194.6918	157.4098	144.7027	127.3424	203.741	163.6134	162.5578
Nmt1	171.5098	154.1635	170.0411	171.2193	173.3741	167.3072	186.204	155.5933	162.3934
Abi1	163.7544	158.2124	165.1815	151.0189	153.9205	134.746	162.3884	165.3108	162.3809
Rpl22	123.5705	132.692	114.5458	142.3381	147.0661	162.946	118.9073	156.6313	162.2916
Glyr1	170.1332	161.0581	174.8538	167.6778	156.917	151.2019	192.2801	166.4985	162.152
Crlf2	130.3024	122.414	146.1111	140.4983	158.9926	158.1292	138.9056	154.6483	162.0161
Tnfaip8l2	120.6126	142.1401	167.973	155.1573	198.941	207.5043	175.642	183.6035	161.7317
Fnbp1	201.1346	181.7126	194.5461	178.67	180.8065	156.1596	213.7601	159.841	161.369
P2rx7	190.4207	220.0533	259.9917	195.5715	203.5194	172.6901	253.8201	192.3451	161.0945
Tuba1a	165.1992	187.5914	152.6031	152.9434	140.4423	149.5487	131.1984	158.1484	161.0694
Cwc15	132.4354	139.3781	136.9268	149.9258	151.0627	156.5168	142.0513	156.194	161.0348
Sell	183.6336	120.4402	152.334	86.89859	96.40829	112.2251	50.39118	141.6317	160.7357
Acin1	174.9089	171.3886	174.9055	174.2124	160.7022	152.4848	214.2234	163.117	160.6393
Xbp1	173.1285	174.8583	185.7803	138.7	158.277	158.2341	155.7701	149.7444	160.6295
Atp5g1	150.3492	143.8137	143.9946	140.4769	150.0959	148.181	150.2464	153.8152	160.4666
Hcar2	138.6948	137.36	96.96381	59.48789	122.8171	92.63413	61.32976	132.9457	160.4661
Rbms1	186.4008	166.9794	174.6669	154.2908	143.8788	121.2129	176.3952	153.1644	160.1217
Arhgef1	163.5109	148.2151	164.6513	153.9279	156.4541	156.3003	188.2774	155.4034	159.8886
Nop53	166.1363	164.5229	168.0061	158.3766	189.8784	186.6751	159.39	181.9945	159.8348
Ccnd3	154.1368	148.584	157.812	111.2369	143.5941	161.7493	119.5472	142.4709	159.5926
G6pdx	161.5718	144.8005	167.543	153.3102	155.1439	143.1671	165.6447	151.078	159.0998

Bag1	151.1088	137.4679	148.4137	141.8441	154.5602	162.163	157.5377	155.452	158.9822
Rps27l	127.4313	141.9738	104.789	110.4717	121.053	140.809	94.44602	135.0153	158.9274
Ndufs8	147.5048	140.3419	165.3434	162.9918	158.0591	179.632	152.9652	150.6051	158.9166
Pgam1	176.0582	151.1132	145.9958	149.2235	129.2586	134.6329	193.5997	138.9285	158.8295
Vps53	188.2493	159.2322	184.6695	152.1433	155.3817	149.8663	195.9186	159.802	158.6162
Canx	184.0288	174.6206	175.9154	153.5223	143.1261	132.502	183.2782	154.86	158.3631
Psma1	142.372	146.3105	150.9151	128.3527	141.82	141.0159	132.1735	141.4402	158.3438
Hk3	139.4955	114.3638	121.5603	177.4977	132.4709	147.3007	157.0448	114.1006	158.3296
Tsc22d3	89.661	111.4149	108.2252	92.05373	105.3317	141.2288	164.9798	137.8242	157.8628
Ppt1	161.628	147.7891	179.7339	135.93	153.4128	157.1471	153.8135	154.9253	157.8184
Adssl1	156.8733	144.1072	156.1795	150.9466	131.2153	145.186	152.8267	140.0459	157.5976
Hdc	102.0984	93.74039	69.25943	48.42034	92.66155	84.5271	50.49387	83.88014	157.4775
Pglyrp1	51.1235	35.56127	36.71876	48.0848	71.09592	160.427	48.80006	50.76728	157.3484
Sgpl1	181.2088	172.5942	175.93	159.2557	148.4767	136.382	196.7236	164.0118	157.3404
Bcap31	181.9506	176.2827	175.3521	165.6034	167.4071	160.0873	190.3965	158.8972	157.2844
Cuedc2	132.8224	139.0203	141.8961	145.1922	147.1631	156.7015	132.8038	140.5795	156.1881
Myl1	167.9691	220.8318	176.6776	248.4853	221.9117	360.3255	126.3122	167.6434	155.9886
Morf4l1	152.9746	141.8115	152.9627	132.5306	134.7411	142.7574	149.0178	141.2907	155.9071
Ddx39b	164.4002	160.4797	162.5051	151.8929	150.209	155.51	178.5574	141.3713	155.8879
H2-DMb1	168.5162	131.6378	164.5199	174.0982	255.5222	221.9191	154.0888	148.3171	155.1378
Arl6ip1	147.229	144.0142	150.8298	163.1362	161.3634	162.855	158.7168	145.5906	155.0474
Bcl2l1	160.9998	124.1176	141.3644	143.1795	112.2608	130.007	168.2586	150.1934	154.8318
Gpr137b	203.5894	231.2896	241.099	157.4193	131.9375	143.4189	193.8234	147.2643	154.7353
B4galnt1	183.6567	161.3483	192.1326	140.5247	163.1297	150.0108	170.1316	163.4598	154.6739
Ppp1r15a	134.8526	103.5659	76.50059	97.11707	112.8166	105.6552	154.3338	143.6416	154.4356
Sirt2	164.8385	157.4424	168.3796	163.9485	168.2975	154.1433	179.6585	173.0677	154.4207
Phb2	176.8311	170.75	167.1489	158.4106	150.476	151.3142	165.9114	162.5001	154.3551
Arap1	182.283	165.179	195.92	177.3897	171.8506	158.0035	205.8859	163.2333	154.1966
Bri3	115.3616	120.2271	135.2117	144.0564	130.0274	142.7839	154.0744	151.6078	153.8363
Eif3c	186.0118	156.8162	168.4882	147.096	149.6571	146.8645	172.9477	155.6369	153.7088
Hnrnpc	144.1742	141.2442	137.4337	129.9732	137.6872	132.3305	133.6802	141.794	153.6039
Eef1d	165.679	162.0126	162.0019	144.5375	156.5259	158.9823	158.2857	162.471	153.3936
Psmb4	152.5861	129.1082	127.5983	142.1465	142.929	157.2056	150.2186	141.9954	153.2786
Trf	164.6501	180.3105	209.8427	179.2827	263.6081	196.4297	232.7793	183.2448	153.0744
Arf4	160.4389	146.6229	155.1396	147.072	167.3988	150.5847	180.85	164.2225	152.9438
Psma2	137.6575	129.8099	130.699	134.3806	138.3033	133.7504	124.711	145.9608	152.9268
Tab2	172.3911	167.6874	193.1672	163.8337	165.8967	144.6726	202.2549	162.2534	152.8458
Eif4ebp1	153.0339	151.8938	162.9989	128.6309	150.1073	145.0747	147.1291	161.0915	152.6952
Tpd52l2	155.7271	155.1205	155.8414	140.0201	146.9378	148.1948	143.9413	162.471	152.6414
Phf11b	95.21219	60.24262	115.855	117.5341	99.01566	92.83042	78.09036	123.6952	152.4625
Dnajb11	167.3698	143.2186	150.0544	143.0674	136.5369	155.8806	152.431	156.0779	152.443
Clic4	179.4066	158.4041	180.655	145.7998	136.1723	128.7297	150.7649	164.3593	152.2211
Hsbp1	157.6535	158.0406	167.2378	167.4646	170.5418	158.1148	156.1063	157.9396	152.1173
Clec12a	130.6861	153.0566	155.7037	161.4378	159.9403	157.6322	147.1646	184.4298	151.7351
Pigt	202.4037	169.9171	200.0767	155.6504	176.9765	159.7566	206.9316	179.4665	151.3322
Atp6v1d	148.9693	136.2223	155.6711	146.3	134.7692	139.3927	153.3315	145.1127	151.2822
Ptpa	180.0988	182.5416	180.2196	143.1991	157.8884	174.4323	173.477	150.2319	151.0864
Fuca1	143.8577	153.0514	194.9897	151.049	175.2838	195.7833	139.2139	213.724	151.0149
Sh3bp1	171.6442	173.3496	214.3392	166.9097	195.5642	164.7184	189.7899	168.9268	150.9927
Pacs2	184.4814	167.8016	192.5885	158.3054	150.1609	149.1257	214.6094	163.8159	150.5687
Stom	183.1858	182.1091	179.9978	140.5791	151.1023	134.7954	169.3303	170.9245	150.4252
Tapbp	145.3256	128.7159	165.4858	135.6213	147.517	138.7411	141.8996	152.218	150.3375
Papola	178.0261	175.2639	181.9957	154.3465	145.1072	138.4318	162.1329	149.4318	150.3247
Hnrnpab	165.4609	151.3358	136.3972	134.1956	126.0007	139.2366	145.9621	140.6508	150.2921

Cd300a	138.0912	142.6063	164.1445	142.272	152.7457	133.5083	139.6583	150.1216	150.168
Bnip2	143.0514	141.9549	145.9006	121.8213	124.012	118.809	133.5652	139.9742	149.9099
Itm2c	150.0169	149.9454	145.624	154.5423	180.5952	173.306	172.262	161.3986	149.8191
Cox7a2l	164.1069	208.4656	201.0377	161.7585	188.6572	189.7368	159.8508	187.2311	149.657
Cltc	193.6201	173.6863	193.3956	155.2362	150.7141	120.296	196.278	148.6083	149.6478
Mrps21	116.2707	123.5164	131.4368	136.6204	167.0316	164.7349	127.7639	148.075	149.3231
Btg1	132.2546	124.3318	127.4228	107.6582	131.3853	109.9025	146.1614	152.8163	149.2841
Cic	194.9932	172.0092	193.8435	153.103	160.9655	137.2519	196.1668	166.9585	149.1506
Msl1	143.8866	143.7877	155.2609	138.4664	139.1294	136.6377	152.5843	145.2487	148.5421
Trim14	116.2935	118.5653	159.0493	168.0162	149.0514	115.95	186.8873	130.3228	148.5299
Arl6ip5	147.1862	149.7674	158.8802	132.5284	142.7416	142.2009	131.9266	154.0507	148.2525
Srp14	119.9287	124.7885	124.1536	125.9559	135.806	139.4603	116.5543	135.8982	148.1454
Krtcap2	180.5182	161.2198	156.4744	147.9905	170.1064	160.958	136.9455	156.1271	147.9043
Vapa	148.3635	137.8392	134.7474	131.8632	137.7006	128.7145	143.4019	151.7295	147.8675
Mrpl30	119.7013	125.5438	115.944	141.8477	132.646	128.4281	111.1134	132.5291	147.7306
Ndufb11	125.6145	129.3258	116.3658	146.6258	153.4379	156.7407	129.759	142.7058	147.4937
U2af2	160.4413	137.7465	135.8011	152.595	142.8938	141.3716	163.2339	147.9355	147.4622
Hdgf	165.7002	164.3405	170.3249	129.7672	138.8796	134.8519	143.4433	149.6762	146.9548
Irf8	133.9315	129.6131	149.6632	172.8568	166.6299	176.3656	183.1041	163.2048	146.7644
Eif4e2	165.6034	164.1584	174.5673	148.5381	152.4729	147.6676	168.1598	156.8248	146.7141
Plxnb2	185.3878	172.3087	209.5527	163.1209	163.3972	139.5256	203.5427	160.4042	146.4265
Grcc10	125.9775	129.1102	139.2074	143.4501	156.5034	163.4492	147.5578	160.2393	146.2971
Atp6v1a	172.6717	157.7696	167.1487	138.8657	118.2632	119.3433	162.094	132.3636	146.2124
Cops9	117.8375	134.4392	120.1298	116.9257	143.0866	141.9802	107.2702	140.2712	146.2076
Tm6sf1	161.7214	174.4398	198.7572	144.159	151.5001	124.2709	176.6968	155.2093	146.1011
Nfam1	163.9368	154.0744	177.332	129.3706	150.6031	137.266	143.2827	151.7652	146.0987
Dock8	171.3568	144.4351	172.2484	152.1192	143.9733	112.4946	189.1542	141.3213	145.9778
Trappc1	155.7475	170.4728	181.1435	163.0887	155.4192	150.3614	144.8986	144.1068	145.817
Renbp	134.8341	119.6442	127.979	154.4868	149.9894	155.7724	154.772	138.9731	145.624
Fam89b	149.489	147.3941	165.1518	140.4741	161.9517	156.1623	167.4001	166.627	145.5393
Vps28	151.104	139.2804	155.1741	149.148	160.5212	161.6769	161.5148	139.4486	145.4708
Tomm6	163.8128	161.172	148.3562	138.484	182.6621	168.7036	170.5713	158.0151	145.4276
Ubl3	158.3479	156.3019	169.8284	144.7686	160.3419	148.9274	175.5364	151.2703	145.4157
Eif3i	146.2685	132.8168	146.7855	138.6887	139.4474	144.1866	139.6542	141.8321	145.41
Pea15a	163.3317	182.6289	186.6214	158.4775	172.1863	179.8254	160.5521	154.7708	145.1889
Anp32e	178.8216	161.6909	172.0808	130.0875	133.9282	134.7957	143.245	129.2527	145.174
Cxcl16	135.4575	165.0937	205.9951	133.111	185.9091	170.9714	167.02	154.4232	145.0286
Dctn2	127.4947	140.7219	141.6915	140.9134	146.9378	139.7689	145.0687	137.6071	144.2326
Cd47	121.8828	120.233	138.828	128.6979	120.6747	101.8402	125.1957	127.7616	143.699
Elovl1	177.2649	167.5097	172.494	146.0879	153.8982	151.2388	152.1227	153.4601	143.2807
Ahsa1	146.0709	128.5922	143.0216	137.2759	133.9165	144.2866	146.8044	148.0308	143.2214
Mrpl54	108.5151	113.5009	108.0554	139.3684	133.4806	150.396	116.7435	140.3636	143.2101
Ly9	135.6686	145.8098	163.3939	166.9584	139.5792	129.8821	162.5425	146.8613	143.1409
Asprv1	86.56562	69.71564	60.20617	39.38193	101.2345	89.326	45.36869	132.4885	142.9394
Lpxn	120.0443	108.6075	119.5965	124.0943	108.1728	104.5781	131.1616	128.7377	142.4818
Os9	157.2791	153.9706	168.8317	155.7168	164.717	156.3761	176.1722	155.8469	142.3508
Skap2	131.0354	118.947	131.9302	121.22	125.6987	124.3993	116.9732	127.3993	142.2967
Pid1	193.9446	178.1582	186.6765	124.3123	127.2906	103.5388	142.6038	151.3646	142.2302
Ap3s1	122.2126	121.5655	109.1376	128.6309	119.7734	121.2859	112.7385	132.8117	141.9099
Pik3ap1	129.2445	86.93444	128.8433	149.3934	113.0625	95.24751	133.7957	117.1755	141.8121
Rasgrp4	133.8119	132.2307	136.6623	112.2751	142.5135	134.2738	123.9219	139.7937	141.4475
Capn2	193.532	172.4369	184.6646	134.3796	128.3047	126.2061	146.6988	141.6123	141.4252
Eef1b2	126.8795	140.1283	127.5826	111.0251	122.0951	135.2366	104.3102	128.5293	141.2095
Sin3b	129.832	125.0505	128.9469	144.2161	137.468	135.342	166.2572	141.0557	141.2025

Lyl1	112.446	122.6153	135.856	137.7117	157.9561	146.0309	139.0932	153.9806	140.7041
Tmem86a	120.317	129.2889	161.5351	172.7391	191.3249	173.4675	192.8169	149.2114	140.6637
Ubap2l	180.254	160.3835	187.9946	169.5472	155.1059	143.0031	203.4735	152.2062	140.6229
Sod1	131.7633	138.2696	147.3588	133.8811	145.0511	158.2287	132.2262	151.8842	140.5126
Cct2	151.9629	143.9597	145.2197	123.7821	130.2154	123.7232	138.2196	140.8588	140.5036
Ik	158.704	142.7968	145.5835	129.7264	137.4721	139.213	158.402	147.2673	140.4321
Oas1a	103.33	72.68983	100.4651	117.2389	111.5744	100.3238	97.72321	112.8919	140.2826
Txn2	138.9843	157.677	143.3686	124.5314	137.2269	132.3274	130.3284	136.1969	139.9243
Ndufc2	101.4329	115.3712	102.2589	104.2647	132.8881	127.1521	91.03736	128.1196	139.4256
Slc10a7	148.3554	150.4913	148.1774	127.5309	113.7773	125.6412	158.9818	121.7342	139.3493
Stx4a	120.0081	119.6886	124.4673	145.4129	132.3813	131.0083	153.6438	130.4173	138.6789
Prkar1a	152.522	145.1878	151.1939	116.8282	118.4124	116.4404	135.0049	131.2836	138.5379
Prdx6	119.9578	101.2185	111.2866	97.81867	102.1025	110.6222	115.7338	128.5354	138.4322
Gps2	141.5924	147.3331	144.1185	136.5481	145.7826	152.384	171.846	155.0291	138.3162
Rab21	145.269	134.2652	141.6005	126.5409	125.0165	113.6667	152.9357	129.8901	138.2982
Tpcn2	166.8088	162.5082	149.6057	175.3529	133.0028	134.842	193.5028	136.5581	138.1143
Slfn1	84.87032	55.81621	101.9558	100.4993	106.8802	87.55808	62.08041	119.1997	137.8852
Psmc3	138.9512	120.4893	131.3034	129.1704	131.2053	149.5691	136.4989	139.1391	137.7004
Trafd1	99.59018	87.40981	117.7842	141.1762	137.7756	119.1727	147.5643	123.6611	137.6293
Rtn3	156.041	159.625	164.721	126.7193	135.7566	122.3	148.0946	136.463	137.4521
Rab5if	123.8521	110.3705	123.6555	110.5698	119.2615	122.5344	108.272	123.2428	137.0957
Zdhhc18	146.7275	140.7003	150.671	111.9282	112.1714	111.0328	122.3101	127.0476	136.9249
Mapk1	161.9239	156.2271	160.02	122.0966	127.9792	114.1044	145.9142	131.7103	136.8795
Dtnbp1	139.758	126.8602	127.9749	144.6099	133.0638	157.2043	144.2284	130.6879	136.8291
Slc9a3r1	133.4882	120.5529	123.0117	149.7499	141.519	145.4093	179.2709	130.3452	136.7849
Siglec1	154.0868	128.0064	213.2732	185.8504	155.9501	124.5561	212.3385	149.8559	136.3531
Abr	132.67	123.3427	148.2408	127.8056	122.8716	105.5659	151.6817	114.312	136.1487
Eif4h	161.9411	143.201	143.281	128.9857	137.6798	135.3931	147.9061	146.7457	136.1312
Metrnl	152.7467	121.5136	131.7593	119.1451	109.8545	117.969	119.3554	135.6133	136.0113
Abcc5	129.536	158.3586	147.3268	182.8119	146.2512	133.5866	200.3395	140.347	135.8006
Dnajc7	123.8957	130.2153	144.1242	130.0926	138.0464	128.7025	129.7887	147.8509	135.7961
Cnbp	133.8595	131.424	115.4193	119.4174	114.6666	119.5287	117.1231	135.2153	135.773
Preb	138.2985	124.8263	121.7829	123.9105	119.1198	124.8369	136.3237	124.4908	135.6887
Copa	170.5152	146.1694	159.8812	143.998	138.1647	123.9366	166.738	130.3372	135.6803
Hnrnph1	146.6744	152.8806	140.1925	131.7176	119.605	115.6534	162.1794	144.5649	135.3423
Ifi209	97.97176	67.25927	101.82	112.5724	102.0491	84.12132	74.43661	108.8252	135.2589
Cyb5a	111.8079	138.3921	128.594	121.0423	126.8941	122.6601	129.2472	130.5938	135.1699
Arf6	125.9578	111.8406	123.461	113.8455	114.7814	117.5261	113.7821	121.0418	135.0179
Nme1	124.4826	122.9176	129.7829	127.2518	122.1436	120.8891	124.1844	127.8342	134.8265
Amdhd2	111.2551	92.40274	98.53432	158.3739	135.3348	163.509	150.8626	133.9224	134.6758
Mbnl1	145.3643	141.0075	145.4186	133.6188	133.4285	112.4236	144.425	138.9356	134.6682
P2rx4	141.8601	148.4381	167.3998	152.2191	154.0805	134.1455	159.4978	153.5471	134.6126
Stx8	114.3028	109.3604	113.7041	120.4774	124.2457	135.0154	113.3312	121.2948	134.6012
Fcgr4	106.0863	118.9851	180.3942	155.0627	187.6674	147.7321	125.7347	146.9886	134.5029
Rpn1	157.4246	134.1728	139.0223	137.728	123.9651	127.5491	144.1103	125.362	134.4873
Eif3l	170.9121	155.2398	151.3374	142.7258	130.839	136.4422	152.7947	140.6696	134.4241
Psmd4	126.5471	116.0428	128.4155	123.9532	124.3736	131.4408	127.6579	131.1139	134.382
Pdlim7	114.7665	121.5542	129.2489	82.08682	105.1341	106.2449	103.4128	109.5005	134.353
Dnase2a	127.5529	119.941	124.2529	149.5972	132.2457	145.6394	146.869	127.8206	134.3048
Mtpn	156.6996	157.8966	161.2033	133.4408	128.2345	120.0382	147.2709	138.1981	134.2242
Timm10b	126.241	115.2403	135.0811	119.6494	136.4458	146.9857	108.4799	138.5787	134.0724
Clk1	98.12442	107.1154	102.4081	107.9092	124.7857	112.2635	156.2433	129.8467	134.0229
Arl11	104.3672	118.4156	123.4968	138.2652	140.2619	129.0679	143.6703	128.8576	134.0187
Cyb5r3	190.1642	208.5107	190.1863	152.0267	136.8395	138.3402	182.948	128.7987	133.9373

Elovl5	150.3733	129.1827	139.7408	121.9993	124.0368	119.0669	146.3467	131.9254	133.9081
Septin7	129.3404	130.0329	124.8349	112.1878	100.4099	113.5072	102.1694	126.2002	133.7628
Eif3e	129.2955	125.0045	134.7075	100.5603	114.7796	115.4431	109.3905	133.9257	133.6879
AI662270	123.9875	122.3616	128.5698	116.0889	118.5344	114.3685	114.6774	125.3209	133.5195
Ifngr1	123.1607	151.2634	169.7725	127.6912	155.3959	115.7157	149.7561	160.4717	133.2693
Neurl3	107.3396	126.8821	144.4106	131.4506	164.2522	147.4085	165.6703	146.6685	133.2171
Rpl17-ps3	117.1051	124.6032	116.4068	108.8393	134.4119	123.3544	107.9741	132.2527	133.1536
Diaph1	164.2721	140.9974	156.8919	104.2185	108.9237	97.24085	124.261	131.6799	133.1039
Fus	155.5697	116.2541	126.9926	123.4533	108.005	111.3298	129.0605	128.0304	132.9827
Kif1c	160.476	146.6214	142.9527	118.0625	120.5524	116.3348	145.5728	123.199	132.6331
5031439G07Rik	161.2783	152.5322	163.6823	134.7476	129.5045	124.2096	159.7375	130.4941	132.2897
Anxa7	141.5593	131.8076	134.8722	122.842	121.1621	115.5986	117.6872	126.2864	132.2208
Becn1	151.6908	133.0019	144.0684	121.0346	125.0676	134.8965	148.0574	144.2859	132.0935
Gdf15	73.38217	60.81551	39.78675	128.9769	89.23385	124.9573	226.0388	136.6	132.0361
Sdhc	132.8204	140.242	140.9696	130.4972	126.8633	122.7391	135.1875	126.4122	131.91
Arpc4	146.15	118.9287	136.6544	103.7965	127.9849	118.4427	124.3704	137.9589	131.6273
Tmem176b	152.3639	168.6127	177.1216	149.4992	262.5113	229.4803	198.5866	201.3535	131.5745
Rex1bd	112.1827	114.025	99.96812	114.8954	124.7967	149.4867	111.6057	116.4946	131.4708
Tbcb	128.479	113.4948	125.718	115.7751	125.5619	131.3093	128.109	129.6961	131.3625
Add1	149.1932	139.5521	158.8034	152.0887	136.2682	130.8686	173.6148	134.949	131.2291
Tmem258	131.5659	135.4484	142.8558	138.3463	153.7223	147.6697	116.7892	134.7342	131.2087
Rps20	112.8871	120.272	111.9992	121.1814	135.2141	148.2083	104.3702	129.7505	131.1889
Ndufs7	132.928	130.0364	129.6276	150.5081	145.1778	164.4264	127.9044	133.9016	131.1749
Mrpl23	120.6712	126.8962	130.0775	135.1578	132.3331	164.728	131.7124	136.1384	131.0829
Cmklr1	137.377	117.0342	174.6822	132.5563	146.6987	110.8269	186.3836	142.8321	131.0276
Blnk	167.8455	174.3177	177.1474	166.9053	174.3783	159.4086	219.4701	144.8394	130.6341
Mrpl33	111.1141	122.3272	114.4584	105.4048	110.3559	121.5675	84.22561	133.6552	130.166
Tor1aip1	132.6696	104.3935	135.5487	121.2291	113.286	97.40435	129.7631	116.4319	129.952
Hnrnpu	143.1178	129.1993	131.8916	123.7302	114.8157	115.4956	136.8857	135.7448	129.6712
Emb	146.3337	155.1591	159.0805	93.77146	120.9213	108.557	101.3314	144.3365	129.6552
Gsdmd	106.6434	102.241	109.2715	114.6694	122.574	115.0413	109.3321	128.7945	129.6501
Gm15772	76.31733	92.83465	68.81702	110.4596	117.1561	123.7456	73.45625	134.7471	129.5938
Evl	117.9996	123.0659	150.4156	163.2782	135.4596	128.9678	148.5435	139.8168	129.5084
Mrpl4	124.8274	115.5241	123.1437	139.3704	120.6326	127.4045	136.5097	131.0823	129.4254
Cmip	166.9478	139.0029	147.6276	113.7071	121.0345	105.9282	140.2437	136.8071	129.3761
Dync1i2	130.2211	127.2625	125.3499	134.0875	113.9184	113.4436	133.3162	119.2268	129.0416
Psmb10	101.7965	83.15339	99.97474	108.8226	121.0626	113.4341	99.16615	122.8959	129.0148
Tmem14c	121.6782	109.9618	127.2802	109.5143	119.9055	121.0783	105.2281	124.3018	128.6913
Reep5	141.2159	137.5775	157.0928	118.3206	121.5206	117.8371	127.23	117.7315	128.52
Itsn2	141.6855	127.2857	131.4625	122.0078	108.2933	94.68104	149.7063	111.3598	128.5185
Fosl2	160.4905	125.8462	104.3367	77.14469	106.1645	80.32823	144.0072	118.8506	128.5073
Anxa11	149.324	149.5959	141.1925	105.8126	110.2267	113.5803	123.5436	113.7413	128.4644
Aplp2	157.3715	159.6896	168.0371	163.9397	166.6874	148.5369	192.8571	146.049	128.1777
Flii	151.1463	122.2741	142.7727	135.4748	120.4334	116.4731	148.9437	130.8958	127.9975
Tbxas1	146.3875	132.0398	143.3027	153.933	154.0021	133.1159	200.6632	147.9755	127.8074
Itga5	168.7907	152.9452	166.3335	115.0178	106.3874	104.7784	142.7773	126.0316	127.6724
Dstn	120.6216	120.7951	120.6064	101.7248	105.2082	116.296	117.7625	113.9824	127.5328
Pik3cd	128.4439	117.735	136.6066	134.9713	149.0142	129.7308	159.1825	125.9991	127.4811
Bag6	151.4851	136.8946	150.9146	137.2498	136.7496	126.1107	165.6024	134.9373	127.4581
Park7	117.7203	94.79585	113.1136	129.3115	118.5524	131.1749	111.8549	110.4522	127.4458
Ifi203	94.97085	75.87583	117.2614	138.4113	111.272	87.23831	120.5902	114.6268	127.255
Ctdnep1	136.0378	139.3046	144.0184	119.8128	142.3437	119.9308	142.1274	134.4564	127.2024
Clptm1	165.5847	142.7575	152.0138	129.0523	132.989	125.3931	153.9076	124.4158	127.1276
Ptpn1	142.4441	111.2503	148.3565	123.8243	133.4565	134.8674	131.4468	136.1094	127.0706

Psma3	116.8968	106.9544	124.7203	112.389	112.2396	119.8995	114.9657	126.8482	126.9802
Atp6v1c1	147.4302	127.7196	123.3539	131.0184	105.8028	115.1583	132.6079	120.719	126.9383
Slc37a2	165.6685	139.7729	149.3165	150.5149	112.4698	102.5582	159.0317	118.8721	126.899
Tmed9	139.2815	124.7268	131.6712	118.4446	122.882	123.3379	120.8223	112.6437	126.8606
Nadk	135.3065	126.6097	140.432	98.47816	111.1344	100.0366	110.0437	121.6308	126.7389
Vcp	153.6379	132.2134	129.7886	125.8038	124.5028	112.5563	146.676	119.1148	126.5866
Ergic3	129.2892	130.2935	146.4069	139.7566	157.9413	152.4046	143.245	130.5931	126.408
Iscu	119.2777	115.6574	128.3074	123.4881	144.3785	125.2762	135.8292	137.3278	126.3655
Maf	111.5307	108.3341	131.8672	133.7168	138.3898	106.0821	171.0445	140.1446	126.3339
Gna12	126.6229	110.8162	138.1984	149.0203	135.5351	127.4594	189.9368	120.3047	126.2758
Crk	125.5652	129.1722	129.3829	114.5184	109.2189	97.19083	134.2862	110.2149	126.2128
Abracl	97.63259	98.60134	93.4856	81.5428	104.4239	120.5496	95.33751	125.3584	126.0759
Copz1	145.6435	135.5024	143.8286	117.8005	132.1055	122.3763	144.3436	130.6639	125.9993
Hypk	103.0744	91.44897	96.84429	101.8581	91.69824	128.5929	101.315	119.1101	125.97
Cds2	135.3817	133.684	136.5535	138.6234	137.2588	108.3072	159.2779	112.5204	125.946
Tlr13	140.3077	120.1679	149.4267	124.9365	103.1901	97.48977	142.7311	120.9763	125.9331
AY036118	94.7444	117.762	125.9208	131.0697	141.121	121.3462	95.52379	127.2923	125.9248
Nfil3	110.5595	74.49573	65.43373	70.92731	83.66632	77.88596	131.2559	130.4763	125.7837
Was	144.1019	128.351	144.2246	117.4158	133.1564	124.3491	136.5685	144.1813	125.6591
Sppl2a	127.1745	120.5069	154.9805	105.8652	113.3302	92.69318	118.7513	117.3111	125.6414
Otulinl	117.6763	121.3167	149.1329	129.9045	157.7859	135.6414	155.7258	138.6048	125.6123
Ak2	126.3462	119.9651	132.3377	127.4086	128.5654	122.8771	142.5879	126.7102	125.4686
Stat6	143.3522	129.3259	138.6623	112.5152	117.267	106.9946	136.6418	120.5604	125.2823
Ppp4c	122.239	122.3057	121.5454	108.7693	114.5093	119.6239	120.3258	126.9774	125.2733
Gm10221	97.32189	109.972	118.705	120.0906	126.4143	112.3591	96.67461	115.3309	125.227
Adcy7	160.7363	163.5535	174.5309	145.9693	135.9365	112.837	182.2871	133.2396	125.1972
Myo1g	137.3938	138.3143	144.1239	122.7281	131.559	120.5388	156.5744	136.2622	125.0889
Mrc1	191.8024	166.7032	184.5161	139.3737	163.489	116.9471	193.6043	136.0255	124.4133
Morf4l2	125.0319	126.328	127.0003	119.4907	110.3555	110.1464	114.4305	118.1888	124.3251
Plk2	88.41995	74.80592	54.96961	137.0515	75.59886	83.23174	169.7264	124.6096	124.249
Cd300ld	120.1461	156.0596	149.4729	98.55175	101.2695	94.61354	93.03881	118.7173	124.1679
Ezr	139.2452	116.7043	124.1271	98.56514	99.767	93.21646	122.5281	127.4	123.97
Ensa	132.6082	149.792	142.8073	107.8174	109.2787	126.2503	121.6608	130.5648	123.9612
Gm15590	136.7392	113.9844	124.1813	131.3297	130.8562	146.7155	137.9855	125.4011	123.5916
Dock2	154.5198	133.6505	143.2636	131.0337	121.0779	112.1228	154.632	124.3043	123.5713
Lats2	136.5714	122.5687	134.134	114.8287	112.2554	94.87869	138.2378	123.8987	123.3262
Slamf7	114.4066	93.42106	111.7829	156.8279	122.154	103.2167	149.0071	122.6698	123.3086
Glipr1	135.3386	160.5521	154.0752	105.9203	101.9829	110.4549	109.4546	130.8576	123.271
Polr2j	120.2419	104.2593	122.3375	136.784	119.2924	136.2173	120.2671	118.8056	123.2572
Csnk1d	136.1848	132.8173	130.8972	112.8916	125.9706	113.3734	145.7051	120.4244	123.1341
Cox17	100.3225	102.7185	96.01056	92.04779	93.85073	121.8188	102.2428	107.8651	123.0045
Usp15	134.2157	131.4081	131.6139	128.2591	109.0052	109.1531	136.6762	123.6676	122.9818
Fcrls	89.26893	84.3381	117.0712	129.8584	196.8213	181.1739	173.7071	111.0261	122.9246
Myo9b	137.9317	129.0439	148.9488	150.551	124.5963	109.766	190.2906	123.1591	122.796
Ccdc124	121.5864	113.3728	103.2205	130.527	131.8516	138.0449	111.9544	129.262	122.6106
Slfn5	99.27669	64.35297	105.594	112.6246	89.13683	70.85171	83.53683	92.50836	122.4389
Tsc22d4	114.9826	109.404	125.2871	109.1051	125.2197	119.4493	134.3539	120.2829	122.3425
Cnpy3	127.2998	133.572	137.3084	133.2112	154.4383	135.5033	138.1833	135.4886	122.2395
Ube2l6	108.7178	93.63902	120.6827	127.1073	121.2807	109.3729	100.4264	114.1273	122.1698
Ddx3x	132.2339	129.0272	122.6305	103.6219	102.9059	91.12831	120.0237	111.3501	122.1005
Cs	166.4355	152.6686	155.4993	116.6461	122.0932	108.7399	135.255	125.2501	121.9902
Ech1	118.427	106.6941	111.8634	129.632	117.1962	117.8519	124.817	114.8877	121.786
Cmpk1	106.0106	103.7285	98.47298	102.4551	108.181	100.3532	95.12078	122.5508	121.779
Map3k11	128.992	132.4219	143.5191	116.3189	131.6256	124.8446	144.0846	124.5293	121.753

Idh3g	153.4393	149.2716	161.9708	140.83	148.8364	154.9379	164.1463	148.0651	121.5785
Rnf114	94.83046	93.05522	106.4468	95.18263	97.2425	87.23414	91.84322	116.0316	121.5234
Trim8	132.9812	137.1997	145.2786	124.8859	119.8551	114.8649	137.5394	133.0697	121.4145
St8sia4	129.9653	136.7586	132.8043	125.8758	118.9673	102.9393	134.0379	129.6465	121.291
Stip1	143.6848	141.394	133.7506	110.5595	114.4415	129.5722	122.9547	125.1671	121.2123
Eif3g	130.3417	104.8862	112.8098	119.2881	120.626	114.9196	115.5617	117.7721	121.2094
Wdfy1	132.4483	119.1406	128.1333	129.4766	95.58241	98.88814	122.5874	127.1343	121.1502
Stfa2l1	85.29963	85.47319	66.15842	32.35746	56.11446	60.43112	12.17929	63.23697	120.99
Brd4	155.7178	134.6326	149.3814	128.6645	120.735	110.025	168.3273	133.57	120.9785
Prrc2a	163.9362	139.8318	154.9569	135.2672	130.1753	114.6639	178.3721	129.2953	120.7378
Ets2	100.5555	84.11739	66.90449	52.55408	72.6352	59.73025	90.78092	93.1857	120.6969
Idh3b	131.277	141.0334	146.5158	127.1381	130.9886	135.6441	140.8944	122.9304	120.6547
Myd88	114.0816	96.19457	114.0663	99.26939	122.4971	105.4727	103.8878	110.5516	120.4594
Ppp1r14b	112.3842	111.9647	102.7443	103.4766	107.8953	128.029	103.8721	111.3084	120.4509
Kdelr1	149.2647	161.847	153.6085	130.1371	141.2233	135.7493	154.8361	134.8462	120.4
Ptpn18	69.89848	74.54547	78.12635	109.0214	110.2863	115.2261	105.9813	129.151	120.3067
Bhlhe40	142.7535	127.6494	117.3899	89.74825	102.9853	93.34518	159.3963	138.8147	120.3056
Snd1	153.6168	125.6289	134.0551	125.0209	128.4926	115.0138	146.7729	116.0607	120.2576
Xaf1	74.61398	49.95758	94.53842	131.4015	107.1777	87.35917	90.26659	94.68075	120.085
Ndufab1	137.8406	122.9235	114.109	126.6632	125.0493	129.5176	118.3396	126.2564	120.0588
Evi2a	121.0746	104.955	132.8576	123.814	119.6152	105.9204	103.2445	131.2093	120.0235
Eif4a2	100.9456	114.0013	101.3749	112.8943	114.4585	111.1115	129.9762	127.9954	119.9429
Pi4k2a	127.2813	112.298	108.7736	127.7178	105.9954	99.60066	139.4319	102.872	119.9039
Csde1	123.6073	123.2216	116.7798	101.3071	105.1647	94.96148	115.5055	115.0242	119.7296
Tnfrsf14	77.65879	95.30232	121.6594	128.2035	121.4151	99.65931	129.0969	135.0971	119.4805
Chmp3	132.4672	133.831	130.0312	125.4501	125.6637	127.2762	130.1737	128.7038	119.3439
Cln3	115.3899	110.4903	112.5554	122.6361	118.4651	118.1671	139.2686	134.8693	119.2739
Arhgap30	120.3011	112.6506	117.0289	112.8837	111.1382	104.3511	118.1835	114.362	119.1902
Tmem59	128.1467	140.5896	156.0993	115.7535	139.2461	123.6527	138.2723	125.7344	119.021
Mmp8	88.95402	59.12226	66.1582	53.41954	45.69565	77.14143	34.96743	47.38157	118.9649
Map2k2	130.402	110.7591	117.7302	121.5496	120.9603	117.4524	129.3426	122.503	118.8112
Foxn2	120.8775	117.9134	119.4342	89.31054	101.3708	79.87177	123.2215	120.4475	118.8052
Serinc1	137.1028	132.628	139.5453	109.2402	109.1	99.64998	120.8851	117.0968	118.6869
Lims1	126.5868	117.1622	122.3808	97.4937	93.61247	86.85298	108.1921	113.7837	118.6008
Hpgds	105.3055	85.07771	100.8204	179.5315	132.8275	128.7891	182.7154	107.3542	118.5323
Twf2	114.5975	111.5522	135.9677	112.2609	134.8782	132.028	122.8636	119.4394	118.4035
Sf3b5	103.0211	103.5755	108.5474	106.9504	123.4365	119.5773	101.6376	116.783	118.3443
Psmc4	123.423	105.3445	114.557	100.3505	107.3988	111.2102	111.9625	105.7855	118.185
Sh3bp2	132.2638	127.4261	144.4607	133.1737	119.1645	110.8889	141.6689	124.9173	118.0563
Rrp1	132.5765	117.2995	121.2158	126.6725	116.0886	117.7516	130.3758	121.6783	117.9632
Ifi213	71.89539	37.24917	80.03814	120.3987	89.0685	60.44923	86.62176	86.73626	117.7045
Eif3d	124.547	112.4047	113.8499	115.7967	111.0153	121.1628	119.4858	125.6554	117.4871
Sec61a1	143.7486	122.1703	134.6614	116.7673	110.3923	116.7392	133.5068	113.867	117.4802
Gm2000	94.307	112.6932	101.2952	109.7595	113.0721	121.544	104.6815	122.1707	117.4296
Ppp1r12c	110.9927	113.7592	109.3537	116.9407	114.9244	120.0911	134.4786	118.4044	117.4164
F10	87.39856	67.10845	73.94078	89.28765	86.52812	76.84987	79.75479	123.5527	117.4118
Rnf10	130.8295	120.9312	131.4154	114.5106	117.468	110.4005	138.5915	114.3422	117.4013
Higd2a	115.7597	120.232	117.5821	117.6294	145.1316	127.6248	120.7651	107.7176	117.0525
Cdc42se1	129.0674	121.2261	126.8509	99.94113	114.882	104.784	100.5727	111.1532	117.0354
Ccl12	66.1947	40.53947	71.9951	74.0519	109.5374	60.6409	51.52544	110.7841	117.0112
Dnajc15	88.29025	83.02103	90.86738	106.2566	102.9899	104.8291	87.62704	113.5475	117.0087
Tax1bp1	113.9709	101.464	112.3561	98.03414	91.93329	93.00588	109.0862	104.5089	116.9277
Ap1s2	96.59988	101.3837	106.6406	93.33181	103.2159	102.8083	100.202	115.5992	116.8983
Il7r	137.4962	132.2587	128.8936	118.2518	78.25092	80.43398	126.9643	106.0134	116.8475

Ywhag	141.4041	129.2369	133.4558	120.3689	109.8158	98.48133	130.5635	114.5873	116.7512
Lypla2	118.3235	112.0387	124.5326	126.3469	127.8701	113.5735	124.8111	125.7767	116.5209
Vkorc1	137.9003	162.9452	168.1868	130.2177	143.9828	146.0906	134.0678	138.3518	116.4189
Rap1gds1	140.6225	135.3587	141.8185	106.6263	113.0747	99.53508	137.759	115.3564	116.2844
Lrrc59	156.2203	123.828	131.1378	114.3011	108.0751	112.3939	137.4626	115.3615	116.2833
Itgb7	140.1794	104.7838	124.5698	106.2134	93.71388	104.7576	100.3223	110.0796	116.1715
Ptafr	99.67267	92.06464	108.3279	110.543	96.95955	80.85502	128.3022	128.7706	116.0476
Nupr1	95.64394	99.80548	89.98661	72.47596	93.78921	96.6382	84.84864	113.6594	115.9722
Rilpl2	96.62062	90.22001	77.71032	98.22325	82.5502	94.84466	109.23	109.5446	115.8229
Mfge8	177.5394	235.9517	186.3376	145.9033	117.5719	146.958	189.3251	111.0396	115.7703
Tmem259	119.7339	120.0057	123.1213	117.9878	123.0823	116.703	140.5172	115.1016	115.6631
Snrpe	96.34054	82.71095	89.02675	100.9551	93.68533	102.0138	81.98739	106.6378	115.6624
Cct4	127.7388	127.2581	125.1309	109.6382	111.8546	109.0279	120.8621	124.6536	115.5113
Mgst1	97.2768	96.94862	98.39838	102.9415	92.11671	100.1607	89.1382	108.0752	115.3958
Txnip	124.587	163.263	182.5032	130.1151	163.0379	121.1874	141.0948	135.5404	115.3623
Cox7a2	78.30443	93.05518	83.97563	102.9437	103.7766	109.7421	81.00752	93.94439	115.2052
Tmem123	130.5377	126.2393	131.7559	96.66394	111.2662	98.33441	121.5627	112.18	115.1235
Cux1	150.8996	135.0882	143.3541	104.773	106.5989	96.59802	130.6402	109.4843	115.06
Ctnnb1	146.9279	136.7644	153.4726	143.4153	128.3794	120.2829	156.6447	110.5918	115.0136
Uqcrq	91.4728	90.34617	84.62988	109.7191	110.5837	115.2092	86.06638	107.1044	114.8113
Csf2rb	156.1457	145.9599	165.007	96.8	123.4701	106.3973	130.3124	111.026	114.7564
F7	123.0994	124.56	115.2229	137.5959	116.8685	122.6548	139.3376	107.9835	114.3908
Tubb2a	101.5962	98.74527	106.7143	122.7076	108.8827	113.573	130.4983	107.9298	114.355
Rab4b	114.3351	94.04106	109.4674	102.8105	120.2635	104.2437	111.9025	112.0612	114.2552
Eif4g1	161.4588	133.7122	146.708	125.2466	105.577	98.48683	146.671	108.6097	114.0692
Jak1	116.9532	104.6904	126.8401	102.9257	103.4239	85.95722	123.5256	104.5309	114.0255
Ppdpf	97.48886	93.95285	124.4421	124.2207	113.6539	121.6746	115.2751	109.6311	113.8939
Pip4k2a	132.1514	130.7584	143.1656	117.5577	133.4879	115.9009	153.1419	123.9188	113.772
Mrtfb	162.5183	154.602	167.1281	171.0007	110.1372	107.2697	195.9811	129.0145	113.7519
Il3ra	117.9151	115.6027	133.0874	137.9465	118.124	128.2397	134.7751	132.5187	113.6567
Mgrn1	127.4918	110.9616	133.1207	127.5592	109.1043	107.3729	144.4402	111.8715	113.5715
Tuba1c	142.0022	128.1721	123.5372	108.4426	98.57342	103.0015	122.0445	105.1378	113.4788
Nceh1	125.5532	109.0154	107.7492	112.0892	94.68367	86.15534	136.4579	105.4528	113.4296
Akr1b3	133.2174	125.3698	128.0918	119.2595	120.0961	127.4737	129.5948	114.3369	113.4007
Wasf2	154.1905	139.0647	154.5068	116.7145	116.5625	95.78774	150.8838	117.973	113.1325
Use1	97.69823	94.78367	102.449	103.4771	114.6237	109.8587	109.564	112.4872	113.0993
Gnpda1	114.4434	115.4128	134.1275	127.0809	128.3924	111.3171	142.5475	114.3314	113.072
Vps35	121.9776	112.0687	113.3524	104.2869	96.24677	93.24697	113.6279	105.5319	113.0482
Eif4b	140.5658	144.6963	149.1851	105.6063	122.2697	114.2542	130.4147	116.6172	112.9684
Elf1	119.4882	107.8338	129.3356	113.5835	106.0953	92.18456	130.344	107.092	112.9524
Mvp	122.2986	99.92445	113.5805	108.3521	104.0594	102.6085	104.0677	105.8307	112.9326
Aco2	143.2016	127.1601	136.0674	120.3724	110.6901	108.0542	131.9427	106.8044	112.9285
Ikbkb	134.7432	130.1661	137.1383	120.8825	115.0204	102.6046	147.1481	121.8887	112.8014
Ifit3	57.80081	30.63391	72.76371	132.031	98.35249	61.41961	62.65852	67.59668	112.7875
Prdx2	107.5874	114.0561	112.4944	107.413	119.6918	118.6825	114.0295	113.1692	112.6153
Atp6ap2	123.6119	127.3922	126.3657	96.89221	97.16905	91.68808	114.3298	105.721	112.6142
Rgl1	139.6218	98.8729	115.368	111.7642	94.02006	81.6343	114.0116	110.4664	112.595
Lrp10	127.6934	115.6641	128.6651	108.5901	119.1955	109.8854	117.905	116.8117	112.5842
Bzw1	118.5123	113.7179	113.8833	87.80932	96.78676	91.49113	106.0604	112.403	112.4718
Ncor1	137.0813	126.6336	135.6354	117.7819	105.7629	89.32368	158.2799	105.9957	112.3582
Mcub	141.1538	138.9705	144.0491	95.9961	118.2892	115.3023	109.2519	124.9016	112.2795
0610010K14Rik	102.7931	99.91371	107.5411	109.6055	121.6156	124.9595	112.8239	118.7023	112.2114
Sec11c	104.6123	116.8999	88.92723	95.36346	90.7176	103.4003	89.14677	91.92375	112.1937
Plcb2	112.0603	112.3836	111.4812	118.5288	115.0351	106.454	138.2367	98.77057	112.1495

Ctbp1	130.5159	119.0015	126.4779	116.765	112.0548	113.5858	134.4382	118.6493	111.9885
Furin	143.0225	123.4745	130.8576	109.6176	109.1841	99.68178	146.9926	119.9209	111.9374
Cdc34	96.26573	99.81732	95.66596	105.9015	107.0517	114.2571	95.70714	103.2869	111.7882
Tspan31	121.2595	117.1384	126.1605	109.076	117.8473	110.3428	119.6091	113.98	111.7359
Rsad2	53.27034	27.75479	44.42885	81.81012	50.23191	30.54371	48.68438	53.54945	111.7333
Ctnna1	141.9922	137.0434	143.0541	121.8682	127.3829	111.606	139.4847	117.9089	111.7287
Fermt3	112.1833	106.1996	124.1983	104.8425	115.5161	109.0745	133.2671	112.4815	111.6182
Scamp2	123.3035	109.6602	126.1824	121.1064	123.7453	108.7517	148.1381	113.8361	111.4955
Psmb9	69.67588	69.83337	86.53745	100.406	108.021	122.0622	76.27886	98.55784	111.3781
Rab14	140.4575	139.7246	155.7521	125.6428	123.2139	106.9111	147.2713	118.7734	111.2655
Hnrnpf	130.6882	116.3324	121.8856	102.8384	101.8137	107.7018	116.7682	115.7164	111.0978
Pip5k1c	141.3868	129.4664	134.0019	112.7561	116.646	110.3893	151.7785	121.5752	110.9083
Sfr1	136.4493	138.633	124.2624	105.2819	112.6911	115.0974	129.003	133.0875	110.847
Alas1	114.3436	125.8667	135.246	89.73015	94.51771	80.54406	88.78858	97.12599	110.8177
Slc38a7	141.1767	112.6482	126.3049	126.2199	116.8676	114.0011	153.5867	124.3312	110.7394
Hsd17b11	112.2466	125.7785	131.644	113.3375	126.2319	112.915	120.9362	122.4653	110.6818
Vps33a	120.123	104.4615	113.2226	126.5828	103.663	98.2192	145.6343	109.3498	110.5344
Tomm22	94.68864	98.37816	106.9364	91.52031	105.1332	102.4346	92.77353	101.4929	110.4735
Rps16-ps2	115.7973	93.35275	102.05	84.38906	111.4318	116.0017	95.39287	115.4138	110.4608
Capza1	111.8794	105.4484	107.433	94.90285	85.26691	81.83195	101.1164	97.83507	110.2963
Nr1h2	122.6768	108.7894	125.2062	114.1299	118.0467	115.7733	136.1613	110.959	110.2579
Mrpl12	97.76539	98.46326	88.45123	99.74654	95.90939	115.8089	87.96207	104.6503	110.253
Agpat1	116.557	121.3999	124.9643	104.9183	111.5241	107.0226	123.6539	110.7562	110.0552
Etfb	90.61637	89.15053	82.52206	125.3342	100.6272	124.1659	105.6491	97.0309	109.9463
R3hdm2	138.5613	129.6567	139.6389	113.264	116.415	98.33732	136.7917	118.1209	109.9147
Hmox2	94.76821	90.14266	99.88066	107.096	114.1626	105.8864	112.3762	112.8071	109.8967
Kdm6b	119.9311	87.24523	74.60429	65.23357	90.479	67.73554	136.3326	102.6209	109.8507
Wdfy2	120.6637	125.7204	133.089	109.7409	109.3962	87.71129	161.8926	96.24625	109.8286
Man2b2	119.0449	119.2271	142.7446	132.9546	125.7234	118.7928	155.4254	120.0286	109.783
Babam1	122.5113	115.1985	115.5195	112.3368	117.867	110.3533	110.3101	113.2696	109.7805
Mxd1	60.42059	46.18213	41.69636	47.33494	69.51476	57.30466	65.07785	79.35485	109.7096
Snrpg	101.1427	91.30666	101.2956	112.0786	111.2398	124.9724	83.83296	105.2963	109.7082
Pmp22	157.4753	166.7465	139.6838	122.2435	93.43434	113.834	135.621	116.8268	109.7011
Eya3	128.7029	115.3166	132.3003	124.7514	107.2633	94.98525	135.3455	109.3323	109.6842
Psmc1	123.6837	107.1943	108.7111	101.4831	101.9765	113.5255	115.6641	110.8713	109.6147
Mir703	122.0655	101.4504	108.3457	113.2896	135.6294	111.4713	105.134	124.9347	109.5528
Sf3b2	123.4663	114.8064	121.8338	111.2614	105.4829	104.819	125.9599	106.7245	109.5166
Ehd1	125.1276	103.6479	102.9783	62.47324	80.01096	66.07402	76.24128	105.2332	109.494
Lrrfip2	125.8298	111.2721	104.0679	98.54045	89.61868	93.36061	113.1636	108.5166	109.2205
Anp32a	99.13215	101.6003	105.4639	109.0414	115.723	119.766	100.6359	114.4481	109.2162
Arhgap25	116.9092	119.3222	122.8854	133.4729	108.2665	108.9267	141.1338	106.1303	109.1837
Hspe1	105.1013	106.6213	106.3408	109.6808	103.9473	122.8281	97.80948	117.1111	109.1147
Ints6l	106.738	108.214	107.6634	116.73	110.7554	104.1105	171.2651	118.3115	109.0388
Cyc1	103.4904	94.27677	101.7458	100.4029	101.351	92.45621	95.4191	104.7931	109.0227
Sf3b6	80.47461	80.55762	74.12473	83.67924	89.93741	96.51682	66.53962	100.5515	108.8943
Tap1	84.5885	66.51833	95.33515	109.6972	106.3525	98.99292	89.8855	105.8044	108.772
H19	76.40938	127.9379	103.7036	136.1775	143.6936	199.7722	84.24111	125.3237	108.7053
Vdac1	125.4795	116.8967	118.6147	105.3422	108.3528	107.659	117.8404	107.0983	108.6926
Selenok	86.80577	90.43088	98.74351	103.9192	99.58086	108.6284	97.71055	108.624	108.4776
Arrb1	131.9838	138.3774	149.4393	110.1646	116.3707	91.82134	137.3522	109.232	108.334
Eif5	115.5406	111.1155	95.74934	83.11623	85.56492	83.31117	97.1985	102.5996	108.2014
Hpf1	90.74557	95.02393	92.65346	96.42077	83.21301	93.56785	99.45452	102.2535	108.1861
Sulf2	171.855	151.4738	189.2332	115.7946	129.1583	122.5321	158.5214	128.1636	108.144
Ccdc12	81.03006	100.7485	105.7565	102.466	111.1279	120.3316	92.54221	110.8368	108.056

Orai1	116.3947	112.3796	111.358	81.02347	110.9217	117.5447	103.7105	110.6131	107.7718
Srrt	109.3978	107.2951	111.3748	117.6684	106.5426	105.1909	129.1552	98.48706	107.7078
Mcfd2	101.1574	89.09715	97.40571	114.0854	92.24941	94.20485	121.7621	100.7973	107.6234
Ppp2r5a	92.22289	90.7754	92.0717	87.68174	82.65842	81.32584	87.11825	97.80684	107.5949
Snx9	114.5345	107.7743	120.9585	121.3241	110.5703	99.32514	149.9831	100.6826	107.5821
Rab8a	104.0704	100.3033	112.0188	100.9393	101.1886	106.6881	94.14871	108.5031	107.5393
Prkcsh	114.6059	111.8209	115.467	106.0685	103.1753	106.6372	108.3464	101.8793	107.4522
S100a1	103.1238	143.8451	122.0681	104.4286	122.5099	106.2027	88.88178	117.4946	107.3072
Kmt5a	109.4195	96.9794	98.49529	104.0831	84.52602	93.06612	117.9344	100.5802	107.2643
Aurkaip1	98.54127	98.64479	98.09413	103.5066	107.4751	106.436	92.93746	107.7478	107.2481
Hadha	127.2871	114.6117	122.6679	114.5581	120.527	119.5043	136.895	113.7855	107.2311
Rpl34-ps1	87.11696	79.25414	81.08591	95.83914	102.2733	107.376	70.20046	120.6456	107.21
Got2	134.725	125.2092	122.1639	103.7368	100.3284	100.6216	106.2836	108.3092	107.1591
Ran	129.7028	123.147	117.8438	95.12389	100.705	103.7448	114.0451	109.3483	107.1297
Commd4	89.55903	89.864	83.98412	102.6022	96.90555	109.1479	110.8653	98.23764	107.1028
Bscl2	137.4052	120.892	138.5527	120.0314	125.2155	118.1206	148.9103	121.76	107.0878
Milr1	92.4393	99.31925	111.1442	86.90991	82.813	91.03058	77.67209	97.54735	107.0733
Icam1	101.3245	92.8159	102.9069	74.20931	107.2363	90.00142	97.03085	114.8433	107.0485
Gyg	125.3087	123.9447	136.4639	122.6162	125.093	125.2274	146.4987	124.9011	106.8442
Nrp1	122.6177	101.8127	106.5581	138.9243	106.137	97.75071	158.8574	104.0707	106.7551
Ppp6r1	123.8843	123.5399	131.1344	103.1479	107.3233	101.7373	127.0889	106.1077	106.7335
Vps26a	111.7579	102.4417	102.108	96.52447	96.7349	91.04949	110.7761	104.2789	106.7119
Cct6a	126.5057	118.435	112.0451	91.16876	93.05123	94.73999	102.9472	113.7047	106.6847
Ankrd40	137.5755	130.3921	133.1348	116.6766	107.1797	104.8048	132.3943	115.3448	106.6106
Pfdn6	84.28739	75.79626	77.94529	95.26893	87.14333	100.0461	83.88873	101.9778	106.5982
Serpinb6a	112.0403	150.7376	129.9995	148.0978	131.3274	139.8066	150.1946	105.3356	106.1722
Zdhhc3	143.523	129.5782	138.3269	107.7578	117.962	98.67876	136.8296	119.8658	106.1264
Tex264	118.5979	129.6364	119.9978	111.4364	115.5912	116.6791	120.6839	109.4765	106.0994
Nlrp3	121.4813	119.9643	71.61198	47.87728	81.45118	54.60462	87.0983	100.4287	106.0609
Ostc	114.2692	102.303	115.9009	102.1572	118.8217	108.3277	107.2584	115.356	106.0231
Tsg101	129.0273	129.1534	126.9444	101.7837	106.9427	98.37795	123.3566	106.7062	106.0012
Ndufs6	95.86622	99.76324	88.31674	104.6987	108.9363	121.1775	106.1032	113.5971	105.9881
Snx20	93.40192	88.64212	111.3916	89.18819	103.7563	103.4286	95.25693	101.8842	105.9327
Ap1s1	116.9613	119.789	114.4986	112.9259	110.3934	113.9836	110.5622	98.8123	105.9148
Neat1	118.7784	119.7642	138.7592	156.5726	116.4885	116.0397	140.2857	133.6383	105.8905
Trim30d	72.9868	52.50606	85.67085	97.56382	86.52049	66.75825	82.30577	84.5954	105.8015
Uqcc2	95.83575	88.2664	92.73902	112.3563	102.4009	124.72	91.47125	117.1629	105.7933
Cd164	117.9087	105.8529	122.5461	90.95068	95.83973	79.76335	100.2411	109.1832	105.6935
Rbm42	103.8364	93.02841	95.04772	95.76828	110.9671	116.7606	99.70729	106.2166	105.5816
Arhgap4	101.0129	101.8827	116.7445	105.3752	110.7799	106.1743	122.8819	112.8277	105.3738
Set	128.4319	109.6942	110.2893	90.49468	96.6563	98.6529	105.5984	106.1753	105.3201
Map4	153.3729	138.2498	147.7784	117.1989	104.3962	99.19663	143.7908	113.399	105.3142
Ndufa2	87.11696	99.46774	96.70553	105.0353	107.7898	121.6962	101.3661	116.4371	105.2359
Epb41l2	119.2585	100.7325	114.128	112.0547	103.2839	88.793	133.5641	118.2013	105.1603
Ncstn	121.3795	111.0598	135.5199	109.3598	108.4934	106.8641	132.9578	114.0414	105.0456
Rrp7a	96.41599	102.1721	96.92588	79.60361	86.73131	98.9297	94.63775	92.31537	104.9835
Anxa3	100.7744	121.2097	121.3738	109.7373	114.5898	109.1799	142.9759	108.4734	104.8477
Hnrnpm	110.7843	100.0036	107.1468	99.41216	93.63827	97.07899	110.183	101.7838	104.8215
Ndufa8	100.2766	104.4607	109.3547	104.4303	107.7888	102.0834	100.9742	108.4862	104.6779
Cited2	84.88865	82.4443	78.39386	93.37242	83.23355	78.78878	100.6862	90.60678	104.6014
Fuca2	116.0038	109.7777	122.0853	111.2161	104.012	102.8327	113.4306	104.8237	104.6001
Eif3a	126.128	120.6541	117.4447	99.68965	92.02013	85.39942	121.8247	105.7791	104.5825
Rasgrp2	73.02899	64.22201	76.55607	99.54759	92.39756	94.25311	89.47304	79.68123	104.5571
Tbc1d22a	107.4549	109.9542	113.4378	117.9271	123.1857	123.8101	139.6729	105.1328	104.5102

Cct8	113.4498	100.8224	104.6048	89.20468	91.42042	92.80018	99.25903	101.481	104.4152
Stx7	111.6785	98.57758	110.8389	105.6424	113.9791	102.071	121.4376	109.3607	104.4082
Napsa	99.93095	85.42172	108.5273	81.25626	98.35034	102.4308	73.22057	107.1709	104.3887
Nfkbib	104.3468	91.36882	89.14925	93.07559	111.6225	99.94702	116.6499	119.1563	104.3606
Rnf187	112.706	113.2043	121.7549	105.6208	110.553	108.854	114.3005	102.0261	104.3396
Srrm1	135.5153	118.4462	120.9931	114.7059	100.6645	96.91724	136.2948	114.1489	104.2731
Anapc13	74.46093	78.19265	89.82554	84.31269	97.83271	115.4773	81.59348	104.53	104.2503
Pisd-ps3	106.0546	123.9835	119.9716	140.6589	128.9997	91.23553	161.267	114.4327	104.2353
BC031181	107.7206	100.3991	98.82241	96.7808	90.62975	101.9626	103.2131	100.322	104.2005
Ubald1	115.836	107.9481	143.7717	131.6935	137.6345	123.1379	146.3456	116.3042	104.188
Arpp19	102.272	100.732	105.5108	104.3242	103.4555	107.8695	93.47558	110.778	104.1838
Aip	102.2859	100.2029	109.2062	97.08961	108.3383	101.7977	91.53036	100.3373	103.9445
Sipa1	112.2648	108.9781	123.3106	104.9021	117.53	114.2947	130.8678	98.40447	103.9051
Epc1	105.7577	87.77528	87.08909	102.6535	88.50414	86.6136	116.451	105.0586	103.8375
Lhfpl2	124.7421	110.8136	110.7594	117.961	82.01186	84.76256	136.5348	96.03991	103.7975
Atp2a2	147.0426	125.6369	134.84	108.3898	99.5893	89.52485	135.8819	107.7423	103.5442
Ankrd13a	113.7051	107.9634	110.2754	97.81619	99.90022	96.24652	117.1853	101.2489	103.4008
Hyou1	132.3417	116.0648	114.7397	98.15602	99.79727	102.9769	121.5925	101.1684	103.3843
Khdrbs1	116.3965	101.1639	112.1467	98.85228	102.9786	89.19066	111.2696	99.72051	103.1761
Rap1a	83.92611	89.58659	94.63447	87.06268	87.01385	83.25908	78.05815	101.456	103.1587
Zfand5	80.79547	68.96339	67.01379	87.00994	81.31366	81.58362	119.5811	100.4582	103.1188
Lcp2	115.9577	97.28497	121.1452	91.65598	109.3435	91.05552	106.6547	104.7634	103.0344
Rtf2	93.14733	91.27316	103.4602	98.90527	97.14253	107.7525	100.2849	103.5443	102.9121
Slc48a1	129.3774	125.9248	123.5539	114.7726	108.5026	116.1939	127.2079	103.559	102.8719
Mmp9	54.72217	46.2002	42.46962	40.69887	52.13342	59.24411	37.84375	52.35023	102.8623
Polr1d	96.81671	103.2709	106.2242	116.2184	106.1049	102.7196	88.08191	106.2628	102.7535
Ifi35	68.19697	55.69702	68.77565	81.4347	74.53921	80.21838	60.49995	78.74679	102.5279
Ciao2a	102.7655	115.4528	115.9588	102.6023	101.735	99.59425	96.97318	108.2852	102.4246
Coro1c	119.8308	113.6866	115.6331	105.6825	93.48856	89.6284	126.3691	101.6016	102.384
Eif3b	128.857	109.5557	107.8233	97.96938	94.59602	90.83281	109.8824	101.988	102.3257
Ikbke	112.6868	112.0913	117.4739	120.4687	91.26885	76.65753	138.4475	118.2277	102.2548
Sec31a	143.215	122.0084	131.7357	112.5156	103.1703	97.97428	140.4597	109.9286	102.1628
Ndufb5	95.35033	96.89518	108.4579	91.6193	113.6626	111.5265	97.17158	111.1642	102.1623
Dbnl	106.1014	85.41052	96.5633	105.1797	99.12339	108.1683	116.5165	98.06298	102.1105
Mfsd1	120.0351	106.7934	119.5836	96.81563	95.82823	86.92042	121.1955	105.689	102.0908
Snrpd3	87.19563	82.11184	92.16272	87.71118	93.27171	96.59318	77.02977	97.43784	101.8257
Rab11b	122.4281	116.6065	130.7506	108.0021	115.5602	108.0185	129.9179	116.8564	101.6227
Rps26-ps1	101.4669	97.39239	100.4614	95.46165	103.5385	112.2944	95.54048	119.3659	101.5544
Klf2	105.1064	90.30371	88.96767	76.13427	78.96531	82.43434	75.78734	100.7982	101.5432
Trim30a	81.10794	59.21555	85.09777	86.96525	81.1495	71.48975	74.48588	79.80056	101.529
Zswim8	116.2078	101.5469	113.9852	124.5453	101.2292	95.57188	142.8401	105.947	101.2125
Tnfsf9	159.5699	138.1589	83.50401	62.37056	93.20046	74.90527	153.4917	112.1788	101.0467
Stk17b	61.91549	65.16119	60.96362	43.0189	62.06938	64.20136	58.98104	70.69819	100.967
Slc25a4	108.5038	119.4639	117.549	100.0738	106.3107	121.9717	96.14192	108.8936	100.756
Igsf8	117.8059	105.8279	109.3084	94.24872	100.1321	104.7384	112.1081	95.14735	100.7155
Il10ra	88.15588	92.98033	111.1845	106.027	124.312	105.7532	134.4985	108.519	100.6993
Adrm1	110.7597	95.41379	102.2755	92.20069	100.2398	95.43124	105.1332	94.41007	100.6832
Hbb-bs	47.8034	69.56735	28.02594	102.5769	127.1628	207.8776	55.86331	44.68751	100.6309
Adprh	109.1251	97.34336	105.1099	85.11161	91.42197	83.91269	87.52153	98.12938	100.5346
Ckap4	104.9615	89.90629	84.27945	90.35997	80.23939	85.01768	98.59581	92.8899	100.4908
Snrpa	99.39283	84.54424	105.4693	89.21336	100.7638	102.8132	108.9951	85.86876	100.4689
Cd300lb	127.7404	124.2833	133.0416	118.1811	106.0367	106.744	112.1246	115.0909	100.457
Oas3	73.2471	37.45287	75.6477	79.82054	75.72298	55.69112	52.92075	75.94642	100.4041
Slpi	56.19749	36.98655	36.58325	23.28446	47.73572	50.79919	22.43518	57.8438	100.3066

Eif4a-ps4	108.0076	96.02755	91.0874	83.96251	75.39688	87.07958	99.44974	90.06862	100.1771
Naa20	91.99223	95.47531	84.91457	110.3033	84.79215	85.61025	81.73244	92.90084	100.169
Marcks	79.43562	86.47557	96.22655	120.0005	132.2362	102.9879	120.3313	103.8627	100.1588
Ubtd1	103.7814	100.3785	107.465	94.79145	108.8578	102.6111	106.1548	99.79186	99.93502
Mtch2	95.26999	99.45045	100.3627	92.25515	83.77782	87.89294	100.1074	96.45111	99.92408
Rpl21-ps15	71.22043	83.18476	77.12281	80.35706	92.27864	111.5874	84.03707	96.435	99.86744
Arhgap9	94.92643	103.2687	112.7865	105.0429	107.8941	102.8619	101.2353	104.3097	99.72906
Ciao2b	101.0114	90.66011	102.1647	87.39334	108.0818	107.0584	85.10439	87.01345	99.69174
Nrbp1	102.7387	97.00309	97.24749	93.42908	100.4797	97.06402	106.3724	99.26843	99.64257
Tmem184b	114.3013	93.50058	121.4352	103.2326	93.81328	80.57574	114.596	91.32961	99.64095
Pik3r5	126.3023	112.1078	118.9987	104.4623	95.79864	88.0412	148.1059	95.4482	99.61575
Ndufs2	100.7313	106.7995	103.6467	88.95809	93.82081	95.11158	91.11901	96.32882	99.52183
Sh2b2	111.7542	98.96235	105.5026	80.0426	85.52593	74.08597	109.5136	102.9175	99.48161
Samsn1	66.9163	65.41957	60.94388	40.84516	56.14681	65.80009	46.55269	75.86944	99.40304
Anapc5	115.0575	113.7672	115.1178	97.01043	94.67953	93.60642	114.8145	92.99555	99.18207
Pqlc3	112.7739	115.5995	124.9802	84.77858	90.42583	86.63337	100.0932	102.225	99.15234
Luc7l3	82.13603	99.58874	82.38585	100.0418	96.98812	98.64667	91.37883	97.78767	99.03434
Polr2f	101.7176	107.9573	94.71364	93.59084	111.3503	123.258	93.95262	106.0288	98.98887
Txndc17	105.5854	103.163	94.93817	102.5158	101.8728	110.0157	111.0694	103.4843	98.95137
Isg20	62.96903	30.16991	56.71036	62.93096	65.81744	59.57785	39.44143	54.59009	98.85901
Vwa5a	107.8468	99.29524	113.7421	101.7178	94.45788	86.98245	108.9097	96.75708	98.8128
Cdk11b	108.9915	104.723	98.32368	96.51221	93.63152	99.1944	109.8888	97.96905	98.78947
Rpl28-ps1	102.0463	96.64685	112.9011	104.4756	105.7037	107.9096	101.6578	117.9092	98.75054
Puf60	104.9603	86.52442	100.5808	89.54432	90.14679	97.34599	102.9662	99.64215	98.72863
Kctd12	107.3706	116.131	117.5255	100.7895	108.7702	83.99827	137.7536	104.7545	98.63504
Piezo1	140.9252	114.5423	120.0171	109.8109	89.71853	73.06049	125.7636	89.86353	98.60987
Tfg	110.9993	102.6297	115.9686	97.7052	97.09549	90.77828	113.3552	89.09423	98.60438
Csrp1	116.1208	128.1884	133.3845	110.3285	108.4464	115.5139	109.1372	97.899	98.5641
Comt	100.1633	110.1346	113.2109	116.0126	117.5581	111.3552	125.2057	105.8046	98.53028
Rgl2	92.64191	104.5441	105.9858	117.2866	112.1342	108.8473	120.014	98.52728	98.52198
Pnpla2	98.38178	102.1988	105.2704	92.75815	108.6577	94.45086	127.1844	103.1687	98.52129
Fbl	102.3141	87.63468	87.83099	90.42056	98.17169	99.57108	92.15141	99.32577	98.47177
Ube2s	120.8078	104.8169	103.1447	79.28766	89.19116	111.875	95.80598	84.99687	98.45511
Med15	125.2347	106.695	121.4968	105.0652	90.11267	84.94149	133.8113	107.0534	98.45407
Rgcc	83.52102	82.81472	49.31771	60.97804	47.31078	53.95976	63.31618	80.61743	98.33306
Stard3	80.10746	72.37186	78.53731	98.31436	88.87074	84.30413	91.9132	89.0769	98.2786
Nsa2	87.14054	88.30831	91.25844	85.72884	88.12217	98.5647	78.67276	99.53209	98.21933
Mafg	106.6481	104.6437	110.0095	94.81665	86.17222	82.10914	118.4765	98.86649	98.16344
Trib1	97.51922	83.37517	69.50532	63.49387	81.8339	63.36476	83.07214	89.41293	98.11535
Ppp1cc	97.44467	94.22528	116.2546	89.10454	88.5654	77.9039	88.8768	82.30732	98.05925
Rnf19b	94.87634	85.55866	102.2169	84.29981	92.30848	83.50539	108.1133	99.02737	97.91385
Scarb1	104.9183	91.06814	103.9963	118.5392	125.4087	110.4257	123.1542	117.3665	97.83099
Entpd1	103.2599	108.491	125.8775	100.1091	118.3014	99.34058	118.0315	91.75013	97.80979
Lman2	103.2189	96.26821	110.6449	101.0565	96.49358	89.57238	103.5215	91.49777	97.67878
Tnfaip8	95.3387	105.1255	106.9287	98.94256	96.55241	102.5037	100.4449	101.0279	97.58287
Rtraf	97.58568	100.1087	95.61423	90.28501	97.38021	105.6583	90.99379	100.6362	97.55673
Actr1a	127.7118	120.7666	118.9158	93.82143	105.0863	109.1364	116.3233	101.6755	97.53387
Ssrp1	114.8142	106.8969	117.1479	105.7623	88.79237	99.62698	107.1813	94.31669	97.49897
Slc39a7	107.3521	90.75191	91.89901	92.94271	93.72504	90.18001	103.2343	85.20813	97.47848
Nt5dc2	114.7971	84.36595	96.32893	78.0522	85.71561	78.05938	102.8137	103.5601	97.33569
Tpst2	91.48582	83.42242	89.18626	89.22636	90.07368	90.98156	93.64727	94.47566	97.33368
Ifi205	64.33977	34.55226	57.67879	73.54894	49.09976	45.29202	34.13725	58.18179	97.25619
H2-Q4	72.53636	69.93797	83.08945	96.94204	89.48966	85.95079	103.3793	119.6363	97.21728
Tank	106.1767	108.5233	111.2329	92.74038	70.37388	75.7881	99.43237	98.87164	97.19023

Rps7-ps3	73.62367	85.65229	70.82795	80.77935	94.83911	84.45783	65.49526	100.8359	96.97037
Ccnt1	103.5971	107.2811	102.9474	84.08044	85.64018	84.3804	119.6115	97.72457	96.91528
Stk38	93.20806	106.1158	113.1128	93.40306	102.4779	94.50427	119.2544	99.55718	96.76833
Ndufs5	82.99372	80.383	70.14983	87.07766	88.06683	100.8225	77.44799	99.86153	96.74746
Plod3	113.8696	103.9522	118.1621	84.49075	90.5211	85.29967	89.89739	93.03756	96.69663
Ergic1	131.4619	110.1274	116.8979	99.92459	83.64338	86.44177	111.1571	83.34434	96.66324
Copb2	100.4371	92.01148	104.0199	89.96493	86.47658	80.58229	96.06783	91.94869	96.59364
Banf1	111.7446	98.81548	117.331	100.1076	113.1793	108.6253	112.726	101.7062	96.56966
Lamtor5	95.63693	88.43974	90.88132	84.06324	98.90779	83.97644	90.35455	91.11544	96.47009
Rab32	104.2905	93.54157	105.5983	78.8533	90.09694	85.61204	85.05434	94.36087	96.43983
Capn1	104.3271	97.71311	109.0177	88.98725	100.7276	91.42818	98.89977	103.017	96.42618
Rp2	88.44729	105.3246	122.5803	86.06041	90.08597	72.30204	95.87893	90.88847	96.42164
Eif4a3	104.0553	85.22216	91.10635	92.771	84.16018	92.02198	93.72121	97.77204	96.39304
Per1	51.18551	40.52338	32.21357	47.65321	54.34149	58.10818	105.6455	75.99814	96.30848
Malt1	119.7367	102.1502	108.2407	80.10877	60.97867	59.86951	114.5609	95.90054	96.25919
Edem1	105.6127	96.79731	100.6424	97.1028	84.39438	75.94204	108.9621	88.07002	96.22784
Pebp1	93.24101	92.43145	90.4374	102.9327	96.75277	90.99744	86.09243	89.89096	96.19804
Zmiz2	137.6074	128.1308	142.2333	113.5847	111.7484	105.3592	134.2022	108.8463	96.19424
Srsf11	99.46738	105.3975	95.16847	102.8599	99.82236	89.58116	111.7136	105.2833	96.17051
Saraf	109.669	113.4292	118.6919	97.46125	99.31633	87.1246	107.2017	96.31469	96.16238
Mea1	82.30116	73.67685	79.61094	93.36324	90.48502	112.5203	79.21007	91.96186	96.13645
Cytip	78.72574	68.20386	68.18612	55.67834	71.8782	73.05922	65.62315	83.39279	96.12799
Mmp14	84.49163	74.44794	102.5745	75.02623	79.85772	73.96747	75.73406	66.26623	96.02756
Mien1	94.70669	89.84702	91.43872	93.28703	85.51387	90.13137	86.87117	95.49086	96.01635
Cept1	91.2176	82.00328	101.8925	93.06853	90.67691	75.84956	87.54007	94.1135	95.99356
Mrps18c	61.95369	71.26083	67.37281	74.67473	85.82403	91.91364	62.206	81.69717	95.99145
Psmd2	119.0954	100.5909	107.0879	91.58132	92.18804	95.60908	110.9	96.65199	95.98526
Creld2	100.7558	96.75832	92.92239	92.10823	83.10748	104.151	97.66705	86.17783	95.87431
Pisd	98.83446	100.5515	98.9491	92.87065	97.2231	92.06668	95.94085	93.09355	95.77086
Ttyh3	113.0656	90.19236	113.0666	103.5671	115.1362	94.87207	124.6979	101.7874	95.75986
Sra1	92.11937	74.95498	75.48392	78.55354	80.35631	89.15552	78.53664	84.11063	95.72965
Arrdc1	97.26513	93.23117	100.909	99.15351	108.3646	106.1765	113.8057	97.84778	95.69035
Ly6a2	56.61481	22.95076	53.28688	43.55595	46.94593	70.91585	21.48675	72.26468	95.64317
Chd4	119.137	116.8912	117.0654	100.339	94.46206	82.88818	124.8288	94.5944	95.62711
Tex261	124.4116	124.1184	128.2011	98.99448	112.6419	95.37708	127.758	105.6012	95.6088
Clptm1l	115.4921	105.675	110.4227	95.18655	94.76217	87.91774	111.6082	98.41777	95.58725
Tprgl	89.93569	95.06025	92.3969	84.36853	96.9894	93.14654	92.09783	101.9895	95.4531
Cln8	98.74765	88.86128	94.44777	94.26318	84.96278	84.13056	114.9654	98.90865	95.41387
Phc2	101.8711	106.6024	102.9977	97.88782	97.4679	91.63028	106.5318	102.3533	95.38688
9530068E07Rik	101.82	113.0239	107.2706	86.72474	88.53986	81.17163	104.0747	95.017	95.33006
Ndfip1	98.49825	101.1298	108.0489	90.05799	92.30936	86.43521	99.66535	102.2734	95.28972
Tfe3	123.726	113.2019	123.779	94.28747	97.05815	91.11621	111.9198	105.9329	95.2878
Trpv2	99.0611	98.06761	107.5529	90.63085	94.48819	88.32479	93.93225	92.63569	95.27551
Naaa	121.6519	105.0473	130.1043	97.10014	120.2708	124.1612	105.4255	107.2676	95.25154
Cast	110.6111	110.8831	122.137	89.3563	85.49118	78.68703	95.35969	88.115	95.22642
Hprt	88.56118	86.11214	85.20123	84.30547	86.77132	87.21916	93.44374	97.03523	95.21699
Septin11	157.9511	148.4731	142.8508	89.53579	95.25098	83.29289	118.101	108.3833	95.15662
Sh3kbp1	114.0222	109.9629	117.1343	95.00768	99.3776	90.77956	122.2817	101.8888	95.13458
Atf6b	90.50275	81.68596	90.77255	80.22065	82.20204	83.37443	84.42859	85.22127	95.13203
Wsb1	85.79531	84.76456	79.62901	87.12643	83.17527	68.4878	105.1319	99.48245	94.91789
Gm10443	79.98185	59.22756	66.43041	75.43767	87.77972	101.4237	82.84263	93.9626	94.8783
Maz	112.4678	117.1748	116.3992	106.4565	114.4125	116.8688	115.1781	111.1836	94.86927
Psmc2	107.0989	95.93068	94.4808	90.41218	92.55199	93.71382	88.49052	96.81024	94.83822
Luc7l2	101.3629	98.43006	95.63831	99.10367	83.276	76.48955	116.9672	90.5757	94.83587

Il4ra	122.4198	96.24116	132.8279	82.03796	150.4114	122.7103	127.8851	108.2842	94.79681
Ndel1	105.4681	96.61671	93.87286	71.28517	81.11465	68.94264	80.24273	96.48965	94.70721
Dclre1c	79.91721	84.83343	93.05899	111.5165	108.3073	82.49705	137.5932	89.21119	94.66292
Serf2	70.47335	75.89989	77.81844	95.14148	98.26455	96.75816	85.09307	92.90959	94.63311
Celf2	105.4803	102.0678	113.506	89.25922	93.39441	74.89844	112.6003	98.76095	94.58101
Bcl3	120.8034	99.9086	124.3956	67.53122	108.8818	103.5184	98.33937	104.6136	94.49293
Psen1	109.9075	97.36467	107.8179	93.14297	97.94239	87.70725	117.429	98.50111	94.36092
Dusp3	125.6984	119.4057	114.2897	91.08838	84.03763	83.02502	112.499	96.58602	94.30518
Tmem65	105.993	104.8872	90.60006	107.6486	87.98627	80.9852	122.6489	92.50606	94.24495
Mcemp1	69.98321	56.60924	56.13022	36.71922	49.48703	63.94836	26.32064	53.71502	94.23914
Tbc1d20	105.9774	102.4916	104.5173	87.49598	88.84748	87.08772	103.5293	91.49641	94.19395
Gmppa	90.93755	84.27486	90.94812	97.34197	92.59901	97.49988	106.0793	83.32415	94.17469
Ssb	85.58958	89.5085	84.43972	82.74754	83.85819	89.14247	87.55486	95.00844	94.10988
Txndc9	89.73613	88.96652	100.0033	92.64888	83.94333	97.52434	88.74691	95.52429	94.10221
Mrps15	105.2023	102.977	100.8404	99.67621	106.3852	112.9741	98.40841	104.2707	94.07085
Smim10l1	96.95813	102.1824	97.43729	112.5811	95.01521	102.9674	86.79318	103.6719	93.85884
Psmc6	87.55244	87.92033	83.41491	80.71741	75.1131	78.76952	73.41765	86.59835	93.74922
Ccni	91.92708	108.2527	116.1474	80.91365	110.5646	93.95779	99.5542	104.4837	93.64808
Tmem164	98.85609	103.7487	106.829	85.75042	90.07176	72.23774	105.6145	80.95526	93.64431
Klf4	117.9243	94.68766	71.56421	74.83145	70.78551	59.57055	102.2103	95.88931	93.61107
Pla2g15	80.02566	79.43405	89.88379	103.8595	115.7794	103.4123	124.36	96.44997	93.49547
Mtss1	117.8918	100.2478	99.11655	108.1154	79.30123	80.07812	134.2373	89.58903	93.43913
Fam20c	115.8639	109.5492	107.8036	103.8624	77.39456	81.31714	134.7964	95.01196	93.36779
Ralb	83.39068	74.52307	87.47565	81.8396	86.31682	83.02148	82.0574	90.83725	93.35597
Rin3	108.2179	105.103	117.199	86.16263	119.6128	112.3381	127.2737	108.4617	93.28197
Ltbr	106.0875	91.16236	95.15298	94.92295	93.04282	97.66838	96.94703	95.24392	93.26164
Inf2	134.4085	130.0595	135.4247	91.26644	82.00458	81.25549	106.9308	105.0887	93.23759
Eif6	96.26569	80.99407	81.33097	74.85544	82.67661	80.45036	79.9106	84.98154	93.23415
Sharpin	93.43058	95.06675	103.5707	100.2319	112.9816	104.3721	105.2801	110.1788	93.20443
Gsto1	93.76039	106.4541	109.4312	95.24577	111.1055	98.53426	108.7642	108.072	93.12648
Gga1	102.0872	89.44154	97.34769	101.4063	94.70336	94.42391	106.7964	92.27364	93.08368
Csnk1a1	98.04107	96.44775	104.0104	82.70026	83.34993	72.38534	94.35233	91.23066	93.08054
Rragc	97.36762	94.49876	103.9106	100.9261	83.67545	81.77994	114.9038	99.48868	93.00274
Gng10	85.03803	79.23322	93.61211	71.41968	83.39351	86.33061	72.64568	99.06138	92.98439
Tmed3	101.4969	104.484	104.9844	98.6383	101.0511	102.2637	101.3967	94.04369	92.98424
Rer1	86.79555	91.08728	97.63436	82.70877	87.01558	79.77816	77.28146	85.61671	92.80887
Gda	119.6706	101.9305	117.1668	52.15416	62.00145	59.33923	63.43342	94.32394	92.7981
Rps6ka1	109.6322	102.1374	112.5787	99.104	111.1317	101.4703	121.0435	97.08015	92.73972
Pdcd6ip	130.8724	113.0853	135.4183	107.7275	102.376	91.31262	126.4603	104.5403	92.72187
Psmc5	95.33437	84.68924	86.01062	84.48282	79.01185	93.82293	84.48876	91.80099	92.6413
Ndufa10	112.103	105.4598	96.75521	90.55374	95.34043	106.3588	107.786	98.42633	92.46545
Daxx	63.58744	46.02715	70.98594	82.52477	69.27823	54.94261	66.13616	68.10915	92.43885
Npc1	119.0717	99.01513	109.4077	103.8291	88.99381	88.56382	126.6158	100.8121	92.43669
Gtf2f1	94.11578	84.60728	95.0293	90.06299	85.67494	87.3632	92.30975	98.44544	92.43473
Syncrip	107.8099	103.4372	98.69613	80.08127	82.25836	79.62324	96.26572	94.72818	92.37193
Sec61g	65.95218	52.79727	66.47458	78.15825	88.51119	72.07351	60.48967	84.77397	92.36033
Gm6136	97.99717	86.0593	89.87045	85.33997	87.86758	92.41122	93.78237	105.2305	92.28412
Nabp2	112.7781	107.9587	115.4208	87.61438	100.5358	100.5172	103.5527	109.4531	92.13221
Axl	85.64997	89.7446	129.0353	111.8044	139.0111	105.4164	129.4402	121.2891	92.10771
Son	103.564	94.73597	101.0648	102.6586	87.23437	72.3274	123.5454	88.0553	92.07747
Srsf3	88.1433	86.51836	87.25271	79.26848	74.89845	78.51744	91.43299	92.08856	92.04132
Naxe	86.48064	87.61729	89.02647	97.71677	102.1382	109.1888	111.6922	98.91004	92.02459
Lsm4	93.77441	89.54498	103.5487	82.90386	94.19597	103.5891	88.45103	94.16718	92.024
Nxf1	91.99223	103.1734	89.79388	101.3487	96.30428	91.24427	118.7353	96.52906	91.7483

Fam234a	100.3929	100.9155	111.4518	99.43679	108.4158	91.35617	126.6372	100.6637	91.6709
Dnajc8	84.94927	81.46606	90.25665	74.29543	84.01502	94.06387	78.4143	94.11357	91.66833
Ccnl2	79.74912	86.7518	81.50564	90.46004	90.43489	79.75685	112.3834	90.94609	91.65049
Tspan14	106.7803	107.8871	116.1368	100.3479	103.2145	96.47441	113.3146	102.8879	91.6425
Midn	111.9364	96.56206	99.56233	78.75361	87.60499	72.88258	109.8309	90.83601	91.61953
Fnbp4	93.05181	85.98593	86.97286	96.46629	78.84205	72.79303	113.9953	92.09922	91.61472
Tmed2	105.4586	100.2227	100.6142	84.82661	82.44602	83.37764	96.66681	92.25574	91.60266
Flot1	84.83344	73.63056	85.24974	86.95129	97.1146	95.92669	81.72929	90.02361	91.53748
Atg3	76.98633	69.2328	72.39137	68.18626	73.48232	85.55881	72.15521	85.34465	91.51664
Naga	113.4369	97.77647	115.4921	99.69066	113.7708	111.7834	121.499	92.97886	91.49247
Mmp19	192.731	188.1866	146.0027	73.08854	77.18454	56.35033	86.22668	94.74437	91.184
2310022A10Rik	98.59116	76.27462	75.24117	91.81038	73.75985	70.75667	100.8974	100.1285	91.16265
Sp110	69.07956	49.38868	64.38415	81.70023	71.33744	69.55659	69.4014	71.25633	91.15935
Ube2q1	86.69282	84.20087	97.14036	87.67441	88.9402	87.22611	93.46958	92.59631	91.04633
Bin1	102.4003	98.33939	100.9994	103.0129	95.7909	98.2383	117.3863	87.39294	91.01436
Emc7	91.63829	98.71825	93.25228	80.73714	82.99034	82.68779	87.17635	89.11965	90.98679
Rps27rt	66.70355	68.59566	60.63316	71.6363	85.12716	101.0269	50.55706	86.08905	90.98204
Myof	132.0072	120.2717	128.6982	115.2724	86.10234	75.7917	135.6945	91.31253	90.95174
H1f2	81.74247	92.6592	84.1227	96.18803	102.1016	99.14051	100.8445	100.3959	90.88033
Ap3d1	109.2239	98.06433	99.56807	107.0273	93.26526	86.01997	129.4564	94.18508	90.87675
Rab31	96.15066	96.13292	97.08335	80.30735	83.54498	77.24033	95.32032	85.79385	90.87577
Trpc4ap	102.0032	92.865	103.3078	88.61394	97.0636	92.10039	108.9817	89.05171	90.84237
Qk	93.18055	87.81926	97.10668	97.0336	88.37475	71.24082	110.863	90.8057	90.81933
Cd177	32.6675	16.26489	31.65349	30.51115	29.12525	59.76097	17.03952	36.06842	90.76199
Rogdi	73.53305	89.88019	95.80811	74.09139	105.8506	99.82785	94.8855	85.80318	90.71125
Arcn1	107.5396	99.94201	104.4077	82.77104	78.61737	71.27887	102.3937	85.22169	90.63082
Ilrun	93.36769	80.97522	89.43073	79.40706	81.53996	72.40433	83.0928	79.06005	90.56539
Arhgap17	83.77913	74.42883	85.66372	109.6958	98.82899	89.72186	126.9081	98.01995	90.55287
H2-T22	66.68304	61.50288	85.74062	93.23756	81.71138	81.19104	74.91773	90.58233	90.52037
Hax1	94.29366	89.89279	98.89439	82.81298	81.41242	85.95492	88.03402	85.34914	90.51175
Sgta	102.7606	99.02971	97.39743	105.3649	89.60151	94.25942	101.5699	95.45875	90.4357
Prpf38b	84.98673	90.19828	81.81267	87.16699	85.1225	88.38971	88.63226	88.47596	90.34672
Phf23	89.69991	86.47907	87.88095	85.13984	89.2484	88.41672	102.7074	97.1251	90.28624
Fgd4	83.28669	76.14288	81.68344	92.41378	69.25531	65.43583	109.7443	81.8471	90.27647
Osbpl9	95.17663	95.39924	110.0851	85.55132	91.91311	79.93252	99.33039	92.1481	90.25691
Snap23	94.9813	94.03247	92.58845	80.98221	81.91783	74.40343	90.5816	89.07811	90.25465
Tubb4b	106.649	107.1944	103.5773	83.34593	78.14193	88.88483	92.82277	85.30967	90.21986
Eloc	78.25184	73.18027	83.3119	73.36867	71.43015	80.25627	68.9976	79.03553	90.01722
Col3a1	91.60632	99.07369	98.27349	139.8598	92.19828	135.8162	75.31948	93.93458	89.96792
Cct3	97.73189	83.82567	88.62055	84.84625	79.0765	78.90514	86.07876	83.90713	89.90727
Ptbp3	96.68524	85.97619	97.08588	73.81091	80.54706	62.50607	86.46074	84.22164	89.88358
Cib1	87.09908	76.57044	98.4359	78.2114	85.63385	80.25718	90.3446	90.96933	89.87129
Dnajb6	89.88886	96.36339	106.4337	87.95793	91.73757	83.99612	94.33707	95.47972	89.87075
Apobr	87.41899	78.33002	91.65816	77.92213	76.30469	76.08813	71.53625	82.17199	89.80873
Snrnp27	62.0031	68.84854	61.34709	74.05288	73.19806	83.27884	65.45406	85.4874	89.74773
Kcnn4	143.4233	124.1486	128.5279	85.0071	82.47077	92.18674	88.88236	85.63631	89.70448
Sf3a2	100.2455	84.55413	93.53362	93.82217	96.13509	91.96891	99.296	102.4749	89.67207
Snx12	108.0488	101.071	99.52289	91.56682	80.99306	76.95234	102.5634	94.5334	89.66507
Pxk	100.2197	92.27365	100.7858	70.07321	71.01412	68.81222	94.54171	82.72186	89.6181
Lsm3	70.19812	72.72756	69.3276	73.99844	80.57854	82.22281	57.11639	69.09401	89.59749
Ddrgk1	83.63544	90.34292	92.35715	93.43139	97.20506	102.5813	105.1764	93.96334	89.50204
Yipf3	100.3634	100.7245	104.7854	85.27539	101.0223	92.88572	99.86456	94.61857	89.50085
Wnk1	129.9516	121.1223	131.4571	102.5889	87.69197	69.71877	138.9619	90.48111	89.49908
Psmd3	106.5898	87.09472	89.4033	83.5001	86.78448	78.79943	103.8943	90.61769	89.45689

Cdc42se2	91.5251	85.24569	97.4949	90.25089	82.15093	74.53462	108.8623	89.66618	89.45685
Daglb	98.93531	97.26033	97.69079	107.9556	91.3581	85.90651	115.3995	98.03001	89.43029
Dpp7	102.1173	99.90416	101.9553	113.9533	102.5518	109.0597	118.0087	97.58966	89.40949
Ndufb10	88.43886	83.87491	83.12581	87.53632	95.45863	90.19122	94.79138	93.16846	89.28506
Dlgap4	100.3202	107.678	108.7931	91.05593	94.86008	86.09762	105.531	93.53785	89.17814
Rnps1	116.1119	87.74803	103.1542	92.35562	89.8554	98.26249	106.355	100.9057	89.17126
4933434E20Rik	69.4072	73.2833	73.98866	73.62795	80.06297	65.69177	75.49093	84.98872	89.07729
Sft2d1	60.18487	63.29765	64.1187	70.74505	81.31878	86.60178	76.43772	82.40984	89.03046
Sec13	85.94817	72.33235	79.94919	88.00912	79.33995	88.93965	82.18146	86.05044	89.0235
Rpl6l	90.63683	86.41696	82.02267	75.0201	91.18235	83.27559	76.63987	93.05354	89.01284
Rab8b	98.42561	100.654	109.7453	78.39225	74.68041	69.47157	107.5167	87.12274	89.00394
Ssu72	75.87062	72.13946	72.49265	76.99692	80.70178	74.24446	79.83181	82.63336	88.88433
Slc39a1	116.6233	109.4723	116.968	94.12131	99.20011	87.99005	105.9915	98.49425	88.8588
Atp2b1	107.7399	96.59733	105.3957	95.21193	80.57557	67.35097	112.1519	89.20565	88.83492
Usf2	68.1998	73.27356	77.64976	67.49028	80.79232	81.34256	75.08308	74.33967	88.82961
Blvra	88.9179	93.47336	95.69732	92.58136	80.45267	81.36846	93.18268	88.79064	88.78456
Ogdh	124.3682	117.9891	127.2165	94.79775	93.12579	85.69869	116.115	89.65582	88.77743
Ttc7	98.92983	82.84556	99.81518	81.5378	79.51355	78.48925	102.7783	91.25056	88.60315
Snu13	97.24815	85.157	89.23169	75.7839	81.65512	87.93356	79.86166	91.91792	88.48268
Gm5805	69.16558	89.30728	73.92601	76.02348	89.60677	98.04277	70.39469	97.62243	88.4649
Spcs2	90.63089	88.83794	90.799	93.37408	84.32772	86.98154	98.19026	85.08947	88.36891
COX3	87.25838	111.2862	76.16548	127.2715	133.1414	235.7384	49.33183	99.977	88.35437
Pon2	106.888	115.9855	126.4476	91.93975	108.0327	93.80721	118.8123	94.95627	88.35249
Vac14	104.644	92.95479	94.53939	89.46661	85.75344	88.87924	117.9849	85.35085	88.29874
Hsd17b12	106.5954	91.06843	99.1911	83.60558	74.9344	67.9941	84.4982	89.34846	88.23472
Slc27a1	107.6758	106.081	97.70127	101.6294	89.64104	99.67221	127.6824	85.90055	88.15678
Zbtb7b	89.15184	80.61864	98.25821	84.57735	88.60706	86.98127	84.97866	94.38184	88.09137
Tmem214	106.6556	87.87545	100.8827	101.5897	94.0586	92.40986	106.5132	94.57352	88.04656
Slc25a11	96.24849	86.19984	101.5369	81.79463	83.07829	84.09704	95.23205	78.63316	88.00928
Zfp385a	94.62242	95.14822	108.1107	107.4199	125.8115	108.9573	135.6515	117.6527	88.0004
Hdlbp	116.6813	103.3371	105.1365	91.92046	85.5649	77.34502	113.3005	84.88701	87.9663
Gpr108	93.05393	91.70511	91.88172	85.57967	93.88088	84.27882	93.26887	85.81844	87.91201
Stau1	94.81428	98.23526	96.80003	91.94214	93.66328	83.39729	114.7922	86.47648	87.90551
Irak1	112.3641	102.5763	111.137	106.2645	97.28771	84.31285	123.8699	95.97008	87.90476
Ola1	86.88379	86.38119	93.58641	74.98209	77.15125	79.08963	84.85053	88.26431	87.88949
Mark2	89.87571	87.19752	92.55879	80.87376	87.87762	79.12756	100.9164	84.25033	87.79802
Slc16a3	81.97419	70.15298	63.07941	42.97667	66.10859	57.60532	59.03736	75.30732	87.77563
Slc35c2	85.70119	73.201	81.88625	80.55828	82.98281	80.20716	89.12641	82.8308	87.7665
Man1c1	104.2576	89.66235	95.25486	112.0101	108.4337	92.78551	144.776	91.35658	87.71188
Cnppd1	83.72164	84.08994	94.51538	73.56886	89.38664	79.19819	93.91155	95.13744	87.70362
Rpp21	87.81389	80.50293	89.26565	93.49628	91.76397	104.3804	81.01938	81.49613	87.60441
Gm5150	51.77971	47.64242	65.99866	59.60489	85.2282	62.78303	52.49893	78.16896	87.50356
Mdfic	81.6135	70.96375	85.13754	86.46334	76.64868	74.04614	94.82345	88.38249	87.48846
Ogfr	68.31242	55.8031	66.85163	84.65057	73.98798	66.18359	70.04776	78.97562	87.43248
Bloc1s1	67.23125	70.57075	70.86816	92.71447	98.2054	86.40885	76.36261	92.51327	87.42188
Commd3	62.52549	69.24787	64.87548	71.85093	67.09942	78.98399	63.94035	78.17295	87.35631
Retreg3	84.78507	92.9157	103.5948	88.67027	86.28871	93.74263	99.38534	94.36365	87.30377
Ndufc1	71.90879	69.58052	82.09537	92.67816	80.09064	86.06481	57.57481	77.1338	87.29657
Ptk2b	87.78166	75.8334	88.51542	79.48915	80.73809	74.68404	97.50044	85.81878	87.27365
Tram1	102.9268	95.75791	96.1754	83.59978	90.65285	80.81189	100.6755	92.2931	87.27237
Rela	104.2296	97.19415	105.3424	81.26428	92.83429	80.98211	104.4925	92.12791	87.20536
Bcar3	88.91801	61.39285	65.38849	98.62601	69.50524	77.04938	109.4196	80.28949	87.19229
Arhgap10	108.2521	100.0758	96.80611	97.28171	78.56432	80.14801	122.3121	80.65161	87.14648
Plekhj1	77.6494	80.50437	85.36407	94.51288	95.27826	92.34809	81.18773	101.9544	87.14442

Scnm1	77.51603	79.05567	79.37319	79.30626	91.05443	97.94769	111.3793	93.98984	87.13536
Irf9	80.66084	64.04539	88.90525	79.73845	86.14984	76.78073	82.55999	78.30639	87.09263
Pdcd5	72.53578	81.90224	65.80797	77.19571	81.91185	94.70891	62.38521	93.06815	87.07472
Stat2	64.24424	47.78006	70.81685	97.557	75.84786	57.37228	77.063	70.43559	87.07074
Ubap1	69.3523	70.60216	76.64664	64.85957	67.21176	62.71797	78.72416	77.96801	87.04909
Cyhr1	88.31544	90.79396	88.72255	84.26958	93.82819	92.15548	101.5311	85.00244	87.03584
Znfx1	86.31411	61.05517	90.38269	106.7374	79.9035	61.71097	94.24577	70.24529	86.9597
Arl8b	102.621	99.42088	97.36873	88.22663	85.92396	79.29986	110.0169	83.26579	86.90742
Ahcyl1	106.5019	110.6442	117.7681	89.60981	84.58644	71.52225	97.2985	89.84186	86.79351
Snrpc	95.65069	90.16488	95.37038	95.25978	94.01066	93.91161	87.26464	105.7037	86.71999
Irgm1	62.49831	47.56405	74.04507	92.41646	75.06402	65.08773	68.42824	72.76552	86.69208
Irf1	68.04048	63.65927	66.88544	88.84782	90.06293	74.31446	91.43644	88.12039	86.62458
Rab2a	78.29245	83.1373	90.26915	88.52028	84.39563	77.96032	89.77709	75.10661	86.58963
Gm9843	74.83043	73.75705	69.14127	71.90547	65.94608	94.22662	66.35698	82.3031	86.56562
Mbd3	87.36781	72.83903	88.77572	87.80493	81.16036	91.89687	99.89187	84.88868	86.54814
Trappc2l	85.33826	75.14066	77.16785	88.6559	86.12231	88.75141	91.30108	87.74004	86.53207
Nans	85.71936	87.34965	89.83524	81.27256	76.20303	83.71339	91.78514	84.49631	86.49904
Idh1	87.40743	92.57428	100.1333	92.91046	87.72548	93.29169	113.4566	82.63733	86.48242
Rnf213	82.10562	50.82077	82.58437	97.11464	67.53565	44.30665	84.49021	60.60326	86.44765
Tnfsf12	109.0762	99.74479	115.5367	97.79115	98.3974	95.02927	108.2371	105.783	86.36762
Snrpd1	66.30048	66.91666	73.85977	69.6833	69.89349	87.16068	62.70606	85.00689	86.3579
Sik1	75.35397	55.96776	49.59312	56.47812	51.70407	55.45815	85.33286	66.52208	86.34465
Bud31	83.00521	82.40552	72.76142	82.54025	81.51237	88.84006	78.80438	80.90902	86.27696
Hspd1	97.91997	89.19501	84.64084	77.46694	68.73781	75.58202	82.38431	81.86715	86.25728
Psmb5	81.78865	76.3656	73.52285	74.81141	68.80186	80.28555	65.1921	84.59739	86.08068
Edem2	90.75468	93.69624	92.63882	91.14325	91.48895	97.7866	97.24167	87.2331	86.03436
Abi3	81.32792	70.27082	99.73679	111.8991	117.0213	104.389	108.6335	92.70424	86.0284
Golga7	94.62283	94.15803	99.36095	85.90554	88.6366	79.15707	92.50255	87.76755	85.9407
Zmiz1	125.1888	99.07323	112.9542	106.0699	89.8417	73.74515	144.7683	91.8478	85.88894
Atxn7l1	84.21013	78.00771	87.59001	85.57493	87.39079	75.02836	99.09106	93.34877	85.78743
Psmd13	71.93115	66.46884	67.8601	70.89336	72.39118	68.45491	65.76623	71.82873	85.72978
Rbbp6	101.3843	94.30043	95.53928	90.47999	80.73167	72.86197	109.1234	82.5423	85.67473
Rasgef1b	89.98266	70.78954	55.48156	84.67131	56.29146	58.58151	135.2141	84.10277	85.6732
H2-T24	59.87659	44.52749	75.11556	81.37873	72.8571	60.28143	60.23636	70.73569	85.65686
Tmem165	90.81784	99.35371	85.76938	83.06927	74.63094	82.41719	93.86477	83.40521	85.63783
Dlst	89.11764	84.68414	87.94577	73.07834	75.81093	70.55519	86.55788	79.2977	85.62447
Cd72	76.77749	95.81613	121.4266	69.00887	71.93651	74.45488	59.64139	78.68694	85.59065
Tifab	92.26224	107.9887	136.8352	101.4417	132.919	110.3727	112.1266	111.8669	85.5492
Commd1	64.18074	62.99599	60.93473	70.02139	70.35511	81.66431	69.346	71.88224	85.52166
Magoh	64.4107	68.47389	53.12096	69.86537	72.55993	73.86356	49.01795	86.03848	85.51718
Fgd2	77.50224	86.94332	92.82644	113.6261	132.5671	114.1557	138.4878	95.21434	85.50913
Slc25a28	87.41804	72.8372	77.25573	70.80014	76.14344	69.52874	92.29241	69.31516	85.50052
Mdh1	90.60358	84.96886	83.76141	93.30573	86.55951	92.03517	95.51618	80.11721	85.40877
Pin1	75.10963	92.64039	84.48186	76.67642	74.73958	89.88817	80.56513	78.22222	85.32187
Spg21	76.75561	75.95208	78.21488	81.72305	77.26433	81.05841	86.16113	92.3496	85.15983
Cops6	90.55559	80.71196	89.14706	81.38113	84.89282	89.08528	92.8081	82.88874	85.14839
Supt4a	76.6647	86.46406	94.79222	85.51338	95.37812	91.38969	80.04041	88.46431	85.1475
Mrfap1	96.18334	99.1158	86.97644	89.4554	89.40132	99.89277	94.24291	87.36632	85.12332
Ubc	82.81176	65.18673	71.05554	97.27444	81.08357	73.36686	123.6856	82.35205	85.0489
Pbdc1	84.00376	74.52832	80.84737	96.19863	66.19108	70.19479	77.31046	64.76893	84.83455
Irf2bp2	68.82832	64.17178	66.52249	79.69617	71.25733	72.43032	111.6973	92.63947	84.81027
ATP8	81.52656	154.217	48.10626	77.82709	126.0437	88.71268	42.41176	110.7397	84.69524
Dazap1	95.24819	93.45238	96.60835	89.47999	79.80893	88.69105	95.8934	82.35318	84.66527
Klc1	88.87918	89.50288	88.4869	85.57919	80.15639	77.27335	91.02894	78.79043	84.60013

Lgals8	82.32179	78.00297	88.77277	84.24325	75.70173	71.61578	88.6506	78.52491	84.57755
Iqsec1	111.9908	106.8794	104.5311	89.34167	71.40527	66.9557	112.2666	75.04534	84.54451
Mia2	97.09444	93.3997	104.6616	77.41795	91.34495	80.28883	93.57315	96.45497	84.25102
Pdcd6	71.1864	72.5137	74.67835	66.64088	69.05871	70.18641	66.37441	73.38165	84.21253
Ndufv2	78.72925	84.94627	78.19836	80.79404	82.11211	85.62319	77.3151	82.46404	84.19674
Mrpl18	79.32456	79.20227	81.82967	74.0056	75.46296	83.82465	80.60411	80.19202	84.18306
Ap1m1	94.7366	89.44593	91.60975	79.42594	85.6173	78.68376	91.75925	81.33816	84.033
Rdx	94.65256	95.27437	99.52818	77.41887	70.35018	69.14811	91.33333	77.31841	83.90636
Slc7a7	100.2706	79.40994	93.33159	98.55044	95.42009	81.47056	100.7282	89.95969	83.88894
Casp8	84.79224	81.82677	87.26165	84.1872	78.93628	72.63081	87.31253	77.44252	83.88155
Col1a1	85.26056	84.93101	102.1282	117.9432	77.77332	101.3676	73.01872	87.55837	83.84484
Gak	99.80172	92.50743	104.6166	98.07743	87.42657	79.38126	114.2037	82.93715	83.84142
Tmem219	76.58818	79.43966	89.0883	73.58148	79.95538	76.87384	83.17649	84.13784	83.83234
Dhx15	103.9487	94.37416	88.37217	77.86374	76.522	69.53214	89.89827	86.91829	83.77774
Plxnd1	116.9762	103.4325	117.4313	95.21809	99.04611	80.16328	117.2257	86.38382	83.76567
Hif1a	117.3265	103.2557	116.283	60.90844	62.01352	53.10955	72.45235	87.06333	83.65383
Gadd45g	63.07939	56.21113	43.54522	90.57029	84.39962	84.22838	98.53561	83.39728	83.58182
Plcg2	98.41091	80.15452	95.86476	78.76821	83.13883	76.54456	89.53311	89.90394	83.49387
Usp12	111.2192	106.2789	117.0659	99.19055	105.924	89.75192	124.1203	92.68434	83.48402
Mknk1	80.90156	84.02051	83.10951	85.95886	97.35356	84.25978	105.8865	92.11315	83.45734
Vav1	85.61356	71.83257	83.63895	74.92771	79.04802	78.502	86.19453	82.3073	83.4364
Pa2g4	105.542	101.7145	91.71597	78.61113	83.47477	85.35429	91.38938	90.41602	83.40575
Eif4e	99.40009	90.3606	83.99078	76.66354	67.65171	71.2682	84.63925	88.78325	83.32267
Gpr137b-ps	106.5595	107.0654	104.121	90.2172	72.95938	66.18914	97.84107	75.6471	83.23552
Birc3	75.74599	75.93053	69.3648	59.19203	70.1113	59.62295	94.29602	75.31282	83.16201
Casp1	72.18307	68.99432	79.30067	79.32097	75.79548	80.20482	67.18219	84.21473	83.08009
Ppt2	87.03215	99.82545	105.8426	82.74048	94.63545	87.2857	98.09855	90.9642	83.0751
Pygl	53.96417	54.58489	64.7606	60.03253	65.91871	68.25959	62.74142	65.61791	82.91782
H2bc4	81.91594	136.6347	111.8348	91.33068	100.4187	84.71689	75.37925	94.42322	82.80488
Tor1aip2	78.98202	70.10042	81.14355	77.49098	72.4956	64.16384	85.56888	72.10462	82.78406
Ctdsp1	88.68892	88.97065	98.0465	86.04739	96.42973	91.15843	93.72847	86.60812	82.77732
Cx3cr1	118.4688	123.8842	156.0956	98.59205	123.9846	92.27583	141.8023	99.2783	82.71242
Rps15a	63.6739	69.37146	57.90583	68.90941	73.73917	78.6579	57.3761	78.47487	82.53786
Slc35b1	89.79107	79.73291	84.01195	70.92627	75.29435	79.24562	77.95498	81.54731	82.51816
4930430E12Rik	60.50655	43.8986	47.14966	83.36652	70.07479	76.81137	100.4344	85.47712	82.49198
S100a13	63.73439	69.05509	68.85975	64.84231	81.59784	82.31463	64.4302	80.83469	82.47068
Map11	75.77231	77.23554	99.08589	81.29638	97.22804	82.16504	87.27701	79.16267	82.44932
Zfp467	98.38174	123.9651	132.5485	111.1754	122.9617	107.3277	137.3622	109.7946	82.38753
Fcf1	73.32381	74.75025	65.89843	85.61705	72.56121	80.5528	71.78256	78.73139	82.3555
Dhx58	56.82941	39.14116	60.02689	85.47033	64.42755	62.00447	60.34762	65.23009	82.33123
Tcp1	104.581	93.14371	88.44008	71.27968	77.30521	78.31396	85.15153	88.06557	82.26693
Ppp1r9b	85.98661	75.81051	94.60139	92.88924	89.48937	83.85118	100.4369	80.8803	82.25795
Gapvd1	88.48291	86.39445	86.56082	85.86888	78.72607	69.34154	103.4846	79.43313	82.23086
Akna	79.30655	74.11034	88.20643	79.88229	90.75778	80.67273	102.4296	82.60728	82.19668
Pepd	97.77258	87.20815	93.64361	92.92926	100.5231	85.78964	99.37855	79.65354	82.17245
Stk24	97.17814	92.55891	93.82916	75.19531	76.79797	74.57074	89.15249	82.65973	82.15045
Smarca4	112.9034	96.22789	106.2081	93.25636	79.69848	71.25356	110.2114	82.30729	82.09477
Oxct1	101.4189	93.5746	98.81924	92.23807	87.7955	82.06904	102.6	81.69004	82.09372
Klra2	73.98092	49.71709	67.76551	58.34803	47.47641	38.11287	35.17953	74.42751	82.07046
Rassf4	109.0148	121.2922	136.3652	83.31695	99.813	81.46698	111.6155	95.07334	82.05455
Mpdu1	83.92227	79.5139	83.98139	86.00483	89.23575	92.90216	86.58479	85.07237	82.00854
Lamtor4	77.62593	79.37366	81.42819	74.11101	84.70654	89.61562	78.66226	83.47695	81.93713
Pdlim5	102.2617	97.27797	109.9441	79.3339	73.99744	62.42029	89.23554	83.12908	81.89135
Pde4b	83.52023	88.12673	56.09015	44.89643	66.55545	55.07326	97.74856	74.90962	81.83259

Ndufb1	73.58743	54.79681	59.73485	86.30083	88.62623	85.7508	46.89569	88.44205	81.77216
Tmem160	61.0839	68.11243	68.71037	78.19315	81.1738	81.52458	74.66317	79.59467	81.7521
Fam111a	94.06692	100.4972	113.188	80.93228	77.25666	69.78476	78.06784	78.49832	81.72937
Calu	103.6142	99.94397	97.29182	75.9949	70.02835	68.67069	87.77166	77.4062	81.61522
Coa3	82.07613	73.77197	69.96201	81.5396	83.46297	81.25666	67.6723	77.4829	81.61097
Nap1l1	94.3133	93.96161	86.1125	74.54654	73.47229	74.49442	72.70675	87.79791	81.56341
Nfxl1	68.06601	60.68804	68.2821	88.52142	71.96205	58.90295	96.30941	65.79642	81.53756
2610507B11Rik	108.6301	97.30788	107.7609	88.40025	77.4886	66.96208	115.3228	80.4428	81.52685
Gorasp2	109.9792	98.56548	104.1009	85.0415	82.78784	81.03699	101.2423	84.83968	81.46247
Rad23b	101.1006	89.32654	93.92541	79.6174	73.44146	75.40113	91.26184	82.83704	81.32883
Fbrs	92.08185	92.03772	95.75862	83.2988	89.30534	75.83519	106.8886	85.48817	81.26706
Ldb1	83.66818	86.62374	87.34614	84.99325	97.6946	90.77441	101.2037	83.8394	81.17589
Lfng	88.03584	75.81863	88.7015	81.19483	91.91211	89.76282	84.51707	93.68657	81.15306
Apobec3	89.02594	85.48711	102.6717	79.54884	102.2722	92.04193	85.26267	83.93043	81.14929
Tfeb	76.38834	68.8968	70.24548	86.41612	85.04356	87.56051	94.13826	86.71942	81.14762
Gmip	77.15709	86.89209	84.05943	80.78467	78.19042	75.15854	98.6742	80.59005	81.13077
Ceacam1	102.5566	82.93571	98.59743	60.54251	60.54192	65.04593	63.43428	67.86832	81.13028
Anapc10	68.77283	69.20545	69.27476	73.78985	73.96508	76.95817	62.27489	76.5287	81.08011
Susd3	57.96665	65.71096	82.11704	82.45408	88.92157	87.18103	76.08504	98.56967	80.93853
Chp1	89.84865	91.88136	88.15041	64.49332	71.71932	63.74495	73.41661	77.3698	80.89487
Mfsd12	88.98164	94.67645	86.19877	98.54171	82.87079	80.03921	96.51371	80.14503	80.87667
Cdipt	93.49487	90.39851	93.4116	72.50502	83.15705	80.22316	83.72578	78.11327	80.82597
Prmt1	79.54157	74.95902	66.18277	69.3452	64.5896	69.75809	60.58188	74.3753	80.82242
Stat3	107.4283	88.29158	101.487	67.54663	82.18628	76.99283	83.80634	80.6143	80.80458
Maf1	73.85046	80.89904	81.02398	80.06141	84.7137	82.13307	86.32761	79.65181	80.7238
Srpr	94.07843	82.06885	90.93353	71.39898	66.16921	64.44244	80.32266	69.8909	80.64276
Sae1	93.845	94.92398	90.55357	78.68777	81.70818	77.62428	91.54509	84.49607	80.60878
Zfp710	85.06983	86.37194	99.85993	87.40606	85.92086	74.24494	93.87394	80.30671	80.53279
Fgl2	63.43974	50.15604	68.63997	69.84185	68.72644	58.92553	55.64094	68.2573	80.45472
Sri	78.80108	78.0612	84.98101	73.62592	83.72067	79.60442	73.94524	77.51256	80.43442
Ncln	93.95009	82.04269	92.59367	76.57345	79.66556	75.46758	91.66885	74.47113	80.36644
Slc23a2	104.8517	81.65302	84.24992	89.52333	72.00982	63.22351	102.108	80.75267	80.35284
Lmo2	69.78116	66.71984	75.81188	85.90313	88.80871	82.65558	89.77568	89.89351	80.31702
Syf2	69.55826	69.67639	69.56634	71.02637	78.49448	80.23587	76.87632	84.30303	80.29131
Rpl22l1	59.43052	74.06193	57.84026	50.70371	79.41518	63.71502	52.93581	74.06923	80.23457
BC005624	82.44742	74.35902	76.5702	79.5364	88.50127	89.10024	67.3079	84.81761	80.21099
B4galt5	92.4191	82.60843	95.618	72.58879	72.44341	65.14017	84.76339	79.95798	80.20715
Babam2	93.56083	76.67139	94.34783	69.83784	79.03623	74.94996	85.22013	83.9994	80.20375
Tlr7	85.68498	84.21814	100.9078	79.94508	82.02292	62.63161	102.046	85.32285	80.18918
Tyk2	81.74628	81.77159	83.37993	81.91413	79.30424	71.67297	99.09135	76.41958	80.11705
Gpx4-ps2	66.99658	77.20741	70.14408	77.54747	80.93068	83.33424	74.2588	83.48733	79.90211
Cav2	96.6192	110.8287	91.76165	61.74678	54.5491	62.43044	55.80908	75.5319	79.87159
Rnpepl1	93.85615	87.16573	95.50905	78.38303	86.3993	80.85705	103.868	78.55713	79.78638
Ap2b1	107.2182	94.05296	98.55428	72.71279	75.14915	71.5412	86.91773	77.25584	79.77434
Hdac5	79.78789	78.25341	72.91592	77.9914	76.33479	64.25571	96.05122	76.29644	79.74492
Nhp2	82.6654	80.4838	82.79795	72.97369	66.67141	81.10705	80.86514	76.89153	79.72472
Cab39	91.54412	86.01255	90.06631	82.5063	70.38337	64.19892	93.30153	73.43199	79.69936
Safb2	79.92493	73.21346	72.25371	75.23467	76.095	75.1555	97.9999	80.94625	79.60112
Tmbim1	106.7705	113.2868	122.8472	73.10545	81.81611	72.44932	89.68168	83.52843	79.47349
Isyna1	82.52369	73.52008	78.55141	71.71377	84.19625	81.1966	77.56117	92.12522	79.45733
Nars	89.18681	93.20079	84.5363	81.70295	69.43753	72.67305	85.94701	82.68553	79.4245
Wsb2	100.1937	94.94439	101.0803	84.63723	71.85259	73.963	94.48936	77.90237	79.34387
Otub1	86.71082	74.87457	80.30692	74.98264	79.84333	78.1392	83.81157	82.33687	79.20884
Stk11	77.04426	81.61525	79.30706	73.50337	78.71499	79.37587	80.26961	79.09324	79.17555

Tlr8	76.94545	86.20698	106.445	85.83527	81.06971	65.11456	108.1692	74.13578	79.16213
Tle3	94.85049	74.45906	87.07725	71.90309	89.04082	73.62472	85.37782	76.09764	79.13363
Naglu	92.18161	86.4641	89.26243	90.94931	89.28237	96.49656	101.8688	82.18748	79.05497
Camk1	76.97154	78.03899	83.70806	77.84745	86.34081	85.98191	89.62303	84.21638	78.92448
Pnp	63.03464	54.95207	66.09603	65.75565	67.59155	66.57264	60.27883	73.99596	78.8339
Ddb1	107.5483	100.6634	101.7363	83.57112	76.02848	73.22516	90.9425	78.53247	78.81558
Prdx4	72.74784	82.1775	80.30096	72.22404	69.67411	87.71698	68.23781	75.72169	78.81021
Etf1	78.98989	67.69165	67.91017	59.05564	58.32785	54.44998	70.81446	72.31412	78.80687
Mbp	91.60546	90.21957	90.6385	74.87345	78.99842	72.54916	85.21094	83.28196	78.79028
Irf2	75.16708	69.26499	80.37813	79.6157	78.26065	67.06363	81.90273	81.76152	78.78713
Tnnt3	92.39677	106.6356	76.92148	134.5314	100.4304	171.4139	58.40775	78.0169	78.74442
U2af1	84.71924	73.26169	80.51443	70.44474	73.69661	75.65803	82.60525	69.9385	78.66748
Armc7	74.12809	69.60836	88.40419	65.81185	83.1535	69.38631	85.20045	66.36493	78.65372
Soat1	86.54069	84.2327	83.47987	83.99644	74.36988	66.17769	97.55844	79.27688	78.59047
Nop10	80.97863	85.85756	77.00626	68.34917	79.19791	71.76044	69.33214	67.15818	78.54014
Hspa9	90.69033	87.12383	87.36655	71.39585	64.0593	66.27864	81.45618	76.16425	78.53551
Nsfl1c	85.03	79.68552	86.01391	83.92048	80.01843	89.12904	81.72709	85.41487	78.50638
Mapre2	91.07048	81.49001	86.59876	84.23568	75.29577	69.84292	104.8884	79.72293	78.49483
Smarcd2	81.17302	77.18448	83.22478	73.74951	79.71727	73.43532	76.36536	72.75541	78.4046
Cyb5r4	77.86373	82.25451	84.21986	69.67349	78.08664	72.58493	77.50526	77.03314	78.36101
Sumo2	80.76848	73.9607	75.94453	67.99798	72.47905	82.1922	62.75222	81.55367	78.35606
Rbm25	68.89915	68.26909	60.51605	66.46999	66.88576	69.2944	71.65994	76.35839	78.24555
Lsm12	99.23594	78.14748	75.55673	77.06143	67.03352	74.3042	84.44902	93.18866	78.22007
Ufc1	72.53949	68.22358	71.24534	76.35641	80.41136	76.03702	75.38287	71.81435	78.20367
Tmem87a	72.59189	78.77531	76.96727	70.40196	71.88333	72.89919	82.76378	75.05355	78.17854
Chst12	74.15539	75.93926	86.98517	77.78352	83.01147	80.74757	94.48075	78.70085	78.1319
Map1lc3b	77.34231	83.89287	88.03368	78.07674	92.32766	85.28554	85.46855	82.47395	78.10465
Gm12481	62.95699	64.35632	72.50435	71.97825	79.94606	84.26947	72.37643	77.14361	78.07956
Paip2	62.01546	73.02091	76.53221	71.0491	83.94083	73.686	64.13492	83.27105	78.05606
Cbl	100.1441	93.56849	95.83621	83.48843	71.85928	59.31605	115.2498	77.14361	78.05548
Rbck1	75.59971	73.23019	79.69824	75.20197	71.80314	73.09551	79.82131	77.67056	78.0474
Zcrb1	61.78109	69.0182	60.77327	67.11177	64.39738	72.97097	61.29706	76.45607	78.047
Foxp1	97.43817	92.62512	97.34571	78.29295	80.02559	65.32573	101.1094	80.19873	77.95803
Sash3	72.66797	78.81526	86.36668	80.08627	85.49942	78.27038	83.58189	75.12186	77.90408
Pmf1	95.69666	96.8406	103.2159	82.28111	94.83321	86.66629	90.46605	83.72933	77.84081
Zfp703	83.7078	65.80455	69.32749	75.1897	77.37468	67.54813	121.4082	84.94132	77.83144
2510039O18Rik	76.79319	76.12309	83.74536	72.83051	76.11428	74.67655	80.67039	76.03304	77.77603
Wdr83os	82.72089	73.00873	81.46138	72.9513	72.21097	69.01661	75.57763	79.58902	77.7065
Pttg1ip	88.8325	90.35386	90.37055	79.43131	77.84381	80.38917	85.57053	80.39314	77.65463
Cops7a	88.35875	82.0826	92.0684	90.66846	86.88211	84.23415	99.82947	80.12018	77.64762
Tceal9	70.68625	71.58432	67.9168	83.55773	77.38263	76.74999	75.08403	66.47065	77.5952
Uhmk1	99.60747	105.4753	116.3835	92.00337	77.53309	63.56463	110.0148	82.44289	77.59005
Dctn3	84.69182	82.09229	83.51133	77.86374	68.78733	82.83882	80.67041	79.0246	77.55455
Ube2n	85.9619	74.85623	71.3037	68.80813	70.31855	66.9461	68.21345	82.76912	77.50879
Zmynd8	87.09365	89.44525	95.87534	100.7426	92.95119	77.67443	124.5462	83.89267	77.50652
Hmg20b	72.31553	79.9833	79.82163	85.67322	80.36958	77.22105	84.11919	71.66995	77.49729
Msra	59.24106	59.77781	59.94435	62.79744	68.86774	67.06864	71.08274	59.76913	77.49485
Gm8995	69.40071	58.24711	71.28979	75.57525	60.13674	47.87284	63.83788	63.27373	77.48019
Yif1b	94.36266	84.60226	79.77521	78.24394	81.117	91.08142	91.2806	84.36772	77.47611
Pdap1	77.45749	74.73925	74.5839	85.28628	76.56416	79.91469	72.78323	85.9353	77.43711
Lta4h	77.97113	77.65202	75.24012	68.24915	67.60245	78.80131	71.36968	66.99621	77.35097
Lman1	98.62374	87.03012	92.30142	83.73561	68.31691	67.80715	86.01279	71.59872	77.34522
Flot2	91.83436	79.17683	77.33742	66.02229	75.12845	75.15839	73.1965	73.54079	77.31385
Psmd1	89.84848	73.18855	78.04121	72.41414	66.38743	66.88693	74.31495	66.95074	77.29255

Gpr35	109.3631	88.16589	106.0115	64.27162	76.46984	61.26548	78.06739	82.02681	77.26805
Asph	75.23962	59.4349	69.45518	89.87571	62.75269	57.43927	97.12353	69.6273	77.26082
Sparc	80.92948	83.41777	82.89353	143.0014	92.60771	127.9741	57.1894	88.25757	77.25316
Gatad2a	84.37816	64.98329	74.13647	76.93404	65.32977	59.97838	82.91641	71.52602	77.23729
Ehmt2	77.77681	76.21552	84.19621	87.53328	83.62676	81.71457	98.5248	81.0295	77.20355
Elof1	77.04533	67.54304	72.17665	68.69454	70.49613	75.70217	68.20887	66.24259	77.19673
Hipk1	92.83452	92.13519	91.82899	68.12431	65.291	59.04568	96.82167	71.52278	77.14022
Trim25	69.2673	56.46836	69.87098	78.72636	61.29714	50.35256	99.15518	64.27698	77.13952
Adam17	91.49853	88.39183	93.4458	79.31146	69.92526	63.9277	89.73982	77.84576	77.11369
Emc10	94.85229	93.53226	96.02825	82.4565	90.42623	85.76588	92.76355	86.86178	76.84076
Cd82	63.14822	67.30126	68.88767	49.02109	63.29334	64.10789	55.85233	64.76815	76.76868
Psme3	96.39231	87.21117	86.74651	69.07495	70.51705	68.83763	77.94593	76.79611	76.75703
Gm10222	73.73076	110.7671	55.07114	135.8699	100.4418	97.35625	45.33681	68.244	76.67649
Csnk1g2	80.85844	80.34895	83.79374	74.58717	79.20961	78.02646	81.97045	67.83308	76.66695
Wdr33	88.22765	71.76202	85.09862	82.88337	75.61054	77.79424	88.79194	70.02413	76.49456
Sigmar1	102.3894	101.4852	95.72939	69.31877	78.13228	77.77961	80.47031	78.9526	76.25809
Fkbp15	84.55015	78.48412	93.33159	87.70931	79.33329	71.61659	101.5203	78.40227	76.11295
Xiap	88.05633	81.47114	92.4335	77.09583	73.34377	58.09342	89.45258	71.78713	76.09774
Mta2	81.25499	71.53504	75.92684	66.12732	72.84871	70.15537	75.88836	71.38469	76.08797
Eif3m	70.1314	80.14871	74.24155	65.25665	73.57464	80.06882	68.57127	84.53778	76.07726
Gm5559	81.81305	75.38176	79.35554	77.04646	83.7007	94.11206	74.29456	81.05557	76.07621
Slc2a1	87.40483	66.5267	65.75018	45.76882	57.50926	50.94974	63.78279	75.17305	75.99332
Tmem135	84.1369	86.81297	84.526	84.85993	80.97391	62.54972	103.2932	78.84268	75.97259
Sdha	92.82337	86.38774	93.28019	80.69559	75.55057	71.06423	95.61692	76.54289	75.95723
Cyth2	79.15616	76.36489	72.12099	64.79986	77.89734	72.22631	73.83866	74.99946	75.85386
Irak4	84.06295	78.50886	97.97914	78.86299	79.71554	79.87492	87.67905	83.57224	75.84584
Maea	74.53397	74.23161	77.72132	63.85778	73.46648	70.3481	79.53868	74.03932	75.77995
Abcg1	75.06662	84.81165	97.20172	94.7235	95.40212	88.73371	106.052	85.07217	75.72967
Mef2a	92.18239	86.38869	94.8517	76.63707	68.0808	56.01547	101.4237	85.46025	75.67027
Asnsd1	72.10787	75.96287	76.39386	63.63385	64.44459	67.49445	64.55235	70.42883	75.57159
Ifit1	34.94826	20.45622	36.1293	57.71881	40.64972	28.80146	27.0417	37.05577	75.5707
Plekhm2	78.05354	67.31995	73.11678	88.11109	69.71369	68.5703	98.0159	76.55396	75.5559
Psmd11	89.85228	79.52565	77.23423	72.83918	73.54849	63.12095	87.21263	78.52263	75.51231
Rraga	65.13027	55.0848	57.98815	65.85911	64.45161	67.41359	81.73758	79.05917	75.43988
Map2k3	93.84034	86.77352	69.50784	57.0732	73.91805	63.11317	78.32974	67.93414	75.41451
Gde1	75.34355	72.10543	77.60055	69.33305	55.76831	67.99886	90.68749	64.10533	75.40301
Nfu1	73.43214	71.61045	74.489	68.73568	72.26532	76.25485	79.14588	78.39749	75.37099
Atp6v1h	75.71821	71.36984	64.1855	66.23741	64.3281	62.88102	67.44209	70.17914	75.34721
Hsd17b10	69.37365	75.07608	71.45099	59.54499	72.33003	71.47045	65.28576	75.20488	75.34114
Fcgrt	88.484	102.6278	115.6104	93.95151	136.2799	115.6023	122.838	96.36293	75.33433
Nudc	86.26442	71.99665	73.7202	72.72577	67.41915	78.70895	75.92943	75.31396	75.19621
Dok1	67.73332	71.4805	69.22905	74.27205	74.30143	68.50488	76.38227	71.44588	75.14838
Gpr65	58.76899	52.20018	71.68791	74.4106	70.70144	71.58809	91.59141	76.01462	75.13394
Odc1	75.00572	58.54668	45.16975	44.19725	47.4918	42.1089	81.70646	64.51191	75.05524
Smarcc2	98.50425	90.15248	105.8552	87.00619	80.38769	77.42011	98.50317	83.87965	75.05142
Zfand3	76.32716	74.38434	84.15459	58.6174	71.27211	68.25286	70.39531	74.99192	74.98985
Szrd1	74.13388	71.46515	83.85042	75.14609	76.34965	69.39429	80.96611	77.25375	74.92356
Brms1	71.79684	68.10957	70.55	76.47276	78.35296	83.16442	80.69134	82.98508	74.90454
Ndufa5	48.74509	53.52722	58.60194	67.4339	65.42697	77.89373	57.42852	69.37468	74.86301
Emd	86.976	73.80567	67.7249	59.99706	64.49296	61.8453	66.85397	73.45009	74.78042
Elmo2	81.36696	74.37713	86.14532	80.59188	76.54498	67.92594	96.29317	72.97524	74.765
Atg5	71.18822	78.29181	74.63443	83.96537	74.70377	76.81438	78.55059	77.8348	74.75866
Tmem43	101.3719	100.0912	93.35103	65.70045	65.25609	59.40226	74.33522	75.3229	74.74514
Marchf5	72.28854	66.29815	70.90661	72.97276	65.51681	65.5646	71.49189	71.83389	74.679

Dctn1	87.9734	78.56218	83.41602	74.99487	69.67106	68.94622	90.56287	70.22698	74.67857
B4galt1	95.69453	93.02171	97.98397	70.90367	78.21384	65.92381	107.583	79.80114	74.62682
Vdac3	71.05442	64.47959	72.15595	60.71792	63.24678	67.83903	56.93485	76.23721	74.60492
Kcnab2	105.6326	92.24587	96.34854	79.72872	72.6378	72.16226	94.08434	80.52768	74.56903
Slfn8	64.7167	45.48058	70.67691	69.43243	62.1835	51.48356	58.69189	62.30146	74.50022
Nfkb2	86.9518	80.13678	84.81723	55.60964	66.20693	57.19943	67.3584	68.54797	74.46643
Plgrkt	50.90043	56.27429	61.23231	69.38352	66.32707	76.93148	64.3668	76.51267	74.46124
Tmem109	116.4199	109.4064	111.3774	78.26395	84.06741	83.38255	93.16121	87.81581	74.43777
Snapc5	81.88224	69.09493	75.84459	80.4094	69.2418	79.49476	67.91432	72.20309	74.43061
Mcrip1	71.67843	77.45254	75.87385	71.96315	82.33077	81.262	77.7446	86.7297	74.41084
Plod1	98.72135	95.21114	107.1382	83.99658	88.35486	79.3604	100.712	74.93747	74.30056
Dctn6	68.81587	73.08006	76.14672	76.25883	72.72261	75.09374	81.59418	75.16391	74.29767
Cfb	36.03474	28.64656	64.93992	70.94956	65.35386	58.04921	53.35031	97.77225	74.22198
Mapk14	89.29243	95.30539	100.1135	79.37383	85.82917	74.06012	104.5702	79.01447	74.13008
Mx1	42.09757	24.99039	42.98645	86.72825	51.9568	38.55034	48.79997	42.15552	73.99587
Sting1	87.91494	86.78504	109.6819	75.50377	121.8244	102.7165	88.05035	97.73443	73.97236
Sirt7	62.07908	67.80122	67.47673	69.67507	79.59512	79.7196	72.98201	76.79116	73.90509
Gm6863	61.23551	70.17915	70.08475	68.6752	69.01778	74.32095	70.92969	74.17144	73.83422
Smarcb1	79.65395	67.94283	78.5067	68.21608	69.51276	67.65408	77.40503	68.26101	73.74545
Mob3a	82.1367	76.987	81.43083	57.88913	72.65569	73.21411	79.06472	71.03098	73.73173
Mgat1	74.85904	74.61572	81.092	73.40827	70.05484	68.47216	79.87962	70.01242	73.68209
Tmem120a	78.0384	69.22125	58.04753	66.77419	59.69795	68.99836	72.90028	72.11126	73.67468
Washc2	75.54161	73.60492	75.21888	73.96482	68.17255	64.47889	98.27165	70.91313	73.67224
Retreg2	79.05805	85.86257	86.2919	69.68327	82.3097	68.56161	89.47134	79.18502	73.58282
Snx8	76.28173	71.97743	70.93763	79.25123	73.85174	71.62947	90.37703	72.39982	73.53274
Ube2b	62.62636	77.54193	77.45275	60.08394	68.3637	69.10906	65.39332	76.15526	73.45529
Tra2b	76.16728	73.38595	66.3595	61.77104	67.52904	62.90464	79.69223	76.41804	73.42283
Elp5	74.24637	63.97484	69.95877	63.95947	64.6852	70.21753	62.87781	75.00085	73.41909
Ldah	87.4048	80.383	81.69623	68.07004	78.62739	70.70669	75.30917	68.85572	73.39104
Gramd1a	68.17191	76.32158	72.33664	66.9999	77.67468	69.50584	96.9286	72.41344	73.3432
Akt2	72.61736	70.51619	76.65644	64.6017	72.59145	67.97866	68.96875	70.14675	73.31742
Sirpb1c	57.00809	50.70093	65.24476	53.67397	74.71209	52.16857	50.58339	87.78178	73.27061
Ube2i	86.94698	67.99234	77.78979	73.53258	72.2938	72.82577	82.39435	84.69111	73.22894
Srsf7	65.02977	62.42792	57.92969	61.89801	61.41299	58.64382	63.17933	68.11328	73.18134
Dpm2	78.36019	73.33533	85.58159	71.90268	80.88427	74.90635	74.33435	80.12391	73.08867
Trim28	85.94439	79.79769	79.91739	68.30576	79.29562	74.27603	88.13854	73.39401	73.01405
Naa60	90.61852	86.75835	96.63735	71.99793	81.70395	82.66782	83.32675	82.11369	72.9234
Lamtor3	78.3946	69.47425	70.72138	73.8962	65.59067	67.62586	71.86068	70.54379	72.91812
Cd63-ps	69.42473	82.49186	81.83151	64.86335	72.47698	67.90577	63.67175	68.52036	72.8938
Actn1	102.006	102.1511	95.21991	65.00254	59.07322	59.61877	71.6142	55.23035	72.85435
2410006H16Rik	42.81117	69.56285	64.22422	65.88995	79.71329	76.62305	53.69357	87.93262	72.78036
Ubl7	78.42458	76.06654	82.06801	76.47263	86.8038	78.26873	80.79368	82.67376	72.77224
Syvn1	84.38091	81.19841	82.03214	74.06595	77.47085	68.78057	98.43882	66.18646	72.74295
Ube2d2a	83.21291	75.04184	83.44588	69.69203	76.48674	65.34888	79.03571	77.44121	72.73388
Dusp6	74.25026	69.19276	82.00834	82.81044	66.64492	64.89683	87.28864	71.22591	72.72756
Septin2	81.43177	84.55162	84.94484	73.62529	66.79482	63.78861	81.24911	74.09558	72.72029
Sec11a	66.74484	65.3749	74.17508	63.20101	64.75301	69.04745	67.13082	70.47147	72.68109
Cct7	85.30498	73.28468	80.36288	72.01202	61.64861	68.09864	78.21224	68.58277	72.576
Prrc2c	113.06	97.56471	111.305	90.58659	73.74572	56.02772	109.6519	76.95092	72.57089
Leprot	74.28154	74.20484	80.45068	75.01206	77.8541	71.55546	87.65623	70.11661	72.53213
Noct	66.20537	44.65013	50.19576	54.80901	50.14694	43.8621	62.26383	65.40334	72.39898
Snf8	75.01146	61.95628	62.78538	63.61325	68.5984	68.65084	73.70411	70.05467	72.34572
Zc3hav1	70.53824	62.71463	67.52605	61.96165	59.48394	50.87488	73.05664	59.85433	72.33247
Stmn1	102.2466	107.6078	107.5937	70.3656	74.86444	101.2773	84.70375	65.8785	72.32742

E2f4	89.12371	75.15398	85.71225	81.93062	73.70081	73.77564	81.36144	76.98878	72.32334
Ubxn6	65.57718	65.85927	64.01469	66.70593	70.18009	69.41513	68.58859	66.11043	72.32022
Paf1	84.59059	77.28776	72.84721	70.82179	70.26297	66.83398	94.79255	73.24142	72.31662
Col1a2	57.25344	66.74667	77.50764	86.32282	57.11592	81.98658	48.19089	67.71156	72.31499
Camta2	85.83801	88.50861	92.83504	80.80269	80.48327	71.34965	103.5883	79.00509	72.07957
Fbxl5	59.12477	62.32453	53.08565	38.73272	45.53634	47.73097	47.05016	56.11816	72.06489
Hgs	86.86887	76.18856	93.57811	84.42766	79.58867	76.0275	98.95494	73.35037	72.04438
Rnf115	70.13187	63.52083	71.04555	70.33068	62.21021	65.86053	72.40457	65.7872	71.97031
LOC115487638	86.8307	80.60418	45.42441	80.36663	62.34052	58.06186	84.22116	47.695	71.93525
Nampt	59.35177	49.39298	58.32221	66.02694	59.88177	54.11996	71.49964	62.47531	71.83655
Selenot	67.40199	60.74514	73.12832	56.45627	58.259	52.8192	68.15379	70.59359	71.79252
Serbp1	77.37218	72.19823	71.79653	66.15802	64.27206	69.68555	70.73561	73.40286	71.75577
Xpo6	80.73	64.27629	75.18597	68.37951	66.85549	59.28464	91.39911	70.7575	71.75302
Ass1	50.33953	37.45921	45.89214	31.53524	40.4649	36.61956	24.06446	42.16772	71.7241
Txndc11	82.29371	75.41258	69.85099	73.87992	73.22561	66.18535	94.54171	70.21799	71.72027
Abhd17a	76.9902	73.72063	79.98692	83.22177	89.33898	79.24935	77.41185	79.68162	71.7108
Mboat7	81.85875	83.12428	83.83492	61.39487	75.08014	68.37983	68.05929	76.01732	71.67529
Fubp1	80.11623	74.53812	77.57839	66.52419	62.8983	62.2315	74.04491	73.64461	71.62868
Psmd7	76.08874	69.32043	69.5482	68.56486	67.9782	70.84512	69.47383	70.20068	71.59467
Plin3	69.84323	73.34503	70.41632	86.53413	71.92251	74.91829	83.43906	68.28228	71.58378
Sphk2	81.92386	72.71674	83.24745	72.59413	72.99599	66.25324	81.17019	74.53192	71.53371
Stap1	75.55221	74.60808	68.79341	58.76977	43.33861	48.97097	51.07415	53.37354	71.47545
Selenon	74.50747	66.60536	66.5495	54.9355	59.74135	54.22884	69.43953	53.02761	71.45702
Psmd14	79.90132	74.0555	67.35374	64.67915	65.8869	69.53201	75.6289	68.9152	71.45564
Rtcb	70.92935	69.51121	74.46766	66.88286	72.96826	74.0385	80.69204	68.7319	71.44663
Prr14	81.94028	78.73674	83.70222	73.8825	80.58856	70.7796	91.53092	79.03468	71.4395
Atp8b4	99.10227	88.57211	97.25218	66.96924	66.04097	53.73535	77.25496	73.13105	71.32621
Tmem104	95.61427	72.95775	88.12106	78.62252	71.67012	67.87371	114.9474	74.76938	71.23676
Pacsin2	89.57312	72.34836	82.81675	76.93582	72.33528	68.19608	96.88167	79.74613	71.20819
Tra2a	57.47667	61.54175	54.31857	58.48601	59.55057	48.84752	64.75115	64.57306	71.19419
Nop16	90.00828	88.38953	88.13288	66.75306	74.70823	73.70413	71.20024	76.95947	71.04893
Sla	78.89106	77.01173	89.59718	68.199	73.96859	76.22154	106.727	77.8522	71.00755
Mir7115	59.76916	69.11309	81.43412	68.04804	57.74847	69.6916	69.83194	67.62754	71.00025
Abcc1	102.7799	87.67428	94.86508	59.21654	60.06298	49.02123	80.81617	64.08141	70.99137
Chmp1a	65.32039	70.03629	70.94649	65.51313	75.73231	67.79763	69.14821	71.06548	70.82304
Trak1	88.54516	81.64551	85.74178	78.50739	70.43004	66.08777	93.22933	68.37566	70.79302
Pstpip2	72.94901	61.36329	70.41014	54.5339	63.94203	54.69012	72.31148	70.81869	70.66481
Emc2	60.73696	66.30416	59.40801	68.5441	67.68399	68.39769	57.8963	69.27434	70.66206
Abcg3	59.74314	56.78919	77.13488	84.36733	104.05	78.91046	111.4058	65.89108	70.64341
Nfic	90.24511	80.50819	95.53433	82.39872	75.92877	73.76798	106.9016	78.15506	70.61415
Micos13	58.30175	65.25008	53.90234	63.88952	65.80594	73.53984	51.30258	67.81324	70.61316
Patl1	82.75084	74.45825	73.60305	67.37118	62.7095	54.47521	88.07873	75.09749	70.38037
Tatdn2	72.33991	61.23137	55.5176	63.34033	52.0503	53.41338	79.24273	66.09117	70.3744
Casp4	61.36888	51.6813	61.47655	54.07795	62.56371	51.73218	47.73968	60.05621	70.37003
Unc119	70.14928	79.33204	75.93936	63.23753	78.7063	82.48607	65.94072	84.08827	70.35147
Ist1	71.79807	66.14304	73.36469	68.32215	63.30567	59.49434	82.41458	66.10968	70.32212
Pkn1	81.09797	68.47704	81.55762	75.60346	70.9645	66.02314	98.60427	72.70487	70.28566
Gm36161	61.77588	73.15245	74.38894	83.24008	78.80794	61.24427	73.56372	100.4121	70.28019
Xist	72.7981	44.42646	46.15699	101.2321	51.83314	8.633846	48.91145	29.99337	70.25002
Eif1b	67.52924	61.62144	70.19746	58.43397	69.7264	60.82761	66.10828	67.17246	70.18097
Itga4	99.5192	102.6481	112.0687	79.68631	94.68159	62.71591	120.1629	83.10431	70.03865
Map2k1	66.84299	64.71577	68.11556	63.08455	57.5507	55.66902	68.84791	61.38616	69.99695
Chchd1	50.87258	55.19755	44.44879	61.05721	61.05116	52.30135	53.63864	66.17768	69.97625
Ly6a	65.58624	45.8055	72.32144	86.03584	68.70446	78.50989	32.96433	76.52354	69.97086

Pigs	91.62119	86.47572	94.17255	70.11938	74.16678	67.40508	88.70762	75.16133	69.9445
Plekhb2	83.61346	83.56896	81.28157	66.59105	69.3336	53.94635	78.54379	60.47125	69.93296
Zfp384	76.32199	65.84376	80.99442	78.11384	68.81658	69.54821	94.26778	70.84462	69.83577
Lxn	73.25744	68.3536	73.92601	66.68815	69.43113	77.27666	77.28934	72.60006	69.82085
Tmbim4	80.2068	66.92337	74.17761	69.6599	72.59808	66.17807	67.43862	74.27434	69.77414
Cat	85.62366	87.21633	91.48017	77.92625	77.30411	69.99777	99.21023	76.01834	69.75945
C1qbp	70.23741	59.08724	69.15096	70.45913	61.68146	60.99279	68.90515	64.19979	69.75444
Dusp5	56.8431	40.39403	32.31972	32.37793	39.5335	31.63607	57.17173	51.21208	69.75119
Ntan1	55.5625	57.8347	55.63119	58.71607	62.33909	72.67195	58.32693	71.32446	69.73825
Lrpap1	80.98695	81.67868	83.59326	73.16412	74.56787	74.82409	82.09776	75.30299	69.70924
Romo1	62.42915	61.0397	73.9669	73.78795	68.80172	89.83135	68.45337	68.31359	69.67055
Gm6344	68.18347	61.33509	54.64225	70.72631	63.91857	74.26353	54.53444	69.83918	69.65702
Qars	75.72028	76.82303	81.11613	65.88412	73.84627	70.92569	74.20561	74.288	69.63297
Rpl10a-ps1	72.31052	59.55551	68.58633	69.97252	63.7582	70.46569	66.21817	67.95835	69.5853
Sgms1	82.04542	75.02928	74.5457	54.29391	56.06163	53.18865	90.41105	66.00411	69.5814
Parp14	58.24399	46.16308	62.28044	68.81247	56.76706	40.4002	61.96814	51.12278	69.53171
Dek	64.68484	66.76505	61.83703	57.66992	60.59056	64.8231	65.51175	63.06714	69.48869
Dock10	92.69499	80.37293	89.53438	81.50017	66.10003	53.82294	99.92476	68.62573	69.4835
Map2k7	86.4663	84.07133	84.55465	81.52363	79.29957	74.99253	96.98	79.3431	69.45507
Trp53	86.75494	83.99659	89.39331	66.10948	74.07299	69.69703	80.51469	79.88859	69.44599
Pgap2	74.04423	73.70913	80.69372	61.28337	68.24623	59.87058	72.811	70.85956	69.41707
Arl4c	76.52605	71.73684	86.68183	66.8145	81.33407	67.68018	90.77841	75.66773	69.37989
Fundc1	60.90668	62.99432	69.66455	61.925	71.6134	68.5883	72.13473	70.74543	69.32058
Rnf126	78.01968	80.75621	78.18212	72.01652	80.51239	76.54653	80.35986	74.02687	69.31711
Atxn10	69.99273	61.59514	64.65629	58.41098	62.27422	63.62218	69.37729	66.86745	69.29732
Irf3	70.78748	67.38526	73.70654	76.48944	74.67703	75.29897	77.53136	74.22535	69.28304
6430548M08Rik	79.2242	75.53017	81.18435	63.10569	58.43205	64.79709	76.64665	61.64944	69.21039
Bnip3l	59.79994	70.79706	64.25723	58.18822	60.7385	64.08423	67.65964	65.15755	69.18145
Rab11a	72.95107	71.2401	71.12962	60.964	63.1637	62.3647	63.65899	72.73709	69.16649
Kars	86.61689	73.88301	76.63989	69.13202	65.38447	66.65011	76.6778	67.76721	69.15673
Arf3	85.84061	77.89269	80.65814	78.85089	82.07218	68.80816	91.00852	74.07135	69.15461
Mcoln1	69.47056	75.05481	72.30207	84.93437	70.7496	68.39064	92.74095	74.65439	69.1397
Glipr2	78.75006	67.7233	70.10888	46.49934	58.44719	51.59749	45.20838	68.04991	69.11519
Vti1b	71.63396	66.19473	70.87096	67.80415	67.96416	75.30684	70.56751	68.89365	69.09506
Slc30a7	83.5178	78.53571	82.80601	83.87772	80.80988	68.81838	95.84081	74.70912	69.07472
Ski	87.99314	87.57257	94.43833	71.13329	76.76652	67.7647	102.776	78.8674	69.05854
Ubb-ps	36.27526	26.97672	26.49979	26.42671	30.56761	28.07673	33.09102	32.43532	68.99677
Plpbp	73.23639	70.72948	68.68674	81.30431	75.8863	68.74408	92.424	71.82306	68.97696
Plec	112.4014	97.65411	96.92413	72.03865	58.36722	48.15587	83.56877	66.16585	68.96048
Pqbp1	70.49927	67.595	69.546	62.24418	73.55181	76.06144	58.89882	65.44416	68.87187
Arhgef2	74.97541	73.64526	79.42556	66.83732	70.60559	64.4758	79.83403	69.83488	68.86529
Nubp2	67.30767	63.4146	63.63782	66.60664	62.77315	68.5287	65.81333	63.06419	68.85276
Hspa4	80.40909	68.38621	68.68427	62.48182	58.26053	60.79163	70.93098	66.52411	68.81388
Eps8	102.2227	85.74525	89.93778	72.08984	68.77752	65.53071	86.46176	83.55989	68.81322
Clint1	78.26157	66.22192	73.97614	63.80039	65.40153	58.83852	82.59685	70.58343	68.79372
Sar1b	53.22585	50.92195	54.29402	55.91946	52.20868	50.94006	51.81057	58.51145	68.77717
Nfkb1	79.56264	66.51505	73.51311	64.83308	68.51766	67.93738	76.87505	67.93276	68.76047
1110008P14Rik	71.13286	66.14659	92.66068	70.39877	72.64831	70.77552	61.22253	70.61764	68.68058
Washc3	48.82225	60.30022	60.22315	55.79758	57.99095	57.95197	53.81581	64.91369	68.65943
Mrpl14	53.82336	65.84755	66.30015	56.64398	64.16141	63.71092	74.07946	68.83927	68.65442
Ino80e	95.56749	89.16144	91.78816	82.83908	88.61106	88.92761	100.6171	88.01795	68.63196
Dram2	64.80873	68.40462	75.79028	73.01241	69.61125	65.74749	73.41893	71.17287	68.59643
Gaa	78.00119	70.52356	80.88755	82.20912	80.32161	72.6263	99.01028	71.66413	68.58079
Plbd2	84.01295	76.92989	85.75006	79.40204	73.05153	75.15414	90.48144	74.55616	68.54899

Pip4p1	65.91926	73.95707	73.37693	72.36186	79.03748	72.31436	92.89163	72.70509	68.49953
Immt	85.08788	74.59123	78.59447	71.34799	60.95724	63.19146	80.51081	68.67215	68.46864
Usp25	74.07926	55.76003	71.70411	63.74486	54.42404	49.43058	63.6239	57.5172	68.4306
Nfkbie	71.13382	63.75901	54.85965	39.35687	54.95956	38.3532	73.90717	64.3857	68.3765
Mvb12a	64.15572	53.10384	61.65721	70.44073	72.03877	66.37726	66.3621	66.84785	68.36025
Dmac1	67.72393	65.27452	71.90341	68.09871	80.53412	72.79828	74.46389	81.08716	68.23325
Myo5a	76.4547	77.73595	77.08197	77.48894	64.82194	51.86694	100.2327	61.96153	68.22804
Ap1b1	82.94731	63.42224	75.73966	79.10327	82.78086	75.21975	115.7957	64.10061	68.19277
Psen2	65.1543	59.11124	61.88573	81.35244	72.99797	71.7538	81.65755	78.12968	68.13035
Uqcrfs1	77.60484	70.78696	73.43336	57.85661	61.88656	64.34779	62.27285	60.94305	68.12926
Ppp3r1	66.21605	65.76615	67.32127	52.57478	54.72093	55.25424	62.38171	63.82779	68.0592
Smarcad1	68.85289	71.92196	73.30831	68.61461	56.04558	54.35165	79.97208	59.83895	68.02462
Mrps18a	75.84235	63.28967	73.79766	74.01381	64.77607	78.56252	71.29092	69.52135	68.00369
Dnaja1	64.38891	66.98621	61.23295	52.53427	61.24318	59.4515	58.41093	62.02541	67.959
Metap2	70.18246	64.08324	68.82242	61.60549	57.57592	61.26784	62.30288	65.16661	67.94562
Nr1h3	72.2275	63.9255	76.4502	81.38184	65.05766	62.7447	71.41836	57.13922	67.92932
Msmo1	81.40675	72.6838	76.32372	50.31732	62.07322	48.38323	53.41674	78.901	67.8818
Alcam	85.06264	85.60963	80.83995	59.41302	50.68835	45.53003	58.68351	56.35826	67.87346
Rab11fip5	84.73043	88.41088	96.6936	74.18379	77.78951	69.4264	103.8622	77.75288	67.84554
Slc38a2	70.20208	71.88901	59.01215	48.62634	53.81419	43.14349	69.0935	64.32523	67.77996
Itprid2	69.17	72.33258	68.62128	58.89496	57.39363	47.53495	83.34982	63.53036	67.72325
Noc2l	75.78612	68.77141	72.63853	70.90184	63.31878	59.0055	76.33296	70.85107	67.62
Snx6	80.605	77.35958	85.33556	62.52089	76.37393	67.06505	75.59433	75.73259	67.60038
Snrnp48	52.22849	53.73846	52.52637	50.58192	56.5964	54.59527	61.82153	62.50556	67.57625
Ptpra	83.46297	79.25023	76.61032	73.90344	61.39629	68.0711	90.56947	68.65027	67.53984
Cd80	86.10783	91.85352	77.98001	34.36579	51.39348	39.5255	50.69025	65.46729	67.49552
Senp3	70.70218	64.24108	67.0058	69.73756	60.30406	58.07238	69.34857	68.45152	67.48023
Dse	72.45597	68.15981	76.49596	76.61652	74.13177	57.28189	90.11335	68.54995	67.47264
Ctsk	136.1262	179.3884	137.8232	52.96839	36.38868	39.86744	47.34415	41.21234	67.45697
Cyp27a1	55.27058	66.21493	72.26211	81.84189	99.24861	86.02388	113.5228	86.14227	67.42754
Ugp2	68.7916	67.131	67.86057	61.87064	57.44854	58.4452	70.92704	65.67514	67.41392
Itpkb	83.12819	63.30442	69.79706	68.53576	56.95532	53.91362	76.08864	68.50988	67.36906
Fbxo6	54.65595	51.34443	57.65308	64.82631	62.04384	60.79759	54.34989	64.30263	67.35124
Npepl1	73.97029	70.31935	67.26315	73.58223	73.57059	73.51503	80.68718	64.0085	67.35068
Il17ra	76.14704	62.11474	72.07786	53.1696	66.88267	55.02527	79.95742	66.50325	67.33108
G3bp1	80.77233	70.014	75.02044	64.01774	60.76264	63.9458	61.07519	62.0022	67.20287
Otud5	60.78284	62.57396	65.81427	65.91681	65.13458	61.52383	72.64272	67.05544	67.17111
1110004F10Rik	78.36566	73.93126	75.84016	60.16776	65.65884	65.03634	67.93906	64.84703	67.14124
Ifi47	37.07641	23.88343	43.75846	69.14083	56.16157	41.02578	41.87034	47.03661	67.12522
Ophn1	69.31001	80.38193	83.19162	78.41458	80.73688	66.44069	110.6905	76.21505	67.10113
Mroh1	75.43502	67.03528	73.48086	83.81112	72.33378	70.41455	103.4058	62.30966	67.06639
Acp2	80.58217	71.94438	89.82371	74.08801	69.59705	59.89926	83.35223	61.93911	67.05908
Herpud1	73.52059	81.31518	82.9576	61.65452	77.86492	74.50582	89.42213	62.58537	67.03572
Bub3	74.33678	74.95719	74.09924	66.11495	67.17691	69.52839	69.02897	68.25005	66.99167
Tor3a	54.42023	41.8739	61.34863	58.06271	54.26882	46.02769	46.42751	50.97352	66.9845
Usp18	35.52919	26.69303	41.48163	53.22872	41.82747	35.11425	32.15935	41.1888	66.97909
1110038B12Rik	57.202	58.55764	45.74172	52.12057	64.79829	48.12143	48.96375	67.65753	66.88734
Card9	65.64249	72.66918	67.85835	67.85611	75.58896	66.36063	74.62926	73.71933	66.88566
Selenos	66.09744	64.8073	68.71208	62.44083	63.61545	70.46575	63.64841	67.89483	66.85639
Acly	78.54881	70.79494	83.09741	60.36821	65.02262	57.6452	79.54219	68.97614	66.84886
Mrpl34	63.76235	73.6921	66.0871	63.40519	71.35167	75.79002	68.11899	68.71526	66.82377
Prdm2	78.97272	76.50932	71.26053	71.20476	69.26309	55.2823	83.83539	67.2219	66.78632
Rftn1	81.73256	71.64872	71.45257	78.77609	69.39945	73.18684	92.38843	73.7862	66.75389
Polr2e	76.61232	70.04557	72.88092	62.34661	75.58372	77.76571	69.46246	69.1199	66.72818

Tmem51	62.46458	55.37955	73.48928	83.90516	74.66097	69.57716	79.30019	71.605	66.68522
Dnajc19	51.15342	55.46489	41.10239	56.4318	55.79663	63.07344	52.68886	61.52405	66.6718
Taok3	79.96251	72.25681	81.40921	75.09712	70.90418	63.22937	84.49531	72.44994	66.62269
Nrm	99.6991	110.2242	98.45871	68.10917	94.01558	80.37403	94.26446	75.00219	66.55808
Kxd1	73.17277	78.43426	85.81132	69.13608	78.72029	71.71531	82.03604	81.25722	66.52472
Slk	79.57519	72.39114	76.90788	62.98988	55.65756	51.36149	73.84165	62.44182	66.50258
Leng8	80.66144	79.1474	71.69169	79.8038	75.26641	62.16824	104.5218	72.52359	66.48058
Skp1	63.36868	67.91526	71.99397	57.62987	64.30972	59.05974	59.74125	60.22429	66.31929
Hras	55.58857	58.04249	62.87056	55.87586	51.79519	57.85559	57.40171	54.16771	66.27388
Arnt	91.79028	84.06522	80.8086	71.41101	72.39032	62.39306	100.0508	73.64091	66.25024
Srsf6	70.4737	68.57141	64.16682	65.68055	62.54856	62.77255	72.11116	63.08004	66.24412
Srp72	80.2737	69.92212	76.36116	62.39121	61.1175	58.79039	74.33641	65.22771	66.23362
Mbnl2	72.56802	65.42944	74.29486	65.3531	56.20777	50.25624	75.17757	67.18332	66.21113
Cntrl	73.95763	72.61763	70.04469	68.65446	64.74867	54.64244	89.12677	60.50743	66.20486
Mia3	72.74074	72.38291	72.38334	60.18376	61.1774	57.06901	73.3664	58.73813	66.19056
Gnai3	73.10439	70.34205	76.2976	59.69201	60.05781	52.7711	68.15189	65.82892	66.15572
Rara	86.37925	69.88988	83.87102	58.77723	73.22301	66.58051	77.67651	76.51223	66.09759
Zfp787	68.41077	65.19626	56.15403	67.3221	63.89886	63.45159	75.31493	61.65996	66.0709
Gm10705	7.588784	1.274708	5.91195	9.293833	71.05749	71.95163	5.44909	18.70964	66.00868
Trappc4	64.22226	59.83312	68.91518	54.86871	62.1561	64.3851	62.61085	55.26052	65.98473
Prcp	80.01486	84.5213	90.98586	71.42028	81.58489	69.39431	85.23175	79.65452	65.97544
Srm	77.89716	71.44462	59.61954	59.94288	60.15383	67.36565	70.93217	67.32944	65.96717
Osbpl8	74.80848	60.33087	62.57111	73.8885	51.98608	48.43464	80.60182	57.89594	65.9635
Rbm5	63.23309	71.34516	71.21909	74.5463	70.1017	62.94381	87.38418	64.01155	65.87746
Yy1	71.92436	67.3135	75.49355	62.88194	63.24775	61.16297	78.43727	68.11057	65.85771
Spop	70.23052	67.28094	73.54766	56.31347	63.7174	63.9686	67.0001	66.53668	65.85425
H2aj	56.10563	57.5602	60.20903	60.07603	70.60043	71.77995	49.08121	70.02994	65.85154
Gm15459	70.18643	73.65059	67.69234	55.54655	56.83688	57.33179	53.40555	65.69599	65.83967
Spred1	89.93838	77.8805	90.69473	60.29144	60.92754	55.45665	67.40438	61.5401	65.81639
Sdhd	67.47481	74.69592	86.29495	65.50952	68.61764	78.99566	72.63693	72.49888	65.78976
Ciz1	75.90046	71.54726	79.65789	76.62379	74.46596	69.36413	92.25339	71.26878	65.77675
Spcs1	70.75793	78.26405	76.33265	66.36127	72.68993	71.39722	62.93668	69.72952	65.70535
Golph3	72.08224	71.61473	68.81667	64.02656	64.58111	55.33654	71.82024	64.37923	65.70233
Tmem176a	64.31254	70.64287	75.82529	72.45021	129.0748	90.47781	84.81464	76.09646	65.68127
Arhgap27	65.55373	54.55291	66.93491	71.30597	70.5826	73.39614	79.3	77.67749	65.67545
Scyl1	75.68445	66.22541	71.24321	70.15846	66.22129	66.46647	78.86177	68.61316	65.66122
Il6ra	89.76234	78.93723	83.29822	64.45705	79.2918	58.77363	97.47793	78.6731	65.64741
Zbtb7a	75.59923	72.50636	74.08079	63.48951	63.98133	57.13551	72.11039	67.65547	65.63127
Azi2	61.96019	63.86818	65.44387	64.85591	54.744	53.78601	58.22396	63.35943	65.54297
Hilpda	44.2421	39.3817	28.35436	31.66391	28.77839	31.06102	52.13434	46.99416	65.51608
Slc16a10	72.21569	59.44981	68.58052	67.59259	53.57472	47.12255	73.2265	66.24622	65.49279
Arl5c	50.69336	43.4603	45.95401	66.17786	70.51705	54.50046	88.07632	74.06189	65.45173
Myo1c	81.65068	78.06206	80.36881	64.12581	60.38697	53.48078	77.14236	67.2849	65.44394
Pdxdc1	74.77401	74.01234	76.47518	63.41691	63.60307	67.09101	79.25846	62.03208	65.44191
Ncaph2	74.22489	75.40725	72.08093	63.81095	70.69098	71.48431	77.60495	65.19499	65.38005
Tpm3-rs7	47.74995	46.18841	42.31567	34.35069	39.77329	49.79224	51.32496	55.77016	65.37382
Cbfa2t3	87.86854	76.74881	87.95729	84.72148	103.5406	103.4425	97.99907	74.94961	65.37318
Tesk1	80.38519	66.53185	78.35748	74.65059	68.87155	68.21961	79.9323	65.30737	65.33155
Ppp6c	69.25674	60.50348	64.62536	53.56823	59.55011	59.96804	62.09253	67.86762	65.32284
Pkig	64.06677	77.11418	84.83066	66.0633	74.76669	67.85236	71.22906	67.87085	65.30767
Kpna4	64.36529	59.20799	61.05443	48.72113	53.91806	44.92432	56.72902	59.92213	65.2739
Timm8b	46.70683	54.87081	50.39453	58.78875	59.71151	58.07709	49.25236	70.30985	65.26973
Tmod3	64.57994	63.82276	68.29404	57.56911	58.01964	57.3652	60.74546	59.28103	65.25934
Crtc2	68.44879	67.34308	72.06599	59.11748	65.77779	57.43704	78.0578	67.81474	65.21515

Tomm5	64.75027	66.48744	54.22595	62.13746	64.56847	65.59328	57.6578	60.81999	65.19654
Psd4	81.11159	71.57883	78.41483	62.76722	57.79929	54.17184	62.1422	59.37644	65.19478
Ndufa12-ps	38.0019	61.75589	61.36902	76.91179	75.79154	68.52878	83.75212	61.08294	65.19453
Sertad1	53.66479	46.40118	42.89015	44.26907	48.39872	44.49215	58.16616	57.49202	65.15723
Ndufs3	62.03922	70.43855	65.844	69.24342	64.42654	72.66031	67.44138	67.07656	65.142
Gm15710	69.29758	54.9128	61.50401	61.67957	58.06404	68.56662	59.2549	74.84727	65.10838
Slc29a1	102.418	122.2429	108.5145	81.12718	77.21937	74.73292	99.99545	84.99447	65.08504
Scyl2	72.11185	65.46126	75.50985	58.3774	60.11893	52.19087	75.34437	58.37188	65.0373
Snrpb2	46.15331	50.6155	52.83005	48.71839	54.21515	62.35448	43.88189	61.49372	65.03725
Arid1a	80.9886	79.54203	83.26962	80.97814	72.38176	59.55831	106.4017	68.68739	65.03379
Mindy1	69.05821	69.2802	69.09017	65.76742	58.65752	60.78594	76.39734	65.65902	64.9973
Mrpl58	60.81294	64.63318	71.8782	71.39091	66.98549	72.14358	71.42411	72.00932	64.98561
Dennd4b	78.26637	65.87111	80.2113	68.79186	63.28234	58.4	84.09918	66.07656	64.97648
Psmd6	61.69921	66.20796	58.63765	59.25075	54.29924	57.55598	53.86955	60.28334	64.96985
Sidt2	73.40737	76.68145	83.01634	60.66759	74.95683	59.79381	73.54528	70.04043	64.90312
Nrd1	76.79611	70.77022	75.13334	59.92925	60.92244	60.16158	75.14964	62.73523	64.89935
Ethe1	52.30564	49.04229	61.47365	60.18186	65.80125	64.9314	71.65305	61.67333	64.89199
Ganab	83.39825	76.95109	77.32153	64.0004	61.67493	55.3735	73.55785	66.2212	64.884
Pfkp	93.88362	85.33336	84.47031	61.1568	60.65127	56.32091	70.11072	61.71743	64.8551
Idh2	72.91912	67.49788	66.02552	78.11081	69.38536	69.32988	81.28713	59.91105	64.84449
Slc35f6	80.38699	72.90736	81.43974	74.28443	73.7159	67.75213	85.85405	66.8772	64.83811
Ddx24	65.08782	58.12785	67.65229	60.23122	55.78271	58.43131	67.72847	61.91508	64.80093
Rbm7	57.24407	58.21742	58.83117	61.60557	53.53576	47.83763	61.66452	62.02477	64.73273
Tmem147	73.51292	66.05555	70.39153	52.77762	57.96897	60.83572	61.06066	57.52559	64.70755
Sumf1	63.56527	56.21506	59.43132	68.31019	56.77174	51.15308	61.4181	52.04693	64.67722
Rap2b	63.64084	55.73838	63.66144	71.6776	64.0452	59.21951	72.65666	69.60948	64.65878
Rbm17	63.72992	58.21783	62.19619	61.69467	58.1798	60.69977	65.71637	66.44094	64.65675
Dennd5a	79.78495	78.45148	87.10139	62.90708	64.71362	57.34837	81.74144	66.93057	64.65403
Nsd3	70.16187	66.06534	64.25821	65.10327	58.3989	49.12649	77.36507	60.26194	64.6199
Mdm2	68.2183	68.73848	62.55814	56.47025	49.01304	45.70975	78.99852	65.42129	64.61932
Eif4ebp2	81.04635	79.12791	79.34835	73.49308	79.02053	59.39094	86.8453	68.03205	64.58233
Gm13394	68.19945	58.70289	63.8606	63.54148	66.88997	101.5941	67.47688	63.96408	64.51539
Itsn1	90.35035	79.58268	82.74358	76.36455	66.44154	64.3866	103.1141	64.2531	64.49594
Eif2s2	60.3355	63.69515	57.05511	54.11843	56.47539	59.57054	57.1156	64.69161	64.44354
Cnih1	84.05367	85.43197	74.3868	61.44267	63.79238	56.03484	60.42546	59.32693	64.40893
Slc35b2	66.14916	69.01597	55.41204	56.16036	71.19156	54.98937	84.9406	66.22531	64.34531
Ddx54	74.72727	62.26603	71.25687	65.19397	62.29955	64.28848	75.76957	68.37888	64.34515
Chmp5	67.11561	67.82352	68.25493	59.98935	71.80918	65.91521	67.4045	69.67132	64.31374
Nsf	81.06676	67.85782	72.41942	56.04973	53.19116	48.14996	60.24963	59.34174	64.3
Supt5	80.13507	72.38283	83.55343	73.15813	69.7669	70.02019	88.43432	73.5018	64.29829
Synj2bp	72.03071	79.03058	76.84686	71.08752	64.90905	60.37362	61.94595	74.92896	64.29166
Bst1	71.48093	70.98293	74.62223	37.92377	53.27325	38.80168	39.24521	64.62237	64.25987
Cdk9	55.14361	57.2156	59.56726	59.74724	60.94663	61.1457	64.99071	61.1149	64.20313
Aamp	66.20836	65.11921	61.76097	57.76709	64.21845	72.28786	69.24954	65.82558	64.17839
Srsf10	65.48906	72.5098	59.71755	53.38629	55.13335	51.55886	65.44207	54.04952	64.15255
Psme2b	44.62088	41.02698	47.22665	52.59352	55.86944	46.31378	46.17166	55.20868	64.13846
Gipc1	74.64927	75.5454	69.8889	74.4358	70.86644	73.03064	74.63632	63.43042	64.11766
Tmem234	75.82953	70.72948	73.12702	67.27824	76.55985	76.95558	78.45482	73.84309	64.10888
Cacybp	55.88366	62.9501	48.86457	47.28195	49.60323	55.92144	47.97861	57.44929	64.02943
Ube2f	70.02247	64.05235	74.48868	61.19543	70.72787	70.1975	62.3524	74.29102	64.01533
Baiap2	78.55292	87.92925	77.16586	66.63356	59.75319	64.20216	78.38589	67.24972	63.99338
Tnnc2	54.87808	74.67804	52.56427	102.2206	80.15026	148.7716	50.93156	66.11138	63.89284
Klf10	54.71541	50.39979	63.31721	50.45657	57.88674	46.23134	56.39105	60.62121	63.85749
Acot7	80.32417	73.24249	70.91841	47.49982	53.2552	64.44697	52.92858	58.13541	63.84519

Olfml3	77.82406	80.32406	104.182	73.768	88.93037	77.55078	86.4751	65.88687	63.8249
Ggh	64.63593	76.05567	78.73454	65.14693	67.77015	54.43777	57.90296	69.73973	63.79538
Fam78a	69.84611	71.11901	80.46037	72.87306	76.13308	75.14256	81.63832	68.51972	63.78129
Rnf141	82.34183	103.205	102.6189	61.55521	62.50423	63.16874	81.55336	64.92465	63.77119
Bag3	84.81276	79.00465	75.16523	71.1878	63.96871	70.09506	88.96095	73.28525	63.75102
Mospd3	80.5843	73.58675	80.99641	69.58793	102.529	76.92177	90.2247	86.26675	63.71192
Phyh	61.83187	62.49249	70.2272	51.20053	62.11179	55.82371	60.60216	64.56363	63.69882
Mfsd5	72.52775	72.23944	71.13575	65.41251	67.55918	62.74516	69.50097	71.08362	63.69606
Gsr	50.39635	38.21912	46.37667	34.64039	44.32158	43.38157	35.6397	46.91556	63.69359
Pdhb	60.44055	65.52945	62.01571	55.86998	61.15394	59.58755	58.10828	60.56655	63.68952
Gdi1	65.62014	66.40267	65.00174	64.85835	69.82849	66.06681	71.42303	68.25765	63.5759
Vps72	68.7957	58.83276	66.64152	71.09762	65.33643	72.25988	67.23338	67.63213	63.5447
Rb1	72.18231	68.40981	76.42212	65.70936	58.07358	48.84866	77.8638	64.42782	63.54174
Ndufa12	72.0101	46.29971	52.20529	58.99236	56.65439	51.8165	49.09664	60.4403	63.53864
Antkmt	56.31386	61.75413	58.24739	73.06792	66.97832	69.4094	70.87573	57.11847	63.52174
Prps2	72.03044	79.49628	79.97805	60.43323	62.09413	59.99247	77.54061	58.85369	63.5041
Aldh9a1	71.26912	68.01528	68.53747	65.83001	69.03143	66.93895	77.05803	68.54455	63.48694
Eid1	58.77219	61.68017	56.90472	55.66927	63.07192	59.89256	56.19439	67.3267	63.47134
Bin3	58.30555	58.62697	66.6768	59.48387	62.69834	62.05858	61.00262	60.36616	63.46561
Kpnb1	81.29477	74.23838	72.7844	63.13216	57.03393	55.13695	71.66857	62.46536	63.46032
Prelid3b	66.57357	65.36138	64.34596	51.75399	58.80449	58.76003	53.29663	58.82452	63.45053
Ppil2	65.59559	60.53879	68.75486	69.12237	67.04723	61.62272	72.19169	63.79338	63.42237
Nt5c	55.50693	50.13919	50.48549	49.64901	60.40685	60.82031	47.54667	63.99465	63.40488
Copb1	72.21163	66.88229	70.36351	62.8936	56.87596	56.68023	67.323	62.73392	63.373
Gas7	95.88526	93.54247	93.75342	69.73003	70.62926	56.98246	99.33307	86.16437	63.35599
Atxn3	68.21869	78.74202	86.32079	65.03067	66.18268	56.89554	81.48668	62.00096	63.33105
Mrps36	60.4353	52.72063	61.84796	54.58401	56.59118	63.32605	57.78221	61.62911	63.33046
Ubqln1	80.15587	72.6973	78.48738	63.05536	60.86058	54.55522	77.6116	59.64038	63.32567
Mt2	86.94603	127.8841	70.1034	43.72525	67.13779	75.70238	62.2446	66.54768	63.29084
Smc6	65.44903	68.22612	64.48061	50.2753	49.89033	51.20916	58.85979	59.82613	63.27886
Abcf1	71.33031	68.7006	69.40244	61.70783	64.38077	64.14119	72.45014	64.42813	63.25391
Tlk1	69.65649	65.45092	69.0019	63.14408	53.98184	53.05459	78.18552	61.01002	63.23121
Acadl	63.29274	63.91896	67.96791	62.7684	57.89416	58.05742	67.53399	65.49036	63.19874
Bclaf1	60.99547	63.52811	58.02806	62.27619	54.09348	53.04335	69.11945	54.99927	63.13923
Polb	54.81164	56.74822	49.64946	48.84652	52.84832	52.90871	48.55763	59.93941	63.13621
Ndufa1	57.90696	55.17855	61.50524	69.74636	61.66962	73.58011	58.3419	68.91638	63.10539
Cnpy2	52.61822	65.47112	56.40336	66.00722	64.29291	63.8365	54.73329	58.58156	63.10387
Otulin	51.73721	51.18936	49.23831	62.71081	60.38195	59.46049	63.98213	65.77343	63.06284
Bcl2l11	59.86541	55.48245	63.60236	67.76092	66.96073	51.95718	83.21143	71.68244	63.04254
Ap2a1	78.21002	69.72743	70.28746	64.48772	60.73673	59.52713	74.42772	59.17868	62.92787
1810037I17Rik	60.49338	63.67468	60.72764	60.21101	69.35226	64.29434	59.70499	62.13838	62.90682
Tns3	76.24388	64.60968	71.76988	74.33398	56.57173	48.00351	82.07577	55.37833	62.87003
Dgat1	40.26863	41.62316	27.74058	36.76383	38.49099	43.06731	50.21623	35.793	62.82517
Scarb2	68.55484	71.42906	82.98472	64.69106	72.14654	61.44267	72.40837	67.78651	62.82066
Rnf166	60.27181	60.97578	68.68051	62.65245	70.95904	61.50236	73.07821	78.65001	62.81391
Dhrs1	67.89067	68.8245	76.44077	65.52595	67.47122	64.48101	68.18834	69.19862	62.80385
Cbx5	88.37829	109.9882	99.1718	75.97974	65.97804	60.06057	92.9228	56.17736	62.78993
Mkrn1	61.18018	64.73679	71.48878	58.36513	67.23154	61.72341	69.56522	61.88484	62.76733
Chchd3	66.02815	71.62951	64.75634	61.7711	64.40753	72.10298	62.4034	60.93668	62.72748
Kdelr2	71.02065	65.45196	62.57737	65.13988	56.25192	53.90003	61.85087	62.48303	62.71942
Nagk	65.78296	63.93236	67.3381	71.12945	72.41914	73.05807	76.48872	69.91362	62.69701
Mapkapk3	56.86586	57.00909	59.21138	58.06505	65.62313	56.73105	68.23857	74.04077	62.69162
Tcof1	69.92483	57.08864	71.91267	67.26386	59.40293	60.03542	69.65154	63.22612	62.69109
Parp1	71.55695	65.51376	72.97565	69.58795	67.78902	64.89755	83.72154	64.41887	62.67938

Gm10073	49.04136	60.60686	53.31203	53.00724	53.70114	65.50832	52.27831	60.11337	62.65974
Eri3	67.18794	79.20706	80.86993	67.02402	69.53342	67.56626	82.41418	68.54602	62.61079
Nucb2	70.21429	66.98584	66.08317	53.97227	57.29357	57.54571	67.95461	55.44406	62.59519
Atf7ip	64.4013	57.27886	66.00058	63.2742	59.53206	44.71371	74.12897	58.71631	62.59371
Dhrsx	59.56874	59.19333	65.22883	61.91578	70.6466	66.6677	70.42037	68.67931	62.53462
Ifit2	29.74725	19.08714	39.84099	73.44919	47.50601	29.25443	40.86572	38.56473	62.46737
Sumo3	63.9218	70.45492	68.11284	63.93765	61.84084	63.63328	65.94493	61.37444	62.44623
Pilra	51.49656	45.69796	60.13629	32.37784	48.47353	42.43228	42.08791	57.96282	62.43306
Cd151	63.48176	71.50146	70.7994	61.4335	59.37856	64.4469	59.91971	63.07839	62.41239
Ccng1	59.87311	69.31342	82.02573	54.64816	58.67532	56.98853	60.30407	65.66445	62.40939
Psmd12	69.36997	63.79423	60.81841	52.24797	54.39206	57.24808	62.55226	67.18047	62.40517
Rab10	67.06854	67.56675	68.51255	57.55985	56.78142	50.75661	64.70287	58.68725	62.35806
Vti1a	68.56813	64.41228	74.91742	72.8152	63.70034	63.40966	72.6084	68.80953	62.35669
Gosr2	66.99549	59.71371	63.41348	56.36089	57.34465	56.32461	61.41569	57.68498	62.31231
Stx5a	59.13801	59.01107	58.50267	66.92536	63.80681	67.30081	76.73445	61.34179	62.30908
Parvb	99.23488	87.6589	84.65575	72.03032	69.32934	67.09731	85.01137	73.12616	62.29251
Ubald2	68.94221	62.75207	66.14432	53.78839	55.75561	51.92466	56.52475	60.83162	62.24973
Lsm14a	61.50065	60.30212	60.04284	58.78921	57.99127	50.96637	61.62952	57.95418	62.2221
Elmo1	74.99707	60.84605	73.45654	64.07427	60.99483	56.30296	80.86086	62.09352	62.20288
Ap3b1	76.77206	67.66894	72.67092	66.00218	63.96249	57.78421	71.46572	64.87517	62.19566
Rwdd1	52.56881	54.57507	52.77989	54.87278	51.52988	50.98309	47.28939	56.8314	62.16452
Tnip1	74.08892	66.90019	82.30915	60.54222	60.70002	55.31168	67.22434	64.45748	62.134
Pou2f2	66.22988	48.05362	67.8785	78.93416	81.82793	74.94569	74.85283	79.31672	62.13185
Usp8	70.13041	65.23137	69.54124	70.48985	64.4277	57.24469	79.54779	64.63546	62.12221
Arrdc4	66.60653	71.72137	77.02591	45.02667	57.22656	49.58495	55.52213	57.49777	62.08515
Rbpj	96.74425	90.21347	86.29155	56.31306	59.2981	47.65567	69.88572	69.32959	62.04525
Mgat2	64.2539	61.48317	68.92413	61.38627	58.57093	60.43907	65.16115	61.05495	62.02739
Rala	58.89513	60.39996	58.65204	52.45606	49.20862	54.96511	47.4183	53.73422	62.02737
Polr2m	65.52003	66.13474	68.82062	59.87377	63.12982	65.85451	63.12805	62.81891	62.00853
Ddx41	66.70099	58.76103	64.10522	52.45964	59.37629	62.07603	68.11517	61.73551	62.00229
Rmc1	65.65451	60.58256	60.87015	51.38673	53.96772	51.23076	64.41933	58.51314	61.93298
Tmem30a	64.6323	66.40899	68.69968	56.66387	56.24638	54.46109	57.92421	62.25735	61.93236
Acsl5	73.21111	74.11597	81.37195	61.3197	58.44089	64.08423	62.25906	60.97569	61.92527
Slc66a2	73.02748	66.93332	65.23499	53.61789	57.28574	59.30363	63.19553	56.57045	61.86815
Araf	73.50572	70.88161	81.808	69.90072	64.52789	59.85002	88.73714	63.09706	61.85103
Tsc22d1	70.37533	80.82366	73.10657	74.90725	64.07143	59.73035	77.30931	63.36777	61.84787
Lrg1	52.73377	42.74489	26.10358	29.94733	35.31614	52.59359	8.469772	24.85028	61.81937
Cybc1	62.06196	66.70353	65.85117	53.92322	63.86207	62.76426	59.80144	61.15989	61.81245
Git2	64.38325	64.62487	69.08474	66.20519	67.18149	59.57791	81.58117	62.21918	61.81028
Bcat2	64.29688	72.46143	60.20315	61.97566	71.84903	65.71257	67.23052	59.22008	61.758
Slc44a2	71.55474	62.29404	72.00713	66.11705	59.97207	59.44948	56.65211	66.10726	61.75596
Srgap2	64.21178	51.1787	60.18622	67.31118	56.87231	44.08304	82.26518	60.33859	61.72754
Fam193b	59.27231	70.18411	61.58579	68.02788	66.27968	62.81121	94.09626	68.92188	61.68485
Azin1	67.80963	54.69209	50.06807	51.22564	42.55887	37.56279	55.68572	56.5755	61.6665
Sf3b4	72.76047	63.22994	69.58429	66.08337	66.11198	61.98613	74.01678	66.97098	61.63857
Gng2	57.46236	60.03184	66.34936	68.02874	70.2959	64.98123	75.49389	69.26695	61.60709
Supt20	62.37123	69.52824	69.53085	64.16616	60.88347	59.82457	79.38778	66.81621	61.60461
Aspscr1	60.21536	63.96628	64.42973	66.22393	72.18639	68.57631	72.40552	63.44079	61.60399
Nek6	71.78709	58.97156	61.76191	60.7191	54.19052	54.61191	72.01934	63.37198	61.5728
Rnf44	81.65435	67.19143	87.37792	70.61827	72.69373	61.76578	88.9457	65.78685	61.54437
Ubp1	60.92387	65.14828	61.92189	57.17018	56.27401	51.93784	75.98105	58.51169	61.54382
Snw1	56.37145	56.02539	58.22042	56.49533	57.89834	61.96607	56.68872	61.50549	61.52268
Arhgap1	76.21949	67.5842	79.1586	60.85047	63.04341	55.85416	75.27258	62.08765	61.4342
Acbd6	59.0706	63.53945	65.22554	53.84937	65.62568	60.88002	66.7129	63.4773	61.41282

Sema4a	69.31181	58.37583	72.69855	51.22085	66.24679	54.37397	61.67235	66.51567	61.41149
Mta3	60.34948	54.97032	65.91058	65.2784	64.63014	61.24181	71.66398	60.39268	61.40397
Eps15l1	63.74238	58.69836	61.82363	66.24776	60.78644	54.81033	76.30459	64.02117	61.38375
Kat5	60.19101	58.63199	58.87326	60.74488	61.77771	68.20039	69.57902	62.46358	61.3404
Adam10	73.72006	73.42738	80.76191	57.41049	59.54228	49.33424	78.4663	63.47011	61.33653
Ear2	57.91574	75.52246	59.08402	52.74072	90.18533	62.43737	50.25579	63.02935	61.32385
Ghitm	63.58399	68.97039	68.32305	55.94325	57.97672	58.58878	60.7485	63.04472	61.27393
Mcts1	54.40377	66.01847	53.72049	59.65292	58.77457	63.82917	53.5209	63.94294	61.24932
Tap2	48.53614	45.72011	55.02144	56.07953	57.08186	55.83821	52.5807	58.39988	61.19801
Ywhaq	71.99066	83.93146	74.73184	59.17718	60.98114	59.3341	62.67478	64.63976	61.17619
Rbm38	51.11333	48.66437	52.96847	41.47809	50.05272	55.17982	53.76749	58.3024	61.17071
Elk3	82.06707	71.55711	85.82351	63.95949	63.57979	52.86529	76.57537	65.32849	61.13316
Dpp3	70.59001	61.57824	64.06173	66.8719	62.54573	64.96849	70.76456	58.725	61.10952
Prex1	73.44482	67.5509	80.41576	66.76931	71.35493	60.83782	90.79935	64.81008	61.07453
Fxr1	57.18224	62.22185	63.09295	60.33075	53.94862	55.11368	60.26552	55.51612	60.97016
Cd200r1	58.86813	52.95137	52.30724	62.1533	52.40771	49.80338	57.0859	49.37715	60.96838
Gm8203	62.92796	72.72038	60.05421	49.71662	49.75019	66.89624	51.93872	52.96532	60.95215
Gnptab	98.32856	91.52743	87.05295	66.79566	48.30606	48.10475	80.11482	62.29252	60.94116
Lrmp	64.14347	61.90401	64.43381	54.78376	64.62142	62.19513	67.14001	55.05654	60.92476
Gm15500	56.03651	62.39847	61.5385	53.61274	52.17683	65.50553	51.73127	59.25726	60.82264
Dot1l	79.60545	56.5032	54.71484	77.9	47.61622	47.36915	103.4108	63.99308	60.73742
Rnf181	73.43743	76.74246	74.61606	65.82191	81.85498	77.65011	79.29635	75.44637	60.7287
Rnf220	63.69355	60.6232	73.33836	65.46941	66.50889	66.12868	68.01671	69.03399	60.70561
Tomm40	65.37307	54.65576	58.09651	53.26416	54.8863	55.77944	58.43081	61.19804	60.68887
Anapc2	67.87674	69.0677	75.87462	62.75005	62.6225	62.8638	73.41503	59.8304	60.68301
Prkcb	76.18941	79.98056	88.80021	52.97552	64.81843	49.02292	73.20599	67.32124	60.68301
Snx4	63.76966	54.72443	57.19276	60.56804	53.74993	54.45847	61.28681	56.28401	60.66997
Uck2	97.28593	83.39026	77.53114	40.74822	60.84862	54.90745	54.55729	66.3134	60.65639
Trim47	81.10852	80.86736	77.67796	74.31653	66.16686	65.06654	88.61325	63.06446	60.6232
Tmem50b	69.6344	72.36035	81.59475	50.41365	65.55866	61.00403	60.03139	59.64752	60.58942
Cops5	53.82954	51.85813	51.70331	55.98308	50.26912	54.96888	51.76925	57.91113	60.57405
Abcd1	77.33671	75.17564	93.76019	66.64265	72.49677	61.25947	86.02554	65.6453	60.55734
Tmem9b	68.86164	60.7703	67.32312	55.88001	62.29295	56.98743	60.01838	63.90281	60.55135
Rnf4	71.28296	65.85735	70.10752	54.53904	55.144	52.96217	70.63597	56.29039	60.53376
Nabp1	55.9409	59.84577	58.46189	62.91955	62.53622	46.96604	62.19194	62.31762	60.50541
Rhbdf2	65.96269	56.47582	59.71428	47.07222	51.97944	42.92288	62.22773	57.11525	60.48354
Adipor2	69.07677	68.03248	74.0972	55.22965	58.94116	51.36236	64.68582	62.83441	60.47229
Iah1	56.50162	60.19634	61.69365	54.59872	56.66037	64.05349	57.36671	61.00995	60.4399
Adam9	78.18367	71.88455	87.0277	59.27765	63.64981	50.03234	80.47602	64.12043	60.43485
Cdv3	69.73604	64.736	69.39333	55.01126	51.3987	52.25597	59.71491	58.82566	60.41516
Pycard	60.34569	56.11918	60.89806	65.43005	56.68122	69.03688	63.84635	70.55792	60.3856
Rasa3	61.38843	55.00851	67.23892	66.02452	59.10674	54.81552	75.48365	63.70298	60.38193
H3f3c	43.30263	48.67907	51.3276	49.4925	51.93305	54.5303	43.81612	58.38138	60.37832
Tm2d2	71.71028	70.44032	73.7096	62.78771	73.65426	66.23538	71.61132	69.01689	60.34435
Med8	55.17175	53.07708	53.9796	58.5261	51.36133	63.41722	53.35275	57.43105	60.32516
Marchf7	47.18046	45.87543	42.33277	41.4014	40.58444	35.52326	49.12161	49.26881	60.28011
Rcsd1	48.19673	53.8332	55.8958	48.86692	60.04306	54.98328	77.37451	56.0558	60.2715
Glb1	70.30622	64.4062	79.65478	65.7023	68.38202	64.2109	79.74358	56.69492	60.24318
Rin2	70.79235	58.27205	68.76745	59.77698	46.93336	45.76023	64.23161	58.03111	60.1658
Zmym2	59.76656	67.20366	70.55438	54.17562	54.78053	43.30016	59.53486	53.93164	60.13626
Jak2	64.27474	62.81015	67.40399	49.70658	53.48947	49.13294	60.20607	57.15659	60.12821
Parp12	46.40727	36.6263	53.04059	53.96659	47.53346	38.9467	49.02892	45.80256	60.11911
Lmo4	62.40205	68.67412	63.27012	46.21842	56.18934	64.07519	52.56698	57.10341	60.1086
Mat2a	65.33983	61.2236	62.67898	50.74484	54.77502	52.38229	62.62948	57.63733	60.08959

Sbds	49.55827	56.92786	57.08646	57.17772	56.25306	57.97593	56.82447	61.03427	60.07889
Chtop	59.97544	62.29505	59.93414	54.98353	55.05122	53.85642	57.84679	58.96379	60.03727
Aoah	45.90224	38.69152	69.30563	57.01223	69.26996	58.4974	79.76421	75.95939	60.00658
Rbx1	55.47156	53.25257	57.22007	47.66616	55.82934	59.78613	50.33915	66.04985	59.95688
Pvalb	50.92432	67.12194	42.5962	97.19247	80.85131	144.8116	43.66132	65.84035	59.95225
Clk3	60.85154	59.08982	55.04346	58.55015	55.94933	56.86075	69.31712	61.75453	59.93971
Fam214b	65.50758	62.23884	66.64861	57.36292	53.78946	53.36344	65.55475	54.2683	59.92016
Lair1	59.09173	52.38614	64.77872	66.78254	59.32737	58.15879	73.07599	63.56284	59.90537
Acox3	60.14149	71.69754	82.30845	68.07263	69.42157	61.71876	83.33208	67.08701	59.86964
Scp2	54.25189	55.14519	61.70907	53.88591	58.01108	63.35294	61.59966	57.57727	59.83875
Hnrnph2	60.06942	59.74864	60.74469	49.51396	53.52277	51.88532	54.91654	58.06949	59.79893
Siva1	52.227	55.94981	60.92284	47.14483	49.47058	56.07097	52.2222	61.32478	59.76837
Bcl6	64.16757	45.79272	50.79578	63.66319	52.8603	47.97237	72.21256	56.40616	59.72185
Septin8	73.17906	77.95486	78.21306	68.71056	60.065	55.53492	88.79813	61.92128	59.71818
Amfr	63.59168	65.96973	63.53357	52.70165	56.68131	53.11459	67.55276	60.98199	59.70345
Rnf5	66.06243	70.25556	72.31454	58.93288	65.21898	61.93063	55.73675	58.82297	59.70002
Ppm1g	70.77635	61.39413	66.14927	57.24096	54.70082	58.03989	61.10905	60.85221	59.67087
Mtx1	67.17609	61.77735	63.32159	60.68575	64.02617	65.31775	66.75139	64.29688	59.66606
Josd2	60.47106	62.10813	54.91861	64.61424	66.24866	66.55034	59.70483	63.00997	59.63896
Frrs1	81.11088	89.91227	86.30782	67.2887	57.46664	51.66344	81.69269	62.90855	59.61782
Tpm2	53.73292	70.18115	51.65947	81.76078	62.66121	102.8809	33.39876	50.16908	59.57585
Rad21	67.80138	74.51648	76.31495	59.63796	59.98341	55.40373	66.86938	55.1067	59.50212
Rsrc1	66.78932	58.06595	60.13202	57.16659	57.68091	58.71707	55.78419	57.03575	59.46825
Ppcdc	56.02427	66.31516	63.23354	57.54248	69.22838	67.37557	76.16653	66.65581	59.46203
Znrd1	51.63983	51.50235	52.21159	58.58304	56.6469	72.41705	52.20299	60.55365	59.46133
Ctdsp2	65.129	75.27578	82.14795	63.60458	74.15549	65.62126	84.81102	70.29155	59.39097
Zfand6	52.42353	54.66717	51.58398	53.66526	58.57564	51.1314	58.43789	54.07753	59.38952
Washc5	77.51468	61.61446	70.55204	62.23636	53.50293	46.75869	82.47912	54.93393	59.37681
Gars	72.23185	64.38217	65.62866	50.90646	51.09756	52.59733	59.04811	54.06338	59.35917
Txndc12	67.39529	66.21847	67.22918	56.7273	64.39359	57.9422	51.54573	63.42176	59.30221
Pak2	73.0832	62.57448	67.41382	53.50072	54.5329	45.08972	61.44464	55.34139	59.25993
Gm5963	53.75812	53.27991	50.4041	50.11593	58.23846	54.06379	36.636	61.57955	59.24193
Npy	87.17293	100.3239	92.96261	43.9267	29.94408	37.59004	30.97737	44.20299	59.18535
Neu1	53.50853	54.52933	53.25553	56.43054	54.37571	51.3606	61.36095	55.51071	59.15044
Aig1	72.31496	73.95866	79.68869	57.67031	50.46066	54.38883	54.23224	46.93939	59.0765
Hs1bp3	69.13274	75.3731	77.25707	68.59418	65.21328	55.16052	79.94461	68.8635	59.07347
Coro7	65.67376	59.18997	64.9184	68.8936	66.08908	65.31888	87.96755	62.52786	59.04822
Tufm	75.3583	69.40948	70.03517	60.46476	58.69942	64.95867	69.0629	55.76627	58.98288
Ptges3	61.39609	57.38438	54.22439	45.30952	49.33994	52.50053	47.24006	64.0737	58.97562
Cd302	46.55456	59.76219	62.27554	49.75228	56.51007	49.29109	49.32912	55.89436	58.94785
Irak2	52.82254	44.96569	52.0421	52.1629	42.81721	41.96449	63.85769	58.32019	58.90385
Mrpl32	45.1878	54.21058	52.1958	53.97903	53.48609	59.61169	57.89486	64.05373	58.89168
Dcaf12	66.2684	64.06411	65.5706	56.48921	59.91219	60.3236	68.01994	59.41979	58.88839
Mfsd11	66.79753	64.07788	61.4162	67.67845	62.3227	71.10993	75.43568	58.21324	58.85216
Tbc1d13	65.51918	61.85884	67.93813	59.29508	61.3672	54.90369	69.84135	55.44778	58.85216
Ap2a2	76.82934	74.13381	76.59923	61.0455	69.01719	53.68764	86.97835	65.5827	58.83984
Slc15a4	56.52388	59.37698	63.35344	63.78577	62.67151	59.47092	69.6427	62.12571	58.81236
Acaa1a	51.81762	53.42059	55.9941	63.93507	67.63763	74.13593	61.70811	61.5539	58.75272
Igbp1	57.41017	65.88963	70.05439	66.20387	66.15477	68.9982	66.99866	67.73402	58.68502
Txndc5	73.20475	61.58075	65.87911	59.63467	56.11761	61.96374	65.1867	55.16409	58.62445
Tysnd1	57.8577	57.27781	58.71851	55.12752	56.39081	66.00923	63.55003	49.46239	58.60271
Mydgf	46.51211	49.59791	48.8859	53.0142	53.14558	55.53499	51.64979	49.83405	58.50888
Tmpo	66.37866	65.41768	69.66894	56.91241	55.04535	54.12875	57.81038	55.09363	58.50728
Lacc1	56.36523	64.12292	69.92914	69.40218	66.56156	55.76141	89.44965	68.94596	58.47461

Ccdc88b	45.12368	42.83639	47.79601	48.23834	51.29353	48.68379	57.99801	50.47181	58.44524
Slc35a4	73.07284	68.69361	71.31765	59.82395	57.06496	53.72297	73.28279	56.95103	58.42058
Ubxn4	56.12133	56.21242	52.12159	51.95683	53.50037	52.95234	55.22099	54.98918	58.40461
Sh2d3c	36.00994	30.19363	42.33278	42.02643	47.78777	43.94917	41.11817	44.38572	58.3657
Tmed7	59.99902	59.7592	62.07793	55.09559	53.91514	49.00996	61.39783	58.92921	58.36298
Wbp11	68.89701	64.15399	74.37469	58.80269	61.12023	54.94204	74.38805	60.83713	58.35529
Cxcl14	66.14941	99.86785	89.94938	42.06227	44.50457	46.74957	42.5266	47.64001	58.33226
Limd2	45.5617	43.89785	48.49298	50.53202	62.87914	61.50363	50.71114	57.60829	58.32538
Rpl17-ps10	44.29654	49.46571	48.25559	45.03124	54.51007	55.75432	41.88233	54.40786	58.3181
Pdlim1	94.3368	91.22009	89.22697	60.07252	65.81407	66.73276	71.57492	70.00253	58.31423
Gm7665	68.80062	51.32542	49.03857	40.30239	48.69672	52.18652	46.68304	57.93454	58.3106
Bcor	61.96019	63.42142	69.71331	59.87011	64.63947	49.45795	74.36101	57.09045	58.27654
Nudc-ps1	59.59045	56.01702	55.44451	54.9102	55.22692	53.49054	51.42515	49.73066	58.25306
Fastk	64.89869	66.64906	68.16058	64.56512	65.28088	67.3163	66.82691	63.62582	58.236
Cxcl3	38.17493	24.06059	26.03386	11.20962	38.63945	24.02214	30.22498	76.68494	58.23533
Mif4gd	53.20027	56.34322	58.35819	61.80329	61.12253	60.16987	69.16781	63.23444	58.22979
Nab2	61.04468	60.90537	59.79538	48.74263	59.20179	56.10799	57.53845	65.67353	58.22535
Riok3	64.53893	60.09309	57.38302	54.57038	50.31804	50.27312	59.95866	54.19538	58.20629
Txnrd1	72.85667	65.79733	60.20168	43.60237	43.96162	42.46984	61.14095	53.18113	58.20095
Tmem192	65.40571	63.72366	64.51724	48.89088	65.54493	58.87981	68.12096	58.16735	58.12114
Usf1	53.00296	56.64671	62.27623	61.70006	74.96619	71.32981	78.38027	65.09737	58.1121
Nacc1	75.78026	67.11969	76.19674	66.24195	61.60255	60.31457	79.32838	62.32237	58.07602
Agap3	66.23699	74.43153	70.86952	58.84877	57.12636	55.42019	64.28759	58.73768	58.03452
Setd3	73.30226	73.20527	71.90805	61.07337	62.99274	61.89405	72.65885	59.71705	57.99684
Tmem208	55.76561	57.16888	53.80656	62.45615	55.52822	53.08484	55.68994	50.28758	57.98846
Cnep1r1	59.7432	64.55577	74.40441	58.35707	62.50952	55.59787	60.88889	49.54649	57.87639
Mfsd10	65.88763	64.77069	68.5324	61.60169	63.99079	61.92426	64.80091	65.58292	57.86362
Csnk2a2	65.74748	68.25902	62.32934	62.53518	63.409	57.5105	71.59673	65.82769	57.84538
Hnrnpul2	65.04746	61.70347	67.54967	59.52919	56.62487	56.06986	67.82148	60.05308	57.83566
Ccz1	55.97354	51.45784	52.29425	50.39783	54.74855	52.89862	51.95887	61.7195	57.82093
Ndufb6	54.09207	46.39701	47.03108	48.35161	51.35646	52.35652	45.03849	52.14171	57.80844
Fam107b	51.19426	50.23647	54.11928	38.21178	40.57102	40.63636	41.10429	45.45157	57.8037
Taf1d	50.31922	51.72645	41.92146	60.98602	50.91294	56.53703	56.44952	62.80562	57.71611
Akt1s1	67.05102	71.37664	63.9248	65.66728	70.5737	64.90459	80.3982	63.13713	57.71267
Ube2r2	59.22097	57.86134	62.49225	56.35924	61.86938	54.10009	62.53994	61.59548	57.68721
Qrich1	62.78662	54.77811	64.60335	56.66008	51.60734	49.42903	72.1881	53.29286	57.64854
Mtarc2	52.71093	49.20121	46.65154	53.06328	57.4117	58.79563	58.09732	47.3182	57.64785
Nf2	73.72722	70.29617	77.27991	59.57715	50.82509	50.73971	70.024	61.53189	57.646
Usp4	65.51487	59.72245	61.01556	58.13052	48.68559	53.51024	62.182	53.76572	57.62453
Sap30	35.24043	35.14661	39.13454	45.15517	44.72037	40.86656	37.34299	50.90854	57.57177
Zc3h12a	52.37464	48.11552	38.40594	35.3563	45.49527	46.42394	76.84674	61.36315	57.56545
Crat	76.30736	74.42293	83.70029	73.41849	70.37782	69.42212	86.16755	64.15975	57.55575
Ahcyl2	74.87119	72.8883	80.06664	63.54485	61.42805	49.80654	73.62486	55.47835	57.49618
Snx17	67.17688	58.93662	64.58528	54.38908	65.50182	60.88167	70.67049	62.43614	57.49232
Psma5	63.50095	60.28797	61.27597	67.67504	60.7813	61.04062	59.65207	57.42316	57.48292
Cpne2	59.53736	49.80685	50.69398	50.2424	45.30021	47.83752	51.5144	44.66685	57.42656
Sptssa	62.55594	64.78275	59.67505	53.02435	59.6938	59.84329	67.05086	64.47748	57.41173
Wars	71.93104	68.82793	70.13339	60.69415	61.94234	61.3149	70.98482	64.21581	57.39828
Pde6d	48.10896	58.10185	54.44212	51.63923	58.70489	58.91405	46.74481	59.52193	57.37485
Nfe2l1	76.4142	72.92759	75.08707	67.24103	57.4484	55.76762	72.23888	58.86926	57.35419
Tmem132a	58.26718	53.46753	56.12802	58.28851	51.48167	49.4889	77.66771	51.05735	57.3516
Hdac1	63.70736	56.83333	60.33915	57.47695	54.16478	53.53033	52.41416	55.67466	57.34104
Rab43	57.8341	59.41329	59.06532	53.2031	62.42799	57.13556	74.55136	61.15547	57.32939
Naa50	56.69657	55.79761	46.67204	49.6034	42.88298	45.1073	51.92426	52.11679	57.28393

Ppp6r3	72.98037	66.72238	74.53625	58.89804	57.34377	49.57459	75.42886	54.27631	57.26128
Rpl36a-ps2	57.11377	62.11474	46.64601	61.37624	56.38944	68.31347	51.81356	69.53459	57.22942
Dda1	65.71322	59.69941	59.74623	53.4939	55.95628	51.59885	60.75564	54.65943	57.22154
Tbrg1	61.49551	64.26413	68.3048	53.78897	56.71211	62.27431	58.83571	55.00844	57.19269
Nagpa	66.05167	55.95848	66.03358	60.96817	64.9678	60.70135	70.82374	65.21774	57.18323
1700017B05Rik	55.97739	43.00868	50.96085	52.17786	44.0024	41.97816	74.81024	53.92712	57.17448
Sec24c	75.27619	69.32991	74.15703	63.802	62.55599	52.37315	76.73439	58.09953	57.08511
Tmem134	52.01085	54.76461	60.07744	51.45735	62.16031	61.75073	52.89945	62.35646	57.07301
Trap1	62.43721	57.86604	57.68932	63.07146	49.39669	51.64681	63.06023	50.00203	57.06991
Mrpl20	56.06033	54.44419	46.16604	43.25823	53.66062	59.89914	43.49971	56.71422	57.04282
Derl1	59.9683	55.09709	62.03791	52.58587	55.36718	48.28426	61.57941	56.12228	57.03751
Rsl1d1	58.96512	53.96759	52.67077	49.52184	47.3149	51.60192	48.16896	56.6285	56.94602
Dnaja2	55.74018	52.16438	56.7853	48.48068	50.42482	45.36535	53.0631	56.34205	56.92258
Emc3	74.83289	75.029	75.37961	66.07983	58.89283	61.29568	65.87222	59.5218	56.91219
Pnrc2	58.72307	72.88948	62.48046	56.22603	61.60299	50.9179	68.41508	54.69227	56.91034
Ddx46	65.03541	66.30893	63.81086	55.34487	52.51474	44.70736	65.06096	59.85797	56.9091
Btk	63.26719	63.75308	63.40577	50.79996	56.17272	51.10559	64.82472	55.82241	56.90181
Emc4	64.10026	61.0499	63.65851	53.46859	52.73749	56.31105	54.71164	55.16501	56.87359
Cdc123	59.66961	57.70884	63.19532	56.49862	50.85149	58.21442	58.3791	63.01303	56.87044
Prpf8	84.05747	70.89981	78.98304	64.3093	58.19068	54.04285	82.51954	57.84129	56.86324
BC004004	62.49942	58.97821	60.19542	60.51516	56.54024	57.08513	59.23536	57.76979	56.80204
Uqcrc2	65.24724	60.54924	61.48389	50.20647	59.39142	50.34119	55.34951	58.72821	56.79599
Cbfb	62.75364	61.98689	68.79448	48.51063	52.45501	46.541	52.20209	58.27826	56.7725
Med25	70.41285	62.9864	69.04638	60.97838	66.50925	58.62634	75.76596	59.14208	56.76745
Ivns1abp	59.01087	67.06914	64.33786	56.15389	61.60782	57.67351	82.22356	64.06465	56.75338
Atf7	80.0672	78.89663	81.45662	67.17921	66.3411	52.34933	87.71432	68.49154	56.72587
Rab5b	59.44773	62.62776	69.02667	54.07005	58.82561	52.54101	59.92867	54.11033	56.72543
Glrx3	54.97658	50.64791	48.9228	48.22411	53.50564	53.15999	46.88773	58.30333	56.71941
Lmf2	75.02348	73.26996	76.57008	62.55163	61.91311	57.66645	74.98929	56.96269	56.71343
Ube2v1	69.58367	65.91106	76.68128	56.77124	61.72956	61.61493	66.62864	57.68543	56.62448
Ube2d1	57.68198	55.466	62.30906	59.19646	54.72617	55.7838	65.43796	62.04035	56.6115
I830077J02Rik	57.83032	45.53943	68.55146	56.1101	64.68572	62.5212	57.13675	68.97371	56.60442
Ncoa3	60.27222	54.93034	60.1392	63.59121	55.5989	45.16137	81.5714	58.31821	56.58792
Ralbp1	60.00202	57.94724	59.26377	58.25809	59.57584	58.38211	68.19541	56.95044	56.5836
Ppp1r21	64.21313	62.18063	65.1176	51.12614	60.83267	56.3336	67.84565	60.88332	56.58014
Eif2ak3	62.50426	62.10867	61.64969	54.442	54.87772	44.83904	75.28898	47.0393	56.56066
Fkbp4	67.32847	67.52689	62.74116	55.6296	56.45315	64.36438	56.64571	56.72433	56.54275
Slc30a1	47.93083	40.78719	48.93398	61.60083	47.89873	40.58668	57.5693	52.48257	56.54002
Ccdc88c	44.78034	33.67449	41.06835	52.58934	43.62922	44.60014	44.38513	47.02671	56.50785
Vps52	68.79335	65.75597	68.20165	60.60233	64.99887	60.99659	77.9467	57.60918	56.47419
Samd1	51.7766	58.47654	54.04365	53.24596	59.35162	60.53915	54.12325	60.33953	56.42079
Itga6	59.92405	48.28556	57.9444	67.65006	52.16498	50.44999	82.35667	46.90971	56.37016
Pofut2	64.55699	61.42001	60.74159	46.9292	48.13501	48.65665	58.58908	62.15439	56.33767
Marchf2	62.13765	60.21386	61.75303	52.24453	58.69414	54.85429	58.43182	56.43807	56.23542
Arpc1a	52.68012	47.76985	53.7686	49.0871	54.86001	52.65561	53.65374	55.04268	56.23124
Rsrc2	45.74342	46.33408	48.19819	49.24184	46.99149	49.12043	50.6796	56.66889	56.21724
Tep1	67.10557	62.85532	68.72567	67.97524	63.91061	55.04272	87.01385	59.4631	56.12039
Ifi27	59.1195	73.79262	61.29217	61.58778	53.76108	62.28186	66.40003	46.66575	56.07094
Tpcn1	81.64807	83.83871	91.7457	78.13623	80.12409	66.30152	116.2042	73.47146	56.04625
Frmd4b	65.72704	54.76722	71.25873	79.86535	64.26329	54.77146	106.9744	63.24461	56.04598
Camkk2	69.16425	66.29258	75.8537	48.31683	54.48281	48.61965	57.44306	58.63531	56.02102
Alyref	58.13161	54.16708	54.5139	51.81598	46.68852	53.93945	51.78079	52.5027	55.9647
Ndst1	70.04175	69.77558	78.46147	64.26578	67.22071	53.33655	85.17271	56.14913	55.96004
Hsd3b7	59.41358	65.18718	65.68186	57.64889	52.41104	56.21754	64.12459	58.05774	55.82088

Gstp1	55.88514	49.27094	58.49992	50.16308	51.30832	57.26236	47.74834	62.64371	55.82015
Ssna1	47.67417	51.77082	52.0434	50.34215	49.31536	51.32672	37.40286	49.85011	55.77281
Mrpl36	49.96801	46.21842	56.30506	47.42678	57.65701	54.62079	47.00723	53.48467	55.7663
Flvcr1	61.96782	59.43079	58.08266	52.63346	47.89764	50.58755	62.03559	54.47052	55.76365
Ier3ip1	47.60356	50.09459	46.45566	44.33139	49.16255	51.45127	41.59654	51.15432	55.75596
Gpr132	61.94531	51.52953	45.44104	38.59613	54.20014	45.50485	72.46023	62.32754	55.75211
Tob2	53.8557	47.05366	42.43712	41.47677	45.30316	38.53397	73.882	51.85739	55.73459
Pgs1	61.49265	48.97866	52.0057	39.41928	49.00185	47.51162	42.82543	47.65842	55.73455
Ptpn12	66.45225	58.20233	69.57228	41.689	47.52041	41.25965	54.08166	53.79784	55.72004
Mrps12	40.11965	46.5604	47.66282	46.18139	51.33198	59.28728	47.07819	51.77929	55.69556
Srsf9	59.73189	53.14927	46.23556	51.23729	56.888	57.40006	53.64908	59.82316	55.65661
Ptprj	61.54799	54.68505	65.58052	59.75976	52.10736	40.44278	78.38411	53.83787	55.64214
Gtf2b	54.46469	49.24514	51.9488	45.97627	49.59613	53.35409	54.82866	51.77147	55.62053
Gm12033	53.3574	56.44332	57.43292	54.25394	59.49201	96.03548	53.71389	54.89553	55.61368
Abhd16a	45.52286	50.74696	47.67222	44.0354	48.8746	44.60727	47.67332	47.67963	55.5511
Lypla1	49.57331	51.0533	51.93448	43.1882	42.00139	38.24235	48.42051	49.02328	55.54419
Mrpl40	52.075	57.38679	48.50737	60.40514	49.14524	61.27789	48.87981	59.73675	55.54187
Rcbtb2	48.10467	55.92253	58.58344	65.4208	72.02684	62.59812	69.46252	65.8847	55.54111
Akirin1	59.25089	58.4166	58.48577	51.82453	55.47195	54.55542	64.30915	53.80694	55.535
Slc50a1	70.45917	69.63679	71.29489	67.02646	68.37753	63.8635	65.78416	62.84204	55.50657
Denr	63.11098	46.72577	52.54777	51.03465	50.23369	59.53783	57.14045	56.3142	55.50106
Slirp	40.48699	54.10285	38.77146	56.83793	50.15237	60.68884	42.25557	51.64872	55.48557
Rcn2	53.55607	61.18955	59.96181	45.66334	47.06297	49.62643	48.51713	47.61173	55.45108
Srp9	53.60078	57.24237	54.1263	50.20259	50.28801	63.82568	44.41834	55.48244	55.42597
Mrps16	45.68978	44.04638	49.51008	53.27308	52.47568	51.81612	36.1836	59.56187	55.42271
Mtm1	58.17397	52.63366	53.64436	40.09275	41.00463	41.28023	47.47549	55.73006	55.37634
Spag7	58.52639	54.93702	53.5204	57.14132	56.75247	58.24349	63.57724	64.25482	55.34657
Tarm1	59.2909	34.26301	49.98568	18.25168	29.81388	33.69989	13.60551	46.66867	55.33646
Myo1e	80.5339	62.47072	62.63199	57.47581	44.27643	54.76155	73.85545	46.71789	55.28279
Serpinb8	85.5357	83.80476	83.94402	51.80208	50.91454	46.43248	62.799	64.80091	55.26154
Pparg	47.94813	36.69362	38.20625	70.5221	46.94867	47.44189	57.07127	63.94168	55.25675
Ykt6	62.06555	58.95268	60.16938	52.14405	53.77952	52.09561	60.58408	49.798	55.22746
Hnrnpa3	64.61626	49.79388	55.79171	54.48023	53.33589	50.26411	60.86371	61.52101	55.2156
Rab11fip1	60.32575	46.94524	38.65441	31.48635	42.42609	31.12399	48.67722	52.02374	55.1666
Orc3	67.79207	64.69692	64.25717	65.47494	60.30511	52.98722	69.70037	61.42497	55.11633
Usp5	58.96466	55.97633	54.22769	54.0404	52.00931	50.289	62.29153	49.42481	55.10618
Ikzf1	57.71282	48.83809	59.54199	50.25081	48.13956	48.12326	59.99037	50.48859	55.05207
Taf9	56.11801	58.45509	56.44187	53.27942	46.49558	52.77648	56.6421	50.0635	55.04076
Snord104	44.04668	44.68682	26.58298	83.82404	55.69168	61.09949	51.01877	70.85866	55.0088
Jdp2	48.6461	47.4881	41.4678	29.38484	38.20194	42.5858	57.62878	53.35803	55.00648
Rheb	63.16513	67.54089	60.07399	53.78649	54.95082	56.55928	59.10623	52.53955	54.99119
Slc25a45	53.16867	59.08232	60.83863	62.83541	62.76325	60.23441	64.3346	64.62415	54.97224
Prkag1	58.65137	60.21567	53.0427	51.22588	52.41204	51.98682	50.16634	50.01292	54.93284
Cnih4	63.64411	61.09861	55.86053	46.02735	57.04842	51.23241	45.53354	62.64768	54.92783
Cetn3	43.62598	46.54662	39.85074	48.60201	42.34888	45.87848	40.91164	52.03272	54.88885
Clcn7	61.62417	60.86259	61.57559	66.68228	53.11707	49.66971	72.69171	55.91307	54.74958
Tmem126a	41.78155	45.49868	42.74616	48.81465	47.8773	48.6733	46.23514	56.43718	54.73701
Hsf1	67.12337	58.00309	63.80652	53.15749	53.02246	56.45312	64.69018	58.31328	54.72559
Srebf2	67.67811	49.07181	53.96437	42.52178	45.28896	39.10681	49.63737	51.78919	54.64104
Smim3	47.30871	44.0509	44.6139	33.35668	45.0563	39.92288	49.98788	50.51917	54.62755
Rars	61.49957	51.00942	59.45129	58.41293	48.61138	47.34595	60.8472	58.75177	54.59782
Polr3c	54.32683	51.47309	54.05531	49.75597	49.77062	48.95521	56.59614	52.48858	54.57315
Timm17a	57.25174	41.12116	55.68636	51.52049	46.4075	65.41204	52.04933	54.73951	54.45075
LOC115487677	58.79795	54.45274	47.04382	49.28067	58.39846	30.29436	51.22094	61.57955	54.43853

Cops4	61.65133	50.18472	53.30024	58.21675	48.08965	55.88024	54.85594	54.61548	54.3804
Ggta1	57.45243	49.14327	65.36253	63.27681	62.73524	54.6746	74.45182	56.35594	54.37986
Map3k20	75.00944	76.04031	63.44417	55.0006	48.38184	43.83106	64.15793	59.79043	54.37832
Drg1	58.01099	53.54554	52.32232	50.35163	47.73438	50.31564	52.86592	49.02629	54.37413
Cmpk2	30.93065	19.57138	36.45569	53.79025	31.7668	21.17459	26.31721	27.39365	54.35523
Llph	45.03746	43.53768	45.90156	48.67542	49.43621	52.89982	51.18359	56.14383	54.33794
Pml	47.92406	42.24477	60.60591	64.58441	54.8852	37.83521	60.46818	45.38118	54.29136
Larp1	75.407	58.16833	63.53176	56.42255	49.864	45.60774	61.37124	54.81662	54.27333
Gm9616	54.06768	51.62515	59.86761	53.70288	54.93428	55.93329	59.03626	59.36545	54.26223
Mrpl13	44.71722	54.72772	67.01308	56.53026	53.60469	59.29014	57.3709	49.62627	54.24908
Mpnd	51.78782	48.43198	45.9267	53.89599	55.62322	63.11223	55.20954	47.29011	54.17309
Mlec	69.49318	69.40106	72.07227	54.31247	56.42673	57.22791	64.48914	52.42775	54.16899
Skiv2l	60.16636	59.23392	61.22787	57.65045	58.69824	56.87404	61.33356	62.25665	54.14067
Tmem37	54.23881	59.47667	67.05678	58.37989	75.38208	71.88524	87.10198	66.38031	54.13625
Gm14681	58.15181	44.75435	56.51075	44.23695	50.9525	55.30066	52.69012	74.09847	54.13062
Zfp106	68.55724	63.37991	67.18236	55.89619	51.91724	42.5181	68.23549	52.02577	54.11371
Chmp1b	47.26155	49.4313	51.93675	53.34008	47.9342	51.16603	51.04484	53.74253	54.11185
Mrps17	53.63899	59.67649	61.44211	50.02558	53.17936	48.67937	60.39706	58.55555	54.04402
Rab20	34.91704	28.58161	33.08785	30.63134	34.27651	35.43238	46.62394	46.52519	54.0305
Mybbp1a	76.15785	58.32716	60.80195	53.02946	53.11145	46.74585	62.56033	59.10963	54.02949
Trim41	57.52444	66.82959	65.66082	57.36388	62.39835	59.78689	79.89795	58.40641	54.02534
Fgfr1op2	57.35253	55.04672	56.66665	51.48206	54.97107	52.32211	52.68806	55.71052	54.02148
Stt3b	69.51596	66.12382	68.00467	52.6223	51.21911	45.44873	66.48348	53.14408	53.98368
Pdxk	58.39824	51.7412	51.34113	57.3244	52.07306	50.83462	68.07372	55.33145	53.97837
Eno3	44.34282	55.21442	47.97056	55.73416	51.60029	58.98923	50.00768	49.91465	53.96392
Mertk	62.55444	70.00647	76.63436	72.83118	62.66872	52.8436	107.7552	61.89147	53.95201
Eps15	63.05851	61.78064	68.08124	59.21587	60.18093	48.47032	63.96969	57.32022	53.88566
Sumo1	48.45697	46.82625	46.36347	46.09841	48.19231	52.44886	48.85657	51.95706	53.8811
Rp9	42.2339	46.33361	41.31635	44.55578	51.45783	55.80154	45.8083	53.84551	53.85528
Ldlrap1	66.52152	52.97204	42.28396	62.55767	45.74166	43.28265	74.65259	39.4831	53.84627
Farsa	68.02882	53.20404	59.82272	50.58172	55.04928	54.74253	65.18599	59.27423	53.83433
Tpra1	60.4061	62.70629	64.82236	69.88273	66.02176	63.2247	82.05346	56.82338	53.80948
Sympk	73.9219	58.24216	66.34236	57.26788	53.68264	57.21395	66.90561	56.41999	53.76055
Rgs14	45.29638	44.54215	44.12498	40.43309	42.51229	46.52363	38.13382	46.30788	53.75689
Stk40	67.75236	51.28597	61.35732	36.61021	48.40248	40.53734	40.3063	55.59693	53.75644
Cln5	55.86852	57.39183	58.11817	46.40895	46.60604	43.49452	52.00608	54.89636	53.7227
Resf1	40.09798	39.47261	39.41886	35.01847	39.8397	32.08553	44.62815	48.25328	53.71848
Dapk3	52.71695	51.12921	49.05823	55.56046	56.42678	60.79614	58.83545	54.78599	53.70188
Phb	54.32869	51.63312	44.1337	55.40332	49.39414	49.52902	48.85778	55.56976	53.68914
Aldh3a2	57.62338	56.69568	62.31755	47.80769	56.63253	55.01671	58.42732	51.22199	53.65956
Smc1a	67.34738	61.78886	65.50887	54.06565	50.40259	55.76981	65.77205	51.11094	53.57916
Gm9493	49.12872	47.40382	46.27387	54.37465	47.11014	54.22362	38.34358	51.66114	53.559
Lmnb1	45.76614	43.89398	34.34322	27.26321	34.71002	38.24767	32.18604	36.20324	53.52505
Gpbp1	52.48658	48.46339	44.08176	42.68051	41.60557	42.45426	48.42214	50.92036	53.51524
Tnrc18	61.08699	53.95232	60.19665	58.19536	55.55777	42.6214	69.00139	49.60917	53.46457
Vamp3	57.14183	58.63666	55.31343	44.36267	46.13325	49.66755	50.66065	52.98212	53.46195
Pik3cg	62.46994	62.78288	69.95844	51.0499	52.81386	38.97269	61.38717	46.74322	53.43279
Ralgds	58.84244	49.70671	55.34769	52.57651	50.40146	43.01626	66.1862	47.35982	53.42175
Tial1	51.54074	58.1916	56.34834	55.68919	51.58824	55.89007	66.08389	57.83754	53.37573
Hnrnpa0	52.02235	45.50418	47.34936	48.74607	47.31764	53.69866	49.46713	53.11753	53.37011
Micu1	54.16849	50.97603	54.26884	45.63568	49.82057	47.78868	50.22688	49.13381	53.35278
Oat	71.46494	62.78227	61.64503	59.98796	57.4292	57.02456	67.24207	49.5004	53.35229
Ncor2	75.67779	64.94221	72.86876	62.98452	56.85605	44.3067	88.50734	60.06549	53.29144
Mapk6	54.60748	40.07737	44.11827	52.32199	42.5779	36.72232	63.59329	55.82013	53.17666

Pbxip1	53.90396	59.54738	65.207	48.02898	53.71766	53.8073	55.57178	57.90641	53.16756
Ipo5	75.22859	63.45532	61.80034	58.80677	50.46891	49.74414	70.23135	49.00226	53.142
Nap1l4	53.59993	56.56152	52.20902	51.1794	44.67574	42.51884	54.00502	45.64214	53.14106
Ebp	58.42784	59.98524	61.5838	47.44193	60.12922	51.06149	52.23553	55.81072	53.14054
Sh3gl1	57.74087	56.28286	57.00833	61.43185	58.02431	65.17531	68.78699	65.72094	53.10836
Mau2	52.06155	52.60309	53.0657	58.68361	52.65603	49.60616	67.9565	50.09029	53.05934
Rbbp7	69.15587	65.09916	63.26796	49.32166	48.74963	53.39042	63.24458	54.42855	53.0535
Pitpnm1	54.69753	52.59691	60.12055	53.38042	58.6897	50.78398	64.21639	56.01743	53.03114
Tlr1	52.39247	45.04921	60.98072	69.92233	61.69636	51.60993	71.27157	58.75821	53.01392
Rabgef1	40.25199	35.36199	26.11243	27.83973	35.69245	32.72736	46.32946	45.63771	53.00668
Dusp2	34.98286	30.00311	21.51987	34.75283	40.36581	35.96321	54.96076	45.83397	53.00068
Dennd1c	53.98656	54.4966	64.0843	55.91905	55.34043	46.59097	63.92793	54.58795	52.99359
Mlxip	69.58534	65.33	66.70504	68.94582	54.29347	44.9775	91.64653	55.19345	52.97702
Suds3	52.04002	51.0483	49.59824	45.29767	45.05873	49.68519	50.59318	44.50198	52.95064
Eloa	56.05377	55.8323	64.7416	55.35562	54.61528	52.50664	56.53686	57.77106	52.92945
Dnajb14	57.41478	63.50488	61.04587	64.92599	57.67627	44.75856	76.64498	55.03521	52.92303
Qpct	69.7615	66.27916	62.54906	50.88916	40.56545	37.43163	53.91167	45.4117	52.9203
Pttg1	64.55979	62.66253	69.12868	56.06587	57.59167	51.99458	45.51331	47.99087	52.91235
R3hdm4	59.54571	55.50289	49.75399	42.45001	59.55057	51.18297	55.53692	48.46601	52.8954
Utp3	52.57938	49.14709	50.44123	59.89915	58.82717	54.9181	55.85588	57.61033	52.86983
Wipf1	62.961	57.62581	71.86236	58.49636	55.74043	46.97111	72.47535	53.25681	52.81366
Rad23a	53.34486	44.75745	50.59153	52.28915	49.17399	44.87363	55.59349	52.69188	52.80717
Scamp4	57.4993	55.4132	62.09046	55.31108	52.0661	53.40115	57.55238	57.9084	52.77876
Timp1	44.41529	37.26448	41.09702	28.6921	47.85838	37.17635	30.91077	43.59955	52.76015
Uba3	49.50558	61.12555	55.11716	53.0933	42.55061	45.6677	58.01687	50.83478	52.75941
Wls	60.98446	55.23877	62.96153	57.91096	54.23026	51.4831	64.63772	54.58383	52.68919
Gm10709	56.62481	45.56814	50.61219	48.3841	47.30884	56.9115	56.68472	52.70176	52.67582
Pgrmc1	62.25665	59.96207	62.08599	50.67605	51.2457	46.79862	46.17545	46.00825	52.66787
Cmtm6	55.43806	49.52103	54.17032	42.9892	55.34015	45.61523	54.20178	51.43832	52.6569
Paics	69.27148	70.46672	70.79355	51.98702	55.98044	64.49456	66.41605	57.97622	52.63839
Emg1	55.49111	48.88842	52.52637	45.31535	50.37341	51.12021	53.40503	54.80285	52.63515
Mrpl24	57.01533	54.30002	58.94995	62.3307	49.84989	60.12708	58.32442	53.33866	52.61469
Nop56	59.38748	49.98025	49.91468	50.0075	42.01599	45.12612	50.76694	51.30623	52.61434
Ube2a	45.97852	45.24641	46.17694	46.71282	49.73426	42.2517	46.22416	46.71693	52.59207
1700037H04Rik	52.9798	48.23121	51.62952	52.80947	48.88311	50.95382	52.08511	48.62605	52.55463
Kmt5b	54.48517	52.13393	55.92101	54.89064	49.20091	45.85425	61.34298	43.74238	52.54548
Gas2l1	67.11619	61.22387	65.49327	52.14766	60.8569	52.75487	76.1772	54.94328	52.50139
Tor2a	57.52487	51.95262	52.49114	57.00687	58.42346	59.35074	55.01911	47.83411	52.49966
Slc38a6	58.25414	64.37705	58.86837	74.23547	54.27108	43.09728	94.62161	51.67845	52.46901
Bgn	58.02349	76.83162	65.8332	73.23793	50.35806	64.91471	40.50825	70.34032	52.46669
Api5	57.0978	55.68952	57.68244	46.53641	45.53015	44.20973	55.32221	52.3733	52.45605
Prkx	57.65767	52.66007	62.84842	54.63416	52.13955	45.32684	56.79871	52.83219	52.45163
Dennd1a	52.21759	44.1568	59.1796	66.90116	56.36834	47.92631	74.79369	55.52659	52.4376
Mical1	57.04401	51.5825	58.01872	55.08377	56.21375	54.46142	58.09381	48.55149	52.39709
Uso1	62.044	56.19475	56.23616	49.83231	45.08167	44.73252	59.00861	50.80929	52.37102
Pno1	46.05079	43.27848	46.83555	40.8613	41.32798	39.76908	42.68976	46.57673	52.36473
Gm13835	42.44678	40.34537	45.34614	45.91829	45.44845	50.73211	40.54731	46.72045	52.34703
Zfx	42.15554	46.1302	49.038	45.57271	40.41762	40.76998	60.41361	45.06914	52.34359
Gpcpd1	45.40479	45.33237	48.05838	47.78363	50.06844	40.66344	55.8417	49.82184	52.32801
Mef2d	66.9788	65.00768	70.09283	53.65732	51.31959	44.97208	74.30491	55.30412	52.32038
Slc6a8	69.37814	64.19116	58.07872	67.08088	45.78014	55.8409	75.28237	46.40642	52.31664
Jtb	51.16586	52.83423	55.59531	52.57856	52.22419	51.08146	54.64869	47.42287	52.31046
Mxd4	44.62317	53.55532	63.29797	62.04769	68.78597	64.44297	78.43849	64.15376	52.2548
Ppp5c	53.68143	57.75508	55.68272	52.69978	49.87795	49.17484	57.39032	49.04064	52.24149

Adh5	59.8773	50.96763	57.33228	61.24508	52.16609	60.3507	62.93387	51.85062	52.23773
Nsmce2	49.96348	47.25735	47.07434	51.87369	55.31568	60.46176	51.8999	57.9349	52.17768
Trappc8	57.71465	57.3769	58.89056	58.24795	46.50887	40.20971	64.20853	49.03321	52.17278
Mbd6	71.03998	63.16739	73.14074	65.03422	64.33222	56.46972	78.45001	60.98809	52.16389
Snord96a	31.67889	21.54174	25.92211	43.67245	26.74215	34.62213	44.6478	30.8469	52.1512
Pfdn1	38.70884	41.99406	47.25911	57.4309	50.88668	53.31427	44.79565	51.16518	52.15056
Pbx2	39.23254	43.39366	43.73113	49.16973	56.49382	49.08118	54.63081	47.42175	52.13221
Actr1b	62.43121	58.11223	57.15295	52.60604	49.64645	48.2807	55.33933	46.7088	52.09966
Med11	44.69236	42.44213	49.82678	47.78124	53.0312	51.88847	56.09904	48.42268	52.02272
Nfatc1	68.0931	65.66945	72.53401	51.34589	53.74078	45.55253	70.64319	50.00573	52.01698
Helz2	46.60535	26.8485	44.84402	48.79041	34.51576	25.97101	36.72805	32.76015	52.00993
Slc9a1	65.97734	57.73084	65.97026	62.76417	54.84822	50.69487	72.39567	56.31908	52.00852
Mff	45.896	51.50391	55.91494	43.0997	52.9586	51.54702	44.41464	52.84645	51.97279
Csnk1e	45.16399	47.67862	48.71316	48.14276	52.2195	46.90462	66.1831	55.45753	51.95811
Pet100	30.01927	32.58106	37.69111	36.64386	48.59419	42.37187	33.8601	44.50894	51.95161
Ddx58	36.90114	26.87014	45.74891	44.99505	39.87035	30.4836	36.46128	38.49886	51.93757
Ppp1r15b	49.02915	44.92103	46.27652	45.41016	40.31704	34.56053	53.27853	46.90726	51.9013
Sp140	36.49233	36.09779	41.42854	47.28149	48.40782	43.41925	37.31364	45.88166	51.89739
Aldh3b1	48.61807	47.0874	50.49724	48.49127	51.82292	50.94256	49.06936	50.30219	51.88889
Borcs8	61.56006	60.77429	69.56075	55.46573	59.97878	58.9208	54.48583	58.82709	51.86493
Ano6	72.58688	64.03858	71.4937	58.1723	55.21429	48.062	73.81061	55.1012	51.855
Sec62	51.17231	50.53752	51.47823	47.15748	47.70442	44.33311	48.30806	51.13252	51.85275
Cc2d1b	58.02426	54.90632	58.19609	60.84547	49.49705	49.02841	65.31688	50.08441	51.84096
Gmfg	35.81531	42.88911	39.75457	38.44882	51.5495	49.65799	38.63314	50.45312	51.82777
Gm9733	45.66015	25.5875	35.70698	41.39025	52.45687	49.94007	24.3841	70.46203	51.81143
Dync1h1	75.88754	62.74804	70.45126	62.62821	50.24632	41.2653	70.56195	48.98454	51.80986
Srp19	46.00613	51.36532	46.1472	50.40424	46.33611	54.78143	40.53641	48.2601	51.80264
Aopep	52.58216	54.38572	46.47258	56.53077	44.03084	50.80132	56.53403	48.91175	51.78065
Ube2j1	63.26367	59.72974	58.38548	49.40705	46.61581	44.77352	54.2255	54.66428	51.78065
Scaf1	59.34863	55.92475	59.12547	59.8908	56.05905	54.646	68.08071	56.35112	51.76698
Tbc1d10b	58.21457	59.92606	63.13064	47.98871	45.05559	46.78468	53.92101	48.88293	51.71164
Dynlt1-ps1	43.51927	49.58155	55.83662	45.0689	54.2922	50.10716	45.06038	48.52702	51.64164
Ppp1r11	47.87683	44.48924	49.46746	42.22394	49.18226	51.20632	39.7571	52.34235	51.64156
H1f0	56.21173	50.66083	61.51366	69.28806	64.97935	53.59264	53.19994	45.16319	51.63917
Inppl1	63.03487	62.12334	64.18418	70.14335	60.68843	53.99346	77.31257	58.25152	51.63806
Wbp1l	59.36415	56.13362	59.98726	52.27782	52.19815	45.35752	61.29657	49.87664	51.62196
Mrtfa	55.55893	52.86616	60.3738	58.73325	52.26141	52.38513	75.20363	47.43773	51.59582
Entr1	56.81636	59.20052	57.06802	48.82289	50.20243	45.60248	61.69824	53.30738	51.58215
Trim12a	39.97601	32.58534	43.12782	43.26757	49.50563	45.84245	42.6824	43.7573	51.57848
Phactr2	61.04483	60.7627	65.33996	62.31289	56.48223	42.97699	68.51589	50.67003	51.55147
Mgat4b	58.03993	58.43172	60.1695	60.8922	56.23301	51.77394	63.14811	52.93458	51.51048
Tomm7	43.94054	45.11982	45.04554	52.04555	54.3221	58.84886	40.14477	54.06752	51.50336
Rab29	50.58898	53.94192	53.71915	51.83564	54.48603	56.22596	52.67176	50.2965	51.4598
Wdr81	57.47755	51.61894	63.20571	64.93619	55.41288	48.49169	79.1195	51.86653	51.45595
Ppp1cb	47.75429	50.53492	47.48693	37.80377	41.95164	38.54351	41.13284	49.19982	51.45487
Tpr	65.85748	59.05462	65.06468	52.3748	47.63763	40.82378	64.05993	51.4332	51.43073
Brd7	53.41754	51.48689	57.08637	53.06809	42.59969	44.30717	50.98087	46.72271	51.41717
Pmm2	61.08647	54.30687	64.94928	55.21668	59.57276	57.7069	63.22858	58.67248	51.41321
Sh2b3	60.62292	57.37535	66.90203	56.96009	57.00311	47.57908	68.32833	56.77618	51.40865
Hmgcl	44.75803	51.56429	48.19784	58.65509	52.65484	52.03517	55.34777	43.9794	51.3627
Samd9l	39.53273	33.08443	43.6777	44.52649	38.31448	31.30197	33.80904	38.33341	51.35521
Pdpk1	64.75091	62.38942	64.47619	50.51278	48.40867	40.77033	68.05758	48.63272	51.34313
Crlf3	45.87885	47.8843	50.42128	51.28766	52.10422	42.45229	56.76773	54.37648	51.2932
Eif5b	50.05314	49.30312	48.29866	50.03767	45.07861	50.44915	49.75595	51.74983	51.26869

Ssh3	48.67978	54.16374	55.56556	60.92514	62.65511	59.81195	72.31058	53.26266	51.24151
Hmgb2	32.38012	36.11982	31.04315	38.26933	41.97568	57.14177	34.97992	35.69886	51.20589
Clip1	52.66694	44.3448	47.19281	53.04137	42.46553	40.84306	52.11999	45.80642	51.15126
Snord89	14.74417	46.46752	28.31209	26.39084	25.27593	35.99625	34.74453	25.82972	51.1511
Gm10177	39.07929	26.93306	34.5392	50.22156	47.23435	45.8645	34.75453	40.0504	51.10502
Rassf2	56.63233	54.96888	60.96268	50.15156	51.05837	42.86271	67.23443	49.68966	51.0845
Zfp672	62.73906	62.69545	70.41947	46.75995	51.14865	56.30257	63.94867	55.49228	51.06829
AU020206	42.69641	50.87039	54.57386	60.91178	65.23511	61.07442	71.85767	62.16351	51.06484
Dnajc5	60.58945	56.17813	58.06096	53.9877	51.21888	47.35156	66.28784	50.9978	51.03802
Ndufb3	51.07694	54.70071	46.94302	53.80446	47.72994	61.24744	49.73467	50.38966	51.0057
Inpp5f	58.59652	52.23445	49.50402	54.09623	51.4068	40.97903	60.75732	53.72501	50.99528
Uvrag	48.59434	45.75831	51.33386	48.59592	45.10076	42.68342	56.38335	49.78666	50.98181
Nfya	57.04708	55.1608	56.43604	52.46085	48.49546	48.35156	63.2109	52.14803	50.9166
Zfas1	29.82237	38.58346	33.13147	36.82638	38.4185	40.43038	32.3757	47.56144	50.90432
Cxcr2	36.39106	37.43876	29.74936	19.12437	35.87484	29.90455	13.5449	31.28176	50.89679
Mrpl39	57.21655	46.87781	54.37729	52.24735	43.79719	45.72242	44.06327	46.15442	50.85176
Polr2g	50.32856	46.50005	50.9093	48.90345	52.43364	47.25105	51.22494	52.47842	50.8286
Tuba4a	46.45886	36.16127	41.51944	37.75006	32.33948	46.27466	39.76499	44.26044	50.80623
Polr2a	80.44939	64.60678	68.49076	59.99701	54.06057	45.23951	74.32749	59.12169	50.77442
Thrap3	58.46103	52.78656	57.13551	53.22965	49.12661	45.98429	61.81247	49.3921	50.74978
Adap1	54.09775	54.27639	63.76465	55.03561	53.39284	52.84879	57.36966	55.81873	50.74505
B430306N03Rik	39.88977	27.65343	45.87774	43.94839	34.62511	33.02817	35.29951	38.91788	50.73831
Ccpg1	46.90757	49.67251	53.94491	41.44639	48.66889	45.10256	45.71694	44.17831	50.71309
Nsd1	58.86891	57.33263	59.13718	56.71962	44.45871	38.375	70.76135	47.32793	50.70937
Parvg	49.4662	42.94122	53.79474	48.33781	49.61677	47.57922	57.42749	47.01715	50.68808
Gfus	48.70563	47.64983	47.31822	53.46539	50.41073	52.59845	55.42646	49.64381	50.66903
Ntpcr	42.15718	40.78084	54.12177	48.77087	62.03574	54.22316	55.4754	54.09416	50.63146
Akirin2	46.87564	50.94829	55.98879	42.75269	52.41851	46.3688	49.87326	51.02075	50.62431
Nras	62.77696	56.84615	60.61046	51.82191	49.92488	43.6293	59.78233	50.49393	50.60741
Dnajb9	51.21704	56.2688	51.46083	41.29392	52.77029	51.15743	60.06306	52.58436	50.59925
Gripap1	58.62681	57.97261	52.71647	51.03976	42.95604	42.47412	54.55818	45.39366	50.56718
Rab6a	60.4575	54.79158	50.14847	39.17588	41.10209	42.19111	49.66778	47.05096	50.55227
Antxr2	57.87058	50.87421	59.47975	41.35289	46.8057	40.86843	50.05672	47.85925	50.53288
Kmt5c	49.24998	64.86535	63.51796	54.21591	64.8038	55.38856	77.35362	57.63157	50.53168
Eif2s1	50.0033	43.99381	42.60361	43.47939	39.06293	40.41861	41.10114	48.49557	50.52409
Gls	45.40549	50.4812	46.34941	40.25951	40.70085	33.74429	43.36653	46.41961	50.52403
Zdhhc20	54.49126	61.57743	59.83023	43.3835	45.70342	39.0093	56.62846	44.69311	50.51018
Mir3064	47.16373	47.03816	41.3765	50.19073	56.7388	32.13776	54.73242	46.48008	50.47154
Pfkfb3	46.76604	32.47923	32.47801	46.08724	36.71699	31.82406	64.86643	44.70047	50.41527
Yipf1	49.67536	48.65205	48.60888	40.73484	44.45974	50.48247	42.06163	46.70036	50.41054
Atn1	63.78656	62.27096	63.06245	49.43419	54.0357	45.76279	66.0048	54.84766	50.39745
Vipas39	56.65747	52.34014	52.82472	55.64399	47.44347	45.00253	57.85815	47.27847	50.38385
Vegfa	36.18085	29.18632	18.16612	26.738	28.49162	26.51828	65.33754	47.91856	50.37286
Yeats4	48.78392	49.12527	52.9243	41.69802	49.61119	54.65365	51.23451	48.39643	50.36065
Cr1l	58.8404	59.38237	62.01645	40.79835	51.49611	47.7817	56.09137	54.27511	50.35766
Wtap	43.68011	45.82828	43.25266	41.7421	45.46189	40.09404	51.76655	50.72367	50.33851
Dynlt3	49.79898	60.01571	53.71911	45.09037	42.76859	43.17128	48.80843	50.37992	50.33419
Plekhm1	54.60455	46.46949	49.90561	63.2226	44.42809	45.57196	76.59516	55.76284	50.33058
Acaa2	48.34199	51.6413	57.81014	50.46006	48.60991	50.68635	53.47814	49.26364	50.26598
Fip1l1	52.94348	56.00509	55.89571	50.4566	44.53382	48.51873	56.0363	57.05257	50.2322
Frg1	41.68046	39.99412	44.38618	48.79394	47.70315	53.76478	44.1321	51.56964	50.21495
Dcaf8	51.55655	56.66502	60.95801	56.86162	55.90598	48.9004	67.0657	53.74653	50.21027
Appl1	55.08674	54.55467	57.48239	46.32389	45.43758	40.4486	52.7955	52.38108	50.19218
Xrn2	53.70297	45.78981	51.48054	45.1474	45.44341	37.81736	52.51585	47.32445	50.16229

Thap4	48.71356	58.95571	55.75731	54.661	48.25599	50.68891	62.0661	48.9857	50.15454
Isca2	51.33926	43.92752	51.90106	44.62704	52.00464	48.89033	52.84628	47.42485	50.1227
Arhgef6	62.96587	63.8028	66.07479	53.08479	53.9346	48.71811	66.84539	55.81087	50.12163
Igtp	34.91255	21.50944	32.31294	46.05315	37.2435	31.47508	26.00078	35.97975	50.09497
Bcl10	42.87323	43.2389	42.28387	36.5547	41.39799	37.05698	42.23498	45.58853	50.05932
Ddx47	52.29532	53.25312	52.67857	47.40842	50.5837	46.9951	49.89214	54.1888	50.0218
Cav1	83.48387	80.37098	70.0232	73.38092	50.74187	55.57689	36.32887	46.18128	49.96668
Hip1	66.65502	56.2723	58.93	60.42949	49.28098	46.66894	68.02235	52.0299	49.96096
Mthfsl	41.52023	39.93632	37.55918	47.55039	46.50424	48.67885	43.63649	52.56038	49.94727
Got1	55.27644	49.89038	53.28923	40.87687	41.84379	41.52708	73.47397	54.06484	49.93622
Glg1	69.8326	69.83702	71.65388	52.32167	50.92268	41.31518	63.65193	51.78315	49.88827
Cops2	51.73392	53.0633	56.08261	43.74249	44.25765	42.81023	45.75781	51.70252	49.86445
Ap2m1-ps	41.17756	46.01238	41.70733	40.56991	41.41121	48.97505	57.47885	41.2875	49.86338
Fli1	49.72838	49.92686	51.8297	48.30387	39.74446	44.2272	52.56979	52.33361	49.80861
Uba7	35.11779	30.89762	43.11766	54.88344	47.68906	40.84204	48.47317	41.41471	49.79616
Man2a2	63.19108	61.97061	70.29686	66.62037	64.00384	52.52394	80.72608	54.50231	49.6379
Poldip3	53.10946	51.41979	52.42559	44.49309	47.8703	45.30161	54.08903	49.41582	49.63164
Etfa	49.00058	47.09509	48.21097	46.05257	42.84477	51.65259	52.4449	43.48064	49.62529
Rab24	40.13716	45.20549	46.08547	56.49469	49.58845	49.05263	51.98075	46.11077	49.62488
Dpp9	65.04375	59.57631	62.76021	48.67058	48.49527	45.54016	62.00072	53.83541	49.61506
Abl1	56.40424	52.50186	66.60075	66.72949	61.35903	52.9596	89.93381	53.4933	49.61441
Ada	42.32583	37.24455	42.21117	50.96621	50.29342	53.84734	56.84489	48.38077	49.57067
Hba-a2	27.86674	30.34592	18.41585	92.62991	81.02663	119.7453	42.94806	27.93815	49.55266
Steap3	68.25924	61.39383	59.07659	54.10997	50.4864	54.90416	61.07225	45.96258	49.54821
Tm9sf2	59.65657	60.2742	66.23645	47.92602	48.07397	45.55799	53.31222	49.69688	49.48976
Sec23b	63.08627	49.26127	57.82626	48.82008	38.0778	40.6158	48.68319	45.89306	49.47641
Ube3b	57.27462	56.32788	60.12584	53.39822	53.43324	45.88664	67.88075	51.18863	49.41404
Macir	51.71978	45.73668	56.0748	37.87276	41.7147	38.19313	40.40606	47.26221	49.41368
Rpsa-ps10	42.54925	51.90205	49.29599	44.50958	49.25473	47.77641	43.14072	46.46706	49.39372
Med21	39.87276	46.14628	42.22312	41.34564	46.63877	43.51155	30.99969	40.66957	49.38265
Sptan1	64.76658	56.65455	57.43669	52.49809	47.20078	41.60935	57.60247	46.85245	49.35457
Sh3bp5	59.29699	53.66428	46.83792	44.33622	34.90574	38.55106	55.31317	52.28224	49.33217
LOC115487644	59.13393	50.68293	21.25373	41.34564	51.91863	36.74163	60.16291	46.62451	49.31491
Mrpl28	48.46276	48.33374	45.08445	49.38184	52.99691	50.06392	52.07931	50.55222	49.31491
Cul1	50.67098	51.69255	51.74452	42.70004	39.77425	39.48882	51.1535	46.84181	49.29385
Chmp2b	50.0198	49.09583	53.32982	45.79724	46.82022	49.55643	51.97113	53.56886	49.24773
Niban1	57.04252	51.40231	65.98727	42.92361	47.5174	43.39311	51.18855	46.21649	49.20223
Syne1	44.56853	46.35013	43.63242	45.49448	33.46098	36.04638	56.38456	28.72984	49.19457
Hspbp1	48.52669	48.10418	46.14248	50.30204	49.57747	48.24166	49.09086	44.45208	49.19344
Spn	61.50219	67.79709	84.38347	42.50887	41.88951	48.6019	39.69463	40.73623	49.12701
Uckl1	41.99984	44.5599	42.91223	48.46754	53.04552	49.62907	50.91171	46.20009	49.08977
Cyb5r1	48.01019	42.35015	45.49702	45.52221	46.05836	43.39597	61.02122	50.23288	49.06981
Zdhhc9	64.43169	70.57575	65.3911	48.17381	55.12959	46.45321	62.69855	60.25413	49.04214
Ddx39a	53.76724	46.19204	46.68835	42.53359	42.00974	47.62088	45.27889	47.8192	48.98368
Mrps24	44.84373	44.44482	47.82019	49.8879	49.54207	55.1552	46.81172	50.00725	48.93737
Acta2	34.00704	36.78202	40.27617	58.59349	58.45079	55.23113	21.17641	58.65715	48.93183
Pin4	43.19696	42.02545	46.38033	51.73326	45.96057	51.81162	35.52233	53.0066	48.91105
LOC118567924	29.32058	34.71013	27.96867	45.26582	41.5349	36.0011	61.30887	63.74949	48.90395
Map3k3	52.98004	51.10854	55.43246	47.43531	49.90679	43.93551	67.70903	50.56838	48.89842
Rfc2	53.39292	45.78401	47.48212	50.29751	48.34655	53.19244	56.12789	46.94093	48.86937
Mta1	62.14452	61.36571	66.54512	54.51597	60.63611	50.67157	69.19347	58.33118	48.84598
Srp68	58.46675	53.56128	57.20047	52.02851	48.93172	47.61413	60.06537	50.37947	48.82125
Agfg1	64.64169	62.82206	61.5023	49.45637	41.63579	43.68367	55.59065	50.92881	48.80759
Smarca2	56.94418	65.67741	61.30945	53.7573	48.46485	51.77117	65.54494	45.67362	48.80587

Limd1	62.64701	58.26055	59.82123	52.2926	48.44051	43.72757	60.43222	46.78328	48.77032
Tgfbr1	53.98333	66.09787	75.69432	52.29341	59.4432	45.52017	71.86287	49.35283	48.74396
Dr1	46.65437	45.7044	52.38426	42.52695	44.02169	44.47612	45.31751	53.63933	48.66873
Mrpl48	51.76776	47.8055	48.48655	52.51278	58.43324	64.28687	50.33392	51.00915	48.66053
Gtpbp2	45.73486	56.5438	57.15461	53.46817	54.21128	48.48588	60.78997	50.01683	48.63554
Cpsf3	60.16644	50.85707	54.87817	50.5101	48.91556	51.37212	59.72688	51.19199	48.62768
Gm10039	51.37261	46.14911	48.56939	59.0232	47.37795	47.23435	51.7974	52.92322	48.61041
Tgfbr2	58.97866	49.74148	67.38513	54.91698	58.39998	51.68074	73.92938	51.71853	48.59964
St3gal1	82.93359	70.90376	74.18099	48.56872	52.86483	45.53528	62.18492	48.83868	48.58655
Eif1a	48.92953	48.62642	43.46587	41.37915	43.77571	45.05262	52.04386	51.04078	48.56122
Cox5b-ps	44.73573	41.20101	55.76561	44.48515	37.71329	37.80722	52.20976	44.63074	48.55329
Stx2	53.53922	65.62398	60.68758	55.74325	49.88937	57.71875	58.91924	52.07503	48.50969
Cdc5l	47.80931	48.29144	48.25931	38.61136	41.47706	39.93171	45.87285	44.5319	48.4784
Trappc6b	43.09221	49.25192	52.08567	43.55639	40.7252	42.64991	48.93797	47.87352	48.46434
Acot13	41.21489	47.79482	48.57456	44.19845	49.56604	53.65887	49.27963	49.9356	48.44712
Lpgat1	60.04786	57.27372	65.93075	47.39769	42.43975	36.94595	60.97255	47.07393	48.44385
Fnip2	49.72626	39.46314	40.65149	53.28606	39.42593	35.42833	66.57713	47.85759	48.39146
Sar1a	55.22791	52.41906	53.65801	45.95354	45.89356	43.33824	52.54127	49.86123	48.39089
Fndc3a	46.67434	41.45151	48.06564	48.37741	41.14385	31.86353	53.39814	39.12553	48.37553
Mtfr1l	54.27888	52.42912	44.11215	51.15074	45.48463	42.87915	62.62944	41.52515	48.36293
Rarg	59.22905	63.99059	62.88467	42.88597	56.00146	56.18679	51.12583	57.73083	48.3501
Gabarapl1	44.40048	53.35702	50.31597	44.68035	47.22989	49.50353	52.77363	47.0666	48.33743
Map4k1	57.10182	53.93843	59.69683	52.28843	58.21736	57.32678	54.96278	57.80844	48.33477
Slc8b1	52.0186	53.3082	58.69337	50.15457	50.78355	52.5718	69.5793	53.59639	48.29509
Sp3	44.15241	43.12846	49.19055	49.94794	41.01678	41.84683	50.03586	48.28815	48.28669
Taok2	53.65553	53.33353	55.04374	56.40541	51.46943	47.3955	62.4394	44.41516	48.28063
Klf7	58.28421	50.68293	47.9364	39.78698	43.331	40.05264	48.30256	43.19994	48.27703
Gm14548	71.38891	81.73374	91.07221	52.34295	55.56682	49.39894	63.81913	68.81295	48.27663
3830406C13Rik	57.68252	53.5743	59.78429	52.30047	61.42024	53.61973	52.96779	65.3192	48.27384
Nde1	61.00203	63.49357	65.80759	52.76205	46.11718	51.60295	57.75639	49.71149	48.26456
Atp2c1	51.25491	48.53898	56.95152	43.31928	42.32456	40.49395	51.27765	47.17232	48.2503
Ppp4r1	51.76446	47.62974	49.34895	46.65914	43.41572	43.02137	51.87886	45.10357	48.20617
Mpc2	43.19492	47.19584	42.64962	41.51663	42.12097	42.36238	48.41184	46.1028	48.18299
Nudt21	43.21275	44.64046	47.75913	41.92868	43.43648	48.17565	39.86621	44.61093	48.17667
Ten1	45.60426	41.5598	39.09391	49.32141	57.07166	54.56396	49.99283	49.85661	48.15115
Apex1	46.02165	48.90267	44.28773	45.17336	43.18417	44.48142	50.28371	44.6652	48.14793
Zranb1	46.45588	51.6355	46.31754	36.97796	44.58625	40.2032	53.02758	44.12792	48.14607
Rras	58.90294	68.68871	70.39993	59.41925	68.60345	55.2586	54.03101	64.12607	48.1446
Tspan3	60.27968	65.52353	60.57529	54.29854	51.02745	44.83302	55.47534	42.15881	48.13123
S1pr2	44.29137	40.06841	48.33422	53.93293	49.62881	43.80038	53.69525	46.42607	48.12614
Baz1a	51.66238	44.70538	45.46311	39.78889	35.82762	31.62695	43.04489	40.66091	48.08686
Bad	41.77002	51.0012	50.55408	44.87533	51.00311	61.50997	46.82483	52.95074	48.06463
Fam76b	56.80796	59.47462	62.80856	66.66386	54.22559	48.49617	69.02308	54.24978	48.06367
Pnkp	39.5189	36.89305	41.55876	44.81355	43.0454	50.41748	38.91108	41.78222	48.05106
Klf13	52.04036	49.34303	51.78635	45.10356	44.28776	39.34026	61.80409	51.61063	48.02123
Snx10	44.38317	40.74525	46.39854	39.81174	33.81159	34.62494	38.48049	43.9483	48.00362
Spcs3	60.86031	58.40116	55.52449	46.17288	46.90332	44.77391	53.36352	49.69106	47.98965
AI837181	47.94741	49.04683	60.00456	45.62774	61.93152	55.55082	52.3611	52.78247	47.97443
Utp11	53.053	53.95607	46.25224	55.93737	54.23907	58.31463	45.27324	50.45627	47.95839
Eftud2	57.21941	50.34256	51.48181	43.22461	41.92177	43.33864	52.29088	48.99418	47.95034
Uba2	52.25323	50.99692	52.3252	46.78414	39.22242	42.61449	49.23905	48.41181	47.93884
Cklf	41.55427	45.60738	38.35478	51.55534	44.66152	45.16199	46.86194	48.43785	47.93305
Cherp	64.73123	62.15813	68.05084	60.11802	55.09118	50.06665	71.20031	54.94933	47.91518
Rpf1	49.29037	48.39951	47.93583	45.98469	48.29077	47.34309	44.02781	56.97813	47.91144

Top1	52.05154	46.42272	48.06536	42.1732	42.46397	40.63277	47.73593	49.00935	47.90664
Trabd	49.30409	46.62382	47.40436	44.82288	52.73222	57.15475	52.27903	48.44661	47.89912
Havcr2	48.43964	56.91368	65.0481	37.42603	42.7814	38.9444	50.69559	47.30084	47.89298
Susd6	51.18924	47.31133	57.25272	40.36243	47.9234	42.59394	52.93	50.68451	47.88322
Rbm22	51.83819	44.59938	49.84405	46.50971	47.5162	46.87797	48.03772	48.89804	47.88151
Med10	48.87811	38.80596	49.65564	47.90083	50.2873	52.33793	46.79559	49.72084	47.8724
Polr2i	40.89561	35.0173	41.86462	37.55063	45.75879	51.78749	41.18813	42.45546	47.85996
Mpv17l2	61.06894	55.69574	60.03101	48.89321	55.83936	53.46839	62.93781	61.07291	47.85796
Aimp1	36.4152	45.28694	32.07084	41.66501	47.38698	43.45391	40.13924	48.31337	47.84563
Mindy3	48.13926	45.59828	47.93981	38.20598	36.55288	41.18075	44.49919	41.38271	47.83504
Brpf1	53.33579	48.80312	52.40205	50.22102	41.78663	39.57984	57.05516	46.38923	47.82828
Ccnk	56.3859	52.55956	60.79515	50.56149	52.97915	47.83917	64.96096	55.75048	47.81865
Gid8	51.69005	50.21974	52.80429	45.5669	52.89172	49.63985	44.93565	51.23096	47.74314
Mtmr6	52.08606	51.35659	51.96207	41.20556	44.22588	37.35825	47.30781	43.35948	47.72854
Man1a	62.96921	61.65687	65.90128	49.59288	55.47896	41.07497	67.84435	47.3186	47.72749
Dhrs9	66.73428	63.25921	56.62543	40.33292	30.73827	29.31905	44.36171	38.65468	47.69808
Slc25a22	42.69994	34.55112	41.70315	44.36618	38.448	34.01045	40.50919	38.83015	47.62837
Pcyt1a	57.96271	56.97679	59.41724	48.19484	42.01167	37.8267	60.21856	47.18115	47.62761
Cul3	51.69166	49.41686	51.44982	39.05168	40.96994	39.79525	48.71008	48.31338	47.62729
Mrpl27	39.2623	49.38272	44.43156	47.65133	55.04074	62.36361	46.93727	55.31314	47.62604
Adpgk	27.62918	23.81433	23.72342	31.18234	26.63463	41.2341	25.72374	21.68615	47.6136
Rnf150	63.47784	59.28569	60.26333	58.97169	55.51991	47.12247	82.7477	47.60824	47.60439
Gm6563	38.35715	48.29697	43.87608	43.74444	41.95349	46.99972	33.07798	48.84518	47.58222
Usp19	51.26556	54.92083	56.61629	51.52527	50.12281	44.44574	63.34043	44.23392	47.53885
Sh3pxd2b	62.93415	58.03894	65.90729	46.3107	46.08021	39.16903	68.55173	43.7917	47.52011
Erap1	53.84575	50.14293	57.25666	42.96999	44.83262	35.56051	51.83114	44.31358	47.51703
Lpcat2	42.8837	43.24062	60.14361	41.50633	58.40994	39.8874	56.78412	43.93016	47.50627
Nol12	55.54306	44.97587	41.11084	54.7621	50.34867	47.28639	49.8102	49.33361	47.48361
Akap13	61.99552	57.14316	63.47043	47.80018	50.58122	39.28228	65.59223	48.18987	47.4433
Ssr1	56.62852	49.52862	51.56923	44.8564	44.02848	42.84874	51.17013	48.1265	47.42017
Cgrrf1	39.04864	56.60691	55.22449	51.4086	45.98926	51.77599	55.36533	55.92428	47.38592
Nosip	49.20368	47.54215	54.45328	50.89917	54.5951	53.36657	57.92584	58.35294	47.38302
Elf4	49.1803	44.54821	53.51006	44.8412	47.34446	37.05421	58.01337	47.2668	47.37021
Gemin7	51.87782	49.13098	48.54119	54.62177	55.35896	49.99074	49.33786	48.77017	47.36668
Psmg4	42.33241	36.32052	38.85755	40.13468	45.17705	44.9394	35.72135	47.48214	47.31047
Cd86	51.66612	48.49376	56.20532	33.31606	43.41017	34.79382	38.52627	52.66175	47.30896
Srd5a3	52.428	51.66162	57.19782	48.54906	51.88502	50.80855	52.37503	47.01923	47.25732
Nmi	30.98913	26.44137	35.82163	36.96992	35.57531	37.22264	29.07999	39.15849	47.23153
Prg2	0.264621	0.263916	0.727792	0.657846	5.04044	10.7062	0.546998	1.826601	47.22579
Kmt2e	59.77026	63.10358	66.19119	50.2923	50.09245	40.22005	67.29749	52.30618	47.16365
Nfyc	56.03282	48.17233	49.87188	46.72299	46.26958	44.52244	49.95377	51.53071	47.10912
Slc66a1	53.95247	45.28037	50.00877	52.78551	57.35378	54.14908	66.17358	46.77976	47.07018
Acer3	42.89874	45.8617	46.32573	40.33769	42.9283	36.90959	42.96839	45.17486	47.06921
Csf2rb2	59.73442	58.98653	69.08891	49.08136	52.28414	45.40062	61.28407	49.25407	47.05438
Cyfip2	48.2489	40.14965	43.41176	38.20111	33.15206	34.06402	45.74393	32.50685	46.99797
Vps29	40.93639	38.72955	37.4215	39.07799	36.09799	40.75579	38.58888	41.42105	46.97729
Fpr2	34.22103	24.78685	26.35325	13.44159	16.82548	25.39634	12.73289	30.3825	46.92581
Vps35l	55.0816	50.06593	56.25052	49.27109	50.24502	45.52005	62.40789	45.67004	46.91406
Lsm2	64.53519	57.61686	57.3108	44.84427	47.83716	46.73745	50.22616	45.23154	46.90894
Fabp3	34.15963	33.58083	40.93109	50.91189	39.98718	56.01716	42.55219	40.90641	46.85434
Strap	51.49617	46.90489	51.46022	41.42904	40.55748	43.87976	45.68237	47.27265	46.80226
Snord14d	29.9493	40.67345	16.56962	44.35548	41.87476	37.71394	77.71454	40.04187	46.76413
Braf	55.98763	46.48647	51.83752	44.81246	42.1342	33.09827	44.83729	42.15403	46.71559
Cox14	39.10569	46.03716	50.3065	52.00134	47.61368	50.6292	42.00269	51.24937	46.69861

Dctpp1	30.86693	40.02891	34.28115	41.04131	37.80846	45.0409	41.57268	36.79787	46.67026
Uchl5	42.89531	40.19911	36.2041	38.66029	38.98254	40.70019	34.09277	41.22302	46.66878
Parp3	55.12376	48.37012	54.11461	53.6129	44.1178	45.74491	51.37579	47.44207	46.6619
Flcn	38.21112	37.79088	38.14041	47.5991	47.34162	42.52882	67.16157	50.83286	46.58858
Cttnbp2nl	60.61458	54.04272	64.36384	48.7131	45.26688	41.33234	70.0388	51.44925	46.54723
Bloc1s2	37.28796	39.52838	42.29341	44.32302	41.50982	43.56015	37.45426	41.73224	46.54163
Ncoa6	69.696	57.47598	65.819	53.17486	46.6731	36.3451	71.56368	47.75621	46.53109
Dck	40.33178	32.75468	49.67697	38.08614	41.76219	35.63729	34.86238	39.50484	46.52871
Tbc1d9b	55.21528	55.0341	58.20388	56.18406	55.70375	49.65092	65.6226	48.03449	46.51651
Stard3nl	41.52113	38.24983	47.63542	37.58902	39.58802	41.32822	50.27223	47.70975	46.48991
Sub1	36.02389	35.71155	36.06147	37.05048	41.33585	40.75456	34.33394	46.2979	46.48927
Psme3ip1	43.42982	44.87777	46.43728	45.74059	48.06516	45.06718	46.128	49.13025	46.47911
Nipsnap3b	54.88447	42.07912	47.49561	45.41533	42.06565	37.22553	46.38605	45.51641	46.46827
Ppp3cb	48.69843	46.00565	48.20577	44.5662	41.01008	39.23871	48.65181	41.82149	46.46392
Fam126a	61.76102	55.77869	64.17528	47.42022	41.10443	39.57284	61.55636	43.88829	46.46114
B4galt6	54.42848	46.43868	52.43761	52.63426	41.94726	38.4531	60.47422	45.86381	46.45081
Cabin1	52.3194	44.83741	54.54725	52.1113	51.72717	46.28946	65.68739	48.89124	46.44479
Rpl31-ps8	48.04632	39.87616	35.95492	45.43844	37.22568	49.50669	35.30483	51.82279	46.43287
Ip6k1	39.6058	43.27244	47.33895	47.74304	51.08732	44.81829	59.01227	46.15475	46.43033
Oga	47.7517	51.29024	52.36669	45.96136	41.71381	41.62356	55.51922	41.42334	46.40127
Pigk	50.62871	50.97729	54.6615	50.45633	46.01681	42.26887	54.67779	43.94398	46.37041
Cnot3	57.44109	49.81761	62.14292	53.23945	51.76529	50.4184	65.08294	50.97471	46.35453
Rnf128	56.51556	62.66534	54.81061	46.32477	31.34917	33.6833	52.86527	30.63556	46.35354
Rps6kb1	42.63337	46.39862	44.72988	43.27018	41.44219	37.15274	54.30228	44.08437	46.29829
Apaf1	51.31215	45.86999	54.02017	44.61018	41.92772	32.36411	55.11172	42.33365	46.29011
Traf1	46.19032	49.01295	45.13038	29.947	36.84374	33.53648	28.77411	36.4637	46.28464
Slc15a2	41.05579	50.30568	24.99338	39.35507	43.37549	37.07099	69.47449	49.09207	46.27526
Shkbp1	50.64273	49.31859	52.704	50.03107	51.9287	48.21715	51.81733	50.9224	46.23575
Haus8	42.97879	42.10436	50.07153	51.6418	45.86765	48.62474	50.24897	48.79204	46.22818
Phc3	55.75851	54.08583	61.09003	57.33361	44.39402	36.08549	65.65449	41.8223	46.21217
Gm7536	39.13275	33.93317	39.24601	35.67075	33.54264	42.32072	33.92941	40.57005	46.20682
Nab1	45.8471	42.20522	43.10716	31.6175	32.9116	32.12333	37.20233	40.64857	46.20353
Egln2	46.73855	46.47802	52.06666	45.62863	50.84912	50.85725	47.98928	44.9869	46.17176
N4bp1	44.90202	42.73925	46.92238	38.6956	40.65225	31.39542	53.09809	43.45846	46.1536
Rab9	38.96172	45.09267	50.64642	47.36919	52.89091	48.62335	48.43284	55.18167	46.14035
Suclg1	41.06081	40.95149	39.87065	40.98922	40.88857	40.21838	38.73378	44.09915	46.11885
Gm12543	44.10248	42.5719	49.86463	42.62256	42.76181	40.21132	39.99877	39.88196	46.00061
Echs1	45.80468	44.06305	47.17582	50.09925	47.31486	47.85411	54.01729	45.96503	45.97768
Tifa	41.12966	45.55308	46.91458	47.28803	53.8674	47.42752	56.28311	53.39684	45.96533
Khsrp	57.60069	55.70525	61.80835	48.27622	47.31938	44.87018	60.02034	49.33755	45.95361
Dpy30	58.90294	50.81383	56.62585	44.39958	49.1987	55.85523	49.72881	50.30829	45.93251
Timm23	47.8523	38.92221	41.03466	40.42053	39.93974	38.86968	44.2309	45.37839	45.92579
B3gat3	49.87494	49.78069	49.68755	55.01228	45.29274	49.48106	45.71862	49.21459	45.91515
Pagr1a	43.07051	48.00717	43.90011	47.90473	47.52622	55.15238	42.28366	47.1005	45.91465
Pdcd5-ps	35.30751	39.83857	39.00089	56.6996	38.74574	51.07245	37.47246	50.57484	45.90626
Arhgap22	48.90717	50.91839	49.59882	56.72886	54.33229	53.11511	76.83912	45.85459	45.87389
Stag2	44.31223	42.71194	43.25563	35.66082	39.40179	32.35873	41.23437	41.36076	45.82546
Ube2c	75.56488	92.46752	93.97576	51.21945	53.36572	65.97028	55.75939	45.18542	45.79998
Ddit3	31.65965	34.59853	35.36861	35.63529	38.62061	38.24809	43.16496	46.04838	45.77529
Ndufa9	46.92526	41.61424	42.77867	39.44829	41.17652	41.72746	46.37584	43.55951	45.72313
Ly96	45.44104	50.55704	45.85028	48.29934	50.29812	38.75403	42.9996	49.89818	45.71542
Gas6	44.70536	41.19599	45.90797	74.30416	73.50673	67.02523	86.44419	54.71627	45.70547
Ltb4r1	50.65892	32.37194	40.22913	26.65289	44.52986	37.12466	25.52066	41.89988	45.70131
Uhrf2	52.32971	58.54385	54.16344	46.50005	37.52507	34.95216	53.41459	40.03402	45.69149

Ing4	42.16996	47.52861	49.54688	52.5365	55.672	49.46019	61.62105	47.49443	45.64864
Rnf7	49.32856	45.85724	46.93547	42.84196	42.23888	43.69237	39.52393	42.8292	45.63426
Traf7	51.35765	52.17972	53.8848	53.72544	48.24104	47.652	62.59724	52.29431	45.62952
Wfdc21	4.429982	8.331441	4.810254	14.97757	11.09012	34.06121	3.139617	3.976761	45.5994
Tnpo1	55.77145	52.91545	56.24235	44.34119	45.14417	38.05414	54.1476	47.14826	45.55183
Ranbp3	47.42406	47.5799	51.6492	49.47883	42.09072	44.20504	51.40255	46.54372	45.53631
Tgoln1	50.58378	50.61416	53.01708	42.92816	40.71534	36.65746	51.1064	42.24698	45.51902
Fibp	56.71363	52.59934	53.7272	56.98137	57.98502	59.29776	57.55154	45.49569	45.49627
Micu2	48.21432	46.19507	50.33209	44.93729	46.05593	45.80774	48.54208	49.67493	45.49506
Atg7	51.68872	48.10941	50.85522	45.649	44.44889	44.67784	56.62323	44.79051	45.47714
Git1	63.42722	57.88079	63.31307	52.03132	54.93948	51.22525	63.81271	49.29738	45.45464
Pafah1b1	46.14312	44.8377	45.41485	37.48112	37.47677	34.85662	44.97016	41.76101	45.4438
Pdcd10	39.38034	40.33933	34.13744	34.02827	40.05885	39.67024	35.6064	41.61114	45.41873
Zc3h15	41.68445	40.02433	40.95493	36.90146	35.01976	35.3361	40.63109	43.47899	45.4084
Mvb12b	54.34757	52.26287	58.86373	51.69854	52.04073	46.60671	61.95861	42.87699	45.39353
Ugdh	46.00488	49.25966	43.39766	40.84055	34.09334	35.00587	43.64848	35.99433	45.32213
Zpr1	49.18481	46.48091	47.31152	41.29685	44.8935	43.20256	51.06681	45.62733	45.30065
Ift22	45.45799	46.49012	49.23667	36.91305	48.79959	45.02075	45.22203	50.52285	45.29748
Rap2a	52.48951	47.11744	50.2773	47.65216	44.60179	42.81699	58.49229	41.22445	45.2675
Cxxc1	47.06882	45.46154	44.64845	52.95458	49.6977	45.24544	55.46862	48.18455	45.25647
Ccm2	42.62962	47.39183	48.56691	46.11785	50.9983	47.01771	53.39056	45.05593	45.22707
Oxa1l	47.64961	51.38479	59.42671	42.5313	52.40272	50.09394	51.75814	53.94893	45.20579
Rnf167	39.36716	44.93989	46.42273	48.07329	61.0145	55.03347	52.36476	46.98724	45.19977
Sh3glb2	42.06752	45.60105	46.1129	54.33042	50.75262	49.72551	50.37187	46.70745	45.16696
Igfbp4	66.05443	65.32408	64.79117	58.76057	67.39765	58.17159	74.01676	47.111	45.16664
Wwp1	49.02851	48.7667	50.58362	60.42379	54.32229	43.18633	75.66476	42.38987	45.16512
Nup98	59.61355	46.49746	48.33407	41.49293	41.71493	34.56961	56.13163	45.10014	45.16051
Trmt112	48.38985	41.29064	41.93879	35.16147	39.09127	51.37693	46.40159	45.56435	45.16017
Igf1	42.77046	42.46996	41.78273	60.71794	50.59027	50.0711	57.84458	40.70617	45.09867
Ptpn7	61.09123	62.6905	64.26178	42.31184	44.85077	45.83724	54.8461	46.98361	45.02805
Wdr91	52.91528	56.69527	53.29989	58.97412	49.47492	51.6951	71.20423	48.51904	45.02299
Tpgs1	35.80483	47.09738	48.5204	41.97501	52.35575	51.84074	48.75403	48.89634	44.95857
Tm9sf3	49.7174	51.25887	51.36558	37.21826	40.3294	34.60382	46.2189	43.92525	44.95135
Pcgf3	54.49843	58.05723	56.20525	48.27552	45.21333	45.26757	62.15377	43.75109	44.94804
Dhx9	52.61388	46.48091	48.49742	46.06907	41.30721	37.7087	59.89399	45.36798	44.93412
Oxr1	48.94834	45.04442	53.64825	50.57921	40.30027	35.47747	53.26363	46.39841	44.89467
Smcr8	52.68415	45.30292	50.46812	52.31124	40.41485	31.7907	58.47857	40.03006	44.88867
Map3k8	36.32546	33.24318	30.37069	41.53169	40.59955	29.30706	54.97752	46.61629	44.88161
Commd6	34.75318	39.72201	35.65666	38.39124	39.70602	40.93074	38.11014	42.385	44.87811
Agpat2	46.72188	37.5521	38.75618	42.7902	40.53377	46.07529	39.32136	40.33448	44.86688
Bfar	40.61088	41.49899	40.05366	36.62752	36.59633	38.2196	44.32852	36.76347	44.86419
Rnaseh2c	43.50944	49.55601	42.57039	41.98567	44.54371	52.37844	37.78475	48.77885	44.82182
Stk10	54.96816	50.25515	63.05132	42.20684	43.85833	38.89773	54.85835	47.73624	44.81753
Coa5	46.94005	46.7435	53.52075	43.55103	45.79889	39.42624	50.63648	45.44322	44.80644
Tmem131	64.14153	53.42438	68.28955	48.74593	49.29912	37.9886	59.1006	49.72741	44.77623
Cyp4v3	49.24002	56.32073	54.56443	44.91238	45.98471	43.87271	50.2791	47.3913	44.74223
Map1s	53.43637	49.83954	58.19497	51.76968	47.27837	49.99576	60.32032	49.46803	44.74175
Pum2	49.73064	51.75802	57.14827	43.66177	41.6614	35.49554	53.08023	44.20532	44.72833
Brf1	43.68398	47.61627	44.59799	46.90358	42.35429	41.32207	57.39194	41.18763	44.72411
Vill	48.98459	42.24671	39.37762	57.51695	42.99081	46.66443	47.93164	51.05029	44.72367
Eif2ak1	53.27251	48.16318	53.97176	46.62152	52.55111	43.61733	53.35877	48.26709	44.70539
Samd4b	53.12762	51.00927	50.34326	42.54775	43.35955	39.11313	60.40366	46.08699	44.70301
Man2a1	65.49864	57.501	65.62484	51.88953	45.58853	36.80151	66.15007	42.45697	44.66577
Pak1	56.41454	55.54772	60.03264	40.25097	48.28905	42.27131	49.89059	47.87084	44.65454

U2af1l4	45.74923	51.19488	53.19849	47.59853	51.63752	45.68968	57.56327	47.71193	44.64271
Cstf3	49.38121	46.69131	45.32481	48.60859	41.60096	39.93321	49.93322	44.302	44.61976
Kat2a	50.14004	43.81475	48.16116	45.76761	44.21558	39.88586	52.4432	45.84881	44.61246
Rassf3	42.61982	49.11883	51.36395	40.65676	44.57361	45.13605	55.59365	45.2398	44.60637
Fam168b	56.02302	51.4929	57.1223	47.87304	46.36565	41.50994	59.76194	50.09238	44.55887
Lrrc8d	54.239	48.38101	53.14974	46.03038	40.40609	36.8249	53.76374	45.14346	44.55554
Rab44	30.86588	37.28323	33.68187	24.19042	36.77197	40.597	34.55411	31.21475	44.45659
Klhdc3	51.58859	50.02204	56.37186	50.23786	55.60846	51.63117	61.42183	53.74556	44.42841
Nrp2	79.95784	70.44355	78.63297	49.84486	46.23251	39.82611	68.15729	42.5317	44.42122
Dap3	40.08804	48.72306	45.47695	40.88109	48.51878	46.53564	44.8819	48.08034	44.39868
Aars	57.67648	54.02541	49.40008	44.20589	40.68975	43.96713	49.3241	40.90464	44.37488
Adar	41.9873	34.10148	46.2703	52.20667	44.09842	38.01979	47.99396	36.97126	44.35732
Lima1	65.26751	55.32457	66.07864	55.82448	42.83476	43.31564	57.54536	44.97008	44.34887
Slamf8	44.96921	38.11739	39.12994	44.31333	44.70322	45.51642	34.81807	45.16561	44.31908
Ilkap	55.1269	41.58423	44.76016	48.6298	45.39001	50.26026	52.68844	45.52426	44.28654
Tmem11	42.33782	38.33714	42.61475	34.16735	40.2789	41.41633	43.19604	42.9424	44.23998
Slc20a1	48.73827	46.47248	38.63815	29.81637	33.13315	32.24843	44.15834	41.90703	44.22744
Dpm3	41.38334	48.05932	49.19724	62.22644	60.97867	61.4796	46.57625	62.55307	44.22607
Jmjd6	37.63419	36.48738	33.62137	32.38562	37.49666	37.41314	43.34751	40.37928	44.21511
Mrps26	40.97345	35.80661	36.36745	42.17657	40.94362	46.68423	46.33872	47.07626	44.21425
Tec	43.00108	40.53554	41.16335	51.75952	40.6957	44.20121	56.73791	45.0431	44.21194
Dvl1	44.11904	48.91852	42.48901	41.97096	42.40178	41.28839	46.35315	42.19724	44.20697
Ciapin1	42.82134	38.97319	39.1447	35.57321	35.98815	41.43289	40.12811	38.52341	44.20109
Tiparp	33.48438	27.19269	22.54352	24.27257	25.6621	20.61062	37.53199	39.5805	44.20076
Mettl9	36.54698	39.52943	38.98555	45.19301	43.28378	42.93559	40.70186	39.22367	44.18121
Stx12	50.89788	46.9969	49.87616	42.84264	43.39954	37.23515	51.27184	42.02122	44.16022
Zmat2	46.96392	42.42937	39.79623	42.49973	43.04361	47.49672	43.86839	48.22218	44.15212
Rpl7l1	47.39678	43.60012	46.50871	41.92738	44.89705	45.50325	40.80779	43.3035	44.1393
Ufsp2	54.30588	54.70642	56.77205	42.93735	44.32108	43.97442	50.11558	44.79076	44.13039
Hibadh	43.44886	45.88408	45.50031	43.65057	45.2168	44.34895	45.07179	44.92902	44.12258
Gpatch2l	49.46781	46.96146	52.42349	41.98173	41.15584	41.34254	55.71864	43.61321	44.09891
Gm12338	35.87714	41.97638	32.72895	43.05109	34.52216	41.32254	31.14022	41.48455	44.09007
Mtdh	45.85675	43.53847	47.49039	39.62473	36.42245	36.0269	43.24286	43.33672	44.08512
Chrac1	41.43197	45.47982	41.50194	42.56026	42.37538	47.83349	48.00644	42.85862	44.08291
Exosc10	53.15951	46.76864	47.6721	45.78113	43.03328	40.29899	48.99242	44.80215	44.05379
Zc3h11a	47.91196	47.64446	47.21586	47.58015	43.91784	40.20231	59.68432	43.64938	44.03522
Atraid	52.20312	59.46572	57.15732	43.83711	60.22907	61.76723	60.83816	52.96364	44.01101
Pdzd11	48.38529	46.62066	46.0944	42.24269	38.94808	44.998	45.99662	47.05656	43.97906
Gramd1b	55.94296	54.20279	52.81872	47.63661	35.60299	36.55019	58.94702	41.5722	43.94455
Mefv	41.39041	33.59602	43.45602	32.74867	36.3481	30.0413	32.40837	39.6295	43.92301
Txnl1	44.68854	43.38076	45.32231	39.58924	43.22137	39.90358	40.58795	41.7225	43.91412
Nav1	73.39713	67.66272	71.26375	54.75754	46.36839	36.49728	77.86676	44.79036	43.85962
Chchd7	36.23027	37.16621	41.41083	41.17408	41.62546	36.69553	39.8233	36.13738	43.84846
Mrpl55	37.65447	37.38768	38.15985	31.964	33.79429	38.06449	29.7708	39.1099	43.84549
Hp1bp3	50.53193	55.56698	55.90263	48.21111	51.65056	46.49625	58.10783	42.87787	43.83011
Hlx	35.2647	35.40237	40.22242	41.35206	40.85811	37.93166	49.13924	40.55089	43.78939
Dnajc13	57.40661	50.36517	57.58399	49.08522	45.20176	34.83067	63.96631	41.62361	43.78173
Dpp8	56.73834	57.0842	59.13919	47.46622	44.7434	37.70322	56.3309	46.84704	43.76864
Shc1	51.62626	54.23932	53.03769	47.96584	49.20174	44.6884	49.36416	45.30663	43.7633
Ubr2	55.71049	59.24707	56.2789	49.54028	43.3448	40.84386	64.95299	44.4501	43.74989
Nol7	36.673	35.2015	32.60541	36.8633	36.58876	40.05707	37.80846	42.34839	43.73454
Eng	66.86509	58.17589	64.7553	63.14754	59.47924	57.61976	59.08544	48.38137	43.73335
Fxr2	40.9159	45.38659	43.1235	41.98601	38.39898	39.58118	45.02813	42.6525	43.72873
Coq10b	39.88879	31.81909	24.93777	26.78021	31.58577	25.52034	39.48965	40.04532	43.7252

Gm12715	60.09428	46.96062	54.48405	32.84501	39.107	38.32271	37.86782	43.00178	43.7122
Krt80	48.24918	39.03526	55.19724	49.49185	57.74338	53.0613	61.07201	59.17431	43.70188
Emc6	47.77589	40.86509	43.51963	38.87041	46.51584	38.68736	36.16592	49.19342	43.69922
Morc3	37.47699	35.90672	42.05257	42.54523	33.21614	31.45953	43.7493	36.88137	43.6905
4833439L19Rik	56.15241	56.30824	57.68984	45.18275	44.97479	43.29298	47.51542	49.04179	43.67049
Stk25	46.97424	53.60221	47.93199	53.75986	45.06522	43.00003	56.06785	42.40729	43.66144
Ndufaf8	39.05353	41.63574	35.691	37.83043	41.68311	37.77597	35.14432	42.09776	43.63898
Gabarapl2	42.14424	40.50873	42.14914	36.22666	42.93927	41.85093	42.85657	38.77896	43.61781
Trmt2a	48.36366	51.27745	46.94898	49.11266	45.68532	45.09001	53.32955	44.82003	43.61341
Tmem251	35.56039	32.63361	31.04634	37.67154	32.88388	34.07879	43.69056	40.18595	43.61084
Rbm6	43.63334	44.20487	49.40354	42.48385	41.79233	38.41228	49.60126	44.27425	43.61057
Klf3	43.23192	37.83789	46.4553	36.81053	42.44179	34.75633	46.67792	40.65391	43.55172
Snord14e	36.55257	22.48074	26.80833	46.04057	35.39247	31.54916	45.96433	59.74279	43.54731
Atp13a1	55.2807	48.43213	53.85498	49.21262	47.08862	42.65248	60.84355	47.64356	43.53874
Fas	25.77121	28.29278	29.1708	20.46175	29.30085	33.71639	29.11358	40.2288	43.50755
Ppp2r2a	53.44464	50.01674	46.44159	40.02066	41.10777	40.42012	47.79894	47.64807	43.49786
Mindy2	42.51844	45.63691	49.9603	43.16843	47.93219	35.01703	53.4123	38.3459	43.49336
Smim15	44.85011	45.38327	47.59185	41.73012	41.38346	38.97311	33.59803	35.9623	43.48314
Enoph1	50.37266	45.18663	51.22284	34.75553	44.16858	49.3416	46.42011	43.08336	43.45004
Tes	46.97025	50.80746	48.4288	27.76694	35.45852	40.87535	41.7182	37.00393	43.44697
Zc3h7a	37.83486	40.3556	40.84357	43.53745	40.75406	34.22456	51.27481	39.44027	43.40368
Cers5	60.07169	56.91343	58.80979	42.56613	44.65554	41.11593	49.03113	45.08831	43.40027
Cd33	47.68386	50.95701	52.28007	29.86813	40.15071	37.46146	42.76624	34.7923	43.38698
Mcee	32.1298	38.25939	39.49915	38.79046	37.76834	48.57304	42.99442	41.07015	43.34981
Cdk2ap1	45.45921	49.4518	45.24272	37.75133	47.56098	47.08397	38.18339	39.56574	43.32667
Tbl1x	57.36467	57.04382	57.01005	53.69067	45.2068	41.44596	59.63771	46.88973	43.28902
Gm21399	40.746	45.32646	41.33848	35.1024	34.8135	32.42707	34.54078	38.57037	43.27812
Rpl39-ps	24.8622	28.4923	28.10836	29.32969	32.50276	41.27096	23.94067	37.63875	43.27074
Tmem242	38.48121	39.68842	41.6262	41.89919	41.0535	41.86924	45.61417	36.96404	43.26028
Ddx6	48.82145	48.55796	50.67272	40.875	40.57789	33.17613	52.20858	44.74402	43.25959
Gpaa1	50.23734	45.0525	45.74989	42.80777	47.48244	41.77436	50.17797	43.44962	43.23532
Nup88	40.58374	40.72373	40.04577	44.24591	37.70406	37.38421	47.71718	37.71406	43.22668
Safb	41.43657	43.1562	42.90505	43.68064	40.96156	45.10641	51.78945	46.66819	43.21924
Gnptg	41.01698	41.44545	39.76369	47.35428	42.18754	46.44808	49.59017	36.57132	43.20335
Wwp2	45.72534	40.09793	43.86861	41.4298	37.95597	33.62842	51.1349	39.17158	43.19563
Abhd4	48.31671	48.32919	49.73733	39.83907	46.7125	42.4357	50.32768	44.47557	43.17339
Cmas	48.8926	54.66404	57.39515	40.26906	41.43289	40.38415	45.13456	40.12978	43.16472
Mcm6	66.93314	58.53408	58.94016	46.94947	34.83711	39.46171	52.14003	35.98319	43.1642
Ipo7	54.53348	56.94251	57.31263	34.89424	34.85223	30.46276	44.79177	42.31322	43.16342
Rcc2	57.90528	54.91989	53.30402	44.07246	47.67445	43.39172	53.64199	45.97594	43.14818
Lemd2	39.48574	37.12299	37.8885	40.39004	43.72766	43.38769	42.88522	44.95456	43.12883
Ebi3	51.64606	42.29348	52.13279	38.45118	50.15359	39.90756	50.72916	50.39251	43.11491
Lilra6	44.87483	54.88818	60.61328	27.17519	42.45017	35.18076	37.67754	47.66984	43.05803
Ctns	45.63607	45.12496	51.39539	49.12389	53.47508	41.97291	56.674	48.79251	43.0552
Ncoa4	39.13943	40.92952	41.31955	46.31358	42.39349	42.74033	55.88862	43.5552	43.02805
Sys1	34.26955	33.00924	36.7906	36.75168	42.5541	41.16057	45.00459	45.16709	42.97967
Naa10	55.66978	47.54865	45.30575	41.70567	43.80574	50.89239	45.14388	46.27429	42.95454
Synj1	48.75197	43.74246	48.4652	44.33075	39.37617	31.78073	58.31543	39.35486	42.95361
Nudt3	44.55984	45.43445	39.63605	40.2728	43.4111	43.7107	41.88482	46.72865	42.93191
Sarnp	35.75394	33.71776	27.24956	35.2219	32.99251	43.19419	30.28691	40.18244	42.92707
Ifi206	28.71451	11.57163	36.66547	41.01283	32.62725	22.84018	29.76658	31.80021	42.92425
Bcl7c	36.30762	47.21176	38.49515	46.95837	46.67068	46.5391	45.36339	42.99173	42.87221
Usp7	51.128	48.09741	49.47888	42.04871	39.30785	35.55254	51.87327	42.84681	42.86786
Gbp2	27.97872	22.25132	36.04458	37.5451	47.80908	34.74905	30.59783	40.89298	42.85102

Snhg1	38.31748	42.74557	33.8957	33.47028	46.36516	39.20447	41.76976	45.46389	42.84713
Vps37b	39.59231	35.48099	34.12309	30.04362	34.40737	32.38907	36.26745	38.41017	42.84625
Taf15	49.2823	44.96188	51.71441	52.17491	49.66035	44.13482	51.38482	49.54174	42.7945
Ppp3ca	44.70268	43.5117	45.76989	40.11265	44.35033	38.8607	50.61926	45.0149	42.79076
Washc1	44.69712	42.11258	42.5425	44.81407	46.70958	47.32576	54.41989	45.3426	42.7899
Zfand2a	40.7322	37.42602	35.68512	39.16815	32.63448	32.15186	60.11199	43.67557	42.765
Bcl2a1d	39.69811	40.91217	39.35445	27.77153	42.84912	34.84379	32.03218	42.23924	42.74785
Slc1a5	72.12539	56.81321	58.51998	38.2343	40.48985	41.78153	42.96239	43.18007	42.73312
Dgka	46.77587	46.14563	52.99345	38.9086	42.17449	40.24802	48.78191	47.15019	42.72902
Trip12	57.42584	51.21601	57.17245	44.83062	37.98924	33.22174	54.37756	41.33361	42.64332
Fh1	44.43198	46.44806	46.57746	48.10147	45.7782	45.8642	44.02678	44.05008	42.639
Hdac2	40.32128	39.65336	35.88804	33.62367	35.75316	35.92	39.65609	40.16425	42.63081
Sesn1	40.32631	40.00727	50.98687	58.1034	49.74631	49.97196	64.42735	42.25427	42.60167
Abcf2	52.61223	46.06105	48.13101	37.17354	39.89684	39.65742	41.95908	39.24798	42.57831
Cant1	48.41726	46.45207	55.84921	48.52832	45.05224	42.28624	52.50923	43.13137	42.55917
Vps26c	40.47785	43.65763	45.69273	46.50883	41.49246	40.07596	50.72243	40.68969	42.55553
Bach1	43.98621	39.42691	48.92104	41.51543	45.25729	32.02724	54.57684	40.87084	42.52651
Xrcc1	39.82929	41.22629	48.98495	46.70675	44.28931	41.81029	54.14569	40.68862	42.50931
Clasp2	58.48289	58.66049	62.46327	48.40736	50.1463	31.45407	63.74547	41.18659	42.50349
Ccdc59	44.12417	36.56967	41.09477	44.84865	36.39087	40.94733	42.52171	45.38901	42.49538
Ube2g2	46.76712	47.5353	47.89152	39.41256	41.66348	37.50641	44.3756	40.87845	42.46719
Sel1l	58.6111	58.15838	59.95219	48.03714	46.66882	40.06359	62.93464	43.35028	42.45034
Dpagt1	59.80161	60.27159	57.38993	48.82798	48.4926	44.09627	51.18513	43.74763	42.44028
Brox	46.04546	44.10925	51.25655	41.8226	38.11732	32.4606	50.14222	38.91575	42.43846
Ddx23	51.02508	47.47043	49.37595	42.96186	43.07946	44.72688	56.82968	45.97364	42.41638
Tut7	44.46714	40.66348	42.89341	37.7338	36.64088	29.49514	44.58745	36.77128	42.41384
Runx3	52.71079	42.76192	57.84148	36.32434	45.80916	37.98433	54.4109	49.17866	42.40855
Ythdf3	43.08977	42.99961	49.05417	43.2524	41.47205	35.25985	51.53385	43.88576	42.3943
Atf1	46.34517	33.21227	37.44706	39.12658	38.15374	29.54093	42.46301	41.72285	42.37882
Dgkd	44.84496	44.63795	46.77071	42.65924	37.56985	33.27679	47.89319	41.05967	42.32255
Rufy1	47.43584	43.60707	47.07008	44.7501	38.04721	40.33873	48.8781	40.66685	42.32103
Dtymk	44.65608	49.70215	45.68016	48.37377	52.47518	45.69314	41.94249	39.60706	42.31847
Gm13456	47.00184	39.69653	49.7034	40.52567	34.5849	35.57628	42.12124	42.9084	42.31828
Sdf2	42.29199	43.85911	44.3556	39.82208	42.68444	44.77784	40.56795	36.48004	42.31451
Stt3a	54.96097	46.14518	50.72075	38.85494	38.23128	35.72834	46.81474	41.6173	42.28811
Mat2b	41.19791	43.67858	42.36713	33.57525	44.35424	38.81634	39.74143	42.29419	42.2813
Ppp1r10	48.18067	42.81103	41.48129	44.67569	39.21322	40.7435	59.5578	39.64015	42.25485
D17H6S56E-5	73.48682	87.699	89.0715	36.85421	41.98589	47.22926	45.60146	34.61089	42.20749
Iffo1	38.98747	47.72487	45.56418	48.02278	42.17168	43.72207	55.33793	44.00639	42.20266
Arl6ip4	38.27196	34.94246	39.40219	40.62274	37.11983	48.18787	39.84684	40.99349	42.1984
Ate1	43.37515	41.85182	47.78666	43.68516	36.99161	33.22886	48.05782	34.33304	42.19653
Zfp655	41.63574	34.41395	33.7853	39.7722	37.14785	29.47698	48.94369	40.87556	42.18997
Parl	45.38127	41.29024	41.36336	41.12688	42.1032	37.82084	40.40744	44.24472	42.17407
Spag9	50.15057	49.24534	48.3104	40.05121	34.8741	30.7172	45.49352	34.28021	42.16698
Gps1	49.71235	44.7023	40.02503	40.03344	35.78604	39.93285	44.69183	38.27368	42.15054
Bop1	51.01835	41.50607	42.92478	38.05578	38.05392	36.07617	44.46536	43.97728	42.14817
Hikeshi	42.9105	41.64737	45.29168	36.22666	36.20476	41.01391	39.66213	39.27838	42.13268
LOC115487513	37.45815	21.66789	23.22675	17.13423	20.69134	29.79051	35.61768	24.96468	42.12712
Relb	45.24012	38.75162	41.97682	25.93882	35.65059	30.69887	34.02851	32.93142	42.10841
Clasrp	37.39758	43.97534	38.35449	50.28054	44.23451	40.59424	50.84148	44.72774	42.10438
Gatm	37.5624	46.72831	52.22181	47.17532	57.79397	45.04656	58.97538	48.2589	42.09955
Ifnar1	47.37607	43.97067	45.01013	41.16094	40.95769	36.37104	53.1236	40.21521	42.08008
Med1	48.52477	47.08227	50.94485	44.78293	36.01425	31.41802	57.68362	38.76147	42.06493
Mast2	61.09057	56.02434	61.95066	49.20734	40.59838	39.62203	57.7474	42.73549	42.06271

Il1f9	22.08098	17.08734	15.55274	10.17533	21.0938	15.53274	8.66254	19.29268	42.05818
Smndc1	36.21766	36.45803	33.97031	33.944	38.72791	34.45585	36.85105	41.52935	42.05535
Vps11	41.40702	36.95155	37.34613	41.03862	38.48809	39.3547	49.12524	39.4562	42.03124
Rps6-ps4	45.96202	39.98915	40.42291	38.97388	41.62495	69.92633	34.1123	46.39947	42.02416
Asl	48.1363	43.48689	43.98874	46.38696	47.65717	45.21899	50.18046	44.44666	42.01925
Prkacb	53.12984	54.61339	57.33204	46.49993	43.7984	38.81153	58.12597	40.90221	42.00593
Osbpl11	40.73551	35.77187	41.80719	41.00727	41.78576	40.90278	58.33725	41.38771	42.00345
Dffa	54.37681	44.30294	49.32893	43.31807	41.68058	35.19083	48.20972	35.65343	42.00019
Ppp2r5d	52.9371	51.87025	58.1542	46.17825	44.49807	40.47425	52.87239	43.16762	41.9846
Tmem243	38.66075	36.99089	38.55301	41.40144	39.59895	39.22889	36.3042	41.88406	41.98113
Kpna2	52.55463	55.53464	48.37111	38.92469	39.76735	38.53858	41.58182	42.11202	41.97436
Etnk1	40.2863	34.54547	42.97349	42.86911	39.09567	30.83271	42.44569	33.7226	41.93471
Tardbp	44.28162	44.14943	43.98559	41.39555	40.35693	37.93999	48.30165	40.1398	41.90364
Gtf2a2	37.53619	46.86731	44.03795	40.69962	43.10511	41.98852	42.37665	44.05412	41.89841
Gm6377	41.18765	31.01083	32.2651	35.26902	34.91004	28.38833	32.89368	48.80635	41.85869
Urod	37.70427	37.2321	39.83909	35.00412	40.87961	38.80759	38.25371	39.75164	41.82326
Nenf	35.16776	43.43671	40.13824	48.16575	46.98378	54.95623	44.08833	48.9071	41.81991
Plcl2	34.48628	35.62284	39.44484	41.83214	38.53998	36.68826	45.07745	39.59662	41.81602
Prdx3	56.05191	54.2191	55.59799	46.3529	43.81005	41.26226	48.93131	50.40817	41.79457
Smarce1	46.81563	44.13388	43.57304	39.71675	38.72621	39.14868	39.96531	45.98349	41.77757
Tent5c	35.05522	26.97761	35.87907	49.7479	40.9586	40.25083	71.38953	44.51001	41.76993
Ube2e3	42.49887	40.24099	36.01523	35.22942	40.30189	38.56008	41.77744	44.52328	41.75579
Vps4a	38.80145	45.3765	43.14081	40.18425	39.82142	42.67482	38.30597	39.43859	41.73066
Tcea1	39.15692	41.3816	39.22634	32.07889	34.59503	33.15184	38.99572	34.76973	41.73
Ypel5	35.29944	37.70034	38.15916	34.49867	41.65651	39.70068	45.59814	45.01185	41.7112
Ythdc1	37.73691	39.18291	38.97843	39.06743	40.6526	34.334	48.40259	40.65677	41.69538
Cd2ap	49.97203	48.39855	52.00006	48.69782	45.43431	37.25361	58.74481	50.58232	41.67695
Mrpl37	46.95189	46.23555	48.32863	43.70404	40.55187	41.15784	45.42476	36.02333	41.67656
Vps37c	47.95087	45.36534	54.20041	49.19933	43.63373	39.74199	54.12017	43.70978	41.67488
Frmd8	47.19374	40.40997	41.97051	37.5715	39.87185	37.6184	41.33686	41.73587	41.62598
Rnaset2a	38.22648	36.38885	47.64359	33.65343	45.67693	40.83507	42.24062	41.55085	41.59409
Dnajc4	38.43875	38.58946	41.86885	49.70308	47.84496	46.4574	49.03765	54.47344	41.59283
Syap1	36.07304	38.19722	34.61691	39.44141	36.95446	33.49203	38.99035	37.44456	41.59006
Ric8a	42.19048	37.71753	42.36356	36.75168	39.93261	39.794	40.88301	39.2444	41.58252
Strn4	52.93248	48.47225	47.71115	46.62987	46.01902	43.2113	51.72427	47.90076	41.57723
Add3	53.96402	49.36799	53.39465	40.83262	39.41511	37.07765	48.86799	42.66438	41.57149
Rcan1	65.03554	69.95063	58.34934	41.33936	40.79564	36.06234	41.81841	45.6406	41.54777
Prkaca	52.7512	53.42205	52.31358	41.27824	49.37104	41.16892	56.02415	46.5631	41.53691
Matr3	39.08681	38.52529	39.89723	37.64746	35.60836	30.51739	38.83503	40.5901	41.53029
Ndufb2	27.68607	29.19021	26.96409	32.84054	37.29176	37.12466	28.81933	34.33057	41.52856
Hars	52.53934	44.78278	45.63231	37.05656	36.4467	36.00831	44.59392	40.8142	41.52079
Ppm1m	38.51746	39.55018	43.53717	36.12964	41.3423	42.81765	41.01799	35.50075	41.51929
Mrpl2	41.22632	40.44452	38.16788	40.99539	39.14681	44.63945	36.848	38.72659	41.51093
Trim11	40.33232	48.47332	40.33701	46.82835	45.7492	44.91135	46.44729	45.12398	41.49967
Golph3l	43.75638	48.9424	51.27556	42.49362	42.00591	38.98722	50.25507	35.1487	41.48961
Smg9	49.97308	39.85631	45.53807	40.8623	41.98865	39.62896	46.24574	42.50114	41.46859
Exosc5	40.22083	38.71942	47.32412	41.54616	35.84774	37.69222	36.62404	43.26012	41.46019
Imp4	47.72099	45.41515	41.54082	36.22505	36.18082	39.38196	39.17612	42.40966	41.45329
Ptpn2	42.8105	34.79789	38.01909	35.74539	39.76657	31.45213	39.33554	41.51383	41.43123
Rab5a	44.39266	42.89455	44.53854	39.94647	41.40804	32.48669	43.4825	42.64573	41.41612
Reep4	52.2512	51.64858	52.62248	45.68652	46.60188	46.69323	55.03353	41.65706	41.41566
Igf2r	71.54981	63.58807	64.17504	43.52043	32.68203	29.15047	51.31088	33.20998	41.39294
Hsph1	55.3722	62.07246	56.21846	40.12996	38.32486	41.38543	48.68714	41.91872	41.38171
Orc4	33.86387	35.56305	31.35575	32.87135	31.52538	32.60207	37.1788	33.22392	41.38075

Dhps	39.33106	31.38108	33.54975	32.77853	37.41883	39.02787	38.32772	35.74308	41.37343
Man2c1	49.81576	46.10713	49.3123	48.71391	46.65594	44.81236	56.99101	39.8747	41.37211
Pgm2	40.87975	38.28517	43.33058	38.14917	35.07336	36.23182	40.6864	43.02437	41.35969
Parp9	35.60998	32.11887	41.56507	43.38471	38.55275	33.04952	39.62418	36.64093	41.35556
Map4k4	47.22539	43.77744	47.0708	38.94339	35.53569	31.0176	45.57373	39.75352	41.34893
Slc4a2	56.42778	49.87564	51.06098	44.3579	39.91203	38.42766	48.63243	42.88828	41.31585
Pilrb2	31.87959	30.3397	32.63985	24.2862	40.44805	27.28264	28.14639	36.88599	41.30175
Gm36220	30.62293	34.22743	21.12172	56.17757	49.78154	52.73217	42.56423	47.25288	41.26349
Plekhf2	33.58559	33.78021	37.8048	36.34263	35.53057	33.76273	37.61653	36.1343	41.2566
Notch2	50.58868	44.80181	52.00363	39.77409	39.20947	30.18656	55.01285	39.35323	41.24496
Tm2d1	42.98204	37.706	38.60061	48.84717	34.3358	40.64076	42.79218	39.50334	41.21651
G3bp2	46.71762	51.19287	49.19935	34.67285	41.64287	37.11381	44.76978	44.16889	41.18076
Ngdn	37.29721	36.9217	40.67162	38.18993	38.77466	34.61936	43.41481	43.59718	41.17886
Grk3	44.44868	46.13662	45.06785	45.45121	45.10977	38.83377	67.3553	39.80605	41.10551
Timm13	44.03366	44.54832	46.79969	54.09248	49.39363	49.46779	52.71416	51.6191	41.10534
Dld	44.87148	41.3768	44.77929	37.26011	32.43972	31.07114	38.34965	42.28161	41.09575
LOC115486430	34.85878	23.0377	33.74274	37.16932	31.33251	35.60235	30.09592	32.65582	41.09575
Pef1	44.25037	43.38395	45.00621	41.69219	42.79036	40.95441	46.62159	39.204	41.0844
AI467606	39.80095	47.93955	48.79579	34.6056	39.17227	41.91202	33.48856	41.5545	41.07433
Fbxo3	36.09002	51.98249	48.57318	46.99975	40.5391	38.01236	47.01375	36.54171	41.05812
Pih1d1	39.40399	46.8767	51.51413	45.2796	46.01164	47.39083	46.84572	44.25147	41.05136
Pcsk7	40.06204	35.51006	38.24669	42.11932	37.62717	38.60907	42.60551	36.10903	41.02314
Rnf34	32.69346	27.29414	37.04465	38.41576	34.37303	32.21551	28.69646	29.84497	41.02119
Gm4204	46.58056	45.82876	44.92219	34.1585	28.73306	36.09091	33.55163	41.65235	41.01005
Metap1d	43.85399	44.44458	41.02184	46.66445	45.59368	43.88882	54.79351	40.1777	41.00813
Praf2	54.88644	55.19878	54.38711	41.87132	40.84668	39.40702	44.75516	40.09017	41.00813
Plxna1	54.8854	49.59484	57.14567	47.6229	36.00224	31.28401	59.7034	35.10256	41.00341
Snx18	42.9592	34.91245	35.69544	27.92946	31.41559	28.53312	41.10201	40.62838	41.00334
Bccip	40.33075	34.31209	38.46351	31.15326	35.69423	42.9014	34.82097	39.0777	40.98721
Sec24b	57.69192	51.49932	57.42947	42.53874	40.18632	35.8374	52.85926	42.93914	40.98532
Ppm1h	44.43322	43.65827	46.79012	46.1672	42.49687	33.01849	52.9817	41.74955	40.95033
Herpud2	43.03319	47.12989	47.3016	40.18041	40.33013	38.0395	47.12886	40.10621	40.91817
Kdm5c	45.15816	37.97624	43.18762	40.13305	38.23778	27.51721	40.78259	35.06347	40.90691
Mgat5	47.95464	36.14187	46.51337	43.13942	34.70011	30.98506	59.47622	34.04333	40.8877
Dcaf11	37.42896	38.51646	42.68014	38.73174	39.71967	37.99698	46.59023	38.84706	40.88333
Coro2a	41.97433	32.77315	40.85235	42.73199	46.48233	42.31175	52.81676	45.77559	40.87524
Zdhhc5	52.71294	45.8421	53.8186	38.99633	35.94538	32.73974	45.14938	39.63139	40.85423
Dhx34	41.10459	32.71592	42.97667	42.95948	36.05625	35.86061	46.31341	39.40909	40.84632
Dhx38	42.53363	36.30182	38.80493	37.30853	33.1369	34.85722	44.41078	38.47019	40.83483
Vta1	44.01573	37.76444	47.99611	42.43779	39.09089	38.30544	40.51942	42.06663	40.83434
Cnp	39.35081	34.74318	40.63871	42.69843	44.83242	40.10415	46.31978	37.89569	40.8114
Batf	31.95048	31.49844	36.30879	26.89296	47.95094	43.66802	37.33244	43.20689	40.79702
Erbin	40.43328	38.37137	43.81154	39.55729	35.90066	29.25782	49.70574	38.36866	40.78781
Fam50a	36.23717	30.10392	36.80264	36.6321	33.74835	38.66369	37.8258	40.45511	40.76465
Tor4a	48.17077	49.4885	57.76451	40.25985	41.90749	41.36251	42.29451	43.32683	40.76373
Uhrf1bp1l	51.80875	46.20771	45.1895	44.71649	35.49032	31.35433	52.79393	36.87965	40.76285
Impdh2	49.9627	49.37462	45.07922	36.91373	35.10595	40.05547	41.83664	39.62564	40.75407
Bex3	47.98003	48.59611	44.6291	61.55495	48.89198	48.7385	49.39705	41.65791	40.75351
Abcc3	49.68103	47.86003	62.80784	51.38584	50.83956	42.95093	80.38573	43.12161	40.75174
Rapgef6	44.71128	43.51655	39.24459	44.46083	35.76559	35.03664	49.00834	40.21699	40.7261
Mxi1	42.3924	46.29025	47.32892	31.44364	39.25699	36.17051	47.3525	38.0599	40.72312
Fbxw4	37.96709	37.96771	41.06497	43.53418	46.32422	44.85503	49.21797	45.82463	40.7214
Luc7l	38.94058	46.75417	42.47369	41.90601	39.60673	38.91125	50.95032	42.33173	40.69445
Fhl3	55.20776	53.78106	53.83217	38.63834	43.6176	38.00176	40.32963	42.82115	40.67069

Pnisr	39.19051	42.04213	40.67716	45.14953	41.11659	36.65494	52.44474	42.97394	40.65332
Mrpl10	44.61374	41.10314	46.08185	42.8002	41.23585	39.22834	50.75938	41.01435	40.63879
Hivep3	78.64355	70.31186	87.68083	57.66437	44.38821	35.20663	65.00535	45.21542	40.63336
Mid1ip1	48.37348	47.67996	51.96574	37.27007	38.70014	40.64486	42.36039	39.20177	40.63031
Carhsp1	37.0106	33.69051	38.84424	26.93489	32.45011	27.04629	26.88924	33.84822	40.61562
Ddt	52.25197	39.76556	38.41967	39.73889	40.05411	42.25981	36.12879	38.39513	40.60411
Lars2	31.84317	32.86279	35.06989	44.32789	46.78186	52.76405	38.54593	41.17168	40.56505
Tcf4	41.30888	43.39597	44.59568	44.05368	40.02218	35.8689	55.80628	40.48729	40.55839
Ttc1	39.41506	41.11923	38.89052	37.20262	38.00636	36.79929	35.11309	36.55555	40.55549
Pnpla7	35.08277	40.1554	42.61946	42.25051	47.34542	39.49919	51.66949	38.44057	40.5487
Idua	48.43267	45.1005	50.41679	43.55584	42.13692	39.6881	58.90966	38.60078	40.52642
Usb1	37.22498	33.58856	38.50757	37.90613	37.21769	35.42916	35.3035	38.39278	40.5036
Eif2b2	45.00673	39.54733	44.21743	39.29339	44.31135	41.14168	41.84004	37.22726	40.49221
Anapc15	42.75661	50.59078	42.20713	45.13566	45.07681	49.60119	44.62082	40.70848	40.48666
Ankfy1	49.98991	42.82054	51.64872	42.27585	38.57322	31.277	49.90344	38.67072	40.48315
Atp11a	47.9498	47.18555	47.76853	41.53528	36.2073	33.30005	48.5609	37.35238	40.44784
4930503L19Rik	36.59637	38.50293	40.22445	39.43764	34.69712	34.08687	39.59452	41.30393	40.43646
Dnajc3	49.32813	48.24866	48.992	38.51491	39.04452	39.26548	46.40145	38.92071	40.40687
Tmx1	47.91091	45.8053	42.7783	35.57151	37.20936	35.77668	38.82434	40.11013	40.40239
Znrf1	40.93122	39.44826	36.80254	39.05141	38.6979	36.5779	44.98065	36.56845	40.38739
Vps4b	44.95024	42.12688	46.36191	40.92444	37.84008	30.91398	46.84357	37.50588	40.38702
Zfp984	33.06509	33	34.24175	33.81734	33.89973	35.85102	33.69912	42.18657	40.36768
Siglece	35.08417	29.02026	30.00825	18.29166	32.03161	29.62637	27.67404	35.64889	40.35422
Gm10250	34.95219	32.5352	38.54898	33.59786	45.77112	42.91736	36.52622	40.67301	40.34519
Phf20l1	34.55281	35.07346	32.74792	33.10366	33.55775	33.86267	40.17385	33.46626	40.28262
Prpf39	34.98404	34.36181	34.1333	34.64154	30.87638	30.38976	42.21958	38.6398	40.28114
Cdt1	49.36543	42.97566	46.00553	43.80512	34.0504	37.78951	49.4787	43.12215	40.27314
Pde1b	34.48216	38.47654	33.62352	37.32999	40.90585	37.77978	54.36017	40.14678	40.26329
Sqor	38.30391	32.91506	42.61805	33.22615	35.2425	37.54126	33.33252	44.349	40.25551
Ifih1	28.8488	22.25505	34.4589	36.33874	27.40549	22.28477	27.11525	29.18793	40.25374
Ephx1	81.70336	117.3253	113.5354	39.79447	56.62549	47.00769	47.81024	80.06015	40.22741
Lpcat3	50.65626	43.69086	50.50788	43.50074	46.129	44.76267	50.85716	44.17631	40.22346
Ccdc97	43.61575	39.50116	46.13848	42.33304	44.27411	39.85164	52.94235	42.82251	40.21898
Xpo1	42.55769	42.32813	38.4008	37.42783	35.63573	32.81285	38.57616	35.38068	40.21686
Leprotl1	42.48464	44.44627	48.32359	34.73994	40.21681	37.38453	44.10949	41.65425	40.20548
Abca1	58.17214	70.46571	77.03328	50.30252	47.95616	38.94522	71.69511	41.01702	40.18938
Abce1	52.47206	44.51659	46.96715	34.0047	32.01426	33.97658	41.3844	40.33782	40.1858
Mink1	55.72312	59.17968	60.61036	47.80289	48.09647	41.71406	59.77283	46.22537	40.14425
Acadvl	41.17315	42.20068	39.53082	36.105	35.83452	40.11415	37.1599	36.83676	40.13517
Upf1	45.84625	41.21044	42.97009	40.21755	39.9591	39.09998	46.5903	40.30121	40.12576
Grsf1	40.80699	41.70156	42.11496	36.00907	35.92482	30.845	44.32621	39.20051	40.11285
Aida	39.27634	42.93281	44.21417	37.65011	33.39796	32.19586	37.98407	36.12866	40.09738
Grk6	45.46801	43.59111	47.98045	37.43039	44.50611	49.40923	51.00848	42.14216	40.08858
St3gal4	36.71377	38.89605	40.96415	34.69489	43.95996	37.45011	41.99603	37.88315	40.07549
Adss	49.77048	48.38187	46.36664	33.46951	36.00563	31.18615	41.02654	39.55067	40.06784
Tollip	50.7262	41.10439	42.67681	32.78367	34.07719	32.20382	45.33619	37.02483	40.06661
Cebpd	33.94968	33.41893	32.92324	30.49089	37.44963	39.31256	28.77577	37.32141	40.04888
Fez2	42.49669	35.23241	43.13128	43.33684	42.33882	42.95463	47.53746	39.02906	40.03641
Clec10a	47.7774	46.83819	42.63823	54.90672	61.11668	58.93354	48.61533	44.99547	40.03368
Pym1	39.71771	39.43381	36.50643	35.05424	40.49216	39.97865	43.38893	42.18062	40.01113
Slc31a2	37.85168	42.68588	45.61012	38.42316	44.63418	37.27742	44.33348	39.81941	40.01104
Fem1c	39.55717	35.97932	35.15097	28.4101	31.94445	26.56388	45.0627	37.38605	40.00599
Tnfrsf13b	41.40079	37.42458	51.75126	43.24443	47.29374	41.63752	49.309	39.74518	40.00515
Rasal3	45.68139	45.08381	51.59972	45.84733	44.79422	43.54418	53.68091	39.2019	40.00077

Zfp638	38.23926	40.77525	40.01698	38.3483	35.05366	31.2496	43.20257	36.81549	39.99419
Ticam1	38.94377	38.15629	40.61359	39.48533	39.79485	36.24948	54.88865	47.76534	39.99212
Cggbp1	38.84821	42.27504	43.8542	37.92914	39.84189	34.51608	41.90917	39.00218	39.99122
Pes1	48.84872	38.99393	42.11192	36.51181	41.39004	37.75928	43.02763	42.37466	39.96838
Hps3	40.83432	43.38649	46.96211	44.51534	46.40607	33.34717	47.89347	43.3704	39.96263
Bcl7b	41.06277	38.29844	44.26242	38.91355	45.38271	45.17438	44.8479	43.65801	39.96106
Triobp	30.42042	32.43852	33.3318	36.16193	35.19218	39.4488	33.14538	36.73081	39.95376
Srf	49.20683	43.04442	47.84979	39.49207	38.23196	38.85051	40.79377	41.17936	39.93813
Sirpb1b	33.45245	32.95816	37.1135	28.91053	38.52406	32.39698	26.31602	42.33773	39.88621
Rest	30.59843	38.2898	37.20618	35.31368	33.85838	25.56268	46.26695	37.02814	39.88419
Acd	45.97857	42.40902	46.34779	38.34958	44.43919	41.59429	49.68334	50.96388	39.87728
Ptpre	51.95516	48.25471	49.98658	44.241	36.30828	33.55254	50.79781	41.67259	39.86591
Gbp9	24.67821	19.3723	35.58379	42.84725	40.70221	30.30132	41.1776	35.17518	39.86463
Colec12	43.95761	38.92625	48.85687	33.878	31.23729	27.15261	39.9546	33.88783	39.86206
Mrpl3	40.28681	39.48212	38.81504	43.03578	34.00275	37.6871	39.06865	38.68988	39.84095
Smap1	43.73284	40.24325	37.64932	37.93125	35.32045	36.00956	46.20317	36.06569	39.80324
Tbc1d1	46.10981	34.27095	43.47582	49.87902	34.47182	31.67562	57.12753	41.2314	39.75468
Rere	49.7308	47.40631	57.55034	56.29381	49.5707	38.9352	72.75119	46.32864	39.74907
Serpinh1	30.34295	36.14097	39.97278	50.92309	31.40913	46.00035	23.16084	36.98798	39.72879
Magt1	49.56036	42.30038	44.57087	40.06281	36.12329	34.04216	43.46548	35.10074	39.72668
Dnajc2	41.47649	40.28034	37.26241	33.50411	39.50175	42.71024	36.62347	40.88892	39.68641
Slc25a1	42.98267	40.85827	46.7875	45.66583	45.40428	47.20388	42.95643	44.32092	39.67528
Reps1	46.34771	42.10532	44.08024	40.91649	37.96005	35.46482	46.29914	39.92493	39.67491
Akap8l	39.23359	50.16143	40.69381	55.09536	59.2059	50.51961	71.10435	49.48741	39.67429
Ppp2r3d	41.79336	42.81327	42.86379	47.91617	50.83461	42.39968	58.11503	44.27445	39.63624
Ccdc115	39.40817	35.57399	38.95749	35.21048	33.63008	37.55599	38.68035	45.10502	39.6358
Zfp414	42.55802	49.28276	57.59959	44.2189	46.45531	46.46673	49.55366	47.70006	39.62805
Tm9sf1	48.95004	49.59071	50.55722	41.92218	41.46584	37.30162	49.59359	38.35141	39.62805
Prkab1	38.69828	38.39616	39.50136	38.42479	39.87055	35.78696	42.67876	40.86297	39.62805
Slc25a24	49.75169	46.33417	48.98718	34.53007	32.68113	32.6123	38.92132	37.1646	39.62414
Ampd3	41.64781	43.59382	40.60594	30.21148	33.94824	31.11341	43.68138	34.23094	39.60605
Dcps	45.22334	39.26827	40.49214	37.67509	38.47134	41.0912	40.40613	42.74761	39.56167
Inpp5k	36.21002	41.64057	43.16316	40.09844	44.68189	37.54302	46.537	39.95159	39.55302
Phka2	46.8667	48.62514	48.18771	44.95857	44.15038	40.5548	65.78416	41.9351	39.55222
Dedd2	22.56341	18.93426	21.68645	20.6879	28.56555	27.31643	25.98471	31.73301	39.54134
Rxrb	39.379	45.31271	51.82547	41.91009	51.19933	47.22111	52.87354	47.3556	39.53788
Trappc6a	30.96318	37.76134	42.7128	38.71177	54.28479	35.39348	44.0558	42.14778	39.5302
Exoc4	49.599	50.60968	53.06454	39.92144	40.06063	38.2264	52.2423	44.32804	39.52305
Nin	44.68637	41.19358	43.30752	35.05014	32.17257	26.56891	46.49121	32.9623	39.5125
Pink1	34.28869	42.18614	51.43901	44.25108	53.22242	52.26371	52.48081	47.07385	39.51086
Ell2	43.64305	30.70347	29.92364	29.23933	28.20648	21.95569	34.26584	36.81187	39.49784
Atic	57.62501	47.1589	49.64736	34.68959	39.51301	37.89585	42.19995	45.44427	39.4611
Arf2	38.57942	36.99028	37.28881	34.62174	29.90137	29.46349	33.45961	33.49791	39.44758
Arhgef10l	41.91381	37.95384	44.67699	37.41425	34.71309	28.96806	40.66563	38.90629	39.44115
Tdrd7	36.67295	28.54774	34.94655	35.67818	30.73871	24.12151	31.53687	29.91309	39.43135
Kansl1	43.26684	39.18294	43.10334	45.20277	35.84923	30.49432	58.24918	41.87302	39.42112
Creb3	36.98242	36.77756	35.03069	37.30567	33.02029	42.98042	38.70096	34.63532	39.41622
Usp14	47.16182	46.40133	40.80023	37.57889	33.05673	30.40248	39.76908	36.56222	39.41568
Slc2a6	51.02214	44.63072	43.40481	30.31874	32.84175	34.07421	31.94851	42.84726	39.40689
Prep	55.68138	46.08784	52.63672	40.55465	36.88059	38.94383	41.97727	39.95216	39.40507
Jarid2	49.50275	37.09937	43.23005	30.81557	29.90508	25.7023	34.5416	38.25289	39.40232
Fcho2	36.37339	45.19217	42.98033	37.29215	40.22337	31.83386	49.22537	41.45953	39.38353
Alkbh6	32.85219	36.45076	33.97076	35.58496	36.3165	45.78801	38.19867	40.6062	39.36246
Rae1	43.24098	40.18223	36.95115	39.32124	35.79739	32.88479	40.35485	41.37279	39.34844

Dcakd	41.33597	44.35496	43.50809	44.93183	43.61892	49.1138	53.01904	43.14366	39.33519
Micos10	31.37529	36.46527	33.66437	34.67626	41.35093	39.20074	32.12089	37.13919	39.33402
Nr4a3	52.33234	47.12952	29.2381	24.04085	44.08444	25.31553	44.11235	45.18647	39.33116
Il13ra1	37.54038	35.90831	38.66348	31.69425	37.10249	30.55078	28.37924	38.18457	39.30466
0610012G03Rik	32.93539	30.53128	32.63458	40.5884	45.36473	39.04913	37.1099	36.97673	39.30144
Sugp1	39.85942	34.83725	42.42022	41.23975	38.74409	37.84557	41.32249	36.2119	39.29455
Ubfd1	38.94835	37.90245	35.1306	44.02516	46.4595	43.92078	51.79456	37.2053	39.2904
Pigq	52.22013	46.55612	47.82649	43.82658	42.77036	36.31283	47.04667	35.85407	39.28835
Hmgn1	48.04632	56.85703	49.34561	41.66722	38.58698	46.73877	45.74291	38.39925	39.2758
Gm45969	42.43025	54.36382	27.56738	23.09812	35.69068	40.95663	50.25579	56.61583	39.25907
Vps51	37.67513	39.29805	39.57612	40.52324	40.65753	39.06922	44.36185	38.4985	39.24146
Chid1	43.21955	37.93825	39.42808	43.25269	42.93182	38.90763	40.25573	36.29265	39.23233
Ddx21	51.58602	38.58651	42.36535	34.138	32.24729	28.40867	39.4384	43.88383	39.21085
Emilin1	45.87316	45.38413	50.38955	42.14886	42.88173	44.49287	51.54506	36.85331	39.20883
Mast3	45.27953	48.98995	49.75083	46.26756	46.40916	41.12938	69.37226	44.53112	39.20194
Rangap1	56.82401	52.79472	48.10966	36.63683	36.11571	42.84743	43.61015	37.38758	39.18774
Nuak2	34.7342	33.27878	35.83164	30.57607	34.81584	34.37484	46.63408	36.84364	39.18303
Dusp11	41.94042	46.27076	42.83617	38.69138	36.85651	34.78082	43.89736	41.65115	39.18292
Dpf2	42.65753	39.7077	44.4861	32.50428	37.8066	35.34882	43.25878	38.43214	39.17365
Hpse	40.08899	33.41907	47.65346	47.00961	53.64555	46.82651	55.20241	54.37424	39.15765
Rabl6	43.3984	44.20119	46.73949	42.42971	37.67547	46.19045	51.57	39.20227	39.1106
Golga2	45.40389	41.2771	42.93712	40.39466	40.27155	36.05588	47.55065	39.55884	39.10921
Pkib	34.13164	28.52204	35.79067	43.60834	40.23923	43.04165	45.80633	44.01822	39.08587
Xpnpep1	43.80618	36.38795	35.83902	37.4781	36.17003	35.12105	38.33142	35.26474	39.0657
Dhrs7	35.03141	38.41501	39.3724	32.12954	30.28632	36.44545	35.50367	36.83439	39.06078
Skil	35.78854	35.33847	32.95587	28.55051	31.48123	24.35244	45.00051	38.53067	39.04364
Tmem127	38.84647	38.03547	43.94232	35.0519	39.59303	35.50963	41.08887	38.17071	39.04083
Ppil3	29.59897	35.74333	30.88251	28.47858	34.74194	33.43847	29.18643	31.83704	39.03715
Hk1	47.14993	44.54161	48.69098	42.33509	39.86515	37.23399	41.82955	36.63123	39.02779
Rps6ka4	47.04801	40.9559	43.51296	36.55807	37.44116	34.93123	42.36979	36.99155	39.02072
Slc44a1	50.17459	66.58821	58.39125	35.92676	37.86897	31.6916	46.53615	38.89235	39.01461
Trim26	40.94978	38.38537	38.88201	38.95913	37.70124	33.21733	44.83157	34.53626	38.99501
Sfxn3	49.95861	49.31996	51.01753	33.6815	37.32859	33.99192	43.95601	40.75185	38.97292
Spsb2	32.86685	36.13627	39.60322	46.90762	40.21179	40.87658	41.69453	37.98453	38.96758
Pdcl3	42.08223	40.44696	42.44462	39.68148	34.91021	41.08002	38.55692	38.6432	38.96216
Stoml2	36.27827	30.2526	33.91857	35.39842	30.8674	33.44965	37.27331	38.15477	38.9454
St6galnac4	46.91147	37.70564	44.00841	40.10789	39.54457	35.34225	49.08335	35.60388	38.93511
Odf2	47.82118	45.92379	50.34134	47.38173	42.84458	36.24506	56.0751	42.08047	38.92911
Dcaf7	47.0404	44.24199	47.28693	42.5968	37.50078	33.1856	51.29915	38.76528	38.9067
Ptpmt1	34.84798	37.39008	35.39725	40.61589	36.50372	41.14576	38.61764	39.28741	38.88865
Hgf	36.76038	36.2239	39.14491	42.0595	39.93772	34.49723	50.6939	37.61028	38.88221
Sdc4	38.19818	34.59026	33.31178	25.12112	37.79607	29.1974	45.65207	39.4152	38.8763
Sltm	41.94253	37.38157	34.68549	32.90946	35.07463	34.66159	42.26934	47.18433	38.85417
Ndufv1	45.71572	44.11547	44.10045	35.10609	39.23777	38.41866	40.15849	40.40747	38.80657
Cstdc4	24.63914	21.50185	22.09745	8.788446	14.79381	19.56581	3.875227	22.69391	38.79848
Stxbp3	43.17994	52.78562	46.80291	42.04448	40.42187	34.07172	48.9867	36.78978	38.78256
Mthfs	26.60133	34.25928	34.42925	42.30889	40.66344	35.96986	39.96998	35.3302	38.77583
Glrx2	36.11309	41.226	35.66955	36.05919	41.14454	38.77829	34.77981	39.92429	38.75691
Pmpca	46.73414	41.67747	46.39335	36.39105	34.02943	35.07768	42.04069	37.69167	38.74428
Ruvbl2	41.52863	29.73605	33.67938	33.45374	36.85761	34.92706	34.6677	34.02663	38.72417
Usp3	43.08574	47.0597	46.30648	36.31649	37.20656	34.59124	48.02066	40.25652	38.68954
Zcchc17	33.86037	32.22518	32.42563	37.00843	36.07729	36.80473	38.65614	39.68343	38.63493
Plaa	45.41771	38.9103	41.56855	32.74241	34.14498	31.1077	39.01575	39.76091	38.6344
Chordc1	35.85246	42.4019	41.48909	33.07651	33.81026	35.61979	38.38057	36.81607	38.60365

Pias4	35.68846	34.46979	37.34965	34.03869	37.87108	35.70024	41.20114	39.81559	38.59372
Rab28	37.10774	40.83087	40.43361	36.96339	33.57699	39.74994	33.7979	33.20188	38.59322
Klhdc4	37.50005	39.42483	33.88686	33.54631	29.10602	33.0405	32.57823	35.98485	38.5903
Nelfe	37.79147	34.13139	42.27743	37.97411	38.82472	39.6057	37.58388	39.68124	38.58869
Lage3	35.1594	35.57505	37.42504	37.13854	44.04515	35.01023	36.79135	36.94773	38.58521
Pik3r6	37.12395	39.08512	41.64535	40.83216	45.82174	38.3041	55.98866	42.7399	38.58469
Erh	37.84326	37.80004	34.09751	36.13979	37.79963	35.52974	32.37546	41.35817	38.54216
Dalrd3	38.00285	47.09711	46.93277	45.78288	48.23924	42.03051	51.31165	42.82245	38.52962
Mrpl22	29.64396	32.63674	34.4218	40.42685	37.25235	45.00137	29.28565	32.40257	38.52727
Aim2	32.868	34.77606	37.72788	44.67505	37.2995	32.9858	45.05531	40.15837	38.52197
Ctbp2	45.08701	43.96723	45.6589	44.85612	38.14616	40.58668	50.13575	46.89009	38.5065
Mogs	45.6919	41.45703	49.60481	36.28401	37.47998	35.75067	38.54267	40.79585	38.50055
Dnmt1	48.31167	41.35993	45.55087	40.57663	32.43731	38.85816	47.99022	33.38232	38.49855
Trmt1l	34.87927	38.43362	38.26409	33.98105	36.17465	35.32448	40.27376	31.97962	38.48578
Tiam1	56.84122	45.05883	47.11724	33.06598	35.17386	27.67802	38.23971	40.9228	38.47996
Stx11	23.92155	22.72423	21.85743	17.75075	22.06913	19.51792	21.14633	23.88247	38.45854
Nat9	32.17026	27.98155	35.88379	41.04385	33.72174	41.16359	34.29516	32.04191	38.43889
Rnf40	41.41799	38.26858	38.93552	34.28315	35.72398	36.12853	43.18888	36.85796	38.43312
Galk2	43.86343	41.00263	43.29922	40.56732	40.29922	38.92578	46.08682	40.49767	38.3998
Taf12	37.63604	37.62395	39.18667	36.41491	36.47521	38.64458	35.61151	37.26316	38.35448
Rap2c	35.74934	31.13111	36.86395	29.14217	32.49832	26.81234	30.30571	34.54311	38.33506
Tnfrsf21	40.43951	41.21626	43.67058	33.5387	39.18396	33.56557	46.39463	39.52135	38.32387
Ube2z	43.97101	46.5809	43.4599	38.02329	40.9342	36.19937	42.34631	38.69032	38.31842
Mfn2	51.29021	43.804	45.71592	38.73373	37.27786	35.86575	45.61208	34.13921	38.30862
Mafk	31.61441	23.23178	25.53391	34.48154	26.91327	23.51948	37.38061	35.06998	38.28607
Fgd3	36.68538	32.78316	39.02509	26.92563	30.23611	25.50181	35.00521	32.94237	38.27738
Ppig	34.24197	33.12095	31.6918	32.26239	31.84775	31.45275	31.88411	38.41279	38.27702
Celf1	43.10328	42.23129	41.47757	38.92592	34.36532	31.97593	49.23203	36.22721	38.26975
Gm42226	69.73069	64.48498	62.94746	36.40816	40.14403	37.7067	50.96709	40.9123	38.25699
Uimc1	33.70229	37.52335	37.45885	38.8918	37.50633	38.45054	40.98205	39.94088	38.24268
Hsd17b4	43.21485	41.34945	45.96628	43.30128	44.5986	40.44109	50.06373	38.33364	38.23578
Commd10	51.1736	45.13255	45.83799	36.3537	41.24521	44.06928	46.5573	38.63093	38.19761
Drg2	44.06456	37.90373	39.25247	42.45808	39.51017	40.63457	43.61586	37.93	38.1768
Zfand2b	32.24647	34.23402	35.16106	37.39484	34.89108	36.27167	34.52205	38.00338	38.17502
Plekha2	40.7788	39.55077	41.83877	33.28412	35.40575	33.98449	39.22344	38.86816	38.17045
Kif5b	46.77759	47.07484	43.94184	35.89232	30.03304	26.98552	39.28192	36.2037	38.16911
Agrn	34.02068	31.83515	38.06146	43.12725	38.71636	34.08938	33.58702	34.31253	38.15598
Ppp1r35	29.14575	35.57291	30.8478	33.10354	41.79889	42.9113	35.31973	39.26343	38.13876
Rps19-ps6	32.71933	31.33127	35.00614	25.94236	29.91921	46.54274	28.8737	33.22191	38.12581
Svbp	36.27429	31.55122	37.72356	36.4492	45.556	43.32059	29.39568	44.1664	38.1241
Ggnbp2	33.5566	32.24651	30.64138	32.0204	28.35414	33.59198	37.01794	34.72814	38.12046
Smn1	45.49807	36.27451	35.42664	31.85445	36.05968	39.56823	37.03123	35.66151	38.1101
Selenoh	39.64661	44.39992	42.00341	45.52197	41.5189	46.68439	37.15689	37.66749	38.10697
Snora65	26.7794	39.82788	31.01133	33.16995	35.79566	26.50263	48.55763	35.33742	38.06084
Nudt4	31.00041	34.63836	33.40488	28.04463	30.64293	31.17568	28.649	31.7517	38.00671
Abitram	41.78809	37.31808	46.94222	33.27893	33.18444	27.23926	36.44549	36.76533	38.00184
Mrps23	41.20848	37.05081	38.72997	34.61496	32.01569	38.77068	36.07652	36.77952	38.00106
Galnt1	40.90465	42.01905	41.53702	35.59817	35.69713	34.89928	41.92321	37.76384	37.99345
BC028528	35.15325	42.26226	39.61839	38.33581	44.90916	40.85677	36.77171	39.73853	37.9579
Gabpb1	37.13717	37.66936	39.42937	36.83235	37.96493	36.99894	38.28251	35.03384	37.94644
Afg3l1	42.24321	39.47916	41.21408	32.72891	34.55823	38.38224	39.48779	40.00874	37.91529
Ccar1	41.13439	40.55928	37.84493	33.56654	31.19749	31.36005	41.4154	32.97824	37.90697
Abhd2	53.94851	51.27177	57.81279	40.9611	34.81107	30.97588	49.34379	40.56414	37.89441
Coa8	43.36164	39.28533	38.82737	36.02632	40.13929	35.75226	39.03636	36.19149	37.85135

Dnajb1	33.25508	34.21086	28.21677	32.49034	33.81401	37.406	38.09846	37.49605	37.84925
Pi16	30.87871	22.88252	20.00168	27.27813	26.67287	35.66138	19.33437	24.71614	37.84855
Ctnnd1	52.9035	51.13288	54.42167	47.19058	43.30184	35.75455	57.75758	35.59403	37.77127
Clec5a	39.76145	40.42331	39.86971	18.4887	21.44371	19.90461	20.56302	32.86748	37.76922
P4ha1	39.22793	36.43385	38.97007	31.91975	30.84893	30.58788	37.48552	32.58828	37.76283
Fam32a	35.55743	35.42783	40.39653	37.63999	37.93492	40.09061	35.8633	37.47305	37.76246
Tssc4	45.68696	40.87278	35.53086	35.73457	40.65428	45.33979	39.74917	34.34431	37.75957
Npepps	41.64102	41.12073	42.50269	36.88184	35.30673	31.08065	41.85743	37.72604	37.74829
Cdk13	40.33969	40.83135	40.56282	37.03223	32.21742	31.42195	46.33505	38.84925	37.73171
ND4L	31.91888	63.85398	23.14855	87.23884	46.221	50.05906	22.57194	41.40847	37.71558
Taf8	36.92954	37.22397	45.07328	45.38122	38.96345	39.20923	45.49471	40.70124	37.6993
Slc35a3	38.01012	39.21175	40.14947	34.05867	28.37166	30.28901	40.24032	33.18622	37.6846
Pak1ip1	33.83841	30.43657	33.08326	34.49794	30.81174	30.91251	31.37826	33.54231	37.67641
Dcun1d5	32.31709	36.35473	32.39214	33.27028	32.65683	36.10472	34.06444	39.43414	37.66335
Bola3	38.73515	36.55783	37.50467	37.09636	34.53883	44.53591	32.44424	30.6918	37.66136
Timm44	38.29644	37.57394	41.00778	42.01076	35.37684	39.04031	40.83528	39.98672	37.65628
Galc	45.03628	44.60695	49.18965	42.93289	43.91849	38.12965	60.25151	33.93936	37.61649
Retnla	27.87274	32.60196	29.51574	59.4073	68.8041	64.91342	49.88421	54.05081	37.60148
Tbc1d17	33.1534	38.69589	40.12202	39.22488	49.00866	37.92728	45.9875	40.32685	37.59539
Brip1os	53.58098	48.85009	51.22338	42.41063	37.3831	41.89991	45.65083	34.19709	37.59159
Cdc16	42.58671	38.18622	44.94063	40.58549	33.67969	36.96497	41.31319	41.17961	37.59016
Nek7	40.87119	43.44926	42.89333	37.3844	36.81075	35.1047	42.60621	34.37627	37.58616
Tmem128	37.99755	33.5927	34.52544	29.0239	30.88005	25.99395	25.76862	31.66948	37.58595
Pot1b	30.19231	41.22118	39.50375	42.48427	44.14424	36.08709	51.57957	42.38645	37.57637
Hipk2	52.88188	48.78888	52.96918	51.59043	41.38617	32.48143	61.96683	36.68633	37.57527
Ang	32.18315	35.89394	42.98507	37.37109	46.47068	47.36313	57.87027	42.10736	37.53368
Tnrc6a	43.07967	42.82301	45.2978	40.21324	35.13745	30.88728	50.22269	34.6462	37.51899
Itgb1bp1	36.92489	42.1887	36.67555	42.16524	41.7365	37.47726	35.17563	43.55882	37.5131
Ap4s1	30.54227	29.82102	30.93085	34.96514	30.67697	30.55868	34.21964	37.69304	37.48765
Cbx1	41.68903	45.98561	35.80075	39.64885	40.77979	37.19042	50.19285	37.02721	37.48694
Ero1b	47.60188	44.29653	48.77713	42.10161	34.80921	34.58385	46.41864	43.53997	37.48223
Ndufs4	27.71903	25.80703	28.48127	33.9965	32.00958	34.08913	25.8476	34.71806	37.47529
Matk	36.10205	46.9908	47.12171	37.08634	38.92877	37.14905	36.96543	40.78116	37.47023
Pigu	46.60063	48.84818	43.09195	46.02195	41.88058	39.09056	44.67924	46.39586	37.44622
Tbkbp1	36.54138	34.04668	35.99469	37.63147	34.80161	33.25202	32.67763	41.14839	37.42052
Txndc15	35.83325	38.59833	35.65586	31.87793	37.72317	36.66273	32.90789	35.7068	37.40486
Aff1	43.09358	32.43572	39.18486	36.40038	32.96678	24.15724	46.91274	33.09901	37.40059
Tspan13	38.83259	38.32668	42.03749	35.80566	53.01826	51.77393	45.64771	37.30181	37.34063
Glo1	46.19839	48.96278	52.24105	44.6533	45.93479	44.88744	48.38498	42.7538	37.33843
Lsm10	33.56361	34.49689	41.15609	34.1913	38.67123	30.7225	41.18956	39.115	37.33148
Ppp2r2d	42.4143	37.19604	35.69762	32.79257	34.2594	32.55737	39.33839	39.42108	37.3048
Ift20	38.21127	34.06983	36.11702	34.21206	38.76236	41.92553	34.55029	36.79905	37.30246
Ostm1	42.26484	39.61657	42.74884	33.344	33.46193	32.18481	42.19753	37.43363	37.27943
Dnttip2	38.7582	37.50456	40.93157	35.94874	31.22215	30.70069	38.31789	39.05419	37.26983
Cdkn2aipnl	38.93156	41.26022	40.48282	34.3364	37.81445	36.14021	38.85444	36.07557	37.24696
Dtx2	39.60237	35.59439	38.45133	36.19458	33.91516	31.42586	38.35248	34.27985	37.24398
Dhx40	33.68698	38.83078	36.02941	28.52356	38.50492	31.17781	38.27361	39.47683	37.23754
Ccl8	14.64606	12.33486	16.16679	41.85808	39.36647	34.51268	32.06894	35.53262	37.224
Rmnd1	33.67927	38.62546	35.14088	33.69767	31.08425	35.14102	42.55655	28.74538	37.20944
Btbd7	43.21278	40.40216	45.88944	42.00192	35.229	27.37748	48.98265	37.8813	37.20184
Akr1b8	49.76494	50.00688	45.39024	39.44791	26.22053	31.63225	37.25017	29.70751	37.17304
Tox4	41.63451	38.32381	41.22892	34.87205	37.24801	32.33824	41.77176	37.01166	37.1687
Lin37	32.38516	35.29061	35.11115	35.18661	46.92621	42.48309	44.60977	40.30084	37.16619
LOC118568626	41.04302	36.61819	41.86189	40.89832	30.56117	31.89354	41.97721	41.80992	37.11684

A630001G21Rik	38.14667	41.51173	42.06742	49.046	35.11526	38.77605	49.84017	39.54496	37.11198
Tmem205	39.77412	40.81693	36.55344	42.22371	45.92455	44.87671	48.01287	50.88172	37.10327
Atg13	41.12277	39.92234	40.73118	41.64911	33.12558	31.28527	54.21764	36.91905	37.09346
Prcc	39.31701	39.37393	41.13102	37.32453	40.83984	43.20145	45.32487	39.07908	37.08655
Nudt9	41.54139	42.1349	44.07318	47.22049	46.28725	45.28848	46.2762	43.93822	37.07825
Srprb	41.57391	37.11911	40.31822	34.24505	33.22199	36.62935	37.5665	35.50596	37.06214
Hyi	34.52548	35.67973	30.84635	37.53642	44.46858	43.23975	36.15959	44.07931	37.04259
Mpc1	29.85126	27.09645	27.79104	26.33263	30.65725	35.81084	32.70109	31.76116	37.03862
Ogt	34.80871	42.24405	37.83021	42.82985	37.82208	32.20972	52.2073	36.07479	37.03698
Tacc1	46.42345	43.87382	47.80624	37.52834	37.44195	31.6255	44.13582	39.1079	37.02624
Cops8	37.6707	34.69776	37.77218	28.57749	34.61345	35.94403	34.88723	33.92498	37.01394
Elf2	37.56646	36.96234	37.19169	42.95675	39.2508	36.19267	46.01006	39.27318	37.00855
Eapp	34.70012	34.28009	32.36316	31.44311	35.66839	29.88699	31.83222	32.72513	36.98618
Necap1	35.47269	32.2468	39.59979	36.44184	35.15342	28.10508	41.39428	37.24211	36.98389
Gm10080	28.21644	27.23935	23.69733	36.69173	27.64821	36.46424	22.15274	29.62018	36.97756
Kdm2a	42.38469	41.57093	43.85739	34.36735	36.2326	31.83663	45.77218	34.83407	36.9586
Tmem171	24.46377	18.29898	26.14376	38.05957	34.76065	24.0829	26.71188	29.86624	36.94857
Qdpr	35.63307	43.27253	39.81345	35.86848	34.05018	41.15366	42.04868	36.5388	36.91795
Ramp1	23.61974	29.91656	29.54437	38.36927	50.38177	46.69424	39.00568	47.76442	36.89936
Fzr1	39.84457	40.03285	44.35069	38.86319	37.36192	41.69171	41.66434	34.02526	36.8971
Retreg1	33.59213	37.48778	42.53222	26.77509	29.29644	26.45999	30.25733	43.37683	36.89623
Pank2	44.46995	37.71159	43.15196	41.50479	37.23135	34.92813	39.78661	35.79781	36.89263
Yme1l1	34.27433	33.63877	34.81202	28.92054	31.30261	28.45446	32.56623	32.18218	36.87405
Sap18b	37.72003	38.03377	39.24723	30.6264	37.11063	39.3626	33.54902	36.37444	36.87404
Aftph	37.72848	35.85748	44.61832	38.09124	41.47185	35.15707	49.39175	38.77952	36.8665
Gm3699	35.88812	40.94559	33.09911	34.85393	36.17436	41.32384	31.17473	35.8974	36.8664
Tbc1d14	41.88493	39.61645	47.975	36.56052	40.3439	34.89382	51.11369	38.78036	36.85563
Dctn5	43.0622	47.02577	53.23686	45.91561	47.90474	38.51747	49.89265	41.44733	36.83443
Rgs18	32.44153	41.87741	35.38865	27.93809	29.85403	28.57682	23.76806	32.94017	36.8231
Prpf40a	39.22678	36.087	37.51984	31.91486	30.26404	27.1973	36.15811	34.05006	36.82015
Nus1	42.24539	42.52837	36.45831	31.19592	28.90535	30.56709	38.06207	33.58676	36.8167
Hdac3	35.61308	38.44136	38.85263	33.57143	33.54884	34.11007	39.20841	32.06436	36.81489
Ppp4r3b	40.52177	38.69364	36.90631	32.87842	29.68475	28.46441	38.59204	32.12745	36.79169
Dcaf13	41.56747	32.48481	39.53357	30.84674	35.32568	33.27667	37.83226	34.1757	36.78767
Arhgap15	30.67135	30.42612	30.78428	32.04899	35.90201	38.55166	30.93753	40.86217	36.73606
Zc3h14	44.92435	43.23307	45.14128	34.07756	33.13073	35.0628	44.92436	35.11367	36.71941
Eif2a	45.83221	48.61485	50.02781	42.65397	45.88856	42.26606	48.90134	46.5556	36.69399
Parp2	34.62212	39.94045	39.16778	41.27745	44.67128	39.26084	47.09209	38.76711	36.68699
Tnks2	39.70473	37.63254	38.95297	34.59403	34.9847	28.71223	42.72575	37.40419	36.65796
Lnpep	38.61181	38.12361	43.65671	34.873	28.96792	21.8441	41.68792	32.2551	36.64703
Raf1	36.50913	33.51289	35.51332	31.8417	30.34148	28.8164	34.60953	33.32115	36.636
Rprd2	44.73369	43.25266	43.46127	40.84737	36.49963	28.80099	50.67356	37.57894	36.61042
Chil1	13.42536	11.39117	7.181066	7.140011	11.22765	21.7732	5.07398	7.376827	36.60422
Pcnp	34.24851	35.85397	37.15482	35.62392	36.45391	31.23239	35.26827	36.9906	36.59991
Fdps	35.22209	28.86651	28.63519	21.03259	30.95822	23.18356	25.22033	39.25829	36.583
Arfgap1	37.19894	32.98077	37.64682	40.35335	31.17758	35.27196	41.627	34.6253	36.56348
Clec2d	27.46302	32.35723	31.14389	34.06705	38.16927	39.55896	25.7384	33.77014	36.55585
Irf2bpl	41.71337	32.9699	40.42379	41.03729	36.46596	34.39852	47.79479	35.26105	36.5313
Morrbid	46.54886	29.26582	34.50943	25.85291	30.49575	28.63902	20.94341	27.11927	36.53068
Stx16	30.66952	34.73852	39.05965	46.66537	40.34582	38.80237	46.4162	40.91011	36.49777
Ep300	46.69283	40.74746	49.52337	45.1743	37.66415	30.25587	56.85807	38.56755	36.49321
Pfkfb4	34.22434	30.83576	39.35809	37.07157	35.84586	33.68244	38.66045	35.01816	36.4854
Rapgef1	47.10571	38.71895	40.04108	38.55616	31.48147	26.78363	42.00707	35.63889	36.45986
Bckdha	35.34828	38.92865	47.55366	40.26556	50.34901	41.52819	44.7607	40.24043	36.43959

Ccndbp1	38.73418	35.19799	40.87673	37.83262	39.85777	36.18874	40.9325	34.25686	36.43363
Mmadhc	47.02412	41.57435	42.16004	38.60626	36.81259	36.41643	39.46411	41.51841	36.43257
Gm12191	36.81764	31.44382	32.58952	29.14184	30.02669	38.93973	26.46206	38.78101	36.40134
Rreb1	44.53591	40.00067	46.23987	46.98378	36.94334	28.21029	62.86927	36.5525	36.40057
Zwint	32.93925	32.18173	34.22538	40.28316	37.47654	36.47437	39.84988	38.59863	36.39845
Stxbp5	40.01346	41.22337	41.24392	35.30787	27.62049	28.354	42.4473	30.84363	36.39311
Gpr137	35.84323	40.49273	44.83948	38.08811	40.74132	38.85136	40.25445	42.60985	36.39056
Ap3m1	45.16481	42.12203	45.579	36.13614	31.36438	29.05257	39.0204	34.58274	36.38863
Peli1	32.53321	33.37697	33.81982	32.83136	30.88909	23.56944	37.80842	34.7971	36.35746
Tmub2	39.00097	41.07754	48.42621	38.11153	43.64753	38.71262	39.69575	37.80088	36.34822
Vps41	42.1031	41.73159	44.62268	40.60848	36.72946	35.00679	50.03942	34.40684	36.32674
Xab2	41.12224	32.54703	41.98475	38.85824	40.12778	38.29293	45.38838	35.27904	36.30082
Zdhhc7	42.54887	37.17772	41.39137	36.18066	36.92542	35.68179	43.56879	39.3843	36.26018
Arih1	40.82688	37.84841	36.56666	32.82808	28.5695	27.02393	37.27264	32.57175	36.2513
Smim11	28.82779	32.84254	43.13583	32.856	41.18291	46.24318	28.07602	34.51973	36.24646
Pdrg1	30.31276	32.84137	30.40651	29.64955	35.27067	32.74605	28.62569	35.00221	36.24241
Zfp598	42.54285	42.41073	39.56	39.82268	36.77174	38.3806	47.58548	40.46896	36.24011
Trappc3	36.01709	42.08415	45.3356	38.0099	37.07673	39.22608	36.44993	40.93163	36.22054
Galnt2	48.08679	51.10669	53.9471	36.85899	37.16456	34.67721	43.95635	32.72256	36.20197
Ppp2cb	40.30634	38.21597	40.54695	33.88081	33.41237	33.53417	34.62262	35.80865	36.19924
Zdhhc6	41.36412	38.84331	45.22899	35.93756	40.02595	35.00748	38.6419	37.15559	36.19102
Crtap	47.41669	47.19368	46.03123	44.80318	43.77294	41.69588	46.76774	38.00091	36.19084
Pmpcb	39.84177	39.21605	37.03248	33.53937	31.39466	36.78017	37.44475	34.36152	36.18501
Atg2a	36.4795	38.57787	39.4941	35.36679	37.05315	28.9435	45.72077	33.73401	36.1812
Plxnc1	38.6152	35.78201	41.72474	37.81695	35.92648	32.09601	49.46553	31.5146	36.17977
Rock1	31.80946	34.98662	31.21274	27.88008	26.4377	26.34217	31.02446	30.14922	36.17573
Sf3a3	41.95401	33.05055	36.70178	35.45304	37.96649	39.61296	36.84889	39.44697	36.15846
Alkbh3	41.49246	33.71231	44.76886	43.71396	37.49466	31.41852	48.28217	36.55432	36.15478
Dync1li1	42.54045	42.04886	44.60492	37.96417	33.03138	29.29207	41.39123	36.5298	36.10297
Acot9	35.53545	35.13177	37.19259	34.4119	27.57989	26.5253	27.97281	32.06289	36.10009
Vcp-rs	43.47043	37.07091	38.49267	37.45539	32.10638	33.47105	42.18605	31.81945	36.09522
Spr	32.0148	40.81393	35.59121	32.68981	32.77303	36.38061	37.62226	32.63386	36.02942
Bcas2	34.03206	34.83261	34.42455	32.56658	32.00476	31.25622	37.14131	32.62378	36.02718
Cdk16	43.78752	42.8892	45.62769	42.90452	44.11194	38.86239	49.80434	39.38332	36.01161
Cd200r4	38.57837	40.19943	37.57181	37.06515	30.99589	40.83296	41.45679	38.50015	35.99159
Oaz2	33.74491	40.77672	40.58899	33.22632	34.2617	33.96464	35.00161	34.59547	35.98112
Wdr45b	35.00356	38.07117	34.84205	32.90248	31.2647	30.75168	34.49125	33.84473	35.97632
Scp2-ps2	31.01754	39.13042	45.72146	47.1864	51.91679	46.66509	41.86702	40.44085	35.96032
Dnpep	36.67753	32.74354	39.92139	39.45829	34.7706	35.79044	38.04755	36.07712	35.95387
Aph1a	37.60632	37.67886	42.28557	33.70392	37.26314	29.1203	34.23566	37.30669	35.93383
Eif3j1	33.40528	33.15595	31.66288	30.49948	32.09928	29.46865	30.63176	33.86128	35.93293
Bmyc	37.74786	42.76321	41.17231	43.29113	62.13074	53.57837	48.59795	44.0167	35.91439
Kidins220	51.23335	48.05942	52.59582	42.76797	37.72	31.22111	53.10516	37.40324	35.91439
Ppa1	32.56772	28.88628	30.16833	25.43034	25.37766	24.00679	26.29854	26.75891	35.90653
Nufip1	33.90736	34.79527	34.23827	33.9032	36.02938	30.36308	39.92035	37.97492	35.8778
Mmd	37.59447	44.8918	47.77214	42.65218	38.67054	33.15918	41.26508	37.48847	35.87126
Polg	38.91441	37.04342	39.39793	38.62499	34.75813	31.11476	42.3733	34.92991	35.85639
Nt5c3	25.65154	22.48114	28.4359	32.93096	25.96367	26.42893	29.04612	28.11581	35.8527
Nsmce4a	33.8037	35.37049	33.49853	35.45833	31.67916	35.47632	36.29539	35.09023	35.82633
Hmbox1	45.83501	46.11136	47.10536	46.8195	41.37027	36.12265	61.90799	38.64799	35.81713
Rbm8a	35.07046	31.48573	33.58923	30.33807	32.75413	36.51305	30.12915	39.0869	35.81134
Gng12	38.85334	36.84701	38.67104	32.70502	36.29189	32.53721	38.80544	34.19745	35.8099
Mms19	44.84083	39.80997	44.03557	45.85007	40.76654	35.99093	53.81889	37.92503	35.76625
Kat2b	36.96752	40.40052	37.31537	31.95189	30.55172	25.87806	36.26871	34.81932	35.7622

Gtf2h5	34.80226	33.34383	38.44278	37.00212	35.21406	41.22352	30.69885	38.26055	35.735
Il18	30.75636	32.42107	36.45216	36.13492	38.15259	33.09859	29.97823	33.50358	35.72914
Sf3b3	54.54827	42.81507	46.79057	38.59824	34.11829	32.52062	44.12973	36.40382	35.67969
Lrp12	43.6552	35.24151	36.57671	36.32875	32.46283	30.61836	51.64625	36.40726	35.66338
Diaph2	47.0788	37.27133	42.15244	34.73062	33.47747	26.63409	43.80843	35.31316	35.65788
Kcmf1	40.8418	38.17428	40.06637	33.6833	34.27452	33.60114	34.59739	34.18152	35.6545
Dnttip1	36.46889	33.08546	32.68777	28.38293	32.65456	30.76043	35.66743	35.14592	35.64933
Ptpn23	42.8045	35.77923	37.4479	35.60147	34.25577	28.3431	48.65391	35.55101	35.63752
H2-M3	22.68098	24.21141	33.42234	36.86774	31.60506	31.26411	32.97148	36.15136	35.6328
Ranbp9	35.87914	35.81971	37.78333	34.4727	30.36264	29.4319	39.38303	36.22765	35.62731
Fkbp3	35.02278	34.16893	27.86893	37.51005	30.89696	35.64304	30.6802	34.68562	35.61632
Ppp1r2	31.73332	29.05668	31.39678	26.53536	30.57239	29.28306	24.37345	30.40757	35.60105
Parp10	23.80249	20.24461	24.15905	33.46849	28.89404	25.67264	28.93628	24.07368	35.5942
Agpat4	49.4879	46.31663	45.9812	27.62149	30.15878	25.92895	29.06899	36.33976	35.57534
Usp38	33.26284	30.63714	32.18662	25.29661	30.10206	27.52015	38.42191	35.23401	35.56803
Myg1	32.25119	32.4882	35.41164	33.80082	35.61015	38.05761	35.46621	35.36811	35.55918
Glrx5	31.59195	33.93577	30.59961	32.95548	29.71897	37.55515	36.7119	35.08296	35.54132
Slc31a1	38.89853	36.2901	37.78497	34.90395	35.15045	31.49687	37.76603	37.10812	35.54118
Fmnl3	36.32697	33.50737	35.7745	38.78563	35.08968	32.18459	44.0796	35.53321	35.5323
Sts	53.81565	59.9726	51.33623	51.6606	57.99299	61.38679	70.14189	48.80571	35.532
Ccdc9	37.18756	31.66792	33.30103	30.49366	33.9731	33.88727	35.95169	33.00947	35.52484
Sav1	38.28389	42.12503	42.06665	37.19579	36.54483	32.76319	40.86229	37.60019	35.50405
Snrnp200	48.69693	43.08731	50.82686	38.312	34.9986	31.10929	49.61024	37.09608	35.49329
Atxn7l3	42.52873	37.20343	39.3715	31.16399	35.19907	34.6176	36.5224	36.91965	35.47475
Fpr1	23.25633	24.48996	20.49309	9.708778	16.88119	13.5628	7.925574	23.41898	35.46456
Ankrd11	42.49968	40.87879	40.50833	37.44585	35.32788	29.29824	51.06834	35.50401	35.45988
Setd2	44.7067	38.15064	43.60124	37.86327	37.05955	29.8026	49.79767	33.69829	35.45199
Osgin1	15.72711	13.19241	17.41418	14.82914	21.87906	18.56949	20.14428	23.31627	35.43907
Il15	19.8399	24.06398	21.13402	32.41889	31.09853	27.80766	23.38912	23.16919	35.40549
Tm9sf4	47.12591	44.78599	45.5798	39.38893	38.60601	33.31393	46.44154	39.50216	35.39224
Far1	38.52782	36.79276	40.33867	30.89207	30.88788	29.08388	35.31685	33.32894	35.38632
Ripk3	37.77034	32.27992	31.8353	40.60008	30.82511	31.35905	40.17305	37.85524	35.37587
Trmt2b	41.74634	37.48507	42.82518	31.90188	31.32121	30.72319	45.08923	32.33405	35.37419
Cnot8	35.07737	36.40438	38.1148	32.5992	34.21108	34.07257	42.11485	32.64377	35.36737
Gnl1	30.42628	33.58936	32.63224	26.4588	33.01837	29.85615	30.96682	30.373	35.3533
Plekha1	32.85838	34.17108	34.08241	34.26981	30.46624	29.69088	44.73977	38.52468	35.34706
Hook3	39.67709	41.55214	49.44365	30.84116	32.43573	27.5484	37.14326	30.2936	35.33904
Rxra	42.49546	37.71617	46.82686	40.74948	42.98003	38.49402	56.49612	37.16933	35.33697
Ddhd2	49.44627	55.45802	50.85213	45.16002	38.82564	35.4211	53.47814	43.55887	35.33167
Snapin	42.0415	39.10156	45.48996	39.82072	37.69754	32.00198	42.79407	36.88712	35.32605
Ckm	43.41016	69.78712	35.46097	57.65988	45.26128	74.99893	25.38929	40.13706	35.32451
Cln6	45.21489	49.01802	53.4514	38.5275	42.37199	35.01596	48.44383	41.96477	35.3236
Zfp513	35.16469	34.48701	33.7464	36.29876	38.69596	32.90603	48.53614	36.02908	35.32239
Man1b1	37.20592	37.21474	34.86143	30.77493	35.49338	32.44934	39.98724	32.02548	35.26911
Hcfc1	50.7853	45.41183	48.91762	41.09839	33.91879	29.11116	50.97537	36.47036	35.25523
Mapk7	40.11206	36.92222	41.06656	43.8687	42.84898	41.76131	49.50301	45.47767	35.2486
Pcmt1	36.34509	37.56645	35.71202	29.68766	32.05228	30.55527	31.78344	32.26719	35.22493
Cdkn1b	28.53735	34.18583	28.33318	31.31152	38.94583	34.74671	39.19136	36.30029	35.21761
Cfap20	28.73446	28.61487	29.34281	32.13982	30.45282	32.24691	30.77032	30.15553	35.2112
Smg5	43.56822	39.97484	36.15946	35.81706	33.92855	32.72028	42.11568	34.66872	35.21048
Itgav	46.34638	38.43295	46.50108	38.73383	28.61078	26.52388	40.16139	33.95884	35.18499
Abhd5	32.30623	28.66304	28.6398	24.00682	25.60408	25.07126	27.21069	28.42284	35.15288
Sh2b1	38.53738	40.2632	43.27842	44.94897	44.7415	39.57637	50.17276	40.96414	35.14606
Prpsap1	40.86628	36.25847	36.30012	36.32868	34.73741	30.29935	35.51986	32.29677	35.13065

Mfsd14b	36.48235	33.84596	36.88086	31.65545	29.75495	29.56039	32.66411	31.85715	35.11534
LOC115487878	29.85126	30.93173	22.87571	41.24927	30.27612	33.34173	44.47607	32.11241	35.10177
Phlda1	41.92628	33.83109	27.45255	22.13005	35.60459	25.67307	36.55572	43.3773	35.08966
Gm14303	28.04902	27.15157	33.1677	42.6582	43.8417	34.33084	31.03642	39.74394	35.06768
Gsk3a	39.37918	34.06562	35.66883	28.85516	31.54617	30.68295	37.7272	33.10538	35.05635
Zmpste24	39.10684	34.60943	36.01119	29.91617	29.83323	31.44239	36.38879	29.21831	35.0487
Pi4ka	46.81648	44.28928	44.28586	40.79668	33.44817	29.03882	50.90972	32.02961	35.0336
Raph1	45.28009	48.59682	48.66418	47.25861	36.88344	27.07002	60.97726	34.84291	35.03044
Pilrb1	23.91446	23.20032	32.28827	17.99752	29.55687	24.0253	21.68336	28.04716	35.01773
Brd9	35.58298	38.65883	39.35221	36.74244	33.30455	35.09804	42.10885	34.96706	35.00345
Kremen1	36.55486	35.49923	36.30842	39.59045	32.50675	29.63232	36.09731	30.66713	34.9993
Tcf3	36.72409	36.58503	37.37018	34.6657	35.09392	32.64036	38.8976	34.09771	34.98159
Phf12	39.02292	37.21205	38.23383	38.23186	36.24625	32.59069	48.45324	36.9018	34.96789
Brcc3	32.10341	30.36603	31.47377	27.99974	24.77544	32.12095	30.10914	32.28172	34.96183
Runx1	41.55377	37.23261	39.1303	36.81738	35.00715	33.44202	49.66715	39.48778	34.95731
Thoc6	35.75659	27.43479	29.54482	36.54821	29.01183	30.19276	31.40486	34.00725	34.95678
Mtpap	40.67185	32.81257	37.90065	40.11282	30.9108	31.10951	42.26588	33.12118	34.95451
Pole3	36.0992	39.76873	36.1034	41.20828	37.01165	48.03124	44.92468	40.77051	34.93237
Dnm1l	43.4933	43.96259	43.66133	37.80497	35.46291	29.8683	45.32302	39.74976	34.91431
Mrpl38	44.12257	44.24021	47.27125	38.17122	40.52508	38.98647	38.86194	37.6697	34.90039
Ap1g2	39.36506	36.52856	36.7365	36.29422	40.28143	37.24789	46.89615	36.20582	34.88766
Lsm7	34.58956	27.76338	23.57576	33.33566	34.86062	35.16417	28.15928	34.17327	34.88623
Alox15	11.23	12.02465	18.7418	46.51813	55.48884	66.3885	55.37541	43.33672	34.86743
Ppp1r12a	35.7169	32.97769	34.43966	33.67434	30.88132	29.46621	35.17334	33.01133	34.83981
Csnk2b	35.87169	30.23509	34.42633	26.60293	32.55339	29.43128	33.89196	36.05077	34.82203
Cdkn2d	39.1354	33.41682	34.95121	34.34834	31.39608	38.7733	29.23083	31.558	34.81002
Clk4	27.83297	29.1874	28.65013	26.83542	29.94918	28.59895	40.94637	35.23997	34.78023
Arhgap18	35.0474	39.75532	42.4727	40.41567	40.24141	35.5866	45.85742	34.06922	34.77683
Tspan4	39.99854	45.34919	41.99699	39.90102	38.53854	33.77293	34.50155	33.59986	34.76677
Lbh	36.26603	36.27761	42.03518	35.2543	35.72683	29.32668	31.97333	37.71159	34.75419
Tlr9	40.93568	31.12578	50.30099	47.04343	46.33378	30.07214	41.12052	39.35495	34.73781
Zzz3	40.75521	39.82556	36.50554	39.05321	32.64627	31.62335	49.4408	36.18996	34.73667
Esrra	28.76816	28.7191	30.56627	31.6544	31.3163	31.81233	29.04849	32.33189	34.73375
Pnkd	33.64637	40.0769	38.53219	49.66678	43.25169	50.62251	37.02748	37.67805	34.72647
Cyp4f16	34.08434	32.79989	39.17571	30.56629	38.62744	30.48875	38.40485	39.6775	34.71505
Pitpnb	36.10796	37.68098	39.39698	28.47909	33.41255	29.03862	38.16263	31.94833	34.71317
Gab2	40.96231	36.39687	39.42515	33.84766	34.66859	32.08374	44.60752	37.94537	34.71166
Ndor1	43.35859	43.03118	45.66643	44.17247	41.93525	38.13114	52.90455	40.10119	34.70725
Gpr146	36.83327	31.4337	40.57776	40.71999	50.28972	46.28638	52.65485	37.80094	34.70186
Ankrd33b	23.23351	22.92902	17.64005	13.78944	23.05414	17.99842	27.87895	26.75315	34.69356
Slc22a4	36.85703	34.27451	33.6582	38.09133	30.50263	30.18217	43.82076	33.37323	34.69196
Ipmk	40.60771	47.51108	41.18161	33.68904	36.12368	33.15662	42.2815	37.83381	34.68252
Kmt2b	44.96387	41.3354	42.64345	42.39965	34.93815	30.55095	48.45105	34.99427	34.66768
Wac	37.4476	36.07388	34.60106	32.42726	30.38145	27.89243	39.20518	36.57723	34.66503
Abtb1	22.82384	25.5398	27.50806	33.64848	36.97651	35.08364	33.89816	31.69278	34.64174
Unc13d	35.17932	36.00208	37.69964	32.785	36.15058	36.09353	38.77904	34.70229	34.63414
Rps6ka3	47.35501	44.1351	46.42498	34.16253	32.18628	26.63923	44.92778	34.61994	34.61025
Slc8a1	39.79919	37.55396	47.40964	39.16905	39.65002	28.79846	52.42072	33.80826	34.60543
Ino80b	36.30704	35.88542	42.17693	39.66775	45.48394	44.38008	39.11274	40.72803	34.55947
Wdfy4	46.22988	36.40342	42.86197	35.88613	35.27542	29.56573	43.40112	30.41766	34.54816
Zfr	39.26233	38.91927	41.97366	32.09513	32.18988	28.41591	36.96268	35.72764	34.54107
Nfat5	44.46093	44.70593	42.29157	33.00406	32.48284	23.91101	47.22804	34.31924	34.51177
Nop58	41.18577	35.03222	34.34417	29.43296	27.44138	29.7849	29.89587	34.88558	34.49405
Ogfrl1	25.15897	21.65196	24.92317	24.54332	25.24558	24.0609	21.58698	27.55597	34.48934

Numa1	40.03042	42.2814	44.17423	40.7079	37.82928	32.99805	50.52926	33.86254	34.45917
Zc3h18	46.01225	41.30078	43.51567	41.72693	36.58888	34.43989	42.1078	36.63441	34.45856
Sars	38.51053	39.6563	38.22496	34.42435	33.37709	36.06502	39.89721	34.44231	34.44905
Adgrg5	40.11965	33.52427	42.76641	36.93466	56.42241	43.84711	62.61621	44.42962	34.44202
Ubac2	36.48442	36.06599	39.01253	35.21221	38.58261	38.05952	39.65077	38.11073	34.44026
Acads	34.53762	38.70333	32.70336	36.96787	37.63665	38.57391	37.93312	38.64128	34.41495
Snrpa1	40.65458	35.86792	40.99102	33.45346	31.4084	36.6759	37.41511	45.3839	34.41205
Coa4	35.09748	30.96001	35.76636	35.09918	43.61899	37.80509	37.92386	34.93516	34.40133
Mtmr14	38.51579	41.31402	38.57903	37.3944	45.3279	38.06338	48.43154	37.39807	34.38491
Med27	36.25982	32.10414	34.7222	34.27841	29.71757	36.38331	31.86763	36.34299	34.38251
Ifi208	17.28604	7.140836	24.31238	39.83693	23.86653	15.92253	19.45172	22.17123	34.38005
Arid4a	30.65208	31.74058	30.9119	32.39798	31.87695	29.15669	38.34269	31.61042	34.34238
Cenpa	40.3441	56.18586	54.32215	37.16086	39.49207	43.48387	45.75721	35.79378	34.33361
Ss18	41.08446	42.09005	44.56325	33.7872	31.08596	30.92289	41.92638	34.34567	34.3053
Mrps5	34.48449	30.61411	34.87514	34.29132	31.33306	34.82178	29.72784	32.86942	34.30378
Tnfrsf12a	27.46118	21.79473	21.98439	19.93565	19.95296	24.97795	38.37634	34.92235	34.28287
Ciita	47.83671	42.14412	44.71498	50.91866	66.50419	55.43715	52.0128	38.98238	34.28092
Tspyl1	30.5773	31.15216	29.7171	35.62023	35.74089	30.00739	37.27771	32.852	34.27767
Ptgr1	31.24772	34.70595	34.0036	31.02854	26.54726	32.63216	35.1862	32.32873	34.26605
Faim	31.45306	26.36763	31.94738	34.90868	30.52663	34.68236	36.62114	35.55161	34.2378
Fbrsl1	33.68185	31.8956	35.40497	34.95327	34.99442	32.32362	42.76993	33.67099	34.23508
Ppard	36.8121	34.38728	42.55399	38.5075	48.57326	41.84154	47.21798	40.32879	34.22857
Supt6	43.78009	39.80025	43.83163	36.96722	35.66206	32.12802	44.15441	34.5191	34.22744
Gcsh	46.17463	44.20395	41.04889	29.33521	33.79654	34.00253	29.36093	37.04271	34.18358
Pam16	31.63585	33.75523	31.53908	41.14591	34.05127	36.6859	32.80103	35.37955	34.17201
Nckipsd	36.50866	34.95501	38.55212	38.57831	35.00663	33.53274	42.7172	32.79229	34.16573
Mtx2	28.73261	29.13957	29.92593	32.42796	30.38648	32.72473	28.7401	32.18136	34.1469
Axin1	39.16176	36.91278	37.84433	34.8814	32.37438	35.43105	41.25049	34.86273	34.14687
Zfp524	36.12254	37.12718	39.18747	35.27164	42.28666	35.5856	37.54186	37.54482	34.14109
Gm34084	15.98397	19.37184	22.66345	34.30381	30.44591	30.8727	15.5792	37.7568	34.13219
Hadhb	38.62622	35.19724	34.06502	39.9593	33.32456	33.3693	35.91014	27.45834	34.13067
Tarbp2	32.37296	28.71636	27.56881	31.83564	28.34759	32.01847	35.09767	28.09547	34.12009
Tmem154	37.58906	37.6048	40.43036	39.5394	34.81991	35.62025	45.00517	39.13673	34.11822
Snhg3	23.97665	32.51636	26.26319	31.53749	38.17931	30.61965	23.07654	40.10018	34.09644
Ldhb	26.26261	36.36051	38.67019	24.43364	41.56771	34.62721	33.60188	41.8234	34.07564
Sephs2	29.81607	24.94536	31.80242	31.06452	31.59201	29.07737	29.87062	29.47687	34.06207
Htra2	26.16034	31.57166	29.76325	34.70637	28.98567	29.92826	34.34559	30.78212	34.06098
Tbc1d5	41.96869	38.04903	49.1712	37.48491	38.08022	34.03347	49.32733	36.20771	34.05045
Smc4	37.92966	46.12225	40.98821	30.63159	30.32191	33.09208	35.12335	26.46579	34.04579
Hhex	32.83711	35.24729	36.97344	33.80491	36.52077	33.92737	37.43297	33.41002	34.04149
Rps11-ps1	30.75396	34.60699	31.26342	34.4044	43.64556	37.42145	32.51766	39.97213	34.03577
Sod2	34.19937	28.62843	25.75038	26.59788	25.77798	27.21569	32.11026	34.72821	34.03204
Farsb	43.41635	36.82356	36.23517	34.40661	29.47878	32.38075	34.47592	32.101	34.03087
Ccny	40.48648	42.82266	44.93101	35.59804	37.34123	31.85738	41.8815	40.79568	33.98382
Atp13a3	41.11453	40.67625	44.77083	31.00013	30.35338	24.5835	36.8146	33.63429	33.98353
Sorl1	28.73795	25.86414	29.36039	23.44009	26.47756	22.24457	26.34463	28.02374	33.97361
Aldh16a1	32.57951	32.64666	31.23851	31.08184	38.29211	39.8316	36.45201	31.62751	33.95039
Auh	33.27453	35.69385	38.54212	31.45991	42.38369	36.61183	38.19867	38.58465	33.94301
Egr2	48.15423	42.37591	39.10723	39.90021	34.72578	24.24516	43.01203	40.89982	33.93935
Ercc3	39.77137	36.52977	38.16345	33.45597	30.78852	29.22465	35.43977	32.62125	33.9358
Ube2j2	33.02618	35.35138	32.39415	29.48682	29.99041	31.19487	33.76913	31.61231	33.9176
Mettl6	25.53069	29.93168	24.6999	30.36159	26.10157	29.77748	35.27274	30.70709	33.91724
Arhgef7	42.91307	37.46196	40.23568	34.98229	33.43002	31.93074	39.95251	35.08088	33.90131
Rab3gap2	38.74276	32.77274	32.85199	32.11277	26.92166	26.1632	44.20381	30.78117	33.88073

LOC115485637	44.0119	78.92609	39.36137	21.46186	36.67689	19.17635	42.86416	42.70948	33.88047
Cd274	19.71269	17.30347	17.5227	26.89902	22.00189	19.36544	39.33413	29.23314	33.84201
Jpt2	47.52442	40.71466	41.57274	33.86768	31.80883	35.5488	40.12619	32.83207	33.83945
Dera	34.28752	28.92268	35.07982	29.45795	31.99821	29.33904	36.21858	32.14477	33.83722
Sptlc1	41.65756	39.56074	40.46121	33.4411	34.91812	27.44588	36.94643	35.65976	33.82919
B3gnt8	25.7834	27.3641	35.7852	36.51498	40.98604	40.14497	38.69072	36.82248	33.81594
Ms4a14	33.55097	36.25242	40.84218	54.67295	44.80361	34.93407	62.08	33.38142	33.78583
Igfbp7	41.97453	32.82872	35.04885	74.71748	48.22587	64.8624	23.02152	43.40416	33.77819
Atg16l1	36.60846	41.24778	35.85608	33.81211	29.46154	30.66715	38.44483	34.2946	33.76947
Gm6169	44.50096	45.88359	46.41282	23.19846	28.19111	27.69831	26.60079	30.52307	33.75856
Wdr41	36.93435	34.52239	39.49622	31.60524	33.41882	31.08138	39.91037	32.69557	33.75674
Pold3	36.77172	35.94074	35.15097	31.43	28.79421	29.17554	43.5033	29.651	33.7562
1700123O20Rik	31.75578	40.63581	37.41158	33.2906	36.90776	35.58852	35.88079	35.51287	33.75438
Naip2	35.44002	37.16093	40.66162	33.32783	31.9309	28.96789	37.06038	34.20595	33.73329
Cacul1	35.95848	35.60424	36.10224	33.6951	29.40429	24.8178	38.65889	33.03057	33.73004
Erp44	37.61297	34.5233	37.10525	31.83331	29.30998	31.88305	36.35011	35.41514	33.72362
Dusp7	42.58361	35.77619	44.02083	44.83294	40.90125	33.81126	48.91641	38.98313	33.712
Stk16	33.00832	34.86393	36.67133	37.41079	31.77764	36.70362	35.22553	29.72327	33.65826
Cycs	31.84964	29.5385	25.29634	24.66231	23.35258	26.07257	23.52597	29.73825	33.65578
Apeh	35.20769	33.09143	36.97115	35.93372	34.27604	34.07281	36.8182	30.79947	33.6492
Rexo4	33.07673	32.79181	36.52118	32.13436	33.65038	33.37856	39.78219	29.88143	33.64823
Kat7	44.03084	38.92883	45.70219	33.08328	34.32476	33.53368	41.92366	36.56412	33.62085
Cpeb4	41.79891	38.09265	34.83779	39.62389	26.48925	23.19551	48.66751	29.63581	33.5972
Ubap2	49.53224	45.09151	50.66116	39.80848	32.03652	30.56983	48.12158	34.63063	33.59632
Inpp4a	35.56404	34.50066	37.32417	34.60329	30.98894	25.78915	47.7419	31.22844	33.59305
Atp11b	33.78896	28.98362	35.83717	24.95674	24.86731	23.58727	28.47448	26.78892	33.57606
Cpd	49.59259	45.80555	52.56687	34.23183	29.18577	25.79054	47.76921	28.31175	33.57184
Twf1	44.66884	41.18767	45.59187	40.6938	41.56106	36.21625	45.7029	36.19879	33.54104
Gtf3c2	43.28091	41.0153	39.31431	40.99133	35.3963	33.80651	48.71149	32.57876	33.52986
Cep20	31.54938	35.71502	31.55305	27.00032	28.70897	27.83446	26.14251	26.58545	33.52561
Snapc3	32.12954	36.06407	37.0409	43.33523	33.71544	34.46638	41.23766	29.03314	33.51751
Abca7	40.27254	37.94444	40.63608	32.94219	35.36994	28.99589	38.47136	33.48014	33.50493
Arl2	36.68226	37.28448	40.19567	41.74213	34.14593	41.82805	37.91295	33.58884	33.50219
Tsen34	29.77004	38.44004	35.01331	32.87458	37.0944	40.52565	32.15405	34.9563	33.48304
Npm3	36.2727	29.95435	33.96137	29.4184	34.91323	37.76658	29.31371	40.10733	33.46777
Slc35c1	47.9097	47.88643	50.42501	37.8053	40.62963	34.98404	45.69143	38.72573	33.46435
Krcc1	30.66195	31.06518	31.22118	33.82237	28.16036	34.45656	32.69557	33.42504	33.41018
Jak3	36.18015	30.7186	39.0779	33.38519	39.78193	34.72637	37.30055	30.68815	33.37704
Ltc4s	35.43034	46.04075	53.56856	28.08188	43.96575	36.90866	46.5277	40.97586	33.37099
Mospd2	36.62329	35.56888	38.0786	32.71815	32.89605	26.64	40.94788	34.24511	33.36629
Scly	38.57817	36.72063	38.08363	44.7601	35.19073	40.72733	44.32385	33.52974	33.3644
Vps9d1	38.1211	38.51363	36.22322	37.90756	38.57053	35.78456	49.57772	37.06846	33.34912
Cks2	28.99714	30.43366	24.84617	24.22751	33.09423	34.35063	27.74082	30.49823	33.34695
Setd7	36.81764	43.08577	36.68084	37.34785	32.07549	26.54685	41.73423	34.71824	33.34268
Nt5c3b	37.27	34.20727	35.57618	31.65823	30.64421	34.30911	26.93796	32.33229	33.33692
Rnaseh2a	35.20131	35.37315	35.25866	29.81441	28.45343	31.6666	32.80462	31.08132	33.29619
Mllt6	35.3821	36.65919	37.60472	34.19948	28.91352	26.68481	42.91666	33.30322	33.26799
Dxo	35.13248	36.94947	34.71508	40.07889	43.23866	46.94491	40.70653	35.6382	33.26529
Frmd4a	31.85592	25.95306	37.56994	39.70585	29.85808	24.6905	38.64857	28.34244	33.25049
Klhl9	27.50212	30.58873	31.18671	37.51861	33.80513	29.65168	42.35188	32.93115	33.22855
Nupl1	43.41106	37.80666	39.90486	28.87295	31.4939	29.92243	38.07608	31.95858	33.21273
Chmp6	33.70322	29.75272	29.69353	31.04234	27.76916	32.05443	33.95301	33.47511	33.20531
Tmem248	40.72224	37.45075	36.1436	31.56902	30.37128	29.37316	39.71198	32.32473	33.20474
Mfsd6	46.8964	43.20548	50.03084	31.85182	32.28771	31.22886	38.80319	31.86807	33.19495

Rnf31	34.16419	31.41504	34.83052	34.43864	32.75298	30.32424	40.36905	30.6206	33.17733
Yipf5	34.75293	37.69913	34.20943	30.08462	33.14365	30.20131	35.42685	32.68026	33.168
Snx29	40.13538	31.54589	40.24253	40.01246	31.03504	37.2423	56.3095	32.35894	33.14139
Ctnnbl1	35.53564	35.86988	30.0414	29.47261	30.47986	32.86225	35.55333	35.50023	33.13681
Pigx	35.77558	33.208	36.45589	32.15772	40.18918	38.03373	30.57058	38.78224	33.13077
Akr1b10	33.99583	34.95568	42.21535	41.26186	40.24088	37.126	38.05354	33.77115	33.12375
Gm13841	29.75536	29.84193	28.51194	26.77859	28.33868	32.35917	27.31749	36.79737	33.11249
Nfatc2	47.02557	38.60928	45.19447	50.41429	38.94902	30.20048	63.04319	37.09422	33.11207
Ppm1a	36.8527	35.08933	37.0359	32.53493	31.17938	29.87074	35.91141	32.57032	33.07784
Pdlim2	34.97461	35.2695	43.46071	30.71667	36.75524	34.07033	27.02053	35.18831	33.07436
Tnni2	37.59681	42.53701	34.3082	57.94482	37.92165	69.74914	24.61282	31.93194	33.05986
Kctd20	34.67226	31.56987	35.57172	32.10866	31.49487	28.73858	36.22356	34.07006	33.0595
Mad1l1	38.79547	37.50395	46.43338	40.28123	31.02672	36.08342	41.63629	37.50427	33.03668
Msl2	33.91598	32.24241	33.53434	28.08809	29.09377	23.08028	36.40164	30.66112	33.02743
Tbk1	33.43477	32.2695	36.56667	36.73956	29.75256	27.28284	42.47727	33.47011	33.01684
Phf10	38.42374	40.3471	41.0922	35.46041	36.30895	29.8059	36.78773	36.51976	33.00354
Dusp22	47.89205	46.40988	49.65855	40.57026	45.56395	42.70731	41.17969	41.52668	32.98741
Ireb2	33.77084	34.47394	41.72696	32.57136	32.16297	27.61494	37.71167	30.68112	32.98654
Khdc4	31.89086	34.6118	34.9973	30.40924	31.14329	27.10382	34.05617	31.15211	32.98435
Tbrg4	39.80774	34.63896	33.72008	35.45254	31.07652	29.06171	39.89874	34.03435	32.96712
Klc4	30.74165	38.58181	39.08767	33.22563	33.20555	34.38772	35.30875	34.11006	32.93963
Timm50	38.74552	34.89068	33.34195	31.00047	30.18343	33.30858	32.02662	33.80048	32.93935
Tada3	35.54196	39.31767	41.95301	38.12987	36.13771	38.42206	41.22273	41.6395	32.93531
Tfdp1	33.86621	36.69441	34.50536	26.63896	27.41466	29.87101	28.74278	29.47957	32.92609
Fbxw2	34.43391	35.62807	37.93006	30.87569	33.02471	31.64469	35.4226	34.95816	32.92098
Gm7027	39.95323	34.54181	38.48014	39.25926	37.657	38.25924	38.91133	34.4943	32.89663
Tusc3	35.70386	41.45797	38.40295	30.2236	24.60664	26.45822	26.8674	28.97565	32.89591
Commd9	29.96014	31.80967	37.37196	39.50415	39.63265	42.97734	42.37614	35.937	32.87161
Crcp	32.49558	30.0084	34.6058	29.80776	34.31551	29.91177	34.40213	35.27187	32.87103
Dyrk2	37.92721	34.64387	32.7945	33.17454	29.57414	23.77444	38.74385	28.94762	32.85957
B3gnt2	32.32452	32.13115	33.74883	37.96437	30.47808	31.46496	36.87892	33.62424	32.85928
Birc5	42.37704	55.97307	47.97205	41.40236	31.55966	37.07919	37.96288	24.0019	32.83312
Tns1	50.96637	49.90326	50.16538	41.48163	36.90953	33.37944	59.48873	37.88776	32.82461
Nemf	33.96118	34.08477	28.60016	30.86133	28.27568	28.15918	37.47959	34.11731	32.75844
Cers6	32.71625	27.82197	31.87724	30.99797	27.09313	22.35983	34.52207	32.31136	32.74282
Cldnd1	34.70063	35.65615	33.43262	33.07651	29.67782	27.56421	29.60635	32.11701	32.7302
Vamp4	29.20789	36.35807	35.56156	26.19211	28.17177	26.72707	28.2643	34.00008	32.69992
Hadh	39.29316	41.63011	46.76677	35.40611	39.48828	36.56634	42.14878	33.63444	32.6948
Hspa13	34.70742	36.14307	36.61902	25.74483	26.8311	23.04574	29.99016	26.70382	32.69184
Mtmr9	41.94691	38.94213	38.72826	35.69095	28.96468	23.69626	36.09679	30.64364	32.6723
Gatad1	37.93818	34.53755	37.81198	33.65658	31.34334	33.19196	31.89971	32.59789	32.65256
A430005L14Rik	28.56412	33.208	30.53956	28.124	31.6577	27.82193	28.76546	30.73613	32.6475
1600014C10Rik	23.31639	17.59881	29.83577	29.36749	31.2129	28.0114	23.20678	30.95185	32.62894
Mfsd14a	35.3824	33.5617	37.94127	27.62674	32.3306	27.56647	30.98369	32.70262	32.61593
Vegfb	34.83069	36.37654	40.93786	42.61764	41.21957	40.93577	50.21459	34.23486	32.61513
Hnrnpll	34.29889	32.24975	34.97549	31.90167	29.221	29.48295	33.75886	37.81301	32.58595
Kctd17	43.01477	48.55821	43.1634	43.52361	30.60541	34.16719	33.88153	35.17692	32.5785
Slf2	36.05832	38.91286	38.48526	36.773	30.05396	26.74389	45.13381	29.37122	32.56876
Tent2	35.83115	38.17229	41.25447	33.85515	31.02291	28.80532	37.1304	37.02228	32.56814
Oas1g	20.48558	12.96903	24.40884	25.97143	28.39766	24.94434	21.34408	26.70667	32.54973
Vars	47.42243	38.55028	40.42543	35.3165	32.97629	33.19836	38.26476	31.60947	32.54345
Mrpl21	33.33045	35.12968	35.36278	36.23626	31.61477	34.57422	35.91607	40.74549	32.54011
Gga3	37.85305	39.09875	39.77169	38.60966	36.18104	35.25818	52.49373	39.24517	32.5266
Mtmr3	32.65393	29.92751	34.07071	33.03496	28.0007	26.60899	40.93743	30.06668	32.51605

Cltb	44.67363	40.48849	37.66746	35.0789	37.76402	37.45048	36.56281	34.34411	32.50803
Nr3c1	35.45948	31.13412	35.84123	32.82031	29.45595	25.3564	35.40952	31.56163	32.50474
Tor1a	36.11874	32.13854	35.05376	31.81602	32.8355	31.28299	31.56915	34.42493	32.49573
Mtmr10	48.10722	38.12169	38.00537	36.39069	29.73407	27.63717	47.53047	31.83227	32.47926
Chpt1	24.32293	28.0426	31.19465	24.18668	27.21242	27.47595	26.43317	30.2027	32.47637
Ptp4a3	37.41823	33.58675	31.90767	34.30375	38.91904	41.73633	30.46395	33.65703	32.47201
Vps16	35.80709	30.46338	33.7747	33.28583	32.57693	31.39942	38.9948	31.78276	32.47003
Hfe	32.18732	39.16165	49.90983	35.48774	45.96365	40.97774	46.95001	38.57682	32.46658
Vav3	35.59553	30.36129	34.33414	31.12891	25.69194	26.74451	32.08916	30.6952	32.45751
Ptger4	29.58735	23.93549	25.15727	29.53984	27.43457	24.76981	43.11394	32.4554	32.42442
Trnau1ap	28.46964	25.93412	30.13522	26.12426	25.5823	31.38516	28.649	33.79303	32.3974
Padi4	21.20926	18.51393	18.94354	24.66121	21.97942	24.75982	22.50683	25.03169	32.38292
Stard7	34.59771	37.63012	34.13856	27.03551	28.12466	26.04937	32.37971	27.49357	32.37751
Ripk1	36.17349	33.18658	35.16625	29.50764	32.13726	29.92783	39.54136	34.05321	32.37246
Dhdds	38.06061	33.00493	40.50394	27.932	29.41858	29.40065	34.03528	31.02625	32.367
Dtx3l	26.13004	21.25818	31.74067	31.86777	31.40809	24.91629	32.0829	27.86336	32.35921
Wdr74	38.62687	34.01745	29.49589	26.42836	31.18866	34.73242	35.80498	38.48818	32.32297
Gbf1	45.95616	40.34558	46.82231	38.47281	33.7567	31.21191	48.22716	36.17796	32.31567
Hmbs	39.93847	34.83515	35.90554	35.05508	33.71146	37.53386	36.58429	35.38279	32.29334
Acadm	31.42924	33.03485	37.05263	35.2367	35.48927	35.13134	38.20793	33.78897	32.28667
Gm10132	29.17793	27.43738	31.05726	38.65144	32.9495	36.42683	31.12617	32.93944	32.27849
Acbd3	34.6428	33.37181	33.38491	34.01992	28.06495	25.81832	36.36632	30.48944	32.27176
Galnt6	38.75017	35.89899	35.16159	32.95646	28.10547	29.65837	36.16597	29.70057	32.2555
Cdc42bpb	42.23459	42.37729	40.07718	33.54589	29.6216	26.69445	44.15477	30.97965	32.2467
Lrp5	41.06708	31.61825	44.88127	40.60548	46.95321	33.50146	54.56462	39.03654	32.24461
Postn	31.38826	44.16716	45.36204	48.20949	37.55759	48.27735	29.53138	41.17794	32.23566
Ubash3b	38.96999	30.55439	31.2563	26.51955	25.38333	22.45838	28.82128	32.34235	32.21715
Yif1a	35.64671	29.07101	31.71441	33.52699	28.99856	33.48004	31.39301	28.63617	32.20498
G6pc3	38.22819	40.4914	40.85574	27.47444	35.55266	35.98352	35.57452	32.08682	32.18869
Sbf1	42.33534	37.50641	36.76893	31.33096	31.84123	28.0604	42.45051	33.53691	32.17192
Rnf11	31.38543	33.81744	35.70605	28.36534	28.13284	26.90816	27.46649	29.04026	32.16836
Naa80	36.6875	32.75379	36.69176	40.96869	37.26546	38.37359	53.32269	37.32449	32.14657
Spty2d1	29.41633	24.27025	22.12051	25.6017	23.53003	19.71822	35.77562	27.47193	32.12101
Chm	32.79242	31.04122	34.793	28.93719	24.64109	25.89926	33.12938	28.0708	32.08823
Smarca5	34.81161	31.77504	30.82375	25.16924	24.90264	25.34083	29.77915	30.77745	32.08514
Dph3	33.71045	30.8771	27.23263	27.86513	30.68447	30.13953	26.98644	30.98584	32.08461
Mrps35	33.72987	36.65905	34.16627	34.40095	35.00687	35.7512	29.31942	31.37304	32.06513
Zcchc8	32.5735	34.95597	37.38377	36.32527	33.35744	32.54716	41.89478	33.99431	32.06333
Pcyox1	39.12157	42.91645	46.71512	35.05538	36.52205	33.98159	42.38425	32.2976	32.05952
Alg3	55.54561	43.33143	50.49698	36.14569	39.38131	33.76387	44.51571	39.98362	32.03043
Vps18	36.6147	33.58889	37.6947	40.58681	29.0157	34.2365	46.33116	33.25545	32.02938
Rab7-ps1	33.70487	28.47184	25.66534	26.1291	19.29517	28.38726	33.05898	27.53106	32.00671
Sh3tc1	35.05284	37.25991	36.53522	36.39095	32.82703	33.58742	45.00907	37.87409	31.9842
Tomm34	33.87218	33.1862	34.92167	34.78143	31.12159	31.0477	38.00315	32.78997	31.97004
H2-K2	14.36198	20.75481	19.34695	25.69009	31.22455	27.72466	34.44805	28.10547	31.96583
Iqgap2	39.09069	33.00276	38.3802	34.80778	36.02414	29.27012	50.84724	32.38882	31.95291
Eed	27.34227	28.692	28.1798	27.08207	26.68124	31.53193	27.42516	30.13213	31.93328
Def6	31.3589	27.68166	36.84126	27.6485	30.74701	37.26286	32.93943	32.54571	31.92985
Plrg1	37.21957	33.56141	38.78924	28.35752	28.56074	31.35098	31.47993	29.50573	31.92322
Ccnd1	45.26993	38.0402	45.40998	34.60734	23.94621	26.81082	28.01442	22.12814	31.91367
Tcf12	38.93414	36.32935	37.77569	34.36937	27.54014	22.72504	42.80336	34.82816	31.91341
Dhx16	37.72617	35.98555	34.12855	33.16482	31.79507	32.33298	40.89558	33.16324	31.91247
Tent5a	25.25738	27.3003	34.43663	30.83095	31.42728	25.88768	35.21987	29.64535	31.90603
Sord	34.87273	40.21348	41.16	31.57814	38.30762	32.90735	38.93424	33.506	31.90359

Nelfa	33.22191	32.33971	33.29399	38.05563	30.21085	28.66179	38.75489	32.95116	31.90183
Tmem94	40.52128	40.62517	47.09155	39.25615	40.37838	33.08734	48.77554	37.83895	31.89132
Gtf2h1	36.76993	35.12399	34.52957	35.89651	29.78536	30.37636	36.35594	35.76746	31.88636
Vcl	44.63885	38.6309	37.07529	29.00292	23.47718	25.11294	33.08206	27.42844	31.881
Gm12751	32.01704	36.52899	36.50616	21.30772	31.32322	37.61528	29.67908	36.41397	31.88054
Rell1	27.62372	28.93287	32.67049	24.79791	26.62212	23.55832	31.63122	29.78192	31.87752
Cdc27	33.78338	33.27811	33.96952	30.7213	23.17685	24.09326	34.67471	26.78772	31.87608
Trp53inp2	36.39972	39.14183	42.87295	32.3142	32.06301	31.61532	37.98505	33.09973	31.87167
Sash1	38.98003	35.68876	37.9816	37.26117	32.80387	26.92079	55.53499	30.13464	31.8543
Prpf4b	35.29502	37.56364	38.0408	39.69004	34.39501	29.4846	44.94837	34.15753	31.83592
C1galt1c1	27.22241	30.19513	30.16888	24.8019	26.17157	30.0052	22.35649	29.17571	31.83567
Sap130	42.10311	37.72308	39.42966	36.08821	34.23495	28.49323	48.69474	33.80391	31.82611
Rbbp4	37.28578	34.01236	36.13266	27.75765	32.53937	32.78228	35.48134	32.26593	31.81994
Mark3	31.79301	32.52498	30.01913	28.33825	27.78774	26.72331	30.09384	26.94264	31.80522
2310011J03Rik	33.68389	34.72735	31.51547	31.20238	33.22588	34.20916	30.42769	36.3655	31.78525
Tor1b	34.07688	33.21577	36.33903	30.90214	34.60487	32.30999	37.95302	31.56765	31.7704
Siah2	31.9884	30.42035	31.39731	23.82523	31.51733	28.40331	41.31603	32.39752	31.74293
Pram1	19.52766	12.74064	22.40699	17.98796	17.62275	25.1377	15.97491	20.17061	31.73962
Snap29	33.21115	31.28616	33.9743	32.88055	29.75306	29.8761	33.74105	31.60489	31.71476
Mcrs1	36.36194	32.41696	37.47752	34.25519	34.45432	36.08128	39.55987	33.91479	31.71421
Gm12183	28.40345	25.03673	28.21118	26.20259	27.26065	28.27844	26.35001	28.5556	31.7071
Tbc1d9	34.73358	30.9472	38.46335	29.8603	31.48023	23.41831	41.77446	29.52292	31.67781
Hgsnat	37.51743	37.04606	40.70897	24.3022	25.26607	24.85865	28.46721	31.34636	31.67483
Xrn1	33.78328	33.03888	37.81542	33.32403	29.97668	23.79225	36.06266	26.06808	31.65931
Pnpt1	27.68762	25.358	30.26721	31.67698	28.06484	25.85643	28.05373	26.14198	31.65852
Srebf1	39.23432	35.22218	37.43235	28.80525	29.15687	28.07792	34.81259	32.06908	31.64674
Ndufaf2	26.57467	23.07997	28.10375	30.47187	20.34442	32.33608	21.15821	26.87548	31.62973
Cpped1	38.8104	39.79267	41.32999	36.27891	31.06431	30.16341	37.35814	32.77016	31.58796
Rnf25	26.06267	31.28748	34.44824	30.56025	33.67859	32.77569	31.63972	31.27428	31.5731
Irak3	32.21401	30.48064	31.91037	26.37946	27.67993	23.23553	31.38882	27.76292	31.54499
Naxd	32.0662	29.83634	38.8953	36.02347	35.65909	34.06298	35.8958	35.60369	31.54206
Tusc1	22.35406	23.51061	21.38109	28.37208	28.56699	32.06091	33.68988	38.92901	31.532
Kctd10	36.41537	34.08537	33.46376	29.67446	32.08859	27.0334	33.47166	28.44625	31.53172
Rubcn	38.61062	33.87283	38.94553	35.19664	34.67465	30.00272	51.37926	32.6509	31.5303
Srsf1	33.90239	36.37178	33.47163	28.78322	27.94177	26.49979	29.74533	29.53926	31.52293
Pdcd2l	28.33253	26.75525	32.24037	23.90338	32.08285	29.55353	29.91717	35.63082	31.52171
Prim2	34.85925	38.00054	37.79457	33.11372	27.37925	27.62326	34.54638	27.25214	31.50036
Pnn	36.56451	36.41935	35.83355	37.0907	32.66238	34.80389	38.18766	37.24747	31.49834
Nasp	29.61051	30.50883	23.1359	31.36613	25.03851	30.88124	31.41967	31.04241	31.48974
Fbxo11	31.99109	31.17471	32.29093	26.97491	27.37954	26.48119	36.10998	32.70043	31.48475
Tmem254a	49.89426	55.85755	50.75305	40.99226	41.02045	37.59608	36.72949	39.2485	31.43148
Med22	37.89274	40.3715	42.51033	34.67976	34.11792	29.89933	31.99064	32.17843	31.43127
Senp7	32.63615	36.059	33.17028	32.26159	27.36345	29.82424	36.8202	31.99077	31.42677
Pfdn4	20.8485	22.81829	19.63236	22.61642	20.95183	25.72759	16.79062	27.8413	31.41858
Memo1	34.54246	33.41772	32.54914	29.08997	28.21326	29.0367	32.52875	34.29628	31.40635
Nt5dc1	31.03257	29.61536	32.56568	23.82523	27.18825	23.27657	33.78606	29.30337	31.39878
Mapkapk5	33.82023	32.12757	35.02948	29.61107	31.37308	33.52813	34.87705	30.57463	31.39614
Sfswap	31.44618	32.54562	33.27379	31.72782	31.51728	30.24301	39.51019	29.24867	31.38855
Gm7730	26.95143	26.45436	22.1778	27.30933	30.62319	30.20832	26.09263	35.08004	31.37729
Katna1	27.35986	26.78862	25.48885	29.78439	28.3179	24.14622	25.95358	30.33133	31.375
Ndrg1	31.3062	25.77033	27.47285	31.89385	35.31478	31.13296	26.13106	34.50038	31.37335
Camk2d	36.05847	25.59653	33.03425	31.55957	28.80774	26.95814	38.2325	29.84306	31.35612
Fut8	32.4496	29.15605	30.43335	28.09231	25.36155	26.51846	31.91768	31.04702	31.33263
Ltv1	40.89693	35.47467	32.56464	30.29053	26.41423	26.02494	31.15923	32.64218	31.33207

Naip5	28.04732	33.78209	35.52624	37.21485	35.78697	29.26896	48.59803	34.9746	31.32056
H6pd	37.63795	34.14261	41.31065	36.37821	33.93951	29.15313	44.94641	30.5853	31.31401
Cpsf7	37.48434	35.84461	33.39813	30.12005	32.96197	27.31222	41.45829	34.03101	31.31105
Camk1d	38.02607	37.09687	42.26808	31.27324	37.87905	29.88105	43.02365	35.74262	31.27908
Ythdf1	36.78388	31.2807	33.1513	31.24067	31.18631	26.11064	35.83158	29.26256	31.27587
Senp5	35.71957	34.01251	37.75584	29.54982	24.18928	24.12834	36.03498	30.82092	31.27111
Mki67	56.69626	61.59443	63.9547	38.89227	31.37136	31.53686	51.28902	23.35668	31.26369
Thoc5	34.42865	31.91262	32.64652	30.14131	26.83634	29.29237	34.91717	29.47316	31.25326
Keap1	30.9299	27.96985	31.7605	33.95828	30.78103	31.87331	30.74926	29.71631	31.23963
Mcmbp	37.18909	33.40494	35.78437	28.79954	29.02594	27.6925	36.08771	30.63686	31.2388
Rpl3-ps1	28.685	28.94372	34.19078	28.10669	33.55911	30.5274	26.60885	34.18864	31.2221
Mef2c	35.24376	37.47548	38.87427	35.02507	30.64214	25.49027	40.21805	33.41294	31.19476
Incenp	43.77965	48.25824	50.18362	40.01012	31.87103	31.75749	43.70612	27.40355	31.18331
Faap20	29.67032	26.65886	29.29951	30.67366	31.058	30.84942	33.98098	31.812	31.18273
Macf1	47.48559	44.30794	46.762	41.99303	31.75375	23.79956	52.51385	33.24138	31.17934
Eef1e1	34.04185	29.53437	28.65611	26.90053	26.52634	22.74713	26.63613	32.00477	31.17356
Ei24	37.09515	34.75109	37.99497	30.85819	32.21032	30.23522	33.05146	31.59154	31.16114
Snhg6	25.74397	25.13203	23.83978	26.8188	27.89115	36.40362	24.35517	28.92012	31.15719
Mbtps1	39.71401	40.46758	41.89978	29.93326	31.79712	30.31474	37.73487	32.53082	31.15328
Anapc11	35.6081	38.56121	37.31214	30.13319	31.83614	33.88188	34.22975	31.81006	31.13954
Rps19bp1	30.8583	30.70953	31.79709	32.81084	27.98962	36.01071	24.02374	31.92385	31.1295
Nsmce1	28.31901	29.6199	30.00244	36.45337	31.82773	34.28922	32.30764	31.70376	31.10773
Pim3	22.91776	21.02821	14.1419	21.78665	24.68591	24.49983	41.04896	37.6176	31.08211
Maff	22.71277	16.43691	12.61311	15.06682	20.25306	14.53077	24.60426	27.58636	31.07751
Rabggtb	32.60286	30.14647	30.89051	29.57635	30.17063	23.8434	35.1064	33.21944	31.07342
Gm14539	15.22478	24.75693	16.5506	21.88645	26.3205	21.73125	20.86674	25.3671	31.07154
Bicd2	39.99714	39.59779	37.79918	35.86757	31.25267	30.68575	41.46549	34.28356	31.05407
Mcm5	45.29083	43.38669	43.38698	27.03427	28.05464	31.88649	36.75878	27.16604	31.04365
Nelfb	33.53193	33.10719	34.58711	33.07233	34.89874	28.48188	37.69891	28.14264	31.03721
Cisd3	30.37802	28.62608	26.81154	29.0488	29.2786	26.81264	28.83723	26.49527	31.0359
Atf6	40.93651	34.33224	38.44204	32.58717	31.07868	26.3803	39.43311	32.80419	31.03556
Slc39a13	33.93481	34.45881	36.15094	34.1902	36.31904	33.42737	38.19867	32.14932	31.0353
Nup214	47.24387	42.92545	49.3351	35.14053	32.35791	28.12417	47.77662	33.34864	31.02006
Upf3a	24.51612	27.36918	33.7729	31.96767	29.07313	27.20609	28.40379	30.38192	31.01653
Pop4	34.66021	33.24715	38.40925	27.56376	28.22594	30.02563	28.08263	26.78254	30.98866
Wdr20	25.73163	25.49621	26.23676	26.41961	27.29588	19.46226	28.41116	23.54297	30.98465
Sorbs3	51.32195	61.00325	59.01414	44.70882	51.53758	45.19835	68.78141	38.88802	30.9727
Tars	37.58263	32.19684	31.80277	28.73491	26.83996	25.36141	30.88788	29.63507	30.96881
Pmaip1	18.77876	15.72883	15.27126	18.71511	20.72952	23.18043	30.37615	30.28926	30.93795
Akap8	33.38447	36.05692	31.49965	35.42548	33.86793	31.84971	40.55852	32.98904	30.93367
Rinl	29.71619	30.13744	36.91797	28.66708	31.42555	31.99527	28.87835	30.38712	30.9153
Cdk20	35.52826	31.20353	33.5861	34.88267	33.78921	31.79408	38.11271	37.5796	30.91297
Mib2	30.67557	38.87423	35.79634	38.01567	42.96064	39.81759	41.00911	38.23684	30.86974
Dguok	30.06375	35.10913	32.95832	34.62239	39.09422	33.98024	33.79454	38.7153	30.85753
Tmed4	42.09881	38.31204	33.13022	31.04104	31.61418	28.89903	36.14182	31.53956	30.85704
2210016F16Rik	28.66199	23.53078	26.288	28.79446	27.24124	29.92111	27.08559	31.83408	30.85614
Uchl3	26.16915	26.27784	29.09346	26.85244	24.63935	22.93251	30.06605	34.05321	30.85022
Rpl9-ps4	23.39517	24.95002	24.5562	23.03378	27.06112	31.48874	21.86581	25.50908	30.83408
Tm2d3	27.23166	32.74897	32.17	29.68405	31.44708	35.0421	30.85277	31.90149	30.83149
2210016L21Rik	31.70889	31.20561	34.19078	33.2018	34.99907	41.71301	35.63762	33.94872	30.82182
Med30	25.77774	23.6143	30.0457	29.11442	30.49104	36.61115	25.78739	31.51192	30.81676
Ino80c	36.71597	37.43821	37.14312	26.44252	24.98985	26.89365	28.47905	30.55121	30.80689
Edc3	34.05246	31.89456	33.29961	30.03387	28.13837	27.68743	34.27679	28.78759	30.79307
Vamp2	30.6139	28.10514	25.00639	26.82499	28.71717	24.63867	33.04432	32.70854	30.78926

Eif2s3x	38.2518	30.335	32.18162	29.10832	24.49314	19.06626	28.28959	24.9271	30.78622
Kat6a	35.47135	31.09902	35.13739	36.34831	29.92636	24.57083	42.74058	28.35091	30.77886
Dmkn	43.78013	22.08273	32.20982	8.84042	17.97229	12.02185	5.981184	20.36237	30.77519
Mapk8ip3	31.37908	33.4291	30.58168	35.94178	32.61772	28.84025	45.83421	31.25633	30.76706
Ube4a	39.85044	37.63071	38.60712	37.1259	33.19955	26.94331	47.53339	29.56874	30.75103
Cfdp1	31.2854	27.67599	28.53429	31.29412	27.99481	31.25086	33.16358	31.509	30.69937
Tctex1d2	20.1593	20.891	25.51707	15.50462	21.14944	15.80259	18.8823	23.09233	30.69673
Samm50	32.15922	28.59188	26.64425	28.07568	29.86201	28.04382	32.53981	28.67347	30.6966
Slain2	41.03993	38.19235	36.84038	30.73817	25.76121	24.37023	36.07602	31.56942	30.67574
Nudcd3	36.71193	30.70728	32.50596	31.41071	30.13903	27.72117	34.07273	27.32415	30.67309
Baz1b	36.70305	35.58255	37.49162	31.04726	29.18574	26.53822	40.43727	28.79118	30.65275
Smu1	30.42064	29.08958	30.18702	27.08373	27.56884	27.97542	31.09113	30.51159	30.6402
H2-Ke6	28.46664	31.37333	35.71684	34.94079	37.68199	34.46189	38.15904	34.54952	30.63911
Fhod1	35.70723	34.08251	33.76203	30.10567	30.20324	26.68268	37.21893	28.65769	30.62133
Itpa	26.83123	33.10542	28.65948	25.65097	31.04971	28.0511	23.44936	27.61218	30.60244
Lyar	30.53499	29.42202	28.69933	28.80194	27.11082	28.52125	25.34259	32.86593	30.59968
Ndufa11	26.41693	29.25554	28.89949	33.41627	32.89744	35.39203	28.65977	31.36005	30.57858
Exoc2	32.16822	30.69011	32.67465	25.56451	27.42762	23.84329	31.45811	24.83151	30.57046
4930453N24Rik	26.69857	27.74909	27.03492	24.10535	23.80102	29.73585	26.21674	32.83538	30.56709
Pgap6	30.77581	27.75697	30.21918	35.28225	32.49614	29.70371	38.98712	26.08649	30.55234
Zdhhc4	33.77356	30.68498	35.54874	29.16107	34.08766	32.29921	38.69531	32.0381	30.54049
Rchy1	32.65572	35.25991	30.57421	27.50278	30.10805	27.42011	35.11404	29.57336	30.53931
Stard8	38.83479	41.23397	44.63236	37.52123	33.97068	31.0879	54.47431	29.47244	30.53125
Dram1	26.93251	29.8559	35.08784	26.59204	23.47109	21.68835	30.64433	30.09009	30.53029
Dennd10	30.8285	28.81572	33.05857	28.9233	27.35572	25.13019	30.71233	26.8405	30.51239
Hnrnpd	32.23748	25.76651	25.60333	30.63011	28.49702	26.52992	32.0789	34.94332	30.47727
Klhl5	34.99939	32.69265	34.97907	34.70633	29.17512	27.56214	34.71155	31.44191	30.47507
Seh1l	30.59509	30.17253	28.48411	27.79565	30.85594	25.27427	30.07904	29.45516	30.46374
Rbmx	30.61226	25.71379	28.07228	28.67745	25.75487	26.41047	37.98163	28.8793	30.44377
Cpne1	29.96307	27.63727	31.96325	27.127	27.71508	25.12412	31.21594	29.74431	30.42984
Nfe2	16.58598	14.42689	18.63299	14.38437	18.32083	20.76609	17.72951	19.72132	30.42481
Erlec1	25.21795	28.78175	31.52611	28.1377	28.13295	22.88283	32.36588	25.86989	30.41042
Gnl2	33.75494	29.95859	30.75978	34.33046	27.43672	25.33742	30.56953	34.40743	30.38822
Hmgn2	27.98429	29.49707	28.91603	29.98164	28.12557	34.99592	24.30824	29.59017	30.3794
Arpc5l	31.71005	34.97525	30.84128	35.69627	33.10817	31.38161	28.22073	29.48509	30.34721
Churc1	28.84833	30.20498	31.11053	28.38047	34.61242	37.8809	30.0284	35.09057	30.33258
Gpx3	37.08012	75.88817	65.57169	42.66769	39.92851	32.53896	38.70062	44.14723	30.30687
Pvr	30.96316	24.81179	24.78492	20.94006	24.24202	18.99449	31.40677	31.10657	30.29734
Atl3	35.92108	36.22977	38.06832	27.98848	27.57827	21.54328	34.68707	28.75535	30.28939
Ndst2	38.17707	34.84669	40.07641	36.95443	34.28499	30.54521	40.4139	34.16088	30.28624
Gmpr2	35.54643	35.16531	31.38504	28.45637	31.18674	28.22362	34.32685	26.97239	30.28569
Snx24	32.53681	28.29335	31.7338	33.78065	27.65844	28.92856	41.27124	30.29552	30.28433
Ammecr1l	35.52401	37.63621	36.92259	33.72756	26.67354	25.78786	40.09921	31.42707	30.27534
Arhgap26	36.17235	24.43595	32.82381	26.95672	28.37999	26.04513	27.48337	31.50398	30.25479
Khnyn	31.89434	32.72862	30.83664	34.24294	33.16869	27.48035	37.8138	27.14907	30.23843
Sacm1l	33.17055	31.99143	33.44787	29.53503	24.49504	24.66966	29.39084	28.81558	30.23003
Nub1	31.90448	33.68231	35.50826	29.07492	32.00258	31.18439	35.04567	31.71798	30.22979
Aatf	31.43431	27.3208	27.15719	29.35141	29.9703	24.31955	28.56052	28.52666	30.22077
Zfyve19	29.13688	30.50874	31.3382	35.6355	34.43387	33.95973	35.97611	32.43219	30.20245
Mad2l2	29.7284	27.43969	31.02007	23.87493	31.45344	25.68134	25.98646	28.40246	30.19981
Specc1	40.16398	36.33404	36.65991	34.33372	28.58439	27.23116	42.16373	29.58385	30.19571
Dtx3	33.12947	38.30127	34.19432	38.73093	37.0081	40.30754	43.48258	32.06196	30.18867
Sil1	35.10922	36.4108	36.04592	30.9252	34.28135	32.03673	36.19075	34.58162	30.17375
Gm20056	48.3817	53.80888	45.01067	39.91877	22.66932	21.69965	38.83842	28.22351	30.15143

Il18rap	23.30377	20.09898	17.07676	12.09672	16.50611	16.09518	13.9742	16.91969	30.13792
Ppm1b	30.86935	33.83613	34.37783	29.54717	27.30473	25.61645	29.74718	29.56812	30.13189
Malsu1	20.05657	22.67966	23.02536	21.77011	29.72864	25.20919	20.58349	25.68523	30.08703
Jmjd1c	38.37185	32.60061	32.26405	33.84006	27.35863	22.11153	45.95478	29.24897	30.07458
Gm12918	24.8093	25.04872	26.42038	27.94448	31.79584	31.2986	22.63429	28.86997	30.06769
Pcif1	32.70432	28.14037	33.51496	28.92138	29.09834	29.51701	32.30496	27.63202	30.06606
Nktr	35.76236	33.11835	34.50058	38.5813	27.0631	23.49261	45.39128	26.37598	30.06081
Hps1	34.54341	30.94825	31.91773	32.89289	31.56064	33.72659	39.4987	33.15626	30.05342
Trmt1	35.07587	32.3169	29.89925	32.38605	32.06754	32.46255	38.70178	32.08398	30.04274
Ppan	36.46106	28.29046	30.42646	28.96587	24.45609	28.83982	28.66615	30.67132	30.03922
Gpat4	36.75724	29.31348	31.74282	27.31217	26.69382	26.20564	33.39962	27.24775	30.03586
Ppid	29.06212	28.78525	28.83686	26.51236	26.75311	24.33879	27.79249	29.95541	30.01382
Bmp2k	34.00045	34.0529	37.51041	28.3413	30.96674	26.04345	40.2864	33.25271	29.99584
Ankrd27	33.2226	31.98764	33.31269	36.63737	27.64468	28.22497	37.45608	32.42469	29.99324
Rnf20	31.94211	30.68621	30.91847	28.03397	24.89299	26.14637	30.31739	26.6241	29.99299
LOC115489587	27.71903	30.63391	23.22675	35.01343	29.25328	22.16968	47.23214	25.67796	29.98879
Phax	29.07156	32.5913	33.14854	29.92902	30.61904	34.90668	29.37206	30.99453	29.95996
Gm38525	16.27821	9.853242	14.28349	11.24948	17.69704	14.20149	8.412172	16.27825	29.95583
Fam162a	35.73761	34.58699	33.86918	27.28043	37.44747	39.82444	35.42211	34.10321	29.94895
Ap5s1	32.56037	37.34947	45.28601	27.23337	35.44016	32.43716	39.78483	32.22081	29.94722
Rnaset2b	24.95534	40.44446	28.57061	28.89705	32.83625	29.4041	30.14791	36.89041	29.93945
Trim3	33.86786	39.46196	39.1992	32.13048	35.99241	36.4487	35.97834	34.52044	29.9391
Tmem106c	38.401	40.5335	39.33276	38.33956	42.90117	40.15647	35.3371	36.70864	29.93506
Tmem222	26.03248	26.67557	28.85337	30.14848	26.68276	28.25761	26.33165	28.33704	29.91724
Marchf1	23.17654	27.09822	33.27796	27.03235	35.33301	30.43269	35.42243	32.09528	29.91707
Mrpl51	32.89691	30.47363	28.54569	32.42153	28.15926	29.67756	29.23522	29.23069	29.88888
Tpp2	37.62209	33.54035	36.39887	26.12988	29.04377	27.6284	37.81274	32.76779	29.86017
Ppox	24.72138	31.70528	27.24109	31.20704	31.05178	32.13623	37.02568	32.346	29.84008
Pds5a	38.53934	35.57671	35.72703	31.74441	27.72097	25.93203	35.62998	29.69197	29.84
Hebp1	31.24596	35.48804	35.06434	33.30513	38.6659	37.6895	34.45441	22.73477	29.82997
Ccl5	16.25627	9.873298	20.21849	27.56376	33.94196	35.27042	21.97142	35.91589	29.79553
Etv6	32.21031	26.39583	31.67789	25.959	26.55039	22.73491	33.57367	29.26109	29.79208
Zfpl1	33.03326	30.14688	35.41815	33.94739	35.38802	35.93435	33.5703	34.04145	29.78182
Arg1	21.39451	17.74386	35.77428	8.771136	36.03317	20.79158	21.10367	45.06344	29.76352
Pigbos1	19.5533	26.48181	23.33336	29.38662	31.63684	27.73987	19.97966	25.91524	29.75413
Rab22a	28.45956	30.81669	28.43382	31.62108	27.63007	26.96305	33.64644	26.12857	29.7539
Clcc1	30.65226	33.02801	36.02426	29.95554	31.84678	29.3499	28.98248	31.19472	29.7455
Mrps18b	40.42494	33.77062	34.44969	23.97692	28.03959	32.0947	31.63817	33.53822	29.73265
Sugt1	29.33231	29.87587	30.58249	30.15242	27.55165	31.95735	26.69738	34.5425	29.70356
Madd	33.97076	35.96981	38.12707	28.92887	29.4834	25.31963	40.41017	29.52904	29.69653
Osbp	34.84367	32.23168	33.1555	26.95684	26.09427	23.25865	35.87527	27.77782	29.69584
Timmdc1	24.16362	24.23062	26.53698	23.89732	21.19469	23.1373	22.59256	24.57331	29.68182
Sec23ip	37.78001	35.02705	38.54713	30.31528	28.86696	28.42763	39.85597	29.50978	29.67095
Gsap	32.3042	32.185	33.58962	24.67581	22.68232	20.51619	29.24245	29.33064	29.64668
Pacc1	34.17906	31.58252	34.09858	31.86732	29.375	29.75304	40.82428	28.86353	29.63804
Pfkl	30.42405	26.32597	30.84379	25.00691	30.32027	33.06952	36.74446	32.49023	29.60594
Gmfb	34.41121	38.60126	38.16379	28.4559	27.92465	23.24302	32.92815	31.86436	29.59198
Svil	34.65214	30.96231	34.17396	27.53269	28.16379	22.41761	34.13518	29.43883	29.5886
Itprip	30.21988	29.6615	28.33631	14.39987	24.18866	23.62565	22.47345	24.65178	29.55869
Lap3	30.37233	24.87705	29.10409	29.52131	29.06602	30.07747	31.59352	31.38543	29.55352
Anapc1	39.6961	36.71306	40.10777	31.00998	27.22588	22.69448	41.54755	28.71065	29.55061
Lyst	33.11046	25.95784	29.90173	25.45135	21.53911	17.04258	29.48964	23.03556	29.54891
Tcp11l2	23.46878	28.65889	26.75197	29.03383	28.29125	25.16374	34.85629	30.32793	29.54491
Nudt16l1	22.74827	23.38047	25.57307	24.17495	26.33728	25.21215	24.58834	29.01589	29.5426

Isca1	29.67311	29.53637	28.25788	27.23271	24.62097	26.12896	29.65134	28.7754	29.52272
Eif4enif1	33.26284	28.70669	34.95069	25.55769	26.77752	26.51508	34.77283	29.00767	29.52233
Snx27	33.99403	32.64547	35.60166	36.86507	29.30088	26.32569	42.10619	27.7856	29.51599
Wdr82	34.8628	33.71223	33.76069	27.90275	29.3069	26.15912	33.24506	28.34587	29.50172
Exosc8	28.06825	29.64788	29.55585	24.78568	28.35327	29.83197	28.82947	29.3005	29.49741
Ercc1	25.20844	26.98093	31.51421	31.28309	28.39999	30.3719	26.95442	29.90356	29.49518
Vrk2	25.65159	24.66688	28.37319	25.27947	23.84688	25.20882	26.31435	27.15673	29.48385
Ipcef1	22.33578	24.16015	22.85787	17.98453	26.08449	23.05722	20.88888	21.85032	29.47913
Pld2	32.24092	35.50899	37.48545	33.19664	33.53551	29.3625	43.49654	26.48449	29.46821
Slc17a5	37.74659	34.46725	36.28248	39.24453	31.65188	23.14981	45.49047	34.19949	29.45651
Rpf2	28.05615	23.19958	24.23824	22.42349	21.26214	24.52521	19.58972	29.67231	29.45328
Ttc13	42.07136	43.14434	40.67191	29.77907	28.23812	32.3886	36.5055	35.11975	29.44276
Galnt10	35.3834	33.48039	35.5968	32.13316	34.01433	31.18941	37.56183	30.5172	29.43537
Atp2a3	35.42149	34.40976	36.07573	23.99023	26.20072	27.19894	26.55737	26.33285	29.43464
Lonp2	33.2273	30.97684	32.91129	30.17879	28.09157	28.95293	32.76271	27.74463	29.43314
Slc35b3	30.05283	41.56994	36.28053	36.56168	34.93911	37.33356	40.03243	37.18942	29.42348
Dnmt3a	35.49081	37.74739	35.5136	39.42982	28.14978	27.79931	46.01583	30.3856	29.4213
Gna15	32.63556	36.38124	40.32585	28.63147	33.36915	31.22555	35.94997	29.61952	29.40797
Ash2l	38.03377	35.84608	36.78865	29.4129	31.04824	27.62205	38.25873	33.22419	29.3818
Stx6	30.52588	28.93812	30.10102	24.95199	26.13091	24.53913	28.31113	28.41968	29.37321
Pts	29.41492	32.13613	30.06168	30.99469	34.47504	29.90597	34.08869	31.40223	29.37269
Cdc37l1	28.90699	39.53269	38.81115	35.28162	29.26551	23.47314	35.03364	28.27632	29.3625
Purb	32.97643	32.6082	34.3013	28.28553	26.74183	23.66838	33.35963	25.7811	29.34788
Snora81	22.64848	30.67947	19.60842	27.56376	28.00902	13.75466	31.09465	23.81648	29.31831
Mcm7	37.98359	34.8396	35.50435	29.68043	24.70993	27.58028	29.45567	24.00431	29.30402
Lmf1	41.07456	38.59562	42.26744	38.37448	41.82747	41.7463	43.08512	35.47393	29.30074
Usp39	32.61063	29.35627	31.70688	28.25462	23.84714	22.5524	31.00833	29.05061	29.29705
Stim1	31.73789	31.40334	35.79777	29.5656	29.18742	26.77251	32.07103	27.77433	29.2932
Arsb	38.78858	38.89322	48.59213	28.62098	26.44563	30.57536	35.73764	28.25033	29.28641
Gins4	29.83251	28.09113	28.77911	24.73464	28.6444	31.23402	24.99436	30.57049	29.28524
Acvrl1	35.97031	26.84785	32.62081	35.29026	34.67172	34.74295	30.08506	31.27631	29.28177
Bsdc1	25.9905	29.78457	29.49264	28.41283	28.64057	25.88406	32.5234	28.23525	29.27692
Impa1	30.20171	28.89604	33.45613	25.42585	22.84039	25.72837	28.96644	25.41649	29.2484
Rpa1	38.82304	34.39529	35.00128	31.2504	25.15872	29.95511	34.98511	27.42329	29.22292
Tmem68	32.31306	28.52041	28.44499	32.79934	29.93887	24.86993	31.36986	31.79726	29.22292
Ergic2	30.68735	29.60411	28.23918	25.14074	24.83813	23.76272	27.199	28.34562	29.21962
Rbm10	32.11823	29.43273	31.79606	30.92569	29.42523	28.81672	34.47984	30.54092	29.21392
Ndufs1	31.54428	31.5498	32.31084	24.54078	26.34588	27.9678	31.9997	25.05619	29.20918
Gadd45a	19.83813	21.77288	14.13291	14.36739	23.42291	26.38762	17.18004	17.93914	29.18144
Gpr34	22.40305	24.033	28.62783	32.82092	40.72558	32.52157	38.06785	30.34019	29.17471
Ddx18	32.41653	29.03943	30.63177	27.60133	31.185	27.97462	32.69709	30.55196	29.1674
Tbc1d15	28.12892	31.30742	29.77196	23.84444	23.42267	23.65944	28.29596	25.93593	29.15335
Ppp1r37	26.1796	28.92002	31.61326	27.70293	25.57294	26.3153	35.02295	27.03969	29.13082
Zkscan3	26.64871	28.88698	31.50157	38.18526	35.15746	31.83507	41.35932	31.07053	29.10429
Slc25a20	28.32231	28.21524	27.91278	26.30082	29.63496	22.43315	29.69914	30.56304	29.08175
Slc27a4	25.87292	26.04953	24.04328	21.81168	24.81039	27.44209	24.08382	23.83934	29.07751
Bud23	25.49637	25.09872	23.95614	22.50401	26.34682	22.76285	29.23755	26.1289	29.06232
Imp3	36.93815	41.02196	44.58996	36.36332	38.52712	39.40574	42.44769	41.86196	29.03412
Sgpp1	33.67312	45.62628	45.55323	29.32174	34.15072	31.35577	35.18208	32.50473	29.0256
Ccdc50	34.16551	31.91262	35.31817	32.7194	28.97221	23.285	34.26678	29.92598	29.02434
Mrpl15	28.36366	28.53904	31.07157	33.70815	27.91168	31.92943	24.73272	33.17242	29.01112
Prr3	27.98815	29.6135	30.32402	26.67173	31.86712	26.46326	40.42384	34.16341	28.98362
Pex19	31.39872	29.74647	29.36326	25.39149	26.29759	28.42601	28.4182	25.86433	28.98191
9130401M01Rik	23.80152	23.69592	23.16894	19.16197	25.77411	25.65327	23.72461	25.20171	28.98186

Vbp1	26.00499	33.49891	28.18977	21.72734	21.76582	22.09525	24.26486	33.06734	28.97641
Ilf3	37.17338	33.16715	32.93751	35.57762	29.27009	27.13085	39.01219	31.90112	28.96765
Fyn	40.57258	36.62665	39.312	29.04024	28.61668	30.1267	26.8783	24.9943	28.9559
Mocos	27.48836	23.75343	26.58799	29.03561	24.14749	24.70932	29.52317	23.81068	28.94698
Cdc42ep4	32.78453	27.62645	30.86737	28.20043	26.02268	22.22051	31.66571	29.13435	28.93135
Mitf	32.85716	30.89296	27.97277	33.54796	24.62461	22.52059	40.93075	28.81688	28.92839
Rbm28	31.54354	29.0396	29.05116	33.27414	28.67719	30.58092	34.34537	30.7366	28.91851
Cks1b	36.25078	39.14887	38.37902	27.3817	28.3871	33.18496	26.11336	21.82419	28.89654
Etv3	30.41544	25.84883	30.83966	28.70063	28.36127	22.71125	46.41807	28.35261	28.89609
Fbxw11	32.3278	27.81744	27.46987	27.24679	23.37609	18.98883	31.16111	25.73367	28.88715
Ttc39b	35.7728	41.64332	38.9441	20.64366	24.68651	20.96973	34.66974	26.57735	28.87799
Polr3gl	31.57195	38.42666	32.50043	30.0815	33.17212	33.26883	41.27823	26.91529	28.87381
St6galnac6	35.85462	36.02441	40.18699	26.71028	28.78249	28.8421	32.78455	29.78653	28.87277
Nfix	44.58963	42.98004	44.76567	39.83792	38.7244	38.07531	43.39286	38.54991	28.86111
Afg3l2	35.01739	28.17829	30.56083	29.79965	27.13652	27.5085	34.32547	30.05863	28.86032
Gm13436	23.68481	23.62175	27.15826	24.67367	30.9851	28.88303	25.46578	28.74538	28.85798
Lactb	31.63213	35.19834	30.50489	26.01025	26.21509	24.21648	26.23467	26.51873	28.8436
Rnf111	31.84631	30.70705	34.56364	31.56659	28.8985	24.46745	40.05067	31.94601	28.83475
Tmem250-ps	27.89864	31.02476	36.61972	32.17161	33.52654	28.36869	35.91335	28.27225	28.82662
Ldlr	44.33272	27.16533	34.15574	20.24784	23.13105	15.42237	23.76729	33.05837	28.8204
Pon3	39.00131	38.7793	44.42332	36.10642	31.92099	30.82305	45.26105	28.57506	28.81453
Surf1	27.57175	29.31981	35.29358	28.00314	29.34503	33.28839	24.72156	29.78479	28.80509
Mrpl53	21.72814	30.85565	19.94214	24.54064	22.82262	30.01623	20.42397	28.34929	28.8049
Atad1	29.83762	30.95716	33.07354	26.78966	26.14591	22.78145	28.88325	28.29576	28.79479
Zdhhc16	33.0751	34.08845	30.53866	31.90149	27.97695	28.9528	28.87728	28.44155	28.78691
Vrk3	25.96386	29.01725	29.446	29.78081	28.49257	29.59646	29.94009	29.41727	28.78478
Pan3	27.76241	29.17387	28.41639	31.4316	27.94167	21.87622	43.45921	26.47384	28.7753
Hmgb1-ps5	22.68723	17.52039	19.51152	22.73571	26.06301	23.67443	30.92997	24.54217	28.77451
Mettl1	33.68784	32.97481	26.62836	29.70796	27.73861	30.51768	32.55715	27.49211	28.77166
Rad9a	31.40406	30.86105	34.3615	33.19507	34.60554	31.72263	34.27799	28.73884	28.76875
Eif4e3	31.03914	32.43062	33.80063	27.6186	30.927	28.96923	33.56254	32.40881	28.76306
Gm8451	27.10533	24.07482	30.17736	35.19211	29.61398	30.26857	33.9348	32.62385	28.75836
Foxn3	29.13099	33.35216	30.87565	28.65111	29.07866	22.71787	35.90178	27.25039	28.73577
Thap7	36.10142	30.16276	39.82097	39.35651	33.88364	32.50458	34.75291	32.64895	28.73374
Filip1l	31.90288	32.0219	43.17274	27.82644	30.61118	26.58717	31.07091	28.62228	28.73328
Paxbp1	24.27689	27.0138	23.90125	30.42217	25.83791	24.36177	39.27674	27.0356	28.71447
Fbxl6	24.34889	26.9537	28.64149	24.9501	30.10043	29.19766	29.83229	28.67878	28.70863
Uba5	28.80379	27.36901	27.1221	24.62608	24.70676	27.27715	31.82162	27.446	28.70641
Klhl24	27.1311	39.37247	37.97758	29.43803	33.29968	24.11454	45.93134	32.03827	28.69305
Sap18	28.95348	26.12627	26.7442	25.07681	27.37649	27.16074	26.29611	28.87925	28.66482
Mpc1-ps	29.69225	22.30361	23.55608	26.84742	24.22173	27.11089	23.30766	26.12285	28.65757
Cstf1	34.17665	31.71607	34.82037	30.28985	30.54776	25.60552	30.60091	27.31928	28.65413
Ocrl	39.31605	41.03517	43.74349	29.18734	28.02538	22.34644	39.57439	29.59798	28.63285
Rit1	28.77432	29.32921	30.77123	32.64743	28.29773	31.25055	29.90936	27.79787	28.63143
Bckdk	37.9742	37.29252	37.99802	29.8117	28.75556	32.94607	33.95363	29.67051	28.62873
Ankrd17	34.57714	30.36756	33.81678	29.84378	24.2936	21.54442	35.70899	26.93049	28.62351
Rmnd5b	30.65332	29.12268	33.99039	28.52694	29.41094	29.7962	29.94454	29.72743	28.61799
Exosc3	28.30151	23.16869	30.0867	22.55527	28.78144	33.65016	24.85983	30.20801	28.60665
Ttc17	32.85092	29.70781	34.26767	29.60333	27.07205	24.94897	34.58639	32.33068	28.60066
Ids	41.31629	41.50522	47.2241	28.50949	31.14777	23.79472	39.52121	32.42242	28.59966
Smg7	33.13539	29.47933	32.68084	27.94729	23.35958	20.82124	32.14419	25.37475	28.59762
Gm18588	30.60461	30.08709	43.8134	24.08569	26.23274	24.25901	30.45651	22.97786	28.58553
Zfp622	19.04633	21.24432	20.88812	25.13485	27.23752	26.6488	29.41557	26.21234	28.57706
Btbd1	30.42535	34.56345	31.57436	28.71187	32.58348	31.36518	29.21225	31.13128	28.57551

Gtf2f2	30.52443	23.21866	25.58655	27.10575	23.64771	28.18932	27.26416	29.62037	28.5737
Ube2k	29.75297	26.45852	26.25085	22.83671	25.62334	27.38452	27.4919	28.14916	28.56951
Srpk1	33.41653	36.89681	33.44237	29.91078	28.18714	26.37479	35.62688	28.13671	28.56358
Unc50	28.37964	33.8384	33.45698	31.37382	29.99806	27.12431	28.94942	29.43541	28.55939
Ell	29.86647	26.20249	26.16765	27.75139	26.97264	23.04955	38.55775	29.30839	28.55104
Kras	31.07855	26.52048	29.34878	23.06621	23.16581	21.73649	27.96192	26.96766	28.54577
Mlkl	24.65131	18.0295	26.04212	24.80375	25.42541	21.7498	24.34599	24.09634	28.54194
Tnfrsf11a	31.02943	28.15148	29.37764	27.49793	23.40416	21.18453	31.16948	29.60609	28.53262
Anks1	31.98505	29.06346	32.63583	32.44924	33.53026	24.69261	43.24167	30.08075	28.51909
Sucla2	29.77389	30.0959	29.28873	24.86311	23.70326	25.48752	26.61431	30.48194	28.51412
Ints11	30.85552	30.18518	28.17803	28.33683	27.57374	29.17878	30.86556	27.15694	28.5138
Acap2	31.23247	30.65543	30.47612	26.85482	23.44346	18.42878	30.86838	27.16757	28.50599
Nkiras2	24.75848	22.00083	24.18295	26.45464	24.18297	23.83819	26.30072	23.52006	28.50542
Rcbtb1	27.71903	28.96981	32.34977	30.84415	29.09228	27.89199	43.0842	25.90175	28.50116
Mrps34	25.2322	27.40326	26.93366	28.5288	27.49687	32.86946	27.20715	28.3547	28.49982
Kmt2d	35.62935	33.74645	33.30256	35.36428	28.64338	19.99672	44.78108	27.43629	28.4831
Brpf3	31.08263	28.91853	29.76029	27.22196	27.10079	23.81731	35.09439	25.52633	28.45451
Gm8129	27.89559	21.65837	26.30965	28.09046	31.6119	40.32817	19.16016	23.70168	28.43796
Ext2	35.25946	36.97586	38.37277	29.99275	28.83602	30.44615	33.88785	27.77928	28.43392
Hs6st1	41.27669	42.7873	44.35262	34.03409	28.70631	35.75897	34.98891	40.46325	28.42905
Fto	40.59312	40.89719	42.02846	30.2506	33.99818	32.51246	44.97762	33.01931	28.41828
Slc28a2	27.22324	22.72304	29.14217	28.43774	28.00902	22.27839	23.89746	26.71308	28.41621
Erlin1	32.48172	30.12159	31.76085	24.81354	24.23653	24.75935	27.26998	25.99583	28.41463
Lats1	35.70315	36.06769	38.73301	34.38746	26.1417	24.08659	35.3065	25.94296	28.41418
Lsr	32.06023	31.94685	44.17575	28.05273	32.70142	33.10423	27.70517	38.87123	28.41383
Kif1b	41.05269	40.0531	37.21955	30.52777	26.44328	20.37221	40.59537	25.60482	28.40878
Asap1	38.10674	35.99774	37.25235	32.19139	30.0048	28.1185	41.10302	34.9363	28.40758
Crybg1	33.61122	26.29319	34.28167	28.30181	22.51875	18.86808	28.1089	24.82651	28.40371
Dym	38.37658	37.07907	37.18237	31.0808	30.17063	29.40431	38.23106	33.2939	28.40094
Eprs	36.1054	32.43954	33.03964	31.46853	25.1196	24.1126	34.91036	29.85419	28.38382
Ank	46.23008	49.33149	47.18648	25.29436	27.56154	27.99334	34.93901	30.85768	28.38235
Zcchc2	29.5402	23.55971	26.06341	26.74146	21.00472	17.54885	29.1381	24.81204	28.38222
Cd209a	62.01293	49.77633	49.01832	38.85673	63.18755	71.3736	40.00049	31.58412	28.37696
Cnot1	39.7203	34.97549	38.45377	28.31481	26.58626	21.98756	41.53435	26.23601	28.36895
Apba3	27.89006	27.21225	29.34059	27.00124	30.87227	28.39537	31.4364	29.45998	28.36217
Tk1	47.09255	48.97368	50.82413	35.41795	30.37339	33.48918	37.73659	29.51278	28.33619
Sf3a1	35.19573	30.65944	37.27504	28.80851	27.56998	25.46683	37.5887	29.36535	28.33185
Trim27	27.07333	30.17517	32.10431	32.03542	28.85914	28.70462	31.03245	29.94812	28.33087
Trip4	27.98524	33.81812	32.24834	24.38715	27.06483	28.00319	31.67555	30.44655	28.32161
Timm17b	28.67009	26.76569	25.83721	22.93828	29.80832	28.3042	27.45157	26.14428	28.31399
Uggt1	45.18426	39.37509	40.60854	33.14123	32.17972	26.76079	40.04324	29.22941	28.31181
Atg101	22.13015	27.24067	24.88241	22.18547	31.03528	27.46765	25.52789	26.13376	28.30882
Erg28	30.76508	28.75915	27.8238	28.62391	30.60578	27.93415	27.54712	34.07659	28.30575
Mthfr	22.95551	17.16253	23.34506	34.39975	25.5888	20.00205	28.1007	24.26562	28.30424
Sec24d	35.21474	27.13634	32.27195	29.01449	24.25697	24.97574	36.05451	22.58	28.30232
Sort1	31.72865	30.7592	34.36979	30.71803	27.8328	25.08536	39.29006	25.40033	28.29587
Mpp6	29.65173	29.34453	27.97659	25.59131	24.4133	27.46803	30.22601	25.47117	28.28497
Ssh2	30.10871	30.96463	35.30589	22.95854	28.94573	22.64148	41.14505	26.9909	28.28431
Hspa14	32.58771	31.82116	34.60412	30.66227	29.1196	30.28618	35.25773	30.43954	28.27481
Coq2	27.83357	24.63701	25.12568	27.94343	21.12671	22.80904	29.31985	22.39256	28.27456
Qsox1	31.68524	29.91669	32.00004	23.88491	26.79079	28.08918	27.28258	22.89003	28.27171
Vps54	26.01392	25.47955	29.67535	26.31745	25.60816	19.56894	27.48918	26.52999	28.26606
Borcs6	24.5195	30.56011	28.24531	25.63644	33.3727	30.78319	32.65541	32.7181	28.26596
Zfp263	30.52342	32.82978	29.36404	25.81233	26.95302	21.12684	32.6079	25.49579	28.26035

Gtpbp1	31.32463	24.83027	37.54497	26.18476	30.20615	27.80629	34.85432	28.73678	28.25355
Zup1	19.46259	17.56868	22.41491	31.71683	26.70123	22.76568	29.91691	27.58547	28.24476
Ccdc86	25.73449	18.9641	24.61123	25.8657	22.6983	24.95935	27.79993	26.92732	28.23977
Mtfr1	32.23903	32.09889	34.20349	24.15214	24.68776	24.12287	26.11618	27.58377	28.23531
Cluh	43.52868	38.09801	33.3856	30.5711	26.59794	25.50832	39.82621	28.12881	28.22765
Nbr1	31.28055	32.58775	35.09964	33.32354	34.12475	28.2709	40.9103	27.59797	28.20652
Vps39	31.74555	28.29827	32.62239	34.3967	29.37397	26.47585	41.32053	26.88567	28.19047
Srek1	29.98087	31.44329	25.16008	31.55718	25.9377	26.55147	34.30247	29.02816	28.17848
Erdr1	26.12791	57.8796	71.69259	46.38373	31.98224	43.36818	124.6361	56.52986	28.17462
Atxn7l3b	27.04897	30.86373	32.5981	27.67864	27.11081	23.74624	28.6888	24.91731	28.17309
Tbl1xr1	23.6423	25.90466	26.89045	26.47911	22.43876	21.08192	30.83326	29.18224	28.17302
Mcm3	48.21305	40.40381	36.24836	30.25796	24.80192	34.47195	40.51685	27.32669	28.1686
Rab3d	27.53786	25.52757	28.06289	23.92463	23.52126	25.30531	27.41316	24.30063	28.16613
Itpripl2	34.66797	33.07764	39.33208	25.4478	28.91041	23.37464	33.53582	27.3946	28.16199
Dnajc10	34.36372	33.00861	33.95941	25.9119	23.23514	22.32696	27.00485	24.82304	28.14953
Zfp971	31.51318	37.93688	38.3326	29.02966	28.03343	28.42725	36.56682	34.69746	28.14616
Ube2e1	30.87892	27.60534	28.84234	24.14315	27.96117	24.11712	25.09371	27.91454	28.1342
Ccdc22	31.79825	29.82646	28.06632	27.22166	26.46951	28.06526	33.34764	27.63125	28.12842
Smim14	26.58721	30.52685	33.88822	23.94964	29.65988	30.04791	33.62607	31.73909	28.11384
Sypl	27.84911	30.72791	29.9834	25.18004	24.29326	20.08307	28.0024	28.07202	28.11304
Actr3-ps	27.19479	24.11606	29.33884	24.26219	23.86131	23.3107	26.03018	27.57666	28.105
Elp2	31.96504	30.50652	31.86442	32.42918	28.43613	27.47639	30.08307	26.26211	28.09908
AU040320	34.44448	32.49829	33.98357	31.74642	28.363	26.77774	38.25033	27.19789	28.08371
Alad	36.17789	28.94313	27.98256	27.39122	23.92049	26.91738	28.33516	22.75676	28.07245
Ccdc6	35.04005	37.08379	37.54427	31.41788	30.97091	26.55647	38.58225	27.34128	28.07049
Aga	25.56311	29.88318	31.81459	25.37616	23.42414	24.73855	32.37792	29.44927	28.05414
Srxn1	36.4527	36.65058	37.79769	25.30926	17.68716	20.74761	25.8167	24.19509	28.05343
Erf	31.77687	34.08629	31.43761	25.65581	29.99956	26.6379	29.11312	30.98541	28.04506
Myc	39.68125	31.89125	32.37348	32.28043	24.85677	27.15288	30.39473	26.54544	28.02852
Ddx27	33.13023	29.65037	28.21424	27.73391	26.13449	27.11712	32.20802	32.05232	28.00871
Acox1	32.42484	33.20911	31.33337	30.3809	29.77763	26.72714	35.48113	27.41122	27.99943
Ube2w	23.90278	26.99541	29.26755	25.69409	23.36755	23.10303	28.70672	23.85668	27.99779
Rpl19-ps11	28.27026	29.68721	28.27355	30.46109	30.1492	31.16824	27.9165	34.41357	27.99481
Gm7331	27.77224	33.32286	40.33295	28.03991	33.9492	42.80482	29.03392	32.01394	27.99064
Taz	32.70712	29.51496	37.56566	30.79285	34.84835	31.4539	33.59684	32.9253	27.98212
Cd300lg	44.28008	37.04411	39.66067	38.33437	35.56606	40.8094	28.93471	35.45948	27.97556
Paox	31.92201	20.67606	25.78814	40.30719	42.33711	38.96321	48.18362	37.07048	27.96696
Spsb3	24.30587	26.6137	26.51544	26.63811	31.16037	25.53804	27.54438	27.57293	27.96308
Plekhg3	31.5458	31.97932	36.9748	31.56845	28.95444	26.72971	37.18703	28.9905	27.88547
Larp4b	34.88569	32.41821	34.49923	27.90943	25.07956	23.41865	39.31425	26.84008	27.88464
Spata6	31.47883	28.17658	33.55291	24.49103	24.76112	26.91467	31.33927	27.97094	27.88399
Zmynd11	32.29615	33.15967	33.83799	31.57635	26.51787	25.93845	32.60764	28.08557	27.84957
Wdr61	33.17196	29.95656	32.85769	26.75041	30.85689	27.81886	32.07749	32.66456	27.84791
Mgst3	42.24815	43.24451	39.22054	33.46849	30.22564	35.1441	37.97233	27.76252	27.83958
Ptp4a1	19.79068	16.84558	14.5426	17.04961	16.75131	16.7125	28.72533	22.40922	27.83273
Ly6c1	34.28054	32.58665	21.78496	66.57914	28.99247	42.43387	19.65294	33.82838	27.82259
Kpna3	33.30009	34.48523	34.15552	27.79528	25.59472	23.80667	34.2523	27.46158	27.80716
Kdm3a	25.887	27.50076	27.0831	24.77603	25.79065	22.26893	33.90675	28.23286	27.7912
Isy1	24.53648	25.11962	24.06018	19.80507	21.38018	22.94374	22.67162	22.8724	27.78856
Ubqln4	37.17918	36.4379	43.65928	29.94368	32.17648	27.05823	39.9377	31.86835	27.78733
Rab12	28.6682	32.82771	37.52077	28.95555	29.72411	28.86208	30.49464	29.43932	27.78333
Ranbp2	36.32109	31.4287	34.28351	29.89346	23.98417	18.96087	32.25545	24.78999	27.76514
Ubxn7	25.20723	29.29061	24.49666	24.47485	27.29107	22.40371	32.84266	25.89596	27.76442
Il10	19.91506	12.50415	5.672399	15.60533	19.21057	14.59149	42.92943	29.23337	27.75996

Zc3h4	31.60497	28.3955	36.07487	30.11287	27.5721	25.76219	35.77535	27.02535	27.75676
Slc30a5	31.15769	29.72841	30.72344	27.39143	25.17446	23.80332	32.71209	26.78202	27.74876
Ddx31	27.27064	23.78303	28.33377	24.96953	24.80758	24.76667	26.79524	26.04194	27.73963
Cysltr1	26.37364	25.65225	32.66912	25.22251	28.45321	23.78572	22.8415	26.29705	27.73869
Dnajc14	30.81785	31.10171	33.48161	27.14031	27.75327	26.85146	32.50182	30.60161	27.7362
Ddx42	29.8488	26.63908	28.8273	28.05739	26.41455	23.63453	33.1011	28.08357	27.73124
Rce1	35.41755	33.88771	29.00912	29.49388	25.46463	27.79168	30.11414	28.82064	27.7282
Spryd3	31.14268	33.66888	31.87433	24.45337	28.25169	27.53755	25.90989	27.93984	27.70874
Triap1	27.97906	28.00831	32.76739	24.0989	28.7272	28.03835	31.39028	29.36211	27.70742
Sun1	36.73607	36.21193	37.0877	25.71732	23.25683	23.39769	29.15985	26.81095	27.70462
Pten	27.46379	29.48146	28.97137	23.39113	24.08835	21.48258	28.95501	26.33615	27.69341
Tmem229b	32.93102	27.39548	40.652	31.67265	34.76181	29.70092	34.6344	30.93631	27.68668
Mrpl16	26.98572	22.62326	26.30436	20.27176	24.6766	24.91034	24.45523	25.36724	27.67673
Eci2	33.05574	34.35794	34.49349	30.96963	34.32668	32.33676	33.54732	28.09756	27.67131
Dpy19l4	34.32873	36.61286	38.8588	30.31718	27.30669	19.63139	32.15704	27.55347	27.66727
Itch	31.42238	33.51325	34.47244	26.95265	25.27452	22.98977	34.37659	28.76731	27.66655
Sowahc	27.07412	27.5656	23.39161	28.37608	27.64527	24.7533	45.83333	33.67178	27.66558
Shmt2	38.00501	39.49319	33.42125	23.74613	27.50311	28.75975	32.34243	26.57515	27.66139
Rnf216	29.39011	31.07576	32.94416	26.29	27.29724	26.10872	35.37611	28.08131	27.65194
Ptpro	26.98601	29.25629	31.10268	24.45913	28.50659	23.11049	26.1096	29.37673	27.64778
Map4k2	22.4348	25.80222	20.59544	22.59184	24.79239	23.01031	24.1201	20.12442	27.63802
Il11ra1	33.81061	38.55576	30.63459	37.90996	29.95219	31.55812	33.74107	29.85312	27.63387
Marf1	26.7454	30.49399	33.77456	32.07303	30.39019	22.65396	38.62862	25.73056	27.63376
Il18bp	25.3581	20.81387	32.3572	41.15725	36.7536	33.47744	37.29492	37.96342	27.61324
Brd1	30.33492	30.00848	28.81331	28.39196	27.04727	24.08244	34.08055	27.49692	27.6038
Ptcd2	26.61027	24.69091	27.7719	29.29164	25.37485	26.4754	27.12676	28.39224	27.60163
Tnip2	31.94879	32.70744	31.13466	28.09653	29.46087	22.21761	37.39234	31.78761	27.60122
Apip	22.04851	25.21245	23.88866	22.05101	28.90346	32.16662	27.43756	25.03397	27.59622
Clpp	30.81934	29.17981	24.8972	28.63708	27.60225	33.21549	27.86572	29.2993	27.57876
Slc12a6	31.5539	32.63029	35.28117	26.64744	26.11563	22.19062	35.99885	28.97896	27.5631
Strada	27.98841	28.74675	28.4766	29.46564	27.06634	24.91127	31.60021	25.44228	27.54836
Arid5b	25.60957	22.0842	24.39114	28.69232	25.74138	21.70935	36.73243	26.77882	27.5474
Ptprs	43.09049	52.34444	45.82572	31.0858	33.78577	28.68902	43.89953	30.36325	27.53949
Thap11	27.02906	30.93634	38.58314	31.32246	30.34205	32.98535	33.70288	36.01661	27.53377
Acsl1	28.98415	28.89081	28.69553	20.35428	21.19359	22.10887	27.15704	21.77393	27.53172
Dynlt1f	33.37894	29.74396	32.80225	29.94493	32.96457	28.72047	27.82403	26.18397	27.52524
Scfd1	26.66868	24.27262	24.80142	24.78701	23.00789	25.65738	25.44519	25.85463	27.51744
Nomo1	42.17406	34.20765	32.81888	27.10426	25.77326	24.2193	31.29259	28.93803	27.51664
Ddx1	32.82724	33.56841	32.17982	29.51745	27.79999	27.76205	33.48766	27.94115	27.49942
Slc35a2	32.98703	31.35877	39.39043	27.15236	34.62117	28.82196	31.18608	31.56444	27.49648
Cacfd1	28.27945	27.73122	26.42884	23.31989	26.23508	23.8796	29.40644	29.67475	27.48695
St3gal3	35.8629	33.68099	32.62907	27.83618	34.58237	34.22432	36.34537	33.71818	27.48684
Akr7a5	28.18061	25.05274	29.98182	30.53514	32.80825	33.29459	31.5365	26.87696	27.46063
Hat1	33.17138	30.90793	31.16994	26.58434	25.36075	27.32526	25.23148	29.17102	27.45801
Ufd1	28.14115	28.71175	28.20071	25.6329	25.94368	26.31032	32.22649	28.0792	27.4379
Flt3l	24.19115	25.35543	26.93819	32.18557	29.81255	24.44263	29.16989	33.80211	27.43275
Mcur1	34.25929	33.68614	36.51305	29.75925	29.29553	24.28536	37.61312	30.09568	27.42717
Fbxo22	27.27067	34.56312	29.9643	26.04264	27.52962	29.99947	39.77059	29.68072	27.42417
Pik3c3	37.79467	42.71405	41.73793	30.57567	30.66322	29.67596	40.24916	31.66276	27.4034
Tmem141	30.34063	32.00293	29.99457	34.41125	32.35829	28.51061	35.26031	24.22802	27.38483
Nup153	34.72192	32.57024	36.29762	28.8698	26.51401	22.18086	33.90695	27.31408	27.36437
Abhd11	32.79358	31.61788	30.55996	27.23821	29.25749	29.72095	32.20193	26.75489	27.35479
Atg9a	28.97066	34.55654	33.29418	25.74237	27.89662	25.60546	34.50185	28.33861	27.33521
Nek9	33.51178	32.25543	37.5482	31.74778	26.4449	26.52406	35.31783	28.60443	27.32619

Poldip2	27.2786	28.99136	27.70804	28.3408	30.01212	26.40888	30.30832	28.10917	27.32595
Hdgfl2	29.37245	29.5125	31.24833	27.34615	27.37191	29.18643	37.49501	28.1275	27.29932
Gm2573	31.07085	27.16768	29.15891	26.24113	30.80763	27.06052	30.24367	28.26547	27.28073
Spg7	30.95292	29.28091	31.9499	26.18557	27.36728	24.9074	30.20082	24.19197	27.25529
R3hdm1	38.19582	35.6265	36.38304	27.40999	24.76699	23.85804	39.67707	28.17028	27.25388
Ubn1	31.51165	29.12158	32.36682	29.1512	27.05353	24.1932	37.12871	27.67165	27.23938
Guk1	26.1523	27.64524	26.69773	26.78479	24.39066	28.75432	17.43852	23.57999	27.22275
Taf11	23.73743	25.84943	27.74762	20.42694	23.38017	22.37378	25.16289	21.15162	27.21634
Mrto4	27.19344	22.60847	25.68822	21.86013	24.46234	23.07666	21.11476	28.89329	27.18099
Spata2	33.82408	29.35725	32.78302	27.99421	29.3989	27.22113	41.4076	27.2013	27.17271
Snrnp40	28.95602	26.02927	29.58667	24.73982	27.77651	27.7121	29.38729	29.99079	27.16593
Ubqln2	39.54393	37.99456	40.99664	33.94373	31.36849	26.91884	39.13328	27.61929	27.1563
Pik3r2	28.64767	31.33243	33.90807	35.45655	35.16013	31.32107	36.47224	30.39496	27.15348
Nsun2	34.6747	28.8854	28.79746	27.31904	25.47409	23.01043	31.75488	27.58747	27.13472
Tomm20	25.52864	25.58265	23.3076	24.32422	22.57654	24.1222	22.98356	26.79351	27.11811
Casp3	22.29021	25.18617	26.53905	22.67111	26.76786	23.06726	25.82638	27.34919	27.10367
Plpp5	30.59064	25.61659	27.36326	22.80455	23.62643	24.45344	31.78184	27.11272	27.10292
Map3k1	32.48916	26.12025	30.63695	32.18588	27.48216	22.04425	37.73942	29.82131	27.07055
Isoc2a	24.8656	22.51257	24.40985	26.44905	29.99038	28.27245	27.17442	27.07042	27.05003
Gapt	25.17173	24.88618	25.88689	29.66101	36.25991	34.09382	35.3583	31.4765	27.04523
Nop2	28.61728	24.99981	26.61252	28.01563	21.0349	24.04075	27.1745	27.60867	27.03309
Phf3	28.95573	29.60185	31.57188	26.39325	24.65385	22.17514	36.33357	26.44325	27.02934
Aagab	23.51525	27.33268	29.65687	26.86527	29.47665	29.76107	30.29088	26.54557	27.02638
Ctcf	33.53936	31.96735	33.32023	29.84781	26.83115	26.94665	34.75679	26.07276	27.0209
Fndc3b	35.66403	34.00616	37.0248	28.99641	23.33409	19.73561	30.49508	26.80836	27.00489
Dlg1	35.1575	33.05703	32.33509	31.20596	24.71974	24.23283	34.60781	29.83544	27.00051
AA467197	20.52941	23.75762	25.29656	12.83438	23.51188	20.98759	19.06949	22.10266	26.99661
Gm9222	20.08161	33.02514	30.66006	31.12209	27.56921	21.23793	30.24981	28.47609	26.97863
Nploc4	35.42527	32.08908	32.33	23.40694	26.58951	22.53217	33.18302	26.29288	26.97661
Slc25a36	25.82453	28.69024	33.32175	27.86897	27.23594	21.8459	35.3108	30.39174	26.9735
Ms4a8a	44.88331	42.20881	56.62309	25.88242	20.93401	18.33686	20.75861	39.31855	26.95586
Chfr	26.65291	27.62658	27.75966	22.07705	26.00741	22.90082	30.15684	26.44466	26.93566
Trmt112-ps2	30.78889	22.51241	28.08346	30.16751	29.66697	32.81447	26.31602	23.81229	26.93503
Bid	25.49558	24.83636	23.66028	29.50944	22.5594	24.50965	24.38995	28.3106	26.92729
Hagh	23.61531	22.19654	21.90587	26.2369	26.13555	25.51728	26.9056	26.65491	26.9226
Ppme1	32.47388	28.52342	32.57159	26.29123	29.49655	26.62169	29.80176	28.77215	26.92223
Dag1	36.51202	40.7739	41.67101	34.23688	26.28149	26.02454	33.49664	26.60715	26.91976
Insig1	24.80895	20.88009	18.48848	19.40289	21.09551	16.37923	21.96279	25.17532	26.89759
Actl6a	32.37876	29.9338	29.48217	26.96181	24.61611	27.54743	29.21645	25.81489	26.88831
Ptpn22	30.29874	31.71463	30.77509	26.30668	25.68475	21.95544	27.72484	30.74574	26.87909
Shoc2	30.05219	28.00479	28.47739	24.37787	21.50814	21.53859	26.16726	27.01944	26.86603
Akap10	28.88382	28.19401	30.68816	33.76721	25.95693	22.53439	39.17358	28.12616	26.86139
Dolpp1	31.03731	29.10025	31.58449	30.46521	29.83203	29.52239	33.52732	24.13069	26.85492
Nsmaf	29.51542	25.19594	27.40387	26.52362	26.13942	24.3089	29.42457	25.65514	26.85215
Akap17a	28.55317	25.00017	27.6153	26.22386	27.92704	29.7873	29.4006	26.92069	26.84678
Fbh1	28.1536	26.56172	28.80879	29.97135	27.0349	22.46177	32.3384	23.3029	26.83288
LOC115489087	9.929205	14.85416	4.13765	24.68397	13.39666	9.182218	18.81427	20.48275	26.81301
Plekhg2	30.91122	31.0256	26.29238	27.96821	29.193	27.75369	41.31112	26.55552	26.80635
Fam241a	17.70698	17.48738	18.43538	20.63413	18.70843	18.00595	17.26446	20.64542	26.79776
Tbc1d2b	30.91128	29.85013	31.62875	32.04928	24.91694	23.48838	36.40555	29.66654	26.79665
Cyp4f13	30.17127	32.84237	32.05796	29.39264	34.69861	25.75518	36.93142	29.76831	26.79432
Clcn5	37.76353	34.31433	35.74935	26.82285	23.3363	22.13621	39.92729	29.56106	26.78725
Tmem63a	31.27056	30.38501	32.38685	22.58544	25.88582	23.50481	31.02831	28.12675	26.78574
Elane	0.060259	1.08177	0	8.928262	6.312877	15.54739	3.736826	0.229489	26.76329

Cdk17	30.25603	28.34808	28.38007	28.54485	23.22244	20.57212	28.13915	25.54135	26.75453
Cog1	31.00432	28.27499	29.06764	27.73666	26.40802	22.34168	31.28785	24.84906	26.74141
Polr2c	26.38571	27.57525	22.94981	22.50459	22.72345	24.30334	26.47313	26.55827	26.71967
Idnk	22.06941	24.49816	23.69714	26.71825	28.64873	27.50336	26.79375	27.45264	26.68883
Ppie	23.28585	24.89933	22.54183	20.74243	23.1105	26.10839	21.89671	25.32492	26.68555
Baz2a	33.1803	31.26643	34.24925	31.72364	28.33591	22.15288	40.24413	26.74929	26.68197
Ccdc107	22.67921	26.55845	26.63243	30.67908	27.89115	30.73524	28.43783	25.15921	26.67834
Ss18l2	18.65807	31.44178	28.41933	14.07511	22.97812	22.70838	18.72991	25.01297	26.6742
Mtg2	21.01864	21.0736	24.74705	26.8173	22.2951	21.49425	22.58587	26.62947	26.65718
Pygo2	28.55129	32.55712	34.15585	28.37188	31.79852	29.19217	31.22954	28.7684	26.652
Prmt5	39.6991	33.25759	36.42805	28.32882	25.16412	25.41008	30.71646	29.59334	26.64916
Fbxw5	31.98445	31.80008	30.91858	30.54396	29.0641	27.39321	37.09332	27.23187	26.64389
Ccnd2	34.51981	24.85421	29.20765	20.55234	21.64951	25.30116	19.58409	25.90003	26.64221
Pcyt2	28.87537	28.10654	28.35005	26.4139	25.80146	27.86404	28.03436	31.58141	26.63912
Rab18	28.05136	27.31379	27.19349	23.95869	25.62243	25.18667	26.60376	26.00292	26.63466
Ppfia1	31.88034	29.38254	31.66282	30.76693	22.02795	23.37077	37.13654	27.04937	26.62952
Sp2	39.92708	31.81295	41.68376	28.5255	30.32705	28.48563	31.68553	29.55332	26.62741
Top3b	27.38151	27.1403	26.75176	30.66836	26.29885	25.12648	32.48631	27.97792	26.61975
Gm6485	24.51953	21.05452	21.59166	29.97225	24.26344	23.17264	24.29139	26.53358	26.61819
Sars2	31.39752	25.42091	27.51948	29.89675	31.28193	23.83907	34.54645	27.85282	26.61194
Zswim4	34.19616	28.3192	26.71487	19.20842	23.90995	17.48609	26.05293	30.43368	26.58883
8030462N17Rik	30.07011	24.51224	25.13379	24.79363	22.99355	21.63242	27.97515	21.7074	26.58817
Tcerg1	36.39032	29.24041	29.89033	27.90552	22.87436	22.46768	32.28307	27.59942	26.56988
Stub1	27.34318	22.81903	27.34636	27.23673	26.44405	27.50191	25.73554	23.57319	26.55303
Trim35	33.99419	34.80418	34.81084	27.32434	25.58239	24.56214	31.96183	25.71784	26.54778
Diablo	27.36164	25.89157	22.53237	22.631	24.45237	26.78384	21.66036	24.98933	26.54132
Sgk3	26.40446	26.46732	30.50578	22.22258	23.37693	21.03691	24.42436	25.12985	26.52637
Slc39a11	33.48709	28.4947	30.53653	29.63292	27.66228	25.98265	35.50203	24.65645	26.51118
Tent4a	23.76314	17.08273	24.30729	25.68661	20.14649	16.40454	22.55194	22.41339	26.49807
Pip4p2	30.61251	28.979	32.31831	26.62079	26.12141	26.21832	33.70027	26.13658	26.48822
Glt8d1	25.82191	28.94165	31.4818	33.01363	26.90119	24.36663	27.99541	24.81913	26.48567
Ssbp3	30.34171	32.74134	31.04621	27.02418	29.43544	28.68632	33.71983	27.4245	26.48335
Ly6g	0.638687	0.764384	0.83039	5.461944	3.102222	16.59693	1.518265	0.304046	26.47957
Gtf2i	32.98715	33.57991	33.08653	25.85375	28.81696	26.56766	34.33366	26.79405	26.47073
Coq8b	26.94491	28.49857	30.47954	27.28016	31.06865	28.10789	34.16544	29.37819	26.45753
Oaz1-ps	32.00785	27.38687	28.03891	27.07717	27.13947	31.40992	30.34474	31.68044	26.44613
Mitd1	20.72659	18.00413	19.72599	23.71315	21.27863	19.83713	20.27025	23.1288	26.44525
Mrps10	25.62034	26.422	21.31642	24.93477	21.90857	26.91916	22.31073	22.05757	26.44468
Cnot6	29.88107	27.28378	28.89714	24.89189	22.94763	21.4269	28.02901	24.51125	26.44252
Zranb3	33.27979	30.72256	26.75579	33.34625	19.19802	26.08836	35.44766	19.85396	26.43486
Ints9	34.46335	30.50195	32.64127	31.00214	30.06195	28.62024	33.96835	27.68418	26.42958
Gm25835	25.02413	29.18108	24.642	41.34564	21.63276	19.58129	45.75882	26.75778	26.4187
Dmac2	27.83263	33.23471	31.81242	34.7936	35.12694	31.41178	35.9678	31.97954	26.4187
Snord55	24.16531	17.01245	31.98722	26.15024	20.30716	37.46463	32.32195	28.42133	26.4187
Gm25767	12.96535	17.38975	16.09679	9.78069	21.71555	15.71097	19.86947	14.89828	26.4187
Med24	33.73207	29.48607	33.58831	27.22931	29.81348	24.95852	36.40687	26.77394	26.41088
Cops3	27.32962	25.20907	25.95199	24.95876	22.82545	24.16049	24.99013	23.82963	26.38496
Usp48	30.78173	29.29791	27.53525	36.9825	26.8353	24.61407	43.72988	26.94988	26.37568
Phf1	27.12733	31.75344	30.08934	23.51293	34.70056	33.88362	32.7182	31.20146	26.37532
Psat1	33.13011	31.7182	30.78549	25.68557	19.25253	25.77531	29.09747	26.36693	26.37005
Galm	31.93715	34.85704	36.15946	27.45481	29.68617	29.93975	32.15935	29.10338	26.36649
Prkd3	34.00183	30.77827	33.95042	24.04089	21.28551	20.05392	26.96124	25.25811	26.36594
Il16	24.85393	25.82058	30.34603	33.22755	41.27562	35.67329	37.76459	28.62611	26.3529
Zranb2	23.88407	25.76259	26.3113	24.91606	20.23	25.00893	22.57007	25.76693	26.34295

Ilf2	31.44206	30.4616	28.52315	27.96891	21.56902	23.64588	32.61311	24.63895	26.32497
Phf11d	16.65619	10.55994	18.08242	24.58778	17.02293	18.3425	15.78575	19.08108	26.32034
Arsa	33.44387	31.97693	33.73364	28.78759	27.82854	28.2257	32.97384	28.22826	26.32032
Nr2f6	25.13651	28.53813	23.45199	27.598	27.97342	24.42365	26.65603	28.12879	26.32024
Cdadc1	19.01037	20.65449	21.80959	22.49456	16.62178	18.13674	23.19728	20.89727	26.31748
Kif2a	30.35067	29.73695	25.62471	22.9543	21.80646	19.2963	27.39259	28.26088	26.30411
Blmh	31.99165	29.51173	33.14899	25.56232	27.39698	29.5631	30.80224	26.04383	26.2953
Rusc2	36.9052	32.87542	31.32579	33.62992	27.06183	25.2708	40.65061	27.5121	26.2827
Nolc1	37.65061	27.33546	31.69989	29.51485	25.94476	26.5094	32.72829	31.07227	26.28152
Tug1	25.88516	29.15868	28.47815	27.30158	27.61018	21.74881	32.47008	28.38271	26.2791
Med6	21.41259	24.10548	21.06305	24.61779	20.56073	23.21951	23.82869	22.78862	26.27892
Ankle2	21.21822	20.46024	24.88714	25.33831	23.57769	19.81905	26.28086	22.68774	26.2765
Pacs1	27.89006	27.05481	28.27486	23.79615	28.93023	24.62392	33.32639	26.91731	26.26824
Ddx50	26.69156	28.87266	26.24298	25.14983	23.78626	22.13582	26.56013	26.91798	26.25728
Mavs	34.54597	34.55654	36.99728	37.8695	35.72131	34.87551	45.18381	30.22854	26.24231
Rps6kb2	31.42565	28.6096	31.17142	27.59581	30.17502	28.13745	34.07899	27.40392	26.23438
Secisbp2	28.51695	25.37318	25.32822	27.09229	23.26046	23.22753	28.63705	23.29739	26.23133
Rps27a-ps3	15.45486	22.37474	15.55637	19.23514	17.85277	19.548	15.14893	26.20137	26.22864
Tbcd	30.50597	27.6337	31.61979	25.68938	24.60411	24.3611	29.25856	26.09396	26.20929
Rexo1	32.2975	26.82889	27.52684	30.19463	26.41439	25.66788	34.95298	27.20072	26.20228
Mbd1	29.84826	32.18459	33.7981	31.0408	32.79913	27.24144	35.67497	28.39987	26.19529
Mir142b	15.90831	23.07776	13.49953	23.96849	18.82385	18.27794	29.39637	27.0797	26.18897
Alg5	27.24794	27.70162	24.27346	26.27083	24.26367	29.20603	27.07146	26.98329	26.18522
Apool	21.1475	26.26544	23.22542	20.09551	26.68165	26.8674	26.60629	27.04984	26.18324
Fam207a	28.40402	25.91456	29.38924	25.92901	26.35581	31.77818	28.6018	28.36949	26.17932
Itpk1	26.34084	30.43299	29.37239	23.02771	27.32365	25.65164	27.84651	28.77532	26.17819
Sptlc2	30.29678	25.89011	31.29185	24.3432	25.49441	23.93751	29.37483	25.817	26.15884
Kdm3b	30.79026	31.31468	32.10355	28.928	27.96574	21.31576	34.53832	26.3096	26.13638
Mrps2	30.43336	32.15707	33.0967	26.8549	24.98925	23.80815	29.69748	24.43343	26.13126
Wdfy3	37.76758	32.63749	38.9279	32.49556	28.7624	21.48859	48.44864	25.20047	26.13097
Hvcn1	26.35763	30.44982	30.98107	25.94367	25.48532	26.7482	27.77246	23.62887	26.1209
Letmd1	32.48451	35.30316	38.99061	31.14392	27.74202	28.70456	36.05511	31.39436	26.11887
Ccl22	41.57089	36.94173	33.34018	18.44687	32.68207	23.52468	35.75193	18.85713	26.11235
Kdm4a	33.53215	33.19721	36.11448	29.8172	30.46555	29.61238	38.66258	30.65949	26.10568
St3gal5	27.38304	27.42184	25.20205	21.04869	18.69284	23.5105	23.75842	23.8854	26.09847
Exoc7	28.3496	25.41397	28.33562	27.24595	27.37827	24.18785	30.10422	26.20208	26.09762
Pmvk	23.52433	21.65261	20.01218	16.85392	23.75397	21.28278	19.39227	26.60711	26.09454
Sft2d2	28.11102	32.29085	36.56588	26.81962	31.36887	25.61015	34.17886	29.96338	26.08392
Ppp1r8	26.29162	25.40813	25.80522	23.06218	24.23712	21.79932	21.94691	24.48858	26.06887
Pwwp2b	23.10117	26.09426	24.57604	24.88412	22.79272	23.71254	30.23175	26.62859	26.06796
Lsm8	20.84471	23.88548	24.09988	22.27152	21.61223	20.57531	25.8987	24.20956	26.06645
Sec22b	31.12473	27.23398	27.76807	25.63506	25.42478	24.1225	30.77992	26.49302	26.05481
Mapkap1	27.80709	25.4301	29.45428	24.77139	22.7185	20.73859	29.04339	22.97239	26.01771
Alg8	41.71854	37.69805	39.16318	30.50708	31.96106	28.99973	31.79088	28.02879	25.99935
Nlrp1b	22.81217	23.62197	26.51899	41.40429	31.54436	25.08829	49.91022	29.19881	25.9915
Sec16a	38.5089	31.80666	36.81958	27.60451	25.82046	20.70146	35.74244	24.2391	25.98913
Coasy	22.69205	23.79947	23.30042	19.1521	23.30368	23.59868	23.16125	22.91213	25.97614
Tmem245	44.16444	46.77343	43.2112	40.63798	30.11695	24.0829	47.51045	28.30987	25.97548
Jagn1	26.92706	28.79181	31.01826	19.56138	22.67664	20.88496	22.17987	27.43715	25.95607
Cpsf1	30.65722	27.19633	28.59756	25.99722	26.81607	26.14243	37.2941	27.58164	25.95396
Paqr7	29.13698	32.36332	31.37421	26.55305	31.35888	27.8501	33.97935	28.21915	25.95228
Phf14	26.26014	25.85445	29.18132	25.62202	26.33517	23.66187	26.97878	25.3655	25.94808
Ptov1	31.18391	33.58767	29.60063	25.95373	29.85342	31.71271	34.87414	28.73438	25.92489
Rpl37rt	28.98829	27.03271	34.47878	27.27876	29.71384	38.99956	25.22128	33.44709	25.9114

Mkln1	27.32135	25.70388	27.1152	24.60133	24.18662	23.67874	25.1517	25.06935	25.90668
Rnf103	24.45036	31.64192	28.66418	23.65232	28.70331	25.88986	29.96598	28.48354	25.87308
Nutf2	32.47968	26.4014	26.74319	26.77699	24.39413	27.35749	29.24499	24.08025	25.86911
Mtus1	28.08313	28.60832	35.97085	27.72118	30.83185	27.41971	34.21192	30.30998	25.84536
Palm	24.27479	23.37608	25.04797	29.25983	24.16705	24.28027	26.47673	27.80561	25.8209
Nr4a2	39.25243	29.93666	15.2501	16.14043	21.14121	13.10754	28.28277	30.62472	25.81951
Med7	23.00185	21.91008	20.93771	16.52932	21.99229	25.59215	24.65579	29.68479	25.81925
Cdk10	26.66946	25.94637	23.88485	24.8091	25.62912	25.36319	27.99102	28.04581	25.81194
Dynll2	32.54877	31.3646	30.10683	29.35109	23.73259	28.60752	31.84627	23.9116	25.80106
Atp9b	30.05082	29.97082	29.91934	27.77998	24.53557	26.07051	33.44579	24.92783	25.797
Mphosph10	24.72306	23.47711	22.8375	22.78204	21.31606	18.99827	24.95404	25.33654	25.79482
Cyb561a3	25.95026	23.85805	26.49758	26.46908	25.58647	22.30509	25.59362	21.59818	25.79389
Znrd2	30.76378	21.69027	24.83534	33.57982	32.68844	33.05686	27.62728	30.04309	25.77802
Plekhn1	30.04911	33.75335	35.47735	37.37631	25.61108	27.31749	37.95302	26.20939	25.77552
Prkch	27.12494	22.07877	26.87792	28.09234	21.72396	22.35177	26.80693	29.01101	25.76307
Appl2	28.04913	26.56351	27.07141	34.32575	28.20477	28.60786	43.35854	24.94551	25.7625
Atpaf2	29.41828	26.21531	31.33358	28.30302	29.48428	23.5221	34.1922	24.16669	25.76076
Ap3s1-ps1	19.46006	17.56384	18.62162	22.73645	25.17841	23.63197	18.85317	21.08898	25.75773
Top2b	25.85404	27.65014	29.35124	23.57575	23.17027	22.78955	29.5899	26.14292	25.75741
Lmbrd1	28.15009	27.83838	31.03993	25.45781	24.28712	20.65947	27.59654	27.28938	25.73397
Mgl2	59.41013	49.32694	43.56964	26.20469	50.27664	52.17582	43.30458	30.01037	25.72668
Ccs	27.2593	26.3776	26.93792	29.31171	28.0734	31.31062	28.34155	23.63625	25.72279
Cse1l	36.47487	33.9806	32.63921	28.04489	23.69397	22.05421	29.63301	27.41657	25.70468
Il1rap	30.75964	25.29445	25.69495	13.28967	19.01378	15.6941	15.03098	19.7261	25.69982
Cert1	31.40616	31.95026	32.87854	23.9703	21.03952	20.15773	27.92803	22.9806	25.69387
Thoc7	23.02245	26.39003	25.05134	23.87028	26.54548	26.45692	22.71615	31.27201	25.68728
Gsk3b	29.35911	25.46698	27.34061	25.44037	22.13087	20.44538	34.0616	25.47906	25.67582
Itfg1	28.95007	30.70228	33.76248	21.70595	22.58301	22.05032	26.84723	23.23687	25.67063
Pinx1	29.30647	26.00114	25.58507	27.68519	22.15448	28.96494	26.8821	27.14693	25.66222
Atxn2	33.34111	32.23151	34.26329	31.52283	26.07675	23.97025	39.31362	26.82834	25.65117
Smim24	27.86365	26.44327	35.77376	24.73548	35.07664	30.13631	24.11499	32.31205	25.63762
Tceal8	25.31082	28.28245	27.57479	36.45845	25.69719	24.49745	28.26803	24.75348	25.62239
Wasl	28.56931	28.87486	30.40094	25.44998	27.2091	24.8867	37.43704	29.26513	25.60831
Brap	25.92641	24.84246	31.06521	24.3489	25.12444	24.64193	30.69594	25.78754	25.60547
Gspt1	34.12573	30.35587	28.3114	23.26134	24.08167	22.18857	29.34182	25.15735	25.58622
Senp2	27.90185	26.90962	27.33766	25.97776	23.63149	21.81516	29.32011	23.68429	25.58354
Ctps2	24.27567	26.09985	26.22422	20.98955	21.70974	22.14687	25.90866	24.93234	25.56542
Zfp512b	35.37214	32.95442	29.58878	47.2059	31.13519	24.53329	50.17783	33.96396	25.54723
Faf1	28.61677	24.29597	24.38583	20.16861	20.62544	21.3392	27.46914	24.56569	25.54195
Pop5	22.37661	26.22252	25.45636	18.85795	27.012	24.3145	24.97754	23.59499	25.53896
Ttc5	22.58588	24.47903	24.73678	27.10829	26.98595	27.16733	30.39569	22.97767	25.53055
B4galt3	27.41382	21.64021	27.195	18.95526	26.08219	25.78765	30.16892	22.27008	25.51956
Xylt2	27.5895	25.04542	27.67367	31.20244	28.75859	26.90639	37.55161	24.156	25.50824
Slbp	29.27351	26.64749	27.30665	26.41591	25.62242	28.23834	30.19201	28.80909	25.49456
Atp6v0a2	28.61621	25.37465	28.21498	24.5779	26.58205	24.16137	27.60381	22.90038	25.49442
Lrrc8c	33.99966	29.12082	34.53277	24.00746	23.19854	18.91946	22.38045	25.2811	25.49418
Mocs2	22.8655	24.92015	24.52215	27.13475	25.33785	27.92776	26.86541	25.0905	25.49353
Ubr4	35.86902	27.67833	33.33028	28.24621	23.49709	19.03996	35.64295	24.32124	25.48831
H2ac24	26.61518	26.422	23.42898	25.73431	30.83812	34.82099	28.39547	22.07055	25.48352
Fcnb	1.096233	0.780939	1.044153	7.111555	4.424742	17.81324	2.266023	2.932359	25.4734
Get4	25.88075	26.73576	26.92605	27.2615	28.42578	25.86125	26.65928	27.53508	25.4668
Ipo13	35.68128	32.67333	33.9179	26.7313	27.78128	23.05312	35.68627	27.77279	25.46124
Lrwd1	25.46742	26.8967	27.70953	22.13703	27.52603	29.59815	26.6111	28.82567	25.46089
Casc3	32.65246	34.38293	36.15302	30.58384	31.29538	24.88016	38.3569	30.52412	25.45853

Rps10-ps1	22.56088	22.02003	22.85278	22.05101	28.92445	26.48072	19.92974	32.86282	25.45384
Dctn4	30.06984	30.26733	31.8751	25.50285	24.31386	20.50695	30.82133	25.57544	25.4485
Stat5b	26.53003	24.5598	24.65077	24.26328	24.69803	22.87699	30.02933	24.99093	25.44659
Sirt6	19.70113	22.14111	23.86905	26.59044	24.51505	20.45303	27.50821	22.76269	25.44611
Herc4	24.32761	27.36058	29.13271	22.18791	27.11885	21.19321	27.34756	26.67896	25.4426
Pprc1	35.45186	28.58888	32.93259	27.93546	25.07544	22.49024	31.75174	25.4904	25.43899
Top2a	40.45859	44.75435	39.52629	26.01103	24.1327	26.07188	30.65243	17.92225	25.43339
Ppih	32.32439	31.02506	28.852	28.94627	26.06828	25.63369	24.42799	27.6322	25.42489
Tubg1	35.60711	34.25494	39.39388	33.30692	24.14392	30.0609	35.74522	22.87132	25.36325
Trpm2	19.34099	16.91259	19.45325	19.19619	19.58996	15.71589	21.37307	18.10034	25.35287
Pop7	25.33911	20.52449	30.17245	23.80507	25.86598	24.92165	21.41441	18.66047	25.35128
Polr2k	13.46674	14.99782	16.16196	19.41739	17.9558	20.44469	20.29788	20.83519	25.34912
Smim12	31.13645	28.02394	25.55221	29.02016	30.94556	31.3022	25.173	29.69659	25.333
Tsn	34.28188	36.38161	35.22748	27.60966	27.1817	24.39597	31.65414	30.3196	25.31902
Gm16418	25.12037	22.0298	21.51362	22.10843	26.12431	32.44264	24.91866	21.03052	25.31792
Usp32	26.72221	28.00095	26.9997	23.69883	25.39768	19.90819	30.60557	24.10127	25.31175
Galt	25.45061	24.64712	28.44159	25.34472	25.85481	28.03845	31.14602	26.86533	25.31133
Tinf2	30.09404	30.39545	26.87735	23.42682	27.87183	24.08594	26.85329	20.91266	25.30969
Slco2b1	32.23276	29.47874	27.40032	40.33201	40.04519	31.03827	58.15563	27.09273	25.30741
Rnf41	27.90249	28.39375	31.45859	24.56193	23.85785	24.50778	26.56324	27.1058	25.306
Aimp2	27.43273	30.21507	23.11489	26.70967	26.07706	29.94208	23.67242	30.43046	25.30242
2310061I04Rik	31.33664	34.69393	33.06544	33.73404	30.66609	30.08691	35.35936	30.97606	25.29984
Relt	24.33515	21.27503	24.13225	19.78045	26.46787	26.49925	21.70733	25.73513	25.29137
Mocs1	25.34077	26.55469	28.26904	24.05713	26.47741	23.63659	27.06681	29.69665	25.27233
Prpf6	28.41768	25.59107	29.38287	27.75324	24.02293	23.15843	30.34637	28.38677	25.26856
2610001J05Rik	23.08611	26.88819	27.32959	23.75498	22.60186	23.38257	21.15535	23.1266	25.26283
Pygb	30.99794	28.52401	28.32899	28.79903	25.8903	26.50195	35.60598	26.01991	25.26228
Pdha1	29.08799	28.5264	30.74209	28.01753	24.83661	24.99622	30.08396	27.01057	25.26201
Dcxr	23.9567	22.86665	26.59664	25.26145	30.44189	28.3398	33.73403	31.13676	25.25407
Sart1	22.62823	21.55796	23.05559	24.11491	23.65618	23.99728	27.10147	23.55564	25.25111
Ctdp1	31.64992	26.1673	29.70353	25.96609	23.76977	23.54203	30.63202	26.0942	25.2442
Mnt	32.6028	28.63386	33.57379	28.98222	27.13613	22.71595	38.04458	31.25974	25.23199
Atxn7	28.55966	25.86098	26.66596	27.62806	22.51947	19.01597	33.23819	23.10772	25.22719
Ilvbl	29.88511	30.27812	30.19322	28.77536	26.56047	26.47876	28.99881	26.069	25.22518
Gm5637	27.76593	20.44157	25.98668	21.54731	26.04195	26.7614	24.18926	27.01641	25.21175
Ankrd52	37.3945	30.09005	32.95658	28.40963	24.33605	19.43743	36.73482	24.29669	25.20859
Swap70	25.65127	26.07627	27.00086	27.66623	23.56637	19.75732	28.3721	21.54467	25.20743
A530064D06Rik	18.83473	10.82611	19.54774	18.40797	15.13806	14.01189	11.25258	13.87232	25.18706
Larp7	23.62848	21.63654	22.42875	19.80536	21.08459	23.10779	25.28568	23.29297	25.18558
Stx18	26.37811	26.50749	30.50449	25.91193	25.84654	30.64938	32.18617	31.21216	25.17883
Ift52	29.35853	25.50308	29.8958	25.20043	28.03337	27.64349	33.28591	25.6047	25.17105
Dnal4	22.46068	26.37161	26.67046	22.9698	26.18467	24.80008	20.06983	21.9927	25.16578
Dock11	29.46237	28.65368	29.95398	24.2093	21.99112	18.56427	26.99132	24.52393	25.16423
Ncoa2	30.64308	29.41079	32.52458	25.74717	25.05806	21.00488	39.71461	26.19199	25.16272
C1qtnf6	28.31073	27.50905	22.3963	28.96684	24.53531	30.30584	22.67458	21.84102	25.16067
Glod4	22.43451	23.38285	24.21714	23.99129	25.196	23.67281	22.57401	23.53558	25.15169
Icosl	26.8919	25.87465	28.97318	24.83547	32.22098	27.07022	31.71407	29.65653	25.14968
Msl3	27.56154	31.10089	33.39271	27.19181	27.88051	24.74161	30.1954	26.85983	25.1428
Zfp148	25.84776	24.18119	24.59862	23.38703	22.50159	19.81974	28.53769	24.62242	25.13562
Bcap29	25.38203	25.31445	24.36339	23.51615	21.63829	25.3045	20.63496	26.14073	25.13538
Znrf2	22.96113	25.56919	25.30054	21.37512	24.84164	19.36768	25.15843	22.52861	25.12307
Capn7	28.03048	30.37543	26.75501	23.9614	25.41577	24.40582	30.61428	25.61089	25.12198
Dtx4	28.93413	30.38188	37.03908	27.54674	28.48017	24.4256	40.75363	25.64713	25.11924
Trim65	28.66974	39.64558	36.2796	33.2065	29.73812	28.68336	34.51391	25.65579	25.11617

Map2k4	29.75481	25.21344	30.09889	20.49001	22.95656	20.48691	26.32092	23.58334	25.11289
Il21r	24.30258	21.60333	36.24676	25.32224	36.89864	30.35655	29.12224	30.11351	25.10951
Meaf6	28.93571	31.9714	30.94946	26.93253	27.91071	26.2941	31.38269	28.85912	25.10785
Prpf19	29.64138	25.63286	28.28996	21.79107	24.10509	23.33544	25.74515	25.81445	25.10481
Mark4	34.25983	24.36425	29.51054	21.69078	25.66947	26.97228	28.481	27.31246	25.09813
Ak3	29.40765	34.66734	36.95642	23.86175	27.27395	25.31407	27.88353	24.40743	25.09165
Nipsnap2	24.89164	24.40371	30.15357	23.75371	25.36749	27.00213	28.53976	24.6553	25.08391
Zfyve1	23.82391	28.64602	27.79779	28.15388	23.88166	20.69454	29.06162	23.01143	25.07154
B9d2	23.52927	23.02386	25.752	19.70023	26.00292	22.7855	19.50082	21.82098	25.07
Tsnax	29.53725	31.56447	31.11274	26.24565	25.00281	25.42758	29.94627	25.1962	25.0659
Mcm2	35.55535	30.17931	29.2272	26.16554	21.47342	23.25358	30.00029	20.24555	25.06289
Mtrex	28.58185	23.47631	25.82048	25.12309	20.51949	19.55257	26.07761	23.22492	25.05345
Epb41	26.78886	22.74484	26.92331	30.28423	26.70154	23.30612	34.08926	22.84879	25.05239
Cc2d1a	29.18676	27.03461	29.59277	28.37274	26.64692	23.42322	32.33306	27.93239	25.0518
Ubac1	25.58423	26.88008	27.02169	26.73453	22.39652	25.0476	28.20082	25.1209	25.05167
Zbtb22	25.26291	23.3081	25.01064	22.83764	22.58168	21.08118	26.5061	22.08887	25.05064
Angptl4	19.72232	22.42998	29.19359	32.64778	26.85709	25.62022	22.28088	16.55866	25.04432
Rabepk	17.81659	21.65738	16.49299	23.41563	19.49316	20.62232	39.89102	23.79294	25.04389
Pigb	28.62873	32.28835	32.16424	25.80777	23.74918	21.72431	27.93001	27.25736	25.04166
Opa1	30.61923	28.92475	32.41132	26.31895	23.54833	22.20388	30.03926	23.30424	25.03292
Cryl1	30.39264	31.05678	33.46332	31.27803	31.00514	29.3424	38.72695	27.32709	25.03219
Gcnt2	25.06825	17.69847	19.50594	15.85691	19.1156	19.85411	17.79743	18.58259	25.02685
Slc12a4	35.42204	31.14129	31.1102	19.87462	19.31554	18.34675	22.36205	22.89951	25.01494
Bap1	30.38736	29.64116	30.5489	25.73083	25.31257	26.03955	29.26589	25.85639	25.01327
Csnk1g1	24.41178	24.08576	26.03274	23.25766	19.75982	19.75885	29.75562	18.62111	25.01273
Nbeal2	24.58596	23.15128	26.13192	22.04976	22.30581	20.45337	25.20253	20.82998	24.99918
Tmem230	28.27956	26.11239	25.17246	26.0714	23.7301	22.03862	27.92016	25.03122	24.98833
Rgs3	21.97363	20.22052	23.3587	23.16788	20.07718	20.61348	17.50194	21.49686	24.97768
Usp45	29.87563	34.12212	31.60946	27.62434	23.76517	20.75484	31.68391	25.96201	24.96712
Fiz1	26.56818	25.8853	27.71129	25.70002	27.82939	27.50883	29.29471	25.60858	24.96666
Trappc10	29.69673	28.43546	29.7501	24.7286	21.58052	21.60084	32.9499	25.26858	24.96189
Sirpb1a	18.58435	16.06655	23.04187	20.00163	27.38499	24.63498	21.53326	32.08934	24.96052
Atg12	23.31424	25.82589	24.05117	23.16152	23.72971	23.49412	27.20046	23.87072	24.95571
Lman2l	30.8999	31.1851	34.14615	25.71966	28.79711	26.57636	32.85052	30.47211	24.95555
1810030O07Rik	29.00636	25.07744	28.51135	24.9003	21.77201	23.48315	29.12112	27.16221	24.95429
Ipo9	37.73722	36.62534	35.3597	28.23605	24.11644	22.86287	34.63607	26.68382	24.95425
Fam114a2	27.17552	27.19167	26.22465	26.82476	26.15633	26.23889	30.01324	28.7674	24.94923
Rngtt	29.43432	28.36374	27.37916	25.00524	22.9687	20.97014	30.38638	26.48922	24.94736
Slc25a12	25.8007	25.6969	26.87738	20.89596	18.99074	18.0982	20.75461	22.00107	24.92583
Banp	23.83517	28.08742	24.26362	27.33451	24.8623	27.71653	26.98102	25.90157	24.91437
Polr2d	28.094	26.00551	25.03945	21.05286	22.14135	27.84763	23.37234	24.11187	24.9067
Pias1	26.48947	25.74485	30.90797	25.59748	25.8583	21.10541	31.61423	25.53549	24.90416
Gnaq	31.15153	29.35691	31.90812	26.46089	25.25821	23.00594	34.28844	26.58393	24.8987
Tiprl	25.51428	23.80857	23.29703	22.71024	21.43224	19.57329	25.86739	25.45024	24.89705
Exoc1	27.27743	25.6972	29.55681	31.78615	25.47727	24.34572	31.94926	26.95722	24.89703
Nifk	32.40229	27.4117	28.16136	28.26232	22.94258	28.55707	27.98345	25.13716	24.88435
Prpf3	24.44185	24.02698	24.66393	23.47661	21.16955	21.48811	25.78297	21.10047	24.88309
Cdk5	29.08579	29.52909	29.61136	33.80951	27.46697	32.72024	33.96613	31.52327	24.88188
Cdk12	29.76678	27.08617	28.7062	23.92348	23.19609	18.54555	34.44265	23.55932	24.88133
1600012H06Rik	22.33906	27.29194	25.48587	20.40863	24.31182	21.31364	24.85049	22.06999	24.87831
Ipo4	30.90487	27.78863	27.306	24.57637	22.6012	19.77636	29.65794	25.96615	24.86805
Tomm70a	29.62664	28.11355	28.32395	27.69509	22.22069	22.29548	25.1911	24.64486	24.8663
Hmgb1	26.52483	29.20421	27.53776	25.31425	26.31608	26.39687	26.51162	27.19545	24.85486
Pycrl	23.9219	23.18497	27.89103	24.54308	21.85878	26.06287	24.15761	24.78446	24.85047

Ankhd1	29.79013	28.0382	28.28471	29.28838	23.74045	20.54183	34.26325	26.86256	24.84276
Des	18.37686	30.08956	30.95124	24.67699	17.72586	30.77716	12.95514	21.78461	24.84133
Ginm1	28.7694	29.75813	32.15568	26.16522	24.55118	21.76477	23.25776	29.32359	24.8407
Rbm47	27.60057	23.09677	25.66085	31.05046	22.98093	20.44123	39.68009	26.32356	24.83809
Idh3a	33.85319	30.19817	26.03862	21.21171	22.75348	24.37547	24.40647	25.53495	24.83621
Mir1938	25.50151	22.11619	18.29669	29.21759	26.3993	26.14637	45.26542	24.80776	24.83358
Klrk1	17.1245	17.54409	19.59217	28.35887	23.09939	27.91361	15.35752	18.6515	24.83104
Esyt2	27.85799	29.27531	27.63622	23.2012	20.22959	17.3543	27.82147	19.921	24.82311
Evi5	30.11214	33.41432	31.67476	23.55226	26.15185	20.58789	31.09413	25.88346	24.81559
Scaf4	30.04328	28.41369	30.47983	27.07619	23.97952	19.43285	34.1445	24.03282	24.81039
Ddx19a	33.8226	31.54241	31.75947	27.14629	26.75813	23.75223	32.91019	26.48348	24.80793
Eipr1	26.5772	22.97225	25.35956	23.68761	23.66187	25.26958	29.4222	25.56651	24.80506
Ndfip2	26.242	23.22593	27.32833	25.99708	22.55429	21.67254	26.20646	23.27398	24.77633
Mthfsd	26.07931	22.53152	26.23853	23.03451	24.29232	24.79127	24.31803	26.46558	24.75668
Tbce	21.64178	26.332	20.6313	28.36952	22.99087	21.60599	23.34492	22.53381	24.74541
Fam91a1	30.33741	29.1974	32.2024	25.08553	24.09587	19.87681	32.6703	24.03845	24.74498
Polr1c	23.02485	22.75171	23.45239	23.06485	24.67981	22.62836	24.76328	25.23851	24.73843
Norad	26.59719	28.35928	28.08873	24.48617	24.45036	21.55169	30.58923	24.46436	24.73697
Pdgfa	38.33807	42.90929	34.96473	28.95557	27.76129	31.23315	32.45419	27.63692	24.73672
Hbp1	25.05273	30.53386	34.65543	25.68182	24.69644	21.35013	33.82549	22.27974	24.73452
Agtpbp1	28.86928	24.35414	24.81802	24.43237	19.73662	17.82152	22.33453	20.62825	24.72934
Zrsr2	22.66971	24.31114	20.65238	28.15244	21.35823	22.54219	27.78521	23.53992	24.72604
Ciao3	26.84461	27.23826	28.27612	31.07082	27.50968	28.16741	30.30588	25.03143	24.72412
Ccnh	24.64783	23.02144	22.34214	22.21441	21.79759	23.84277	23.45296	26.48436	24.72209
Hopx	33.40569	29.15216	34.83827	20.76161	21.82063	18.34244	20.72723	30.96851	24.7168
Slc16a7	35.34348	35.52242	38.35938	28.31241	28.55036	24.96279	36.38777	31.9562	24.70964
E130309D02Rik	32.04596	27.14963	30.20719	24.92429	25.32578	23.7843	28.33583	25.17957	24.6975
Nipbl	28.64008	28.30802	28.71337	24.17264	22.87384	17.5204	32.61347	23.14991	24.69634
Kifc3	35.39405	34.22384	32.97519	25.67222	22.89322	21.23159	27.07424	23.3908	24.69594
Slc25a38	24.10351	25.28137	26.35623	26.36534	28.80967	30.38893	27.27883	26.27649	24.69574
Rpp25l	19.16214	23.98776	19.49479	27.3995	23.47304	23.09782	22.40017	22.20764	24.68684
Smc3	26.0448	25.89568	24.89345	22.83059	19.63486	20.86205	25.77288	21.6962	24.68142
Gpr107	31.53163	28.49023	29.34633	25.10647	25.0948	22.47355	29.13062	26.79456	24.66912
Itpripl1	28.96795	29.16763	35.28602	23.14804	26.92795	24.79955	32.80831	26.55639	24.66627
Sap30bp	29.04803	20.7204	28.45464	29.47866	26.24432	24.90632	27.16329	26.2363	24.6609
Psmd5	26.28656	26.27549	25.25576	25.69877	24.27554	23.07988	23.16952	21.17485	24.65933
Nfx1	25.58092	24.8731	25.35479	27.0626	24.61026	23.17625	29.97491	25.57696	24.65745
Chd1	28.39876	27.91267	27.71602	23.39044	21.07193	16.27408	29.19685	24.9841	24.65349
Pias2	27.89286	31.41928	30.16951	25.1039	23.33021	20.9719	28.9945	26.02204	24.64726
Selenom	24.29308	25.78151	25.35496	30.35111	31.97578	40.09225	31.99675	29.41585	24.63766
Slu7	25.38951	25.98788	27.63878	24.52292	22.7787	24.78462	25.51981	26.90418	24.63238
Shtn1	36.92437	46.01424	41.25589	26.12228	32.86773	31.8737	31.47055	30.91004	24.63033
Huwe1	39.58296	34.07386	36.66675	30.13503	25.48397	19.96608	39.19538	24.29089	24.62959
Htatip2	22.28847	26.54148	25.32841	25.79784	24.56091	31.65535	25.74952	24.45539	24.62802
Socs7	27.0155	23.80016	25.44256	19.61644	19.53816	18.68527	28.61992	23.36361	24.62169
Phrf1	28.41663	25.28448	29.30246	27.19024	24.59922	23.30135	31.56382	25.49998	24.6173
Id3	17.80068	12.7638	10.82128	25.27883	21.10836	20.92656	26.27411	26.97278	24.61679
Zbtb18	29.02555	30.16248	28.70226	28.93678	24.95703	23.68405	33.03766	23.62065	24.60745
Opa3	32.67412	26.51985	27.45239	18.85556	24.13317	21.95877	25.96157	23.16823	24.59511
Usp21	24.53483	27.55667	27.86182	29.09018	30.47096	26.60742	29.71105	26.01681	24.59297
Ccdc82	25.69819	28.71224	26.45773	25.33932	21.6958	19.66749	27.83365	24.05564	24.55446
Agpat3	27.79157	27.83013	31.98659	29.83202	32.51743	30.47122	33.92228	22.46977	24.55195
Uxt	23.5557	22.18175	22.4627	23.64165	26.50365	24.11391	24.23275	22.53165	24.53911
Anapc16	25.74338	27.01829	28.80001	26.40287	26.51334	29.23237	26.82363	26.02073	24.53573

Ifit3b	12.22164	5.65504	14.92624	23.86814	17.87934	11.35653	10.08105	15.3844	24.52865
Rnf38	30.35234	28.49763	29.73212	23.79671	24.79334	20.17585	31.25129	24.42289	24.52536
Rfk	30.35468	27.64524	29.48701	21.81113	22.97441	22.0396	25.53247	24.13703	24.52405
Cyb561d2	25.08798	22.52967	27.64252	22.30471	25.61466	26.83796	27.1932	23.38015	24.51898
Eef1akmt1	20.98917	22.92694	28.05545	19.34764	26.01854	28.28333	22.58864	24.36114	24.5135
Plcb3	28.71066	31.15648	34.87746	32.15	29.33397	30.07112	34.54389	25.14296	24.50945
Cep83	22.31633	23.70121	22.61601	16.43118	16.90404	20.07085	17.90323	23.26247	24.50763
Phf11a	15.77469	9.855538	21.48871	22.80828	19.47029	17.64805	15.17952	17.52223	24.49616
Sap30l	22.82058	20.16202	23.7175	27.7184	22.54863	24.84275	23.94783	22.4307	24.49195
Vwa1	28.22764	28.65974	30.77424	34.1386	30.03223	50.79704	12.35324	30.02306	24.47971
Gnl3	26.74488	23.96509	22.34904	18.0823	20.94513	21.48426	26.97095	23.52446	24.47739
Foxo3	26.1784	26.16577	29.18949	28.14486	26.68342	21.68452	37.61581	24.72209	24.46738
Cep170	28.82048	29.60346	31.13148	25.62863	22.20742	17.60178	31.05629	22.13432	24.46437
Ggps1	20.32965	22.07387	27.47473	21.05956	17.50974	20.00997	21.85217	24.91009	24.46425
Aaas	31.50445	30.3854	35.59928	27.62448	27.88208	26.42416	28.96452	27.14693	24.44021
Slc25a30	30.10074	30.07114	30.2056	22.9782	22.29703	18.97924	29.74887	26.10175	24.4385
Tmem39a	26.3958	23.92607	26.15826	20.09608	22.94595	19.54708	24.86584	26.11309	24.43689
Mapk1ip1l	34.13592	28.07348	29.9052	25.68981	23.59352	18.93849	30.94191	23.01852	24.42934
E2f1	30.25951	31.18424	29.97501	24.66931	23.86223	26.9558	30.27546	24.48166	24.40911
Dus3l	27.68111	26.27117	26.49605	23.10178	24.43711	27.63249	28.92334	23.61131	24.40688
Il6st	36.94176	27.92919	30.73245	38.70656	27.80352	24.74977	37.08816	28.52112	24.37382
Brd8	26.15924	25.04671	22.92032	28.66423	24.15026	22.82132	30.69343	20.22692	24.36733
Kdm5a	27.5262	24.52012	26.99906	24.62363	22.61923	18.2928	31.33952	22.9183	24.36646
Wiz	34.24288	29.81892	33.39837	34.08016	28.21034	23.80755	37.05473	29.09303	24.35539
Pafah1b3	17.01375	16.7778	18.67269	22.74802	28.21994	28.87473	19.88965	22.93307	24.35379
Exoc5	28.33204	26.34378	25.49452	19.62719	18.57836	18.46614	25.68185	23.90062	24.34199
Ankrd16	24.25671	24.60079	23.03016	30.47701	28.76022	29.21445	35.40365	21.69036	24.32942
Gpbp1l1	23.32571	27.661	27.15327	23.80792	24.08146	21.4832	28.551	24.35998	24.32509
Med12	31.44356	28.15906	32.14298	27.15587	25.22732	20.56279	33.52276	27.03797	24.32009
Lactb2	26.82392	29.88437	26.47483	22.0335	22.97536	22.85185	24.23563	25.03605	24.30688
Slc26a11	16.81931	20.18362	20.33041	24.42359	31.41183	24.05317	42.74539	25.62612	24.29355
Exoc3	26.00661	25.94966	26.97067	24.13361	26.34064	22.71715	26.9046	23.0718	24.29346
Slc12a9	25.00148	22.88342	27.88114	23.4978	28.39816	24.32688	31.00833	26.63803	24.29086
Slc36a1	28.9703	30.03661	33.42452	26.675	29.16427	23.25672	39.54926	28.49807	24.28617
Rel	27.44054	23.98266	20.89289	21.68177	21.28212	15.36991	35.89089	25.10084	24.26344
Ascc2	28.38593	25.30878	26.63343	23.36373	22.51785	24.08924	29.37362	23.25989	24.24023
Snhg12	17.47305	19.43378	16.74314	19.1036	20.64896	21.65751	26.28522	20.72975	24.23894
Gtpbp4	29.87937	22.8108	24.61573	20.41364	19.61266	19.26254	18.81792	25.11209	24.23677
Cd81	34.804	42.50592	39.44668	22.40691	31.58177	30.09173	24.61021	20.65401	24.22584
Kpna1	32.50198	31.2607	28.27367	21.98522	20.57071	17.94741	26.18442	24.25211	24.2248
Arfip2	27.47115	27.07808	28.08683	28.53524	29.10728	28.50585	28.72435	24.73918	24.20903
Washc4	26.75874	26.90272	29.68623	23.21837	21.89837	18.35967	26.87586	21.21366	24.20771
Tmem184c	24.10551	23.41958	25.54003	23.99345	22.84788	19.06779	23.02898	22.00552	24.18505
Arap3	20.75587	22.76855	23.36643	23.12657	23.8291	22.05081	22.0156	20.48412	24.16239
Gm39701	16.33562	16.216	15.72677	9.450433	15.97589	9.44167	23.84623	20.38621	24.15424
Fbxl3	23.60793	25.48078	25.97527	25.89695	20.83713	19.22119	27.75543	22.67865	24.14847
Rgs5	38.44898	31.50962	26.60442	110.3291	58.54684	63.73969	33.57786	43.8435	24.14612
Ccnc	23.27639	21.92684	23.81076	19.97429	20.57699	16.15274	20.52524	22.81215	24.13873
Coq10a	26.42834	26.56231	26.18554	27.70637	26.96514	28.60819	31.21111	26.99591	24.13416
Phf21a	25.4383	29.64728	30.15703	27.99697	27.18843	21.81826	33.70782	24.46374	24.13098
Ppp4r3a	24.5418	27.08451	25.39463	24.1443	20.67115	19.64847	28.48684	22.69397	24.12576
Chd7	21.12714	17.19231	17.17814	17.12107	18.44668	15.15066	24.45855	19.60961	24.12514
Sgms2	22.09698	21.68187	19.53062	13.80303	15.56903	12.38031	14.97941	22.65808	24.12459
Nrf1	28.00959	24.71843	26.4146	22.53641	23.18932	25.39186	29.91028	22.297	24.12022

Pex11b	31.5275	35.4731	31.13371	21.99832	29.96337	31.8507	31.14766	26.61195	24.11456
Zfp91	27.00246	26.48698	23.35806	22.27117	23.05291	21.15042	28.52131	25.16319	24.10606
Gpr141	13.95934	12.82699	16.59009	15.68454	16.57483	12.77069	13.12076	20.48251	24.1035
Eif2b5	31.14595	27.86155	27.5921	24.22074	23.03225	22.16372	24.72602	26.39123	24.10345
Srcap	35.62495	30.91581	32.50704	29.06531	25.33888	21.94285	38.81173	24.46466	24.10246
Pcbd2	22.03308	24.80983	23.0511	28.06859	25.14219	26.47876	18.57463	19.91426	24.09618
Nt5c2	26.08254	22.74882	21.68245	24.49632	19.32363	21.16052	27.52639	21.88239	24.08601
Exosc1	26.09828	24.66104	24.43881	24.75366	20.54159	22.86664	21.04649	22.8302	24.08181
Tubgcp3	27.44204	27.25059	25.96117	23.88466	24.10043	20.76677	26.27692	22.17165	24.06157
Pycr2	22.08508	25.89904	24.13344	19.27222	25.35918	24.68786	23.57854	20.23493	24.05605
Snx15	26.33768	22.68563	26.4789	21.1078	24.99602	20.99238	28.26828	22.57722	24.04892
Pcid2	27.32765	24.61436	27.28478	21.65724	22.91302	22.99368	25.60326	25.33909	24.04892
Ube4b	31.62312	29.6608	33.01635	26.68455	26.35556	23.09581	37.01584	23.51597	24.04386
Strip1	27.17855	26.49014	26.54678	28.13534	22.30582	21.62719	28.58693	24.34103	24.0393
Snx14	31.07976	31.71723	33.75004	21.73615	25.68509	20.47099	28.01746	25.54502	24.03625
Rai14	38.15774	38.37795	37.66019	20.32077	20.04276	17.46119	27.69177	23.87101	24.03253
Actr8	24.74967	26.33739	25.65703	24.64101	22.1645	25.03432	26.57705	22.76047	24.01961
Arl2bp	29.13868	27.54512	28.36762	22.13086	23.92736	22.96821	21.78688	23.83812	23.9996
Slc9a9	28.22706	23.8452	28.88132	31.39996	31.8727	24.13619	37.46029	27.11677	23.99774
Tmem161a	29.53855	26.4191	26.55828	25.10242	23.35503	22.79898	25.81968	25.72014	23.99712
Tsc2	34.24298	30.10969	31.65155	30.18546	27.16735	24.61064	38.09537	27.43468	23.9946
Czib	25.62861	21.59914	25.84065	26.857	27.9522	25.09241	23.76614	25.51551	23.98802
Tmx3	24.8275	25.80462	27.70422	20.85066	21.92785	17.87854	18.95022	23.69545	23.98366
Nae1	23.3474	24.97813	23.63839	18.75801	20.4362	19.1031	19.69515	22.65591	23.9615
Dgcr2	27.30369	24.73199	26.04026	21.59872	23.69517	21.06862	29.27533	21.32821	23.95918
Mrps7	27.5445	27.50282	24.91353	24.89732	25.68907	27.67324	29.58374	23.38502	23.95376
Mob2	21.66501	21.15164	21.70561	21.12717	23.35323	25.59848	22.15575	20.19219	23.95102
Rbm14	29.24148	26.75514	29.73492	24.60553	24.1327	26.24864	34.16538	25.34158	23.95029
Rfng	31.03751	29.64771	31.43157	28.39567	27.88659	27.72255	31.61766	26.20538	23.94693
Crip2	33.4695	32.52908	32.48491	35.51571	26.52766	31.03463	19.45532	26.30569	23.93526
Zfp131	23.36289	21.43582	22.68745	15.8558	19.5304	18.48894	23.31846	23.75015	23.93154
Tjap1	24.9721	24.83446	23.52407	22.23075	21.29159	23.35734	27.87471	24.86278	23.91397
Usp1	28.47043	29.39931	28.55632	24.38617	19.56552	20.81544	23.29812	23.85195	23.90825
Xylt1	26.81613	21.46783	23.83933	22.76928	16.44152	16.46568	26.29736	16.90071	23.8887
Ndrg3	32.34069	38.18246	37.19612	27.3356	29.11519	27.07856	32.81592	27.92041	23.88825
Rpl9-ps6	18.42686	19.39879	19.6891	17.46227	20.09947	20.31761	18.14979	22.19263	23.86689
Tfpt	23.22933	21.46399	25.47714	20.43018	25.23736	22.20383	21.23456	22.81355	23.86055
Acap1	13.27891	11.71755	11.69554	12.85132	14.52027	19.5569	12.59483	13.76142	23.85365
Tfip11	25.91483	22.57422	25.62102	23.34648	22.6726	23.05516	28.60864	24.8668	23.85052
Taok1	27.1333	28.38573	28.62983	25.99689	23.07582	17.87492	31.4798	22.51895	23.83973
Ube2q2	24.65233	23.97499	24.81349	21.8582	22.14074	19.57748	29.38517	25.09145	23.83529
Nmral1	16.12816	14.62293	20.01392	25.02214	20.37973	16.23406	18.59421	18.71344	23.83159
Wdyhv1	20.22016	21.76895	24.10631	23.63559	24.16748	24.9526	23.94337	24.86131	23.80235
Ccdc47	29.85518	27.76359	28.62089	21.88242	22.8471	21.41628	23.09443	23.35665	23.78491
Mir7050	28.643	31.33127	19.40558	21.13222	14.9596	19.6525	22.9192	16.71445	23.77683
Ap5z1	25.78118	25.88262	27.61372	34.1618	28.9093	25.24661	35.49079	27.39493	23.77387
LOC108167922	24.61494	19.22675	19.93388	23.93553	24.79149	22.25951	20.8254	24.16172	23.77164
Ampd2	33.70198	27.48396	29.84164	25.98119	23.82989	24.2249	30.64572	24.48705	23.76628
Ak6	21.44725	22.95392	20.16164	16.87236	19.51948	21.3873	18.17331	20.20662	23.75014
Tom1	29.74861	26.89657	29.36279	27.14906	25.1548	25.71082	31.7524	28.05889	23.74238
Manbal	23.14941	20.98434	25.61295	21.92118	21.569	20.85059	25.17168	18.59817	23.73854
Tmem260	26.21039	27.22829	29.52193	27.87103	23.54298	21.95253	29.64467	24.81641	23.73352
Ddx52	24.54324	24.52204	26.91438	20.57654	21.09836	22.04191	21.82128	21.93475	23.72937
Tradd	25.3374	23.31382	25.82073	24.00137	25.54156	26.87373	25.40143	24.1999	23.72914

Rufy3	22.08098	21.88791	23.54792	29.48837	24.29954	22.47423	36.04173	23.92357	23.7239
Rpl28-ps3	33.1497	26.06551	28.96693	27.67627	23.27449	30.23729	29.70141	33.28527	23.72291
1110038F14Rik	23.26567	21.41883	22.311	25.2047	27.07316	26.44196	23.53004	25.87609	23.72129
Tlk2	23.11277	20.8129	22.60862	20.93154	20.73677	17.90927	24.61775	22.38473	23.71904
Pole4	24.50143	26.14769	22.16827	22.70515	24.10667	22.04101	21.18499	26.45931	23.7155
Utp14a	23.28353	22.88038	23.788	18.39096	21.67545	19.10664	22.74479	24.90395	23.71271
Arih2	25.14068	22.8798	20.80062	16.76127	19.90321	19.08822	27.66721	23.39239	23.70263
Rps12-ps3	17.58339	18.09506	15.45726	15.81436	18.13285	21.43909	16.08974	17.27426	23.69677
Klhl18	26.38244	25.40608	28.70953	27.74619	29.80217	25.35094	33.67815	25.83397	23.69607
Arl3	30.04152	23.52402	25.70115	24.41448	23.84268	28.72982	25.93686	26.87943	23.68771
Ralgapb	31.41051	28.42692	31.45007	24.28562	21.93769	17.43954	32.94666	23.17279	23.6873
Sdc1	34.55237	41.40375	35.1498	17.83827	15.55892	17.39396	15.57655	20.84559	23.67598
Prkag2	34.16197	35.55691	36.7016	27.99262	30.17063	26.83017	34.25454	30.1863	23.66597
Hexim1	21.22327	22.97131	23.34506	21.28269	23.59714	22.00835	23.40104	24.1176	23.66109
Alox5	31.60962	33.64406	30.92053	15.03895	20.62686	22.71907	22.19373	20.22485	23.65689
Mir6385	11.61559	11.58467	6.336515	17.85082	11.56541	9.276955	27.83046	16.58878	23.65103
Cd109	38.33513	40.37232	45.0965	26.42212	17.15257	15.62401	32.39432	21.77153	23.64039
Mrps28	22.42093	22.28466	16.66407	19.85202	16.01509	21.44425	22.06211	17.3261	23.63778
Prmt7	26.09614	22.04497	21.587	25.56265	19.07303	21.40143	23.48279	22.25022	23.63453
Rpl17-ps5	17.53312	15.40472	17.61866	24.07678	18.20916	19.30248	16.13664	22.01919	23.6336
Pabpc4	29.6278	30.18039	27.86194	23.33244	23.69464	22.97302	25.92557	21.98531	23.6233
Ggct	13.27225	11.01124	14.44846	15.53382	18.23342	10.6445	11.77523	13.41927	23.62011
Tusc2	22.19525	22.70205	25.00177	23.33212	24.76226	23.39595	26.69997	22.21249	23.57165
Il15ra	14.71629	13.33673	17.87665	20.51412	15.1676	14.78986	18.75207	15.73877	23.56868
Dnajc11	27.64371	25.54179	28.13658	24.86731	22.41789	21.92447	28.0262	23.27162	23.56505
Gatd3a	25.119	23.9973	25.29821	22.17371	25.35005	26.73403	25.00525	25.46817	23.56262
Zfp335	27.17186	27.21115	25.894	23.30987	23.21338	23.03467	31.47663	24.8402	23.5623
Arhgap39	26.4474	28.96346	29.60799	35.14458	31.17802	25.28032	39.51944	26.3972	23.55891
Chst11	31.85728	30.41388	29.33233	16.69629	15.873	14.9164	19.31621	16.5068	23.55651
Hscb	27.88601	28.72773	29.14177	33.20935	33.07864	35.20772	35.89755	28.14005	23.55402
Fam104a	24.55867	24.42762	24.82492	21.71492	25.68863	24.47683	27.67362	28.37632	23.55301
Mier1	20.00367	23.59187	24.12453	21.18916	22.18659	20.20362	26.7003	25.5763	23.54921
Mrps11	25.07785	21.71175	23.80005	25.52102	22.10529	31.92493	22.33464	24.8418	23.54544
Def8	24.70165	27.64524	29.23112	25.60582	18.46003	21.09341	27.19541	26.07473	23.54286
Cnot9	30.83128	30.49507	28.1045	24.12508	25.1166	23.22685	29.40144	23.01219	23.53918
Cd2bp2	25.71095	23.63976	27.38441	24.91376	23.2178	24.1585	24.40111	21.83486	23.53894
Smim20	26.73114	28.39867	27.89661	26.35026	27.3955	24.50516	24.50481	27.05516	23.53867
Tmub1	22.00723	23.62411	25.74809	20.67282	22.27941	21.59444	22.39831	26.07134	23.53666
Mtln	21.39111	23.32352	22.63181	19.49298	24.63061	24.48876	17.48798	25.21247	23.53428
Sbno1	27.01217	24.78336	27.99998	22.02423	19.13829	18.18638	24.37861	21.91258	23.53141
Nedd9	27.62659	24.66866	28.55432	20.72207	23.22988	18.97797	28.53981	23.13476	23.51126
Fam168a	29.46059	29.1639	29.81966	22.66757	23.77674	19.51196	29.77988	23.90837	23.49874
Birc6	36.99249	32.54197	33.70819	29.43942	23.24082	17.84934	36.63907	23.7698	23.48329
Exoc6b	34.74085	33.88853	31.62089	28.73803	24.04114	21.72347	33.93785	25.55706	23.47791
Stra6l	27.15051	30.06649	31.11838	29.29032	26.77978	28.85922	31.65048	19.95435	23.47515
Impdh1	31.48598	32.25277	29.66518	20.66104	21.00662	19.87159	25.97999	18.94755	23.46071
Oip5os1	21.70188	23.44444	25.7163	20.98197	21.20349	19.92083	22.55435	20.62412	23.43656
Cnot4	28.20416	28.56285	30.51623	25.6674	26.25591	20.49767	33.92236	29.60851	23.40534
Klhdc2	19.79931	23.18449	20.8273	20.02835	19.5892	16.99253	18.66749	20.85803	23.40485
Ruvbl1	25.57658	20.57243	22.68939	23.12119	20.37286	24.23605	26.77798	26.78655	23.39535
Snora28	20.44847	25.83243	22.2687	18.52646	3.894979	9.665163	23.01313	14.27723	23.38705
Eaf1	28.89634	26.10577	26.11308	21.80126	20.04553	20.04485	28.06764	22.46804	23.37662
Slc9a8	27.71903	22.22201	26.0358	24.49275	22.10468	19.36106	31.15555	21.17793	23.36692
Pitrm1	31.19537	27.40412	29.41477	25.74828	23.97771	21.35499	32.53493	23.24016	23.35969

Kdsr	22.24129	25.25682	24.83165	27.64588	19.34725	17.15701	24.80827	20.02274	23.3492
Trip10	27.94078	25.90359	27.74998	25.16572	25.00014	23.8137	21.83054	24.25354	23.32771
H3f3a-ps1	14.38949	15.5044	19.53086	15.71422	22.51435	18.41586	16.00095	21.95702	23.32682
Slc35b4	27.71153	26.54601	26.88991	21.88997	24.29278	19.44195	29.35418	25.3847	23.32449
Orai3	26.35808	27.50673	29.94448	22.92377	28.88581	29.02412	30.51492	26.28546	23.32152
Tnfaip1	31.27391	29.63485	29.40239	23.92439	22.24227	23.55304	28.77302	24.87745	23.3165
Rbl1	31.38619	31.97605	33.74174	25.53787	23.26154	20.58447	28.75729	21.52532	23.30083
Thoc2	24.22556	27.27193	22.98056	23.24206	21.0422	19.78775	26.25263	23.1938	23.27739
Adamts10	21.21047	25.59351	23.11075	28.69241	26.78778	23.63285	36.25574	24.9729	23.27482
Dhrs4	32.5002	33.34981	34.61615	31.03475	34.08164	31.03169	29.71008	29.65872	23.25964
Gm11942	25.46834	30.48064	28.55155	28.85949	24.59422	25.96233	25.83307	27.9702	23.25749
Mylip	21.50246	23.79918	25.35243	30.06788	27.68254	27.30938	34.13294	26.11697	23.25696
Myo18a	30.46336	27.43134	32.90951	28.82161	25.97828	24.13202	31.94683	23.99506	23.24412
Gvin3	20.78208	15.02881	22.13306	21.46637	23.79741	18.07212	17.95227	20.25508	23.23627
Zfp511	28.78106	31.82909	24.58889	31.68249	27.1579	27.00868	30.2955	30.89089	23.23024
Naa15	25.98944	23.94105	22.11407	20.01428	18.47518	18.80646	21.19744	23.71311	23.21722
Lbr	22.4787	23.52653	21.10458	16.83069	18.50335	21.10584	18.17239	19.01897	23.21327
Gm9104	16.23543	19.25293	17.72244	18.40866	24.51364	25.63369	13.09669	22.99808	23.21071
Pias3	24.19162	26.91605	26.34961	24.61734	26.63141	25.38459	25.40097	21.73827	23.20347
Rptor	28.66623	24.70264	26.47007	23.57968	21.76439	18.85778	29.36917	19.47727	23.20197
Urm1	21.18702	26.54871	25.6654	21.47993	24.68698	23.68395	23.71597	24.35924	23.20112
Hcst	24.08169	19.63938	17.18529	23.82208	23.41296	22.50197	23.33403	22.6893	23.19108
Zcchc24	26.78237	28.69994	31.40284	25.50264	24.31015	22.83394	30.80377	22.33503	23.18764
Dnajb12	28.71426	29.86935	31.42716	24.06331	24.55627	22.54879	29.69667	26.21576	23.18662
Mrpl9	25.49637	26.56434	26.16069	22.79627	23.93257	25.92247	27.52886	26.30379	23.17983
Ing1	25.50685	26.80122	26.7571	21.20062	26.78108	22.87399	27.16054	21.34481	23.17827
Ints12	21.89857	22.57485	23.5181	18.75693	22.10492	21.38079	20.51662	21.92713	23.16559
Rtca	25.32263	21.7925	23.2497	24.31766	21.8886	23.76657	24.45599	23.78625	23.16357
Pde8a	31.23472	31.6352	29.26991	18.47985	18.9624	16.7506	21.40383	21.34655	23.16309
Ftsj3	28.74762	25.18145	24.44271	22.58952	19.55876	21.30044	27.22246	25.37844	23.14255
Odr4	22.74433	24.9462	26.7073	22.33991	21.96467	20.00274	24.20666	23.58728	23.1377
Eif2b4	27.49523	25.06246	30.91974	21.74581	25.91212	25.1902	26.93072	23.19506	23.12779
Cdk18	42.0828	47.81034	42.53814	34.70055	23.6857	25.9506	30.50092	25.99038	23.12453
Ankrd44	23.93588	24.74564	26.1149	23.02068	24.36131	20.15626	28.42504	22.86667	23.12282
Wbp1	25.43951	30.28245	30.91396	25.79569	25.9651	30.44001	28.22492	24.61156	23.11636
Zmym3	26.43913	27.93701	29.62404	28.18195	28.21034	24.82207	37.87111	20.75089	23.10765
Gfm1	31.02654	28.64549	27.70091	25.37818	21.33893	21.11474	24.17191	22.93742	23.10365
Rnasel	24.61591	26.45861	29.84484	23.32687	28.16028	21.48532	29.5796	22.68666	23.09338
Rnf6	24.50408	24.68481	26.19828	21.68665	18.5894	20.85232	21.2842	23.17934	23.08593
Cab39l	25.17566	23.63815	23.63027	23.09198	19.85213	20.2138	22.64871	19.6355	23.08123
Rdh12	8.676968	10.43852	7.023421	5.942492	8.360315	11.61478	4.152535	5.52898	23.07212
Manba	27.48521	27.80321	29.29884	22.89105	24.38074	21.13472	32.61699	24.1004	23.06155
Pcnx3	28.59571	27.08883	30.15333	27.38941	26.62701	22.61926	36.16133	23.80371	23.0461
Fam174a	16.20781	19.8122	20.70946	19.62299	23.17926	22.21506	18.68236	25.12616	23.04583
Dennd4c	29.69659	29.298	28.13107	26.45418	18.71836	15.07984	34.35825	22.22586	23.03871
Cdk14	27.70729	26.39183	25.90151	22.36636	18.53544	16.74879	24.13314	21.77277	23.038
Ric1	23.58718	21.48798	26.11363	24.36491	18.54739	14.90103	27.39474	20.71501	23.03145
Med16	27.42824	25.95191	26.43699	24.60684	24.282	24.37586	32.73064	23.09568	23.03031
Hjurp	25.08603	27.73484	28.48944	24.94551	22.03562	23.22434	30.49894	17.71259	23.02662
Htt	33.72482	25.88531	30.8732	26.01236	22.46826	17.34827	35.92084	22.23786	23.02613
Ints14	27.81475	26.11787	26.51611	23.6424	23.77396	23.68638	24.19733	22.21748	23.02514
Secisbp2l	27.37081	28.56341	33.90979	27.76773	24.53213	18.14782	30.33018	22.3265	23.0135
Timm10	17.23075	20.09053	23.31	21.52126	17.83737	17.78193	19.95967	19.81324	23.00728
Osgep	25.7194	24.51624	26.61888	19.50719	28.17239	22.19036	28.04324	24.42174	23.00136

Arfip1	18.70315	21.10221	21.161	18.63654	17.92229	15.29152	20.75814	20.38243	22.99981
Ncbp1	27.96584	25.29728	27.58934	19.89877	20.79397	21.20925	24.33203	22.99291	22.99874
Usp47	24.85707	23.95255	26.82153	23.38442	19.27054	18.47106	27.25275	23.10424	22.99476
H2-Q10	12.23175	10.99804	10.76519	5.688652	16.81096	14.75721	5.718131	13.72416	22.99306
Marchf6	30.81275	27.8137	28.55801	23.0641	20.37945	18.2675	26.50814	21.42278	22.98715
Gm10288	37.99055	28.81466	19.51346	44.8669	24.74655	28.36622	43.14317	23.31002	22.97667
Aebp2	27.11688	29.26672	27.94307	21.09696	23.28338	20.65916	28.10963	24.15534	22.97528
Slc25a19	21.3693	21.31241	23.98524	21.41959	25.79456	25.36268	29.12859	23.71723	22.97379
Mov10	23.23886	20.15531	27.25484	25.60866	25.8928	23.04052	25.1345	21.71139	22.96276
Sppl3	26.19121	25.78978	30.80943	23.97748	27.53763	21.97174	31.23783	22.16587	22.96158
Commd8	18.56834	20.98908	20.30592	20.3668	20.73525	19.73616	21.10737	21.78795	22.95557
Btaf1	27.69318	24.17476	23.63192	25.92361	19.90412	17.10825	28.84673	21.70963	22.9445
Fkbp1b	26.09211	24.73682	24.37593	23.87028	18.35965	22.53948	27.79239	25.54368	22.93717
Miip	24.24939	27.75932	32.18374	32.75803	30.06688	34.13124	29.91003	23.48711	22.93012
Ppp4r2	21.24035	20.74342	19.91799	18.81997	18.09741	17.81193	22.11033	20.07792	22.92862
Wdr46	26.51497	24.83472	24.03278	20.50724	23.34948	21.96158	27.13977	25.29363	22.92713
Rpain	17.6141	22.55999	26.45513	21.87893	27.19393	28.46283	28.93645	25.30263	22.91388
Dhx8	25.86425	22.60777	25.50781	24.55785	22.38348	21.48506	25.71047	21.32563	22.90151
Gas8	22.07534	21.86713	22.1778	23.54094	22.07077	18.98279	22.24814	21.73116	22.89621
Vps13c	37.40027	29.38618	30.19237	27.18213	22.37868	16.81245	33.55606	22.57812	22.8907
Flad1	22.2262	19.31989	20.85223	21.7722	23.25566	21.66194	22.44501	20.70044	22.88406
Pip4k2c	26.89052	28.65117	29.50556	26.81154	21.562	22.46904	28.79793	23.04732	22.88247
Rbmxl1	24.49973	22.4916	25.82347	21.70297	20.66032	22.25963	19.89561	21.7543	22.87837
Atrnl1	30.06096	27.26213	34.05487	20.82376	23.76763	20.2136	27.1506	28.07829	22.86393
Psme4	26.31454	25.90081	27.60215	20.19647	19.14434	16.18462	26.29082	20.52044	22.86071
Mfap3	26.48652	27.31911	32.13719	27.46371	24.24328	21.12105	27.84309	22.79896	22.84666
Adk	25.69688	24.13916	23.98862	22.61404	19.19949	18.29745	22.47548	20.17108	22.83097
Tspan32	23.80194	35.38391	30.59179	18.50816	21.95586	21.87285	23.15644	19.43117	22.82804
Ncbp2	18.37049	23.3702	21.17615	21.54222	18.8696	18.87606	19.04308	20.72949	22.82617
Nmd3	27.33646	22.23412	21.11343	17.39595	17.09164	20.42333	19.30303	23.44416	22.8056
Erlin2	30.59036	29.31902	32.28032	28.56824	26.36143	22.15457	34.11574	23.89978	22.80271
Ddx56	29.16089	20.45513	24.01418	24.87176	23.73695	25.96024	23.63087	27.96014	22.80085
Exoc6	22.2603	21.11017	21.0677	16.9995	21.56699	22.40255	21.4161	23.56601	22.79904
Cul4a	28.81156	29.40419	28.44028	25.90311	23.51559	18.66237	31.40744	24.02851	22.7891
Mesd	21.60332	24.08493	26.08936	21.09706	19.02858	20.81405	21.96582	21.02516	22.78778
Gatd1	22.5084	23.18259	24.83573	23.26836	26.01189	21.38828	23.10339	22.42609	22.78174
Rhot2	27.61253	26.80516	25.98901	24.28076	24.54591	22.95661	28.69904	23.69035	22.77379
Papss1	25.06485	25.74529	24.10445	22.11486	21.0379	23.43652	26.52522	21.62247	22.767
Sde2	18.06213	15.32668	14.72609	15.74825	16.12601	14.16324	24.83093	21.6327	22.76531
Smchd1	26.13936	23.75693	25.82723	19.01863	19.32332	16.02059	22.5317	19.16355	22.76452
St6gal1	32.33302	33.66177	36.69142	29.30265	31.59623	25.70513	41.89563	28.13639	22.75743
Hectd3	26.78291	26.68735	26.89545	29.17165	26.16773	25.35262	27.65634	23.08076	22.75716
Kdm7a	23.5877	21.70614	22.61552	18.85282	16.98271	14.23958	22.74786	21.88957	22.75295
Stfa1	10.21227	11.6401	8.223826	2.373913	8.589247	13.73672	1.919072	10.48073	22.75295
Dus1l	23.99146	21.54095	24.48518	27.1214	24.0764	21.67586	25.55738	22.60854	22.74903
Sec63	27.91071	24.9735	25.50129	20.98547	21.83542	20.43259	24.81722	21.96228	22.73372
Ints1	30.20332	26.05719	29.18966	26.8011	23.46164	20.59176	34.78377	22.07339	22.732
Gm14326	19.16609	24.03392	28.22175	19.80369	22.83183	20.49541	23.74512	23.53813	22.7296
Tars2	27.63782	26.83532	28.72394	20.53823	26.86335	22.45451	33.51709	22.42224	22.70357
Etfdh	24.45011	25.03948	22.75145	21.70299	20.90186	23.24002	24.86849	20.87233	22.70086
Zfp444	19.73157	19.53354	22.36019	23.06652	23.02992	25.70115	20.88361	22.81453	22.69922
Tmc6	24.42748	24.83741	26.7837	22.64713	23.50458	22.79702	25.5076	24.45757	22.69418
Ube2h	23.35362	22.59698	24.53486	22.74343	27.19001	20.37074	27.84628	23.53537	22.692
Klhl6	24.17562	25.41457	29.83347	24.24012	26.20783	25.51749	32.25935	30.41093	22.68224

Ulk1	23.05446	27.93838	30.36795	22.46783	28.82137	22.02914	37.49863	23.13035	22.6794
Ascc1	21.96602	23.352	26.51519	20.72283	24.10038	27.19627	24.65727	23.02051	22.66103
Zfp236	25.15905	22.398	23.14992	23.53456	19.77391	16.40974	26.72739	19.96157	22.65383
Jup	31.91168	33.94286	37.53454	31.69917	29.22838	30.35858	29.98233	25.64782	22.64922
Snora31	10.39464	16.78461	12.87105	18.70398	21.21372	12.81685	30.18377	20.73597	22.6446
Wdtc1	26.37358	28.67798	29.26512	22.69545	23.80298	20.49393	27.52052	24.31884	22.63884
Acot8	15.50281	16.08	20.71417	23.86393	24.74565	24.25739	18.77888	23.7344	22.63616
Gm4366	26.93931	25.00125	27.44932	21.36095	20.38427	25.0929	25.90901	25.94581	22.62867
Mrpl1	17.51119	23.92982	21.30214	22.18496	23.25223	22.22754	22.10128	22.96458	22.62741
Arglu1	22.69981	22.12265	19.52864	22.63706	18.22662	24.19629	21.18554	23.95559	22.62255
Mpv17	24.12643	28.79575	23.86553	19.27169	22.60107	25.05457	23.09634	18.76571	22.61767
Ccdc71l	23.53616	21.17139	24.84661	19.34439	21.33546	18.66848	21.82978	22.61198	22.61008
Eif2ak2	21.11926	16.34686	22.41619	23.10361	19.02751	16.46103	20.58278	19.22912	22.60796
Ncdn	31.19548	27.0156	29.43122	23.60456	23.2487	24.10019	27.64851	24.62006	22.59188
Btbd9	26.29729	21.82673	24.61367	22.07441	23.02818	21.32665	28.41592	22.27431	22.57785
Rsl24d1	23.42094	22.48673	22.0758	19.23306	20.25912	19.06527	21.6844	21.22396	22.56525
Kcnk6	29.0816	28.00356	27.39005	23.40459	23.38427	20.72511	31.80887	22.56389	22.54861
Gch1	17.95993	15.56103	15.56442	16.90577	15.90617	12.74757	15.34141	15.92033	22.54396
Abca9	31.14027	39.33862	42.05876	27.61183	34.74212	21.47647	36.03746	28.37019	22.54368
Numb	26.27096	20.69761	23.8917	25.61257	18.39383	19.50855	29.3132	21.1293	22.5396
Ints3	28.2191	28.19448	28.46295	25.54322	24.24709	21.29604	27.7527	20.97317	22.53945
Mib1	27.54625	27.66985	29.07998	21.5503	21.12179	17.73587	31.31492	21.72635	22.52529
Spopl	19.75317	24.69283	24.76162	20.33831	21.68331	19.36215	23.80927	24.44392	22.52002
Tmem97	28.11413	25.42615	25.49699	23.07664	21.70565	21.07616	25.1028	21.69902	22.51436
Camta1	28.81115	27.28217	28.19036	24.0989	30.01497	26.83602	33.8628	24.06405	22.50298
Abhd10	20.34478	21.27898	22.32939	25.79574	20.48652	22.74956	28.85988	21.25729	22.50173
Tmem175	26.74588	26.95645	31.26995	25.44107	27.23643	23.51449	31.76373	22.9541	22.49928
Gm21188	19.14081	20.45047	25.96506	19.19803	19.21381	17.73905	17.51841	23.9312	22.49825
Slc25a37	24.1093	27.4194	27.28264	23.47092	23.82407	19.83193	32.42114	22.30763	22.49588
Msi2	19.97192	22.82504	25.44761	27.63444	21.11944	22.80741	26.1514	21.11299	22.48976
Dleu2	15.80672	18.57159	17.6087	17.99618	20.35271	21.6691	17.82529	18.94436	22.48864
Atf2	26.12457	25.82567	25.97428	22.18212	19.60966	17.09858	25.82847	22.04135	22.48804
Dennd4a	28.3344	29.0407	25.75904	16.40904	20.3127	15.36813	26.01612	22.72206	22.48358
Cog8	24.24098	22.91506	25.37693	24.3672	20.55231	20.17557	23.66538	21.42406	22.47598
Ccdc84	16.61046	22.94189	17.45087	21.62375	21.00965	23.40468	26.53688	21.65179	22.47337
Sppl2b	23.33733	23.5602	25.05482	24.67478	24.3808	23.69575	24.9079	21.53924	22.46951
Hes6	18.82295	22.66149	25.81733	28.20996	28.89624	28.88712	22.83583	27.22329	22.46764
Bptf	29.00927	26.95695	29.08466	25.65417	20.14669	19.61236	32.76121	21.60893	22.46076
Ttyh2	32.37095	33.48465	34.65282	35.39583	26.55208	21.7471	36.78943	21.45815	22.45895
Rbm43	21.76264	19.35746	25.01977	21.69852	20.64348	19.85671	15.22541	18.61772	22.45866
Oma1	23.81783	26.54687	26.24747	24.20414	24.46158	23.19979	27.6651	19.86896	22.45145
Trim30c	20.35908	12.44759	27.8674	22.18295	20.49549	15.52976	18.77077	21.67358	22.447
Ctbs	22.33037	21.7183	25.87964	22.12264	17.81062	19.39501	20.58834	22.44772	22.44467
Map2k5	30.29799	32.77037	30.83647	28.76054	25.68974	23.88474	29.49801	24.08132	22.43131
Men1	24.82552	23.60928	28.6659	21.5381	22.02604	20.68922	26.2652	21.87957	22.42192
Pros1	29.48195	32.26524	35.69381	23.59145	27.43527	26.03259	27.00139	25.78413	22.41478
Mon2	28.00153	24.268	26.67123	23.02037	22.84424	19.80611	31.53376	21.81159	22.40151
Mrps33	17.88896	23.02298	23.79567	23.60092	17.31839	24.29604	20.1977	24.11467	22.3954
Ttc4	26.04769	25.50658	25.10457	24.71018	24.02667	24.32139	26.07418	25.8508	22.39128
Tmem185b	21.84221	20.96349	21.06108	19.44073	18.47018	17.15555	19.78097	21.56061	22.38588
Exosc7	26.63691	28.8075	26.22375	27.14989	24.10026	26.32649	27.35851	27.97629	22.37259
Cdca3	33.64286	42.99131	42.62669	27.23033	25.86706	33.42027	32.11461	20.32551	22.37067
Nudt2	18.6319	19.49511	17.65332	14.49698	19.36364	17.77431	19.25699	18.48631	22.36866
Pdgfb	43.57509	51.83643	45.9065	22.46222	31.11302	28.20304	25.48359	23.74596	22.3666

Zfp740	17.89296	22.8085	23.0147	21.41747	24.45218	24.13876	26.9946	20.83836	22.35545
Eif2d	20.53677	22.77654	21.38093	21.56558	21.29579	20.93764	22.35666	20.56807	22.35428
Aco1	29.04051	27.25384	27.81036	28.62299	27.35276	23.43398	34.51787	23.57749	22.31964
Cebpg	24.67713	27.73931	25.88275	22.6044	21.77297	21.32688	27.16233	25.26868	22.31711
Sprtn	22.84947	24.99618	27.07517	21.87493	21.24268	21.4139	28.26185	21.43072	22.29686
Fkbp5	28.50407	26.63452	24.23913	15.59865	16.92437	19.25827	36.43823	28.47046	22.29673
Cpsf4	21.35925	19.35337	22.45897	21.2737	22.62797	25.14226	22.71368	21.57651	22.28516
Gnpat	29.79982	26.60761	28.76277	24.64481	27.2663	23.33147	25.86475	21.72147	22.27528
Ssbp1	20.13085	24.13432	22.53248	21.67762	20.36447	23.4875	18.81186	22.19545	22.26825
Phkg2	22.58724	22.27028	23.25211	20.74141	21.40673	21.9766	26.72893	17.44238	22.26393
Gucd1	21.38658	23.28632	24.61394	22.88932	22.55133	21.75361	25.83831	20.08471	22.25317
Bms1	27.15334	24.05369	24.2035	22.47359	20.07871	18.13115	24.14274	21.92482	22.25009
Caml	22.26316	23.47952	25.06395	23.01286	23.90593	22.32552	24.6212	24.58362	22.24733
Pde2a	19.60195	19.86509	20.56657	19.01373	18.47231	17.97473	20.62899	14.04304	22.24595
2310033P09Rik	22.47851	20.99526	21.36598	21.90909	22.34277	24.70568	26.6899	21.78392	22.23166
Fkrp	28.08493	23.94671	25.63542	21.87007	24.6946	19.0035	27.35096	21.29753	22.22451
Arfgef1	26.19177	23.77443	25.45471	21.33236	18.44427	16.29316	23.57176	20.22254	22.22442
Tgfbrap1	29.11798	26.37134	28.07065	24.67707	24.71939	22.1003	32.02653	22.5639	22.20794
Gtf3c6	23.07495	20.75904	22.2942	22.32253	19.50372	19.49692	21.53591	20.92547	22.20579
Gm36459	18.22411	18.63381	14.70352	20.86318	21.58617	17.70283	25.64165	25.808	22.18587
Tmcc1	19.59845	20.80457	23.05465	21.56193	22.64688	18.77662	23.36123	23.0268	22.18457
Trappc9	27.27874	24.74702	24.22884	24.4795	22.73713	23.43497	29.4479	23.66175	22.17567
Yipf4	20.68856	27.81373	25.10989	19.30626	20.22338	18.72353	23.31677	23.70633	22.1704
Asb6	23.60182	23.38206	23.52589	20.80973	23.00261	23.59658	24.39366	22.27151	22.16971
Stx3	23.46916	25.3935	25.61604	18.77204	17.59953	17.84358	21.55048	20.4663	22.16928
Zfc3h1	26.15909	31.10255	32.48264	30.4673	22.94543	18.08784	35.38751	22.50681	22.16172
Stambp	26.083	27.94049	26.27303	21.25772	26.08767	21.46948	28.2464	22.10396	22.15666
Wdr36	32.14433	30.49803	30.27511	24.64285	24.84784	20.3314	27.65471	23.10607	22.1521
Peli2	20.6205	26.84954	31.05711	17.73845	20.45702	15.66398	21.56604	19.7496	22.14881
Gart	26.43559	23.10696	23.43806	20.61895	20.06537	19.77632	25.32413	20.99555	22.14532
Cetn2	18.93121	24.10217	24.40306	19.15054	23.19399	24.38011	19.27648	24.92258	22.14181
Mepce	23.669	23.13387	25.1622	20.9387	24.77961	20.83447	27.36242	21.08294	22.14091
Mrps22	20.92605	18.50594	20.7005	20.76349	18.88766	20.44793	17.48155	21.5731	22.11697
Abcb1b	32.29082	33.46456	34.62879	21.8435	23.71884	21.5582	27.15908	23.50245	22.08755
Tmem115	29.5398	27.65793	31.35149	24.46173	28.61844	26.25776	33.53131	28.48847	22.08637
Vcpkmt	20.49731	19.20666	19.2125	21.23571	20.52018	20.85079	19.85477	24.73469	22.07994
Rcor3	24.09137	25.69708	22.79179	22.70773	22.72289	18.10702	27.25501	23.55728	22.07468
Phf8	28.26866	24.0155	25.01837	21.10858	20.64125	18.88871	26.02361	23.22315	22.07221
Rrp9	24.23837	22.08741	20.70602	22.12992	21.19502	22.11469	20.91359	23.93584	22.07001
Lmbrd2	24.35198	24.41421	28.12271	22.93059	19.95115	17.16767	27.65202	20.96743	22.06473
Tet3	25.71387	21.52917	21.66031	25.87815	21.69647	15.33274	34.47757	20.6397	22.02729
Pum1	27.57609	26.97994	28.7468	21.48718	21.26012	19.57502	30.82815	23.3631	22.02504
Xpa	19.33076	24.83729	20.66832	23.14779	23.60733	24.37214	23.21919	26.03198	22.02019
Agps	27.05421	26.87486	26.75757	20.88013	19.63935	17.49189	23.97413	21.22129	22.01986
Prpsap2	19.83893	19.75357	21.14658	25.47093	21.97093	23.98915	27.82275	24.35636	22.01817
Tacc3	25.60338	29.91653	27.2236	21.49357	20.99624	20.18793	26.76878	16.21707	22.01344
Marchf8	26.32693	26.87677	27.08116	24.14485	20.81851	19.42436	28.16048	20.5409	22.00633
Tmem156	20.02345	21.75851	22.86525	18.94544	23.69533	16.78972	15.28976	21.34062	22.00371
Aqr	31.36131	27.44482	31.61618	25.44059	21.01657	17.75891	30.14181	22.66035	22.00162
Cd5l	14.8869	8.631502	13.0922	34.0995	21.6171	28.71678	29.25377	18.7861	21.99613
Usp10	32.11285	26.81209	27.7331	25.95633	21.40875	22.01187	31.28404	24.68691	21.98284
Gatc	29.29451	28.65646	32.76124	25.81097	26.71128	24.09019	29.56796	29.10907	21.97346
Spns1	23.08886	22.59447	27.16409	24.74778	27.71828	23.18693	30.2513	23.08495	21.96633
Arfgap3	23.17163	21.69332	22.47464	22.97859	20.29162	20.44814	23.92712	22.76812	21.96339

Mllt1	27.98785	25.23231	26.547	24.5164	22.25178	22.88494	26.2596	22.91774	21.96068
Rrnad1	26.0885	26.22888	26.67018	32.32335	25.49762	23.29893	31.09531	21.33333	21.9571
Usp16	26.25892	26.51261	24.80168	26.20401	20.1085	19.35387	25.48707	22.26485	21.95668
Rpl21-ps8	17.63478	22.03532	19.66405	20.96257	15.54036	22.40019	18.43592	22.86919	21.92704
Cars	26.98664	32.65926	26.46836	25.05797	20.49995	23.21152	25.58267	23.16472	21.91108
Rpe	22.04768	18.30901	19.88622	18.75332	17.15846	16.57659	18.57359	20.88312	21.90342
Ticam2	27.49904	28.76055	31.84759	21.89423	25.10727	22.37862	30.07008	25.37887	21.89327
Cdca4	25.18472	25.56526	25.9005	20.23968	22.82911	20.33606	22.67364	22.797	21.88978
Brwd1	26.09278	27.57261	25.99872	27.49135	21.33674	17.84482	36.70183	19.82809	21.86136
Crebzf	20.79229	23.52428	23.44524	23.76641	23.12951	21.3447	22.53572	23.60416	21.86058
Gm6055	20.81953	17.70583	20.17631	24.47437	21.28852	18.507	21.51803	18.59295	21.84253
Alpk1	20.8228	19.95075	22.56183	19.4287	18.20456	15.62696	26.52604	21.00815	21.83731
Bicra	25.07124	22.23997	25.36379	25.7536	23.97608	20.59304	35.59745	24.10662	21.83486
Rasa1	24.46185	30.07833	25.61209	25.93774	23.08369	22.10936	29.72573	22.03455	21.83125
Nxt1	21.9801	19.17423	19.9164	18.88945	20.4417	24.20075	21.59055	24.69739	21.82414
Hpgd	16.38309	23.44779	23.70664	17.5741	35.16835	31.13621	27.03239	28.7709	21.82347
Ep400	30.48316	24.49539	28.94629	23.54136	20.23041	16.25306	32.07938	20.87105	21.82199
Akip1	23.65828	20.66056	19.77638	17.26393	20.40201	22.80365	27.43248	18.43464	21.81927
Ankrd13c	23.59452	22.85843	24.17361	18.95981	19.77399	20.02275	21.1698	20.34798	21.81289
Gigyf1	20.93964	24.70685	26.60072	30.18937	26.52552	24.62494	34.8972	22.7514	21.80344
Rnaseh2b	24.27728	25.87358	23.87297	24.3621	22.02879	22.17708	25.58903	20.92976	21.79807
Fads1	37.79722	36.8177	38.00993	28.08955	26.3993	22.64063	33.02087	25.90307	21.79161
Pum3	25.32703	20.56631	22.17155	21.95151	18.41846	16.71323	23.7045	20.85399	21.78462
Tmem183a	22.92117	24.90342	22.451	19.41478	19.89537	18.97832	23.34631	20.23939	21.77916
Fech	28.39799	29.4804	30.07427	23.30738	22.30399	24.51444	29.10665	24.99532	21.77644
Ncoa1	24.54036	25.11422	26.81765	23.51888	21.59988	17.20703	28.78255	22.00664	21.77338
Lztr1	24.89496	28.18343	28.5138	23.23512	24.17224	24.26967	29.85743	23.75382	21.75557
Chmp7	22.35141	23.31354	21.90324	20.43345	21.91162	22.94339	23.99063	21.33925	21.75198
Sgf29	29.52576	22.2869	28.19776	19.66814	22.23337	23.96289	21.37619	20.09898	21.75124
Dapp1	21.62558	24.55464	25.55186	20.88249	24.35054	21.96172	26.95455	25.38972	21.74914
2310009B15Rik	11.35946	11.91021	16.31292	19.11602	12.39057	15.98178	17.05895	16.7314	21.74569
Setd5	29.23872	28.42471	30.89225	27.33061	22.96706	20.05242	34.59062	22.8691	21.73418
Tbp	24.28801	23.50295	27.99315	19.42332	19.34802	20.2342	22.52104	20.1731	21.71439
Ebna1bp2	22.53108	21.35034	21.12885	20.95218	20.0492	23.03568	21.62225	25.01818	21.70617
Abcf3	22.47839	21.18331	21.75998	21.19441	23.34476	23.00964	22.69758	21.68323	21.68818
Pspc1	20.41334	17.25339	16.34322	19.66383	18.09803	14.12745	18.67755	18.67531	21.68794
Rab40c	22.63833	22.80227	21.31443	23.56221	20.51138	19.72942	23.21197	25.83473	21.68347
Pja1	26.54635	20.43511	26.00814	20.52866	21.99328	22.61866	26.61121	20.02348	21.66554
Ugt1a7c	27.55073	25.5303	30.34804	18.71056	21.12585	15.2059	25.30923	22.59359	21.65467
Zfp358	20.41141	22.87405	22.68815	21.92415	24.59005	22.38941	25.82566	23.02467	21.64774
Fbxo38	24.68169	22.71941	23.69401	20.77459	20.91569	17.37815	22.6162	21.94031	21.6359
Slc7a6os	19.08523	17.76546	18.39667	19.88206	19.04212	17.01069	22.20533	20.87071	21.62002
Ide	29.72087	27.78427	27.21476	21.42554	17.31276	16.09905	25.36973	19.28243	21.61965
Noc4l	28.24921	24.13365	25.61487	20.08816	23.12367	22.02561	24.67271	25.96411	21.61177
Gm13391	16.28898	18.18401	13.3289	26.50539	16.13046	20.19884	27.93158	23.52741	21.61129
Wdr70	22.34022	24.97945	22.73885	16.53826	20.58517	21.81915	22.64635	24.38047	21.60672
Kti12	20.73583	20.28976	19.63363	20.33625	18.9342	17.82497	17.49136	20.01392	21.59678
Sptbn1	30.62921	28.73574	30.91541	33.74294	22.9548	22.11485	28.58989	20.95539	21.59285
Abraxas2	23.59229	23.87326	24.15044	21.15602	18.49319	20.50695	24.75986	22.73566	21.59124
Prkaa1	25.57371	24.32975	27.35149	18.05756	18.66068	17.69236	21.70817	21.68699	21.58213
Arl10	26.57846	26.57032	26.07922	23.05825	25.51235	23.68867	24.20403	23.87067	21.58175
Hspb6	30.13313	32.58956	39.83727	30.90742	27.38466	45.72604	22.05483	23.43607	21.57048
Cript	15.481	16.95071	16.6665	15.86505	20.06437	18.60686	15.46214	20.41884	21.56813
4833420G17Rik	20.95333	22.99632	21.90624	22.72604	21.20564	18.37256	31.01108	22.81495	21.56629

Gm43305	17.98222	22.35418	18.02692	11.61026	17.85656	18.28445	24.09071	18.09453	21.56546
Nup50	24.54964	22.1907	23.23322	22.56642	22.11338	20.66241	24.6011	21.76207	21.5622
Synj2	28.06621	27.28513	27.65281	23.61425	18.39751	17.90249	30.37139	20.08431	21.54792
Mpo	0	0.282316	0.021777	8.076421	7.009398	13.33033	1.912934	0.331704	21.54172
Prxl2b	20.19782	18.5945	18.22253	24.78983	22.86819	26.97252	26.49577	17.28038	21.53881
Scaf11	27.74438	26.43542	27.82728	23.27527	21.14703	19.36842	28.90724	22.71267	21.5353
Map1lc3a	21.97343	26.94642	24.38661	21.5159	23.11607	22.54936	21.60986	23.53596	21.5303
Nlrc5	20.19818	14.58316	20.95915	28.32608	18.24511	14.95485	22.37708	18.86186	21.51182
Wapl	23.48091	23.49823	23.13672	20.33342	17.87342	17.00962	22.31984	19.7225	21.49778
Srpk2	19.17914	19.7263	21.70089	19.53396	18.78014	17.75014	25.71203	19.24554	21.49512
Mycbp2	26.9032	25.84414	27.12759	25.94948	22.56702	15.95872	34.72866	21.44897	21.49237
Ube3a	23.28652	24.45474	24.64969	20.40486	18.70843	18.06991	23.46013	20.20639	21.49094
Arfrp1	18.25474	21.10813	19.81911	21.32004	19.13585	18.72251	22.82423	20.38395	21.48526
Fnip1	21.27719	22.31128	19.00319	20.94581	18.62982	16.68348	28.48818	21.21457	21.48241
Irgm2	16.45472	15.87595	25.27664	24.92818	22.17722	15.97547	17.72423	18.89594	21.47177
Kat14	30.90269	26.34976	26.79676	27.26829	23.10849	22.67384	29.272	23.32775	21.45862
Lin52	23.33611	30.83507	24.87895	22.61642	19.85589	19.71822	26.93496	19.51147	21.45108
Cipc	23.6644	18.32214	19.94377	25.94157	16.12968	18.06565	30.05522	20.67474	21.44463
Slmap	26.15123	26.18289	25.55754	23.83347	22.65734	19.21793	28.76053	20.77698	21.43986
Lcorl	22.65268	23.96802	24.14091	20.83768	21.47312	21.29191	27.08633	21.1635	21.43833
Septin6	21.38727	17.36804	22.87976	20.75718	22.46581	22.31945	22.31012	21.38722	21.42526
Tmem19	22.9642	25.06983	26.83598	21.79328	23.46806	21.20133	25.46797	23.27945	21.41468
Alg9	25.59067	24.54284	24.40969	21.81557	20.3662	17.42299	23.32531	21.51107	21.41343
Usp24	28.65169	27.09513	29.19663	21.19139	20.16242	14.82933	28.83557	20.87237	21.41023
Ddx28	18.90405	21.09962	23.35129	16.05799	16.33907	20.90701	22.17197	22.96362	21.40607
Serpinb1a	15.00123	17.55573	12.71928	11.75556	10.59826	19.77991	8.006186	9.108966	21.40493
Rabif	20.41573	20.47279	23.18335	19.69039	18.51276	18.10501	20.66769	18.66665	21.40114
Rrm2b	24.91238	25.11264	25.343	20.89211	19.34761	16.36403	19.55978	18.62911	21.39991
Ppil1	25.00416	26.25729	28.3383	24.18352	24.55244	24.22015	21.36296	18.68022	21.39588
Ormdl2	20.75857	20.94831	20.76098	17.75155	22.92879	16.66001	21.24343	24.79295	21.38471
Tob1	20.98333	23.05018	18.63176	15.08911	17.4326	17.08913	21.16972	18.38089	21.38316
Col6a2	25.64512	23.79759	25.94547	34.51938	21.36581	31.4286	18.48471	24.36005	21.38151
Tma7-ps	21.69316	22.83737	23.26259	26.2455	22.95591	20.28405	25.7841	23.29313	21.36469
Thbd	22.42573	18.15752	22.33886	22.83389	20.59117	20.77998	23.70208	22.45881	21.3595
Mrps9	19.33886	19.87184	24.18615	18.91974	18.93903	17.37816	20.18957	22.05779	21.33603
Fam53b	25.04929	22.23553	24.17159	24.77427	22.74931	19.39621	29.89157	21.15168	21.33508
Chpf2	22.76867	23.12444	25.91785	23.53076	21.50002	20.61451	25.18153	19.36398	21.31686
Cox19	23.16597	21.72385	17.55864	21.29762	22.27116	27.28422	20.85617	23.3741	21.31548
Med4	18.06839	18.7835	22.36489	23.6744	22.39082	21.89872	20.65189	21.0887	21.31325
Ccnt2	21.56626	21.96514	21.26898	26.55973	22.71812	20.30854	28.81206	21.43443	21.30637
Foxred1	24.936	25.49135	26.58664	26.5232	23.4095	26.60139	26.96918	23.27516	21.30472
Actr10	20.4688	19.96066	18.67909	17.98292	18.56221	17.10612	18.61971	20.28884	21.29523
Eepd1	22.26611	19.53079	18.78448	22.67932	16.63094	19.33033	27.91097	23.0331	21.28345
Plscr1	19.63644	18.97322	18.75379	17.90206	19.12288	20.70837	19.2752	18.63101	21.27768
Faf2	27.77742	24.79191	28.30619	23.32542	22.90998	21.8676	25.57138	22.05523	21.2713
Smyd2	32.31647	30.40708	28.82469	28.82031	22.99377	29.48745	26.89838	25.85368	21.26821
Pip4k2b	24.10398	25.47323	26.31227	23.11799	23.29074	19.05406	25.24151	19.50225	21.25835
Rnf14	26.32773	28.4351	26.84456	20.62493	23.07646	19.02238	23.02982	20.66464	21.25736
Tnfrsf23	27.9652	25.53379	27.96845	10.77092	15.28627	12.88514	10.7879	14.81284	21.25696
Arid1b	23.85811	22.88949	26.64744	25.86972	21.59254	20.87063	31.77554	22.50304	21.2518
Zfp639	22.8682	21.54525	20.67116	23.15955	20.74641	17.58137	24.35165	19.36313	21.24982
Camk2g	26.49224	22.48724	22.69424	22.00636	23.62726	22.27469	26.50261	19.80297	21.24501
Minpp1	26.29022	25.90355	24.8537	18.94417	16.89394	17.95044	25.02822	22.37058	21.2439
Mfsd13a	27.71903	21.49312	23.07563	21.79521	25.02434	20.97302	24.38963	22.2676	21.24003

Smyd5	28.90874	21.84561	21.37271	22.02447	22.08943	19.8859	25.78755	21.74392	21.23669
Fam3a	28.88219	24.84323	28.20547	25.1615	31.08607	28.3642	31.62672	28.35056	21.21682
Cdip1	21.46789	24.37634	23.85849	25.15571	24.62934	21.49855	30.08087	22.24113	21.19078
Galnt9	23.5816	29.96524	29.40939	19.88264	19.41473	18.98305	22.1704	29.58659	21.18912
Zfp592	25.33071	22.07212	24.00792	22.42373	21.42563	18.5005	30.70014	22.93502	21.18724
Spindoc	22.60649	26.32714	24.52219	23.34447	21.62954	19.5871	25.98899	20.76199	21.18137
Ern1	32.34817	30.44035	32.53321	22.50301	24.32773	16.96878	31.64648	23.90876	21.15622
Dyrk1a	22.2691	21.33598	20.33139	21.59464	18.30815	16.63899	25.88382	19.41481	21.15484
Tmem273	17.09173	18.24386	25.98086	30.88734	27.82887	29.24277	36.11636	23.0566	21.13496
Casp6	27.32439	26.42697	27.10203	19.73379	27.67005	26.65904	24.39535	26.19442	21.13496
Ythdf2	21.28887	20.86612	21.87597	20.25038	17.50459	17.04722	21.58159	18.41821	21.13237
Gpr183	31.39383	25.95066	33.80811	27.70129	21.75167	21.23047	28.19977	27.9337	21.12743
Golga5	26.59865	25.34836	24.73423	24.69598	21.81499	21.41258	27.25515	21.01817	21.11914
Eci1	20.07153	22.39999	21.34436	18.89798	22.69711	21.99188	22.32049	20.02928	21.11559
Chd8	25.62598	24.32677	25.04114	23.59268	20.1641	18.75194	28.61863	20.43774	21.1001
Slc2a3	12.31957	9.343661	8.209225	10.8973	13.99777	13.24739	12.62925	11.59305	21.094
Isoc1	23.51197	24.78687	25.7645	20.75617	20.22951	17.12247	22.80295	19.44075	21.08961
Tprkb	25.47283	24.02276	26.38394	20.86292	25.39795	19.22527	24.54923	22.93307	21.08941
Gpatch2	25.1486	25.047	26.29793	22.93526	19.88218	20.65472	24.73579	19.77689	21.08861
Ip6k2	19.00608	19.43703	22.65191	21.21297	19.39711	23.21848	22.77403	20.85388	21.08476
Mterf3	16.0548	21.0863	19.48661	20.34959	19.02329	21.71284	19.47587	20.84746	21.08467
Fam193a	25.71302	23.51916	25.98617	25.49934	20.79165	19.27269	31.32208	22.92861	21.08347
C130050O18Rik	24.96254	24.55457	34.5886	20.05566	27.00262	26.21951	25.37534	25.52728	21.08274
Timd4	31.10243	25.44724	28.31209	6.605426	15.60899	11.53947	7.571293	9.161571	21.06396
Anapc4	24.43597	24.5367	25.29084	21.11707	22.36223	20.86821	26.99535	22.29604	21.05178
Mir6236	18.93007	17.98064	22.98919	19.27222	26.18467	44.59846	23.75771	20.59804	21.04904
Foxj2	24.95985	23.60295	25.18395	21.30102	19.50589	17.97887	24.89429	21.61628	21.04853
Crls1	19.98396	18.04818	21.54579	17.995	20.00954	18.10107	14.6744	21.56619	21.0411
Scoc	21.32002	19.25105	20.72016	20.96104	20.41967	21.21985	24.55629	21.38822	21.03738
Calcoco1	22.39902	27.54734	25.87672	27.42711	26.51148	24.05428	29.29827	24.70907	21.03018
BC064078	17.73771	21.08065	20.89213	26.67263	27.25279	25.03357	22.10157	19.53599	21.02893
Parp8	22.1555	20.14586	24.45936	20.78927	24.98268	20.57991	23.90107	20.89078	21.00651
Plscr3	28.81947	24.67901	34.86103	22.50646	24.75643	23.32336	28.12643	25.93817	21.00457
Dcn	33.4951	37.76455	25.70498	51.75423	29.92115	37.0233	19.07828	29.9411	21.00097
Wdr18	22.98972	20.30811	23.0487	21.98383	22.52073	23.3428	23.64464	19.44053	20.99902
Umps	23.39053	24.75618	26.22375	22.00135	19.51804	18.76725	23.9688	20.36801	20.97629
Hacd2	21.17625	19.51241	21.45003	16.90006	18.22266	18.29924	18.40661	20.11629	20.97374
Crebbp	20.84743	21.70521	20.65044	21.28876	17.27366	14.40295	29.75191	17.64806	20.96745
Kctd13	20.07703	20.15729	17.56525	22.76427	21.54719	17.51274	22.03236	19.17912	20.95607
Tmem69	28.1344	29.27468	29.5224	19.49565	25.00154	18.84755	25.87283	22.14649	20.95549
Tpst1	17.45217	15.60308	23.17349	18.94548	18.73772	16.52284	16.23086	16.81366	20.95422
Dennd6a	21.66711	22.49523	26.06966	20.4777	22.42564	19.06291	26.85577	20.96353	20.94597
Zfp706	27.22326	26.34966	27.56535	21.84133	27.36156	25.3029	25.23084	25.51292	20.94596
Ahcy	25.61531	26.28635	22.15164	19.70543	18.47847	19.8578	21.38254	20.32011	20.94381
Lrif1	21.49004	22.08858	22.0809	17.85967	22.60914	23.52155	19.27817	19.63692	20.94366
Gk	20.32157	15.54296	17.26847	14.83152	15.7742	13.03112	13.90763	18.41667	20.93866
Nup85	24.72854	24.9837	22.42458	23.86992	18.82563	21.256	26.61156	21.22024	20.93557
Tspyl2	22.28373	23.28653	20.09587	28.19316	22.65805	23.35741	31.69147	22.80417	20.93196
Senp6	23.68827	22.79223	21.85095	18.48818	18.19131	16.90257	22.18108	20.60591	20.9273
Deaf1	21.15184	21.61404	21.86582	23.72715	21.70841	19.86421	26.01884	17.66363	20.91589
Fbxw17	19.79473	18.75013	23.03771	26.28766	23.92437	23.43821	24.60366	20.77088	20.9148
Bcl2l12	23.03301	20.51609	26.53075	24.32561	27.15573	21.81435	26.84305	23.82017	20.89272
Gale	19.37915	19.97047	18.37405	18.62958	18.303	22.20593	16.19835	17.22335	20.8892
LOC100042019	5.654884	16.21451	7.170419	7.430668	6.63589	10.73907	11.16737	18.55559	20.88473

Agfg2	23.46956	22.20173	21.32781	22.58213	20.19862	20.83864	21.41624	22.17829	20.88229
Tom1l2	29.75214	30.187	30.09926	25.05797	26.3993	22.42948	31.40144	24.07382	20.87841
Utp4	20.66889	16.15615	19.8563	16.45231	14.34617	14.50102	17.15112	16.92801	20.87607
Ramac	18.97283	19.73827	22.55964	18.86655	24.19163	23.63416	20.33415	21.62486	20.86762
Papss2	26.64847	20.64751	22.0145	21.78142	17.98585	17.02265	24.37434	22.24813	20.86189
Rnf2	17.33737	16.96334	15.36664	15.58849	16.2977	16.35308	19.457	19.06034	20.86121
Slc25a17	22.11536	25.24058	23.84746	21.03246	20.14392	19.89802	23.54477	23.39907	20.85603
Cpeb2	18.73929	14.61821	15.34333	17.72437	16.48299	14.87174	28.99031	18.94775	20.85587
Nob1	19.55648	17.96017	21.57858	19.04527	20.80528	20.85558	19.23849	22.72178	20.85265
Atf5	22.5776	27.51784	23.02736	18.5452	19.55617	17.36476	19.73745	18.48603	20.84886
Ltb	10.37882	8.280947	9.519239	9.011714	14.22428	15.02907	8.21533	14.60556	20.84544
Pik3cb	24.68745	23.83875	24.12259	21.36642	16.48375	17.51356	24.21746	18.19213	20.82415
1810013L24Rik	19.62241	21.30732	18.59038	17.27979	18.31233	15.25186	19.92913	18.21604	20.81591
Oser1	21.18856	21.09675	20.55209	21.74043	20.14595	21.89203	23.36673	23.51895	20.80346
Slc12a7	27.61364	23.63427	25.87484	23.68597	21.07929	20.84757	29.41152	21.43199	20.79871
Ecd	27.33762	22.15157	22.65684	21.3101	19.85228	20.45774	23.43259	21.34945	20.7968
Slfn9	21.27809	20.37546	25.9968	23.54643	18.77253	13.60128	20.72892	15.65953	20.78691
Syne3	28.32268	22.80176	23.82254	19.08968	13.72536	13.05573	21.38136	20.05879	20.78458
Prpf38a	20.07303	18.38162	22.0829	21.23081	20.1601	17.58414	19.12448	19.35473	20.76625
Setd1a	28.88684	26.01466	26.75053	24.3308	22.11064	20.25122	31.36231	20.76964	20.76455
Pex14	25.40092	22.3628	24.61702	24.02923	25.47606	21.54114	23.58134	25.89632	20.75468
Ap1g1	26.77044	23.49463	26.14378	20.96082	20.64428	18.02981	25.35731	20.53805	20.74408
Desi1	23.47026	23.5188	22.39676	17.78545	18.43003	19.37111	22.89619	20.42053	20.74239
Acbd5	25.84045	22.56205	26.6679	26.9726	25.18389	19.48366	28.52612	21.64428	20.73003
Vapb	22.46479	22.41291	23.02431	21.92761	21.06944	22.03269	21.05613	21.19176	20.72858
Nod1	15.29279	10.41705	16.84718	23.35912	21.89242	17.78176	20.39421	18.56657	20.72742
Alg12	25.50515	22.80581	24.32522	24.63851	19.81392	19.71607	25.57723	24.88976	20.72452
Pgp	23.19718	23.64718	18.17502	14.71201	18.73302	18.54563	19.06987	18.97156	20.72335
Polr2b	24.83951	23.07637	26.8932	17.8022	19.11387	15.97735	21.77986	19.80035	20.72191
Pelp1	22.44919	20.97502	24.93391	20.19206	19.47716	18.033	24.28503	19.0109	20.72025
Cnbd2	14.38898	24.29053	17.8793	20.28569	18.86512	15.35141	23.40205	17.02086	20.71938
Raver1	30.37	25.41668	28.89971	22.82512	25.72044	21.80456	24.96544	25.72783	20.71666
Pgrmc2	24.94436	25.15376	25.40822	21.61468	22.38717	22.2153	24.37118	22.81916	20.69919
Fbxo7	17.85792	20.41996	17.72916	17.43266	17.91103	19.7508	17.54434	19.15116	20.69854
Setdb1	25.25258	25.46067	24.11518	23.57152	22.9415	20.58909	30.20581	22.05459	20.68197
Adprhl2	20.38402	21.58268	21.31857	21.31759	23.92919	23.98474	20.31399	22.26278	20.66375
Cdk2	22.18056	25.12961	22.10306	19.40213	16.06582	19.12962	25.35237	19.54247	20.6567
Dpy19l1	24.64444	26.322	30.46349	21.04611	25.24164	22.32019	26.6044	22.28391	20.65641
Synrg	26.34232	24.19181	25.92814	23.06561	19.51426	17.83501	28.19934	22.08443	20.65328
Cd22	17.42166	19.89191	24.7226	26.57961	24.24793	21.5745	29.28148	21.62225	20.65108
Ino80d	21.00314	22.65364	21.84096	22.57348	18.46347	14.65138	31.51042	23.22942	20.64703
Neurl4	21.95567	23.22669	23.63646	23.68846	20.99995	19.14369	27.68458	20.00806	20.64171
Eif2b1	22.2634	19.70341	23.14781	18.22058	17.37211	19.87503	21.04534	22.50863	20.64125
Eefsec	24.6595	20.61059	23.74623	18.72806	22.43853	24.22748	27.9484	23.02474	20.63906
Angel2	19.13016	18.63262	24.56039	19.11206	19.46469	17.12778	25.52955	20.26028	20.6228
Hmgcr	21.84986	18.34695	17.65964	15.49295	15.05297	11.66536	17.33168	20.45767	20.59824
Adi1	20.90505	20.35455	21.30445	27.8269	23.50105	25.20544	22.7004	22.10817	20.58641
Zkscan6	14.20812	13.26821	17.60203	17.01421	17.62346	14.28935	17.76199	16.21868	20.5818
Acp1	18.99999	20.47049	20.06808	18.6012	16.80035	16.99284	18.47894	19.12205	20.57804
Grpel1	21.04255	19.3467	20.74601	21.40362	17.76298	19.27519	20.46112	17.85243	20.57778
Prrc2b	29.64568	28.02048	30.12922	24.39648	21.32703	17.89499	31.85383	20.14084	20.57604
Rps18-ps5	19.04361	16.9881	12.69722	20.1994	15.71867	18.58932	13.88711	20.85109	20.57028
Ess2	25.42343	21.96044	26.95696	30.12603	22.72571	25.3593	27.11568	24.26507	20.56938
Gadd45gip1	20.26974	18.20695	19.02508	19.80649	19.79186	22.44057	18.93412	20.36417	20.56819

Fscn1	18.29227	18.40943	21.82855	17.52836	21.36097	19.28053	18.69114	18.16809	20.56439
Smim7	20.79415	23.59864	22.64992	19.10648	18.26215	21.48468	20.20148	19.07375	20.56234
Extl3	25.43673	25.62403	29.01981	21.63101	21.07795	17.94446	26.30971	21.91906	20.56091
Tm4sf19	38.6488	38.45049	36.40489	26.39843	16.90102	14.99538	28.22878	18.17101	20.56054
Adamtsl4	26.16804	30.79846	33.48944	19.35791	27.30832	24.58554	36.49973	29.23104	20.54367
Cpsf6	20.12536	21.79802	22.85155	20.07168	19.52272	17.5599	21.79148	21.16469	20.5317
Gm11560	20.66961	20.74478	22.06465	22.15486	20.59726	20.60353	24.82614	19.43091	20.52944
Acat1	22.54996	20.98186	24.14704	22.53805	21.89898	23.01181	22.3892	24.09873	20.50523
Phf5a	24.08595	24.52508	22.94487	26.25917	24.25276	25.57147	26.59768	27.80048	20.48732
Dstyk	24.22715	25.65262	24.572	22.67746	19.23378	19.04217	29.72799	20.68456	20.48307
Zc3h3	31.03976	23.80677	24.17881	23.93004	23.81892	20.89242	26.79651	23.81164	20.47284
Ints4	26.48688	23.92426	29.19583	23.9737	17.9059	20.30297	25.63004	18.9505	20.46484
Efr3a	28.23816	27.203	27.89531	19.6377	22.73245	19.53281	25.1056	21.53105	20.46083
Errfi1	29.71975	29.53179	23.86588	16.31399	19.33177	14.83525	26.29918	25.0405	20.45542
Baz2b	22.16413	21.84432	23.32378	20.98408	18.1731	15.19848	24.79146	19.21626	20.43711
Naa35	20.0018	17.70462	21.15172	16.86938	17.8281	16.04186	18.27769	18.35819	20.43589
Coa6	11.11668	16.73932	11.55397	12.64393	13.70134	18.47776	15.77009	18.88547	20.42861
Gm18446	21.13756	19.26063	20.08302	13.47137	16.74548	13.32774	17.9739	17.0148	20.42069
Gm20559	13.6851	12.79228	16.63872	23.81922	17.82102	15.62505	14.72664	16.5041	20.41716
Sin3a	23.38089	21.90267	24.04849	21.07619	21.20529	18.69384	28.39279	19.83634	20.40469
Abca3	29.12023	29.55607	32.06935	25.00454	26.59902	18.37023	33.82119	22.15303	20.40454
Slc4a1ap	22.06049	22.05377	22.61072	20.89971	19.61942	20.59377	20.86014	21.47546	20.40428
Bbip1	17.41638	16.66665	18.00106	18.38601	17.40485	20.1043	16.66966	22.56684	20.39851
Scimp	16.81534	14.93629	25.85658	15.57157	21.0694	16.32781	14.06652	20.41256	20.38372
Rnf214	22.84048	22.65066	24.09988	22.19802	20.89065	18.90058	26.93006	19.70546	20.37762
Terf2	26.00445	24.43248	22.94166	23.74049	23.94988	21.47753	27.8972	24.31357	20.37731
6030458C11Rik	23.8325	29.03481	24.78868	24.6105	20.53279	23.32802	23.15415	21.25961	20.37315
Spen	25.58146	21.65567	24.93661	22.34639	18.36186	15.12093	27.46306	18.42232	20.36855
AI839979	21.2678	20.83409	20.84487	15.60302	14.89884	13.76746	12.99341	24.20884	20.36019
Tmco1	20.14803	18.84622	20.89393	14.03448	17.5838	17.452	17.35333	18.88796	20.34842
Psd3	31.46582	27.47497	28.07271	25.35687	19.98988	14.75703	36.77473	19.21385	20.32998
Epb41l1	24.56258	22.88021	25.74101	27.17582	21.02873	18.39479	30.07857	21.2442	20.31985
Lonp1	23.15058	22.97713	24.77904	23.2067	20.64253	21.33981	27.67376	20.52948	20.31934
Gtf2h2	21.52176	23.44798	21.95048	21.6131	18.77209	20.26392	28.59394	21.33616	20.30906
Tle4	30.38403	29.22196	30.46286	23.04217	22.57061	19.27052	28.83574	22.40603	20.30552
Arid5a	21.814	17.68294	17.94295	16.36365	23.26005	18.90166	27.09859	21.63842	20.28382
Galnt7	28.5621	28.97017	29.83017	21.16186	18.32136	17.31751	23.63213	20.94272	20.28274
Tcea1-ps1	18.42663	14.78882	19.57563	16.3967	15.96762	10.78736	13.7319	20.06637	20.27569
Fam102b	27.32041	28.70217	28.71407	18.07935	16.87111	15.36583	24.76013	19.52266	20.27503
Tent4b	22.09844	23.17299	23.2529	19.31754	17.22902	14.85476	23.26839	19.63625	20.27141
Sart3	21.64527	18.29218	19.65223	18.20653	18.91203	19.94306	21.86002	18.5415	20.26473
Golm1	28.53728	30.07789	33.79356	22.67404	26.65906	22.70063	30.8156	23.23794	20.26152
Arhgap31	30.88507	27.47339	27.46377	23.68013	19.5482	17.69329	28.95323	19.07059	20.26049
Ccl24	25.6669	28.77548	32.47026	7.857957	21.11944	10.19683	17.60093	30.44469	20.2592
Tlnrd1	21.6305	22.79456	23.44898	23.47255	21.73979	23.65469	26.9734	23.68145	20.25869
Ece1	26.40928	19.77204	22.9947	23.59733	21.53448	22.79284	20.83452	22.68422	20.25661
Tfec	23.30954	21.50703	27.769	23.33391	30.24271	28.09764	25.63756	24.29952	20.25582
Rock2	24.78944	25.04041	25.37098	20.76095	17.6254	14.65751	23.20388	21.57696	20.25576
Foxk2	25.03099	25.17057	24.24117	19.57907	18.81757	17.23077	26.44067	20.94456	20.24375
Parp4	24.14545	22.53629	21.34885	20.33503	19.11361	15.87027	24.60444	19.03015	20.24139
Rrm1	30.9854	32.2862	30.25381	19.06086	18.94251	21.92043	24.24091	15.83342	20.23869
Mtmr2	23.15255	22.46911	21.47	18.06306	19.41833	17.46831	22.62313	19.31614	20.2356
Tmem119	24.03535	22.64252	31.82441	15.92515	21.64693	22.10634	23.30263	15.52133	20.21865
Asxl2	24.95314	23.3995	24.48699	23.86866	18.63413	17.22629	31.04781	20.12833	20.21804

Gamt	22.38845	23.67193	24.80412	23.37898	23.88763	22.77974	20.9938	20.78176	20.21512
Khk	14.06951	13.36186	17.72544	14.99302	18.63951	19.4583	19.49	21.39289	20.21431
Cdca8	25.43468	32.33778	25.84682	17.90119	14.80699	24.0572	21.28413	17.49677	20.21209
Gm13212	13.46183	15.20192	15.03057	16.76255	18.06685	15.98401	15.8275	20.59307	20.20719
Gpihbp1	11.00609	19.37877	8.289497	27.0233	15.91723	30.65991	7.583559	13.97183	20.20253
Stk19	19.07933	18.41306	22.01322	17.02919	27.67274	24.11184	24.8752	25.06922	20.19389
Thoc1	22.15971	21.30127	20.79169	17.17593	18.91293	17.52752	21.754	21.31978	20.18369
Rusc1	21.60113	23.42326	23.48852	18.27973	23.91401	24.10532	23.52455	21.89147	20.17327
Trappc12	24.57782	19.64378	23.62547	23.42709	20.98199	19.82391	25.31466	21.0362	20.16803
Tk2	18.64376	19.03684	21.11232	20.18236	21.20869	16.32894	18.53009	21.03785	20.16659
Twistnb	21.13517	19.65143	20.06404	20.09166	18.33411	17.31708	19.72393	23.23428	20.16643
Gm8730	22.67003	19.7394	20.34985	18.52646	19.85958	21.1513	18.73405	21.29566	20.16291
Stk11ip	22.38898	22.53703	25.44561	21.91851	23.26629	21.91122	29.33761	21.26364	20.14806
Unc45a	23.53102	20.3675	20.23733	20.15542	19.74082	19.08976	22.38889	19.63773	20.14395
Emsy	24.55193	28.68897	28.97547	26.22186	22.49121	19.02471	32.43471	20.55711	20.13228
Bcl2l2	21.26196	26.86249	29.72025	20.53979	19.77399	19.13455	25.77304	22.72295	20.12853
Tmem131l	20.57554	20.72677	22.68967	16.34084	20.65605	17.39334	22.07236	22.2886	20.12437
Plekha3	25.75486	24.80633	24.16111	21.14023	21.52759	16.08541	22.49365	19.62092	20.12186
Aifm2	20.23135	27.37482	24.36388	21.03059	19.66539	15.77755	22.63465	21.20831	20.11717
Bahd1	24.90807	22.86208	23.61739	23.4951	22.5757	19.67085	26.00845	21.53242	20.11458
Fam20b	25.5868	25.19252	26.0215	21.59313	19.7839	17.22623	24.03866	19.09286	20.11013
Nol9	25.77894	24.50098	26.33978	21.87256	19.8168	20.94652	24.88929	20.48034	20.08561
Psmg3	14.63735	17.13723	13.15393	13.35999	18.51992	17.72125	13.37441	16.22522	20.0836
Eea1	24.35851	21.47833	21.63015	16.73665	14.85719	14.1319	21.58547	17.59416	20.08037
Nprl2	18.85552	22.36943	22.71391	24.73491	24.31691	24.04653	20.5573	21.17566	20.07732
Armc10	21.83025	22.65915	25.55245	19.91237	21.68927	21.13502	23.23118	18.86005	20.06126
Ripor1	30.08715	27.42907	28.48496	26.54998	20.97877	20.56337	26.79881	22.97482	20.06077
Sdhaf1	15.59375	16.40989	18.92108	23.48763	18.07274	21.25979	19.13884	20.96015	20.05308
1700066M21Rik	20.13706	22.92928	25.03156	23.99669	23.68173	19.22527	21.73954	22.19969	20.04713
Cactin	21.55246	22.79413	22.81692	20.68294	22.21263	20.96618	21.43417	21.46952	20.03709
Nfatc3	25.01752	24.89399	25.73394	20.53567	19.02489	16.59944	25.69955	19.25455	20.03614
Fgd6	22.94042	22.7874	21.97496	23.04111	16.31664	14.69248	24.31354	21.03692	20.03369
Armc5	23.46417	19.51526	22.50091	23.39898	20.69756	20.75952	26.17911	21.45229	20.03028
Rtf1	22.72407	22.38748	22.34151	21.18448	20.82828	21.07016	25.55148	22.68982	20.02911
Treml2	14.87051	9.57245	16.44649	14.57087	16.74637	16.63941	12.96215	17.716	20.02747
Grk5	28.15652	26.10939	26.70717	15.62643	15.83291	17.08913	21.90274	27.82409	20.01417
Mrs2	23.44766	24.76686	23.96352	23.49489	18.03118	14.44787	24.1813	19.22249	20.00046
Asf1b	25.96817	32.43431	33.18348	20.92708	18.12785	23.95043	24.04438	17.13202	19.99164
Jkamp	25.17852	27.08039	26.24956	18.37584	19.38723	20.83018	23.68184	20.30569	19.98753
Wdr55	23.01686	20.4374	21.0067	18.41223	19.37691	18.95032	19.2128	21.80979	19.98405
Nelfcd	19.02263	18.24416	22.28485	17.58566	18.02427	20.15602	19.50998	20.52008	19.96183
Gm14586	16.79244	18.23897	15.41403	17.0415	15.33569	22.54914	15.81045	22.887	19.95105
Ring1	18.09777	19.27283	21.07109	19.29734	22.79114	23.97015	22.3389	19.95561	19.9504
Lemd3	20.50186	19.7466	19.0351	19.52962	17.82257	16.00629	26.3716	20.06707	19.93577
Nrbf2	18.31776	17.92364	17.76579	21.14197	19.52328	19.94977	22.90489	19.25357	19.93366
Hectd1	25.10945	23.26185	24.72525	21.52953	18.38438	13.93688	24.53877	19.08015	19.92708
Plagl2	22.085	19.01984	20.31359	18.05638	20.63315	15.94817	28.77087	22.33517	19.92518
Dido1	22.35387	24.20497	24.93063	23.50253	20.24861	18.5093	26.38356	20.11933	19.89384
Crem	21.17426	23.84402	17.67294	15.16007	15.72958	15.41582	16.03548	18.3639	19.88741
Pdk3	25.70923	23.89194	26.79143	16.49802	18.98694	21.1057	21.16262	21.24141	19.87509
Trpm7	21.26874	22.39581	24.53114	18.95169	18.33126	14.17289	25.9201	18.39116	19.86935
Tstd3	18.86361	23.69094	22.6739	18.619	15.66991	18.70436	18.99102	19.29038	19.84316
Dlat	23.31559	24.00716	23.64808	18.04795	19.4838	17.33054	21.28617	19.4386	19.83858
Tmx2	25.55675	26.21219	30.19172	19.97575	23.22395	22.67943	25.9528	19.01975	19.83729

Pwp1	22.42028	17.19386	17.24176	17.44244	17.11502	19.26254	18.97344	21.18257	19.83195
Nubp1	21.17324	18.81918	18.39617	15.2728	16.71724	18.46437	18.70878	18.03517	19.83145
Crkl	24.11507	21.08371	24.62588	22.71725	19.40786	18.35292	26.52408	18.57289	19.82555
Edc4	24.80186	23.04159	24.52353	22.36789	22.05893	17.7464	27.11121	20.63558	19.81961
Fbxw8	24.12191	21.24762	23.95764	20.56485	21.71128	19.03217	22.82482	20.69734	19.81668
Plekhm3	23.33912	19.40767	21.67407	20.94101	17.08743	13.46359	23.79151	18.89023	19.81159
Zswim6	22.98998	22.72614	24.9231	20.0427	20.10546	16.99518	28.41275	20.54425	19.81039
Cops7b	19.01271	22.40406	19.95626	19.99816	19.84056	20.97698	21.20707	16.78763	19.79534
Mtres1	19.9793	24.05495	21.84178	22.55217	18.91379	25.57821	23.44009	21.02159	19.78543
Dvl3	24.5679	24.29842	25.00733	21.43245	20.43607	18.06469	29.9461	19.49471	19.77502
Mak16	24.62919	22.15192	21.04921	16.06031	17.68483	16.50937	16.93947	22.85251	19.7749
Zfp219	17.95274	21.03575	22.94114	25.09506	23.87857	19.80872	26.14672	20.88248	19.77003
Dhrs7b	20.66879	22.85672	22.31347	20.34003	19.27676	20.00517	20.91949	19.44081	19.76724
Commd7	22.13727	22.97317	23.46857	19.54256	24.09049	23.58332	25.23395	23.24531	19.76675
Lzic	20.0074	19.39867	18.63861	16.23152	16.15784	17.63059	20.01445	17.09108	19.76298
Psmd10	17.43259	18.06878	16.38774	15.81363	18.9032	14.85526	16.76913	18.28412	19.76126
Nup62	28.69578	26.27257	28.3587	21.82438	22.00177	19.83087	26.52289	22.12186	19.76113
Gcn1	29.69896	22.78624	25.5641	24.11276	21.03893	18.02176	27.44188	20.63007	19.76102
Ash1l	25.42926	25.04951	23.12795	23.59914	17.93365	15.58977	28.22272	17.21501	19.75416
N4bp2l1	16.07105	20.70317	23.82739	20.11646	26.41608	17.92566	24.69679	20.70361	19.75104
Ghdc	20.37624	18.93581	18.28043	18.61923	18.50698	18.43104	19.9486	18.22668	19.74529
Carm1	27.12964	23.355	23.05056	20.29449	20.35871	18.79208	24.69397	20.11798	19.73647
Rn7sk	8.850052	10.15879	9.686088	18.92933	29.42091	29.80303	19.32945	32.59158	19.73445
Pphln1	21.6979	20.64447	21.24801	17.91567	16.74283	14.49739	18.2986	20.27239	19.72854
Chd3	26.62176	29.03406	30.38394	29.67856	26.3575	22.88472	31.14399	22.44414	19.7218
Apmap	23.09245	20.28663	22.60949	20.4951	16.39198	19.40926	20.18674	18.13354	19.72082
Nsrp1	15.47853	15.84544	17.25968	17.49715	16.46991	19.94097	15.97945	19.88659	19.72076
Llgl1	22.21728	19.59981	22.73856	18.95662	18.03563	16.21631	21.23865	18.07016	19.71884
Amz1	18.27288	18.92437	19.84438	18.54009	23.20386	21.59531	22.1723	19.40272	19.71809
Smpd1	30.33924	30.4877	32.52652	22.26128	21.08004	24.2096	25.79836	22.27718	19.71545
Mysm1	21.31227	23.81142	22.1255	19.37264	18.00632	16.54099	25.16247	19.02161	19.71433
Guca1a	17.38961	19.35513	18.64382	17.98389	18.74781	19.36196	20.12979	20.99378	19.68972
LOC118568032	37.37774	41.62457	44.79983	18.8342	24.46375	24.52929	29.10375	20.70605	19.68922
Sdhaf2	19.70853	19.58465	21.5906	19.33112	19.79308	18.02718	19.24118	20.96301	19.6882
Lig1	29.17132	26.03588	27.84777	18.16784	17.96382	20.32788	24.20155	16.38783	19.68586
Hmgcs1	21.71271	18.65973	21.84372	17.82222	18.88942	12.71289	21.08009	21.10076	19.68392
Tbc1d10a	15.30125	15.75186	16.08557	14.03403	15.84234	17.47325	19.97792	18.67942	19.66557
C2cd2l	27.62949	29.44403	27.03144	24.61277	22.79331	23.22055	32.28466	21.16657	19.66468
Thop1	21.38172	20.68313	19.15356	19.21466	16.66679	18.20637	20.50315	17.60096	19.66328
Fnta	21.89861	23.93813	21.41126	18.68147	20.82856	19.02542	19.26809	19.039	19.66298
Tlr4	22.23564	22.66692	24.65527	17.29066	16.8546	15.98898	18.92265	18.42804	19.65918
Slc46a3	21.02174	23.5953	25.003	28.34795	27.66602	21.90021	32.72434	24.02667	19.65417
Nme4	17.34447	19.28439	20.94427	27.85122	22.20845	20.49507	21.57804	19.38823	19.65331
Zfp217	19.58745	22.05663	25.56221	25.3072	24.09588	21.43658	29.23536	20.86661	19.64565
Nup205	28.4546	23.76399	25.08773	19.61785	18.94475	16.47673	27.92774	18.75939	19.64175
Gm19765	20.5891	18.63296	24.09968	9.023625	18.3742	14.52693	23.48666	18.15078	19.62324
Ncapd2	34.19617	35.73956	36.86935	24.90982	24.3926	25.93679	37.03722	19.39221	19.62064
Myo7a	29.20133	26.94496	29.4388	20.79113	18.14441	15.63417	28.13295	18.3617	19.61512
Cisd2	21.89311	20.3509	21.47049	17.13968	16.93089	17.86943	16.65616	17.90078	19.61374
Kctd2	20.33352	23.12317	18.74641	23.70577	22.25911	25.28788	24.73573	21.85176	19.60241
Impact	19.94069	20.13232	18.11067	28.25509	19.08477	17.42698	31.22733	21.89795	19.5977
BC029722	20.67711	21.24461	22.8255	17.49649	21.32751	20.74507	21.81437	22.9925	19.59557
Nsun3	28.10526	25.9563	27.93024	18.31676	22.01357	19.17719	25.30201	18.3296	19.59458
Fbxo33	18.56516	19.12543	18.51904	18.94109	19.25939	18.41817	22.71302	18.31916	19.5802

Sntb2	19.70327	15.60202	15.8653	16.04458	16.04398	12.14731	18.05261	16.88842	19.5799
Ccdc90b	17.32697	17.65026	19.96332	19.07159	17.08997	19.41081	19.48217	18.22112	19.57377
Pfdn2	20.53382	17.50272	18.91407	17.12856	21.10091	20.54895	17.33357	20.90915	19.57055
Cog3	21.96641	22.19377	19.45062	22.16908	18.05447	15.25905	23.25351	18.32183	19.57013
Rnu11	8.213046	12.28677	10.2675	13.78188	18.57729	12.34215	13.26343	17.10543	19.56941
Ptdss1	25.50086	24.70631	26.7021	22.36336	21.49569	19.33008	24.8336	21.967	19.56712
Gstm4	16.67074	20.46924	21.86322	22.12921	22.76706	13.45314	20.96863	23.65853	19.56108
Tbc1d8	16.8441	16.75281	17.94843	20.91789	21.02482	20.96062	27.06853	16.49545	19.54584
Csgalnact2	21.77372	21.62335	23.30523	16.09294	17.98682	13.82363	22.71172	17.99603	19.54542
Ptpn11	23.80302	23.9569	25.59815	19.94791	18.92602	18.04751	24.43787	19.36331	19.54389
Gm4617	19.79253	21.68068	17.8011	21.09761	18.9858	20.93711	17.31695	24.35766	19.5426
Dhx30	23.85278	21.00079	22.18072	20.20039	19.37474	19.09565	24.7584	18.84197	19.54165
Dpysl2	13.5468	18.46342	18.77757	20.73501	20.10922	14.52884	16.30252	22.03239	19.53982
Commd5	13.27625	20.49847	16.33335	22.03996	20.89724	20.08572	16.76455	19.93887	19.53607
Clip2	21.39118	17.01767	21.12172	20.3813	19.52027	18.66078	21.699	18.84549	19.53461
Cd40	14.41529	9.630796	14.278	19.99927	23.81892	19.36982	17.12478	14.25391	19.53262
Gm6421	13.76075	16.48208	15.93536	14.46934	16.2409	14.43037	15.24317	13.10159	19.51595
Gpank1	17.50446	18.2415	22.13415	18.88226	20.99472	21.9602	21.47547	25.64314	19.51239
Ripor2	16.1944	9.349819	12.67152	10.53135	13.52692	13.81905	11.07349	13.82478	19.49912
Zdhhc8	29.17308	28.25101	30.07846	26.79505	23.38424	21.85502	26.78472	24.1832	19.48949
Dmap1	17.29462	19.1123	18.50593	18.54588	18.21604	18.53487	21.24533	19.78034	19.48663
Ddx49	22.8088	17.87725	18.66632	17.90332	20.01319	18.92011	22.07337	18.72521	19.47436
Mideas	18.45164	18.31129	18.27082	18.41443	17.52562	15.67166	23.43658	17.36193	19.47225
Mad2l1	25.70841	34.03809	27.36133	20.47203	19.5417	22.12157	20.95835	16.03435	19.45779
C9orf72	19.26096	19.94048	20.35199	16.80057	17.48655	16.34051	26.24716	19.71369	19.45486
Rasa2	16.04935	17.30293	18.08587	11.21095	12.80945	12.69926	13.20015	12.32129	19.44373
Calhm2	21.07675	24.23174	29.3864	24.77914	26.56679	25.09575	25.30732	23.33866	19.44334
Sik3	23.11119	19.89021	22.89786	18.37286	17.6481	14.48969	20.55659	17.48848	19.41946
Gm45700	14.49405	19.18513	16.03215	9.032944	12.59533	17.23325	11.39057	18.63285	19.41615
Ppil4	20.17637	19.39432	20.09524	19.64839	17.86449	15.3204	21.47866	20.22302	19.40884
Hmga1b	24.70994	22.66697	24.42957	18.12942	15.74518	19.38077	17.37966	18.36938	19.40071
Ift46	26.57243	26.55887	27.4069	24.96986	24.97969	24.7324	30.871	21.04203	19.39739
Stag1	20.07568	21.96225	22.8587	20.12092	17.56933	16.46344	22.36633	19.01336	19.39588
Cenpt	24.4396	22.27194	23.19298	22.36159	24.65174	20.75968	29.89987	22.78567	19.38612
Creb1	19.88777	21.11147	22.2007	17.86186	19.30762	17.06346	20.7271	17.97398	19.3714
Nit1	17.84593	18.86962	19.87243	17.17087	18.09788	15.5101	17.71883	18.91831	19.37109
Gpd1l	24.59566	25.17131	24.41683	21.6091	22.78579	19.82459	26.04192	20.57013	19.36617
4930523C07Rik	18.89247	18.51055	18.19934	15.66055	15.1141	14.08875	18.40159	16.40458	19.36027
Bet1	18.01549	16.12951	18.43135	20.75697	17.08612	16.72972	19.51397	19.0862	19.35698
Ccng2	14.782	17.95721	20.14143	16.04755	20.21759	16.09557	24.81229	19.70348	19.34264
Wdr45	17.8498	20.23993	22.11114	20.36441	21.76135	19.93161	22.92686	19.00451	19.34195
Col4a1	24.3961	19.88451	19.31967	46.62714	26.65072	35.34853	15.44404	21.59174	19.33777
Slc38a1	24.66666	23.78016	27.38661	18.66454	18.36308	15.56938	25.13085	19.04977	19.33614
Sdccag8	18.61604	20.01144	21.27013	21.25726	20.51543	19.16207	23.78941	21.75845	19.33203
Trem3	8.747281	6.008578	7.473711	7.258169	9.875601	12.21417	3.352861	11.25144	19.32402
Ptges3-ps	22.1046	21.27098	21.57745	17.97789	13.08195	16.49046	18.79521	20.67594	19.31762
Ccdc28b	15.37746	17.44283	21.78177	19.75403	21.30801	25.32853	20.80463	18.47386	19.31081
Gclc	21.53127	20.0567	18.16968	16.5633	14.85282	12.87507	19.57651	19.30105	19.30399
Nod2	24.82045	17.90195	23.32019	16.5468	18.83327	15.05061	19.52837	18.99106	19.29731
Egln1	22.45675	21.51545	22.1156	16.37387	18.41014	17.21022	19.47151	17.90583	19.29727
Hk2	20.53099	17.59342	17.90625	16.96232	19.56396	16.10999	26.29756	20.28002	19.2955
Mier2	21.20349	23.0898	22.25842	18.59107	18.57334	20.41585	23.32649	19.24983	19.2912
Oxsr1	22.02553	18.91967	19.63506	18.97373	19.18887	14.24094	18.27499	18.08483	19.28483
Gm23969	17.23075	13.44903	10.4895	10.42953	8.561936	26.32649	45.68354	16.40536	19.27851

Slc25a44	21.99445	19.99939	23.18558	20.65618	18.60386	14.76724	21.45215	20.4309	19.27161
Arhgef3	18.50506	17.9944	20.38877	22.48038	20.92851	17.74037	24.64053	23.78515	19.26838
Esco1	18.56777	19.76549	17.67933	16.50434	17.52134	16.50508	17.89828	18.56226	19.26777
Zgpat	19.22211	20.29549	20.11038	22.21119	20.5058	17.42843	22.69722	17.89236	19.2671
Vezf1	22.74134	21.55882	22.88916	18.62043	17.69554	15.35896	21.62897	21.35254	19.26496
Ambra1	22.93017	20.35184	24.26261	22.76812	20.16434	17.70392	29.13813	20.79114	19.25558
Prkra	20.99145	23.20072	25.12383	23.06391	22.28619	19.25311	22.40704	22.4432	19.25236
Hsdl2	19.02104	20.32543	18.35717	19.92114	19.63773	18.32914	20.45767	18.79472	19.24591
Psmg2	21.05629	19.935	24.56853	18.00495	17.14744	19.89551	21.71016	17.82014	19.24445
Gm12250	11.63338	9.88353	16.41584	19.09673	16.72672	13.22478	14.25028	15.33466	19.24204
Zdhhc14	29.50898	33.96844	31.11852	24.61941	23.7068	21.37946	39.48963	22.26611	19.22809
Yars	24.95753	24.24576	23.55063	20.31377	19.43358	20.19495	18.74903	19.44965	19.22762
Mrps30	19.80429	21.45548	19.84146	17.78643	18.29687	16.57323	20.61287	19.88472	19.20756
Slc35e4	27.13004	24.50928	20.93442	14.39911	15.86202	16.50813	17.08263	21.91361	19.20732
Pafah1b2	22.6511	20.4993	25.07372	18.04611	17.21972	17.26731	21.79936	19.86382	19.20545
Htatsf1	19.58984	19.31884	19.81158	16.52437	17.78319	15.03524	18.76257	15.25165	19.20323
Pus1	19.90602	16.34955	18.61611	13.77184	20.15038	17.13891	21.36677	18.29895	19.2031
Mars1	25.2197	24.51292	21.9967	18.79475	16.00665	15.31567	21.77675	18.60587	19.20052
Fam149b	20.78014	22.80095	21.8783	20.84532	17.14737	16.34612	23.13813	20.51493	19.17879
Tbc1d23	18.83059	20.05169	20.31152	17.72801	18.35488	15.44937	23.74828	18.91561	19.17826
Cdk5rap1	15.42135	18.32725	15.36685	17.51768	20.2082	21.65201	19.9525	18.51405	19.17703
Rhno1	23.98903	28.26525	22.78491	23.81829	21.94137	22.35339	26.26788	21.86503	19.16924
B4galt7	20.91035	22.75831	21.93847	21.13316	18.80238	18.78399	26.67626	19.74596	19.16917
Pom121	31.09108	23.94201	26.67146	23.21504	20.06536	16.52277	30.75831	21.1905	19.15736
Fam110a	24.75406	23.89731	23.89493	16.01029	21.86808	19.5441	24.40866	22.94462	19.15684
Eif4ebp3	20.66284	20.31961	21.00244	19.39761	21.88092	20.80093	29.02236	21.46151	19.14265
Tle1	24.26573	23.22464	25.61827	16.39353	18.49841	16.73837	17.09368	19.24859	19.14252
9930021J03Rik	24.47977	23.49845	24.33756	21.45807	19.5846	17.61571	29.28195	20.94421	19.1382
Wdr11	22.3581	22.24861	24.85357	22.85563	20.70908	19.26291	26.75892	19.99914	19.13655
Armh3	22.06398	17.91063	20.39764	16.33018	17.40613	14.89277	19.68336	16.31642	19.13557
Agtrap	22.77666	22.95207	25.71723	24.51804	23.24358	20.63828	22.97964	23.26299	19.13399
Zdhhc13	19.7425	15.74729	18.36453	20.77168	17.10942	15.5749	18.56745	18.485	19.12848
Dync1li2	19.20844	21.88	23.28965	19.49333	18.41921	16.5619	20.19283	17.4428	19.1224
Mnat1	15.89351	15.00202	15.70612	15.42818	14.91155	18.02232	15.64451	13.28569	19.11524
Peak1	30.01889	25.87519	25.08079	19.33484	15.65755	12.42596	24.4648	18.45237	19.10214
Mrps27	17.94711	18.37707	19.83355	19.30734	17.94423	18.69369	16.79993	17.05701	19.08355
Fars2	20.71795	24.12118	21.29104	19.4234	20.48402	20.09292	19.50763	23.48674	19.07669
Kansl2	15.11277	16.07737	16.63827	13.3176	15.79745	14.52121	19.22633	14.55261	19.06456
Zscan29	16.83024	17.06211	20.69342	21.40272	18.25273	17.10338	22.10202	17.65285	19.05936
3110082I17Rik	23.56402	22.89578	23.30111	20.8662	19.98469	23.05453	24.19772	21.85722	19.05908
Rnase6	13.28657	11.63096	14.9999	17.82774	20.55581	21.46201	13.76232	25.68692	19.05022
Mgll	14.50414	17.0831	10.77576	28.93737	25.12757	25.29305	16.75158	14.5108	19.04604
Pigp	20.88317	18.58	16.0774	19.12283	20.03199	19.58998	19.56517	16.80743	19.04437
Trim34a	13.0944	11.11914	17.42479	18.97726	17.69669	14.43411	15.65754	16.40086	19.04313
Tnpo2	21.54198	20.2904	23.02991	18.30715	17.56664	16.01374	21.91961	19.86331	19.03902
Strn3	21.05853	20.38059	19.96966	22.33567	20.17738	16.53871	20.82765	23.16654	19.03065
Gm7964	21.32456	18.06463	18.02815	12.86117	14.4397	14.02093	17.16548	16.41876	19.02808
Scrib	22.33965	21.66269	22.82949	21.53829	20.88938	19.65469	24.63122	19.32716	19.02804
Cyb5b	24.89161	23.35746	25.07527	19.01348	16.69784	16.55982	19.4952	18.18008	19.02343
Kifbp	22.94221	22.6351	28.19326	23.88413	21.31594	18.27126	25.28808	19.02475	19.02317
Galk1	23.37528	18.72386	19.36508	21.15784	18.23059	22.05927	19.02324	15.45622	19.01585
Atg4b	20.36908	17.37908	17.64556	16.00885	17.91103	16.23215	19.62518	18.92626	19.01523
Slc39a6	24.03459	19.69776	21.45225	18.85867	14.38973	15.61001	20.32441	18.83146	19.00077
Coq9	19.6663	20.9172	22.77243	20.95301	22.02794	20.42236	22.2169	18.50652	18.99282

Tbl3	20.12523	17.91719	18.89302	16.3081	16.20697	16.45041	17.42862	17.60815	18.99205
Wdr5	20.74148	18.76063	21.03067	16.44321	17.49637	19.05732	20.03454	17.1452	18.98502
Ctso	17.69606	19.08382	21.02351	16.35704	18.37598	15.69953	21.01588	17.51806	18.98368
Cep250	22.24742	23.27857	25.38585	23.59971	20.23537	20.53557	28.87819	21.19155	18.98214
Olr1	23.67405	22.55961	18.10714	11.5998	17.65774	11.48695	23.2366	22.54001	18.98127
Stard5	17.64874	17.25975	18.10806	23.09673	23.55798	23.0439	24.12423	21.06946	18.97789
Tfpi	24.18726	27.70928	22.39187	20.26309	19.89631	19.2797	25.39695	22.00968	18.9725
Dvl2	16.15154	17.35625	16.17209	18.10352	16.73408	14.8501	19.99351	19.16453	18.97092
Spry2	20.63985	14.80947	17.98559	13.57717	10.82789	14.7882	16.80229	14.92173	18.96273
Phf2	25.18954	21.47499	24.73921	23.94798	20.58744	17.94358	26.88431	21.58462	18.95517
Ccar2	23.14782	19.9374	22.7315	22.75033	20.83443	19.08171	23.47594	19.09002	18.95301
Fmnl2	18.26412	15.17958	14.97586	14.75265	13.28018	11.31693	15.6354	12.33933	18.94586
Snord37	8.36801	23.99398	14.64572	31.20426	18.9278	23.21542	31.35174	6.97127	18.94171
Dhcr7	24.88233	26.28093	22.95547	21.76463	20.43375	18.88238	25.18136	22.86783	18.93912
Fmc1	19.1079	19.18241	21.49889	20.37593	18.07843	22.553	14.81173	14.36258	18.93041
Trim39	17.1847	18.56415	15.83082	13.62703	14.34252	16.40014	21.32107	17.26855	18.92787
Fundc2	15.3853	18.48102	17.27434	16.76986	17.47001	17.78087	16.67458	18.71099	18.92489
Hmgn5	17.61692	19.57057	14.21729	14.68526	16.05831	17.25939	15.77423	17.76945	18.92393
Nxpe5	17.75689	14.63648	21.59802	16.38505	25.09364	16.90399	20.97058	20.36215	18.91494
Kif3b	22.30516	20.04672	23.52847	18.5194	19.00262	18.63358	24.89493	19.72782	18.90401
Itgb3bp	21.51802	26.21804	23.95881	19.07855	27.96408	18.62486	15.66657	16.55129	18.89215
Upf2	18.31174	18.86201	16.07877	16.87577	17.14724	15.79743	20.16763	15.93182	18.89047
Otud3	21.98888	21.15596	22.1778	20.63036	16.20932	15.65307	20.22283	16.58878	18.8853
Klhl28	20.6096	21.17466	22.76838	22.09737	17.83387	15.16106	22.70639	17.16262	18.88372
Zcchc9	19.39579	20.1705	19.47338	17.2648	19.83535	16.02106	17.67207	19.68585	18.88076
Snrpert	13.63442	13.21327	12.60687	7.546459	16.41534	16.77193	9.97065	16.04293	18.87926
Fam71a	7.7921	6.088387	3.77241	14.53452	13.2351	11.58909	27.64872	19.79933	18.87388
Ppp1r3b	15.52477	13.04976	13.44229	8.525551	11.9717	9.099564	13.41452	14.73087	18.8696
Txlna	22.81816	22.16458	23.01493	23.11255	20.03343	18.11747	27.31999	17.24381	18.86885
Med13l	22.68154	18.00616	20.93431	18.14428	15.03387	12.47012	22.76685	17.04904	18.86875
Tmem181a	22.47937	21.50951	24.25179	19.41149	18.77576	17.74542	22.89062	18.71737	18.86862
Rab27a	13.55031	10.68441	14.87979	14.2883	13.63271	16.33223	10.15122	14.65025	18.86059
Pigyl	16.82369	14.06232	17.06601	15.69382	19.37983	21.85532	16.97412	18.76371	18.85959
Dpcd	17.03991	18.85696	20.48529	16.71234	19.39654	18.67212	19.48132	17.61268	18.85461
Mon1a	19.63675	20.10827	19.14295	18.30814	18.89634	19.94031	18.78771	20.75184	18.85461
Uap1	19.4321	17.71591	19.99072	15.12669	16.07708	14.17392	19.29099	17.5698	18.8496
Rbm26	20.54593	18.98253	20.99475	19.13619	18.43405	13.96638	23.43951	18.818	18.8494
Shld1	11.80335	15.71677	13.18612	14.35938	19.43323	16.25693	13.36737	17.51445	18.84913
Ankrd12	17.17931	18.15473	15.60232	19.19709	16.19018	16.00976	19.81559	16.61684	18.84656
Rhov	14.45338	9.743402	11.87858	14.53876	9.94156	13.27321	16.70183	13.88845	18.84545
Rpl7a-ps5	15.05486	13.59707	17.51219	16.51256	18.89181	20.13649	17.22947	20.67006	18.84411
Cd69	10.15737	7.674495	8.140526	12.10697	8.82691	8.381158	12.51295	14.39226	18.84023
Pja2	21.36573	20.48928	21.73621	18.33112	19.02852	16.51445	22.72905	17.16598	18.83209
Gm19253	17.0346	13.26971	16.43174	20.84823	16.31957	19.10875	22.18995	15.93071	18.82933
Snord14c	15.29326	14.61702	10.19669	20.91044	15.77889	9.428485	18.44074	24.8745	18.82712
Ctdspl2	16.33636	20.57542	17.27393	16.98433	14.89601	15.97253	20.77704	16.14764	18.81661
Mecr	22.84695	22.66206	18.99296	19.99764	19.42925	18.55902	23.18491	17.80471	18.81122
Igf2bp2	23.03402	15.4853	17.17798	22.21219	14.2322	15.01599	20.00066	13.93002	18.80952
Sipa1l3	25.89284	21.31703	25.34606	21.93983	19.69965	17.50846	28.58159	19.66701	18.80834
Scaf8	21.67238	22.27277	21.44734	18.50782	17.09752	16.05459	23.15202	19.81103	18.80157
Wdsub1	21.40574	19.08132	18.81965	20.17285	19.85361	22.15657	21.907	20.58017	18.80145
Nnt	19.11815	19.49198	21.70626	22.88284	21.4526	18.2284	23.50083	20.04141	18.80113
Thumpd3	19.66643	19.32251	19.72187	21.3269	19.13506	19.09624	19.33739	19.93892	18.79451
Trio	27.60431	22.29925	25.12292	23.88571	18.97344	16.35991	29.52806	19.33675	18.78378

Tmem126b	14.21489	15.53031	15.83206	17.73333	12.92274	16.49161	15.96063	14.51825	18.78253
Bambi	12.80388	11.92753	8.282619	12.92178	14.14017	12.21852	15.85198	16.21083	18.77221
Ppp2r5b	20.55385	22.74505	22.54225	20.07228	18.21045	16.12992	20.5875	18.71166	18.77015
Nipa2	21.52374	21.26082	21.32005	18.08199	21.38255	18.3411	21.37222	18.90276	18.76523
Dnajc9	20.1902	23.66202	22.87808	18.89411	19.77924	19.36279	21.14862	16.99007	18.75711
Gm10282	14.42403	14.98312	14.19527	17.82594	12.99118	19.56087	14.09826	11.71481	18.75442
Wdr48	24.02655	21.821	22.14298	19.13088	18.75104	17.56321	22.44834	17.11528	18.75098
Gtf2e2	18.06208	15.65969	16.16833	12.76825	16.24834	14.35487	14.78658	14.9153	18.74875
Pex6	20.183	18.75958	21.55885	21.14173	22.20224	22.15317	22.38737	20.06032	18.73913
Gtdc1	14.78348	14.93413	17.94802	14.81434	14.62394	12.68471	17.05157	15.6715	18.73821
Atp2b4	34.71705	37.48797	39.74589	16.75703	15.21694	14.79006	18.67467	20.21867	18.73772
Mfsd4a	19.74278	22.97192	20.28661	20.95757	19.3185	15.13755	22.23473	17.1315	18.73723
Ncoa5	22.41915	23.18229	24.59218	23.97004	20.9418	19.91981	23.07862	21.80628	18.73497
Ttc9c	18.2741	23.15821	24.43452	19.13958	19.42822	17.28506	22.56199	17.96609	18.72311
Eri1	19.92957	22.29287	20.29665	13.83369	15.79244	17.00882	19.66926	18.00755	18.72152
Rabggta	23.71797	22.24081	22.99974	22.60539	22.3141	19.55583	25.38299	22.49032	18.71181
Fam98b	20.55703	19.09763	20.3796	17.28322	16.55308	15.01996	21.27769	19.03022	18.7073
Orai2	17.96591	16.7982	17.9392	13.58505	17.19279	14.56414	17.82472	19.47647	18.70095
Snord47	17.05787	17.01245	7.676932	11.0255	8.935148	6.309832	11.4596	16.24076	18.69631
Ppp2r1b	25.67889	21.19996	23.11613	18.0033	17.4987	17.01381	20.25253	19.86612	18.67589
Cstf2	24.2793	22.56959	24.32235	18.74256	16.74382	15.38394	20.39526	16.85362	18.6733
3300002I08Rik	20.36151	21.92847	23.78638	28.92494	21.02167	19.8582	26.08474	16.86107	18.67248
Trim12c	12.00975	13.31351	13.65801	16.6365	14.82543	13.66453	13.02537	14.98813	18.66573
Atp11c	22.35381	20.73959	23.50678	16.01302	15.14915	13.03029	20.05352	18.74693	18.66546
Mrpl47	12.71515	16.42228	13.47963	15.74169	14.77392	16.462	13.93025	14.10357	18.66275
Gm22748	11.00609	11.78988	8.561284	17.83538	7.376276	3.769661	18.11628	13.19562	18.64849
Zfp362	20.56325	21.42937	25.99081	24.25458	23.79784	22.37834	31.37275	18.84565	18.64526
Tmem87b	19.0231	19.85768	18.94134	23.75899	18.44867	17.23702	29.40521	18.03192	18.64513
Stambpl1	22.84947	21.12827	22.26938	20.48658	19.8193	18.39776	25.89595	22.82485	18.64383
Fam122a	22.91396	22.23355	20.759	19.0801	21.13812	20.01121	21.18269	18.67304	18.63017
Cfh	24.68994	28.48758	27.93184	20.28289	28.13506	18.92136	23.92996	23.737	18.62927
Wbp4	18.74166	16.5455	18.56716	20.76066	15.69275	19.88616	20.05098	18.34848	18.62779
Usp22	21.25417	19.79105	22.70454	18.34275	18.23347	15.98499	25.26943	17.61	18.6275
Iars2	24.62011	20.57862	22.20895	17.99387	18.67232	16.25867	25.20468	17.94011	18.62667
Ift27	18.59135	18.31846	18.4815	18.1531	17.38621	19.31589	21.35653	18.82042	18.62652
Mob4	16.8742	19.17057	19.40457	17.13881	16.64408	15.69751	16.79746	19.27525	18.626
Rbpms	26.68584	24.2595	31.88502	22.01578	25.07793	22.11213	25.68952	26.41934	18.62532
Atrx	23.23268	22.71745	23.37069	20.96611	18.14243	16.28505	25.3042	18.12658	18.61997
Clns1a	23.04068	23.17477	20.83864	19.82642	17.79134	20.18984	22.46352	19.44945	18.61371
1600010M07Rik	11.06961	12.47626	15.48126	14.85579	17.91381	15.72977	12.18513	20.05048	18.61317
Arhgap19	16.48369	16.52843	18.68517	24.3109	23.34733	20.4823	32.14474	17.68229	18.61113
Irf2bp1	17.25013	18.49145	16.78088	15.87005	18.57512	18.92687	22.29667	16.77492	18.60829
Ppa2	18.65014	20.389	23.9303	17.73048	18.54295	17.48398	21.76477	15.56156	18.60541
Smad4	22.44448	21.70379	22.86064	19.5074	17.83155	16.43402	22.51171	18.51278	18.60536
LOC115488690	15.96616	23.88548	7.540453	18.08183	16.89555	9.433199	21.54405	19.00169	18.59876
Tmem39b	21.65205	18.51398	22.69474	16.86106	18.88023	17.63318	19.28392	19.38458	18.59372
Lpcat1	23.3673	24.9432	24.70786	18.67331	21.01301	21.15523	23.18872	18.50772	18.59153
Otud6b	24.16992	25.07338	27.06626	19.45993	19.18549	18.52938	20.7833	19.60736	18.58301
Heatr1	25.91091	19.37125	22.62457	16.64565	18.24887	15.63141	23.22869	18.5393	18.58198
Mrpl49	22.90112	21.20643	23.35351	21.14393	19.14944	19.87131	17.6276	18.59313	18.58187
Ccdc134	18.62463	19.21257	18.4815	16.20888	20.2284	16.60547	19.72997	19.58595	18.58171
Pip5k1a	19.59076	22.15754	20.64699	17.29929	17.57211	16.83884	20.89083	20.09977	18.57295
Ap5m1	20.04397	23.2802	22.05093	17.10593	19.62127	13.74951	19.3266	16.71646	18.57168
Snapc2	22.51942	21.87516	22.66853	19.9901	24.62075	22.72154	20.43654	22.62604	18.56638

Acyp1	16.12961	12.27353	15.77301	17.94021	15.93061	13.92175	15.55937	15.24321	18.56141
Pithd1	19.39251	17.50745	19.79131	13.44137	18.5722	18.36692	17.65881	16.54171	18.55149
Znhit2	21.78865	21.64302	18.18861	15.68208	19.78902	21.61192	19.7501	17.6081	18.54752
Ppp1r7	19.54237	16.97303	21.01087	16.38195	16.13611	17.66684	19.22951	16.8075	18.53919
Rxylt1	20.1238	21.91088	20.76507	17.71704	19.70652	17.16571	17.38749	20.106	18.53706
Mrpl46	19.88012	19.23614	18.85868	19.92948	19.8572	22.27067	18.43114	20.62056	18.53674
Ccdc88a	22.89081	24.16101	20.87512	17.76553	17.22176	14.25375	21.68931	17.69878	18.52183
Mkrn2	18.4876	19.96875	21.21247	20.03295	16.64099	17.5545	22.20298	18.63881	18.51668
Ulk2	21.16241	20.16442	22.87217	19.04285	20.18209	16.8804	25.75601	17.1341	18.51536
Treml4	12.84304	11.61808	17.58862	11.48758	12.13938	10.44446	10.27228	16.74415	18.51462
Ace	21.09746	19.07416	18.24667	28.63199	26.29867	26.07341	20.25816	27.01161	18.51323
Arid3a	16.69424	18.18947	17.62256	16.29369	17.91725	15.94044	19.81827	16.64467	18.50882
Whamm	20.61962	16.89431	19.31682	20.63389	18.29556	17.47532	21.01466	19.9798	18.50802
Nudt22	15.62607	13.40722	17.409	21.76538	20.40817	22.63203	15.55656	15.42451	18.50678
Pnpo	18.57012	20.7475	18.0004	18.19533	19.39752	18.48245	21.86667	16.87691	18.50347
Trp53i11	19.32929	13.94567	20.89452	28.79064	18.25234	33.77258	22.69817	15.42752	18.50093
Abhd17c	24.25106	19.73954	20.83504	15.37068	17.90623	16.31027	18.45928	15.77814	18.48601
Acsl4	24.06726	24.89014	23.85881	16.49294	16.72785	13.50819	16.99293	20.53298	18.47773
Pold2	25.41198	21.80711	24.69175	19.07207	21.28341	19.29692	22.45655	19.08037	18.47668
Zfp865	21.91908	22.57238	26.25837	22.79696	22.37406	23.4341	25.75574	21.49623	18.46693
2810004N23Rik	22.78496	18.56352	21.5038	21.93932	18.49097	18.73374	19.65214	20.70051	18.46633
Chuk	21.05787	19.93029	19.09314	19.78937	17.23747	15.85101	20.63685	18.16069	18.46316
Rrs1	19.84185	15.28047	18.30102	17.84646	15.95817	17.04741	18.4372	18.71097	18.44665
Ext1	27.49497	23.65487	24.55308	22.28018	17.3455	19.18087	21.98298	19.15345	18.44123
Dmtf1	20.71928	22.01803	20.22952	19.91138	16.5329	15.72388	25.22024	19.20978	18.43729
Ints6	16.30238	17.48545	14.49269	15.35176	15.4629	12.57096	18.49819	16.5499	18.43607
Gm12411	16.11572	14.14407	16.22504	15.49126	22.61336	23.24916	17.76682	15.95749	18.43165
Creld1	27.00195	28.58236	27.45022	20.70356	21.45385	22.52381	21.69759	18.2455	18.42946
Vgll4	18.64128	20.83234	22.93488	19.80485	21.9946	22.44235	23.84802	20.6582	18.42268
Napg	18.80993	18.41367	18.72946	17.86631	15.12804	14.32246	18.04016	15.7057	18.4159
Rpp30	22.63476	22.86705	23.02854	19.6884	15.50435	17.08913	20.21094	16.89598	18.40456
Cep41	19.20607	18.74078	23.56911	15.58849	20.07138	13.53689	24.78622	15.22191	18.40404
Lpin2	17.95586	17.20844	18.24067	18.87558	16.09385	14.28128	22.60539	15.98925	18.39081
Aqp1	36.11509	28.36642	21.81621	50.87312	32.80783	39.78795	16.878	24.74656	18.37823
Chka	15.1559	13.42298	12.77675	15.53977	12.95272	12.48989	22.43685	19.36704	18.37627
Gins2	15.43507	19.38895	20.72492	19.06626	14.0898	15.50863	16.44972	14.3906	18.37601
Ddhd1	16.29426	18.36768	20.29833	24.32646	19.81395	18.29883	33.79611	21.86026	18.37456
Gse1	20.46476	18.72169	22.58593	19.12411	18.42369	16.90178	24.05888	20.03006	18.37103
Txnrd2	19.79494	16.14164	17.47175	15.63772	21.3001	16.94766	20.5223	19.0215	18.37062
Thra	19.6976	19.26646	20.60181	24.63747	27.05476	22.69284	25.23957	23.20531	18.36642
Gpr84	21.58903	19.21442	15.22847	4.97605	11.8465	7.966112	5.319737	14.32716	18.36193
Kank3	23.57711	22.94943	27.47442	35.62519	22.79173	25.86286	22.68503	21.77361	18.35475
Supt16	22.24435	22.28362	20.41978	17.16061	17.85818	15.12565	19.69898	17.10053	18.35367
Rbbp5	22.82076	21.01297	22.92074	20.03171	18.81472	15.5992	23.51934	19.22591	18.34932
LOC115487892	11.5496	13.05469	11.55094	21.43848	11.36637	5.340353	29.44481	21.25961	18.34632
Klrd1	14.45098	14.22119	14.13291	11.76308	19.36558	17.26652	6.939205	17.168	18.34378
Rcor1	20.54602	20.67663	22.19149	18.55412	17.11833	15.17493	23.52968	17.4576	18.34256
Gm7367	21.72412	23.12649	22.67645	17.82496	15.30067	15.84495	19.7128	19.36487	18.32903
2310001H17Rik	18.15401	14.30933	9.468516	9.414384	20.35721	17.69086	9.583293	15.33294	18.32565
Ap1ar	21.24853	20.93523	17.38931	19.29114	13.81812	16.16578	22.37259	15.4181	18.31131
Cavin1	20.92446	24.90182	18.43448	32.0759	22.18974	26.61208	18.54045	21.75766	18.30711
Stam2	18.30079	17.61671	19.54821	15.47717	14.87848	13.76413	19.46845	17.65849	18.29783
Akr1e1	17.31428	19.9149	20.42352	16.44493	15.78102	18.19648	18.06242	16.39245	18.29106
Vma21	20.04757	19.51971	20.35388	15.21756	15.55639	12.88101	17.11428	15.89136	18.28842

Pcnx	22.27653	20.31543	21.4845	23.15909	15.52177	14.40116	24.72786	16.78411	18.28768
Creb5	23.84895	24.31329	27.49068	15.8541	16.44443	13.48981	22.56365	17.47868	18.28502
Serpinf1	17.81143	18.87426	21.0793	24.09523	23.11264	28.17304	13.9218	21.65706	18.28443
Pcf11	16.91606	16.73766	13.86113	15.71951	13.83644	12.64018	21.70888	15.94205	18.28006
Crnkl1	17.53489	14.89342	15.43393	14.10527	14.83317	15.55654	18.08635	16.32168	18.27484
Hmces	19.89708	16.91869	15.25457	15.55627	12.94357	14.28615	17.98773	17.91997	18.26478
Pikfyve	20.8807	21.0804	20.96846	21.46676	16.5431	13.93282	27.22537	17.30402	18.25144
Gbp7	13.59242	12.07629	18.52108	17.80529	20.08684	12.97237	18.19958	16.91601	18.24418
Eif1ax	19.48594	16.9565	17.50519	16.13599	16.59385	15.82808	16.65352	18.65017	18.23515
Rps13-ps2	19.75379	15.67623	16.67584	15.63003	16.89151	15.61593	17.89191	17.8975	18.21979
Nudcd2	15.47707	17.19578	17.79519	14.9517	14.25002	17.10046	15.92033	17.43082	18.21979
Mir27a	17.84214	13.34598	12.74586	3.801898	21.8477	15.91057	3.951587	9.100426	18.21979
Smarcc1	22.65211	20.18448	20.83689	17.18059	17.2831	15.87816	22.05867	18.55248	18.21787
Rrp1b	23.64149	18.62185	18.17943	16.27341	16.76271	13.63575	21.42645	19.60716	18.21438
Gm5431	13.86418	14.42279	24.27913	25.51341	22.48073	18.03245	21.9472	18.90291	18.21121
Paxx	11.71307	14.00692	14.89893	17.86697	17.59953	18.24593	17.51262	17.91897	18.20858
Snrnp25	15.10737	16.32828	18.30401	15.02275	19.07849	18.83292	16.37086	14.19361	18.20448
Gabpa	21.08695	21.48613	21.95321	19.04249	17.92074	14.85647	20.78695	19.13702	18.19262
Gm46162	20.12008	22.69819	26.00944	22.68176	22.71427	20.01422	29.02924	21.11299	18.18477
Tagap	13.63138	12.173	12.81536	14.27342	13.6161	14.48708	20.73025	18.4811	18.17418
Rbfa	14.82213	13.87423	15.40355	19.10319	17.19208	19.41192	18.48599	16.63495	18.15176
Entpd6	15.00617	16.01037	17.45106	23.39945	18.70742	16.78177	19.16804	17.56251	18.15098
Magohb	13.3816	12.68601	14.7803	12.72174	15.26538	17.20245	8.739086	18.06084	18.14972
Ranbp10	22.5973	24.93733	22.95788	20.78941	19.26467	16.8	28.52145	18.78877	18.14962
Gpkow	19.70959	20.01283	21.34032	17.68707	19.40626	15.57381	21.28441	18.0863	18.14946
Ppfia4	19.14244	21.79095	25.19464	21.72673	30.90271	27.08124	25.59873	23.90735	18.13566
Gm6560	17.20842	14.57681	14.592	14.00305	13.26622	13.75122	15.02726	13.23797	18.12122
Dnaja3	23.30545	17.76023	22.21285	19.31968	17.83596	18.33148	22.79695	18.93579	18.12045
Rmnd5a	19.57932	22.65373	25.14804	17.93832	19.47472	16.31605	25.04514	20.31706	18.11568
Mad2l1bp	16.2566	13.47488	20.18145	17.37904	18.9278	15.83969	16.48669	20.20838	18.11093
Chic2	13.26165	14.59235	14.26337	11.45788	13.45847	14.0734	13.18978	15.81404	18.10976
Mrpl44	18.80378	16.85117	19.15639	21.4858	21.13427	19.58529	18.38847	17.60651	18.1072
Ankrd28	23.87123	20.86872	18.06452	17.34989	10.8222	13.21385	21.1663	14.89616	18.10413
Wfs1	29.52341	33.74208	28.12673	24.64033	17.3625	19.70716	29.80654	22.02232	18.1017
Ubr5	25.4475	22.3063	24.30026	19.39099	17.70644	15.48892	22.88637	17.77363	18.10097
Scap	24.94579	24.46517	25.31046	20.43635	20.33856	18.79474	24.81344	20.38337	18.09343
Tpx2	28.49405	32.23528	34.65999	19.77131	17.97012	21.41303	22.34958	12.91777	18.09275
Sumf2	20.51733	18.48004	21.42004	19.43264	17.46855	20.22252	19.57746	14.99908	18.08912
Riok1	14.86039	15.74572	14.68114	13.09026	12.23566	15.56219	16.90186	16.25898	18.08673
Gpatch4	21.3484	15.58002	16.0301	13.41122	13.13511	20.61977	17.96692	18.16414	18.08615
Fhl1	15.90436	19.48763	15.73579	23.55346	18.12247	22.21937	11.97622	13.5201	18.08165
Nudt5	21.40816	18.32584	22.49164	14.666	16.57013	16.12182	20.72098	16.53187	18.0777
Rrp36	17.3991	17.03487	16.28533	18.64879	19.80896	19.9223	18.64176	19.42354	18.07662
Heca	16.82644	20.23447	18.41598	16.02562	15.62217	11.92557	19.0785	18.52496	18.06875
Immp1l	15.15114	18.29507	16.19792	14.25811	13.48991	17.28597	17.39939	17.07831	18.03664
Arhgef11	24.43178	23.79498	26.31327	20.06181	17.48337	16.60409	25.46578	17.76836	18.03098
Slc2a8	19.92024	20.69351	21.61579	20.48924	21.64777	21.48633	26.32209	17.76564	18.02445
Fam13b	19.0205	21.15785	18.56195	17.2778	17.42542	15.09416	24.53361	17.62897	18.02368
Xpr1	31.04126	32.04983	32.58117	19.92257	19.6882	16.2493	26.63822	20.30997	18.01762
Parg	20.32729	18.59346	22.92642	19.0504	17.82231	14.94758	22.4115	17.3269	18.01376
Cnot10	20.80466	19.60336	18.98082	17.48493	16.45315	14.42014	20.86648	15.08071	18.01008
Cnr2	18.81999	20.12879	24.31552	17.24676	19.01885	17.74539	20.65427	20.26977	17.99123
Golga4	22.6478	21.83162	22.39777	21.33134	17.02633	16.19585	26.68685	17.20506	17.9878
Wdr43	22.2395	19.66418	18.06366	16.25064	16.05384	16.0192	17.08975	19.70546	17.98003

Lhpp	26.43376	28.54253	28.70774	26.75432	24.52238	24.84131	27.36489	20.39508	17.96635
Mrps6	17.69754	22.32884	15.49603	11.09618	11.84584	15.18303	14.17758	15.56406	17.95117
Mrpl41	14.20843	13.48604	13.6609	17.24584	15.40595	17.24429	14.04664	15.92405	17.94683
Nck1	18.27904	23.44975	21.31133	19.39672	14.95245	18.5248	17.77946	17.80872	17.94658
AW112010	9.728486	10.5587	13.80747	25.60762	24.79831	25.20371	11.4596	25.40369	17.93063
Mprip	22.24428	23.01286	21.90818	22.05365	18.78211	18.13916	22.6543	18.53614	17.9289
Dkc1	22.34289	19.84786	19.24701	15.29508	13.85052	13.88702	17.66783	18.28821	17.92511
Bcl2a1a	15.73995	17.22618	14.48801	7.133336	18.30705	14.55582	15.04433	18.43408	17.92223
Lysmd3	16.41165	16.52704	15.49185	12.74207	16.40673	12.58739	17.01721	16.57046	17.92215
Myo1d	36.08296	40.53087	39.3799	16.40267	14.88623	13.59339	17.18596	18.42795	17.9083
Xpo7	25.09954	24.54583	25.70977	20.3413	17.38408	17.55159	24.51409	19.15859	17.90752
Gigyf2	20.8179	22.00822	21.33042	18.75537	15.74243	15.03561	23.90465	18.51251	17.90678
Slc25a51	20.31147	22.15107	23.06232	20.58276	19.84409	18.22892	25.4084	19.96885	17.89423
Ttc14	18.94794	20.06254	18.15808	21.02721	16.53728	13.67741	20.13616	17.68213	17.88923
Nsmf	23.05645	23.43684	22.94255	21.39727	19.47198	20.632	23.66133	19.28846	17.8865
Btbd2	18.67602	21.56849	23.05838	22.72346	23.27634	23.44072	26.05734	20.0932	17.88642
Polr2h	17.08133	18.63296	14.18525	13.34587	14.01669	13.32811	16.39338	20.03846	17.87895
Parp6	19.68202	18.77616	17.85697	19.70976	18.26034	17.28162	21.96782	19.99436	17.87698
Hmgn2-ps1	15.39422	16.76611	17.28508	18.26628	19.78823	18.82133	17.37486	17.71405	17.8675
Sema7a	10.23433	9.725855	10.61649	21.1621	21.28391	24.98786	27.87471	17.844	17.86257
Cpne3	17.7598	17.12537	21.64789	16.14797	15.70875	14.3095	17.32124	16.06829	17.86185
LOC115489586	21.8629	15.57478	17.9609	27.17554	12.64192	24.55058	29.85952	20.07221	17.86053
Mphosph6	24.01813	18.17907	18.53186	17.12408	16.68839	16.06471	16.86154	20.61441	17.85242
Suv39h1	24.59237	25.54636	24.7155	16.89103	17.92404	18.82214	22.9627	15.48553	17.85125
Tmem168	20.1593	21.58203	22.93969	20.70797	17.47281	15.78168	22.21093	20.2705	17.85026
H2az2	17.18981	22.31274	19.27235	18.50891	21.48093	20.31795	17.23844	16.74862	17.84436
Ceacam19	33.27884	36.70075	29.5224	12.48921	13.1806	11.98458	13.31166	17.59416	17.8412
Prrg2	15.90018	18.7411	19.04842	17.12166	17.45107	20.09021	16.16999	16.7981	17.82857
Gabpb2	21.88682	22.57719	22.83764	19.22048	17.14802	14.57736	26.03063	16.68201	17.81956
Cdpf1	20.84628	20.72581	15.44107	16.64838	17.7443	19.03701	16.36124	16.18828	17.81943
Ezh1	22.30964	22.0531	25.33024	21.63431	24.17165	19.91227	27.63434	22.00629	17.80923
Elavl1	20.23535	18.65939	18.95044	16.25485	15.9359	16.33245	18.89979	20.3427	17.80524
Zzef1	23.66541	20.68387	23.45967	20.69202	18.89131	14.78757	25.89583	17.36382	17.80135
Cog7	21.10349	18.65689	19.80022	19.24135	16.86957	16.90292	23.2213	18.20021	17.792
Cdc73	17.18353	16.44382	15.76349	15.6132	14.00279	13.69462	17.97891	15.5395	17.79027
Gm20900	11.84216	9.511215	11.42429	18.02847	14.57202	15.79694	12.02283	15.66512	17.77894
Cnot7	18.82593	19.34946	18.629	13.5069	15.78059	13.80905	19.36609	16.68145	17.77411
Stat5a	23.32211	21.29741	22.89431	17.68789	21.17215	18.18386	22.6745	19.32137	17.77365
Rbbp8	19.46443	17.24757	18.66395	18.15816	17.98176	14.18752	19.28788	17.90679	17.76894
Runx2	16.85962	16.28499	18.25852	15.06323	14.63297	11.75257	19.25522	15.11549	17.75622
Rfx1	19.97205	21.54897	21.3565	17.13816	19.78049	17.97799	26.19063	20.71067	17.756
Cog2	19.83996	17.00794	18.42917	17.97255	17.34411	18.60144	21.43858	18.27301	17.74963
Atp7a	21.11314	19.14757	21.88688	17.90047	17.3182	12.89974	26.14942	18.1953	17.73712
Smg1	22.81612	19.79764	20.53725	20.98695	15.70753	11.65243	23.53739	15.47535	17.73363
Heatr5a	26.944	26.21685	26.93999	22.87924	19.967	16.8249	28.63055	20.16493	17.70778
Nfs1	17.43922	16.37482	18.72455	17.34154	16.7478	15.93847	17.54205	16.18736	17.70511
Hipk3	22.53327	22.42901	23.87502	17.42689	16.17635	13.79171	22.28765	18.55204	17.70412
Map3k14	20.63151	20.2586	24.79258	17.4511	20.68673	18.7231	21.69443	17.82183	17.7011
Pbrm1	24.32406	21.10432	20.63582	16.67074	16.35325	14.02754	20.78811	15.77165	17.69622
Gm5617	11.64812	13.80078	11.4743	17.50495	22.85437	14.33543	14.39239	19.76216	17.69594
Luzp1	28.33016	25.91832	26.77635	20.51728	16.28303	16.55089	26.25783	18.45308	17.69567
Golt1b	20.56126	21.75233	21.1982	17.46888	17.84502	15.31054	18.66899	16.38597	17.68806
Kif21b	16.32069	14.53065	17.58753	18.88376	18.2192	15.77891	24.87501	16.62732	17.68781
Btd	17.95928	20.26214	19.38898	18.02373	19.03279	17.77474	20.10577	19.97516	17.68629

Plpp2	19.23135	20.24981	20.56514	13.38654	14.33558	16.2797	19.76189	17.95801	17.65946
Kif13b	22.43358	22.6984	22.49536	26.62242	20.10705	17.14384	31.92055	18.84283	17.65946
Cmc1	14.74615	12.10061	14.79454	17.03014	19.33282	14.80195	13.11874	17.8163	17.65694
Ly6i	7.408204	3.201675	5.30983	10.19061	9.230936	7.3076	3.126506	8.816671	17.65169
Pmm1	14.93411	17.09278	15.48698	19.12476	15.69263	13.1481	16.74514	18.67849	17.64748
Hdhd2	23.52653	23.46389	22.00454	19.43245	18.14952	18.00767	20.23336	18.50685	17.63448
Gm10698	19.677	17.35701	18.05105	13.76095	14.48954	15.8864	18.59647	15.60758	17.6303
Pccb	19.38121	19.66691	18.44681	21.83371	21.33252	18.78922	23.36554	15.95115	17.629
Cdc40	18.79228	18.3973	22.25357	17.58971	17.44268	13.86017	21.87402	19.7111	17.62816
B230219D22Rik	22.3802	23.56857	25.79147	19.28041	18.47724	16.46463	21.00024	18.58506	17.62761
Atpsckmt	18.68538	17.63984	15.16878	16.92595	19.19949	17.63141	16.95353	15.70799	17.62757
Arrdc3	14.49331	17.5315	19.71869	18.67957	16.75592	13.54419	22.77714	18.94643	17.62702
Zfp800	18.94169	17.46603	18.41144	17.52601	17.39939	12.70996	19.05589	17.43409	17.62582
Zfp276	14.17807	15.45148	15.37316	17.57806	15.6797	17.07666	18.75093	14.38472	17.61889
Cdk5rap3	22.84671	21.01637	20.33784	24.18445	20.58912	20.14346	27.84497	20.08904	17.61247
1110032A03Rik	20.19173	27.39618	27.90065	14.75743	22.83183	18.67268	21.60658	21.1945	17.61247
Oard1	14.6055	17.69922	19.10977	21.81155	19.39439	14.03923	18.99112	18.8402	17.61247
Lpcat4	17.37176	14.33134	17.54868	11.62257	14.62927	14.44759	12.74293	14.37504	17.61247
Gm10154	12.14727	10.43946	9.434613	8.995167	12.79966	13.86251	10.55138	14.64129	17.61247
Prtn3	2.224367	1.42208	3.365459	4.480529	5.757872	13.13331	2.652685	4.887266	17.61247
Hltf	17.73834	21.23851	24.21097	20.96249	19.05481	17.93508	22.35826	19.03439	17.6076
Zcchc7	17.78244	16.57178	14.90112	17.70471	15.78299	17.17732	18.37903	17.83163	17.59848
Nedd4	11.70142	13.76816	17.45071	22.41449	16.5413	15.8171	13.73346	13.26402	17.59421
Vps13d	23.63889	21.08918	21.74707	21.51004	17.19861	14.46886	26.91422	16.78228	17.59189
D6Wsu163e	21.47668	18.28193	22.51969	19.50894	19.34533	17.43274	22.0692	16.39134	17.58886
Zbtb17	20.46497	18.29906	17.02153	22.47618	21.52665	18.61826	21.43044	17.92351	17.58609
Surf2	19.16972	20.1755	21.77317	21.40921	20.32127	20.40005	21.40586	23.93784	17.58186
Dnajc21	20.60362	15.75496	18.28345	16.08446	14.1705	17.58024	17.02474	19.77093	17.57815
Gm10131	13.67373	14.37849	16.64822	13.96662	11.04099	17.18076	14.20929	17.83	17.56525
Akap7	18.1561	19.28987	19.88243	16.39361	18.16395	18.43434	20.38005	18.51746	17.56112
Gm6180	24.02316	19.52445	17.15314	20.95994	17.32454	19.38548	20.23336	22.59749	17.55743
Uqcc3	17.61209	18.36362	21.88423	16.05398	21.41171	21.65445	21.51261	18.35636	17.54888
Dph6	23.12979	19.72044	20.71956	15.48995	14.03634	14.1628	20.27384	17.67738	17.54582
Alkbh7	13.11672	14.96095	10.72652	19.67296	16.01213	15.07864	16.77745	20.97499	17.5434
Mfhas1	22.47294	20.62258	21.14921	19.06363	15.83529	15.11741	25.3342	17.4195	17.53221
E2f2	21.78126	20.11205	21.78379	14.35965	14.94071	15.45098	15.49624	16.46079	17.53143
Ptcd1	17.68454	17.41098	17.4027	18.71457	15.68008	14.46407	17.95514	16.405	17.5313
Thbs4	21.33032	16.64585	18.26794	23.29405	20.67753	30.74122	14.74502	18.61068	17.52446
Lifr	19.98222	24.12	25.01898	21.54609	26.63901	20.09811	36.57753	25.44311	17.52207
Irs2	19.99165	16.59365	15.28519	17.67262	14.34593	12.57915	28.55623	16.36148	17.52173
Fem1b	18.11807	16.6314	15.09019	18.35604	14.22376	13.67709	24.26521	16.95767	17.52028
Ugcg	15.95018	15.68471	17.57706	14.70463	13.44455	12.80651	18.08762	16.27897	17.51775
Nup54	16.9605	16.33467	17.87389	16.46274	13.28403	14.46723	19.67493	17.06392	17.51596
Alkbh5	19.68166	19.11536	18.15635	16.23174	15.16043	15.19727	21.99673	18.00298	17.51308
Car13	17.02448	17.5804	16.39942	13.45217	13.77954	13.04542	15.80119	13.77533	17.51287
Gm46367	18.87253	16.80561	15.33571	25.97193	10.59182	6.077289	29.43271	11.55118	17.5054
Letm1	21.15414	19.23941	20.23019	18.10094	16.52585	17.63032	18.70228	17.5463	17.50297
Rabep2	16.50696	15.48307	17.42604	18.45545	17.67578	20.091	20.96489	19.99871	17.50148
Tecpr1	20.26001	18.8714	20.39401	29.72349	20.90424	20.55159	27.96877	20.61587	17.50095
Anks3	16.87128	17.36395	19.94601	24.87037	20.25187	18.7673	24.93034	18.19289	17.4926
Notch1	21.5758	19.93347	21.51403	18.32356	19.43572	14.91179	23.17523	17.66091	17.48996
Tm7sf3	21.05007	23.18633	22.71064	19.70957	18.75273	15.03912	21.98734	18.16171	17.48818
Gcnt1	21.08432	17.7593	19.50893	18.88662	16.23676	13.35689	17.87324	15.67289	17.48425
Slc35e1	20.51834	18.3581	18.73841	15.09501	14.72489	13.41445	17.69717	15.86684	17.4824

Mpi	19.81508	15.23084	17.38755	18.73144	20.88505	16.36938	19.76243	14.29963	17.47213
Utp18	18.52179	17.22405	17.14472	16.94122	16.79129	15.12712	15.37157	20.66455	17.47092
Csnk2a1	22.08294	23.0366	23.39086	19.04882	16.81268	17.55649	21.57021	20.04604	17.4533
Zfp516	21.06738	17.1122	21.25296	16.47908	17.46583	13.8858	21.73634	16.51032	17.44376
Nsd2	23.6229	20.11435	20.46701	16.10145	15.31434	14.33648	20.51328	16.13391	17.44184
Lrrk1	22.57364	21.20618	26.79835	19.43987	19.75893	16.11233	24.20577	19.50721	17.43211
Bmi1	17.8937	17.52283	16.61629	13.85821	13.43521	12.9667	15.5498	16.6143	17.42821
Cdc14b	19.74373	20.33784	19.29209	20.92267	15.40731	14.96048	22.0145	16.35948	17.42707
Fam53c	16.3626	15.94345	18.9101	17.04576	15.06411	11.90114	16.96526	14.67622	17.42681
Kat8	17.88669	18.33558	19.13351	16.58423	15.91748	17.85644	18.81756	16.79288	17.42041
Inpp5b	19.93632	20.1655	23.13071	18.66769	17.02553	16.85164	21.51519	17.4245	17.41267
Vamp7	12.98448	12.90486	13.257	13.98959	12.34477	13.40679	11.10951	14.51249	17.41159
P2ry12	18.26803	16.0415	22.70853	18.14235	27.23791	17.78398	26.11294	17.2141	17.41105
Arhgap24	15.90006	12.1905	16.85157	16.03198	14.662	14.94676	20.44269	17.71598	17.40707
Rnf180	19.86642	23.99714	27.05101	21.12332	19.59173	21.13383	27.12291	17.02969	17.39924
Racgap1	29.47942	34.25997	34.94546	20.68465	22.71083	21.8762	26.39682	17.79347	17.3948
Pdf	19.33299	23.09465	19.47953	20.33657	17.14151	16.51097	18.19221	18.09783	17.39475
Ncoa7	17.56075	15.24701	18.99115	15.00205	13.77438	13.87717	16.96487	17.02599	17.3888
Ccnb2	28.74433	35.16201	40.22515	21.82206	21.07147	23.2217	21.34267	18.94141	17.38388
Ctu2	17.59023	14.23604	13.9152	12.61696	13.69743	14.80021	14.56666	17.53696	17.38344
Coq5	18.06378	17.10385	16.09858	14.10559	15.0137	16.26926	16.1786	14.42879	17.37482
Ormdl1	16.0368	18.15061	17.90083	14.2149	18.27644	16.69106	14.06067	19.75768	17.37394
2510009E07Rik	26.91387	25.23618	26.63784	18.9274	17.43254	16.85748	26.13029	18.76619	17.37372
Prkar2a	26.89967	25.16033	23.6807	17.73659	18.66478	15.65959	20.62406	16.51509	17.37164
Ddx60	10.10768	5.450451	10.5372	16.40538	13.85229	9.996823	12.60698	9.460393	17.35666
Iars	26.44532	25.95986	24.72048	18.45944	17.53548	16.99194	24.2174	17.87832	17.35605
Polr3d	23.21852	17.75008	18.95888	16.40942	15.84608	16.80536	17.30217	18.11354	17.3525
Lamc1	26.56983	25.08455	25.61999	20.79397	15.21891	15.42326	19.04922	17.35409	17.35135
Stk3	19.90337	19.65403	19.05905	14.58452	15.05585	15.96644	17.62076	17.75035	17.33727
Rars2	20.49196	14.9631	19.03046	17.3206	13.03411	12.25003	15.58203	12.29849	17.33346
Rnf145	20.35978	21.99506	24.50171	17.16279	21.61257	19.46358	22.00085	18.13684	17.33323
Icam2	11.85841	8.328066	11.21738	20.8325	15.76429	14.62171	13.07334	19.00545	17.32991
Wdr83	14.64317	18.44504	18.45164	19.81563	17.22992	15.48789	17.03204	19.35641	17.32794
Saa3	14.92965	10.93314	14.61814	18.6873	13.12508	22.01311	8.632468	25.84452	17.31395
Wdr13	19.00018	21.46346	24.8402	20.65267	18.76941	16.53479	24.66511	18.98822	17.30808
Gm11425	20.08905	16.37523	17.48337	17.95966	13.98151	15.6303	21.4618	18.20719	17.30563
Cebpz	18.47935	18.90463	18.21387	14.81303	14.72527	13.45958	18.76129	17.31106	17.30074
Zfp771	15.21387	15.08331	15.21563	22.40117	16.76829	19.91412	17.12408	17.4079	17.29693
Cog4	20.20958	21.40755	23.59476	17.09249	22.87399	20.7745	21.83305	18.16803	17.29482
Zdhhc12	23.77292	23.37366	30.56186	26.60669	24.33686	20.73837	23.27731	21.30542	17.29141
Zeb2os	9.111243	10.12679	10.019	11.08861	14.28973	10.56368	11.71259	13.07693	17.28124
Fam53a	17.52052	21.06718	18.4815	17.36459	15.82851	19.15809	19.82006	18.50706	17.28016
Sesn2	19.08421	16.53722	16.37469	11.07528	13.66646	13.50157	15.08977	15.98566	17.27453
Gins1	16.64858	16.86103	18.10958	19.45677	10.70684	16.1897	17.65062	15.60596	17.25804
Cdkn2c	22.30555	28.995	25.91755	16.04979	20.09784	19.7931	21.34428	13.50582	17.2462
Cryzl1	16.81865	16.58398	16.28107	16.12488	13.3281	12.79484	13.15197	15.43895	17.23945
Ccdc71	19.42608	18.84779	21.17634	19.78798	19.43752	19.31156	19.32514	16.65811	17.23072
2300009A05Rik	14.83293	16.18032	16.13268	12.90611	17.5701	17.7464	15.90547	16.24076	17.22959
Sfxn2	21.51153	16.35352	19.32749	15.95592	13.90611	14.08048	15.53502	16.54099	17.22797
Recql5	16.18814	19.67065	19.4168	21.20289	19.07804	17.01994	21.86371	16.21405	17.22029
Pank4	17.86847	18.82183	16.10313	18.89653	15.72945	17.12948	23.72196	16.26473	17.21738
Kansl3	21.61043	22.53149	22.51049	17.13439	17.55015	19.033	23.95559	20.30183	17.2095
Smurf2	18.68022	19.99743	19.61592	18.38759	15.73155	15.70146	22.29884	16.84044	17.20706
F11r	18.04752	18.3559	24.48315	23.67685	27.80329	25.46845	25.00151	24.07323	17.20089

Col6a1	20.81716	21.23454	23.14836	28.79775	16.93114	24.0477	13.51749	20.48374	17.19607
Ints8	17.2174	17.08948	18.2236	15.12406	12.74503	12.80162	15.05604	15.73444	17.19412
Hacd3	17.18113	20.8983	17.81203	15.67292	15.30058	14.06899	18.61713	15.06415	17.18714
Zmynd15	19.70793	23.99971	27.99643	20.5971	25.03582	21.0985	25.81558	16.70478	17.18667
Yeats2	21.28236	22.80408	21.23758	20.24709	16.21977	17.759	26.90345	15.94494	17.17722
Rnf185	17.06933	16.11322	18.18953	15.08363	17.01979	16.11261	19.20205	16.16297	17.17437
Gm10052	19.37226	16.14575	16.57899	15.13305	15.75084	12.85275	19.42033	18.85088	17.16845
Zbtb45	20.76398	16.82424	19.32464	15.86928	17.174	17.19048	18.42842	16.90387	17.16251
Ddx10	15.91704	15.30009	15.07933	13.32358	13.40345	12.96907	14.98799	16.15758	17.14705
Mis12	19.1025	18.83733	20.28667	18.39721	18.41812	17.04939	18.34424	16.28482	17.14144
Hccs	15.06162	14.76293	14.2383	13.68813	14.95063	13.10021	16.29777	16.27011	17.1407
Zfyve26	22.34114	21.78715	23.45949	21.1161	19.55201	14.18803	26.43159	15.97818	17.13989
Med17	16.07281	14.77904	15.33075	16.40338	15.07142	15.68639	18.46621	17.53861	17.13983
Snhg15	8.989956	10.83394	8.52272	8.287753	14.71583	12.29724	21.00282	16.94032	17.13645
Yrdc	19.15717	18.82153	18.41014	14.50911	14.74556	14.7468	18.17755	17.05071	17.13645
Phpt1	18.82802	17.63984	18.4498	17.02051	17.84104	21.63255	25.94627	14.30468	17.12911
Atat1	15.51329	20.60986	21.77968	22.12087	20.35004	16.45753	26.25906	17.16684	17.12891
Ecpas	18.16883	18.14645	18.93322	16.33096	15.18813	13.03674	19.08811	15.27032	17.11783
Nsmce3	15.41986	16.88414	17.66554	17.8485	18.88162	16.74961	19.94257	16.07943	17.10703
Tmem33	20.11586	20.89359	20.99247	15.54568	16.53466	15.12454	17.82815	17.98127	17.09901
Prkar2b	16.98279	15.15296	16.17131	11.74372	15.43058	15.07333	14.66076	15.45684	17.08533
Ttc7b	19.65318	18.48033	19.99088	19.19292	14.78872	14.82505	20.36454	17.49836	17.08505
Tyw1	20.20996	18.37165	22.11913	18.90087	15.86752	19.31343	22.07034	14.88521	17.0813
Lrsam1	19.7704	20.15018	22.02748	22.21771	16.79205	18.01575	25.48238	16.67684	17.07987
Fance	17.04836	17.98865	18.34765	19.62478	20.79588	21.79221	21.67429	19.55451	17.07281
Ankrd10	17.88247	19.91036	18.46537	21.22325	17.79159	16.42526	21.9088	18.75379	17.06664
Rsbn1	16.9105	17.31365	13.86113	14.11114	12.1635	12.37941	19.4823	14.09634	17.06396
Cbr1	16.76989	19.6918	18.0028	17.23691	19.39042	21.29422	18.07458	18.31029	17.05851
Zfp644	19.10797	19.83155	20.52473	18.11845	15.90033	15.02886	21.46884	14.97132	17.04859
AW549877	16.64889	18.54267	16.18132	17.35898	15.65866	13.49832	18.97898	18.741	17.03325
Rassf1	23.79577	20.21887	17.71277	15.25481	14.57986	15.46018	16.78226	20.00306	17.03251
Zfp277	16.58335	19.30905	19.68333	18.32273	21.08384	17.68181	17.44332	18.76371	17.02708
Lrrc45	16.53692	19.09586	16.62885	20.76231	17.39954	18.58027	25.60272	18.63667	17.00489
Chst14	20.19548	19.26829	22.53345	18.7365	17.28781	17.13003	22.60103	17.66153	16.997
Kdm6a	16.84313	14.16363	15.44914	15.81726	13.74834	8.888195	18.20214	12.79872	16.99046
C330007P06Rik	16.69781	17.69626	17.97796	16.53826	17.98946	14.01411	20.34594	16.89355	16.99023
Smpd4	24.84994	21.72846	22.33333	19.9325	16.97257	16.19273	21.93879	16.73049	16.98504
Fam98c	18.14767	16.87068	14.54826	18.65855	20.35228	22.69787	21.98872	18.54153	16.98022
Blcap	17.85745	19.13901	18.68586	14.17944	16.42341	14.02459	16.74865	18.24548	16.96893
Fam117b	18.86337	17.46436	19.50677	15.08187	15.00763	13.34953	22.00185	16.86292	16.9473
Sipa1l2	19.04374	15.58163	16.36909	16.93026	15.98699	14.24226	22.52855	14.58988	16.94415
Pdcd2	17.57189	16.04411	19.50459	16.8828	18.62094	19.77456	15.14304	18.00259	16.93627
Rpl36-ps12	14.78348	14.74413	17.48634	15.54879	14.07963	19.98113	12.19419	20.57163	16.93506
Cd244a	18.66392	15.02483	18.15848	11.79174	14.61135	15.95839	13.06671	14.98029	16.93084
Zfp768	17.27042	18.65161	20.62822	20.09321	19.40883	18.59498	20.09383	17.94651	16.93054
Riox2	19.60141	17.43161	19.96624	18.10119	16.59667	14.7265	16.56584	15.28497	16.92968
Sertad3	10.84945	13.98757	19.89298	14.34105	15.16332	15.90635	15.99968	18.85388	16.89788
Rrm2	27.99876	26.30102	24.74656	21.09007	14.24109	17.144	17.37339	10.87125	16.89343
Cxcl12	16.59009	12.92466	13.7578	27.97472	14.31016	23.09489	8.207222	15.26138	16.88096
Maml1	18.3895	16.0206	17.03373	17.00584	15.62185	13.10074	21.04951	16.04481	16.8798
Gpn2	18.82396	15.80955	17.27525	22.35942	16.32777	15.89408	18.69865	19.1116	16.87348
Trak2	20.89214	19.88517	21.68022	15.53949	14.21501	12.98116	19.86292	14.54901	16.87267
C1galt1	17.55762	18.66221	15.2506	15.11001	13.09099	9.657633	14.37306	14.87622	16.86191
Nol6	23.68144	20.17738	21.55694	18.75295	16.92429	15.81593	23.72186	17.31943	16.85422

Bnip3	16.6693	16.05817	15.53457	12.77728	16.41688	16.23273	14.42239	15.99104	16.8502
Slc16a6	20.87662	20.37094	21.40321	19.09485	18.48228	15.15942	24.55629	15.77375	16.83082
Fam76a	20.45906	23.09531	18.96243	15.70548	14.88139	16.25171	20.58391	19.72876	16.82083
Gmnn	20.65451	21.78795	17.98967	11.39791	12.91593	22.51147	13.78202	15.72129	16.81927
Nif3l1	18.68655	16.85449	18.72758	14.75733	16.72387	14.90958	16.06111	15.16525	16.81022
Tmem167b	20.88606	21.33239	21.99195	18.34376	17.84534	15.75255	20.06631	18.06104	16.80687
Pdcl	15.5568	16.67613	16.45099	15.3925	13.00567	13.29221	15.61762	13.59269	16.80518
Psip1	17.36	21.94873	18.98127	15.89128	13.24962	14.19627	15.58729	12.30354	16.80281
Unk	16.71295	15.51135	19.33345	20.75077	16.72354	15.61509	24.43591	17.92753	16.7956
Hps5	17.67245	17.55035	17.26055	17.74807	18.02559	15.21783	21.24693	17.4827	16.7956
Nqo2	20.0543	20.00091	20.07413	18.08002	16.61623	18.43231	19.85897	17.22761	16.79522
Mrgbp	14.59232	17.06378	14.75334	20.15006	20.51256	17.23645	16.49787	16.92679	16.79258
Tapbpl	15.16551	16.64031	21.32481	17.49239	19.67256	17.1795	17.6577	18.753	16.79112
Herc6	12.83298	10.36893	16.83606	15.41357	14.24232	10.65359	12.59058	11.87831	16.78506
Slc30a9	15.78744	19.09584	19.51316	15.53444	15.47708	14.81398	19.8063	16.90615	16.78179
Cdk8	20.23158	18.61372	18.90856	18.44905	15.58716	15.91127	19.42653	16.21326	16.77745
Cwc25	15.42275	16.48515	14.97186	14.05277	16.42872	13.40982	17.46489	15.65758	16.77631
Mettl3	17.20873	18.18222	21.8242	16.59045	18.30983	14.84123	19.95581	17.01557	16.77002
Fbxl19	17.82059	17.2804	18.28272	16.33157	19.74674	15.97576	23.94754	16.23702	16.75728
Tbccd1	20.31418	22.7436	23.89925	22.89378	20.09592	16.60433	24.02091	18.75878	16.73809
Pex2	16.6148	16.21154	17.55835	16.10869	14.68972	14.95512	18.86083	17.37445	16.73272
Cwc22	17.99393	17.50832	17.07826	17.27818	14.53434	13.70697	13.73173	14.18802	16.73153
Taf13	16.12822	15.87081	14.92571	16.12341	15.11457	14.4376	15.51358	15.68323	16.72433
Tbpl1	16.17615	18.22255	17.12494	13.22099	14.38915	14.49347	17.23937	14.53819	16.72288
Hyal2	21.82743	20.24427	18.29159	20.12498	17.36157	19.71396	18.43824	16.44278	16.71708
Knop1	22.81234	23.92701	22.35986	17.92718	16.78087	17.50278	20.3335	17.26457	16.71535
Osbpl2	17.79469	18.51551	19.41644	15.43649	18.05724	15.41492	17.37107	16.35937	16.71522
Tnpo3	22.85276	21.87802	20.59209	15.68366	15.04754	12.85215	19.85986	15.73031	16.71266
Tia1	19.05288	19.88539	20.67465	22.79577	18.29034	16.80054	21.88883	19.87473	16.71215
E4f1	15.02879	15.82886	15.9173	17.98643	15.91769	18.34639	18.78623	19.79606	16.71107
Irf4	29.18628	26.1028	23.16137	20.7024	25.07745	19.17943	27.70633	18.84009	16.70539
Zfp330	17.59771	17.19914	16.78854	21.11118	14.879	17.67616	19.82832	17.86277	16.70495
Il1a	20.38907	15.5732	9.325013	4.384509	21.82556	7.947224	16.7482	26.79232	16.7024
Slc39a14	30.74456	23.43361	26.93681	11.73988	16.14857	12.89424	13.34647	20.47067	16.70035
Nup188	25.0562	19.92299	21.1381	20.49939	15.65616	14.36614	21.5096	18.56087	16.69782
Mrpl45	20.88904	19.12764	19.50023	17.71065	16.20229	19.31753	19.63358	19.41075	16.69236
Tut4	18.31244	19.8475	21.53393	19.76055	18.85762	14.87085	24.14649	17.53059	16.6898
Fbxo42	18.70796	18.50117	17.12472	15.95355	13.90902	12.78825	19.98632	15.09294	16.6838
Ccdc130	16.63464	16.46199	17.08708	20.22089	17.65061	16.03779	22.11444	16.45049	16.68227
Dele1	18.72534	19.73345	20.2497	17.70318	16.97726	17.18865	23.47693	16.40121	16.68202
Ago2	26.98386	23.25628	22.53404	15.50655	12.77396	8.777569	18.87103	13.48644	16.67822
Slc11a2	24.74159	22.18516	22.63119	18.28033	16.90433	17.28453	24.45986	18.78295	16.66044
P2ry10b	19.24959	22.93393	18.00316	17.10343	14.32218	12.20078	13.63596	19.17911	16.65932
Med28	17.29405	15.08142	16.20368	15.04904	15.17613	14.74723	14.28687	15.04439	16.65627
Ascc3	19.01079	15.49369	17.91726	15.8162	13.69439	11.0822	17.60252	15.09304	16.64266
Gm10257	11.30187	11.00467	13.60667	12.30382	12.54923	10.60021	11.84712	15.96223	16.6425
Csrp2	13.46977	17.91187	21.20487	13.64236	20.84624	22.60609	13.59941	22.7398	16.6417
Ap4e1	19.99451	18.05006	19.71794	14.78017	15.51025	13.61629	17.92724	16.88614	16.63793
Smarcal1	20.12267	21.06395	23.29053	18.40763	15.60208	16.71068	23.43469	17.18626	16.63738
Hint2	19.6988	22.26588	20.63924	19.30863	22.2451	25.85056	23.36518	24.64968	16.62903
Ago3	24.58381	22.53188	21.4142	24.59282	17.81031	14.34327	30.7648	15.68022	16.62814
Pex13	12.9377	14.55918	13.24112	13.40128	13.82087	14.53673	18.3416	16.79349	16.626
Rfc3	16.1123	18.12082	17.33865	16.65514	16.3713	15.76064	14.78005	15.01409	16.61671
Fam118b	20.32853	21.46265	19.80959	15.8089	17.90685	19.72882	21.3184	16.34165	16.60705

Atad3a	22.94942	18.79784	18.36628	18.07798	16.56814	17.43006	21.33791	18.12066	16.60227
Siae	21.85555	23.89576	25.29992	18.39206	19.7121	16.89305	21.49307	16.6469	16.60204
Aggf1	18.75762	18.72468	17.99305	17.365	13.9866	14.82563	17.97826	15.31243	16.59491
Zhx1	20.87208	20.7407	24.10017	17.8395	18.70963	16.51001	19.6755	17.701	16.59125
Pold1	23.34746	22.17109	19.75534	19.12719	19.10557	21.56105	23.10997	16.94157	16.58884
Nlrx1	18.25249	16.21381	18.31649	15.65843	15.10005	13.40333	17.28213	15.69925	16.57066
Nme3	17.61154	22.29947	18.53256	22.38604	24.13859	22.80124	18.75639	21.87119	16.56642
Thyn1	12.89583	14.46919	13.80856	16.30827	13.61683	14.19413	13.61824	16.28182	16.56596
Nop14	22.97951	17.20921	21.93567	19.09673	17.07845	15.1032	20.2091	19.61273	16.56302
Mpst	22.18381	17.75971	18.40998	24.87566	21.78147	22.61796	21.59762	20.22239	16.56281
Trub2	18.32701	16.04472	16.72934	21.17842	16.01804	14.79273	15.06848	16.1872	16.55252
Unc119b	17.88079	19.59374	17.41107	14.54229	14.98593	16.08527	15.8698	16.18392	16.54885
Pdpr	18.37165	21.59137	22.62099	18.08513	15.84837	12.52871	20.41927	15.22825	16.54193
Ero1a	17.21617	16.3819	17.00482	11.21767	14.00633	12.10319	14.96648	16.2959	16.54009
Hbs1l	20.95622	18.84187	18.96722	18.49658	17.09074	16.97685	18.74797	15.65209	16.52471
3110040N11Rik	13.62763	14.0183	17.26843	12.48706	16.2405	15.4396	14.30607	16.64312	16.52444
Prrc1	19.24917	16.77947	17.727	17.64862	16.12221	14.33052	19.04762	16.43612	16.51925
Cryzl2	16.5	21.456	21.32107	20.37637	22.29686	20.07485	26.96377	15.47791	16.51459
Limk1	24.22519	22.8451	25.54726	21.32425	20.74595	21.8587	26.25391	19.85707	16.51381
LOC115487643	17.32439	20.73393	18.01946	23.4292	16.49956	12.176	35.81125	14.51518	16.51169
Tubgcp2	21.85723	19.48731	20.69111	17.56309	15.28861	16.95331	21.02643	15.35689	16.50484
S1pr1	18.0462	19.45845	20.61093	20.8207	14.81638	16.33312	20.60004	14.82928	16.50187
Mrps14	16.4801	17.06733	16.06928	14.22646	17.55586	16.89407	15.3553	18.022	16.49366
Gtf3a	17.08415	15.46259	19.81992	15.5869	15.74196	12.99458	15.84745	13.62259	16.48625
Ap4m1	17.29616	19.21328	19.33135	17.96939	20.65231	16.59177	23.37945	18.28031	16.48478
Rybp	16.24122	12.93817	12.56877	12.63549	10.70209	10.16912	12.28188	14.72747	16.4815
Ptgr2	16.37943	16.50214	17.30788	16.78515	15.59959	15.19859	18.844	17.99402	16.47637
Gtf3c1	21.30681	18.25576	20.76848	18.48285	16.84113	15.86759	23.3563	15.60652	16.4689
Vps36	18.69334	17.68319	18.78723	16.39994	15.95458	17.56972	17.48798	17.89976	16.44704
Mcm4	26.352	23.00047	21.56604	18.4936	14.54941	15.28451	19.97208	14.47143	16.44452
Bbc3	13.51806	17.56861	16.88203	20.92965	17.24376	18.42776	18.89423	23.77378	16.42198
LOC118568007	13.82865	13.17613	17.53479	26.33598	23.22433	24.09225	16.97246	18.86768	16.41607
Gm9118	18.58465	14.5971	13.95327	14.7635	14.94206	18.93923	15.56242	12.73195	16.40819
Rbl2	20.58699	22.01935	23.36392	17.80495	19.57379	16.60097	23.30868	16.9485	16.40427
Clec16a	25.16074	22.02861	20.35048	18.68929	19.33108	14.36674	24.29449	16.27044	16.40003
Serpinb10	15.34365	9.078446	14.49291	8.839779	10.49593	10.05077	5.443468	18.63763	16.39381
Smim4	12.65584	12.34775	11.41909	11.90098	15.45964	17.42837	17.06144	21.47971	16.38877
Iqsec2	20.93678	19.41284	23.00743	22.07425	19.69516	18.43249	24.57354	19.29981	16.38015
Trappc11	18.91975	20.81388	19.79428	16.26275	15.45355	14.04456	20.8272	14.43519	16.36957
Tubgcp4	19.36676	18.13722	18.37525	16.05167	16.87341	14.35556	22.76806	15.02971	16.36762
Gm9892	17.79688	17.8673	17.20827	12.80305	16.01208	14.85589	14.44659	16.11965	16.36158
Pcbp4	13.99539	14.54537	17.39435	14.59258	11.9487	16.37708	10.95403	15.26552	16.3606
Snap47	16.76106	15.85445	20.34406	19.09205	14.05219	14.90065	18.18478	15.34101	16.32782
Kpna6	22.93821	21.00864	21.77588	18.55281	16.1234	14.69527	21.46042	16.83937	16.32029
Mlx	17.11727	14.53661	16.894	16.76542	14.43305	15.74025	15.39697	16.81809	16.31428
Lin7c	19.98022	19.49935	20.04955	15.07112	15.31504	11.87193	18.21293	17.03278	16.30973
Rhot1	16.63982	17.04254	17.65035	15.697	15.62081	12.97876	18.15354	15.416	16.29976
Naa40	18.00979	19.22451	21.70343	17.18191	16.26924	13.93404	20.54914	17.53757	16.29819
Dcaf5	16.98148	15.51574	15.25802	15.13089	15.61891	13.93226	18.07992	16.00568	16.29002
Cnot11	19.5584	18.67807	21.13705	15.58941	15.54419	15.67331	16.97394	14.8133	16.28251
Zbtb21	17.27165	15.86154	14.77065	18.78098	13.92719	13.37527	24.22758	15.18362	16.28113
Proser1	23.23216	19.98758	23.66892	19.0909	18.06556	14.13011	26.65023	18.69202	16.27825
Pgghg	16.35383	12.81953	15.28949	11.16152	14.3108	14.212	13.0355	13.09986	16.27683
Hmgxb3	21.21121	17.40309	17.33714	16.2282	13.79902	15.71353	20.08978	16.0962	16.27654

Atg4c	15.94142	14.43564	16.57783	17.31115	15.37127	14.33873	21.18961	16.65026	16.27074
Sp1	20.33722	20.16992	21.77215	18.11494	15.85039	14.51199	21.61318	16.57469	16.26754
Rpusd1	20.41517	19.40141	18.60028	17.32478	18.49308	17.39664	15.65016	18.62312	16.2655
Atg4d	17.60437	14.45577	15.00968	12.14338	14.15753	14.06357	14.30238	12.90359	16.26184
Dna2	19.18906	19.37989	21.415	18.47857	17.24874	15.83881	21.35096	20.25851	16.25964
Fam204a	19.59738	16.10448	16.91609	16.91467	16.29136	18.25597	17.13493	15.60208	16.25766
Setd1b	19.5175	17.54871	21.18183	19.20633	18.91447	13.90789	26.14467	16.3058	16.25627
Tepsin	18.65225	19.5178	20.52983	21.58285	18.69515	17.3966	23.36043	17.68279	16.23719
Rab3gap1	17.93584	19.67529	18.35772	16.02134	13.39184	15.54145	19.03815	16.40038	16.21742
Angptl2	30.92233	30.40655	39.03976	15.4626	14.51808	14.76691	10.84736	16.18417	16.21636
Vps50	15.63011	16.35669	15.91376	17.01836	14.61757	12.89068	19.10581	16.52694	16.20395
Ahsa2	15.92696	18.43016	17.71569	15.73622	14.63387	16.71147	15.08953	13.21992	16.20152
Gm8942	14.44278	12.52069	14.66668	12.26284	11.32154	12.22609	15.96077	12.5874	16.20065
Mmp13	15.41904	15.75003	17.51425	15.33266	16.61922	17.8813	15.44369	11.77986	16.19688
Fcho1	17.83961	18.22963	20.1633	20.04803	19.42738	20.92615	21.08295	20.34379	16.18396
Tax1bp3	19.40528	16.89539	16.7077	17.62369	21.12689	19.35642	19.12629	19.33246	16.18308
Fen1	24.87264	23.47988	20.53894	15.71293	14.94778	16.01491	21.55549	15.95631	16.17575
Gm18373	19.43132	17.48072	19.6576	19.32248	15.35959	14.29273	22.39831	14.39522	16.17145
Hint3	9.797244	13.74318	13.86113	11.24728	10.46869	12.52221	13.08963	14.48484	16.17007
Gbp3	10.38417	12.34013	16.41559	19.61108	20.41759	18.60572	16.33686	17.02274	16.1625
As3mt	17.85131	17.89933	16.76749	15.81423	17.6705	12.52153	20.5956	12.34429	16.16167
Snrnp35	17.05787	15.34159	17.05985	18.98164	14.89191	16.76049	16.10851	14.83903	16.16089
Npl	26.27677	22.02949	22.96027	26.75826	28.78898	26.09046	34.22361	15.49991	16.15627
Lipe	14.35836	12.71989	16.62307	15.01432	16.49589	17.86274	16.62599	15.92287	16.15511
Ddi2	23.26237	18.93531	21.29107	18.38103	16.85855	15.06586	24.12377	16.94289	16.1543
Taf1	18.67207	18.39812	20.1412	15.4587	13.41888	12.59407	19.40917	14.18949	16.15326
Ciao1	18.20892	16.3444	16.94889	15.00544	17.30764	15.47191	14.77636	16.54709	16.15192
Siglecg	11.78339	9.812007	9.727106	15.80918	21.34033	18.09234	23.39089	11.89564	16.1507
Cnot6l	17.98965	18.8982	20.71942	15.73816	17.61897	13.27757	19.7812	16.47388	16.14746
Arg2	11.85443	11.62778	7.082185	4.474005	12.3706	7.95965	6.510218	11.47283	16.1458
Nipal3	24.20122	27.35287	17.65624	22.44617	18.55086	13.76568	30.53425	13.55226	16.14304
Marveld1	29.40361	31.93083	28.37995	16.66188	20.59094	20.26036	17.98826	16.72503	16.14142
Tmem199	14.81926	14.77981	13.29347	10.87773	13.99576	12.63552	13.86586	13.44125	16.13055
Snx21	16.88673	18.84241	18.12889	16.79213	21.32786	18.75307	19.30038	18.99474	16.12931
Kri1	18.28731	15.94017	17.54249	19.35193	15.62822	18.86513	18.96701	15.29838	16.12781
Smim8	12.63596	15.35353	13.88338	15.04282	21.44149	18.68009	21.88913	15.92794	16.11413
Psmf1	19.1901	15.72133	19.19233	15.82645	15.5386	17.04218	18.02369	16.91142	16.1125
Tepp	12.43157	16.33582	16.54934	16.0371	15.51959	15.14718	20.0543	19.11365	16.09139
Adgre4	11.24324	14.41258	19.29678	12.47515	15.41157	12.17435	17.44709	19.56474	16.08884
Gab1	19.42375	19.98336	21.07436	13.89701	19.03085	13.08767	19.73754	16.82036	16.0849
Bnip1	20.59128	15.49152	18.60074	20.01866	18.83231	15.6401	20.12031	13.47534	16.07036
Vwa8	17.95455	18.76323	19.73799	18.80282	14.97929	14.45664	25.06582	16.18602	16.05129
Iws1	17.55685	18.85367	16.35803	17.3129	14.57331	16.10061	17.52538	16.21395	16.04939
Pex5	18.40551	17.14133	16.70905	14.19034	17.2479	13.74301	19.8053	16.90867	16.04652
Zfp950	14.24789	21.51415	19.75405	20.83282	14.33045	15.31044	19.68042	14.9177	16.03313
Nln	17.17407	15.13906	15.85596	16.18824	16.37036	12.8711	16.91655	16.03023	16.03294
Gm40645	15.20076	11.59316	12.16202	16.53826	11.75195	11.74188	24.76752	15.83474	16.02166
Mmgt2	21.66228	23.33612	19.33652	23.30658	15.29468	15.47144	18.43324	20.09866	16.02045
Casd1	21.69069	20.72685	23.58616	18.74599	17.97208	13.74015	20.12962	17.1917	16.01899
Rabep1	17.4094	18.78465	16.7585	17.57156	14.6634	15.90849	19.33175	17.5907	16.01196
Gle1	20.02079	19.52059	19.53912	15.63105	15.36332	15.6305	17.93226	14.45417	16.00667
Rps6kc1	16.33029	16.42158	15.67581	17.7936	15.07481	15.1553	19.59145	14.11453	16.00576
Pusl1	17.5289	15.19789	13.83775	17.29126	14.4239	15.34563	16.42607	12.10525	15.99378
Txndc16	16.70397	20.45377	22.42202	15.34351	19.59935	17.1164	21.94625	18.7847	15.98782

Pira1	15.08103	23.00646	22.03684	15.46374	20.44827	12.42582	17.72354	16.0584	15.98555
Lrch1	14.66331	14.3291	16.58895	13.5645	13.19645	12.10463	17.11571	14.16584	15.97559
Socs6	15.72977	14.90349	18.6719	17.30824	18.07512	16.87555	23.93142	19.79008	15.97347
Gga2	18.6796	17.17159	15.03157	16.92937	14.95115	15.51334	21.82502	14.07245	15.96681
Snx19	18.22963	16.40658	18.96539	16.32718	14.09152	12.94675	18.30891	14.07235	15.9613
Gtpbp3	14.92348	15.10714	13.86113	16.12062	14.50628	15.04361	12.81971	15.43487	15.95796
Uspl1	17.34235	16.18607	17.36232	15.12079	15.16592	18.37856	17.96129	16.47743	15.94704
Tsr3	14.01816	13.7507	11.01967	11.01403	11.75744	13.60373	14.54809	13.8959	15.94469
Topbp1	21.57009	21.55593	21.30145	17.13569	15.56485	14.24094	22.96628	14.08359	15.93804
Dhx37	18.00287	16.44664	20.26335	17.57241	14.0086	12.836	21.2191	13.76762	15.93415
Nhlrc2	18.86923	16.59839	17.603	16.21315	13.89084	12.68001	19.50229	15.77839	15.92912
Rps27-ps2	13.13007	13.41845	14.9149	15.47439	18.83459	14.09104	12.73289	15.89647	15.91302
Cdk19	16.87202	16.3531	19.43875	18.10672	15.93671	13.83551	21.40872	16.51632	15.91067
Gpr160	14.22834	17.52554	20.82038	17.44129	19.38967	19.61561	22.73623	21.13172	15.90434
Gm4786	14.17349	14.43397	18.00302	10.94225	20.21953	17.974	13.84547	19.92866	15.90252
Ubr3	21.9932	20.97706	21.88573	18.73719	16.53588	13.3371	24.26094	16.15945	15.8903
Rps12-ps4	21.2566	19.38895	14.31515	19.43803	20.24455	19.26231	17.77453	17.69586	15.88176
Zfp512	20.72451	18.48316	19.65071	23.66005	18.67499	18.75996	22.98511	16.54433	15.87781
Prmt2	18.67815	18.30398	17.94829	15.58128	17.71141	20.35654	19.19273	15.87346	15.86414
Suco	11.17172	12.17649	12.00013	11.16489	11.08036	11.38123	15.60302	13.97301	15.8579
Trim44	23.80715	23.16249	22.35761	16.30643	18.39296	15.29252	21.0222	19.64764	15.85499
Cbx3	17.28019	19.59546	18.4815	16.90822	13.90978	14.24568	15.25404	14.84232	15.85415
Utp6	21.48207	19.868	20.31008	16.46278	13.91232	14.71404	18.65035	17.9714	15.84712
Abhd17b	16.86661	16.53129	16.91337	13.85427	14.89027	12.9256	12.10782	14.20328	15.84055
Pcgf1	16.11129	16.19492	18.01629	16.21027	18.84801	15.72043	11.0138	17.0305	15.83913
Apc	22.79646	23.07923	24.04846	21.816	18.63064	14.0314	29.88429	16.83124	15.83835
Ier5l	14.76935	15.39958	13.64027	14.30013	17.80144	19.40907	16.98124	14.6674	15.82765
Tasl	13.15827	15.57959	16.83178	13.4474	15.48556	13.8133	12.68051	15.71967	15.82296
Gm36193	9.950422	15.40572	14.45348	18.75278	12.81607	15.20494	23.06612	13.39863	15.80606
Slc35f5	14.73801	18.03846	16.74499	14.65545	14.56785	14.01156	18.13796	14.62244	15.8
Metap1	19.79154	18.67599	18.07547	15.72292	16.03222	13.5761	18.07281	16.08944	15.79525
Bud13	17.9772	16.17647	20.64103	18.57558	15.51915	15.74243	18.42463	16.877	15.79379
Padi2	20.37856	16.7568	21.73266	11.33138	16.31231	16.86285	15.90808	16.29623	15.79125
Crtc3	21.28561	19.22723	20.86493	13.65782	17.5031	13.20311	18.0528	16.28923	15.78918
Rnf113a2	15.39723	15.23595	14.31345	14.4315	14.77901	15.09395	12.54822	17.49209	15.78608
Tlr6	15.59058	15.1532	19.2534	16.7093	16.50744	12.50076	20.23891	14.99073	15.78396
Chek2	17.02539	23.15464	22.60935	19.30863	19.38629	20.25539	21.32921	20.09485	15.7797
Rb1cc1	14.86753	15.64088	16.4127	14.09259	12.89689	13.49776	16.557	12.46871	15.7756
Mthfd2	22.7912	28.60681	18.45472	13.02288	12.26866	15.47928	12.37305	14.94228	15.77464
Ermard	16.38653	17.5381	20.27059	22.74529	17.61608	17.61112	23.26385	18.80127	15.7668
Cdc42ep3	13.51763	12.98329	15.80747	13.72958	12.49834	12.84117	11.95974	15.22379	15.766
Specc1l	20.54921	18.31463	20.75282	16.81471	16.33081	14.20805	22.66589	17.51869	15.75883
Ufm1	16.32273	15.20005	14.99178	13.79604	14.50416	15.58984	13.84476	14.85761	15.75243
Pianp	17.30921	23.45738	22.01036	21.83082	26.69506	18.62906	24.56027	26.76403	15.75085
Fig4	19.0117	20.46917	19.48969	15.89795	17.31367	14.36052	18.40279	16.12753	15.73949
Fam160b2	18.56681	19.91812	17.50392	17.11225	18.77055	14.97202	21.27938	15.95817	15.73943
Ocel1	14.66161	13.80401	15.48417	14.394	16.37965	16.31862	16.85009	16.09048	15.7161
Rnpc3	14.25621	14.24298	14.08429	13.43672	14.85256	12.83977	13.09449	14.61196	15.71416
Fam117a	14.66704	15.17684	17.06169	19.39099	18.41229	19.25016	20.57486	18.17886	15.71211
Rabgap1	17.67684	19.483	19.0848	19.39345	15.60413	14.22517	23.79763	14.18404	15.708
Cebpe	4.970618	2.76899	5.553408	4.764657	7.847289	13.29308	6.942408	5.244139	15.70611
Nbn	16.53572	16.86903	16.35958	13.89807	13.01419	14.38615	15.91739	17.20569	15.69638
U2surp	18.21725	16.43177	17.99154	14.64416	14.66939	14.3459	17.53726	15.63044	15.69573
Paip1	14.45021	15.47836	16.10602	14.08485	16.95998	15.66312	17.28708	16.87192	15.6924

Ube2g1	15.30073	17.53675	16.54521	14.20931	14.59977	12.13208	14.18637	14.51277	15.67083
Phospho2	12.99025	15.23054	16.10756	12.36428	14.14674	15.09983	14.71981	14.27484	15.66829
Mrps31	18.18009	19.02709	20.15756	19.72985	15.60445	20.38242	18.65085	17.65114	15.65869
Chchd5	12.76394	18.71305	20.29703	19.46175	19.89571	17.78423	19.2166	17.25659	15.65252
Thoc3	17.44654	17.30501	18.35437	14.38625	15.52633	15.2083	14.87876	15.22659	15.65132
ND3	7.646629	14.14038	9.400074	17.58378	19.72362	19.00429	4.939483	11.22386	15.63866
Txnl4a	20.98128	19.25418	20.84406	14.54561	19.05139	17.6272	16.9224	16.85183	15.63827
Rlim	14.82576	13.86355	14.56631	13.51849	12.74841	12.08889	16.37967	15.34457	15.63788
Wipi2	16.44866	17.0831	16.36555	15.94796	16.23223	15.8925	20.28054	14.16345	15.63652
Snora68	24.32486	23.13173	14.70977	18.00082	15.08532	13.60155	16.95553	10.77193	15.63556
Dip2a	22.16156	19.10406	21.75411	19.04776	14.27341	14.00691	23.82316	14.98467	15.6298
Crot	14.18091	20.33695	18.33645	13.23611	14.93731	16.71723	17.82514	16.9853	15.6269
Fam114a1	12.21931	12.74924	14.75888	18.18891	15.1288	13.99466	17.44782	14.8378	15.62367
Rlf	15.38014	12.23572	13.04079	12.33054	12.61544	9.789228	16.02245	13.27394	15.61736
Gm5424	18.52704	11.27212	17.84955	11.27447	10.55972	11.57651	9.500379	12.53158	15.6126
Zfp64	18.90552	16.92898	17.03055	17.42008	14.74112	13.83565	18.75259	15.07331	15.60751
Fam3c	16.61915	15.6167	16.41664	15.63166	13.78371	16.20064	17.83168	14.77208	15.60574
Cyp20a1	20.98593	19.25459	20.29498	16.67879	15.1869	15.53557	20.06395	16.90867	15.6047
Gm10335	13.88542	9.714587	13.26523	16.38369	10.95448	16.67388	16.81455	16.37736	15.60432
Sh2d1b1	12.46351	14.34612	14.47544	16.0585	19.99625	19.00322	19.73803	18.35265	15.60428
Scamp1	18.52571	17.42852	16.59627	14.78057	15.18622	12.33103	17.35868	20.18326	15.59498
Prxl2c	15.60001	18.60772	18.2359	15.56757	15.29581	14.54563	16.73254	17.13238	15.58791
Gatad2b	19.09882	18.97353	17.82465	15.88219	16.54754	12.83755	21.60632	16.15166	15.56384
Sfxn1	21.41791	20.16106	19.5466	16.55199	13.42974	13.13145	17.97824	14.04528	15.56363
Gpalpp1	19.58852	20.67303	21.51426	14.63187	13.19965	12.82626	17.01692	15.59834	15.55639
Ifit1bl1	7.53357	5.29191	9.887395	17.99422	13.51549	7.753758	9.286674	9.650971	15.55304
Cracr2a	15.17439	14.92134	15.42495	15.90217	15.09499	11.00934	25.41681	15.80091	15.54639
Gm38503	19.24027	15.93667	20.54708	21.07817	14.59726	13.87235	12.1337	14.28232	15.54041
Aldh1b1	16.06307	15.15564	16.76341	15.47019	14.10562	12.49392	13.39111	13.64273	15.53906
Ptcd3	16.68467	14.89951	14.67475	11.67856	11.66415	12.66638	14.95129	11.68875	15.53544
Abcb8	18.57364	18.20448	18.36835	18.5821	15.39061	15.95567	19.46963	14.14714	15.52773
Spire1	20.48216	19.20824	19.70429	15.05267	12.38732	13.75099	20.9166	15.04428	15.52605
Trdmt1	15.41224	14.59118	15.46004	14.13644	12.35619	11.57239	12.2204	11.47635	15.52236
Ints13	13.99646	13.865	15.15042	13.89927	13.88774	14.3933	14.58317	12.48076	15.51817
Zfp410	17.08122	17.69625	16.11727	16.29953	14.61226	16.66381	16.14269	15.26462	15.51721
Ano10	15.78833	15.96933	15.43232	12.85345	14.2273	13.73197	16.64161	14.98945	15.51659
Lipo3	16.51909	17.61738	19.42871	15.04673	14.09641	15.39855	16.4359	17.21669	15.51324
Lsm14b	17.41464	19.26657	18.72948	16.81672	16.68969	13.51484	20.66708	14.78909	15.49258
Ncbp3	14.69234	17.3995	17.23614	15.81637	14.74473	14.13904	16.59831	14.88689	15.48967
Mtif2	16.98624	17.41788	16.20802	15.43971	16.05768	16.19705	18.25837	14.37562	15.46987
Zfp146	17.31948	15.90091	16.86929	17.41795	15.57746	15.45294	18.40851	14.61812	15.45775
Map3k7	18.4724	17.9132	19.02774	15.90936	14.10415	12.71068	19.10293	14.51717	15.45733
Ckap5	21.66799	22.22993	21.8833	17.12897	16.32428	15.2842	22.21903	14.3989	15.45214
Ttll3	15.98146	20.59093	19.1188	21.55676	19.73575	20.61302	27.26595	18.41926	15.45038
Mdp1	17.27032	17.35709	18.63681	15.71763	17.77912	18.86369	15.16712	15.39753	15.44527
Pwwp2a	17.07557	19.83021	20.39179	17.04857	16.24067	14.76943	23.45199	15.14018	15.44107
Tmem120b	17.03914	13.53433	17.58887	13.07085	12.34479	14.01271	14.40312	11.70369	15.42782
3110053B16Rik	14.4943	15.51088	17.45867	14.41311	16.82704	16.04552	14.10581	23.67153	15.42771
Gm11478	12.98724	15.85251	13.27954	15.03478	18.46105	22.58633	13.6233	16.51759	15.4263
Zfp451	17.29604	18.1817	16.23026	15.28813	13.3204	12.41574	18.89703	12.98596	15.41621
Cryab	19.40967	17.77646	23.91599	19.17479	19.63335	31.03026	16.8485	15.33953	15.41595
Slc10a3	17.98758	16.80785	18.58554	12.9214	13.69954	15.37497	13.43014	12.58784	15.41316
Fam219a	18.07328	16.9766	17.0239	18.51966	19.35843	19.42975	25.04416	15.03077	15.41223
Atp8b2	17.82381	16.23822	18.12609	13.80247	14.5206	11.95537	15.26592	12.91968	15.40926

Actc1	7.32344	12.86705	14.30704	15.31949	20.85202	28.70412	9.059438	13.15046	15.40639
Eral1	18.41513	17.15817	18.6375	18.7773	18.61321	18.42978	18.4924	19.2103	15.39998
Ccna2	25.89628	27.54697	28.26422	19.7094	13.55936	17.53964	19.39918	10.74098	15.38482
Homer3	15.10255	16.12307	17.25827	14.17187	14.28221	12.95823	15.00466	12.02478	15.3824
Sik2	19.1973	19.03723	20.65121	16.06334	13.37802	12.31168	22.98695	14.33983	15.38116
Tmem70	16.44866	16.7886	15.74509	11.7971	10.10777	13.81758	11.1017	15.20282	15.37144
Cnpy4	16.68434	19.21662	16.09478	13.15508	15.65281	16.58058	14.47732	14.78858	15.36764
Tmem185a	19.85637	19.91732	20.52063	15.41029	18.51972	15.70314	16.20573	15.60223	15.35743
Gm10382	11.81283	11.85595	10.1318	21.1886	13.74259	13.65517	24.37966	16.72809	15.35598
Adck2	17.90187	20.09736	22.58513	16.86467	15.05223	14.92488	16.52344	15.65533	15.35297
Sike1	17.27734	18.95052	18.15124	13.89024	15.94525	13.61389	16.23921	14.9783	15.35268
Fam222b	17.52463	15.59324	18.95892	14.29939	16.0294	13.06602	15.5643	14.57668	15.35169
Ednrb	20.34187	15.46864	18.39533	13.01073	21.77848	13.01912	18.0649	29.66647	15.35089
Hinfp	13.88591	15.29703	16.29012	18.69086	14.98475	16.82058	22.45536	17.59416	15.34801
Zmat5	14.62949	15.5679	15.89129	17.33176	16.99955	19.22527	17.14599	19.32691	15.34419
Casp9	15.9774	15.1031	17.55962	15.36412	13.8929	12.58344	16.90662	13.24786	15.33944
Topors	14.86995	13.83766	15.5504	15.79143	15.81373	12.53792	16.30406	17.11553	15.33664
Ehd2	17.04113	14.64529	15.82834	31.04303	15.90174	19.68212	9.331068	14.94755	15.32596
Mrpl57	14.71342	16.27031	15.52733	14.72601	14.80909	14.33543	11.80028	15.2139	15.3208
Dhrs11	12.59597	12.6412	12.08406	13.74262	16.13291	12.96291	13.141	13.75953	15.31682
Iba57	14.82301	17.08978	15.35842	10.49487	16.54544	16.61392	18.50695	11.79843	15.31437
Lig3	20.54093	16.40278	19.73126	14.6709	14.38017	10.87974	20.71227	14.42512	15.3128
Parp11	16.24786	16.15708	18.39414	19.2129	16.85834	16.43553	18.71357	14.3581	15.304
Evi5l	24.15159	21.73905	23.54786	18.83701	18.48291	18.90361	21.68033	17.89985	15.3004
Foxp4	22.64245	20.87955	21.58172	16.52932	17.62806	17.62922	19.8113	18.42111	15.30029
Naf1	18.41378	15.42169	17.98307	15.25886	14.87654	11.20678	15.81897	17.19457	15.29997
Cpsf2	19.55717	18.78732	19.67419	16.31526	14.53328	14.71948	16.21377	15.51199	15.29948
Bmt2	14.01682	16.51638	13.7105	12.9798	12.87771	10.85764	13.18643	15.47402	15.29873
Gtf3c5	19.96915	17.10535	18.87051	19.15671	14.3005	17.26558	19.45584	14.84114	15.29725
Cavin3	8.699345	11.37065	12.42908	23.21159	15.43819	18.08849	9.717205	17.33693	15.29504
Tango2	16.88041	19.20924	15.34429	17.73257	17.05318	17.81152	20.77715	13.84058	15.28585
Acrbp	11.88902	18.26913	16.99887	27.54187	23.98793	19.30162	27.9891	15.97309	15.27626
Rnf26	22.36075	20.57263	20.43463	19.63812	17.80878	15.35087	22.17743	14.82836	15.26259
Mphosph8	15.07421	15.13338	12.70603	13.02942	12.55484	14.14273	14.94194	14.33317	15.25908
Nufip2	14.32465	15.72208	13.7185	13.18417	12.43425	11.10736	17.35084	14.29324	15.25318
Clpx	13.09929	14.75579	14.69942	13.39421	12.10432	12.74474	14.58641	14.04934	15.25299
Tbck	16.41173	17.5065	19.53697	15.59162	13.33298	14.26086	20.59077	14.98989	15.25182
Inpp1	13.98438	14.88111	15.95278	15.05453	13.85369	12.67251	15.13106	13.87917	15.2324
Lrrc41	17.81737	16.74223	18.40031	17.494	14.61374	14.15527	18.20214	14.68063	15.2316
Bola1	12.09143	15.9273	13.99422	20.94695	13.25397	16.7375	16.3484	14.40838	15.22065
Foxo4	14.77223	16.122	15.68149	14.62653	15.71698	16.04179	16.96911	14.92858	15.20559
Gm31223	10.76854	15.03583	14.83839	8.090658	13.0667	12.46646	10.80006	10.48055	15.20501
Snx13	14.4212	14.43746	15.19015	15.56649	12.94305	11.75687	17.00061	12.79127	15.19837
Rmdn3	17.87417	15.91386	17.46707	18.96916	14.34427	16.24571	16.83525	15.75196	15.18344
Dgkq	19.09236	15.70676	17.0547	16.6912	14.33014	12.30665	19.17997	13.1797	15.16924
Cd200	18.67516	24.09346	18.77525	14.652	9.021218	10.88725	11.30782	10.78953	15.16305
Ripk2	17.98754	15.71072	15.64598	12.44954	12.6262	11.27682	14.41981	13.53132	15.15844
Nup93	24.40187	25.20534	23.36797	18.43082	17.8299	18.40564	25.24198	17.0941	15.15568
Dis3l	17.9943	18.19418	16.78953	16.30532	14.0616	15.27847	19.20327	15.22311	15.15439
Map3k5	15.61312	13.63656	13.24973	12.59177	13.10945	10.83951	16.1844	12.11078	15.15423
Nme6	18.6822	11.34942	15.94562	13.37342	15.12671	17.10789	18.36555	16.28087	15.13783
Asb8	18.04424	15.90303	20.58051	16.23793	16.60012	12.82868	15.87202	14.35313	15.12428
Nol11	18.85979	15.76795	16.76621	12.24229	12.49133	11.6619	14.67742	12.27375	15.12187
Tmem80	16.5759	9.729225	16.02259	13.0918	18.20381	14.88881	14.83682	15.2533	15.118

Zkscan8	23.73868	23.4609	21.08756	24.31887	22.33524	14.12672	21.79251	20.55307	15.10616
Usp33	16.30531	17.7491	16.20964	15.21619	14.10883	12.23045	18.26392	14.50257	15.10209
Pkmyt1	22.01217	22.30709	20.49036	15.01555	16.84559	18.55329	17.36525	10.462	15.10123
Rnf123	18.56989	15.58342	19.18683	15.60857	14.37734	14.91223	18.91218	14.10073	15.0964
Gm4149	17.97168	15.18969	15.53665	13.63043	8.558016	15.77458	10.3892	13.63064	15.0964
Snord99	9.899654	9.873298	8.910724	26.57934	13.19965	13.73234	8.185429	12.25307	15.0964
Dgat2	13.39774	11.24968	11.97069	7.396052	10.50812	10.545	8.018157	10.10628	15.09305
Uqcc1	14.46327	14.29095	15.35056	14.16879	15.79358	16.03036	14.69891	14.25587	15.08909
Tmem198b	19.2828	19.81621	21.50022	17.29618	19.85376	16.38629	18.54947	14.35857	15.08753
Eif2b3	17.17312	13.41072	12.21854	15.16675	11.92817	10.6585	15.52565	15.8384	15.08594
Bzw2	21.0078	16.60783	16.20959	15.32139	20.28979	17.39967	19.53828	14.72605	15.08026
Prune1	17.26987	17.14479	18.04524	16.3056	13.78504	12.89019	17.78779	15.02677	15.0802
Suz12	16.42275	18.85837	17.48099	12.5907	12.35321	11.11349	15.07001	13.28146	15.07535
Dhx35	21.14465	18.99724	18.94354	17.49239	18.10721	15.24876	22.11115	15.80091	15.07221
Fmr1	13.40574	13.04679	11.99396	12.72471	11.94019	11.43805	13.9225	13.61531	15.06286
Ankrd23	12.00156	13.14498	10.36728	17.93737	15.59907	25.08877	10.56471	14.72413	15.06038
Endod1	25.81467	26.65402	29.88334	15.37909	17.28199	13.94644	20.19944	14.71267	15.05799
Ddit4	10.7257	9.089294	7.206141	5.856277	10.12104	12.32243	11.11955	13.72031	15.0516
Lrrc42	17.41112	15.12415	13.31593	14.32396	13.15245	14.51252	11.98546	14.46957	15.05142
Arel1	18.79143	15.1065	17.22191	14.9844	14.54244	12.61586	20.04393	14.73962	15.04177
Dglucy	20.87218	20.34969	26.84435	20.15394	16.98027	16.27135	19.45552	21.52529	15.04062
Tmco3	18.66139	18.46146	18.83305	14.39982	13.93496	12.71236	16.69894	14.14585	15.03999
C2cd5	14.14562	15.15576	17.14933	13.43209	13.29346	13.82745	16.4041	12.77139	15.03765
Acsf3	16.51268	16.46871	18.11039	18.85553	16.71602	13.31442	16.83656	15.79235	15.03072
Sms	16.16876	17.04857	15.45627	13.89892	10.68341	10.67382	11.67772	13.27676	15.02346
0610009B22Rik	16.31762	18.84744	20.08556	17.75176	20.45531	18.44336	14.1263	13.61057	15.02044
Mettl23	14.92133	14.99308	20.28867	15.11561	15.7012	15.45257	13.86242	15.74961	15.02031
Prc1	23.99317	29.47445	25.47263	13.19115	13.99613	16.12906	19.33778	13.55255	15.01787
Camsap2	18.5058	20.06144	19.65265	16.28358	13.84722	12.34525	21.29349	14.50115	15.01308
Thumpd1	15.02366	15.97747	15.31288	12.63379	13.08559	13.53898	15.15139	15.81832	15.01291
Junos	16.32709	14.19177	13.1619	13.70009	10.65372	9.241821	15.59968	15.27089	15.01241
Siglecf	9.39316	12.05461	11.53559	13.0035	18.26463	16.05299	17.25127	17.18499	15.00863
Hsdl1	14.11411	13.88053	13.77625	12.64931	13.39107	13.30011	15.0308	13.28492	15.00153
Kdm1a	17.06352	15.9728	17.2494	14.48126	13.7469	13.21219	16.2867	14.46047	15.00126
Mapk9	17.99337	15.28556	17.79393	17.10285	15.22885	13.95411	17.77623	15.50633	14.9915
Cdiptos	17.04435	22.60688	20.6489	21.31714	16.65122	14.29962	22.33805	18.98831	14.98874
Stam	15.21979	14.75126	16.90774	13.54945	12.21798	11.66521	14.20965	13.90702	14.98788
Mctp1	13.70761	12.64094	13.38106	14.24712	11.36524	12.6539	14.82062	13.28239	14.9872
Mgp	31.44785	38.35384	33.60818	29.21669	22.76245	30.07908	14.11555	30.19807	14.9863
1500011B03Rik	17.92773	17.05477	19.86072	21.1185	25.47992	26.95151	22.33007	22.05835	14.98374
Amd1	17.96636	16.46987	15.20141	16.12901	13.27376	13.88025	14.36918	16.77646	14.98103
Gpr89	16.8984	17.9185	19.47626	18.3342	14.59814	14.58143	19.08851	14.41425	14.9681
Zc3h13	17.08552	16.59658	16.46304	16.3407	13.08252	13.21929	17.11118	14.39358	14.95857
Dcaf15	15.22315	17.33391	16.88627	13.36275	14.97062	17.21905	16.8614	15.13129	14.95799
Rnf144b	10.91666	11.30166	15.03352	20.10819	17.00255	13.52827	19.92809	15.31157	14.94344
Tmem159	17.56259	20.14125	20.47242	14.92467	16.91351	14.6063	14.29405	18.18021	14.94126
Mt3	57.49896	97.83741	40.39823	19.98117	16.4873	15.24681	13.31273	13.14656	14.92804
Acvr1b	16.80855	14.98468	16.83563	14.67819	16.19971	13.02305	17.1907	14.74752	14.91851
Mtap	18.68163	18.15637	16.42961	13.01682	13.99431	14.14964	15.59007	12.73135	14.91345
Atmin	22.38644	20.48653	22.53993	15.61389	16.86637	15.42207	20.00692	16.96066	14.90995
2310039H08Rik	13.26784	10.94365	10.40033	12.69431	13.31918	12.86659	13.26823	14.1345	14.9011
Rrn3	18.98586	17.19652	18.54016	13.32858	12.3085	14.71756	16.3992	15.28956	14.89387
Gm4950	12.01909	15.5083	10.14229	14.34212	13.66468	15.49136	11.72358	16.80743	14.89184
Lime1	12.33844	14.39415	13.95311	14.8864	14.25751	15.8853	17.6954	13.55262	14.88833

Gdap2	15.92696	13.65079	16.12026	11.59277	14.18416	13.50726	18.00455	15.65613	14.88789
Ap5b1	19.8988	16.57126	22.90618	18.08563	17.01836	17.40809	17.83107	18.3583	14.88778
St3gal2	20.55165	18.11416	17.85632	18.08632	14.56977	14.7174	16.71413	13.08555	14.88653
Coq7	15.43057	16.94085	14.3745	13.24051	11.31822	15.88081	17.74888	15.58001	14.88461
Zc3h10	20.89507	18.06964	18.46387	13.59777	18.41402	18.6627	18.75307	18.45875	14.87312
Mllt10	13.78104	13.59528	14.61977	13.51063	13.4808	11.00265	15.55651	13.14937	14.87254
Ctc1	21.5271	20.20007	22.47875	18.87456	17.70241	16.20435	21.55553	15.88984	14.86948
Exosc9	17.17654	15.6605	13.33823	14.72795	11.68949	14.36248	15.30782	13.54652	14.86767
1810055G02Rik	21.47228	17.76247	20.05691	14.56753	15.02102	13.90256	17.73582	16.95675	14.86316
Trip11	19.00095	18.86461	18.53883	14.91042	13.28972	12.03766	20.90915	13.0892	14.85642
Ccdc58	11.20636	13.24899	13.65701	11.73397	11.30894	13.45168	14.65049	16.39295	14.85389
Msto1	15.67454	14.39067	13.45858	13.41185	15.36222	17.3045	16.57718	15.55998	14.85238
Inpp5a	17.21476	15.33812	15.27886	13.4675	13.03453	13.26011	13.36532	15.53568	14.85201
Gm13493	12.51102	13.15017	14.61038	10.87651	13.271	12.95541	11.76932	11.41243	14.85159
Anapc7	16.92765	15.39531	13.96694	14.9692	14.89339	13.97693	17.5164	15.6563	14.84671
Xpot	18.7983	18.78681	18.16252	14.46425	13.44818	11.87837	18.06046	13.0944	14.83979
Scaper	14.05714	15.87073	13.40568	14.73029	14.0098	11.88717	15.701	12.8602	14.83902
Ndufaf3	13.67652	12.71796	15.21931	13.10189	11.88794	14.60711	13.29919	10.41711	14.83413
Supt3	17.37301	15.15445	16.07839	14.49096	17.63246	15.44256	17.84868	15.70143	14.82808
Lrpprc	18.15801	18.71552	17.24881	15.21982	12.81605	13.14047	18.9037	12.61096	14.81411
Dohh	17.27139	15.53362	17.27339	15.41117	16.96311	15.61552	17.293	17.98568	14.8077
Rpl9-ps7	13.10488	11.74831	11.85467	13.17789	14.72471	11.09772	12.25097	12.75752	14.8057
Cblb	18.91189	16.4656	19.61559	15.48418	13.37709	12.48644	20.38007	14.45821	14.79079
Pira2	20.25622	14.49416	15.01154	12.55435	11.54301	8.925091	10.98985	14.21066	14.78698
Timm9	15.99844	16.99644	15.1655	12.31204	11.9244	14.28025	13.58761	16.15925	14.78386
Avpi1	17.6652	15.46547	15.05409	9.093782	10.603	11.08342	8.92346	11.78953	14.77931
Rnmt	15.65588	16.11113	17.1002	15.67249	13.31204	13.50801	16.42507	15.03077	14.77148
Bloc1s4	14.34768	11.34598	14.77388	13.4653	15.32211	15.30956	16.01361	17.54027	14.76696
Kbtbd2	14.06273	14.54415	14.01559	13.3858	14.29124	12.14781	17.05498	12.19724	14.76658
Phactr4	19.26662	17.60231	19.02495	13.9893	14.57188	11.76337	18.36083	13.58974	14.76609
Eef2kmt	16.91638	16.5613	16.7629	16.17761	15.4695	15.78748	15.26162	15.53876	14.76484
Upf3b	12.77157	16.88907	13.26978	17.87411	15.0467	15.20087	19.31119	15.83024	14.76472
Fubp3	18.03992	16.8501	16.09903	16.50721	16.10721	12.91309	18.50173	14.43425	14.76339
Hdac7	17.46058	14.86178	15.41506	19.38407	17.79916	16.29575	20.1926	16.52672	14.75991
Rpia	13.54666	11.13922	15.51946	11.88415	15.1066	14.63956	12.86942	16.08495	14.75988
Pex7	14.16479	15.49716	14.68547	14.63187	12.06576	12.84508	13.91433	12.52712	14.75141
Ngly1	14.2906	14.29967	16.8436	12.58984	14.64503	14.72272	16.13719	16.14734	14.74584
Tex10	17.96	16.00127	19.12025	16.87535	13.47537	12.34467	18.95426	15.29549	14.72945
Snapc4	13.89975	14.44454	15.02796	14.70201	13.73607	13.24469	21.14371	13.57865	14.72392
Klf16	17.89404	15.01099	15.86817	12.90882	13.00056	11.90826	15.64244	15.58396	14.72354
Cby1	13.29873	14.32865	16.93247	19.06626	15.56491	16.10132	13.4689	17.18736	14.71099
Cep120	17.86658	17.71117	18.30125	13.44137	16.59254	12.26684	18.67711	15.88965	14.70378
Adora2b	18.06848	17.57062	17.6797	8.998582	14.00137	9.814201	11.83869	15.77177	14.69934
Snx30	17.17521	14.52813	15.74643	18.32868	15.13048	11.49752	22.51401	14.97535	14.69714
Taf5l	17.31506	15.57373	15.67316	13.50327	12.59482	11.95318	14.94227	14.51162	14.68695
Tex2	19.71028	15.97487	18.04655	17.89704	14.17874	12.46404	19.45652	13.25385	14.67767
Rcc1	17.13056	18.26905	17.27794	15.32391	10.75356	13.06332	14.82536	14.32601	14.66423
Kdm2b	17.6614	18.27452	18.53532	15.41131	18.11204	13.9888	21.67446	16.63094	14.66338
Gm4705	17.10888	16.79878	13.79481	15.43043	22.48362	19.74653	16.31211	16.4156	14.66301
Acadsb	16.60824	16.2007	18.21653	15.03328	14.08278	14.82283	16.10437	13.40057	14.66127
Vav2	17.67573	15.92797	18.68929	20.21966	22.26636	19.08661	30.38715	16.40355	14.66086
Tmem63b	19.80427	20.13238	21.47303	18.74405	15.52219	15.47331	20.00228	15.56703	14.65684
Mbip	16.90329	15.85622	14.59999	17.22001	15.6482	14.21058	16.24869	14.96816	14.64576
Ppbp	26.05538	18.88963	17.20162	11.19857	25.20487	12.49731	17.40632	32.21534	14.6364

Tmem38b	12.60503	12.75164	13.32916	12.97353	9.519806	10.41311	11.84546	13.85492	14.63454
Mis18a	16.802	19.9471	20.94096	16.49585	20.55084	22.08441	19.65762	18.18965	14.63189
Qtrt1	16.88729	15.53804	15.297	14.13526	14.6753	16.66847	15.98467	19.1641	14.63189
Gm3362	10.95073	13.74948	12.51657	9.430988	15.73715	14.19573	14.65293	15.54616	14.63046
Sipa1l1	15.10897	14.07953	15.92934	13.59755	13.09648	10.68627	15.17795	12.07478	14.62964
Dapk1	22.21447	24.16648	23.00195	19.54285	16.205	15.16047	28.16457	15.62926	14.62742
Togaram1	18.86583	17.56946	18.12501	15.60361	13.43282	11.54427	18.84726	13.34298	14.62282
Ankra2	13.73466	16.93582	23.02695	15.39598	14.17476	12.65519	19.15095	15.59698	14.61359
Brd3	16.94983	18.19671	18.00524	14.67579	15.451	15.73066	18.00651	13.42904	14.60846
Ltn1	20.33609	19.82477	21.54709	18.4403	14.26131	11.4841	18.69256	14.73307	14.60277
Elac2	20.52744	17.98582	19.93129	19.2619	17.28922	16.08678	18.57701	16.71413	14.60206
Gosr1	17.4393	16.74725	17.33774	15.06287	14.22924	12.10447	17.449	14.05232	14.59753
Grwd1	14.91007	14.00112	12.82602	10.46216	8.982581	10.36286	13.47999	11.14329	14.59629
Cars2	16.056	19.37635	17.60487	19.50382	14.26034	18.9964	18.60906	15.08071	14.59515
BC147527	6.550517	4.848232	11.03373	13.1305	13.75785	13.58203	9.763466	11.062	14.58943
Zfp110	15.23817	14.88919	15.75538	16.46822	15.05259	15.1721	17.93337	15.47326	14.58882
Gm46174	11.84391	9.713191	12.77293	12.45158	13.93013	10.8869	14.63791	13.3145	14.58639
Gas2l3	18.12324	17.82542	17.2494	18.39498	12.64967	11.81998	24.29197	16.42121	14.58532
Pgam2	17.56151	24.59389	14.19771	26.77275	21.15441	33.44263	15.93719	17.30286	14.57986
Clec4b1	12.46345	18.6454	16.431	19.79762	25.20459	24.02457	14.88912	20.49655	14.57849
Rbm41	12.9193	15.69805	14.27905	15.47865	10.9776	11.72194	16.05208	13.39455	14.57011
Foxk1	16.2455	15.25998	19.45843	15.91564	14.06895	11.60994	19.14071	14.01129	14.56005
Nle1	15.39946	12.08199	14.00487	11.27052	11.61569	13.40547	13.49686	14.11442	14.55964
Ptpn9	17.83255	15.0594	19.1466	12.97678	11.62168	10.0336	15.60619	13.33548	14.54754
Slc25a25	15.44372	12.63989	11.17219	13.4128	9.018181	11.45228	16.54195	13.53176	14.54668
Ikbip	15.08274	15.89321	15.66812	20.75652	14.45014	16.23121	15.91353	18.50592	14.54633
Zfp395	16.10043	17.14555	16.31262	19.04239	18.28799	16.14108	22.75348	16.19263	14.54344
Uevld	14.29231	20.31734	19.72971	13.81191	15.72072	10.05243	16.10336	12.15108	14.54276
Gmppb	18.30284	13.50078	16.08718	17.9041	13.61731	15.3343	14.40147	15.15723	14.54211
Bet1l	14.96685	14.46387	15.57591	15.06061	17.69025	16.05409	14.21374	14.72604	14.53709
Apex2	18.01737	15.72323	18.77028	20.8451	11.98968	14.79278	18.56217	15.99969	14.53028
Pag1	12.81843	11.75319	11.20748	9.935608	10.5969	8.898916	14.41417	11.65372	14.52546
Zfp652	13.80235	14.13877	16.25707	16.16413	15.06552	14.2057	22.19304	14.01199	14.52186
Scmh1	18.77741	17.23237	18.43767	15.11561	18.03368	14.32471	18.99967	12.82003	14.51274
Socs1	7.316007	6.662049	9.316495	16.08639	11.38199	10.08539	10.94298	9.907529	14.50863
Adrb2	10.31065	10.2101	8.161509	11.29762	12.44921	12.33671	13.38384	14.1203	14.50758
Epc2	15.46872	16.06189	17.557	13.32017	13.16936	12.28159	16.29009	17.16218	14.50058
Cog6	17.03012	17.62857	16.84222	12.91343	14.57386	11.71073	17.69959	13.01486	14.49409
Pigl	15.19454	13.01275	15.41655	10.29193	13.84194	10.28402	15.07842	11.05968	14.48175
Mir22hg	13.40433	13.08687	10.76867	8.178602	10.49395	9.434824	14.11361	14.8843	14.48092
Slc9a7	16.14749	18.82962	19.82042	15.90973	16.20955	12.11664	18.90086	17.27027	14.48008
Cog5	16.83838	16.17231	17.84513	15.06566	12.13277	11.14912	17.2787	15.03698	14.4781
Psmg1	17.52353	14.93478	16.99448	13.04262	14.91915	14.48647	14.26962	14.40901	14.47461
2900089D17Rik	16.06024	13.87288	12.76904	18.70995	12.03168	10.31562	27.01688	9.468855	14.47424
Adam19	17.31513	16.09736	18.23805	17.61807	15.03924	13.10558	22.92795	12.92858	14.46522
Mthfd1l	19.54111	20.667	16.17877	10.74791	9.884826	11.91495	15.35098	15.53829	14.46292
Spg11	15.9624	15.01823	16.00766	14.73663	14.25183	11.02934	19.04701	14.46784	14.46221
Cnn3	13.58966	13.7191	14.44934	19.07308	14.47151	13.41194	10.29819	14.01911	14.45576
Calhm6	5.892216	6.643033	10.65846	15.183	21.76357	15.54131	19.91132	25.12289	14.45459
Sos2	18.46562	20.09069	19.31236	15.5459	15.67382	12.83828	18.62744	14.90421	14.44857
Mdm4	16.13727	17.33234	15.598	18.34947	16.34658	11.88414	23.23337	16.58401	14.44743
Slc25a46	16.47866	18.51105	17.10249	15.91587	12.69432	12.75121	18.95962	15.86949	14.443
Apoc1	7.314744	12.15878	12.44935	13.01622	18.94394	12.81685	13.39606	17.22761	14.43244
Copz2	15.55842	24.13756	17.28915	23.82932	18.62144	20.23132	21.13249	16.06176	14.43086

Cdkn3	15.78795	19.12518	18.31327	9.175973	9.131618	15.00021	11.02744	8.991552	14.42894
Med20	15.91719	13.63605	17.07555	15.35454	17.58874	14.05922	17.50577	15.28425	14.42493
Leo1	16.03455	12.78844	16.26406	16.32197	15.58426	12.18536	15.8406	15.96477	14.41458
Mir3101	14.48949	9.424512	7.560614	19.41992	10.19973	12.81685	13.02227	11.99602	14.4102
Cyth3	15.75704	13.76206	14.71132	18.00853	14.21167	13.84163	17.68205	17.54598	14.4102
Snord12	17.63938	11.72828	16.80137	23.38743	7.999789	7.767786	15.62673	11.19628	14.4102
Lcmt1	18.24853	18.07807	17.43589	15.716	18.73749	16.31064	15.82957	21.4853	14.40314
Igf2	11.90785	18.51388	15.57915	21.67896	21.27705	19.76173	8.366029	17.09179	14.40085
Bambi-ps1	9.718772	11.14911	14.83083	14.92754	11.03773	11.68812	12.73289	15.29171	14.39439
Gm6969	8.222623	3.249347	3.672164	6.222417	3.546175	4.543285	17.21078	6.499334	14.39228
Gfer	16.78339	15.75021	14.43936	16.13088	18.40714	18.63713	17.34648	17.61513	14.39016
Scyl3	15.45895	15.99179	16.77257	14.95976	15.11809	13.18198	18.24627	15.27914	14.38933
Med14	19.61525	20.48992	22.32145	16.29882	15.14087	11.44629	19.63297	15.05581	14.38702
Ncapg2	12.41518	12.92909	13.45685	13.11665	12.59854	7.677429	18.29471	8.912612	14.37415
Brix1	16.06325	17.31213	16.94254	12.91986	12.11543	13.44465	14.14099	14.23973	14.374
Gm15448	12.71796	8.221745	11.12568	10.42212	9.544026	7.85023	9.28044	9.453577	14.37037
Ctr9	17.09555	17.20434	15.91128	14.74341	13.46978	12.35186	17.85566	14.76682	14.3644
4931406C07Rik	17.17646	13.73058	16.37491	19.23957	17.18591	13.55483	22.33118	12.69426	14.36316
Gfm2	18.04793	15.70508	16.36008	18.57247	16.11008	14.17999	20.6414	14.25674	14.35724
Fam133b	13.01736	12.16678	13.62048	12.34627	10.74763	11.97129	11.31536	15.21161	14.35599
P2ry2	16.78869	11.05809	13.4679	9.83303	10.39121	8.690003	12.43489	13.34535	14.35229
Pdzd8	16.78968	15.94797	16.71327	14.04677	12.78926	10.62878	17.2477	12.89724	14.35182
Acap3	18.43117	18.98763	17.26225	17.40794	15.46097	16.4831	17.83933	16.70871	14.34256
Galnt11	18.78106	16.97079	18.05907	15.95323	14.20175	13.32952	17.39988	11.84913	14.34158
Aff4	19.27711	17.66863	17.05108	15.83167	13.5619	9.980515	21.96747	15.028	14.33762
Gm16089	13.54387	10.76046	8.609395	9.701531	14.32012	17.40756	10.91391	12.67654	14.33064
Rpsa-ps2	15.7317	12.46588	13.20107	11.08979	12.87896	12.45563	14.2549	13.79833	14.32618
Gm34181	16.3142	15.66814	18.65776	22.23126	15.75326	17.74103	26.7755	14.66979	14.32513
Abcd4	17.16755	17.776	18.16777	22.06235	14.9922	16.85178	25.79	14.7161	14.32372
Kdm4c	14.86098	12.1104	13.82536	12.23475	11.5646	11.90726	14.41692	13.11048	14.31723
Fam220a	14.70754	15.19857	14.55735	9.993437	11.23477	12.07944	15.67192	12.55693	14.30711
Rspry1	14.96922	15.00786	15.71085	14.86972	14.03166	14.04686	14.65801	12.67858	14.297
Gm8399	20.68947	16.65292	15.65778	10.49391	13.62118	14.73496	13.81323	13.88143	14.2929
Mapk1ip1	14.1328	19.27379	14.9544	15.95592	16.88068	13.10568	19.8122	17.35874	14.28842
Gramd4	19.04247	16.53862	16.69282	12.37749	14.94373	14.49785	15.57466	15.78842	14.2849
Gtf2h4	16.32855	15.33293	16.69262	15.71331	14.67325	16.98765	16.7743	13.0389	14.27614
Ccdc163	12.88483	17.16565	17.30858	15.31845	13.40342	13.36645	12.58001	17.88085	14.27425
Eif1ad	18.38954	15.38459	17.7629	15.67417	12.16784	15.30339	15.52546	12.52755	14.27402
Eln	16.40914	13.17061	11.01698	23.04565	33.21705	23.35145	6.790624	20.97605	14.26859
Gmps	15.86546	15.53266	13.56066	12.74681	12.61294	12.89823	14.37782	13.53367	14.26506
Dnlz	19.21081	18.88113	19.2729	16.28633	16.45325	16.88005	20.14399	15.42259	14.25982
Traf5	13.58273	14.32758	14.82661	15.35945	15.97074	15.18294	15.62927	14.80891	14.25812
Wwox	16.04252	19.78035	16.45057	19.65475	15.72999	17.52731	18.04992	13.27618	14.25771
Polr3a	18.40969	17.6975	16.87276	17.32417	14.09531	13.95007	20.79458	16.18928	14.25053
Spg20	15.46859	14.60012	15.74692	16.60394	14.9223	13.18264	17.6387	14.47898	14.24516
Fmn1	22.02676	19.76134	20.25184	17.68737	11.72623	10.62707	21.73296	13.55749	14.24508
Trim21	10.37044	9.611317	13.91716	15.2558	11.93353	11.39903	12.88731	13.26836	14.23367
Utrn	19.89794	19.14509	18.2136	21.0358	15.51188	13.65944	20.85797	13.17945	14.22877
Mtf2	15.26819	15.42896	15.87591	15.1425	14.36814	13.29777	18.53575	15.9405	14.22471
Eml2	12.71019	10.88646	13.00672	12.56565	13.60354	14.2321	12.22188	14.06752	14.22275
Ralgapa1	18.33025	17.02237	17.66629	14.21434	12.36417	10.42446	16.02741	12.65379	14.22072
Srrd	11.54747	10.65042	11.70211	11.27955	11.43564	12.52153	14.8922	11.18891	14.21983
Nit2	14.35527	10.79153	14.31382	11.35987	12.11165	11.80027	15.59432	13.79075	14.21597
Zfp287	13.87669	16.16921	14.90887	20.42519	9.944719	11.37158	13.98725	17.92119	14.20783

Dmxl2	15.19024	15.06062	14.82904	13.02349	11.782	8.36135	18.80397	12.61972	14.20694
Pdlim4	12.92017	14.92383	15.88838	18.28844	15.82075	12.92353	14.98878	12.30127	14.19887
Plxdc1	19.9743	19.50733	21.04362	18.40859	15.27885	18.81875	19.41925	15.63159	14.19793
Dbf4	15.6039	20.06663	16.92013	15.68283	14.95202	13.96594	17.58456	11.7168	14.18866
Prmt9	12.87474	13.60509	13.14064	16.71965	13.74728	12.27288	18.55286	13.13898	14.18572
Stim2	15.95064	15.35203	20.21656	12.86925	14.71545	13.73003	15.14099	13.63805	14.18372
Mx2	8.972634	5.912565	9.545978	14.38505	10.07141	8.297611	9.652182	9.850403	14.18014
Ccdc25	14.36361	13.03435	12.39846	13.34249	10.83474	11.14208	11.50077	12.89343	14.16431
Rdh11	19.04512	16.25843	16.10634	12.89384	12.59347	10.31188	13.40149	13.51036	14.16396
Dcun1d2	13.19345	14.11491	17.39435	15.34499	14.79822	12.73308	13.79029	13.37318	14.15904
Prkrip1	8.781604	9.393363	11.49786	10.63221	10.27881	12.80135	9.214794	10.81817	14.15173
Efcab14	17.06415	18.00944	20.18267	15.87251	14.38548	10.74677	17.11627	14.53656	14.14963
Chsy1	16.82165	14.90945	15.71502	13.39088	15.52145	13.7969	16.11652	15.75915	14.14803
Fbxw7	12.88423	12.35799	12.27283	12.32157	11.28857	10.14566	18.41446	11.84012	14.14601
Cbx4	14.95558	12.73479	13.89047	14.65966	16.78339	13.96856	17.82776	13.90402	14.14216
Gm6789	14.4308	12.64407	17.78231	14.69237	12.67047	10.68191	14.13847	15.796	14.14169
Ttll12	19.96947	17.70175	17.22162	14.57882	13.47275	13.55798	17.38698	13.42663	14.14136
Capn15	17.79295	18.34817	16.58649	16.00121	13.8338	13.67206	18.93791	13.35998	14.13776
Pik3ca	16.379	17.23049	17.38134	15.83173	14.02324	12.5791	19.17985	14.62422	14.13155
Gm5854	13.31062	11.76975	11.39083	11.25748	12.21948	11.54785	12.48075	14.24081	14.12485
Itpr1	16.53538	11.30695	14.19039	17.25012	13.01341	11.62879	13.75398	13.03771	14.11765
Msantd2	15.21782	16.20831	16.61412	17.28413	16.43946	13.60609	22.13899	12.74928	14.11441
Cep350	17.98183	15.70733	17.53234	15.39912	12.62229	9.29463	19.22897	11.60339	14.11388
Nepro	17.46705	16.72604	17.50777	14.59937	13.44835	10.84021	17.53327	14.03358	14.10349
Aldh18a1	16.83031	16.45212	15.34461	12.64759	12.90916	13.58578	14.71749	11.95045	14.09741
Acp6	17.07087	15.87915	15.85578	18.92488	17.83563	15.06761	23.26858	15.54508	14.07923
Rpa3	12.16509	14.55925	14.68671	12.35617	12.74449	14.78558	9.340114	14.89161	14.07893
Als2	19.03181	15.39664	18.23318	16.31419	15.5363	12.31313	19.5356	13.59898	14.07459
Wdr62	15.65553	17.80138	17.56484	18.42852	16.6358	13.86042	26.21778	15.81356	14.0728
Pdcd4	11.7875	18.88938	16.36319	13.79714	16.81019	15.07363	17.57646	16.3082	14.07242
Zfp637	14.65933	14.28694	16.28473	14.38729	14.9164	15.72023	16.58237	14.86638	14.06267
Map4k3	15.707	13.14589	15.45351	15.85952	11.99038	10.42495	17.48381	11.24474	14.06095
Trerf1	17.14508	17.11482	15.58123	17.57056	16.09373	14.74971	22.53498	15.96398	14.05469
Vdac3-ps1	15.79119	14.14897	12.6682	11.29421	12.10387	11.7138	11.56433	16.72316	14.04437
Ttc27	15.14777	15.24541	16.01423	14.95975	12.05508	11.44961	11.90283	15.40998	14.04163
Brat1	15.37761	14.36426	17.43226	14.15184	14.14045	14.66364	16.26478	14.08727	14.03672
Cass4	15.66728	14.23305	15.91898	10.43506	11.36598	10.05776	12.38014	12.98572	14.03581
Wdr3	15.71046	13.52382	15.87647	15.10008	12.17948	13.37476	19.74974	16.02074	14.03469
Pex16	15.05491	14.21215	12.07374	13.79365	14.69884	15.06062	16.29397	15.19017	14.03282
Scamp5	16.83006	18.37012	18.98116	20.56059	19.22764	16.49908	25.32684	12.82597	14.02687
Dyrk1b	15.61213	17.66351	21.51819	19.76702	21.04038	17.38058	23.6676	18.20161	14.02257
Nudt19	18.17169	18.12331	16.01025	11.12876	13.1035	11.73655	12.69977	12.60518	14.02033
Card11	13.15845	11.18718	16.16682	23.46137	19.97924	23.003	25.98754	20.7048	14.01887
P2ry14	9.873941	12.6515	18.10433	14.52339	20.04845	19.11826	16.05195	13.80766	14.01809
Elk1	18.68769	18.34185	17.95539	14.76075	13.63121	12.5134	16.82766	13.40389	14.01353
Nbdy	13.92428	19.18373	18.65423	12.55826	19.55275	12.21793	17.47054	14.12054	14.01179
Engase	16.22508	21.25467	19.12565	20.06179	24.03135	20.18111	23.87662	19.85768	14.0015
Gm10146	5.384713	7.079133	7.955613	7.788436	8.158725	8.827497	9.233453	7.224091	13.99666
Atl2	13.67461	13.62181	12.64062	9.93701	11.14515	9.773178	13.0632	14.14627	13.98867
Rrp8	17.46777	19.04185	15.9403	13.06903	12.9038	11.62355	16.39908	14.12841	13.98369
Kcnk13	22.63887	17.37649	17.09886	16.28072	14.95472	18.12196	21.67628	16.51931	13.9818
Rfc1	17.0651	16.53908	17.74882	17.23831	13.67816	13.49885	19.33823	15.37484	13.97945
Ttc39c	11.64548	11.86948	13.20439	11.07173	11.1353	10.51143	11.89204	11.88435	13.9792
Trappc5	14.22747	13.56438	15.75562	14.25618	15.91226	15.60202	12.93008	14.06495	13.97567

Mfng	17.61351	17.56661	18.32729	13.58727	18.46934	17.80209	17.39535	15.55016	13.97241
Cables2	13.99031	13.41195	14.37953	12.13923	12.40149	13.95921	14.8402	13.20945	13.96193
Rps15-ps3	11.83129	10.11411	13.41036	15.2385	16.09714	18.75636	14.79034	12.55193	13.9611
Vps26b	16.09987	16.86951	16.65029	15.36801	13.66633	13.44156	16.31488	12.87883	13.96082
Ezh2	17.33591	16.80757	16.92347	13.78188	12.05948	15.62718	15.51424	13.2833	13.95539
Tctn3	21.68582	21.83847	24.26225	16.52987	15.51009	17.16716	24.46285	19.96415	13.95326
Atp2a1	25.02771	33.5761	17.58429	25.78494	17.78883	27.21344	12.04257	14.48449	13.94662
Gm14253	10.96839	11.21663	8.783682	11.38117	9.878449	11.4295	10.84349	9.534898	13.93847
Ggcx	15.7709	14.32511	14.10908	15.26902	12.33897	11.98113	16.26695	10.59913	13.93346
Nsdhl	14.99677	16.22818	18.44817	13.91858	14.80646	12.38923	12.60659	15.89661	13.9121
Srek1ip1	13.75014	11.81234	12.72036	12.92731	13.75026	13.12029	13.92077	13.77581	13.90928
Tpgs2	18.65392	18.77837	21.67399	14.87922	16.24572	15.22109	22.03769	13.15407	13.90458
Polr2l	15.8584	11.58089	13.07309	12.53648	16.8728	14.05174	14.05876	12.85605	13.88128
Osbpl7	16.19135	17.41802	19.18275	16.62358	18.71543	18.65897	21.25433	15.17858	13.87542
Pomgnt1	16.71606	16.05956	16.59103	15.0233	13.30041	13.8148	14.98059	12.55095	13.87486
Kctd5	17.91527	17.14747	17.39834	14.44938	13.64555	14.04271	15.55465	14.95181	13.87062
Hspb11	16.54584	14.13084	16.45266	12.29259	17.38822	17.23569	17.8872	14.75736	13.86642
Dusp16	15.06963	12.1223	10.37746	7.286513	9.258221	8.952626	9.563488	11.99254	13.86568
Pwwp3a	14.72539	15.10961	14.41423	15.3095	13.19433	14.79542	17.18247	11.5096	13.86423
Impdh2-ps	15.93429	16.18984	16.06895	8.813802	12.04567	13.13919	11.01357	11.88396	13.85795
Smagp	19.1901	19.88618	20.22975	22.69283	35.12589	33.4144	31.09102	24.41601	13.84098
B3galt4	12.86641	14.72543	16.03215	14.01784	12.45471	15.11689	11.5904	13.82318	13.83757
Thap3	13.09259	17.15534	17.64143	17.86742	17.42563	19.80785	15.62673	17.36814	13.83588
Phlda3	13.15095	16.24417	16.17604	9.732808	12.92072	13.31484	9.178617	15.21948	13.83065
Wdr77	13.54355	13.52306	15.80785	12.72293	13.5823	11.8609	12.69168	13.86041	13.83022
Cep63	14.11701	14.2305	14.7549	13.67645	12.49278	14.65183	13.43216	11.45062	13.83012
Gt(ROSA)26Sor	7.434575	12.08335	11.84162	10.95249	13.11224	8.148458	9.581289	11.88479	13.8217
B3galnt1	11.54246	13.53604	12.84004	19.67277	14.34669	13.25218	18.45061	12.31669	13.82116
Pik3r1	16.02432	15.9437	15.91198	14.2408	13.47606	13.27883	21.46954	14.95775	13.81245
Cirbp	13.74333	15.56079	15.33854	18.08975	14.73302	16.14769	19.00784	15.86635	13.79467
Hivep1	19.51384	15.87398	20.06086	14.88878	14.84489	11.74939	20.49584	14.11673	13.79338
Fbxl14	14.44096	13.48516	15.6911	13.15796	13.11518	12.62546	13.14635	12.03525	13.78857
Klhl20	18.15419	19.3426	21.09967	16.60567	14.4397	14.61724	17.29364	13.42781	13.78847
Gm35154	10.48126	12.61314	16.54672	13.60961	22.19191	17.94358	15.39884	17.73161	13.78726
L1cam	23.42229	22.05686	22.57512	12.71767	14.15314	13.58551	16.21559	14.81631	13.77883
Las1l	14.32597	13.13282	13.76997	10.89755	11.45842	12.99038	13.43234	13.068	13.77656
Smim19	11.28561	15.12589	15.00962	12.32494	13.42593	15.67317	13.2604	14.55288	13.77546
Tspan17	17.50935	22.52939	20.07644	12.4086	15.12558	16.6026	19.32023	17.09326	13.77345
Fam172a	16.30025	15.44187	14.83969	14.45547	14.21343	11.41485	15.60248	15.43754	13.77298
Mettl17	12.12601	13.5586	13.32308	15.01316	14.28132	14.5305	14.61576	14.50461	13.77093
Fadd	13.28123	10.16991	15.76968	14.8553	11.69427	14.20727	14.42412	15.32453	13.7605
Gdpd1	14.3172	17.20512	17.22958	15.98745	15.02145	15.71447	21.37153	12.93863	13.75741
Lyrm7	21.12289	18.02454	12.58369	14.63496	11.40217	11.35357	12.45266	12.19732	13.73627
Acss1	14.70019	14.2303	14.87159	18.72863	15.51345	14.23619	21.49074	13.482	13.73125
Ankzf1	14.60492	16.83411	17.2348	13.81957	16.46046	15.98309	20.26584	15.97428	13.72713
Snord83b	16.55942	11.48893	13.68111	15.75072	8.91405	16.64525	16.37086	15.08071	13.724
Mob3c	17.17125	15.10178	16.96265	15.05728	15.58799	14.94119	22.13966	14.7995	13.72021
Ermp1	18.041	16.11785	19.50216	13.99642	12.66149	12.64583	14.21832	13.9586	13.71853
Prpf18	15.41585	14.52323	16.72185	13.86293	14.1644	11.95904	15.09869	13.40624	13.71612
Nxt2	14.04612	18.15946	18.39102	11.85321	12.96808	11.06505	13.90955	11.15517	13.71056
Enox2	14.33702	13.94609	14.43302	18.14917	12.8684	12.75688	17.64515	13.89721	13.70941
Chchd4	17.81143	17.09786	17.8135	14.83351	16.28487	12.68302	13.3005	16.78864	13.70802
Patz1	14.26305	14.63485	17.08243	13.45357	15.34486	12.94576	18.98812	16.42991	13.70584
Rps4l	12.60759	11.5164	11.84309	16.34493	14.70057	18.41485	21.2508	21.25883	13.70066

Ankrd54	14.43447	14.45641	15.55594	12.03658	14.1219	15.36342	15.66938	12.96513	13.69965
Dis3l2	16.44866	13.907	15.2997	11.48771	12.1628	13.08289	17.7934	13.64138	13.69858
Kmt2c	19.27027	17.85372	18.67205	14.86848	13.0647	9.323362	22.41384	12.77261	13.69689
Tspan5	17.80832	18.59103	16.02935	13.5894	14.71134	13.65818	13.52425	14.15396	13.68902
Dusp23	19.24878	15.80285	18.35108	15.75628	20.34442	19.46496	15.4466	15.94276	13.68477
Ccdc174	14.60889	13.59447	14.23191	11.79735	14.57446	14.93961	14.25113	13.84901	13.68299
Ago4	13.88618	16.38079	16.25576	14.44473	13.79386	10.96755	17.17397	13.58656	13.68202
Urgcp	15.04133	15.62772	15.53901	12.45875	12.71952	12.33535	16.99807	13.64413	13.67408
Larp4	16.78369	14.58406	15.91786	11.96107	10.88055	11.16287	16.12468	13.42037	13.66946
Slc30a6	12.38296	15.52953	16.27759	12.58056	13.90278	13.62372	14.9835	12.58855	13.65712
Appbp2	16.53699	13.18518	12.71515	12.77987	11.49417	9.478183	13.99719	11.64416	13.65387
Cdc20	17.46423	23.39927	21.84837	14.2454	14.62024	15.863	16.21945	9.645227	13.65361
Trrap	17.29024	15.29028	18.04757	16.58376	15.18505	11.71274	22.43433	13.25589	13.65076
Lrrc47	13.9779	13.45211	12.73835	14.14317	11.49283	13.12264	17.09658	13.24615	13.64901
Alg2	13.94317	13.9246	14.63274	12.33067	12.00451	11.02025	14.14196	11.34593	13.64346
Gclm	17.47155	15.69054	20.39031	13.51582	13.40351	13.55913	17.17281	15.00428	13.64286
Pura	14.89158	14.84264	14.2316	15.28077	13.71219	12.42689	16.10393	13.87658	13.64233
Pgam5	18.02538	17.4181	16.74552	14.39264	14.52216	14.96533	16.91892	15.94153	13.64203
Arid4b	12.61686	13.99347	11.98741	11.98377	11.79863	11.17482	13.60223	11.94	13.63178
Aifm1	16.22031	14.3134	17.19403	14.79152	13.16999	13.96316	16.53273	13.16596	13.63086
Trip6	11.18211	9.518757	12.09698	17.91645	15.53959	14.88502	18.5893	13.16563	13.62965
Gtpbp6	13.55153	13.47157	13.28908	13.65063	14.16344	13.46786	14.82472	13.44696	13.62869
Hdac10	16.98753	18.0222	16.05099	17.39484	15.33084	14.08518	15.66742	13.81416	13.62214
Ccdc125	9.895264	8.642967	11.18725	13.59853	11.58994	10.48651	13.43518	11.9375	13.6194
Cfap410	14.44789	15.90905	15.33224	13.13179	16.28165	17.29065	19.62862	15.09404	13.61435
Cpt2	16.91451	14.44611	17.45912	15.95179	13.88491	11.47517	15.09254	13.45391	13.60282
Golim4	12.21201	11.47398	14.22418	11.52655	10.34224	11.15556	12.87826	9.913699	13.60264
Tmem71	10.74646	11.43615	13.95116	12.25056	13.66446	13.07537	14.59385	10.32191	13.60224
Cwc27	17.56716	12.85415	12.21309	14.8938	13.67607	15.81016	16.24052	15.29683	13.60198
Lmnb2	16.87637	14.81957	15.70748	14.23859	12.00748	13.80788	16.60488	11.95237	13.59559
Snhg5	12.33785	12.7972	12.28444	15.04965	10.65372	11.46036	12.58176	13.20867	13.58825
Orc5	13.81098	16.56778	13.61844	14.80915	13.62885	12.07951	11.66598	13.96795	13.586
Tex30	11.17763	15.18805	10.20359	11.04042	13.57474	11.37734	9.769349	17.49892	13.58472
Cmbl	9.795646	9.558334	10.06156	16.37503	9.581409	8.177319	14.99489	8.771872	13.57522
Arid3b	12.1986	12.69797	11.95557	13.63274	13.86096	13.62336	15.80001	13.83557	13.57466
Fbxo28	15.26719	13.71852	15.30282	13.34147	11.62794	10.80027	16.51414	12.68799	13.57232
Atp6v0c-ps2	13.89621	11.07761	14.534	12.01809	14.53942	14.07251	14.56472	12.85787	13.57077
Aktip	14.23368	15.56663	14.96849	12.8479	14.1669	12.5132	15.15319	12.06872	13.56597
Zbed6	16.55281	17.99949	15.63564	16.73534	11.56782	8.088922	21.58259	10.12007	13.56023
Plk1	25.36529	27.07085	30.42686	17.25536	14.76167	16.64916	17.7277	10.96655	13.55539
Rnf215	15.53898	12.61725	15.85474	14.92127	14.61977	15.64729	15.80075	15.27284	13.55342
Smad3	14.1892	9.940144	15.44634	10.35217	9.33516	10.04944	14.65214	12.3143	13.54774
Zfp292	13.43149	13.01895	13.19992	13.5321	12.57	11.28373	16.26642	10.8672	13.54452
Trim56	15.72773	9.901154	13.53524	13.19864	10.65489	7.211987	11.7874	8.562673	13.54062
Rbm45	15.9674	16.91057	17.54456	13.45173	13.11876	12.99535	14.04519	14.93788	13.5405
Aven	19.21172	15.08385	16.14789	12.19411	18.78365	15.30934	19.01132	15.08071	13.53806
Relch	13.78868	12.78651	13.79029	15.62494	12.46884	10.56789	18.05814	12.46503	13.53565
Snrk	15.14308	13.37236	13.46864	13.74374	13.08443	12.8032	16.38306	11.58585	13.53276
Tfrc	16.40015	14.8935	13.77191	14.84961	10.82403	9.471328	16.08988	13.01003	13.52738
Gtf2a1	11.6117	11.86253	12.0612	13.32902	12.82159	11.53989	13.05843	13.31851	13.52734
Pck2	12.78342	18.63869	19.92368	12.90539	16.23305	17.84257	19.65078	14.68354	13.52374
Ubr7	14.03452	14.6272	14.66795	11.9525	11.39877	11.63661	14.1348	11.50908	13.5226
Ppp6r2	15.94647	14.5431	16.08212	19.52877	18.32152	12.54467	22.09513	15.89588	13.51536
Lars	20.38626	17.52904	17.14984	14.86712	13.54612	13.52266	16.58782	12.22445	13.51528

Ccdc32	13.07017	14.71971	13.80605	16.93985	15.35006	16.19506	15.78541	14.57635	13.50678
Txnl4b	9.855656	11.32138	13.08373	9.742113	10.95082	12.12514	10.55011	10.94748	13.50289
Nop9	14.71397	14.45814	13.68732	11.54971	11.5859	13.4463	14.51909	13.91262	13.49921
Rab10os	10.79828	10.718	11.4196	11.66258	13.97465	11.56263	10.78676	12.49464	13.4925
Slc39a9	18.52326	17.31559	19.77082	16.28822	14.44834	11.9587	19.07046	13.70292	13.48894
Eml3	15.82353	14.39219	14.74805	14.03582	14.36264	13.72283	16.16245	10.751	13.48814
Sergef	12.61384	9.849315	11.26848	13.88232	11.02999	10.6686	15.68156	13.37726	13.47675
Gm7072	11.08437	9.90567	12.46528	14.39513	12.61231	12.45665	12.39556	14.81803	13.4723
Rad51d	15.22776	16.31101	16.83942	15.36656	15.45325	14.11193	17.33581	14.34506	13.47016
Stn1	19.61052	17.28031	20.16757	18.49481	16.03536	15.63067	18.91947	16.34113	13.46592
Arsg	10.31226	10.2848	15.43141	16.92376	16.50418	12.18945	19.75379	16.35149	13.44952
Tet2	15.02795	13.95954	14.55373	16.55767	12.78417	10.64168	22.85992	12.92549	13.44487
Fryl	19.51022	17.5935	19.5835	15.70589	13.32926	11.06039	18.90908	12.6717	13.44432
Lrrc57	15.33786	15.26631	16.78736	14.70067	13.34631	13.50041	17.57139	13.10765	13.44418
Gpc1	11.95422	15.49298	15.92032	8.755009	7.272978	9.733414	9.226757	8.806831	13.44366
Mex3c	15.05524	12.80625	13.72523	13.20088	11.2197	9.2608	16.10804	13.76484	13.44246
Ercc6l2	18.66508	15.73278	16.64728	13.95502	12.50319	11.59313	15.18829	13.92502	13.44167
Snord52	12.21517	8.43414	3.75895	8.409283	6.264241	3.475755	8.740374	13.41927	13.43324
Kdr	11.43238	6.643109	12.19227	16.01715	7.932925	10.72534	6.157398	7.418432	13.43279
Col4a2	17.28464	13.73261	13.35826	32.74319	18.39356	24.31425	9.460833	13.78891	13.42643
Ormdl3	13.34569	13.03685	13.1828	13.51619	12.3449	13.96358	14.72811	13.46305	13.42483
Pgm1	16.4604	15.2795	13.6117	14.52589	15.11117	15.66137	17.89642	13.43307	13.42441
Lpar6	11.97264	13.91223	15.86054	17.89187	18.33404	16.5975	17.34263	20.14631	13.42344
Arl5a	17.89079	20.12395	21.45656	14.53285	18.14319	12.86603	19.08468	14.70456	13.42224
Vps8	16.45003	14.58583	15.32944	15.01633	12.90273	12.21401	16.83788	12.94196	13.42005
Dnajc25	12.59266	11.59072	11.77475	11.04361	11.21284	12.48713	13.17196	11.38275	13.41902
Aurkb	20.96208	24.19837	23.41931	13.99931	14.13337	16.69777	20.03243	12.22189	13.41774
Yaf2	15.5881	17.57843	15.34294	14.42647	14.49316	15.87119	13.59073	15.34101	13.41529
Gm8186	12.63873	13.60776	10.0547	19.42317	16.27729	12.08635	17.21909	18.32345	13.41468
Morf4l1b	11.08761	11.5431	11.0889	10.47745	10.1892	11.57264	11.19154	12.03811	13.41019
Id1	8.606552	8.761231	7.182854	10.97829	11.58856	12.95407	8.281832	13.789	13.40735
Ctla2b	11.74304	12.66506	17.13863	12.28828	11.89919	14.20586	13.47773	13.26062	13.40456
Rbm27	15.75239	15.02899	15.24632	12.52397	11.60795	10.96391	16.41449	12.29012	13.40296
Mdc1	20.99631	18.92411	21.3804	18.27896	14.02705	12.61412	22.52312	14.03822	13.40281
Noa1	15.45429	16.01335	16.94873	14.67354	13.27416	15.24887	12.41498	14.96611	13.39863
Elp3	14.26053	15.41266	16.21859	13.14968	12.57553	14.90625	15.58813	13.96854	13.38647
Lonrf3	14.06637	13.5713	12.53332	14.66078	9.49945	11.00475	16.74626	13.02015	13.385
Fbxl2	14.03432	14.04677	16.13385	11.52215	11.17808	11.08484	13.93735	13.26694	13.37595
Isg20l2	17.22738	15.93925	16.2328	15.81605	14.91063	12.90102	16.59721	16.47514	13.36916
Zfp609	20.54178	19.84533	19.56647	16.31676	12.5161	11.34062	21.79371	14.1146	13.36267
Pex3	16.41866	14.37312	15.53731	11.85581	15.29286	15.18346	16.5546	12.19233	13.35283
Herc1	16.86512	15.93937	17.62136	17.25418	12.98109	10.72334	21.33355	12.38189	13.35004
Tmem201	15.27246	12.57177	13.81349	13.35564	10.61415	13.27197	18.14765	10.45976	13.34553
Zfp809	15.69402	18.44426	17.51995	13.11757	13.29054	13.14045	17.09734	16.07301	13.34074
Lmbr1l	13.78916	12.52774	14.20014	12.87242	15.66659	10.88279	15.60029	13.46964	13.33736
Metrn	12.7715	13.30541	11.87806	9.868757	13.63544	12.4125	11.56049	10.72688	13.33534
Uck1	14.88561	15.73962	15.61003	15.37707	15.05779	16.17141	18.28278	15.54854	13.33332
Sms-ps	17.29227	13.73108	12.92481	11.4636	9.44081	12.66406	12.71181	11.43038	13.33182
Rpph1	9.893562	7.825728	3.582568	10.85588	16.7331	13.0929	6.346856	8.932418	13.33128
Rcn3	10.36584	13.20998	14.78264	14.04912	15.50319	19.10101	11.03106	13.96323	13.31912
Dnajc17	9.789658	11.08003	12.92605	13.01622	12.15206	12.3591	12.27814	12.51489	13.31419
Fbxo30	10.78923	11.79559	10.03364	9.847272	10.17259	9.99754	14.63736	12.28983	13.31239
Pign	17.22634	16.62277	19.40325	15.7088	15.62211	11.21878	17.70796	14.168	13.30928
Spin1	13.67935	14.5214	14.72202	12.39507	11.5918	10.0955	14.91072	13.50692	13.30475

Lyrm2	13.36453	13.94603	13.65486	14.80732	13.63178	16.82211	14.87872	13.90253	13.28798
Gm31718	10.15006	9.22863	9.498948	9.282502	9.550336	7.690108	11.88091	10.01314	13.28705
Fyttd1	14.74114	13.75178	14.0241	13.26252	12.36796	11.74652	13.87106	12.03001	13.28501
Pxmp4	18.35347	20.1878	23.30331	17.19919	21.36401	18.19503	19.28148	17.25857	13.28134
Lrrc14	14.93363	13.47705	17.22245	12.98942	12.34167	12.56051	13.10692	13.98735	13.2754
Pak4	14.5204	14.59638	15.19264	12.91516	13.84732	14.15433	16.45288	14.51791	13.27325
Rnaseh1	11.57934	13.1305	15.15007	16.79852	11.78338	15.32887	18.11568	13.29001	13.26604
Arfgef2	14.41986	13.29859	14.92957	14.52851	11.50188	9.77668	18.91339	11.05101	13.26533
Zc3hc1	13.59932	14.73951	16.55008	15.73101	15.00035	11.96667	14.37829	11.16425	13.25895
Maged1	14.29535	17.69377	17.72255	13.37908	11.38793	12.93539	12.10402	10.29092	13.25333
Cfap36	12.15779	17.17163	13.03292	12.45165	13.93634	12.15202	14.25866	13.68948	13.25272
Clybl	16.38355	16.58111	17.83656	15.81084	12.20644	11.46108	13.62155	13.64171	13.25256
Eola1	7.185444	10.56639	10.33356	10.37879	10.53974	10.57359	10.78742	8.539075	13.24684
Spred2	20.42252	16.51784	19.80713	13.93544	10.056	9.996426	14.3245	13.28751	13.24614
Bpnt1	15.63982	13.2397	15.0235	12.42574	11.41453	10.18886	16.4145	12.79265	13.24136
Mrpl50	16.13319	16.66864	16.53053	12.42532	13.9089	12.82294	15.06695	15.18469	13.24076
Ivd	15.50955	18.03593	18.10178	17.0208	16.18285	13.85215	19.49713	12.12229	13.23537
Tbc1d10c	8.325279	8.144051	6.571673	8.532396	11.17945	12.27113	8.868333	6.711695	13.22455
Tdg	15.72322	12.62434	13.28397	9.915429	10.84804	9.098648	12.26138	12.03368	13.21837
Dhx57	15.09019	15.43239	16.90176	17.35411	15.84688	13.79894	21.60365	13.18467	13.213
Mir5107	23.30919	27.01693	19.53159	21.29927	22.7994	26.79886	42.9735	27.59084	13.20935
Cd2	16.67019	16.09417	20.64629	15.03478	20.07639	17.34308	11.61988	12.27285	13.20935
Snord72	5.543806	0.921508	9.240751	5.512752	7.039814	6.835651	8.594701	5.278247	13.20935
Tmem106b	14.3016	16.30896	17.7674	15.27292	14.08947	11.11833	14.78142	13.80745	13.20501
Uvssa	14.83631	13.6523	13.30777	15.51396	11.31956	9.399442	16.83076	10.50967	13.20284
Psd	15.18187	17.77913	11.99346	10.68021	15.53579	11.63131	17.86129	13.87798	13.19973
Tubgcp5	15.55201	15.10927	16.45343	15.62967	12.44442	12.10541	19.40108	12.98151	13.19807
Gm43828	13.98347	14.71543	12.89236	15.16509	16.17711	8.567206	12.42184	12.94648	13.19622
Cd4	10.8052	9.980451	15.71849	20.78729	27.89029	22.45432	21.574	16.8635	13.19472
Dcaf6	14.79902	17.60695	17.13157	13.84706	14.31638	13.20075	17.19241	12.86971	13.18853
Pitpnc1	17.43234	17.54709	18.32325	15.99893	14.95752	14.43968	21.10704	12.48163	13.18769
Tbc1d7	15.10027	13.32237	16.42213	13.80813	13.32536	13.13422	14.84291	14.37694	13.18419
Pcolce	18.33527	22.38204	17.22058	24.78769	18.01122	20.75346	11.76489	16.66815	13.1836
Gm12966	11.70649	10.26239	7.606375	8.82337	10.19045	9.239851	7.436796	10.40031	13.1827
Tbc1d2	11.10693	7.802314	10.72185	14.23327	8.775238	8.610062	12.05853	9.600156	13.18173
Ccdc69	11.17356	13.71547	14.69143	11.26637	14.21358	14.30718	12.47716	14.24643	13.18142
Pomt1	16.37432	17.57545	19.52166	17.49883	17.13993	14.31147	19.16813	13.64121	13.17961
Chchd10	16.72983	21.96663	17.66913	13.37413	12.94302	18.80815	11.2368	11.06513	13.17585
Rfwd3	18.72891	17.91994	18.34423	14.88927	13.67796	12.78511	18.44163	13.00851	13.15286
Adsl	15.9952	12.51185	13.86113	12.43043	11.91951	12.05723	11.88563	12.02963	13.15241
Esf1	12.43761	13.15431	11.58908	12.28668	9.759648	11.13917	10.74338	12.58874	13.14704
Dmxl1	16.55942	12.91059	14.13054	15.20295	11.13681	8.794057	19.92171	11.16101	13.13451
Ptges2	12.46788	13.96424	11.64562	9.009058	10.73824	14.70786	11.50657	9.680391	13.12814
Polr1a	18.60561	15.76294	17.3221	14.3012	11.8059	10.57191	17.20126	12.02085	13.11636
Tdp1	15.53884	13.64101	15.51367	13.06428	12.30683	11.82518	14.16418	12.61129	13.11293
Ppp1r16a	15.15427	15.27113	18.28633	16.09939	15.56937	13.07714	15.68597	14.68562	13.11277
Prps1	16.16827	15.11739	14.963	11.47211	11.97659	12.74546	11.98181	13.46955	13.10903
Cyria	14.82586	14.21144	17.21507	14.48558	12.30395	11.40718	18.22598	14.92489	13.10897
Casp7	13.3995	15.6225	14.01448	14.63812	12.91881	13.52586	14.79995	11.32016	13.10817
Dis3	18.74306	15.90132	16.5238	15.09804	10.20039	11.76167	15.75538	14.02125	13.10785
Maco1	17.76571	16.13751	17.27365	12.15958	13.72411	14.29699	16.2128	13.89631	13.10634
Naa25	15.21769	10.46702	13.43922	10.53848	9.270134	8.687291	11.48512	11.55964	13.10148
Hmg20a	14.80114	16.71334	17.09833	17.01521	14.19453	12.73521	18.5519	14.33027	13.09717
Rbm18	11.24332	11.67015	15.3669	10.95264	11.10057	11.3504	11.09986	11.07538	13.09477

Dnaaf5	13.58252	13.62257	13.62231	10.65698	10.86174	9.681091	13.46562	11.60414	13.091
Tnfsf14	13.58514	9.969443	9.047926	10.26452	16.35655	14.94956	12.53873	12.56725	13.0892
Hspa4l	17.68471	21.41379	16.76737	12.24023	11.30763	11.18142	14.89619	12.68381	13.08312
Selenbp1	15.92405	20.27864	18.55828	15.1478	19.52138	21.11547	21.76304	18.19977	13.07608
Phtf1	12.53126	12.20351	14.51862	11.66057	10.14571	11.0674	13.14582	10.75577	13.06869
Fxyd2	23.35303	30.68317	24.98049	14.23623	18.56654	30.23461	16.58074	16.4341	13.06419
Decr2	14.8934	13.47891	15.30063	13.38543	13.41203	11.67327	14.75053	11.73516	13.06122
Tomm40l	12.2452	14.6669	13.74299	13.95807	14.71836	14.45536	14.83318	12.22094	13.05924
Gpn3	9.358135	11.89296	10.93884	12.28983	11.31936	13.58367	14.24288	11.87981	13.05882
Traf3ip3	9.252746	13.32081	13.07037	14.21724	12.07323	17.16164	13.91253	16.27522	13.05365
Gar1	15.39704	14.52952	15.35521	9.71563	12.17151	11.57651	9.872748	14.03657	13.05346
Tnrc6c	16.32224	15.76363	17.45325	15.75387	13.79524	12.33065	19.94334	13.95964	13.05287
Slc7a11	6.286326	6.166915	5.572765	2.949604	6.342451	3.677257	4.382473	7.216545	13.04991
Zbtb2	12.21182	13.79127	14.44036	12.16127	12.72503	11.69169	17.1133	13.06311	13.04247
Hcfc2	15.19414	12.23859	13.60142	11.67406	9.593472	9.49396	14.20591	10.69449	13.03092
Rnf135	11.14302	12.58695	13.39165	12.76412	11.28676	9.166073	14.41199	13.24691	13.02599
Sbf2	21.11293	20.0347	21.31879	17.4787	15.60788	11.53824	25.33013	14.44871	13.00991
Tti1	15.35364	15.00968	16.3685	13.55524	13.54764	12.17784	17.02039	11.60085	13.00592
Apbb3	10.89273	10.69019	8.736077	10.48565	9.644943	7.17679	12.44514	9.741397	13.00205
Usp31	18.73281	17.44201	14.2644	14.0912	9.260603	9.205116	18.05149	13.36015	13.00072
Tmem223	17.49115	17.00108	15.12123	15.18218	18.56422	19.73931	14.55431	12.27827	12.99743
Exosc4	12.64323	10.60069	13.23354	14.60617	11.4215	12.17923	11.14573	10.35587	12.99522
2510022D24Rik	12.38671	11.0355	11.44393	14.11986	14.56637	15.43609	12.3339	12.44055	12.99339
Zfp746	14.22087	14.33801	14.45567	15.28487	13.63261	14.08524	15.50114	12.82938	12.99086
LOC118568010	11.44273	7.477003	7.103211	8.435885	11.83741	17.14994	14.68134	10.8946	12.97431
Slc41a3	14.20115	14.40385	16.40021	12.65615	13.01464	13.38056	15.5084	15.12808	12.97313
Prdm4	19.1617	16.21564	18.20741	12.93094	12.97593	11.51344	19.978	14.55352	12.96947
Creb3l1	16.08705	15.5086	16.08892	19.80804	12.1825	13.04824	15.56709	15.7348	12.95924
Fdft1	16.02961	12.54647	14.6252	11.91291	11.14181	10.05583	11.14182	16.17211	12.95472
Mzt2	14.1667	15.28237	13.73462	11.85925	13.69873	15.23985	13.22262	12.79992	12.95102
Smarcd1	16.77056	18.36929	16.69927	15.45683	15.48387	13.56181	18.01443	13.89288	12.94761
Ints10	13.24552	14.73854	13.1001	11.87715	11.0955	12.44316	14.05873	11.59176	12.94426
Taf4	16.60068	15.08735	16.65385	14.34233	13.87061	12.85238	19.13023	13.53709	12.94077
Stard9	16.26947	18.18406	18.94644	18.89506	13.98355	10.30265	25.45967	13.27058	12.93746
Snhg8	8.968961	10.07202	11.0889	10.4637	10.29068	14.95571	11.09465	14.85978	12.92329
Pold4	8.592487	12.60722	13.71652	14.37752	11.40953	11.84269	12.97958	14.63121	12.91406
Abca2	21.51818	18.93528	19.90526	16.01421	14.38599	12.79153	20.92438	13.97106	12.90929
Gab3	11.40529	10.56986	11.58892	14.70758	12.94545	12.64226	22.47244	15.4455	12.90533
Sqle	17.511	13.27937	13.1146	8.425604	11.31673	5.820751	8.56968	12.21603	12.90171
Ptgir	19.38294	18.88414	20.81207	12.82931	14.11198	13.77811	10.04822	12.55524	12.89854
Pam	20.02434	21.25418	20.19163	15.69683	11.86044	12.37068	16.05913	14.07114	12.891
Uhrf1	20.40225	20.67298	18.4978	12.91494	10.04136	14.78345	16.25296	10.31756	12.89096
Fbxo34	12.34949	10.05281	11.7353	10.86483	10.02111	10.86897	13.71812	10.18288	12.88851
Kif23	19.1901	19.84786	21.8757	12.73941	13.84271	14.60792	16.71192	11.1486	12.88758
Lrmda	10.94596	12.13693	12.8147	14.34212	13.123	10.24157	17.56873	13.42551	12.88717
Cand1	16.46461	15.10065	16.22338	13.65932	11.8538	10.21725	17.09378	12.62176	12.88591
Cdc14a	14.83199	12.25266	12.84656	9.684189	13.45951	11.95635	10.5861	11.64358	12.88262
Faap100	15.88933	13.56576	14.7561	9.948999	12.40559	13.43448	14.23878	11.13835	12.88068
Gm15501	11.71227	10.4351	14.83726	15.99475	11.89828	16.67995	15.25257	17.17289	12.87446
Aarsd1	17.45426	18.40253	16.95754	14.00917	17.05862	16.67919	14.64664	14.60025	12.87268
Ankrd46	13.01006	14.75899	15.20253	12.22586	13.02933	12.54811	12.93826	10.51393	12.86846
Ccdc102a	12.15114	11.35086	14.43867	13.15978	16.10999	14.10743	15.84151	14.24943	12.86536
Hars2	11.56968	11.69914	12.64242	13.50225	10.89131	10.74881	13.50794	10.22501	12.86476
Pus10	11.76645	12.57627	12.11756	13.66426	10.99073	10.00618	15.15899	13.12217	12.86135

Nav2	14.13175	11.54801	12.35461	18.98543	10.93537	10.57348	23.83457	9.515129	12.85764
Rps27a-ps2	6.738328	6.414917	7.24965	6.903631	4.958985	7.458791	7.06992	8.845681	12.84445
H1f4	8.50712	6.928985	8.64991	9.305413	12.55824	13.76746	16.55927	16.80669	12.83773
Nostrin	14.50343	15.99387	15.21044	11.76948	13.78371	14.17793	15.02171	13.39997	12.82943
Dnajc24	10.94103	9.241184	14.18835	10.9318	11.31755	10.11793	13.90518	14.80302	12.82267
Dnmt3aos	13.64937	12.09604	13.69099	20.02104	13.95256	12.51146	14.64512	11.54736	12.81831
Cisd1	17.80912	15.92824	15.57043	15.02439	13.62368	13.28171	12.7065	14.16649	12.81238
Dusp12	12.08624	12.28677	13.30108	12.761	15.55514	12.04007	15.43381	13.19562	12.80907
Aamdc	11.19961	16.59512	13.92113	15.59162	10.36163	16.1274	12.89825	14.77604	12.80907
Pelo	16.08127	13.64676	13.08519	11.88819	11.95694	10.50133	12.61139	13.12846	12.80601
Vps45	13.49007	14.4648	13.00602	12.84769	14.91872	14.52511	14.74998	13.62777	12.79687
Chd9	18.76239	16.79475	19.11521	16.66899	13.30921	9.321343	18.84266	13.00785	12.79166
Mfn1	16.58029	14.51466	14.37247	13.01925	12.69532	11.68545	15.47575	12.70882	12.77426
Prr12	17.21768	16.37315	17.09123	19.32243	16.55623	14.45519	23.11285	14.2422	12.75932
Nudt7	13.59127	16.90818	17.38454	19.13459	16.21428	14.02411	17.3003	16.61796	12.74915
Lpar5	14.37837	16.46593	17.55899	14.1305	17.3443	13.08918	22.59068	15.61222	12.74156
Amz2	16.1235	16.33582	15.98755	14.0168	13.3309	13.59969	15.78951	13.45803	12.74027
Akt3	15.95051	18.41383	18.26051	12.95586	10.82629	10.83018	14.21031	12.09306	12.73745
Casp2	13.25074	16.4799	15.67185	12.79915	15.55646	11.69821	17.04558	15.356	12.73463
Rnf19a	11.63096	12.7995	10.9389	9.047218	9.2849	9.238074	10.9536	9.996067	12.7306
Atad2b	15.75329	14.78179	14.74073	12.68469	12.4273	10.267	17.13179	12.7715	12.72382
Fam217b	15.68314	14.13787	17.11971	10.03418	14.21857	11.91742	14.82712	12.31591	12.72269
Prickle3	12.51382	11.79224	10.90485	11.48299	14.17456	12.16803	11.84	10.88502	12.71605
Rfc5	18.23368	17.22634	18.23579	11.85401	12.45193	12.65386	14.80474	12.47863	12.70541
9430038I01Rik	13.79792	11.00895	10.44821	12.44657	10.51279	12.39531	13.75152	14.40375	12.68098
Mir23a	13.30514	11.7953	11.0889	4.410202	11.2637	8.886347	6.875761	2.111299	12.68098
Sdhaf4	12.05175	12.84861	11.2219	17.85239	11.3988	15.14194	11.59705	13.76934	12.67464
Hnrnph3	13.15479	13.58834	14.47196	12.56721	13.78133	12.94719	15.14998	11.22746	12.67202
Dhx36	13.15991	13.2572	12.42555	12.22257	11.28116	9.805055	13.76349	11.2892	12.66902
Pi4kb	14.69138	13.48785	14.59578	14.45429	11.15646	10.23908	18.08035	11.56064	12.66688
Slc45a4	14.01311	12.93497	15.31942	15.53312	13.81084	13.35583	19.64155	13.02133	12.6659
Gbp5	9.39566	6.643642	12.49841	16.8795	13.68464	9.163981	11.94894	11.49648	12.66279
Smpdl3b	14.96664	15.94917	16.28067	12.35476	6.951296	12.43765	7.586052	13.78122	12.66222
Traf6	15.04284	12.33296	14.26642	10.89655	11.32722	10.08216	15.088	11.93228	12.65979
Sgsm3	11.73154	11.51968	10.82728	9.484736	10.61721	9.025293	12.4579	8.793885	12.65796
Serping1	20.6522	22.32884	19.86254	24.38333	13.02559	17.18302	11.23922	15.74774	12.65775
Ppwd1	13.64567	12.10622	13.64725	14.62236	12.41407	10.43429	11.07228	12.21637	12.65613
Mir7678	14.05472	7.008651	0	1.552888	1.487285	0	4.035071	9.664396	12.65121
Rbm12	14.17247	14.75899	16.39488	14.19682	11.99323	10.9839	13.93943	12.34429	12.64121
Spast	14.99564	14.70325	15.05766	12.46632	12.44481	11.1698	13.64238	11.77535	12.63778
Drosha	17.14004	15.01208	17.84616	14.19531	10.99441	12.64004	16.58593	13.45277	12.63377
Usp36	19.02425	15.17375	17.26285	12.09554	14.06083	12.91505	18.90422	16.23793	12.62356
Ogg1	13.41996	13.52738	13.13443	9.990749	10.49137	13.14038	12.23841	13.18708	12.61942
Ttc37	19.16154	16.6901	18.02263	16.04659	12.74687	11.15455	18.13844	14.55355	12.61814
Mettl5	15.17055	12.45281	10.73925	14.58893	12.4267	11.14257	12.77591	14.97881	12.61433
Tnks	15.88984	13.48799	14.33435	13.20015	12.24102	8.783239	19.29495	11.27074	12.61079
Rad17	14.07854	13.89882	14.20243	13.1285	12.43804	11.92659	14.4877	15.24698	12.60253
Xxylt1	15.00712	12.85415	16.40184	11.56781	11.62092	11.21134	15.87556	11.8415	12.6017
Tcf20	15.65545	14.40565	15.51158	14.1421	11.98209	10.94687	15.28528	12.4164	12.59738
Disc1	11.9947	11.27393	16.34784	13.91924	11.59903	9.878765	17.98891	13.08602	12.59496
B3gnt3	23.14533	24.58231	23.35893	18.74283	16.16847	15.28514	15.28492	15.009	12.58751
Aak1	17.03999	16.50116	17.5668	15.84124	11.36651	11.02994	18.60345	11.20601	12.57523
Mest	5.597224	10.79403	20.62619	11.03936	8.228354	10.22341	8.737526	14.55001	12.57295
Gm6254	13.82301	10.48529	12.70502	16.84318	9.456466	12.46801	12.57638	9.268212	12.5715

Hps4	14.25884	12.12272	13.3059	18.49852	15.21253	12.96996	16.69011	12.50013	12.56884
Jaml	10.96481	9.565018	9.386965	7.90859	11.80728	9.490956	7.675999	8.99195	12.56839
Ankrd55	11.2531	8.637417	10.26137	17.66275	12.92384	11.52878	16.3057	17.54164	12.56531
Aasdhppt	11.82728	11.248	13.25688	13.5452	12.24063	13.68033	11.95916	13.03873	12.56371
0610010F05Rik	17.53207	15.54499	14.07976	11.0429	10.4931	11.62411	16.1772	14.50685	12.56347
Rhoq	16.20642	17.70583	19.28855	14.52414	13.46224	13.96732	18.66842	14.25203	12.56202
Ppif	12.95665	12.03097	12.2879	13.40239	12.80074	14.08218	12.19843	13.32855	12.56175
Rufy2	16.14961	14.8608	15.34837	14.72828	10.93362	12.45929	15.24668	10.98691	12.55739
Amn1	8.37663	12.3796	10.43392	11.13152	9.936001	12.18305	14.2458	11.89056	12.55614
Zfp143	14.74923	13.9887	14.86514	9.500178	11.96261	13.78042	14.37859	11.39724	12.55183
Rpl15-ps3	9.068815	9.752516	12.30348	10.35111	9.312983	9.990941	9.047053	11.1121	12.5517
Prdm10	14.62354	16.80648	14.59667	14.88244	13.30166	9.535047	20.72293	13.16163	12.54922
Wrap73	11.5034	12.92415	13.44529	13.19615	11.3847	12.46438	12.39069	11.41421	12.54888
Gpatch8	15.41651	14.54011	17.05219	15.66826	13.00812	11.28879	17.12512	14.28324	12.54345
Lsm6	15.15631	15.25069	15.23913	13.00279	12.94235	14.31589	12.59327	13.04128	12.53987
Spata5l1	12.32222	10.93449	10.24984	11.96879	9.284019	12.29888	13.57317	13.16158	12.53923
Habp4	11.5325	13.59107	12.11701	12.62757	11.60047	10.0258	13.25867	12.00817	12.53525
Bod1l	15.18242	14.01398	15.53046	14.35714	12.09636	10.98244	18.22657	11.75752	12.53424
Cptp	12.68286	11.44442	12.89262	9.04986	10.96831	11.40131	11.96758	11.87705	12.52457
Sc5d	14.83936	14.23441	17.15846	11.15297	13.40507	11.80814	12.99332	14.76219	12.52216
Ing2	8.358436	11.2392	10.57612	11.3123	12.25864	13.48362	11.15048	12.07691	12.52216
Ppp1r12b	22.62611	25.55013	23.10017	11.535	10.52068	9.514317	18.23792	13.52246	12.5208
Gm1673	6.563492	8.416309	9.494725	14.33549	12.39037	8.99618	9.327582	7.811359	12.51118
Cdkal1	10.59427	10.84993	12.24017	13.17652	9.818793	9.651019	12.7801	10.35777	12.50857
Tmtc3	16.60358	16.30157	17.61974	13.14914	12.36639	9.451734	17.36615	13.32814	12.50814
Larp1b	12.06438	13.13807	12.40901	10.29047	8.573488	11.74267	10.66834	11.51161	12.50485
Actn3	18.72259	25.96948	13.92354	18.25491	11.59484	21.75357	7.561669	12.37746	12.50015
Mmp25	4.786132	4.928875	4.443668	5.61197	6.132121	8.679124	5.542889	4.749828	12.49613
R3hcc1l	12.85388	11.47022	15.34233	16.92735	14.0181	13.4932	16.93239	14.11827	12.49486
Med9	12.30935	11.19389	12.89963	11.25238	12.30157	13.3741	13.08365	11.40438	12.48597
Gm32695	11.73382	15.26424	10.88481	9.131553	13.60455	10.69381	14.58814	11.9813	12.48
Rhof	9.390582	12.56484	13.94521	11.70463	14.04349	11.88982	13.42212	10.34467	12.47584
D230025D16Rik	12.33973	11.65082	13.18055	13.10519	11.23375	12.79587	19.24782	13.00125	12.4743
Usp9x	17.87543	16.94528	18.18028	13.37548	10.90748	9.100898	17.38243	11.67573	12.47359
Ehmt1	15.63843	14.03369	14.40459	13.19602	12.75839	11.26698	17.39277	11.81778	12.47338
Ppm1k	7.11479	8.619789	12.41651	13.01029	10.68706	10.15633	13.76929	9.13805	12.46981
Katnbl1	13.28744	16.16714	14.15441	12.39079	10.95164	11.41646	13.07738	12.65095	12.46252
Dnajc1	14.92438	15.13453	15.42728	13.57335	12.08693	12.02854	14.78331	13.58716	12.4592
Kmt2a	17.76303	18.3162	18.04199	15.88057	12.5687	9.420357	21.12963	11.64854	12.45229
Hdac9	16.43278	15.12834	14.95376	14.20387	10.23295	11.45584	20.88484	14.9579	12.44347
Tgs1	13.06535	12.05966	14.23264	12.54635	12.76048	13.24328	15.72783	12.92731	12.44023
Copg2	14.57639	13.09177	14.46656	13.08487	11.22729	9.767321	13.1061	11.99739	12.43501
Snord100	8.967922	9.757142	0.81536	4.864193	3.1058	5.277525	7.583559	6.985915	12.43233
Tmf1	14.12726	13.82058	13.14773	13.4384	10.48809	10.50146	14.15339	12.14285	12.4282
Ube2o	14.53883	12.52212	13.41601	11.10315	12.16599	8.578261	15.23643	12.44477	12.42795
Katnb1	15.39227	16.0618	18.0281	15.62805	14.02206	14.17439	16.19873	13.83279	12.42749
Poc1b	14.36576	13.64162	15.68571	12.49962	13.045	11.83637	15.65724	11.91334	12.41733
Slc39a10	18.18368	20.38007	21.85622	14.50956	12.10144	10.80716	15.28711	13.06894	12.41679
Ino80	14.16053	12.54782	13.36811	13.76887	11.56683	9.351416	15.46523	12.58506	12.41521
Gm10557	11.7005	14.031	14.41836	13.15858	13.99561	12.94566	19.00336	13.39455	12.41281
Mkks	11.81819	15.21559	13.84578	9.920513	15.43614	14.10847	12.75404	13.21023	12.40479
Agap1	22.67065	25.10429	22.98517	12.39813	11.01138	10.67572	15.64718	10.76153	12.40462
4833407H14Rik	7.580493	4.918757	7.39869	2.58836	5.914835	5.658838	4.71977	6.304331	12.40417
Maged2	10.56597	12.42244	13.06925	17.33487	12.42022	13.90594	15.43168	11.22546	12.40398

Mettl16	14.48161	13.42594	13.85093	11.25672	11.32509	11.80772	13.21775	11.24395	12.4026
Dppa3	9.962586	9.26014	7.930327	13.27643	9.036436	10.96796	14.56966	10.06609	12.40193
Rgp1	15.01467	11.9873	14.51556	12.56272	12.29298	10.24998	15.86999	10.89476	12.40107
Ttc33	14.05846	12.92977	14.8891	12.79275	12.25231	14.47261	13.12508	13.4166	12.38772
Trps1	14.9208	15.0423	15.05427	10.35985	10.8916	8.111753	14.02901	12.05121	12.38332
Impa2	10.91527	10.40874	11.33149	9.64811	11.15549	11.42227	11.38043	10.54434	12.38044
Zfp646	13.93871	12.89058	14.95418	13.71663	11.29244	10.60502	15.93118	11.05631	12.37905
Cd276	19.30015	20.89174	24.52253	12.88091	12.35325	10.92058	11.72127	8.83285	12.37886
Gm13192	8.070098	8.048613	7.018292	8.024893	6.516283	8.760882	6.708944	11.85935	12.37331
Spaca9	10.24489	13.59602	11.9813	12.32359	15.1078	10.3146	11.61331	10.4621	12.36285
Mus81	12.19304	13.87802	15.19446	16.01902	12.08866	14.82028	13.17624	13.24851	12.36226
Inpp5e	13.12557	13.98916	14.38543	13.19106	11.98287	12.29613	17.62769	13.39897	12.36179
Tmem60	12.34045	16.18105	14.9105	12.64507	12.88644	12.51272	9.841013	10.67578	12.35858
Rab11b-ps2	14.08357	15.92953	14.27728	11.52203	10.69995	9.560834	11.64486	11.54997	12.35517
Cenpo	11.23447	17.90532	15.42165	12.55885	12.69264	14.70118	16.24076	10.73127	12.35205
Tnip3	15.19435	16.7089	13.96042	9.931438	11.80929	11.00433	11.67548	12.1698	12.35069
Cep95	10.56301	11.54831	9.595256	12.97118	10.51021	10.86099	17.26756	10.73199	12.34224
Helz	15.42419	14.79114	15.44345	14.70415	11.89652	11.25283	20.29022	11.58538	12.33788
Zfp445	13.57064	13.90972	14.36952	14.63476	12.30176	10.23857	17.63656	11.55603	12.33641
Pdcd11	17.56079	14.6579	16.61714	14.33262	12.5178	11.69256	17.59149	13.07987	12.33388
Gm6419	13.41762	13.3819	13.09776	11.50487	13.08487	9.36187	12.37305	11.78044	12.32873
Retsat	17.53797	18.53343	15.56287	13.25532	14.90088	10.83849	17.16313	11.61591	12.32783
Mrpl11	10.23354	10.89694	11.27359	12.28032	11.9264	12.66564	11.67034	11.24512	12.32017
Mzt1	14.35099	14.9745	15.01622	11.38716	11.37417	11.43063	14.42609	12.82127	12.31936
Batf3	9.732806	12.73545	12.61518	16.4918	23.06231	21.31341	17.54349	16.30919	12.31883
Gmeb2	11.52564	11.04444	12.321	11.35219	10.92475	9.671181	12.70459	11.22116	12.31568
Pola2	19.9278	16.72927	17.52355	12.66098	13.55008	13.84846	16.10525	11.98894	12.31314
Sgsh	13.19282	11.86623	14.54072	14.58508	14.70155	12.41373	20.27427	13.67168	12.3051
Ecsit	14.80088	13.49727	15.0636	14.82919	16.12008	13.20471	15.49011	16.25714	12.29438
Snapc1	15.1881	15.32835	13.98192	12.34064	11.41669	11.50445	11.42215	13.39686	12.28844
Ttpal	15.62159	14.41693	15.27736	12.08065	10.98445	8.663074	12.43708	12.51845	12.2872
Fam98a	17.07568	16.37925	15.37201	14.67694	11.65058	12.11102	13.33979	12.99014	12.28123
Elp1	15.82375	13.26095	15.97212	15.15233	13.08191	11.77101	14.66995	12.45018	12.27112
Smpd2	11.76588	13.73633	12.321	14.55614	17.83024	14.72097	15.16501	11.30112	12.26936
Mre11a	12.2509	12.65336	14.16107	11.92923	10.42123	9.345792	13.24429	10.40074	12.26521
Pigv	12.46518	14.89408	16.12432	14.78957	13.0908	13.55669	16.06486	16.27871	12.25804
Btf3l4	12.73887	11.49892	11.80203	10.42841	11.77494	10.68151	10.08479	10.36196	12.24063
Ktn1	12.41802	13.09719	12.90884	10.95449	9.320364	9.649324	12.57494	11.54617	12.23882
Tmcc2	15.26989	16.89928	18.8603	11.89375	14.04925	11.13466	15.76159	13.03903	12.23563
Fbxo9	12.35614	14.8595	17.23477	10.6209	12.45169	16.32367	13.45096	12.27698	12.23273
Septin1	2.977909	2.481765	3.91661	7.829001	8.314129	12.41131	7.251845	6.253111	12.2082
Mrpl43	14.62505	14.95076	11.79281	12.498	13.53699	16.94825	12.13981	14.3161	12.1966
Icmt	14.07696	15.22657	15.0973	11.08468	11.13959	11.08112	13.35455	11.51756	12.19492
Mir1956	7.676039	5.103736	5.970947	6.784926	2.436859	4.732374	5.289047	6.496304	12.19325
Phf20	13.47954	13.45472	14.22451	14.16525	10.397	11.56261	16.61493	11.73531	12.19162
Wdr7	17.03986	16.18912	14.38491	14.47723	12.12685	9.946111	16.74065	11.32088	12.19153
Smc5	12.06693	13.75548	13.19537	10.23414	9.678319	10.03398	13.44065	11.06163	12.1757
Arhgef37	11.65674	6.01801	10.95518	11.18233	8.195702	7.419028	9.337059	12.81207	12.17188
Tmco6	10.96544	9.870035	10.72233	11.96708	12.42149	13.33234	12.75394	12.59218	12.16861
Abcb7	14.54539	12.95135	15.20175	9.620275	10.80909	8.020822	13.4216	13.52786	12.16572
Higd1a	10.96796	11.73431	12.69772	8.559969	8.198344	12.23287	11.85121	11.77163	12.16401
Sphk1	16.35247	18.19565	14.01345	5.802897	10.56583	6.760534	7.257526	13.89012	12.16269
Cdk1	10.2106	13.33633	12.6723	6.984425	5.875954	10.53875	7.812765	9.393799	12.16147
Heatr3	15.59526	13.19871	12.2133	9.374833	10.23749	10.57614	14.24254	13.77445	12.15966

Bnip3l-ps	11.31297	14.92393	12.66659	16.4038	12.96356	14.3382	16.21114	14.80484	12.1569
Trp53bp2	15.21058	11.58949	13.00472	11.0255	11.15174	9.490919	14.64574	11.61939	12.15139
Snta1	12.47684	13.43702	12.58315	9.826514	12.68165	12.33811	12.32652	11.33014	12.14136
Med23	15.28683	13.34037	16.31406	13.06932	11.01913	10.87196	15.65519	11.13779	12.13772
Knstrn	20.46527	27.66453	26.34872	15.81117	12.63777	15.95621	17.91312	12.63391	12.13085
Aph1c	11.53668	11.79388	13.45211	13.69417	13.97101	12.58696	17.00706	12.69426	12.12662
Cyp51	14.22566	12.07931	12.31676	7.940491	11.26807	7.20782	8.674336	15.94508	12.12602
Nusap1	16.14127	17.84719	15.50925	11.99612	11.99237	12.40655	11.92253	8.911065	12.12199
Ccdc43	13.45701	12.65426	13.17017	11.42217	11.64911	10.40015	12.6915	11.78282	12.11573
Scd2	17.54742	16.32453	18.08835	14.98239	12.47104	11.20681	13.13451	16.21321	12.112
Morc2a	15.77853	13.5492	14.89081	16.28382	13.58839	10.53517	17.47904	13.59584	12.11196
Xrcc6	17.41219	15.24805	15.02915	13.44891	14.31194	13.41351	15.90298	12.19253	12.11181
Ear6	0.544273	0.387731	0.388811	0.309271	1.258873	3.235669	1.28579	0.074029	12.07924
Igfbp5	6.988946	9.492129	13.38283	19.54984	10.91327	10.98211	7.791119	12.8034	12.0766
Clp1	12.94009	11.50187	11.30686	10.70043	12.23751	12.47044	11.21558	12.83898	12.07341
Kansl1l	11.73196	11.03384	12.30074	12.63339	12.04179	10.45585	13.78	12.7712	12.06995
Timm29	15.26733	14.00576	15.44401	13.82536	12.675	13.11604	13.82744	13.98652	12.0676
Clcn3	14.25503	13.93473	15.37257	12.03896	11.5145	10.10255	14.97425	9.988872	12.06261
Slc25a13	16.58719	16.42049	13.71367	15.10142	10.43802	12.08965	16.33095	12.47033	12.06173
Alg1	19.34623	13.94914	14.52722	14.85416	15.94832	13.93135	15.96346	15.49051	12.06071
Prmt3	14.00612	11.29203	10.08287	9.778519	10.31055	11.0544	14.31285	11.5314	12.05931
F5	7.719413	8.465028	9.950622	13.26828	14.81742	13.33242	21.20635	11.32625	12.056
Prpf31	10.91021	11.73043	11.03567	9.896493	9.275659	12.04168	10.81786	10.82674	12.05538
Sbsn	19.91832	17.52376	17.57258	18.52646	9.737447	9.525088	16.75106	9.157614	12.05443
Mcm3ap	16.57439	14.36415	15.6662	16.61427	13.99411	12.69061	17.09284	13.08876	12.05003
Bloc1s3	11.06985	13.25436	11.9296	10.09543	12.4033	10.78449	8.657413	12.48405	12.04569
Pkn2	12.56668	12.09674	13.0237	10.90649	10.81151	9.366395	12.80366	11.28441	12.04526
Taf6	13.2878	15.10104	16.10611	13.04581	11.87567	11.68709	18.2866	13.08675	12.045
Ccnq	12.95367	15.08743	14.26881	14.05211	13.71728	15.28807	9.877352	11.85881	12.04382
Vrk1	14.69002	14.62051	14.66125	10.80427	13.45363	15.5155	15.08837	13.95732	12.04019
Kiz	12.44464	15.40524	16.48012	13.02788	13.51978	14.19757	11.6018	13.51565	12.03966
Ppp2r5e	13.05363	11.61453	14.10026	10.93744	10.72841	9.794855	13.91261	11.46712	12.03612
Actr5	13.87848	11.83715	12.1356	11.04813	12.71211	12.37851	14.73601	11.62514	12.03478
Orc2	12.95384	13.08359	11.03401	9.406248	7.09209	9.018322	10.43845	9.371937	12.03228
Setd6	12.36013	12.89938	10.79681	14.41717	12.06967	12.25016	10.83434	12.01633	12.02883
Gsdme	8.335184	8.235302	6.466362	7.074914	6.850217	7.852194	6.494668	6.650344	12.02762
Bcs1l	10.23991	14.25282	13.24216	12.04749	11.71715	12.66075	13.56854	12.96349	12.01175
Phykpl	13.91317	13.34106	13.12783	13.01721	9.469685	9.988871	15.74771	12.7359	11.9992
Pde3b	11.55586	15.5953	17.13173	9.141643	12.86723	10.44814	14.86285	12.56677	11.9964
Ap3m2	11.39696	12.69813	12.81493	13.84259	11.97078	12.13554	16.28912	11.14555	11.97958
Nucks1	15.94993	15.19059	15.79709	13.13723	12.27028	11.91443	14.08705	12.95089	11.96711
Fstl1	14.08609	13.03746	12.79725	19.64367	12.88877	16.98969	10.3038	11.27606	11.96125
H4c4	2.20649	3.851078	5.654788	7.679456	9.193787	3.443332	6.271423	3.413792	11.9607
Tsfm	12.1212	11.43294	12.31056	9.950985	10.51498	8.64594	9.857199	10.10918	11.95558
Ddx55	10.23348	12.21534	13.24329	14.52974	10.30903	11.50039	15.93462	13.4002	11.95497
Mettl7a1	16.92878	18.32249	17.40186	16.59974	18.36595	15.00177	23.94771	14.43599	11.95365
Cul2	14.74549	14.77728	14.35537	11.49302	11.78767	10.19419	15.5347	13.90351	11.94909
Gskip	12.71077	15.07742	14.12472	12.60001	13.09072	12.96861	11.0402	13.48443	11.94387
Adck1	14.92795	11.94658	11.64876	14.72462	12.0961	12.7751	15.58431	12.32164	11.93288
Pus7	15.55505	14.94155	12.24976	13.6225	10.09054	8.612172	14.15885	12.46476	11.93103
Rbm33	14.57576	14.42056	15.93716	14.86546	12.69256	10.90531	21.35483	12.24424	11.92887
D10Wsu102e	15.57764	13.34598	15.18443	11.28001	12.05235	10.47555	12.4426	12.69547	11.92568
Sac3d1	11.98334	13.96346	12.3479	11.1539	11.37437	13.58175	13.88663	10.60259	11.9211
Haus7	12.04701	12.83043	11.66679	9.215024	13.08284	13.61081	16.33866	9.082529	11.89751

Pctp	15.29739	13.78278	14.99958	14.67552	13.9224	13.92493	16.53303	12.87238	11.89603
Lrch3	14.44505	13.26506	13.69419	14.30091	10.38611	11.3884	15.72929	10.5242	11.88867
Rnd3	13.88403	16.4874	13.96409	10.43269	11.72887	9.448287	11.81632	12.24807	11.88701
Spc25	20.81827	22.09305	21.69063	16.26147	11.92939	14.15752	13.66521	9.606851	11.88289
Zscan26	14.24557	14.04114	14.90115	16.64201	14.985	11.9774	16.37702	13.02012	11.88013
Cox10	16.99325	14.37037	13.80728	11.11546	10.1946	10.49647	14.66954	13.57498	11.86481
Lrp6	15.87645	15.91609	18.75951	15.54251	15.29505	11.28175	22.78861	12.1144	11.84401
Myo9a	16.53907	14.2436	15.79464	12.06813	12.5711	9.049215	17.96167	11.21727	11.84241
Slc9a3r2	16.38838	15.32935	11.8575	23.97133	17.79763	20.8046	10.45485	11.69469	11.83207
Rapgef2	13.51097	10.29797	11.24777	11.35992	10.10746	8.126517	15.89428	11.28697	11.83023
Narf	10.6428	11.40397	10.97077	10.42075	11.53623	11.28301	12.67517	11.76001	11.82015
Rpl10-ps1	12.47541	10.01266	10.26203	8.661848	7.100744	8.396656	10.91027	11.45612	11.81982
Rnf169	11.29366	12.83956	12.85209	12.87875	10.88874	9.097328	17.70898	10.66417	11.81957
Sec23a	12.57623	12.46191	12.87491	10.867	10.49797	8.781913	14.81315	9.440139	11.81889
Chaserr	6.650843	8.632753	8.613681	10.34378	10.32771	10.37708	10.19399	12.10004	11.8147
Gm5086	8.078484	10.99841	10.98631	11.34853	17.87092	11.62113	15.94802	15.38302	11.81407
Rffl	11.95425	12.4269	11.92192	9.422649	10.59826	8.606933	15.45722	12.89061	11.81128
Cop1	9.9261	10.29639	11.43899	9.213277	8.596748	8.754901	9.367297	9.613762	11.80515
Tsc22d2	11.99922	11.50173	9.865497	10.83984	10.71532	8.969888	14.72891	11.38522	11.79617
Samd8	13.53142	14.24333	13.5493	11.26706	9.921573	8.714174	16.73193	10.65583	11.79344
Phldb1	16.83539	20.24555	17.78714	11.68656	11.1723	9.251482	12.64862	11.14833	11.78736
Gm9531	18.6416	12.32992	13.53255	10.88999	9.317401	12.87311	13.38127	12.14136	11.78288
Zfp62	12.63234	12.23914	14.81109	13.7612	11.64792	10.22014	15.93681	12.74014	11.77542
Qpctl	16.12984	11.84796	15.76208	9.109167	11.41456	10.42437	13.91523	12.1651	11.77519
Med13	13.93315	13.72819	12.57233	11.69499	10.41445	8.653195	16.38484	11.29264	11.77061
Tbc1d22b	14.06993	14.33339	15.04037	12.25407	10.45083	11.33727	16.19969	11.70256	11.76014
Rtl8a	8.428671	11.78606	10.68914	11.96734	9.392855	12.49542	11.85476	7.487168	11.76005
Serpine1	18.47935	15.63272	11.38593	10.66455	10.70289	13.54585	22.43414	16.40725	11.75912
Neil1	8.934233	9.889618	10.86634	12.65915	11.78151	10.92534	13.15757	10.06419	11.75897
Tipin	13.58353	13.06866	13.4891	14.08019	10.01116	14.11518	10.61625	12.65865	11.75689
Ndufaf5	13.16515	9.418217	11.61112	10.21903	10.63379	12.27746	11.48257	12.42874	11.75341
Slc39a3	11.9464	12.66404	13.48153	11.92523	14.0066	12.19639	13.78956	12.80504	11.75139
Lysmd4	12.05175	15.31395	14.64235	17.75444	15.64403	12.79621	13.74783	12.23941	11.74164
Rps13-ps1	16.03668	13.4519	14.65774	12.03934	13.45252	14.83022	11.30593	17.39192	11.74164
Smyd3	10.64096	10.74365	13.26989	11.66997	14.09631	12.18916	16.47431	16.34339	11.72773
Med19	15.29326	13.31912	16.29137	12.85149	13.30863	11.32912	14.33146	13.07385	11.72739
Tmem209	18.27226	13.7114	19.24625	13.59179	14.29204	13.28827	15.87221	14.33317	11.7214
Zfp608	15.07267	13.27352	14.47373	9.783595	9.960459	6.69165	9.853483	10.0028	11.71237
Bcr	13.72081	11.79989	11.90775	9.215258	10.96817	10.19246	11.96485	11.22181	11.70512
Dnajc12	8.565951	9.023098	8.951978	7.991577	8.753935	8.500061	8.455435	6.047991	11.69578
Arl6ip6	10.0757	10.93681	13.81226	10.34316	10.11301	10.42216	10.07709	11.30905	11.69335
Rnft1	13.60321	14.61444	13.94241	11.55017	11.43136	11.34265	13.7753	12.54685	11.69001
Traf2	11.20053	9.33617	13.98863	10.79369	11.36107	10.56	11.16219	10.54262	11.68186
Taf1b	13.63902	12.3147	12.53802	9.929219	15.1196	10.66129	14.71517	13.76543	11.67827
Cdk6	16.23457	13.81182	13.72522	12.77141	8.855506	8.340681	15.21815	12.41132	11.67695
Yju2	14.82008	9.643197	10.38793	12.80571	12.58642	11.6357	12.65531	12.78357	11.6701
H4c9	5.22741	6.85986	7.01655	7.797193	6.288667	6.233503	5.687147	8.251354	11.66881
Alg13	8.95798	11.76608	10.58179	10.89105	10.75289	8.409101	13.97512	11.03926	11.66289
Ccdc34	11.01004	16.2479	11.8767	12.13518	13.41278	13.52046	12.36625	9.601978	11.65949
Bloc1s6	12.61398	12.54875	13.36127	12.7011	12.45165	13.19101	13.90633	13.09742	11.65931
Taco1	15.74237	13.94969	15.46103	12.21928	11.54127	12.7252	12.29069	12.88561	11.65769
Tjp2	17.8874	17.46758	18.17262	12.9885	8.615928	8.746873	12.31614	11.61508	11.63944
Nat10	14.22772	11.06665	12.31306	11.94406	9.600984	10.44654	12.60645	11.51766	11.63867
Ibtk	13.36309	12.31708	12.68577	10.4976	10.99971	8.909957	15.31926	12.24067	11.63543

Acad9	12.82505	11.7421	12.96373	13.54887	10.82099	11.18369	12.90091	10.60003	11.63512
Rrp15	13.9395	11.16749	10.83293	10.68011	10.5336	10.14359	11.1006	15.14287	11.63033
Casz1	10.52396	9.072761	10.63222	10.00384	10.36266	12.04157	11.6882	9.034837	11.62831
Calcrl	13.94646	12.97646	13.40838	9.301205	8.120227	7.61858	6.085512	10.89216	11.62354
Zw10	14.0318	15.75091	12.63583	12.25902	10.46492	10.90494	15.41269	11.48237	11.62204
Ric8b	11.5819	13.19432	9.232673	10.60145	11.93045	10.35207	13.54817	10.0582	11.61591
Mvk	11.98926	9.546322	11.89263	11.53228	11.94736	13.56456	11.8175	12.41026	11.61364
Lsm5	10.06959	8.608097	10.40276	12.54399	8.957847	9.5167	8.691913	13.90676	11.60103
Gm32287	8.440544	8.177557	8.313838	5.007746	7.471246	6.690472	6.744392	9.170751	11.60043
P2ry13	10.19727	8.726621	13.35669	10.72893	14.72429	11.82313	14.8458	12.84067	11.59998
Rflnb	4.072614	5.179546	5.22555	12.1651	12.92904	15.15249	9.625804	7.529618	11.59955
Met	14.37513	11.37531	12.45078	8.030679	7.194431	4.998053	5.894169	9.87749	11.59297
Polm	13.19563	15.04447	16.428	16.55187	12.41093	11.19021	14.31743	13.1109	11.58509
Rhou	7.392839	4.97565	7.937111	9.152446	8.013094	8.633385	9.00227	10.0329	11.58123
Armc8	14.22233	12.05146	13.04756	11.57541	12.23577	10.79313	12.66272	11.73752	11.57502
Poglut1	16.97363	14.13954	15.07856	12.3089	11.65853	11.62784	14.63272	13.46093	11.57391
Slx4	17.47949	14.21309	15.97554	15.21338	12.96446	10.61665	19.23629	13.2587	11.57325
Gpsm2	11.98334	10.98566	11.08544	9.491701	7.328565	6.140573	8.971802	8.281217	11.56951
Zfp429	8.262535	8.675681	11.42708	9.680534	10.25846	9.078337	10.00531	10.9044	11.56549
Med29	12.3851	11.3036	8.770593	9.179421	11.13606	11.33479	11.68752	11.45004	11.55971
Mir6935	21.65549	25.91741	8.663204	22.39556	21.44943	13.6179	28.649	28.86541	11.55818
Vcpip1	10.36087	10.73596	12.44928	11.06056	11.01573	9.854865	11.98974	10.99612	11.54954
Endov	11.78834	14.4869	13.46874	14.9523	11.50516	12.96029	14.40937	11.74488	11.5484
Lenep	9.377996	11.0912	9.711089	11.80125	12.88349	8.442234	11.49391	13.82774	11.54829
Sirt1	11.48809	10.74323	10.26155	8.851216	8.700007	8.98835	12.23606	10.18633	11.54817
Gm13012	12.84988	13.30389	18.11432	15.45518	11.77187	14.37298	11.76317	13.98211	11.54725
Armc1	13.85575	13.9539	13.01479	10.26255	9.599746	11.57516	12.78126	11.25835	11.5425
Gfpt1	13.88615	12.25529	14.32287	11.20385	10.27391	9.204154	13.3885	9.898826	11.54231
Slc35a5	11.72676	12.53287	13.05533	10.16641	11.45216	8.953635	12.37211	11.25527	11.53802
Prdm1	10.70282	10.00083	12.22047	14.07646	11.83145	9.379186	18.6472	11.45177	11.53655
Fam160b1	14.21893	14.77031	15.619	12.56281	12.97927	10.32633	15.78494	11.54089	11.53413
Gm36587	6.287488	8.293571	7.775754	13.98357	10.30217	11.56642	16.56052	12.48756	11.53402
Arsk	12.3348	14.11308	15.90003	11.0732	10.73593	10.42458	11.73936	12.21696	11.52757
2410002F23Rik	14.88642	12.81298	14.42198	15.35344	12.56845	13.52403	13.45886	11.81573	11.52261
Cir1	11.17312	11.76877	11.11741	11.13888	15.25595	13.25835	14.75902	15.65835	11.51828
Ube2d-ps	12.23253	12.32016	11.05877	10.42629	10.78928	12.00883	14.04424	11.38839	11.51511
Pced1a	9.98884	11.93477	13.4865	12.11812	11.37786	11.87925	14.01994	13.06723	11.50046
Gm39714	0.287244	0.286479	0.574554	0.57127	3.009247	12.21917	0.296881	1.093937	11.49829
Serf1	8.376889	9.387177	11.57839	6.738849	10.0398	14.53591	8.949773	10.75364	11.48249
Eef2k	12.27557	12.55468	15.11175	16.95275	15.34335	16.44026	21.79909	12.52095	11.48121
Dcun1d1	12.67066	13.44819	12.14022	10.39085	10.10086	9.50414	12.28738	12.91264	11.47991
Natd1	13.14305	14.88948	15.63751	13.95751	15.47523	12.00636	17.55173	14.02371	11.47203
Inip	14.36025	14.76791	15.61389	12.46593	12.80792	14.05719	13.28944	11.79944	11.47083
Klhl36	8.489115	9.318329	9.810209	9.960015	9.761086	12.27832	11.5024	9.782745	11.4703
Atp8a1	12.96194	11.75511	13.05941	14.53528	13.57091	11.56529	15.19021	12.14309	11.46982
Lcor	13.71982	13.48172	13.29336	13.37106	10.39441	8.773616	15.22458	10.74213	11.46718
Bhlhe41	11.87364	13.34534	14.05766	13.39797	9.863959	11.8954	13.99237	13.34749	11.46646
Gba2	11.21307	11.26143	12.2966	11.49335	11.14222	11.60224	12.10795	10.36239	11.4643
Ptrh1	10.86902	13.14133	11.05246	10.14395	10.2937	11.79206	16.69361	11.85149	11.45871
Eno2	10.37437	12.61011	11.12382	10.51465	7.267824	9.686146	9.407905	9.616239	11.45642
Dock7	17.47317	15.62893	14.19752	14.8196	13.26536	9.530884	19.11122	12.21026	11.45029
Pla2g4a	14.30158	11.35654	13.21495	9.410131	11.28887	9.793454	9.700293	12.45096	11.44533
A930005H10Rik	5.91457	5.370571	7.681006	7.988224	10.08891	7.67378	8.302736	7.312221	11.44249
Tsen54	12.41066	13.42084	11.988	10.17652	11.52361	10.77112	11.80438	10.71261	11.4243

Clec2i	11.93595	11.73201	13.93512	14.27234	14.28005	14.29922	14.5539	14.88616	11.42303
Batf2	8.147957	7.130399	11.10488	11.99461	12.28671	10.30519	10.60921	11.90267	11.42019
Gm5561	10.32751	8.981612	9.750301	11.41986	10.22924	12.83595	10.84478	9.596813	11.41792
Stoml1	10.19038	9.89658	9.03276	16.51462	15.19445	13.70977	14.43197	13.60577	11.41129
Xpo5	15.28607	15.52992	14.82691	11.87228	11.1408	10.12764	15.39062	12.03591	11.41045
Gopc	12.08712	10.11018	12.86344	9.823563	10.31867	8.813261	11.37151	9.909776	11.40933
Btg3	9.785865	10.48612	9.240751	6.291236	11.05389	7.660644	10.53756	10.61716	11.40906
Hirip3	14.85698	14.71271	12.54852	10.67574	10.09973	10.60788	11.82857	9.696779	11.40807
Zfp653	13.1065	10.87948	12.64667	13.0061	9.898123	14.2033	12.95726	10.30847	11.40543
Zfp664	15.40248	16.05295	16.15205	12.17997	11.36762	12.04369	15.68038	11.84305	11.40189
Tmcc3	10.49051	11.72548	12.22147	14.3219	11.69707	10.76758	14.51337	11.30143	11.4015
Gpr180	10.78777	10.17397	11.40833	10.40325	10.70871	11.06122	11.35181	8.967745	11.39996
Pofut1	13.71368	11.86275	12.05401	10.78561	10.83845	10.43269	13.40734	9.809048	11.39298
Ptar1	14.26241	16.04602	14.31779	12.90048	8.850929	6.855522	15.57373	13.06775	11.39178
Wdr24	10.80497	11.73964	11.91547	14.17768	11.6024	11.7953	12.79452	11.01977	11.38928
Zfp628	15.69032	12.0127	15.30667	13.22294	12.46549	12.50496	15.03824	12.85923	11.3879
Pknox1	12.23769	13.32063	13.46303	12.91499	11.50466	10.13666	12.97467	10.63668	11.38769
Golga3	13.59539	11.57662	13.65954	12.59307	11.45205	9.19162	13.76416	11.0912	11.3876
Klhl2	13.70542	13.54237	11.78513	8.833384	9.496152	9.941648	10.69888	11.13299	11.38648
Apoo	11.26183	10.98363	12.88105	8.290784	8.177562	9.263803	8.617208	9.359854	11.38584
Foxred2	15.05069	14.3203	14.69827	14.5342	12.01919	9.928962	15.43918	11.04999	11.38367
Gys1	12.33463	11.47566	12.01975	11.14232	10.3417	10.40389	12.5461	9.162727	11.38279
Rprd1b	14.58557	16.28462	17.54021	12.54649	12.64646	10.81748	13.1572	13.81008	11.37942
Chn2	15.04779	17.38886	18.22593	9.561827	11.76547	14.40186	13.03977	12.3458	11.37747
Emc9	9.198244	9.834927	10.19383	14.3381	12.94316	13.64065	13.61791	13.64928	11.37307
Pcp4l1	11.79159	10.07515	8.97673	18.62085	9.621079	16.08006	14.22446	12.03105	11.37262
Rbks	10.11686	12.07292	10.64441	9.129769	8.855462	10.05879	10.27496	14.19782	11.37007
Pecam1	12.80747	10.9851	9.25905	24.65274	19.44661	20.38851	9.039091	14.00565	11.36963
Zfp868	8.707696	9.885266	9.408765	9.744765	8.986429	8.80349	8.044871	9.010341	11.36805
Zfp260	10.6951	12.66947	12.75041	11.42187	11.15668	11.78981	11.25833	11.39951	11.36787
Arhgap6	13.82536	15.88681	11.87316	13.28603	8.756397	8.060274	14.83987	11.02995	11.36694
LOC115486178	4.172757	1.783564	5.365597	2.963845	3.974088	1.653787	12.32215	3.405321	11.36288
Cdc25b	15.83683	18.18669	19.97687	12.48926	12.18832	12.42743	16.55738	11.99285	11.35969
Sema6b	15.1505	13.37864	15.59748	11.52425	14.49983	12.88546	14.7846	12.68701	11.35665
Cgas	10.6132	10.39569	10.47888	8.909363	9.242983	9.338436	10.05867	10.20805	11.35552
Gm16477	12.36249	10.98103	7.985039	9.604098	9.321008	10.83701	10.11533	12.32204	11.35298
Rpsa-ps7	8.932891	8.693131	11.53289	8.721347	9.332566	10.56388	9.344499	9.432805	11.35178
Ccser2	14.53085	14.59228	14.16859	13.53856	9.010898	9.79395	13.87706	11.7082	11.34964
Virma	13.87344	14.21755	15.06912	11.36585	11.12068	9.694063	16.0747	10.60196	11.34885
B4gat1	13.92081	15.83997	17.51883	13.84283	15.41148	12.57122	14.95098	11.38649	11.34641
Thap12	13.47614	15.17051	14.26778	11.30584	11.3888	10.74337	13.72014	12.44717	11.33812
Atxn1l	13.49552	11.8527	13.97015	11.4062	11.09327	7.969307	12.31361	9.098485	11.33387
Strn	14.13057	12.77685	13.4759	11.24255	9.830106	8.311491	13.35135	11.2397	11.33298
Uri1	14.1356	14.85059	17.00887	10.85346	12.814	11.65942	15.54199	13.14305	11.33232
1600020E01Rik	7.702372	6.259299	9.795513	7.564669	6.792273	6.947038	6.879692	11.86021	11.32877
Ctnnbip1	12.22103	11.54132	13.26082	14.66913	10.38256	11.56167	10.66373	12.00631	11.31788
Fzd7	12.65772	12.45157	11.80602	15.02853	11.41829	12.11057	18.95636	15.59657	11.31681
Wdr37	12.56907	11.44492	12.17679	11.0632	9.943135	10.59868	12.80992	10.89801	11.31237
Mto1	12.80422	12.91047	13.43896	11.91631	11.63624	10.84336	13.98517	10.56096	11.30953
Smurf1	15.23188	14.34087	14.73261	12.27412	10.80133	10.71479	14.67428	10.54432	11.30706
Gm14292	9.189733	10.06187	10.5561	10.26398	8.593647	8.098091	11.32195	10.14438	11.29737
Zfyve21	10.28047	12.66808	12.91581	17.61546	12.01623	15.98914	15.71853	10.92051	11.29627
Zfp326	12.42294	13.20858	13.57378	14.47574	12.1442	14.01882	15.18708	13.49522	11.29249
Primpol	9.98687	12.03231	10.82644	10.07591	10.24036	9.606315	15.40755	10.23723	11.29004

Dipk1a	10.96957	10.45323	12.13103	10.48313	10.40854	11.85699	10.60139	13.37001	11.28905
Helb	14.29989	13.20042	13.12709	12.68712	11.69678	10.36208	15.15894	11.39039	11.28566
Zfp688	10.25822	10.01323	12.0057	11.61151	13.66736	10.69753	10.54599	12.83196	11.28515
Slc33a1	10.85947	11.60417	11.72777	11.63803	10.86391	9.599449	13.34596	11.03436	11.28502
Arhgap12	13.69846	13.97718	14.53884	15.12049	11.12463	10.6896	17.06001	12.79926	11.28341
Ralgapa2	14.38613	12.44991	12.78384	11.18736	9.335504	8.201453	11.66494	9.389627	11.28156
Gm12854	10.63019	10.15211	13.65977	9.48155	9.725251	11.70719	12.01893	12.69724	11.26748
Man1a2	13.26633	14.25414	14.09843	11.42938	10.66739	9.467429	13.87731	11.29534	11.26054
Commd2	9.829444	11.05436	9.624612	10.03503	10.05688	10.45778	8.301535	12.01465	11.25984
Rubcnl	9.89189	8.058677	12.17605	9.548231	11.31892	9.951904	14.56792	10.97047	11.25816
Spidr	13.20041	10.84628	11.5723	9.376049	9.938971	8.58407	10.27504	8.506554	11.25499
Ppip5k2	12.85581	11.58714	13.19018	11.05653	10.50029	8.541358	12.35186	11.08135	11.25149
Kcna3	21.88789	15.84544	17.52362	10.92466	10.16802	7.450443	16.12962	10.03081	11.24943
Pank3	14.76843	14.25616	14.86801	11.14527	10.6099	8.137349	13.48234	11.28749	11.24185
Dhx29	14.38205	13.92188	13.4754	11.23335	8.957752	9.848492	11.9328	11.07532	11.24099
2610524H06Rik	10.55963	13.80799	10.44352	10.90883	11.95651	11.71826	11.6415	10.44478	11.23843
Mtmr12	11.81488	10.91812	11.90558	9.020868	10.59618	8.283016	12.2772	11.3704	11.23631
Kif16b	12.98174	12.82248	11.96209	11.44821	9.109024	8.247488	12.99731	10.247	11.2308
Tmem42	7.851743	9.729225	9.042452	14.19593	10.08385	10.12146	10.57431	8.381765	11.22511
Dennd2a	10.5533	10.32637	12.24272	11.22111	6.911541	7.682118	10.96231	9.693448	11.22367
Golgb1	15.24394	14.92416	14.71122	14.72834	11.82782	9.66794	20.674	10.38495	11.22043
Hdac8	8.50434	9.11942	7.73793	8.011613	9.195605	8.810658	8.723572	7.001135	11.21347
Fastkd3	10.0755	11.09447	13.63315	8.160324	12.02731	10.37153	13.6177	10.15715	11.21057
Mtg1	13.00647	9.088296	10.55895	9.221186	9.099253	8.872487	10.74597	10.93105	11.21025
Pcmtd2	10.92291	13.71414	13.11524	11.32525	13.24404	10.35981	13.68215	11.21332	11.21018
Sertad2	15.69141	13.9518	12.95432	10.16752	11.71176	9.220893	13.39831	12.36255	11.20641
Ccr7	13.62192	7.697582	7.949446	3.923362	9.435179	9.747461	12.1442	5.785507	11.19878
Btbd19	10.68073	8.665556	9.516278	5.584401	8.799767	7.309914	9.155415	8.353189	11.18957
Fem1a	13.89205	12.41927	12.64096	11.87312	10.73634	11.83904	13.03832	11.48421	11.17058
Gm6467	9.877776	9.393271	8.730212	5.786867	10.20967	9.984394	8.388934	10.86269	11.16591
Ptdss2	14.73598	14.98102	15.82093	11.98917	13.22001	10.78065	16.82116	12.80891	11.16513
Degs2	9.803601	10.38319	10.7158	17.55626	13.92264	12.0346	12.91223	11.56423	11.16283
Trmt6	15.17648	12.94698	14.13291	11.329	11.44766	11.54096	14.02202	12.3597	11.15722
Otud4	14.42573	13.45074	11.95707	11.44926	9.845718	8.830396	15.9211	12.0592	11.15414
Zfp777	11.29601	9.777982	12.48153	13.54051	11.3897	10.66519	14.93028	9.537371	11.1498
Gm10269	11.55995	8.429937	11.43698	8.899511	9.115454	14.13876	11.17697	10.53282	11.13613
Fam120b	11.65438	11.88029	13.59427	11.94287	10.46859	9.983469	13.33868	11.11948	11.13105
Pnpla6	13.49334	16.50828	15.28386	14.26845	13.87553	13.06557	19.15739	11.95843	11.13082
St7	12.04025	11.51335	10.48682	9.374311	10.01787	8.534368	9.982707	12.59725	11.12864
Fcrl1	5.855862	2.920136	7.012439	12.41231	12.21816	9.334298	9.596122	8.876551	11.1256
Ttc19	10.46135	10.7529	11.60135	9.305866	9.759947	10.79313	13.09248	11.5864	11.12366
Gm36897	12.93555	13.53465	17.44192	12.80566	9.404751	10.09327	12.71299	12.97569	11.11787
Tmx4	10.995	12.39417	11.10398	9.33784	7.979276	8.106381	13.20036	10.15047	11.11556
Gzf1	13.11354	14.4883	13.64516	13.11816	11.19914	11.54605	15.42014	11.73774	11.11382
Pdhx	12.15863	12.67347	10.07483	8.314959	9.343221	9.681133	10.57042	8.253791	11.10715
Arhgap23	14.82838	13.82579	15.10968	15.02329	12.07724	11.19822	14.19646	10.69441	11.10518
Rps2-ps6	10.09594	9.771161	9.917875	11.40569	8.420467	9.723125	10.92861	10.35173	11.10269
Fancg	13.53662	15.0612	15.37641	13.01622	11.56744	12.88575	16.53222	10.05043	11.10248
Chd2	12.24148	11.11173	11.20992	11.3707	9.970677	8.048149	14.81246	10.45641	11.1022
Szt2	12.70564	12.34424	14.68666	13.11578	12.20772	9.711549	18.21494	12.56608	11.09635
Msantd4	12.14544	12.48431	16.02601	11.72677	11.43659	10.54333	13.90944	12.4962	11.09025
B4galt4	9.302863	9.743455	11.2289	12.06368	12.13729	11.62348	9.162858	10.13446	11.09002
Bmpr2	14.80582	14.55196	15.32763	12.63453	11.30072	8.989248	14.12424	11.41629	11.08027
Snora61	8.941623	4.458909	13.41399	17.78307	12.77386	5.788253	17.55907	11.06729	11.07881

Rhbdd3	9.973349	11.90186	11.07514	10.22527	11.39821	11.98995	11.65865	10.5434	11.07881
Erich1	10.81376	10.60989	13.06229	11.3467	10.2989	9.90282	13.20866	9.326351	11.07611
Haus4	12.12035	12.73183	14.09424	13.47879	12.05557	11.37433	12.4158	10.75451	11.0733
Arhgap11a	13.76835	14.50314	15.09611	12.07597	11.43952	9.145766	14.94979	9.963125	11.06879
Cox18	10.97907	8.410147	10.98035	7.804198	9.740706	8.415881	6.860228	10.25443	11.06084
Orc6	11.78604	15.52777	10.18665	12.44348	7.517911	11.16849	9.324085	11.29074	11.0598
Hspb1	13.38372	12.18472	13.63088	21.00096	13.79897	17.99752	12.9444	16.95118	11.05899
Trnt1	9.982323	11.19661	11.2278	8.545342	10.91245	8.722947	9.898559	9.66944	11.05835
Col15a1	10.84122	10.09973	10.15251	20.38493	15.76918	17.22584	10.58103	11.64733	11.04889
Ufl1	10.64753	10.65038	11.44143	10.97158	11.00468	9.566826	12.44906	11.02117	11.04258
Fam174c	14.64723	15.71235	15.07477	14.22646	14.92311	15.6716	16.89895	17.0266	11.03823
Hsd11b1	2.790646	3.406325	3.290846	3.272032	3.094133	6.355461	1.808051	2.22075	11.03591
Ints5	15.51727	14.55277	15.06461	12.93673	14.60216	11.85967	13.23734	12.9793	11.03586
Dynlt1b	8.125164	10.61348	10.35539	13.44225	11.23083	10.30667	11.51905	11.02202	11.03349
Tdrd3	11.92371	11.31688	11.98689	12.67605	11.80712	9.06511	12.64302	10.1369	11.03069
Rfxap	11.00043	11.20131	10.41187	8.541962	10.64764	11.76162	11.28999	12.84162	11.02205
Nom1	11.09099	12.17436	12.17446	12.89503	9.773941	9.881362	11.21846	9.416817	11.02189
Lyrm4	9.550716	8.904868	10.06418	8.29642	9.061147	10.25348	10.02242	8.605459	11.02087
Mbtd1	12.43302	13.35837	13.27545	12.81133	11.11173	10.44233	14.74353	11.88034	11.01972
Edem3	12.97505	12.73791	13.44212	10.86559	9.947096	7.827082	14.0402	10.42756	11.01922
Sugp2	12.1882	11.4133	12.91953	13.80365	13.38732	11.74371	16.53465	11.97959	11.01823
Gpr157	10.56569	9.877379	10.52873	16.81041	12.51163	11.93688	20.89305	11.38065	11.00425
Sass6	9.865503	9.722935	12.26917	12.12945	11.03951	9.338989	11.83564	9.681597	11.00316
Ncoa4-ps	10.547	9.890428	10.86334	13.29129	11.05565	11.44046	14.84298	10.45229	11.00055
C1qtnf12	13.38739	14.98409	13.0112	11.76833	10.43182	15.36857	14.7474	11.14785	10.99946
Traf3	15.42949	14.52425	14.09566	9.952044	9.940431	8.46125	11.42838	11.89006	10.99327
Gstz1	13.13871	15.76088	13.39574	12.91995	13.45165	15.42409	14.67342	11.74488	10.99185
Zbtb20	17.52472	20.92072	17.2612	21.80206	13.69187	14.71548	24.6207	14.35582	10.99047
Tapt1	13.03398	12.14427	11.46361	10.48244	9.994238	8.162848	12.45395	10.35395	10.98509
Hnrnpr	11.63838	10.81663	11.49813	10.56652	8.778417	8.412539	11.02645	10.68995	10.98446
Snx11	9.258003	9.715858	10.4687	10.36511	11.41422	8.276805	10.54592	8.688903	10.98247
Slc43a3	16.19643	15.23026	15.08757	15.73757	10.68753	10.86971	9.398212	8.745676	10.98206
Coprs	9.822076	10.73784	11.24003	15.02624	11.24331	10.65523	10.9325	7.912874	10.98154
Exosc2	14.24519	13.61802	12.57267	10.15078	10.90984	12.26289	12.48411	11.06276	10.98042
Arl15	10.95766	10.25098	12.19956	9.163446	9.592075	9.313894	14.95792	10.06719	10.97847
Cyld	11.77093	11.8045	12.78112	9.698885	11.64436	10.4265	13.85578	11.59433	10.97839
Zfp282	13.84188	13.62917	13.13809	12.41421	11.99052	10.12629	14.87124	10.7781	10.97418
Naa16	10.7032	10.01693	9.705615	8.619531	7.788781	10.42077	10.32221	11.69754	10.97337
Zfp281	9.350112	9.555989	9.950769	9.998466	8.003431	7.01267	11.13131	10.18398	10.96644
Zc3h12d	12.38701	10.18038	12.80231	14.64946	14.58235	14.21047	14.89834	12.69984	10.96452
Faap24	12.74331	11.87446	13.67507	8.509618	9.921885	11.2685	12.27357	11.27679	10.96346
Nr1d2	6.813464	7.805071	8.127847	12.19009	10.45027	13.41151	16.54459	10.62944	10.95346
Pnpla8	11.78971	13.41478	14.1191	12.19912	11.12054	10.22788	13.58974	11.15308	10.94883
Rhbdd1	14.7719	12.69995	13.82558	10.76157	9.870585	9.788871	11.52818	10.13833	10.94693
Sdad1	12.55639	9.967704	10.73547	10.65213	9.371226	8.752221	10.478	10.38826	10.93587
Mtor	17.36521	15.22802	16.04385	13.91005	12.80685	11.77592	18.35632	12.65569	10.93272
Btf3-ps1	10.68133	11.43715	12.12439	9.578896	11.67061	10.78675	10.97196	13.60116	10.92972
Plvap	20.67928	15.16648	9.708289	31.33737	25.53504	24.54019	7.787526	21.65233	10.92916
Hira	12.63472	10.90682	12.49966	10.45236	9.375183	11.86654	13.52891	12.30388	10.92888
Rbm3-ps	10.83606	7.912423	7.789324	8.322043	9.905497	12.34712	10.54963	14.32404	10.92711
Depdc5	14.49249	14.32998	15.75252	14.51944	11.64262	10.31381	14.24022	11.43201	10.92575
Fbxo31	12.85948	15.18145	12.53878	11.18568	14.11372	11.79274	15.17773	11.68116	10.9257
Rps15a-ps7	10.46471	11.05809	7.600258	10.77774	13.76323	10.13827	6.953016	11.50539	10.92369
Acad8	13.6685	14.39415	14.15123	12.49445	12.10136	14.34126	14.65355	13.06764	10.92351

Smad2	13.11509	12.48622	12.16364	11.97566	9.793391	10.0907	13.5551	12.05844	10.92318
Sec24a	13.73112	13.57326	14.51667	14.19178	10.52883	9.536083	18.22864	12.05684	10.92299
Rbm15	10.73159	11.44699	8.087777	10.8232	10.54357	8.356427	15.19186	10.66948	10.92296
Fbl-ps2	13.07594	12.40617	9.550953	10.12944	11.61383	11.14115	12.95785	10.58447	10.92222
Gm52147	11.44364	10.14504	8.901641	9.609385	13.80514	12.22892	14.98159	12.59031	10.9068
Fam136a	11.11668	12.53637	12.7893	8.995251	8.961231	9.709223	8.52336	12.20984	10.9068
Rcl1	11.75027	9.743114	11.54667	8.627475	9.759446	8.939415	11.01477	11.80532	10.90606
Psmd9	12.68317	11.64684	13.56166	10.01739	13.87186	11.84767	10.19073	10.16144	10.90587
Gm10136	10.19311	10.16598	9.466135	10.34288	11.29276	12.11949	6.020053	15.3495	10.90453
Dock4	17.32215	16.11297	14.74034	10.79358	9.901874	8.005344	12.71126	10.61628	10.88382
Desi2	14.01081	13.42244	13.3151	10.62258	10.29911	9.635446	12.06059	12.92632	10.88345
Vars2	14.11245	11.48759	13.2579	13.74964	11.66165	11.03556	12.62862	12.02857	10.87902
Chaf1b	14.8156	14.3557	15.05826	13.7744	11.29965	11.77952	16.0285	10.49342	10.87744
Gm5526	6.004122	6.886358	5.504418	6.667047	7.91026	7.310692	5.998708	7.336192	10.87268
Decr1	10.36647	10.98383	12.56272	10.69813	9.014396	10.07588	11.72695	7.630976	10.87005
Zfp120	10.50463	11.54627	11.90847	8.572658	10.69853	9.968658	10.70074	11.70326	10.86364
Fbln2	14.76484	12.49394	13.14709	19.60057	15.28159	16.97568	8.444077	13.66514	10.84528
Xpo4	16.19185	13.26305	13.94924	10.99231	8.796823	9.244938	12.61361	12.07869	10.83617
AU022793	7.805252	8.79083	7.539028	12.24728	9.920937	12.59729	11.71726	10.42652	10.83336
Bloc1s5	11.23275	11.58047	12.4332	11.64047	10.96841	11.05882	11.18567	10.84489	10.8261
Gm4735	10.92912	9.38522	11.16155	8.044219	9.654063	8.427515	9.862492	9.148123	10.82553
Zdhhc1	16.30903	16.108	14.88846	13.67188	15.32183	14.84825	17.0522	13.0602	10.8145
Usp30	12.71698	14.07033	15.48074	11.69731	13.22804	12.23802	16.9635	13.9429	10.81368
Cul5	11.82679	11.18569	12.6385	10.77107	8.718539	8.952074	11.32737	9.785058	10.81134
Cdc42ep2	13.05081	9.901111	13.1267	6.100632	9.561115	8.561756	8.070142	12.07164	10.80846
Cinp	13.88001	12.1263	12.10543	11.95471	11.91807	11.09235	14.88223	12.51275	10.80706
Ap3s2	10.71701	9.993057	10.99528	8.244433	9.560877	9.672258	10.77052	9.148719	10.80599
Kin	13.99017	12.42933	10.97632	9.834207	12.86463	14.31323	13.29613	12.59277	10.80504
Rpl10-ps5	7.386866	7.65897	8.558057	7.490943	8.079997	8.048574	6.274056	6.893753	10.80448
Itga9	17.62674	17.06746	22.63806	15.33567	14.91204	14.63793	19.21364	9.720941	10.80209
Vps33b	11.52801	11.45426	9.650971	10.66915	9.601615	10.88034	13.2312	9.413696	10.80204
Sephs1	12.4433	13.43631	13.53427	12.00047	10.97473	9.481108	11.32199	9.876545	10.79821
Spata13	10.86047	11.02371	10.96314	6.060297	9.879819	7.689639	9.023886	8.486517	10.79557
Zc3h7b	14.1277	13.86804	15.45523	10.93896	10.3419	9.95774	13.21055	9.587177	10.79318
Aldh4a1	11.10414	11.04161	10.72533	10.20393	10.10334	9.306062	10.22996	9.722672	10.78796
C1d	9.557024	9.275944	9.594756	10.88714	10.0959	9.921627	7.304171	9.395558	10.78385
Ccdc61	12.4233	11.56659	11.49109	11.85385	9.164525	13.18209	12.58033	9.195909	10.77965
Zbed5	8.066594	6.467644	8.146625	8.257333	8.812321	11.04333	5.885102	6.776637	10.77853
Trmt10c	11.57695	12.2008	12.65257	14.65716	11.53726	13.68602	11.31774	9.317896	10.77792
Zbtb41	11.30181	13.57666	9.837383	9.248644	6.281074	6.646255	11.91402	10.38476	10.77163
Rbm19	13.85952	10.84264	11.53223	12.49855	10.59446	10.87097	13.64307	12.16721	10.76268
Lrrcc1	10.37354	12.01119	11.24523	8.778718	9.219878	9.428862	10.70322	9.927366	10.75032
Rtl8b	10.4518	11.46637	9.886805	12.25537	14.0188	16.06888	8.641961	9.287724	10.75011
Birc2	9.946345	11.57611	11.13131	11.1028	10.85249	10.14565	12.2338	10.59686	10.74259
Fam189b	12.53714	15.06539	13.37789	9.710536	11.31053	11.68802	12.35324	10.70177	10.73714
Bysl	14.60032	11.76393	13.43673	9.211761	10.01447	8.87269	9.529845	10.86053	10.73474
Nup43	10.65786	9.600826	7.1777	7.649478	7.940255	7.392041	9.993838	9.369911	10.73132
E2f6	15.22843	12.93783	13.89051	11.42745	10.74325	9.149457	12.02311	10.04635	10.72875
Pskh1	14.14132	12.72396	15.59483	12.5506	12.31317	11.6244	16.11617	11.38254	10.727
Dgcr6	10.43299	12.07821	11.04798	13.30384	10.91041	12.59929	12.55905	10.94603	10.72346
Etv5	15.56385	12.39768	14.21654	8.22153	8.343892	7.216602	12.51801	11.29672	10.71402
Cdh5	13.53267	9.945627	10.05679	28.55954	17.43175	21.33783	7.863023	11.79859	10.71064
Naa38	13.69898	9.713191	12.73725	13.51582	11.41591	15.14268	11.61446	15.69209	10.71028
Btbd10	13.59484	13.17468	14.17396	10.95851	11.66427	11.08123	12.1609	9.988349	10.70966

Gpn1	14.56424	11.71408	13.18105	11.26156	9.278613	10.58733	12.03718	10.42936	10.69946
Klrb1b	23.45967	21.72976	17.46409	9.76409	16.22628	17.42453	10.17595	9.65194	10.699
Nudt8	8.745954	10.6923	10.01669	9.468468	10.54629	11.34927	10.27865	8.931385	10.68848
Gbgt1	12.7902	11.26517	17.1525	18.45843	15.10792	10.06146	13.73435	10.12158	10.68622
Ankrd13d	7.871635	11.98261	10.20324	12.33353	12.1824	11.39807	14.47838	11.46373	10.68594
Rab11fip4	7.253989	7.45391	7.958331	8.962048	10.50948	7.88729	10.08736	8.83196	10.6848
Sepsecs	12.83978	15.63557	17.77204	13.86124	12.73517	9.87299	14.44364	9.894602	10.68242
Smim26	7.231052	12.38026	9.160397	5.992122	8.14935	8.247362	11.58416	11.47445	10.68234
Agpat5	12.68314	12.05734	11.56964	9.372687	9.997124	10.00176	10.26546	9.925144	10.68063
Gm5884	13.80425	12.61612	13.87955	12.04159	12.09432	11.90257	13.93735	11.03825	10.67517
Gm15542	10.33147	10.26663	9.845986	6.365163	9.162216	9.450369	9.169228	8.945577	10.66737
Lin54	11.38161	11.13358	10.233	8.24959	8.400093	7.093177	14.59151	10.2544	10.66736
Ago1	13.8691	14.26061	15.45879	12.02935	12.52935	10.18679	14.85768	10.8092	10.6596
Srfbp1	10.47024	9.131872	10.09599	10.43233	8.620991	10.04902	10.79996	11.87506	10.65495
Zfp869	9.414011	10.43216	11.43761	8.147779	9.397487	9.39902	10.0722	10.02535	10.65056
Cad	19.62999	14.18487	15.30596	11.80761	10.54932	10.33429	15.08377	10.7229	10.64036
Ankrd49	13.01428	13.99583	14.96122	12.91835	13.45328	11.11436	11.56012	14.13265	10.63811
Gypc	12.48722	13.10262	13.16514	12.85705	11.22373	11.21098	12.44907	8.743412	10.6369
Polr3e	13.25114	11.22495	13.70902	12.17494	10.19397	9.792844	12.90974	11.0235	10.63634
Nedd1	13.60453	12.56673	12.42911	11.10976	8.607981	9.940039	11.61205	8.754749	10.63328
Gm46295	11.614	11.41642	9.06666	11.38277	10.46423	7.070031	17.87594	13.32489	10.63119
Ldlrad4	16.24173	18.69493	15.90225	13.52024	14.30357	12.36309	16.78664	11.48269	10.62712
Tmem129	12.64703	14.14084	14.15703	8.053372	10.18843	10.68696	11.32049	10.38416	10.59402
Akr1c13	7.874725	7.943517	11.43093	8.99402	9.685458	12.48394	7.627332	10.62504	10.59321
Rps2-ps13	7.620552	8.934662	10.82128	8.792638	7.590146	8.499734	9.499564	9.027851	10.58966
Etfrf1	13.06937	13.50314	10.33711	11.08496	10.86073	8.531398	12.40427	11.38605	10.58376
Rfx5	13.57667	13.29369	13.71969	17.29767	10.60686	10.6916	15.49385	10.19967	10.58096
Gm52186	12.08288	12.8898	14.25051	11.7304	12.17249	10.37929	15.05992	10.63489	10.57772
Bag5	10.73497	10.44778	10.01009	8.483141	10.42143	10.50286	10.53233	10.36887	10.57737
Zfp35	9.843274	10.41906	10.77314	10.52686	11.62984	9.682408	12.06905	12.20097	10.57633
Grap	12.59793	14.31341	13.709	14.94742	14.4523	14.16555	14.27826	11.79002	10.5743
Mcph1	11.08761	10.95125	10.49964	11.55814	8.238623	10.03058	12.51145	9.613039	10.56748
Polrmt	11.24681	11.37858	11.85253	12.54697	11.56768	11.2731	14.15311	10.53965	10.56467
Gna11	8.841274	10.04242	8.744054	8.742906	10.3383	9.871924	10.01784	9.041297	10.56436
Slco3a1	12.32173	8.399043	10.61538	7.197258	8.944531	8.577462	7.320186	10.14266	10.56008
Dnmbp	10.96561	9.677247	10.55545	12.28661	10.59215	9.301068	15.21887	9.116726	10.55937
Gmpr	15.21028	16.07544	16.89218	12.68701	10.98349	11.79864	11.54407	9.856186	10.55882
Lsg1	14.61812	12.92193	13.34329	13.33885	10.43587	12.42711	13.86403	12.77143	10.5583
Gm9761	13.19377	9.416281	10.35045	11.13383	10.89404	11.02585	11.8224	12.33128	10.55594
Dhx32	12.29692	13.80003	13.09106	11.07955	12.25066	10.89013	14.53516	9.724221	10.55453
Cstdc5	3.394167	2.006003	2.640215	0.500023	2.03532	4.06884	0	2.274075	10.54352
Ms4a4b	8.674582	4.128121	7.046164	7.750256	8.806757	11.36108	6.417012	9.391003	10.53391
Tmem35b	11.87611	13.68912	16.74531	11.61598	14.50919	13.63829	12.77761	13.90232	10.53037
Snai1	6.326984	4.672915	6.259312	2.788684	5.146317	6.198894	4.100289	6.284403	10.52837
Prr14l	13.89133	12.3039	12.36545	12.70157	11.19524	8.810794	15.02452	11.75444	10.52762
Ing3	9.980328	11.17589	11.10367	8.808659	10.30662	9.721006	11.06286	10.71112	10.52527
Dcp1a	14.17691	11.78766	13.76539	12.58455	12.13926	8.889328	13.49659	10.66778	10.52523
Ppp2r3c	9.825407	10.04608	9.504772	8.687507	7.943361	8.262288	9.259767	9.284059	10.5203
Nt5m	12.31014	15.02919	11.16532	12.83213	11.36019	11.89435	15.66262	9.053042	10.51893
Prnp	16.44671	15.34304	18.27062	13.98946	9.856061	11.23156	9.989885	9.250784	10.51443
Taf7	5.019982	4.420559	4.751906	8.414377	7.531236	6.458883	6.07338	8.44008	10.51307
Zbtb42	12.41698	10.2219	11.7967	10.00157	10.21664	8.859028	10.21414	10.48668	10.51279
Osbpl1a	12.01569	11.64834	12.35339	12.34964	9.58616	9.204496	15.26866	9.284421	10.51193
Gm3375	9.813174	11.69362	9.36821	6.843417	7.889447	10.90169	9.417948	10.92051	10.50675

Cfap126	11.10359	14.42014	14.69998	14.77481	11.71612	12.48442	14.44834	13.91814	10.50657
Adprm	10.1395	12.0387	13.8282	9.166097	12.62484	12.94877	13.56461	13.70756	10.50054
Sema4b	11.49382	10.17418	14.44973	9.960594	9.862187	8.423617	12.11798	11.32413	10.50002
Rap1gap2	11.10218	9.228704	11.76841	9.418864	8.804104	9.449976	12.16182	9.330366	10.49456
Slc25a10	13.54075	13.60146	14.22152	13.6165	13.85963	12.71431	14.52504	11.63853	10.48822
Atp10a	11.51532	8.203334	11.84571	9.102234	5.852834	6.563268	7.76037	9.061848	10.48237
Septin10	9.721367	9.358577	12.31266	12.83945	12.40428	10.23959	9.232854	10.5422	10.48162
Csnk1g3	12.60661	13.71956	13.61568	9.6075	10.60401	8.80337	11.14721	11.03119	10.47638
Mon1b	13.03485	11.52027	14.70301	10.16971	10.76191	9.056499	13.17724	10.28046	10.4761
Dcaf17	11.38761	13.03646	13.34832	10.89766	9.35856	12.22944	14.01602	12.29939	10.47412
Ankib1	13.75978	12.20228	11.47372	10.05044	8.283293	7.633126	12.19633	9.994314	10.46946
Agl	12.23661	13.03699	12.45318	10.58257	9.665483	9.361768	12.69947	9.710721	10.46858
Bbx	9.918352	10.51563	12.78482	9.891717	10.51263	7.491063	12.89656	10.57865	10.46491
Gmcl1	12.27491	10.63871	9.957128	15.12702	9.894217	9.607273	12.11151	9.567374	10.46139
4930402H24Rik	13.16225	13.17	11.06101	11.09377	9.010907	8.7099	15.17821	10.28482	10.45911
Gpsm1	9.962333	9.652666	9.963491	10.41982	9.807562	8.329757	11.92375	8.305621	10.45433
Cep192	13.38033	13.74909	15.05923	12.27907	11.00556	8.777494	15.81029	11.03729	10.45155
Eef2-ps2	12.76298	12.11944	11.61386	8.329905	12.97709	11.43708	10.99884	11.84354	10.45098
Sestd1	13.37711	12.8217	13.71996	12.66668	9.466521	8.555083	13.69765	11.7616	10.44921
Mexis	13.74362	16.69865	15.40883	11.1177	10.57011	11.97836	16.79764	9.086997	10.44793
Cnnm4	11.21586	11.01707	10.56374	8.470052	10.9008	8.559483	11.57965	10.18326	10.44755
Haus5	11.69377	11.05809	13.46329	9.995788	11.56999	13.85037	12.08609	8.801086	10.44712
Ano8	10.98856	10.46537	11.46968	9.726576	9.109307	9.274502	13.66066	10.02012	10.44357
Hrh2	11.07171	8.167685	7.991643	6.00888	7.307372	6.305024	5.464779	6.794158	10.43865
Aar2	11.58022	12.75782	14.72031	15.09044	11.42614	9.974271	13.17998	9.569305	10.43117
Cdc26	10.05568	11.53137	11.86482	12.71453	12.46434	11.85907	12.22254	11.90474	10.42749
Gm9392	8.484145	7.756428	9.940913	9.181028	8.238642	9.730419	8.854755	9.701204	10.42478
Det1	12.75297	11.14638	11.56477	10.59087	11.11827	10.77024	12.32307	10.40373	10.41456
Smad1	10.59385	9.665267	12.27186	11.37727	10.61587	10.35911	12.66307	9.875882	10.41295
Hmgxb4	13.67105	12.90314	15.89368	12.05471	11.02154	10.58885	12.97154	10.20345	10.40825
Ap4b1	10.96022	9.81384	11.8841	10.48384	9.957264	8.348215	12.80352	10.49794	10.40419
Grhpr	8.878752	8.59594	9.442892	9.518112	7.589799	7.650055	8.505173	8.824569	10.40236
Atg16l2	10.68284	11.67641	11.58083	9.986132	11.54055	13.50389	12.1798	10.80528	10.40145
Ttc3	10.91322	12.60513	12.19963	12.13901	10.0702	9.090624	14.19169	10.69631	10.40127
Zfp422	13.29695	12.2641	14.34419	11.79851	12.05774	13.74697	12.00463	11.31826	10.3985
Rassf5	12.44962	11.14299	13.05411	11.32178	11.1475	10.10259	12.83065	10.73885	10.39507
Cul4b	11.61517	12.29168	12.50328	10.58025	9.626615	9.070699	10.96245	9.781957	10.39424
Gm51686	13.39234	12.52837	8.098636	19.20172	10.184	7.584501	17.91904	9.983201	10.38938
Cox15	12.94094	13.12544	13.48556	10.98414	11.85162	11.06982	13.32734	10.48389	10.38467
Bub1b	15.12497	16.90812	18.61751	11.64454	9.814295	11.3322	12.9307	8.732341	10.38315
Zbtb38	13.22753	13.23629	14.74306	15.30183	12.00986	9.418955	16.95243	12.13213	10.37978
Fbxo25	10.41115	11.41124	12.13014	8.749984	10.39362	10.82529	10.07768	9.031891	10.37608
Bcas3	13.16359	13.47362	16.54684	10.66581	11.57825	9.293577	14.22291	12.53303	10.37262
Fgfr1	19.64451	22.96216	18.77282	14.99265	16.07927	16.09783	18.88257	11.57043	10.37203
Cracr2b	8.5441	14.55185	14.59239	17.05784	17.27613	11.70003	16.98222	10.26239	10.36703
Rad50	13.17905	12.29631	13.7078	10.66605	10.58431	8.556171	12.42031	10.26354	10.36651
Trp53i13	10.79541	10.32143	11.36491	9.806727	12.21403	11.52203	10.6123	10.70333	10.36634
Pik3r4	13.91627	13.07545	15.33692	12.77731	10.24838	8.869971	14.82897	10.74238	10.36409
Snhg16	11.12056	11.0088	9.886093	8.764703	6.433114	11.1219	10.04631	10.98001	10.36335
Gm31814	3.934314	3.852497	4.93635	3.841144	3.883252	6.747449	2.957316	5.176087	10.36295
Ptger2	13.19954	13.93877	14.85768	9.857069	10.35267	10.00456	12.35839	12.46869	10.36027
Adamtsl5	17.82204	29.53773	19.79629	12.24645	9.709274	9.806178	11.99797	10.02513	10.35471
Ttf1	10.68524	12.15053	12.22909	12.73713	12.6887	10.62558	14.25519	12.30172	10.35443
4932438A13Rik	13.29497	11.29077	12.00026	11.37151	9.162963	7.380549	13.71525	8.742497	10.35273

Rpusd4	13.1731	12.36882	14.87159	13.19134	12.98663	12.55295	14.12522	13.1589	10.3499
Ice1	14.68477	13.32014	13.58842	11.74378	9.631016	8.530091	14.14719	10.24702	10.34718
Spryd7	10.79677	8.825131	7.903003	8.990111	7.199216	8.246424	9.542578	10.17509	10.34258
Toe1	10.43605	9.582213	10.7686	8.236184	8.046002	9.6254	11.10006	9.646995	10.34118
Klhl22	11.34298	13.14889	14.28431	12.13573	13.00876	12.10976	13.01251	10.37556	10.32975
Gm10156	8.332047	7.440859	7.461589	8.826902	9.128246	9.820373	9.568429	9.643948	10.32873
0610030E20Rik	11.68313	11.44928	11.69645	11.34419	11.59383	9.167528	12.00094	10.52006	10.32586
Klhl17	11.56952	12.21747	12.7114	13.31547	11.77195	10.90325	14.18192	10.96278	10.32556
Gm12174	9.668007	10.00843	9.852721	8.822838	12.06326	13.07149	9.423182	11.24395	10.32254
Rsbn1l	9.013427	9.128801	9.104319	8.51628	8.498731	7.227649	11.01805	9.978681	10.32203
Homer1	11.51584	13.23585	10.72622	10.95262	9.020221	9.061876	9.629414	11.22133	10.31377
Bpgm	11.43634	11.20131	10.46316	9.638392	11.77101	9.674881	6.466565	9.667834	10.31331
4921524J17Rik	9.140989	9.411929	12.17706	10.08341	10.75005	10.26717	8.414927	11.66288	10.29941
Trim33	11.10338	11.68976	11.34779	10.50546	8.453533	8.269129	11.34009	9.864654	10.29446
Snip1	10.09039	10.98268	13.51844	8.976434	11.32042	10.92655	12.74917	11.47445	10.29271
Foxj3	13.41301	12.71669	14.62117	12.37341	10.8819	9.330292	14.21753	11.72318	10.28114
Phf19	26.11345	25.68137	16.96662	9.700034	10.50202	15.57632	18.59837	11.59484	10.27875
Prkd2	11.24508	11.02737	11.22927	12.45858	12.7799	10.96891	12.55992	10.49457	10.27711
Gm14494	8.275145	7.875521	7.518816	9.358233	8.911374	10.05341	7.155263	8.754166	10.25819
Pde12	13.11725	11.67226	11.62622	11.40354	8.812235	10.67768	10.4562	10.90349	10.25605
Pan2	12.39039	12.98036	13.1695	12.71394	12.27395	10.35014	16.45555	10.68029	10.25403
Tmco4	11.44448	11.39356	13.02525	11.6863	9.981534	12.97332	11.99803	9.958956	10.24299
Cd7	11.43876	7.481724	9.843794	12.64441	14.79577	19.18837	10.06289	8.003484	10.24295
Rmdn1	10.51332	11.77867	11.99676	12.48083	12.39466	9.893706	14.16893	11.068	10.24083
Snora70	8.595049	11.57242	9.02585	30.34151	11.86945	13.90976	17.32265	7.774162	10.23981
Atg4a	9.068739	8.156287	9.420707	6.334029	8.508441	7.313215	9.317202	9.122579	10.23821
Ube3c	13.77966	12.20499	12.89996	9.364998	10.18626	8.627752	12.53429	10.35094	10.23574
Tcp11l1	12.81768	12.12234	11.0668	11.83547	8.524148	8.057972	13.78611	10.3551	10.23048
Klri1	14.75261	12.47005	11.71083	8.94671	17.57853	10.88973	12.51257	7.999252	10.21439
Stradb	11.34018	13.55183	14.27159	13.03471	11.76242	11.07095	12.39934	11.73478	10.2064
Tbcc	9.234664	8.820075	9.596743	10.19994	11.31603	10.06988	10.41499	9.336454	10.20625
Cmc2	9.047184	11.82602	12.39801	11.10207	9.496416	9.042997	11.02191	6.561154	10.20055
Oit3	12.67564	11.35938	12.12593	12.21899	7.717616	8.097831	10.90687	9.794685	10.19396
Lias	10.89317	11.10658	9.612738	10.30483	9.532789	10.44146	9.591535	10.37137	10.19263
BC051226	7.523737	8.885969	10.75887	10.3036	12.25682	9.032825	9.140396	11.56187	10.19007
C330018D20Rik	7.536511	8.705489	9.112999	11.47432	10.6246	8.938323	8.493483	9.283553	10.18601
Snx16	9.179679	9.82842	8.303175	10.15743	8.035502	8.052142	10.92941	8.461475	10.18578
Kctd6	11.79457	12.15887	12.48133	14.13158	13.36282	11.8412	20.98814	14.25161	10.17558
Srsf4	9.420044	9.517614	10.28208	9.244989	9.369761	7.164697	11.33758	9.320063	10.17366
Polr3k	9.173341	9.999542	9.307095	8.141911	8.646336	10.28853	8.246995	9.058735	10.17007
Mcrip2	11.34578	12.87401	11.14326	11.82269	10.28796	12.56554	13.48188	12.35472	10.16602
Krr1	8.810097	9.872507	10.16668	8.528747	8.253088	8.024013	9.897972	9.054468	10.16511
Zfp692	10.25325	12.0426	9.549266	13.8184	10.38065	9.835079	14.90902	12.75506	10.16427
Panx1	14.80129	13.69605	16.56655	10.7864	10.96684	10.25348	12.45369	10.75999	10.16006
Vopp1	15.46967	16.72943	15.36954	9.606782	10.30737	10.66814	12.0073	12.08951	10.15954
L3mbtl2	12.75939	12.45243	12.86616	12.56229	10.16669	11.7216	12.8828	9.943071	10.15409
Zfp90	12.47566	12.73521	13.16912	14.99118	12.30104	14.01903	17.14173	11.7982	10.15181
BC024978	13.294	10.51664	12.56464	11.55844	11.79931	8.367558	13.99179	9.608861	10.14956
Tut1	10.83514	9.232564	10.75224	10.14681	9.718149	9.961632	10.30712	11.35775	10.14638
Cacna1d	13.84447	14.75613	15.78453	11.1931	9.699809	7.915551	17.08364	10.87743	10.14294
Myog	6.218053	8.181495	12.32516	7.412417	8.633285	10.877	7.007391	11.94738	10.13907
Myoz1	14.18083	16.32223	11.3974	11.11923	9.792631	18.18526	6.597684	10.44228	10.12649
Gm6210	11.33541	15.32779	13.6568	7.339851	8.912746	10.48263	11.36152	10.86772	10.12528
Znhit6	11.18417	11.31489	8.690866	9.96136	8.314439	8.929007	10.43672	13.13816	10.12269

Rusf1	10.66884	10.19294	10.74487	14.20327	10.9918	10.28114	16.33405	10.22898	10.12083
Gnpda2	8.092643	12.14114	11.24109	9.28536	11.56102	9.338787	11.4739	10.56966	10.1192
Plpp3	13.13191	12.31009	13.76469	16.92879	10.887	13.14922	12.8839	10.08242	10.10962
Mppe1	9.076684	9.296358	10.33089	9.360131	11.84621	10.48523	11.52909	9.427519	10.10726
Nr2c2ap	8.493297	10.93488	10.65646	11.15859	9.951826	13.66178	9.363462	9.115635	10.10632
E2f8	16.08913	15.01998	15.64992	10.86633	11.01397	10.78518	13.60701	10.35743	10.10356
Thap6	11.81813	14.36077	8.966522	7.759578	9.156741	6.964995	12.32383	9.728759	10.09852
Lrrc8a	11.75485	12.60737	13.51574	12.22032	10.54979	10.40051	15.0451	9.082456	10.09798
Tmem170b	9.612794	13.38889	13.0253	11.05559	10.23558	8.414006	14.03137	10.47728	10.09173
Fam210b	15.03998	13.25576	17.31548	12.4776	14.69867	13.98937	13.82744	12.40471	10.08411
Ylpm1	14.57623	13.26253	14.58413	12.20531	10.72638	8.171564	15.66812	10.26227	10.0837
Zxdc	10.97283	10.91636	11.16543	10.66678	10.77576	8.959471	13.72779	10.59032	10.08039
Paxip1	16.16474	15.18858	15.06179	12.13971	10.36004	10.76844	15.27403	11.67697	10.07757
Susd1	7.726479	6.836994	7.10827	9.324714	9.7387	10.41037	9.478578	6.83247	10.07363
Olfml2b	7.558922	8.5928	9.781163	5.949142	4.759551	9.375554	5.239204	7.725512	10.0724
Plaat3	8.941623	10.47844	10.73119	12.89273	14.42381	15.50425	11.89858	11.65258	10.0668
Snord22	14.95948	8.337452	11.00089	11.37552	12.5711	4.882608	13.64238	16.33743	10.06427
Eef1akmt2	9.386339	10.38525	10.04748	12.68808	10.47591	8.002052	8.564384	12.91634	10.06427
Efemp2	10.83109	11.83273	13.57606	13.95903	11.5709	14.16772	11.23129	11.05854	10.05133
Cpox	12.09336	11.67937	13.34775	9.430164	9.263785	9.6066	11.6898	9.907067	10.05005
Epas1	14.91772	12.51804	10.37747	20.91397	14.43143	15.86136	10.05601	11.90824	10.02958
Rpl7a-ps11	9.752993	7.807219	6.224673	9.762166	7.883125	12.1048	8.289642	9.224714	10.02932
Smim27	9.684963	5.828815	7.515069	8.468385	6.679341	10.65497	7.075958	6.20035	10.02637
Pfkfb2	14.03407	13.55568	12.91843	12.28913	11.14933	8.307813	14.4568	8.907873	10.02624
Bicral	10.42137	10.60241	12.60723	11.89256	9.93387	8.568412	13.75303	9.34157	10.01924
Nfkbil1	10.03017	10.15856	11.39217	9.007513	10.25611	10.67771	12.17482	12.43682	10.00428
Ramp2	10.80788	9.447569	9.410306	18.0176	13.56294	22.2825	5.12528	9.563796	9.997902
Tmem41a	12.42181	11.74636	12.37724	9.653035	9.551947	9.870567	12.36305	8.410153	9.99745
Gm39556	6.858901	7.066778	7.143157	5.974967	7.989973	6.028373	7.03043	11.00984	9.994804
Gstm2	14.24936	13.88965	15.21901	13.68831	10.80553	9.249014	10.67043	9.675933	9.993494
Trappc2	9.72376	10.11769	8.756611	8.120531	10.26305	9.109011	9.70194	7.414394	9.989759
Tbc1d4	14.06916	13.69717	14.51807	9.487493	11.05665	10.01222	18.33536	8.995197	9.981384
Htra3	15.15826	21.67567	15.9632	12.07864	13.93462	13.87863	15.12211	10.05949	9.974285
Wdr75	13.39318	11.2739	11.54783	8.996248	8.260885	10.02233	11.45189	10.38955	9.973681
Acvr1	11.48303	11.6118	14.91969	9.830016	9.338658	8.569188	10.34089	10.21044	9.97363
Cep162	14.37821	13.94677	15.54189	11.55367	9.899738	9.497513	15.43958	9.857205	9.966336
Gm28913	13.85952	13.53162	14.73657	14.65232	9.216598	8.364679	13.4198	11.2973	9.964948
Mterf4	9.809416	11.80914	13.08074	11.38021	14.57978	10.99569	13.97887	10.94328	9.963084
Tma16	7.813084	7.941181	7.764222	11.08489	7.156723	6.02875	7.35513	11.7505	9.960371
Mettl2	12.93646	10.93808	11.48825	11.93931	9.819967	10.64804	12.26805	11.84806	9.957517
Tmem9	10.74587	9.694768	13.29149	12.49809	12.40406	11.16646	9.693567	9.182704	9.95225
Fra10ac1	9.670068	11.83622	11.92414	10.59935	8.163114	11.38146	10.90309	12.81636	9.94956
Flt3	16.60265	14.57062	15.33047	13.6082	14.26619	15.48663	12.74233	8.510146	9.948628
Pcnt	12.53732	11.93149	13.28503	11.83925	10.62844	9.652918	15.56818	9.727401	9.94853
D17H6S53E	11.47354	10.74081	11.39664	8.479163	13.7336	9.862822	11.69677	11.09773	9.941185
Yars2	10.71612	10.08196	9.824252	9.910167	7.654437	7.927946	8.085221	10.30482	9.941057
LOC115490257	11.52673	5.474304	4.9406	6.549805	6.273101	4.568381	12.48075	9.929375	9.939709
Pclaf	14.9569	19.31808	17.8621	10.64675	10.2412	11.81276	11.85807	8.314289	9.938832
Stk38l	15.34446	13.78971	13.79512	10.27078	10.90543	9.582118	12.75563	8.859914	9.938463
Aqp9	7.105594	6.834866	7.286786	6.38432	10.0307	10.57369	7.120312	9.546862	9.934944
Ttc32	7.705891	6.745437	8.094898	6.174283	8.394978	7.690108	7.104953	10.18702	9.933431
Cry1	14.61315	13.65763	10.79109	7.219951	8.420467	7.292343	8.530107	11.02552	9.933291
Ccdc117	9.842619	11.03534	12.01135	8.102223	9.187764	9.011727	10.61074	11.21744	9.924485
Haus3	8.937047	10.0168	10.35683	9.507664	9.673439	10.28496	10.40982	9.400358	9.923913

Penk	5.826566	6.366523	6.084328	11.58786	7.426079	8.59739	8.855951	8.158032	9.922324
Tnk2	8.541568	8.539306	8.255071	9.249174	8.056676	10.76615	8.594701	9.754982	9.921689
Nxpe4	7.373341	6.567218	8.695244	11.78223	10.27057	8.587105	8.41539	12.85775	9.921105
Taf3	12.65672	10.0685	12.04663	9.534021	9.636707	10.1041	12.21085	10.74324	9.918696
Gpatch3	10.12997	8.888433	9.577533	9.77048	9.146838	8.830378	10.3268	13.12315	9.918553
Nr2c1	12.75651	11.86162	14.72445	11.56438	13.67922	10.88767	16.90192	11.71168	9.918439
Syce2	12.66946	14.89994	10.25394	13.39956	11.02005	11.30998	10.44534	10.38915	9.911375
Zkscan17	12.90821	14.36244	17.2731	12.78698	12.99658	11.8006	14.90087	12.05564	9.91092
Snord68	16.16944	10.36696	10.39584	17.22735	8.799767	6.408423	5.968542	9.896713	9.907012
Zer1	10.04843	11.18318	12.4836	11.44716	12.9117	10.54896	13.95028	10.46267	9.904344
Il6	8.855757	9.240605	5.11953	4.632137	8.092861	5.58592	4.973234	19.25122	9.903963
Trp53bp1	12.44336	11.96845	12.10919	10.0511	9.792671	8.354225	12.73597	8.230487	9.902215
Zfp691	10.24817	9.348799	12.03338	10.99072	16.42253	12.38827	13.70091	14.58594	9.900762
Flywch1	11.13622	10.4014	11.3806	10.65638	9.469658	9.624128	12.78871	10.729	9.897799
Ddah2	6.699464	8.978438	9.673475	9.701445	10.08916	10.29994	10.60273	11.40165	9.897035
Rrad	9.40789	8.80765	11.60802	8.925067	10.29882	10.30014	9.31372	10.08976	9.894129
Nup133	11.63181	10.30185	12.02007	9.194332	9.763873	7.691896	12.12868	8.889162	9.893953
Zfp821	8.85491	8.45285	9.520135	9.245575	10.05975	8.744408	9.478848	9.063048	9.88629
H2-DMb2	15.75525	14.10671	12.73141	9.767457	17.88642	14.35196	11.00479	11.40939	9.885948
Gm28809	11.60126	12.89522	9.255513	10.30339	10.37417	8.271575	15.33134	10.66611	9.87536
Ntmt1	9.344582	9.754301	9.490937	9.195945	10.65195	12.31313	10.95918	10.4643	9.875287
Smg6	13.65623	13.59602	14.42323	10.55937	9.657726	10.79313	15.86942	11.4992	9.869971
Col18a1	16.09936	19.16716	19.49579	9.456514	7.338802	9.529569	6.494617	9.141586	9.865118
Abcd2	10.36867	11.18916	12.15778	12.05945	12.24942	11.02736	13.63287	11.80406	9.863841
Gm20234	7.142327	8.770881	4.859291	6.474223	7.033644	4.942517	12.70499	7.077754	9.863598
Gng11	7.029228	7.53961	8.489111	11.01232	8.210309	13.85936	4.934756	5.74546	9.85961
Zfp954	9.607087	11.4001	9.47211	11.15	9.071925	8.708157	11.08268	10.44248	9.856756
Sbk1	14.60918	13.3033	15.82229	9.591866	10.07296	10.3409	12.2138	10.33709	9.855097
Josd1	11.25422	11.35458	12.07233	11.045	9.427211	9.440812	12.67849	10.06195	9.854471
Sh3bp5l	10.5027	11.30024	12.36188	10.37071	8.215841	9.678547	8.668842	8.195818	9.852229
Polk	11.52899	13.60442	12.44362	10.42391	9.494327	7.934657	14.11804	10.56012	9.845816
Gm7392	11.00609	8.266468	10.73558	10.80932	7.117459	10.17808	9.128359	12.67814	9.842261
Gon4l	11.29205	11.68122	10.34748	12.30228	11.36403	9.196855	14.28747	9.696039	9.833602
LOC115488689	7.305791	3.857475	11.17486	6.837522	7.367247	6.358745	10.66009	8.592495	9.830214
Ntng2	9.234388	7.848905	7.299506	8.835551	9.580514	8.569019	9.24902	7.492875	9.82951
Ankmy2	8.725942	8.499271	10.9257	10.43501	11.03029	9.389938	13.77401	8.998483	9.828707
Zfp991	5.588028	6.555469	6.573732	6.496173	9.01673	8.643703	8.060778	9.071389	9.827986
Cd3eap	10.62892	8.632987	9.594267	9.784645	8.337858	10.08015	8.97193	11.03548	9.824748
Epg5	12.51136	11.59843	12.70183	11.25103	9.779741	8.272692	15.74393	9.21294	9.822953
Enpp1	9.358135	7.876136	12.22228	9.786706	10.45442	10.24252	14.13065	9.088434	9.822337
Ppfibp2	12.82254	13.03397	12.23681	12.75071	13.61905	11.85788	15.03412	9.070578	9.820138
Heatr5b	12.16072	10.52211	12.31717	9.985815	9.268669	7.972449	12.97817	8.060458	9.817233
Gcc2	11.52212	11.76228	13.32117	11.02036	9.5572	8.694049	11.24041	9.985207	9.810723
Igf2bp3	9.890228	9.157354	9.766346	7.541876	8.572662	6.397183	9.877481	9.363565	9.810201
Col5a2	8.732647	10.03838	12.05279	12.40057	7.015869	11.04399	5.716808	9.415467	9.808666
Tmem268	13.11025	12.49286	14.24925	13.64815	14.81347	13.64477	18.20447	12.81881	9.799895
Zcchc10	8.423763	6.24602	8.865828	8.376577	6.132535	8.034745	7.977049	7.936266	9.794044
Pggt1b	10.45542	9.033202	11.10511	10.90058	9.552379	6.577066	13.40304	10.26326	9.791141
Septin5	3.15374	2.677442	1.981092	4.328301	3.053234	8.701235	2.20895	2.258206	9.787464
2500002B13Rik	13.25287	5.21257	6.533864	6.125281	3.555462	7.077316	5.9806	8.352781	9.784703
Gm4754	8.543116	8.949455	7.74502	5.867231	8.0193	9.207668	8.130981	8.192401	9.782534
AI504432	14.50949	11.15982	12.91105	8.342712	8.704532	5.724118	8.877883	11.08203	9.77835
Slc35d2	9.55918	9.301201	10.88163	10.04661	8.511925	7.3068	10.12086	9.495912	9.777388
Ankrd13b	16.51592	19.97368	17.81155	8.590706	9.503749	8.352311	10.79112	9.54483	9.774919

Adgrg3	4.227749	4.057808	4.160043	3.028737	4.242938	5.423118	4.087681	3.678975	9.770261
Ckap2l	19.21359	23.64309	23.22911	11.21201	11.48609	13.75466	16.07139	11.54487	9.769307
Rps6ka2	10.83567	12.48907	11.00776	9.915099	7.20859	8.598862	9.301409	7.419835	9.767746
Gm9512	10.59253	9.301201	13.41492	8.87307	10.14303	8.411471	8.924923	11.07171	9.766414
Dusp10	7.765416	5.357265	6.832024	7.228412	5.727495	5.561391	10.2889	9.311283	9.766154
Slc25a43	13.01493	11.04705	10.41315	7.599998	8.597575	7.989722	12.53573	11.81146	9.765154
Csad	10.21018	10.39514	10.34432	10.52314	12.53492	9.245341	13.21839	8.505952	9.756546
Spsb1	11.54128	10.89521	15.5898	10.1413	14.88039	11.17646	19.73077	9.416185	9.754597
Marchf3	12.83288	12.21851	13.9638	7.112131	10.52713	9.842072	13.33417	11.30587	9.752088
Dhdh	13.64257	16.66564	15.6827	11.33857	12.32031	7.875805	15.66465	11.54797	9.751933
Insig2	9.769137	12.83997	10.7258	10.09444	7.279428	8.283184	11.05968	8.596197	9.743389
Borcs7	9.885096	9.045684	9.605965	8.334998	8.856384	8.411055	9.532113	9.169013	9.737039
C87436	10.59128	13.61268	11.30164	10.90652	10.25584	9.364187	12.43518	11.05436	9.735428
LOC115487640	2.917793	7.275062	4.377198	9.429708	2.778874	4.047425	5.277448	9.723086	9.733205
Zbtb8os	10.88381	9.773677	10.97181	11.44807	12.07946	11.82939	9.926874	9.763828	9.732661
Hsf2	10.50428	11.0876	10.27298	8.785095	6.723116	8.326308	8.934382	7.284504	9.729494
Dpp4	10.78065	9.848269	10.67094	8.495302	12.94429	12.9793	7.969736	8.169135	9.728567
Nedd4l	10.24415	9.332937	9.883393	12.48558	8.757562	8.927055	14.21381	8.944751	9.728389
Rundc1	9.567203	9.52454	10.56825	10.35355	9.374379	9.564811	9.353685	7.713856	9.726287
Cd79b	7.141174	5.763329	9.16244	10.46489	13.69184	11.07897	11.02258	8.40941	9.716708
Suclg2	8.947574	7.750786	7.97995	7.888464	9.487936	6.589693	10.60633	7.816372	9.714696
Wipi1	9.122719	9.646164	11.38184	7.281566	8.223448	7.194927	11.57312	8.714772	9.712543
Rprd1a	10.38549	11.96258	12.43478	9.454589	9.658848	7.890758	11.94338	8.634599	9.712415
Mcoln2	13.70379	12.95732	11.0355	9.158425	10.57243	9.360618	14.27852	10.5692	9.710886
Telo2	11.39341	9.961139	11.51805	10.12797	9.089439	10.2854	12.5658	9.344939	9.707227
Zfp574	10.98334	10.78078	10.27201	10.43794	10.92385	10.06062	13.81259	10.53995	9.70618
Heatr6	12.01989	10.77935	11.171	11.55414	9.735121	9.760052	12.87766	10.82073	9.705018
Rwdd4a	12.18811	10.76965	11.00626	9.150572	8.638796	9.430281	11.70417	10.90695	9.701175
Map3k9	7.905518	6.760479	6.713012	5.867085	7.24501	6.253242	10.46609	7.148119	9.698703
Med31	6.780538	7.381587	7.020105	7.834661	7.32177	9.71475	5.626161	7.956014	9.693683
Phtf2	13.50607	14.43012	11.25135	8.405228	8.36527	6.092001	10.19737	7.933122	9.690201
Gm11427	13.81478	13.42106	10.59495	13.98652	11.79362	11.78256	14.16729	9.949955	9.687425
Dip2b	12.44236	11.40234	13.24201	11.23307	11.04437	7.973665	16.95604	10.4305	9.687181
Mtmr4	11.98607	11.54776	13.13055	11.51373	10.58244	11.10273	13.69276	10.08715	9.680857
Megf9	9.396589	8.793749	7.188592	9.559999	6.983486	6.529811	10.10481	5.705747	9.680529
Smg8	11.35955	9.252271	11.55022	9.687109	10.01551	8.976965	12.4878	10.01245	9.678381
Gm22571	5.465725	1.557478	3.904543	0.776444	2.974569	2.888303	12.10521	5.94732	9.674453
Gpd2	12.07406	10.37926	10.43777	8.229892	7.309131	7.306971	9.084079	7.607438	9.674321
Foxm1	15.95194	19.81084	18.20206	12.74311	10.37828	11.24007	14.32122	8.343621	9.659621
Nemp1	14.83451	15.95826	16.58596	11.59816	11.45532	10.81974	12.54453	9.820801	9.657329
C2cd3	12.45679	11.64715	12.58353	12.09335	10.7387	8.424568	14.06784	9.499992	9.654872
Cbll1	13.47475	11.35569	12.23549	10.41645	10.27554	9.730637	12.20626	12.919	9.654426
Clk2	7.536897	10.38039	7.489914	9.992905	8.909907	8.496623	10.93039	8.975526	9.646189
Zfp68	7.702721	7.861148	8.014632	6.922162	7.233466	7.764769	8.517438	8.233382	9.644108
Bcl11a	12.43611	10.57243	8.316676	9.610069	12.73588	14.27173	8.943135	6.419489	9.639255
Tmem86b	14.74159	11.18343	13.7282	11.63109	14.26989	8.939387	13.58767	11.18234	9.627739
Gm30489	21.37229	15.54492	13.99398	12.44626	10.12114	3.82185	20.62484	12.14165	9.622146
Mamdc2	20.92629	33.31011	25.8287	9.397113	9.300653	10.15211	12.56505	9.803814	9.608239
Abcd3	14.23587	13.03928	14.54846	10.93439	8.555618	8.56474	11.82152	10.68736	9.6068
Snord34	6.719765	5.864141	10.92089	0	3.199915	6.991007	7.813364	5.598141	9.6068
Bphl	9.163735	10.50266	7.848309	8.155853	7.859518	10.39394	12.97708	8.098132	9.602413
Hspg2	12.51323	10.89741	12.77836	20.64794	12.11276	12.6148	8.199847	10.63087	9.599608
Ssbp2	9.05387	10.37749	9.19007	7.760182	10.55329	8.841359	7.995882	9.488622	9.595091
Pcmtd1	9.991178	11.91651	11.81994	10.35456	10.22606	8.469568	13.79086	9.728776	9.59462

Poglut2	11.28322	12.22862	10.16324	8.753036	7.906159	9.44167	11.36591	9.629848	9.594399
Haghl	9.378804	11.26378	10.80407	12.15833	14.02974	11.3978	12.68778	10.00483	9.594036
Slfn10-ps	10.00196	8.665278	9.480805	10.96885	8.289818	6.644417	10.28845	8.889996	9.593087
Gm5776	7.906887	8.065061	7.997668	7.683901	12.06581	8.724595	6.221988	11.54884	9.59123
Eml4	11.33172	9.809792	11.17746	8.721881	8.763679	8.775099	11.89741	10.08256	9.579843
Gnpnat1	7.530604	9.641209	7.93209	6.320015	6.790572	7.655534	7.369252	8.720803	9.569779
Kif13a	12.02262	12.36104	13.11417	10.40566	9.458532	7.39218	13.43574	9.371079	9.565524
Smc2	14.91899	17.22063	16.43515	10.36862	8.56586	9.943335	14.09604	8.534214	9.560131
Zfp687	12.15337	10.34948	11.74573	10.66759	10.15015	9.293834	12.91138	8.87867	9.556174
AF357399	9.436266	5.881965	1.17967	14.07511	6.740247	4.363182	11.58151	9.545766	9.5557
Rc3h1	9.918535	10.70057	11.20874	9.407376	9.50864	7.328568	13.47239	10.15287	9.554015
Gm4459	10.11134	8.606536	5.143437	7.974422	8.799767	9.511873	8.738847	8.216139	9.553932
Leng1	9.594568	9.693946	9.495467	8.967876	9.925088	10.77241	9.036152	11.32927	9.550366
LOC115489078	8.469704	10.75092	13.09106	9.187921	5.133198	5.696376	14.3245	9.530168	9.540086
Optn	10.45212	9.896133	8.975933	7.039915	5.946147	8.969859	9.160767	7.722322	9.536765
Ttll5	13.04922	13.0707	13.07893	9.360749	8.562661	9.044119	12.8753	10.8946	9.535982
Sfxn5	9.313468	6.450029	7.163822	5.912136	6.987608	5.644384	6.373708	6.277892	9.524597
Clasp1	12.17076	12.64199	13.70193	10.91634	8.468687	9.129994	13.91793	9.615816	9.521195
Uros	11.15612	12.93202	12.03826	13.13719	13.421	14.16301	15.37387	13.78959	9.51353
Abcc4	13.41839	11.15222	12.8454	9.487157	9.907066	7.447242	9.425967	10.3338	9.513469
Zfp142	11.03036	10.03032	10.92667	10.07667	9.19659	8.938702	14.16178	10.15676	9.512551
Ttll1	9.113477	9.628633	8.654752	9.385125	7.546337	7.177434	8.916129	7.853002	9.510732
Ccn1	6.369363	6.979468	6.479462	12.58582	7.78441	9.125999	5.876718	10.98333	9.50969
Zscan21	9.427221	10.00067	11.55406	10.74203	12.97936	8.97237	11.42342	8.689942	9.509302
Twsg1	12.12839	14.92829	12.84331	8.610633	7.673983	7.451429	8.776152	7.778189	9.506199
Gm10175	11.51737	6.772011	10.48718	10.17081	6.957956	9.856055	7.817402	7.528662	9.50254
Ccnyl1	9.327529	10.81124	10.13083	8.705545	7.923065	8.477451	9.0483	7.811544	9.501558
Mical2	18.32522	23.19569	18.07042	9.605167	8.628502	10.31049	12.9394	7.6312	9.499959
Gm23201	15.57249	13.66731	6.852692	8.052335	11.27161	10.36868	13.51975	7.116737	9.498858
Aurka	16.3947	17.22544	16.68888	9.996768	10.60425	13.70193	12.35366	7.902067	9.497919
Gm15564	5.268907	7.082664	6.014125	9.738437	9.272482	15.25311	9.0564	6.815918	9.497632
Bod1	11.50676	11.75218	10.47988	10.26268	10.16806	12.32319	10.78935	8.922571	9.497164
P3h1	11.64846	10.81558	11.15951	10.8227	9.076803	9.484557	11.39069	8.009791	9.494658
Nudt13	9.429951	8.585053	8.220767	11.03459	8.828762	9.058364	11.44544	10.30432	9.4881
Serpinb2	9.249048	11.29922	10.85273	10.20362	15.20771	12.94683	18.77004	20.44919	9.48501
Crbn	9.616122	10.95104	10.60133	7.560855	7.773897	8.685882	9.846289	9.751662	9.484729
Taf1c	9.397449	7.861855	9.346901	10.8167	10.31056	8.212968	12.54117	8.046008	9.481533
Wdr44	10.19487	9.274619	9.507134	7.79512	9.000955	6.905299	10.80745	9.535998	9.480269
Gm9522	3.711733	7.546081	6.234532	7.050645	7.613876	5.940856	13.23018	6.478878	9.478984
Adnp2	11.0532	9.941195	10.28662	10.83332	8.320697	7.540761	10.43896	9.906165	9.474901
Gm6552	8.00137	10.83009	8.881673	6.688748	9.965161	8.497132	8.587885	10.54812	9.469669
Tirap	11.59256	10.06034	11.74802	7.967972	9.008614	8.714469	10.11934	8.407727	9.467124
Cep89	10.7059	11.0343	12.76506	9.726642	8.60569	10.78297	10.75686	7.495735	9.464687
Vhl	10.45243	9.60266	10.75519	10.09405	11.12255	9.740431	10.3876	10.6407	9.463987
Eva1b	13.17823	13.06202	14.31876	9.787866	12.16343	11.0208	11.98087	11.73374	9.458786
Aacs	10.87808	7.975759	8.574253	7.604994	9.212733	8.041309	9.763219	9.392786	9.452486
Mdk	7.991072	6.760096	5.708572	8.017259	6.455428	9.96316	10.47145	10.86898	9.45225
Fanca	10.43725	9.467905	8.73369	10.0137	7.692512	7.930196	9.784569	6.741375	9.447747
Tmem143	10.13911	12.16717	15.79923	13.56235	12.3041	12.43121	13.45404	11.58412	9.445269
Dnajc19-ps	7.815516	8.834004	6.670016	9.844201	10.917	8.769421	5.385151	8.036429	9.43525
Soga1	17.61975	16.22986	17.94366	13.35659	12.45357	10.23233	17.32683	10.91773	9.43179
Tmem203	6.671977	6.533229	7.946641	7.11712	12.9397	7.628407	7.146578	6.814367	9.422862
Krit1	8.026254	10.18715	9.14589	8.293521	9.055194	7.404281	9.542907	8.090198	9.417214
Ulbp1	14.87227	18.76223	15.8175	14.07159	9.301856	8.72418	14.22413	7.555449	9.414471

Mmgt1	13.07361	14.08837	12.80527	9.109659	9.187394	7.136759	10.86278	9.531417	9.412747
Nes	11.91923	14.13097	14.09243	8.937767	7.261779	9.392567	7.890679	9.058225	9.411053
Jag1	12.35321	6.935215	7.206877	10.98938	6.180503	7.522576	14.86431	6.976165	9.407423
Fam151b	7.455878	8.235602	8.178365	9.60646	9.353332	8.044123	6.545976	9.808456	9.398409
Pla2g12a	8.497203	12.21821	8.291924	7.055011	7.581942	7.285767	8.270694	9.661077	9.395936
Fbxl20	10.71755	13.808	12.92771	11.31186	13.91168	11.04397	16.16788	11.4882	9.395109
Rmi1	7.977094	9.121016	9.913193	10.03804	9.266268	8.676798	11.65959	10.16719	9.394862
Dtd2	11.36913	12.20278	13.5904	18.08872	12.37467	9.762832	11.02315	12.57707	9.391022
Med26	11.21527	9.566707	10.08579	8.940089	8.9445	8.971792	11.74232	10.45232	9.385591
Bcl9l	11.32365	10.39215	12.67546	11.82059	11.27059	8.593727	11.89038	10.03211	9.373723
Arid2	11.4818	11.37775	12.14019	10.44669	8.508359	7.546168	13.63755	9.131268	9.368997
Dock1	13.27585	12.11279	12.89502	11.13875	9.250408	8.568386	14.58724	8.658958	9.365749
Tab1	12.85711	12.99185	13.44223	14.15158	12.44218	11.22834	12.7437	12.00824	9.365406
Tatdn3	6.579894	8.668214	9.232566	8.691496	10.75614	7.537986	9.490458	11.45412	9.360035
Zfyve27	9.921348	8.208757	9.102376	9.181209	9.400774	8.547685	10.03448	8.000265	9.359437
Gm30408	8.724679	13.23346	7.634981	8.495061	11.59838	11.5982	19.72554	11.42178	9.354818
Ogfod2	12.2785	13.5564	12.52635	11.18885	12.94544	15.15236	11.7567	14.11442	9.354176
Snora41	5.887228	9.785924	2.453297	13.65992	11.68111	7.259099	8.112992	14.94725	9.351752
Donson	10.61986	13.73336	12.41117	7.332934	9.796039	7.216717	10.95149	8.611518	9.34833
Dus2	10.78457	8.266903	9.151612	9.660611	8.516817	9.149003	7.123868	7.778767	9.344234
Ccdc91	11.44116	11.11686	9.870988	9.228611	10.63922	9.399777	11.49513	8.906165	9.336635
Gdpd5	10.54528	10.70502	10.84784	11.21026	11.19101	10.86646	14.49318	9.74133	9.332693
Mmut	10.60862	11.07733	11.41363	10.34141	9.185028	8.829465	10.36647	8.462947	9.329654
Nip7	8.40667	9.301022	8.733228	7.159913	9.045978	8.730508	8.629337	9.480789	9.326394
Abl2	11.97552	9.934425	8.944038	8.765711	7.700008	5.643257	11.67222	8.458794	9.32604
Snord2	21.19691	13.82262	10.59968	24.32097	11.64675	8.293253	15.16712	11.64319	9.324247
Trim5	7.40322	9.401097	7.83455	11.98424	9.797257	9.353909	11.21048	11.14661	9.312181
D5Ertd579e	10.52799	11.26529	11.42771	10.38527	8.244211	7.269514	11.31645	8.50899	9.311655
Rdh14	7.401916	9.094579	8.242267	7.322514	10.68327	10.33816	10.05574	9.226942	9.309273
Wipf2	10.9868	9.777285	10.61548	8.775076	8.668739	8.187649	12.19347	10.18926	9.308684
Cox16	6.315729	7.444172	7.145897	8.627553	9.174441	7.138497	7.121041	7.1065	9.302787
Mir3061	7.310514	3.645526	6.092803	5.452173	0.580204	2.253511	3.148242	1.160054	9.290092
Map3k4	11.55224	10.17345	10.68759	9.639441	8.910393	9.579677	11.67788	7.852021	9.289318
Pi4k2b	8.734023	9.022525	8.532753	8.64853	6.496943	6.410549	8.171855	6.617504	9.288166
Xrcc4	12.94771	10.65797	11.19833	9.465976	9.413435	7.85875	12.25122	10.73012	9.281306
Gm5905	14.51759	10.73988	13.00353	11.18415	14.35818	13.20412	15.25199	14.58163	9.275054
Zbtb25	10.39257	11.91633	8.407704	8.260901	10.46516	8.57007	11.35698	8.224612	9.274146
D11Wsu47e	9.564736	7.833927	9.993371	9.936238	10.05082	8.202782	10.65881	9.793374	9.268826
Creb3l2	15.18782	13.531	17.74625	10.6334	11.39355	9.440739	14.66124	8.685723	9.268043
Rfx2	15.94817	13.17323	13.58204	8.459166	11.69365	8.539351	13.03975	12.52738	9.252186
Gm8355	12.49531	12.17284	10.15033	9.830158	9.289341	9.873177	9.536045	9.88887	9.245917
Nudt14	9.065914	8.363644	11.28837	8.699601	10.53382	10.22833	9.135823	9.408486	9.245465
Arfgap2	11.57498	9.770665	10.72004	8.497531	8.35808	8.374536	9.665954	8.9042	9.243014
Gm12070	8.347686	7.016216	7.305075	6.79471	7.469384	8.186595	9.706219	7.403006	9.239327
Swt1	6.086727	9.574189	7.872331	6.893276	6.58417	7.157629	9.319906	7.691109	9.238935
Ppp1ccb	21.29334	19.47006	9.309852	13.04173	21.06919	23.03721	20.21595	20.84746	9.231279
Ceacam10	1.067583	1.82527	2.059149	1.668233	3.268139	8.109696	0.394072	2.032887	9.230192
Rab13	8.730404	8.469693	10.04113	10.06265	8.654889	10.89937	7.874887	7.254284	9.228579
Mgat4a	12.33173	12.2989	14.49811	8.571421	9.199193	7.212412	13.06046	8.14728	9.228297
Trappc13	9.553952	11.09571	10.73373	10.53173	8.096385	8.210338	9.93945	8.782117	9.225578
Gm6252	6.820914	6.127304	7.208753	6.686497	8.246902	8.410356	5.299815	8.474672	9.221186
Cd101	6.433494	7.048462	7.092017	4.090567	9.065507	6.568772	7.032477	8.732561	9.220331
Tmem241	16.1826	12.67205	15.9316	10.43463	11.5591	9.996263	15.61485	11.4352	9.217927
Klhl25	8.573393	8.334402	11.22378	8.740898	8.646861	10.73453	12.54483	7.95635	9.204082

Dnajc30	9.271538	13.25065	11.32789	10.12255	11.15143	11.75614	12.94145	11.05702	9.200995
Gtpbp8	8.544398	9.975343	10.00313	8.496536	5.313368	6.321273	7.532376	7.27374	9.197444
Kptn	8.640135	8.315365	10.45679	7.95655	9.445475	7.212876	9.138493	7.778132	9.196078
Phkb	10.20002	9.56151	10.53225	9.984397	8.920419	7.981477	11.88534	8.730935	9.195717
Col6a3	14.03504	12.6496	14.15479	16.70986	9.442383	11.19521	8.456695	10.62022	9.192919
Kdm1b	9.448666	11.4139	9.502754	7.25316	8.981986	7.088259	11.82832	7.802187	9.190576
Dcaf4	10.78721	9.069816	11.47128	9.803466	10.19559	8.76344	12.87088	8.840414	9.187981
Rnf139	7.777649	9.206601	9.461781	9.05268	8.111653	8.348047	7.999054	8.873962	9.186999
Nat2	8.0234	8.68563	7.943686	9.702444	8.793368	9.917909	6.167349	8.905842	9.184101
Nadk2	9.980139	11.71952	10.91503	8.48362	11.37531	10.06294	12.0785	9.502071	9.174438
Erc1	13.11083	13.35814	11.36718	9.637351	7.074249	8.548199	11.38161	7.880326	9.16957
Fut11	10.16254	10.94566	12.02071	8.374636	9.094496	8.554785	11.82285	8.965443	9.164381
Cib2	5.942825	8.34014	8.278468	7.513552	10.22821	9.931584	9.871402	9.497611	9.143378
Cspp1	9.571898	12.59733	10.40201	9.282777	9.360521	7.738473	11.64726	8.15507	9.141679
Gm26728	7.273313	10.2625	5.631606	6.965933	4.564813	5.021352	8.348742	6.159018	9.136334
Pptc7	10.83272	9.916552	10.80994	7.938555	7.248214	7.460511	9.120958	9.12332	9.132814
Cryba4	3.949413	2.990646	6.509975	10.76369	7.592411	6.966412	7.634695	7.38119	9.131529
Ebag9	8.82102	8.535357	9.932103	8.945879	10.48739	9.102797	8.633946	8.871237	9.131015
Tmem161b	8.952453	9.933088	9.669773	10.17088	8.61148	7.16129	9.993031	10.99546	9.129756
Anxa9	10.25752	11.17636	11.47434	11.40874	9.147993	8.279546	12.31754	10.21778	9.12646
Rad52	8.744283	10.51346	10.57732	9.485784	9.381298	8.917457	7.358721	8.325414	9.124663
Lratd2	8.071552	7.176302	8.948686	8.240565	7.167479	6.481472	8.683765	8.2729	9.116505
Cdyl	10.00492	8.998449	8.983414	9.05168	8.153708	7.119955	8.972888	9.258408	9.115168
Spdl1	10.0937	9.73641	8.592794	12.58489	7.046826	11.25431	13.62825	8.663895	9.114978
Scn1b	10.17443	9.717067	10.1037	10.6537	10.47754	13.19919	6.874274	10.50857	9.114623
Tmem138	9.110451	11.14831	11.11475	12.46473	11.99968	12.84672	10.8185	8.551006	9.114143
Crispld2	10.91353	9.522248	4.540021	2.183796	3.507154	6.191713	2.435857	3.914062	9.112537
Snord14a	3.186096	1.906568	4.461052	3.801898	1.213761	5.303523	7.244575	1.820085	9.109896
Gm7336	8.152656	8.447155	8.108306	6.350475	7.462548	5.445065	8.519801	6.166579	9.108408
Sfi1	9.059088	10.96597	11.88477	13.31822	12.26498	9.051051	15.14606	10.68841	9.108145
Slc7a6	11.45492	10.04294	9.852996	6.422775	7.455839	6.678289	8.895507	9.1132	9.10785
Mfsd7a	10.28821	12.31298	12.27632	7.808165	9.888476	8.770572	11.4156	11.37165	9.106787
Apba1	9.012226	9.929857	11.23655	9.038558	10.31612	8.072291	13.10252	8.523319	9.104819
Rpa2	11.72848	10.67336	8.929615	10.2088	8.888654	9.321343	9.613975	8.767458	9.102739
Zfp52	8.200067	8.999555	8.025785	7.71855	8.39371	7.421132	7.225633	8.924975	9.101259
Atad2	14.2424	15.42062	14.7904	11.2816	9.086228	8.795654	15.30501	8.581911	9.092904
Gm47754	4.423985	8.41077	11.47604	10.44811	11.85006	9.332965	9.71637	8.555762	9.091722
Hdhd5	6.711841	10.57706	9.415105	8.350029	8.772095	7.792213	9.849971	7.524545	9.083159
Nup210	13.80632	10.57867	11.19924	10.14002	9.606569	10.7053	10.66956	7.968015	9.080372
Iffo2	9.797244	8.828239	10.31792	10.34158	8.796469	8.214695	10.00782	8.259626	9.08019
Thy1	8.17991	7.64825	8.883904	15.53738	9.768503	15.98597	7.562676	9.126652	9.075242
Alg10b	14.36897	15.20599	14.47478	12.80935	9.434899	7.194216	14.32834	10.25342	9.073232
Bcl2	10.40442	10.6888	11.05761	10.86474	8.170621	6.052734	10.07971	8.019162	9.071331
Rpap1	11.66561	10.20513	11.49072	12.89697	10.41651	8.430492	13.854	8.769115	9.069356
Nol10	11.9565	10.38779	10.47244	8.270975	8.2752	7.451455	8.826886	9.315593	9.059862
LOC115489083	6.335779	9.478367	3.168257	18.90087	6.034126	6.591521	11.4596	11.31053	9.05784
Senp1	12.05098	10.46779	11.22578	9.173192	8.811053	7.99666	9.754806	8.520671	9.054694
Ubxn2a	10.90833	11.39016	10.65345	8.21935	8.005164	8.505089	9.108482	7.969466	9.054036
Btbd6	11.81375	11.67089	10.4744	11.19214	9.070188	7.903184	12.06578	8.987645	9.050235
Col5a1	9.92369	9.963077	12.47204	13.52927	7.452886	10.22053	6.826387	8.807548	9.043106
Gm6166	6.704898	4.485215	6.378571	10.48887	8.176775	11.87165	6.929847	6.461571	9.040027
H2-Q5	4.834988	4.073049	8.121846	12.46321	6.795417	10.11456	11.88655	13.85484	9.037387
Mrrf	9.654252	9.775925	11.55197	9.208329	9.288426	10.59055	9.876333	10.88008	9.037049
Aatk	7.425239	5.835288	7.615942	10.87006	9.528207	9.086666	9.139984	8.281479	9.035036

Ptgs1	7.425259	8.967586	12.2415	10.72939	13.92248	9.799277	13.90981	9.407688	9.030459
Zfp944	8.133597	10.6525	8.91671	7.976937	8.959376	8.513568	7.898395	11.12666	9.02985
Tamm41	12.00899	11.87377	9.52548	12.40498	9.120207	12.30226	9.897414	13.40507	9.028239
Zfp280d	10.38825	11.60468	11.30978	8.093233	10.88691	8.377518	11.3497	9.968508	9.023871
Kif11	12.4976	13.46878	14.25029	9.093083	7.989549	9.292478	12.15986	6.930151	9.020753
Sparcl1	10.57354	8.739274	7.536714	32.70474	14.66937	20.79868	5.695587	11.97663	9.019661
Rn7s2	3.708232	4.253113	7.231892	4.609325	7.59311	8.916067	3.066114	11.82751	9.012399
Rft1	10.35374	10.11638	11.87429	10.20278	11.10729	9.50997	11.11175	8.678399	8.999287
Iqcc	9.326377	9.523895	9.290299	11.08462	7.537602	7.456449	8.525574	8.030577	8.995107
Me2	10.74126	10.43574	9.427101	8.494013	8.838042	9.054738	9.591204	9.308415	8.990784
Haus1	11.54768	10.92044	11.82509	9.607286	8.587989	7.913472	8.321287	7.884518	8.988935
Oas1c	5.777752	8.130952	8.898063	9.023431	6.920533	7.506542	8.352906	5.872218	8.986412
Rbsn	10.91423	9.843211	11.07112	8.454803	7.081449	7.585271	10.4763	8.275038	8.982781
Gm22107	9.978851	16.58714	3.881115	5.512752	16.89555	2.050695	11.4596	8.97302	8.982358
Iqce	12.44397	12.87287	12.29097	10.55715	11.60638	11.01987	14.08509	10.38986	8.977942
N6amt1	10.859	9.153607	9.683455	8.357721	10.72622	9.544625	9.6597	10.27482	8.971784
Spint1	13.18329	11.03379	15.15889	8.723476	13.59999	12.21403	8.437289	8.909217	8.964939
Slc7a5	17.75344	13.07896	14.31488	7.453907	9.348344	7.720025	10.76478	11.31375	8.964159
Nsun4	12.93261	12.19384	12.97825	10.6656	13.32576	10.08204	12.08915	11.55667	8.960482
Tcf19	11.66395	14.74641	11.73392	10.23407	10.74922	12.3727	11.73616	8.459567	8.958816
Rbm4b	9.153797	12.87793	9.852086	12.1166	12.90376	8.549236	14.06605	12.75552	8.955491
Usp40	11.39397	12.11703	11.96191	11.5388	9.512542	8.518691	12.20998	9.909199	8.93956
Gm5869	9.462993	7.976193	6.197724	8.785429	7.536961	6.931165	9.347711	10.5246	8.939258
Ippk	12.16933	7.998285	9.466135	7.696526	7.371378	7.028245	10.55363	7.813048	8.932142
Utp15	10.10441	7.64641	8.38954	9.167609	7.275858	7.896008	10.45748	8.129356	8.929533
Anln	20.08047	19.31794	23.84617	13.88103	10.37145	9.156608	13.5852	7.90829	8.924678
Prpf4	11.16203	9.159743	11.5746	8.139635	9.548882	8.674347	10.70669	9.974787	8.921327
Ngrn	7.008446	8.831408	11.58417	9.055771	8.992949	9.585953	10.10631	9.78933	8.919561
Ksr1	10.72016	10.79248	9.119939	8.849904	9.760277	10.80216	13.81493	8.489488	8.91601
Kifap3	11.75386	12.34642	13.18098	10.03897	8.949855	9.40328	10.78002	9.224121	8.914838
Spout1	9.32011	10.16767	10.37699	9.387876	8.244396	8.674732	11.4409	8.615202	8.91164
Ppm1d	9.04173	10.50786	10.28808	9.429207	8.921395	10.57589	11.50316	11.16201	8.909693
Gm32026	10.09334	11.11819	12.95714	8.538774	9.469315	11.00577	12.14469	7.028101	8.901953
Fastkd2	11.76338	13.15489	12.59078	11.79704	7.913831	8.309251	11.40786	10.00841	8.897675
Gdf3	7.715607	4.227536	6.906747	11.27178	8.028654	13.03707	8.712841	7.346014	8.897019
Pdk2	13.13071	16.36969	17.10131	11.93658	17.05514	13.32091	15.09042	10.72909	8.895735
Nkapd1	12.2128	11.22189	11.51526	10.09426	10.59966	8.514942	11.25013	10.92912	8.892269
Ankrd50	13.63675	11.29433	12.23953	9.511321	8.368283	9.523321	11.26142	8.559029	8.890443
Mtx3	12.01273	22.99753	17.745	17.4376	12.09831	9.895946	18.90906	12.75193	8.883
Rabgap1l	7.823456	9.753285	11.12835	7.975758	8.875425	8.648426	10.93979	8.168529	8.881946
Itgb2l	0.180462	0.107989	0	1.1126	1.959323	5.573993	0.559551	0.343636	8.875032
Pik3c2a	10.36891	10.29955	10.78185	8.145968	7.31671	5.943016	10.12541	7.998753	8.871629
B230307C23Rik	7.271288	10.19665	9.784325	9.596922	6.16963	7.865346	8.562818	9.901958	8.862235
Procr	7.372083	10.69782	9.916604	5.681361	6.17856	7.328782	6.400309	7.089135	8.853075
Zyg11b	12.70753	12.0025	11.8459	7.928223	8.498147	7.351107	11.3745	8.148793	8.851509
Cd300ld3	5.682401	6.012839	4.643477	7.648944	8.249782	6.088002	7.806853	5.938028	8.850264
Trim13	8.607304	9.460347	6.781236	10.96961	8.766308	6.302834	11.98247	7.559466	8.839717
Pld1	10.39745	12.46614	12.14113	9.400294	8.596366	7.286788	12.81421	10.19905	8.838373
Zfp830	7.847243	7.89382	8.829174	7.937825	7.763553	7.303804	8.320272	7.27812	8.833098
Airn	13.69173	16.46665	11.94809	12.14674	8.948916	8.813521	10.82141	9.073984	8.827556
Mcat	9.255802	7.655109	7.54742	9.941555	7.340818	9.245427	7.166417	8.044798	8.821602
S100pbp	11.15434	13.74305	11.85195	10.39951	9.587188	9.362803	12.24816	10.08142	8.820058
Cox6a2	13.3758	12.58508	10.93746	16.56335	12.17813	17.58183	12.43341	11.69384	8.819596
Flrt3	15.09797	12.26827	10.20101	2.781283	7.660038	3.783286	9.77978	7.578206	8.819495

Rai1	11.79566	10.35969	12.03049	13.15323	9.966894	8.51938	14.62808	8.000711	8.817357
Psph	9.071683	8.160519	10.37731	6.190792	8.13155	7.895726	6.924786	6.040347	8.815652
Rtl8c	10.87819	11.25535	8.785016	13.42034	12.85339	15.38559	9.198945	9.747864	8.815474
Gpatch1	9.557036	9.839063	9.739516	9.575635	7.340336	7.865359	10.79609	8.046003	8.814875
Gorasp1	9.056634	9.63575	10.28342	9.164179	10.06554	9.817785	10.11722	9.304748	8.813818
Fbxl8	9.536163	10.2379	9.18728	10.70071	11.08187	10.84139	12.14682	11.52274	8.810866
Rpl36a-ps1	5.659302	5.068293	6.584035	5.8573	5.27986	4.592703	4.416721	7.477516	8.806233
Dipk2a	9.571838	9.882297	10.23267	9.280963	8.568077	8.488321	9.704396	8.284843	8.801774
Ceacam16	5.603516	9.482293	7.395663	11.37262	7.173961	5.606707	14.81108	12.28821	8.798937
Ercc2	10.16154	7.439311	9.688564	8.674571	9.22621	8.797881	10.07778	8.81714	8.796192
Naip6	9.243811	9.837663	10.97313	12.51366	8.811579	7.256189	15.58711	9.580353	8.794413
Yipf6	10.46879	10.56853	10.91207	8.351253	8.663136	7.723454	8.115013	8.605115	8.791451
Tmem167	8.221595	8.220167	8.41747	7.47682	7.410073	7.574615	6.885938	8.267251	8.789124
Pkp4	12.4355	11.22121	11.15377	12.7584	7.224224	8.709718	10.41914	8.913418	8.788316
Oas1b	5.905359	5.07831	6.086843	7.729838	8.235434	5.795443	6.726288	5.335619	8.787089
Pcgf6	7.215521	10.47384	10.43157	11.3665	6.123549	5.813827	10.11576	7.073939	8.783537
Rfx7	9.286255	9.428765	11.19591	9.194537	8.53234	6.660403	11.70223	8.674175	8.782134
Psrc1	8.989956	8.902434	13.61403	8.495789	10.14073	9.669754	9.159773	10.53221	8.780913
Taf2	10.17244	10.62574	10.82739	9.73912	7.591028	7.689047	10.33592	7.643303	8.777085
Neurl2	10.31071	11.3355	11.75078	9.63301	12.78859	11.92987	17.14975	9.953787	8.775762
Pcm1	11.18551	11.75607	11.33897	9.866518	9.241803	8.900375	11.82741	8.955575	8.775762
Cstf2t	9.471162	11.18483	11.1421	8.903859	9.808269	9.578475	11.78946	8.834592	8.773374
Adck5	8.411292	9.85968	9.69596	11.56863	9.727428	8.965479	10.16122	9.41244	8.771529
Hacd1	11.0751	9.610308	11.20154	8.773116	5.899618	9.026763	9.312543	7.565884	8.766476
Lrrc58	9.504221	9.240819	9.589215	8.360238	7.527734	6.277152	9.062848	9.153384	8.763186
Stx17	8.982175	11.0447	9.789406	8.311593	9.285681	7.729599	10.54315	8.451408	8.762027
Rbms2	11.33888	11.83962	11.95778	10.95775	10.51511	10.24954	11.76331	10.17738	8.757187
Gmeb1	9.296612	9.186687	9.895575	8.819919	8.040633	8.913124	11.42429	9.11845	8.755845
Prim1	12.22313	10.60365	11.32037	9.205901	8.541457	10.83538	8.559193	8.194539	8.754533
Asap2	11.18279	12.3395	13.70645	14.95305	9.2001	9.37335	10.72186	7.475629	8.75332
Gm12350	7.89939	5.536145	4.341611	10.19045	6.845519	8.029038	6.619795	8.740614	8.74971
Ndc1	15.74231	15.84115	14.4225	7.705861	6.915994	7.332387	10.09113	7.402494	8.743055
Fcrlb	7.685339	10.89446	13.55886	3.263	5.715737	3.713292	5.399578	6.741686	8.723932
Poll	9.784571	10.57955	11.62053	11.01615	9.65473	11.20183	10.93937	8.890386	8.7203
Ccdc127	11.15226	11.07421	13.31961	10.12546	8.897257	9.745287	11.84381	10.49494	8.718941
Cyb5rl	12.91701	10.84852	13.28937	10.87801	11.18365	8.344524	13.09486	8.8265	8.717876
Trmt61b	7.397544	7.377849	8.268804	7.442649	7.542658	7.88729	7.375881	8.368963	8.709461
Gm35853	5.877226	4.959798	6.612648	8.064016	11.70611	8.831996	8.731959	7.882397	8.698512
Gm41236	6.795634	11.23645	5.186744	6.046245	8.00495	4.134466	5.360136	8.683568	8.692604
Gstk1	8.464737	10.16631	11.62546	8.832259	7.266905	8.544564	7.393291	8.229525	8.692604
Raet1d	8.898005	11.83242	17.4491	4.293961	4.237181	5.60674	4.823672	5.938547	8.688447
Jmjd8	11.36851	10.99466	10.2567	11.12037	10.12057	8.650758	12.59899	7.140267	8.688368
Pogz	10.15785	9.576171	10.35022	11.13783	8.383227	7.690108	14.14488	8.438577	8.687528
Eme2	7.872861	5.380006	6.707291	9.749707	8.157575	6.505329	7.421241	9.681991	8.684648
Fam214a	7.334882	10.50519	9.198101	11.91603	11.87292	9.530475	15.70553	9.812126	8.684301
Hook2	9.76578	7.804942	9.503833	10.52851	8.893715	8.453341	11.8946	8.734466	8.680877
Zmym5	7.952226	10.11372	9.740983	10.06236	8.925306	6.883075	10.69369	9.324868	8.673718
Pimreg	13.03557	19.72207	16.38245	10.27221	9.978796	13.05567	10.57496	6.837835	8.673397
Arl16	8.138954	7.188035	9.181369	7.384854	7.77755	8.337602	6.146153	8.401362	8.671288
Klhdc10	11.06866	12.60812	11.26898	9.169074	7.761846	8.272891	11.62611	8.165493	8.670753
Ado	9.840412	7.624985	7.434179	8.793119	6.924991	6.75876	9.706501	8.110332	8.665571
Trit1	7.385761	6.680186	8.642644	7.671468	10.53694	8.876392	9.858718	7.829115	8.663737
Fbxl18	8.067991	9.745654	10.57578	12.28734	8.11282	9.652249	11.1722	8.576452	8.660033
Ndufaf6	6.432407	7.341199	7.759578	6.989854	5.936685	7.481604	6.648213	6.692514	8.65876

Mplkip	7.346216	8.688797	8.257692	8.9307	7.745372	10.73572	8.298479	8.846334	8.658312
Gdpd3	8.305328	8.697378	10.90201	8.465275	12.95247	13.53689	12.76866	11.96007	8.657813
Nfrkb	10.16219	8.596861	9.200637	9.517874	8.480281	7.617273	11.00058	8.217318	8.653323
Fuz	7.215437	8.140529	8.19586	8.571064	9.950267	6.823574	8.773546	8.361887	8.650646
Ctse	8.68181	8.789099	11.40274	8.529164	9.239756	9.358716	11.62177	12.84705	8.648386
Gcc1	9.833625	8.925576	9.419403	7.913424	6.673192	6.145148	8.695782	6.428798	8.644495
Pds5b	10.47512	10.51772	9.314049	9.120248	7.336463	7.820818	11.69496	7.835131	8.644079
Ccdc167	9.752243	8.798046	9.10585	8.409965	9.943095	8.195297	11.20866	6.39554	8.639107
Trpv4	13.17836	11.36865	13.15208	15.89685	13.37176	12.28977	17.70089	11.25002	8.638411
Dennd3	6.063538	5.442656	5.11129	5.185433	5.741848	5.479202	5.550744	4.766917	8.635612
Oxnad1	12.47909	13.21685	14.9656	9.938329	9.676238	6.68927	9.644784	9.88357	8.63081
Slc36a4	11.87311	12.11268	11.5522	10.0942	8.881291	6.556265	13.5725	9.636037	8.628669
Nrg1	13.56876	9.537284	11.95482	3.726569	9.538209	11.17366	6.077061	10.27351	8.621487
Ifrd2	10.07965	9.66187	8.288674	9.744765	6.959816	9.217772	12.70395	7.037663	8.619434
Arrdc2	7.127068	8.324579	11.00279	12.10522	11.00161	9.510343	11.96386	8.51624	8.61628
Zcchc4	9.103169	8.402098	10.6489	9.773559	7.599968	8.050429	12.14166	9.290962	8.609217
Elmod3	10.94172	10.28543	9.056271	8.954471	10.06144	10.21161	10.76287	8.693301	8.606455
Zfp942	5.702517	6.092259	8.250231	8.937119	8.102582	7.372673	6.160871	8.20553	8.601028
Dock5	11.45274	9.683487	10.85094	7.267107	6.803459	6.367863	8.233188	7.349458	8.600708
Wee1	11.87716	13.26498	9.065651	9.687753	6.519143	6.831473	16.54848	8.904622	8.590922
1190007I07Rik	10.49363	9.478367	7.524612	10.92706	9.522605	9.612635	13.50596	11.49904	8.586077
1500002F19Rik	2.369148	3.307977	1.421654	9.894684	7.220322	9.201838	1.958906	9.022644	8.580432
Map6	12.46466	13.97061	13.51089	9.845044	5.58512	9.616751	11.06698	9.900951	8.576177
Gm4202	9.074456	9.962932	9.151775	3.943097	6.754154	7.686582	8.748389	6.897297	8.576084
Slc26a6	10.47256	9.470669	10.03737	8.843546	9.835422	9.896066	8.225313	8.863023	8.575185
Ift140	11.80561	12.68651	11.25461	9.439645	10.55315	8.299826	11.63085	8.019254	8.57045
Ahnak2	13.50351	14.1336	14.80157	12.01184	7.655564	7.052103	11.98234	8.462026	8.569112
Zbtb11	7.315139	7.397781	6.417136	5.893996	6.533462	6.396587	7.725177	7.5713	8.565882
Tbc1d31	11.72595	12.14453	12.07427	9.866378	10.73814	7.844102	12.76476	9.892584	8.563759
Zfp715	9.315924	9.6506	9.608163	8.340123	7.948177	7.44845	10.48816	9.344833	8.561799
Dnajc16	11.47581	10.89419	11.633	10.79164	10.55162	8.267242	12.4319	9.172522	8.560928
Cdkn1c	5.103451	8.580057	10.26642	12.64366	10.02646	10.62564	5.696645	9.551378	8.560715
Alkbh2	5.433907	8.585855	8.609775	9.956954	9.303719	9.824367	8.140355	10.81227	8.554072
Mtr	10.85034	11.0433	12.08755	10.26114	9.709953	7.428399	14.01157	9.212812	8.551037
Zfp180	12.16425	9.610575	11.60051	8.739369	7.463751	6.435927	9.652132	7.90038	8.546393
Zfp53	6.571904	7.308614	9.363801	6.714135	8.865501	8.625042	8.727757	7.251375	8.545964
Usp34	12.19943	11.22157	11.65278	8.871265	7.62192	5.921613	11.75658	8.592604	8.545081
Ldb3	8.702702	12.77923	11.11112	13.77267	11.99167	13.49322	7.387118	9.167296	8.541517
Mga	11.2226	11.1649	10.79436	10.80475	9.105223	6.685032	13.81286	8.560755	8.537767
Tvp23b	10.3632	9.967671	12.47753	9.304646	10.60444	10.70009	11.40414	13.02798	8.534534
Ssh1	10.4223	10.37449	10.68511	10.39727	9.676976	8.776113	13.70411	9.450527	8.52711
Tpd52-ps	7.867275	6.878616	8.34028	7.275582	6.318849	6.135595	8.266741	8.813724	8.522967
Pbx1	5.987753	7.200839	6.24126	6.441233	5.762857	5.347353	4.229177	6.01681	8.522647
Clock	11.15735	12.7142	12.09579	10.03304	8.945047	6.166596	12.81547	9.447177	8.522384
Mrpl19	7.405004	7.517564	8.058023	8.132849	7.126127	8.084629	7.916172	8.754987	8.521821
Crybg3	13.7361	13.21799	14.42675	9.55925	8.339069	6.426609	12.5597	8.69808	8.520994
Ahr	12.92869	11.09913	12.52902	7.762856	10.53033	6.753218	12.62895	8.294388	8.518683
Prmt6	10.97562	8.243307	8.759112	7.617622	8.383778	6.918508	9.213982	8.914373	8.518029
Mks1	10.17945	11.09791	12.02712	11.16444	9.409652	9.644359	11.91345	7.981507	8.512956
Dhx33	10.74244	11.2876	10.65517	8.426997	9.135181	8.45078	12.16625	7.878924	8.498692
Fchsd2	10.76757	11.04998	12.15222	10.18133	10.97818	8.515302	13.77787	8.947713	8.491725
Gm8566	7.424741	6.664476	11.0559	6.398731	7.385519	12.05394	6.821191	9.739622	8.491725
Cpq	5.852276	9.603233	10.49496	7.052195	9.60973	10.10418	8.670681	7.246267	8.490253
Gm9816	12.12708	9.830741	9.947748	9.385936	12.0902	10.74516	10.68163	8.446333	8.483591

Gm6794	14.1544	8.769475	12.11843	10.12982	10.2125	13.78369	11.30444	12.55753	8.4826
Tyw5	7.252331	9.584865	9.211086	7.875361	8.432522	7.570786	4.230671	9.997322	8.481123
Polr3b	10.13036	8.400947	9.573634	8.098831	8.978632	8.068242	10.60861	8.710329	8.474825
Mthfd1	12.09164	9.198445	10.10811	8.292347	7.259014	6.740026	9.670331	6.558111	8.472423
Gm33695	6.549645	11.66466	8.655893	6.512957	7.351704	10.59959	14.26406	9.576566	8.471819
Net1	11.58486	11.09678	11.5333	8.346279	10.0089	10.76224	10.78894	8.159122	8.470117
Nhej1	7.141843	7.240887	6.905899	7.376494	7.140025	7.151891	6.81051	6.649464	8.461505
Fabp7	7.493596	7.981564	8.949704	9.043229	6.928951	6.526163	6.767481	5.541467	8.459531
Prag1	12.06184	9.218934	11.71526	9.526221	7.952928	7.518501	10.8123	8.646166	8.456195
Cep104	9.947385	10.2246	10.79451	8.966424	7.121447	7.791015	10.05382	8.536683	8.448824
Parn	9.943871	9.897798	10.57396	8.168488	8.871513	7.269392	9.972818	8.700407	8.447241
Nkap	8.636995	8.211841	8.911189	5.924075	7.209945	6.844473	9.020873	7.913655	8.442578
Slc25a26	9.094689	8.991603	10.12396	7.864126	12.80423	8.995561	9.726765	9.788475	8.441924
Prg4	45.84528	38.00486	31.31261	9.714617	8.51952	11.3474	9.945036	11.93489	8.441644
Arhgap21	12.25834	12.31332	11.61284	9.021156	7.134124	6.668748	12.32295	7.367648	8.440892
Tinagl1	9.930052	7.72482	6.441097	17.29436	15.13164	17.26404	5.160926	7.806619	8.436678
Gm7887	9.812858	9.283677	10.13502	8.43556	8.03552	8.94741	7.488077	8.687933	8.434754
Lpin1	9.373077	11.85846	11.16474	10.57728	11.75531	8.307576	18.14509	10.96961	8.4315
Mob1b	8.286863	8.090438	6.801612	9.492157	6.826688	5.20596	8.402262	8.605656	8.431322
Rhoh	7.679489	6.742195	8.128871	8.773582	9.855028	9.424075	10.59057	8.368343	8.430477
Cenpc1	11.52868	11.10459	10.31067	10.53699	8.384254	8.273747	9.802925	7.050181	8.424592
Wrnip1	10.23988	9.291813	9.254742	8.951441	7.733936	9.624763	10.86537	9.429722	8.419585
Usp37	11.79	11.83009	9.228804	10.53018	7.747436	7.98671	11.4633	9.399851	8.419146
Scrn3	8.196367	12.71097	12.11582	9.941763	9.39309	10.28679	12.38121	8.44691	8.412778
Kbtbd4	9.133202	11.42151	12.32889	8.846756	8.270204	9.365105	9.68417	10.43034	8.411588
Stac2	18.03616	28.42357	21.85404	8.047814	11.2314	9.640478	11.25248	10.89774	8.411376
Pmepa1	14.69457	15.07034	18.45295	8.71139	14.89221	14.24534	18.98131	10.7988	8.407805
Borcs5	11.74506	9.733035	10.75329	9.091445	8.870426	12.03311	11.25433	9.519754	8.387407
Abhd8	9.886687	10.19462	11.20063	8.998145	8.006237	10.02103	8.919438	8.296287	8.384776
Brms1l	9.640407	9.20366	7.809408	7.013332	6.629812	7.009295	8.023141	6.21355	8.380653
Ptbp2	6.944191	7.94564	6.450105	7.856615	7.776051	5.979415	8.336425	8.527486	8.379153
Rpl27-ps3	6.986097	6.405603	3.718839	5.938534	6.546168	8.440362	5.590049	10.29902	8.376661
Adam33	10.55403	13.06937	10.86916	11.08013	13.48924	12.04584	19.30209	10.49673	8.376205
Cfl2	7.316316	8.989403	8.505263	8.587893	8.584263	8.841008	8.16594	10.17948	8.374907
Gm26740	7.693668	6.149432	15.38912	4.449269	8.210807	6.862563	4.511654	7.844639	8.372855
Cntln	10.12942	10.51202	9.605818	9.823135	9.820975	8.333587	11.10591	7.015941	8.371357
Kif22	14.31613	17.6533	16.33249	9.962484	10.92297	14.38169	11.82604	7.671871	8.371041
Gm42059	6.33796	11.11902	8.324313	10.40285	4.290796	6.214228	9.549667	8.215453	8.36956
S100a16	5.515665	6.511365	6.923527	11.24937	8.147602	7.807216	6.398762	6.912628	8.368157
R3hcc1	8.805451	9.966099	9.30122	9.707167	11.40148	9.911898	7.737446	9.576831	8.360945
Gm12164	9.239677	7.029451	12.67641	9.364612	8.23568	9.640021	9.733315	5.864719	8.354631
Cnnm2	10.49655	10.97678	11.0923	8.05632	8.508599	7.4922	8.90015	6.61398	8.352971
Otud1	5.545368	6.605568	5.202316	7.735561	5.430118	7.280575	9.880636	6.796728	8.35215
Tefm	6.954835	9.270658	6.487475	10.50681	6.49633	7.421093	9.054329	4.902594	8.349456
Atg10	11.70146	13.20084	14.13291	12.27176	12.4232	10.40722	11.56534	10.41038	8.349162
Arntl	12.38117	13.66135	12.12849	10.47423	8.31578	9.035877	14.34838	9.742764	8.343906
1300002E11Rik	11.20056	6.195537	10.63706	11.13782	8.605545	6.354022	6.90689	6.272959	8.342747
Gm48909	11.27329	6.481419	8.489111	9.099998	8.841872	8.708096	6.442599	3.914489	8.342747
Trim37	12.422	11.40717	11.43895	9.105053	6.503098	7.065882	9.205147	8.033408	8.339612
Mapre3	10.71329	14.28927	14.74863	10.10778	13.39941	10.14605	12.44014	16.12968	8.334654
Slc49a4	8.363368	9.424628	10.05566	8.418439	8.521572	9.099037	13.00831	9.650927	8.332465
Hspbap1	6.450774	5.113887	6.865083	8.744744	6.747509	7.214654	7.94935	9.396382	8.328919
2310022B05Rik	10.14213	10.58327	12.10491	7.801537	9.116421	7.704835	9.460148	10.6778	8.324247
Prepl	9.011753	11.83797	10.53831	10.14681	7.781198	7.49068	9.38658	6.56025	8.32164

Flt1	16.67005	14.37167	15.99584	20.47594	11.53475	11.61108	8.824292	11.31053	8.32143
Fbn1	9.943242	9.258039	9.427433	15.36317	8.061325	9.427569	7.101035	9.275552	8.320419
Ltbp4	10.04796	7.315634	6.729732	25.06208	15.87711	18.41589	6.892051	10.21596	8.31994
C5ar2	7.560889	4.428299	7.206517	5.550597	4.180393	5.6312	5.611795	7.150394	8.318565
Ahctf1	13.05127	13.29826	11.69665	9.332539	8.310504	6.594921	11.75028	8.899683	8.31661
Phf7	7.282396	8.048199	7.31605	7.404703	8.341555	6.734532	6.780829	7.964219	8.316419
Gatb	10.42918	9.155163	9.036469	7.288208	9.886865	8.06678	9.835288	7.618796	8.313817
Cep164	9.277701	10.6182	11.38298	8.797214	8.920477	7.173214	12.3792	8.507462	8.310958
Gm11964	5.59763	7.51521	8.828057	7.278974	8.099203	11.24896	6.230463	9.429101	8.310348
Champ1	9.932711	9.809822	11.74379	10.64636	9.341393	7.537443	11.55098	8.786117	8.308098
Arl5b	8.539438	7.819664	6.009635	5.440059	6.495583	4.977416	8.626667	8.023302	8.307286
Zfp629	11.77714	11.88333	13.93009	11.87573	11.87312	9.284243	14.82336	9.374797	8.306775
Vps13b	11.16073	11.71602	11.96965	9.428289	8.697096	6.241699	13.5449	7.914119	8.305263
Ap1s3	9.605789	9.283155	6.106715	7.404637	5.886211	6.541842	7.195921	5.459033	8.303527
Snord4a	7.127751	8.688503	7.128579	8.662897	1.508532	7.323912	13.09669	4.524212	8.30302
Frs2	9.802121	9.241018	9.354553	9.194387	8.007107	7.459206	12.09669	7.828223	8.301161
Ear1	0	0	0.388811	0	0.592411	2.804247	0.482171	0.296115	8.299844
Adgrg6	14.4634	14.18696	16.29931	10.21594	9.258927	7.073741	13.762	8.56047	8.299365
Htr2b	12.11543	9.825876	10.3326	11.06787	7.303553	10.19438	11.03578	5.704157	8.298669
Mlh1	9.903541	10.72379	9.295172	8.982302	8.644294	6.984602	10.2583	8.620929	8.297982
Cep128	12.81228	10.91775	11.83187	10.13709	8.353026	8.791721	11.80461	7.851938	8.296782
Hip1r	2.876503	2.682557	2.783434	5.850799	6.137304	7.272092	4.671872	3.09439	8.295899
Fasn	15.83945	11.79752	12.15173	7.709688	8.817713	7.962209	8.800413	9.704451	8.279768
Nup37	11.0969	10.0486	9.240751	7.387273	6.456111	7.471126	8.301972	7.515092	8.275204
Rilp	10.01534	11.28832	10.4261	11.55123	12.6589	10.26036	12.50628	10.35089	8.268042
2810006K23Rik	9.013768	13.1129	12.06494	10.41223	10.19826	9.40111	9.526319	9.872516	8.267955
Mmachc	9.852464	11.68835	9.853609	6.426784	6.893911	8.181531	9.292371	8.040438	8.267821
Nhlrc3	14.21489	12.15682	14.03883	14.59466	9.375137	9.497612	17.88726	9.609116	8.264311
Ltbp3	11.27972	16.7386	13.95524	9.861257	13.4129	11.13934	9.887293	10.40572	8.263962
Tprn	9.377877	8.939414	8.687886	6.164545	9.871534	8.508049	8.284541	8.214366	8.260548
Mrpl17	8.589926	8.86361	9.053513	7.802615	8.778791	8.860275	7.717133	9.161439	8.257812
Fggy	10.01404	9.402529	9.473837	8.881408	8.205479	8.092655	11.98176	10.00677	8.2545
Ncaph	12.54813	13.99187	15.03894	10.94368	6.954918	8.845764	13.96838	6.365233	8.254005
Slc37a4	12.46485	9.765665	11.1877	10.14138	10.59846	9.404394	12.70286	11.89542	8.252382
Bcl2l13	10.68734	10.57808	10.29151	9.644497	8.442221	8.234852	10.96216	8.431926	8.247638
Fam102a	9.333893	8.496141	8.467219	7.725959	7.712578	8.266972	9.878029	8.536316	8.244489
Wdr4	8.812147	8.231869	8.586806	7.20229	6.840538	7.032869	8.390399	8.332562	8.240563
LOC100861749	7.33263	7.285035	7.882438	9.992651	8.524984	7.145416	11.75344	7.276182	8.236142
Bc1	6.479774	11.48893	2.880234	5.011593	2.742785	2.663241	5.580974	10.2823	8.2344
Mex3d	9.918977	8.327745	9.92013	7.693464	9.206263	6.50432	9.562094	8.94589	8.233284
Mgst2	0.533058	3.189835	2.985473	1.060145	1.523037	4.732374	1.322262	0.203009	8.230441
Clpb	9.907794	10.75055	9.305559	7.505876	8.133666	8.13335	10.03235	8.475831	8.229089
Bri3bp	8.869455	8.932877	9.822596	7.675885	9.41429	9.221967	7.674718	9.411413	8.226544
Scnn1a	5.725395	4.5596	2.820299	3.271537	5.493496	5.689791	3.024981	3.742572	8.225938
Shfl	7.806786	8.870129	10.17965	9.957675	8.188019	8.194252	10.82636	7.620998	8.22543
Siah1a	7.552584	9.20636	7.579689	10.61348	7.093095	6.790382	9.486425	9.213212	8.223039
Timm22	7.17903	6.489983	5.689308	7.201437	8.436581	6.871887	7.051061	5.755907	8.222708
Mapk13	3.195277	2.270574	1.597824	3.574551	4.907075	5.57736	2.353016	3.99291	8.222535
Zfp729a	6.622331	8.622213	8.092534	6.542887	5.5871	6.104432	7.603787	6.406476	8.219376
Snord45b	13.55153	8.60074	19.7136	13.47562	4.693209	3.417826	6.366445	16.42121	8.219151
Zfp426	9.721136	10.81207	12.14066	12.1358	8.840984	7.218856	9.879545	8.065705	8.213308
Slc37a3	7.384197	7.928871	8.644193	8.130571	8.231678	6.239996	7.354146	6.936201	8.210762
Mrpl35	8.762065	7.197488	6.73737	6.713772	5.872862	7.811512	6.636954	8.570902	8.208029
Angpt2	7.023193	6.057102	6.650226	6.24056	5.027732	7.27248	6.953016	5.485818	8.207607

AU022252	8.997179	8.469778	9.56247	8.651508	7.607298	8.677286	10.43456	8.990265	8.207202
Tdp2	8.075955	8.585824	9.198684	9.257632	7.39768	7.727709	7.303539	8.810427	8.204897
Zbtb40	9.626313	12.0965	11.27158	10.58957	11.09901	7.077771	15.36757	9.060846	8.199807
Mospd1	9.294824	10.71009	9.205653	9.452142	10.17011	9.374449	10.53935	8.334074	8.199401
Tfb2m	7.336631	9.223667	8.913492	7.064338	6.962724	4.801838	7.155576	6.296511	8.198907
Arhgap5	10.26196	12.07518	10.80725	9.228583	7.162404	6.79685	8.136603	6.835213	8.194524
Mccc1	7.541207	6.338446	7.912702	9.488862	6.758645	6.031457	9.077291	6.509603	8.194035
Slc41a2	11.42636	11.87497	10.10036	7.528816	7.403361	6.393812	10.59477	6.871589	8.191845
Asb13	7.850144	8.343575	7.765096	12.02264	8.81341	8.292864	9.446028	8.565217	8.191845
Itm2a	5.197318	9.926537	6.353016	11.24845	7.829205	10.39798	4.599288	6.46844	8.191092
Vps37a	9.973035	10.7044	10.01173	7.659451	6.755076	6.732694	8.872512	7.896379	8.190736
Snhg20	5.7286	10.96594	9.333159	10.65799	10.82371	9.313575	5.920794	8.005341	8.189797
Mtmr1	9.785454	9.644134	10.1462	7.368214	9.136103	6.769147	9.290851	8.644245	8.188144
Sec22a	8.086806	11.36471	9.558245	6.911709	7.860913	7.66384	8.32358	7.222194	8.185179
Sgcb	9.029291	9.034875	9.728409	8.225564	6.619273	6.289971	8.764308	7.154548	8.181092
Cdc25a	8.320943	7.3941	7.432146	6.799871	7.226996	7.372308	7.247891	6.842787	8.179987
Pde7b	7.690763	6.738814	8.268243	8.140712	6.577863	5.747321	7.877284	5.895213	8.17617
Insl6	9.239677	9.382625	9.408765	8.102076	5.119865	6.991007	9.115592	8.237264	8.16578
Pald1	10.81652	12.43698	15.0744	7.642373	9.191346	7.583562	10.56301	7.923823	8.164605
Cep44	5.825157	6.560556	6.023992	5.674313	5.094933	5.423569	7.576934	7.658929	8.157883
Ppfibp1	10.751	10.8594	12.10344	8.447878	6.106406	6.130487	8.105975	7.194636	8.151495
1700025G04Rik	12.2303	12.76756	10.82105	9.995748	11.10386	12.74667	8.968383	8.482593	8.151083
Gm36043	4.275521	3.690809	3.593293	6.359494	3.592906	3.455482	13.66537	6.6705	8.149903
Cbr3	5.605563	6.159178	6.746461	6.991323	8.369959	7.292533	5.891826	6.558233	8.149727
Gm15753	5.977377	3.694428	7.662035	6.278761	5.973418	6.345166	4.91623	6.57276	8.144261
Mir7036	0	8.506226	8.529924	4.240579	8.935148	10.25348	14.10412	8.12038	8.128831
Gm23301	12.7934	7.655604	15.35386	13.56985	12.18429	6.309832	13.22262	4.872228	8.128831
Adat2	10.66117	15.52386	12.47501	12.61572	9.950506	9.563339	10.13734	6.496304	8.128831
Ndufaf7	11.34509	10.29842	11.561	9.094575	7.279924	8.33374	9.203163	8.911989	8.128831
Cep19	5.871842	6.90313	6.594287	4.632017	7.123125	6.795204	4.238018	6.340143	8.128831
Nlrp1a	10.16249	9.514195	9.644529	7.685277	7.294689	7.413823	7.76134	7.885521	8.124602
Clcn6	9.983345	9.43385	8.818041	8.629304	7.724637	7.094907	11.41315	8.730858	8.117848
Umad1	6.987134	8.011269	9.742319	9.204826	7.698785	7.947152	9.646306	7.74499	8.117613
Sod3	7.780305	8.949216	8.51324	8.599355	6.144776	7.646236	4.286843	7.149508	8.112931
Nudt1	10.99305	9.490156	10.69878	10.8727	11.37028	10.38465	10.3845	10.74782	8.111498
Nmrk1	6.597793	6.124992	6.806062	6.313257	7.113584	7.943375	8.663321	6.439777	8.107535
Mvd	8.153582	8.351656	7.272959	6.980942	7.855329	7.714853	9.826234	7.643117	8.101134
LOC115487273	10.92575	9.685922	5.665862	9.254986	13.48869	16.09122	6.691738	8.090743	8.099163
Tatdn1	7.306174	9.157638	8.195715	7.461606	7.61654	6.52826	7.398229	7.1912	8.092638
B3galnt2	9.811841	8.88349	9.001033	7.704014	6.516983	7.572128	8.103232	7.884243	8.091418
Nuf2	9.65844	11.21902	10.05148	7.197523	7.859418	7.972558	8.767429	5.047804	8.084891
Zbtb48	8.115409	10.05281	8.168048	8.249853	8.418239	10.30128	9.723298	8.686346	8.079565
Ercc8	10.74214	8.6139	6.37045	7.51495	6.117852	6.988738	7.252912	7.400853	8.077382
Efl1	10.009	9.114325	10.94279	9.334742	7.697021	7.876234	9.445256	8.567718	8.072998
Cyb5d1	10.4953	12.71284	11.91684	8.524875	10.24303	8.552572	10.50732	8.689897	8.071693
Klc2	9.611553	12.02049	11.19571	10.39212	7.573794	9.02955	8.672557	7.93462	8.070108
Dnajb2	9.620746	10.15781	8.493374	8.887726	7.974936	8.320309	6.690394	6.983005	8.065698
Gxylt1	10.52696	10.32156	11.4006	8.472088	7.525374	6.523943	10.84513	8.135886	8.063636
Lancl2	8.610561	8.69347	7.95963	6.813684	6.886774	6.098253	7.928026	7.822832	8.062146
Disp1	10.56665	8.308276	9.339057	8.662548	7.688095	8.169603	10.00683	7.978201	8.056766
Ldlrad3	10.50545	8.237573	10.44757	9.153638	8.851365	7.952457	11.13279	9.439509	8.055168
Eef1akmt4	10.7045	10.33887	10.0296	4.87408	8.638796	5.991615	9.724357	9.440767	8.054481
Rpap3	9.855656	9.010299	8.155329	11.55053	7.402979	7.947801	8.51891	7.233153	8.051413
Pcca	10.29816	10.68861	10.47579	8.025726	7.791679	8.830063	11.91999	7.012468	8.049612

Coq6	9.620338	7.489408	11.10967	7.639395	8.404272	7.616503	8.655504	10.18089	8.047644
Dlgap5	11.29534	16.12099	14.00179	9.166403	7.151099	8.564575	11.80849	7.602159	8.043164
Klf9	7.249878	8.698903	7.065122	6.72947	6.209521	5.194241	10.60032	9.258429	8.041755
Kif3a	7.587121	8.595535	9.467132	8.874831	6.778528	7.18631	8.907629	7.583409	8.038993
Scfd2	9.253325	8.956457	8.844914	6.731475	5.303257	7.295064	8.181399	7.848501	8.038777
Hadhb-ps	9.225528	8.946952	8.519038	10.10249	7.627363	8.348288	9.096314	7.705863	8.037542
Trmt61a	11.32695	8.410147	9.602588	9.132572	8.21665	7.823938	6.87461	7.366228	8.037014
Cradd	7.613811	6.881647	8.430556	9.463953	9.582089	7.952574	9.836567	7.216732	8.034135
Haao	7.337391	6.685449	7.428839	6.53063	11.56048	9.884888	6.881378	8.150234	8.029213
Kctd3	8.403467	7.815282	7.890248	7.527807	7.159133	7.820448	10.59106	6.869992	8.026148
Zbtb1	7.618464	9.523647	8.896667	8.698128	7.029442	7.306255	8.534072	8.10189	8.025396
Zbtb4	10.46402	11.70698	12.47332	9.263114	10.97711	9.453624	11.09444	8.997743	8.022673
Prxl2a	7.510261	9.263325	7.873991	10.78739	8.465745	7.448534	8.512204	10.60485	8.022432
Ints7	11.92487	11.09734	12.02464	9.075608	7.567939	7.247381	10.96931	9.022555	8.011033
Ift57	8.730404	9.556109	9.364446	6.706459	6.464711	5.691891	5.842592	7.668004	8.008818
Ccdc66	8.129846	10.42864	9.949521	8.562983	7.360732	7.963385	11.94054	6.721935	8.003349
Gm10774	13.75096	6.207546	7.962005	6.908933	7.7199	12.44874	5.834523	5.512529	8.001486
Afmid	8.021258	11.78045	8.259133	11.07262	10.37921	7.073522	12.90779	8.539288	7.999801
Gm6204	5.216497	4.488525	5.319399	7.526636	6.331936	4.918642	4.122919	9.154155	7.99385
Caap1	6.151023	6.082214	6.940422	6.03815	6.108516	6.17445	5.460834	7.958665	7.992001
Zfat	7.087672	7.33061	7.465892	8.434723	6.187703	6.903421	9.004214	8.403956	7.990503
Ipo8	11.02928	12.34663	12.97522	9.205824	8.755186	8.211573	10.09302	8.989064	7.989443
Alkbh8	7.37546	8.281392	7.498442	6.678445	6.419566	4.968645	8.253942	6.138578	7.983801
Srbd1	12.11441	10.06098	12.04826	9.001326	8.149257	8.933269	10.96156	7.975255	7.983555
LOC115488691	6.38136	6.762144	3.988813	6.345614	7.217075	8.851923	8.656533	5.69595	7.982629
Herc2	11.72686	10.76317	11.38754	8.803539	8.580032	6.09927	13.65968	8.039165	7.981908
Cutc	6.3333	7.614337	7.505399	8.238934	7.808244	6.097529	8.652985	6.153825	7.979357
Tigd2	8.849441	8.494911	9.016416	6.434906	9.341156	7.655157	8.517528	7.84628	7.977446
Itpr2	9.028116	8.902531	10.23079	7.877289	8.423727	6.647748	10.07468	7.322461	7.977043
Cald1	6.017812	6.933576	8.107127	12.02286	7.013644	8.550917	4.799684	9.130648	7.974715
Rtn1	10.98738	8.382005	11.18144	7.245551	11.19859	10.41035	7.37144	5.505821	7.973377
Selenoo	8.151313	7.446451	7.581374	8.241883	7.676239	7.136893	9.109156	8.282587	7.964781
Zfp799	9.544573	10.07445	10.34116	14.31576	12.5747	8.752968	8.96052	13.93397	7.959723
Tfcp2	7.613537	10.08182	7.678408	9.129602	7.220582	6.182852	9.257612	6.456944	7.957623
Nup160	11.14808	10.17485	10.08614	7.681386	7.579814	6.566266	9.677355	8.905417	7.957216
Dicer1	10.98616	9.797825	10.3838	9.392239	8.891495	7.172921	12.87446	7.770039	7.955596
Mocs3	5.240578	7.133194	5.801565	6.915522	6.905882	6.949443	5.791118	6.997914	7.947599
Pigc	8.160591	8.458366	9.61173	7.125669	8.671304	8.684895	7.162251	9.102086	7.938466
Coq4	10.49791	9.888302	7.906756	10.21357	7.498574	8.869168	6.898532	7.465434	7.936418
Hyal1	10.95483	9.228874	7.158815	15.82441	6.599826	8.710851	15.63258	7.407719	7.929565
Tmem41b	9.405629	8.555586	9.023712	7.829662	7.148747	6.927259	7.932108	7.686201	7.9278
Il27ra	10.11502	13.53162	11.52662	6.33483	7.50296	8.679478	6.634506	6.667259	7.92561
2410004B18Rik	6.450453	8.041601	9.175044	7.982266	9.35153	9.743123	10.55587	10.20165	7.923902
Tbc1d25	10.03493	9.591204	9.150557	10.17438	9.62743	8.552146	11.03254	8.50046	7.923266
Rtraf-ps	8.427503	8.633776	7.28175	6.556014	8.244663	8.164609	4.799523	8.296727	7.922878
Serhl	11.4044	14.84944	13.75024	11.65553	11.10279	11.65967	10.87025	7.841967	7.910513
Agk	9.966393	9.343468	7.400907	7.581583	6.881616	6.244252	9.219185	7.686076	7.907799
Akap1	10.78414	9.971742	10.6499	7.274893	6.735306	6.013769	8.961565	7.765163	7.902368
Mir24-2	10.36225	8.267734	8.80894	7.728158	7.401673	9.582689	10.70991	11.34576	7.900919
Setx	9.376887	9.403563	10.57417	8.171045	6.351415	5.908653	11.30227	7.098791	7.900689
Gm30631	3.657372	5.375462	3.272766	4.59396	3.483241	3.382223	5.371688	3.115632	7.888917
Hs2st1	9.291694	10.21855	9.81003	7.874094	7.3631	5.623992	9.032016	6.647688	7.88694
Prr7	4.850831	5.244464	4.280642	5.147938	5.43515	7.162355	5.392753	7.529264	7.886759
Nadsyn1	7.104117	7.764607	6.715631	8.12881	7.136616	8.399571	8.482385	9.852316	7.883872

Qsox2	10.56831	10.13129	8.952215	9.19547	5.965331	6.592724	8.427562	6.332161	7.880023
Rras2	9.089406	9.867223	7.969386	6.687999	8.14607	8.473203	7.631339	7.238739	7.873353
Cramp1l	9.809597	10.25362	9.234515	9.836495	8.316368	6.933448	13.94362	8.456309	7.873193
Stfa2	1.965889	3.267758	5.111905	0.912276	2.496388	3.635985	0.135456	2.745188	7.8694
Zswim7	7.003479	5.661391	8.994986	3.225547	6.740247	6.681122	7.467027	6.948756	7.8694
Cd99l2	11.61007	11.53091	12.09522	9.540686	9.813353	9.006835	12.23291	9.257658	7.868746
Spice1	7.816333	10.1878	8.14813	7.916485	6.774606	7.037075	9.211279	6.418159	7.863529
Wrap53	9.681112	8.805182	9.590895	6.054643	8.031419	7.657731	8.27533	7.826066	7.863226
P2ry10	6.346307	6.390664	6.203724	5.150393	6.902033	7.57273	9.055962	3.488945	7.863173
Nprl3	10.86464	8.02571	10.37937	9.251749	8.011488	7.647932	9.846458	8.588007	7.862823
Slc39a8	10.71009	12.76878	9.931584	6.773626	6.56563	8.765926	7.549256	6.055724	7.861079
Phyhd1	10.8456	14.30311	15.55315	8.556576	8.792654	6.838414	9.727228	7.253856	7.859403
Sos1	10.06467	8.778252	9.689255	7.909907	7.471148	5.992197	10.45099	7.872184	7.857946
Alkbh4	6.541758	6.524342	6.342237	8.29734	8.805065	9.07442	7.968597	10.35947	7.857217
Lrrc75a	11.12306	14.27564	12.80852	11.72176	9.496152	9.425658	8.931344	9.282289	7.85592
Fcor	8.89101	5.08568	2.484542	2.470337	4.23385	6.166596	5.810883	5.850887	7.85084
Gm6542	7.056725	6.877985	5.988221	7.123518	6.822578	7.712355	6.40942	6.515097	7.846634
Nme7	9.900978	9.245898	10.273	9.1449	7.310576	9.464748	10.1565	7.308342	7.846089
Kcnj2	10.55236	12.61491	7.592071	6.597494	5.57254	5.872035	6.036904	7.799172	7.846082
Ccdc93	9.034577	8.812324	9.998816	9.052376	8.123982	6.757427	10.56217	6.818858	7.84584
Armt1	8.521484	9.875023	10.16886	7.053434	7.885206	7.634139	8.131813	9.427087	7.844854
Slc52a2	8.448471	7.027381	6.483662	6.869509	6.89677	7.504618	7.009294	7.080709	7.843993
Nectin4	6.337626	4.982431	7.049812	11.02872	9.315589	7.251292	9.858799	7.527159	7.843172
Smad7	6.577104	5.972683	7.858822	7.968793	6.676065	5.298003	8.890761	6.04782	7.835705
Ggn	8.993754	6.376975	7.589362	8.733765	8.164043	8.642031	8.932389	4.816651	7.835204
Tchp	7.396438	10.49137	8.501021	8.522468	9.347656	9.250278	12.1828	7.914017	7.832736
Pdp1	8.297671	7.662574	7.109298	8.702947	6.808208	7.759922	7.885553	7.811144	7.832008
N4bp2l2	7.540317	8.606737	8.253299	8.496408	8.130687	6.933542	8.337868	8.494276	7.831282
Fam8a1	8.601656	7.651323	7.982645	6.627016	7.365531	7.280915	7.44858	6.861573	7.828857
Mbtps2	9.645923	9.839146	11.65733	7.742436	6.887253	5.330782	10.51876	6.313219	7.828171
Tceanc2	7.903232	8.360436	9.698126	8.10621	8.846282	8.696486	8.856731	7.727563	7.811776
Gm34455	5.976383	8.373044	3.785482	4.358131	5.610529	5.053048	8.882956	6.367495	7.811689
Cnnm3	9.549447	9.907541	10.98207	9.357871	10.45799	9.048348	13.89946	7.922455	7.808533
Tmem62	11.36555	10.37006	8.934656	5.700295	7.917427	7.182942	9.464174	7.040813	7.804987
Ftsj1	7.06961	8.053423	7.654228	6.772027	7.211743	6.37281	7.122449	8.676147	7.804295
Fkbpl	5.469371	7.778753	7.703323	7.176555	7.890509	7.841246	7.927395	8.596794	7.803772
Snx32	7.320753	9.126578	8.752644	8.702604	7.510966	8.831776	9.320384	7.79381	7.801921
Mrgpra2b	2.815902	1.228677	1.452118	0.87504	2.095183	2.359927	0.59117	0.50269	7.799806
Pibf1	7.458846	9.060636	8.001768	10.03485	8.013196	7.184117	7.735764	8.502204	7.797698
Cacnb1	6.513972	7.671553	6.376118	7.476671	7.226809	9.38834	9.275115	6.102973	7.793516
Snora7a	6.446286	9.429383	4.298024	7.264868	4.502207	3.179373	2.665023	6.955829	7.782252
Wdr53	9.207871	8.659953	9.018082	10.53211	8.542287	10.01523	5.818558	7.949167	7.775555
1110059E24Rik	7.415035	7.976076	9.163098	11.07955	7.46871	8.257352	8.867548	7.983903	7.770206
Klf11	9.439228	8.98366	9.120078	9.20638	8.485964	6.115522	10.13003	7.847117	7.7624
Dolk	9.726433	9.232416	9.075582	9.360789	8.965332	7.837206	8.327885	7.224694	7.754124
Adcy9	12.57067	13.54675	12.05418	11.90333	11.71434	9.088804	13.67366	9.491879	7.751729
Dnajb5	9.555697	9.249405	9.838443	7.000617	9.619251	11.05903	7.101615	8.204251	7.747577
Lrrc28	8.676468	9.336529	11.00669	7.038523	8.741779	6.545671	8.731574	9.130411	7.747164
Nlrc3	8.988049	9.006338	9.454777	7.352207	7.579133	7.033118	11.25835	7.429042	7.746078
Rnf168	8.834356	9.318202	11.01941	8.649148	6.948429	7.400954	8.656256	7.8205	7.745859
Eny2	6.139497	7.420623	6.642384	6.218585	7.129659	6.331912	6.321888	8.148796	7.743974
Hps6	7.587776	8.982651	9.460116	9.467375	8.538647	7.359228	8.990006	8.986333	7.741384
Flrt2	9.151126	8.240977	8.160835	8.350744	5.082744	3.461337	6.540382	5.873947	7.739307
Mtfmt	7.520247	8.881133	9.09594	7.964065	7.343195	9.13875	8.586283	8.374887	7.73672

Zfp318	11.91897	12.31027	12.37285	10.0104	8.698775	6.64214	13.18103	8.319196	7.734932
Zbtb37	8.375805	8.709373	7.606716	10.27105	8.352625	5.922147	11.54889	5.18527	7.732302
Rnf146	9.357651	7.920826	10.17993	9.755714	9.168297	9.779555	10.95919	9.62377	7.731315
Aste1	8.597826	8.798241	7.120902	6.902073	8.956144	7.702531	7.544083	6.608467	7.725015
Nacc2	9.33081	9.270785	10.42561	9.252233	8.273405	7.960065	11.76769	6.683876	7.724717
Atg2b	11.13716	11.42663	11.62363	9.23753	8.532711	7.626632	12.22557	9.532488	7.722465
Cep70	11.11938	14.17908	10.88237	12.71566	8.736732	9.65854	13.79092	7.25948	7.721224
Ccdc186	7.315592	8.817952	8.851238	6.390215	5.979081	5.813744	8.876775	7.471567	7.720343
Snord35a	4.983197	6.833654	8.098636	7.432925	2.966214	7.488494	9.656967	9.488983	7.717822
Asb3	6.067434	7.267063	8.700437	6.694647	6.807616	7.635023	8.18952	7.095694	7.710405
Dph5	10.46333	8.374687	9.189454	12.02226	5.987471	5.529234	10.90525	11.46899	7.709819
Abtb2	9.49915	6.841559	7.310695	4.100095	5.305345	4.847793	4.437516	7.805043	7.708835
F630028O10Rik	5.829281	7.745046	7.866451	5.240787	7.624141	6.738421	3.961547	7.108622	7.70585
Bclaf3	7.093889	6.003549	6.071438	5.595842	5.473125	5.077852	5.851411	7.306094	7.703761
Tsen15	8.269226	10.05281	8.270187	7.687687	8.341527	7.828118	8.496085	6.522106	7.694767
Gstt2	9.699891	9.179772	6.01888	5.843658	8.968345	9.101107	9.147191	4.988382	7.692801
Vwf	10.99327	11.08162	9.874128	11.79815	11.73025	9.075696	9.276562	9.187967	7.692706
Ulk3	8.801085	8.8943	9.591666	10.84524	11.41742	9.409836	12.9948	9.103306	7.69152
Pbx3	9.364401	10.6708	12.03556	8.306336	7.281238	7.126185	9.385919	8.742518	7.691398
Ppp1r13b	8.597826	8.33249	9.48174	9.542036	8.785551	6.897966	9.005295	6.943459	7.682336
Zfand1	6.847621	6.691423	7.055945	5.708777	7.180347	7.73968	6.934381	4.478113	7.680046
Lsm1	8.080802	5.739901	6.460695	7.17125	6.711687	6.299806	6.239656	5.993941	7.679334
Cd300ld4	11.19685	8.579556	9.286272	16.7012	15.21536	10.10195	19.33455	12.28557	7.678341
Gm2981	7.193656	8.188293	6.725286	6.842344	5.138369	6.507849	7.354187	5.136799	7.675955
Tbcel	8.608036	8.942352	9.025044	7.456808	7.25183	6.635483	8.586341	8.162691	7.675629
Slc40a1	3.455161	3.876708	3.403729	4.466549	5.347319	3.770365	3.963901	3.190963	7.672866
Pot1a	9.920709	10.03622	9.128927	11.09828	8.189882	8.102766	9.266014	8.032536	7.672758
Nmt2	10.53462	10.81469	9.469903	7.557186	7.693973	6.870887	8.764727	8.574026	7.670182
Phf13	7.957702	7.689052	7.568671	5.569149	5.620823	6.047847	8.279635	6.918418	7.668855
Sel1l3	13.32137	14.0097	13.43364	5.210634	5.459097	4.868525	6.712524	4.977269	7.667109
Ccnb1	15.34361	17.40361	17.38027	8.188199	8.412213	10.51468	10.86089	5.993864	7.665529
Phc1	8.76819	10.70948	11.4059	7.290442	7.969867	7.300432	9.628759	6.345742	7.665164
Chd1l	10.87587	10.87091	9.697976	9.618605	9.143508	9.278863	12.73289	8.797078	7.659588
Map3k2	8.226957	9.303167	8.696168	7.019486	6.561237	5.619191	10.30035	7.210747	7.659584
Gm11221	7.953811	6.74274	8.11383	8.383813	7.272118	6.619892	6.740942	10.82911	7.656495
Mpz	4.644179	3.553971	4.615507	15.53911	11.62793	16.96308	2.354713	13.01486	7.655577
Cttn	5.140873	6.859344	8.406999	9.533331	6.450957	9.797339	5.169745	5.75448	7.647518
Ikzf5	8.726128	8.904411	8.600845	8.16239	6.891481	6.714976	11.32908	7.394355	7.642767
Mb	8.418797	9.310276	3.72303	12.69983	13.41783	20.17859	7.221443	9.269649	7.641773
Cox6c2	5.477013	4.396592	3.34003	5.180659	5.470699	7.78276	2.76135	5.34184	7.639142
Dbr1	7.038499	6.993469	8.067532	8.073836	6.854027	7.435355	8.25549	8.332753	7.637931
Nkiras1	6.570846	6.718563	6.240268	9.279434	6.415767	9.753058	6.563018	5.467507	7.630899
Ikbkg	7.415335	7.650047	7.811288	7.242853	7.117152	6.454089	8.591298	7.256022	7.624399
Actr6	6.607143	8.236942	9.361208	8.315325	7.964035	8.242242	5.44171	6.65105	7.60911
Gm52666	4.435045	13.71204	11.53246	0.88204	2.534333	3.281113	5.500608	4.644857	7.608585
Eif2ak4	10.30142	9.732045	10.02511	9.231992	8.071194	7.184918	12.30802	7.903611	7.603296
Gm5560	7.753262	7.822015	8.785063	7.13048	6.615832	6.714079	6.160578	6.57114	7.603118
Gcdh	7.217408	8.86734	8.028992	7.17698	9.140136	5.828038	11.48663	6.747193	7.601607
Gm13736	4.992406	4.384594	6.259864	5.409018	5.464372	8.889103	5.853022	7.732916	7.598927
Sec61a2	7.408095	9.394487	6.893764	8.220343	7.265649	6.44245	11.00325	8.781508	7.598298
Pkd1	11.75911	11.14596	11.56996	9.041246	8.523219	6.352913	12.50228	7.768685	7.595211
Prr5l	5.806379	5.372463	6.348504	8.20991	8.700942	8.32344	7.637869	7.139035	7.585056
Mmp27	6.162674	4.123445	4.316974	10.60145	7.08274	9.498413	11.1801	5.991256	7.583604
Tmem150b	7.355684	10.78838	11.52164	6.238822	9.168928	7.302476	6.428557	9.475097	7.577656

Cenpw	6.217353	7.062023	6.218075	7.728158	3.618596	4.951056	7.853932	1.644314	7.571714
Slc19a1	9.881132	8.003643	8.462726	6.188614	6.99302	5.654305	9.902314	7.640594	7.570499
Trmo	7.784759	6.030317	6.803007	6.764113	5.434621	5.941006	7.616299	6.476377	7.563636
Hibch	7.23892	7.520467	7.149264	7.198371	5.860128	5.271782	6.45304	7.007031	7.56052
Ncapd3	11.68351	11.47356	10.59502	9.93091	8.578624	8.508421	13.27306	7.394011	7.559469
Socs5	7.312777	8.615863	10.10774	7.271815	6.044986	6.929312	7.571427	7.906338	7.558717
Rpp40	9.622386	7.579266	7.491023	6.687067	8.174932	9.656874	8.080488	7.756004	7.555644
Slc7a1	13.97585	11.83807	11.01379	6.128888	5.56675	4.574884	8.732651	8.394208	7.554869
Flnb	15.66382	14.03612	13.78772	9.627854	7.933771	6.764761	9.2357	7.296606	7.554862
Snord49b	7.919723	8.776265	6.160501	15.75072	20.95183	7.323912	11.8234	20.94542	7.5482
LOC115489601	4.751834	10.26823	3.168257	3.150144	6.788392	0.732391	12.27814	5.278247	7.5482
Gm51644	4.061397	3.645526	2.335574	3.735748	4.738336	3.474163	5.98166	6.573641	7.5482
Emc8	7.511473	8.370175	8.043294	8.042239	8.563099	8.649021	7.728459	7.280711	7.546662
Klra17	8.007197	8.267734	9.65686	4.355871	7.760542	9.582689	4.770777	4.798406	7.541787
Rrp12	11.92175	9.138286	8.932726	7.452425	6.172059	7.001796	8.952813	8.45497	7.534222
Abhd14a	7.483085	10.8794	7.958289	5.135454	7.076619	5.701791	7.298415	5.920467	7.53385
Nt5dc3	9.387307	8.037187	7.16407	7.687982	6.510422	5.564799	9.02558	7.397612	7.53378
Xpc	8.131119	7.91168	8.864408	7.858024	8.252506	6.799912	10.39058	9.165035	7.531583
Socs2	9.16654	9.531163	12.1422	8.193977	10.58758	12.98593	9.373288	9.563051	7.528284
Rnf144a	3.501472	3.892889	3.341137	3.127965	3.498769	3.758263	2.005252	2.360947	7.52788
LTO1	7.382727	7.255189	8.059738	6.211931	7.237272	8.853002	7.99377	6.8231	7.526107
Il27	4.18146	2.892324	3.844692	5.365209	5.588173	5.925063	4.182336	4.944343	7.520652
Ccdc80	9.862123	10.81945	8.747588	10.85187	8.45332	9.792982	6.333025	7.835018	7.519583
Rps12-ps9	4.601578	5.682025	3.944668	4.575802	6.469394	6.484412	3.963301	5.945851	7.518365
Enc1	7.974142	10.38438	10.14662	9.987602	8.803798	7.033707	10.91891	7.096498	7.515351
Stk35	9.916744	9.037377	9.459678	7.795812	6.971937	6.52494	10.03899	7.144294	7.510771
Utp25	9.161375	8.68972	7.453826	7.432448	7.342179	6.694778	8.012009	6.973955	7.510401
Armcx3	8.191027	7.922168	7.911206	7.931664	7.297752	7.177739	7.37347	6.147735	7.507727
Hnmt	6.355095	6.068466	5.950142	6.185039	7.726625	6.220859	7.197188	5.728829	7.506777
Pole2	10.02555	12.92089	11.53759	9.514164	9.155827	6.688891	7.854227	6.930151	7.504569
Ccl17	13.58988	13.33856	10.13966	5.3626	14.89455	5.585552	12.03927	9.447446	7.504144
Cst7	9.839413	8.018801	8.828385	5.087834	7.657404	7.591317	3.370471	6.42383	7.502268
Ccdc28a	7.814558	12.70721	13.36558	9.291156	9.168297	10.99709	11.47131	7.49414	7.501939
Gm9791	5.918388	5.080746	7.642351	5.512752	4.994463	4.364656	4.490925	7.346749	7.497198
Gm8325	8.442508	9.79669	9.278206	8.874031	7.062234	9.380714	8.228086	9.321745	7.495751
Trp53rka	8.428485	6.304534	8.963726	6.669604	6.868341	6.751486	6.993547	7.883463	7.495669
Nrde2	8.217658	9.515177	8.747842	10.64788	8.641658	8.362254	12.00009	8.238926	7.490984
H1f3	4.857975	4.275036	4.644192	4.688681	8.028654	12.02405	8.565166	7.618088	7.489837
Zfp943	7.238986	8.311772	7.706259	7.460565	7.145385	7.838247	7.77526	8.398089	7.479178
Tef	8.793813	11.12377	10.15316	9.440299	10.03448	9.806901	10.8187	8.320325	7.479087
Spred3	8.967922	9.408673	9.590191	6.871638	7.283841	7.108503	7.382936	7.65124	7.474198
Msh6	8.921068	8.923257	10.01461	6.724446	6.576603	6.037116	10.4838	7.948943	7.473833
Nup107	10.98576	10.23091	10.45951	8.446376	6.374635	6.156123	9.587265	7.792687	7.47143
Tmem181b-ps	6.391267	10.25985	8.499137	9.808252	9.786238	6.890075	10.17009	8.685013	7.471275
Tnfsf13os	6.698172	4.477249	6.912747	7.298374	6.650724	6.391949	6.260065	7.734192	7.470583
Dclre1a	7.755164	7.491802	9.979038	8.728121	7.633161	6.286519	10.31263	7.862533	7.468675
Pdcd7	7.542286	8.71585	8.401367	8.128784	8.581117	8.582849	8.728902	8.277495	7.46826
Emc1	9.471114	9.809886	10.22002	8.355874	8.460663	8.421088	10.34087	8.483502	7.465785
Avl9	8.492604	8.552629	8.65932	7.357477	7.030871	5.876862	10.36177	7.336377	7.462463
Gmds	8.686664	6.544131	7.158941	6.524845	4.828924	4.757834	5.856958	4.507985	7.461906
Zfyve9	8.657553	6.509848	8.051593	7.512911	5.308171	6.585958	5.83733	8.219254	7.460508
Cwf19l2	6.773738	6.643808	6.409851	7.000763	6.892001	5.875063	8.406993	7.34388	7.458454
Scarf1	7.400712	10.16087	9.900805	12.21446	8.934022	8.443831	9.814568	6.80828	7.456595
Dedd	7.274229	7.353905	8.268258	7.529734	7.471724	6.635143	8.801261	8.816776	7.453552

Fbf1	9.844566	11.04	11.50179	9.530024	7.885225	7.373357	11.33535	7.364493	7.447825
Uba6	8.374987	9.339827	9.235312	6.294726	6.028799	5.572323	8.476103	6.855404	7.443055
Ftl2-ps	6.081065	5.770465	6.317958	7.866974	7.309711	8.13508	6.590186	8.094436	7.427622
Epb41l4aos	6.592635	8.06942	4.795201	4.171813	8.704635	8.729311	4.336065	8.274009	7.425797
Hrob	11.28889	15.44817	13.43446	11.70427	7.125936	7.364376	11.0345	5.332404	7.423092
Tubb2b	6.28798	6.184938	6.000238	8.375254	4.724953	6.695168	5.694026	7.057802	7.422527
Izumo4	3.346655	6.022453	3.783615	6.221742	4.018791	4.238642	4.586848	4.363905	7.419425
Castor1	7.502101	6.990345	7.326847	6.549458	7.614538	7.426279	7.426171	8.484094	7.41872
Gm10145	2.779846	1.584254	3.653936	5.054673	6.051416	4.99453	2.79102	5.595849	7.418431
Slc4a7	10.28541	9.766627	9.693742	6.729217	6.63557	4.492393	6.819107	7.131974	7.418221
LOC118568402	6.188645	8.300892	6.573376	7.838462	7.894515	8.490606	11.62534	8.687764	7.415964
Lyrm1	7.037794	8.188901	7.162097	8.71727	9.113345	9.07741	5.742562	8.757893	7.413711
Crebrf	7.064363	9.3306	9.478803	8.171541	9.550154	6.886031	10.73499	9.765375	7.409901
Camkmt	10.00965	11.92109	12.13765	10.13588	10.02196	9.527867	11.25497	10.01889	7.408418
Slc2a9	7.627326	7.031259	8.887917	6.57564	7.997264	6.48729	9.402008	8.094755	7.402905
Car3	6.239938	7.950029	4.148418	11.11876	7.076456	12.07469	6.001938	4.258312	7.391048
Gpr171	7.699039	6.460909	8.4475	2.551971	6.762967	7.71891	5.639669	4.886826	7.385362
Guf1	8.073504	9.073663	9.01212	8.183666	6.63083	6.342191	8.290535	6.843703	7.380357
Dcp2	8.332066	7.695758	8.147	8.374689	7.245071	5.267319	7.816076	7.200108	7.378774
Sufu	9.488956	10.08727	10.45868	8.740919	8.558465	8.253573	10.47887	7.738783	7.372932
Smtn	7.378728	8.235165	8.101926	9.550261	7.306286	7.942834	6.415763	6.040247	7.367468
Usp6nl	10.15314	8.691382	8.572912	7.436246	6.612564	6.12394	10.64123	7.776444	7.365282
Rdm1	7.068605	10.92717	8.533808	10.49331	7.741872	9.992004	10.01932	8.268292	7.362588
Slc16a13	8.420972	8.52978	9.211508	7.981279	8.312438	8.132208	9.224797	7.245062	7.357106
Fmo5	5.865616	6.032102	7.167384	5.878151	7.499184	7.429444	7.100329	5.562912	7.352425
Nfyb	8.623699	7.645102	7.689218	5.376635	6.431435	6.66687	6.97951	7.472086	7.349399
Map3k10	7.175076	7.735342	8.353576	6.369875	7.431579	7.506439	7.058803	7.399412	7.347256
Rbm15b	7.213801	7.032718	8.883025	6.303619	6.569768	7.229802	7.586301	7.486388	7.339482
Lrrc20	9.993579	10.49155	9.615992	9.037985	8.455792	7.257205	8.937184	7.191236	7.339085
Slc25a33	6.907759	4.914709	5.984486	6.387793	6.034126	5.08605	5.184105	5.990391	7.338528
Gm5619	7.635567	3.967603	5.197922	3.31786	5.316526	7.654505	4.17798	6.658899	7.338528
Fpgs	10.22453	9.31697	11.50087	8.923783	7.986343	7.437285	8.286797	9.431277	7.337878
Pde4dip	10.93561	12.63655	10.45397	7.771203	7.161875	6.573802	8.557734	7.031987	7.337581
Hsh2d	4.751834	3.294861	3.801909	3.825175	4.137687	4.896558	3.274172	5.429054	7.332537
Gm10157	6.566268	7.390774	7.12992	6.622765	4.824238	9.108419	4.944498	8.484492	7.331077
Txlng	8.922749	7.877798	8.631203	8.914326	7.662067	7.845668	10.1508	9.649264	7.329123
Tmppe	7.447266	6.782276	8.273006	7.357305	6.938673	6.341123	8.04813	7.244502	7.329108
Extl2	9.911594	8.826077	9.716065	8.096061	6.855016	7.038126	9.410776	6.759302	7.328635
Mettl14	8.701136	7.414189	8.765512	7.749355	6.094467	7.323912	7.225006	5.328516	7.326786
Tstd2	9.872322	8.426378	8.786622	7.473113	7.040786	6.595969	9.744774	7.490389	7.323129
Gm4739	4.988865	5.925976	5.21369	4.905179	4.431025	7.412777	3.823729	5.550432	7.319235
Arl14ep	7.118771	8.55706	7.969192	8.329105	8.559749	6.331828	6.743008	7.182169	7.319186
Zadh2	10.48332	10.38881	10.68488	9.578013	7.504047	8.891904	8.316061	6.309738	7.31864
Tada2b	8.143359	8.106667	6.985135	5.52323	6.307734	5.442723	7.311991	6.406126	7.31661
Spata5	7.159474	5.154844	5.322366	5.006402	4.740206	5.116117	6.390627	6.105707	7.316228
LOC115485611	4.264466	2.551868	5.544451	3.816521	4.467574	3.943645	10.13734	4.466209	7.315947
Thada	11.17919	9.779374	10.32774	8.918381	7.173151	6.249627	11.83169	8.403703	7.313864
Fbxw9	4.782774	6.720057	5.625677	8.494963	5.586396	6.036366	5.471757	7.503531	7.310655
Micall1	11.76185	11.35879	11.75493	8.837866	7.517865	7.74411	10.35011	7.547112	7.302345
Tsc1	9.651998	9.861988	9.978091	9.876984	7.870206	6.795131	13.43339	8.359978	7.2918
Ifitm10	5.168555	3.601295	2.478362	4.08352	6.001374	5.761852	4.976072	4.651329	7.287917
Gm2810	2.190562	3.417142	1.29202	3.686339	2.460725	4.311239	2.612371	2.459973	7.280533
Kmo	11.0521	8.01247	10.9868	9.09229	10.5259	12.10879	9.404396	5.620551	7.276247
Srp54c	8.636299	9.287581	7.023262	7.677083	6.376543	6.231952	7.618696	6.229245	7.275016

Smim13	7.704197	9.583024	9.003703	6.308369	8.079376	6.461314	8.755104	8.159324	7.272067
Tnfsf8	7.673245	8.875456	13.57732	8.488104	5.534989	4.954588	5.068128	8.861922	7.270011
Terf1	8.799693	9.893245	7.627287	8.511753	8.075977	8.433596	9.618568	8.225839	7.268637
Myod1	4.066654	5.576763	7.625862	6.303687	5.923468	6.304727	8.097129	7.316664	7.26728
D2hgdh	5.617072	7.829047	8.775515	6.713138	6.828265	7.033537	9.772032	6.261482	7.265558
Loxl1	6.493793	7.884441	8.238608	9.991657	6.137165	8.124759	4.102523	6.261791	7.265538
Rfc4	8.251883	8.593667	6.702584	8.885687	5.644588	6.83003	6.78529	5.903302	7.256452
Zfp935	5.058637	8.530923	8.577686	6.356471	6.657311	6.421713	7.533583	7.749894	7.253911
C1ra	9.192843	9.989118	12.1448	10.39878	7.856708	7.870156	7.717182	8.98181	7.250316
Zbtb24	6.728974	8.464594	7.619821	7.209316	9.240789	6.905239	8.7495	7.729304	7.240834
Kifc1	11.62719	12.85825	12.36876	7.359833	8.322261	8.721196	9.179525	3.659766	7.23613
Fam43a	8.455431	10.10512	8.961275	8.300502	8.430606	7.802646	5.944086	7.930244	7.233998
Zfp51	6.464891	5.326344	6.681885	5.525653	5.271624	6.33844	6.793523	7.287439	7.233201
Areg	4.289035	3.04894	2.920534	1.542663	4.171742	1.09708	2.829531	5.126199	7.218937
Timm21	7.935634	9.080856	7.490996	6.368323	8.114703	8.162612	9.410571	8.668944	7.218369
Zfp398	9.407562	9.526035	9.768451	8.424745	8.360065	5.871962	10.29951	6.88467	7.217568
Bdp1	10.19737	9.288551	9.912145	7.818761	7.025979	4.870545	9.297655	6.94085	7.215079
Egfl7	7.755584	6.44578	6.27222	24.46938	10.71474	15.27396	4.007892	6.654785	7.209248
Tada1	7.694625	6.940774	6.303497	5.901857	6.577941	7.382892	6.758505	6.775959	7.208513
Zfp995	5.366584	7.094905	6.615396	6.205259	6.180836	5.978502	5.28865	6.250243	7.208344
Sdhaf3	5.039824	8.243307	5.544451	8.920636	5.759848	6.058873	5.625622	7.005673	7.2051
2610507I01Rik	6.660201	7.251521	7.596184	8.900106	6.997405	7.465096	7.278129	6.904419	7.20262
Cep135	8.12924	8.595168	8.68896	7.30589	6.478438	5.55429	7.853392	6.396666	7.202074
Kctd11	10.46847	10.68435	10.73448	6.764362	8.204926	7.891636	6.988588	8.532067	7.20157
Hspe1-rs1	3.413164	5.364001	5.378945	4.011145	3.053651	3.730276	5.451862	3.348142	7.196138
Gm30989	6.578884	9.273402	6.053277	9.246072	10.19214	7.219616	8.068865	7.349458	7.189899
Rasgrp1	8.063718	7.534205	9.777311	7.048563	10.51843	9.150953	13.05028	9.215004	7.189605
Mecp2	7.724671	7.878958	8.63946	7.369707	7.129203	6.829101	7.736741	6.535213	7.185076
Gramd3	7.512336	7.364988	7.726048	8.18977	7.762712	7.754069	8.578204	8.510033	7.180207
AI987944	6.867328	8.15659	8.928563	7.573827	7.531332	6.812558	8.065598	6.677062	7.179857
5830432E09Rik	8.94282	8.474912	7.385179	8.044043	6.714682	7.27489	7.248543	6.783289	7.179379
Slc41a1	8.545488	8.358596	9.394799	6.030163	8.958521	7.750402	9.800458	7.159805	7.179322
Sirt3	8.877052	9.200612	9.487364	6.447411	10.56801	11.10659	7.645731	11.10338	7.174937
Tasor	7.635619	8.142994	7.828104	7.901279	5.803763	5.42454	9.61724	6.653179	7.173088
Exd2	8.378549	9.29632	9.44442	8.348973	8.844099	6.602129	10.69827	7.777769	7.170314
Pola1	8.236512	8.929619	9.704873	8.124056	5.081369	4.841491	8.047569	5.556049	7.166817
Pigo	8.694017	8.873972	8.992695	7.523762	7.399861	6.794124	9.374271	7.06948	7.166417
Pde7a	8.804237	9.476837	9.127404	11.22836	8.777044	7.479941	11.18957	8.07582	7.163237
Fbxo4	6.692465	6.097034	7.224197	5.343179	6.067396	6.099718	5.802961	6.877344	7.160495
Cdr2	9.68245	10.00703	8.453915	4.453867	6.419474	6.883027	9.803065	4.285309	7.15659
Lum	7.347213	8.606661	6.466299	11.90223	6.97196	14.1078	6.654853	9.666187	7.15533
Thap1	6.235865	7.243613	6.481162	5.374143	6.591092	5.585816	5.990131	5.937155	7.155307
Trmt5	9.832035	8.878278	11.49419	8.885143	9.174113	9.891011	10.09324	8.633681	7.154735
Chchd6	9.736243	8.861333	8.407132	7.618391	6.029783	10.2338	7.423446	8.408723	7.149782
Phip	7.967704	7.967105	6.909246	6.82864	5.905334	4.658934	9.361861	5.805137	7.145779
Gpr155	8.724679	9.990555	11.0485	11.20625	11.59838	11.3554	12.79551	10.13346	7.141229
Mir6955	3.745815	6.724517	5.994001	5.214766	3.567473	6.235222	7.742974	5.706213	7.140189
Qrsl1	7.023403	6.101876	6.368626	7.449666	5.589041	5.686754	7.162251	5.557614	7.140189
Zfp386	5.72193	6.400261	6.357084	6.733225	5.658653	5.821736	6.657867	5.715003	7.13956
Wdr59	7.225288	8.770669	8.375236	7.734113	8.375386	7.519489	12.2863	7.636523	7.139051
Hif1an	7.917164	8.530275	8.795881	7.445333	8.06053	6.849455	8.164271	7.009239	7.136036
Fdxr	8.794034	7.585402	9.894438	8.685687	8.969538	8.132447	10.40944	8.118204	7.135597
Ak8	9.428002	12.39633	10.45323	9.445417	8.878237	9.273845	9.707815	11.66593	7.134732
2810025M15Rik	9.750413	11.66935	8.915699	8.633875	6.98675	7.213501	8.493925	8.708665	7.124641

Fbxo8	8.088893	8.194546	7.39746	7.264587	7.009739	5.964066	7.550608	6.487231	7.119071
Cela1	5.263107	3.899328	4.56189	9.47037	9.2135	6.906727	8.185429	6.394983	7.118239
Mllt11	8.31387	6.897551	6.512062	6.730899	6.972078	6.327627	6.615695	6.3395	7.117447
Fxyd1	10.94441	12.03741	8.081395	14.54472	14.61216	16.26935	5.074362	9.738463	7.116476
Mycbp	7.466406	8.712438	10.23016	7.090476	5.368747	6.62851	7.099551	7.748538	7.116148
Mipep	9.608343	8.43375	9.724681	8.042295	7.241704	7.521773	7.430165	8.489948	7.115261
Inka1	7.151615	6.351636	5.795047	6.696281	8.50147	5.020535	6.582257	5.864719	7.11464
Abhd13	8.205358	8.684767	9.047704	7.713942	6.794933	7.668889	7.712759	7.583449	7.112326
Zbtb49	6.066072	7.052851	9.311043	9.290069	8.094344	7.079639	9.957582	7.227091	7.110943
Nudt16	6.455117	7.12648	9.079642	10.50375	8.449092	8.938274	9.739947	6.375965	7.1064
Limk2	7.753308	8.646874	7.716011	8.298563	7.984357	7.717481	9.829282	7.74555	7.098376
Armc6	9.22201	6.977383	8.507498	9.170353	7.874362	7.793035	9.367073	9.612868	7.097184
Mllt3	7.948562	8.153897	6.763372	7.828761	7.978669	5.717118	7.980514	6.006198	7.094689
Nhsl2	6.890088	6.471934	7.792968	6.444555	6.108666	5.753872	8.476445	5.096951	7.092215
Zfp523	7.99273	10.47909	9.322528	10.10265	8.021027	9.149489	9.634178	8.524602	7.091745
Cluap1	6.812452	8.25024	7.647518	8.191363	7.514284	7.264219	7.687632	6.916324	7.089156
Aen	7.968824	5.916271	7.320457	5.139328	6.280064	5.67414	7.552022	7.247742	7.085428
Dph1	7.539366	6.546518	8.041167	8.10005	6.377007	6.728387	7.656081	6.575847	7.085186
Exo5	6.757306	9.575445	10.53136	6.943436	9.117077	8.904746	7.595114	8.71219	7.084344
Numbl	6.507114	8.250093	7.571248	7.950882	6.197305	5.840588	8.000182	6.094178	7.073361
Ppcs	6.147478	7.235816	6.868251	7.214491	5.696553	7.067831	5.781317	6.907596	7.073138
Bank1	4.416672	5.60305	7.950933	6.008162	7.605153	7.057843	11.10172	7.300061	7.071927
Pde4a	8.720902	9.299411	9.709303	10.45828	9.494085	7.506874	11.8054	9.282299	7.070253
B3glct	9.704849	10.51268	11.03789	6.861003	7.110878	6.826043	9.532584	6.258897	7.06209
Ccdc181	8.4577	11.32901	8.736523	8.502408	6.937901	9.105955	10.84706	9.345671	7.060677
Rora	10.8381	11.54801	8.188007	6.551724	7.871522	6.814019	10.27496	7.200984	7.059849
Zfp758	5.397308	7.170843	7.248656	5.175394	7.123038	6.702551	4.701783	7.616385	7.054785
Galnt6os	9.969788	5.875554	7.704822	12.76796	8.77579	4.470181	16.08091	9.060751	7.054585
Gm36738	4.229607	4.971623	6.496223	2.553591	9.638982	6.006805	3.278632	5.177572	7.054585
Wrn	7.799708	8.087632	8.357802	7.430652	7.040937	5.528274	7.87859	6.836715	7.054539
Rpl36-ps2	4.468559	6.684994	7.262253	4.99897	4.787783	7.489945	4.907134	5.318133	7.053859
Amacr	4.515901	5.233078	6.882173	4.319535	4.464738	6.204586	5.156376	4.627168	7.050452
Pkn3	9.239677	10.01043	8.360679	11.75835	8.249782	11.69792	7.560153	6.754899	7.050228
Pwp2	9.709896	7.170193	8.364658	5.724946	6.069589	6.767666	7.563751	7.772154	7.043167
Cfap298	11.20202	9.88858	9.773107	9.006216	9.624509	8.155173	8.326193	7.806178	7.041958
Zbtb46	11.71375	12.13864	10.06163	6.401229	8.978443	11.74335	7.925739	6.932723	7.040516
Ubxn8	8.541043	8.910853	8.994725	8.551909	8.115653	6.169557	9.234396	7.925802	7.033732
Kdm4b	8.463592	7.614305	8.372458	6.085704	8.148354	6.956162	10.7361	7.960731	7.032631
Fntb	8.101336	8.030046	9.149341	6.79179	7.193335	8.09121	8.527716	7.286069	7.032316
LOC118568331	3.723452	4.607539	3.792846	7.268057	6.63266	5.866417	9.264603	7.287132	7.031843
Riok2	8.895754	7.24639	8.116368	6.707728	6.440868	6.205967	7.742489	8.13943	7.024052
Ptgfrn	8.307086	11.28552	12.21772	7.765111	6.697922	7.044171	8.268896	6.841834	7.022461
Dennd1b	7.811929	8.676487	9.304378	9.480698	6.636814	5.29503	10.07417	6.237544	7.022424
Abhd14b	8.419069	10.30839	11.50239	9.156775	10.09438	7.889964	10.99345	9.053535	7.021105
Septin3	10.52651	8.209167	7.142161	6.10991	7.883355	10.39928	5.559656	4.5917	7.005209
Wdr12	7.442531	8.279933	6.6424	6.837522	6.027748	5.871001	7.651848	5.97011	7.000304
Mcm9	7.984247	7.23644	7.854031	9.344876	7.895507	5.780212	9.336337	7.809863	6.998699
Mir6945	4.076328	2.439285	4.076802	0	0.77645	5.277525	1.685235	1.552426	6.993185
Gm22806	4.891594	0.406548	0.81536	0.405349	0.388225	3.392695	0.842618	0.776213	6.993185
Gask1b	11.78697	10.51245	9.479195	10.2735	7.282017	6.885163	7.2887	6.64261	6.992364
Nck2	6.007862	4.586878	4.76541	6.59223	5.544248	5.498408	6.487779	4.260468	6.989701
Ecscr	5.795163	5.431558	5.027713	10.90053	5.984728	12.07431	4.690643	6.980049	6.987313
Cox8b	2.138107	8.529659	6.262327	9.87132	12.79966	18.92515	8.681516	5.234625	6.986763
Gm19810	7.168715	6.037465	5.416992	3.168249	12.28933	10.16508	8.232472	6.825319	6.984254

Fbxl12	5.324377	5.92168	5.615451	5.936317	6.361648	6.923186	6.103338	7.680802	6.981426
Slx4ip	7.172286	8.153333	9.28799	8.736293	7.121479	6.673026	9.868866	8.364662	6.981269
Ddx20	10.82167	9.609525	9.00206	8.299642	6.955388	8.059006	8.689845	7.22594	6.979996
Rpap2	9.957194	8.007767	8.426297	9.192298	7.345018	6.594661	7.861756	6.839844	6.972825
Ebpl	7.873967	7.613421	6.62024	7.27248	8.185436	5.652494	6.207054	6.429449	6.970365
Pou6f1	6.997412	8.096931	9.472736	10.84098	8.910225	7.436273	10.46867	6.993493	6.963925
Tsga10	6.595876	6.360851	7.141819	5.881353	4.750317	4.814194	4.704408	7.473624	6.96186
Slc25a32	8.301591	8.016649	6.36341	6.72013	5.503963	5.100626	6.478858	7.402092	6.96126
Tgtp2	3.287698	2.650644	4.351291	10.61048	5.793597	3.841413	3.153832	4.461018	6.961177
Snrpf	6.614381	5.916693	4.978412	6.848561	7.143723	8.134677	7.047725	7.141539	6.954
Olfr111	9.053286	9.719651	9.320642	9.955787	8.723689	4.186098	10.23768	5.678806	6.953625
Prkci	8.743418	9.374465	9.678183	7.92913	6.885207	6.755534	9.649641	7.976231	6.950391
Slc9a6	8.906605	10.38388	9.413475	7.446019	7.190197	6.593802	7.726433	6.894372	6.948576
Znf41-ps	4.990154	5.521782	7.249315	6.555901	7.412156	5.557896	7.642244	7.467691	6.943154
Gm40315	7.777282	8.651567	9.972033	7.436267	6.077537	5.993489	4.122159	7.499667	6.937284
Tmem237	9.974594	12.13175	10.21906	8.043841	7.037883	7.058757	7.543347	7.643759	6.927119
Odf2l	6.257045	8.115736	6.744127	7.640482	6.144399	4.916872	9.851236	5.772118	6.92139
Agap2	8.385589	7.466056	8.06524	6.134155	6.961276	8.39353	7.205834	7.173277	6.920459
Cercam	11.12626	12.28065	13.14322	8.895079	11.49053	8.323291	12.89776	8.06983	6.920436
Pex26	8.604203	9.254339	9.0439	8.153371	7.519704	6.490332	7.463039	7.549531	6.914206
Gm7666	5.659188	5.808513	4.670746	6.228482	5.860697	7.062603	6.814431	5.492725	6.912326
Arl13b	8.801184	8.512234	8.990154	6.945445	6.741517	6.183947	9.161206	7.440241	6.910654
Zfp871	7.051242	8.604979	8.675483	8.399699	7.079858	5.454689	10.2585	7.087538	6.90276
Spag5	12.01698	12.04966	13.12079	8.424947	8.645385	8.134825	10.65542	5.021022	6.900791
Prorsd1	5.415893	7.057203	5.824803	5.953881	6.220749	6.16618	6.919641	7.073939	6.899729
Mief1	9.023512	9.64073	8.281753	7.584031	7.063041	5.82281	9.658512	7.377514	6.899184
Tlr5	12.36244	8.973927	13.13566	5.294821	5.438609	4.595796	8.709552	6.818225	6.89887
Dnajc27	9.738675	8.039267	6.745474	7.884708	5.828214	8.397506	7.889102	6.421606	6.898651
Tulp4	11.16055	10.45985	11.19329	7.409109	5.976297	6.053005	10.93711	6.034335	6.897567
Gm33168	3.747561	10.05281	10.21006	8.866665	7.999789	10.27738	9.616448	8.489488	6.89719
Uxs1	9.274715	8.843022	7.940079	6.468231	6.713184	8.130964	8.026578	8.029811	6.894909
Gm14121	4.902686	6.975421	4.663236	5.863905	8.881398	3.26564	8.539777	5.124828	6.894676
Rpsa-ps9	5.937865	6.460425	6.262476	5.153108	6.786189	6.888899	5.634955	7.966195	6.894071
Layn	10.05707	13.96193	10.83434	8.364815	5.764685	5.913259	8.822481	5.230961	6.893121
Slc35a1	6.14468	7.484144	6.689234	6.245443	6.991478	6.461853	8.205501	6.549272	6.892961
Tasor2	7.34952	7.344643	7.622883	7.820961	6.16498	4.590093	7.558039	5.672292	6.892445
LOC115488839	8.436227	3.6059	2.410631	7.590022	0.765197	4.458033	9.96487	7.649633	6.891835
Supt7l	8.51141	9.170328	10.62497	7.701791	8.461188	6.396454	8.47591	7.4136	6.888402
Dhcr24	10.90043	7.988842	8.107451	6.172421	7.458393	5.816583	6.87064	8.32097	6.886702
Arhgef18	5.527982	5.818388	6.847476	6.787347	7.013021	6.380375	8.48294	8.03539	6.877408
5033421B08Rik	6.423776	6.494436	8.272672	9.975457	8.799767	12.12514	8.367328	8.713296	6.877249
Msh2	7.899159	8.506226	8.70988	5.403607	6.117852	6.988738	8.368745	5.584903	6.876922
Fnbp1l	9.855892	8.097509	7.640536	6.304224	5.489918	5.498213	6.045859	8.044685	6.875056
BC003965	8.325796	6.767276	6.80447	9.592153	8.138984	8.3778	7.903827	7.333324	6.869037
Entpd5	9.62261	10.35704	9.521348	7.442329	7.041258	5.92194	9.040247	7.320671	6.862141
Blzf1	8.357355	6.927148	7.468842	5.811764	5.75447	6.109788	6.959803	6.209702	6.862
2700062C07Rik	6.983477	8.732894	8.488597	8.012722	6.088211	8.743275	6.773601	7.825308	6.86067
Gpr68	11.08761	15.39423	15.19028	5.05965	5.91272	5.17941	5.788668	8.025827	6.858005
Gimap4	5.285669	4.41343	5.101878	10.38989	11.47287	15.28928	4.001967	6.378369	6.853632
Slc26a2	11.33732	9.933106	11.1377	8.020106	7.804298	5.613799	9.151662	8.307466	6.852585
Mir7-1	9.239677	5.631437	12.83438	4.08352	6.844264	5.221678	6.896982	6.353445	6.849292
Scamp3	9.853984	9.677708	10.72027	8.901188	9.205727	8.799626	9.718115	8.486734	6.846637
Rsph3b	7.247479	8.838254	9.893443	6.182207	7.491252	7.686931	6.461113	5.167057	6.842018
9430015G10Rik	7.885015	9.604241	8.3719	8.69741	9.045179	8.456055	8.948533	7.318048	6.841496

Zfp319	8.386176	7.570606	8.847676	7.645423	7.734404	5.535794	10.12414	6.164376	6.835747
Zscan25	7.170287	6.928985	7.454724	7.774653	7.793437	6.8369	8.562252	8.716725	6.83392
Rpl3-ps2	8.554995	8.82789	8.217138	7.033076	8.470364	8.812194	8.185429	6.854866	6.821635
Ficd	6.436498	6.436853	7.700139	6.505083	5.679065	5.96849	7.39563	5.109597	6.819886
Ndc80	8.639371	10.62602	9.219759	7.664254	6.308965	7.337222	7.419324	5.395754	6.817729
Sh3pxd2a	8.647548	8.790619	8.611541	7.574821	6.878857	6.502539	8.599801	6.248392	6.80161
Lmo1	6.01562	6.528981	6.488187	5.102228	6.347066	7.962807	4.023051	4.772883	6.80141
Gtf2ird2	6.143532	8.704481	6.859449	6.965688	6.423753	6.868717	7.139153	7.504983	6.80024
Afdn	10.49741	10.57431	10.63381	8.631643	5.778311	7.166185	7.628869	6.577255	6.791642
Mcoln3	5.806754	3.533054	6.610972	7.989496	7.930225	7.70024	8.568279	7.85826	6.787413
Naprt	6.229829	7.803538	8.752444	7.596167	7.769694	8.127338	5.595658	6.037326	6.785806
Cenpl	6.53846	6.017497	5.832277	4.694375	4.327754	7.657424	5.583685	4.735039	6.78513
Ankrd37	4.108794	2.385319	3.55728	3.232034	6.190987	8.733603	3.993113	6.364258	6.78002
Cds1	8.152213	8.205653	9.057393	6.862845	7.046511	4.807624	9.500349	7.776053	6.778813
Bbs9	8.71951	11.61645	11.35921	7.93478	9.530102	7.379171	8.280443	8.087389	6.777171
Slc37a1	6.904374	5.037914	6.34667	8.657848	6.067971	6.291053	7.477075	7.371178	6.774025
Surf6	8.7339	8.673739	8.994002	8.243368	6.696752	6.519651	8.644425	9.65447	6.772217
Cyb561d1	7.103941	7.820781	8.689676	7.471695	7.299166	5.786215	8.40126	7.19288	6.770687
Slc13a3	13.32646	16.44651	16.92227	8.036581	11.9714	8.968612	10.57454	7.940291	6.768563
Tfb1m	8.383904	7.9215	8.866307	6.821134	7.335262	5.972539	7.794519	5.614344	6.767163
Herc3	9.662223	8.568899	8.254695	9.916232	5.714483	6.173969	9.491438	7.348779	6.765764
Rabl3	9.386339	7.342809	7.949979	7.350337	5.81064	6.483018	7.791316	5.808864	6.765424
Tgds	7.905737	6.957079	6.070745	6.790543	5.408069	6.948824	7.386763	6.734717	6.765053
Gm34020	3.186096	3.304718	2.931549	5.153684	4.04587	3.417826	3.336895	3.963741	6.761567
Palld	8.727941	10.29253	10.36386	8.608375	6.890895	8.321091	9.285215	7.227138	6.760477
Gm35857	4.724835	6.125933	5.512948	7.360777	10.34973	2.767274	2.767233	5.698107	6.754781
Ttf2	11.22357	8.842436	9.879624	8.413671	7.273066	6.955137	9.507313	6.375759	6.754585
Mis18bp1	9.893382	10.91797	9.191961	6.985536	8.363031	7.903947	7.704272	5.220653	6.751135
Spring1	6.436629	6.895778	7.475664	4.542343	4.647068	6.470827	5.708341	6.46437	6.748147
Snord21	5.897666	4.705572	2.359341	4.105241	2.808436	3.272386	12.80062	3.369094	6.7452
Manea	9.187769	10.16685	10.33894	8.647922	7.016236	6.159176	9.929343	6.694751	6.735969
Mettl8	7.919723	8.776265	8.515986	6.511328	7.764501	6.462275	9.683416	9.11742	6.734178
Rc3h2	6.827531	7.24189	7.330225	7.245191	7.009917	5.872717	9.35543	6.288122	6.731631
Spryd4	6.426578	5.564264	6.639215	5.3723	6.994974	5.649209	7.992524	6.118732	6.730879
Snord58b	13.10354	4.021125	3.024246	11.0255	2.879924	12.11775	13.54316	16.31458	6.72476
Gid4	7.811727	8.532325	9.047535	6.677948	6.383831	6.886142	8.620745	5.890044	6.72476
Elmod2	7.895724	7.268957	7.534766	6.566472	6.756744	4.839928	7.319186	6.471697	6.72476
Tlr3	7.647083	9.136317	12.37366	8.991598	8.586668	7.692542	8.311748	6.904829	6.723847
Rmdn2	7.317555	5.549226	5.935665	4.292167	3.372174	3.742145	5.402184	4.270115	6.717163
Taf6l	6.995815	5.596811	7.298641	9.408965	6.758605	6.725499	7.178506	6.397149	6.715602
Cenpn	14.26943	17.10425	13.60629	12.64716	9.285126	9.384677	12.50388	6.117872	6.715545
BC002059	5.157685	5.902897	5.891155	6.894444	6.120021	6.620191	5.447243	9.016222	6.71548
Cd59a	7.838123	11.80501	8.505511	5.111997	4.926258	6.162651	6.493993	4.924752	6.714312
Nbas	8.724258	7.282063	7.905116	7.702723	6.370461	4.285226	8.98632	5.785282	6.714145
Cbx8	4.059356	5.239298	5.850928	4.788536	7.694269	6.367019	7.938632	5.494227	6.713725
Mir7036b	0	0.877627	0	0	0	0.813768	0	0.837817	6.709511
A730008H23Rik	6.463181	5.321677	5.536934	4.508849	4.008209	5.397786	6.110406	5.271092	6.709132
Zbtb12	6.873808	6.217787	6.778682	6.072294	6.607438	8.219338	7.765301	7.062014	6.703707
Zfp369	8.618116	10.56088	10.24245	10.49133	8.410234	7.147771	11.4636	7.175031	6.703664
LOC115487261	5.465725	3.893695	3.123634	3.105776	3.718212	12.99737	9.68417	12.63806	6.697698
Gm20658	5.912251	6.836534	6.255717	9.968965	3.753842	3.40726	6.022047	5.547462	6.696548
Adcy3	6.987134	7.337305	7.16647	10.38396	8.245228	8.536703	11.86021	5.232117	6.695816
Tada2a	8.015142	8.437903	8.053185	8.413036	5.972533	7.823938	8.015584	5.970708	6.684249
Phlpp1	9.563611	9.248291	9.083307	6.999317	6.87662	6.67719	8.917612	6.978285	6.682581

Poglut3	9.772443	10.65731	13.01621	8.423522	9.459387	6.946857	10.85547	8.391282	6.680841
B3gnt7	8.972634	6.754173	9.593288	8.816155	13.1437	10.27323	14.1313	8.868268	6.678484
Gm5547	4.227303	6.022927	6.764471	3.843313	5.981541	6.031457	5.492619	8.164609	6.676621
Dgcr8	7.595513	7.18279	7.989992	7.67037	6.434283	4.913225	8.162186	6.070109	6.676567
Ogfod3	6.556231	9.296628	10.08082	4.257637	8.835165	6.310466	6.914482	5.817537	6.67375
Ublcp1	5.756431	5.896271	6.223892	6.549294	3.580822	4.460119	5.601161	5.678189	6.672637
Mmp2	10.35517	11.15525	8.76864	11.57086	7.542658	10.47703	5.07161	6.458252	6.671302
Plxdc2	7.900343	8.293173	9.297949	8.070346	12.75314	11.40915	12.2316	11.15369	6.670275
Usp2	8.659308	5.962397	7.612447	5.914181	8.335077	5.614049	7.548431	7.863092	6.667087
Dcp1b	7.299984	8.201478	8.224326	6.687374	6.852867	6.186887	7.544898	6.883949	6.658641
Lrrc40	9.946967	8.688823	8.331272	6.006199	7.035537	6.104743	8.795441	6.285154	6.655501
Rbbp9	8.033386	7.909609	8.599032	8.141519	7.72424	6.028665	8.117217	6.500063	6.653598
Nmnat3	6.525463	10.58272	8.171964	9.479452	6.971361	6.979081	11.84667	8.211807	6.651996
Cnksr3	6.161853	6.23828	5.250286	8.145101	6.73723	4.940812	8.388828	5.051378	6.635724
Atrn	8.848141	8.054999	8.403281	6.249378	5.536259	5.264699	7.700582	5.518241	6.635318
Cbr4	8.40272	9.818398	8.900957	9.245603	9.612661	10.02358	11.4596	9.988431	6.634292
Klhl21	6.618408	6.941474	6.049786	5.774591	6.235522	5.35708	7.85549	6.897632	6.633501
Tedc2	7.895292	7.163598	6.784469	8.219528	6.759307	6.879582	7.482619	4.749065	6.628445
Trpm4	7.602603	7.35084	7.139151	6.867496	7.378816	6.923311	9.117296	7.860043	6.623689
Gm11353	7.952814	6.083492	6.718206	6.295901	6.765281	8.568365	6.224574	9.483201	6.623072
Riox1	6.92385	7.353208	7.822733	8.224482	6.706728	7.058553	6.643247	6.952167	6.621567
Gm21811	2.844214	4.086687	4.146285	5.992122	5.738979	2.897722	5.082084	5.232349	6.616161
Polh	8.024655	9.724823	10.74364	6.57367	6.138559	5.519013	8.692443	4.89536	6.615613
L3mbtl3	6.037937	5.198359	6.103154	6.927825	5.934774	5.011008	7.947315	5.55217	6.615434
Alkbh1	6.286204	7.347477	7.424842	8.032949	6.609984	6.707636	8.878398	8.314119	6.614841
St3gal6	4.631977	4.154775	4.312027	5.272312	5.687711	4.903134	3.342133	4.382359	6.608145
Zfp579	7.164067	10.57664	7.883957	8.911304	10.17333	8.90471	10.00526	8.532229	6.607734
Syt11	10.90067	14.00724	13.49995	6.03542	6.566588	7.364004	7.602932	6.922859	6.606121
LOC115490046	7.462816	7.442948	4.264962	6.360868	7.107504	6.408423	18.73204	8.627904	6.604675
Snora3	8.315709	9.675832	6.468526	7.350337	2.63993	2.563369	14.80198	6.157955	6.604675
Snord43	0	1.151885	0	2.29698	1.099971	0	5.968542	2.19927	6.604675
Dhodh	7.135084	7.013699	7.674957	8.039431	6.624269	7.713843	7.454046	6.377882	6.604675
Mier3	5.004973	4.375658	5.48481	5.612291	5.121647	4.352705	5.558105	5.38369	6.597063
Trp53inp1	3.977481	5.934962	7.863375	8.278327	6.920395	5.911812	8.47679	7.123774	6.59243
Gnl3l	8.675073	8.880261	9.500194	5.269208	5.885889	5.313011	6.872449	6.548775	6.588313
LOC108167594	6.427602	6.891276	4.339135	11.18529	8.723248	4.903837	15.44555	10.4035	6.585531
Entpd7	8.778457	7.740666	7.468971	6.278159	5.995478	5.677545	7.538713	6.805013	6.585025
Ubr1	9.85218	9.293062	9.501421	7.28295	5.920295	4.808577	8.930084	7.519082	6.582939
Osgin2	6.415536	6.284488	5.730572	4.999787	4.026749	3.713715	8.149275	5.828453	6.581337
Aspn	5.883029	7.002987	7.520714	7.666429	6.619594	7.919352	4.70741	6.16586	6.57924
Comtd1	5.83819	6.044697	5.566717	8.757429	7.209448	6.565703	8.130641	7.042373	6.57815
D030056L22Rik	7.137948	5.932454	7.376737	6.962765	7.25131	6.160887	6.850467	6.472406	6.576329
Tram2	7.936816	6.52348	7.25964	5.909353	4.482184	4.721025	7.461107	6.873113	6.576165
Epx	0.023541	0	0.023543	0	0.201778	0.370083	0.02433	0.134478	6.573825
Sacs	10.70634	11.25501	9.618098	8.479856	5.41441	5.084239	8.602617	6.304078	6.570202
Pla2g6	7.897085	9.298493	7.713099	7.826585	7.999026	6.496962	9.88179	8.122314	6.570062
Pigm	7.558737	8.064563	7.383811	7.079397	6.745442	5.737016	7.872893	6.018136	6.568898
Slc25a23	8.51038	10.54952	11.6816	6.5783	9.844364	7.39219	8.119295	8.529175	6.567731
Qser1	9.470345	9.584716	9.634747	7.383841	7.642098	6.068678	9.161252	7.780417	6.558359
Fbxl15	4.480301	6.093236	4.345039	4.14019	5.516915	6.654297	5.004805	6.980212	6.556151
1700071M16Rik	4.273351	3.110089	4.851394	2.526678	3.574905	3.471229	8.654386	3.408868	6.549636
Gphn	8.499157	9.987198	9.728878	8.418077	7.966274	7.346146	8.645145	6.601815	6.544523
Zmynd19	7.948915	7.295978	7.193685	5.648895	6.539009	6.972969	6.631616	6.556466	6.543813
Hbb-bt	5.103822	3.071693	2.112172	8.925409	8.464538	18.87942	3.183222	3.93774	6.541773

Ovca2	5.938294	5.734137	7.051232	5.696372	6.215127	5.763215	6.397771	5.913554	6.539678
Zdhhc21	8.260426	7.507554	8.65356	6.200568	5.289656	4.844958	7.554248	7.393463	6.53728
Lrba	10.2456	8.496133	8.963885	8.258499	7.659008	5.155634	8.549658	5.746415	6.536101
Ppic	5.431723	3.998456	5.992835	6.901657	4.885718	7.614363	3.029755	6.854333	6.532773
Paqr4	11.36792	12.78722	11.0318	8.129761	8.008777	8.64057	9.066819	6.548387	6.530465
Upp1	4.991634	3.039875	4.37371	2.943063	5.511249	4.902061	1.141395	5.59368	6.525734
LOC115487645	9.581887	15.01717	4.107	14.97291	14.9922	9.49396	9.195976	11.0778	6.523136
Gm4294	4.324955	3.686031	5.976996	4.613509	5.242415	5.308538	2.438213	5.540288	6.52036
Tmc8	4.691764	4.149544	6.086722	5.501749	5.90164	4.727651	5.398135	4.593444	6.517667
Rab33b	5.896652	6.087024	6.708022	7.238649	6.327356	5.144376	7.063688	5.387202	6.513785
Timm8a1	4.793177	6.403953	4.296271	3.687159	4.608035	5.101648	2.991087	5.080205	6.507706
Gm11605	6.877654	6.339697	8.129082	8.445288	8.386244	6.649341	6.78529	7.58996	6.505356
Poc1a	7.891886	10.30016	9.029627	5.813096	7.206839	7.027866	6.612599	6.431607	6.500207
Maip1	6.848956	5.955775	6.3264	4.928113	6.464238	5.622048	5.535732	6.110575	6.498325
Gm6545	3.313032	2.486026	5.238354	8.597007	4.747968	3.55983	4.956913	6.098373	6.495662
Pheta1	6.114137	5.905042	5.679799	6.200044	8.53892	7.017419	6.119447	5.153999	6.495269
Nxn	4.897571	6.424882	5.81273	5.779499	5.496629	6.314458	4.977869	6.675202	6.488464
Ubiad1	8.424503	8.105312	7.9419	7.230749	5.49063	6.741635	6.785037	5.913903	6.487249
Gm38843	5.643396	1.986484	4.648042	2.145682	8.536301	5.372331	8.062893	7.111411	6.486028
Col5a3	7.069283	6.833247	8.331651	9.096177	5.514079	8.526381	5.111706	6.955293	6.478182
1110059G10Rik	5.360717	5.872865	6.301637	5.576721	6.880627	8.435247	5.216659	6.627254	6.476944
Pradc1	6.793286	9.618364	8.249948	6.694918	8.14508	8.862414	7.334646	7.103111	6.474605
Zfp839	6.885441	7.816223	7.837998	9.221939	7.606127	6.988654	11.07003	8.598692	6.473515
Top3a	6.287354	5.834399	5.249184	6.774053	6.112974	5.389648	8.656858	4.913871	6.4718
Cacnb3	7.639103	6.772236	6.621326	4.364865	7.968293	5.472109	7.644747	5.472198	6.471772
Gm52302	5.46838	3.385131	4.714669	10.31297	2.155045	4.882608	10.13434	5.565498	6.469886
Rps3a2	4.651266	4.388133	5.091842	4.062686	5.267888	4.824482	5.911699	5.984407	6.469886
D3Ertd751e	11.18933	10.82138	9.240751	8.007821	6.377813	4.625039	7.622212	6.053035	6.46329
Lag3	7.555962	13.88473	17.10669	6.522827	10.12215	6.782754	3.861823	13.38667	6.462887
Rint1	5.625437	6.601583	6.531235	8.382066	6.270257	5.612499	7.59327	5.237697	6.460911
Fbxo46	6.906362	7.989305	7.450005	7.984371	8.823534	6.802188	8.51152	8.464441	6.457508
0610042G04Rik	5.429952	3.668562	3.737176	5.602745	7.312287	5.237426	5.944027	8.449642	6.455122
Zmym6	7.911679	9.29464	8.475773	9.015268	8.701446	6.053665	11.05802	7.867778	6.45373
Mettl22	7.730812	6.645002	8.18951	9.407082	6.151764	9.924237	7.779913	7.215218	6.447132
Rasgrp3	11.52943	15.29864	9.855146	8.305535	6.619033	7.356703	10.19743	9.263814	6.44647
Snora73b	6.760739	4.854773	5.950142	5.10938	2.833096	7.002374	5.590049	5.66446	6.443585
Nlrc4	6.023364	6.727632	7.035298	6.679077	7.538727	4.501592	9.360565	4.703388	6.442914
Msrb2	7.54703	9.942992	7.920644	6.253963	9.361769	8.357876	9.100271	6.431477	6.43817
Tmem88	7.72985	5.302304	4.197691	7.095278	7.26189	6.080926	7.410783	8.225407	6.433828
Lbp	6.3891	6.722445	7.356004	7.510443	9.095997	9.217977	6.1723	5.121467	6.424191
Pstk	4.75482	6.044953	5.486968	5.819305	5.822278	6.861422	6.264438	6.616464	6.424151
Gm7332	6.202325	6.949493	5.130914	5.406152	5.979952	6.656263	6.806117	6.196837	6.422225
Dut	6.981815	6.547877	5.904607	4.920795	5.24953	6.68311	6.026039	6.833965	6.420806
Cip2a	8.778538	11.4424	10.88028	7.058397	5.311592	6.095286	7.351273	5.585811	6.420013
Gtse1	9.699602	11.80981	11.82212	7.699833	6.217369	8.01767	7.747576	6.274291	6.418213
Cnrip1	8.277926	7.879063	10.37437	9.3587	7.785668	6.670478	10.04668	6.21355	6.416437
Cep68	8.932328	8.63267	10.05148	7.529893	6.73978	5.691639	9.08906	7.077899	6.415185
Ccpg1os	5.069883	8.57387	8.818212	5.808666	7.347718	7.950012	5.126064	7.240538	6.407716
Lrrc61	8.043792	8.331723	8.024046	6.806121	8.113773	6.635503	7.28781	7.638791	6.40513
Dubr	5.9867	8.407807	8.156299	6.013912	5.439856	5.451573	6.376968	5.001975	6.404533
Rcc1l	6.821181	6.599185	6.464267	6.274884	6.253107	6.780525	5.703396	6.689023	6.403795
Tmem101	7.59811	8.630365	4.995634	6.611105	8.1074	6.018062	7.162251	5.291643	6.403517
Nol8	7.865479	7.628137	8.788659	6.270621	6.186529	5.618344	7.961171	6.417047	6.397875
Thg1l	6.342803	6.203321	7.818782	4.864912	4.846701	6.092975	5.615459	7.630636	6.396723

Megf8	7.758016	7.770404	8.3719	7.64867	6.726037	5.887579	8.840101	6.976328	6.394167
Ogfod1	8.045484	8.590861	9.450641	6.194228	6.410122	4.894876	7.394034	7.150831	6.393591
Gm12166	5.126894	3.933265	4.676722	5.098176	5.151083	4.16808	5.182441	6.061401	6.389889
Mpg	6.004466	5.703315	7.403192	7.613601	6.596043	6.610408	5.844725	7.440967	6.388935
Gm9762	6.249265	5.654342	5.186848	3.555581	5.706299	4.498184	6.125992	5.029823	6.385926
Mul1	7.234281	8.637284	7.888808	7.577819	7.087916	5.659743	7.338812	7.453473	6.385227
Ctdsp2-ps	7.78542	5.801544	7.580648	4.937229	5.624017	5.75977	8.896829	5.874043	6.384169
Sh3d19	7.578046	9.114642	10.52489	12.24222	8.559992	5.657572	9.419563	8.749138	6.382719
Rad1	6.527111	7.989219	8.011477	2.95025	5.893422	6.623997	6.833707	5.003843	6.382499
Mir7669	1.911657	3.813136	0	1.900949	0.910321	0	1.975793	0	6.376927
Lrig2	6.186963	8.136438	7.180589	7.597383	6.810513	5.442087	8.83338	6.602946	6.376691
Foxo1	7.578799	7.64825	8.758075	7.715073	8.149929	7.49804	8.679327	6.797454	6.376271
Gm12663	5.365844	4.087622	6.013679	3.405251	5.341493	4.513325	4.43112	5.468223	6.373383
Oaf	8.891448	9.238232	8.520994	4.709387	5.240062	4.916345	5.206728	6.697273	6.37235
Nsun5	6.658909	7.220967	8.192468	7.357344	7.046525	6.793295	7.155423	6.968896	6.371717
Gm41235	4.959899	5.178312	3.0526	3.018653	3.80744	2.837961	2.674593	2.969211	6.371476
Ears2	7.738816	6.034239	6.860202	9.514395	6.70033	5.985532	9.161864	6.530826	6.369991
Ccdc142	6.112538	5.813539	7.530813	8.368672	7.272674	6.308067	7.43464	5.667916	6.366155
Pcgf5	6.352186	5.568698	6.688684	5.666495	6.049752	5.212525	5.84394	6.342308	6.365749
Acbd4	5.766417	6.52679	6.30356	4.213909	6.232636	7.168009	5.488633	6.460554	6.365028
AA414768	7.606541	8.083209	6.7437	5.251813	5.251389	5.774876	3.913704	7.558424	6.363282
H2-T-ps	5.247047	4.117832	7.44135	6.586221	5.406839	7.039819	4.267346	6.387948	6.353605
Pomk	8.190239	8.107017	8.837864	6.644084	6.993872	5.452765	6.619264	6.18556	6.3534
Speg	7.746615	11.52424	11.85932	11.40421	7.913608	9.485066	7.559222	9.188517	6.352037
Mfap1a	5.561642	6.533299	7.508279	4.804914	5.698358	5.488114	6.97162	6.947093	6.351615
Zbtb34	6.320384	6.022072	6.201368	7.373583	7.510076	5.38616	9.308053	6.612059	6.349943
Myl9	4.70942	5.105307	4.198014	10.94406	9.311665	11.92925	3.280218	10.91715	6.342439
Zfp346	6.823146	7.536517	7.165136	7.225946	7.099382	6.988139	9.837627	7.893009	6.340488
Prkab2	7.569752	8.609646	7.324327	6.947331	7.740174	5.44199	7.904097	6.824575	6.337523
Wdhd1	10.21026	9.667235	8.218968	7.657659	4.925924	7.493471	10.05784	5.872825	6.335376
Mcf2l	5.514955	5.533474	5.204859	8.76126	6.235223	6.20832	7.523517	5.345863	6.334988
Slc17a9	5.658579	6.415505	6.700323	6.29043	8.210857	7.528432	8.634702	6.378623	6.334382
Mpp7	5.543806	8.150578	5.209872	7.081036	3.231639	6.673599	7.014066	6.825319	6.331378
Hddc2	9.110991	7.238585	9.498154	4.683536	7.941851	9.925023	8.538839	7.865911	6.328713
Phf6	5.92734	7.636879	7.349683	5.600765	5.428013	4.489307	5.197569	6.077517	6.326684
Zfp408	6.508987	6.841699	6.701206	7.963746	7.657697	6.506163	7.568283	7.321194	6.325283
Rbm34	7.052427	6.950707	7.269502	8.468435	7.144366	7.183279	7.856342	8.250725	6.325269
Naa30	5.995672	6.429125	5.958813	5.601126	4.947261	5.452007	6.584937	6.113661	6.322832
Gm52507	5.924004	7.495518	6.720546	8.704346	6.905081	5.151268	12.06274	6.650423	6.320263
Rev1	6.586885	5.694677	7.035536	6.327326	4.958199	4.469287	7.675734	5.578491	6.313456
Snx7	6.730587	8.082601	7.170969	5.709441	5.756042	4.801554	6.076201	3.975686	6.310115
Ppm1f	7.301376	6.475358	7.062994	7.022615	6.856116	5.561779	7.4757	5.726144	6.307485
Acot1	6.436363	5.568059	8.001933	4.809072	5.723518	4.768294	4.33686	4.739927	6.303885
Brf2	7.014939	7.436029	7.697285	7.852583	8.666148	9.749329	7.747453	8.892491	6.303811
Zbtb9	6.909845	6.275786	8.065742	7.881018	6.637756	7.679059	7.079926	5.820481	6.301012
Sh2d2a	1.961252	2.021232	3.072986	5.78579	4.420638	9.612635	4.459515	3.796852	6.293134
Gm6451	6.388509	5.451173	5.04041	4.023392	3.785815	7.417688	5.575734	4.865989	6.291777
Mlst8	5.860595	6.160938	7.181384	4.81272	4.944631	6.77055	5.657041	4.993389	6.290166
Lnx2	9.850411	9.948073	9.926095	9.338205	6.979874	7.168014	9.1153	5.9843	6.286502
Pbk	10.32012	12.262	9.017184	6.001787	6.990138	8.441202	7.701345	5.072509	6.285094
Slc25a16	8.329409	8.161493	7.17947	6.339211	6.314957	5.946003	7.117413	5.982593	6.284778
Tnks1bp1	6.650286	6.450555	6.377259	9.233293	6.355388	7.036079	6.437183	5.549762	6.283954
Acot11	7.724733	8.582107	8.665368	5.793062	5.934544	6.091718	6.736732	7.345286	6.278271
Rpp38	8.474952	6.191241	6.640384	7.461272	6.374904	7.687612	6.806579	7.298063	6.276692

Papolg	6.347982	7.506225	7.365104	5.668	5.807285	5.12129	7.626541	6.408499	6.274792
Kctd18	8.654002	9.180206	9.625418	10.11801	7.942269	7.251158	11.41081	7.690161	6.273504
Elac1	5.810346	6.439989	6.491391	6.631716	6.149747	5.713565	8.299047	6.031069	6.271186
Ttc38	5.979479	5.435812	7.215018	6.700196	7.693847	6.279969	8.404194	6.650289	6.270553
Mir7059	0.939628	3.748507	9.397374	0.934365	2.684675	2.606816	6.798068	4.473091	6.268844
Prdm9	6.346702	7.85506	6.156252	8.630309	7.71952	4.949954	8.930586	9.82846	6.267609
Chd6	7.838879	8.215182	8.838979	8.093707	7.618702	6.144333	11.82065	6.16627	6.26591
Pgm3	8.27609	8.488214	6.193113	7.470962	5.869617	7.490629	8.493087	5.728113	6.265525
Cish	6.708939	6.196175	7.583173	4.737059	6.72979	6.332706	10.54464	8.352972	6.26179
Cep57	6.433163	6.416036	6.117859	5.791084	6.06238	4.13312	6.905623	5.437282	6.260457
Usp46	5.724302	5.006145	6.532996	4.80336	3.716367	4.912131	5.152379	3.895264	6.258569
Eogt	8.910439	8.719043	8.47711	7.523089	6.551457	6.231896	7.211121	7.096354	6.249154
St7l	5.855161	7.344508	6.865469	6.329574	6.639042	5.503112	6.425021	6.525722	6.248931
Rhbdd2	7.509591	6.031447	6.726186	6.833117	6.088739	5.076344	6.813668	6.20077	6.245226
Golga1	8.153591	7.830703	9.58516	10.32061	7.583744	6.481914	10.06986	7.823548	6.241115
Ndufaf4	6.105514	6.24149	6.579455	4.872229	4.491952	5.067453	4.780092	5.391601	6.240981
Saal1	9.380985	8.101268	7.576778	6.935135	6.407724	5.412541	6.303629	6.171409	6.229965
Get1	8.863	6.784242	7.822506	6.984582	5.085717	5.716867	6.778661	5.252932	6.229829
Tnrc6b	6.367689	6.796289	7.203739	6.415717	6.295752	5.352764	8.301101	5.563349	6.229414
Poli	8.341778	7.591607	8.933692	8.606115	9.037002	7.424932	11.42368	9.29898	6.227856
Gm38515	8.577809	8.653989	9.273848	14.11754	9.587712	6.775668	12.49802	10.58674	6.226183
Nrep	5.049437	6.117019	5.922541	9.779418	12.06034	12.00395	7.978549	6.267446	6.223575
E2f5	8.082736	9.673462	9.034697	7.028838	6.55077	5.774542	8.517714	6.850555	6.223273
Fuom	4.509793	4.721434	4.186372	6.29321	4.840861	6.659	5.588165	4.886826	6.221752
Supv3l1	7.849448	7.913875	6.909173	6.699526	6.620195	5.795271	7.847528	7.045808	6.217364
Mgme1	6.339284	8.443473	8.385189	7.340847	7.692899	5.975837	8.813452	7.787897	6.217311
Bpnt2	8.135583	7.509423	7.735251	7.190178	7.236027	6.331451	7.464446	7.558931	6.216165
Snord73a	4.076328	3.252381	4.892162	0	1.5529	3.015729	5.898324	1.552426	6.216165
Rbmx2	5.915938	6.027074	3.976227	6.072567	2.877721	5.382634	4.40501	6.329051	6.214381
Mrm1	6.736721	7.187538	7.229946	6.053568	5.947037	5.629685	4.718931	7.372925	6.207381
Gstm5	4.259785	6.797511	7.668505	4.962082	5.795676	5.402491	3.836639	6.489173	6.205929
Rabl2	7.606178	9.548457	6.509526	9.219279	7.820681	7.208929	9.073814	5.764638	6.203435
Mybl2	8.756792	9.078506	8.325326	4.773132	4.344971	5.138736	5.944333	3.726064	6.20283
Kdm5b	5.706018	5.405334	5.849825	3.966146	5.27986	4.535531	6.449724	5.205569	6.202256
Ift74	6.472628	7.875584	7.282553	6.75819	5.548014	5.237474	5.251481	6.40908	6.199835
Cdca7	8.100861	6.908382	7.491231	8.405722	5.00927	7.578279	6.867995	6.103183	6.199051
Chaf1a	8.077735	8.808367	8.397127	6.749289	6.68761	6.369678	8.279458	4.67511	6.197373
Gm16464	4.861988	2.708646	3.684904	5.74127	5.335933	3.635611	4.239935	3.634559	6.19061
Gm10232	5.412363	3.315886	3.093138	6.304682	3.681911	5.076687	5.833688	5.447563	6.190155
Ak1	5.910475	8.732947	6.294292	8.81605	7.08846	10.34963	4.694703	5.913929	6.18088
Rps2-ps10	9.171524	6.641848	7.945683	10.97903	7.399594	7.130974	8.33206	8.620951	6.180138
Rsph3a	7.099466	8.124314	7.383171	5.400247	6.276569	8.500969	8.887037	6.220791	6.173349
Tnfrsf22	6.650817	6.851305	7.833123	7.440258	4.042198	4.653314	6.331271	5.415723	6.172009
Aldh7a1	6.89405	6.685759	6.152036	6.55243	7.091808	5.142589	6.810412	5.348962	6.171321
Mid1	3.555012	7.927309	11.84024	6.770047	6.579862	8.249924	17.01955	14.2733	6.169158
Cwf19l1	7.618211	7.016557	8.262397	7.335679	6.489749	6.022709	7.416747	6.545179	6.168833
Zbed3	9.679662	9.512339	10.02146	7.169683	10.21908	7.455165	8.684183	6.567404	6.168421
Rdh13	7.243354	6.490375	6.697108	6.189889	4.922361	6.175112	6.998974	6.249847	6.166461
Gm38733	5.352768	6.571899	4.587301	6.864556	8.139089	5.556029	8.889919	6.598907	6.165976
Cdc23	8.797138	8.131738	8.690866	6.774258	7.318239	6.151094	7.207296	5.554033	6.160529
Mief2	5.53941	5.393123	5.034414	6.579455	4.103302	4.512831	6.338677	5.797282	6.159475
Spata2l	6.785037	7.4383	5.708711	4.899629	7.282566	5.577413	6.79005	6.818911	6.158803
Rexo5	6.087317	9.850067	8.231257	8.678339	7.335604	6.060178	14.47697	7.865623	6.156963
Fchsd1	5.640694	4.950595	5.68117	5.424327	5.776625	5.866845	7.229123	5.319947	6.147708

Gm46076	4.092743	6.803078	6.325883	7.153012	7.677649	5.963991	5.255522	5.904079	6.146632
2510002D24Rik	5.354813	6.686916	5.580454	8.0096	6.385545	5.03519	5.673991	5.655265	6.132912
Ggt5	7.811344	7.314147	5.901341	6.705832	8.777467	7.171717	5.546842	6.768355	6.132673
Plekhg1	6.353402	4.767746	4.773317	4.983712	3.833223	2.880671	5.442912	4.661595	6.128844
Dimt1	6.730987	6.039588	5.991057	3.660728	4.937551	4.351181	4.435248	5.70341	6.124571
Rilpl1	5.122489	4.141495	5.729367	6.510413	4.878603	5.578026	5.544968	4.877112	6.1244
Mettl26	7.792773	10.63541	8.614066	10.19621	10.80711	7.649018	6.146642	8.656065	6.124187
Taf9b	9.389946	8.663124	7.499634	5.685504	6.094563	5.755125	5.841169	5.757707	6.12
Gbe1	6.475195	6.642998	7.069731	5.479543	4.417554	5.387536	5.925396	5.546752	6.118387
Eef1a1-ps1	7.338792	6.146906	7.212552	7.803151	5.264732	5.493983	5.976067	6.745267	6.116421
Lclat1	10.95288	8.622029	10.19311	6.959168	6.93843	6.241146	7.929404	6.366202	6.111256
Gm6061	6.788334	7.522513	6.034776	5.625258	5.387613	9.137452	5.06717	5.24234	6.110447
Gorab	5.624467	4.413671	5.973966	5.788065	4.110941	5.483574	5.02456	4.213465	6.10861
Gm16184	5.397435	7.572787	4.391644	3.456841	5.401837	3.045587	8.509605	4.529189	6.10333
Gm3219	7.36835	5.68652	6.141005	3.837992	4.678357	6.814019	5.349023	5.261544	6.103054
Ercc6	7.850062	8.084288	6.76367	7.424529	4.895386	5.123042	9.526258	4.833002	6.102777
Saysd1	6.929758	6.263374	6.545532	6.292769	6.439413	7.12047	5.844198	7.78908	6.100151
Nlk	6.707808	6.53008	6.831908	6.682496	5.542914	5.678638	7.646531	5.963855	6.099335
Pgpep1	7.104816	7.154035	8.358239	6.997086	7.026801	6.675605	8.119865	6.298339	6.09871
Noc3l	7.945611	7.312745	6.873055	6.889208	5.164359	6.471267	6.958747	6.423615	6.098156
Gm10254	10.59326	9.782461	8.554071	7.958963	8.145857	9.288358	8.921133	8.143367	6.095185
Gm12989	5.595191	4.327576	6.566548	5.677397	5.491925	7.2334	8.851391	6.033835	6.09453
Ubl4a	8.474928	7.352057	7.673459	7.430123	7.11623	7.744598	6.919298	6.970819	6.093865
Inafm2	6.137591	6.501565	6.556	5.326767	6.329607	4.584342	5.818009	6.396827	6.091963
Shmt1	5.959927	6.292136	5.20883	5.793062	4.960256	4.071599	7.297518	3.655154	6.089734
Atxn7l2	5.191446	4.451352	5.450477	4.835338	5.772051	4.453311	6.36105	5.434805	6.089734
LOC118567384	1.310205	9.718709	12.20271	8.46863	7.954886	2.726183	2.115879	9.043968	6.087618
Ddx51	8.122374	7.267856	7.894492	6.370494	6.450015	5.723412	6.36119	7.264942	6.084042
BC048403	7.465335	6.475124	6.998395	5.544382	4.846851	5.519506	6.78758	4.506907	6.080848
Ppp3cc	7.061385	9.451891	7.526824	5.882322	3.510075	6.301019	5.761811	6.133382	6.080729
Katnip	7.440099	7.844013	8.860758	7.872417	7.125344	5.404636	9.30691	6.856788	6.073832
Rgmb	6.880529	6.885316	6.533786	7.072357	7.413427	6.362897	6.776154	7.322933	6.071995
Rassf8	10.90921	11.4119	10.31573	7.075736	6.366689	5.62263	8.954469	6.667362	6.068434
Slc16a1	6.957915	5.591452	5.63206	3.882571	3.635118	3.900025	4.656271	5.945475	6.059006
Morn2	5.51576	6.060507	7.105873	3.625588	6.23255	7.780885	6.473805	6.052627	6.058926
Pcdh7	8.230804	8.936136	7.422571	4.449934	3.224996	3.713421	4.563192	4.869302	6.05488
Serpina3g	2.179111	2.062802	3.028947	6.243624	6.82407	5.225789	5.267905	5.063741	6.054652
Dnajb4	5.398573	4.755068	4.606926	5.149515	4.023815	4.327678	6.701698	5.866271	6.05414
Zfyve16	6.559815	6.735071	5.990977	5.542081	5.230462	4.777808	7.568256	6.332734	6.04853
F9	6.270629	7.959554	8.836914	7.156658	6.922182	5.535296	5.965474	7.530661	6.044381
Gtf2e1	6.231829	5.981718	6.592817	5.605885	5.197523	5.996185	6.031369	6.019053	6.042484
Gm19705	1.348858	2.0179	2.563128	4.828688	3.340058	3.991621	1.254701	3.595886	6.042233
Inpp5j	1.390803	1.387101	2.134969	8.474972	9.395318	8.255425	10.74755	5.881827	6.042083
Polg2	5.345268	6.663796	4.009417	6.340425	5.854391	5.861147	7.339827	6.397875	6.040639
Crtc1	7.725832	6.414111	8.20704	7.433391	6.632141	7.105988	9.020929	6.968081	6.039981
Mxra8	4.584918	6.047779	6.878176	5.46618	4.20229	6.815895	5.095421	5.74998	6.037889
Fktn	8.986802	10.50519	10.82627	7.427709	4.751874	5.504498	12.12305	7.028402	6.034587
Espl1	9.59923	10.52437	10.52024	7.001113	6.569962	6.085585	9.178051	6.146013	6.032867
Gm12261	6.961401	7.195335	8.164773	4.656891	6.08751	5.618344	4.840242	5.181841	6.031666
Snora23	4.820701	3.6059	4.519933	3.295667	2.008643	2.229017	6.539446	3.155474	6.030355
Scai	5.90712	7.533685	5.960088	6.783727	5.302264	5.486891	8.104385	5.176216	6.028595
Tmem38a	3.902885	6.676907	5.926237	4.730882	5.96901	7.165842	5.064366	5.506085	6.027699
Gm8242	5.49799	3.746958	6.415067	3.553675	5.585307	6.101242	5.114202	6.107063	6.026083
Rnf121	6.120764	4.128022	5.694979	5.537697	4.610919	5.265926	6.689088	5.134946	6.024898

Shprh	7.798743	8.78647	9.607347	8.45892	6.861652	5.680791	9.628051	6.245805	6.023415
Polr3f	5.570459	6.51258	5.930963	5.751285	5.127557	4.461248	5.275273	4.618466	6.020415
Mbd5	6.93368	7.405439	7.645715	6.447664	5.926272	4.35207	8.668742	5.695448	6.020334
Hectd4	8.704185	7.799802	8.540879	7.794968	6.578962	5.145646	11.39998	6.225856	6.009628
Recql	8.073504	9.048926	8.100076	7.034303	5.663096	6.044005	8.238409	6.369036	6.009246
Hdac6	10.05881	9.317495	8.411973	7.622958	7.287272	6.148376	9.670612	6.807561	6.008905
Nsa2-ps1	4.440072	4.553579	4.691942	4.352721	5.245951	5.01634	5.021963	4.626193	6.008333
Glis2	5.683843	8.215523	6.549537	8.928529	5.138646	4.151668	6.215088	4.705718	6.006034
Gm12184	7.307148	9.076889	7.5268	6.091439	6.584198	8.497357	7.733196	7.540353	6.005198
Terf2ip	5.976651	7.020768	6.51654	6.463967	6.719022	6.367469	6.670704	6.482315	6.004583
Rpl18-ps2	6.209063	5.838674	4.612983	6.262487	4.646277	5.906003	4.400487	4.898213	6.002328
Tbc1d19	7.807414	10.78773	7.645648	9.138499	7.822972	7.295261	9.722449	7.22694	5.994267
Slfn3	5.891821	5.388994	7.419061	5.798104	4.768156	3.670022	6.341905	3.894742	5.99367
LOC115487911	8.572896	2.850024	2.857964	4.5466	1.088631	5.813827	10.04192	3.264895	5.99187
Gtf2h3	7.241909	5.728292	7.013813	4.925057	5.985427	4.34926	6.000805	5.943972	5.989825
Timeless	7.493332	5.978706	6.133911	7.589114	5.805208	5.951302	6.664375	4.304613	5.989519
Cnst	8.245573	8.089805	8.807686	9.582122	7.713591	6.429573	9.581185	7.420902	5.987076
Rps4x-ps	8.031876	7.950264	6.58328	6.365488	8.10959	8.991339	8.238929	5.174752	5.985936
Daam1	7.536379	6.927917	7.204169	5.412452	5.5463	4.373479	7.710545	6.387168	5.985699
Gprc5c	6.983358	6.26218	7.719346	6.396011	10.26802	7.647621	9.876783	6.06561	5.984346
Smarce1-ps1	4.02489	5.161081	5.654669	4.097638	2.464239	6.602282	3.91231	3.421508	5.982454
Pabpn1	4.792471	5.057243	5.133178	5.226815	5.771627	5.86158	5.84804	5.622672	5.982148
Psenen-ps	5.500831	3.171701	3.868221	6.495646	4.993356	5.166481	4.53054	4.664497	5.980047
Eef1aknmt	5.989154	8.248717	6.648075	5.759267	7.187463	5.295128	7.119736	6.099122	5.980014
Mcu	8.609114	7.931641	7.258751	6.948525	5.147496	4.944661	8.060026	4.704844	5.979162
Rita1	5.110311	5.76455	4.018229	5.992884	5.571881	4.432527	6.083132	5.133959	5.979057
Grb10	4.13109	6.023458	6.238089	6.09828	4.277973	5.283855	3.536023	5.528642	5.978035
Capn11	6.599769	11.37258	8.580697	12.72454	10.58067	14.0375	17.43193	10.92653	5.975658
LOC115487678	9.835785	9.8096	15.20253	7.113229	11.92227	15.71097	18.48323	8.513301	5.965513
Scarna17	0	0	0	0.889154	6.812723	0	0.924161	0	5.965513
Gpatch11	5.586782	4.928996	4.539788	5.448651	5.947517	6.333907	5.03856	5.779476	5.964687
Ppp1r13l	6.042927	6.43574	6.738914	5.9559	5.466608	4.945407	6.429905	6.177755	5.963321
Proz	7.779826	6.077068	7.78073	5.355864	5.285042	5.232393	9.39035	5.542418	5.963004
Fdx1	6.393187	5.51083	4.43008	5.585408	4.740567	5.025386	3.587025	4.739118	5.962705
Eif2s3y	16.41859	26.81975	27.02962	7.860963	20.72048	41.39505	37.39559	33.29059	5.956684
Gm30732	3.993982	2.011525	1.977317	2.09796	4.347228	2.405076	3.757696	4.573302	5.956538
TrnN	5.465725	10.12361	3.904543	5.435108	8.180065	3.610379	8.070142	13.38147	5.95351
Mfap4	4.392931	5.720952	5.991057	11.8053	7.019068	8.259186	5.328302	6.844092	5.95156
Zfp169	6.704138	7.149187	6.266519	9.05117	8.300432	5.126738	10.31364	6.112946	5.947279
Mtrr	7.855874	6.754867	8.200629	6.307614	5.058843	5.182377	7.248864	6.432096	5.947279
Pygm	7.66861	9.728811	5.563769	8.470815	7.597848	9.003053	5.570085	6.694362	5.946509
Rfxank	6.056897	7.251954	6.270148	7.54758	6.433565	6.541786	9.099902	8.483929	5.946378
Thap2	6.472973	6.133509	5.905464	4.79707	4.605023	4.749651	5.976219	4.635438	5.946331
Klhl11	5.416362	7.830547	6.600536	6.472279	5.418576	6.69254	6.468371	4.961899	5.943123
Galnt4	8.207826	6.488223	7.818402	6.04861	6.350702	4.9633	7.609103	6.493286	5.942011
Atg14	6.409675	6.360567	7.518988	7.603797	6.441093	6.640545	7.63752	6.530894	5.940571
Prr11	10.35752	12.8438	13.0969	6.06446	6.511874	5.907739	8.668812	6.109912	5.936786
Nudt18	5.394685	5.832328	7.222992	5.626148	5.235305	6.070426	5.862771	5.887918	5.935847
H2ac23	10.04313	8.313603	5.725248	8.48884	7.651972	9.008943	5.70904	4.49416	5.934635
Samd10	5.513318	4.865156	5.00576	5.0782	4.331325	4.628632	4.306541	3.701062	5.932705
Gm6286	6.865593	3.647635	4.556204	4.33874	3.60546	4.924992	4.310614	4.520721	5.931976
Glb1l	7.162484	7.645857	7.229035	7.253049	6.487699	6.299548	7.92347	6.339736	5.928811
Tesk2	5.9501	5.845689	6.705743	4.967438	5.074756	6.364793	8.169039	6.278091	5.924899
Skp2	6.603132	6.638378	6.692187	6.513475	5.27986	7.265601	8.68594	7.043266	5.923173

Hace1	4.880038	5.052654	5.43898	5.161137	4.411372	4.780621	5.855895	4.803776	5.912428
Gm36599	6.159785	5.631437	7.06878	4.476167	7.220322	5.294709	5.713476	5.52637	5.908456
Cep85	7.526308	7.272187	6.91687	6.75285	4.336549	4.369955	6.307147	4.484201	5.905619
Fer	6.682411	6.923441	9.181273	10.9997	10.84395	11.27942	15.79132	7.937446	5.905174
Slc22a5	6.408687	7.615553	7.052323	6.275937	5.417144	4.683598	7.368612	6.620991	5.903827
Maml3	7.407155	5.260879	6.174774	6.122442	4.974845	4.695947	6.991879	4.459687	5.900747
Rpl30-ps10	5.94829	7.949488	6.781839	5.91497	5.098578	8.581236	4.918284	9.061368	5.896019
Gm2962	6.070697	4.797933	6.873286	6.264491	5.890753	4.978444	8.168517	6.216117	5.895082
Pls3	6.49031	9.745507	9.08749	7.188988	5.837894	5.918674	4.136636	5.321159	5.893733
Hus1	7.831081	7.660857	6.89044	5.957432	5.318578	4.570732	5.816046	3.972436	5.893481
Hmgn3	7.32802	7.816929	9.559398	7.033512	8.435639	7.7785	7.573874	7.159002	5.891066
Yipf2	6.587278	7.994153	8.482835	6.724283	7.161956	7.304659	6.476904	6.96551	5.889342
2610002M06Rik	6.39597	5.631262	6.652961	5.454247	5.494959	4.177212	5.521872	5.285475	5.887906
Lrrk2	5.147917	5.228541	5.263377	4.554772	4.523205	3.929708	4.419892	3.151768	5.885131
Fignl1	7.686282	7.605458	6.416067	4.79457	3.227862	3.582001	4.879088	3.11163	5.88364
Pacrgl	5.149984	6.382575	7.195667	6.439076	5.085111	6.128273	4.65742	5.624361	5.882852
Adora2a	5.272208	2.804358	3.834779	3.526891	6.573198	4.698279	4.722544	6.145279	5.87759
Gm46224	3.346539	5.577355	5.945201	9.457939	6.919595	3.94991	6.553545	7.797887	5.875485
Zfp951	3.969811	4.723307	4.214061	6.613918	5.240062	5.474532	4.606875	5.603164	5.87451
Afg1l	7.822367	5.673848	7.20097	8.161584	8.042545	6.274843	7.656067	6.911654	5.87396
Pex10	6.631025	5.6734	5.520886	6.225695	6.475603	5.073821	7.758018	6.890243	5.870822
Cebpzos	6.159785	5.714777	5.730698	7.620989	6.071157	5.100244	6.070331	8.183329	5.870822
Gm6807	6.616065	5.972736	5.875292	5.558125	4.236925	5.063445	5.600114	5.973325	5.870822
Socs4	6.978049	7.392933	7.679161	6.250667	6.224226	5.559921	7.436762	6.843023	5.868265
Nup155	7.869019	7.450918	7.921881	5.345752	5.367253	5.539824	7.139883	6.264003	5.866238
Hexdc	6.303164	7.622858	5.857164	7.353627	7.18477	7.068198	6.925005	5.386931	5.865566
Wwc2	8.152656	7.724404	7.189996	7.204165	6.802761	6.048592	9.680529	6.015649	5.862973
Rps24-ps3	5.598695	5.58379	5.379764	5.239844	7.945929	9.339801	5.899993	8.361579	5.859197
3110009E18Rik	6.370906	5.462163	7.713046	7.002085	2.980566	7.44204	7.970892	8.087636	5.858986
Rab3ip	6.502292	6.941332	7.442377	5.02901	6.215648	6.213554	5.177231	7.06212	5.85297
Gal	2.962339	2.532388	6.278067	5.26026	2.149561	3.196058	4.228097	3.760583	5.848414
Repin1	5.661919	5.946016	5.606326	6.431855	4.99953	4.90655	7.208271	6.247502	5.843026
Tubgcp6	8.56493	7.977718	8.707421	7.716122	5.814687	5.958047	9.189727	6.154846	5.839705
Shld2	6.125753	5.871855	7.045434	6.835948	5.023808	5.916678	7.73822	6.901575	5.838387
Gm32276	6.120362	7.784898	7.274319	7.938364	4.223888	3.855307	4.675517	6.164992	5.833249
Nckap5l	7.216112	6.888462	7.858457	6.640361	7.003451	7.255818	9.411552	6.183401	5.829581
5430416N02Rik	5.224993	5.122759	2.922793	5.107662	6.157026	4.58622	6.315595	5.311974	5.823931
Akap11	8.306452	8.074204	9.009812	7.282192	5.788814	5.095445	7.66129	6.607687	5.823493
Tbl2	7.875887	7.293854	7.641521	6.377299	4.656408	4.956478	6.205522	4.859493	5.819913
Ints2	7.007656	6.722631	6.970312	7.158105	5.426422	4.845644	7.913145	6.296601	5.818296
Ro60	8.465438	10.29996	9.080766	6.890941	5.448055	5.822612	7.797132	6.707468	5.799673
Mir7035	4.732518	2.697096	2.70461	9.412016	5.794969	4.376484	10.48134	10.94271	5.799227
Gm22513	4.732518	5.394192	1.690381	8.067443	5.473026	4.063878	2.795025	3.540288	5.799227
Xlr	3.705192	5.856746	5.243806	6.256592	6.658084	8.857039	4.190775	8.785985	5.796789
Gm52860	3.934314	4.042744	4.411713	6.283352	2.95218	1.764039	9.611278	2.724256	5.79507
Bcl9	6.912568	7.122751	9.159759	7.338789	6.967635	5.875359	8.281537	6.066449	5.793153
Trmt44	5.057999	4.685633	4.418765	5.049546	4.912384	5.380025	5.868241	6.033914	5.792488
Sult1a1	10.76854	15.82253	12.01076	11.23703	11.39538	10.94196	11.35884	7.97433	5.78635
Zfp518a	5.022415	6.467483	5.536616	6.379178	5.305559	4.499681	5.397834	5.262501	5.782331
Cd209c	10.82629	12.75218	10.45418	6.774932	11.82191	9.235034	6.366445	6.309016	5.78187
Tti2	7.23995	6.940013	7.675751	6.995879	7.138898	5.394076	8.143879	6.673925	5.778709
Snupn	4.995158	8.411992	8.844914	5.781469	6.278121	6.171775	6.64386	4.249106	5.775432
Gm38396	4.479582	5.448991	5.205206	5.484429	6.954323	5.507472	4.308724	6.212603	5.774849
Ighmbp2	6.29618	5.68702	6.296912	6.497173	5.61928	5.895749	7.10086	5.862624	5.774373

Utp20	8.594124	8.114194	7.446176	5.512128	4.896621	4.110215	7.850819	5.761782	5.773762
Itpr3	8.084333	7.115867	7.891669	7.461557	5.434376	4.620373	5.840319	5.336172	5.772227
Gemin5	8.265642	7.898639	7.820498	7.160242	5.756695	5.825457	8.458277	5.962946	5.769601
Btla	8.468291	6.797177	7.045013	4.931132	6.563496	8.160451	6.25547	4.866641	5.768487
Mrps25	5.009134	5.751083	5.310846	4.373184	4.753265	5.32418	5.111178	5.490211	5.765503
Usp35	5.629046	7.988005	6.080076	8.348294	8.087515	6.083075	9.092547	7.916605	5.763522
Bach2os	5.573845	5.026406	7.744206	6.471925	6.738895	7.284228	7.554644	5.592844	5.757718
Mtf1	6.945196	6.06449	7.142177	5.504806	6.06793	4.689377	7.313361	6.128328	5.746959
Ptprcap	3.407541	2.548852	4.259921	4.170404	6.865067	8.968762	5.418251	2.370844	5.745911
Fyco1	8.874985	7.810443	8.537676	8.660647	6.731788	5.757763	8.942119	6.57211	5.742002
Sgsm2	7.208767	8.327375	6.862353	4.965259	5.322376	4.495261	8.014543	5.383717	5.736002
3110056K07Rik	3.621876	3.961804	3.739145	6.273733	4.673428	3.781577	3.441503	3.670857	5.734723
Dnajb13	5.050141	6.055354	8.285464	8.802348	8.970899	6.874132	8.797032	6.861181	5.732614
Pidd1	7.208541	8.798017	6.731409	4.811777	7.282566	7.108193	9.014557	5.555052	5.731643
Pdss1	8.013477	5.787414	4.663557	4.877326	4.704175	5.078825	6.640143	5.360463	5.728167
Dph2	6.083164	7.147724	5.788298	5.681735	4.010911	6.126578	5.778164	6.776603	5.727377
Tcta	8.260024	7.898639	7.363723	6.367967	7.248023	7.123649	6.107098	5.507992	5.72012
Shb	5.939792	6.065026	7.173032	4.580567	8.042936	6.763409	7.224894	5.770678	5.699661
Grpel2	6.237543	6.645781	6.192622	7.064916	6.273819	5.655732	6.462536	5.533179	5.698435
Igfbp6	9.762948	8.450134	6.279983	8.339695	7.332002	9.823929	6.223212	7.780193	5.697749
Wdcp	4.718133	6.482676	7.17842	6.263924	5.831987	3.713346	6.743993	4.778856	5.692757
Ydjc	5.921793	4.398106	6.384519	5.638042	5.239861	4.00041	5.55617	6.477848	5.684023
Ing5	6.448843	7.357445	5.692254	5.489676	5.304288	4.664779	6.715695	5.977129	5.68069
Hemk1	5.466299	3.702305	4.93656	4.259301	6.060767	5.658652	7.420492	6.214272	5.676424
C1rl	3.867772	3.783999	6.097054	4.285661	5.700846	4.803124	5.482334	5.383461	5.669927
Gm40096	4.758632	5.457858	7.376737	8.754156	5.438483	7.261046	8.115168	7.02256	5.669249
Tpi-rs11	5.690874	4.336508	3.83699	4.387312	3.897475	6.859304	4.956575	4.383319	5.667679
Zfhx3	7.778949	8.149691	7.667102	7.141624	6.975126	5.521756	10.62497	6.452207	5.666977
Trim59	5.943914	7.405316	8.233952	3.290054	5.624279	6.012622	5.149266	4.889979	5.665078
Snord98	5.939792	5.923979	3.960322	27.56376	0	0	0	7.540353	5.66115
0610040J01Rik	5.937629	6.103101	5.392963	7.350337	11.1079	8.404489	8.453804	8.076006	5.659087
Sigirr	7.852947	7.505705	7.059123	6.228574	7.211951	7.824112	5.797437	4.138929	5.657968
Hes1	7.545097	5.827294	6.99383	8.509311	12.13711	12.67857	14.40771	12.44002	5.656452
Adat3	3.261063	5.485943	4.793926	6.407452	3.367735	4.360089	6.213037	5.424138	5.654463
Serinc5	6.69134	7.640251	9.027062	5.441239	6.630846	6.33252	8.014611	4.862457	5.65228
Sirt4	5.4974	7.512099	6.46198	6.24755	5.167668	7.097545	6.198368	4.826214	5.647784
Cd209d	11.68736	10.88939	7.843744	10.24561	12.8152	12.87018	9.933773	6.222621	5.642829
Ndufaf1	7.763847	8.203582	7.638834	9.139234	7.953764	7.296191	6.115835	7.112248	5.639722
Cavin2	5.189946	4.995146	3.901987	13.11873	7.829057	8.978085	3.938652	6.928239	5.638295
Fahd2a	5.543806	4.496958	4.657339	6.174283	6.969416	4.648243	4.88943	5.348624	5.635989
Kbtbd7	4.051667	4.736742	5.288591	3.785529	4.255131	5.388226	5.149103	5.908746	5.6349
Gcat	7.578317	5.958701	6.258342	5.003065	5.420617	5.234333	5.915052	4.52079	5.634391
Tmem29	5.48846	6.623817	5.996494	4.49459	5.886034	6.525715	4.051856	5.708587	5.626611
Hdac4	5.680442	5.50622	5.528496	5.20032	4.201165	4.258919	5.892526	5.078158	5.625501
Miga2	6.767808	7.310856	6.444657	5.339843	6.689122	4.303812	6.483907	5.46977	5.621691
Gm17971	3.885846	5.748659	2.39655	5.087704	5.181171	2.036321	9.705854	3.329735	5.61706
Zfp81	5.314967	5.448062	6.349171	6.043719	5.038482	6.098384	6.179945	4.849521	5.605033
Tagap1	5.99521	5.3696	5.690233	3.857524	5.84412	6.500826	6.852433	6.513867	5.601929
Gm14584	7.323615	6.36987	7.665139	4.318746	7.38045	6.300139	6.07306	5.351218	5.600277
Mynn	5.76733	5.510119	6.189792	4.86481	5.522867	4.192654	6.82171	5.230062	5.597267
Nol4l	8.46789	8.275037	7.745554	4.275155	5.998319	4.040825	7.070111	5.585244	5.591056
Nmnat1	5.271566	5.726513	5.791971	5.832689	4.478453	5.080964	6.113492	4.477084	5.590385
Cdc45	8.478141	10.35215	7.501782	7.380102	5.357838	6.814483	7.534183	4.777832	5.588329
Bckdhb	7.977113	7.841402	10.90523	8.388971	6.06692	9.28757	11.15116	5.354743	5.579104

Gm13822	4.017251	1.946041	0.803544	1.826171	4.044614	2.759735	1.304924	5.354743	5.579104
AW554918	7.724489	8.148381	9.003047	8.212997	6.08344	6.154284	9.856731	6.873601	5.578225
Carmil1	7.327334	7.318653	7.860157	3.832048	4.373176	2.730513	4.52146	4.06178	5.576534
Fam122b	7.644337	7.524543	7.977627	5.915963	6.140845	5.83981	6.835913	6.233901	5.575169
Them6	7.488609	9.438024	6.334386	5.594258	6.670791	8.131117	6.238089	5.675534	5.574914
Gm4610	6.917043	6.036299	4.934052	5.158723	5.667373	6.34963	4.783595	8.232128	5.574588
Simc1	6.246105	6.632377	5.25231	5.871205	5.179235	4.856608	6.134484	3.609563	5.568067
Morn1	6.639506	8.141594	7.801419	6.313689	6.046961	5.804488	8.587201	5.596047	5.567291
Uprt	6.579929	6.421284	7.099601	5.448651	4.761757	4.245626	6.524432	5.538716	5.566958
Ocstamp	11.42205	9.667352	13.49263	5.946783	8.610815	9.85508	6.620322	6.179543	5.564675
Mir12181	1.945195	2.910025	1.945421	0	0.926291	0	3.015684	0	5.561831
Rnf138	6.17773	5.315617	6.057302	6.464335	7.11416	6.683803	5.946807	8.649713	5.560819
Zfp61	3.16519	4.994282	3.141934	4.368862	3.891844	4.761947	4.833833	3.103528	5.560647
Cd38	4.655105	5.450139	7.268696	5.52556	6.641444	7.963205	4.906367	5.255473	5.559063
Nek1	7.588315	8.649272	8.044549	5.303626	5.678379	4.872106	7.730861	5.305082	5.558569
Cdkn2aip	4.322344	5.045275	4.595025	3.947914	4.223278	5.181514	6.105389	4.648759	5.553801
Bcam	5.896196	7.212799	6.610957	11.54311	11.47224	9.86157	4.5229	6.710193	5.553744
Oxld1	3.649088	5.878987	5.544451	3.419302	5.747696	7.268287	4.569335	4.810554	5.551271
Chp2	3.740057	4.768862	4.829496	5.931741	11.22703	6.65469	8.758619	5.408964	5.549957
Reps2	5.822935	6.093551	7.048819	8.380926	7.229347	6.496259	10.65823	6.134578	5.549774
Hells	9.108075	7.820149	8.962212	6.719751	4.039906	4.41521	6.225156	4.825426	5.548014
Prelid2	4.211455	4.658451	3.599298	6.396011	5.542395	4.177867	5.935567	4.665854	5.546467
Gm7413	3.842242	1.532805	3.183942	3.384066	4.704826	4.060783	4.08461	4.285309	5.545311
Pogk	7.937132	6.441372	6.77619	6.341604	5.748734	5.116221	7.195837	6.033166	5.543901
Zfp24	5.892717	7.172295	6.358671	5.89826	5.79677	5.619694	7.232371	6.007236	5.542384
Gm36393	5.911406	6.292016	7.203811	5.285524	4.11602	2.848188	4.518122	5.628238	5.539405
Gm11263	5.276635	5.329202	6.54646	4.981403	5.216248	5.68265	4.072991	5.53262	5.538378
Gprc5b	12.78673	15.82198	10.25225	7.327349	4.396444	7.295281	6.540664	5.983939	5.53574
Rtel1	8.168673	8.337021	8.877657	6.904479	6.081175	5.791502	8.752298	6.04043	5.527685
C030006K11Rik	6.01971	5.689355	8.132281	6.205372	5.973236	6.703524	7.296619	8.401985	5.52705
Tshz1	6.479162	7.636805	7.316413	6.091439	6.159463	4.695334	7.536648	5.686509	5.524012
Lox	6.488619	7.372886	8.582319	5.073161	3.137018	3.710215	3.186778	5.387891	5.52332
Meak7	5.276739	5.004926	6.311282	6.532205	6.235733	4.222489	5.565074	4.039684	5.521857
Mir194-1	0	0.825231	0	1.645598	0.788039	6.886664	9.407135	0	5.520325
Cbfa2t2	5.52421	5.918993	5.70217	5.1221	4.763517	4.306081	6.982622	5.375087	5.514086
Gm26637	4.906671	3.505869	6.848166	6.190013	4.498692	5.485678	5.714662	4.148688	5.514075
Nbeal1	5.822971	7.206604	6.190269	5.138412	4.860909	4.074531	6.754228	5.470208	5.509495
Zfp654	4.631145	5.665266	4.866886	4.569225	4.44511	4.190726	6.020544	6.1403	5.508745
Usp42	6.220773	6.214945	5.67254	7.095622	5.54534	4.757486	6.974539	4.55993	5.508383
Gm24727	1.539946	1.535846	0	1.53132	1.466628	1.424094	1.989514	0.73309	5.503896
Aars2	7.640709	7.898639	7.190828	7.683279	5.866512	7.998744	7.431216	7.622091	5.502617
Snord17	10.03404	10.25751	3.261442	15.21619	13.61774	12.75885	13.74115	8.598049	5.498915
Dmpk	4.430805	4.54587	4.537332	8.221696	4.603473	6.685345	5.236797	6.943468	5.495898
Wdr25	5.9479	6.475461	6.130228	6.998171	5.837684	4.534707	6.100525	6.527562	5.495782
Syn1	11.55905	9.595291	10.05859	5.72979	7.017641	5.118665	7.886371	5.502676	5.491761
Zfp526	6.617013	5.745744	6.97995	7.267405	5.753292	4.779679	8.302086	5.35974	5.490822
Kif2c	7.942416	10.25805	9.949032	5.192886	5.729934	5.196515	6.50554	4.55608	5.488125
Cox7a1	2.726462	4.83414	1.96934	5.573001	3.173687	5.042694	2.974487	4.326432	5.48585
Wdr47	7.325496	8.804068	7.580574	7.904906	4.797872	6.304243	7.738455	6.578557	5.484165
Lrrc8b	8.493874	7.496033	7.906436	4.746406	3.740888	3.793327	6.586273	5.309806	5.481189
Trmt11	4.773368	5.011221	4.131816	4.607839	5.210738	4.569148	4.183389	4.438405	5.480616
Mdm1	8.385589	8.150311	7.221763	5.230938	5.305742	4.020971	7.082001	7.762128	5.476145
Cacna1a	9.024346	12.46574	8.124813	10.93985	9.498901	8.463101	12.74638	7.939736	5.47591
Prdm15	6.036842	6.832195	5.679522	6.553169	6.276324	5.973922	7.786609	5.763158	5.474494

Castor2	8.939373	9.205417	9.138054	7.166925	6.830937	5.385817	9.587714	5.699931	5.473197
Gm16217	6.268057	5.358316	5.294226	7.254311	5.166989	4.456975	10.18026	6.243627	5.471997
Macrod1	8.108443	10.63104	10.20507	8.017725	7.852545	9.815366	7.533016	9.368129	5.470429
Fbxo5	5.890486	7.924866	7.977627	5.979521	5.697691	7.178001	5.961276	4.99491	5.467954
Tuba8	2.453012	2.446481	3.132671	3.67767	5.57099	7.677893	3.510429	3.305641	5.467178
Tlcd2	3.886981	4.022006	3.207132	3.816893	5.275233	3.909082	4.670214	4.811023	5.464341
Alg14	8.076877	7.708906	8.714776	6.563486	7.775042	6.398384	9.03603	5.429841	5.463084
Zbtb14	6.401412	7.178597	7.137331	6.396011	4.769377	4.985116	6.42624	5.52612	5.458891
9930111J21Rik1	4.061344	4.007593	5.109564	5.779614	4.141335	3.278034	4.242099	2.978664	5.457448
Ccr3	2.523762	2.871793	3.811492	5.99615	9.340175	9.037972	8.543057	6.353893	5.456474
Tbx6	3.976024	6.963698	5.75459	4.050186	9.051189	5.829236	7.71769	6.278498	5.453189
D16Ertd472e	4.365795	3.640988	3.876975	3.405706	4.579105	3.967741	3.909334	4.568747	5.443364
LOC115485831	4.076328	5.691666	6.522883	2.432097	2.32935	4.523593	3.370471	1.552426	5.439144
Vps13a	7.559413	7.681004	6.615255	5.581282	4.621485	4.066963	7.306358	5.391212	5.437451
Dtd1	4.753069	6.981338	3.889326	6.702957	3.827179	5.594259	7.145503	6.788081	5.436116
Trim36	7.120864	7.12938	7.300767	5.957882	6.099715	4.725515	7.374004	6.045396	5.434704
Mettl25	5.167107	6.416753	5.367748	5.801153	5.079842	4.285127	6.132919	4.6022	5.43307
Lpp	6.511808	5.392831	6.240119	6.382562	6.095154	4.189459	9.021273	5.521268	5.430985
Gad1-ps	4.168275	4.087892	5.054621	4.144927	5.557748	5.187771	4.559428	4.464683	5.429407
Dpt	5.793003	6.971828	5.340539	11.61765	4.993213	8.499671	5.452174	5.854448	5.428711
Mgmt	5.046378	6.111431	6.561053	5.734983	5.08075	7.066766	6.407839	5.765575	5.428029
Insr	6.35271	6.761336	6.466056	6.74141	5.864003	4.444452	7.72344	5.168225	5.427765
Mreg	2.824161	2.084759	2.579849	2.211293	2.837951	2.035889	2.482224	2.392467	5.425742
Haus2	7.670908	7.428733	6.912032	5.767017	5.364564	5.585952	6.013226	5.768672	5.421484
Cep43	5.342114	5.472869	4.852076	5.366396	4.79358	5.242682	7.004967	6.17612	5.420553
Zswim1	8.969431	7.922033	8.416234	7.837456	6.020722	4.745223	8.230887	6.097049	5.418719
Soat2	5.820997	5.321708	4.227644	1.68828	3.563906	3.236254	2.685844	3.265915	5.415833
Kat6b	7.473732	7.416094	7.862523	6.895644	5.60422	5.502933	9.914706	5.971805	5.409973
Myo19	8.758617	7.875004	8.660093	7.224972	7.172521	5.645242	8.983888	6.475408	5.407059
Map4k5	7.2593	5.68232	6.335444	5.889215	4.628951	3.708999	7.089611	6.257286	5.406396
Zbed4	5.957114	5.152592	6.137068	5.902776	4.950497	4.582668	5.764672	5.119639	5.406337
Gm2848	3.781587	4.224101	5.370477	5.86623	4.465912	3.497093	7.191564	4.464547	5.406282
Ube2n-ps1	8.637917	6.654912	6.993413	6.044485	5.78913	4.691409	6.707598	8.137116	5.40135
Fam118a	6.104613	5.476611	6.397443	7.11604	5.507968	4.618927	5.615084	5.728761	5.400661
Aplf	6.716951	5.896415	6.593903	4.401584	4.480954	3.664001	7.422187	4.3617	5.398796
Gm11914	7.893192	7.038653	8.358468	5.632796	5.837032	7.041751	4.798828	4.066991	5.398796
Zfp280c	5.568312	4.942468	6.372306	5.103899	4.901246	3.651164	6.289835	5.988581	5.397927
C2cd2	6.047634	5.828509	5.971991	5.735422	4.661076	5.184783	6.969387	5.410688	5.392066
Mir1249	2.262778	5.641885	4.526082	8.437886	8.62018	3.138819	6.431409	2.692983	5.391571
Snord13	0.565695	3.949319	3.394562	3.375155	5.387613	0.523137	0	4.84737	5.391571
2010320M18Rik	4.911291	4.898216	5.87935	6.585704	7.512285	7.776127	4.691514	7.226593	5.390124
Rec114	6.875478	4.908292	5.42864	6.189252	5.789925	3.61415	11.59424	3.445331	5.38031
Ifitm5	7.036072	6.246204	5.954291	2.998568	5.522867	5.577205	8.151181	4.416943	5.37954
Tmed1	6.047051	6.516666	6.331876	6.134249	5.913753	4.841503	6.459658	5.139142	5.376573
Crebl2	4.415808	5.668581	6.143496	4.73888	5.288188	4.750724	4.925459	4.76624	5.375414
Dagla	8.668962	9.16601	9.860738	6.125281	5.875883	5.368789	7.485149	5.78977	5.373772
H2ac11	5.285409	4.802774	4.228818	6.890941	6.040518	6.95151	6.919462	3.913954	5.373295
Gm11451	2.629176	4.420241	1.577689	1.867464	4.650284	1.597764	2.717385	5.149509	5.369654
Kctd21	7.813168	8.108525	9.567115	7.75086	8.844166	6.966708	9.019666	6.941791	5.36727
Gm39876	5.522727	4.561989	4.258475	6.246389	5.440465	5.146232	9.629551	6.201438	5.364101
Slco4a1	6.941726	5.228244	4.16552	2.78494	2.074557	1.372443	2.894588	4.831556	5.361308
Ets1	3.246532	2.93287	2.988098	5.474153	4.032993	6.428819	2.662479	3.023821	5.358853
Csnk2a3	8.354808	6.998534	7.265	6.11846	5.526803	4.224707	5.93398	5.544706	5.354347
Zfp407	5.201042	4.830663	5.573193	5.130087	4.679709	3.681866	5.665323	4.724995	5.351215

Rictor	6.201729	5.641397	5.998698	5.243431	4.862128	4.122669	7.202505	4.506934	5.351129
Abcg2	6.650489	7.047333	8.52193	4.649932	6.729719	5.861875	6.766212	5.639364	5.348115
Rpl19-ps1	4.817268	4.09559	5.133751	6.125281	5.716088	5.331224	6.284824	6.466228	5.343953
Nr2c2	6.562385	6.504761	6.865133	6.78585	5.291363	3.881354	6.865774	4.23563	5.343215
Adgrl2	7.478577	6.582894	7.347714	7.771409	4.432407	6.0227	4.689119	5.517914	5.342324
Tmem18	7.290536	7.215051	7.628774	7.193788	7.371813	4.228953	6.857165	6.031263	5.3409
Micall2	9.611166	8.322914	8.48877	5.177625	4.941909	4.600708	4.496852	5.908105	5.336424
Dclre1b	5.67561	6.679926	7.021357	4.587273	4.470333	4.701376	4.545475	5.288491	5.33245
Nkg7	1.36379	0.816096	2.318712	5.356795	5.97475	8.891391	3.030522	2.596923	5.329233
Gm33887	3.117	3.158046	3.34003	2.213957	3.675405	3.248536	3.093734	3.20322	5.328537
Tmem144	7.781228	7.709624	8.139345	8.828522	6.463152	7.502544	10.99552	5.878213	5.325076
Slc9a4	5.996482	8.677614	9.11336	4.23833	4.703163	4.920147	4.162156	5.737225	5.322962
Cep78	6.78313	6.434448	6.50338	5.147602	5.464437	5.706857	6.957991	6.433926	5.322627
Ccne1	12.04559	10.51537	9.722631	7.32779	4.102959	5.373115	6.678909	4.12869	5.321558
Ybx1-ps2	6.078589	5.731127	4.052864	5.152723	5.251389	6.972856	5.286934	5.502786	5.318564
Trmt10b	6.06766	6.765659	5.842215	5.621434	6.065917	5.192957	6.388085	6.243474	5.316067
Cdh15	1.754986	3.461732	5.577604	2.326874	1.281429	2.97542	1.713095	3.861679	5.315334
Pdss2	5.435104	6.679621	5.365597	5.404659	5.510291	5.058641	6.088593	4.640584	5.313818
Loxl3	4.685674	4.279238	5.081278	9.657475	6.110109	5.617998	6.616723	5.991524	5.309704
Dcstamp	10.01122	13.81323	10.35115	4.303914	6.523656	5.951611	7.857568	6.700829	5.308849
Nsun6	5.475081	6.168772	6.117224	6.606191	4.821691	4.936075	6.984674	5.932575	5.305573
Cul7	6.086255	6.060052	7.821797	7.109048	5.748736	6.268177	7.036597	5.021447	5.303808
Gm8615	5.772002	6.024385	6.336515	5.846454	5.701738	4.692269	7.158782	4.95874	5.296533
Pramel12	6.616005	6.005115	7.313277	6.102838	5.679201	5.917016	6.972932	5.677465	5.296185
Gm9774	7.353612	3.911485	7.517899	6.134308	4.474458	4.911393	5.066883	5.484398	5.295421
Ptk2	8.323043	10.25726	8.085657	7.164391	4.81892	6.137167	6.40434	6.283627	5.294223
Zdhhc17	6.072023	6.498969	6.134443	5.424391	6.441148	4.998912	8.278363	5.957416	5.29315
Rab2b	6.054738	6.760845	7.845921	6.74092	7.778024	5.301598	7.650822	7.201092	5.291409
Hivep2	9.471713	8.743528	10.43408	7.211702	7.090114	5.764012	8.994769	6.48342	5.290957
Ercc4	5.764632	5.551318	5.632957	5.559631	4.781255	4.214281	7.026549	5.181391	5.289258
Gpat3	5.78082	2.594443	4.374662	2.740089	2.881259	3.492669	1.991589	6.421225	5.289249
LOC118567481	8.622043	7.206148	9.274896	6.09236	7.555326	5.338559	8.102943	6.897004	5.289064
Zfp946	4.533755	5.780888	5.490881	5.021969	4.354336	5.112586	6.386217	4.626207	5.287386
Harbi1	4.648894	4.018315	4.261982	5.085139	5.128586	3.976715	5.765837	4.352433	5.280048
LOC115487194	4.951209	4.50595	3.837633	4.615757	3.345041	2.518297	5.996861	3.609184	5.279316
Capn6	2.573799	6.798519	7.737894	2.730007	3.149495	4.125625	2.095875	5.762408	5.277793
Emcn	6.268327	3.815936	4.111524	12.74976	5.969886	6.700143	2.944831	6.510613	5.275981
Coq3	5.306571	5.686783	5.473688	5.401008	4.360245	5.080552	4.9469	4.240033	5.275806
Srp54b	0.320549	1.918172	2.210356	1.090471	2.474432	0.436849	3.853579	0.562199	5.274092
Rhoj	6.935636	6.846827	6.959955	7.270848	6.426293	6.979148	3.887905	6.558636	5.265814
Il1rl2	7.0772	6.988335	8.201518	5.585362	6.726874	6.025346	8.490206	6.45743	5.25965
Gm14760	3.999263	5.684924	3.31012	3.519751	4.465554	4.251027	6.128891	3.676391	5.257453
Rpl10-ps2	4.558568	2.566534	2.573684	3.875012	2.380839	4.147627	2.58373	3.570167	5.255709
Amigo1	6.806182	7.660271	6.274584	5.784987	3.295386	5.415074	6.130651	6.045729	5.254748
Tkfc	7.200068	7.03385	8.724645	7.892815	6.389193	6.794746	7.644814	4.562314	5.246267
Ppip5k1	8.345515	8.373894	6.507214	6.419563	5.882617	4.926173	8.691049	5.591005	5.246267
Rac3	9.925368	12.1557	6.326228	8.131054	7.395723	9.701806	11.58717	5.337003	5.244528
Bmf	5.514218	6.706753	8.003271	8.385482	9.248082	8.233627	9.049248	8.156825	5.242721
Fam234b	5.790461	5.104141	6.041776	7.004068	5.225843	4.635167	5.725711	4.32005	5.242254
Chst15	3.317299	2.619204	3.340724	4.111943	4.684189	4.931809	4.964319	4.869191	5.236534
Tmem218	5.717576	5.478732	6.727341	6.354437	5.819057	4.406196	4.171341	4.429671	5.235657
Lss	8.572618	6.068466	6.707433	5.727884	5.89799	5.451849	8.468925	5.201004	5.232191
Atp23	6.575352	8.483086	6.033135	6.358561	7.009173	8.20055	8.417008	7.581378	5.231995
Bmp1	4.387185	5.521092	5.329055	7.932018	3.190707	5.975048	3.363681	5.847842	5.230522

Mxra7	4.301929	5.221428	4.424196	5.286754	4.406326	4.954096	2.307021	4.289059	5.221851
Gm8818	2.531229	2.69003	1.618515	3.548628	2.371195	2.571044	5.017864	3.713737	5.220462
Gstp-ps	5.273255	5.096817	5.587042	5.604922	5.702154	5.629451	4.867612	5.199538	5.216887
Gm11249	7.576134	7.256125	7.39661	6.816202	7.158162	7.84024	6.338825	7.442214	5.214971
Tnfaip6	4.155258	5.421989	3.012912	2.444756	4.88082	2.43368	3.113633	4.780424	5.214434
6530402F18Rik	6.006221	4.830831	6.019837	4.020251	4.034935	5.148239	4.859383	5.620128	5.207414
Klhl7	5.795268	5.945926	6.462141	5.613767	4.313974	4.358267	4.819297	5.914047	5.205968
Itpkc	4.930872	3.688308	4.588983	3.268834	3.782976	3.847429	6.104945	4.580566	5.205414
Helq	8.94881	8.110257	8.652759	6.683243	8.558113	5.665853	9.095531	6.151473	5.202343
Acsf2	6.984623	6.147615	10.32547	8.40671	8.851263	7.235672	8.915215	6.86808	5.201892
Mbd4	6.305141	6.716135	7.828721	6.54706	6.781097	5.334981	6.339354	4.124588	5.19176
Poc5	6.379124	6.544438	6.087379	5.525476	4.334604	6.000634	6.857625	4.768347	5.191409
Gm12537	7.016308	6.261036	5.632165	7.068808	5.275464	5.762778	5.295652	4.219082	5.191351
Gm5436	5.598234	5.971061	5.210073	5.025651	4.369029	6.471339	3.777007	3.553378	5.187402
Strbp	5.729686	6.364233	5.404546	5.48798	4.416613	5.493567	4.436486	5.619426	5.186941
Garre1	6.752472	5.755903	6.681888	5.002723	5.437024	4.677169	7.255596	4.84087	5.185972
Rtn2	3.593208	4.19798	6.263176	4.747092	3.128806	5.981195	2.440471	5.864719	5.185893
Eri2	6.837851	6.782982	5.882706	7.640353	5.636986	4.827565	7.181249	6.790318	5.185633
Dlg4	7.035574	8.780435	7.751319	7.912773	6.378114	5.566869	7.602145	5.5881	5.181543
4930438A08Rik	0.247019	0.105583	0.14117	0.421089	0.403299	2.675955	0.437668	0.33598	5.179478
Smim5	5.956643	6.76426	6.37022	4.750351	5.27986	4.85404	6.583175	3.874458	5.17132
Trim24	6.975726	6.54454	6.35589	5.519609	6.063521	5.016212	6.330812	6.214851	5.169828
Ythdc2	6.691413	6.236567	6.230252	4.640086	4.139519	3.756615	5.765298	6.190467	5.169735
Isoc2b	5.422086	7.559675	6.418725	5.831854	6.797425	6.242137	3.831304	4.161492	5.16773
Gm13370	1.904744	2.699535	3.208362	2.791267	6.683368	6.58225	5.284264	4.772375	5.159529
Lrrc32	9.182992	9.893489	5.527443	8.37059	6.140942	7.915516	9.373907	7.353386	5.15948
Spc24	8.923053	11.08106	9.269538	6.354263	7.182365	10.27477	9.579417	6.248392	5.157545
Ctps	7.849456	5.580147	5.944143	4.605849	4.58694	4.510772	4.003447	5.853878	5.156774
Abcc10	6.850352	6.832115	6.88549	6.777834	7.390824	5.255344	9.688669	6.783749	5.154469
Dbt	6.786507	6.929168	7.360366	8.262006	6.412234	5.61358	7.458364	5.881767	5.154036
Ddx11	6.064328	6.728603	6.545181	6.558015	4.812999	4.322911	8.383163	3.921396	5.153693
Gm2225	6.210903	6.997342	5.406414	5.146761	6.681981	5.956377	6.894781	7.665504	5.152194
Pgam1-ps2	7.001693	4.878571	5.052037	5.690788	4.6587	5.35144	5.320057	5.296511	5.149666
Mob3b	6.818402	6.014291	6.876307	5.871638	5.895518	3.823402	6.917312	5.969834	5.148762
Rpl7-ps8	5.711424	6.439204	6.146703	5.247301	5.025623	8.898594	4.234791	4.137484	5.147652
Acad11	6.047185	6.942376	5.49959	4.807345	4.240343	4.731901	6.267238	4.159961	5.145986
Rcan3	7.14058	7.755084	6.232304	6.860992	5.726281	4.577807	8.715817	4.567112	5.145958
Rab34	5.237608	5.826394	5.157628	6.610495	5.141725	5.290675	3.872612	3.375623	5.145502
Sinhcaf	5.46209	3.924808	4.150811	3.67802	4.97675	3.718775	4.756312	4.29958	5.14437
Tnc	7.258345	5.04775	8.711026	8.709993	4.717497	6.512736	4.632907	5.154976	5.141644
Ppat	7.109572	6.621613	6.252724	4.016277	4.38671	3.504806	5.375262	5.188696	5.141364
LOC115490142	2.762039	4.525555	3.551605	4.185245	3.945803	2.554247	5.369564	4.195048	5.139581
Lins1	4.542064	4.964352	5.330804	3.712291	4.286306	3.337271	6.537932	4.284997	5.13944
Mtbp	7.337831	7.557261	7.907806	8.071075	5.134386	8.032167	6.77918	5.104301	5.138159
Pcyox1l	7.558487	7.291654	8.548955	5.520952	4.711824	5.617299	7.556405	5.67863	5.134422
1110051M20Rik	8.26708	5.596202	7.856509	5.058936	5.522121	6.641928	6.34067	6.197132	5.133998
Ccnf	8.067947	9.830607	10.62731	6.421761	5.843795	6.997775	7.76546	4.888212	5.131996
Als2cl	4.648773	6.175449	5.401692	7.039597	5.456223	5.958005	7.874987	4.591377	5.129309
Myl4	3.825551	7.954068	7.327753	6.51214	7.039814	6.295995	5.763308	7.284598	5.129245
Esr1	5.956715	6.31216	6.465141	4.644443	5.125139	2.848188	4.897265	4.833559	5.128905
Zfp87	3.334467	4.61094	7.365322	5.49241	5.513655	4.975396	5.32808	6.154709	5.127762
Gm9320	4.171577	2.627666	2.525195	3.165739	4.182068	3.147107	4.538456	3.344632	5.126797
Abcb6	7.05813	6.462039	6.274622	5.700286	4.712573	4.196017	5.924718	4.391132	5.125352
Zc3h8	4.316173	4.022408	4.068997	4.678979	2.863996	4.024179	5.009462	3.199958	5.125261

Gm4613	5.221313	5.66689	6.143436	6.413729	5.265235	8.94694	7.142411	6.43332	5.122739
Dtwd1	4.854623	5.403682	4.681788	5.775675	4.417084	6.052678	5.73428	4.869688	5.122625
Pfkm	5.748549	7.166556	5.946108	7.047553	6.187336	7.209476	6.043149	4.760919	5.122376
Cep55	9.483428	11.874	11.40429	8.362296	7.595512	8.53752	8.998574	5.809118	5.118215
Fut7	4.609315	6.171374	5.588655	4.709071	5.442225	3.094728	4.633438	5.380445	5.115238
Cbarp	3.89246	2.646884	3.36206	2.844346	4.128401	3.572355	3.931618	3.284866	5.11511
Zfp729b	5.067968	4.517009	6.077681	4.412482	4.990478	3.541532	5.309102	5.665791	5.114354
Snord49a	8.047461	7.134254	11.62546	7.113229	4.257952	4.96136	17.55907	5.107981	5.113297
LOC115486842	7.749407	5.945212	2.980887	0	5.109542	2.756311	7.393291	9.648408	5.113297
Pigg	6.102255	5.993797	6.620792	5.258997	4.438041	3.659513	4.758907	4.084567	5.111023
Shq1	4.898408	5.248469	5.892013	4.21273	5.138013	4.438072	4.925918	5.136443	5.110244
BE692007	4.192514	5.464078	7.600859	6.627262	5.011015	5.267303	6.404809	3.943975	5.110215
Akap9	7.524555	6.901603	6.513184	6.066077	5.347467	4.014992	6.906054	5.075489	5.10696
Rab19	3.452478	3.878644	5.675422	4.61698	3.477073	4.660671	3.650338	4.042752	5.105976
Phlpp2	6.183328	4.629953	5.548304	6.416859	4.017684	4.382132	5.614328	6.088874	5.103821
Dnajb6-ps	7.223081	5.265759	6.380519	7.036781	7.402979	6.204981	6.139072	6.458172	5.102024
Fbxo10	4.204196	4.497949	6.383476	4.503696	3.658228	6.326689	5.530314	5.058093	5.099783
Gm39487	0.364724	4.72879	4.498787	3.626811	1.852583	2.92314	1.507842	3.35678	5.098345
Ck-ps3	6.465778	5.333998	4.909773	2.500384	3.421076	3.174223	5.940182	5.776051	5.097344
Nid1	8.509183	9.68452	7.822389	12.7561	7.309367	10.16699	5.591436	6.626182	5.094972
Eps8l1	4.289325	3.457486	2.791324	6.470984	4.67269	4.79528	3.886669	1.664312	5.082135
Mettl4	5.718588	6.329549	7.789724	5.686555	5.607933	4.660671	5.594755	5.977813	5.078342
Lrrc27	7.506121	8.377344	9.508858	5.402569	5.310498	6.313392	6.945213	6.976406	5.075933
Ubn2	7.88733	8.058561	6.727877	7.343322	5.485382	4.249856	9.093309	5.346973	5.075185
Gm18679	7.785491	7.095387	4.385441	2.669614	3.536952	1.489609	2.312268	3.152464	5.074778
Muc3a	3.372753	3.637859	2.048873	3.080583	2.64112	1.940721	2.866191	2.545166	5.071819
Rab3a	4.743584	6.047395	5.115416	5.947538	6.560541	7.209476	4.604304	6.951263	5.071447
Zfp12	4.701042	4.570329	4.714758	5.460375	4.615175	4.919721	6.355859	5.26571	5.070382
Zmym4	7.697025	7.360726	6.423053	5.538588	6.085604	4.835412	7.485328	5.132917	5.068614
Mapk8	5.030768	5.551402	6.214467	4.247246	3.700172	4.203189	5.765837	4.837207	5.067738
Cemip2	6.515229	5.178262	5.268066	4.338586	4.44242	3.446221	4.639234	4.25768	5.060258
Gm2606	3.738953	3.86711	2.031272	3.304897	2.98943	3.927227	3.912104	3.471953	5.059368
Rad51c	6.404164	7.67744	4.269939	4.502832	3.758127	5.324151	6.017293	5.851032	5.05562
Ift172	6.991265	7.248695	7.699487	6.279319	6.404564	6.095586	8.507821	6.285486	5.051208
Thumpd2	5.910676	6.708035	6.115203	6.53626	4.513116	4.240868	8.162859	7.325508	5.050634
Pigf	5.987081	6.373045	7.254421	4.465158	5.263412	4.631633	6.187947	6.138771	5.04781
Gm10819	5.479343	6.153591	6.769387	5.677586	5.788551	5.705838	6.37702	5.041515	5.046761
Hmga2	9.008499	6.232636	6.175417	5.057435	2.130694	3.779194	4.917424	3.720475	5.046348
Slc20a2	7.77764	6.308708	7.221513	5.736269	5.664436	4.488425	6.393857	5.4165	5.042967
Tacc2	9.212304	10.45352	8.765399	6.85939	5.147615	4.735985	8.178816	5.743097	5.037554
Deptor	6.986878	8.996013	8.338864	6.361628	6.212714	4.648423	8.330148	5.933378	5.036942
Col11a2	4.094181	4.961661	4.546973	3.952897	6.460971	4.20441	4.871191	4.328661	5.036454
Cenpm	12.18651	9.905917	10.21532	7.214911	7.245552	11.92113	9.246311	6.639726	5.03533
Phospho1	3.976447	4.023757	4.441366	3.607822	4.450563	2.898889	3.779868	4.532107	5.034768
Itfg2	5.835586	4.785909	4.099855	4.987614	5.534782	4.972869	7.078135	3.948928	5.033228
Cped1	4.573946	4.012526	5.018156	6.115139	4.210485	4.767414	4.774834	4.252892	5.03005
Dusp4	6.167399	4.168997	4.294836	2.407712	1.370545	1.373045	3.257983	2.196551	5.029023
Prpf40b	5.364201	6.926236	6.46653	6.286681	5.473721	4.78348	6.435197	3.898835	5.021265
Kif18b	8.422864	9.179179	8.80599	5.74899	5.775095	5.776607	7.108593	3.400728	5.019315
Hlcs	6.225298	6.044617	5.691187	7.553957	6.307624	5.680878	6.363296	6.109868	5.018442
Pgap3	5.6143	6.478175	6.697656	4.531372	5.538818	5.42475	4.007217	4.626256	5.014994
Dph7	5.956874	6.114476	7.349115	6.226168	5.921726	5.347892	6.156727	4.222598	5.014373
Trmu	5.179615	6.780145	5.706332	7.283272	6.436034	7.446868	6.733561	5.740575	5.013768
Prr33	5.506765	6.107812	7.754822	5.844254	5.315138	3.699473	5.053454	4.81978	5.013081

Hotairm1	2.629473	1.311236	3.506372	4.357907	5.425944	1.215827	4.303012	2.920769	5.012243
Igf1r	4.376287	4.766508	3.581014	3.748205	3.716159	2.820673	5.224245	3.593617	5.011994
Ahi1	9.409458	9.43788	10.24168	5.651371	5.182842	4.264018	6.84362	4.874977	5.0078
Slc38a9	7.267162	7.508916	7.213835	6.445459	6.878177	4.207582	9.003839	6.257228	5.00755
Tnfsf13b	3.132463	4.979071	5.482447	2.011717	2.983323	4.37538	2.326994	2.143608	5.006958
Rnf157	8.110273	7.684841	7.789626	6.584512	4.65036	5.649875	7.274569	5.828182	5.005648
Mfap5	6.830694	8.092206	6.793745	8.556212	8.446339	9.038974	6.903844	6.934661	4.99959
Mras	6.356818	7.374061	6.20726	5.439528	6.454912	6.114841	7.455419	6.66756	4.998713
Lrp4	5.51648	5.297393	6.029547	5.672395	5.562884	4.232797	7.766828	5.04084	4.997252
Tex9	2.925242	3.435068	4.482747	3.659529	3.190384	3.141491	3.023384	3.279251	4.991448
Cul9	5.42083	5.871617	5.463871	6.493859	5.84931	5.124124	8.334654	5.349575	4.991396
Dcun1d3	5.931478	4.845614	5.081823	4.670289	4.665789	4.511754	5.387707	5.300412	4.987573
Espn	5.923925	9.206873	9.627497	6.283458	9.826276	5.249999	11.12285	9.964277	4.987323
Mfsd8	4.978661	4.773692	4.94079	4.931658	4.247322	4.106368	4.649006	4.246024	4.983278
Ccdc171	5.552093	6.570392	4.889725	6.30382	4.774762	4.444706	6.337896	5.246688	4.975719
Gm2296	5.106137	3.525607	4.377198	4.742753	2.244475	4.566326	3.421642	4.487578	4.973561
Pitpnm2	6.642199	7.141551	4.828306	9.472526	6.850549	8.509607	6.329236	3.948659	4.971842
Pus3	5.071993	6.04666	5.001802	4.504036	4.852978	4.930395	4.754515	4.581968	4.968964
Tmem150a	5.984374	6.151523	4.075722	7.082609	6.818363	4.821171	8.120379	5.977352	4.968815
Gca	1.922123	2.270138	3.119597	2.531708	2.232058	3.305561	2.840503	2.712968	4.965558
Fbxo45	6.248086	7.857642	6.605104	5.054946	4.710899	5.461256	6.188638	4.918189	4.962484
Zbtb44	5.213892	6.457081	6.2202	4.999763	4.965653	3.987251	6.449496	6.013359	4.956173
Fbxl4	8.050035	6.482017	6.363232	6.576299	7.905673	4.871561	6.693802	5.167514	4.955544
Pdk1	6.726571	5.69543	6.13911	5.136303	5.550779	4.52941	7.526995	4.327266	4.953506
Itga7	2.136474	4.124103	5.734688	5.9075	2.139774	4.10445	2.493076	3.871414	4.952685
Casp8ap2	5.14191	6.416591	4.982068	5.583278	4.478956	4.356869	6.509947	4.051533	4.94898
Gm26797	2.824451	1.703261	3.08755	4.702822	3.753455	2.247504	2.376102	2.06377	4.945681
Stat4	4.887161	6.269832	6.459554	3.361173	4.100862	3.981933	3.09298	3.259189	4.94517
Zbtb33	5.904579	5.944731	6.039731	5.448133	5.431384	4.755806	7.353321	5.387055	4.944162
Rnd1	2.994612	2.760759	2.164425	2.952813	3.738803	3.700188	2.054717	4.791872	4.940764
Ifi214	1.670854	0.940023	3.127859	4.558458	3.101	2.931829	1.328393	2.732941	4.940746
Cask	6.34329	6.62894	5.744008	4.960405	4.866408	4.388648	6.172912	4.332219	4.940657
Clmp	6.871159	10.0856	8.559807	4.834813	4.502998	4.508656	5.218977	4.96071	4.940341
Tst	2.716566	2.157432	2.31438	4.752373	3.25799	6.280487	3.015684	4.406522	4.938523
Gm18392	7.080874	4.219989	6.131713	4.8945	6.08582	4.791344	11.2454	5.426235	4.938075
Haus6	5.754331	6.00341	6.191693	5.722098	4.381319	4.326362	6.205269	4.068185	4.934463
Cdan1	4.706678	5.724781	5.21095	5.492028	5.24347	4.705935	7.449459	4.489486	4.932815
H4c3	3.843706	3.980914	0.73926	4.116189	3.519907	4.374817	7.028555	3.659585	4.931491
Gm13680	4.250251	3.870333	4.127535	5.4515	4.517214	4.84192	3.756203	5.102305	4.931491
Cd34	5.847422	5.248669	4.700974	11.94243	8.374951	8.090472	4.346745	4.625158	4.930062
Znhit3	4.61058	6.925159	4.94445	5.855228	6.295625	5.958934	5.454219	5.288825	4.923726
Slc27a3	4.140804	5.077201	5.066985	3.899618	5.822693	5.000597	7.325887	4.823705	4.921585
Lztfl1	4.509154	6.058074	5.897272	3.987555	4.221955	3.966514	4.310685	5.573855	4.918482
Tfam	5.100592	5.144985	6.205715	4.840818	5.82653	4.891567	4.926277	4.883935	4.916717
Lin9	5.606907	6.239282	7.194263	7.422047	6.19847	6.235448	7.93787	4.188267	4.914308
Ahdc1	5.667198	5.467516	5.932298	4.65385	5.128767	5.102287	7.670099	5.227898	4.91413
Gm15708	3.453107	4.25793	3.704673	4.307813	3.468085	4.035202	5.093877	4.991321	4.906757
Capn5	7.075273	9.352681	9.624525	5.13203	4.755077	4.904253	6.149576	5.800407	4.906506
Lyve1	17.67128	29.0556	18.82178	4.546382	7.513725	7.669459	11.91236	6.802804	4.904737
AW209491	4.964603	5.890441	5.421748	4.823304	4.429322	4.854966	5.249122	5.460255	4.89487
Ifit1bl2	0.723555	0.584175	1.137146	1.627444	1.591505	1.561281	0.160249	1.262974	4.89296
Dennd2d	4.059272	4.655735	4.059744	4.555517	4.363065	4.129277	5.364196	4.085672	4.891328
Zbtb43	4.71908	5.217129	5.164877	5.566984	3.7736	4.034695	6.142769	4.726156	4.890191
Lipg	3.721935	1.809223	2.971638	2.674735	1.995772	0.954484	0.921294	2.397173	4.888764

Tmem265	5.761566	5.022293	5.85298	5.052605	5.011979	5.663745	6.611308	4.708093	4.885946
Mustn1	5.454466	7.315788	5.502127	10.28673	5.597816	9.262047	4.033702	6.446714	4.884882
Melk	7.374894	9.234173	9.282876	6.282664	5.579629	5.162857	6.672499	4.506087	4.883025
Tsacc	3.073568	3.633049	4.326265	5.546712	1.843072	5.15832	3.411996	5.419145	4.882306
Cep57l1	6.184839	8.51364	5.251281	5.349388	5.614262	4.915234	6.958328	3.987055	4.881549
S1pr4	1.71434	1.663566	1.506015	2.60318	2.912418	3.984845	2.059184	2.801243	4.879676
Smad5	7.416998	8.068966	6.853732	7.082443	5.171617	5.325268	6.647787	7.14988	4.878533
N4bp2	6.655276	6.087644	6.994657	6.252467	4.91964	4.258118	6.968541	5.212856	4.877156
Pop1	6.328269	5.020907	4.893352	4.664327	4.062912	4.823846	5.746518	6.005883	4.875223
Col14a1	5.378452	7.376792	7.272868	6.0025	4.724442	5.376663	5.430263	4.494466	4.871954
Pdia5	7.096593	6.882901	6.250937	7.02447	7.099754	6.050936	4.878574	5.268949	4.871093
Fam160a2	5.26157	6.017205	5.100268	5.956542	5.504459	4.236933	7.35119	4.449491	4.870731
Pkd1l3	3.347679	2.824144	1.749554	2.553011	3.691707	1.862152	4.812772	3.582737	4.869917
Prps1l3	6.102892	6.210862	6.43577	5.43569	5.061074	4.351201	4.863036	6.008189	4.86695
Pxdc1	4.51102	3.907035	5.283256	2.449456	6.105192	4.638216	4.693038	3.616786	4.865114
Gdap10	3.104057	2.109872	3.875579	4.38425	3.765949	1.864933	6.743346	3.237726	4.861644
Gm10053	3.015831	4.202076	3.63716	4.983528	3.252394	4.30646	2.979496	5.447151	4.861041
Il2rb	2.117212	1.640058	2.919214	7.092789	6.029651	8.516051	3.250499	3.953303	4.858611
Aph1b	4.37861	4.985604	4.610239	3.168805	2.89593	3.171874	3.859256	3.879361	4.857146
Dab2ip	4.765257	4.150132	3.946897	4.484951	4.627868	6.169465	2.663733	3.757396	4.848759
Nbl1	4.050121	4.944151	4.763496	7.346041	4.505316	5.663083	2.946946	5.768744	4.848318
Gm12168	2.833662	4.058016	2.543326	2.601037	3.044743	3.359593	4.130262	2.697924	4.847468
Gtf3c4	7.110782	7.189222	7.591682	6.08393	5.052053	4.574489	7.458663	5.383595	4.846401
Gm6712	2.430767	2.727333	3.757077	3.543324	2.999024	3.857187	2.912006	4.86535	4.844086
Cyren	5.080491	5.608716	5.304777	3.93995	6.421041	4.107301	5.614214	4.350371	4.842159
LOC115487282	3.385531	3.798582	6.771848	2.104104	3.224342	2.348124	6.123451	2.417518	4.840067
Gapdh-ps16	8.354945	6.951591	5.216677	8.721257	5.539237	5.762778	4.580024	6.76811	4.839395
Ptrh2	5.602083	6.374622	6.335978	5.701558	5.926191	5.841248	6.178625	3.919756	4.837605
Ercc5	5.502331	4.936157	6.360218	5.14157	4.884858	3.84825	6.101309	5.989033	4.835742
Cox20	3.217388	3.825903	3.836562	1.230525	6.246263	3.54752	4.604304	6.362173	4.835565
Ift43	5.429501	5.628798	5.001437	3.694112	3.946287	6.276291	4.282583	4.353193	4.83435
Cenpk	3.208091	6.17457	4.953418	2.238681	2.465722	5.256859	3.315722	2.143451	4.827783
Fkbp9	4.621373	7.583847	5.634679	6.078492	5.190431	6.282851	5.004774	5.592032	4.825734
Zfp367	5.601875	6.489732	5.158423	4.364687	3.318212	4.232797	4.713052	3.935107	4.818198
Pced1b	4.920696	4.34749	4.600316	4.494236	5.9599	3.9075	5.887349	4.786833	4.817303
Ddr1	8.705318	7.528571	8.198968	7.385313	8.928607	9.758067	7.067872	7.225711	4.815707
Gm10240	3.938223	3.625605	2.625787	3.61492	5.674167	4.015478	2.087359	5.768576	4.812149
Gm16599	6.127365	4.489753	5.502763	6.963477	6.312014	2.659736	7.883861	4.365467	4.807011
Klhl26	4.202886	5.149798	5.744611	5.174425	4.860521	5.58944	4.447318	4.916201	4.806868
Gm15931	5.142958	9.202507	9.385729	6.806332	6.458765	5.303925	8.800546	8.857086	4.805584
Piga	4.971021	5.523901	4.23188	5.131324	4.472237	4.103936	6.257803	4.159711	4.8034
Gm16894	7.365896	8.312903	6.07434	4.690337	3.384526	1.852318	3.205483	2.276167	4.8034
Gm22270	4.199853	1.675469	5.880478	2.505797	2.799926	1.165168	1.736303	5.198273	4.8034
Mir6516	3.527877	1.507922	0.504041	0	0.959975	1.864269	1.562673	2.399203	4.8034
Polr1b	6.101238	6.071165	4.964766	4.72955	4.186383	4.347084	6.062295	5.096056	4.797393
Mfap2	5.211358	6.810496	4.762656	10.81107	5.733398	9.056961	5.479064	6.159381	4.795615
2610301B20Rik	6.040657	6.754826	5.858287	5.20071	3.611225	4.546353	5.32439	5.15376	4.785274
Gm12696	4.577455	4.97107	4.544076	3.944905	3.778249	5.173773	4.555805	4.648731	4.782835
Steap4	5.720361	3.69777	2.59565	5.42497	4.79223	4.930812	2.572936	3.916661	4.778924
Ager	3.206798	3.548754	3.031435	4.630363	6.359261	5.037366	5.856928	4.642515	4.77295
LOC115487884	4.168275	6.651485	3.335008	3.730434	11.1155	13.10595	12.06274	9.524656	4.767284
6330537M06Rik	5.22453	6.336372	5.483168	4.906638	5.743653	5.368313	9.066351	7.36928	4.76428
Fry	5.69688	5.020683	5.597586	6.365898	4.198238	3.818664	7.040603	4.802959	4.762836
Dop1a	6.300375	6.605614	5.952015	5.90931	4.554326	3.808942	6.647038	4.461544	4.757279

Dclk1	2.76192	4.148445	3.793922	5.195091	2.915649	3.631183	3.680004	5.671106	4.757269
Lancl1	4.742875	6.306997	6.298433	4.521418	6.15963	5.437267	4.915502	5.386474	4.756985
Acot2	6.098187	7.238025	5.368036	5.638042	5.615852	4.054784	6.667404	4.558486	4.755366
Abcb10	6.033875	5.84168	6.991277	4.785426	4.83555	3.851518	6.099416	4.89012	4.754945
Gm10420	5.86026	5.201746	5.626504	4.603673	5.581248	5.527773	7.08654	6.416474	4.747546
Zfp160	4.235258	4.704642	4.133508	5.024795	5.132425	4.26778	5.388669	4.574666	4.746458
Gm6206	7.832726	10.13659	6.426522	6.870059	6.939817	9.263084	7.726549	8.957025	4.743357
Nubpl	7.031169	6.796682	4.976482	4.463985	4.842018	4.051374	5.198746	4.068112	4.742479
Tgfb1i1	5.512127	4.360049	5.924642	6.615303	4.103206	6.79659	4.289165	4.976633	4.740269
Gm11223	9.430186	8.151069	10.23151	6.706235	7.72928	9.196417	6.084221	5.550321	4.739024
Lix1l	6.717087	8.241784	8.264745	7.646225	6.691892	5.496583	7.193651	5.574872	4.738308
Prokr1	5.924467	4.707628	4.402335	5.423282	3.559456	2.931394	5.03593	4.6127	4.736236
Zfp329	4.644873	6.752859	5.200089	5.354195	3.807486	3.248288	5.923262	3.080261	4.735323
Pxylp1	4.202748	4.001033	3.802018	3.818274	2.874631	2.614601	3.711931	3.273895	4.733881
Bivm	5.144107	7.472168	6.937285	5.400466	4.984543	5.07204	5.668668	5.921603	4.731962
Tmem216	3.397469	3.22707	5.285566	3.056682	4.00612	4.48839	3.678666	2.567241	4.728639
Ammecr1	5.528506	6.266593	4.733441	4.026794	4.157829	3.037369	4.712458	4.360502	4.724743
Trdn	4.816674	4.942423	2.408617	5.019967	5.513639	6.295995	2.680608	2.42526	4.723143
Gm4737	5.376936	5.653206	4.476886	3.871833	3.657811	4.237581	5.858468	3.681911	4.72078
4933412E12Rik	4.329379	4.391037	5.504087	4.487548	5.20648	3.359015	4.209185	4.121993	4.720747
Letm2	4.235717	5.404799	7.008435	6.441868	5.012901	5.241946	4.892863	5.930615	4.719745
Gm14719	4.686152	2.380929	3.890843	4.308212	3.031499	3.434179	5.117525	4.209128	4.71913
Ifi44	3.16729	2.238396	5.475224	6.090517	4.275029	5.023932	3.663777	6.849938	4.717982
Zeb1	5.069817	4.628562	4.564465	5.096097	3.477313	4.281605	4.06917	3.947429	4.716689
Rbm12b2	3.133958	5.149038	4.619001	5.363493	4.131518	4.728619	5.199611	5.700695	4.716221
Pgk1-rs7	3.203229	3.194701	2.949851	3.595273	3.473592	2.199688	3.867943	3.744294	4.715466
Egr3	6.435427	6.432848	5.516721	3.729343	5.378536	4.34538	4.630294	4.571054	4.714893
Gm32031	2.729258	2.041494	3.582568	2.374724	4.71126	5.205611	5.465348	4.385005	4.714722
Ccdc126	4.07425	3.405743	3.862744	3.700148	3.633549	3.397499	3.48072	2.982187	4.713617
Gm44764	4.132656	3.417956	3.830711	5.813448	7.295807	5.872446	5.938157	6.525833	4.707332
Dgkg	3.860886	3.803361	3.115126	3.639053	2.92135	3.165194	3.194783	4.172082	4.706942
Tsen2	5.312554	3.771222	4.656838	5.003118	4.285794	4.017005	6.201362	3.897691	4.705924
Gm19710	3.378257	4.060394	2.858857	3.790017	2.309939	2.483264	5.28216	4.371048	4.705831
Gm9396	5.728894	5.537837	5.288824	5.433874	5.204314	5.705432	5.283441	6.293617	4.704125
Car9	4.904952	5.344846	6.419571	5.268879	4.844438	5.907929	5.601498	5.102401	4.703712
Mpp5	4.716974	5.378028	5.48017	3.932264	3.973642	3.334546	5.032857	3.360487	4.703349
Elk4	6.996127	6.619282	6.028614	7.857613	3.911559	3.567055	7.634354	5.545876	4.703273
Nthl1	3.419335	4.640151	4.148527	6.242955	4.003939	3.732307	2.433282	3.148803	4.701407
Rpsa-ps12	2.734373	4.759044	3.539011	2.98563	3.676499	3.619458	3.158594	2.399203	4.7012
Card6	3.756232	5.161746	6.473216	4.951866	4.696058	4.265682	5.361808	4.915957	4.699508
Cxcr3	5.995167	5.979206	6.650584	4.591105	5.578473	6.340714	4.700644	5.44555	4.695928
Rnf170	3.537978	4.855413	5.950927	4.274128	4.483426	3.555728	4.805054	4.440297	4.695729
Tecpr2	6.251109	5.166561	5.920013	5.968649	5.199427	5.006798	7.232392	5.190659	4.692984
Kif20b	5.846851	6.82002	8.123448	6.39663	3.701589	4.604793	5.204675	2.981818	4.692124
Nf1	5.877455	6.26784	6.144901	5.086046	3.979839	3.02922	6.432544	4.254854	4.6919
Opn3	6.25393	6.156799	5.487957	4.17268	4.88022	3.395438	5.337805	4.571407	4.69153
Csta2	2.6589	3.049594	3.456972	0.925402	2.405692	1.598264	0.961837	4.55676	4.68821
Phgdh	3.585252	5.348784	4.119073	4.036596	2.370434	4.411571	4.042403	3.244246	4.687872
H2-T10	2.219819	3.072753	5.30139	4.890498	4.374065	3.574737	5.518172	5.119736	4.687336
H2-Oa	3.699394	4.005792	3.488406	7.199686	9.059818	9.823398	5.62602	4.679475	4.684345
Tyms	7.118574	7.878849	6.858935	5.12774	5.059935	5.940787	5.99359	4.083079	4.683052
Dnajc18	6.402887	6.749524	6.832685	6.143056	5.260453	5.69307	6.218646	4.789123	4.681665
Gm6430	4.962684	4.308348	5.88905	4.142235	3.428481	3.495503	3.003096	4.431181	4.680864
Fbxo21	6.347978	7.679626	6.234838	5.675517	6.276188	6.067847	8.179125	5.46119	4.680078

Tm7sf2	4.273116	4.074822	6.372932	4.286454	4.426658	3.951644	5.423746	5.246128	4.679986
Gpr19	8.270268	7.79505	5.544451	7.86245	5.539526	5.546963	8.641666	6.403119	4.677409
Ccdc137	5.689059	6.076318	5.770425	5.677252	5.110782	6.32447	5.942479	4.993975	4.672303
Gpt	4.557827	4.236462	5.178542	5.642247	6.230708	6.107356	5.255522	4.634702	4.669077
Cox6b2	6.571322	7.626272	8.24498	8.67308	5.803295	9.612635	7.162251	5.232745	4.668822
Adal	3.278973	5.302466	5.434358	4.285367	4.818124	4.20189	5.059266	4.995046	4.665406
LOC115490074	1.630531	2.439285	9.784325	2.432097	3.88225	2.261796	6.740942	1.552426	4.662123
Cspg4	15.24719	17.0954	13.63039	4.581405	3.397475	3.814803	6.619521	3.809517	4.660193
Pxmp2	5.440506	6.731014	5.372263	3.90344	3.279416	4.01223	2.135329	3.475119	4.660193
Irgq	7.042915	5.471265	6.645374	5.662184	5.785085	4.888986	7.737663	4.783519	4.659078
Lims2	5.137118	4.696488	3.389465	10.25216	4.314944	6.63111	3.023447	5.230695	4.658123
Zdhhc24	5.399653	5.945736	7.330697	6.341245	6.817978	4.677104	7.676771	6.978731	4.657329
Gm14150	5.490329	4.017893	3.779497	4.892346	4.549848	3.923356	4.200625	4.20902	4.655128
Chac2	2.913283	3.375539	3.213553	2.385735	2.652171	2.733732	2.258268	3.507954	4.654918
Palb2	5.82274	7.16342	7.845921	5.062024	6.442094	4.997764	6.738822	4.780299	4.65235
Fam83f	5.143738	4.340806	4.594727	5.442805	6.154952	4.817752	7.270167	5.734495	4.651032
E2f3	6.22301	5.675152	6.211625	5.345408	4.750606	3.50441	5.868697	4.138219	4.650245
Gfod1	4.428634	4.416844	4.226673	2.952661	3.703615	4.321688	4.350533	3.790829	4.64699
Tspan7	6.968821	7.386607	4.875616	11.83968	6.160829	9.796868	3.778955	5.504372	4.646355
Cep97	3.587567	7.56109	6.363594	6.93301	3.674628	2.691694	6.926132	5.284692	4.645046
Rad51	6.496218	8.181013	6.111559	4.95436	4.037232	3.996514	3.556232	3.328388	4.643605
Atp5pb-ps	4.563311	4.428158	4.872209	4.108503	4.81589	4.56217	3.760381	5.049268	4.643108
Zfp182	3.4955	4.350695	4.136665	4.871909	4.450732	4.487203	5.65774	4.368637	4.642579
Slc35e3	6.175702	7.032351	7.370315	4.81159	5.260154	4.415795	5.82357	5.243392	4.642045
Pim2	4.943522	3.684225	4.482285	4.051508	4.294252	3.164963	4.828641	4.448106	4.633951
Osbpl3	4.881147	5.07057	4.830969	5.005172	4.233167	3.613023	5.695189	4.386462	4.632823
Npdc1	4.196507	5.947581	3.792021	7.211237	6.801414	5.446734	3.918788	4.416063	4.631166
Gm13092	4.619839	4.371255	4.264962	5.536311	6.656234	7.010924	6.978603	5.413587	4.628917
Gm10163	3.641917	2.118795	2.731755	1.609563	3.08313	3.742145	1.777493	3.756417	4.628093
Tmem238	9.230886	6.944189	5.750191	6.609009	7.033116	9.02423	5.506278	7.834505	4.625158
Rida	6.636951	9.344868	7.753306	5.102346	6.639664	4.8998	6.11025	6.000422	4.624601
Zfp652os	1.651786	2.368121	3.71695	2.669117	0.589929	2.10034	3.947907	3.047033	4.624502
Paqr3	5.455906	6.469197	5.880937	5.334922	5.340482	5.129541	6.798068	5.714013	4.622189
Brca1	8.390715	8.23685	7.831145	5.966821	3.485367	3.948336	5.963218	3.609863	4.619148
Vcam1	3.475076	3.905154	5.923001	4.850833	3.93115	4.25468	3.92891	4.908552	4.618219
Carnmt1	5.089016	4.689989	5.166918	5.124567	4.429537	4.551276	5.086646	4.489515	4.616979
Gm13669	8.432327	9.662412	8.433307	5.084578	2.990212	3.733062	3.059602	4.868286	4.61686
Wdr73	5.399088	5.551252	5.260548	4.676984	5.301065	6.27974	5.493935	6.067864	4.615315
Fkbp10	3.710086	3.530084	3.881115	3.540883	2.538394	5.146457	2.203769	3.755676	4.613111
Cep295	8.251732	8.064821	7.965414	7.781386	4.932512	3.863769	9.257285	4.997304	4.612591
Accs	4.258094	5.816571	6.230465	5.420489	5.428189	5.255443	6.952478	4.716664	4.611034
Raet1e	5.222736	7.898639	4.409872	1.873059	2.976292	2.4545	3.097189	3.546004	4.610522
Fancl	7.301075	4.890119	5.333268	5.658668	4.601557	5.924378	5.474567	6.508361	4.604938
Ttc12	4.738561	5.807044	4.986908	5.05079	3.628061	3.608766	4.993569	4.835936	4.604824
Trim23	2.886608	3.863818	4.011332	3.67114	3.70415	3.554576	4.019811	4.296079	4.604629
Mapkbp1	4.83898	5.130298	4.518006	4.992217	4.412315	4.05565	6.952442	3.790919	4.604121
Ptgs2os2	3.907669	4.530967	3.050242	1.453218	2.844165	2.232849	2.068639	4.446431	4.602455
Mdn1	7.32284	5.058005	5.729145	5.413564	3.902953	2.915488	6.587262	4.641684	4.600698
Myef2	8.170306	7.481017	8.12509	7.45897	5.649143	5.826809	7.370976	4.988185	4.597426
Lonrf1	5.301494	3.925257	4.788055	3.402573	3.846256	3.612483	5.130258	5.145417	4.590905
Fastkd1	6.618789	5.986576	6.048907	4.334853	3.738724	3.081531	5.000896	4.346025	4.589828
Nuak1	5.963372	6.053701	5.985365	7.475996	6.450233	5.977584	8.573789	4.897029	4.587496
Mosmo	6.72961	5.971093	6.312644	4.68434	5.237869	4.978666	4.868771	6.716564	4.578209
Trim62	4.715182	4.529738	5.478569	3.913054	5.085044	4.76123	5.554216	3.779608	4.576598

Rtn4ip1	5.522929	5.848941	5.637459	4.246386	3.95854	3.720878	4.295879	2.92734	4.576468
Nt5e	5.605748	5.420937	4.553264	1.324292	3.215111	1.245883	2.848895	3.626952	4.575305
Gm52877	10.498	11.39335	10.27449	6.274242	7.216152	5.228143	10.03272	6.486562	4.574444
Rwdd2b	5.638662	5.413421	5.40215	4.192207	4.74284	4.11796	4.71129	5.368559	4.570535
Lrrc49	5.167361	6.086383	7.31933	4.882657	4.253283	4.908349	5.050731	5.209236	4.568396
5830417I10Rik	6.173566	5.375541	5.238274	5.034528	4.274147	3.853005	6.415652	4.444613	4.567455
Sfmbt1	6.851984	6.613805	7.018192	6.422016	5.325616	4.857983	7.033027	4.866575	4.563878
Mios	4.653505	5.969282	5.596827	4.79111	3.876173	3.749922	5.752998	5.541803	4.563554
Ddx19b	5.150168	5.111237	6.137084	4.559734	4.768492	3.414188	4.634718	4.14106	4.56312
Zfp317	5.339844	6.893651	6.487885	6.028253	5.220506	5.069105	7.261066	4.531147	4.562862
Krtcap3	4.599397	6.483175	6.930563	2.683198	5.665337	6.351711	8.176375	5.254892	4.558979
D730003I15Rik	3.806487	3.504326	3.709316	5.532164	6.785736	5.686347	6.960497	5.761467	4.558156
Gm5555	5.144211	3.618846	3.950478	3.425488	3.368262	4.927106	3.845185	5.597478	4.552684
Gm8494	5.260546	5.515594	6.115534	5.275789	5.010087	5.821114	6.366445	7.748278	4.542388
Sec22c	5.406652	6.413219	4.880468	4.477011	4.136427	3.575298	4.550559	4.759696	4.53731
Zfp212	5.224208	6.374955	6.679567	5.296806	5.775457	5.607962	8.109806	5.286049	4.530036
Arl6	2.742257	5.876461	3.187318	3.758695	3.141093	3.221346	3.217268	3.457675	4.520847
Ice2	4.020593	5.317773	5.231702	5.287771	4.419327	3.758098	6.657709	3.581031	4.518717
Bco2	4.794345	3.847249	4.82246	5.507273	6.088109	4.051448	5.325411	4.066244	4.516915
Ctsw	1.979931	1.057853	3.323842	3.726733	4.714161	8.239401	4.01963	1.481141	4.515441
Caskin2	6.52849	5.491875	6.288761	8.440692	5.931398	6.960178	5.020255	5.139737	4.514842
Abraxas1	4.502881	4.111791	4.649618	7.443379	4.539121	3.920765	4.442409	5.150188	4.514588
2900060B14Rik	3.528721	2.685802	1.671694	7.387273	5.483272	2.662125	3.455156	6.630963	4.513748
Depdc7	6.571591	4.876744	5.606748	5.3579	6.318035	6.422824	7.532434	4.952063	4.511958
Adgra2	6.200551	5.269244	7.045232	6.329997	5.94028	7.532336	5.005517	4.733305	4.510936
Hoxb4	3.694431	4.331015	5.358627	5.413927	5.596734	5.354505	5.292138	6.212122	4.509505
Purg	4.960489	6.642186	5.236681	3.973566	6.036626	4.12008	5.933928	4.766602	4.508908
Ska2	4.808194	9.098951	8.26119	5.762032	3.209404	6.384671	4.969508	3.717075	4.5043
Gm16437	5.606804	3.204334	5.04041	4.071919	5.219862	5.476289	5.20891	6.297908	4.503187
Fastkd5	5.455391	5.159443	4.665842	5.443539	3.511546	3.235743	5.055134	4.298538	4.500233
Lrrc17	1.766347	3.422624	4.996819	4.667146	1.634185	4.433684	2.29505	3.675793	4.497312
Pgm2l1	5.819323	6.56783	7.37157	4.948163	4.218003	3.958756	6.673235	4.805092	4.497191
Gm13292	4.615992	3.86711	3.739388	3.167193	3.209241	3.927227	3.864395	3.296158	4.487439
Pyroxd1	3.833073	4.20772	4.322356	5.320893	3.209567	3.568495	4.759324	3.184093	4.486888
Fkbp7	2.477679	6.980808	4.893984	7.206615	4.9554	8.305889	5.889851	5.897483	4.486751
Gm34432	3.204906	3.468406	3.614463	3.797222	3.441975	4.035809	2.889567	5.453539	4.484355
Apod	7.856492	7.863166	8.410743	6.271994	5.032202	6.318884	3.230876	7.111411	4.482214
Ddx59	5.711101	5.494527	6.923351	3.929485	6.565487	5.441491	6.856681	5.519915	4.481007
Gm17241	2.755693	4.365037	1.783303	3.707407	4.785838	3.597711	2.177994	3.241029	4.480364
Gm5620	6.144091	4.789493	3.602127	2.914385	5.178971	4.310379	5.182367	3.765374	4.476034
Tlcd3a	9.390161	9.049988	9.210651	6.105328	5.503438	6.888533	6.205729	5.458773	4.474829
Cd1d1	6.771061	7.251838	6.233178	7.077441	5.642599	4.518361	6.40179	4.688519	4.473395
Gemin6	6.017299	5.607752	4.14354	4.561263	4.556463	6.47684	4.282057	4.883787	4.465794
LOC115487311	3.123271	0	4.685451	7.76444	1.487285	2.888303	3.228057	6.690736	4.465132
Zbtb5	4.899118	5.20501	4.989238	5.571263	4.763326	3.891779	5.67163	3.629251	4.462041
Tubd1	5.392692	6.530836	5.185884	5.892841	3.612091	4.822587	4.838634	4.288047	4.46195
Gm33370	4.87938	4.96993	10.48669	2.993817	7.020039	7.776513	5.79418	6.91905	4.45259
Gm38965	2.803048	4.659309	4.049318	5.419841	4.152699	3.312219	2.89709	3.261838	4.45259
Pkd2	6.116056	8.550292	6.638022	6.176991	5.287965	4.318185	6.947878	4.972408	4.450426
Gm52232	5.187396	5.057626	4.901765	4.913763	3.722351	3.209122	6.02699	4.121435	4.449645
Ch25h	6.047051	3.966666	5.763631	2.946054	3.362722	2.92742	1.593942	2.472971	4.448244
Mms22l	5.523753	7.072671	7.074143	5.275274	4.184302	4.467562	6.368539	3.575531	4.448002
Msantd3	3.508738	3.534391	3.719695	3.209957	2.439429	1.55749	2.901165	3.674729	4.447705
Prune2	7.319486	6.823809	7.917233	3.480189	4.615501	3.377793	5.359197	3.623084	4.445968

Zfp1	4.061862	4.240941	6.347396	5.553775	5.621374	6.30938	8.429071	5.529017	4.445963
Gm15191	4.389789	4.829453	3.84717	3.915179	4.223888	4.310645	3.040302	4.394948	4.442655
Alyref2	6.14043	3.300924	7.273553	7.058748	6.884971	5.477113	4.636052	7.048719	4.441164
Snhg17	5.185533	5.792335	4.356354	6.094029	5.728828	4.429223	5.417975	5.134621	4.43906
Pick1	4.626849	6.460343	5.973536	7.445144	5.448111	6.612637	7.071681	6.193997	4.436524
Mtrf1l	4.272602	4.451083	3.511556	3.302183	4.57279	4.850939	4.67828	4.269318	4.435035
Smim40	3.057735	2.51923	4.188254	5.684613	3.038769	3.196528	4.190861	5.189643	4.434794
Shcbp1	3.910569	6.500263	6.978492	5.388214	3.578329	4.065463	5.362607	3.066202	4.433567
Zfp597	3.551485	3.849142	4.044646	2.93959	3.23576	2.496449	3.522114	3.30318	4.43165
Map2k6	4.441006	5.469595	5.693495	5.601668	6.472087	4.52035	7.393291	6.044444	4.431524
Ccl27a	2.996652	10.90866	7.042951	8.492619	9.703527	8.175069	8.052693	6.7048	4.426917
Tdrkh	7.722715	7.656309	7.493743	5.531037	3.808855	5.993948	4.8461	2.888593	4.425023
H4c18	1.911657	2.587485	2.458131	3.530334	3.641283	5.682346	1.129025	3.250152	4.424807
Gm7658	5.724093	5.798757	4.687991	4.033721	4.936455	4.751611	5.496882	5.278247	4.424595
Arl4a	4.03241	4.293818	3.456753	2.577743	3.623858	3.616895	3.305955	3.954716	4.421177
Cchcr1	4.399502	4.495863	4.746813	4.353307	3.653383	4.729907	3.919918	3.425289	4.42033
Tm4sf1	4.636936	4.747368	4.227079	8.405825	5.158709	5.160891	2.120578	5.547824	4.419412
Cilp	6.899788	7.054118	8.8189	7.602883	5.391496	7.144422	4.446722	7.076554	4.417928
Ofd1	4.556553	4.631337	4.158649	5.583318	3.782339	3.626595	3.885919	3.508558	4.413993
Art2a	4.939589	6.114062	4.984271	3.113806	3.486304	2.406345	7.156553	3.485239	4.413625
Bbs5	4.146783	4.09559	2.496412	2.402071	3.489232	2.159411	3.079196	1.456597	4.412709
Tnnt1	4.238725	5.506797	6.80506	3.771299	4.090033	5.931337	3.631564	5.575613	4.411976
Gm40960	9.255746	9.808049	10.7032	2.396849	3.030181	2.67482	5.879274	4.773371	4.410774
Cdca5	9.04577	8.267463	7.010559	3.875702	4.653812	5.836843	4.930154	3.156979	4.407737
Troap	6.881612	8.630333	8.346209	5.617441	5.0133	4.867908	8.757916	3.520461	4.405156
LOC115485682	1.421489	3.544261	2.487895	0.353382	2.707621	3.286371	4.407539	2.030095	4.403117
Ftl1-ps2	4.082419	4.130558	4.556272	5.236232	4.845976	4.869581	4.341684	4.450176	4.398417
Serpinb9	6.328943	6.44224	5.220354	6.553017	3.728422	5.168975	4.877861	3.89294	4.39448
Myh4	5.142507	7.848921	4.61961	6.492574	3.489588	6.003982	3.103602	3.881964	4.393635
Tbc1d24	5.582412	5.128615	5.922832	5.174924	4.4342	3.884326	6.870972	4.307873	4.393305
Ctdspl	6.512953	6.017115	5.913921	5.259896	4.67214	4.41463	5.950463	4.590773	4.38978
Heg1	6.935557	5.864491	6.785572	10.89864	6.958812	6.510314	5.861658	4.45007	4.388378
Tgtp1	2.348569	1.614033	3.11863	5.377338	3.702857	2.93843	3.059701	3.363497	4.382739
LOC118567383	5.353832	5.081212	5.743084	5.302375	4.605486	3.693328	7.162251	6.495039	4.382541
Pgap1	4.737311	4.834454	4.900332	5.112024	4.421927	3.653995	6.158222	5.139044	4.380225
Rab11fip3	6.471503	7.158065	8.486228	6.760438	4.147174	4.36115	7.079977	5.522413	4.379877
Snora78	3.884195	2.817349	3.884647	4.5647	1.345187	3.591982	5.109376	3.025747	4.375071
Rdh10	3.575091	4.6497	4.251953	3.579085	5.314369	3.060406	4.119464	4.714774	4.374338
Usp20	4.72097	4.961113	5.535114	4.177332	4.419994	4.180814	5.182596	3.174313	4.3724
C4b	4.949641	3.915844	5.712591	6.770229	7.677627	5.175988	7.047579	6.939567	4.369112
Gm32908	3.674797	4.72905	2.608223	3.339871	3.349306	3.909915	2.450378	3.987842	4.368595
Tigar	6.480168	4.78286	5.949698	4.980039	3.873536	2.736693	4.627132	4.638153	4.368162
Il12rb2	8.02393	8.730074	8.754396	6.760375	5.641114	5.909241	8.745485	6.306116	4.366038
Igflr1	6.357147	9.510334	5.750147	5.020044	6.98936	7.39184	11.98137	7.966325	4.365991
Tjp3	4.505167	4.605973	5.108963	6.279402	5.349876	5.70025	5.24079	5.581553	4.365688
Pdgfrb	7.106839	6.546316	6.021443	8.217476	4.587272	6.244664	3.392003	5.619939	4.365611
F3	7.417353	7.191298	4.551415	1.674983	3.827617	2.568835	2.504497	5.711536	4.365563
Gss	3.608933	4.718443	5.338202	6.061615	3.379342	5.440849	4.168837	4.013547	4.364577
Gm7353	3.440536	4.467263	4.853017	4.84141	5.162393	4.037156	5.937791	4.929042	4.36187
Igsf9	6.136749	7.030202	7.409189	5.140288	6.344784	5.713418	4.942685	4.500525	4.360304
Ankrd66	3.399306	3.139126	4.742793	3.046411	4.156741	3.609286	6.202585	4.435166	4.359785
Ssx2ip	5.104231	5.411818	5.781173	4.211019	4.508507	4.318194	4.842788	3.617968	4.358356
Coa7	4.452779	4.517824	4.588245	3.411926	3.543161	4.795176	3.964639	4.184424	4.354125
3830403N18Rik	8.060462	3.662212	3.582844	4.809186	3.582458	12.34061	4.443141	9.720842	4.353324

Clstn1	6.301856	5.608424	5.780635	8.199717	6.18821	6.889545	5.839029	5.540358	4.352339
Jrkl	3.774746	4.794838	5.59704	4.911429	4.542969	4.532787	4.949523	4.917066	4.34942
n-TRtcg4	0	1.514807	0	0	0	0	0.784904	0	4.3428
Islr	4.419168	7.495224	8.204199	8.841455	7.182626	9.078854	3.473435	5.99629	4.337963
LOC115490270	3.723452	4.951385	3.723885	4.113994	2.758136	6.121479	8.551941	6.696283	4.337398
Plk4	6.33069	7.05664	7.254421	6.118125	4.117181	5.000966	6.258602	3.607213	4.336247
Zfp994	3.37014	4.726183	5.666614	4.800061	4.260891	5.076383	4.654417	3.460916	4.334153
Gm18541	5.343428	5.329202	6.479659	4.184378	3.689541	5.867954	4.832362	5.214654	4.328847
Cenpe	7.067235	8.739758	8.53702	5.560027	4.527719	4.212679	7.120999	3.276526	4.328163
Nrip1	6.47776	5.715341	6.605404	5.922792	5.209373	4.308677	7.56931	5.355424	4.326419
Gm52148	2.393916	2.513203	3.528287	5.888622	3.839898	2.097302	7.943587	3.478845	4.32306
Gm15387	5.669802	7.204516	5.586454	4.677487	4.999868	5.0879	6.163876	5.478181	4.32306
Fpgt	4.555031	5.35018	5.333339	5.097678	5.638099	5.122335	4.773467	4.669277	4.311096
Iqgap3	8.663418	8.757247	9.201678	5.846859	5.897519	5.112287	7.31872	3.77549	4.310027
Gem	5.084483	6.761263	2.972435	3.908021	2.807192	3.588944	4.696558	4.419976	4.307524
Slco4c1	1.042069	1.247153	0.972711	0.96715	1.521764	2.719698	1.43604	1.543347	4.303798
Ddb2	3.939344	5.484029	3.734604	2.366689	5.588601	5.047048	5.174209	2.617635	4.302083
Gm8717	3.398715	3.000689	2.897602	4.26615	2.918516	4.482676	4.261359	4.456007	4.301336
Stxbp6	5.983403	5.358548	5.400615	2.53646	4.251283	3.588466	3.786212	4.275819	4.2932
Enkd1	5.893188	8.186516	6.230666	5.316018	3.808555	5.138417	5.220775	3.326458	4.292788
Tcap	4.912233	6.357238	2.807317	6.745562	6.516283	8.003769	2.115433	3.062274	4.291645
Vasn	5.754155	5.565457	4.746425	7.104864	4.817935	3.761859	6.989314	3.5089	4.291278
Cdca7l	6.199891	7.835111	7.241125	5.4473	3.821883	5.321126	6.693179	3.113175	4.29013
Fam71f2	3.888441	4.099694	2.055558	2.099044	2.380699	1.541104	5.798696	3.01463	4.288406
Gm15610	3.227164	1.954133	1.613769	2.177595	3.073515	2.77121	2.144208	2.962841	4.284113
Acaca	6.450997	5.417634	6.470163	4.797413	4.115392	3.16164	5.836378	4.254389	4.282216
Camsap1	5.934034	5.596592	5.128375	4.938708	4.691674	4.078426	6.649768	4.383186	4.280167
Zfp932	3.927419	4.716344	5.397489	5.233794	4.325669	3.681369	4.224865	4.883968	4.277919
H2bc6	3.363658	5.466923	2.367294	5.574694	2.254322	2.995398	3.476508	5.456165	4.274486
Snora16a	7.337391	2.845833	0.40768	7.70164	6.988051	0.376966	1.685235	6.209702	4.273613
Rttn	5.324063	6.261662	6.731202	6.680232	4.472634	3.99456	7.436411	4.542998	4.272469
Nfatc2ip	4.221701	4.349078	4.256943	5.14823	4.004156	3.968362	4.650637	3.787899	4.27203
Gtf3c3	5.911531	6.069882	5.877304	5.565982	4.178227	4.315328	6.795418	4.808479	4.270025
Stxbp1	6.568423	6.051409	6.626434	5.805893	3.979657	4.155384	4.83646	3.74682	4.269
Asns	5.431139	10.25712	3.178163	2.47054	4.0445	3.259313	2.896251	3.768212	4.267742
Atm	6.347012	6.43415	6.773073	5.792819	4.172534	3.398533	7.886873	4.591441	4.267371
Fam216a	3.459791	2.395109	4.274356	3.966591	4.845687	2.936018	3.407591	3.875365	4.267338
Gm15921	1.6069	2.976299	2.066255	3.994748	1.202453	0.849149	3.677512	3.387695	4.266374
Sytl1	0.660692	1.048303	0.570664	4.001673	4.347447	5.94324	4.469617	2.887882	4.264774
Kctd12b	4.185314	5.823084	5.73524	5.277067	5.384467	4.5868	6.357151	4.48018	4.264457
Usf3	6.186184	6.362792	6.468526	6.029026	4.521404	3.26321	7.55788	4.344081	4.256346
Tcea2	3.767184	4.684847	3.302483	4.763536	3.897884	4.343965	5.479843	4.870865	4.255361
Armcx2	4.860098	4.288503	5.28906	5.422648	3.781416	3.80887	3.96744	3.466546	4.255256
5730455P16Rik	5.130738	5.78201	5.160325	5.34701	4.003031	3.80652	5.302874	4.981561	4.254619
Apoa2	2.447914	3.015844	2.448199	4.152463	3.977038	2.530079	3.869476	3.427433	4.25444
A130077B15Rik	3.896118	3.907454	4.527859	3.592921	4.622728	4.086093	4.836698	2.942721	4.252716
Gm6170	6.039342	5.498159	6.287841	5.635942	4.630939	4.210227	4.257338	5.219272	4.250606
Slc25a40	4.458447	4.446577	3.591384	4.79457	3.861907	3.376782	5.004193	3.956764	4.249296
Tns4	5.619589	5.279048	6.89121	5.066399	5.574111	4.614604	6.049127	4.573371	4.244771
Rcn1	3.637953	4.422516	4.688256	5.02141	4.033586	4.870653	3.984484	3.877263	4.243552
Zkscan14	4.725419	5.942274	3.972553	5.039453	5.511405	7.1882	5.37943	5.183701	4.242657
Gpr176	4.266647	3.392921	3.756787	5.356292	3.577507	3.95877	4.93721	3.630397	4.242123
Zfp948	4.94023	5.31877	4.444657	5.200322	4.705021	5.122915	6.06735	4.369019	4.23566
Ctsg	0.388844	0.055401	0.722223	0.497142	1.375515	3.493168	0.80378	1.004877	4.235463

Zfp959	4.442869	4.347436	4.750789	4.028977	3.273301	4.780477	4.332006	5.640062	4.234449
Tfap4	3.646854	6.570327	4.794406	3.772653	5.237846	3.78046	5.167459	3.248152	4.232598
Nmb	1.613917	2.33395	2.824684	2.407315	2.075054	3.805876	2.418693	2.996385	4.230068
Prodh	5.181444	3.276222	3.353089	4.276845	4.192925	5.292723	3.780198	2.934151	4.228283
Gm2451	4.01926	4.930067	3.188059	2.480739	2.551933	3.503271	2.817152	3.518831	4.226992
Exoc8	4.872442	4.792289	5.352075	3.877608	4.00605	4.26363	4.115369	3.883684	4.222997
Gm7846	1.000171	4.275036	3.572455	2.557462	2.857656	5.021033	3.544206	5.713566	4.221545
Myo10	6.721269	6.307312	5.868142	4.827607	3.08244	3.470464	5.438385	4.121267	4.220179
Gm13373	7.596165	8.270982	6.063698	7.103169	8.960165	5.864654	6.842628	6.137496	4.217693
Rps15a-ps3	3.643816	3.634115	1.561817	5.305701	5.453377	3.249341	2.690047	4.708295	4.217069
Gbp6	1.769728	0.869137	3.580093	5.759393	3.983837	2.71525	2.508087	3.90603	4.216769
Pfas	5.57079	5.330248	5.301571	4.976971	3.664152	3.694729	5.739697	3.82878	4.214382
Nr1d1	2.822754	3.539749	1.847458	3.900824	4.625561	4.568191	8.516401	4.900806	4.21354
Card10	4.185996	3.449807	2.333641	3.941012	3.707516	3.903608	3.090311	3.036555	4.213134
Nkain1	6.844765	7.346444	6.482882	5.206238	3.25944	4.485372	4.122833	3.754763	4.212303
LOC115489593	6.37783	3.91437	4.906593	5.366396	5.606931	5.898018	7.60593	6.539421	4.208288
Rnd2	6.641432	8.60691	6.681978	6.999693	5.188959	4.670701	5.590049	3.786404	4.207282
Gcfc2	5.872993	5.185813	6.210383	7.588404	5.058976	5.43116	6.456658	4.630041	4.207026
Arhgef40	6.307257	7.195568	7.215614	4.57986	4.61326	5.717357	5.006394	3.780205	4.205801
Mturn	6.257842	9.761849	8.906426	4.547422	5.195811	6.677373	4.809384	5.270608	4.205582
Wdr92	3.37702	4.238238	3.63597	3.454508	3.554788	4.28847	4.124922	4.353669	4.2042
5031425E22Rik	4.279205	4.331512	6.259864	3.112038	3.467189	4.429786	5.016876	5.047172	4.200208
Iigp1	3.330652	2.014853	4.696219	10.44274	8.008193	6.193827	4.740347	5.49312	4.19785
Stxbp3-ps	7.322008	6.09458	7.543096	5.20071	6.553948	4.683813	4.551977	4.612569	4.197609
Ska3	4.527105	5.397884	5.235863	5.381976	3.63713	4.163136	4.783547	2.96179	4.19421
Mir7014	1.759939	4.388133	0	0	0.838073	3.255072	3.637969	0.837817	4.193444
Gm30744	4.019678	3.507855	1.968196	4.330259	3.549151	1.626307	6.361636	3.388603	4.190277
Pisd-ps1	2.987795	3.671933	3.527936	3.181909	3.653311	2.869975	4.761552	2.826321	4.188778
Gm13588	2.25358	1.910443	3.606147	1.568669	2.682856	3.542868	2.096268	3.111162	4.18833
Ckap2	5.690874	7.227513	8.206895	2.90368	4.019271	6.031457	5.37513	3.307783	4.184738
Mr1	6.501416	6.952273	8.403528	5.811496	6.795417	6.402997	9.630528	6.167638	4.183147
Cep131	4.694727	3.925036	4.9742	5.284724	5.150004	4.484822	5.492794	3.820754	4.179146
Sox18	7.019644	5.737368	5.890337	16.13987	8.185577	8.866503	3.645271	7.694047	4.177385
H2ac20	2.013776	1.77213	3.672606	2.591465	3.497343	5.039102	4.652402	3.609058	4.177316
Dcaf10	5.749019	5.657059	5.619012	3.974257	4.304115	3.653325	4.790059	4.741861	4.175422
Gm13414	3.531555	3.595532	2.943305	5.670051	3.608664	3.504008	3.497954	3.222287	4.172296
B3gnt5	6.423892	5.288337	3.218415	2.084858	1.938735	1.330156	1.927571	2.599664	4.170762
Dlg3	8.052828	9.757142	7.371523	5.211636	2.994879	5.123661	4.453837	5.116668	4.170539
Hdac1-ps	4.82314	3.551791	4.739566	4.071127	3.952551	4.667744	3.622787	4.245024	4.169264
Tmem221	2.915774	5.242072	5.832225	4.921419	5.955829	5.747624	5.234143	5.552204	4.168487
Fahd1	3.900759	4.361935	3.585963	3.134472	3.339784	2.878553	2.443412	2.363394	4.168409
Cbwd1	5.229467	5.158544	4.972858	5.114925	4.89884	4.994606	4.341653	4.652475	4.167073
Ppp1r3e	6.0467	4.657297	4.909773	3.363612	3.535112	2.740535	3.867306	4.189537	4.165367
Plppr3	4.703079	5.084897	3.392438	4.800896	4.736812	4.618683	7.527891	4.854244	4.16511
Dbn1	4.654813	5.137105	5.284972	5.121965	4.923751	5.151437	3.056156	4.014083	4.163718
Mcm10	7.80639	8.181217	8.251615	6.453108	4.488032	4.783391	6.461195	3.625585	4.158639
Plcg1	5.237553	4.774803	5.425686	6.326925	4.285794	4.115262	6.356396	4.213076	4.157892
Slc26a10	5.321586	5.744843	4.649618	5.524383	5.402389	4.623799	4.109983	4.092312	4.152306
Pex1	3.837002	4.354619	4.485844	3.920764	3.062067	4.050001	6.085349	3.527229	4.148808
Akt2-ps	4.389411	3.259187	4.119177	3.288039	3.20438	3.004158	3.237627	3.829352	4.146639
Cdc42bpg	6.292661	5.651158	6.805977	3.341062	3.782951	4.45002	4.782191	3.944453	4.138522
Zfp84	4.89245	4.889107	5.359021	4.749211	4.086322	4.910394	5.317447	5.26808	4.135583
Rps15a-ps5	3.736043	3.846294	2.531162	4.314328	2.63993	5.238189	3.363144	2.983357	4.135101
Nradd	3.857803	4.132535	5.04907	5.304367	3.538051	4.712723	5.808561	4.262502	4.130759

Tmed8	5.096688	4.990699	5.383037	4.407745	3.552267	2.999346	4.565324	3.418839	4.128957
Snord71	5.197318	7.775223	3.465282	2.584103	9.07476	4.806317	7.162251	0.824726	4.127922
H1f5	1.466246	3.551403	3.421638	1.249742	2.127903	2.970151	2.597894	3.323833	4.125842
Cit	8.61333	9.508552	8.538656	7.373745	5.873184	6.017737	9.501876	4.490592	4.122663
Gstcd	6.091383	4.976466	4.569068	4.704044	4.24302	3.191103	4.755299	3.701868	4.122614
Car5b	7.361738	7.678787	6.832101	4.251644	2.801434	3.299901	4.618395	4.254429	4.12098
Chtf18	5.666255	5.723007	6.435309	4.225886	3.567164	5.750651	4.441898	3.383199	4.118983
Bcdin3d	3.969208	4.089146	4.667633	4.250588	4.569509	3.831945	2.704863	4.111302	4.11558
Gm8971	3.48229	2.083812	2.925464	4.155341	4.245114	3.220313	2.879297	3.978578	4.115476
Thoc2l	4.3429	5.159942	4.330815	4.381161	3.776467	3.154737	5.451377	4.218762	4.115174
Thtpa	5.155213	5.219389	5.976055	4.796231	5.504891	5.128544	4.904338	4.610797	4.113091
Cry2	7.667285	6.661143	4.612887	4.64608	3.59407	3.268261	4.550409	3.393362	4.110527
Btrc	4.048898	4.056268	4.103967	3.700502	4.350057	3.634911	4.927647	3.846285	4.110443
Jade1	4.556833	5.91015	4.414306	4.714186	3.376542	4.053393	5.681225	3.900805	4.109359
C920009B18Rik	3.957762	2.628227	4.996766	2.786088	3.610719	2.282979	3.229911	3.245856	4.108227
Pbld2	4.36181	5.531732	4.4431	3.668029	5.128568	3.510783	6.205661	4.1016	4.105869
Zc2hc1a	3.826727	3.372373	3.497243	4.608996	2.701982	3.29478	3.324341	3.10947	4.103112
Chml	4.785237	6.010816	5.701258	4.271775	3.840997	3.067494	4.702239	4.495613	4.102953
Nup35	6.249972	6.172619	6.717471	4.03569	4.67689	5.047923	4.257502	6.143787	4.10012
Lnpk	5.116273	5.038629	4.635356	3.383232	3.527651	3.609376	4.485115	3.832169	4.099242
Acy1	7.748157	7.534341	5.501831	4.160568	6.346163	6.950295	6.206283	5.901604	4.098498
Agbl5	3.796302	4.887998	5.685071	5.253094	4.132065	3.603577	5.70904	3.690948	4.096813
Mir6403	0.859505	5.1433	2.578814	5.555487	5.32079	4.769059	5.330047	2.045832	4.095922
Stimate	5.374578	5.766587	6.2998	5.656103	5.461925	4.593488	4.745143	5.713874	4.091986
Stard4	4.558737	4.059086	4.346713	3.529345	3.552293	2.918615	3.844029	3.561324	4.091683
Neil3	5.151324	6.880728	5.703915	7.287322	3.475129	4.792139	5.609373	2.685834	4.091391
Fads3	5.189369	4.499011	5.495265	5.025391	5.297627	5.47333	5.310889	4.020445	4.089282
LOC115486847	3.959862	6.253089	4.950402	4.59396	5.656993	5.187771	11.59602	5.655265	4.088608
Cenpv	2.787988	2.994455	2.359341	3.305519	2.808436	2.77657	2.992352	3.164906	4.088
Mrm3	6.023657	5.24394	4.237471	5.228485	3.50046	5.523282	4.220847	4.568648	4.086871
Tha1	3.589014	4.344003	6.203094	7.761512	6.438045	6.380228	6.086337	4.545065	4.084852
Proser3	6.153395	6.309079	7.361926	2.887905	5.394878	5.477739	5.706025	3.805375	4.083388
Meis3	4.509779	5.108184	4.027056	5.829872	3.681483	4.736499	3.495811	3.987055	4.083309
Hgh1	4.08669	4.160137	4.03079	4.400113	5.36844	5.655832	3.874242	5.447301	4.083012
Rpl36a-ps3	2.855179	1.423789	2.141633	7.807761	3.625655	3.850555	2.828013	4.417417	4.081859
Apobec2	4.278471	4.630237	2.91334	4.707112	3.597934	7.913192	2.869605	3.856847	4.077763
4933427D14Rik	4.782579	3.961623	4.597124	5.654105	4.985059	3.894503	5.972833	4.439647	4.077077
Psmc3ip	2.908787	3.469875	2.795042	2.155191	2.172782	3.850328	3.065325	2.443633	4.07696
Utp23	4.67527	3.753004	4.219637	4.157715	4.416476	3.479925	4.439269	4.161799	4.075562
Zfp397	4.112136	4.343028	4.355127	3.99868	3.627679	3.840149	4.208328	2.77043	4.073304
Clcf1	2.947873	4.65004	2.918132	1.884446	3.924801	2.587014	4.943235	3.064419	4.071031
Gm13077	3.085606	2.513203	3.497427	3.528571	3.036654	4.042419	5.262062	2.154386	4.068186
Dmac2l	4.318545	6.410489	6.327906	6.19186	4.782482	5.990482	5.70904	5.020072	4.068097
Fcsk	3.59938	3.931682	4.30452	4.45035	3.627523	4.068281	5.019234	3.626415	4.065811
Vdr	7.537743	6.768448	7.564087	3.051386	6.26938	4.121172	6.856284	4.53391	4.065349
Snord16a	4.873676	6.075876	4.874242	2.423188	1.160409	3.380267	9.444726	6.380298	4.064415
Gm26202	6.823146	8.506226	2.558977	3.392463	9.747435	2.366187	8.815078	9.744456	4.064415
Mir8094	2.369148	1.890273	1.895539	1.884702	2.256351	1.314548	5.386992	4.962454	4.064415
LOC115489081	6.823146	3.402491	5.117954	5.088695	3.249145	11.83093	2.644523	12.18057	4.064415
9830107B12Rik	0.480503	0.718836	0.800932	1.85152	2.440672	2.925333	1.46918	1.353397	4.064415
Mir7052	2.55868	4.253113	1.705985	5.088695	1.624572	6.309832	0	3.248152	4.064415
Rnf24	4.293586	3.765515	3.277851	3.209581	2.647046	2.321542	3.17121	3.566255	4.063685
Gm8894	4.007571	4.829589	3.173029	4.400239	2.544511	2.161878	4.400901	3.656617	4.058294
Zfp952	3.188426	3.71299	4.128846	4.306838	3.528098	3.272386	3.581125	3.755136	4.057659

Invs	3.685281	4.868941	5.094328	4.033208	3.691364	4.025004	5.166124	4.849736	4.057428
B9d1	2.418764	2.991282	5.031613	3.944552	4.607208	3.131516	3.499878	3.408292	4.057322
Gm11826	2.766583	3.289836	2.128388	6.242848	4.763022	4.624889	2.749424	4.153707	4.056614
Mcpt4	4.927828	9.326776	5.65646	7.51739	11.99968	21.59444	3.356853	3.358884	4.056204
Zscan22	6.609265	5.208934	6.705005	6.440043	6.366944	5.181185	5.083996	4.592925	4.054668
Platr25	4.118698	4.184873	4.680004	4.999357	3.683195	3.808843	4.676572	3.626839	4.054492
Mtif3	3.644873	4.005418	4.06158	3.288858	4.392741	3.745182	3.709023	4.155763	4.052869
Gm17455	4.252351	2.905891	2.992743	3.523776	3.824899	2.039044	1.05806	2.999004	4.052869
Tab3	3.981017	4.152867	3.319352	3.620884	3.310283	2.747042	4.55575	3.375623	4.051642
Lmtk2	5.144576	4.150672	4.067331	2.493848	3.201872	3.008723	4.000142	3.504204	4.050501
Dars2	6.00722	5.228708	6.616513	4.856435	4.354064	4.27108	4.72511	4.025907	4.044968
Carns1	3.401491	3.62586	3.074182	2.094629	4.591829	4.213362	2.918925	2.971149	4.044968
Tbc1d8b	4.624606	4.878571	5.483418	3.840153	3.432727	2.945185	4.937444	3.622326	4.044142
Gm11993	2.191412	3.348119	2.471454	1.576397	3.330442	2.112785	2.698644	4.306055	4.043905
Sccpdh	5.422662	4.602745	4.932888	2.351955	3.818421	3.921075	4.948735	4.3665	4.041154
Tcf7l2	3.819994	3.553701	3.611759	4.006082	3.699265	2.924052	5.673398	3.132717	4.038527
Zfyve28	5.987198	8.027399	3.84129	7.526325	4.787647	5.223371	9.515614	4.705518	4.037499
Pfkfb1	2.519912	2.67362	2.73469	3.252204	2.961624	4.462345	3.324836	2.960719	4.0369
AI597479	4.939232	6.173596	6.367217	3.093648	4.597159	4.004106	4.193345	4.046096	4.035023
LOC115489590	0.423191	6.33097	1.692962	6.733133	1.209128	1.174062	2.624336	1.208759	4.033389
Dock6	5.657208	5.256579	5.181007	6.778815	4.12374	6.041972	4.368607	5.472102	4.028514
Gnb5	3.878772	2.736218	3.437686	3.041735	2.282534	3.12037	4.367425	3.092488	4.027424
Tspan33	6.076544	4.399992	4.07925	6.042506	4.518799	7.492531	4.531073	3.460712	4.019662
B3gntl1	4.818955	3.664671	4.27732	4.312765	4.073199	3.305177	3.818483	3.135214	4.018781
Asb7	3.342372	3.220475	3.240778	3.098318	2.762404	2.242228	4.09857	3.214628	4.017068
Gm38922	3.288306	0.692863	2.732854	2.671175	2.602437	1.413387	1.627512	3.218981	4.016878
Clu	3.496094	2.583958	3.902344	7.884229	9.27543	8.082696	3.129452	4.606578	4.016356
Gne	6.137095	4.609326	5.428359	3.986539	5.189781	4.482666	5.143268	3.888588	4.01556
Miga1	3.841145	5.930557	5.097496	3.231993	5.170816	3.518015	6.336755	4.11429	4.012967
Gm12867	4.926765	4.675122	3.492191	2.140413	2.414431	1.902069	4.350496	3.688852	4.011813
Kif4	5.950159	6.006687	6.567707	3.499588	2.747048	3.321643	4.799833	2.642578	4.011216
Nlgn4l	3.482805	4.753973	3.633466	4.563402	4.227531	4.534366	5.20891	3.537037	4.009342
1700047M11Rik	0.080814	1.128377	0.323292	2.089381	0.307864	2.316748	0.417624	1.461905	4.005167
Oxsm	5.628483	6.126827	6.011055	5.16769	4.648934	3.930653	6.349285	4.995286	4.003553
TrnC	3.359883	5.026406	7.560614	4.176328	6.399831	17.86591	10.41782	5.598141	4.002833
Jmjd4	3.548341	3.564263	2.925496	4.451684	3.464189	3.293156	3.667388	3.087756	4.000078
Gm19708	4.577455	3.614171	7.375645	7.01738	5.207202	3.762744	8.870677	4.358186	3.999161
Map3k15	3.564915	3.736823	4.159551	2.676796	3.730085	3.195802	5.063078	3.913661	3.998631
Gm34872	2.641011	2.379824	2.201098	1.451331	2.140186	2.52802	3.088777	2.558615	3.996477
Zfp623	4.705322	5.760351	6.222453	5.322046	5.330833	5.91864	5.393296	6.369566	3.995749
Gm17082	3.897367	2.908633	4.560715	3.822808	2.49979	2.525366	3.288264	3.180579	3.992496
Usp54	4.156863	4.689993	4.46493	4.025059	3.694391	3.477154	6.121494	4.014415	3.990226
Zfp961	4.385277	3.824359	6.242097	3.9145	4.52615	4.564646	4.195108	3.029457	3.985161
Kank2	5.57465	5.707918	6.649228	5.270796	4.699979	3.950959	4.769914	4.926944	3.984852
Gm6745	3.993026	2.315346	4.922209	4.247682	4.333554	4.379626	4.318945	5.481596	3.982718
Zfp617	4.400443	6.001679	4.908757	4.375794	3.456625	4.678062	3.945555	4.565648	3.978936
Tpsb2	6.090855	14.23041	9.193748	7.795245	12.56854	24.61669	4.721402	2.201507	3.977586
Rfesd	3.86848	5.437909	4.874242	4.331448	5.076789	5.661947	3.935303	5.33625	3.977321
Stfa3	0.134558	0.671001	0.538296	1.204242	2.050431	4.479674	0	1.537353	3.975629
4930430F08Rik	2.841802	2.399958	3.2547	3.099357	2.946594	3.327399	3.150324	1.898336	3.975364
Nars2	3.922854	3.91241	3.553186	4.833165	3.195643	3.764628	5.81395	3.57051	3.973974
LOC118568080	4.951193	5.700638	3.842878	4.182088	4.56981	2.351228	4.702388	2.366111	3.971915
Clic3	2.220298	4.290374	2.63691	4.346726	5.088226	2.374084	4.804714	2.378184	3.967765
5730409E04Rik	4.473949	4.888842	5.07755	4.584289	2.7974	4.119379	4.181749	3.222509	3.96744

Ptpn4	4.097526	4.555043	4.648721	4.138993	2.606767	2.785783	4.268475	3.361548	3.96705
Cd163	9.279494	12.26448	11.10399	4.538737	6.682155	4.499995	8.473191	5.051985	3.9667
Tmem107	3.841714	5.853659	5.496424	2.918204	2.845738	4.391528	4.02575	3.556086	3.966619
9930104L06Rik	4.58905	5.856795	5.523058	4.041267	5.037257	3.848201	4.743013	4.832068	3.966048
Gm11110	4.268623	2.748357	4.485277	2.149221	2.47011	2.748252	3.406607	2.263576	3.96538
Wnt11	4.909512	4.145892	3.619842	4.418754	6.2116	5.998317	9.000268	4.742575	3.961951
Pecr	3.076893	3.930092	4.372936	5.475178	3.033221	3.843806	4.519122	2.826715	3.961519
Pex12	4.080589	4.55135	4.998699	4.393875	5.312055	3.617298	3.369003	5.425376	3.958202
Gm13657	3.529764	4.76285	3.322517	2.580877	4.053825	0.960063	2.78979	1.779184	3.957858
Gm6311	2.982427	4.724186	5.088262	4.768415	3.898631	4.055964	5.500125	5.233705	3.957231
Rsf1	4.533079	5.097315	4.902273	4.41357	4.288789	4.311822	5.375067	4.39182	3.954852
Gm6793	4.631359	4.722441	4.70103	4.158623	3.95002	3.867427	4.358078	4.277881	3.952923
Coil	3.97657	4.856739	3.870694	3.4045	3.665726	4.169039	4.417683	3.482388	3.952164
Gm24097	2.072451	6.200801	0.518173	5.152105	5.427894	4.791344	5.890449	0.493294	3.95046
Pard6a	4.992723	5.261285	5.464369	4.496383	4.620439	6.18519	5.354954	6.143754	3.95046
Gm25596	2.072451	2.066934	1.036346	1.545632	4.441004	0	1.070991	0.986588	3.95046
Map3k12	4.323226	5.851615	5.10395	4.864669	4.612719	4.629245	5.173786	4.147083	3.950025
Tsr1	5.666638	3.78948	4.094868	4.022599	2.573639	2.499001	3.385406	3.212168	3.949147
Gas1	2.443453	3.51415	3.506232	4.17286	2.883603	3.897042	3.001237	2.343265	3.948883
Adgrf5	7.019692	5.178027	3.743622	11.81442	5.250229	7.453641	3.587406	4.952406	3.947515
Coq8a	4.837331	4.824453	6.728047	3.624456	5.164149	4.993576	5.301926	5.548157	3.94565
Tspoap1	3.681633	3.387822	4.109261	4.085768	4.959252	4.853049	5.476474	3.80545	3.945115
Gm42266	4.284157	3.332208	4.688867	4.420919	5.337599	4.609704	5.569672	5.566851	3.94194
Gm10243	5.685955	2.964291	3.231032	3.726569	5.292168	4.063149	4.407539	4.552334	3.941251
Ubxn2b	4.718382	5.301346	3.852427	3.737262	3.445569	2.750141	4.610462	3.366485	3.939092
Hmmr	7.008914	7.894538	7.858956	4.536715	3.412994	3.912931	5.756575	2.754225	3.936743
Gm51710	3.639873	3.141504	3.780307	2.575402	3.599905	3.236577	2.966185	5.264918	3.936119
Gm35248	2.477679	2.368121	2.581215	2.258483	4.621107	2.48222	4.694809	3.145324	3.935747
Plekhh3	4.262365	4.768769	4.782054	2.74415	2.88844	4.017503	3.812336	5.072736	3.932207
Gm12366	4.300721	4.5243	4.360142	3.573623	4.488723	3.813727	4.5059	4.711719	3.930518
Nudcd1	5.102749	4.703864	5.26433	3.88966	4.215916	3.513096	4.325633	4.413865	3.927519
Atr	5.198058	5.444021	5.435504	4.615547	4.668651	3.284967	6.094491	4.182464	3.927256
Sclt1	3.377063	4.572711	4.264962	3.65229	3.451043	4.787083	3.770611	3.590804	3.923453
Cdk7	4.129764	3.543888	3.58295	3.693065	3.425864	3.562672	3.348291	3.897205	3.922123
Ndufb4c	4.915127	2.964025	3.886831	3.750945	3.810209	5.073885	1.890243	4.679683	3.921951
2700097O09Rik	4.766168	5.153772	5.569539	5.288251	3.440271	4.825166	6.429821	5.923101	3.920965
Cenps	6.020693	5.231787	2.503945	2.015415	2.611544	5.181865	3.080538	4.710693	3.920194
Zbtb6	4.107429	5.059657	4.865913	4.473413	5.006262	3.86625	5.622406	4.085258	3.914744
Nupl2	6.14023	5.031725	5.710686	5.814155	4.153118	3.960338	5.477164	4.84072	3.913881
Mettl21a	3.758145	4.788085	6.452593	4.493153	3.827485	5.283433	5.051744	4.426116	3.913115
Usp11	3.771524	2.746215	5.324141	4.364401	4.974703	4.953892	5.778095	2.367426	3.912703
Capn10	5.010044	4.881397	5.010626	5.365209	4.698103	4.686591	4.222168	3.797696	3.911841
Rad18	5.380753	5.474841	6.278275	3.837308	2.381114	3.065991	3.904129	3.492958	3.911004
Cdc42ep1	2.81334	2.457838	3.075403	6.636122	2.762476	3.630263	1.645458	2.595519	3.907723
Mag	3.38308	5.492105	5.23574	4.051689	4.58607	3.722309	4.491959	3.667735	3.906908
Trim32	4.375318	4.692677	4.688386	5.317657	3.803219	4.142495	4.665669	3.88471	3.905301
Npat	5.656708	6.117589	6.728307	5.300946	3.602676	4.111724	7.326157	5.00896	3.904842
Maml2	5.35453	5.700433	8.256897	7.12002	5.609555	4.445016	7.271647	4.505242	3.904651
Gin1	4.380099	5.221447	3.680748	4.974106	3.727251	4.338194	5.143159	3.553378	3.902903
Ccdc51	3.65424	2.952516	4.140412	4.783698	2.995665	4.299017	3.968072	3.633336	3.901635
Mfsd3	3.552739	3.543281	3.706525	2.821187	3.529644	2.789097	4.041768	2.141474	3.897641
H2ac18	2.692136	2.983299	3.09133	3.172807	3.323653	2.305188	3.194673	4.082097	3.896283
Gm2991	3.562285	4.292968	5.344049	5.018302	2.403149	5.490483	3.374983	4.734171	3.890304
Zfp668	4.540219	4.299438	4.21204	4.408378	4.433248	4.142105	4.163249	3.886094	3.890138

Gm12657	4.818237	2.430972	2.664511	2.987483	3.401137	4.613016	4.686953	3.238188	3.88987
Bub1	6.578925	8.153611	8.356592	4.340044	3.273857	4.226642	4.803665	3.052214	3.889795
4931414P19Rik	4.924163	4.300109	5.513818	3.37372	4.420453	4.531923	5.454634	3.970462	3.884773
E230029C05Rik	3.189215	3.045375	4.41113	3.441253	3.683624	2.44728	3.085817	1.48592	3.880348
Bahcc1	5.729125	4.711227	6.723116	4.733475	4.798824	4.054794	4.494207	3.909385	3.878821
Map2k3os	3.194143	2.400139	2.669388	2.393065	2.416984	2.913379	2.035051	3.374412	3.878357
Diaph3	5.441773	7.256571	6.77871	3.237111	3.412706	3.3025	4.243367	2.879675	3.878293
Stard10	3.684577	3.294619	4.532133	3.369138	2.86379	3.799035	2.80143	3.225819	3.875012
Rnu12	1.847935	0.737206	2.58741	2.940135	0.703981	4.443173	0.381987	0.703766	3.874743
Pms2	5.101036	5.168855	5.489353	5.660868	3.439585	4.90597	4.38645	3.555095	3.869946
Eme1	6.901318	7.147674	7.319282	3.959227	4.189219	3.787194	4.075918	2.057866	3.867033
Elp6	2.900829	3.750323	2.175875	3.365343	2.353426	3.576794	3.192476	3.017602	3.865527
Osgepl1	4.085887	4.908534	4.148277	5.817478	4.716793	3.349126	4.446914	4.302759	3.864712
Cox11	3.730228	4.258761	3.14161	2.537965	2.898197	3.676546	3.17054	2.289811	3.859305
Rps25-ps1	6.522125	5.158949	3.486369	4.584642	3.534186	6.343429	6.973388	6.102638	3.858309
Lamb1	4.533282	4.414718	4.562934	5.444217	3.892175	5.134818	2.618592	2.892823	3.858028
Fn3krp	3.41704	4.553012	4.264962	4.186212	3.098143	3.918365	4.181511	2.706793	3.855984
Gm34076	4.043718	3.512571	2.609153	3.242796	3.851192	1.326921	4.718659	1.862911	3.854022
Mamld1	6.909047	8.476991	7.539525	4.31662	3.29794	2.374909	5.702402	3.801726	3.853056
Gmfg-ps	1.347453	2.49575	2.117672	2.488395	1.558292	1.335088	0.596854	2.19927	3.852727
Vamp5	1.802278	2.840018	2.739781	3.476833	3.501598	4.533397	2.98039	4.633052	3.847717
Eno1b	4.541002	3.868446	3.090763	3.323958	3.483867	4.461837	4.269647	4.98401	3.847092
Gm8524	3.537424	2.59088	3.150884	2.143543	2.632034	3.935781	3.427597	3.631097	3.845593
Zfp7	4.032251	3.741946	4.011154	4.309853	4.333141	4.68605	4.390396	4.762945	3.843094
Ctla2a	2.450072	3.644615	2.658014	4.624931	4.034051	4.377889	2.145993	3.874668	3.839122
Nek4	4.594866	5.265759	5.751386	5.122532	5.667186	3.78732	5.545444	5.420902	3.835302
Zfp157	2.999759	4.034203	4.170882	4.957736	3.912114	4.059635	4.634397	3.134705	3.835294
Rps18-ps3	2.539148	2.954453	3.809165	2.41972	2.418257	2.837317	3.061725	3.727006	3.83172
Snx25	4.934988	4.92185	4.799876	4.215932	4.037825	4.155967	5.100557	4.375665	3.830671
Rev3l	5.106548	4.937205	4.570918	4.601725	3.678551	2.671681	4.965827	3.008353	3.830091
Gm26165	12.05175	2.003278	3.615946	9.98687	3.825986	3.343525	11.62568	1.147445	3.828797
LOC115489086	6.427602	2.403934	2.410631	0	2.295591	2.229017	0.830406	9.17956	3.828797
Mxd3	4.014775	5.1116	5.04041	3.185335	4.393104	6.398549	4.458473	3.049835	3.826437
2700049A03Rik	5.135146	4.866741	5.337408	5.186586	4.071279	4.326152	4.918403	3.404495	3.818027
Gm8590	4.58984	6.193251	5.490446	3.44547	5.014153	4.619058	4.976369	5.955165	3.816987
Dynlt1c	2.559911	2.872232	4.000325	4.534299	7.085527	3.698945	4.878185	2.741946	3.812222
Gm5518	3.599304	4.552818	3.643622	3.055366	3.135309	3.00379	3.901527	3.343308	3.80697
Serpine2	5.152637	5.865364	4.235906	7.028424	4.085147	5.014474	4.126572	4.417803	3.805321
Zfp568	4.014617	3.712304	3.983468	3.269145	3.198775	2.572505	4.361673	2.738819	3.80369
Gm22504	0.398835	1.591093	2.393288	10.31162	2.279076	3.688301	0.412216	1.13919	3.801252
Zfp790	3.675037	3.814856	5.000632	4.338107	4.08341	3.167365	4.160081	3.237166	3.80048
Firre	4.366403	4.925273	4.776904	6.930047	4.848573	4.390569	7.508354	3.585396	3.798114
Gm10481	5.081441	4.474373	3.021748	4.597754	3.662331	3.175106	4.494889	4.140656	3.795915
Prkar1b	4.825535	6.099029	5.737939	4.953295	5.527652	3.98952	4.803563	3.599286	3.79378
Dzip3	4.605589	5.343722	5.016571	5.169268	4.918315	3.289209	6.739556	3.18019	3.79195
Zfp677	3.707457	3.98646	4.770043	3.725092	3.567722	4.249961	3.033541	4.081403	3.791189
Egln3	3.045364	1.79756	2.25839	1.545054	2.111155	2.931207	1.862828	3.077005	3.791024
Cdc7	3.83521	5.605952	3.653006	4.802471	2.976202	3.602979	4.194583	3.187814	3.790677
Gm3756	3.265238	3.681312	3.798055	1.905817	2.568946	3.446271	3.301422	3.17641	3.788597
Pdgfra	6.237616	5.256254	6.972263	8.093331	3.303884	4.688714	3.482009	4.668486	3.783183
Gm7984	3.514478	3.335519	2.267669	2.59291	3.239178	4.403334	3.04652	3.831856	3.781818
Gm16589	2.543244	1.931443	2.520205	2.250577	1.733288	2.52453	2.604455	2.79907	3.780454
Bvht	2.582805	4.783868	4.151419	1.192442	2.459835	3.156228	3.718173	2.459083	3.78038
Taf5	4.032787	3.309282	3.880094	3.925594	3.062902	2.87966	5.205712	4.293233	3.778734

Ppp1r3d	3.420212	3.020781	2.859017	2.267369	3.257372	3.603509	2.866659	3.30498	3.778734
Fbxo32	5.203313	7.405543	6.626802	4.156819	3.699556	3.762269	7.046597	4.467028	3.778453
Gm13422	5.219818	4.218591	3.960322	4.832608	5.228433	5.659387	7.813364	5.14115	3.7741
Gm14328	1.237457	0.411387	0.660054	2.707155	1.099971	2.212432	1.193708	1.885088	3.7741
Arhgap35	4.267126	5.228316	4.938545	4.54582	3.471166	3.172233	4.43888	3.772998	3.770333
Morf4l1-ps1	3.094817	3.032992	3.439085	3.569024	3.776446	3.796112	3.742862	3.979915	3.768979
A530032D15Rik	3.873402	3.621647	2.259747	3.370242	2.997301	2.462625	1.668064	4.532992	3.768606
Rpl7-ps9	3.210222	3.817382	5.742026	5.095306	4.58607	4.56725	3.445537	5.525114	3.765694
Ptprb	4.725177	3.249291	1.948986	11.21555	5.41028	5.733486	2.597385	3.53889	3.76488
Btnl2	3.812228	4.53501	4.869194	3.562508	5.030522	4.417405	4.319899	4.110648	3.764719
Tmem202	3.391898	3.937436	2.224454	2.322323	3.601108	4.165154	1.264349	1.800004	3.762744
LOC115488151	4.614121	3.575405	2.653285	5.685595	2.287068	2.093841	7.392719	3.162811	3.760087
Myl6b	3.350058	3.610586	5.403948	4.352173	4.785838	6.345963	3.462453	4.012702	3.759386
Lzts2	3.466176	4.98877	4.940394	5.531328	4.190679	4.952072	4.397638	3.892979	3.758557
Etfbkmt	5.048566	5.754429	6.732204	4.733413	3.617597	4.846613	6.162144	5.996967	3.757733
Zfp874b	3.204003	4.192226	5.335725	2.732992	4.26924	3.873596	3.114022	4.141997	3.754089
Gm31134	1.083159	2.160551	1.969609	4.21045	4.032575	3.915626	2.849634	3.375078	3.753989
Kyat3	3.478227	1.97792	2.593717	3.094666	1.946894	1.636493	2.585821	2.382038	3.751252
Arap2	6.071997	3.998308	4.009447	4.219314	3.455807	2.191955	3.357128	4.833866	3.75119
Gm16238	2.647549	3.120591	2.407142	4.467643	3.514813	3.264493	3.98018	5.728922	3.746791
Gm7868	2.456635	1.143378	3.685381	2.361445	1.559782	0.454364	4.147123	0.623722	3.746226
Lgals4	3.814015	4.956547	3.814459	4.405605	4.439632	4.524641	6.649595	4.695035	3.74525
1110002J07Rik	3.901302	3.242429	4.297585	2.192732	4.173271	1.359461	2.454377	4.145079	3.745231
Cplane1	4.746576	4.63138	4.617218	3.035431	3.412348	2.637695	4.614942	3.236102	3.744992
Ttl	5.079595	5.296347	5.323256	3.371455	3.541511	3.88832	4.898339	3.424728	3.741008
Mtrf1	4.021586	6.165484	5.484618	4.62701	4.020038	5.225093	4.15651	5.411152	3.737897
Tnnc1	3.383183	3.144118	2.537682	4.05237	4.027633	2.63092	2.225165	3.0015	3.737458
Mirt1	2.40944	1.61943	1.812528	1.395897	2.045297	1.685662	1.310102	1.705556	3.734162
Rad51ap1	5.371675	7.725275	6.440823	4.013567	3.080861	4.693106	4.26361	3.305968	3.733692
Zfp472	2.63313	3.927351	4.602582	3.420407	3.275908	4.541014	4.143501	4.675212	3.730497
Hyls1	4.79136	4.677648	4.623188	5.20071	3.920529	4.150068	4.429	4.786724	3.730455
Gm15267	1.410362	0.649203	0.759514	0.64729	4.339611	6.721947	1.569808	1.342803	3.7224
Rad54l2	5.248961	4.677695	5.374419	3.6531	3.595015	2.957522	5.615524	3.169438	3.7223
Zfp54	2.102202	1.886945	2.643075	2.657828	2.059317	2.749443	1.396755	2.687731	3.720944
Ccdc122	3.37716	3.718109	4.518025	4.535809	2.673347	3.32589	1.405252	2.756047	3.720355
Traip	5.358572	3.199986	4.135181	3.355016	4.378882	4.190711	1.914492	1.92108	3.720056
Ypel2	2.816009	4.105616	4.094433	4.981945	3.823644	3.053871	5.283292	3.381007	3.718666
Fermt2	3.333464	4.483933	3.675784	5.490654	3.842267	5.027144	3.16261	3.564404	3.714748
Lgi4	4.527705	6.642208	5.265385	4.397637	6.117217	4.67395	4.842851	3.659184	3.71317
Zfp707	3.375549	4.276443	3.52801	4.71744	4.576072	3.65593	4.557439	4.314091	3.709922
Dand5	1.764967	3.430266	3.711387	4.3652	3.965283	2.72031	3.882232	3.403931	3.709401
Egfl8	3.122595	3.401753	3.41123	5.015936	5.673334	5.331097	5.511333	4.162222	3.708691
Gm8034	1.094172	2.667523	3.039721	2.901449	4.863029	2.473426	2.51307	2.430772	3.707888
Htra1	4.350216	5.613108	5.384018	6.461735	3.366267	5.808124	2.753901	3.908854	3.705615
Obi1	6.508573	4.624294	5.069212	5.326608	5.403286	3.755169	4.673136	4.770987	3.70548
E130311K13Rik	3.663797	4.133867	3.479161	3.496071	2.431978	2.977478	4.092715	2.536941	3.703556
Slamf6	3.367733	2.731305	2.812939	2.576053	4.47625	2.327224	2.983474	3.276882	3.703556
Gm10359	3.282821	3.320195	2.913264	2.43683	2.642132	3.548952	2.198255	2.377192	3.701703
Rpl30-ps1	3.881877	2.782671	2.911746	1.930067	2.65726	4.262934	3.761357	5.428394	3.699774
Ube2t	6.198404	8.377344	4.113438	3.917107	4.137822	5.357093	4.310822	4.136557	3.69917
Gm42573	3.880664	4.312657	4.657339	4.520457	3.590305	4.408995	4.01086	5.383812	3.698618
Dhfr	4.024237	5.090068	4.150477	3.147167	2.814595	3.149698	2.97863	2.793779	3.695621
Dpysl3	3.993151	4.901563	5.201233	4.265695	3.510492	4.055158	3.44839	3.701025	3.694782
Gm20570	3.99342	1.874253	3.75895	3.620664	3.467705	6.51704	3.641822	4.808572	3.69414

Kctd1	4.187269	4.900903	4.395413	4.748813	5.042563	5.440359	4.685417	2.998255	3.694
Them4	4.364648	6.213596	5.139618	3.815175	3.653999	4.003739	2.946755	3.25073	3.690231
Spata24	1.658404	2.284079	2.211462	1.963231	1.35381	2.629097	3.836191	2.556416	3.688081
Pemt	7.477692	6.857733	5.329549	7.179399	4.911498	4.61009	4.619374	4.909997	3.68633
Gstt1	4.365202	3.1097	3.305465	3.782654	2.96955	3.575453	2.191375	2.90927	3.684948
Cd28	2.504152	2.20291	2.799838	5.222876	6.286422	4.655273	5.680692	4.095929	3.684053
Zfp931	2.711136	4.941644	4.176258	3.718551	3.413063	2.56481	3.34963	4.302112	3.682877
Npr1	4.676766	4.873947	3.494843	11.54804	5.429999	6.438795	3.991852	4.50277	3.681089
Atp6v1g2	4.155999	5.14416	6.012055	5.756288	5.265724	4.838488	8.130641	5.617411	3.678105
Trpt1	3.781489	3.700263	2.140715	3.121765	3.873257	5.080552	2.802219	4.551384	3.672097
Sfrp2	2.631992	2.182882	3.103341	5.675297	1.926186	2.510846	2.405314	2.875207	3.670364
Gm8226	4.106523	2.623738	2.37436	3.381665	2.199942	5.103004	5.239054	3.543268	3.669264
Mir7013	1.539946	0	0.770063	0	0.733314	1.424094	2.387417	1.46618	3.669264
Gm8116	3.03466	3.026581	2.701902	4.342489	1.550827	2.293001	2.677477	3.065471	3.668284
Nicn1	5.546423	7.48861	6.175037	4.91699	5.805604	5.250129	5.299863	4.309281	3.665454
Cntrob	4.30873	5.697018	5.319865	5.387145	4.370922	3.737976	4.30823	4.048883	3.665177
2310057M21Rik	3.711475	3.634898	3.561423	2.659953	3.455156	3.153965	4.216138	3.13575	3.664838
Rbm48	3.735866	4.73219	4.009022	2.928565	2.519166	3.429594	3.382076	4.751201	3.664571
Fbxl17	4.310615	4.263682	4.382227	4.211345	3.859906	3.378099	4.657329	3.240992	3.663675
Zfpm1	4.379982	4.083044	3.593791	3.626577	4.035237	4.562979	3.695453	3.812729	3.663348
Zfp595	3.176804	4.427198	3.375746	3.412858	3.52531	3.514878	2.785014	3.41621	3.663069
Fam120aos	5.502066	3.973648	5.161159	4.678859	4.445057	5.895223	5.412132	6.069442	3.652688
Ccdc191	3.181317	3.425233	3.199764	2.678188	3.460228	1.404128	3.063865	3.5108	3.652275
Gm52945	4.2098	4.657603	4.257672	5.478428	4.157624	3.0982	5.743091	3.71172	3.651076
Fam210a	4.64568	4.799401	4.921892	3.648901	3.057222	2.718966	4.783736	3.313256	3.650564
Polr1e	3.923021	3.538465	3.188802	3.263823	2.694262	3.295451	3.085401	3.065461	3.649609
Rhbdf1	3.981739	5.258486	5.973303	3.198006	4.542264	4.167751	5.291134	3.853495	3.648992
Nsl1	7.217866	7.319636	8.189287	5.66957	7.105283	8.245411	10.21835	5.197398	3.641965
Cystm1	3.726929	5.57551	3.540994	4.076657	4.081909	5.85913	1.540787	3.193561	3.640896
Gimap6	4.436421	4.630405	3.783434	5.7453	6.943737	6.64695	2.985752	4.06019	3.638307
Tpt1-ps6	2.807902	2.656815	2.592211	3.221738	4.182747	3.728537	3.646237	2.673398	3.63686
Ciart	2.980541	3.051875	3.219358	3.754204	3.519907	3.638331	5.503894	4.502591	3.636122
Fam187b	5.177095	3.155358	4.818135	3.289061	7.532875	3.391228	5.425103	4.039125	3.632143
Kctd9	4.741298	5.359165	5.602407	4.086107	2.776233	3.394008	3.466557	3.511029	3.631839
Gm32435	2.667123	7.220062	2.667433	6.8199	3.447332	4.756768	10.04192	3.264895	3.631436
2810408I11Rik	2.565857	2.869211	1.633008	1.855625	2.295591	3.091862	2.330494	1.332517	3.631182
Ccdc114	4.912233	5.39907	4.411498	4.685341	5.060264	4.913511	5.750523	5.392784	3.63078
Alg6	6.225877	5.457858	6.186941	4.968575	3.436819	4.180602	3.606741	4.455173	3.628319
Trim16	4.627068	4.758961	7.216173	3.450862	3.800838	3.476661	4.528246	4.130084	3.624475
Gm12669	3.41666	2.857957	3.527285	2.685138	3.149022	2.751927	3.303463	3.987542	3.624036
P3h3	5.081002	6.206675	5.987613	4.954623	5.739386	6.447124	5.801041	5.006366	3.6226
Satb1	1.897635	2.108879	2.318095	2.924859	3.033661	2.958216	2.689784	1.30343	3.61723
Stxbp4	5.613491	4.903554	6.194917	5.697537	2.986513	4.689963	3.921232	3.317334	3.615967
Gkap1	3.537824	4.474163	3.465282	4.533514	2.63993	3.845054	5.465928	4.410114	3.61519
Lipt2	3.698878	5.93844	5.819643	3.588448	2.921322	5.256054	5.967571	3.908222	3.611344
Gm12708	4.037977	2.684818	3.407439	5.061025	5.408051	3.384114	3.91261	5.806873	3.608017
Spint2	3.053609	3.731785	3.269032	2.822666	5.529722	5.607992	1.911415	3.480613	3.607208
Unkl	3.76732	3.336215	3.946402	4.666624	3.402371	2.44774	4.565043	2.922052	3.606067
Ctu1	3.299061	3.497358	3.899343	3.165875	2.438887	4.5443	4.458896	3.448543	3.606048
Il1rl1	4.218944	4.759855	4.620376	4.195311	5.345313	5.931763	6.175715	2.817246	3.60255
Cyb5d2	4.736501	5.375462	6.230506	3.457071	5.288749	3.344463	4.581911	4.531828	3.60255
Mpzl1	3.283282	3.912062	2.702484	3.698283	2.794895	3.708018	2.942979	2.766377	3.600032
Urb2	4.965314	4.666945	5.02308	4.09405	2.686675	2.661638	4.629545	4.192109	3.596976
Leng9	4.067645	3.009895	4.757073	3.653422	4.405091	3.822302	5.56028	4.153887	3.595444

Idi1	4.099361	3.452038	3.674383	3.345724	2.725564	2.485583	2.618081	4.105508	3.593754
Vgll2	3.534303	2.551351	3.265402	4.183935	2.50048	4.295628	3.513721	4.038003	3.593071
Haspin	3.392155	4.543614	5.029651	3.569267	3.005257	5.015486	3.669029	2.591242	3.590161
Peg3	2.627952	4.256966	5.700137	4.127906	2.399212	2.569561	1.643512	3.1236	3.589497
Gm15539	2.719603	3.338293	4.812165	7.385008	6.475301	6.287509	7.243333	5.178657	3.588955
Mrm2	2.881396	2.950358	4.034424	3.591121	3.951663	3.41072	3.891341	3.511516	3.588403
Ncapg	5.127046	6.459027	5.712373	4.39768	3.612235	4.713605	5.051149	3.796684	3.586313
Gm25500	2.818885	0	0	3.737459	2.684675	4.344694	8.740374	6.262327	3.582197
Gm14648	2.639908	2.126557	3.655682	2.827053	2.320818	3.755852	2.098828	3.28682	3.580556
Cd55	1.497662	1.836486	1.571501	2.856475	2.782566	4.223088	3.451076	2.033691	3.580213
Hspa2	3.294655	4.162119	2.811766	3.756709	3.0332	2.498371	3.859216	4.370656	3.579713
Nvl	4.6361	3.910142	4.21674	4.080907	3.570594	3.177211	4.180462	3.411859	3.578853
Hmgb3	4.959316	6.005994	4.67647	5.072437	2.93513	4.127598	2.745911	1.956155	3.578763
Tmem26	5.256301	3.239505	4.576476	2.477029	1.599008	1.552635	2.517846	1.723893	3.576879
Jam3	2.407272	4.87234	2.478362	3.872304	1.955504	1.898792	2.414858	3.168296	3.576478
Dexi	4.470168	5.254386	4.869856	5.199212	4.86553	4.318419	6.146438	3.914035	3.575749
Rpl15-ps6	3.213801	3.739452	4.352528	3.528694	4.399884	3.776945	3.806027	4.271045	3.573544
Gm8459	2.999547	3.151824	2.678479	2.077269	2.040526	1.238343	2.103695	2.855863	3.573544
Apoc4	1.712373	3.415628	2.675893	2.660595	3.975184	3.365041	2.101664	4.687246	3.570094
Znrd1as	2.780415	3.565302	3.291486	3.667645	3.404618	4.407841	1.935347	4.376029	3.569364
Lcat	3.337681	3.815936	3.582318	2.590427	3.643957	2.220834	3.996557	4.611684	3.569046
Mok	2.975896	2.824362	3.744304	5.679806	3.794185	4.216798	4.514388	3.518831	3.56824
Nphp1	4.200719	4.788041	3.572455	3.40995	2.96652	3.594002	3.455601	2.530294	3.567886
Qtrt2	3.684113	4.0261	4.390092	3.663476	3.266085	2.591458	4.719136	3.619583	3.567318
A130051J06Rik	7.001693	5.261204	3.165517	9.919139	3.1058	3.37052	14.4732	6.48366	3.565153
Zfp747	3.779868	4.841006	4.152141	3.183594	2.576981	3.26629	3.671854	4.542753	3.563178
Zfp930	3.682301	3.727862	3.238581	3.385669	3.031162	3.764628	2.734851	3.206412	3.562468
Etaa1	3.366308	3.952152	4.281276	3.729641	3.206035	3.615558	4.301117	3.369094	3.562072
Zfp704	4.12984	3.980713	4.432538	5.984773	4.732648	4.242202	7.035912	3.840105	3.560094
Bdh2	3.32931	5.634698	4.893646	1.9563	1.777569	2.285807	3.180326	4.946856	3.55775
Gm3815	4.416769	3.341732	2.894081	5.300724	3.046073	3.873223	3.935303	5.945278	3.556363
Gemin2	4.707005	4.103318	5.05626	3.016412	3.38708	3.321095	5.477545	2.854901	3.555724
2310040G24Rik	3.931063	6.835913	5.746067	7.417158	7.871792	5.686019	6.250691	7.005673	3.554516
Gm8121	4.303084	6.938132	5.236027	2.92397	5.122762	5.173164	5.559315	5.940588	3.554392
Pkdcc	4.672291	4.708141	4.115968	4.525753	7.331859	5.417776	4.628878	4.863363	3.553257
Klk1b11	2.569551	3.13932	3.276558	2.363521	2.569573	6.415849	3.452487	3.241565	3.551065
Pus7l	5.587495	4.380124	5.2202	2.126445	3.898031	3.976359	4.087622	5.035242	3.550252
Pdik1l	4.63418	5.97039	4.954354	4.241519	3.933555	3.842212	4.395811	3.733394	3.54983
D130040H23Rik	3.177978	5.378575	4.623051	3.766675	4.524721	3.562297	5.175732	4.003766	3.549009
Ptges3l	3.947777	4.106007	4.343059	2.691883	1.450216	1.616774	2.214979	3.060631	3.547577
Rps3a3	5.133154	2.943706	4.171172	1.595125	3.60546	4.331619	3.846394	4.031994	3.546955
Mlycd	2.012747	3.709305	3.413316	2.871678	2.500329	2.832452	3.436976	3.624368	3.544735
Dmwd	2.652864	2.216142	2.834586	4.058468	2.936855	3.564603	1.687303	2.309908	3.544104
P2ry1	3.365445	3.081864	3.809515	3.696465	1.879421	3.395191	3.826191	3.306187	3.542905
Gm21057	2.33647	2.542091	1.593233	1.478516	1.314908	0.58928	0.439065	0.606695	3.542737
Sp4	4.193878	4.560897	3.739281	2.948682	2.990236	2.777276	3.770224	3.501983	3.541768
Hsd17b7	5.543806	4.43958	4.834324	2.036731	2.002706	2.02039	2.455629	4.394206	3.539845
Zkscan5	3.601336	3.883578	5.425142	5.81939	4.373088	2.955732	5.04818	4.628913	3.539655
Rsl1	3.143116	4.161648	5.717884	4.580488	5.006319	3.934008	3.752655	5.133779	3.537988
Gm51701	4.975003	5.592079	5.510416	6.091261	5.000511	3.986298	5.292654	4.320109	3.536909
4930522L14Rik	2.973794	4.190321	4.311138	6.97233	5.742368	5.096462	4.596247	5.143174	3.536361
Tmc4	3.048385	4.289372	3.781381	3.571775	3.80348	3.387231	3.56586	4.027307	3.536007
Rftn2	3.308033	3.441434	3.878834	3.31786	3.422132	3.454747	2.711634	3.040965	3.533365
Aptx	3.094075	3.849659	4.085062	3.838314	3.092642	3.446785	3.261205	3.665314	3.532874

Plekhg5	4.894813	5.318491	5.083065	5.318368	5.302201	4.902579	6.028817	3.722318	3.53243
Glce	5.541478	4.272762	4.657245	3.033056	4.035845	2.971398	5.197971	3.912686	3.528411
Uhrf1bp1	4.031506	3.872334	4.886261	4.491539	3.981312	3.464902	6.427382	3.721327	3.527838
2610008E11Rik	2.738054	3.300924	3.08443	4.084074	3.023204	3.492025	3.451874	3.165516	3.527273
Gm19897	1.849077	1.536795	0.821907	1.974921	1.858876	1.963297	3.043625	1.662697	3.524669
AF357359	7.391742	9.215079	1.84815	1.837584	0	10.25348	1.909933	1.759416	3.522493
Gm22574	0.923968	0.921508	0	1.837584	0	0	0	1.759416	3.522493
Wdr76	6.253044	7.63854	7.324047	5.946762	5.096005	4.288453	7.612263	3.59608	3.519827
Cep152	4.077836	3.907936	2.813806	3.737843	3.4823	2.949428	4.01451	2.852227	3.517427
BC049352	4.315143	4.763588	3.426161	4.880571	4.297926	2.741571	4.698232	3.888904	3.516214
Cd320	3.689178	5.281254	3.262918	3.593646	2.796486	3.991847	2.126952	2.580582	3.516115
Gm5451	3.925528	4.314574	3.285007	4.620515	3.128241	4.296977	3.560425	3.966313	3.512313
Fam78b	5.263408	5.960374	5.359081	4.962186	4.232036	4.515837	5.796112	5.237523	3.510414
Gemin4	4.694871	3.896631	4.936619	3.005793	3.307569	3.761789	3.589434	3.597413	3.509212
Suox	4.224527	5.413712	6.420139	6.078344	4.630274	3.841234	3.951587	4.516508	3.508997
Ms4a3	0	0	0	0.437086	0.837242	1.625923	0.170361	0.052312	3.508529
Mmaa	3.063069	3.368537	2.935297	4.794705	3.649315	2.951106	4.249201	3.526224	3.507693
Gm2541	3.601908	4.138976	5.40349	2.335912	3.877864	3.113697	2.185093	4.845848	3.507567
Gm9625	4.687036	3.870333	3.175458	3.257536	3.215915	5.266559	2.969078	4.654454	3.506482
4930557K07Rik	2.756589	4.582083	3.216394	4.416293	2.479492	2.974075	4.748453	4.811662	3.503032
Scgb1a1	8.055103	12.6412	8.885338	5.654105	1.240993	2.081368	1.836475	9.58656	3.499913
Gm19680	1.655942	1.97466	3.348272	3.32913	3.668474	3.096017	3.088139	2.741946	3.49962
Kcne3	6.79388	8.19871	8.493337	5.877567	8.21743	5.40318	3.932216	7.438706	3.496593
Edrf1	4.199278	4.755763	3.984718	4.226066	4.023439	3.84827	5.294477	4.335316	3.495972
Rhobtb2	4.292184	4.988932	4.695453	3.920367	4.249324	4.126088	5.684891	3.935225	3.494884
Iftap	4.096763	4.922142	4.217041	3.668815	3.354103	4.320285	3.862787	3.695229	3.49357
Ung	5.728837	5.82005	5.373633	4.989076	3.185538	4.639731	4.670633	3.150687	3.493101
Pomt2	5.226892	5.476369	5.788767	4.639554	3.856667	3.297069	5.743448	3.78215	3.492428
Gm38500	3.238287	3.229666	2.605012	3.605165	3.251724	3.352724	3.41969	2.211837	3.488946
Gm7206	3.050237	2.509746	1.525296	2.50235	2.687137	1.833497	2.600872	2.178094	3.488577
Ccdc85b	2.846325	3.958621	3.734604	4.206622	4.762568	4.696894	3.441119	3.953091	3.484332
Armc9	6.151381	6.034431	6.278164	5.540329	6.218662	4.406337	5.966914	4.008491	3.484049
Llph-ps2	2.893745	3.569577	4.188802	3.861956	3.481227	3.661956	3.463066	2.900136	3.483785
Cep85l	3.16675	2.561659	2.289579	3.442496	2.636132	2.227734	3.982005	2.878353	3.481947
Rpl10-ps3	3.067719	2.549626	3.506372	2.178954	2.226028	2.566747	2.793183	2.503516	3.480725
Ccn5	0.874255	1.743855	2.468772	2.966045	0.783653	2.520567	2.019781	2.252313	3.480014
Zbtb32	9.466544	10.49038	9.467644	3.322418	5.421285	5.836242	3.069536	4.477084	3.479248
4833419F23Rik	3.272167	3.326215	4.027751	4.067298	3.595819	5.470073	3.83721	4.074016	3.478512
Cfap97	5.12493	5.446453	5.557655	4.893329	4.938081	4.506291	5.39611	4.605182	3.477499
Gm25128	5.835586	2.182519	0	7.978984	4.863029	0.674571	0	4.167037	3.476145
4930581F22Rik	2.792675	3.061462	2.146686	1.675399	2.19811	3.265574	2.194595	1.823874	3.475566
Zfp553	5.527307	4.278429	5.709464	5.397903	4.3056	4.836834	4.280297	4.131485	3.475317
Rbpj-ps3	5.211306	5.611951	5.307836	2.193656	2.618616	1.53743	3.304383	2.648255	3.473739
Phldb3	2.769474	3.610114	4.203286	4.058468	4.372891	3.819218	4.469345	4.140255	3.473098
Dusp28	2.416817	4.524103	3.718612	2.181438	2.620454	3.782302	3.420203	3.292267	3.472881
Kif15	5.931172	6.442721	5.65596	3.863384	3.240387	4.15624	4.763944	2.725033	3.472829
Zfp46	4.972729	4.368213	2.843618	4.511746	4.113728	3.569248	7.102852	2.822284	3.470986
Mir6942	2.726462	1.812802	0	2.71119	4.327754	0.840449	3.757246	0	3.464747
Scarna2	0.908821	0.906401	1.817853	0	0	3.361796	0.939312	0	3.464747
Fblim1	5.770233	5.663813	7.52409	6.10107	3.165134	3.377298	3.699081	3.790046	3.463321
N4bp3	3.973793	4.036156	4.266838	4.751537	4.225746	5.11772	3.981128	4.874371	3.462081
Pex11g	3.71237	4.854372	4.620376	5.004135	6.835534	4.195991	5.286423	4.71272	3.459592
Bcorl1	4.570812	4.540589	5.033003	4.077187	3.81874	3.189041	4.555776	3.515959	3.459232
Ralgps1	4.497021	5.050391	4.705413	3.832998	2.717311	2.393876	4.335432	2.80643	3.457662

Slx1b	4.096934	4.784752	5.23981	4.831231	5.265355	4.661952	4.045491	4.176195	3.454753
Tpk1	5.051804	5.060261	4.06388	2.730167	3.765352	3.83896	4.245137	4.224271	3.454109
Vezt	4.047558	3.379747	3.708613	3.915702	3.431814	3.207673	4.229777	3.896437	3.453363
Zhx2	3.209988	3.030873	3.460348	2.747219	2.781512	2.38453	2.175529	2.705509	3.448098
Zfp322a	3.490629	4.257503	4.246472	4.11977	3.400736	4.318145	4.625004	4.347689	3.446866
Ccdc77	4.774348	4.669179	3.337796	5.116351	5.01057	3.257794	4.527309	3.287871	3.445917
Slc25a35	4.906785	4.59323	3.917275	3.595273	4.181256	4.497837	3.603368	4.302919	3.445917
Atxn1	2.799836	3.382322	3.31944	4.078195	4.049875	3.269929	6.317385	3.673184	3.445236
Ankdd1a	3.881045	3.567127	3.615119	5.713285	2.645366	3.061265	3.85395	1.992475	3.445129
Eid2b	1.943991	2.531866	2.790524	2.751828	2.98408	2.664889	2.789259	2.395244	3.444022
Ccdc112	2.59487	2.467029	2.983235	2.532104	2.032492	1.771708	2.782187	2.90927	3.443907
Zc3h12c	3.657889	3.493185	3.315145	2.755411	1.991586	1.939815	4.389524	2.588887	3.4431
Pcbp3	4.792916	3.632919	6.327385	3.68576	4.199543	3.664067	4.524396	4.137415	3.441283
Tiam2	4.282862	3.946222	4.152902	2.637474	2.153355	2.794575	3.932216	3.725821	3.439611
Cdk5rap2	4.99442	5.164893	4.679782	3.910472	3.246516	3.300341	5.026668	3.314774	3.438382
Gpt2	5.175628	6.004599	4.829135	3.706178	3.578349	3.502481	4.663066	3.016962	3.437163
Cdca2	5.75703	7.094966	6.436215	4.838115	4.301425	4.08705	6.471068	2.823678	3.436278
Fam72a	2.497579	3.920152	2.989253	2.361445	2.807607	2.120365	4.02017	2.962679	3.434041
Ttll4	4.962856	4.970239	5.204376	4.606509	4.752792	3.399154	5.527756	4.29259	3.431085
Dst	4.937981	5.948501	4.800851	5.912299	3.707466	3.623096	3.894039	3.104501	3.429838
H2bc18	0.562252	0.785057	1.124635	0.447282	0.963869	5.095541	1.85957	1.713021	3.429608
Slc22a15	3.197226	2.456059	2.846947	1.743228	2.703136	2.408587	1.924026	2.598986	3.42914
Ubxn11	5.120615	3.925201	4.570997	3.198238	3.707994	4.265759	3.761548	3.70686	3.428381
Meg3	5.361235	5.520376	3.767792	11.46929	6.044378	7.369854	6.080238	5.65625	3.424902
Cmss1	4.241436	5.971968	4.291834	3.274902	3.564262	4.429945	4.177442	3.800718	3.424206
Myot	4.62204	5.716071	3.80372	4.307248	2.968192	5.642099	1.992737	2.28833	3.423481
Ubox5	3.337855	4.409034	2.804124	4.322281	2.93875	3.1279	3.603166	1.850282	3.421635
Zfp324	2.819558	3.445753	3.00523	2.566826	3.013101	2.423816	3.817131	2.129952	3.419039
Gm8738	3.447641	3.576001	1.241295	2.60553	1.444738	3.060739	4.561035	8.403179	3.417344
Gja1	6.527503	8.133097	6.601407	4.000018	4.701723	3.601568	3.080335	4.804737	3.41561
Zfp738	2.670972	3.203999	3.619157	3.586227	2.743089	2.11717	3.091344	2.812565	3.413784
Alg11	4.289167	4.460754	5.058136	4.34497	3.516994	3.987699	4.113034	3.363053	3.410275
Rnu7	3.576649	4.458909	4.471331	3.556615	2.554771	0.826893	0.924161	5.959311	3.408864
Lif	5.233132	3.745706	2.955199	2.11448	2.906225	1.608889	2.911281	6.402257	3.40844
Snx33	4.743297	5.04108	4.457474	4.617688	3.687484	3.016406	5.108311	4.800561	3.40542
Selenoi	4.794329	4.764991	4.595445	3.164545	3.276172	2.528023	4.147922	2.855886	3.405288
Sh3bp4	3.687447	3.712	4.411664	2.798641	3.369658	2.730153	3.288758	2.559283	3.404979
Tuft1	2.943997	2.374835	2.792792	3.422599	2.927529	2.021869	3.132261	2.720533	3.404206
Rab26os	1.127554	4.873058	1.127685	0	2.14774	2.085453	1.553844	1.431389	3.403087
Gm13408	2.612598	2.859852	2.230526	1.710854	2.913026	1.944625	2.239232	3.579501	3.401028
Laptm4b	4.968092	4.226209	4.910215	5.608652	4.425397	3.756545	4.017203	2.949363	3.398083
Gm8283	3.332029	5.271216	4.079337	7.197998	3.118674	4.51578	3.621946	4.813324	3.394734
Zkscan1	3.683617	3.593147	3.742872	3.53869	3.102093	2.733354	4.339146	3.101145	3.391684
Gm9294	4.557408	3.673579	3.371625	4.780202	2.913436	4.56095	2.83909	4.457979	3.39159
Nipsnap1	3.020462	3.828285	3.901884	3.566707	3.416028	2.851421	3.284392	2.516304	3.388551
Ndrg2	4.36344	6.527735	6.014414	5.395933	5.754036	6.673555	3.20892	3.941375	3.385646
Mrnip	5.543806	5.335045	4.912189	5.512752	3.797794	3.372854	5.478493	4.954144	3.383447
Il23a	5.833439	6.428178	2.692669	1.460332	3.690852	2.753877	1.138371	7.068734	3.382527
Mtmr11	2.957222	5.348154	4.081441	3.822855	3.43604	2.497735	4.584655	3.247286	3.382195
Atp10d	4.069858	3.876184	3.309436	4.50281	3.919738	2.68713	5.125367	4.36363	3.380964
Gm8330	6.628958	7.924515	3.678137	2.934717	2.118863	1.931449	8.306099	2.636962	3.375362
Gm18101	2.124451	3.430431	2.832931	2.112551	3.853913	1.871072	3.032194	2.311641	3.374651
Slc35e2	2.826568	4.16651	3.929208	3.659259	4.07185	3.674286	4.795504	3.90135	3.371699
Rpl35a-ps2	2.778217	3.022714	2.273351	4.269558	1.683782	1.751733	2.08831	1.322568	3.370039

Gm42759	1.290562	2.910025	1.62742	1.115942	1.282557	2.075603	1.449848	2.40406	3.369186
Twnk	4.138973	5.483851	5.891923	4.296041	5.956028	3.520267	6.088889	5.551508	3.368924
Gemin8	3.730263	4.868094	4.484774	2.88068	3.401485	4.293689	3.609323	3.400445	3.366162
Gon7	4.102989	5.471949	4.437469	3.985122	3.589578	3.926676	2.416181	5.132891	3.36486
Tcim	2.904642	4.26749	3.904543	6.987996	3.123298	6.036554	2.098237	3.746812	3.363733
Nlrp1c-ps	3.159184	3.51863	4.811499	4.799938	4.276427	1.705452	7.392222	4.27512	3.362519
Ankrd39	2.863848	3.367337	2.502389	3.297459	2.038445	2.843542	2.399101	2.296138	3.361596
Fam219b	2.858583	2.832579	3.725812	3.392745	2.652225	3.052848	4.193467	3.090391	3.357267
Gm5641	3.302069	3.586013	3.55931	2.079596	2.585769	3.121509	2.388997	2.410318	3.356976
Serinc4	2.543629	3.187333	3.978959	2.788804	3.41638	1.749123	4.449022	2.359687	3.356729
Cep76	4.315916	4.48063	4.707671	4.16625	3.245077	3.687797	2.947723	3.664615	3.35571
LOC115488834	2.639908	11.40914	8.800715	7.000321	7.542658	4.06884	7.275937	4.189085	3.354755
Glud-ps	4.772494	4.471316	3.32667	2.684471	3.443387	3.343525	4.135421	3.442335	3.354026
Gm8069	2.163437	2.966806	1.71292	0.986021	1.545325	3.417826	1.397512	2.574755	3.350664
Aplnr	2.488802	2.389094	2.084614	7.764876	6.059099	6.09915	2.234686	2.725021	3.35052
Fbxo36	3.757469	4.668973	5.236426	4.042685	4.223888	2.620333	3.565209	2.932359	3.346369
Asrgl1	2.608563	3.01496	3.218415	3.53941	2.205746	2.705403	2.796868	3.24958	3.345904
Cma1	2.187006	3.398589	2.848525	4.501238	6.975228	13.07553	0.893639	2.324366	3.344753
Abt1	3.160069	3.486675	3.322191	2.523792	3.37693	3.739206	3.81901	3.518042	3.343839
Mri1	3.534209	2.315562	3.379819	3.180927	3.513355	3.387608	4.399335	3.880703	3.343828
Fth-ps2	2.262778	1.241215	2.489345	1.575072	0.862018	0.523137	2.338694	2.369825	3.342774
Hspb7	5.34753	7.830153	6.229495	6.034552	5.55072	6.667723	3.022221	4.404895	3.340515
Dlc1	5.140411	5.149715	3.919154	6.624472	4.083385	5.307933	3.04954	4.27966	3.339411
Gm9450	6.52769	5.755493	6.528449	4.515565	3.6941	4.636811	6.746693	5.674566	3.33615
Slc4a11	6.6616	5.85561	5.533158	1.656071	1.747408	1.670628	1.837971	2.042499	3.335966
Pvt1	4.16081	3.442392	3.641132	4.584165	3.647494	2.732803	3.665118	3.241226	3.334727
Cdh23	3.661004	2.868845	2.321085	5.558259	3.953669	3.204212	3.243283	2.085157	3.333492
Kif9	3.091116	3.062737	4.000733	4.761379	4.117675	3.493805	4.552101	4.193359	3.331221
Rpl3l	2.629685	3.157926	2.522645	2.881787	2.760043	4.814072	1.331222	2.401526	3.324715
Gm14048	4.601285	4.107927	5.937832	3.689928	3.569384	2.505033	4.257081	2.685052	3.324441
Dbndd2	3.146191	3.369674	5.425098	4.931733	4.29533	5.002046	4.821554	4.116944	3.323571
Porcn	3.481197	5.989078	5.135362	4.240579	5.884931	2.816889	2.923368	3.480163	3.31789
Fut4	3.010965	3.455654	3.525811	3.249876	4.006008	1.637086	3.768783	3.500584	3.316758
Spef1	4.064805	4.493572	3.306099	2.654108	3.24161	2.694708	2.378981	3.077423	3.314006
Styx	2.629988	3.181345	3.046974	2.731777	2.293974	2.468251	2.946985	2.565987	3.313195
Acpp	1.953947	2.114596	2.411535	1.963673	1.841121	3.171773	2.492152	1.227039	3.308529
Actg-ps1	4.760335	4.401478	4.711295	2.218908	2.644653	3.118231	4.458789	3.352017	3.308245
Slc9b2	8.288464	8.581035	6.826587	2.138937	1.44766	0.795666	1.4821	2.047949	3.307463
Esam	5.92057	3.548849	4.037221	9.485265	6.749336	9.180567	2.874172	4.412774	3.3059
Serac1	4.717255	3.936583	3.988813	4.171147	3.759166	2.708994	5.955808	3.064028	3.303157
Traf3ip2	6.43672	5.454232	5.05801	5.101281	2.857716	3.088127	4.151667	3.202428	3.298013
Mcam	3.597279	2.650913	1.958746	6.617688	5.519681	7.521927	2.850441	4.775919	3.295195
Gm11423	3.210222	3.817382	1.234844	4.542803	4.233296	3.539619	7.018687	2.93889	3.294983
Arv1	4.622125	4.254165	3.82707	4.268905	3.461561	3.006029	4.11094	3.891434	3.294167
Zfp341	3.817385	3.91012	3.680249	3.984092	3.832157	3.132653	3.323426	3.094258	3.294143
Tmem186	4.092873	2.921225	4.481344	4.166391	3.953429	3.89259	4.571009	3.490513	3.290783
Aldh1a2	4.432107	3.443444	3.25712	3.77419	6.576505	4.551565	3.922787	2.914226	3.290668
LOC118567760	2.710305	2.334487	2.34099	3.797674	2.698595	2.620333	1.018631	2.697771	3.28766
Zfp658	3.525343	5.102226	5.41162	4.092579	5.137752	4.3064	7.049976	3.96532	3.281826
Gm14199	0.602588	1.030257	0.258282	1.369628	0.573898	1.114508	0.088972	1.311366	3.281826
Zfp866	3.347059	3.165485	3.510972	3.548284	3.682345	3.184199	4.204902	3.543861	3.281712
Snord15b	3.058652	1.906568	1.529504	8.744366	8.010823	1.060705	1.185476	4.004187	3.279563
Gm46046	1.99577	2.875105	1.774224	2.205101	1.90075	5.331808	2.062728	2.322429	3.275919
Gm51534	3.641319	3.325589	3.498528	4.210848	3.99399	4.010585	4.376637	3.973293	3.275529

Zfp141	2.978321	3.676228	2.875446	3.621409	3.08928	3.354196	3.154438	3.046222	3.274232
Fam126b	4.510897	3.911453	4.008556	3.942782	3.488895	3.188435	4.528606	3.638284	3.272386
Gm14325	2.258401	3.221293	3.205031	2.584495	2.619503	3.056908	2.529771	2.642727	3.270772
Rpl17-ps8	2.425785	3.009407	2.071033	3.235874	3.54996	2.298004	2.812922	2.53491	3.270618
Btbd3	3.623165	3.359727	3.21154	3.831815	2.7236	2.745466	3.431618	2.630471	3.268412
Lrp8	4.162966	3.410475	3.605845	2.337791	2.619575	2.509232	3.188558	3.671593	3.268019
Atad5	4.222286	4.375111	4.688927	4.457641	2.297862	2.38335	4.60481	1.957807	3.266407
2010315B03Rik	3.088118	4.872987	4.907717	4.837593	4.431779	2.992716	3.475919	4.108212	3.265799
Tnfrsf26	2.402254	3.547356	3.668985	1.592532	2.997301	2.152645	1.67448	2.07442	3.265731
Gm51814	1.845007	1.840095	0.878677	2.271499	0.585721	1.706205	2.179322	1.756627	3.265703
Usp28	3.93343	4.01864	4.057249	5.601338	4.411849	4.005066	5.45358	4.240866	3.265599
Parpbp	5.543806	8.200385	7.662555	4.397814	2.313647	4.665908	7.081776	3.854899	3.265232
Trmt13	4.586712	4.361024	4.556664	4.317765	3.436423	2.120824	5.372676	3.551826	3.264086
Plekha5	3.424468	3.630303	3.005739	3.691044	3.02195	3.133622	2.982466	1.402212	3.263822
Gm32633	3.14738	2.423088	4.307442	1.81196	4.364825	3.012725	2.282789	3.36462	3.262867
Snord32a	8.897467	7.508583	8.214001	2.04176	4.562842	4.430515	6.366445	7.819625	3.261568
Tstd1	1.307976	1.806222	1.811254	3.201599	4.503692	4.931346	3.951587	5.07708	3.260384
Gm12791	3.835338	3.060102	4.60294	4.923339	4.78176	4.127186	3.747794	5.44423	3.256645
Mphosph9	6.041585	6.586012	7.126285	4.411	4.320233	3.471036	6.493221	3.592724	3.252121
Jade2	3.762264	6.345713	6.059055	5.309188	4.382319	3.965292	6.423629	3.819091	3.251803
Mybpc2	4.910491	6.743485	3.947208	6.20641	3.190644	6.25281	2.90917	3.116846	3.251308
Zfp26	3.076979	3.731366	3.855413	4.233224	3.596499	3.354772	4.065611	3.412301	3.249225
Arhgef15	4.961834	3.653657	2.666716	9.798912	4.129376	4.288363	2.132799	3.178869	3.248472
Ccp110	3.7212	4.045644	3.520463	1.866846	1.990191	2.831539	3.199264	2.436441	3.248419
Acvr2a	4.515793	4.783074	3.921144	4.22362	3.678455	2.212127	3.786902	3.177391	3.247426
Lrr1	3.833106	3.681312	2.910659	2.964604	2.704154	2.494444	3.448151	2.703327	3.247369
Tnfsf13	3.076217	3.199986	3.738204	4.243109	3.402297	3.823641	4.410169	5.038899	3.247167
Gm33144	6.916853	4.736241	6.607911	3.798358	2.949643	3.91427	3.734507	2.653867	3.246991
P2rx1	1.294539	1.064585	1.635397	4.381294	4.174572	5.838727	6.103024	4.06518	3.24687
Wdr34	5.031457	5.752412	5.062725	5.796475	4.441277	3.972017	6.246656	3.7973	3.245684
Fsd1l	3.531087	5.911402	4.616171	2.006461	1.369209	2.052561	2.346142	2.569483	3.245245
Efnb1	2.591952	3.490643	4.226859	4.662364	3.191816	2.992379	2.61066	2.876472	3.241365
Nemp2	3.35019	3.475593	3.586298	3.247719	3.270852	3.160425	3.04499	3.061479	3.241164
Gm8623	3.400476	4.008045	4.637552	4.201168	4.023685	4.574042	3.40806	3.139478	3.240956
Mill2	5.317173	7.476384	6.363913	8.147779	12.12043	7.738473	6.216293	7.220243	3.240029
Mccc2	4.097823	4.321195	3.941676	4.64587	4.002154	4.658477	6.069704	2.808107	3.233927
Gm6139	2.573075	3.149457	3.099746	2.267905	3.118905	2.487658	1.631905	2.616852	3.232668
Parp16	3.842242	4.712662	4.200703	3.702064	4.522997	2.537989	4.51379	3.521861	3.229837
Pafah2	3.308559	3.515806	4.49071	3.348777	3.207304	2.932167	3.704525	2.193801	3.228431
Dok4	3.316807	2.610376	2.256594	5.47461	3.653139	3.881047	2.168789	3.394233	3.225726
Zfp275	5.004749	4.24381	5.589653	4.614971	3.538105	3.017528	4.785278	3.673467	3.225508
Plpp7	1.915874	3.415	2.568385	3.648145	4.542233	4.749772	4.086696	2.095775	3.224635
Gm24175	2.505675	2.93633	3.884248	3.301422	4.534117	3.591613	3.496149	3.757396	3.223977
Ankrd22	0.790166	1.733738	1.517295	1.100036	0.421426	1.782959	0.130668	1.083335	3.223262
H3f3a-ps2	2.039348	3.318499	3.274071	2.24139	2.862267	2.382221	1.941365	2.401526	3.222416
Gm24119	5.183234	3.596128	2.028458	2.465052	2.575542	1.667232	6.288805	3.862132	3.221793
Gpx7	2.60294	2.095026	2.374888	1.453114	2.826943	1.815896	1.038349	2.086951	3.220731
LOC118567792	1.630531	2.206973	1.747201	2.432097	2.662114	2.692615	4.092715	5.655265	3.219085
Rab4a	3.337277	4.581434	4.947598	4.060384	3.926242	2.940976	3.124608	1.94383	3.218142
Stau2	4.293324	5.695334	4.877449	3.730434	3.949971	3.816144	3.963471	3.035984	3.217917
Dsn1	4.808032	4.577266	4.395732	2.873489	3.284013	3.390878	3.814847	2.473817	3.216994
Gm46287	2.010707	3.15127	2.370037	4.284523	2.051759	1.992256	2.671928	1.982761	3.216785
Gm13597	3.595263	3.320084	3.329334	3.045476	4.311816	5.623404	4.037009	3.80338	3.215086
Gm7743	3.27273	3.857475	2.529222	4.240579	3.730849	3.806076	3.587532	3.257594	3.213724

Ganc	3.187286	3.385365	4.798746	3.260968	3.287584	3.554028	4.87592	4.031538	3.213233
Gm4708	3.587169	3.360793	3.587586	3.026609	4.96928	5.528836	3.932216	3.311841	3.211685
Rpl9-ps1	1.363889	2.080394	1.444285	1.116911	1.910224	0.964509	1.575488	3.360968	3.211535
C77080	6.803456	8.823186	6.736878	3.248706	2.352307	2.969314	4.931481	2.704327	3.210049
Mmab	4.527967	4.398106	5.020721	5.520583	4.443633	3.622944	4.639185	3.879961	3.208521
Gm19221	3.081838	4.470739	2.171547	4.666512	2.501513	1.910787	5.248396	2.434062	3.204289
LOC115489589	16.37943	7.120743	6.720546	6.264491	5.599852	8.156175	9.115592	10.39655	3.202267
Trim2	4.418053	7.456799	3.993704	4.224331	2.022935	2.592833	2.502671	2.143656	3.198586
Gm15361	4.006642	3.189096	3.043861	2.873221	3.155441	2.600778	3.026163	3.007757	3.194478
Paip2b	3.984871	4.755812	4.568961	4.128347	3.509321	3.546315	4.359818	3.603495	3.19408
Acsl3	4.363786	4.759675	4.266219	2.161545	3.190953	2.463615	2.41557	3.75017	3.193298
Gpr18	2.725186	3.261517	2.881245	2.864773	5.302107	4.572302	3.742075	4.15143	3.191023
Sntb1	2.394677	2.824873	2.781239	2.509288	3.384212	2.571703	2.262114	1.912231	3.190368
Gm25099	4.779143	2.859852	3.823759	0.950475	8.192887	6.187443	9.878966	3.64017	3.188464
Gm23287	1.433743	3.813136	3.823759	0.475237	0.910321	1.767841	3.951587	5.915277	3.188464
Orm1	0.640491	0.496834	0.142348	0.70767	0	2.500848	0.882639	0.135513	3.187879
A530010L16Rik	2.600982	4.298723	2.601284	2.290822	3.680332	1.786799	2.304209	2.051865	3.187243
Rpsa-ps1	1.941411	1.990027	1.510162	1.501528	1.643536	2.742905	2.898343	1.643034	3.186691
Zfp764	3.746725	4.542277	3.724722	5.153565	3.055524	3.029153	4.289814	4.079908	3.186067
Epm2aip1	3.166141	3.439923	3.671315	3.825237	3.423278	2.949165	3.96794	4.121241	3.185259
Pir	3.470301	3.936958	4.034694	1.984246	2.106987	3.69061	3.003886	2.188944	3.183474
Zfp213	2.508603	2.977497	4.167669	3.154268	3.31719	3.220988	2.95704	2.605567	3.181309
B230354K17Rik	3.576197	3.664585	3.071679	2.942552	2.724745	3.04777	3.971579	2.790674	3.181204
Gtf2ird1	4.010709	4.225141	3.924353	3.055926	3.092183	2.761663	4.611834	2.380418	3.177194
Spsb4	4.893055	3.492812	4.42165	4.001191	2.980566	4.272282	3.516947	3.192488	3.172138
Magi3	3.167408	2.722096	3.530048	2.529785	2.08595	2.041036	2.67292	2.005108	3.171368
Zfp958	2.14913	1.823674	2.826257	2.76287	2.352133	2.547448	2.454402	2.588817	3.16866
Tle2	4.546508	6.874741	5.451554	5.080094	4.376604	3.797584	6.568554	4.328721	3.16838
Smpx	1.98376	5.347241	2.305719	3.945297	3.165874	3.718621	1.052865	2.603391	3.1682
Cdin1	3.626036	4.741479	4.029397	3.004771	4.009778	4.135668	4.101638	2.97285	3.16794
Srr	5.088043	5.329202	5.167224	5.274427	4.639991	4.505426	4.507497	3.198371	3.164252
Gm16104	4.147486	5.791022	5.66889	13.33509	5.135026	4.091163	7.430165	5.791593	3.162338
Zfp949	2.733261	2.669584	3.072919	2.230593	3.087848	3.085456	2.766514	2.297231	3.16198
5830408C22Rik	0.789716	1.575227	2.843308	0.157058	1.35381	1.022426	0.816211	0.902264	3.161212
Gm38543	1.876207	2.973433	1.645085	3.705837	2.423302	2.234184	3.40016	2.447031	3.159956
Zfp266	3.348904	3.877559	4.179989	3.391652	3.18946	2.647535	2.958966	2.793079	3.158117
Xylb	4.70601	4.546152	3.82012	4.763589	4.220673	4.449548	4.536728	3.435783	3.157774
Fbxo48	5.025657	5.517391	4.558684	3.25419	4.823485	4.971819	4.952674	2.670652	3.156134
Dcaf1	5.10185	5.098423	5.520008	4.101148	3.559347	3.107685	5.304591	3.325567	3.154327
G2e3	3.316509	3.533994	3.875529	2.794563	2.70144	2.655379	3.833698	2.933283	3.152609
Pdgfc	4.408593	3.43897	4.283131	5.808666	4.213692	3.654676	6.67202	3.582792	3.151336
Smpd5	1.695354	3.254867	3.391101	2.908103	2.66415	2.430105	2.540737	2.582628	3.150854
Etv1	5.279119	7.102733	7.107895	2.544795	2.715834	2.217845	4.141296	3.327621	3.149895
Dcbld2	6.401156	7.481835	7.647518	4.550757	3.117159	3.508896	3.652224	4.136557	3.147055
Atpaf1	2.824263	3.44972	3.522805	3.376443	2.780465	3.228055	3.640572	2.900468	3.145443
Aldh6a1	3.698102	4.455948	4.167124	4.87545	3.553905	4.193619	5.25765	2.708427	3.141856
Fth1-ps	2.702688	1.577849	2.571148	2.425349	1.946203	1.402081	2.657101	2.133893	3.141344
Gpr162	2.689009	2.823	3.356597	3.156463	3.21567	4.001174	2.862811	3.07994	3.140954
Nek2	4.040372	6.55461	5.98322	1.845056	3.43513	2.742172	3.602408	1.56845	3.140164
Srgap3	8.236288	6.688837	7.491971	5.187096	4.202224	4.062219	6.445265	4.144927	3.139966
Cdc42ep5	1.004644	3.188083	3.196965	1.907213	2.957418	3.547331	0.849559	3.304339	3.133684
Bend6	2.988575	6.482845	7.248136	2.451763	2.9886	1.796431	3.01162	2.560875	3.133215
Tie1	5.102188	3.976809	3.530496	8.299684	5.567061	7.45419	2.570212	3.619525	3.132921
Polr3h	4.674968	3.606857	3.440471	4.232145	3.465302	3.018128	2.643822	2.51945	3.131572

Klhl15	3.167219	5.33484	5.021212	4.129315	3.195176	4.209002	4.243399	2.814469	3.130443
Bmx	2.25073	1.416761	1.180848	1.320859	0.913653	1.398977	0.762702	1.282236	3.129803
Nlrp12	2.100218	1.979677	1.306385	0.827741	1.914849	1.622461	0.847095	1.524095	3.124596
Pif1	3.676869	5.372699	4.874556	3.259468	2.986039	4.349161	2.474567	2.496651	3.124062
Gm30368	2.958575	3.126335	4.825856	4.272909	3.220245	4.592567	5.897253	5.331901	3.121905
Map3k6	4.925838	4.77236	4.18425	3.409688	4.008941	4.401446	4.932415	3.264644	3.109517
Sdr42e1	2.731669	2.93559	3.176729	4.000852	3.18647	1.860352	4.070828	1.834685	3.108082
Gm22249	5.299227	5.691666	5.707523	5.269543	3.494025	3.392695	10.11141	11.25509	3.108082
LOC115487216	2.371682	2.069697	1.8778	0.884399	1.411727	0.913857	1.12349	1.505382	3.108082
Zbtb39	3.855026	3.168455	3.158702	3.593265	3.848424	2.224908	3.696336	3.015888	3.107561
Pdp2	3.023355	2.640857	3.626472	3.075194	3.180525	2.841589	3.676222	2.944379	3.107528
Cgnl1	5.711204	5.008551	6.040136	3.639265	3.258884	3.437556	5.249773	3.296951	3.097041
Snora73a	4.597303	2.966806	2.434149	1.344574	3.348204	4.751611	1.677015	3.089706	3.092921
2210408F21Rik	3.708232	4.715408	2.132481	5.346817	4.414599	3.772182	4.120091	2.559685	3.09249
Lrrc56	3.555545	3.74466	4.238703	3.394204	2.76319	3.314361	4.057016	3.439391	3.09054
Dynlt1a	2.869999	2.974169	3.442156	3.032293	3.54482	2.954743	3.441356	2.508368	3.087969
Pxdn	3.100459	2.953748	3.387761	7.822771	4.045836	4.955562	2.458362	3.2956	3.087327
Lockd	3.333904	3.325028	2.667433	1.515533	1.814385	6.078092	3.051956	3.899736	3.086721
8030453O22Rik	4.725258	5.306401	4.688596	2.626882	3.578966	4.300955	5.26834	4.924002	3.085137
Plekhf1	3.627122	5.213406	4.552211	3.394639	3.7931	2.926746	2.16843	2.979382	3.084159
Ctnnd2	4.615903	2.602043	3.294084	3.967867	3.575374	3.668194	4.002075	2.630132	3.082932
Pip5k1b	0.926481	1.289323	1.098978	1.349799	1.580059	1.793263	1.581095	1.620604	3.080299
Ndufab1-ps	4.064069	4.157178	2.709694	2.27971	2.778874	3.854691	3.23109	4.067822	3.078871
Brwd3	3.2484	3.332456	3.429809	3.171827	3.047149	2.194201	4.297856	2.510567	3.077363
Cenpu	3.265582	3.256888	2.804885	2.979866	2.707621	2.55804	1.985378	2.77995	3.075774
Wars2	5.30042	5.861689	6.202572	4.625334	3.931994	3.817961	5.70185	3.724812	3.075738
Vsig10l	2.909515	3.686031	3.633385	3.33111	3.340167	3.35965	2.714544	3.039671	3.072813
Plxna4os1	2.933077	3.967938	5.924924	5.371252	7.135694	5.478547	6.183022	6.801722	3.072264
Gabbr1	2.083943	2.568948	2.123021	2.252467	3.118853	3.650841	3.09032	2.15665	3.071798
Knl1	4.82764	5.37187	4.977835	3.560774	3.125358	2.92403	4.536006	2.288696	3.070615
Gbp4	1.945873	1.156704	2.049204	6.894144	4.528751	3.563214	2.97012	4.196096	3.070514
Zfp846	3.400738	4.17438	4.809886	2.901449	3.296319	2.679675	3.806002	4.176616	3.068597
Ipp	2.538948	2.218928	3.272221	2.47267	2.592566	2.154295	3.246346	3.090192	3.068461
Synpo	5.866855	5.579659	6.053218	5.132435	4.962762	4.795944	2.750407	3.653174	3.067141
2310010J17Rik	2.047844	3.063588	4.973913	2.327283	3.900688	2.434863	3.174823	3.620961	3.067079
Rgs12	3.542498	3.411237	3.522548	4.21099	2.792141	3.74668	3.556138	1.91901	3.06585
2610318N02Rik	5.487044	5.378083	4.115761	3.480748	2.92825	3.718198	5.622244	4.18837	3.065651
Clspn	5.757074	6.61347	4.254203	3.383905	3.307543	3.459498	4.023026	2.352298	3.065613
Tgm1	5.795982	3.711723	5.898352	2.871647	5.771821	4.19392	5.086617	5.402168	3.062476
Slc2a4rg-ps	3.063389	3.314466	2.952336	4.412417	3.448	3.725728	4.029665	3.37624	3.061242
Tnfaip8l1	4.547562	4.582212	4.829416	3.379912	3.817573	3.164921	4.966635	3.325407	3.060771
Yae1d1	4.388578	4.344711	3.501588	4.428171	3.180824	3.521276	3.185075	3.056959	3.06014
Trgc1	0.535116	0	0.856286	0.85139	2.242412	0.692802	0.774297	0.815173	3.060081
Xcl1	0.64214	0.960645	1.284429	4.150528	3.465545	5.047561	0.331842	1.426553	3.060081
2900026A02Rik	4.966445	4.79222	4.663113	3.597737	3.726109	3.609266	3.19958	2.820851	3.059103
Vmac	4.071407	4.783683	3.43042	2.523443	3.585418	4.022994	5.130304	2.708155	3.056489
Gm13453	2.717552	3.84865	2.935297	3.513029	2.381114	3.015729	2.977249	3.622326	3.056281
Trim7	3.530828	3.966666	4.708318	2.824983	3.942502	3.978289	2.726479	2.782092	3.05575
Csta3	0.942824	4.889634	3.771735	0	1.795871	1.918167	0	0.718129	3.055224
Dqx1	2.690334	3.282604	2.633399	2.675265	3.570788	2.567339	4.880832	2.67046	3.055131
Grap2	4.69884	3.864167	4.534495	4.590545	4.455496	5.669906	4.100322	3.345506	3.054355
Ppp2r3a	4.287769	4.729555	4.008598	2.467878	2.796395	2.618322	2.829977	2.507112	3.053864
Cmah	2.604991	2.6173	1.775459	4.298143	3.271198	2.177035	2.433121	1.984165	3.052909
A730063M14Rik	1.011029	2.016674	2.359341	0.502683	2.407231	2.804902	2.612371	2.887795	3.0514

Wdr86	2.941011	2.236551	1.985413	3.728785	3.046073	2.617764	5.053471	1.540072	3.048311
Gm24430	0	0	0	0	0	0	1.652827	0	3.048311
Rhbdl1	3.44916	2.54415	3.844823	3.679932	3.14807	4.286126	5.792929	3.1129	3.047653
Asah2	3.758708	4.448	5.529506	3.634782	2.835339	3.869237	3.564048	4.705881	3.045573
C1qtnf3	2.058148	4.131653	4.670956	2.204051	1.0806	1.756902	0.92724	3.341299	3.042992
Plcb1	3.976837	2.101638	3.16124	2.867451	1.508963	1.663003	2.292575	3.816509	3.042794
Trmt10a	3.727116	3.405573	3.214174	2.352463	1.721694	3.776945	1.89148	2.379886	3.041766
Gm35413	0.455655	0.908884	0.455708	0.906206	0.867922	0.98321	2.040751	2.89219	3.03996
Frmd6	3.710141	3.24959	3.448929	3.417386	2.208436	3.387034	4.449137	2.807819	3.037414
Dusp19	3.789341	4.610689	5.760468	2.373913	4.005909	5.256396	3.916473	4.437624	3.033726
Gm22358	4.998514	4.532006	8.6348	2.71119	9.088284	4.622469	6.575181	3.461146	3.031654
Prss46	2.822622	4.222661	4.41086	2.192821	1.72215	2.691844	3.646651	1.721624	3.026486
Gm36470	3.389704	1.908448	2.460555	1.848457	1.874507	1.719025	3.051373	2.862954	3.022258
Rwdd2a	3.54356	1.95307	3.963653	3.106429	2.753165	5.562231	4.144347	4.261663	3.021819
Endog	4.727277	7.286341	4.298024	3.258507	4.706852	3.626472	4.719312	4.245102	3.020743
Zc4h2	3.571781	3.813136	4.175947	3.526761	2.946565	3.442637	2.911695	2.993561	3.02065
Traf3ip1	2.439275	2.653943	2.645495	3.118643	2.564504	2.724495	3.192317	2.910576	3.01928
Gm24457	0	0	2.640215	0	0	0	0	0	3.01928
Ropn1l	2.375917	3.888561	4.02125	3.695362	4.641636	4.168996	5.226082	4.176195	3.01928
Zfp65	3.825731	3.773617	4.919368	5.058466	3.136419	2.902905	3.997535	4.042742	3.018517
Nid2	3.359883	3.158485	3.076466	4.323063	3.005326	3.600474	3.988805	3.361046	3.018353
Fndc1	4.089928	3.679693	3.8425	7.138612	3.277781	3.746971	3.320624	2.786624	3.016684
LOC118568697	2.399287	2.537923	2.47228	4.012561	3.081361	2.319639	2.89308	4.014929	3.01433
Gm12346	3.031769	3.239676	3.032121	2.562569	2.474935	3.444527	2.820136	2.948396	3.013383
Depdc1a	4.004354	5.480235	3.693334	2.521885	3.411146	2.592171	2.184314	1.842727	3.010799
Dnph1	5.764356	5.04913	4.662151	3.638616	2.721085	2.827586	4.869812	2.052121	3.009725
Gm25861	0	0	0.450768	0	0	2.500848	0.931675	0.858252	3.007006
Cenpq	3.406057	4.603815	3.092701	1.960882	2.817064	3.094555	2.316007	2.617077	3.004228
Zfp606	1.957761	2.381157	2.817593	2.967675	3.16064	3.709268	1.850711	1.591203	3.003676
Tnni1	1.922707	3.94171	3.311714	2.071265	2.390688	4.050025	0.828006	2.695059	3.003282
Gm31619	2.570873	1.657943	2.37785	2.229705	2.743024	2.788603	2.677103	2.282087	3.002962
Gm38935	4.829831	5.23584	3.255265	4.280736	2.099944	1.262265	6.402618	3.698772	3.002125
Zfp583	1.523795	2.219617	2.466428	1.535197	2.00501	2.466026	2.528158	1.813503	3.000171
Tmem204	1.900912	2.362044	1.527139	2.355083	2.047838	3.515807	1.578191	1.335138	2.999762
Cmc4	3.308531	6.19704	3.631736	3.931949	5.533478	4.552126	5.254402	6.0696	2.999503
Gzma	1.824862	0.815864	2.454411	8.322316	5.034146	9.194378	2.146236	2.39648	2.998717
1110065P20Rik	5.29077	5.429631	3.450903	4.574898	4.016491	3.403644	3.011513	3.942259	2.996312
Slc25a27	4.026502	4.897296	4.02697	6.298893	4.115392	2.99703	8.22168	5.703686	2.995211
Adgrg1	6.3967	5.032309	5.046329	8.319304	5.673602	7.255103	3.095371	4.742054	2.994016
B230398E01Rik	4.154683	3.795685	2.347193	6.780559	2.899695	4.751325	7.703107	2.476065	2.992507
Sh2d4b	4.242116	2.723954	2.440953	2.311427	3.9848	2.525752	5.946019	3.762272	2.990796
Ablim1	3.31749	3.338556	3.517748	5.494867	2.55999	3.770204	1.745379	2.368932	2.990437
Zfp788	3.026215	3.03634	2.771314	3.480593	2.534889	2.427656	3.316162	2.72504	2.988501
Fxn	3.466031	4.468103	4.111781	3.318285	3.529272	3.273475	3.582316	3.721278	2.987149
Pigh	4.80636	3.454186	4.100019	3.865721	3.769726	2.222386	4.407539	3.16291	2.986559
Siah1b	2.023049	2.147835	2.839124	1.42767	1.149822	1.780327	1.888575	1.864007	2.985515
Zcchc14	2.797514	3.752836	3.211604	6.053449	2.626796	1.694338	3.787288	2.982378	2.985482
Spink2	6.368591	7.601138	9.91945	1.764911	4.474458	3.475755	3.668799	1.789236	2.985164
Stbd1	2.681136	2.367602	2.597652	2.055132	2.87267	3.409217	1.818526	1.675212	2.981254
Rpl18-ps1	3.493609	2.842462	4.321545	7.770878	4.377994	5.016212	4.180949	4.639255	2.979223
Btbd17	3.19068	4.706983	4.254734	3.205857	3.323653	4.487433	3.778645	3.132772	2.977647
Snord73b	5.465725	9.344868	0	1.552888	5.205496	8.66491	3.228057	2.97366	2.976755
Mir6920	0	0	0.780909	0	0	0	0.807014	1.48683	2.976755
Tulp3	3.286192	2.097564	2.070542	2.418161	1.909137	2.522343	1.817097	1.845978	2.975431

Perm1	3.116768	3.525811	3.795394	4.118063	2.679783	2.642098	4.384595	2.994945	2.970557
Gm15417	2.217523	2.928901	2.45754	2.205101	2.968138	3.159179	2.539695	2.453672	2.970319
Rpusd3	4.960017	3.852384	2.194953	2.531589	3.469742	3.084973	4.491293	2.382107	2.970273
Bbs7	2.932954	4.857831	2.776154	4.296666	2.56885	2.324832	2.814829	1.944749	2.970076
Abca6	4.539689	7.827381	6.464037	4.284721	4.28691	1.95677	3.618403	4.981552	2.970041
Zfp810	3.663912	3.790761	4.143783	4.545722	4.174318	4.338253	4.193832	3.227588	2.96986
Rpl17-ps9	2.936527	2.12997	1.957912	1.946718	1.864477	3.044772	1.747451	3.897261	2.968393
Ffar2	1.174535	1.142123	1.233405	0.554779	2.321125	2.769742	1.669169	1.146229	2.966506
Prorp	5.157856	4.168515	4.48252	3.077379	3.082883	2.927685	4.595605	3.488351	2.966489
Slc19a2	3.399731	3.742543	3.913352	2.870395	3.405869	2.713894	3.596664	3.374291	2.96513
Apol9b	1.703508	0.775618	2.037039	2.540948	1.410784	2.363026	0.842055	2.150789	2.964824
Gm22716	2.072451	1.033467	0.518173	0.515211	1.97378	5.749613	3.212972	0.493294	2.962845
Gm22753	2.590564	4.6506	0.518173	2.576053	0.98689	1.916538	1.070991	0.986588	2.962845
Snora30	1.554338	0.516733	2.072692	4.636895	2.467224	0.958269	1.070991	1.973176	2.962845
Gm41846	3.43817	4.533276	2.253526	2.259958	2.275483	2.496905	4.416805	3.236486	2.962153
Gm31493	0.876176	1.588807	2.150864	3.56428	1.289621	2.283461	1.234871	1.820085	2.960716
Mast1	3.473228	3.834065	3.918969	3.689928	3.25132	3.129575	4.494864	3.518831	2.956631
Rpl21-ps11	3.402336	2.495057	2.702169	2.985245	2.287304	2.683672	2.482224	2.096055	2.956605
Fmo1	2.308416	3.957028	1.839733	6.38432	4.534428	4.302858	1.379327	1.95745	2.956419
Gm6477	3.291933	4.85338	4.055751	4.506984	3.816767	4.103156	4.536503	4.40611	2.95562
Gm10320	3.328579	2.518407	3.214174	3.994748	3.060789	3.927315	2.728476	3.606256	2.953643
Vis1	3.516054	2.930251	3.757317	4.294615	3.470977	2.687343	3.169394	3.210055	2.953263
Cd226	4.206892	3.655143	3.071646	3.131079	3.662473	3.24598	2.85423	2.752159	2.95181
Gm12220	2.65395	3.38213	2.506799	2.932315	3.650967	2.726989	4.876426	1.824926	2.951025
Gm39138	1.619704	2.423088	1.104472	1.830263	3.085177	2.92762	0.076093	2.733753	2.947106
Bora	5.3079	4.989529	4.901734	3.397441	4.106128	4.682898	4.624197	3.078655	2.946179
Slc46a1	2.836497	3.025011	3.314312	2.373779	2.968918	2.908788	2.69945	2.139107	2.944333
Cenph	5.019765	7.592124	4.192818	5.485326	3.572443	4.540097	3.705841	1.943235	2.944173
Gm4949	1.923878	2.625666	3.493763	2.013791	1.542973	2.106879	2.250059	1.301486	2.943453
Setmar	2.365632	2.461913	3.188831	1.465976	1.991784	1.775494	2.515868	2.05646	2.940857
LOC118568652	1.44621	1.217993	1.060678	2.013353	2.815926	3.744748	2.956245	1.376934	2.940516
Snord19	2.053262	5.119488	3.08025	14.29232	4.88876	0.949396	11.67182	3.909813	2.935411
Mir202	0.769973	0	0	0	0	0.712047	0	0	2.935411
n-TEctc4	0	0	0	0	0	0	0	0.73309	2.935411
Taf1a	5.061026	5.163856	4.595106	3.502001	3.909362	4.119508	4.507668	3.575085	2.934176
Pcdhgc3	3.832288	3.562561	4.30591	2.317073	1.644676	1.870434	2.02933	2.477521	2.931027
Ect2	5.771901	6.963326	6.454152	4.370371	3.165797	3.228328	4.456192	3.350217	2.92952
Hjv	2.249342	2.626366	2.03013	4.828091	2.952124	7.178956	1.445917	2.298297	2.928149
Zscan2	1.95876	2.930318	2.629168	3.383	3.092805	3.265287	3.08999	3.288169	2.923129
Atp5l2-ps	2.330361	2.935777	1.962637	1.585526	1.284922	3.062432	1.014124	4.203913	2.922422
Gm19605	2.128958	4.501375	4.258411	3.556615	3.649673	3.071318	5.016876	2.918846	2.921884
Vsig8	3.391934	4.255911	2.662795	1.34192	1.945212	1.484056	1.545538	3.576707	2.919961
Gm36913	1.795137	1.369097	1.689737	2.62512	0.502844	1.953043	2.401059	2.915603	2.918637
Ndufb4b	3.295681	4.460802	6.356695	3.511307	3.923463	2.176959	3.162947	4.818782	2.916714
Esco2	3.690771	4.424763	5.029978	3.27076	2.058035	2.953313	2.905418	2.366927	2.916172
LOC118567430	3.730691	4.018419	4.551972	12.83588	4.405805	4.071031	10.48793	3.978221	2.915657
Rapgef5	5.056339	5.526027	5.026646	5.329094	4.584914	4.479946	3.630021	2.50796	2.91457
Prss30	3.094569	2.032461	3.396872	3.414979	3.091001	3.315454	1.989243	2.083991	2.91343
Gpx8	1.855211	2.612148	1.964569	4.177972	2.078685	3.027601	1.804662	2.805367	2.912298
Gm9824	3.053792	2.29596	2.349343	2.242476	1.924017	1.781324	2.282209	2.996971	2.910535
Slc5a6	3.281709	3.360484	3.808629	3.315679	3.376169	2.677784	3.591328	3.191343	2.910322
Ipo11	4.550407	4.320966	4.333004	3.607188	2.929865	2.28777	3.881691	2.709297	2.907584
Rif1	3.975127	3.272999	3.609854	2.531342	2.614374	1.941248	3.62745	2.373922	2.905989
Gm35891	0.571526	0.760006	0.190531	0.189442	1.451508	1.409413	1.575203	0	2.905149

Kntc1	5.480976	5.54638	4.438769	3.030884	2.482693	3.276069	3.758152	1.896452	2.904974
Gm10293	1.846397	1.657333	1.985107	1.468843	1.758488	2.433173	1.383549	2.15349	2.903637
Tead2	2.132233	3.068317	4.264962	3.422753	2.813992	2.704214	1.668568	2.726128	2.903154
Uchl1	3.963314	7.664503	5.413936	1.00931	2.531755	3.307573	2.697552	2.162839	2.902141
Chil4	0.731983	0.53792	3.197981	0.306477	0.330221	0.463152	0.159272	0.47684	2.900733
Agmo	3.870987	6.652781	7.328077	3.814935	3.061266	2.716788	3.036366	3.488118	2.89881
Matn2	3.874365	2.27759	3.118753	2.631086	3.134917	3.364422	2.278898	2.339223	2.897051
n-TKctt10	0	0	0	0.755172	0	0	0.784904	0	2.8952
n-TRccg1	0	0	0	0	0	0	0	0	2.8952
Ccdc189	1.932602	2.257878	3.258193	2.85521	2.682001	1.940399	1.940371	3.942914	2.894479
Cenpj	4.701371	3.738411	3.990276	4.359156	3.400506	3.442893	4.937898	2.6857	2.89437
Hal	3.355041	1.712889	1.99728	2.124872	2.415498	2.382382	2.229173	3.403481	2.893114
Llgl2	3.00073	3.979175	3.75554	3.31732	3.017519	3.798158	3.759803	3.096401	2.891917
Gm45734	2.426173	1.6938	1.091905	2.774471	2.310661	1.907102	1.003029	1.616968	2.890448
Trub1	2.898136	3.605576	2.360612	2.837337	2.404464	2.666291	2.686568	2.289943	2.890324
Snora21	0.43311	2.591741	1.299481	2.153419	0.412489	2.002632	0.895281	2.886541	2.889545
Exog	3.60605	3.873099	5.270993	2.108165	3.03809	3.408053	4.566639	4.602903	2.88925
Rpgrip1l	4.404733	5.030701	5.139452	4.097475	4.059696	4.314257	4.258801	3.697704	2.889016
1110025M09Rik	2.241166	2.775577	3.399086	3.208221	2.978864	2.186437	3.156354	2.086912	2.887161
Dnaaf2	3.759693	4.322552	3.794946	2.527039	4.194049	3.557329	3.83186	3.447018	2.886564
Naif1	2.830434	4.07752	3.704455	3.422667	3.444472	2.391293	3.738004	3.709578	2.880829
Gm16353	1.435045	2.561139	1.963974	4.28102	1.798318	4.120948	2.888319	2.516875	2.879422
Camk2a	5.662872	6.458761	5.053611	2.165827	4.176317	2.739274	4.081995	3.732652	2.878517
Ptpn21	3.912239	4.278205	3.2459	4.49076	2.036706	2.338267	4.433542	3.425411	2.877462
Zfp446	2.471655	2.200369	2.588074	2.639259	2.764738	1.779478	3.600413	2.495401	2.877441
5031425F14Rik	1.931522	2.838875	2.914564	2.897902	4.195488	3.384399	4.062695	3.839312	2.874406
E030030I06Rik	2.735614	3.674895	2.791768	1.609968	2.180003	3.149356	2.538884	2.9235	2.873333
Mfsd4b4	2.965366	3.511008	3.298758	4.446785	2.9299	2.991575	3.966281	2.2194	2.871598
Wdr5b	2.826254	2.63288	3.758423	3.057493	2.336745	3.216777	2.34328	3.282271	2.871276
Apbb2	4.278777	5.693713	4.279274	4.624792	2.75731	2.913897	4.866966	2.612555	2.870152
Ogn	3.244738	6.45091	5.273313	3.969521	4.981	4.678618	3.155031	4.024231	2.868723
Fdxacb1	4.072752	4.425663	4.863553	3.44547	3.415699	3.344747	4.36646	4.456415	2.867819
Gm51746	3.867772	3.857475	3.008617	8.333231	3.274332	2.185819	4.885876	3.068748	2.867146
Gm12286	4.920538	1.799394	5.030468	3.968747	2.915899	3.690847	3.729456	3.64376	2.865934
Wdr90	3.508276	3.990973	3.84676	2.680063	2.358013	2.754312	4.050897	1.974558	2.864783
Syt8	2.06199	2.291529	1.826546	1.581767	2.188252	2.070309	3.836104	1.234022	2.863663
Capns2	1.857039	2.015515	2.075755	1.900949	1.924678	2.171919	3.21772	1.664078	2.86311
Cd59b	2.771903	2.418958	3.465282	3.215772	2.199942	1.922527	2.148675	1.979343	2.862026
Gm12844	2.627396	2.732704	2.702779	2.276763	2.93127	2.15205	2.870719	2.823165	2.861877
Gm2574	3.508154	2.393925	2.539091	2.937687	3.429052	1.963613	2.194595	2.724824	2.859643
Arhgef39	5.92057	6.134865	7.228549	5.173781	5.931605	7.276508	5.854304	5.051304	2.858056
Gm14321	2.762539	2.661788	1.779469	4.702601	1.96211	1.299005	1.306627	2.942266	2.856076
Cdk15	1.816836	1.469189	1.62061	2.832061	2.291525	1.226058	3.958587	1.262291	2.854811
A930006K02Rik	1.211372	2.203091	1.140247	3.89719	1.832342	1.383824	2.135787	2.713752	2.852405
Ywhaq-ps3	3.59113	3.544261	3.516723	1.264734	1.888209	2.87125	3.015684	2.528715	2.852221
Fam209	2.391781	2.04464	0.768876	1.359076	1.138953	2.211844	3.354891	1.870565	2.849474
Dgkh	2.817914	2.447383	2.585092	2.110748	2.134536	1.573385	2.358708	2.157247	2.849438
Lipn	3.727859	3.791557	4.208172	2.27557	1.406088	2.11623	2.861085	1.370517	2.84942
Bex6	1.973888	1.51866	2.143328	3.14053	1.87991	2.764162	1.107489	1.181296	2.848812
H2bc8	2.748007	6.077185	3.106804	3.089042	5.120554	2.983231	5.92738	2.957638	2.846843
Gm13342	2.031136	1.191605	2.150864	2.613805	0.910321	2.541271	2.222767	1.251309	2.846843
Msrb3	2.636604	2.629584	2.463429	6.65808	2.874517	3.304468	1.470099	2.213032	2.843565
Kdelr3	2.195567	2.111517	3.1761	2.066308	2.053694	1.81285	1.580355	2.165052	2.839917
Gm6883	3.621876	3.437448	3.680721	3.659678	2.39235	1.728721	4.890557	3.782095	2.839523

Gm35144	2.761004	3.550764	1.816661	3.179045	1.937563	2.822058	2.027583	2.905457	2.839231
Gm12091	3.89039	3.455654	3.222104	3.626811	2.315728	3.766354	3.832432	2.8359	2.838851
Mylk	2.435093	1.676269	1.813296	5.987831	4.625707	4.809759	2.147478	3.843078	2.838008
Rap1gap	1.532434	2.217612	1.983381	3.346495	1.659794	2.222976	3.416141	1.172944	2.835177
Gm41041	3.502047	3.229122	2.643361	2.95678	2.517216	3.971847	2.868315	3.082647	2.833949
Hdhd3	2.201142	2.858972	3.839647	4.835748	2.486361	4.023756	3.068591	2.778025	2.829704
AW011738	1.757053	1.289104	1.636067	2.650941	3.539141	1.736928	3.110165	1.653658	2.829544
Zfp28	3.276007	3.333965	3.209523	3.895891	3.34926	3.462341	4.0631	3.322775	2.829209
Gm38984	2.967776	0.591975	0.831074	2.833106	1.695887	1.756484	3.190039	2.373516	2.828555
Hddc3	4.190086	2.893106	2.127522	4.487124	4.972892	3.397955	2.598398	2.761873	2.826186
Hmgb1-ps2	1.482301	3.193246	2.194061	2.063597	1.581135	1.480449	2.328689	1.750007	2.825529
C8g	2.627077	2.821628	4.715815	3.751083	3.143538	2.616319	3.341803	3.014309	2.824843
Pole	6.07338	4.824053	5.039703	3.464319	2.273695	3.106678	3.817724	2.169345	2.823825
Col16a1	2.251562	2.722972	2.588711	3.067537	2.769099	3.787261	2.21716	2.160587	2.821825
Per2	5.343061	6.532122	4.512217	2.359152	2.674692	1.790796	5.386096	2.500121	2.821602
Tns2	4.162667	4.847515	4.223312	6.555964	3.632196	4.405791	3.108616	3.218723	2.820747
LOC115489084	6.652568	1.474413	3.6963	8.08537	2.111944	2.050695	4.58384	3.518831	2.817995
Snord59a	0	1.474413	2.21778	0.735034	0	0	3.819867	0	2.817995
Gm32029	2.830171	2.61509	3.57975	2.359061	2.53687	2.270853	2.624008	2.853106	2.816408
Chst1	1.961364	2.710317	2.977838	1.73889	3.825986	3.343525	2.833149	3.55483	2.815292
LOC115486982	3.280359	2.6173	2.624592	2.609587	1.874507	1.820144	8.136995	7.183413	2.813826
Myoz2	2.504732	4.829589	3.395698	5.14745	3.233649	5.301748	1.610787	2.649722	2.811629
Uaca	3.628821	2.765201	3.194278	4.500482	2.342865	3.732879	2.472285	2.60095	2.810913
Setd4	2.119239	2.634338	3.010283	3.664988	2.076843	2.584671	2.063363	2.193177	2.810189
Parva	2.882303	3.125171	2.76363	3.641861	2.833219	3.399078	2.364072	3.159647	2.810098
Pyroxd2	3.379777	4.316537	4.547422	5.512752	5.580905	4.811939	8.56957	3.217879	2.80409
LOC115488553	1.679941	1.675469	1.680137	3.341062	0.799979	1.553557	4.774834	6.797742	2.801983
Entpd3	3.169536	2.030766	1.287173	0.744967	1.00621	1.687596	1.111812	0.731566	2.801151
Bbs4	3.313593	4.711058	3.478502	2.687438	3.043921	2.173268	3.619077	3.915613	2.799777
Hepacam2	3.611212	6.728261	5.357916	2.920141	5.328993	5.027653	3.937443	2.720356	2.798831
Plppr2	3.103511	3.328452	2.870019	2.578818	2.409139	2.358929	2.26292	2.732221	2.795844
Gprasp1	2.823189	3.517156	3.468335	2.836518	2.521308	2.403017	3.352059	2.483334	2.793166
Thnsl1	2.263264	3.611582	4.360268	2.913917	2.858884	1.916744	2.585428	2.222897	2.792866
Aspm	6.361573	7.663818	8.336099	5.282054	3.099331	3.989944	6.195259	2.368764	2.791447
Ankrd9	3.193962	3.223381	5.095723	4.04571	2.897043	4.50084	3.419017	2.280724	2.790452
Gm10509	1.726575	2.451947	2.120931	1.623593	3.092132	1.162801	1.881485	2.215649	2.79033
Immp2l	1.921121	2.82839	1.097911	1.000665	1.394023	2.199591	1.229165	0.522599	2.790094
Gm44140	1.60902	2.334162	0.292583	0.872731	0.557241	0.676351	0.907092	2.506819	2.788253
Podxl	5.793434	4.263487	4.597837	12.38042	5.131271	6.886951	2.096268	3.842326	2.785611
Snx22	2.854596	3.795995	3.149484	2.320448	2.783416	3.729299	2.739627	1.251076	2.785462
Gtsf1	2.44812	1.811511	2.448404	1.649114	1.80508	1.095457	1.958906	3.985001	2.784877
Abcb4	5.065323	6.499921	4.851394	2.772783	1.571387	1.937785	2.813741	2.576287	2.783399
Smoc2	3.641059	4.080406	3.817683	6.501622	2.796536	4.616237	2.306487	3.876679	2.779934
Gm10029	2.404847	2.524679	2.02537	2.391377	2.049261	4.447855	2.48553	2.048635	2.774567
Pigz	3.318116	3.411106	4.365106	5.304628	3.257372	3.375339	5.724524	3.426486	2.773234
Rpl19-ps7	1.258169	1.882229	2.437985	2.033072	2.246749	2.545189	1.544202	3.069619	2.773027
Celsr3	2.959177	2.529685	2.359161	3.220046	2.238526	2.345618	3.294395	2.487385	2.772009
Dusp18	4.679681	4.965638	4.12961	3.380012	2.804071	2.147209	3.760491	3.133674	2.77191
Tbc1d12	3.134307	3.546766	3.285377	3.821016	2.970729	3.023926	3.955883	3.199372	2.771845
Vnn3	1.010191	1.404396	1.163386	1.461138	3.381909	1.670224	2.119805	2.768821	2.771702
Rab37	0.631989	0.453821	0.455085	1.96076	1.829774	1.65982	2.194725	0.914607	2.770771
Pcgf2	3.75571	4.45245	3.685276	2.959552	2.924507	3.669757	3.710821	3.328422	2.769067
Gm38560	2.524242	3.46753	2.42927	2.415381	1.496868	2.730737	1.279852	2.494017	2.76897
Gm11699	4.838734	4.825852	2.488781	4.261729	4.47669	0.767093	5.286848	2.500915	2.767046

LOC115487043	4.964603	4.53877	9.930359	3.702595	3.940194	3.443332	5.986359	8.665779	2.760163
Mir6981	0.48207	0.480787	1.928505	0.47937	0	0.891607	0.996487	0	2.756734
Sema4c	3.773988	3.490752	3.470038	4.282484	3.101538	3.293046	2.736715	2.506552	2.755725
Spata1	1.601804	2.454757	2.070866	2.136726	1.488333	1.589686	2.866919	3.273331	2.755439
Shld3	2.550831	3.256371	2.585143	3.246774	3.692663	3.868643	2.144281	3.464861	2.755324
LOC118568705	1.550715	1.898084	2.749314	4.555994	3.255858	2.574776	3.241909	2.080428	2.754397
Gm13523	3.79218	1.020562	1.80601	3.830794	0.573275	0.946304	2.986215	1.547369	2.753741
Gm14633	1.385952	1.267073	0.346528	2.526678	2.529933	2.776983	4.774834	1.979343	2.751948
H2-Q7	2.004374	1.170169	4.058108	3.889067	2.141742	3.029025	3.183222	4.933811	2.749036
Ift88	4.637141	4.808867	4.476169	3.05117	4.636082	4.13894	4.554273	3.778021	2.748512
Gm38499	1.816347	3.032312	1.935029	1.177939	2.557197	1.351064	2.734306	3.007548	2.747244
Spa17	3.779868	3.859562	3.330271	5.280071	4.114177	4.161314	2.97652	2.399203	2.7448
LOC115488830	2.879899	0.718058	0.720059	4.295651	0.685696	5.326482	1.48826	2.05646	2.7448
Igfbp3	4.010027	5.46804	4.237075	8.78616	3.085493	4.567344	1.428352	3.472815	2.742276
2810002D19Rik	3.025932	3.874804	4.371299	5.20071	3.771329	2.314633	5.057978	4.837207	2.741563
Gtpbp10	3.500081	3.310206	3.460252	3.140429	2.758708	2.436875	2.931429	3.389876	2.741563
Aif1l	1.332917	1.348091	2.478386	2.296202	1.591289	1.85764	2.095559	2.198525	2.737596
2610044O15Rik8	1.498326	2.552826	2.528719	2.731544	2.348587	1.732006	1.903481	2.853106	2.737072
Rbfox2	2.615688	4.311642	4.141988	4.479563	2.387355	2.788462	2.140227	3.631821	2.735357
Gpc2	3.906265	2.927402	3.354922	3.994112	4.455933	4.530796	4.83566	3.803195	2.73442
Depp1	1.3079	1.388573	1.308052	3.440226	3.937795	3.745562	2.267501	3.012698	2.734355
Gm12251	4.552282	3.1097	3.4302	3.534611	5.048235	7.208575	3.609323	5.581048	2.733994
Gm26226	1.911657	0.953284	1.91188	0.475237	2.275802	0.88392	0.987897	0	2.732969
Bag4	3.32767	3.019196	2.819604	2.091123	2.365923	2.179772	3.355719	2.673226	2.73107
Lrrc51	2.793391	2.785954	3.796588	3.205089	4.843283	2.58324	1.628625	3.614303	2.730615
B3galt6	3.468114	2.73613	3.226929	3.157007	2.054101	2.090267	2.817642	2.727012	2.72985
Il33	1.529025	2.897412	3.058405	2.367573	2.309159	2.019992	1.354563	3.410688	2.727226
Ccn2	2.953005	3.704439	2.145792	3.028229	3.054102	3.434893	1.478351	2.877446	2.726524
Gm4045	2.734613	2.479393	1.864726	2.348482	3.551476	2.988682	1.798592	2.721966	2.724799
Lat	2.611749	3.440296	2.661336	3.479159	3.285246	5.468521	3.106825	2.439723	2.724061
Trim68	2.880812	4.765212	3.302778	2.794805	2.565205	3.097269	3.897329	3.211101	2.723347
Klf5	1.219124	0.927907	1.34761	0.414733	1.099971	1.987798	2.221624	0.885817	2.721371
Gm15429	2.256674	2.060851	3.099889	2.784765	2.278704	2.011472	2.332386	2.122689	2.720906
Pcnx4	2.790239	4.086756	3.77922	2.996578	3.021836	3.671435	3.163997	3.324521	2.720131
Gm7059	2.604473	3.401539	2.790831	4.008153	3.248236	2.179151	3.012311	2.95204	2.718703
Gm5417	2.451683	1.497034	2.101687	2.686721	1.143652	2.26724	2.275372	1.429128	2.718169
Tspyl3	3.952128	3.275672	3.032121	3.032732	3.162416	2.98726	2.555282	2.766269	2.717548
Tpmt	3.799916	5.887725	4.852243	4.116009	4.362186	4.894561	6.136567	3.133353	2.716243
Cep72	2.939897	3.650129	4.500366	3.907849	1.885664	3.218083	4.185731	2.94188	2.716208
Arl4d	2.045833	1.560295	0.882619	3.191174	2.559701	3.375783	3.109515	2.97904	2.714512
Klhdc1	1.678217	2.411334	2.674081	2.687078	1.869217	1.999139	2.087305	1.4895	2.711
Prdm11	5.071806	3.97647	4.808513	5.247464	2.429127	3.795576	4.423854	3.098283	2.710327
Tbc1d16	3.294687	3.460108	3.20773	2.881505	4.143439	2.929355	4.045241	2.743812	2.708834
Gm30461	2.189571	3.205918	1.817089	2.362608	2.972695	1.507864	2.26303	1.862911	2.708472
Gan	4.47806	3.089479	2.472048	3.2719	2.476368	2.241278	4.080865	1.894912	2.707649
Gm2999	2.99254	3.237502	2.333438	2.168786	2.801756	2.579786	2.883248	2.849191	2.707131
Smug1	2.870927	2.250817	2.825198	3.129643	2.748861	2.96729	2.983114	2.39721	2.706984
Fbln5	2.562943	2.18767	2.286133	3.85732	4.486012	3.758042	1.551175	1.582815	2.706789
Crocc	3.652827	2.505184	2.759841	3.280563	2.805031	2.453768	3.757096	2.391548	2.705936
Zfp938	1.801532	1.878406	2.129331	2.388588	1.481792	1.994152	1.467009	1.949131	2.705608
C1qtnf9	3.424388	2.338384	1.974651	8.497676	5.905687	6.362063	1.530498	2.790392	2.705087
Cables1	2.71672	2.811093	3.124591	2.043011	3.218047	2.30821	4.439937	2.489587	2.702556
Grasp	5.092374	3.733617	2.697896	3.229915	3.224542	2.800105	4.580426	3.826336	2.702211
Rtkn	2.357162	3.392419	3.998689	3.204399	3.239527	4.249288	3.238047	3.66466	2.701589

Cpa3	3.318476	3.747682	4.148577	2.086693	5.71675	10.4701	1.714905	2.323172	2.697684
Marchf9	3.856802	3.90188	3.46875	2.64877	2.378316	2.437638	2.781735	2.721018	2.697405
Tmem140	1.257812	1.411271	2.10708	3.3458	1.976578	1.977415	1.625014	2.305304	2.697315
Zfp74	3.423325	4.317143	3.607642	3.136881	2.667549	3.126539	4.532376	2.53205	2.696474
Slc30a4	3.574638	4.207672	4.354498	3.461616	2.642404	1.950755	2.212444	2.700959	2.693865
Ptger1	3.518185	3.748056	3.625218	4.108061	2.995305	2.66196	5.123764	3.781052	2.692675
Gzmb	1.613277	0.731355	2.420197	7.036781	4.260205	5.153864	1.250552	1.535998	2.690793
Antxr1	1.821958	2.415807	3.68647	2.576248	1.935815	2.386115	1.708926	2.566929	2.69005
Myb	0.340409	0.743673	0.421508	1.434605	0.741033	1.469065	0.167538	0.339536	2.688219
Dennd11	3.075324	4.395683	3.618449	2.346722	2.153607	1.786982	2.388125	2.004199	2.688109
Zfp58	1.645046	1.9688	2.326836	3.411877	2.081505	1.738614	2.477489	2.640242	2.687786
Intu	3.240913	4.38855	4.479832	3.275162	3.437932	2.38793	4.656825	2.182545	2.687073
Rpl31-ps11	2.936338	1.757112	1.879475	1.751934	2.460952	2.932668	2.306487	3.131163	2.686647
Tmem64	3.434531	5.397937	3.695511	3.980582	4.534296	3.471857	4.90846	4.273565	2.686456
Aebp1	4.397469	4.01915	4.289052	5.02267	4.110304	3.827428	2.814244	4.264595	2.685988
Gm5138	2.540911	2.810599	3.003244	2.480739	2.771927	2.477924	2.339669	3.606802	2.685901
Inhba	1.721031	1.924504	1.869014	1.676817	1.291405	1.085152	1.248734	2.209614	2.68412
Zmat3	3.390097	2.954886	3.967445	2.556659	2.428302	2.443499	3.415503	2.875099	2.68124
Mettl18	3.750567	4.247119	3.086762	3.224513	2.306916	3.251631	3.068815	3.273331	2.680967
Cd248	4.066467	4.271367	3.33143	6.237605	2.863444	5.840841	2.883903	3.809893	2.680009
Ryk	2.227422	3.438024	3.288482	3.867365	1.868828	2.370463	1.991544	2.608827	2.678937
Zfp503	2.802826	2.559136	2.131975	2.682445	1.992637	2.640641	2.86966	2.656037	2.671342
Cilk1	2.573396	3.241952	3.564229	2.542146	2.724985	2.692927	2.860963	2.264754	2.670511
LOC118567331	2.122437	1.862772	2.292499	4.052253	2.102242	3.532974	3.071103	4.203198	2.67019
G430095P16Rik	2.965483	3.039744	4.531126	3.030785	3.92263	3.961224	3.064975	4.470432	2.669349
Fitm2	3.171746	2.658519	2.63218	2.751343	1.767452	2.527485	3.382779	2.088168	2.669205
Tasp1	2.641165	2.15999	2.509399	2.468789	0.930704	2.686714	2.838967	2.489502	2.668301
A230028O05Rik	2.580361	2.811044	1.647653	2.269841	1.455601	1.853923	1.682219	1.663035	2.667409
Rps6ka5	2.716954	2.618381	3.531432	2.074365	2.093337	1.759729	2.860693	2.247712	2.667086
Glmn	3.101855	3.305903	2.524352	2.81232	2.316999	2.756009	3.0802	4.285137	2.666506
Urb1	3.428285	3.334639	3.498027	3.807446	2.560688	2.358186	3.861803	2.897315	2.665366
G530011O06Rik	1.048199	2.834217	7.035395	6.601405	4.081909	3.705038	10.83366	7.362933	2.664071
H2-Eb2	2.412015	1.871017	2.603748	1.02793	2.91705	1.770283	1.978522	1.421627	2.663764
Arpin	4.822908	4.025268	3.604908	3.382366	2.441694	2.887311	3.226948	2.561787	2.661224
Slc25a15	4.043529	3.632688	3.434189	2.760365	2.872978	2.700626	2.587117	3.131811	2.660986
Nectin1	3.687416	4.057034	3.678089	2.260199	2.592084	2.273338	2.379435	2.47055	2.65907
Tonsl	4.767367	4.824187	4.837627	5.41988	3.624345	3.05516	4.307796	2.216412	2.657149
Trip13	4.108335	4.829075	4.549042	1.945391	2.724939	4.070635	4.246169	2.654257	2.65702
Otud7b	3.895878	4.325107	3.434175	2.870194	2.559358	2.044647	3.166894	2.822556	2.656099
Nckap1	2.13856	2.574585	2.695658	3.25908	2.097	3.101086	1.412505	2.578281	2.653327
Myo1b	3.057421	2.835713	2.177327	2.03475	1.461592	1.661239	1.106614	1.347879	2.653208
Timp3	4.239869	3.543504	3.233572	8.420462	4.241033	5.563732	2.643958	2.841526	2.652593
Gm3222	2.827527	1.80295	1.807973	2.627315	2.074862	2.829137	2.826574	3.00101	2.650706
Rn7s1	0	0.369836	0.741733	1.659357	2.119008	1.714628	0.766528	2.82448	2.650706
Zfp251	2.839741	3.603164	4.070769	2.965026	2.193681	3.078377	3.244822	2.568527	2.646381
Nutf2-ps1	5.16582	4.900746	4.158338	3.132246	3.059919	4.427638	4.036905	4.378546	2.64187
Gm25588	0.395986	0.394932	3.168257	0	0	0.732391	1.637086	0.377018	2.64187
Sh3bgr	1.846537	2.427591	1.972666	1.961388	2.118339	2.871905	1.431366	0.998911	2.63987
Gm15987	6.507055	4.13912	4.458107	5.196865	5.611682	4.975116	4.342363	5.707531	2.636987
Gm44851	2.996652	2.679501	2.118569	2.414719	2.878582	1.982849	3.791387	2.779319	2.634484
Zfp993	2.562622	2.612595	2.676828	2.463325	3.172797	3.21244	2.060021	2.22299	2.632372
Tmed10-ps	3.299223	2.717385	3.262532	2.93055	2.188909	2.742488	2.57659	2.161769	2.632154
Gm13268	1.329695	2.550298	1.534442	3.458184	3.31209	2.55391	1.79717	1.947693	2.632121
F8a	4.541914	4.907307	4.252972	3.542331	4.156035	4.282577	5.085486	4.175962	2.63126

Cthrc1	3.641059	5.037056	3.230347	3.854255	1.957575	2.606816	2.185093	4.193522	2.630676
Arl9	3.585546	4.263693	1.103373	1.371331	3.940194	3.443332	1.567856	3.676391	2.628726
Mrgpra2a	0.918138	0.174418	0.349808	0.43476	0.208196	1.172519	0.406689	1.498556	2.625202
Gm15621	1.376122	1.274426	0.786447	1.857133	1.02976	1.272595	3.657319	1.590961	2.623133
Wfdc18	2.038164	1.422917	2.038401	1.216048	2.232294	3.298453	3.159816	1.552426	2.622444
Myh10	2.934663	2.848706	2.279615	2.33033	2.130125	2.061762	1.847848	2.102347	2.620485
Prr5	2.265861	3.103498	3.474724	3.304647	3.280131	3.212942	3.559658	2.099793	2.620274
Usp53	2.776047	2.865077	2.652054	2.966504	2.604415	1.851961	2.226828	2.669368	2.619492
Ffar4	3.97976	5.596523	5.293697	1.503838	2.84271	3.017893	2.015508	2.917624	2.617215
Ttk	4.858094	6.027473	5.83504	3.282226	3.276391	3.417826	4.468524	2.987687	2.614177
Homez	3.262198	3.118752	3.301188	3.051976	3.253953	2.499106	2.912782	2.646475	2.612434
Camkk1	1.593506	1.493908	1.561817	1.299355	1.623872	2.033601	1.498741	2.033013	2.612254
Gm24494	10.26631	6.825984	4.107	15.65349	2.607338	0.632931	8.488593	4.561448	2.609254
Gm5425	2.851752	3.981824	3.308417	4.196951	3.69373	4.325026	4.715885	3.366783	2.609254
Blm	4.536666	4.554752	5.414384	4.240579	2.18914	3.761846	5.157758	2.807578	2.60872
Crybb1	1.463921	2.539172	3.055495	1.772182	1.636696	1.942392	1.710388	2.908793	2.608505
2900076A07Rik	1.552266	2.801384	3.548448	3.895678	3.379111	3.349469	2.215523	2.674312	2.606645
Dach1	2.231351	2.246306	1.959207	1.875086	1.336926	2.170048	2.663513	2.353867	2.605926
Pla1a	4.850831	4.350059	3.832194	1.418723	1.979948	1.168595	1.390319	2.212206	2.603019
Siglech	2.612265	2.084248	3.541482	3.752135	2.100887	6.01251	2.639908	1.713358	2.600375
Gm52889	2.54104	2.549728	2.2934	2.773324	1.800288	2.235247	4.06755	2.581591	2.599045
Zfp748	2.426541	2.635732	3.29183	2.986323	2.974569	2.032921	1.738184	2.847852	2.598936
Armcx5	2.766018	2.210836	1.981562	2.243335	2.615642	3.156591	2.473587	3.119134	2.598867
Mir6975	0	0	0	1.80746	0.865551	0	0.939312	0	2.598561
Kitl	2.816786	3.560488	2.889347	4.09378	3.596555	3.912084	3.07125	3.231981	2.596142
Rps8-ps1	2.082933	1.518091	1.362076	1.911937	2.13636	1.926231	1.738812	1.983156	2.596057
Gm15793	2.020722	2.45346	2.196692	2.009403	1.087768	3.818648	2.451737	1.923925	2.595815
Cdkl3	2.107295	1.325678	1.815727	1.418486	1.45119	2.308531	1.809411	2.284154	2.595521
4632415L05Rik	2.991553	3.61525	3.652275	2.854196	3.298309	2.479876	2.757658	2.732782	2.59435
Gm36496	1.906823	2.48905	2.944701	2.370177	2.24334	2.385736	1.217257	2.830016	2.592427
Ncs1	3.969673	4.191993	2.78871	3.1279	2.05386	2.375372	2.370854	2.641739	2.592122
Dus4l	4.134883	5.078476	4.594849	2.398504	2.989976	2.974075	3.600911	3.025514	2.590784
Tulp2	1.087021	1.951428	0.761003	1.152994	1.24232	1.775929	2.958524	2.690871	2.590069
Gpr85	3.522442	4.995642	5.171158	2.506527	2.462191	1.763204	3.039416	3.369094	2.5872
Dpy19l3	3.081199	2.67421	2.979623	3.765605	1.396314	1.645835	2.998199	2.045311	2.585453
Tal1	2.236518	3.131955	3.048758	2.178291	1.41516	1.81327	1.599088	1.721009	2.5842
Ctif	3.236531	3.616711	3.391046	2.316889	2.262181	2.590617	3.635589	2.460017	2.583546
Gm11836	1.224184	1.046507	1.661586	2.347702	1.415735	1.293814	1.355631	3.330124	2.583532
Zhx3	2.722405	2.653449	2.388569	2.645629	1.932806	2.002632	2.916059	1.861525	2.5829
Tgif2	1.571538	1.959192	1.840567	1.439361	1.851201	1.300329	2.094444	2.342825	2.58176
Yy2	2.417076	2.757774	3.074878	4.230225	2.522989	2.324646	2.977817	3.497966	2.580131
Wdr6	3.524344	2.860082	3.243309	3.03821	2.935383	3.556621	4.18274	2.845175	2.579926
Hspd1-ps3	4.564028	9.177971	5.432817	2.466556	3.567154	2.110335	1.666385	6.022161	2.576857
Lysmd1	2.447495	2.924569	3.694761	3.834359	3.386499	3.65128	3.22167	2.330255	2.57475
Ccne2	3.736299	4.662817	2.46507	1.77015	2.142498	2.062273	2.30486	1.229232	2.572887
Trim17	1.918356	3.124166	2.938583	2.825195	2.728969	2.762106	3.287796	3.329249	2.568967
Dgke	2.307006	2.525528	2.322812	2.788432	1.797683	1.817381	3.387944	2.048584	2.568947
Tcaim	3.400025	4.09559	3.665387	3.622478	2.859662	3.082985	2.875169	3.342259	2.56717
Rpl31-ps9	1.795565	1.007316	1.122358	2.231884	0.855038	2.075603	2.087782	2.350636	2.566999
Fzd4	3.099116	3.781058	3.415442	5.086393	3.281107	3.923311	2.44119	3.294428	2.566593
Chst10	4.184691	1.161336	3.857642	1.411207	1.403573	0.067302	3.591703	0.571651	2.566437
Wdr19	3.047301	3.981824	3.520286	2.317236	2.188302	2.154978	3.823235	1.799755	2.56266
Rhobtb1	4.460205	4.704965	4.546507	3.369062	3.853018	2.657232	4.609845	3.797398	2.561483
Ccdc57	2.089471	2.695697	2.128057	2.344635	1.825678	1.950004	1.961446	1.806869	2.557827

Mir7656	0	0	0.894266	0	1.703181	0	0	0.85133	2.556648
Sco1	2.953243	3.397522	2.888814	2.576053	2.41788	2.419629	3.279909	2.750115	2.555454
Acss2	3.189694	3.376368	3.601055	1.537266	2.590542	1.719168	2.649502	2.4407	2.555144
Sfn	2.680824	2.502297	4.57168	1.332904	2.68412	2.065952	3.339127	2.912362	2.555063
Syp	2.255941	2.227658	2.323219	1.554765	2.084715	2.086223	1.823748	1.807619	2.554588
Myl2	1.088663	1.837448	1.675061	3.330968	2.392686	2.013523	1.21174	2.710882	2.554074
Tctn1	3.082206	3.29555	3.457471	3.023523	3.847832	3.351061	3.888745	2.141438	2.553874
Gm9727	2.679163	1.644326	1.717612	2.664155	0.915961	2.414078	1.420025	0.523246	2.55348
Zfp40	2.351259	3.144739	2.786498	2.378633	2.951234	2.463449	3.146544	2.61389	2.551843
Gm3839	2.400316	1.196963	2.44676	1.927857	1.846412	1.579428	2.099178	1.538207	2.551681
Gm15432	2.889629	3.308889	4.067358	4.789071	4.484824	4.750646	1.106139	2.547416	2.550067
Col20a1	3.533531	4.038884	3.891307	3.697979	3.541754	3.989775	3.542655	3.162664	2.5479
Ccnj	3.020325	2.048353	2.552471	2.627981	2.315602	2.388102	3.246523	2.300516	2.547595
Gm13552	1.60358	1.446996	0.916438	1.518665	2.472662	0.847395	1.578457	1.454063	2.547258
Mir1964	0.667928	0	0.668006	2.656748	0.636128	0	0.690337	0	2.546381
Kif3c	3.237732	3.212967	3.157155	2.905685	2.420611	2.320469	2.877867	2.481524	2.545823
Pla2g2e	4.299544	3.118616	2.475783	3.498104	2.233548	3.494134	4.780445	0.992385	2.545633
Cdc6	4.679993	4.715528	4.018764	3.708666	2.348896	2.647925	4.066069	2.359633	2.545552
Cyyr1	3.487013	3.341881	2.806282	6.772423	2.828033	3.652959	1.464126	1.582177	2.544504
Adgb	1.611572	1.375118	1.898294	1.673769	1.415466	1.059791	1.869218	1.142256	2.542885
Gm17803	2.241	1.543237	0.266817	1.591748	1.72777	2.96058	1.21324	1.117627	2.542704
Pkp3	2.226348	2.080394	2.988868	2.39338	2.693416	3.375783	2.383962	2.883557	2.542465
Pdlim3	2.563802	2.058864	2.0646	2.913647	3.297931	3.78732	1.479768	1.933772	2.538734
Scrn2	0.961567	1.770475	1.294568	1.83881	1.585015	1.881058	1.376069	0.809871	2.537887
Gm52406	2.662773	1.697896	2.837711	3.906675	1.995538	0.847728	3.609323	2.618343	2.537859
Glns-ps1	2.823045	2.489091	2.90521	2.481756	2.454843	2.421485	2.917758	2.337231	2.534635
Slc36a3	0.787752	0.638345	1.231006	1.90939	1.453603	0.956141	2.137226	1.921919	2.533943
Trmt112-ps1	2.752918	2.272211	1.613967	2.265515	2.260214	1.931306	1.471695	1.988381	2.5333
Itgb3	1.841223	1.931962	1.803073	2.116989	1.854025	2.49198	2.259807	1.469984	2.531735
Adcy4	4.631079	3.340746	3.484955	5.789955	4.367768	4.864788	1.74264	4.08818	2.52831
Prrx2	1.620422	2.350702	1.817048	3.173861	2.385057	3.269488	2.689809	3.179104	2.527209
Plpp1	4.028729	3.77761	2.720569	3.287107	2.230003	2.356389	1.637086	3.409551	2.527006
Dtnb	2.823634	2.773122	1.810785	2.807817	2.771311	3.468322	3.096587	2.195849	2.526827
C1s1	3.965388	3.62687	3.095297	5.558917	3.352877	4.990789	2.199156	3.333436	2.525724
Slc18a1	3.467101	3.830257	3.165209	5.321804	2.319887	1.98953	8.012165	2.843956	2.524943
Tsr2	3.54624	3.402491	3.307216	3.957874	2.508112	3.279452	2.984754	3.689787	2.524216
Cdh13	3.176139	2.63721	2.325386	4.911306	3.299913	4.426871	1.193708	2.155863	2.520205
2700081O15Rik	2.686364	2.876627	3.040186	2.699435	2.248755	2.288139	2.425776	2.194221	2.519917
Focad	4.351418	3.182544	3.771667	3.650632	2.829517	2.50694	5.128078	2.761984	2.516975
Mir12193	0	0	0	0	0.838073	1.627536	0	0	2.516067
Mir6908	0	0.877627	0	0	0.838073	1.627536	0	0	2.516067
Ska1	2.638894	3.056364	2.937175	2.349004	1.824136	2.460047	1.979586	2.086984	2.515101
Ap3s1-ps2	2.724335	2.889596	1.513696	2.064057	2.388704	3.239203	3.039208	4.611261	2.514104
Nlgn2	2.671046	2.774472	3.181242	3.052844	3.261649	2.347107	3.276135	2.300395	2.514055
Gm21370	1.641411	1.959837	2.288993	2.64373	2.488008	2.22344	2.508878	2.069038	2.51187
Wwtr1	3.204512	2.627807	2.991226	5.039489	2.339823	4.456125	2.257083	3.582111	2.511219
Gpr137c	0.395046	2.429629	1.38282	1.898691	2.257423	0.487101	3.402494	2.382107	2.51009
Gm42085	2.632942	2.917703	1.316624	1.745463	5.154481	2.164322	6.198465	2.785355	2.509428
Zfp33b	3.098568	3.600513	3.157399	3.83698	3.173763	3.33853	4.305281	3.172793	2.507443
Rian	1.46948	2.422044	2.134861	3.506997	2.902155	3.175603	2.158267	1.752543	2.506238
5530601H04Rik	2.391621	2.548221	2.377042	3.028173	2.107983	2.280382	2.656097	2.658924	2.505954
Gm13226	2.753132	1.996947	3.128922	2.115503	1.191842	2.083099	1.940113	2.382956	2.504707
Dctd	2.627113	2.828538	1.582423	1.929612	1.108856	2.042965	2.15986	2.10334	2.503872
6720489N17Rik	1.323957	2.850456	2.227869	2.089747	1.501098	1.496432	0.781929	1.48063	2.503667

Dffb	2.625729	2.591741	3.059194	3.176293	2.70696	3.003948	4.78416	2.938087	2.502553
E230016K23Rik	5.767221	6.943696	6.625281	2.2733	4.305041	3.099106	2.470205	3.512236	2.500739
Gm4674	1.692765	2.87004	1.35437	2.861581	1.450954	1.095791	1.66208	1.611678	2.500701
Slc29a2	2.768229	4.153504	3.30756	2.436037	2.566437	2.741208	3.747284	3.055459	2.498277
Zfp41	4.453864	4.301239	4.626911	3.415255	3.002127	1.986883	3.809061	2.807102	2.496137
Angptl3	1.412654	0.824718	1.137146	1.644575	1.312582	1.43383	1.317605	1.67303	2.495738
Ppm1j	3.329516	4.997098	4.817045	2.282247	1.262241	2.152333	1.670496	2.308271	2.495527
Snord3a	5.230006	4.955278	3.66143	2.080284	6.724351	7.254819	2.97301	5.975374	2.49233
Pheta2	2.340671	2.776091	2.383123	2.180777	2.490311	2.496092	1.743568	1.94747	2.492141
Bbs2	3.034365	3.44599	2.348029	2.026261	3.332872	2.089202	4.418104	3.120977	2.490936
Pyurf	2.787274	3.362396	3.33077	2.700332	3.054707	3.041928	3.505664	2.907426	2.490487
Clec2g	1.040068	1.157916	1.427236	3.58377	2.948613	2.52758	3.074893	2.049581	2.48972
Egfr	2.980986	2.993696	3.229777	3.787701	2.050431	3.503335	2.631686	3.528029	2.486002
Creb3l4	4.449365	4.029472	4.75677	2.898772	3.896576	3.310624	4.545783	4.723155	2.485892
Gm11847	2.585632	2.168254	2.205959	2.109391	2.201198	2.088563	2.585118	2.140237	2.484454
Slc35d1	4.092043	4.770005	4.457457	3.667393	2.668477	2.217489	4.027293	3.077116	2.484128
Ankrd26	3.210749	3.54342	3.237443	2.368403	2.619252	1.776653	3.672077	2.806379	2.48322
Dpep3	4.386236	5.09817	3.760068	2.361203	1.821725	1.707896	3.238138	3.077146	2.483138
Fgfr4	1.119181	2.540321	3.686008	2.206636	0.955631	1.481097	0.757857	2.002537	2.482788
E130308A19Rik	2.68884	2.64752	2.946078	2.537535	2.201981	2.027255	2.548938	1.9078	2.481089
Pex11a	2.344966	2.770879	2.855073	2.990827	2.985852	3.417826	1.369883	2.790797	2.477892
Zfhx2	3.755174	3.947961	3.997088	3.588818	3.842649	2.74993	4.787421	2.940089	2.476848
Mir7078	4.331099	3.455654	2.598961	6.890941	2.474935	2.403159	5.371688	0.824726	2.476753
Gm34403	1.766536	3.471848	2.130482	1.601643	1.484497	1.537541	2.201704	0.840958	2.475979
Zswim3	2.742009	2.302913	2.28871	1.8246	2.336569	2.917036	2.834003	2.669548	2.475832
Gm46430	2.341854	3.73699	3.761596	2.836823	3.054912	2.664434	3.637969	2.608043	2.475485
H2-Ob	3.945031	3.665323	3.052563	5.099812	4.765717	5.60677	5.064543	3.182763	2.473661
Gm14173	1.458896	2.586689	2.10754	2.901449	2.006964	2.998093	1.67538	2.006351	2.471925
Gm6627	3.968198	2.421141	2.848097	3.992393	2.489871	3.259526	4.246084	2.7558	2.469453
Fam20a	4.324714	5.909424	5.959944	2.099451	2.789115	2.078407	1.900548	2.464046	2.466611
Notch4	3.482349	3.14784	4.566018	4.377774	2.79522	4.071233	2.756564	2.195573	2.464265
Gm7232	3.688844	3.12717	3.320346	2.043701	1.731513	2.509763	2.777755	3.135841	2.461058
Hexim2	1.404069	3.297553	4.348589	2.972563	1.811717	2.094256	2.621477	3.23422	2.460565
Nudt6	3.678661	3.089572	4.130906	2.887938	3.872307	4.595563	5.669768	3.748231	2.460414
Gm5913	1.985604	1.425829	2.780169	2.685295	2.155817	1.579153	2.339532	1.285533	2.460194
Gm14295	2.664072	3.626987	2.62209	3.321948	3.141336	1.75975	1.442284	2.455935	2.458491
LOC118568657	0.322314	3.857475	0.322352	1.282035	1.841812	0.298066	0	2.454999	2.457553
Hoxc10	2.05648	2.166555	2.607108	2.592203	2.068911	2.410692	1.466877	2.068279	2.456911
Ercc6l	3.842896	4.636076	4.371637	3.125381	2.402223	3.236269	3.275732	2.71582	2.454334
Tnnt2	3.066155	4.932245	6.330862	1.426136	3.20277	5.899637	2.300098	2.872195	2.450977
Ddx3y	7.359881	10.71253	9.798383	2.483115	7.589799	11.46887	12.89205	11.50066	2.448285
Zfp983	1.689773	2.426795	2.866188	2.392758	3.205767	1.837675	2.207531	2.355326	2.447965
Zfp580	2.9681	3.512099	3.169695	3.251624	3.449637	1.581752	3.743608	1.676394	2.445288
Frat2	1.770119	2.405046	1.693354	1.30102	3.273953	2.514735	1.802991	2.979852	2.445044
Rps15-ps2	2.170173	2.361159	2.170426	2.746567	2.44264	3.284032	2.039075	1.87838	2.444435
Zfp189	1.214394	2.114726	2.024226	2.089326	2.643602	2.049982	3.008344	1.982096	2.443454
Cryz	3.105912	3.220565	3.944474	3.40717	3.756237	3.442141	2.598665	2.792847	2.443348
Gm39469	2.525051	2.875405	3.109566	4.178599	2.081794	2.160828	2.064442	2.278509	2.442517
Mmp23	1.281311	3.32254	2.221197	3.39769	2.074521	2.527822	1.677445	2.805848	2.442407
Hic2	2.69363	2.485847	1.967978	3.017708	2.615359	2.450547	3.66982	2.364761	2.44224
Rps12-ps24	2.911663	1.277721	0.93184	3.126981	3.771329	3.554251	1.083366	3.215739	2.442065
Gm10566	2.33279	1.961626	1.052165	1.773906	1.394023	1.988092	1.796472	2.351694	2.441332
Gm7143	2.560512	2.791248	3.990099	3.967287	2.552027	1.982412	3.508041	2.778025	2.440395
Rsph9	1.66731	1.959813	2.322595	3.079088	1.417793	2.257747	3.015684	1.81422	2.440395

Lsmem1	0.822659	1.413029	0.502794	0.908945	1.61051	1.732863	1.606045	1.827588	2.439319
Rem1	1.971285	2.552399	2.559509	2.304145	2.206804	2.142804	1.930189	1.942711	2.439156
Cdh24	1.668983	2.552295	1.863915	3.34693	3.311503	2.443754	4.829937	3.125104	2.439057
Gm24620	0	0.425311	3.41197	0	0	1.577458	0.440754	0	2.438649
Ikzf3	0.931599	0.866128	1.184374	2.527921	2.842192	3.081008	3.280233	1.518379	2.437955
Gm7600	1.85433	1.913165	1.726646	3.052043	2.131432	3.07486	1.453929	1.826383	2.437712
LOC118567393	2.307321	2.238984	1.995753	2.542439	2.435031	2.133738	1.611305	2.968643	2.43682
Traf4	1.6727	2.254883	2.316316	2.22996	2.170765	2.44778	2.260763	2.257606	2.435145
Aasdh	2.905963	3.858964	2.85813	3.735836	2.492375	2.12315	3.750173	2.537472	2.432999
Gm5421	2.32933	2.52514	3.099382	1.571044	1.292476	1.629617	2.009722	2.429883	2.432412
Ccsap	2.903602	4.012851	3.484728	2.557356	2.074019	3.030395	2.465122	1.994399	2.431351
Inpp4b	2.493689	3.118093	3.75958	3.756592	2.339515	2.736326	2.981266	1.665509	2.429917
Glcci1	2.598816	2.641934	2.900174	2.544347	2.608872	1.948625	3.339359	2.426561	2.429086
Gm7446	2.548363	1.96192	1.699106	3.156495	1.532863	1.198995	2.079363	2.298591	2.428816
Tmem67	2.482547	3.377769	3.995556	3.400512	3.147248	2.463024	3.466427	2.520159	2.428765
Mex3b	3.180872	3.981691	4.041476	2.259325	1.715646	2.506339	2.851482	2.765826	2.428417
Gm4824	1.648159	2.764524	2.172825	1.266443	2.283183	2.563369	0.387149	1.925847	2.427664
Stil	2.748578	3.143647	3.366769	2.482431	1.957283	1.795583	3.25779	1.80063	2.427372
Ift122	4.026073	3.932564	4.109563	2.889139	3.215088	2.46933	3.174484	2.555478	2.426274
Frat1	1.633026	2.178622	2.714957	2.21438	2.302617	1.706298	2.082368	2.382682	2.425588
Prkce	3.769694	3.618671	2.992542	2.881719	2.558028	2.157021	2.958652	2.938591	2.425176
Cenpi	3.604906	4.653741	4.784637	3.450705	2.26211	2.476911	2.907556	1.523651	2.424323
Mblac1	3.136404	4.269371	4.196488	3.498153	4.036591	3.723549	3.592077	2.82475	2.423734
Mir692-1	1.017212	0.507252	1.01733	1.517271	0.484391	0.470343	0.52567	0.484243	2.423734
Arhgef25	2.201795	2.927911	1.677753	2.293722	2.096966	2.47247	1.354563	2.146238	2.423275
Rbak	2.755501	3.402491	3.313547	4.142719	3.046073	3.33693	3.882025	3.404313	2.423017
Prkdc	3.944275	3.688933	3.748315	2.663611	2.015928	2.154219	3.687551	2.420452	2.422971
Gm30646	1.616944	1.151885	2.194678	0.574245	1.319965	2.029334	2.268046	3.078977	2.421714
Doc2g	1.904815	2.612148	1.984413	2.525527	2.947954	2.752365	3.527293	1.926919	2.420611
Gm26347	0	1.266194	2.116203	5.04985	0	1.565416	0.437389	1.208759	2.420034
Ank1	1.77356	1.65472	1.563966	2.446319	2.161346	2.010486	1.63596	1.579661	2.417397
Top1mt	3.672553	3.895333	3.906185	3.999789	2.581635	2.425901	4.338019	3.829643	2.416853
Gm47815	3.251128	3.495133	4.518326	3.358874	2.975702	2.577035	2.967452	2.291394	2.414504
2900093K20Rik	1.313254	2.338852	1.876295	1.399176	1.518742	1.301203	0.678657	0.446552	2.413891
Jmy	2.715076	3.444634	3.2711	2.963575	2.212971	1.973619	3.602335	2.560972	2.413189
Fam185a	2.424098	3.679024	3.056826	2.724935	2.810572	3.021462	2.323873	3.445244	2.410832
Gm5063	1.887102	2.343719	2.635127	1.770312	1.865076	2.008549	3.164822	2.712009	2.409413
Itgae	2.768299	2.904727	2.263924	1.505433	3.432939	3.33338	1.624313	1.331573	2.404823
Swsap1	3.063208	2.456023	3.183704	3.404408	2.45974	2.33286	2.390003	2.201652	2.404302
2700099C18Rik	2.478408	4.076317	2.478696	2.767186	2.981568	3.417826	2.561558	1.117746	2.403584
Mthfd2l	2.718764	2.834777	2.595485	2.113672	1.718367	2.239948	2.631161	2.494401	2.402771
E130102H24Rik	0.963496	1.256602	2.520205	1.547698	2.823455	2.056179	3.447072	2.822592	2.4017
Mcm8	2.519912	3.987893	2.624346	2.381542	2.08259	2.176264	1.894149	1.58625	2.4017
LOC118567369	1.511947	1.340375	3.024246	4.009275	1.599958	1.864269	1.562673	0.319894	2.4017
Gm22208	0	0	0	0	0.479987	0	0	0	2.4017
Map7d3	1.963638	1.800155	1.448103	3.313569	2.02127	1.907624	2.808525	1.472999	2.400841
Gm38592	2.249596	3.198806	2.940408	4.141763	2.460682	2.760076	5.455857	2.947675	2.398805
Msh3	3.492918	3.707338	3.33308	2.565183	3.341877	3.111604	3.461064	2.669634	2.397535
Gm11956	0.804908	1.204148	1.308128	1.300649	0.574939	0.093044	0.519946	0.862146	2.397341
Zfp874a	1.437283	1.757691	2.447088	2.160863	2.216238	1.756383	1.892249	1.759416	2.397252
Gm7099	3.335031	3.053517	1.804407	2.881419	2.967969	2.325739	2.994866	2.65474	2.396963
2810013P06Rik	3.471713	3.053517	3.882209	3.805648	4.087466	3.438049	2.429797	3.747868	2.396963
Stk26	1.518389	1.951824	1.77166	1.409225	1.333623	2.106238	1.412398	1.172587	2.395853
Dhrs13	2.733758	3.836093	2.797659	3.066152	2.997168	1.851975	1.872699	2.935722	2.393437

Lpar1	3.022032	2.162578	2.236905	1.561975	1.54477	1.057873	2.205806	2.162017	2.392084
Smo	1.872559	2.261649	2.14768	2.647898	2.552396	2.812001	2.148453	2.682468	2.390536
Gm14276	3.52418	2.636098	3.117906	0.606538	0.774552	0.376044	2.661766	1.096946	2.389956
Sh3rf1	4.779412	3.928247	4.079321	2.616274	3.143304	2.116345	3.620346	3.75006	2.388885
Gm5171	2.763706	2.149017	2.951419	2.492034	2.699041	2.360855	2.444909	3.077305	2.388509
Ncf2-rs	2.940672	2.320772	3.759383	1.907087	2.313592	2.743089	2.193604	2.921539	2.388406
Gm16973	2.161636	2.208464	3.611934	3.224292	2.962547	2.364687	2.942551	2.007892	2.386853
Arhgef12	4.051529	5.193729	4.402041	3.933914	2.777811	2.589361	3.935333	2.595197	2.385618
LOC118568152	2.453735	2.186168	1.783255	1.398928	1.464464	3.161665	2.823478	1.837808	2.3854
Myom1	3.792118	4.914709	4.755136	3.962291	3.813233	4.966528	3.859657	3.830394	2.385021
Slf1	3.13245	3.816028	3.973838	2.592269	3.283645	1.924714	3.606928	2.562061	2.384395
LOC115489609	0.833655	0.831436	2.918132	0.828985	0.793964	0	0	0.396861	2.383642
Acad10	3.067006	3.187363	2.990034	3.17797	3.534635	2.526426	4.128866	3.190014	2.382719
Lsm11	2.199309	3.170227	2.732272	2.229705	2.233693	2.184803	2.432923	2.57655	2.382719
4930556M19Rik	1.985183	1.026614	3.382556	1.023588	1.890666	2.039816	1.367857	2.030095	2.382588
Grk4	2.568827	3.08131	4.061996	3.486462	2.115912	1.348297	1.327505	1.817806	2.382149
Lamb2	4.159826	4.099596	2.967425	6.567468	4.365461	4.849617	2.557219	3.65085	2.376931
Alas2	1.232594	1.686731	0.34402	4.731732	3.276025	4.373898	2.399761	1.064383	2.376863
Dnm1	3.486793	5.633955	3.545643	3.060488	2.059257	2.539951	3.744704	2.596467	2.376383
Gm5165	1.789786	1.694641	3.421381	1.937461	2.07138	3.456934	1.592262	2.847277	2.3752
Mir687	0.6229	0	0	0.61941	0	0.576038	3.218989	3.558369	2.374715
Gm12693	2.560809	2.692045	2.041964	2.546465	2.241139	2.112139	2.646724	2.602881	2.374715
Gm13464	2.686426	1.997277	1.856292	1.457115	2.232893	2.393984	2.726072	2.046193	2.374191
Arhgef10	2.608318	2.841963	2.548306	3.088461	2.728239	4.09916	3.693127	3.244177	2.371001
Gm17898	1.841796	1.285825	2.486714	1.648331	1.140169	0.340647	4.092715	0.789107	2.369784
Jsrp1	2.384433	4.260735	2.185984	4.742153	2.176287	5.88013	2.00235	3.074248	2.367267
Arhgef4	3.000567	2.43534	2.131685	2.572206	2.621207	2.755694	3.700095	2.088444	2.366737
Mrpl48-ps	2.421147	3.962587	4.47033	2.839716	3.133624	2.526053	3.464829	3.960163	2.366737
Zfp873	1.253382	2.067383	2.145461	1.677794	1.12484	1.783213	1.644204	1.262189	2.366197
Gins3	2.941987	2.773693	3.126224	2.856941	2.232777	2.784423	1.995453	2.078912	2.365854
Sox4	3.072806	2.972109	2.597693	5.061908	2.948594	3.377793	4.542134	2.815212	2.365029
Xpnpep3	3.450737	3.244084	3.866028	2.456363	2.020355	2.266925	3.517786	2.70196	2.363305
Hspa1l	2.433306	2.861826	2.640215	3.10449	2.951475	2.483761	3.084365	3.234702	2.362915
Gm45855	3.929518	2.930466	1.841069	2.393788	3.236696	3.077353	3.000279	2.831244	2.361825
Slc25a14	2.28411	3.552181	3.94926	2.733278	2.839031	1.503652	2.280717	1.658667	2.361448
Katnal1	2.953335	2.595249	3.656078	1.808634	1.852282	1.548763	2.605724	2.031744	2.359962
Cd200r3	4.640463	4.566401	6.311763	3.014787	4.301672	4.005264	3.261382	2.474178	2.358812
Gm23656	0.494983	0	0	0	0	0.457745	0	0.942544	2.358812
Cibar1	3.325473	4.327784	3.082504	2.661607	2.510541	3.787861	1.3832	2.23949	2.357777
Cd300e	1.421489	1.105809	1.762851	1.894125	1.35381	1.866659	2.438838	1.190989	2.357361
Gm46272	2.854317	3.568421	3.839703	4.077598	4.001054	3.60619	3.677211	3.751038	2.356418
Medag	1.81224	2.608429	2.389139	3.440351	2.765454	2.304366	3.150113	2.921467	2.355308
Tmem220	1.67045	2.394879	1.287788	1.626487	2.386378	1.930975	2.877489	2.15371	2.354963
Ppp4r1l-ps	2.172338	2.3894	2.50779	2.925487	2.222601	2.00891	2.96369	2.434659	2.354376
Prss23	2.222461	2.308901	1.574426	5.739893	3.557144	3.568164	1.658962	4.408335	2.353559
Ccm2l	3.098721	1.979833	2.324311	6.114581	4.472895	3.805876	1.751467	2.304911	2.353456
Mctp2	1.620267	1.392206	0.860088	1.412891	1.780528	1.53295	1.365465	1.13919	2.352024
Mlh3	2.558058	2.248728	2.952726	3.75024	2.532561	2.412363	2.717031	2.541414	2.351327
Scarf2	1.995502	2.341399	1.760942	2.467899	2.156023	2.093496	1.958462	1.979742	2.349394
Sp3os	2.513321	2.265608	1.595178	1.057372	2.853979	1.430302	2.198005	1.748678	2.349353
Gm10182	2.463914	1.323191	1.895539	1.130821	1.128175	1.621276	1.175344	2.481227	2.348329
Zfp296	1.847935	1.445503	2.174294	1.333149	1.587409	3.283793	1.984833	1.276439	2.348329
Rps11-ps2	2.566577	0.307169	1.84815	2.245936	2.737705	1.613973	1.591611	1.46618	2.348329
Phf11	1.355153	1.228677	1.909755	1.408815	2.229274	0.683565	0.763973	1.524827	2.348329

Mboat1	3.040338	2.993861	3.136916	1.798677	2.180852	1.7795	2.128041	2.454999	2.347514
Dusp13	2.943082	1.976799	2.943424	3.344682	3.374992	3.443746	1.924418	0.972158	2.347057
Tctex1d4	2.285502	1.928737	3.692393	2.505797	1.562749	1.950979	1.998768	1.897044	2.345846
Plekha8	2.959897	3.330992	2.620213	3.480273	2.628502	2.163883	3.224563	2.303997	2.344517
Gm45871	1.404829	2.259256	2.722172	2.601838	1.7895	1.315381	1.724204	2.691789	2.343122
Plac9a	0.98338	5.639383	2.089926	4.767125	3.277962	2.273498	0.127047	3.159926	2.343122
Rdh5	2.063016	3.151951	4.521603	1.920516	4.473041	2.922606	3.72006	3.008977	2.342751
Tmlhe	2.505398	2.278252	2.751345	2.442513	3.158091	1.839902	1.98017	1.800731	2.341046
Rpl35a-ps3	1.938394	2.190998	1.03393	1.156522	1.599958	0.478018	1.869865	1.845541	2.340118
Rpl19-ps6	1.900734	2.527564	0.792064	1.732579	2.187371	1.098587	1.637086	1.508071	2.339942
Mrpl27-ps	3.373824	0.994158	3.067469	2.897436	2.77503	4.183645	1.743508	3.285216	2.338585
Gm52308	3.855545	3.320924	1.110059	0.697081	1.057085	0.594248	0.966036	2.057904	2.338431
Apbb1	2.913238	3.9411	3.346287	1.634895	1.098826	1.413721	1.758888	1.675198	2.336722
Gm38485	0.591565	2.107106	1.859419	5.88251	2.656027	1.641182	3.930503	2.172449	2.3358
Galnt3	1.938138	1.385909	1.846931	0.563639	1.375689	1.301975	1.26615	1.305635	2.335162
Best1	2.050303	2.204183	2.130433	0.820823	2.764192	1.945304	2.972231	3.270384	2.334794
Nphp3	2.815317	3.332648	3.341933	2.721055	2.330456	2.287202	3.072935	2.580254	2.332168
Abi2	2.919615	3.86711	3.762471	2.065561	2.384949	1.846223	2.862515	1.757951	2.331709
Snord53	0.815266	2.439285	1.630721	0	3.1058	0.753932	5.898324	0.776213	2.331062
Gm23344	1.630531	0.813095	0	0	0	0	1.685235	0.776213	2.331062
Zfp955a	2.528284	2.705242	3.53331	2.830468	1.770053	2.988404	2.23239	2.518765	2.329888
Rab23	2.324822	2.635711	2.702671	2.430353	1.532863	2.021295	2.217987	2.364806	2.329391
1700094D03Rik	2.102513	2.294062	2.12073	2.090736	1.831264	2.609069	2.804538	2.309768	2.329299
Gm38639	2.572532	3.07882	2.315548	1.918591	3.675077	2.497947	4.387086	2.816698	2.329259
Gm6682	4.994096	3.511644	2.587171	3.358385	2.361052	3.455482	3.193538	2.565577	2.328544
Gm50597	1.691331	1.030839	2.067422	2.896531	3.758545	4.431587	2.524997	1.520851	2.328428
Mblac2	1.699853	2.806551	2.457216	2.045443	2.013448	2.417401	2.258849	2.339239	2.328059
Iqcb1	2.31522	2.841915	1.984705	3.187732	2.059606	2.470434	2.550668	1.93786	2.327852
4933424M12Rik	1.709544	3.653555	4.005682	2.282814	2.139855	2.75534	3.432832	2.836767	2.327639
Erg	1.608305	0.985604	1.162765	2.967368	1.457913	2.974619	0.540736	1.217631	2.326988
Marveld2	1.660877	2.40939	1.88758	1.651574	1.030567	1.885001	1.534536	1.725074	2.326476
Nkrf	2.358409	1.87965	2.81188	2.642189	2.069572	1.98098	2.075629	2.000302	2.326298
Cfap43	2.44019	2.08385	2.531991	2.183869	1.946358	2.256611	3.02647	2.381382	2.325718
Gm51868	0.128329	0.127987	0.128344	1.53132	0	0.118675	0.795806	0.244363	2.323867
Gm6483	2.198406	1.620583	1.864083	2.04352	2.139254	2.033017	2.123978	1.592574	2.323024
Mir7063	3.655257	3.645526	1.218561	4.240579	1.160409	1.690134	3.148242	1.160054	2.322523
Gm7496	1.792773	2.017231	0.873504	1.691309	1.050718	1.742921	2.803136	1.663129	2.322042
Gm38561	2.835468	3.066899	3.374999	2.051811	2.104591	1.889673	2.400889	2.103948	2.321826
Hr	5.627666	6.791649	4.92099	2.863376	4.888225	3.998987	3.686203	3.693907	2.320724
Tmem267	3.521948	4.423238	4.348589	5.23171	3.312854	4.704536	2.471679	4.1812	2.320701
Spry1	2.460497	3.246366	2.614581	3.491665	3.173286	2.417602	2.596026	2.245024	2.320644
Tmem40	0.229652	0.045808	0.459358	0.730771	0.393693	2.293653	0.284829	0.655954	2.320118
Kantr	2.275934	2.834063	1.868325	2.106201	2.342985	2.08038	2.597038	2.104284	2.31963
LOC118568730	2.526793	2.10199	2.515441	2.964219	2.37322	2.196712	2.864298	2.250544	2.319474
5430425K12Rik	3.981984	3.177106	2.25672	1.495879	1.68551	2.086701	1.417588	1.263746	2.319279
Fkbp11	2.172338	2.426541	1.303554	4.665966	2.896475	0.803564	0.269426	1.240967	2.318883
Gm4356	3.472113	6.69488	6.250529	5.178995	4.409069	5.244471	4.186702	4.848494	2.316462
Sirt5	3.401605	2.867512	3.1995	2.335571	3.008248	3.0708	1.674156	3.315772	2.315737
Mir7060	0.809315	2.0179	1.214113	0.402391	1.541565	4.116359	3.764103	1.926367	2.314047
Siglecl1	1.81963	3.266614	0.727937	1.326921	1.848529	2.916744	1.504543	0.923982	2.312359
Chrna1	1.167793	1.971003	3.426779	1.812062	0.574429	1.495299	1.657928	2.309233	2.311636
Gm3150	2.309919	1.727827	0.577547	1.607886	0.76998	1.708913	1.074338	1.099635	2.311636
1700030K09Rik	2.60616	3.967234	3.246647	4.955794	3.352984	2.854061	4.4657	4.135534	2.309594
Zfp280b	2.957303	2.972118	3.41267	2.284727	2.383195	2.345635	3.080032	1.97095	2.309062

Rad54l	5.169335	5.17587	5.230998	3.784452	2.267781	2.898376	4.05966	2.267088	2.308242
Svip	2.833835	2.152473	2.552625	1.530283	2.180579	2.377668	2.637958	2.340726	2.307388
Gm10155	8.265311	2.613731	2.116972	2.70626	3.455909	3.169256	1.562673	3.166948	2.305632
Ankrd1	1.476254	1.879563	3.895251	4.77876	2.841851	2.904668	2.986638	3.020413	2.305088
H2ac19	2.232405	3.618001	2.790831	3.237355	3.454942	3.78484	3.941641	3.01108	2.304987
Gm17383	1.987536	1.522081	2.094255	1.517595	2.230927	1.247222	2.0909	2.399203	2.30022
Ss18l1	2.750332	2.890917	2.66975	3.740413	3.287073	2.543421	4.33358	2.785456	2.300072
Il18r1	1.48412	1.973558	0.927683	0.768649	1.884613	2.60197	0.798913	1.413028	2.298559
Gm9949	5.451983	3.949319	3.788134	4.051816	7.160059	3.396597	3.143679	3.059853	2.297278
1700096K18Rik	1.912977	2.365776	1.607087	1.179402	1.931212	1.16758	2.095786	2.732012	2.297278
Gpi-ps	2.994892	2.467455	2.734785	1.521429	2.666283	1.866458	2.657981	1.983604	2.295929
Gm12435	3.850473	2.923806	3.063232	2.915189	1.75023	1.659008	2.170722	2.999477	2.293652
A230083G16Rik	5.482271	4.630786	4.643687	4.116485	3.836024	3.052246	9.077484	3.888113	2.292642
Chac1	2.014002	1.832444	1.130799	1.054064	0.975882	0.784205	0.584301	1.278352	2.289957
Gm34795	2.614602	1.547624	1.530677	3.064989	1.842283	0.943571	3.603095	3.015563	2.28935
Spata7	2.754248	3.380819	3.425552	3.300629	1.816003	1.56741	2.919644	1.512873	2.288499
Cbx6	3.978029	3.541269	3.805514	4.411011	3.187877	3.358865	2.681408	2.692884	2.288425
Gm4285	2.521761	3.732005	2.888158	2.305406	3.060227	3.422841	4.287892	2.788216	2.287165
Sag	1.292119	1.472776	1.169196	1.835545	1.6408	1.365613	1.462657	1.288806	2.287079
Hspe1-ps3	0.378848	2.267035	1.515567	2.511505	1.804053	0.116782	0.783116	1.442801	2.286812
Noxred1	1.531221	0.587363	0.589	2.693909	1.907032	1.633876	2.150704	1.420491	2.282635
Gm10362	0.920898	2.112427	1.381508	1.648331	2.894276	1.788397	1.903588	1.841249	2.282014
Gm14305	2.872793	2.6946	3.523001	1.904467	2.833731	1.707859	2.297576	2.637495	2.281689
Il17c	3.701011	1.845579	2.853194	2.606865	2.349868	4.278224	2.789194	2.936438	2.278108
Ssc5d	4.677667	5.626453	6.81168	4.050864	3.377936	3.279972	2.589965	2.777381	2.278108
Rnu5g	0.477914	3.336494	1.91188	0.475237	1.820642	3.093721	7.903173	2.275106	2.277474
LOC115487699	0.477914	0	0	1.425712	5.006764	0.44196	2.469742	0.910043	2.277474
4933430I17Rik	2.528425	4.763199	4.682813	2.2349	2.541825	2.598009	2.758434	3.388064	2.275935
Rnf125	0.799313	0.89968	0.799406	1.510188	1.413763	1.995785	1.546043	1.967791	2.274566
Fzd5	3.184411	2.786048	2.239935	2.518681	2.24939	1.604224	3.139732	2.884542	2.274329
Rnf217	2.36484	1.604584	2.578363	3.006956	2.898385	2.31241	2.544352	3.248152	2.272378
Gm36556	2.303185	2.055258	2.343864	4.741289	2.193528	2.242014	3.758615	0.96178	2.272162
Prrx1	2.002156	2.010317	3.206527	2.905697	2.048555	2.239351	1.999418	2.33129	2.269249
Gm5345	2.0649	1.701245	2.783449	1.473043	1.966588	3.154916	3.526031	1.795031	2.267516
Eid3	2.209026	1.779463	1.189614	3.252735	0.971009	0.982134	2.546578	1.051605	2.267352
Zfp933	1.382721	2.203886	2.016164	2.968405	1.907642	1.601359	2.043495	2.300774	2.266219
Nipa1	1.971647	1.995315	2.928815	1.441619	1.601631	1.796504	1.97786	1.794383	2.266039
Gm26947	2.159179	2.382519	2.228348	4.545423	3.412713	3.706716	4.53446	3.641301	2.265867
Abhd15	1.757501	1.803875	1.962486	2.986286	3.298897	2.603607	3.209676	3.086694	2.26051
Gm8292	0.903268	3.153021	1.467981	2.357796	1.505459	2.401527	2.217234	3.117498	2.259848
Pms1	1.700776	2.652643	2.859084	1.547313	2.033367	1.422235	2.150545	2.032745	2.25904
Snora64	2.369148	7.56109	6.634385	10.36586	9.025402	7.88729	5.876718	6.766983	2.258008
1110019D14Rik	1.776387	3.063491	3.257087	3.128064	2.431978	2.737911	2.486229	2.184588	2.257406
Gstt3	3.966204	4.782198	3.937063	3.355333	3.269962	4.215257	3.273298	3.156239	2.256803
Gm11585	2.366814	1.089467	3.277508	2.715642	1.733944	2.357121	2.44622	3.293487	2.255784
Ctsf	4.873989	5.116856	4.01937	3.769646	3.148914	2.530421	3.3878	2.360964	2.254758
Sulf1	1.679447	1.918905	2.315623	2.221315	1.739248	1.914988	1.820054	2.126823	2.25336
Gm7476	2.435264	2.500215	2.650448	3.205089	2.660395	2.980662	2.516967	2.659582	2.252757
Gm5785	2.377275	1.827784	2.213285	2.415536	2.041799	2.070521	1.965626	2.156402	2.249276
Tbc1d32	3.342682	3.031855	3.292609	3.085636	2.45072	2.193008	2.789937	2.269826	2.248144
Pou2f1	3.38695	2.821727	3.351066	2.335492	1.982053	1.870062	3.903594	2.262044	2.247113
Aldoc	2.936294	3.196577	2.274859	1.891732	2.461703	3.116965	2.244047	3.70127	2.246723
Plscr4	2.881033	4.719281	4.278395	2.378731	1.92902	2.309051	3.212298	2.526909	2.24663
Rps15a-ps6	2.960285	1.878802	1.480314	1.739461	0.768912	1.555442	1.668874	2.177917	2.244307

Ralgps2	2.225196	2.181008	3.248652	2.441634	2.301946	2.282493	3.00038	2.313418	2.242695
Gm14335	1.935493	1.792459	1.060036	2.657852	1.316673	1.065407	1.381249	1.886655	2.239989
Gm26782	2.26959	2.385601	2.815063	2.356444	2.41583	2.078268	2.322737	2.287982	2.237345
Gm10297	1.656928	1.170533	2.899962	1.922255	0.526013	2.553793	2.354713	2.169143	2.2372
Gm11696	1.463645	1.699707	1.784651	1.495322	1.794962	1.464782	2.113706	1.622608	2.235796
Hic1	1.968317	1.894795	1.694661	3.318885	2.494037	2.73827	1.574413	2.411795	2.234864
Gm4540	1.792773	1.788	2.344668	1.645598	1.838757	2.295555	1.140259	0.787798	2.234417
Psma5-ps	4.286448	4.332037	3.029442	3.353117	4.027935	4.333944	3.721417	3.917874	2.233333
Xlr4a	4.433053	2.384495	4.433568	1.783101	1.944962	2.533429	2.882918	1.896944	2.231229
Eif3j2	6.944081	2.651383	1.398146	4.329983	1.353251	0.635809	4.003044	4.82221	2.227951
Cldn15	1.078212	0.955859	1.048383	2.114562	0.827207	1.938799	0.154776	0.769923	2.226535
Mir221	1.750676	0.582005	0	0	0	0	0.603137	4.44484	2.224733
Il1r1	2.438799	2.728929	2.593756	3.97486	2.334016	1.749252	1.401711	3.194132	2.222346
5430437J10Rik	1.616547	2.133852	1.569184	1.560213	3.305573	1.363027	2.555314	2.987687	2.22044
Zfp709	2.259363	3.004463	3.654455	2.912398	3.108144	2.0249	2.782016	3.359488	2.219835
Bard1	3.629704	3.42207	2.722595	2.566038	2.835731	1.809437	3.253237	1.592924	2.216198
P4ha3	2.162044	1.83081	1.387133	1.054684	1.08783	2.753877	0.632428	1.475889	2.216138
Gm6988	2.102442	1.876124	1.660015	2.090665	2.634661	3.786214	1.601142	3.265981	2.214741
Gm25360	0.870755	0	0.580571	0.288626	0.276432	0.536831	2.399916	0	2.213085
Baiap2l1	2.662666	2.178935	2.560553	2.698669	2.357081	1.436371	3.739894	2.941388	2.212403
Mfap1b	2.820378	2.490562	3.378971	4.645086	2.434276	4.319827	4.342138	2.601362	2.212059
Sytl2	1.56297	1.889465	3.149987	4.356511	1.98474	2.102379	1.444076	2.547808	2.211903
Magee1	3.27926	3.039125	3.589042	1.999603	1.414248	1.730846	1.998396	2.533087	2.211387
Ssc4d	1.465323	1.441125	2.035408	1.902345	1.841361	1.693856	1.009656	1.957059	2.211257
Sned1	5.734258	5.739638	4.264962	6.216771	3.903706	3.292752	3.423331	4.020771	2.209779
Tpbgl	3.742115	2.840077	4.034651	2.541275	3.216247	4.389042	5.471327	2.67649	2.209532
Cdon	1.900179	2.393108	2.968507	2.78603	1.809709	1.398085	1.304508	1.795951	2.207617
Gm34931	1.462103	2.106304	2.843308	1.615459	1.972695	2.629097	2.644523	1.701413	2.206397
Rab11fip2	3.539491	3.09142	3.435172	3.061483	2.144258	2.41133	3.495893	2.392858	2.205717
Recql4	2.954324	4.064693	2.509687	3.73416	2.389921	1.382491	2.979864	1.474181	2.205092
H3c6	2.069039	1.395917	1.947557	1.270777	2.49214	4.4458	2.956099	2.839013	2.203975
Cgref1	1.422629	3.103715	2.045266	0.928371	0.762129	1.43894	1.286563	1.989395	2.203324
LOC115489645	0.769973	3.839616	3.465282	1.91415	2.933256	4.628306	4.376931	1.46618	2.201558
Zmat1	1.898564	2.256432	2.120309	3.209479	2.501304	2.662861	2.943391	2.364968	2.201558
Zfp119a	1.493921	1.679573	2.607875	2.322865	1.836698	2.034619	1.740557	2.094748	2.20048
Gm12671	2.584955	2.393925	2.262099	2.937687	2.945468	2.47586	4.198355	1.933746	2.199725
4930579G24Rik	3.292514	4.350266	4.643829	1.98683	1.527675	2.75855	2.152311	2.197037	2.199323
Gm15694	2.249828	1.553426	1.500059	1.032566	1.043885	1.227003	1.43096	1.977283	2.199267
Ctxn1	2.080058	1.713734	1.537613	2.383164	1.679564	1.714488	1.074922	0.861052	2.197967
A530040E14Rik	1.595765	1.915715	2.275707	3.144267	2.589271	2.732803	2.565582	3.263735	2.196864
Gm38958	1.698321	1.451829	0.849259	4.34265	1.848529	1.234007	5.892793	1.963462	2.196741
Mtss2	1.678151	2.380873	3.191221	2.162325	1.722061	2.688505	2.332961	2.306631	2.196396
Nrg4	2.962842	3.151951	3.687521	3.011718	2.696366	3.653258	3.742744	2.131359	2.196329
Cetn4	2.512773	4.333436	4.92142	1.874024	2.542709	4.30859	2.867606	4.98418	2.195322
Plxna3	1.615681	2.346572	2.151126	2.199076	2.596653	1.624868	3.22497	1.70173	2.19434
Gbp8	0.879254	1.079277	1.916545	3.923268	4.186957	3.085634	3.332092	3.842237	2.19171
Lig4	1.905859	2.431886	1.976157	2.243562	2.095395	1.866154	2.751926	2.108097	2.190428
Tubb4a	3.5409	2.507076	3.189884	2.177147	2.188143	1.999703	3.687634	2.753644	2.189751
Gm10179	2.841427	2.532388	2.176665	1.923752	1.669749	1.509509	2.624336	0.863399	2.189554
Gm16174	4.276214	5.178722	7.026026	2.733596	2.909014	3.672044	4.577527	3.635156	2.183364
Gm32725	1.832663	2.132415	2.940239	2.088164	1.63632	1.87133	2.920146	1.308656	2.183364
Mir466i	0.458166	0	0.458219	5.467193	0.872704	0.847395	0.473537	2.181094	2.183364
Acad12	1.867387	2.560822	3.198273	1.949774	2.289792	2.633525	2.243669	1.955729	2.180238
Gm17907	3.075511	4.561661	2.997	4.783469	2.40335	2.114871	5.949864	3.528842	2.179637

Fdx2	2.727671	2.720409	2.064423	1.832697	2.597804	3.136037	3.504931	1.965305	2.178137
Lmbr1	1.586249	2.94954	3.629975	3.448812	2.048442	2.287391	3.251148	2.354989	2.178069
Ccl25	2.618682	5.556831	3.677726	3.213464	3.183835	3.091501	5.009976	4.668198	2.177219
Prdx2-ps1	3.148487	1.539267	2.654915	1.350563	2.939789	2.854531	2.679862	1.763334	2.177042
Cbfa2t2-ps1	3.422931	1.137939	2.080847	1.001105	2.173308	0.931006	1.595465	2.108743	2.174905
Zfp27	2.986125	2.127268	2.578383	4.297328	2.367017	1.852418	2.54956	2.154386	2.174306
Slc6a12	2.013724	4.887823	3.906593	0.241258	0.716305	0.583349	0.601817	0.923982	2.173617
Gm6457	3.355041	2.907928	3.475268	1.946145	2.206282	2.733276	2.600702	2.890106	2.169836
Zfp593	2.2164	1.371069	2.272776	2.343478	2.084155	2.776118	2.000791	2.323925	2.165906
Cenpf	4.379222	5.412792	4.689895	2.573469	2.296699	2.38338	3.807709	1.797088	2.165885
Gpr55	1.012455	1.723979	1.432419	1.792565	2.587014	2.443479	2.169412	1.974934	2.165274
Ptrhd1	1.927323	1.867272	2.326824	2.559932	1.927339	2.291564	2.518437	3.02775	2.164929
Mical3	4.315425	3.888644	3.236945	2.729085	2.361426	1.750337	2.914784	2.901324	2.164728
Mars2	2.827687	3.088746	2.520205	1.664155	1.538891	1.618783	2.664099	1.666622	2.163363
Gm19696	0.147966	1.180579	0.641262	1.128054	3.100274	2.782305	1.121491	1.643582	2.162385
Gm51425	1.258223	1.692177	2.135414	2.331735	1.579601	1.445642	1.733915	2.305149	2.162191
LOC108167564	0.226741	0.113068	0.34015	0	0.43189	1.467778	1.054565	12.84481	2.161039
Cdkl2	2.129591	2.535003	3.126053	1.571169	0.556119	1.969379	2.165538	2.94326	2.160637
Hhat	2.238183	2.403934	2.353235	2.082976	1.831007	1.194116	1.364238	2.650052	2.160535
Gm11714	0.367545	0.427661	0.673911	0.060914	0.408387	0.283245	0.189938	0.116646	2.160203
Ccdc106	0.923268	4.185501	2.728155	1.669266	2.99765	2.018095	3.079605	2.397387	2.159894
H2bc21	2.785414	4.934058	4.240975	4.258069	3.484272	2.61431	3.609323	2.849897	2.159459
Gm5858	0.476887	0.951234	1.31159	0.948431	2.725089	2.425554	2.217987	2.383724	2.158947
Amot	3.133374	2.786173	3.001592	2.064701	2.139269	2.077228	2.633718	1.401782	2.158831
Gm42127	1.859058	2.293865	2.216827	1.694591	1.566253	1.388584	2.869827	1.554428	2.158019
Dbp	2.586032	4.352311	1.899338	4.821649	4.348573	3.886157	3.925665	4.347244	2.156629
Rpl17-ps4	1.085248	2.796092	1.537613	1.348961	1.033578	1.254504	0.560829	2.152629	2.154869
Gm3386	1.32945	0.928137	0	1.586404	2.025846	0.983547	1.099242	2.27838	2.154043
Sbk2	4.155121	7.924604	7.071817	3.805648	3.506022	1.314548	3.5411	3.50495	2.153793
Clec9a	2.033043	1.971307	1.882666	2.246283	3.137438	2.611242	2.626564	1.810197	2.152967
Gm15927	1.432081	1.702936	1.790309	1.451445	2.281907	1.808239	1.992479	1.783014	2.152343
Rufy4	2.681576	2.195854	2.314893	1.459588	0.833379	0.757003	0.671005	1.746874	2.152236
Anp32b-ps1	2.64691	2.248772	1.470677	2.534599	1.727275	2.221133	1.621164	1.773416	2.149001
Rassf7	3.162696	3.625605	2.944921	2.638891	3.531448	3.025616	2.479783	2.249745	2.148143
Myh11	2.028829	2.755834	1.605824	6.374197	4.504596	4.247343	0.308745	4.278058	2.147187
LOC118568702	1.572711	1.568524	0.607709	2.09705	1.702083	1.917156	3.80509	2.041875	2.1462
Zfp366	3.43724	2.104967	2.754132	3.078187	2.584413	2.528051	2.243722	1.033449	2.145681
Nectin2	1.945195	2.63288	2.556839	3.97913	2.831805	2.441304	1.751969	2.672195	2.145278
Zscan20	2.002045	2.573086	2.487365	2.647599	2.594719	1.860972	3.10382	2.043699	2.144183
Piwil2	3.216082	1.962644	2.519182	0.92811	2.109802	1.310282	1.336566	1.680902	2.143505
Camk2n2	1.081085	1.380105	2.119174	2.021056	0.947245	1.199705	1.385521	1.441019	2.143171
Cyp2j6	1.661432	1.876318	1.392832	1.773605	2.04772	2.485417	1.363637	1.302696	2.142371
Dancr	3.65217	2.61509	2.622375	1.303691	2.764792	2.68461	0.967872	2.318149	2.142057
n-TTagt5	0	0	0	0	0	1.385605	0	0.713277	2.142057
Gm13611	2.526291	1.539735	2.386219	2.233014	3.341684	2.076654	2.175874	2.67253	2.140249
Zfp775	2.397916	2.588998	3.190259	2.690748	1.927568	2.176829	2.773951	2.618178	2.138657
Xrcc3	3.524964	3.812349	3.159101	3.209323	3.051942	2.9846	3.903456	2.571565	2.137929
2310015A10Rik	1.556156	2.031311	2.311618	2.491832	1.678222	2.031649	1.608365	2.276891	2.137488
Ghr	1.370379	2.816294	2.159636	2.436348	0.949188	2.112139	1.030076	2.214096	2.137243
Galnt12	2.615758	3.221063	3.416898	2.999235	3.711409	3.20884	2.648343	2.210917	2.1369
Spib	1.837168	1.409444	1.696045	2.409069	2.557252	1.642946	3.025568	2.364255	2.135816
9330133O14Rik	2.397534	2.46955	3.69499	2.345021	2.676427	2.380726	2.78264	3.442742	2.135223
M1ap	2.799902	2.699366	3.03358	2.273778	3.066586	1.941946	3.231453	2.132625	2.134844
Kyat1	3.290999	3.992825	3.25745	3.508729	4.006748	3.168914	3.68194	3.036446	2.134191

Six1	1.10915	1.998918	2.004487	1.354485	1.056343	1.673523	1.287144	1.9453	2.132784
Clec3b	2.906027	3.399918	1.620858	4.556912	2.820893	5.374806	2.656964	3.564945	2.13054
Rpl28	2.906027	2.229454	2.123882	1.33373	1.277386	1.963872	1.617282	1.915493	2.13054
Cxcl9	1.603028	1.446498	3.950779	4.725216	4.743696	4.764955	3.254448	4.033634	2.128047
Flnc	2.832196	3.749761	4.186942	2.871674	1.248556	1.675554	2.019651	2.166642	2.127641
Gm2614	2.231617	2.577098	1.174672	3.620664	2.125368	4.018842	1.699517	3.019336	2.126929
Tarsl2	2.758725	3.119399	2.741472	2.201606	1.891044	1.8687	2.342771	1.790087	2.126894
Zfp947	1.355199	1.060479	1.000879	1.554932	1.707665	1.773825	1.120533	2.362209	2.126213
Nek3	1.895318	1.751282	1.142898	1.136364	1.194539	1.623854	1.411565	1.008413	2.125184
Zfp438	1.961463	1.867321	1.997358	2.305107	1.681268	2.341579	2.488012	1.816573	2.124373
Dync2h1	2.191521	3.056257	4.030639	3.084186	2.264061	1.75185	4.434117	2.104226	2.124117
BC035044	1.740399	1.799269	2.88685	1.287435	2.769299	2.983401	1.557488	1.697445	2.124015
H2ac13	1.237457	0.740497	1.485121	0.123053	3.064205	1.144361	0.255795	0.706908	2.122931
Gm34962	2.420172	2.397637	2.178408	3.28904	2.059084	2.282861	2.734829	1.474714	2.122107
Gm20075	2.379982	2.012439	2.018045	2.032233	3.104351	2.416242	1.604237	2.019675	2.1204
Gm5881	4.002213	4.730736	5.04041	3.390195	4.094009	5.071907	3.983284	3.52824	2.119147
Gvin2	1.538504	1.120053	1.551667	1.755818	1.749649	0.864462	1.476061	1.192859	2.115971
B230217C12Rik	1.479444	2.828052	2.301625	2.411063	2.583179	2.394251	3.185582	0.899923	2.115063
Orm3	2.463914	0.737206	1.10889	1.837584	1.877284	0.341783	2.29192	0.938355	2.113496
Gstm2-ps1	1.675461	3.096266	2.316348	3.185146	1.689555	2.643118	2.190057	2.627394	2.113496
Gm6285	1.542624	1.634675	0.385701	1.342235	0.918237	0.802446	1.494731	1.376934	2.113496
2010204K13Rik	2.116726	1.60845	1.612931	1.703942	1.823952	1.118561	1.250138	0.575809	2.113496
Mir674	0.554381	0	0	0	0	0	0	0.527825	2.113496
Zfp428	2.647627	2.047795	1.567145	1.558185	1.492358	1.998728	1.898764	1.954906	2.111436
Ndn	3.08735	2.911786	2.517154	3.93768	3.739368	3.630923	2.115725	3.610423	2.110937
Robo4	4.288182	2.57165	1.934111	7.720083	5.285199	5.676217	2.491218	3.549074	2.11029
Gm36298	1.246657	2.004565	2.442713	2.023957	2.035375	2.329266	2.445486	2.15587	2.109616
Tnfrsf18	1.608843	2.40684	2.346503	1.733151	2.745272	4.02948	2.771367	2.042369	2.108385
Zfp60	2.525783	2.547684	3.042774	2.126327	2.323521	1.592567	2.580858	2.227166	2.106383
Zfp382	2.74079	2.85901	2.272602	2.294373	1.911111	1.95914	2.695442	2.190116	2.105766
Sesn3	1.849648	2.357147	1.849863	3.11657	2.642377	2.209391	2.124115	2.372521	2.105661
Serpinb9b	4.07508	4.600483	4.075553	2.054267	1.078073	2.224465	1.169944	1.616615	2.103786
Cadm1	2.557681	3.780988	2.687825	2.646638	3.066523	4.034155	2.978959	2.632943	2.103597
Gm4221	2.309919	1.884902	0.840068	4.385144	1.999947	1.747752	1.519265	2.79907	2.101487
A930013F10Rik	2.795769	3.445737	2.29598	3.684209	2.359593	1.639547	3.829264	2.618564	2.101364
Gm52546	1.563329	2.35874	1.603601	3.906361	2.786911	2.224182	5.054488	2.747894	2.10127
Cp	2.551479	3.192151	3.216144	3.678171	2.962013	3.02969	3.13641	2.630499	2.100833
Rps6-ps1	3.940778	2.198296	4.676043	2.789586	2.353673	4.694363	1.311641	2.8617	2.100764
Hecw2	2.105863	2.295629	2.987734	2.824555	2.23881	2.807931	2.480214	2.098243	2.100427
Gm40158	1.91901	1.913901	2.914391	1.837584	3.316835	2.30046	3.819867	1.421066	2.099948
Gm31292	0.979039	1.586702	0.856758	3.894218	1.864851	2.376634	0.758914	1.398211	2.099499
Dennd2c	2.991196	2.635494	3.505429	2.883201	1.258358	2.698283	2.162189	2.026735	2.098804
Rab39	1.548758	2.382087	2.426047	2.913168	1.759361	1.949921	1.832148	1.758823	2.098557
Gm41453	2.414337	2.651748	1.833887	0.820531	1.047822	1.130483	1.421395	1.39667	2.097185
Ephb4	2.212918	1.977393	1.432807	4.439203	2.448666	3.714342	1.150191	2.374846	2.096916
Gm25777	1.319954	1.755253	0.880072	2.1876	3.771329	0	0.454746	0.418908	2.096722
Dtl	3.490398	3.46797	2.621396	1.85985	1.580571	1.997589	2.463991	1.178796	2.096423
Gimap3	1.03381	0.850623	0.930537	1.901835	2.092252	2.629097	1.442467	1.328789	2.09379
Gm19391	1.060291	1.133001	1.439133	1.280284	0.577034	0.560299	0.626208	0.144214	2.093285
Denn2b	1.384106	1.619694	2.19637	2.752706	1.247903	1.587173	0.896473	1.669219	2.093093
Popdc3	0.810794	2.088976	1.520416	1.310161	0.933065	1.968218	0.663414	0.964945	2.092889
Gm25008	0.548892	0	0.548956	0	0	0	0	0.522599	2.09257
Rbm12b1	1.621095	2.79568	2.837246	3.106486	3.216485	2.795267	2.984453	2.797487	2.092252
Ankrd24	2.161652	2.620244	2.444614	3.637689	3.056428	2.383457	3.609052	2.374736	2.091943

Ablim3	2.128484	2.576549	2.453728	6.091171	2.909185	4.447581	1.696102	2.212161	2.090577
Pde5a	2.304455	3.195225	2.7919	2.235655	0.99923	1.975154	1.684666	1.765956	2.089211
Gm15925	1.13462	2.526361	2.929243	1.285697	3.31719	1.756902	3.163526	2.537377	2.087341
Enpp4	1.582885	1.566337	2.547751	1.807661	2.437946	1.932676	2.441204	1.74254	2.086152
LOC115489082	2.917793	2.182519	1.459066	5.802897	0.694718	0.674571	6.031369	2.778025	2.085687
Jag2	2.691954	2.301246	2.125474	3.320935	2.737277	3.799664	1.443433	2.774982	2.083402
Gm12712	2.060198	1.867921	1.810688	1.365772	2.616147	2.886677	3.548863	1.783192	2.082555
Zfp235	3.175991	2.262526	2.609153	3.073606	2.646682	2.67482	3.927331	3.266843	2.081064
Ifnlr1	0.594177	0.771751	0.290213	1.112993	0.342164	0.907274	0.228506	0.092093	2.080835
Slc22a18	2.910612	3.583222	3.183852	2.261178	2.208965	2.859871	3.055267	2.20829	2.080554
Gm11703	3.213801	1.488149	1.607087	2.396849	2.732847	4.35189	3.084365	4.261939	2.07849
Ubtd2	2.576671	3.320986	4.142975	3.626552	3.341206	3.409272	2.191951	3.226958	2.077981
Gstp3	2.254844	3.256942	2.332868	1.623672	2.962054	3.091862	4.660988	2.072804	2.074961
Adh1	2.176369	3.63078	1.860023	1.967435	2.60036	3.695936	1.18604	1.130246	2.074273
Klrb1f	1.676698	1.387599	1.284429	1.986577	2.752051	2.672238	2.027922	1.52845	2.074055
Sgtb	5.031582	4.159218	4.760158	2.614388	1.899541	2.766678	3.982282	3.366339	2.073749
Glis3	3.325565	2.653369	2.76863	2.341669	1.677777	1.944969	3.827299	1.540344	2.073063
4931440P22Rik	1.519259	1.901446	1.906743	3.258478	1.957605	3.112958	2.586261	2.524256	2.072612
4930481A15Rik	2.652331	2.688635	4.43556	2.161864	4.099656	2.81468	3.640109	2.690871	2.072055
Gm6242	4.021977	3.252381	1.630721	7.998896	4.03754	5.528836	3.595169	3.208346	2.072055
Gm22573	0	3.252381	0	6.485591	2.588167	2.010486	3.370471	0	2.072055
Ccdc14	4.004205	3.683968	4.594506	3.24098	3.133624	2.899219	3.368584	2.186956	2.070895
Sgo2a	3.146242	4.264936	4.495153	2.822141	2.287083	2.778913	3.623432	1.870678	2.068455
Gm2223	2.059128	1.737701	1.996002	1.96884	1.41802	1.14253	2.193695	1.658878	2.068207
Cep295nl	1.955918	2.499349	2.261794	3.130174	1.60084	1.441365	3.095482	2.35688	2.068057
4933439C10Rik	1.995423	2.913061	2.140268	2.070517	2.451265	2.48715	4.035071	2.61572	2.067191
Zfp825	2.167666	2.414116	2.998953	2.442927	2.253704	3.374393	2.707139	2.390604	2.065978
Hspb8	2.747481	4.171142	4.946041	4.523128	3.256302	5.053338	1.388278	2.848393	2.065779
Gm19019	1.60008	3.003897	1.129599	3.275829	2.42031	2.001952	2.237443	1.881888	2.06326
Nudt17	1.229771	1.373676	1.328307	0.733729	2.998324	1.546665	1.830282	1.592373	2.062862
Gpam	2.814882	3.137115	3.089173	2.667612	1.772248	1.581086	2.606674	1.708753	2.06164
Fam120c	4.060998	3.507058	3.890297	3.388416	2.430247	2.129546	3.730883	2.355434	2.061296
LOC118567621	4.101975	3.282642	3.709402	2.491364	2.830585	3.361796	3.24951	2.82972	2.06012
Zdhhc23	1.640831	2.344931	1.851152	1.721181	2.66804	1.915241	1.923376	1.752748	2.059715
Mst1r	3.241089	2.370471	2.377075	2.762515	1.499292	2.026717	2.105606	2.67439	2.059364
Rab6b	3.730779	3.2639	2.029256	1.822398	1.807744	0.726338	2.97652	2.679629	2.0586
Gm7694	3.341036	2.380101	2.849613	3.00591	2.451905	2.233669	2.137651	2.547549	2.053945
Klhl8	1.494568	1.681246	1.946641	2.29842	2.168219	2.298193	2.263181	1.853178	2.053868
Rtl6	2.129343	2.535916	2.605616	2.12987	1.77745	1.447904	2.498535	2.075045	2.053329
Kdm8	2.584842	2.577961	2.539789	2.20961	1.943507	2.096826	1.96852	1.446391	2.052987
Gm26908	2.464446	3.054458	3.230474	2.759945	2.574986	2.168409	2.769692	1.822442	2.052381
Gm25323	1.614701	0.536801	0	2.140875	1.537823	0	1.112583	0.512451	2.051938
Ttc8	2.003785	3.232062	2.375133	3.985122	2.638752	2.996889	2.122148	2.237543	2.051251
Cplx2	1.433181	1.494337	2.801544	2.07295	2.419678	2.168773	1.885246	2.791082	2.048924
Lcmt2	2.652305	2.839986	2.375961	2.637448	2.742986	1.926101	2.3713	2.137946	2.04712
Gm7285	1.864889	2.367176	1.865106	2.191614	2.583418	1.62007	1.985864	3.443505	2.046709
Tarbp1	3.1225	3.376434	2.827012	2.189848	2.139074	1.479257	2.887548	2.806025	2.046666
Nanos1	1.707741	1.294991	0.861027	1.473623	1.586109	1.435696	1.575403	2.002186	2.044624
Gm51556	1.084995	1.700453	2.118569	2.286277	2.361913	2.365084	1.441795	1.524936	2.043571
Mid1-ps1	1.499905	2.778123	5.591206	4.687583	2.448846	3.945031	11.73743	6.212975	2.042204
Gm11449	0.787153	1.121511	1.012172	1.789129	1.178062	0.623944	0.581116	1.070638	2.03633
Csrnp2	1.773588	1.902871	1.773794	2.298094	1.59957	1.68986	1.902527	1.663044	2.036148
Gm20324	2.285081	1.634933	1.788532	2.025294	1.892423	2.15911	2.46443	2.695879	2.03585
Rnf113a1	2.135656	2.307468	2.269398	3.052808	2.033975	2.715608	0.919711	1.863907	2.03547

Tedc1	4.081431	4.268326	3.553076	2.234678	1.935687	2.551909	3.347365	2.09242	2.031602
Sh2d6	2.477125	1.432908	2.081027	0.68971	0.849307	0.916307	1.53614	1.132064	2.030392
Rab36	1.224856	1.434047	1.890759	2.72725	1.699091	1.526694	1.788843	2.307015	2.030255
Gm41077	2.425074	2.832615	1.758929	1.933537	1.550146	1.070806	2.653208	1.528872	2.030205
Meox1	1.51358	1.407208	1.795981	2.474489	2.174491	3.273901	2.20071	1.392226	2.029386
Nek8	2.198809	2.612883	3.134837	2.51214	2.628791	1.579122	2.538519	2.650259	2.028778
Dcdc2b	2.335873	1.294253	2.439974	1.858231	2.07635	1.296086	1.287596	0.988436	2.028402
Gm1943	2.223452	3.301659	1.680137	1.130065	1.505843	4.020971	2.553387	2.21103	2.024963
Cmtr2	3.115194	3.014158	3.952572	2.928216	1.889355	2.207206	2.530916	2.110118	2.023686
Ypel1	1.131389	2.679895	2.404481	3.44547	1.750974	3.138819	1.315515	1.144518	2.021839
Trim30b	1.580515	1.416681	1.180522	1.0743	1.733913	1.18409	1.033887	1.14289	2.021207
Fam221a	1.345188	1.829464	1.630721	1.094444	1.979948	2.148707	3.075555	1.280751	2.020253
Gm5888	2.117233	2.500574	1.393078	2.160777	0.795959	3.349126	2.994469	0.848763	2.017911
Adcy6	2.336851	1.809957	2.386849	2.311401	1.728385	1.919653	2.184005	1.763361	2.013982
B330016D10Rik	1.08197	0.719393	1.056331	0.999058	1.030456	1.119687	1.171521	2.133864	2.013321
Snora33	2.639908	7.372063	6.336515	4.200192	10.05688	0.976522	1.637086	3.518831	2.012853
Gm22572	0	2.63288	2.112172	4.200192	0.502844	0	0	3.016141	2.012853
Gm5880	1.861058	1.035135	1.216987	0.355891	1.567938	1.092204	1.886506	0.477053	2.012528
Gm7816	1.574072	1.761331	1.036705	0.87807	1.06036	0.710075	0.833281	1.571777	2.012505
Mfsd9	2.180939	2.14005	2.374685	2.116253	1.591328	1.675298	2.690389	1.892265	2.011576
Gm5763	1.026631	0.369741	0.399292	0.51044	0.733314	1.107629	1.23792	0.624484	2.0113
Gm32092	2.025135	2.188055	2.194151	1.594251	0.562542	1.794749	1.13375	1.205079	2.010555
Gimap1	0.295867	0.627043	0.702766	1.140062	0.598783	1.744254	0.267569	0.352118	2.009161
Epha2	1.810791	1.371843	1.584626	1.592881	1.144191	1.497446	2.051499	1.574854	2.007954
Fut10	1.979931	3.193067	3.1177	4.440363	1.323977	1.869935	1.567423	1.925196	2.0075
Ston2	2.417517	2.039677	3.180992	2.458611	2.302417	2.122733	4.0508	2.266839	2.007367
Gm17251	2.321346	2.061182	2.272637	2.746636	1.576495	1.249982	3.695013	1.883756	2.007074
Hdgfl3	1.772277	2.243042	1.969425	2.26735	1.875441	1.84022	2.474451	2.338651	2.005233
Oip5	2.06513	3.870688	3.098055	1.133	1.729434	1.053665	1.803213	1.017003	2.002188
Gm26387	2.939897	1.675469	2.520205	6.682124	1.999947	1.553557	6.511137	6.397875	2.001417
Cyp7b1	1.740438	0.765796	1.331078	0.814442	0.877539	1.088781	0.290987	0.877271	2.000308
Msl3l2	2.02064	2.428647	2.824043	3.503431	1.20894	2.180062	2.061657	2.145829	1.99992
A430046D13Rik	1.337437	1.490803	1.337592	2.007956	1.473566	2.788907	1.572036	1.822668	1.999523
Gm2308	2.279526	1.909704	2.006216	2.901449	2.605194	1.602106	1.743443	1.692859	1.998783
Gm35034	1.310593	0.784262	0.262149	1.042601	1.248194	0.121199	2.031844	1.9965	1.998578
Noxo1	1.789974	2.65231	2.761996	0.81369	2.43536	1.418839	2.272891	2.09377	1.998462
Olfm4	0.684918	0.502275	0.483528	0.500795	0.709865	0.577503	0.249846	0.326054	1.996762
Gm51604	1.570854	2.154174	1.440117	1.496969	0.810368	2.602713	2.367686	1.807192	1.996218
Iqck	1.689694	2.086433	0.965652	0.720098	1.379354	0.669676	0.582128	0.536252	1.993864
Gm22297	0.523001	1.043216	0	0	0	0	0.540547	0	1.993864
Mrpl23-ps1	1.094827	1.786766	3.08578	3.859916	3.412477	4.694141	4.011889	2.558576	1.992074
Tcea3	2.311772	2.172601	0.800322	5.87968	3.38724	4.111258	2.343383	2.074051	1.991466
Tanc2	3.071063	3.640966	4.081202	3.38853	3.125179	2.7406	4.647612	2.185176	1.991426
Nat8l	2.097289	2.430443	2.471183	2.305072	2.353254	2.26145	2.369499	2.045331	1.99081
Zfp607a	2.045203	2.886041	2.480641	2.401552	2.507312	2.877251	3.440579	2.423685	1.990734
Gm6576	1.913657	1.21454	1.565901	1.21096	0.773201	1.555182	1.85799	1.821989	1.989693
Thbs2	1.600795	2.34345	3.08025	2.299307	2.077351	1.679479	1.354563	1.836067	1.989655
Gm30074	1.768924	1.446656	1.167625	1.829375	2.425973	1.472261	1.170093	1.381035	1.989411
Tfdp2	2.351869	3.309556	3.471827	1.922215	2.132502	1.817411	2.214335	1.794435	1.988215
Prelid3a	2.166176	1.280242	2.62111	3.377325	1.808346	1.929019	2.377052	2.495264	1.988093
Rpl10-ps6	2.455646	2.597539	2.75362	3.033864	4.181366	3.371949	2.614942	3.542448	1.985835
Gm5898	2.659573	1.960538	2.329462	3.022486	2.155366	2.092858	2.048796	1.918791	1.983764
Prkcq	1.003515	0.829967	1.174983	2.652936	2.564171	3.14621	2.833279	1.980799	1.98286
Gpd1	2.456869	3.517407	2.912917	4.807404	2.566337	2.803399	2.744079	2.829653	1.982819

Serpinb6b	4.893317	4.675727	3.780307	2.942854	1.534843	2.11356	2.422749	2.399203	1.982799
LOC115487312	2.771903	0.691131	0.693056	4.134564	0	0	0	1.319562	1.981402
Gm8696	1.583945	0.888597	1.782145	2.067282	1.037115	1.281685	2.046357	1.413816	1.981402
Gm6493	3.046474	3.077069	2.212346	2.238289	2.328535	1.417614	3.128627	2.364773	1.97886
Hspa12b	2.311703	1.771857	2.055086	4.810355	2.262797	3.464012	1.017648	1.650726	1.978852
Nnmt	1.697084	2.507504	4.023184	2.250103	2.334632	1.220652	1.169347	2.154386	1.976909
Cxxc5	2.48383	2.170758	2.099977	2.979178	2.511897	1.989127	1.085089	2.633028	1.976826
Col12a1	2.117299	2.769273	3.736844	1.806743	1.535046	1.659906	1.627999	1.813591	1.976078
Gm26497	0.518113	0	0.518173	0.515211	0.493445	4.791344	3.748468	0	1.97523
Gm25629	0	2.066934	0	0	0	0	1.606486	0	1.97523
Gm9755	3.074323	2.532897	2.30601	1.59501	1.654929	1.236103	1.795959	1.018107	1.974635
Zfp101	1.644263	2.955844	2.821965	2.099327	2.281302	2.740768	2.45473	2.918401	1.973422
Gm10074	0.920134	1.605947	0.34509	0.91498	0.109541	1.063639	0.356627	1.095072	1.973181
Snora52	4.137169	3.300924	2.896355	8.227989	1.576078	2.295555	2.993179	1.969495	1.971545
Per3	1.474526	2.389725	1.269878	0.997875	1.813916	1.306779	2.49766	1.754866	1.971399
Sox13	1.169436	1.521291	1.305175	3.387537	2.550345	2.256956	1.891833	1.952516	1.970701
Meox2	2.020201	2.366245	2.39633	4.905415	3.288727	3.953671	1.529565	2.124718	1.970208
Xlr4b	5.284391	3.353841	2.210093	4.777082	3.56871	2.932103	2.780492	1.921026	1.968811
Acyp2	2.065502	3.131204	3.800965	2.875504	2.045848	2.826965	1.622447	4.169107	1.968607
Efcab7	2.98293	2.057173	2.475485	2.33511	1.904014	1.584682	2.820623	3.534945	1.965902
A630001O12Rik	1.505572	1.069607	1.196347	0.799841	1.51246	1.182507	1.683982	1.963634	1.965677
Gm31597	1.45273	1.8695	0.937354	0.605797	0.758729	1.47345	0.532779	0.53541	1.965212
E230013L22Rik	1.822048	1.727827	1.34429	1.990056	1.280139	1.491616	2.037537	2.019157	1.964321
Tcf7	0.794119	0.963249	1.781608	1.451286	1.185567	1.627536	0.865127	1.369116	1.963759
Gm7895	2.294312	0.747169	3.184313	2.002098	1.739143	2.035107	1.88735	2.18441	1.963552
Kif18a	1.905942	2.479393	2.221095	1.236043	1.862552	1.440698	2.295346	1.861982	1.958696
Mrc2	1.939998	2.449518	2.867325	3.259566	1.793023	2.147556	1.244535	1.719684	1.95829
Zfp605	1.737925	2.276049	2.633526	1.937668	1.220487	1.590945	2.068389	2.039095	1.95756
Gm23318	4.619839	2.559744	1.540125	0.51044	2.44438	0.474698	1.061074	1.46618	1.956941
Jcad	2.983064	3.133907	2.413546	4.61618	2.17068	2.890377	2.035222	2.209907	1.956645
Klrb1c	1.817641	2.234384	3.128398	2.374918	1.449295	1.641807	0.917467	1.50922	1.953956
Zfp719	2.147722	1.910765	2.074745	1.626037	1.917625	1.760447	2.434188	2.114552	1.953925
Rbp7	0.838559	0.278775	0.372736	4.447263	1.86348	2.326419	0.674094	0.79839	1.953652
LOC118567770	0.30742	1.328607	0.512426	0.713295	1.268728	0.852877	3.389161	0.195129	1.953323
Slc52a3	4.046953	3.053007	3.450688	2.682856	2.520102	3.38264	2.868922	2.519332	1.953278
Kbtbd11	2.516559	2.25642	2.811987	2.396495	2.974459	2.713844	2.846685	2.044804	1.953179
Adat1	1.961044	2.21953	2.710522	1.643309	1.909647	2.221043	2.459533	2.034936	1.953052
Bex1	2.944747	2.362295	2.560947	1.145838	1.707114	2.368009	2.183411	2.37704	1.952421
Dio2	2.238263	1.69793	1.650803	2.758525	1.242804	1.454507	2.179379	1.102549	1.95206
Kifc5b	1.498326	2.715063	3.186951	1.657813	1.426989	2.497992	2.813644	1.74803	1.950981
2610203C22Rik	3.470441	2.218719	2.536386	2.964321	2.245848	1.604677	1.931393	1.440292	1.950658
P4ha2	2.238938	2.718408	2.920694	3.024996	1.668788	2.880696	2.263749	1.181697	1.94835
Adamts1	1.941013	2.286789	1.839068	4.21019	3.189105	3.443018	1.759767	2.831492	1.947324
Zfand4	1.685349	1.581988	1.626055	1.399876	1.472922	1.906941	1.639428	0.868381	1.946442
Fads2	3.318274	2.301392	1.926349	1.934299	2.361135	2.327931	1.261463	1.634132	1.944823
Rasip1	2.550292	1.89447	1.477582	2.8858	2.395368	2.3259	1.363373	2.26067	1.944524
H2bu2	1.559945	2.154174	1.920156	1.431884	2.97135	0.66581	1.860325	1.256725	1.944233
Gm4811	2.311808	1.808356	3.037434	2.524237	2.892483	2.724759	2.389369	1.726328	1.94414
Bsn	2.967225	2.709535	2.678815	3.351859	2.08385	2.039501	3.555734	2.119645	1.94282
Gm26493	0.407633	0.813095	0.40768	0	0	0	3.370471	2.328638	1.942551
Gm22581	0	0.813095	2.038401	0.810699	2.717575	0	2.106544	0	1.942551
Rbx1-ps	1.936256	1.829464	2.548001	2.026747	1.649956	1.88483	1.685235	3.492958	1.942551
Rasl11b	0.564415	0.625458	0.12544	0.43653	1.164675	1.014909	0.939843	0.328398	1.942551
Mpzl3	1.486968	2.385711	2.052902	0.916113	1.385386	1.076167	0.935478	0.938697	1.940966

Sema6d	2.873715	2.587646	3.005434	3.592433	2.674337	2.930866	3.377459	1.313308	1.94071
Gm26064	0.508606	0.507252	0	0	0	0	0.52567	0	1.938987
Zbtb3	1.345584	1.640224	1.614889	1.992203	1.395432	2.544013	1.792494	1.651232	1.937941
Cabyr	2.50759	1.081476	2.440101	2.021792	0.903644	1.316155	3.082044	1.290525	1.937802
Coro2b	2.084266	3.731468	2.238284	2.837071	2.440613	2.575218	2.878143	1.740439	1.937643
Gm46226	0.841033	1.25819	1.962637	0.20908	0.533993	1.037014	4.128933	2.468965	1.937149
Gng5-ps	2.25817	1.57651	2.032589	3.143729	1.828058	1.461799	1.050269	3.224998	1.937013
Prelp	5.448383	3.960284	4.094458	7.422779	5.071715	5.949408	2.751959	4.469365	1.936296
Tnfsf10	1.300731	1.073601	1.670961	2.988304	2.178583	1.451746	1.251655	1.217071	1.934379
Plcb4	2.341439	2.514836	1.981448	3.06861	1.703919	2.498411	1.228612	1.680533	1.934052
Gm41608	2.412735	2.406311	1.535555	5.071078	1.984517	1.8509	4.930689	1.72287	1.933713
Rnu1a1	1.352148	2.697096	0	0.672287	1.609714	2.813454	1.048134	1.287377	1.933076
Snord33	2.028222	0	1.352305	0	1.931656	3.12606	0.698756	1.287377	1.933076
Syde1	1.944221	2.326854	2.694447	2.623805	2.751029	2.285971	1.492734	2.750189	1.93243
Gm41756	0.868935	0.866622	0.695229	1.440113	3.365428	0.803564	2.155411	0.551541	1.932402
Lrfn1	2.05922	1.764931	1.769848	0.415936	1.225737	1.338962	1.496466	1.960579	1.931954
Arhgap33	4.095228	3.93225	3.681777	2.859267	3.900251	3.182808	4.165081	2.675418	1.930797
Mtfp1	2.343013	1.940711	1.826968	1.619075	1.626318	3.19503	1.149244	1.73925	1.930306
Notch3	1.459259	2.093816	2.73987	3.737227	1.711005	2.463432	1.180962	2.411715	1.928755
Cygb	3.115567	3.107272	2.901857	3.925855	2.944581	4.552702	2.703896	3.283337	1.926718
H4c14	2.021179	2.591741	1.443867	0.717806	3.299913	2.670176	2.238203	2.061815	1.926363
Dnaaf3	3.694124	3.292344	2.934681	3.46501	2.545112	3.198154	4.278395	3.392446	1.922722
Ccl11	3.048318	1.545866	1.808535	4.572553	2.361913	2.580092	0.640798	2.262809	1.920465
1700102P08Rik	1.107754	0.803495	0.6043	1.802535	1.438654	1.303803	1.196961	1.102631	1.919615
Gm4518	1.658404	0.649781	1.421654	1.236835	0.733314	1.314548	0.73459	1.578963	1.919307
Klra3	0.869079	1.094861	0.686194	3.866204	1.698965	1.099795	0.992787	1.437146	1.918189
Phka1	2.596552	2.275743	2.518161	2.783202	1.541565	1.725545	2.730136	1.765837	1.917659
Gm14567	1.885648	2.256754	1.257245	2.500115	1.676146	3.952587	2.078839	0.478753	1.917003
Tacstd2	1.697084	1.535846	1.257245	1.250057	1.885664	2.063483	1.364238	1.974854	1.917003
6820431F20Rik	1.999054	1.886925	2.023088	2.401993	2.005871	1.749627	2.226001	2.265828	1.916617
Chpf	3.585903	3.898229	2.886988	2.620875	2.356471	2.453937	1.834574	2.236256	1.913903
Gm14176	1.389812	0.872737	2.265142	0.818979	1.078523	1.856479	1.915254	2.00936	1.913332
Kbtbd3	1.457011	1.310201	2.078273	2.92145	2.274821	1.833345	1.60464	0.977874	1.912254
Slc6a13	0.981929	1.550581	1.06388	1.098482	1.402767	1.475593	0.9303	0.973846	1.910725
Ranbp6	2.335074	2.953672	3.030252	1.948209	2.397471	2.49648	2.660643	2.895607	1.910701
Kynu	2.419367	1.964811	2.21225	1.271645	4.147521	4.666483	3.214975	2.468008	1.910579
Hey1	1.510099	1.969488	1.974975	3.523079	1.742437	3.00783	2.791364	1.686606	1.909785
Gm24091	0	0	0.668006	4.649309	0.636128	0	3.451687	0	1.909785
Mir1898	0	3.330751	0.668006	1.328374	1.908383	1.235359	1.380675	1.9078	1.909785
Vamp1	2.830536	3.730393	2.588219	2.714156	3.177159	1.570554	2.591157	2.348454	1.907696
Gm7160	0.941623	0.352169	1.177166	1.170436	1.120989	0.761936	1.094866	0.336194	1.907082
Pakap	3.659196	4.031857	2.976047	5.371017	2.341842	3.021623	2.076202	3.333128	1.906627
Atp5k-ps2	2.18117	1.450242	3.09035	5.783871	3.981534	2.185167	1.878623	1.730573	1.905611
Bend4	2.948313	3.706935	2.634225	2.132848	1.803204	1.492472	2.35402	1.456756	1.90441
Cdk5r1	1.212125	1.208898	1.345482	0.940426	1.725279	1.601336	1.84477	1.496476	1.904279
Xlr4c	0.95091	1.659663	0.618163	0.851025	0.633946	0.351749	1.818204	0.769556	1.903234
Mrip-ps	2.466524	1.932824	2.231876	1.518343	1.733852	2.280964	1.942305	1.956977	1.903042
Fgf2	1.276272	2.466194	1.435973	2.617566	0.683723	2.434279	3.462613	1.442974	1.900626
Rhebl1	1.507526	2.037017	2.091328	1.644154	2.223099	2.158627	1.005228	1.481613	1.900292
Lrrc29	0.979579	1.190684	0.918462	1.765542	1.603492	1.415444	1.993249	1.457274	1.896428
Plat	1.645702	1.861797	1.793287	2.320388	1.263236	1.885331	0.660057	2.40877	1.896247
Pigw	2.774358	1.297783	2.013485	2.075217	1.753718	1.839087	2.131547	2.290825	1.895407
Zfp119b	1.72422	1.923654	1.987468	1.423958	1.892623	2.216091	1.60084	1.752923	1.894013
Gm9836	1.168347	1.223498	1.811148	1.394163	1.112721	1.134473	1.147168	0.945524	1.893015

Ackr3	1.849749	1.926214	1.985991	2.97548	2.098472	2.490418	1.237052	2.330924	1.892606
Arsi	2.42692	2.6405	3.550535	2.911946	2.521496	2.893528	2.425422	2.692593	1.892512
Gm3200	3.277354	2.209777	2.354429	2.799983	2.769619	3.158856	2.910223	3.340106	1.891764
Prrt1	3.520519	3.430431	3.075754	4.225102	4.239304	2.357551	2.467578	2.46575	1.889805
Jade3	2.312768	1.829381	2.062362	2.050572	1.68183	1.548768	2.461012	1.572844	1.889377
1700010I14Rik	1.550715	2.31988	1.852458	1.542029	1.640982	1.951905	2.136989	2.132625	1.888516
Tspan2	1.758856	1.767772	1.731789	1.382933	1.649145	1.702188	1.099175	1.259198	1.884265
Gm20568	1.317713	1.877435	1.976799	1.216737	0.896411	1.827869	1.361922	2.150729	1.883846
Ccdc166	2.457489	2.979582	2.120433	2.539556	2.065134	2.161207	1.917404	2.683854	1.882949
Oplah	1.767831	2.21768	2.707305	1.73046	3.156867	3.295213	2.369574	2.051337	1.882349
Gm7432	4.295724	2.026352	3.36731	2.770808	1.658595	2.57679	2.279921	2.044975	1.881122
Gcnt7	1.223633	1.444526	1.34865	2.2349	1.189158	0.692802	1.471165	1.069915	1.88025
Fv1	0.843623	2.243671	1.165138	1.518004	1.109536	0.965907	1.079528	1.223941	1.876111
Ldha-ps2	1.83349	1.928351	2.267124	1.690622	2.158933	1.479756	2.859732	1.682183	1.874568
Gm8539	1.152063	1.327288	1.668699	1.481392	1.210717	1.193973	1.108596	1.191435	1.874204
LOC115489839	0	0.392131	0	1.563901	0.374458	0.727197	0.812738	0.374344	1.873667
Pars2	2.612656	1.935663	2.438762	1.583555	2.630451	1.495682	2.365986	1.895241	1.873498
H3c3	0.230992	0.691131	0	0.689094	1.319965	2.136141	1.790563	0.659781	1.871325
Gm6402	1.430923	1.590213	1.839973	1.626181	1.946851	2.797778	2.070503	1.868406	1.87035
Hbegf	3.314613	2.700503	2.26447	2.599698	3.779269	3.194768	2.677928	3.955907	1.868775
Lck	0.551897	0.550428	1.214315	2.030581	2.339012	2.245659	1.540116	1.31365	1.867325
2010001A14Rik	2.394576	2.967159	2.322283	1.803911	2.902541	1.54339	2.849901	2.279871	1.86729
Gm19807	1.788325	2.208222	2.044037	2.455758	1.622077	1.968794	1.320231	1.054028	1.866759
Angel1	2.56311	2.699898	2.08817	1.875768	1.974805	1.651209	2.098446	1.604039	1.866464
LOC115487308	8.478763	4.553333	15.00263	14.91686	6.2116	4.22202	7.415036	10.55649	1.864849
Mrto4-ps1	2.123635	1.2262	2.40334	1.222586	2.075752	1.705467	1.097442	0.85133	1.864223
Gm51749	1.791828	1.299678	2.335077	1.673803	1.344529	1.054471	2.637616	1.499208	1.863023
Xkr8	3.237758	2.956637	3.12883	2.377358	2.862418	1.743445	2.640396	1.74294	1.861939
Gm7774	2.068156	1.145917	1.321475	1.256793	1.422553	0.903156	1.54378	1.093937	1.861629
Slc7a2	2.031912	2.134296	2.550995	1.597795	1.550882	1.499241	2.182001	1.776795	1.861052
Gm15452	3.799797	1.360398	1.656514	1.065734	3.154925	1.982219	2.618187	1.576981	1.857202
Rpl31-ps17	1.68781	0.906401	0.77908	1.420147	0.865551	0.840449	1.073499	2.843084	1.856115
Kansl2-ps	3.056735	2.979311	3.369748	3.108695	2.911201	1.895223	3.015684	2.215805	1.853944
Sfxn4	1.864145	1.374178	2.553365	1.128341	2.199942	1.611475	1.717265	1.389012	1.853944
Snord65	3.89039	3.880033	0.972711	0.96715	1.852583	5.396567	0	8.334074	1.853944
Adgrl4	3.307921	3.016334	2.187533	5.42414	1.607361	3.308782	0.92101	2.159633	1.853041
Gm4900	1.325565	1.43955	0.942733	1.874687	0.981908	1.443768	1.248256	1.654711	1.852959
Nrp	1.184574	1.275934	0.852992	2.921288	1.985589	1.928004	2.546578	2.436114	1.851567
Gm4691	1.524547	1.612639	1.386113	1.010671	1.583958	1.452576	1.671192	1.319562	1.849309
1700120K04Rik	0.408384	1.832833	0.714754	0.50762	0.19447	0.18883	1.266254	1.458079	1.848822
Tmem177	2.022921	2.451055	2.524765	1.778844	2.133599	1.221388	1.797042	2.005606	1.848353
Gm3511	1.231957	0.877627	0.176014	2.975136	1.508532	2.604058	1.273289	1.508071	1.845116
Zfp963	2.226234	2.36511	2.105488	2.237826	1.912826	1.723085	2.551024	1.635773	1.845042
Ccdc74a	2.150846	1.716096	1.559545	3.208197	1.177854	0.795614	2.889909	2.150207	1.844953
Ctrl	0.187291	1.245281	1.810688	1.11745	1.248616	1.385605	1.61312	0.475518	1.844549
Gm33877	1.98638	1.620893	1.599609	1.770032	1.8181	1.145107	3.43948	1.22807	1.844023
LOC118567870	2.681778	3.28849	2.374309	2.010996	2.219132	1.870182	2.908067	0.753437	1.843652
Sgce	1.896565	2.255269	1.677926	3.590543	1.87574	2.698283	1.583234	2.083519	1.842357
E130307A14Rik	1.699123	2.159784	0.93296	2.517844	2.379745	3.235021	2.375939	2.791381	1.841688
Iqsec3	0.910625	1.686659	1.93155	1.044836	0.743374	1.027198	1.023917	1.07661	1.840725
Ncam1	0.680918	0.871518	1.225795	0.609393	1.048406	1.51126	0.645116	0.994061	1.838763
Ggact	3.253973	5.288654	4.821261	3.954801	5.50942	4.680935	2.615778	3.32759	1.837823
Gm5088	2.837246	2.520999	2.493627	1.863803	2.096222	1.733296	1.972729	2.750451	1.835542
Asb2	1.275268	1.727827	1.588254	2.201273	2.818675	3.515732	1.815432	3.253086	1.834632

LOC115487891	0.769973	0.383962	0	0	0	0.356024	0.397903	0	1.834632
Grik5	1.783279	1.669323	2.471849	3.064369	2.368786	1.634654	1.972448	2.368062	1.833804
Gm33989	1.94789	2.426639	2.307715	2.652064	2.403715	1.917564	2.568867	1.847935	1.833223
H60b	3.610462	3.775085	2.446081	2.432097	3.216722	1.88483	2.828788	1.718757	1.831548
Creg2	2.96358	3.022431	2.734459	1.701643	1.556917	2.077571	1.778521	1.738481	1.831405
Mymx	0.48028	1.094861	3.156494	0.545817	1.63362	2.855238	0.85096	1.175847	1.830999
Aoc2	2.684599	2.416237	2.204683	2.192079	1.725309	1.473433	1.962569	1.620879	1.830587
Utp14b	1.665392	1.451468	1.665586	1.357674	1.328896	1.026735	1.628225	0.999939	1.830362
Gm9917	1.388602	1.913494	1.102529	1.581095	1.140773	1.284135	2.651265	2.028542	1.828598
Fosl1	2.27605	1.005281	1.495864	1.228649	1.672214	1.383167	1.209811	1.300213	1.828391
Zfp202	2.936432	2.266365	2.420255	2.523805	1.4475	2.087812	3.522981	1.615647	1.828284
Itk	0.758204	0.711704	0.57987	0.887008	1.911454	2.350958	1.19851	0.849275	1.827842
Gm12263	1.652225	2.000932	2.714686	0.70413	1.657859	1.527926	1.585682	1.376445	1.827797
Tnxb	4.756469	4.599354	3.10568	5.104294	2.985061	3.878941	1.940072	2.410486	1.827691
LOC118568393	3.355041	3.505448	3.515214	3.336248	1.825888	2.807148	4.293222	3.346439	1.82723
Gm16315	2.018753	1.832691	1.371876	1.286824	1.25711	2.273764	2.434229	1.232084	1.825382
Senp8	2.107911	2.522759	2.273797	1.407384	1.806796	1.364531	2.085261	1.505203	1.822474
Mir5110	0	0	0	0.63365	0	0	0	0	1.821979
Snora75	1.911657	5.243062	0.47797	1.425712	5.006764	0.44196	1.975793	3.64017	1.821979
Mir7671	0	0.953284	0.95594	0.950475	0	0	0.987897	1.820085	1.821979
Mir7042	0	0	1.91188	0	0	0	0	0.910043	1.821979
Shisa4	0.931732	2.416054	2.096641	2.13098	2.218429	3.144974	0.818543	1.995976	1.820448
Col8a1	1.996204	1.406607	2.267691	2.902016	1.281219	2.72891	0.874608	2.179472	1.81984
Fam171a1	2.715334	2.877361	2.305473	2.545429	2.316673	2.014076	2.382545	1.885088	1.819655
Gm35339	1.843585	2.440721	2.382254	2.611987	2.377336	2.112252	3.642251	2.392143	1.819298
Gm7308	2.008625	1.853032	1.255537	1.348228	1.673869	1.114508	1.868413	2.008029	1.818679
Mamstr	1.195773	1.877694	2.951612	3.035935	1.066149	1.71754	2.12994	1.816744	1.818635
Ccdc152	1.833269	1.389575	2.346857	2.62512	1.955504	1.763164	2.046357	2.862541	1.817159
Ccdc180	1.537721	1.581553	2.002474	1.8636	1.418743	1.392411	1.092652	1.586067	1.816715
Gm33103	1.737611	2.970831	1.572307	3.949435	1.812489	2.754665	2.993179	3.623871	1.813821
Kif20a	3.719283	4.839707	4.023039	1.857161	1.900324	2.317587	2.887173	1.291825	1.810438
Mtcp1	1.661447	1.318855	2.238127	1.483554	2.228198	1.348317	2.418081	0.839354	1.809721
Tincr	1.466844	1.850187	1.553309	1.716032	1.438094	1.196904	2.184904	1.026896	1.809218
Tshz2	2.210949	1.832586	1.195247	4.100026	2.376008	2.818256	1.775605	1.635673	1.808232
Gm14057	1.635111	1.501333	1.323815	1.4711	1.137049	1.224081	1.609495	1.334388	1.805773
Jam2	3.482391	4.005399	2.628777	5.492851	3.125982	4.515972	1.641026	2.972587	1.805755
Epha1	1.618429	2.106564	1.344276	1.74575	1.724249	1.471305	1.559322	1.253616	1.803949
Klk8	5.189687	3.653555	3.358423	3.278509	2.674819	3.444175	2.713452	2.964654	1.80392
Lilra5	0.73602	2.385696	2.129448	2.064995	3.479851	1.969018	1.521426	2.652888	1.803837
Thbs3	3.454031	3.5487	3.384996	3.331125	2.033259	1.829832	1.811866	2.164841	1.803155
Gm5960	1.839177	0.733712	2.049607	0.940564	1.15106	0.874704	0.162933	1.250769	1.802982
Sdc2	1.204812	1.785717	2.326226	2.562523	1.529932	1.795055	0.760976	2.373857	1.802181
Gm11772	1.378172	1.374502	2.089729	2.807216	2.667452	2.220079	1.401435	1.798921	1.800793
Klhdc8b	1.164122	1.960838	1.914556	2.64962	2.04493	1.794239	2.058771	1.40392	1.799874
Rpsa-ps4	1.563914	1.398397	0.86295	1.179772	1.181292	0.99742	1.003272	2.207827	1.798939
Fxyd6	2.165549	2.252346	1.268541	2.122656	1.856201	2.746467	1.470819	2.650905	1.798594
Shf	2.099387	2.164775	1.814936	1.592258	3.185538	2.040165	2.390482	3.89601	1.797421
Gm3724	1.17828	0.940114	0.589208	1.17168	1.346617	0.762745	0.852468	0.673103	1.796808
C130074G19Rik	1.807129	1.298444	0.913387	3.709949	2.831471	3.234535	0.923837	1.295048	1.796435
Gm6978	0.389951	0.648188	0.649994	0.258511	0.742771	1.562664	0.335862	0.680665	1.796348
Trp53rkb	1.362756	1.677221	1.507905	2.681412	1.380717	1.474742	1.708152	1.490718	1.79625
Atp1b1	1.134447	1.408946	1.434279	1.085523	1.182363	1.425194	0.840666	0.957829	1.795248
Mutyh	2.208832	2.744661	2.100445	2.376497	2.345072	2.477979	2.282938	1.999597	1.794608
Klhl12	1.724817	2.328386	2.178053	1.247386	0.978981	1.192265	2.196844	1.625607	1.79335

AB010352	1.701564	2.06655	2.731054	2.292432	1.542137	1.268995	2.297593	1.489406	1.791763
Ly75	0.96821	0.523051	0.847282	1.219529	0.79148	1.193826	0.508686	0.645281	1.79175
Snord69	6.577397	4.685633	5.638424	8.409283	6.264241	5.213632	4.855763	10.73542	1.791098
Prss34	0.17618	0.05857	0.469869	0.759171	0.671169	1.955112	0.242788	0.223655	1.791098
Snord110	0	0	0	0	0	0	0	0	1.791098
Klhl41	0.961101	1.80816	1.398128	1.759389	1.726668	2.646189	1.467443	1.559766	1.790393
Ecm2	1.96988	1.707362	0.914694	3.311383	1.920761	2.168671	1.017985	1.339657	1.788068
4932438H23Rik	2.455299	3.157614	1.098551	2.441545	1.230737	2.44984	3.606168	1.907059	1.785879
Pde8b	1.686055	1.408491	2.118623	2.550741	1.893997	2.105601	2.561803	1.824816	1.785511
Kif19a	2.309246	3.543229	3.843808	3.067101	3.38354	2.314723	3.004265	2.72138	1.785359
Gm52691	3.108676	1.311708	2.750303	2.02121	1.897865	2.174533	2.595093	1.517828	1.785304
Zwilch	3.863141	5.362222	5.496654	2.613805	2.958542	2.633346	4.774834	2.42678	1.784021
Mns1	2.055534	3.151587	2.638753	2.410113	1.197976	2.808783	1.648863	1.19761	1.783664
Gm12774	0	0.219407	0.330027	0.21876	1.15235	0.406884	1.818984	0.104727	1.782214
Scml4	1.353286	1.628928	1.213432	0.742458	0.799979	0.819933	1.109305	0.622016	1.779037
Gm12397	2.463914	1.793204	1.8315	2.350783	1.712387	1.939847	1.583008	2.219083	1.777114
Gm20958	1.491607	1.859545	1.24315	0.247209	0.828678	0.919594	0.256942	0.236693	1.777043
Tmem79	1.480542	2.050834	1.919444	1.690359	1.906181	1.394513	1.331853	1.383517	1.776925
Cldn1	0.752398	0.886831	0.632772	1.275313	1.058578	1.359961	0.759967	1.074535	1.776458
Dennd6b	1.912926	1.834455	1.876357	1.975372	1.588279	2.06385	2.395338	1.727893	1.77644
Gm16892	1.533635	1.404922	1.158881	1.818756	1.557992	0.703876	2.547883	0.919367	1.775683
Aqp7	2.483625	1.592366	2.129069	10.40808	3.0412	6.070058	1.283475	2.026847	1.775337
Fan1	1.668163	1.873706	2.283867	1.835974	1.650438	1.587596	2.527607	1.834973	1.775139
Gm51557	1.269574	2.363562	2.539443	3.02991	2.901908	3.287374	2.274425	1.611678	1.774691
Spaca6	3.823315	5.368494	2.867819	2.451224	2.587227	1.907407	2.443744	2.634334	1.774032
Rpl7a-ps10	0.962466	1.791821	1.090922	1.02088	1.22219	0.830722	0.92844	0.916362	1.773477
Zfp784	2.376655	2.782559	2.557785	3.031243	3.444457	2.006738	2.80349	2.951489	1.772737
Smco4	2.905559	2.144389	3.08025	1.906927	3.099289	3.493061	2.162189	2.379084	1.772324
Ushbp1	2.240403	2.139356	1.930785	4.526809	3.155205	3.107782	1.305587	1.361543	1.771847
Lmcd1	1.445313	1.853312	2.753296	2.634897	2.556357	2.57769	1.63607	1.605426	1.771086
Rpgr	1.680611	1.94079	1.636575	1.209419	1.389991	1.083834	0.891353	1.30535	1.77038
Wdr60	2.413112	3.198361	3.334292	2.431168	1.905104	2.495835	3.51139	1.738254	1.770325
Gm12013	0.795762	1.587286	0.530569	0.263768	0	2.452985	0	1.010191	1.769674
Gm34189	3.015459	0.751858	2.261857	1.037966	1.380717	1.76969	4.195461	2.594954	1.768616
Tmem158	1.983358	2.871404	1.823622	1.113366	1.736596	2.366642	1.686208	1.126919	1.768361
Nynrin	2.273462	2.110779	2.385916	2.015319	2.499974	2.289155	2.890794	1.801425	1.767662
Tmem170	1.768996	1.565093	2.168699	1.645598	2.119553	1.134584	1.503962	1.439769	1.767592
Gm32342	1.390919	0.990869	1.523565	3.885931	3.027877	1.837541	3.080538	3.720628	1.767559
Eif5a2	2.834776	3.168256	3.08883	2.281442	2.22708	1.530065	2.770278	2.425935	1.766153
Gm19366	0.772118	0.61605	1.389974	2.456937	2.500212	1.428061	1.915254	2.940528	1.766153
Med18	1.71901	1.747403	2.149012	2.301089	2.014974	2.231675	1.435013	2.045832	1.764397
Gm15519	1.523582	1.519526	1.361225	1.313041	1.721904	1.089596	1.280754	1.392575	1.761892
Mir6954	0.923968	0	0.924075	0.918792	1.759953	0	5.7298	7.037663	1.761247
Gm4799	1.256596	0.589765	0.961038	1.617074	1.478361	0.751922	0.993165	1.548286	1.761247
Mir7072	0.923968	0	0.924075	0.918792	0	0	0.954967	0	1.761247
Rpl7-ps7	2.54595	1.460024	2.546246	2.531689	1.636696	2.589856	1.973525	1.878595	1.759225
Hpn	1.431275	1.681236	2.067638	3.131168	2.69597	2.323651	2.465491	2.937406	1.758215
Gm36486	2.561833	1.328607	0.819882	0.611396	2.342267	1.800518	1.482758	3.805021	1.757991
Ftx	1.90763	2.316712	1.713173	1.535622	1.507825	1.560103	2.427654	2.137492	1.756299
Gm39908	1.42681	2.018089	0.882131	2.502279	1.284758	1.559373	1.984114	1.185568	1.755477
Ube2e2	0.880605	1.676679	2.922346	2.18918	1.067409	0.851372	0.951519	1.371963	1.754888
Wtip	1.387367	1.976677	2.095451	2.280556	2.696558	2.173234	1.931393	1.455696	1.754051
Gal3st4	1.873586	1.401448	1.840343	1.796552	2.867758	2.75365	2.835508	1.337878	1.753806
Slc39a5	1.463297	0.833944	1.714347	1.579823	1.5529	1.043906	0.993857	1.154368	1.753277

Myl3	0.306288	0.54985	0.306323	2.558404	1.925253	0.453192	0.69644	0.991494	1.751516
BC055324	2.310708	2.9657	2.557229	1.356058	1.930116	1.611411	2.779746	1.514769	1.751017
Large1	2.613242	2.994125	3.438057	1.840678	1.955504	1.985101	2.861685	1.747568	1.749386
Apoc2	3.187206	0.674274	3.090983	1.536656	1.839673	2.67948	1.796802	2.114977	1.748973
Lrrc70	1.559052	2.28662	2.155411	0.683964	1.615838	1.908215	1.374394	1.70266	1.748136
Zfp955b	1.901615	2.460392	2.809919	1.959105	2.088449	1.029784	1.98312	1.239638	1.747096
Apoo-ps	1.282864	3.564179	1.832876	2.460237	0.872704	1.779529	2.367686	2.879044	1.746691
Mmrn2	2.817691	1.826623	1.916252	5.057443	3.421511	3.361369	0.990156	2.964403	1.745581
Tmod4	1.740809	3.071693	1.919576	3.417729	2.253081	3.962696	2.168282	1.104947	1.74423
Rps15a-ps4	1.431686	2.141812	1.431852	1.660944	0.833263	1.618196	2.219578	1.59029	1.743558
Snai2	0.764286	0.598912	1.173862	0.678576	1.221829	1.186394	0.507811	1.949131	1.743036
Gm10575	0.313505	0.521117	0.99288	0.831329	0.64692	0.531519	2.592181	0.497479	1.742987
Cpt1c	1.306269	1.433071	0.99288	0.80535	1.268958	1.159677	0.972068	0.746218	1.742987
4933432I03Rik	0.627009	0.729563	0.470312	1.143078	0.199052	0.338239	0.270019	0.895461	1.742987
Zrsr1	1.71547	2.658669	2.752477	3.141729	2.5506	2.031521	2.321513	2.16206	1.740858
Elovl6	1.863151	1.8489	2.254675	0.972675	1.526023	1.309468	1.559784	1.614251	1.740234
Gm40462	1.678831	1.718231	0.945827	0.597786	0.921637	0.664401	1.204758	1.130754	1.739819
Amotl2	2.090006	1.517157	1.891807	1.657377	1.524369	2.165194	1.394511	1.863948	1.739814
Gm6436	0.912561	1.59273	2.281667	1.02088	0.869113	1.898792	0.471589	0.434424	1.739503
Gm39607	0.22814	0.455066	1.369	2.04176	0.434556	0.210977	0.235794	0	1.739503
Gm11405	2.674643	2.728148	3.100515	2.478321	3.213073	3.11989	4.240806	3.009527	1.738072
Pou3f1	0.939316	0.771495	0.957845	1.135517	0.578855	1.124135	0.837579	0.946928	1.737841
LOC118567981	1.66207	2.67362	2.627448	0.266574	2.757374	2.132009	1.55159	2.705484	1.7374
Caprin2	1.886081	2.599667	2.437354	2.275907	2.058661	1.195455	2.759766	2.421214	1.737009
Wnt6	0.851989	0.849721	1.821706	2.132649	1.175168	1.71163	1.457501	0.811177	1.736046
Gm32581	1.024523	1.078562	1.536963	0.113198	0.975744	0.473723	2.000136	0.75868	1.735931
Hmgb1-rs16	2.547482	2.419714	1.698519	2.050696	2.079595	1.682737	3.5106	3.464932	1.734269
Mir6999	2.726462	4.532006	3.635705	7.229839	2.596653	5.883142	4.696558	6.922291	1.732374
Lppos	1.493063	1.812802	0.58431	1.161938	0.989201	2.341251	1.341874	2.225022	1.732374
Gm24233	0.908821	0	0.454463	0	0	0	0.469656	0.432643	1.732374
Mir7683	0	0.906401	0	5.422379	0.865551	1.680898	0	0	1.732374
Lama4	3.181774	2.249662	2.439338	4.905493	2.602379	3.552933	1.506845	2.217457	1.730368
4833447I15Rik	1.209558	1.60845	2.016164	1.804174	1.727954	1.677842	1.458495	0.575809	1.729224
Gm30927	0.798192	1.375025	0.798285	1.876068	0.691081	0.067104	2.774903	0.829044	1.728972
Snn	1.641761	1.485075	1.374657	2.315964	2.054491	2.206757	1.519265	1.563117	1.728496
Fgf11	2.784856	2.411422	2.353369	2.211112	1.624256	2.33578	1.918858	1.479882	1.728326
Gm3571	2.57408	1.949191	1.382537	1.23243	1.31656	1.190214	0.443407	2.223853	1.726415
H2bc3	0.678833	0.789864	1.810433	2.475113	0.96977	0.523137	1.637086	0.215439	1.725303
Gm7589	1.428816	2.470021	1.810044	1.704975	3.447332	2.114119	1.673653	1.632447	1.724932
Zgrf1	2.075435	2.056641	2.355094	1.971203	1.153029	1.341047	3.052593	1.114677	1.724475
Vkorc1l1	1.611774	1.642177	1.472799	1.291421	0.883474	1.018699	1.450127	1.468326	1.72404
Gm4832	2.309919	1.402295	0.200886	0	0.860847	0.185751	0.622804	1.338686	1.722959
Zfp956	2.309083	2.723471	1.867562	2.775344	2.447744	2.618146	2.511068	1.81613	1.722335
Gm31620	1.654868	1.800504	1.5046	0.972399	0.931319	0.417374	0.388725	0.931034	1.720621
Nnat	1.567657	1.705619	1.045226	2.078501	2.352637	1.142204	1.718449	1.990085	1.720498
Igsf5	0	0.039102	0.039211	0.155948	0.410739	0.761397	0.20261	0.074657	1.718897
Hsf4	0.95231	1.492504	1.564692	1.420464	0.90697	0.975024	0.98426	1.230512	1.718026
Hspb9	2.14212	2.30733	1.713895	3.493398	2.284947	1.505534	0.619917	0.489482	1.71497
Fxyd7	2.534779	2.60958	1.553755	0.162618	2.024725	2.646546	1.267655	0.700652	1.714488
Gcsam	2.218867	1.844133	2.034198	1.805268	1.905104	2.378384	0.799184	0.720197	1.714252
Gm14117	4.655105	3.376517	7.406709	2.998348	2.418257	3.27759	2.952378	4.432116	1.71419
Oaz2-ps	1.031561	1.659378	1.131521	1.687577	1.394441	1.323228	1.61645	1.394015	1.712617
Zfp69	1.770807	2.708009	1.676559	1.666974	1.978823	1.091725	2.098649	1.483664	1.710239
Mycl	1.727199	1.187424	2.129901	1.434049	2.794844	2.496688	1.698489	1.548669	1.710103

9030025P20Rik	0.985964	1.251522	0.851613	1.247834	0.853656	1.699242	2.177046	1.152083	1.708566
St18	2.652299	3.523657	3.283228	2.219433	1.973156	1.416124	2.33785	1.88679	1.707509
Fam149a	1.807185	2.12044	1.701078	1.568025	0.944941	2.162766	0.897284	0.809702	1.705522
Gm25965	2.384433	1.189042	2.38471	3.556615	2.838635	1.653787	7.393291	0	1.704432
Mir6948	3.576649	1.783564	8.048396	2.667461	2.554771	2.48068	4.620807	2.55399	1.704432
Gm10051	2.123635	1.337673	3.465282	2.556317	3.193464	2.997488	2.541444	2.447574	1.704432
Mrvi1	1.262347	1.39013	2.077854	2.458248	1.653087	1.434903	1.984229	1.627543	1.704432
Ust	3.661491	4.063207	2.819504	1.709381	3.388934	3.004507	1.776682	1.636666	1.703904
Fam83d	2.625279	3.481768	3.100423	2.16622	1.516131	1.317197	3.059742	0.664767	1.703574
Maoa	2.038458	1.993168	1.918772	1.82831	1.738382	1.466191	1.6662	2.143772	1.701565
Gm21092	1.783659	1.105809	2.748119	1.869542	0.734589	1.069928	2.391569	2.386686	1.699986
Armc2	1.422461	1.702409	1.403658	2.828987	2.095326	1.490841	2.528718	1.318207	1.699184
Cenpp	1.960832	3.318613	2.495894	1.59533	1.301573	1.373724	2.210855	0.905166	1.698952
Bicdl1	2.189014	1.878556	1.858333	1.366798	1.35754	1.8831	1.446919	1.429828	1.698172
Gm12734	1.278637	1.320777	0.867748	1.453114	1.696166	1.351364	2.312687	1.217388	1.697412
Jph2	2.10456	1.901619	2.356661	2.45051	2.00436	2.844491	1.561659	1.900991	1.697243
Gm12034	2.288727	0.887691	2.034661	0.758636	1.210977	1.058272	0.657087	1.573789	1.696614
Ube2v2	2.334642	2.032549	2.283313	1.859816	2.014651	2.003937	1.999698	1.817544	1.696501
Gm7890	1.414469	2.593874	1.551534	2.132505	1.781681	0.632931	1.886354	1.346713	1.696015
Ttbk2	2.317968	2.656538	2.669023	1.723683	1.63634	1.640594	2.695202	1.814912	1.69568
Fcna	2.452637	4.090904	4.229177	1.934299	3.544071	2.776495	4.632791	2.93907	1.692731
Zcwpw1	2.193498	1.927223	2.010941	2.778448	1.765756	1.690399	1.916231	1.168524	1.69239
Sft2d3	1.854512	1.987309	1.775803	0.94168	2.217166	1.550793	1.488525	2.066218	1.692301
Cd300ld5	3.550221	2.042751	3.004382	3.734007	6.502291	2.841173	13.83055	9.230432	1.691838
Gm16861	2.715777	1.300103	2.245303	2.412504	2.758908	2.377521	4.116774	2.068549	1.691073
Gm13461	2.026986	2.072129	1.343034	2.191999	1.351335	1.335576	1.70218	1.616282	1.69041
Lrp11	1.882493	2.965877	3.21971	2.468802	1.247211	1.942711	0.817737	1.922196	1.690173
Zfp85	1.115552	2.055857	1.576506	1.905107	2.540615	1.457734	1.403626	2.170408	1.687284
Pdcd1lg2	0.811685	0.825714	0.925428	1.000851	0.881265	0.690571	0.855695	1.190117	1.686465
LOC118567692	2.285346	1.249918	0.884753	3.738702	1.54464	1.090795	4.266873	1.895115	1.6863
D030028A08Rik	2.280431	1.941048	1.592555	2.521791	1.198266	1.436214	2.092799	1.684547	1.6863
Atg9b	1.737244	2.345935	1.829699	1.482909	1.259201	1.052076	0.762702	1.683301	1.685053
Smyd4	1.716813	2.377191	2.333804	2.204438	1.666823	1.834281	2.463504	1.475878	1.683934
Fancm	2.104037	1.908333	2.302245	1.232022	1.235831	0.942365	2.303437	1.046994	1.683921
Zfp97	1.62522	1.200662	1.785943	2.015157	1.528733	1.298848	1.161306	1.948538	1.682842
Gm8369	0.738627	0.777586	1.02599	1.224149	1.446002	0.83485	1.738874	0.97673	1.681723
Grrp1	3.572273	2.435306	1.944628	5.890461	2.971536	4.808931	1.028186	2.453997	1.680798
Slc36a3os	0.489736	0.879177	1.371419	1.460977	2.425378	0.452892	0.809866	4.009976	1.680341
Tnni3	1.094172	2.910025	2.978926	1.450724	2.199942	1.068071	2.32459	2.257145	1.680137
E230032D23Rik	2.818885	3.279943	0.234934	0.934365	0.335584	0.651704	0.849759	0.670964	1.679155
Gm52503	0.63398	1.826623	1.62036	1.330906	1.341769	1.17257	1.16489	2.61565	1.678443
Rpl38-ps1	0.703975	1.755253	3.520286	1.225056	0.502844	1.79029	0.909492	1.508071	1.677378
Mir7045	0.879969	0	1.760143	0	0	0	0	1.675634	1.677378
Mir7211	0	0	0	0.87504	2.514219	0	0	3.351268	1.677378
Mir7679	2.639908	0.877627	0	0	0	0	0	0	1.677378
Chrnb1	1.107058	1.840185	2.441489	1.55249	1.216558	1.391281	1.378907	0.729712	1.677378
Tle6	1.882919	2.124188	2.037493	2.547653	2.028454	2.797441	2.488443	2.762557	1.676911
Stac3	0.949764	0.841987	1.37204	2.30864	1.708584	1.984334	1.854187	1.507114	1.676313
Shank3	2.065781	1.693177	1.269664	5.049622	2.28937	2.60505	1.428568	2.245758	1.675332
Tjp1	1.764318	1.901392	1.965228	3.683483	1.648463	2.644562	1.140775	2.157473	1.673583
Tnfrsf8	1.274629	1.898598	1.67211	1.333332	1.592314	1.393052	1.437155	1.828237	1.672369
LOC118568603	0.526311	0.34994	1.754573	0.872271	1.002505	0.811193	0.725291	1.336265	1.67207
Cyp2s1	3.550741	3.646684	3.360914	1.324069	1.288262	1.934987	1.660179	1.106762	1.671942
Cfap157	1.721062	1.947545	1.324048	0.559503	1.040211	1.132474	1.505142	1.039893	1.67187

Cacna2d1	1.035481	1.429926	1.566679	1.768939	1.112614	1.620521	0.548831	1.263948	1.670148
Prss16	0.772835	1.233245	0.901746	0.589188	0.637901	1.524681	0.665637	1.12825	1.669583
Gm11488	1.611572	0.918446	0.921005	3.250876	1.97337	0.936779	1.427691	1.709731	1.667626
Prr15	0.743138	2.334651	1.300642	0.628129	2.194044	2.886367	2.765051	1.06131	1.664449
Rnf227	1.492319	2.090018	1.937065	1.610254	1.542227	1.732403	1.476753	1.995214	1.664408
LOC118567909	1.164054	1.354447	2.037331	2.411528	1.662948	1.345601	1.303367	1.523903	1.66417
Gm15454	1.412964	2.403934	1.662504	1.405049	1.741483	2.152154	1.803985	1.899219	1.663546
Gm17709	0.705508	1.29616	1.00268	0.886176	0.848738	0.721109	0.460533	0.671713	1.663334
Gm6063	1.516773	2.874197	2.123729	2.262416	2.094609	2.805329	1.01898	1.227499	1.662463
Gm4342	0.872059	0.248497	0.498378	0.61941	2.372971	0.46083	0.386279	0.948898	1.6623
Ift80	2.783854	2.846555	2.868547	1.691715	2.30316	1.456238	2.39771	1.619751	1.661638
Scrg1	0.475183	0.473918	0.475239	0.393768	0.452559	1.025348	0.409271	0.226211	1.660604
Bcar1	1.937399	1.684064	3.021983	2.350741	1.658949	2.1697	1.285944	1.709211	1.660168
Gm25939	0.870755	0.578958	1.451427	0.288626	1.10573	0	1.199958	1.38174	1.659814
Srl	1.666294	3.53332	2.537266	3.254211	2.287511	2.915286	1.675652	1.772279	1.659664
Npff	1.740208	4.338938	3.978081	4.326151	7.339716	7.701603	6.166601	5.562278	1.658573
Gprc5a	1.926419	1.97466	1.177393	1.356903	1.29958	1.08869	2.239932	1.324657	1.657544
Gm30181	1.179533	0.928731	0.807143	1.234659	1.06425	1.435257	2.053232	1.773207	1.656716
Fam229b	1.189265	2.007245	1.097911	1.546482	2.265287	0.676797	1.701921	0.435499	1.656618
Mymk	0.640374	2.235341	2.058583	2.274238	2.30885	1.522794	1.560094	1.916195	1.656618
Clcnkb	1.73749	2.45825	2.060984	0.602706	0.269381	0.074734	0.334099	1.423434	1.655983
Gm15774	0	3.197521	0.668006	2.523911	0.381677	1.235359	4.280092	0.38156	1.655147
Hacl1	1.778761	2.682158	3.303798	2.863768	2.762952	2.976563	2.101073	1.794362	1.654953
Gm46370	0.347211	1.500576	1.736258	1.265977	1.212494	1.17733	1.07658	0.881544	1.654616
Sgo1	1.827966	2.926151	2.396604	1.283101	1.887232	1.818295	2.381463	1.360147	1.654371
Spon1	1.123866	0.591821	1.195932	1.198036	1.258741	0.9728	0.492506	1.463802	1.653847
Mir6998	1.732439	0.863914	0	0	0.824978	0	0.895281	2.474178	1.651169
Neurl1b	2.244742	2.344535	2.58087	1.942162	2.466123	2.198458	1.872484	2.2466	1.650905
Nexn	1.067759	1.235303	1.430964	2.909272	1.098276	2.113101	1.236012	1.626578	1.648625
LOC118567915	1.033229	1.332515	0.748287	1.204586	1.357291	1.515617	2.319906	2.187962	1.646924
Zfp626	1.997393	2.479719	2.351371	3.237927	2.437316	2.097258	2.182679	2.08	1.645901
Gm41610	0.523873	1.137158	0.092459	0.367721	1.408745	0.398968	1.019197	2.229832	1.644744
Pabpc1l	0.862849	1.5367	1.571801	1.256381	1.262001	1.082913	0.828098	1.466995	1.644744
Pbx4	1.489849	0.078204	1.019489	1.091634	0.821478	0.217542	0.405219	0.74657	1.644162
Gm16675	1.13482	1.886331	1.59734	0.668719	1.040761	0.971709	0.825369	1.800767	1.642406
Akap17b	1.660798	1.625891	1.5489	2.229012	1.397353	1.573544	2.28518	1.173806	1.641154
Gm34758	0.918229	1.946041	3.214174	1.369628	2.842161	0.743006	1.186294	1.092805	1.640913
Gm9197	2.458451	1.225953	0.614684	0.977872	0.70242	2.046148	0.889326	1.287377	1.640185
A930037H05Rik	1.739608	1.105809	2.007473	2.129063	1.438307	0.88392	2.529015	0.910043	1.639781
Gm6123	2.967592	2.359174	2.322733	1.881777	2.867263	3.340931	3.200509	2.743542	1.63964
Niban3	1.123899	2.058809	1.49106	3.170346	1.76942	2.524129	2.607687	1.681527	1.639555
Gm9920	0.059292	2.365368	1.363876	1.532958	1.581135	0.548314	1.041782	1.016133	1.638807
Actn2	1.895695	2.264381	1.565233	2.476903	2.015374	2.955774	0.9113	0.818496	1.638695
Car2	0.908289	0.938223	1.265264	1.322544	1.575617	2.15989	0.771126	0.463275	1.638609
Gm22728	0.429752	1.714433	1.289407	1.282035	0.818583	1.192265	1.776682	2.864165	1.638369
Snhg7	1.942574	2.682557	2.764753	1.857396	1.209672	1.243683	2.625515	1.636114	1.637817
Zfp59	1.733891	1.420476	1.997303	1.493262	1.238504	1.274168	2.240078	1.695053	1.637797
Kctd7	1.919129	2.500574	2.383711	1.431284	1.429778	1.273813	2.33543	2.195586	1.63734
Kit	1.6097	1.926497	2.092853	0.949739	1.625044	2.222976	1.319873	0.643689	1.636466
Gm38948	2.448678	2.979434	3.183651	3.993337	2.378736	1.856858	5.415977	2.564519	1.633665
C1qtnf1	0.901432	1.695318	1.545491	2.228151	2.685922	2.357942	1.47737	1.679721	1.632375
Lmln	2.777972	2.918519	2.050004	2.065103	1.798055	1.783322	2.815421	1.450844	1.632291
Derl3	3.079892	4.038707	3.536584	3.346218	3.150534	2.7427	2.829531	1.737694	1.630784
LOC100861738	1.574167	1.706496	2.429975	3.436963	2.216238	1.930439	3.218592	1.857161	1.630784

Plcd1	3.651359	3.433545	3.067498	3.361181	2.146123	2.180359	2.566226	2.006409	1.630661
Gm15190	1.866078	1.163194	1.399721	0.773177	1.481027	2.013305	1.848323	0.962373	1.630326
Gm8910	2.827802	2.525128	1.775803	2.027228	1.691058	1.915685	2.514859	2.066218	1.629623
Ston1	1.486773	1.112111	0.985102	1.607809	1.06199	1.168683	1.037242	1.026277	1.629581
Gm6565	1.108761	1.494337	1.31868	1.817718	1.198671	1.884423	1.331791	1.683333	1.627963
Car15	1.956638	2.551868	1.455105	1.546564	3.392489	1.020708	2.125989	1.71961	1.625766
Tspyl4	1.435704	1.942255	2.047182	1.144956	1.814106	1.446003	1.954009	1.204523	1.625766
Rccd1	2.274382	2.055671	2.890696	2.544347	1.714826	1.402185	1.909933	2.323331	1.625766
Mir146	0	0	0	0	0	0	0	0	1.625766
Galnt15	3.054895	3.070381	1.894729	5.4633	1.28557	2.759372	0.7098	1.578288	1.625072
Mybpc1	1.161243	2.075649	1.613863	1.799593	1.22086	1.924619	0.52996	0.918955	1.624218
Nol3	1.490271	1.019179	1.490444	1.016176	0.324415	1.1419	0.044008	0.810791	1.623269
Prkcg	2.674643	3.450991	2.450483	2.67826	2.155409	2.542613	3.208998	2.315021	1.622201
Napepld	1.134518	1.483179	1.671308	1.234881	1.124307	1.105878	2.012402	1.226141	1.621944
Mbnl3	1.319954	0.962769	1.044264	1.057884	1.144533	1.542515	0.990939	0.800303	1.621048
Gm34226	3.514478	2.600574	1.700752	1.014617	1.511616	1.362937	0.468695	2.050853	1.620779
Ttc21b	1.863888	2.86375	2.607227	1.465225	1.2474	1.77025	1.82229	1.510811	1.620411
Insl3	0.566658	0.470958	1.133448	0.93914	1.529091	1.397407	1.952232	0.89919	1.620227
Clec4b2	0.31861	0.264801	0.47797	0.475237	0.050573	1.276774	0.274416	0.556137	1.619537
Mir652	0	0.564188	0	0	0	0	1.169347	0	1.617471
Gdf9	1.414236	0.48359	1.091109	0.96433	2.424426	2.428848	0.87701	1.885088	1.617471
Tmem53	0.816189	0.939249	1.75815	0.624321	0.896919	0.638665	0.908462	0.597763	1.61564
Xrcc5	1.388121	1.362793	1.475049	1.207802	0.908896	1.484267	0.807014	1.796586	1.612409
Cxcr1	2.156896	2.034875	2.098846	1.449199	0.333114	1.02427	0.301251	1.110041	1.611235
Gm46353	0.620946	1.312901	1.341399	0.790359	0.662348	0.826893	1.155202	0.874978	1.609742
Gm5835	1.527662	1.283028	1.769078	1.639034	0.842327	1.375557	1.204962	1.301381	1.609261
Fcrla	1.543936	2.076974	2.765043	2.570714	2.393719	3.486448	1.521514	1.948576	1.608392
Gm10095	0.96414	1.44236	1.325847	1.198424	1.147796	0.780156	1.992974	0.688467	1.608095
E330020D12Rik	1.470766	1.251712	0.86295	1.63803	1.326035	1.686546	2.107886	1.10158	1.607363
Gm34458	0.06243	0.871697	0.312188	0.496644	1.248616	3.059878	2.258367	1.129355	1.606543
Zfp82	2.060787	1.852665	1.335313	1.183366	1.10573	1.986276	2.189924	1.879166	1.604486
Gm39852	0.224446	0.223848	0	0	0.10688	1.037801	0	1.602707	1.604374
Asb5	0.656557	0.900363	2.298218	1.428174	0.70346	1.631752	0.296881	1.758113	1.603504
Gm14461	1.027784	1.335669	1.931213	1.269791	1.66108	2.102539	2.382052	1.186123	1.602932
Mrgpre	3.264554	4.084628	5.026019	3.285616	2.713419	2.250495	3.067345	1.940255	1.602848
Depdc1b	1.851997	2.721992	2.266523	1.647768	1.392491	1.93802	1.863759	0.928043	1.602541
Zfp113	1.332417	1.661087	1.871946	1.246087	1.057483	1.833597	1.901736	1.19308	1.602508
Zfp518b	2.730519	2.510495	2.483354	2.800074	1.739095	2.059533	3.113159	1.53546	1.602119
Chek1	2.382678	3.027385	2.252382	1.736428	1.196789	1.856311	1.737325	0.870126	1.602076
Gm24252	0.839971	1.256602	3.360273	0	0.399989	0.388389	4.340758	0	1.601133
Gm23639	0.419985	0.837734	1.260102	0	1.199968	0.776779	3.038531	1.199602	1.601133
Prkag2os1	1.287955	0.837734	0.728059	1.169372	1.65329	0.362497	1.504796	0.746419	1.601133
Hmgb1-ps3	2.003007	2.126557	1.615516	1.285024	0.492295	1.852318	1.46918	1.660987	1.601133
Snord23	0	0	2.240182	1.113687	0	2.071409	1.157535	1.066313	1.601133
Bag2	0.885787	1.553617	0.763698	0.728959	0.581803	1.497064	1.010213	0.639788	1.601133
Mir6913	0	0	0	0	0	0	0	0	1.601133
Dync2li1	2.127512	2.570701	3.273476	1.627381	0.701384	2.875514	1.987458	2.72677	1.59877
Gm41005	2.235406	2.251749	0.983693	1.133671	1.554152	0.310085	1.363138	0.85133	1.597905
Zfp455	1.287831	1.936797	1.799084	2.723853	2.414095	1.682447	1.098634	2.179807	1.597591
Ehhadh	2.15201	1.367542	2.114167	1.893766	1.451009	1.32087	1.535295	0.743415	1.597283
Zbtb16	1.347706	1.256932	0.961718	1.876219	1.602691	1.933474	2.296438	1.636881	1.596925
1700012D01Rik	1.116738	1.272874	1.037091	0.475921	0.911631	1.327788	1.071761	1.594866	1.596526
Shpk	1.554897	1.928506	1.455393	1.922823	1.974489	1.769748	2.410596	2.087764	1.595952
Eef1a2	1.722887	2.688309	2.174703	2.113913	1.554846	2.589067	1.005228	1.203811	1.595716

Stk39	1.864889	1.500621	1.504801	2.233761	1.755917	1.822579	1.336077	1.896618	1.595625
4930555A03Rik	2.17901	1.465652	1.672458	3.073838	0.72393	1.640181	0.890371	0.627214	1.593815
Src	2.704296	3.871638	2.806396	1.156623	1.453935	2.08404	1.337404	1.688818	1.593575
Fancf	2.098188	2.288784	1.868916	1.564826	1.686058	2.395105	1.423132	2.309818	1.593558
Gm5846	2.424596	2.908305	2.195498	2.248108	3.182895	2.696689	4.199145	2.713992	1.592617
St8sia1	1.084658	1.751437	1.653004	2.670774	1.869267	1.671763	1.97518	1.016308	1.591686
Ufsp1	1.391518	1.221275	1.280345	3.486982	1.431287	1.338305	1.035506	1.483845	1.591488
Gm24313	1.66731	2.494307	2.918132	2.901449	2.778874	2.312814	1.723248	6.349771	1.589095
LOC118567630	1.146276	2.598236	1.667504	1.450724	1.687173	0.674571	1.723248	2.083519	1.589095
Gm25821	1.250483	0	0.833752	1.657971	1.587928	0.385469	0.430812	0	1.589095
Adamts6	1.877448	1.712866	1.909672	1.485059	1.828699	0.710266	1.664806	1.614857	1.586037
Gm42376	2.217523	1.382262	0.554445	0	3.167916	0	3.43788	1.319562	1.585122
2610306M01Rik	2.037376	1.604172	2.144855	1.972648	1.480812	1.53703	2.050315	2.399203	1.5841
Gm30881	1.984877	0.966778	2.031272	1.468843	1.714526	1.920926	2.480846	1.406361	1.583802
Gm30377	1.468981	0.95548	0.638762	0.63511	0.973246	0.885957	0.792138	0.85133	1.582687
Kcnq1	3.925666	5.697752	3.09621	1.523386	2.462111	2.007013	2.638941	2.674069	1.581776
Mogat2	0.031286	0.062405	0.187735	0.435545	0.953474	0.433979	0	0	1.580351
Fhad1	1.463427	1.480995	1.399027	2.760656	1.639708	1.791174	1.779441	1.557247	1.579379
Cyp2ab1	2.107911	1.982168	2.168389	1.227718	1.778117	1.336684	1.213809	2.207632	1.578521
Klra8	0.301021	0.050037	0.301056	0.898005	1.433446	3.015729	0.362974	0.812038	1.577949
Gm23442	1.241151	2.475693	3.723885	0.411399	4.334214	0	2.137985	0.393899	1.577236
H2ac15	0.827434	0.618923	0.206882	0.617099	1.871592	1.721666	1.603489	1.772546	1.577236
Gm22200	0	0	0	0	0	0.765185	0	0	1.577236
Mir7026	2.482301	1.650462	0.82753	4.936793	3.152155	0	0	1.575596	1.577236
Gm11400	2.6245	1.613535	2.085461	1.251273	2.225622	1.762109	2.563919	1.711494	1.576213
Gm25432	1.970849	1.26813	2.034661	2.023028	1.695368	0.881893	1.314174	2.239623	1.575427
Gm4828	1.051671	1.348548	2.178714	1.419272	1.287771	1.319892	1.086954	1.287377	1.575099
Gm33312	1.560343	0.366162	0.275387	0.182541	0.611904	0.509279	1.422964	0.436941	1.574624
Rpl21-ps6	0.61942	0.514809	0.412994	0.410633	0.491607	0.19094	0.853602	0.786331	1.574299
Zfp667	1.089462	1.583276	1.151851	1.114313	1.51192	1.079465	1.672934	1.807822	1.572365
Serpina3n	7.418331	8.697973	7.818074	2.934847	2.127138	1.278611	1.236648	2.025227	1.571184
Ltbp2	1.859203	1.474974	2.070717	2.64713	0.482914	1.966813	0.756986	1.005764	1.570624
Gm8149	2.000477	2.138594	1.66072	2.795382	1.282608	0.931035	3.473016	1.344459	1.570168
Cep170b	3.294565	2.822637	3.838264	1.559638	1.201675	1.255958	2.11913	1.334787	1.570006
Zfp286	0.548892	0.703839	1.568444	1.871373	0.896157	0.797654	1.296702	1.493139	1.569428
Snora5c	4.940025	2.737152	4.9406	2.183268	3.659309	4.568381	6.240377	3.658191	1.569428
Spef2	1.023922	1.385909	1.974936	2.000009	1.602068	2.029052	2.456709	1.531945	1.568392
3300005D01Rik	1.931391	1.212823	1.716991	1.707175	1.703181	1.719938	1.552591	1.566447	1.568078
Gimap9	0.52111	0.559701	0.68153	0.797217	1.450721	1.964694	0.994325	1.488443	1.566402
Gm16374	1.688237	0.455066	0.912667	0.998194	0.956024	1.350252	0.518747	1.390156	1.565553
Plod2	1.412919	1.621288	1.534638	1.480542	1.114139	1.109927	1.020655	1.316307	1.563836
Zfp316	1.777428	1.86093	2.163727	2.247328	2.198346	2.008152	2.610122	1.937323	1.563736
Supt4b	1.171227	0.778739	0.546636	0.776444	0.966735	0.649868	0.56491	0.594732	1.562796
4930599N23Rik	1.196958	1.570752	1.827148	1.879347	2.519933	1.631235	1.823118	1.559482	1.561105
Gm36025	1.454113	0.845974	1.636067	2.470195	3.05828	4.426364	2.066485	2.422802	1.559136
Gm5921	0.759426	0.524356	0.817938	0.697081	0.55636	0.756315	1.08679	0.834286	1.558954
Gm7808	1.306988	2.063888	1.960709	0.97475	1.867141	1.913714	0.787988	2.177666	1.557094
Mapk11	1.29295	1.560983	1.996365	0.744357	0.972151	1.762054	0.515776	0.79908	1.556585
Gm42031	2.36601	2.929296	3.467832	1.216943	1.709447	1.546698	3.035656	2.019638	1.555185
Rpl31-ps16	0.815266	1.084127	1.494827	0.945815	1.5529	0.879587	0.421309	1.423057	1.554041
Snord95	2.445797	4.065476	2.446081	5.674892	0.77645	1.507864	2.527853	8.538341	1.554041
Gm2830	1.739233	1.517778	2.609153	3.242796	1.86348	3.317301	2.134632	4.139802	1.554041
Tmem14a	2.391446	1.084127	1.84815	1.567351	1.604663	2.060748	2.022283	0.62097	1.554041
Gm14044	2.174042	2.710317	1.956865	1.297118	2.070533	2.010486	1.235839	2.587376	1.554041

Cbx7	1.648003	2.197858	1.70569	1.638772	1.770295	1.64807	2.020185	1.331877	1.55243
Mucl1	0	0	0.07081	1.40811	0.606881	0.851183	0.365888	0.067411	1.552056
Bach2	1.780794	1.929047	2.141204	2.58659	2.039022	1.893538	4.053324	1.758992	1.55105
Heyl	0.795118	1.598202	3.547861	1.362375	1.269869	1.742096	0.97351	2.480729	1.550612
Gm46289	1.312275	1.408604	2.369043	1.747272	1.313345	1.182698	1.793077	1.228238	1.547495
Gm5093	1.049602	0.475822	0.381718	0.379535	0.636128	0.794159	0.788957	0.545086	1.546017
Clba1	1.360621	1.587977	2.026692	1.928744	0.937416	1.606289	1.196825	1.295444	1.545115
Rpl22-ps1	1.245799	0.993986	0.872161	1.238821	1.186485	2.41936	1.802634	3.321144	1.543564
Rasal2	1.740208	2.281042	1.918003	1.892912	1.19059	1.366254	1.695815	1.318716	1.543488
Retn	1.238307	1.710015	1.714779	0.284162	0.907193	2.202207	0.393801	0.997607	1.54336
Ppp1r1a	1.16339	1.41253	1.972934	2.917333	1.734261	1.637188	1.411538	0.866865	1.542698
Gapdh-ps15	1.124048	1.704005	1.25908	1.028332	1.070531	1.081064	0.836467	1.755135	1.542698
Snord118	0.404657	0	0.404704	0	1.156174	0.374214	0	1.541094	1.542698
1700008J07Rik	1.390511	0.863914	1.003108	1.065376	1.454567	1.285901	1.036642	1.085166	1.542539
Enkur	0.554843	1.060618	0.924846	0.183912	0.880711	0.940686	1.05134	0.836419	1.542376
Rab38	1.371011	1.472543	2.601708	1.153588	2.276668	0.747717	2.652349	1.640039	1.54123
Spatc1	1.615328	0.902176	0.387724	1.477777	0.615368	0.597522	0.200343	0.492144	1.539551
LOC118567761	0	0.536801	1.34574	0.267609	0.256304	0	0.278146	0.256226	1.538953
Gm13578	1.613052	1.566422	1.27361	0.802008	1.212832	1.648721	1.711043	1.697445	1.537382
1810021B22Rik	1.610586	2.620802	2.543326	1.1801	2.744882	2.273324	1.05134	2.582628	1.535031
4833438C02Rik	1.713166	2.016154	2.535781	1.70357	1.729497	2.091253	2.97468	2.414031	1.534832
Zfp780b	1.478751	1.635338	1.971897	2.150684	1.197573	1.962878	2.422507	1.647357	1.53402
Zfp759	1.289628	1.460863	1.369393	1.757368	0.758165	1.104266	1.53036	1.621977	1.532618
Tlr11	1.624939	1.930943	1.659705	1.37518	2.634168	2.190094	1.286391	1.267306	1.532235
Pycr1	0.964451	1.353762	1.393258	0.426244	1.15667	1.783788	1.033727	0.51014	1.532012
Ccdc9b	0.892722	1.64714	1.517805	1.775444	1.445372	1.774958	1.568544	2.50738	1.531519
Gen1	2.955953	3.198392	3.569868	2.038166	1.991899	1.457046	1.916659	1.181499	1.528245
Zfp661	2.150964	2.975	2.070036	2.804805	1.410797	1.182227	3.376639	1.912688	1.527875
Acat2	1.709073	1.384925	1.495613	0.584203	1.297076	1.382935	1.600811	1.271254	1.527092
Smyd1	1.118107	1.814167	1.385278	0.879518	1.366851	2.191441	0.569185	1.366434	1.526909
Sycp2	1.221458	0.815968	0.426408	0.527098	0.98771	0.820534	0.250106	0.811869	1.526585
Tmem178	4.085942	4.955278	4.544096	1.430195	1.400906	0.544111	0.844605	2.520861	1.526552
Crim1	2.344709	3.102746	2.03613	2.60454	2.374684	1.988502	2.186947	2.036377	1.526147
Septin4	1.095281	1.890632	2.401472	5.403838	2.086267	2.571161	1.175567	3.168552	1.5257
Kirrel	0.791972	1.268569	1.920156	1.527343	0.708553	0.776779	0.578768	1.112012	1.524889
LOC115487912	1.599175	1.063278	5.331203	1.060145	1.523037	0	4.407539	7.105332	1.524156
Gm24762	1.599175	0.531639	1.066241	2.120289	4.061431	0.492956	2.754712	0	1.524156
Avpr2	2.821275	1.688259	2.163229	1.96383	2.328692	1.913287	2.624336	2.372749	1.523725
Tgfbr3l	1.443968	1.182959	2.063051	3.025604	1.964599	2.718364	1.279211	1.129299	1.523683
Gm14010	1.357993	1.832393	1.917389	2.462469	1.293338	1.034212	2.641983	1.140831	1.522692
Gm38555	0.86518	0.464626	0.26624	0.860334	0.570453	0.553909	0.687851	0.950465	1.522326
Gm24644	0.398835	0.397773	1.595525	0	0	2.950641	4.122159	0.37973	1.520501
Snora43	1.59534	0.397773	0	0.793202	1.519384	3.319471	0	0	1.520501
H2ac6	1.252194	2.724787	0.455396	2.150766	1.62624	1.894893	1.17655	1.300595	1.51894
Gm36681	1.564561	1.85038	1.232408	1.789855	2.254885	1.86939	2.618765	1.951	1.517558
H4c6	0.723735	0.433085	0.289527	0	0.137855	1.338574	1.496031	0.551253	1.517523
Gm21596	1.176588	1.035402	1.661259	1.238821	2.900298	2.816186	1.716794	1.252019	1.517179
Pfn2	1.207562	0.766403	1.097911	1.173507	0.705724	1.928872	0.822595	0.966808	1.517113
Ica1l	2.03676	3.237444	2.227965	1.898767	1.272986	0.882906	2.631366	0.666598	1.516573
Gm18445	0.471312	0.470057	0.412446	1.17168	0.897745	0.5993	0.182672	1.570573	1.516057
Sorbs1	1.558328	1.68192	2.327089	1.347939	1.64678	1.727338	1.224505	1.382059	1.515744
Gm3325	1.722384	2.491752	2.214752	1.261026	1.478145	2.135412	2.132292	1.513734	1.515309
Abi3bp	2.731437	1.643147	2.500495	3.046672	2.092124	1.577047	1.508628	1.224619	1.51515
Mlf1	1.788325	1.387216	1.142674	1.53132	0.520416	1.837541	0.513423	1.276995	1.515051

Adora3	2.407302	2.027939	3.295816	2.091685	2.715611	2.334265	1.835855	1.4464	1.514731
Smpd3	1.610603	1.794642	1.655226	1.745171	1.766653	2.085209	2.973389	1.501725	1.513875
Psma8	1.371515	0.50395	1.299481	1.292051	1.649956	0.801053	0.671461	0.96218	1.513571
Olfml2a	0.575322	1.308243	1.519028	1.212851	0.810937	1.383304	0.665979	1.6652	1.5134
2810414N06Rik	2.059618	2.139724	1.931117	1.920076	1.634632	2.063393	0.665225	1.307306	1.513145
A730017L22Rik	1.657306	2.281459	1.657499	2.414005	1.689555	1.230417	2.412548	1.400124	1.512818
Mif-ps	0.793389	1.978192	1.388592	0.690327	1.322326	0.366851	1.537514	2.077306	1.512341
Skint3	1.364141	1.091572	1.522939	1.798151	2.296248	1.68691	1.983708	3.005354	1.5118
Gm16124	0.528701	0.075328	0.075537	0.525739	0.647394	1.117545	0.234188	1.150572	1.511697
Gm10060	2.639908	8.951791	4.752386	6.825313	5.028438	8.788694	6.548343	1.00538	1.50964
Hk1os	1.583945	1.015539	0.905216	1.237557	2.586054	2.092546	1.754021	1.184913	1.50964
Gm23369	0.527982	0.526576	0.528043	0	4.022751	0	1.637086	0.50269	1.50964
Gm23851	0.527982	5.792335	4.752386	1.575072	3.017063	0.976522	1.637086	1.00538	1.50964
Mir7087	1.583945	0.789864	1.584129	0	2.262797	1.464782	5.7298	1.508071	1.50964
Gm32046	1.94947	2.065798	1.584129	1.332753	0.812286	2.02816	1.637086	4.408206	1.50964
Mir140	0	0	3.960322	0	0	0	0.818543	0.754035	1.50964
Mir6905	3.167889	0	2.376193	0.787536	0.754266	0	0.818543	0	1.50964
Gfpt2	3.201247	4.507375	2.259966	2.284497	2.941227	2.786271	2.024114	3.155474	1.507589
Serinc2	0.669028	1.819763	1.49028	0.33264	1.100574	1.490494	1.665822	1.853032	1.507156
LOC118568405	0.997386	0.248683	0.95594	2.355524	2.770541	0.999214	1.546273	1.266146	1.505113
Mypop	1.710239	1.731927	2.210412	2.380923	2.230221	1.313924	1.686035	2.129335	1.504625
Ino80dos	0.525978	1.258986	0.806593	1.569095	0.534331	0.518835	0.906041	0.567553	1.503911
Gm17669	2.323751	1.655403	2.24102	1.155367	0.790398	1.534952	1.88706	1.659329	1.50286
Prom1	0.147106	0.27247	0.084071	0.689616	0.600439	1.243788	0.152042	0.200085	1.5022
Gm15421	0.34221	0.273039	0.88985	1.429232	0.782202	0.759517	0.778121	1.368434	1.500321
Elmo3	1.009672	1.006984	1.409009	1.21416	0.670884	1.737141	1.189158	2.05675	1.499409
Zfp566	1.638241	2.124043	1.605662	2.182946	2.246749	1.333194	3.555727	3.119531	1.498933
4933417E11Rik	1.065141	0.910548	1.3189	0.756553	1.883939	1.688587	2.044485	1.448741	1.49859
Lama5	1.489095	1.648632	1.85817	4.605281	1.90611	2.286692	0.826006	1.554338	1.497363
Bbs10	1.570712	1.064437	1.067402	1.601962	1.457571	1.694636	1.332028	1.591335	1.497028
Epop	1.951039	2.111954	1.451551	1.112014	1.019716	1.826263	1.131205	1.087364	1.496682
Plagl1	2.061697	2.701761	2.529468	2.562685	2.397342	2.095035	1.635316	2.385197	1.496584
Afap1	1.500861	1.241718	1.705722	1.72989	1.55935	1.230228	1.401382	1.729375	1.495475
Tpt1-ps1	0.871668	1.043216	0.871769	0.433392	1.4943	0.967309	0.900912	1.493843	1.495398
LOC115490527	0.523001	0.521608	0	10.92149	0	1.934618	0.540547	0	1.495398
Ppp1r14a	1.774853	0.937127	2.297136	3.114549	3.877864	3.379206	0.431623	2.584452	1.492582
Cbx2	1.255592	1.782613	1.96486	2.012382	1.491248	1.243094	2.152683	1.617368	1.492343
Gm9795	0.900276	0.661595	1.421654	1.272174	0.406143	0.482001	1.322262	1.443623	1.490286
M6pr-ps	1.31402	1.513879	1.404806	0.49563	0.884652	0.963751	1.170781	0.884381	1.489898
Zfp456	1.427065	1.247052	1.563157	1.405556	1.423841	1.457959	1.011389	1.345765	1.489655
Suv39h2	1.449484	2.159051	1.807359	2.321159	1.272904	0.940047	2.354179	1.684738	1.489136
Fmod	1.100596	0.814397	1.52681	2.01234	4.2435	1.493862	1.761322	1.521109	1.488854
Gm24347	3.51368	1.557478	1.171363	1.552888	2.602748	0	3.228057	0	1.488377
n-TGgcc14	0	0	0	0	0	0	0	0	1.488377
Gm46116	2.109515	1.809353	3.122446	1.887929	1.687627	1.36557	2.877982	0.843555	1.487811
Enah	1.777486	2.032182	1.712655	2.133969	1.47609	2.054705	1.971544	1.516916	1.487504
Tesc	0.860339	0.80442	0.376442	1.176339	3.482352	3.779167	3.556811	1.433472	1.486212
Slc9a5	3.039644	3.377117	3.150256	2.756376	1.664956	1.485589	2.783511	1.439522	1.486052
Iqcn	1.621858	2.040307	1.115157	3.115585	1.992235	1.729933	5.714562	1.684547	1.484295
Gm9803	1.661066	0.724781	0.415315	0.722645	0.395495	1.536101	0.965697	1.976872	1.484197
Gm14023	1.213972	1.893722	1.058458	0.742875	2.460575	0.431786	1.447732	1.392912	1.483363
Tgfb3	0.704235	1.388387	1.24484	1.87287	0.982662	1.13591	2.065098	2.463702	1.482881
Phldb2	0.583086	1.103424	1.435456	1.992203	1.352715	1.272006	0.695364	1.295363	1.481955
4933407O12Rik	0.971745	1.308363	1.312008	0.821357	1.203123	1.572619	2.008694	0.601378	1.481855

1500004A13Rik	1.878159	2.01849	2.445129	1.899839	1.588276	1.242755	3.212972	1.371974	1.481422
LOC115488583	0	1.033467	1.036346	10.81942	4.441004	3.833075	2.677477	0.493294	1.481422
Gm23217	0	0.516733	0	3.606474	0.493445	1.916538	2.141981	0	1.481422
Gm26058	0.518113	0	1.036346	0	0.493445	0	2.141981	1.973176	1.481422
Col27a1	2.135026	2.708755	2.178851	1.415378	1.756726	2.377345	2.867152	1.382826	1.481164
Zfp3	1.398876	1.97141	2.311455	2.93328	1.737749	2.531028	1.382947	1.795125	1.478172
Nat8f1	1.951236	1.659859	1.147919	0.913085	1.749022	1.379867	1.660812	1.366006	1.476822
Gm30970	2.347081	3.043083	3.333243	3.220829	3.218882	1.519355	1.601045	2.50281	1.476405
Gpr141b	2.261531	1.666233	1.895016	1.377682	2.076241	2.487062	1.326635	0.562547	1.475797
St14	0.827702	1.292085	1.709584	0.697817	0.685474	0.898552	1.283208	1.027896	1.474851
Tssk4	1.194483	2.032223	1.264894	2.515324	1.003776	2.209241	2.832221	1.003469	1.473286
Gm10291	1.590436	2.356643	1.681514	2.394884	2.683208	2.353257	1.831658	2.076687	1.472518
4930589L23Rik	0.487725	0.36482	0.569079	1.010402	0.774173	0.902065	0.882154	0.619149	1.47201
Spns2	3.163533	2.052723	2.458694	4.169149	2.958464	3.806722	0.728782	1.705587	1.471237
Zfp619	1.886976	1.501854	1.877899	1.580618	1.673195	1.573094	2.565152	1.46913	1.470659
Gm5879	1.865827	1.690918	1.48261	2.050232	1.931161	2.001216	2.130954	2.100916	1.469736
Gpr15	1.417027	0.347115	1.168561	1.804623	1.610002	0.758665	0.873602	1.396487	1.469022
Gnb4	2.146953	2.131928	1.577727	1.577989	1.120159	1.407069	1.186674	1.128704	1.467953
LOC115487267	0.769973	1.535846	0.770063	0.76566	0	2.136141	4.774834	0	1.467706
H2ac14	1.077962	2.150185	2.002163	1.684452	1.613291	1.566503	3.024061	1.46618	1.467706
LOC115487269	1.539946	0.767923	0	2.29698	0.733314	2.848188	2.387417	0	1.467706
Snord88a	0	1.535846	0	3.06264	0.733314	4.272282	0.795806	1.46618	1.467706
Gm36208	0.769973	1.228677	0.924075	0.76566	0.146663	0.284819	0.477483	1.46618	1.467706
n-TDgtc14	2.309919	2.30377	0	5.35962	0.733314	0	0	1.46618	1.467706
1810014B01Rik	1.98482	1.399327	1.43745	1.054909	2.379196	1.265861	1.733088	1.726834	1.467706
Rbp1	0.864901	0.967794	0.696221	1.237642	1.486719	1.092456	0.414255	1.46618	1.467706
Mgam	0.606674	0.511318	0.358919	0.424842	0.398756	0.63215	0.150133	0.21152	1.465896
Gpr4	2.018376	1.687088	0.865119	2.140875	1.70259	2.044296	0.774834	0.713771	1.46567
Ldb2	0.9277	1.084752	1.407708	3.053804	2.559194	2.425808	0.925761	1.431492	1.463471
Bbs12	1.181041	1.531266	2.527721	2.0435	2.114644	1.681972	1.538038	1.191935	1.463328
Gm7079	4.093888	3.572615	2.21778	5.767187	3.736517	3.312662	2.820825	1.299261	1.463189
Gm6651	1.705787	1.87137	1.194189	0.848116	1.78703	0.946475	1.234111	1.136853	1.463189
Tmem47	0.325946	0.880422	0.950788	1.120917	0.620856	1.01731	0.350919	0.801694	1.462672
Slc24a3	2.871415	2.469813	2.810971	2.205705	1.95336	2.149605	3.203287	1.345237	1.462477
Etl4	1.813449	1.310091	1.174228	3.340712	1.306403	1.741522	1.225497	0.785816	1.462474
LOC118568399	0.789981	0.633392	0.371799	0.477495	1.13593	0.28649	0.944561	0.737391	1.461554
Mindy4	2.542072	2.118542	1.532386	1.350486	1.824072	1.674563	2.015508	1.79036	1.46033
Fzd1	2.165351	2.450057	2.317575	1.662136	2.110497	1.370097	1.073192	1.446756	1.46033
Afap1l1	2.476223	1.998451	1.450062	4.61691	2.451419	2.726828	1.81845	1.830247	1.459511
4930404I05Rik	1.587184	1.526424	1.700752	3.720262	1.187699	1.467778	2.519237	0.917487	1.458701
Upp2	1.615526	2.628841	2.040902	2.029234	1.174202	3.145238	1.933368	1.740526	1.458701
Irgc1	2.22587	1.769018	2.017429	1.41796	2.418004	2.508693	2.264601	2.019906	1.458498
Tubb3	0.828861	1.971253	2.008628	1.172926	0.151807	1.680415	0.395386	1.365849	1.458422
Gm38933	2.471761	2.46518	1.883465	2.457915	3.587166	1.523871	0.851563	0.784453	1.458357
Gm27042	1.39269	3.046801	1.527644	1.250868	2.139328	0.332366	1.114386	1.582619	1.455812
Rom1	2.107082	2.173937	2.761325	1.481146	1.626169	2.788462	1.727201	1.314373	1.45424
Gm8991	1.523432	1.185855	1.85806	1.145411	1.13241	0.996484	0.844877	0.70754	1.451966
Gm8666	0.609209	1.924027	0.91392	1.817391	1.934015	0.75117	2.413652	1.353397	1.451577
Ly6g5b	1.284831	1.186491	0.475919	0.993715	1.3143	1.012146	2.164045	1.540433	1.451328
Grin3b	1.564352	1.517442	1.693126	1.683446	2.163375	2.001548	2.524922	2.387147	1.450118
Mir1943	3.797128	0.757404	4.557083	1.510343	0	0.702293	9.41885	5.78438	1.4476
Mir6994	2.278277	0.757404	0	2.265515	0	0	3.139617	0.723048	1.4476
n-TYgta3	0	0	0	0	0.723269	0	0.784904	0	1.4476
Gm51550	0	0	0	0	0	0	0.588678	0	1.4476

Prg3	0	0	0	0	0	0.594248	0.060377	0.055619	1.4476
Plxna2	2.373177	2.46249	3.036741	3.075	1.689434	2.253755	2.254591	1.453077	1.446974
Gm8493	1.233314	1.363994	1.294519	0.874269	1.069928	1.422839	1.43876	0.906835	1.443136
LOC115489415	1.802278	1.653681	1.802487	2.078931	1.71647	0.466673	1.788234	2.127772	1.442894
Gm12115	0.567625	1.037876	1.229998	0.846668	0.5406	0.437435	0.586669	0.36029	1.442659
Scx	1.805641	1.557478	1.51301	1.067611	1.719673	3.700639	2.168851	2.880733	1.441866
Gja4	1.019324	0.721465	0.855016	4.773795	2.755799	2.706285	0.543753	1.033109	1.44159
Gpr153	1.834725	1.903034	1.49731	2.072573	1.328003	2.036036	1.592875	1.523243	1.440893
Gm8783	0.922659	0.920203	1.197633	0.663717	0.803945	0.980325	1.055062	1.270966	1.44068
Ndufa4l2	2.615003	1.622781	1.917892	3.987211	1.9924	3.654279	1.621642	1.881136	1.440013
Rps19-ps7	2.049267	0.968121	0.970818	1.287024	1.232652	1.49613	1.672121	0.821517	1.439151
Gm16470	0.96056	0.729907	1.006418	0.95518	0.827701	0.930596	0.661858	0.914548	1.438642
C430042M11Rik	0.625795	0.917837	1.030841	1.317789	0.385647	2.212736	1.141395	1.331829	1.438468
Gm50340	3.415239	3.326934	3.336202	2.527336	2.571852	4.039694	1.723865	2.495446	1.438267
Ifi203-ps	1.222207	0.93961	1.404526	2.576799	1.690063	1.204523	2.127622	1.079743	1.437422
Klf8	1.907375	2.76939	2.697252	1.587927	1.411004	0.959879	2.228103	1.123391	1.437407
Asb1	2.78535	2.777934	3.035423	1.604543	1.710558	1.714242	1.905963	1.646023	1.437192
Srd5a1	3.014759	3.006733	2.456756	2.720291	1.860978	2.478182	3.577519	2.976655	1.436666
Abhd18	1.506552	1.836439	1.871994	1.54351	1.724687	1.505795	1.509912	2.158822	1.435878
Gm16433	1.456947	1.354555	0.938482	1.399674	0.752586	0.822105	1.046422	1.058	1.435671
Sapcd2	2.061091	2.081958	2.193467	0.945944	1.082145	2.443631	1.665957	0.930863	1.435525
Yap1	1.331159	1.448307	1.277523	1.818419	1.165334	2.200904	1.195156	1.126572	1.435311
Cpe	1.99087	2.138306	0.995551	4.34015	4.08387	3.115697	1.134353	2.381529	1.43527
Echdc1	0.940904	1.463902	1.072755	0.374253	0.698963	0.504669	0.739078	1.057083	1.434824
Gm13868	0	1.000733	0.37632	0	0.238908	0.11599	0.259267	0	1.4345
LOC114841036	1.384447	1.290711	1.474908	1.376692	1.146549	0.528817	1.244256	1.060234	1.43424
Gm17828	1.025365	1.295338	1.70914	0.339874	1.106752	1.517161	2.119532	1.366747	1.43332
Znrf3	1.582438	1.069686	1.608999	1.80961	1.339641	1.146321	2.162532	1.607078	1.432793
Rad51b	2.039348	1.980394	1.932238	0.587031	3.015603	2.183703	2.0523	1.686178	1.432185
Rpl13-ps3	0.949011	0.946485	1.660962	1.179619	0.903828	1.682097	0.817375	0.978848	1.432112
Gm2423	1.840424	0.936492	1.727945	0.746985	0.894285	1.945104	1.436332	1.108575	1.431908
Klhl42	1.611424	2.120408	1.974435	1.644346	1.6713	1.615028	2.145078	1.494071	1.431298
Zfp934	2.173027	2.476848	2.923579	2.212304	1.527538	1.674623	2.032034	0.812796	1.430037
Gnmt	1.679941	0.359029	1.020083	0.954589	0.914262	1.054199	1.612282	1.370973	1.429583
Selp	2.580015	1.849449	1.741712	4.040761	2.242175	3.385019	1.416617	2.870949	1.429284
Plxnb3	1.321516	1.280608	1.79972	2.646867	1.990548	1.490784	2.760766	1.909628	1.429245
Il20rb	2.147722	2.878318	2.080847	2.847588	1.661942	0.744805	1.225502	1.235425	1.428614
Htr7	3.375975	2.629277	2.54176	1.904851	2.077263	1.964402	0.725291	1.065401	1.428038
Snord87	0	1.494337	5.244751	0.744967	2.853979	0.692802	0	2.853106	1.428038
Gsg1	1.133239	1.537102	2.266742	1.712875	2.072226	0.712629	1.358661	1.208429	1.425702
Mmp28	1.59534	0.729251	1.662006	2.346555	2.120807	0.983547	1.648863	1.487276	1.425469
Rpl13-ps6	1.643716	2.011917	2.465861	2.377467	2.134714	1.243683	1.4672	2.63201	1.424189
Dtwd2	1.261279	0.870868	1.222612	1.44717	1.145787	0.789557	1.123097	1.422559	1.424039
H2bc14	0.114779	0.343419	0.114792	0	0.437256	0	0.593147	0.655683	1.422125
Gm39792	0.438592	0.612394	0.350915	0.348908	1.086047	1.054551	0.090661	0.668133	1.421259
H4c12	1.084363	1.351845	0.677806	0.808717	0	1.128133	0.280186	0	1.421055
LOC115490200	1.087313	1.79746	1.236404	1.792163	1.659709	1.818188	0.90827	1.304671	1.419597
1700020L24Rik	0.833655	0.890824	0.47643	1.421118	1.64464	2.092546	0.861624	0.566944	1.418835
Zfp760	0.961506	2.036119	2.318421	1.571704	2.747183	1.071877	1.388548	1.943759	1.418538
Gm34620	1.511732	1.36636	1.582779	1.996523	1.214795	1.485378	2.07513	1.641721	1.418303
Loxl2	1.402005	1.654445	1.862422	2.90537	1.386218	1.761698	1.338428	1.395984	1.417838
Mdga1	1.817161	2.075402	1.846685	2.593894	1.933018	2.032807	2.059881	1.792902	1.417657
Gm38514	0.826201	1.318402	0.247889	0.657258	0.157373	0.305618	1.280879	0.786624	1.417397
Lynx1	1.739453	2.518291	1.964127	1.938949	2.805594	2.451806	1.188876	1.442436	1.417197

LOC118567914	3.039861	2.313127	1.103485	1.164351	1.372506	1.249408	1.117102	0.878993	1.416437
Yod1	1.529127	2.489006	2.005409	2.481671	1.236501	1.360727	2.430282	1.414675	1.416147
Gm16580	3.607453	3.280392	3.395644	3.21797	1.818899	2.698283	2.63187	2.980063	1.415739
Ift81	0.580139	0.995182	1.137204	1.361458	0.950331	1.287586	0.767491	1.524483	1.415485
Snora2b	0	0.493665	0	0	0.471416	4.119701	0.511589	0	1.415287
5930403N24Rik	0.758522	1.545897	1.715119	1.869285	2.041588	1.768893	0.954399	1.821168	1.414446
Gm4596	0.723735	0.90226	1.121919	0.827633	1.171771	0.267715	1.122024	0.654613	1.414056
1810044D09Rik	2.395472	1.365197	2.0535	3.06264	2.064143	3.270142	0.471589	2.606542	1.413346
Cpeb3	1.261269	1.411549	1.800647	2.029704	1.311254	1.175286	2.189226	1.402521	1.413157
Gm20257	1.776861	2.244699	2.606366	1.825805	3.271708	1.15023	1.591611	1.973703	1.411255
Scara3	1.255813	1.555486	1.01287	2.719114	1.871196	1.51723	1.130468	1.40779	1.409255
Ticrr	2.294839	2.695785	1.933126	1.631082	1.298146	1.303227	2.244484	0.982477	1.409193
Gm10327	1.43215	1.013659	1.339909	1.378188	1.715955	1.153516	1.384702	1.14362	1.408997
Tm4sf5	1.023472	1.587829	2.502111	0.848116	1.028896	1.209384	1.234111	0.270679	1.408997
Mir6951	0.739174	2.211619	0	0.735034	2.815926	1.36713	0	2.815065	1.408997
4933428G20Rik	1.517252	1.901216	1.361795	1.160579	1.593221	2.158627	2.211502	2.25946	1.408997
Fam110b	1.828483	1.105809	1.653608	1.702183	1.519118	1.385119	1.427424	1.222331	1.408997
Kcnh7	0.396773	0.263811	0.286592	0.657585	0.293909	0.530001	0.569563	0.272832	1.407597
Gm7363	1.022423	0	0.227232	0.338899	0.757357	1.470786	0.821898	0.757126	1.407554
Eif3s6-ps1	0.830187	2.207939	0.276761	0.733811	1.581323	1.108945	0	0.702595	1.406653
Mmp15	1.642609	1.420658	1.540125	4.262174	2.199942	3.607705	1.737509	1.734979	1.406551
Trmt12	1.690103	1.926404	1.82445	1.89405	2.19728	1.562954	2.329074	1.813479	1.40627
Nfia	1.739021	1.932436	1.46841	1.717312	1.535876	1.38004	1.579691	1.804676	1.405097
Spag1	1.283729	1.62805	2.28245	1.118941	1.735803	1.275089	2.162189	1.373129	1.404768
Gm7517	1.113346	0.718482	1.309971	1.43273	0.810847	1.090151	0.981478	1.40296	1.40442
Gm7407	2.649253	1.174311	1.471978	0.195142	1.588631	0.635171	2.332485	1.868406	1.402763
Gm8834	1.962409	2.6422	4.121539	3.122267	2.42967	2.268468	2.231073	1.588145	1.402763
Dnase1	1.255038	1.122211	1.688006	1.850495	1.236501	1.200641	1.341874	1.359736	1.402398
Lrrc1	1.269574	1.748554	2.176665	1.502931	2.072791	2.161765	1.666245	2.110531	1.402083
4732465J04Rik	0.459685	1.742154	0.459739	0.365688	1.400958	0.510123	0.380086	0.087533	1.401987
Sez6l2	1.470587	1.206949	1.394156	2.848535	2.086267	1.728273	2.279887	1.837688	1.4016
Cmtm4	2.179102	2.616539	2.337071	1.418461	1.870548	1.577662	1.911415	0.969112	1.400526
Gm28071	0.489736	0.879177	0.783668	0.486992	0.932838	1.449255	0.809866	1.305573	1.400284
Ccl21a	0.702556	0.127397	1.66078	6.732163	2.798083	0.885957	0	1.094567	1.400069
Gm13450	0.815266	0	0.489216	1.134978	0.31058	0.603146	0	0.776213	1.398637
Lgalsl	2.104032	2.495861	1.418793	2.536056	0.926025	1.2382	1.021414	0.682924	1.397654
Gdpgp1	2.535661	2.27759	2.252433	1.66009	2.369937	2.577934	2.457954	2.264248	1.395988
Gm6142	0.941401	0.521608	0.313837	0.936128	1.29506	1.450964	1.081094	2.19097	1.395705
5730552O08Rik	2.391998	2.203199	2.29377	1.259258	1.742086	1.340239	1.745117	1.862123	1.394693
Gm20605	1.400385	1.617182	1.31455	1.661277	2.199454	1.828949	2.196445	1.461956	1.393231
Lca5	1.95826	1.540431	2.179162	2.27641	1.497274	0.816197	1.225778	1.102917	1.393225
Erbb3	1.413906	1.373276	1.802247	2.435205	1.883464	1.922847	1.566407	1.530947	1.391617
Gm5786	2.684369	2.269819	2.101055	1.68284	1.945212	2.698283	1.749097	1.833496	1.390458
Gm25177	0	0.363753	0	0	0.694718	0	0.376961	0.694506	1.390458
Gm22847	1.945195	0.970008	1.945421	0	0.463146	1.798856	0.502614	0	1.390458
Wdpcp	2.586169	2.321356	2.51593	2.548304	2.933256	2.76122	3.839307	2.216058	1.389279
Klrg2	1.409031	1.38105	1.895123	2.246652	1.804685	1.280561	1.205217	1.202756	1.389239
Slc39a4	0.716446	0.911652	0.667113	0.417633	1.599958	0.868164	0.510677	0.870299	1.389219
Zfp945	1.037257	1.782854	1.721605	1.711762	1.797086	1.561288	1.733538	1.218704	1.388244
Irak1bp1	1.36547	1.936831	1.244239	0.935388	1.473853	1.767841	1.411281	1.791195	1.388175
Fgfrl1	1.917516	1.089348	1.335135	2.051594	1.571937	1.683474	1.003468	0.808838	1.388023
Fam199x	1.50302	1.625602	1.282726	1.282035	1.545977	1.013116	1.877944	1.221139	1.387945
Zfp420	1.437925	2.038836	1.490071	2.205101	1.385963	1.634148	2.094958	1.484507	1.386982
Gm5500	1.282794	1.428639	1.454002	1.126787	0.509049	0.988573	1.679386	0.854942	1.385632

LOC118568499	1.041887	1.256602	0.508888	0.698732	0.369221	0.358513	1.67787	1.56871	1.385596
Yes1	1.089394	0.80481	0.605289	2.246828	2.305616	2.798438	0.875734	0.96038	1.384386
Clip3	1.335053	1.298211	2.019502	1.692657	1.446319	2.098845	1.604068	1.874874	1.383761
Gm23971	0.580503	0	0	0	1.382162	2.415741	0.899969	1.38174	1.383178
Pnp2	1.269228	2.170027	2.493416	2.208707	1.597341	2.054049	1.358661	1.251588	1.382499
Dip2c	1.713013	1.508673	1.581958	1.765235	1.138071	0.594053	2.462972	1.203488	1.382489
Gm14269	1.076013	0.746538	1.403658	0.790859	1.604008	0.432636	1.305524	1.469892	1.382244
Gm6085	1.318384	1.314874	1.648172	1.769849	1.820642	2.621281	1.362616	1.129708	1.382191
Gm33862	0.895538	1.658714	0.725044	2.968405	0.893515	0.985911	0.705206	0.568427	1.381901
Lzts3	1.881855	2.293922	2.119076	1.358435	0.995699	1.044169	1.930584	1.340465	1.381717
Plekha4	1.470506	1.864665	1.722793	2.903648	3.101093	2.797462	1.498129	1.960092	1.381501
Fhdc1	1.879098	1.613027	1.355726	2.091685	1.273221	1.201714	2.05809	1.068111	1.381079
Ryr1	1.643064	2.052864	1.859948	1.522542	1.619863	1.365839	1.511575	1.664064	1.380133
Slc22a21	1.447746	2.042578	2.330788	1.36941	1.546965	1.502102	1.715291	1.916306	1.37983
Zc3h6	1.302573	1.456139	1.903981	1.466082	1.472308	1.456084	2.100779	1.54	1.377892
Lncpint	1.615526	1.611225	1.275564	2.282888	1.214692	2.280298	2.812172	2.428641	1.377662
Snhg4	2.022935	1.633255	1.107927	1.341069	0.825695	0.668124	0.796497	3.210055	1.377169
Mss51	1.132186	3.387515	1.171363	1.979932	1.487285	1.660774	3.308758	1.524001	1.376749
Ly6d	0.320915	0	0.080238	0.079779	0.382045	0.74193	0.08292	0.687471	1.376372
4930579G18Rik	1.973155	2.157123	2.011334	3.094084	2.529707	1.543987	4.235581	1.914764	1.374279
Klhl13	0.990287	1.185181	1.224497	1.432349	0.703067	0.732629	0.614107	0.634281	1.372846
LOC118568520	2.159925	1.566672	2.683854	4.751251	1.683072	0.605282	3.991242	2.118777	1.3724
Rpl30-ps8	0.719975	1.077087	1.200098	0.477295	1.028544	1.220652	0.496087	0.913982	1.3724
Gm13199	0.89851	0.806506	1.88709	1.250868	0.513439	0.498548	0.557193	1.11211	1.370176
Aplp1	1.691881	1.872294	2.387452	1.56717	1.412672	1.328837	1.365379	1.434306	1.369531
Gm6851	4.669729	3.701954	3.472766	2.381319	1.93861	3.321861	0.247508	4.218037	1.369436
Shox2	1.487238	1.636721	1.23096	1.376913	1.514114	1.04337	0.795069	2.29489	1.368591
Gm10054	0.716872	1.549086	0.238985	1.306903	1.137901	1.104901	1.728819	1.137553	1.366484
Gm31204	0.390868	0.324856	0.195457	0.194339	0.372258	0.120487	0.471311	0.620241	1.365949
Lhfp	1.93728	2.540874	1.910964	3.404237	3.083499	2.527784	1.481136	3.082557	1.36583
0610009L18Rik	1.34341	1.518478	2.060135	2.493652	1.876526	1.656458	1.851309	1.53487	1.365749
Zfp560	0.969551	1.645292	1.736711	0.575594	1.516013	1.150873	1.420859	1.51555	1.365414
Cep290	2.390387	2.14158	2.282612	2.148698	1.52415	1.45497	2.812557	1.697268	1.364376
Rundc3a	1.043751	1.30865	1.103522	1.21583	1.420081	0.744604	0.924658	1.64679	1.364279
Cd46	1.368841	1.241088	1.929046	2.660475	1.244412	1.3234	2.057841	1.184792	1.363928
2310047D07Rik	1.144739	1.671762	0.94043	1.097672	0.934489	1.739159	1.2254	0.233551	1.363797
Zfp458	1.335091	1.220575	1.525996	1.027319	1.286663	1.6462	2.053398	1.422463	1.36335
Fam161a	2.231253	3.081203	3.375878	1.336942	1.961558	1.613679	2.631333	1.933723	1.363194
H2ac10	0.549981	2.194066	2.420197	0.98442	1.047591	1.932699	1.250552	0.523636	1.362869
Pank1	1.270645	1.777458	1.312052	1.02559	0.950831	1.045339	1.645857	1.052664	1.360451
Dyrk3	2.852638	3.301324	2.987544	1.177481	1.050846	0.871048	1.474172	1.844824	1.359409
Gm39321	1.507054	1.395682	0.376807	0.56198	1.102107	0.995483	0.222517	1.30675	1.359409
Lrfn4	3.038743	2.310873	2.640215	2.096319	2.025846	2.177854	1.766639	1.410426	1.35759
Tcaf1	0.850862	1.126585	1.320457	1.925598	1.075812	1.153143	0.894571	1.271025	1.356239
Gm26457	4.975211	9.923931	6.397443	7.067631	2.030716	7.230016	0	7.443682	1.354805
Mir6973a	1.421489	2.835409	0	1.413526	0.676905	0.657274	0	0	1.354805
Mir7228	0	0	0	0	0	0	0	0	1.354805
Gpr82	0.976882	1.062852	1.265654	1.788278	1.14182	0.759669	1.19323	0.803257	1.354263
Elp4	1.501875	1.71069	1.132693	1.256793	1.461634	0.736186	1.408063	1.265842	1.353201
LOC118568784	2.05852	3.752106	1.845784	2.117575	1.960512	1.969298	2.421042	2.635744	1.35307
Stard6	1.261748	1.926908	2.366053	1.92123	0.863704	1.750238	2.159882	1.163767	1.352878
Angptl1	2.17702	5.150345	3.569715	3.196893	1.759953	2.01324	1.517481	2.988596	1.351093
Tmem191c	1.732439	2.356128	1.496372	2.114266	2.099944	2.621628	1.709173	2.28674	1.350956
Dusp8	1.674265	2.101855	1.018727	1.618316	0.992413	1.191006	2.335484	1.181614	1.350227

Ngfr	1.691276	1.394831	1.349925	1.24518	1.31648	2.000165	1.445476	0.727714	1.348446
Zfp109	1.69523	1.670589	2.502773	1.665666	1.306991	1.026467	1.543521	1.767742	1.346421
Gm39712	1.078472	1.098486	0.608145	0.707348	0.546343	0.816968	1.197661	1.004964	1.344992
2210417A02Rik	3.326284	3.317428	3.32667	3.608347	2.687929	2.703189	0.729247	1.439522	1.344952
Hoxb3	1.446364	0.967539	1.234844	1.964455	1.797471	1.712719	2.151182	1.477842	1.344891
Scarna3a	0.469814	0.937127	0	0	0	0	0	0.447309	1.343324
LOC118568495	2.167666	3.242843	2.059522	1.347202	2.116073	1.353098	2.184381	2.528193	1.342886
Klre1	0.384097	0.319229	0.832308	1.90973	0.73162	2.368009	0.926296	1.158045	1.34229
Rps6-ps3	0.737387	0.334283	1.13973	1.066554	0.702279	0.991872	0.138568	0.702064	1.341699
Car4	1.663142	1.531121	0.895642	2.71397	1.827644	2.563369	0.57298	0.730834	1.341257
Mettl15	1.759008	2.514532	2.638818	1.574239	1.647339	1.572453	2.181627	2.093435	1.341193
Gm46209	2.532703	1.777528	1.78248	1.679011	1.786755	2.775899	2.229872	1.607588	1.341051
LOC115488402	1.407057	0.280662	0.281444	0.839505	0	2.602405	0	0	1.341051
Trim46	1.609558	2.15391	1.263344	1.985482	1.028418	1.676882	0.947597	1.0669	1.339868
Aoc3	2.248658	1.854517	1.578568	3.827106	2.718181	2.159469	1.184397	2.099771	1.339482
Mir3108	0	0	0	0	0	0	0	0	1.337656
Mir7001	0.701748	1.399759	0	0.697817	0	0	0	0	1.337656
Bend3	2.248379	1.998451	1.79703	1.814821	1.406649	1.18316	2.119628	1.397262	1.335953
Arhgap29	1.704062	1.598895	1.80517	3.121478	1.537512	2.249752	1.297751	1.846586	1.335627
Cdo1	1.237748	1.266938	1.368196	1.101255	1.209839	1.265118	2.322892	0.558216	1.334905
Gm4963	1.167117	1.629614	1.634154	0.696348	1.667324	1.834833	2.050665	2.22242	1.33484
Mir1931	0.465866	4.181632	0.46592	0.463257	0	0.430818	0	0	1.332035
Uckl1os	4.936266	4.418188	3.417812	7.551716	7.59432	3.862611	9.026398	4.940825	1.326967
Gm6181	0.126571	0.504936	0.506343	0.755172	0	0.234098	0.392452	0	1.326967
Ddias	2.1612	2.122788	2.095953	1.774631	0.764067	1.286984	1.895268	0.826187	1.326397
Gm20636	1.137839	1.13481	0.885089	1.508621	1.023462	1.052238	2.809366	1.083335	1.325454
Casq1	3.372433	4.632126	2.573998	4.441972	3.042822	5.444082	2.415444	2.422248	1.325164
Tspan9	1.562858	1.313347	1.736711	4.014769	2.715042	3.144828	1.585379	2.025326	1.324038
C330013E15Rik	0.757696	0.377839	0.505189	0.879027	0.541216	0.817475	0.456818	0.480934	1.323944
Krt83	0.094658	0.157343	0.031556	0.407887	0.450756	1.429768	0.097834	0.150206	1.32319
Gm52552	1.214605	0.865266	1.908888	0.431358	0.413135	0.481384	1.52436	0.743415	1.323002
Adamts4	1.554831	1.384233	2.046993	2.271139	1.756886	1.437859	0.871591	1.43017	1.322819
Epm2a	1.541373	1.639754	2.158174	4.087305	1.908383	1.282872	1.274469	1.614292	1.322159
Tmtc4	2.389284	2.266276	2.088777	1.877459	1.909534	1.591483	2.279487	1.368081	1.321731
LOC118567327	0.519732	0.518348	0.259896	0.086137	0.494987	0.560737	1.342922	0.082473	1.320935
Nectin3	0.873752	0.570934	1.717574	1.707755	0.832152	1.588174	1.121048	1.290876	1.320935
Calml4	0.652213	1.300952	1.712257	2.837446	1.164675	2.035617	0.926879	0.776213	1.320935
Gm26361	0	0	0.693056	0.344547	0	0.320421	0	0	1.320935
Osbpl5	1.261354	0.951168	1.230733	1.942618	1.39175	1.835042	1.63754	1.420616	1.319469
Gm46442	1.284661	0.788456	1.680137	1.867064	0.658806	1.462171	1.327761	1.787642	1.31858
Cd79a	0.442619	0.05518	0.332003	2.585822	2.318502	2.609418	0.400285	0.526771	1.318298
Snhg18	0.747648	0.52196	0.672961	1.932995	1.24609	1.659362	0.425002	1.566034	1.318263
Tango6	2.263291	1.805813	1.924021	0.962762	2.023807	1.406975	1.442522	1.556299	1.318239
Gm34288	2.113475	1.621421	1.05686	0.969986	2.399937	0.601377	1.26022	2.55399	1.317061
Tube1	1.608821	1.680045	1.665796	0.696387	0.93736	1.767841	1.506298	1.135301	1.316876
Necab3	0.899343	1.345423	1.252802	1.437276	1.040065	0.623763	1.029107	1.070328	1.316343
Syne2	2.006369	2.016302	1.641533	1.87329	1.225274	1.277081	1.74126	1.22733	1.316191
D430020J02Rik	1.706081	1.950079	2.51149	1.391532	1.332745	1.347276	1.268006	1.387091	1.315454
A530013C23Rik	1.301974	1.064777	0.807318	1.191107	0.793591	0.722415	0.807393	1.016478	1.315351
Dapk2	0.658112	0.812636	1.065638	2.056765	1.253556	2.173575	1.846233	2.297485	1.314215
Smarcd3	0.571526	1.240599	1.176809	2.306731	0.89652	1.834309	0.972919	1.216333	1.313725
2010310C07Rik	0	0.981488	0.262459	0.06524	0.374901	0.182014	0.678083	0.687109	1.313119
1700048M11Rik	0.688672	0.343419	0.229584	0.570678	1.093139	1.061437	0.474518	0	1.31273
Gm20544	2.178071	1.31156	1.582367	2.084139	0.958907	1.121118	1.741452	1.467267	1.312122

Gm8797	1.238071	1.234775	1.513374	0.547171	1.703181	2.798716	0.568715	2.226556	1.311102
1700064M15Rik	0	0	0	0	0.49115	0	0	0.491	1.310695
Map10	1.359389	2.337534	1.406428	1.817903	1.547648	1.27157	2.018673	1.666188	1.31051
Gm15764	2.363843	3.437545	2.971656	3.532469	3.043655	2.112734	4.558726	2.137451	1.308978
Map1b	0.986958	1.338877	1.749599	1.572148	1.02461	0.96029	1.024905	1.523085	1.306223
Tssk6	1.121219	1.449563	1.453601	0.371646	1.344684	1.075271	0.987157	0.948898	1.306093
Mtfr2	2.114673	2.588371	1.970719	1.362058	1.533379	0.933346	1.539868	1.029567	1.305475
Gm24837	0	0	0	0	0	0	0	2.606542	1.304627
Mir6933	0	0	0	0	0	0	0	0	1.304627
Crry-ps	1.602934	1.87161	2.267829	1.127432	1.824493	0.795404	1.495082	1.675043	1.304167
Gm14414	1.367848	0.882722	2.333658	2.000273	1.072831	1.339351	0.914772	1.378932	1.30368
Ccdc173	0.976595	0.681797	0.553468	1.359575	1.736184	1.445	0.538325	1.518697	1.303095
Asf1a	1.416301	1.715215	0.885291	1.71016	1.445217	1.309751	1.359259	1.15582	1.301651
Syngap1	1.331943	2.089795	2.258068	2.374376	1.902792	2.223139	2.770047	2.257908	1.300422
Gm6278	2.297951	1.360776	1.508205	2.285079	0.820704	1.394579	1.707065	1.435793	1.300402
BC049715	1.071738	0.874542	1.802677	1.986141	1.391879	0.315353	1.762241	1.80889	1.300041
Gm5900	1.935932	1.404202	1.056086	2.1876	0.879977	0.976522	1.455187	1.298616	1.299968
4930487H11Rik	1.424665	2.779968	1.610678	1.909445	1.179857	0.114564	2.496785	1.179497	1.298796
Gm34058	2.323271	3.915077	1.842809	1.513617	4.501615	4.148806	1.904413	1.754331	1.298029
Wdr78	1.531438	1.527361	1.906011	0.812192	1.004762	0.535019	0.844169	0.842446	1.297419
H2-Q1	0.340111	1.27202	0.722819	0.338206	1.133712	0.511101	0.659103	0.647638	1.296623
Gm23050	0	0	0	0	0	0	0	0	1.296623
Gm24145	0	0.339205	0	0	0.323918	0	0	1.942913	1.296623
A130010J15Rik	1.832663	1.873479	1.451027	1.230118	2.00722	1.553557	2.162487	1.424981	1.295462
Gm7935	1.216061	0.963124	1.64545	1.422646	1.328481	1.223802	1.515625	1.021596	1.295368
Lrrc39	1.810222	2.538848	1.357825	3.318902	2.047293	2.72031	3.508041	2.262106	1.293977
5031434O11Rik	1.474417	2.058688	2.064423	1.172926	1.348049	2.017972	2.499168	1.235334	1.29283
Gm3226	2.066457	1.24368	0.962083	1.41716	2.578852	1.581513	1.436133	1.356876	1.290373
Gm16166	0.966943	0.578621	0.128941	1.217934	0.184181	0.834585	1.199261	0.306875	1.290216
Ica1	1.317477	1.141079	0.658815	1.585908	1.684008	1.891667	2.042518	0.495145	1.288717
Gm22516	0.901432	1.348548	0	0	0	1.667232	1.397512	0.429126	1.288717
Gm26244	0.676074	0.674274	0	0	0.321943	0	0.698756	0.321844	1.288717
Gm22068	1.014111	0.337137	3.380763	0	0.965828	2.188242	0	3.218443	1.288717
n-TScga3	0	0	0	0	0.643885	0	0	0	1.288717
Trib3	2.189412	2.264456	1.216481	0.806351	0.746543	0.724892	1.73207	1.38969	1.288089
Sema3f	2.477542	2.519875	2.281566	4.195546	2.990363	2.880955	1.369067	2.358863	1.285866
LOC118567469	0.688159	0.768686	0.495532	0.958026	0.655395	1.043676	0.938845	1.022103	1.285518
Gm2245	1.230085	1.294032	1.449309	2.144779	1.588773	0.592146	1.654502	1.267421	1.2848
Cpne8	4.672637	3.001483	3.801909	4.016434	5.581567	4.321108	2.046357	2.865334	1.283194
AI506816	1.223798	0.61027	1.346335	1.70372	0.34966	1.13173	0.8854	1.514729	1.283027
Gm13470	0.269117	1.342002	0.628012	0.267609	0.256304	0.995483	0.649007	0.939494	1.282461
Tlr12	1.886361	1.637462	2.288352	1.910532	4.241854	3.440439	3.339676	2.261631	1.281815
Ptn	0.523001	0.81967	0.934038	1.151586	1.850086	1.243683	0.849431	0.995896	1.28177
Dnmt3b	1.063963	1.633582	1.960159	1.252898	1.626624	1.294631	1.736303	1.759416	1.280907
Zfp174	1.200824	1.767926	1.887229	1.279391	1.007503	1.242685	1.418414	1.27941	1.280741
Gm3379	2.014573	1.934794	0.820847	1.038742	1.705473	2.69102	0.848288	2.344309	1.280045
Pacsin3	1.128898	1.521477	0.610286	1.881071	1.743487	1.580062	0.630688	1.481511	1.279497
Spcs2-ps	1.412435	1.831277	1.412599	0.702261	2.017781	1.240867	2.773661	0.672388	1.278867
Rragd	1.465721	2.035301	1.680137	1.614473	1.524792	1.178201	0.885631	1.159346	1.278757
Nos1ap	1.296732	0.773757	1.163869	0.980878	1.002772	0.696958	1.076772	1.02357	1.278154
Vipr1	1.658938	2.266467	2.670518	1.367172	2.380753	1.922921	2.477942	1.287377	1.277887
Rps2-ps8	2.273485	2.093015	1.982243	1.97091	2.553876	2.048539	2.22926	2.275585	1.277876
Nfib	1.014984	0.757659	1.15211	1.863797	1.328416	1.330199	0.958935	1.861585	1.275979
Dysf	1.93585	1.438304	1.61123	2.85521	2.289136	2.715357	0.832547	1.70705	1.275428

Gm20305	0.729448	1.697514	0.729533	0.96715	0.347359	0.674571	2.010456	1.041759	1.274586
Dmd	0.943627	1.019541	0.275256	0.899243	0.561687	1.454394	0.731464	1.010728	1.274093
Strip2	1.72182	1.943188	1.676703	1.46436	2.459763	1.215165	2.08399	2.157028	1.273971
Gm6644	1.98427	0.928912	1.3365	1.167785	0.809913	1.273259	1.17191	1.310887	1.273655
Samd11	0.945152	0.975141	0.651905	2.527894	0.713914	1.115164	1.381081	0.837817	1.273565
B3gnt9	1.285934	0.760006	1.190818	1.444493	1.22471	2.026031	1.476753	1.156317	1.271003
Gm12502	0.111098	0.221605	0.222222	0	1.163897	0.20548	0.114826	0.211553	1.270639
Gm30603	0.799587	1.240491	1.066241	2.650362	1.946102	1.232389	0.550942	1.437984	1.27013
Gm30431	1.156007	1.779521	2.613884	1.974195	1.962595	2.161318	1.324659	2.488385	1.269439
Gm16581	3.829055	1.494337	2.4975	2.372856	2.748276	1.744836	3.556032	1.479389	1.269367
Gm26944	0.400739	0.791803	0.657893	0.819544	0.633698	0.608328	0.8987	1.087037	1.268328
Zfp939	1.531917	1.844938	3.006376	2.586797	1.514037	1.790884	2.330159	1.18334	1.267216
Rfx3	0.904664	1.038961	1.089835	1.097235	1.325024	1.216879	1.473357	0.978782	1.267209
Gm11824	0.443061	0.773293	0.443113	0.495652	0.843934	0.768243	0.343445	0.632757	1.266831
Tppp	1.753542	3.519472	2.265709	1.581261	2.012363	1.269094	2.08254	0.995504	1.266437
F2rl2	1.732439	1.480995	1.507623	0.626449	1.842808	1.290007	1.813817	1.156759	1.265181
Gm52824	1.660307	1.393368	1.184625	2.154363	1.0606	0.777062	1.977597	1.339803	1.264006
Ppargc1b	1.486865	2.028959	1.642721	1.958927	1.318943	1.186377	2.313446	1.395199	1.263636
Gm5781	1.12099	2.744196	2.242241	2.736109	2.232294	2.827245	1.263927	1.649452	1.262658
Il5ra	0.358469	0.435236	0.467623	1.115879	1.424983	1.109808	2.28741	0.756791	1.262631
Dock9	1.686812	1.519516	1.36049	3.877775	1.278291	1.601844	1.199762	1.459224	1.261943
Zfp267	1.397458	2.119816	2.100614	1.572695	1.546103	1.423879	1.677859	1.362385	1.260122
Gm6652	0.440334	1.010072	0.836732	0.875735	0.754865	0.325766	1.046747	0.796558	1.259033
Gm28068	0.879969	0.548517	0.880072	0.43752	1.15235	0.508605	0.454746	0.418908	1.258033
Dnajc28	1.707607	2.208921	2.164348	1.664417	1.545796	1.407156	2.306599	1.207284	1.256882
1700109H08Rik	1.860858	0.850623	1.395806	0.616811	1.107663	0.430216	2.083564	0.88586	1.256274
H2-Q9	1.122426	0.778739	1.22017	3.591054	1.022508	1.353892	2.320166	2.323172	1.255818
Kif14	2.60634	2.358887	2.597367	0.959221	1.67839	1.492475	1.342098	0.821276	1.255297
Ehbp1	1.226154	1.596551	1.40797	1.016072	1.211027	0.734941	1.361165	0.853945	1.255199
Gm15470	0.783909	1.344734	0.97216	0.654795	0.656996	0.492956	0.680576	1.015047	1.255187
LOC118568180	1.580182	1.575975	2.897338	3.07719	0.689768	0.669764	3.130295	1.191053	1.255045
Cdyl2	1.384752	1.367391	1.432905	1.43153	1.247005	1.109409	1.367443	1.520751	1.254455
Nccrp1	2.688946	1.198246	2.918132	2.389428	3.705165	3.968064	2.660898	2.01543	1.254138
Pcsk4	1.073442	1.036049	0.311681	0.895263	0.461699	0.864597	1.073667	0.659369	1.254104
Cbx3-ps6	1.090164	0.575609	0.577213	0.542029	0.671816	0.474423	0.795345	1.098999	1.25294
1700092M07Rik	0.291779	0.436504	0.145907	2.031014	1.250493	0.404743	1.507842	0.138901	1.251412
LOC118568796	0.967881	0.890225	0.978749	1.261891	0.891072	0.84534	1.711715	0.808887	1.250468
Gm26803	1.414013	1.948179	2.624247	2.638235	1.568825	1.887308	2.757175	1.970842	1.250425
Stab2	1.625069	1.551282	2.008354	2.019957	1.901457	1.663828	3.347182	1.381451	1.250131
Gspt2	2.445143	1.174978	1.200482	1.613596	0.444575	1.151152	1.585229	1.058189	1.249963
Adam22	1.06922	1.408418	1.432518	1.344084	0.9991	1.548469	1.605512	1.44057	1.249793
Ntn4	0.597839	1.85754	2.02369	1.41763	1.730025	1.488477	0.736723	1.094618	1.249163
Gm9247	0.964739	0.652902	0.516885	0.959335	0.853178	0.509806	0.498553	1.902662	1.247869
Brip1	2.650097	2.084884	2.156541	1.685907	1.011137	1.286251	1.913903	0.955977	1.247201
Amigo3	1.242615	1.087111	1.264562	2.34124	1.079641	1.229772	1.306836	0.871751	1.246655
Prrt2	1.099844	2.579234	1.189158	2.157807	1.840166	1.209525	2.242764	1.386778	1.246566
Exo1	2.364383	2.684003	2.518457	1.720346	0.915371	1.155472	2.046357	0.878488	1.245819
Gm5529	1.802864	1.573306	1.667843	1.389393	1.373622	1.292105	0.978259	1.33029	1.24576
Gm35816	1.244401	0.372326	1.493455	2.412989	0.414804	0.863087	1.414766	0.888594	1.245326
Tmem255b	1.935011	1.543888	1.548189	5.580099	3.040757	3.578875	2.449915	1.934436	1.24486
AA465934	0.725629	1.302655	0.145143	0.721564	1.10573	0.134208	1.949932	0.138174	1.24486
Zfp862-ps	1.731873	2.007603	1.732074	1.226258	1.407617	0.955918	1.405741	0.958269	1.244453
Mybph	1.424598	1.692874	1.758218	1.687885	0.721687	2.410604	1.221773	1.673802	1.242213
Klhl38	0.676685	2.249612	2.656925	1.271023	1.312804	1.460147	3.056584	2.457773	1.242109

Ttc28	1.680264	1.750034	1.925083	2.575015	1.88892	1.568607	1.483835	1.498524	1.241623
A230056P14Rik	1.302104	1.101874	2.091501	1.490994	0.977056	0.656806	1.508915	1.202163	1.241021
Atp1b2	1.431979	1.576548	1.673937	2.699973	2.07227	2.476519	0.864948	1.646686	1.240731
Catspere2	1.233439	1.196907	2.000403	1.325978	1.460454	2.096321	2.239549	1.269572	1.239121
Gm30698	0.696458	0.787218	0.975155	0.600216	1.326598	1.245188	0.911777	1.060954	1.239068
Gm23608	0.43311	0	0.43316	0	0	0	0.895281	0	1.238377
1700030J22Rik	1.453287	0.753697	1.860424	0.905625	1.199549	0.680937	1.321799	0.479673	1.237367
Gm7390	0.842097	1.364765	0.596555	0.593144	0.367585	1.589938	1.450582	0.734946	1.237331
Gm4535	1.77511	0.680917	1.229065	1.222039	1.495527	1.073332	1.411281	1.170055	1.236343
Mastl	2.591981	3.341354	3.171409	1.686318	1.155503	1.41524	2.194452	0.813856	1.235194
Gm8806	1.087021	1.407058	1.503501	0.850946	0.79297	1.15496	1.243011	0.814748	1.234416
Gm15706	2.03499	2.152577	1.295144	0.919814	0.880956	2.224058	2.29447	0.587124	1.234244
Gm7030	1.418326	1.353048	0.740082	4.047182	1.057147	0.855407	0.892294	1.585235	1.234244
Atoh8	0.776839	1.197373	1.082992	0.983166	0.851952	1.763337	0.997545	0.91893	1.233994
Daam2	1.271918	2.012153	1.425591	1.875383	1.148704	2.048262	2.2892	2.317585	1.233151
Slc25a42	1.136026	1.53391	1.101199	1.477251	1.647876	1.228348	1.57154	1.514251	1.232651
Spns3	2.155446	1.332819	1.250304	1.800432	1.929653	2.910201	2.806978	1.764888	1.232599
LOC118567600	0.791972	0.956151	0.833752	0.787536	0.714568	0.61675	2.024817	0.595291	1.231548
Cldn5	1.722652	1.249502	1.448762	3.464936	2.162655	2.643022	0.445112	1.304651	1.23138
Ddr2	1.399368	1.619216	1.270448	2.262924	1.20335	1.375758	1.17952	1.688056	1.230132
Rgma	1.132352	0.894059	1.384147	2.173823	1.16831	1.207147	0.731464	1.152979	1.229125
LOC115490276	1.71901	0.857217	0	1.282035	1.637166	1.987108	3.109194	2.454999	1.228777
Gm25613	0.429752	0.428608	0	0	0	0	0.444171	0	1.228777
Gm527	1.174696	1.511701	0.947445	1.017391	0.902232	1.752132	1.48826	1.262738	1.227937
Tcf7l1	1.522595	1.732694	1.971794	2.271099	1.747559	2.130124	1.291223	1.802782	1.227911
Chst7	2.322542	2.694026	2.297564	1.25518	1.923446	2.544693	2.400463	1.610394	1.227098
Amotl1	1.418271	1.458307	1.675762	1.934518	1.464304	1.688794	1.426937	1.266684	1.226134
Fanci	2.138192	2.920598	2.417368	1.371255	1.578941	1.561807	1.777551	0.855613	1.225686
Gm6983	1.44621	0.72118	1.124961	1.358214	1.300835	1.931814	1.577771	1.606423	1.225215
Zfp808	1.122521	1.282046	0.995901	0.540113	0.982861	0.719953	0.598803	0.982561	1.225165
Srsf3-ps	1.626754	1.024688	0.984729	1.575072	0.652338	0.91054	0.929157	1.630347	1.224032
Cdc25c	4.040785	4.180683	3.739105	1.802535	1.762351	2.514477	3.278632	1.54608	1.223754
Lyrm9	1.601812	1.860158	1.305738	1.603749	1.368814	1.410284	1.734929	1.316167	1.223427
Mansc1	2.403249	3.141504	2.613546	2.459393	1.799952	1.790906	2.411532	1.555039	1.223088
Gm35498	1.07493	0.772886	0.875045	1.715226	1.301514	0.947826	1.02487	0.944102	1.223051
Srpx2	1.177863	2.714924	2.068043	2.342529	1.17164	1.524951	1.163274	1.719542	1.222395
Gm6501	1.333385	1.432131	1.12838	1.733891	1.416429	0.379407	1.325116	1.562478	1.221956
Rtn4rl1	2.026954	1.987001	1.819273	1.050868	1.765453	2.066716	1.772657	1.468012	1.221865
Gm41640	0.449105	0.579648	0.858683	0.551669	1.169948	0.842852	0.901041	0.804879	1.221165
Bcl7a	1.659212	1.698342	1.528399	1.17231	1.095062	1.17098	1.474194	0.859153	1.220712
E2f7	2.478692	3.034846	2.025508	1.262462	1.218725	1.444283	2.09322	1.160792	1.21962
Sln	1.705787	3.402491	2.132481	0.742101	1.523037	1.478867	0.110188	2.030095	1.219325
Gm7926	3.138004	2.202346	1.511066	1.964713	1.217578	3.116885	2.642675	3.208997	1.218473
Trim45	0.978709	1.571692	1.443349	1.072199	0.647739	0.644294	0.565779	0.742303	1.217379
Tceal1	0.89874	0.566114	2.034227	0.940743	1.4416	1.049844	1.124449	1.576268	1.217244
Neurl1a	1.595799	1.909861	1.611944	0.761693	1.595813	1.44623	0.989603	1.109131	1.216751
Gm19142	1.275354	2.504169	1.116065	1.545632	2.201523	1.584829	1.194567	1.290154	1.215526
Ppp1r9a	0.755759	1.350463	1.716402	1.925784	1.589506	1.106595	1.790054	1.259224	1.215515
Rarres2	2.230267	1.986008	1.115263	3.643486	1.289621	1.178561	1.893469	1.137553	1.214653
H4c8	0.764663	3.050509	1.529504	3.675168	2.427522	2.474977	1.053756	1.698746	1.214653
Gm16385	2.184751	1.059204	1.274586	0.995735	1.213761	1.290804	0.87813	1.21339	1.214653
Gm36774	2.428321	2.26727	1.70519	0.205508	0.295239	0.605207	0.391599	0.819858	1.214653
Gm22720	0	0	0.637293	0.63365	0	1.767841	0	0	1.214653
Cdv3-ps	3.881877	0.967886	2.851085	1.749123	1.617463	1.626646	1.692611	2.252206	1.213988

Ccdc17	1.660651	2.346326	1.660844	1.706394	1.370708	1.177384	1.573335	1.475696	1.21344
Pter	1.676034	2.266266	2.014699	1.778824	0.936254	1.698977	1.715609	1.534374	1.213417
Gvin1	1.038652	0.479098	1.077727	1.090931	1.236501	0.678362	0.899055	0.791119	1.212662
Epn2	1.382376	1.236072	1.255407	0.932222	0.99877	2.204101	0.853969	0.922823	1.21152
Rpl27a-ps4	1.011382	0.85694	1.35165	1.148119	1.031423	1.208434	1.017562	1.337883	1.211319
Gm39260	2.722	3.031474	2.994548	3.654116	1.469028	1.426425	3.28221	1.641353	1.210677
Edn1	1.295887	1.369984	0.959068	1.572127	1.012035	1.462043	0.616108	1.159783	1.210394
Exoc3l4	0.804746	0.713425	1.448711	1.511561	1.073004	1.025348	0.462081	0.936463	1.210147
Gm26175	0	0.422065	0.423241	2.104104	0.403043	0.782708	1.749557	0.805839	1.210017
Gm30198	0.923006	0.575343	1.211587	0.458918	0.934002	1.066959	0.775103	2.471604	1.209597
Mfap3l	1.464789	1.293399	1.548938	1.632858	1.084051	0.923193	1.350003	1.065954	1.209338
Zfp39	1.510652	1.776686	1.71037	1.643906	1.506593	1.858278	1.517145	1.723232	1.208876
Slc39a1-ps	1.458896	1.78895	2.344073	1.40316	1.822212	1.371259	1.581998	1.024681	1.208103
Txnrd3	1.844972	1.973076	1.422794	1.237827	1.354896	1.562278	1.677127	1.608447	1.207591
Gm34823	1.265709	1.009872	0	1.006895	0.24109	1.404586	0.784904	3.133206	1.206333
Myo18b	2.062189	1.709464	2.973111	1.058608	0.675924	0.693472	0.719685	1.262189	1.206071
Mybpc3	0.892229	1.022668	0.932288	1.244772	1.116089	1.305391	1.431418	0.633948	1.205754
Gm5048	0.105396	0.105115	0	0.524026	0.301133	0.194933	0.326795	0.401388	1.205416
Sh3yl1	0.717313	0.74947	0.717397	0.441564	0.65063	0.284292	0.529556	0.747996	1.204549
Gm10273	0.874571	0.581495	1.020451	0.483151	0.509014	0.763844	1.004347	1.341535	1.204007
Shcbp1l	1.118139	0.97127	1.298635	1.111876	1.133607	1.567708	1.416606	1.167602	1.203194
Rpl27-ps1	1.577933	1.049155	1.472909	0.418425	0.70131	0.486408	0.761074	1.70266	1.203129
Itgb4	1.580582	1.056506	0.824016	2.758884	2.161908	2.13031	1.129624	1.360786	1.201943
Mir29a	1.259956	0.628301	3.780307	0.626449	1.199968	0.582584	3.906682	0.599801	1.20085
Snora17	1.259956	1.675469	1.680137	3.341062	0.399989	0	1.736303	1.999336	1.20085
Gm23951	1.259956	1.256602	0.420034	0	1.199968	0.388389	0.868152	1.599469	1.20085
Cxcr6	0.585716	0.759404	1.230148	0.553314	0.669396	0.595817	0.635636	0.362479	1.200216
Rgs16	2.279252	1.326024	2.706927	3.753866	2.419465	1.998001	1.472326	2.803009	1.199307
Dscc1	1.803205	1.923874	1.425956	1.584604	1.437783	2.28803	1.473625	0.958229	1.199033
Dnase1l3	1.602343	2.0312	1.752298	1.682715	3.793741	2.31271	2.213294	3.307815	1.198904
Bfsp1	1.048262	1.254565	0.658984	0.684999	0.45639	1.107885	1.114386	0.484766	1.198904
Slc25a18	1.906064	2.103222	1.338456	3.064881	1.35183	1.312625	2.93406	1.583088	1.198215
Gm8445	1.013122	1.697514	1.175359	0.523873	0.694718	0.374762	1.759149	1.813433	1.197339
Mir17hg	1.113976	1.229202	1.137809	1.296286	0.699768	0.482207	0.979872	1.602204	1.197256
Gm13416	0.513844	0.484005	0.799406	0.709675	0.516567	0.633583	1.268699	0.597948	1.19714
Dnase1l2	2.825727	3.086604	2.242901	2.542289	2.947495	2.6961	2.271523	2.60496	1.196964
Gm15760	1.432217	1.87779	1.078311	1.520208	0.827612	1.160771	1.147623	0.903967	1.196318
Rnf8	2.721214	2.501109	2.641485	1.83051	2.108895	1.776348	2.42648	2.08285	1.195071
Lama2	2.202074	2.223242	2.209106	2.081937	1.290604	1.353429	1.008422	1.59986	1.194686
Gm52102	2.294654	1.807758	1.446378	2.300975	1.505908	1.408739	4.105527	2.148017	1.194585
Apc-ps1	0.894884	1.026377	1.073986	1.334807	0.340911	0.331024	1.109889	1.278026	1.194066
Gm36198	0.715907	0.847876	0.268496	0.845378	1.022733	0.496536	0.416208	1.320627	1.194066
9830166K06Rik	0	0	0	0.14058	0.307751	0.522946	0.041747	0	1.193413
Tesmin	0.625958	0.868579	0.353843	0.460073	0.622075	0.553698	0.562572	0.388678	1.193186
Uggt2	0.554064	0.678895	1.108257	0.487994	1.160906	0.673415	1.750681	0.783749	1.191934
Gm24095	0	0.415718	0	0.414493	0	0	0	0.396861	1.191821
Ajuba	1.756673	1.167997	0.949663	0.975708	1.296226	0.863481	1.095908	1.205423	1.191594
Gm8775	1.153624	1.374272	0.865319	0.828506	0.51883	1.185373	1.126088	1.098364	1.191132
Pkia	1.053669	1.516247	1.776392	2.140439	1.662948	2.088001	0.808986	1.433138	1.190742
Gnb1l	1.477879	1.456398	1.495647	1.80201	1.038881	1.464318	1.545646	1.122318	1.19056
Gm572	0.241736	0.562548	0.564116	1.322099	0.959277	0.558874	1.124307	0.115078	1.190377
A630089N07Rik	1.925113	1.842151	1.248867	1.319335	0.991058	1.299127	1.801486	1.362288	1.190143
Adnp	1.901388	1.827784	1.821421	1.879347	1.199968	0.86858	1.823118	1.025114	1.189933
Zfp770	2.314796	2.60694	2.276047	2.495804	1.684403	1.924181	1.948912	1.869611	1.189864

F830208F22Rik	1.129948	1.079984	0.49441	1.006575	0.851952	0.653088	1.800447	0.784453	1.189122
Gm5575	0	0.829357	0.277223	1.516007	1.055972	0.512674	0.143245	0.923693	1.188841
Gm46877	0.742341	0.503448	1.069096	1.240148	1.244316	0.878713	1.166215	0.819867	1.188629
Pdxp	0.692629	1.298677	0.831252	1.873399	1.292919	1.281044	1.288561	0.94961	1.188247
2610528A11Rik	1.173292	0.877627	2.713554	1.53132	3.282453	2.509118	1.440029	1.745452	1.188143
Cyp4f17	0.831052	1.174189	1.212091	0.998562	1.681904	0.576398	2.004178	1.318738	1.188099
Gm10284	1.615597	1.381111	1.061802	1.101632	0.747357	1.70749	1.431257	1.318463	1.187852
Gm14966	1.245799	2.174344	0.622972	0.825881	1.878602	1.536101	1.931393	0.691905	1.187357
Itgb1bp2	2.01515	2.437399	1.715221	2.430216	1.960041	2.577247	1.32942	2.449303	1.185062
Gk5	1.91901	2.339212	1.367297	0.773282	1.290102	1.067104	1.698199	1.397183	1.183462
Wdr54	1.241507	1.714433	1.910233	0.284897	1.773597	0.839001	1.480569	1.227499	1.183266
Vangl1	0.83691	0.40295	0.865869	2.180995	0.687124	1.414457	0.95447	0.87925	1.182722
Ermap	1.578575	1.049582	1.014916	2.429349	1.539214	1.98118	1.903459	1.89659	1.182125
4933416M07Rik	1.458896	0.873007	0.693056	0.181341	1.493645	0.843214	0.829313	0.937583	1.181889
Crppa	1.52714	1.523074	1.959577	1.031492	1.289779	1.572129	1.072104	1.289385	1.180877
Fgf13	1.348122	1.399411	0.990572	0.902833	0.864692	1.628344	1.364915	1.047791	1.179992
Gm46522	1.031726	0.411592	0.412738	0.547171	0.589563	0.826893	0.639804	0.916817	1.179992
Gata2	1.167117	0.75837	1.078825	2.268405	1.498908	1.504497	1.242828	1.212229	1.179782
Kalrn	0.934491	1.05795	0.732524	1.732938	0.962161	1.938584	0.652597	0.7214	1.179514
Gm12345	0.916332	1.964868	1.420479	1.230118	2.356301	1.059243	0.899721	1.526766	1.179016
Gm9833	1.369253	1.232378	1.235811	1.859724	1.463094	1.729502	1.449709	1.653427	1.177701
LOC118567667	1.502924	1.300952	1.176955	1.367614	1.215313	0.98339	1.553347	1.174444	1.175666
Mdfi	0.739722	0.860712	0.986411	2.329332	1.369867	1.178124	1.231758	0.626034	1.175035
Gm34483	0.692976	1.612639	1.617131	0.76566	0.879977	1.495299	2.546578	1.392871	1.174164
H4c2	0.739174	0.982942	1.72494	0.245011	1.994614	0	1.655276	2.580476	1.174164
Gm6788	0.821305	1.556324	0.32856	0.408352	0.469321	0.45571	0.594202	0.938355	1.174164
Ttc23	0.73699	1.061708	0.982768	0.97715	1.871738	1.337849	0.677081	1.14349	1.170696
Prss36	1.16118	1.32353	0.962233	0.758787	1.232283	1.165865	0.754372	0.821271	1.169948
1110006O24Rik	1.15496	1.367863	1.804834	1.363832	1.237467	1.66886	0.522247	1.855634	1.169578
Adtrp	0.076678	0.076474	0.613494	0.152497	1.387515	1.489094	0.317001	0.14601	1.169292
Micu3	2.01593	1.910034	1.545726	0.985613	1.167969	0.994277	1.962023	1.599469	1.168827
Gm7592	0.612952	0.271698	1.362273	1.422209	0.324316	0.566838	0.985469	0.58359	1.168395
Ramp3	1.725345	1.584903	1.135227	0.090299	1.124295	1.427593	0.516198	0.259373	1.168395
Gm34545	0.612575	0.458208	0.153162	1.370574	0.291705	1.132981	0.474845	1.020655	1.167677
Plcxd2	1.171173	0.911837	0.921934	1.194668	1.187375	1.062102	1.257323	0.848893	1.166643
Gm4875	1.561041	0.799482	0.928295	0.797126	0.683087	1.092456	0.784904	0.923894	1.166122
Nrip2	0.699093	0.522924	0.594298	1.737942	1.797683	0.937424	0.939312	0.998407	1.166021
Mir3473g	0.407633	0.813095	1.223041	2.026747	1.5529	1.130898	1.685235	0	1.165531
Ube4bos3	0.647137	0.501989	0.431475	1.787533	1.917459	1.196904	1.932228	1.779954	1.165027
Aunip	1.527821	2.786291	0.698513	1.258817	0.914621	0.242208	1.30838	0.374049	1.164919
Gm45995	0.152722	0.609261	0.15274	0.151866	0.436352	0.282465	0.157846	0.145406	1.164461
Sox17	1.554598	1.301278	1.277139	4.665274	2.53814	3.260369	0.459073	0.951512	1.164169
LOC112841584	0.244221	0	0.244249	0.242853	0.348889	0.564619	0.631035	0.116261	1.163819
4931428F04Rik	0.976404	1.136106	1.736032	1.537314	1.084903	1.881142	1.289485	1.136218	1.16325
Mrgprf	0.697334	1.246064	0.871769	2.687033	1.189906	2.310794	0.690699	1.46618	1.163087
Mir6395	3.046047	1.822763	0	5.452173	2.320818	0	1.888945	0	1.161262
1700029I15Rik	1.827628	2.43035	1.523201	2.574637	1.740613	0.563378	1.731533	4.350203	1.161262
Gm13991	1.879256	1.509815	1.931682	1.315032	1.756639	1.399957	1.510682	1.308793	1.160897
Gm6594	1.170567	1.214149	0.936563	1.396812	1.204022	1.212404	1.113052	1.471133	1.160281
Gm5141	0.879164	1.163477	1.606352	1.445858	0.998327	0.703578	0.733918	0.853148	1.160199
Gm7733	1.014111	1.011411	1.149459	0.672287	0.386331	1.500509	1.607139	1.287377	1.159845
Myrf	3.425528	2.78847	2.753548	1.135447	1.321236	1.233575	1.81986	1.737402	1.159473
Tmigd3	0.405503	0.751072	1.448394	1.497718	0.496539	1.553557	1.137578	0.661849	1.159441
Acaa1b	0.962818	0.404318	0.709527	1.158988	0.820454	0.79666	0.418998	0.337731	1.159139

C2	0.901602	0.899202	1.635655	2.335205	2.116737	1.512427	2.622186	1.457307	1.159065
9130019O22Rik	0.857732	0.898221	1.057992	1.435761	1.307031	0.819644	1.462739	1.27941	1.158117
Plcl1	1.223877	1.309069	1.534458	1.296392	1.461232	1.328638	1.924905	1.460785	1.15801
Gm4482	0.800015	2.009488	0.563039	1.67946	1.495631	1.205647	1.439341	0.761692	1.157848
H3c13	0.331303	0.991263	0.552236	1.207974	0.420706	1.327641	0.684837	0.736011	1.157792
Gm36430	1.071612	0.997509	0.285796	0.390723	0.918532	0.264265	0.775295	0.646177	1.15752
Gm22781	3.237259	2.825061	1.618818	6.840642	1.926956	2.619501	2.509402	3.467461	1.157023
Tnmd	0.04184	0.333829	0.334759	0.208028	2.351032	1.199463	0.562169	0.995896	1.156441
LOC118568041	1.159351	2.417644	0.895966	2.672532	1.154342	1.656931	0.980384	0.702428	1.15519
Sncg	1.817641	1.963869	1.439133	6.702664	3.317945	3.361796	0.704484	3.172717	1.154916
Gm52364	0.880843	1.262841	0.825886	0.985398	0.576747	1.120042	1.706991	1.572467	1.154342
Gm15790	1.245399	0.56781	1.209957	1.273807	0.643885	0.855553	0.772309	1.727796	1.153063
Gm10263	4.663015	1.894689	4.663557	2.404316	2.796188	6.069036	4.997957	3.453059	1.152217
Gm15326	0.818706	0.699879	0.935772	1.628239	1.262414	0.504742	1.813228	0.816606	1.15187
Cyp1b1	1.1081	1.07216	1.042066	0.970323	0.535547	0.734139	0.632466	0.929047	1.150695
Dcun1d4	1.698229	2.687537	2.161634	1.82828	2.00501	1.739197	2.190379	1.603518	1.150384
Gm46591	1.97718	2.023134	1.489477	1.40436	1.662948	0.973582	3.158158	1.393516	1.150235
9130008F23Rik	0.898718	1.792651	1.232671	0.53621	1.00266	1.021073	1.326957	1.24683	1.150235
Gm10791	1.441256	1.40399	1.60903	1.066554	1.596088	1.39482	1.732104	1.404129	1.150028
Gm4654	1.065226	0.877627	0.185278	1.427697	1.235055	1.499046	0.813756	0.573243	1.147679
Anks6	1.646432	2.181229	1.450011	1.39285	1.334007	1.795267	1.574679	1.240014	1.147621
Cdc42bpa	2.023848	1.731021	1.582116	2.286366	1.183333	1.226011	1.21798	0.99394	1.147578
1700056E22Rik	0.22932	0.514596	0.573366	0.627097	1.528812	1.537491	1.244321	0.927923	1.147451
Scd1	1.306582	2.537623	1.570374	1.424631	0.905992	2.077405	1.030582	1.265819	1.146977
Gm12589	1.943571	1.384569	1.110741	0.736261	0.440723	1.797354	1.817466	2.907882	1.146722
Gm4609	1.257471	1.145069	1.366975	1.685358	1.041393	1.516787	1.299659	1.197235	1.146374
Rpl31-ps14	0.481024	0.479744	0.962161	0.717495	0.916245	2.001763	0.372872	0.686974	1.146147
Gm13710	1.335857	1.06584	1.402813	2.324655	2.353673	1.235359	4.072991	2.28936	1.145871
Ghrl	6.00722	2.567669	1.373239	3.413469	3.269263	1.904671	7.627908	3.758504	1.145083
H1f10	0.783023	2.238691	1.200776	0.363364	1.491486	0.917213	0.539529	2.236545	1.144313
Lhx6	1.413964	1.809313	0.773768	3.183495	1.931037	2.195764	0.579046	1.397034	1.144217
S1pr3	0.717085	0.739837	0.692438	1.487607	1.236363	0.971839	1.022266	1.059416	1.143003
Gm16201	0.939289	0.797267	1.598976	1.291741	0.913602	0.757737	1.487187	0.837213	1.142842
Tceal7	0.685125	1.081894	1.37041	1.532897	1.631265	1.267165	1.062167	2.283073	1.142724
Gm41590	0.489303	0.878401	0.648402	0.510891	0.885413	0.916297	4.488259	1.595587	1.142556
4933407K13Rik	0.968852	0.802498	1.10035	1.338998	0.453543	0.820035	1.476578	0.891173	1.142515
Arc	1.469399	1.809243	1.360713	1.136464	1.485825	2.046669	1.593695	1.260838	1.141122
LOC115488070	0	0.397773	0	0	0.759692	0	0	0	1.140376
Polr3g	2.771903	1.752672	2.083699	2.287972	1.639172	1.474356	2.078457	1.759416	1.13963
Zfp273	0.859875	0.857586	1.45718	1.116326	1.000921	0.640566	0.987471	0.90965	1.138246
Tpt1-ps5	1.061016	1.256602	1.724351	0.857246	1.136812	1.717089	0.41123	1.452149	1.137647
4930512J16Rik	0.894643	1.15994	1.073697	1.808918	1.675695	1.07554	1.541097	1.504826	1.136899
Ccdc157	1.293652	1.406181	1.206579	1.705571	1.564301	1.330748	1.427192	1.134809	1.13599
Gm40723	2.067538	1.607688	1.682261	2.683198	1.034612	0.810167	1.448749	0.767381	1.13557
LOC115489488	1.406234	1.024897	2.434149	2.097535	1.287771	2.100712	2.23602	1.493358	1.134071
Zfp105	0.885239	1.296733	0.774674	0.687719	0.579625	1.279126	0.686204	0.869172	1.133737
Il17rc	1.109554	1.185643	2.100471	1.970247	1.320909	0.952789	1.761125	1.810978	1.133039
Gm29733	0.659977	0	0	0	0.377133	0.732391	0.409271	1.131053	1.13223
Gm18069	0.494983	0.296199	1.386113	0.098442	0.377133	0.640842	0.920861	1.131053	1.13223
LOC115487658	0	0	0	0	0	0	0	0	1.13223
B4galt2	0.606013	1.184623	1.260653	1.398075	1.269752	1.569181	0.901936	0.969333	1.132065
Sh3bgrl2	1.393473	1.816168	1.900412	1.905293	1.673992	1.083629	1.734815	0.738743	1.131907
Aldh3b3	0.169535	0.169084	0	0	0	0.07839	0.175223	0	1.131076
Bicc1	1.451633	1.743439	1.216651	1.534991	1.294895	1.058814	1.764478	1.829818	1.130212

Npas2	1.5371	2.569613	1.259104	1.164564	1.073539	1.380848	0.922941	0.850206	1.130137
Gm32131	1.641258	2.182519	1.641449	2.176087	3.647272	2.698283	2.827204	3.212091	1.129747
Spats2	1.562394	1.504503	1.831986	1.473282	1.128833	1.669055	1.475605	1.590142	1.129663
1700113A16Rik	0.676762	0.899947	0.67684	1.009456	0.966811	1.04308	1.748667	0.966515	1.128775
Gm15186	0.148032	0.073819	0.370124	0.147203	0.281969	0	0.152999	0.140941	1.128703
Hoxc6	0.661635	0.972445	1.393078	1.904531	1.061278	1.577953	0.647842	1.359348	1.128437
Xcr1	1.693145	1.216813	1.157947	1.584622	2.110521	2.866737	1.170923	1.469802	1.127229
Gm41368	1.182048	0.864528	1.379217	0.901161	1.801232	0.619435	1.628941	1.425539	1.126597
Mipol1	0.495664	0.53237	0.305059	0.189572	0.290501	0.599412	0.394072	0.617127	1.12652
Gm18271	0.277885	0.762149	0.416876	0.483575	0.396982	1.284897	0.502614	1.124439	1.125609
Gm18285	0.9185	2.35557	0.984222	0.913355	0.812286	0.910072	0.813699	0.936967	1.12553
4930473A02Rik	1.259062	1.334192	1.337909	1.134633	0.824393	0.83687	1.585963	1.048906	1.124998
Zscan12	1.543432	1.564352	1.819704	2.096293	1.744816	1.438922	1.815691	1.290291	1.1242
Klrb1a	1.321077	2.823343	2.312154	0.750673	1.527789	2.225223	0.731464	1.841771	1.1242
Gm30836	1.040765	0.553597	0.069392	0.827948	1.255536	0.834138	0.717121	0.858789	1.1242
Samd14	0.73894	0.842254	0.580663	0.647324	0.754026	0.79724	0.763731	0.921306	1.123486
5430405H02Rik	0.498625	0.361671	0.634688	0.72121	0.3022	0.67071	0.468504	0.47474	1.123281
Slc36a2	0.53724	0.918531	1.22812	0.305275	0.80404	0.875355	0.608147	0.779437	1.121606
LOC118567854	1.237839	0.900673	0.654029	1.424444	0.622817	0.791941	1.432249	1.0303	1.120411
Abhd3	1.121579	0.852262	0.694392	1.540172	0.71212	0.543296	1.104008	0.610202	1.119868
Alms1	1.543711	2.365729	1.724639	1.123218	1.233545	1.162952	2.17144	1.218829	1.119619
Tmem253	1.174727	0.811107	0.677806	1.887006	1.290919	0.668523	0.79386	1.118455	1.119619
Tslp	0.538234	0.634401	0.391488	0.243281	1.071816	1.312228	0.455147	1.304421	1.119239
Gm9830	0.728383	0.565012	0.323764	1.207172	0.770783	1.085222	0.460057	0.077055	1.118456
2210408I21Rik	1.26788	1.865145	2.028844	1.512934	1.388643	1.34837	1.703543	1.840898	1.117772
Gm4951	0.125615	0.229681	0.711901	0.707831	0.757684	1.47142	1.493037	0.657788	1.117407
Gm33023	0.334973	0.501122	0.083753	0.666194	1.116587	1.084205	0.173106	0.398659	1.117407
Gm15740	0.344764	0.034385	0.172402	0.651382	0.19701	0.255062	0.997726	0.459549	1.117209
Etaa1os	1.431499	2.206427	0.520606	2.329332	1.363344	0	0.403507	0.619513	1.116283
B230208H11Rik	0	0.253859	0	0	0.921188	0.423697	0.052615	0.436219	1.115941
Gm15526	1.06407	0.530619	1.596291	0.634866	0.405364	1.968038	2.19954	1.418339	1.115569
3000002C10Rik	1.34369	1.685949	1.647296	2.456817	1.197154	0.561175	0.716785	0.742834	1.11541
Rpl18a-ps1	0.989965	1.256602	0.540044	0.894927	0.771408	1.081942	0.74413	0.685487	1.115075
Fhl2	0.806372	0.804225	1.008082	0.561298	1.382363	1.155846	1.29181	0.998069	1.114389
Smad6	0.846383	0.884326	1.672808	1.462853	1.189936	1.267242	1.29134	1.841918	1.113984
Pear1	1.350564	0.984323	1.181881	1.782056	1.607828	1.681292	0.818735	0.92731	1.11393
Gm8518	1.240062	1.16401	0.729533	1.305652	0.625247	1.349142	1.281666	0.833407	1.112366
Gfra2	2.710864	2.256023	1.867302	1.410322	2.359523	1.460988	1.892615	1.521256	1.112186
Fbln1	1.880467	2.42148	2.59487	1.372862	1.133504	1.144656	0.984079	1.178484	1.11165
Rps18-ps1	0.530001	0.211436	0.318037	2.108127	1.615254	1.078282	1.205121	0.40369	1.111303
1700001K19Rik	1.573951	1.162786	1.690737	0.985455	1.610052	1.50945	2.590762	2.220083	1.111197
Ccn4	0.673568	0.614845	1.039014	0.385983	0.53277	0.327284	0.41298	0.69565	1.109847
Gm46589	0.811117	2.56756	1.093371	0.420821	0.806086	0.945772	1.348617	0.671533	1.109182
Gm5422	1.449361	1.350404	1.277875	1.782589	0.690178	1.322688	2.365243	1.325463	1.108731
Cdh1	1.988866	1.50951	2.089177	1.368237	1.036435	1.064215	0.775695	0.905115	1.108727
Samd4	1.091823	1.974296	2.163489	1.958331	1.574337	1.34939	1.423749	1.845881	1.108681
Zfp335os	1.109818	0.896032	1.532784	0.262762	0.805317	1.075198	0.273108	1.30824	1.108125
F2r	0.63111	0.778506	1.112877	1.403787	1.154673	1.42836	0.875434	1.265008	1.108034
Gm32098	1.594757	1.765737	2.581643	2.096512	2.419829	1.637257	1.955563	1.569835	1.107756
Nim1k	2.013227	1.927014	1.756711	1.545129	1.466985	1.386956	1.45235	1.839603	1.107486
Gm16378	0.663929	0.993242	2.24102	0.247579	0.790398	1.458204	1.972835	1.185235	1.107371
Cacna1f	1.019985	1.167976	0.953986	0.563484	1.106342	1.222731	0.917549	0.962134	1.107155
Msh5	1.478348	2.071199	3.20346	1.995091	2.111944	1.82284	2.437439	1.273482	1.107069
Gm12722	0.677254	1.254409	0.677333	2.693841	0.184288	1.252606	2.799903	2.026552	1.106542

Gm52380	0.986856	0.839489	0.783771	0.490664	0.635795	0.50999	0.899969	0.773774	1.106542
Amer2	1.626344	1.490942	0.909106	0.451954	1.136912	0.653249	0.481068	0.828962	1.106433
Efr3b	2.132564	2.812979	1.75441	1.693074	0.827154	0.946304	1.137606	1.260819	1.106414
Nphp4	2.14147	2.59486	1.841478	0.995082	1.258017	1.369598	1.572075	0.762202	1.106343
Tlcd1	1.836658	1.494337	0.966774	1.201559	1.19683	2.279544	1.848323	0.920357	1.105578
Zswim9	1.407898	1.519786	1.424627	1.465895	1.64059	0.964997	1.660564	1.671629	1.105055
Gm7809	1.291096	1.05115	1.396653	1.048052	0.301133	1.047765	1.443343	0.551908	1.104965
Tmem240	1.227411	0.816096	0.545579	0.949305	2.013231	1.008952	0.845727	1.298462	1.104841
Eya4	1.255566	1.145196	1.405968	1.355245	0.858514	0.873312	1.419695	0.776513	1.104615
Gm12841	1.241151	0.742708	0.82753	0.987359	0.709235	1.683407	1.624869	0.709018	1.104065
Pisd-ps2	1.157293	1.489306	1.530791	2.004637	2.168832	1.519034	1.543381	1.350663	1.103003
Gm17160	1.512756	1.153734	0.978956	4.158898	2.033975	1.728114	1.471538	2.033354	1.102546
1700049J03Rik	0.231233	0.80716	0.520334	0.574844	0.715727	0.427674	1.374196	0.275195	1.101927
Gm42162	1.101054	1.317747	1.321418	0.930653	0.891337	0.560021	1.87769	0.891065	1.101872
Lysmd2	1.385952	0.645055	1.570928	1.699765	1.011973	2.520646	1.814437	1.803401	1.100779
Spef1l	0.668661	0.666881	0.851122	0.060447	1.273651	0.449714	1.068055	0.983884	1.100779
Prrg1	1.044963	0.786207	0.953411	0.82035	0.593635	0.28821	1.061074	1.134544	1.100779
Gm22890	0	0.383962	0	0	1.099971	1.780118	0	0	1.100779
LOC115488700	0.384987	1.151885	1.540125	1.91415	0	0	0	0	1.100779
Trpm1	1.273981	1.191177	1.134771	1.0689	0.663537	0.533844	1.316723	0.530668	1.100384
Arhgap42	0.520545	1.854141	1.710562	0.924338	1.416462	1.547305	0.768585	1.380628	1.098564
Gm6209	1.357667	1.31302	0.966936	0.22501	0.979566	0.36144	0.382695	0.509219	1.09792
Pnmal2	0.414415	0.759961	1.751442	0.451974	0.407416	0.469776	0.621753	1.018229	1.095736
2700038G22Rik	1.148975	1.227768	1.06703	0.32644	0.625298	0.910746	1.017877	0.820453	1.095076
Tsku	1.987819	2.841622	3.202969	1.427605	1.409365	1.838273	1.689264	1.724368	1.094639
Pdgfrl	0.430587	0.858881	1.327797	0.749306	0.546782	1.161397	0.927152	0.751595	1.094367
1700099I09Rik	0.573215	1.061708	0.409487	0.162858	1.637771	0.378637	0.592446	0.623722	1.092649
Gm40378	1.35469	1.299118	0.885861	0.725362	0.645096	0.289102	0.807773	0.595291	1.092503
9930012K11Rik	1.09031	1.198936	0.894717	0.778402	0.745517	1.059991	0.837944	0.798524	1.092453
Mrln	0.716254	0.857217	0	0.569794	0	1.589686	1.184455	0.545555	1.092246
Abcb1a	1.713365	1.367043	1.606466	1.799604	0.795498	1.158641	1.217458	0.672908	1.092061
Gm12922	0.891083	0.317397	1.145811	1.898767	0.36371	0.824045	0.920978	0.545399	1.091932
LOC105242404	0.381673	0.190329	0.095429	0	0.545252	0.529439	0	0.090848	1.091306
Gm26799	1.962409	1.141691	2.12619	2.764507	1.245985	1.512312	1.521186	1.089903	1.091038
Grhl1	0.822629	0.713425	0.930037	0.586841	0.834559	0.512674	1.312309	0.664038	1.090837
Fndc5	1.023287	1.320728	0.822738	1.137267	0.879021	2.041047	0.870979	1.337232	1.090022
Gm31090	1.183876	1.221439	1.67395	1.623786	1.866225	1.547837	2.742541	1.515844	1.089431
Gm8680	0.253721	0.126523	0.507501	0.5046	0.604103	1.290483	0.655584	0.724702	1.088184
Sdr39u1	1.232972	1.68513	1.415799	1.135246	1.870132	0.802373	1.887908	0.217391	1.088085
Gm6225	0.684823	0.813095	0.945818	0.648559	1.335494	1.357078	0.471866	0.869358	1.087829
Gm10863	0.815266	1.138333	0.81536	1.540328	1.319965	0.980112	1.011141	1.474804	1.087829
Gm17655	0.589767	1.333245	1.533571	1.133829	1.385495	1.854352	0.975285	1.235334	1.086727
Xkr5	1.433743	1.136608	1.654511	1.242928	0.840296	2.379786	0.835913	0.525025	1.08618
Gm45095	1.282047	1.044636	1.064307	1.641494	1.181105	0.728406	1.247115	1.388173	1.086138
Gm8692	1.139138	0.757404	1.645613	0.62931	0.723269	1.638684	1.831443	1.084571	1.0857
Csf3	0.203368	1.379219	0.447461	0.040446	0.968426	0.150455	1.219106	1.936261	1.085434
Zfp831	0.847348	0.536066	0.474317	0.792297	0.800983	0.766058	0.594744	0.51175	1.084833
Ankle1	1.748237	2.142716	1.832702	1.508048	1.644941	2.006284	2.176976	0.902436	1.08405
Gm17944	0.087442	0.784881	0.874519	0.173904	0.333114	0.889497	0.723003	0.083253	1.083417
Cobl	0.649348	1.335714	1.461202	1.775704	0.618432	1.951613	1.090592	1.429686	1.083051
R3hdml	0.162337	1.780952	1.54238	0.080714	0.309216	1.501241	0.083892	1.159205	1.083051
Gm10634	1.006194	1.262487	0.616771	0.74235	0.803726	0.690369	1.576702	1.236123	1.082734
Gm51716	1.135735	0.63715	1.561817	0.564687	1.149265	0.459503	2.494407	0.810998	1.082456
D630024D03Rik	2.175897	1.698342	0.756922	1.31704	0.1802	0.612409	0.195556	0.270217	1.081995

Gm50596	0.41795	0.774126	0.388141	0.118745	0.597076	0.773014	1.14164	0.4832	1.081218
Col23a1	0.915971	0.435016	0.654341	0.737347	0.996984	1.573114	1.172107	0.539868	1.080859
Fam177a2	0.545339	0.85877	2.396902	1.926538	0.505708	1.327139	1.928227	1.243387	1.080547
Mapk8ip1	2.09735	3.222451	2.39995	1.803764	1.421639	1.747355	1.386557	1.313265	1.080519
Cers4	1.859019	1.266194	1.209259	1.232404	0.993213	1.076224	0.718568	1.079249	1.080372
Atp9a	1.304173	0.959534	0.876677	2.848858	1.425338	1.36423	1.149054	0.52925	1.079978
Gm5931	0.883565	0.474499	0.770377	0.608273	0.906229	0.75424	0.655637	0.582397	1.079636
Fam131a	0.737862	1.373676	1.820272	1.491916	1.569435	1.228234	1.499815	1.849962	1.078314
Gm19246	0	0.161197	0	0.482165	1.69325	0.896806	0.334099	0.923308	1.078314
Cap2	1.388928	2.810038	2.480517	1.775761	1.681845	2.348685	0.758778	1.360178	1.077928
Tcf15	1.903453	0.949193	1.487245	4.02218	1.472922	3.96057	0.307393	1.868907	1.077157
Mospd4	0.439486	0.626166	0.690702	0.374592	0.478357	0.522544	0.778682	0.358658	1.077093
Gm3507	0.161392	0.482886	0.322821	0.962926	0.230562	1.194	0.667226	0.845134	1.076745
Zdhhc15	2.717552	1.751282	1.379841	1.829269	1.075085	1.469201	1.080279	1.154368	1.075875
Tenm4	1.617195	1.944512	1.677846	1.613146	1.943242	1.327808	1.666245	1.448592	1.07557
Nos3	1.507872	0.93483	0.951021	3.687776	2.328779	2.173305	0.814331	1.733117	1.074615
Adgra3	0.879576	1.383806	1.300933	1.749298	0.589929	0.836317	0.460945	0.648723	1.074459
Gm6363	1.614405	1.124037	1.279489	1.029855	0.841296	1.295769	0.850025	1.160054	1.074167
Asgr2	1.532434	0.943984	1.126921	1.479031	3.09065	2.334125	2.096268	1.459028	1.073931
Gm19774	1.001139	0.873664	2.252824	1.742179	2.2645	1.851644	0.776045	0.595739	1.073446
Appbp2os	0.606354	0.518348	0.346528	0.086137	0.907476	0.160211	0.716225	0.494836	1.07326
Rsad1	1.796286	1.326534	1.714213	1.36353	1.018623	1.420219	1.332182	1.462191	1.071646
Msantd1	1.255451	1.779312	1.784269	1.401733	2.09768	1.568371	1.957739	1.488898	1.070603
Palmd	1.648094	0.78612	1.266074	2.303908	1.615123	3.158654	0.666543	0.955133	1.069952
Gm16062	1.036225	0.344489	0	0	1.151371	0.479134	0.624745	0.57551	1.069916
Msc	0.85797	0.98733	1.320107	0.853164	0.377133	1.525815	0.409271	0.816872	1.069328
Gm3604	1.338228	1.393548	1.12188	0.880631	1.012114	0.800769	1.118704	1.573918	1.069127
Cox7c-ps1	2.403384	1.118593	1.922931	1.274625	2.13636	1.037201	2.484018	2.135707	1.068965
Cxadr	0.639978	0.75795	0.760062	1.233011	0.419037	0.924736	0.496087	0.837817	1.067422
Gm23136	1.679941	1.675469	0.560046	4.454749	5.866512	4.660671	2.893839	3.732094	1.067422
Far1os	1.055372	1.114478	1.428022	1.543324	1.478124	0.975975	2.438213	1.241245	1.065032
Gm30274	1.536067	2.228331	0.698294	1.805183	1.32994	0.774822	0.144327	0	1.064733
LOC118568450	1.153257	1.204526	1.216964	1.72471	1.297263	0.948929	1.623678	1.080722	1.064537
Trim29	1.312491	2.871979	2.174679	0.720749	0.541046	0.942016	1.316032	0.988506	1.064216
Gm52508	0.167487	0.556802	0.893366	1.332387	0.478537	0.929318	0.230808	1.1694	1.064197
Npas4	2.486088	2.698742	2.790831	2.724423	0.789241	1.360666	1.99267	1.062734	1.06384
LOC118568045	0.939628	0.468563	0.528602	0.992763	0.447446	0.380161	2.488579	0.782791	1.063465
Rps6kl1	0.875108	1.221889	1.225293	1.240043	0.562574	1.355531	1.130584	1.41642	1.06342
Gm9234	1.114706	1.259971	0.891869	1.18236	0.707756	1.992968	0.921684	0.919802	1.062414
Ptk7	0.412793	0.699879	0.853204	0.752547	0.183465	0.63623	0.469306	0.903944	1.062256
Etohd2	0.671977	0.793643	1.184938	0.703381	1.364174	0.948487	1.224551	0.740807	1.061804
Gm32856	1.132159	1.336155	1.132291	1.895126	0.988401	1.256382	1.345665	1.203685	1.061064
Gm14270	0.70815	0.857608	1.163525	1.106574	0.86713	1.075867	1.045584	1.252139	1.060605
Il1bos	1.191362	2.05953	0.754617	0.631834	0.642963	0.257071	0.246266	1.096484	1.059776
Mast4	1.547429	1.240363	1.134913	2.085876	1.108045	1.298512	1.374252	1.222296	1.059698
1700037C18Rik	1.364474	0.604818	1.768968	0.100506	1.54016	1.916101	0.67901	0.866075	1.059638
LOC118567625	0.849876	0.912814	0.915357	0.260035	0.124525	0.604568	1.081094	0.311217	1.05924
Gm8909	0.676074	0.674274	0.772746	0.864369	0.965828	0.759186	1.098045	0.689666	1.058589
Tubb4b-ps1	0.39394	0.214304	0.931238	0.534181	1.023229	0.496777	0.481185	0.579652	1.058113
2500004C02Rik	1.051512	0.873927	0.99321	0.755172	0.584178	0.999417	0.271698	1.112381	1.057862
Gm12359	1.0684	0.7893	0.910224	1.298507	0.942159	0.512308	2.085795	0.489773	1.055994
Ltbp1	0.943801	0.736661	1.241448	1.877029	0.615528	0.901259	0.848231	1.123239	1.055966
Abca13	0.142443	0.138118	0.14246	0.24001	0.380605	0.541544	0.241281	0.2298	1.055918
Gm8741	0.923198	0.46037	0.923306	1.285238	0.571509	0.811058	0.620212	1.230565	1.055868

2810001G20Rik	0.615295	2.27053	1.107659	0.428294	1.758	1.991519	2.289376	0.29291	1.055575
Azin2	0.392671	0.60524	1.035345	0.319477	0.135991	0.660237	0.922376	0.849686	1.054707
Rpl5-ps1	0.940566	0.993242	0.885341	0.825262	1.001171	0.665146	0.514653	1.738345	1.054639
Gm9845	0.979806	0.346748	0.94831	0.78574	0.451527	0.409204	0.88201	0.752316	1.054338
Ebf3	1.164258	1.528858	1.513711	2.836453	1.145787	2.261005	0.701936	0.591193	1.054159
Six2	0.810929	1.396967	2.949176	0.659771	0.140422	0.272699	0.609553	0.350947	1.053937
L3hypdh	0.45521	0.61614	0.975563	0.387994	1.114809	0.571308	0.235241	0.216702	1.053649
Ccdc8	0.414175	0.458969	1.165961	1.02201	0.978834	0.425573	0.539052	0.715645	1.052654
H3c1	2.319122	1.101404	1.325367	1.207974	3.786354	3.370167	2.05451	1.68231	1.052538
Orc1	1.508268	1.430875	1.342883	1.207172	0.665676	0.64637	1.064595	0.612935	1.051839
Gm5127	1.195182	0.641846	1.471164	1.401805	0.933972	1.020246	0.791846	0.671087	1.051491
Trim6	1.058483	1.363568	2.381864	3.026093	1.638143	1.18278	2.415906	1.931578	1.050863
Bmpr1a	0.687792	1.170169	1.355512	1.066141	0.597247	0.682817	0.606328	1.088199	1.050771
Sfrp4	0.440985	0.12566	0.630051	1.754058	0.659983	2.679886	0.716225	0.20993	1.050744
Padi3	1.138396	0.919105	1.174672	1.024208	0.32698	0.91907	0.933801	0.722576	1.050548
Pcna-ps2	1.586463	1.274582	1.674794	1.358468	0.965316	0.774309	1.32086	0.797192	1.050028
Gm41399	0.367139	0.70617	0.340955	0.234696	0.324684	0.291016	0.542081	0.274649	1.04975
Ddit4l	0.346361	0.690877	0.794684	0.709101	0.834377	1.130483	0.315866	0.562547	1.048592
Kcng2	0.60146	0.765338	0.912667	0.989944	0.750597	0.824728	1.1361	1.026819	1.047655
Itpa-ps1	1.099203	1.191605	1.43391	0.950475	1.183417	1.237489	1.43245	1.683579	1.047638
Gm33074	0.274718	0.164392	0.16485	0.819537	0.68026	0.457291	1.022165	0.62774	1.047322
Inka2	1.010399	1.286954	1.180966	1.44053	1.066638	0.889355	0.830406	0.799734	1.044217
Nxpe1-ps	0.810948	0.743714	1.067406	1.561831	1.260597	1.099049	1.2765	1.242462	1.043866
Spic	1.138538	1.222854	1.401441	1.611152	1.793318	1.700814	1.538809	1.876154	1.043393
Dnd1	0.679296	0.602211	0.452916	0.525381	0.898547	0.628191	0.390048	1.077927	1.04308
LOC118567734	1.661521	0.970008	1.337477	1.208937	1.813987	1.536522	0.544499	1.234678	1.042843
Lad1	1.167312	1.145725	0.889484	0.736999	1.535254	1.079494	1.397979	0.987907	1.041914
Acy3	2.357843	1.032395	3.048298	1.143725	1.533569	2.605915	3.506828	2.244898	1.041401
Ppp1r16b	0.995893	0.653449	0.637795	0.94688	0.940158	1.373378	0.668146	0.598856	1.040762
Gm10689	1.855211	0.761877	1.527998	0.86815	3.118028	1.816561	1.579079	1.454635	1.040106
Slc5a11	0.12465	0.466193	0.747987	0.15494	0.831007	0.115272	0.515328	0.652734	1.039522
Armcx6	0.872182	1.141691	0.872283	1.328048	0.622992	0.403283	0.732423	0.882303	1.039084
Dnmt3l	0.494983	0.740497	1.138593	1.033641	0.942832	0.732391	0.46043	0.424145	1.037877
Gm14698	0.642798	0.758838	0.91839	0.352211	0.324838	0.630834	0.637247	0.674457	1.037744
Synpo2l	1.028031	0.799126	1.572465	0.871938	1.123068	1.481959	1.281275	1.237876	1.03744
Dsel	1.711984	2.028824	1.45032	1.562197	1.275607	1.313116	1.228186	1.102631	1.036592
Synpo2	1.720461	1.813677	1.203571	1.577858	1.129155	1.821851	0.847631	1.349479	1.036527
Gm25321	0	2.168254	5.435736	0	1.035267	6.534078	2.808726	0.517475	1.036027
Gm51948	0.815266	1.084127	1.087147	1.080932	0.517633	2.010486	1.685235	0.776213	1.036027
Zfp953	1.612613	1.632147	1.517227	0.831486	1.410693	1.082569	1.06176	1.273785	1.036027
Olfr99	0.30195	0.421605	0.120794	1.441242	0.517633	1.116936	1.061074	0.862459	1.036027
Fam227a	0.374835	0.897208	0.749757	1.006385	0.892471	0.450626	0.581116	0.678071	1.036027
Tanc1	0.977328	0.966323	1.019572	1.399133	0.890676	0.7246	0.426687	0.866338	1.03587
Fap	1.37365	1.962975	1.537847	2.752299	2.011189	1.782224	1.271405	1.952014	1.035644
Jazf1	1.722188	2.1879	1.865921	1.529055	1.679246	1.535746	1.271405	1.112648	1.035644
Mst1	0.434468	0.649966	0.738681	0.216017	0.70343	0.241069	0.987897	0.496387	1.035215
Mfsd4b3-ps	1.14785	1.268556	1.551329	1.233968	1.477297	0.717227	0.961914	0.827034	1.034868
Mirt2	1.141876	1.024952	0.913607	0.113548	0.761257	0.580784	0.236037	0.543589	1.033893
Carf	1.239581	1.136937	1.815312	1.551803	1.148941	1.084916	1.692974	0.790314	1.033752
Itih5	1.048904	1.213489	1.077	2.21122	1.298654	2.541383	1.084094	1.205049	1.033022
LOC102632541	0.62264	1.379962	0.622713	1.628142	0.680847	0.938338	1.596908	0.834332	1.033011
Cd3g	0.541917	0.432379	0.921365	3.556615	1.445123	1.503442	0.616108	1.289894	1.032989
Gm5611	1.08084	1.302538	1.261126	1.164351	0.729144	0.999527	0.884372	0.814677	1.030136
Nudt15	0.888542	1.233697	1.080314	0.623693	1.045353	0.612244	0.684263	1.293851	1.029516

Gm38877	1.079962	1.675469	1.440117	2.386473	1.028544	2.330336	2.97652	2.741946	1.0293
Gm4130	1.48927	1.745233	1.377735	1.036649	0.851018	0.826338	1.115945	1.311586	1.02907
Gm27219	1.079612	0.753716	0.917777	0.268391	0.257053	0.848632	0.725291	1.747424	1.028966
Khdc3	0.629467	0.896845	0.31477	0.715361	0.984889	0.748429	0.929408	0.727739	1.028465
Gm21927	0.856286	0.877729	0.903963	1.107367	1.029694	1.167804	0.750922	1.01291	1.02839
Gm10651	2.217523	1.852978	1.43856	0.953557	1.769467	1.496453	2.106089	1.255367	1.028187
Apol8	1.427181	1.36012	0.919846	1.293037	1.208206	0.938533	2.523997	2.385478	1.02773
Gm46775	0.965432	0.70363	0.70559	0.73848	0.848738	0.618093	2.264288	1.060599	1.026312
Gm26446	0.538234	0	0	0.535219	0.512608	0.497742	2.225165	0	1.025969
Rpl29-ps6	0.089706	0.089467	0.89716	0	0.512608	0	0.370861	0	1.025969
Taf4b	1.337736	0.949474	0.599178	0.914032	0.750358	0.402246	0.950019	1.03924	1.024678
Gm9001	1.612092	0.625256	1.522709	1.959298	1.108856	1.656458	2.962094	2.472846	1.024311
Arvcf	1.763501	1.966665	2.501256	1.960869	1.893299	1.764262	2.137491	1.740081	1.023744
Gm13889	2.361807	0.770897	0.429469	3.544217	3.558078	2.620951	1.065184	2.821061	1.023187
Nr2f2	1.510986	0.949387	1.057813	2.674489	1.88515	2.333511	0.905774	1.82703	1.022474
AW146154	1.007669	1.053615	1.885536	1.34142	1.253793	0.946891	1.159063	1.547419	1.02236
A430033K04Rik	1.037047	1.367361	1.599699	1.957604	1.288996	0.942774	1.380675	0.769814	1.021903
E430024I08Rik	1.173426	1.549177	1.528164	1.057723	1.037159	0.991466	1.448374	1.133292	1.021829
Apof	0.405005	0.356406	0.476532	1.042377	0.340343	0.352505	0.566332	0.567066	1.02178
Gm20957	0	0.089035	0.446413	0.266317	0.170044	0.082556	1.845346	1.784914	1.021013
Wfikkn1	1.071161	1.504354	1.530408	1.521659	1.14508	1.536404	1.333037	1.39449	1.020912
Carmil2	0.574488	0.846416	0.652903	1.168506	0.895313	1.424765	0.79618	0.758297	1.020411
Amt	1.461711	2.453404	1.533192	2.091655	1.731658	1.318775	1.805532	1.697186	1.019371
Gm10059	1.247802	0.711131	0	3.190661	1.358163	1.318775	1.105428	1.697186	1.019371
Gm12138	1.027392	1.557478	1.150813	1.838946	1.487285	1.406148	0.679591	1.21294	1.018363
9130230L23Rik	0.838815	0.342238	0.228794	0.34123	0.399439	0.352595	1.182215	0.217809	1.017501
Chrm3	0.647477	1.047168	0.945077	1.235497	0.499987	0.695864	0.108519	0.899701	1.017387
Mak	0.999037	0.624846	1.049957	1.397552	1.128864	0.782947	1.050055	1.434832	1.016725
Gm6736	1.927211	1.30865	2.296518	1.182469	1.210619	0.872152	1.398546	1.717773	1.016104
Gm6543	0.815266	0.813095	0.25088	0.685976	0.836177	1.101901	0.3889	0.716504	1.016104
Gm46610	0.266529	0.26582	0.26656	0.265036	0	0.246478	0.137736	0.253762	1.016104
Pcdh1	1.903819	1.076903	1.534599	3.263572	2.056737	3.021911	1.248163	1.609717	1.015583
Cfap45	1.825622	2.093876	1.338945	1.966679	0.956286	0.900415	1.47805	1.361568	1.014988
Cmtm8	2.060647	2.121457	1.130164	1.916904	2.152461	2.458867	1.099242	1.51892	1.013667
Gm13717	0.132945	0	0	0.528801	1.89923	0.860604	0.137405	0.75946	1.013667
Gm33721	1.062286	1.19189	1.394413	2.64084	1.327869	0.30699	1.166546	0.189638	1.012453
Mterf1a	1.103042	2.077976	2.002049	1.787481	1.906508	1.246738	2.026753	3.422887	1.01236
Gm16364	1.132834	0.353068	0.637293	0.492839	0.809174	0.523805	0.731775	0.808927	1.012211
Gm33614	0.589767	0.823475	0.235934	2.815023	0.44935	0.10908	1.95057	1.123031	1.01178
AV051173	1.503105	3.350938	1.326424	2.110144	3.873582	1.471791	4.112297	3.114755	1.011242
Eif1-ps1	0.751553	0.485004	0.574784	0.747343	0.631562	0.53148	0.41123	0.9681	1.011242
Cd247	1.223798	0.366162	1.142344	2.271627	0.815872	2.263461	1.011885	0.737945	1.01087
Ttc30b	0.551411	1.2691	1.102951	1.307539	1.373491	0.607991	0.986385	0.848073	1.010662
LOC118568352	0.87304	0.995104	0.498938	0	1.069038	0.692023	0.515618	0.237491	1.010389
Cacng1	1.474662	2.160143	1.797453	2.199602	1.185006	1.747267	1.619395	2.106034	1.010191
Cd5	0.896792	0.623373	1.44047	0.891769	0.983503	1.030374	1.011141	1.009077	1.010127
Kif24	1.61422	1.945735	1.634216	1.743046	1.131803	0.870025	1.780967	1.112599	1.009932
LOC118568514	0.706217	0.528253	0.882874	0	0.168148	0.81636	0.364955	0.336194	1.009632
Drp2	0.47074	0.796451	1.219022	1.053232	0.52038	0.862878	0.582103	0.912388	1.009479
Abhd6	0.820716	1.240999	1.694579	1.790197	1.437211	0.97932	1.778591	0.983055	1.00931
Myct1	1.75677	1.154788	1.377627	3.335047	1.635103	1.624606	0.288863	1.007372	1.00842
Tmod1	1.376971	1.67185	1.057797	0.555641	0.969305	1.051923	1.072533	1.881017	1.008057
Rnps1-ps	1.501092	1.054772	1.978942	1.39091	0.909761	0.820278	2.045098	1.461668	1.007975
Vash1	1.379457	1.670596	1.513996	1.059973	0.759263	1.043737	0.925804	0.776087	1.007402

Gm33804	1.249038	0.766592	0.480455	1.528666	0.183011	2.132439	1.48955	1.189206	1.007299
Gm31544	1.378485	1.443556	1.999035	1.964761	1.9474	1.572228	1.733425	1.443698	1.007261
Tmem95	2.493754	0.860924	3.069592	2.289032	2.831759	3.015729	3.96526	1.187149	1.005556
C130046K22Rik	1.271745	1.41856	1.171481	0.948466	0.876524	0.80468	0.91663	1.035575	1.004756
4930526I15Rik	0.733065	0.868197	1.099726	0.546719	0.785434	0.635546	1.041782	0.872438	1.004347
Kifc2	1.071706	2.029008	2.506993	1.625648	1.885664	0.99108	2.102679	1.729439	1.004118
Pomgnt2	0.912657	0.886888	0.748934	2.071064	0.690906	0.476101	0.532105	0.646134	1.003665
LOC118568191	1.578638	1.91402	0.866804	1.200432	0.795959	0.658375	1.183711	0.707303	1.003055
4930549G23Rik	0.573497	0.667299	0.191188	1.330664	0.819289	1.149097	0.493948	0.182009	1.002089
E430018J23Rik	0.794535	2.121641	1.743054	1.42725	1.318134	1.493225	1.589404	1.537353	1.001541
Entpd4b	0.589767	0.312479	0.129026	0.60479	0.684556	0.068175	0.247631	0.087737	1.001241
C030037D09Rik	0.157494	0.680659	0.997581	0.678653	0	0.679681	0.976671	0.599801	1.000708
LOC102634078	0.968821	1.422522	1.157337	1.311286	1.358411	2.015853	2.197351	2.075427	1.00032
LOC118567623	0.569143	0.922395	1.191781	1.043479	0.728373	0.98686	0.863974	1.286964	1.00013
Fah	1.783811	1.95348	2.028884	1.35645	1.032654	1.681958	0.433802	0.79923	1.000077
Gm3435	1.731016	1.362953	1.6401	1.585426	1.388295	1.348033	1.03579	1.864952	0.99857
Gm35399	0.805786	0.763459	0.40294	0.360572	1.151132	1.005973	1.540717	0.805546	0.998381
Amer1	1.361568	1.299186	1.440287	1.672898	0.972557	1.023047	1.684638	0.810216	0.998227
Gm2629	0.475863	0.569516	2.474776	1.514232	1.94879	2.420349	1.770582	1.268592	0.997788
L2hgdh	0.930057	1.407936	0.697624	1.123029	0.284714	0.706501	0.995591	0.917131	0.997231
Kcnd1	1.060807	1.405813	1.453329	1.459471	1.480852	1.23633	1.892411	0.857802	0.997193
Gm25600	0	1.564825	2.092246	5.720781	0.4981	0.483655	1.081094	0	0.996932
Snord70	2.092002	0	1.046123	0	0.9962	0	0	0	0.996932
Brca2	1.764318	1.893052	2.015404	1.629713	1.202502	1.074837	1.667951	1.218057	0.99618
Eml1	1.028748	1.14001	1.404485	0.860606	1.16639	1.479883	0.877613	1.103845	0.996051
Tln2	1.031228	1.085621	1.693448	1.601477	1.097319	1.165569	0.986876	1.2485	0.994986
Gm13205	2.013218	0.929564	0.633865	2.001941	1.384765	1.275651	1.656903	1.810293	0.994921
Bhlhb9	1.595198	0.510676	0.925716	0.979175	0.693978	1.165582	0.305318	0.975023	0.994807
Me1	0.91767	0.646043	0.647842	0.840959	1.490905	0.682234	0.557916	0.668133	0.994667
Gm14320	0.069558	0	0.069567	1.106701	0.529973	0	0.287568	0.198679	0.99443
Gm10910	0.069558	0.693732	0.069567	0.830025	0.662467	0.128651	0.071892	0.132453	0.99443
Gm16372	1.490271	1.040412	0.931527	1.222586	1.4548	1.102524	1.309229	1.773604	0.994252
Gm52116	1.644689	2.040386	2.407142	2.872056	2.941746	1.743536	3.109515	3.284582	0.994047
Gm35574	1.250483	1.247153	1.320107	4.352173	0.992455	1.284897	1.938654	1.521299	0.993184
Gm6473	0.624459	0.691996	0.416354	1.207424	1.222496	1.15496	1.900372	0.792728	0.991941
LOC118567783	0.86622	1.727827	0.86632	1.378188	0.494987	0.480632	0.895281	0.164945	0.990701
Zfp979	0.757646	0.928345	0.324743	1.119341	1.051436	0.560518	0.447466	1.051115	0.990314
Muc1	1.18637	0.862758	1.310102	0.61444	0.635561	0.685698	0.178817	0.329449	0.989376
Thsd1	1.193611	1.216313	1.012093	1.303038	1.396265	1.36777	1.247066	0.741153	0.989233
Gm5239	0.345768	0.919592	1.267962	1.031492	1.866063	1.385605	1.429472	0.548674	0.988642
Gm19378	1.081718	2.337483	1.262151	1.210116	0.944365	0.583531	1.86335	1.459028	0.988016
Gm24400	0	0.516733	0	0.515211	0.98689	0	0.535495	0	0.987615
Gm24927	0	3.617134	2.590865	0	0.98689	0	0.535495	0.986588	0.987615
Gm25618	0	0	0	0	0	0	0.535495	0	0.987615
Gm41774	1.036225	0.516733	0.777259	0.128803	0.740167	0.119784	0.535495	0.246647	0.987615
Gm24384	0.518113	0	0	0	1.480335	0	0	0	0.987615
Tnfrsf10b	1.357375	1.193448	0.732353	1.740495	1.054611	0.858861	1.015267	0.357098	0.987297
Tek	1.742722	1.349141	1.096946	3.793123	1.810636	2.287817	0.969872	1.810082	0.987289
Fgd5	1.682959	1.311001	1.045747	3.535212	1.545926	1.869459	0.33965	1.867819	0.987083
H3c11	1.721679	1.259203	0.459168	1.141357	1.093139	1.16758	2.372588	2.29489	0.984548
Gm18584	1.588483	1.74268	1.509234	1.737544	1.13464	0.587592	1.559688	0.604957	0.984078
Gm46761	0.90248	0.128582	0.128941	0.512814	1.105087	0	0.666256	1.718499	0.983021
Gm24826	0	0	0	0.512814	0	0	0	0	0.983021
Lpar2	1.13774	1.542043	1.118421	0.97665	1.537374	1.618686	1.587982	1.305443	0.982418

Serpina11	0.379713	0.270501	0.488259	1.780047	2.27313	1.956387	2.578971	0.619755	0.9823
LOC115488029	0.901641	0.668008	0.61834	0.256169	0.343485	0.214408	0.559135	0.392435	0.982108
Eml5	0.663078	0.850259	0.414472	0.800656	0.902155	0.503695	1.162604	0.698956	0.981814
Gm38762	0.34327	2.396491	1.201584	1.024041	1.798095	0.317445	3.193078	0.980479	0.9815
AW551984	0.245084	0.260727	0.212431	0.422433	0.653564	0.423073	0.23642	0.684476	0.981066
Art1	0.447081	1.605207	0.357706	0.666865	0.425795	1.901855	0.277248	0.97903	0.980049
Gm7591	0.370135	0.36915	0.946012	0.081792	0.705026	0.646547	0.510071	0.50903	0.979922
Tceanc	0.671064	0.491714	1.136832	1.525267	1.147796	0.873876	1.033289	1.251758	0.978954
Gm7488	0.461984	0.972703	0.975413	1.225056	1.124415	0.901926	1.273289	0.977453	0.97847
Gm24671	0	0.682598	0	0	0	0	0	0	0.97847
LOC115487515	0	0	0	0	0	0	0	0	0.97847
Gm41949	0.614148	0.956117	0.7865	0.707527	0.577775	0.533314	1.068242	0.713084	0.977942
Cmtr1	1.094858	0.762627	1.094986	0.924553	0.70343	0.554459	1.113629	0.752853	0.977243
Gm33938	0.854427	1.107798	1.42421	1.189497	0.569623	0.724302	0.883093	0.840615	0.977214
Otub2	1.300715	1.552772	1.754199	1.646183	1.182479	1.020609	1.548044	1.013243	0.976731
5033430I15Rik	0.585098	0.510598	0.438875	1.381825	0.975172	0.27054	0.831501	0.835606	0.975889
Gm36884	0	1.604737	0.585166	0.727276	0.278621	0.27054	0	0.557071	0.975889
Glo1-ps	1.023472	0.680498	0.767693	1.356985	0.243686	0.394364	0.705206	0.568427	0.97546
Clec1b	0.481024	1.139391	0.60135	0.95666	1.374367	0.166814	0.683599	1.087708	0.974225
Gm11944	2.169038	1.272508	1.850277	1.839699	1.518947	1.002929	1.978067	0.607393	0.97284
Rpl31-ps13	0.226741	0.565342	0.453534	0.901882	0.323918	1.572619	1.054565	0.431758	0.972467
Glrp1	0.708202	0.593306	1.869871	0.788743	0.863339	0.995483	0.849076	1.105816	0.971971
Abca8b	1.56722	1.506552	1.472981	1.070255	1.043024	1.117545	0.956268	0.898884	0.971805
Gm9242	1.083272	0.826179	0.764752	0.38019	2.245458	0.766064	0.395159	0.303348	0.971722
Meis1	1.109214	0.632149	0.520712	0.967939	0.625218	1.088568	1.146428	0.905212	0.970879
LOC118567810	0.339417	0.112838	1.018368	0	0.862018	0.313882	0.116935	0.107719	0.970483
Got2-ps1	1.112549	0.873504	1.136352	0.87093	0.608694	1.02885	1.418994	1.262091	0.970114
Mir6992	0	1.014504	0	0.505757	0	0.470343	2.102679	0.968486	0.969494
Mir697	0	0.507252	0.508665	3.034543	0	2.351715	1.05134	1.452729	0.969494
D830050J10Rik	1.663142	1.036696	1.039584	1.676796	1.561959	1.602106	1.862185	1.803401	0.968686
Pcyt1b	1.109228	1.711815	2.183682	1.288786	1.056417	1.014982	1.749834	1.27843	0.968166
Srpx	1.244372	1.165076	0.838144	1.540439	1.257685	1.150757	0.603689	0.991334	0.968161
Gm32240	1.269574	0.844129	0.931129	0.757477	2.015214	1.8785	1.924513	0.967007	0.968013
Rbm24	0.550254	1.131877	1.633756	1.504722	1.097241	1.319849	0.995251	1.22788	0.966938
Gm24924	2.704296	0.674274	2.366534	1.344574	0.321943	0.937818	0.698756	1.287377	0.966538
Ggnbp1	0.30377	0.605923	0.607611	1.208275	0.819704	0.608654	0.784904	1.06047	0.965067
Car6	1.820958	1.331814	1.82117	0.643825	1.002017	1.010379	0.961937	1.194348	0.964186
Gp1ba	0.860505	1.265866	1.333938	0.770117	0.840024	0.855451	1.111719	0.798803	0.963662
Efcab9	0.842524	0.084028	1.93803	0.586463	1.043134	0.934967	1.306186	1.283464	0.9636
Gm11805	0.538234	0.939401	1.81675	1.204242	0.704836	0.311088	1.460265	0.576508	0.961846
Rpl5-ps2	0.784765	1.34173	1.233346	0.501666	0.64063	0.881239	0.347612	1.067391	0.961651
Snord42b	10.07965	7.036969	14.11315	12.02782	2.879924	12.11775	14.58495	7.67745	0.96068
Cyp2r1	1.623943	2.457354	2.016164	1.169372	1.279966	1.760698	1.273289	2.665781	0.96068
Mir6481	1.511947	0	0	0	0	0	0.520891	0.479841	0.96068
Mir6539	0	0	0	3.006956	0	0	0	0.479841	0.96068
Rmrp	1.411151	1.005281	1.411315	0.601391	1.343964	1.491415	0.208356	3.07098	0.96068
Gm7180	0.738279	1.472628	1.006861	0.734144	1.342337	1.241341	1.664833	1.341927	0.959517
Klri2	1.062575	0.748887	0.835989	0.633974	0.984998	1.283977	0.307503	0.863296	0.958716
Gm32164	0.695788	0.991336	1.595789	1.056043	1.355216	0.880501	1.557217	0.986216	0.957326
Rhobtb3	0.815819	1.026448	0.99165	0.611557	0.896512	0.812478	0.713468	0.728941	0.956983
Xndc1	1.196378	0.824707	1.038155	0.612334	0.888076	0.82978	1.400173	0.854302	0.955802
Gxylt2	1.001893	1.725935	1.093101	1.751039	0.896362	1.572275	1.161022	0.693745	0.954893
Uchl4	0.524801	1.189554	0.524862	1.091164	0.636128	0.926519	0.739647	0.9539	0.954893
Gm32940	1.412647	1.774975	1.145823	1.327308	1.101737	0.658329	1.666976	1.461473	0.954126

Ampd1	0.333534	1.378104	0.619492	1.350352	1.225236	1.454081	1.477387	0.884622	0.953662
Gm14410	1.43188	1.61668	1.161849	1.114909	1.042078	0.762015	1.438035	1.170372	0.952721
Rny1	1.498326	1.494337	1.998	3.476511	2.853979	5.080552	0.516198	2.853106	0.952025
Gm4335	1.362115	1.449054	1.407682	1.128737	1.340505	2.099401	1.220105	2.074987	0.952025
Prf1	0.863689	0.161511	1.052744	2.683911	1.156737	2.720616	1.227416	0.513948	0.951793
Gm34606	0.434127	0.519566	0.086836	0.04317	0.372112	0.722641	0.493562	0.743997	0.951652
AI225912	0.405842	0.841905	0.754954	0.895923	0.487015	0.390323	1.300321	0.633699	0.951537
Rapgef3	1.647594	0.902607	1.85666	3.299806	1.812258	1.80262	0.575618	1.966362	0.951029
Gm16230	0.570057	1.137079	0.570123	1.41716	1.357291	0.922549	2.209435	1.221188	0.950802
Gm7623	0.83074	0.276176	1.107782	0.385507	0.263729	0.307297	0.286204	0.105459	0.950123
Apold1	0.523319	0.488253	0.607796	1.225429	1.045039	0.921064	0.924724	0.642905	0.949271
A230045G11Rik	0.942645	0.683735	0.916384	0.871815	1.004492	0.780288	0.90614	0.941423	0.948686
LOC115487929	0.745804	0.743818	0.24863	0	0.236765	0	0	0.236693	0.947756
Gm10224	0.764663	1.754043	0.841227	1.064532	0.50978	0.565709	1.027412	1.528872	0.947429
Cyp39a1	0.900212	1.720813	1.650581	1.920885	0.785906	1.04061	1.33747	1.392772	0.947355
Gm12002	0.24823	0.165046	0.331012	1.481038	0.630431	0.612148	0.598636	0.07878	0.946341
Gm35113	1.177336	1.874602	1.19813	1.088584	0.963909	1.126966	1.003356	1.238933	0.944931
Zfp229	0.746381	1.167928	1.389974	0.998131	1.250106	1.118648	1.157132	0.820897	0.944401
Kcnq4	0.528296	0.8068	0.924625	1.214841	0.393081	0.595422	0.887283	1.021698	0.944087
Eid2	0.387378	1.159039	0.559611	1.112823	0.45092	1.154312	0.266916	0.532742	0.943525
Gm18301	0.928093	0	0.68068	1.168999	0.530343	0.572181	0.255795	0.353454	0.943525
Mir6979	2.969896	0.98733	0.99008	0	1.885664	0	0	1.885088	0.943525
Eif3s6-ps2	1.4059	1.440053	1.10205	1.02018	0.868517	1.019023	0.981803	0.651189	0.941585
Frg2f1	0.987373	0.782002	0.668006	0.895209	1.410544	1.101081	1.410689	1.493061	0.941054
Adgrd1	1.632765	2.669848	2.050687	2.322153	0.705187	1.334357	0.706414	1.500324	0.94094
Sec16b	1.529666	1.574806	2.048017	1.643767	1.127874	1.597115	0.764993	1.15102	0.940586
C130026I21Rik	1.315603	0.728945	0.877171	2.14405	1.252966	0.777291	0.755412	0.452322	0.940415
Gm18750	1.027392	0.245918	0.575406	0.980771	1.330728	1.254132	1.614028	0.508652	0.940028
Gm52903	0	0	0	0	0	0	0	0	0.939332
Gm6297	0.265254	0.377925	1.023241	0.527536	0.252625	0.315384	1.135777	0.541174	0.93901
Gm12416	0.895607	1.027206	0.940496	0.623413	0.980911	0.745406	0.786806	0.639529	0.938952
Efs	0.725584	0.96487	0.933002	0.89331	0.707491	0.49526	0.767783	0.871758	0.938527
Gpx4-ps1	0.60588	1.057468	0.530207	0.677797	0.216388	0.420224	0.861036	2.235323	0.938369
Tvp23a	1.126645	1.016631	0.858496	1.547127	1.345513	1.108037	1.349276	0.85133	0.937438
Gm36195	0.86518	0.929252	0.495502	0.647088	0.500027	0.46501	0.634352	0.816696	0.937358
Stap2	0.779386	0.777311	0.508354	1.617433	1.452284	1.128133	1.681115	1.096946	0.936604
Txlnb	1.691626	1.439016	1.492785	1.273981	1.397854	1.196277	0.989891	1.148732	0.936539
Gm2539	0.726076	1.490883	0.085431	0.849423	0.569476	0.750447	1.103583	0.609967	0.936256
2610005L07Rik	0.981817	1.103327	1.701091	1.086324	0.921901	0.690556	0.814664	0.710964	0.935758
Gm7803	0.758204	1.067555	0.669081	0.487854	0.382291	0.371204	0.829738	0.721884	0.935175
Gm40464	0.70086	0.489296	0.280377	0.069693	0.533993	0.972201	0.507062	0.734017	0.935175
Fndc10	0.804972	1.116979	1.260102	1.113687	0.566652	1.585923	0.940498	1.132957	0.933994
LOC118568273	0.55998	0.139622	0	0.139211	0	0.647315	0.578768	0	0.933994
Serpinb1c	2.146718	3.305409	1.958637	2.022341	1.076059	1.427964	2.335516	1.111587	0.933269
Gm10658	0.489303	1.268802	0.391488	0.681187	0.559209	0.678739	0.606863	0.978316	0.932699
LOC118027456	1.291077	0.819407	0.82169	1.167132	0.409869	0.578884	0.970467	0.335245	0.932205
Gm13033	1.416301	1.378899	1.214113	0.536521	1.02771	0.623691	0.975878	1.187927	0.932047
Nscme3l	0.434382	2.382743	1.031778	2.051759	2.275356	0.702981	1.964182	1.085634	0.931511
Gpc3	1.832461	1.705744	1.466139	2.308116	2.303685	2.327255	1.186869	1.860995	0.931466
Gm5539	1.042314	1.136996	0.912131	1.036475	1.147796	0.421706	0.774297	1.178457	0.931329
St6galnac3	0.719975	0.615478	0.360029	1.227329	0.930588	0.9036	0.797282	0.342743	0.931271
Gm15915	1.526378	0.730711	1.09912	0.789271	0.465186	0.734004	0.252414	1.046349	0.931055
Gm18284	1.666014	1.145917	0.517099	1.942317	1.422553	1.275044	1.009395	2.187874	0.930814
B630019K06Rik	0.933872	0.571532	0.424537	0.464321	0.727699	0.608457	0.921331	0.646646	0.930521

Gm11787	0.746061	0.343419	0.57396	0.228271	0.327942	0.477646	0.593147	0.546402	0.929851
Rbpms2	1.687898	1.907858	1.725607	1.230858	1.035967	1.075297	1.783294	1.857029	0.929481
Zfp111	1.40572	0.797374	1.581618	1.843409	0.995726	1.299965	1.570928	1.045612	0.929469
Osmr	1.720966	1.395993	1.216291	2.281768	1.32215	1.156487	0.675908	1.103276	0.929466
Oscp1	0.729885	0.981618	0.895872	0.967728	0.642473	0.859059	1.131558	0.852859	0.927527
Gm9157	0.34327	0.513534	0.514964	1.365388	0	0.581983	0.354786	0.599182	0.926972
Gm9009	0.19452	0.194002	0	0	0.370517	0.179886	0	0.740807	0.926972
Fitm1	3.160942	2.30377	3.708459	2.29698	2.142049	4.497139	2.450244	3.067402	0.926972
Gm51283	0.972598	0.970008	1.945421	2.901449	0	0	1.005228	7.408066	0.926972
Mir7077	0.972598	1.940017	1.945421	0	0	0	1.005228	0	0.926972
Mir7686	0	2.910025	1.945421	0	0	0	0	0	0.926972
Gramd2	0.890246	1.81611	0.323764	0.88526	1.233252	0.935536	0.418234	0.385273	0.925619
5830416I19Rik	1.201509	1.135242	1.496791	1.278623	1.084078	0.877199	1.699332	0.923191	0.924152
Gm10358	1.846397	1.427148	0.78481	1.101632	1.494715	1.792864	1.860635	1.318463	0.923885
Riiad1	1.356876	2.416542	1.163171	1.542029	1.93841	1.523681	2.303941	1.753264	0.923731
Plag1	0.95686	1.061672	1.267987	0.820669	0.831564	0.652595	1.087859	0.842697	0.923372
Gm2546	1.937104	1.103969	0.461269	1.330032	1.449546	0.853035	0.953378	1.668664	0.923116
Pkd1l2	1.703823	1.564542	1.606434	0.783698	0.79348	0.770468	0.682673	0.864701	0.92283
Rasl12	0.597332	0.226949	0.711192	1.187971	0.812703	0.71022	0.205791	0.785373	0.92174
LOC118568220	0	0	0.322352	1.923053	0	0	0.333128	0.306875	0.921583
Gm26698	0.05686	0.62379	0.341197	0.226164	0.920591	1.63004	2.056851	2.544386	0.921267
4930506C21Rik	0.454876	0.680498	0.511795	1.583149	0.270762	0.683565	1.410412	0.974446	0.921267
A230072C01Rik	0.56124	0.590843	1.060244	0.713123	0.504824	0.663189	0.870105	0.564042	0.921237
Gm11539	2.284294	2.015342	1.493751	1.397845	2.008188	2.193692	0.998856	2.425819	0.921096
Gm14393	0.527145	0.087624	0	1.135749	0.083674	0	0.908051	0.501894	0.921096
Gm36266	1.433491	1.488029	1.170333	1.280006	1.727448	1.650296	1.783948	1.25341	0.920124
Gm51770	0.608636	0.953882	0.652186	1.210454	0.886049	0.675417	1.285071	1.291415	0.919848
Trim72	0.989965	0.607588	0.99008	0.832971	0.725256	1.291074	0.39353	0.942544	0.919332
Zfp689	1.653496	1.572747	1.531193	1.522439	1.283148	1.911377	1.629852	1.53056	0.919292
Gm49668	1.663954	2.175595	2.070036	1.775704	2.009903	1.276055	2.348967	1.748468	0.918659
Iqcd	0.465307	0.530364	0.365641	0.330501	1.297807	0.860604	0.79008	0.537951	0.918636
Pgf	1.664018	0.6551	1.445236	1.393429	1.626497	0.971894	0.678886	0.917231	0.918185
Gm7125	0.405397	0.404318	0.304083	0.755862	0.337834	0.468623	0.628497	0.482472	0.917651
Gm30541	0.560911	0.375728	0.468875	0.88244	0.566098	0.394841	0.839309	0.629691	0.917593
5830428M24Rik	1.710721	1.492896	0.908926	0.265803	1.680187	0.988763	1.326087	1.374278	0.917139
Foxd4	0.37422	0.69313	0.080199	0.265803	0.534605	0.54382	0.221014	0.178147	0.917139
Gm7429	1.082305	0.359808	0.48108	0.358747	0.343592	0.667254	0.745744	0.915965	0.916918
H2bc11	0.721537	0.719616	0.12027	0.95666	0.916245	2.001763	0.248581	0.801469	0.916918
Gm46808	0.320451	0.426131	1.495613	0.84975	1.017314	1.185373	0.331202	0.305101	0.916255
Gm19135	0.695875	0.528779	0.530252	0.823783	0.852099	0.15322	1.164454	0.694091	0.91589
Zfp850	1.087298	1.168902	1.186281	0.435291	0.874149	0.74433	0.948642	1.384767	0.915166
Gm614	0.516733	0.662602	0.812103	1.174488	0.421826	1.160513	1.754799	0.49198	0.914629
Chrng	0.389775	0.956893	1.439338	1.192591	0.314107	0.47136	0.774716	0.884941	0.914438
Gm15337	1.375221	0.528531	0.784055	1.114919	0.542251	0.538676	0.937011	0.871507	0.914156
Gm15478	1.060049	0.742576	0.984446	1.167052	0.865354	0.583512	1.213001	0.696878	0.9141
Ceacam18	0.410991	0.546529	0.274026	0.681147	0.478406	0.506762	0.094395	0.521738	0.913991
Gm36723	1.112935	1.14199	0.813393	0.601236	0.963125	0.781802	0.906947	0.575661	0.912836
Ptges	1.550502	0.636742	0.653719	0.604634	0.448795	0.604469	0.612729	0.60786	0.912738
Mir100hg	0.851256	0.84899	1.064194	0.105811	1.11475	1.279225	1.649655	1.317029	0.912738
Snord66	1.911657	5.719704	4.779699	1.900949	3.641283	1.767841	6.915276	6.370298	0.91099
Mir7048	0.955829	0	0.95594	0	0	0	1.975793	0	0.91099
Gm40950	0.260273	0.432633	0.694141	1.12153	0.743642	0.481384	0.448341	0.99122	0.909564
Gm6238	0	0	0	0.829877	0	0.330757	0.616108	0	0.909031
4930469K13Rik	0.873666	1.267403	1.112067	1.10571	1.361569	1.028286	1.477599	1.134294	0.908379

Nrbp2	1.140071	1.00127	1.446526	1.455177	1.231643	1.195924	0.685888	0.696638	0.908193
Gm31954	0.612199	0.339205	0.748331	0.879335	0.712619	0.12581	0.703043	0.064764	0.907636
Tspan15	0.648651	0.560668	1.12446	4.34312	1.029614	0.999754	0.625719	0.494064	0.906726
Gm41046	0.203816	0.067758	0.067947	0.135116	0.194113	0.251311	0.070218	0.646844	0.906524
Zfp970	0.843933	1.375149	1.153113	1.18198	1.347134	0.934333	1.289942	0.927994	0.906302
Gm29728	1.046001	1.232892	1.997143	1.418375	1.086764	0.791435	0.687969	0.362144	0.906302
Gm18002	0.570546	0.569027	0.47551	0.472792	0.633946	0.527623	0.589688	0.090536	0.906302
Rnase2b	1.387778	1.019851	1.972334	0.145264	0.556507	0.33773	0.377457	2.016721	0.904988
Gm4924	0.371513	0.802803	0.536693	0.513101	0.314511	0.496259	0.874616	0.805689	0.904885
4732471J01Rik	1.45155	1.447686	1.116707	3.330968	1.914149	0.722803	1.731058	2.073027	0.904568
Lrrc4	1.484675	0.870115	1.316463	0.761009	0.889209	0.50956	1.313013	0.714064	0.90445
Prss57	0.151781	0.189221	0	0.565991	0.108416	0.842175	0.078437	0	0.904131
Coro6	0.923968	1.697514	0.924075	1.499082	0.578932	1.911284	0.502614	0.833407	0.903798
Tbx15	1.064517	1.24416	0.964831	1.356273	0.68117	1.23054	0.601689	0.712635	0.90361
Snora44	3.790637	4.253113	1.421654	1.413526	3.610161	2.190914	6.366445	5.864719	0.903203
Adamts14	0.559763	0.762257	0.990465	1.402273	0.594625	1.124896	0.956821	0.778926	0.902853
Glt1d1	0.059102	0.23578	0.118219	0.646485	0.112577	0.655873	0.366512	0.225085	0.901278
Gm14388	1.102461	0.785376	0.708808	0.704755	0.712481	0.910287	1.383617	1.349552	0.900637
Mmp11	0.761512	0.911381	1.188097	0.545217	0.783276	0.422533	0.629648	0.580027	0.899978
Gm15163	1.664135	1.139499	1.813373	1.407827	1.064488	1.217371	1.694296	1.726308	0.899561
Capsl	1.41544	2.744917	1.651538	2.033072	3.295232	1.817992	2.925856	2.246063	0.89936
Gm7860	0.353109	0.234779	0.294291	0.468174	0.448396	0.598664	0.669085	0.112065	0.897451
Gm20947	0.654619	0.530462	0.450103	0.976428	1.28587	1.097236	0.761154	0.5064	0.896871
Tmem91	0.392066	0.782043	0.235267	0.467843	1.642955	1.232738	0.810439	1.119854	0.896816
Gm6091	0.537364	0.491272	1.343566	0.623413	0.042648	0.786818	0.185131	0.042635	0.896273
Snord38a	0	0.937127	0.939737	0	0	0	0.971153	0	0.895549
Pik3ip1	1.260518	1.306462	1.062913	0.81106	0.800336	1.805672	1.302808	0.964816	0.89515
LOC118568764	0.239036	0.201723	0.551687	0.420542	1.015695	0.510123	0.361082	0.630238	0.893767
Cyb561	1.071698	1.091113	0.982504	2.086986	1.041938	0.660715	0.461522	0.850302	0.893746
Gm36154	0.42176	0.327163	0.843619	0.605797	0.624836	0.520041	1.307731	0.892349	0.893278
Popdc2	0.802965	0.200207	0.334608	1.696743	0.382368	1.485114	0.829905	0.732647	0.892847
Gm15975	0.65288	0.566211	1.163966	1.467809	1.027315	0.393759	2.347076	0.675659	0.892798
Naip1	0.580814	1.465901	1.766353	0.919381	1.546592	0.887889	1.457871	1.478406	0.892485
Gm12009	2.069688	1.130383	1.182816	0.049002	1.220234	0.91142	1.069563	0.609931	0.892365
Gm20346	0.560925	0.65267	1.262227	0.325373	0.890364	0.561953	0.676368	1.112615	0.891019
AV099323	1.245799	2.484965	0.830629	2.271171	1.483107	1.344089	0.965697	0.889592	0.890518
Pask	1.811554	1.840397	1.260358	1.051753	1.039469	1.487972	1.314121	1.285548	0.890096
Pax7	0.70027	0.970008	2.036208	0.528708	0.45697	0.431725	0.254658	0.666726	0.889893
4933440N22Rik	0.933475	0.749964	1.037315	1.3408	1.506415	1.127019	2.519183	1.234389	0.889685
Sfmbt2	1.574779	0.785293	1.370801	1.362964	1.083191	0.889965	1.356344	0.777438	0.889425
Cadm3	1.17301	1.435771	0.77321	2.028014	1.612268	1.799721	1.446571	1.573702	0.889305
Gm4013	2.132233	1.063278	1.332801	2.385326	1.523037	2.464778	1.652827	2.030095	0.889091
Gm49249	0.898062	2.308076	0.967256	0.893032	0.888201	0.8305	0.856793	0.690612	0.888853
Gm5801	0.559509	0.83703	0.699468	0.185458	0.532871	0.258708	0.722851	0.621492	0.88877
Lrrc18	0.577017	0.708285	1.242951	0.926884	1.09909	1.108262	0.367001	0.887455	0.888379
Slit3	1.353772	1.549373	1.29844	1.180674	0.834886	0.656748	0.309657	0.802936	0.888379
Gm4972	2.058708	2.053227	1.421654	2.485857	1.540543	1.586524	1.925132	1.21339	0.887631
Orm2	0.214876	0.571478	0.214901	0.498569	0.341076	0.198711	0.222085	0.136389	0.88745
Adamts2	1.44986	1.446	1.579768	2.617893	1.431703	1.517204	1.088386	1.017143	0.88729
Tmem200b	1.335369	1.00895	0.647527	2.454583	1.464487	1.983337	0.292764	0.963184	0.887051
Gm15938	1.330794	1.147623	0.965688	1.119366	0.729012	0.892934	1.328904	0.876453	0.886902
Gm4707	1.037898	0.428332	0.28635	0.284713	1.227082	1.125301	1.1467	0.340752	0.886877
Rgs11	0.711095	0.627371	0.984708	1.142258	0.468858	0.708183	0.961091	0.677032	0.886271
St8sia6	1.017212	1.311859	1.473375	0.645276	1.018891	0.843374	0.706935	0.350659	0.885917

LOC118568351	0.132786	0.331081	0.664006	0.594189	0.126464	0.491185	1.372407	0.316063	0.885897
Gm18046	0.30971	1.132579	1.238983	1.642533	0.983214	0.09547	0.746901	3.047033	0.885543
Gm41130	0.154639	0.231341	0.927942	0.307545	0.220915	0.071503	0.239741	0.147231	0.884308
Zbtb26	0.729723	1.09167	1.02173	1.181748	0.794263	0.761588	0.635687	0.734468	0.884266
Gm46989	1.631472	1.371893	1.407708	3.46734	1.157731	0.414162	2.975661	1.34012	0.88418
Nat14	1.76695	1.321685	1.457903	1.405642	1.682824	2.124226	1.050083	1.261732	0.884132
Gm33641	0.987761	0.615707	1.296586	1.59612	0.940732	0.742178	1.786575	1.410667	0.882585
Tspan18	0.670429	0.445763	0.303324	1.206361	0.957763	0.6938	0.478446	0.66871	0.882398
Btbd8	1.375548	1.26813	1.537556	1.310371	0.902728	0.983444	0.872134	1.364684	0.881358
Olfr1033	0.45159	0.621964	0.387122	0.406293	0.716816	0.596595	0.511192	0.552803	0.881306
Gm32392	0.615978	0.460754	1.540125	0.306264	0	0	1.43245	0.146618	0.880623
1810041H14Rik	0	1.290111	0.36963	0.183758	0.351991	0.683565	0.190993	0	0.880623
A930003A15Rik	0.271755	0.054206	0.163072	0.432373	0.46587	0.351835	0.112349	0.465728	0.880623
Gm33741	0.307989	0	0	0	0.439988	0	0	0.146618	0.880623
LOC115489080	0	2.764524	0	3.675168	0	0	0	0.879708	0.880623
Mafa	0.92364	1.273974	1.041673	0.703506	1.160409	1.490225	0.609337	0.860685	0.880311
Gm35941	0.051284	0.511475	0	0.254984	0.097685	0.047426	0.265023	0.048827	0.879809
Prkcz2	1.022474	0.795904	1.496478	1.289533	1.995089	1.083926	2.268207	1.662066	0.879435
Gm40103	1.094931	1.14949	0.74925	0.916882	0.987916	0.479632	1.548595	1.810625	0.878792
Gm6825	0.230512	0.114949	0.115269	0	0.219537	0.106585	0	0	0.878792
Gm14541	0.614272	0	0.460758	0.916247	1.316309	1.278134	0.476161	0.438635	0.878184
A4galt	0.697861	0.723843	0.809613	1.221355	0.451952	0.748617	0.634721	0.451814	0.877963
4632404H12Rik	0.413296	0.674503	1.127303	0.934048	0.876697	0.955509	0.873741	1.144723	0.877341
Eya1	0.766627	0.786431	1.248651	1.241513	0.584102	0.465883	0.543324	1.355536	0.876796
Emp2	1.176447	1.060806	1.418348	2.147409	1.611585	1.087942	0.499692	1.012687	0.875503
Gm41307	1.530381	1.259203	1.186183	0.570678	1.275329	0.778387	1.186294	1.4935	0.875154
Gm7327	1.42759	0.169499	0.985831	0.844996	2.395522	0.942993	0.843134	0.420706	0.874684
Gm51767	0.083366	0.748292	1.083878	0.58029	0.158793	0.693844	0.603137	0.714349	0.874002
Gm2058	0.991636	1.156939	1.197586	2.083794	1.211713	1.418807	1.372986	0.694741	0.873788
Vsig10	0.820494	1.582066	0.724854	0.870291	0.664215	0.872583	0.353342	1.119707	0.873238
Rnls	0.814268	1.21815	1.221544	1.147084	1.066312	0.690259	0.63119	1.065986	0.873078
Med12l	0.806187	0.412886	0.697324	0.664448	0.830058	0.537324	0.803208	0.532458	0.872201
Olfr431-ps1	0.243952	0	0	0	0.058084	0	0.126068	0.058067	0.871904
Zfp651	1.574353	1.581707	1.134592	1.25473	1.135572	1.841301	1.08877	1.631198	0.871613
Gm11878	0.342916	1.026009	0.685911	0.795655	0.217726	0.105706	0.354421	0.10883	0.871545
Akap12	1.588509	1.230251	1.482189	2.409127	1.445264	1.534657	0.797971	1.706749	0.871178
Clec4a4	1.319954	1.468337	1.066241	0.252415	1.063708	0.328637	1.154355	0.676698	0.870946
Ednra	0.517402	0.424961	0.593559	0.953345	0.666686	0.928808	0.550489	0.521595	0.870229
Gm36423	0.331964	0.413851	0.622506	0.907789	0.276639	0.575607	0.857755	0.395078	0.870077
Gm2619	0.317469	0.079156	0.396883	0.315691	0.302354	0.110095	0.205075	0.151131	0.869907
Gm14636	0.651555	0.835484	0.670249	0.721952	0.744641	0.464815	0.923541	0.726689	0.869386
Gm43471	0.607873	0.485004	0.668739	0.604468	0.521039	0.674571	1.068055	0.578755	0.869036
Ccdc138	1.114333	1.515499	1.621036	0.730333	1.374838	1.569171	1.413473	1.567319	0.868957
Gm6900	0.497202	0.743818	0.331507	0.412014	0.868138	0.53643	0.256942	0.31559	0.868776
Pdzd9	0.538636	0.454555	0.538698	0.494417	0.394608	0.153266	0.171294	1.380707	0.868776
Pdzd4	0.825924	0.403457	0.590015	0.351985	0.770548	0.763789	0.592317	0.690072	0.867504
Tmem263	0.821739	1.02444	1.027294	0.890095	0.754665	0.637789	0.940306	1.089739	0.867104
Gm31400	1.591741	2.403934	0.955156	0.994918	1.342704	1.64022	1.974172	1.255695	0.866897
Ppm1l	1.105403	1.130371	1.623312	0.779188	0.999469	0.931664	0.983408	0.905908	0.866843
2200002J24Rik	0.090882	0.18128	0.818034	0.722984	1.038661	0.672359	1.127174	0.951815	0.866187
Krt7	0.384501	0.348616	0.629257	0.382347	0.699099	1.292998	0.830929	0.532484	0.866187
LOC115487886	0.45441	0	1.817853	0	0	0	0.469656	0	0.866187
Snhg9	2.120582	0.906401	0.302975	1.80746	1.154068	0.840449	0.313104	1.153715	0.866187
Pdgfd	1.498326	0.950942	0.181636	2.122026	0.691874	0.503856	0.281563	0.38906	0.865477

Nacad	0.667607	0.612563	0.61427	0.637312	1.017314	0.543296	0.855606	0.533927	0.865352
Itga1	2.065957	1.422917	1.658517	3.067758	1.517607	0.736797	1.168175	1.120216	0.865318
Catip	0.697773	0.66112	0.662961	0.693864	0.531641	0.451695	0.360592	0.166087	0.864552
Fancd2	2.267531	2.273718	2.292311	1.206638	1.038929	0.668754	1.583518	0.431782	0.864463
Clic5	1.378868	1.328102	1.265684	2.601418	1.007401	1.877766	0.556386	0.647417	0.864121
5430431A17Rik	0.767068	1.599599	1.39483	0.762771	1.527507	1.870131	1.513532	1.925398	0.864008
Neil2	0.700184	1.519872	1.276954	0.532435	1.098336	0.609419	1.87304	0.431357	0.863613
2410003L11Rik	0.54351	0.090344	0.905956	0.810699	0	0.16754	0.093624	0.172492	0.863356
Gm5497	0.503068	0.200691	0.20125	1.400699	0.191646	0.837398	0.311967	0.47897	0.863043
Myf5	0.319374	0.982116	1.756763	1.217411	0.861811	0.369184	0.632671	0.937567	0.862444
Klra7	0.266018	0	0.478887	1.534259	1.266761	1.968038	0.604873	0.20262	0.86203
Far2	0.375273	0.646473	0.562975	0.474945	0.422389	0.709856	0.617055	0.828279	0.861656
Ociad2	0.625764	1.271311	1.182136	1.428891	0.198657	0.321493	0.71862	0.507524	0.861479
Ccdc18	0.903783	1.02757	0.921966	0.736951	0.688603	0.501474	1.251701	0.688392	0.861386
Fancb	1.889001	2.184315	0.93092	1.143386	0.599688	1.493716	1.018631	1.954906	0.861054
Gm34360	0.60177	0.600168	0.52661	0.373999	0.644759	0.765185	1.088429	0.429708	0.86031
Gm20008	0.812081	0.953905	1.173142	0.717806	0.429676	0.767676	1.380225	0.412363	0.859984
Gm14846	1.577506	1.12379	0.676153	0.896383	0.214628	0.833616	2.795025	0.214563	0.859145
Gm39671	0.788753	1.348548	0.450768	1.232526	1.287771	1.56303	1.74689	1.716503	0.859145
LOC118567477	1.352148	0.749193	0.676153	1.792765	0.715428	0.972552	1.319873	0.643689	0.859145
Gm16998	0.315501	0.719226	0.225384	0.672287	0.772663	1.417147	0.791924	0.257475	0.859145
Gm7380	0.200137	0.199605	0	0	0.190609	0.462702	0.413704	0.285826	0.858369
Gm35189	0.225053	0	0.075026	0.372987	0.214338	0.901862	0.85288	0.214273	0.857982
Zfp14	0.916194	1.329098	1.199521	1.623347	1.316792	1.186174	0.912498	1.157789	0.857338
Gm38951	1.512691	2.324157	0.490659	0.853745	0.350433	0.415886	1.436676	1.323454	0.857244
Pdk4	0.417431	0.576443	0.481707	1.149488	0.351685	1.48472	0.44803	0.397435	0.856905
LOC118568508	0.449387	0.896382	0.347294	0.365621	1.050525	0.604479	1.118937	0.583447	0.856612
Gm35887	0.280841	0.44815	0.056175	0.055854	0.481446	0.207771	0.290263	0.213911	0.856533
Gm35062	0.449255	0.896118	1.25806	1.340215	1.625889	0.498548	0.835789	0.684376	0.85636
Gm33363	1.447078	1.492992	1.646867	2.183268	2.233604	1.430503	1.701921	1.85285	0.856051
Rab15	1.108379	1.410374	0.688039	0.741115	0.691605	0.583186	0.158009	0.473059	0.856035
Gm17812	0.617225	0.055962	1.178476	0.502174	0.267199	0.830241	0.521945	0.106847	0.855666
Fam71e1	0.896572	0.745155	1.419738	2.526059	0.853886	0.483655	1.158315	1.067031	0.854513
Rptoros	1.037989	0.376446	0.707802	0.844507	1.123375	0.130895	0.633935	0.35937	0.854392
Blk	0.201593	0.067019	0.067205	0.089095	0.511986	0.35214	0.046301	0.021326	0.853938
Gm32528	0.864011	0.877378	0.864111	1.546507	0.583493	0.421296	1.266434	0.673055	0.853424
Gabbr2	1.669701	1.655344	1.987971	1.82836	1.656464	1.461369	1.602454	1.069276	0.852522
Gm4813	0.756599	0.685986	0.619107	0.273586	0.65507	0.063607	0.853072	0.589382	0.852216
Gm11517	0.487011	0.647619	0.243534	0.887852	0.309216	1.200993	1.0067	0.154561	0.850968
Gm52290	0.668227	1.258846	1.039584	0.935199	1.107828	0.938376	0.869702	0.942544	0.849172
1700007L15Rik	1.335857	1.406317	0.668006	1.254576	0.706809	0.960835	0.92045	0.353296	0.848794
Tmem17	0.890246	0.887876	1.133172	2.253388	1.31033	1.347172	0.50188	0.308219	0.848484
Gm51653	0.645079	0.437486	0.722572	1.244443	0.675802	0.334067	0.880072	0.798432	0.848448
Usp27x	1.043741	0.597274	0.8214	0.833719	0.651835	0.506345	1.184866	0.765672	0.848008
Vtn	1.588483	1.425829	1.207388	2.306194	1.149769	2.14471	0.361191	2.026605	0.847821
Inca1	1.630531	1.478355	1.334226	1.105498	2.188177	0.685393	1.149024	2.258074	0.847659
Rnu2-10	0.29646	0.591342	0	0	0.564691	0.548314	0	1.129037	0.847659
Vsig4	1.43041	1.6965	1.778555	0.307545	0.515468	1.787566	2.517276	1.177851	0.847462
Gm33547	0	1.063278	0	0	0	0.821593	0.367295	1.015047	0.846753
Gm33377	1.141745	1.391751	0.126875	0.12615	1.268616	0.762558	0.196675	0.966269	0.846366
Itga8	0.797913	1.098412	0.809245	0.480536	0.685001	0.519637	0.534301	0.770391	0.84617
Zxdb	0.601409	0.580142	0.828266	0.558824	0.40376	0.638221	0.580826	0.797885	0.845699
Nr6a1	0.972101	1.186006	0.698484	1.032351	0.692117	0.576038	1.131523	0.835677	0.845542
Ndufs5-ps	1.219637	0.110581	1.10889	2.425611	0.950375	0.922813	2.062728	2.322429	0.845398

Fndc7	2.168244	2.211619	2.119212	1.73958	2.158876	2.27855	2.037262	1.66558	0.845398
Chit1	0.539731	0.346046	0.886804	0.076672	0.036717	0.356519	0.039846	0.477171	0.845104
Col9a3	0.464447	0.4211	0.654524	0.92369	0.46244	0.527121	0.523668	0.542699	0.845076
Npr2	0.829685	0.65822	0.773206	1.912588	1.508532	2.336677	1.032923	1.1131	0.844679
Gm8388	0.663487	0.882295	0.958482	0.439847	0.070211	1.090795	0.457165	0.491326	0.84315
Sytl3	0.774069	0.441147	0.368647	0.513155	0.421265	0.613572	0.342874	0.245663	0.84315
Gm34643	1.290925	1.120592	1.19544	0.78448	0.136607	0.309506	0.64241	0.591783	0.843029
Gm26725	0.165817	0.606376	0.442229	1.264141	0.526407	0.306684	0.742646	0.841994	0.84287
Nova1	2.503179	1.541965	1.47263	1.976684	0.420706	1.770189	2.05451	0.630866	0.84203
LOC118567844	0.784964	0.880733	2.355165	0.975708	1.775528	0.362955	1.419774	0.467101	0.841658
Gm36608	0	0.391437	0	0.292713	0	0.362955	1.014124	1.027623	0.841658
Gm1818	1.324626	1.370029	2.109835	0.780567	1.822253	1.088865	0.912712	1.588145	0.841658
9130023H24Rik	1.439749	1.080797	1.207671	1.83194	1.164784	1.403017	1.536054	1.473959	0.841031
1700012D14Rik	0.176274	0.175804	0.088147	0.876431	0.251822	0.244519	0.091094	0.755234	0.840022
Gm51536	0.739644	1.86175	0.845405	1.506025	1.308225	0.586285	1.419709	1.173689	0.839222
Zmym1	0.863252	0.895392	0.966955	0.875585	0.624836	0.463019	0.820837	0.460264	0.839211
Kcnk12	0.994023	1.076351	0.852118	0.734281	0.70326	0.499017	0.998019	0.324482	0.839119
Gm36501	1.173292	0.48757	0.488929	0.486133	0.465596	1.446699	0.404219	0.465454	0.838689
Gm51287	2.639908	0	0	0	0	0	3.637969	0	0.838689
Gm31414	0	0	0.146679	0	0	0	0.303164	0	0.838689
Mir6924	0.879969	0	0	2.62512	0.838073	0	0	2.513451	0.838689
Mir7664	0.879969	0.877627	1.760143	0	0.838073	0	0	0.837817	0.838689
Spire2	0.499014	0.094797	0.261419	0.732513	0.859986	0.791096	0.687674	0.339364	0.83797
Lyplal1	0.773391	1.332301	1.476645	1.433246	1.171814	1.105321	0.908339	0.736344	0.837625
Sertad4	0.627736	1.091142	1.434992	1.016587	0.580768	1.028333	0.871241	0.887961	0.837604
Lbx1	0.439287	0.679083	1.076379	0.698923	0.60664	0.568735	0.227013	0.669191	0.83736
Bloc1s2-ps	0.564415	0.250183	0.6272	0.561253	0.418088	0.753932	0.259267	0.477669	0.836791
Tmem8b	1.000984	0.752158	1.467366	0.968106	0.757437	1.179289	1.403043	0.913869	0.836407
Htra4	1.267341	1.166739	0.974991	0.904789	0.433283	0.781332	2.28386	1.175694	0.836231
2310043M15Rik	0.701748	0.839855	0.982561	1.535197	0.835421	0.129791	0.326381	0.40088	0.836035
Gm16845	1.296351	1.597111	0.95331	0.568716	1.053067	0.740451	1.261029	0.980142	0.835805
Npb	0.76693	0.546349	0	0.108948	0.20869	0.810552	0.566186	0	0.835374
Gm15471	0.26274	0.52408	0.26277	0.174179	0.08341	0.161982	0.181036	0.583692	0.834714
2200002D01Rik	0.984108	0.109054	1.64037	1.304793	0.833114	1.314548	0.339041	0.41643	0.833726
Stag3	1.6813	1.475605	1.591816	0.847087	1.473152	1.430428	2.757162	1.472701	0.833263
Gm7879	0.145698	0.217965	0.218572	0.79685	0.555044	0.741053	0.150586	0.346797	0.833178
Osbpl6	1.22322	0.958542	0.480606	0.651626	0.790523	1.211995	0.812738	0.124781	0.832741
Myh1	1.81086	2.172748	1.985743	1.462511	2.136107	1.640624	0.798049	1.076479	0.832292
LOC118568250	1.587346	2.058056	2.06379	2.01253	2.154274	1.321135	3.322218	1.813571	0.832085
1700052K11Rik	0.849713	0.458082	0.803876	1.530051	1.137335	0.849501	0.712071	0.787145	0.83174
Cd300c	0.930754	1.218363	1.454473	0.867695	2.437711	1.613874	1.382848	2.215424	0.831648
She	1.149592	0.632569	0.535217	2.46369	1.26475	1.063106	0.737479	0.792586	0.831193
Ttc30a1	0.929779	1.622781	1.511066	2.330689	1.475852	0.967309	0.960973	0.719258	0.830777
Pgpep1l	0.290252	0.057896	0.058057	0.057725	0.718724	0.483148	0.899969	0.165809	0.829907
Gm23472	0	0	0.580571	0.288626	0	0	0	0	0.829907
Eomes	0.157943	0.157523	0.221146	1.130821	0.330931	0.890972	0.473402	0.60151	0.827936
Prkca	1.251977	1.070266	1.059998	1.119809	0.858001	1.243551	1.239209	1.009104	0.827064
Ppp1r42	0.048207	0	0	0.527307	0.321383	0.04458	0	0.183591	0.82702
Gm16409	0.5337	0.798418	1.134244	0.53071	0.50829	1.233872	0.758457	0.825719	0.826578
Slc16a2	0.960197	1.077346	0.733569	1.299614	1.041493	0.937292	0.59269	1.269725	0.82618
Trim34b	0.505624	0.432238	1.444808	1.149238	0.791119	1.26916	0.671898	0.894035	0.826122
Gm25820	0.43311	1.727827	0.43316	0.430684	2.887424	0.400526	2.238203	0	0.825584
D330023K18Rik	0.974497	0.971903	0.97461	2.045748	1.237467	1.301711	1.342922	2.68036	0.825584
Gm15133	0.399794	0.531639	0.26656	2.05403	1.078818	1.17077	0.482075	0.253762	0.825584

Gm24907	0	0	0	0	0.412489	0	0	0	0.825584
Mir7028	0	0	0.86632	0	0	0	0	0	0.825584
Gm33251	1.069054	0.710805	0.560046	0.303733	0.290901	0.517852	1.420612	0.969375	0.824826
Gm5855	1.409985	0.639196	1.506296	0.955969	1.281816	1.481716	1.622891	1.006833	0.82463
LOC118567733	1.080532	1.084391	1.18197	1.020756	0.855429	0.637017	1.305241	0.951615	0.823875
Mapk12	1.901227	1.680692	0.950724	2.749931	0.905354	3.15676	1.250463	1.275336	0.823654
Cxcl11	0.483572	0.275591	0.310904	1.442589	0.098689	0.063885	0.071399	0.460408	0.823012
Gm10416	0.086352	0.602856	0.086362	0.515211	0.164482	0.319423	0.356997	0.328863	0.823012
Gm38855	0.647137	0.430276	0.755081	0	0.513605	0.598452	0.222949	0	0.822372
Rpl19-ps9	0.156605	0.078094	0	0.856501	0.596594	0.289646	0.080929	0.596412	0.82092
Xirp2	1.815187	2.05298	1.413016	1.451459	0.842104	1.535858	0.575398	0.730492	0.820429
Zfr2	0.286317	0.285554	0.465319	0.711782	0.272685	0.364068	0.443884	0.204451	0.818656
Entpd2	2.175164	1.798296	1.001837	2.049139	2.453213	2.646742	1.035328	1.171733	0.818339
LOC118567796	1.501805	3.380764	1.029929	1.237383	1.144242	0.714251	1.108708	0.204267	0.817916
4930426L09Rik	0.596911	1.674341	0.447735	0.445175	0.746144	0.552004	0.848288	0.568317	0.817806
Rtl3	0.350997	1.12798	1.38465	1.357343	0.687143	1.226234	0.786009	1.503827	0.817744
Gm41659	0.536151	0.962503	1.394156	1.332871	0.970187	0.198326	0.942037	0.714656	0.8176
Gm6382	1.715099	1.609914	2.472048	0.6521	1.104975	0.932982	1.824777	0.960554	0.817321
Pde4d	0.84152	1.270685	1.305687	0.797703	0.764003	0.989128	0.983552	0.733818	0.817035
Tead1	0.840312	0.950569	1.308558	1.435671	0.880872	0.818818	0.868505	0.853755	0.817018
Dlk1	1.324592	1.826179	1.75334	3.796555	3.265128	2.233716	0.724782	2.151359	0.816882
Gm17227	0.085685	0.085457	0.514169	0.17041	0.816053	0.475432	0.177119	0.407902	0.816652
1110046J04Rik	1.284279	0.782748	0.856286	0.070949	0.339759	0.923737	0.073743	0.271724	0.816022
Ptprm	1.363802	0.893543	0.826341	2.47475	1.128216	1.46373	1.028874	0.947791	0.815948
Gm7159	0.155506	0.853009	1.166434	0.541224	0.444308	1.15046	0.88398	0.888345	0.815163
Gm16001	1.307976	1.153975	0.754689	1.050525	1.149879	0.697832	1.403853	0.910043	0.815096
Tmem52	1.160048	1.40053	0.671685	2.185673	1.395558	1.750318	0.820346	0.639435	0.814674
Gm13127	0.736816	1.205162	1.414852	1.172302	0.785944	1.035705	1.340304	0.729582	0.814612
Kazald1	0.815837	0.542443	0.582808	0.193159	0.406997	0.395194	0.44168	0.480849	0.814592
Pacrg	0.801128	1.438191	1.068295	0.743531	1.119046	1.531107	0.496804	0.508502	0.814449
Lipt1	1.590595	1.934586	1.357983	0.771554	0.923698	1.614438	1.122704	1.477466	0.813452
Bok	0.49784	0.851168	1.742643	0.813299	0.711206	1.315392	0.220518	0.846415	0.813404
Mir7086	2.55868	1.701245	2.558977	0	1.624572	1.577458	3.526031	3.248152	0.812883
Mir7031	0.852893	4.253113	2.558977	0.848116	0.812286	0	1.763016	3.248152	0.812883
Gm8093	0.590465	0.610703	0.787378	1.717978	0.770631	0.910072	1.06233	0.936967	0.812883
Snord85	0.852893	0	0	0	0	0	0	0	0.812883
A330040F15Rik	0.651623	0.649888	0.601568	0.448596	1.336674	0.185415	1.243356	0.572685	0.812148
H2-M2	0.61942	0.926656	1.819757	1.077912	2.728419	1.897466	1.040327	1.179497	0.811748
2810021J22Rik	1.650698	1.988228	1.460403	1.79297	1.354431	1.197726	1.456729	1.354017	0.810835
Zfp870	1.113172	0.984524	0.913747	0.97118	0.970158	1.116831	1.161373	1.319812	0.810727
Gnat3	0.755544	0.470958	0.661178	0.798269	0.809519	0.349352	0.780893	0.269757	0.810113
Sox12	0.999785	0.934803	1.199881	0.658647	0.821259	0.936127	0.632913	0.749616	0.809951
Rps13-ps5	0.849625	1.482886	1.805664	0.528041	0.910321	2.258908	1.756261	1.314506	0.809769
Gm5903	1.323819	0.90926	1.373935	0.68304	0.999118	0.8431	0.684117	0.820453	0.809404
Rasgrf2	0.988467	0.771928	1.01656	1.36312	0.817068	0.905479	1.224028	0.843454	0.808781
Gm16259	1.060001	0.422872	0.212025	0.105406	1.009534	0.588154	2.191128	1.009225	0.80822
F730311O21Rik	2.230011	1.645815	2.051848	1.818366	1.189349	1.77353	1.982151	1.656087	0.807651
Cdh2	0.263282	0.536579	0.572419	0.865103	0.196237	0.211717	0.402257	0.457746	0.807344
Kcnmb3	0.384987	0.537546	0.770063	0	0.586651	0.712047	0.795806	1.099635	0.807238
Gm23747	0.423191	1.688259	0.846481	0.420821	0	0	3.499115	0.40292	0.806678
Gm18332	1.04109	0.843634	0.780909	0.841148	0.371821	0.060173	1.076019	0.557561	0.806204
Fancc	1.439653	2.438615	1.325549	1.772443	2.111074	1.141154	1.346243	1.196635	0.805847
Gm20762	0.12078	0.963668	0.241588	0.600518	0.690178	0.335081	1.12349	0.804961	0.805799
Gm6749	1.09862	0.505706	1.267786	0.840359	1.368257	0.703364	0.524067	1.287377	0.805448

Gm5741	0.676074	0.168569	1.014229	0	1.126799	0	0	0.643689	0.805448
Golm2	0.276826	0.494054	0.466288	0.434645	0.471788	0.471579	0.602344	0.485515	0.805406
Myo1h	0.931186	0.626337	0.476476	0.689094	0.433114	0.360474	0.33573	0.453599	0.804945
4921531C22Rik	1.600574	1.596313	2.735844	1.331163	2.494422	1.453249	1.503885	1.163708	0.804349
Peak1os	0.832091	0.778009	0.791734	1.022795	0.5008	0.283216	0.686812	0.473139	0.804071
Susd2	0.957479	0.496564	0.727769	1.31392	0.76599	1.221917	0.57397	1.549744	0.803055
Gm52003	0.30064	1.139391	0.300675	0.896869	0.343592	0.778464	1.056471	0.343487	0.802303
Smt3h2-ps3	0.505003	0.27981	0.392825	0.446377	0.320639	0.570791	0	0.213694	0.802187
Gm7997	0.420782	0.349718	0.666316	0.627638	0.834893	0.389126	0.761074	1.135107	0.802086
Il12b	0.619667	0.728377	0.442671	0.132042	0.906323	0.573049	0.983559	0.611055	0.801525
Ntn1	0.937172	1.002128	0.888967	1.69091	0.901754	0.956014	0.619114	0.708304	0.801125
Mir6922	0	0	0	1.670531	0.799979	0	0.868152	0	0.800567
Fhit	0.969197	1.933233	2.00324	1.09227	0.123074	0.119504	0.667809	0.061518	0.800567
P3h4	0.893725	1.296503	1.598059	1.292683	0.954347	1.076941	0.279912	0.825129	0.800176
Chst13	0.201471	0.334891	0.302241	0.634417	1.183252	2.080506	0.48587	1.31077	0.800082
Trnp1	1.441256	2.473697	1.13973	1.566502	1.021497	1.518804	1.66282	1.850897	0.798631
Gapdhs	1.140701	1.630652	0.608445	1.436794	1.158817	0.527442	1.375467	0.796443	0.797272
LOC118568009	0.949933	0.795819	1.10205	0.453414	0.868517	0.28111	0.74617	1.01296	0.796726
Farp1	0.706067	0.516404	0.988609	0.631901	0.650036	1.251486	0.814894	0.549	0.796318
Gm18935	0.834911	0.832688	0.918508	2.822795	0	0.38605	2.847642	0.079492	0.795744
Rad54b	1.629974	2.797603	1.933449	0.923504	1.173302	0.876366	2.017673	0.812038	0.794819
Trim69	0.486299	0.242502	0.138959	1.001691	0.430064	0.289102	0.753921	0.992152	0.794547
Mir155hg	1.315777	0.699879	0.175457	1.090339	0.710108	0.486716	0.453307	1.628573	0.794233
Gm19345	0.66659	0.166204	0.555556	0.718094	0.952279	0.15411	0.861192	0.317329	0.794149
Gm17344	1.203163	0.646132	0.555371	0.736261	0.352578	0.256765	1.243529	0.704941	0.793884
Gm33973	0.388455	0.498112	0.8325	1.21402	0.951326	0.975055	0.688264	0.739694	0.793354
Gm39058	0	0	0	0.662192	0.158554	0	0.172066	0	0.793354
2700012I20Rik	0.907581	1.357747	1.891013	1.052913	0.504216	0.76936	1.407045	1.728212	0.792921
Hoxa4	0.253142	0.649203	0.506343	0.719211	0.826593	0.40131	0.074753	0.619755	0.792733
Gulp1	0.425257	0.539795	0.541299	0.461318	0.368191	0.625648	0.319654	0.920196	0.792193
Atp8b1	1.214555	0.589723	0.89504	1.096512	0.213082	0.990174	0.165172	0.502111	0.792028
Slc18a2	0.758028	0.596084	1.122595	0.811765	0.61087	1.348077	0.828659	0.929904	0.791936
AU041133	0.979697	0.40358	1.128912	1.143637	1.156174	0.768124	1.012566	0.9936	0.791648
Gm9169	0.553689	0.414161	1.107506	1.307644	0.065916	0.512034	0.071533	0.790749	0.791572
Gm30823	0	0.331081	0	0	0.15808	0.153495	0.343102	0.158031	0.790979
Gm13803	0.860458	1.28725	0.95276	0.947313	1.317038	0.483118	1.333989	0.672947	0.790804
Rab42	0.754259	0.300901	0.452608	1.500069	0.790183	2.022795	0.779565	0.646316	0.790764
LOC115487221	2.606063	1.535846	1.540125	1.413526	1.35381	1.095457	3.550517	2.030095	0.790303
Gm4521	0.769151	4.307582	1.834343	2.176861	1.803154	0.87543	1.773364	2.309585	0.78946
9330151L19Rik	1.00163	1.715612	1.110632	0.887757	1.513864	1.087368	1.552852	1.015845	0.788618
Morn3	0.591024	0.23578	0.354656	0.528942	1.463501	0.655873	0.794109	0.787798	0.788618
Gm22362	1.241151	4.126155	2.48259	0.822799	0	0.382592	0	0.393899	0.788618
Gm23116	0.827434	0	0	0	0	0	0	0	0.788618
Gm24507	0	1.650462	0	0	0	0	0	0	0.788618
Treml1	1.4879	3.13276	2.039211	2.739937	2.624185	1.681733	2.733901	1.836368	0.787834
C230037L18Rik	0.381455	0.760878	0.572248	1.074734	0.54494	1.117065	0.459961	0.363182	0.787714
Rcor2	0.412992	0.557266	0.607411	0.773042	0.509014	0.494252	0.652826	0.393209	0.787236
Zap70	0.330234	0.205847	0.454125	1.68297	0.982848	0.877997	0.298649	0.550227	0.786856
Gm34434	0.164994	0.164555	0.825067	0	0	0.152582	0.17053	0.157091	0.786271
LOC118567835	0	0	0.165013	0	0	0.152582	0.17053	0	0.786271
4933439K11Rik	1.413936	1.2339	1.178417	0.234336	1.346617	0.326891	1.15692	0.841378	0.786104
9630025h16rik	0.206089	0.411082	0.103057	0.102468	0	0.571755	0.639011	0.784869	0.785686
LOC101055663	0.117703	1.526064	0.470866	0.234087	0.336297	0	0.486607	0.280162	0.785269
Gm6474	0.126716	0.442324	0.380191	0	0.181024	0	0.523867	0	0.785013

Gm9058	1.013725	0.568702	0.760382	0.945043	0.784436	0.527322	0.19645	1.327102	0.785013
Hmgb1-ps7	0.606828	0.907819	0.563549	0.387918	1.155872	0.320672	0.313593	0.371417	0.784918
Zglp1	0.65867	0.492687	0.439164	0.218327	0.522758	1.624313	0.397115	0.470339	0.784714
Xlr3b	1.577088	0.820638	1.645848	1.125052	0.195913	0.570694	1.665434	0.816055	0.784229
Chrnd	0.785295	1.212101	1.421175	0.353262	0.552026	0.778088	0.734342	0.890092	0.784096
Gm30671	0.164505	0.820333	0.164524	0.490749	0.470017	0.152129	0.510071	0.313249	0.783938
Rab33a	0.740271	1.4766	1.562976	0.899707	1.253379	0.608515	0.510071	0.783123	0.783938
4930444A19Rik	0.17618	0.878556	0.646069	0.52558	0.894892	0.651704	0.728364	1.118273	0.783605
Hid1	0.308275	0.290373	0.719391	0.868552	0.407774	0.395948	0.442524	0.260896	0.783502
Gp9	0.063213	0.12609	0.189662	0.691451	0.481629	0.409204	0.130668	0.601853	0.783223
Amd-ps3	2.111926	1.257931	0.762729	1.108384	1.033623	0.976522	1.273289	0.726108	0.782776
Gm38552	0.410652	0.546079	0.4107	0.816704	0.521468	0.506345	0.707383	0.130327	0.782776
Gm9057	0.985566	0.409559	0.8214	0.08167	2.033724	1.443082	0.254658	0.781963	0.782776
Gm36551	0.598252	0.643457	0.832958	0.233295	0.234611	0.379678	0.36372	0.30155	0.782611
Ptch1	1.08583	1.347994	0.979639	1.185457	0.911194	0.919878	1.326306	0.737407	0.781597
Zfp772	1.43995	1.31644	1.140093	1.511433	0.72379	1.498073	1.736303	1.142478	0.781506
Gm8349	0.318871	0.408886	0.865609	0.543575	0.347074	0.589764	0.659139	0.390339	0.78149
Gipc3	0.81903	0.816849	0.819125	2.239715	1.170052	1.183458	0.952321	0.146212	0.780608
Pknox2	0.6231	0.798995	1.584637	0.814344	0.932538	0.790249	0.699205	0.915303	0.780514
Gm51535	0.691298	0.535501	0.604117	0.400442	0.677561	0.695151	0.749175	0.651793	0.780407
LOC115490496	0.818274	1.836215	0.818369	0	3.117261	1.32425	0	2.142462	0.779888
Cep83os	1.555215	1.524332	1.783342	1.306529	1.608857	0.781099	0.762126	1.136068	0.779463
Ip6k3	1.274715	1.319295	2.57378	1.387157	1.282743	0.778464	0.770602	1.671636	0.77938
Tnfrsf4	0.767973	1.244634	1.248101	1.861449	2.468506	0.976522	1.885129	1.00538	0.777693
Hap1	0.90446	1.521107	1.312506	0.687771	2.094378	1.476028	2.474482	1.924889	0.777518
Gm18686	1.811701	1.174471	0.452978	1.711475	0.862722	1.089013	2.434229	0.689967	0.777021
Gm11628	0.815266	0.813095	0.135893	1.080932	0.258817	0	0.280873	0.129369	0.777021
Gm23751	2.038164	0.406548	2.038401	1.621398	0.388225	0.376966	2.949162	1.940532	0.777021
Mir7061	0.815266	0	0	0	1.5529	0	1.685235	2.328638	0.777021
Snord90	0	0	0	0	1.5529	0	1.685235	0	0.777021
Gm24608	1.630531	0.406548	0.81536	3.242796	1.164675	0.753932	1.263927	0.388106	0.777021
Mir133a-1	0	0	2.446081	0	0	0	0.842618	0	0.777021
Mir151	0	0	0	0	0.77645	0	0	0	0.777021
Tead3	0.565233	1.195104	1.15321	1.281513	0.279927	0.689977	0.397254	1.270051	0.775753
Cavin4	0.867915	0.811504	1.600404	2.373396	1.033241	1.605241	1.149324	1.704326	0.7755
Gm41485	0.56943	1.095262	0.366105	0.485349	0.542319	0.977955	0.67261	0.580878	0.77531
Rnf122	0.822809	0.884562	0.822904	1.030719	1.343372	1.255003	0.99399	0.92583	0.774025
H2bc15	0.115979	0.578352	0.231985	0.230659	1.104573	1.394301	0.11987	0.552118	0.773769
Shroom4	1.217357	1.657365	0.637737	1.652481	1.417042	1.447424	0.998571	0.791093	0.7735
Gm6663	0.259663	0.938773	0.616771	0.839178	0.680076	0.540289	0.63739	0.772577	0.773381
Rorc	1.0913	0.808522	0.717224	0.40307	0.504824	1.0092	0.290035	0.801534	0.77265
Mterf1b	0.891548	0.970008	0.810592	1.450724	0.578932	0.374762	1.172766	0.42442	0.772477
Asb4	0.708581	0.437478	1.012377	0.486518	0.771252	0.577265	1.464709	0.497948	0.771818
Gm10167	1.113485	0.538434	1.383582	1.107248	0.64271	0.374442	0.767228	0.931644	0.771818
LOC115489602	1.61863	1.21074	2.023522	0.402391	2.697739	0	2.091168	1.926367	0.771349
Scel	0.922086	0.844559	0.941013	0.748507	1.362082	1.218166	0.739078	1.074999	0.771218
Gm11634	1.176405	0.586636	0.220601	0.438681	0.560197	0.407963	1.139881	0.910043	0.770837
Rin1	1.32487	0.892335	1.066902	0.752828	0.540768	1.24111	0.497934	1.081205	0.77075
Gm6681	0.969762	1.208975	0.404114	0.96433	0.846625	1.270475	0.16705	1.61579	0.770224
Farp2	1.275416	1.681893	1.190526	0.859606	0.769305	0.393155	1.142445	0.742085	0.76987
Ptx3	1.384509	1.121919	0.923114	1.175977	0.796649	1.173655	0.864538	2.471604	0.769744
Gm15784	1.098701	0.765723	0.701662	0.342243	0.642963	0.440694	1.012429	0.768799	0.769599
Gm52549	1.076467	0.872301	0.471009	2.140875	1.473747	1.493225	1.807947	1.024902	0.769477
Acvr2b	0.796431	0.928485	1.216313	0.84013	0.932765	1.114725	1.134614	0.952974	0.769327

Gm6685	0.749163	0.747169	0.922154	0.343831	0.603726	0.106585	0.416929	0.164602	0.768943
Rpl31-ps10	0.461023	0	1.037423	0.11461	0.878147	0.746095	0.119123	0.329205	0.768943
Gm38794	0.773842	1.049624	0.68106	1.415894	0.560119	0.687	0.73582	0.766245	0.767042
Ahrr	0.437347	0.418735	0.734828	0.20875	0.616456	0.4368	0.723231	0.28315	0.766968
Gm30716	0.080462	0	0	0.160022	0.076631	0	0	0	0.766871
Tdg-ps2	0.440288	0.57276	0.631792	0.989859	0.58341	0.407165	1.483892	0.473876	0.766288
Anapc15-ps	0.401725	0.734535	0.602658	0.199737	0.510131	0.86684	0.692005	0.063747	0.765759
Mir7079	1.6069	0.801311	4.017718	0	3.060789	2.972022	4.152029	2.29489	0.765759
Mir7019	1.6069	0	0.803544	0	0	0	2.491218	0	0.765759
LOC118567590	0.80345	0.600983	0.502215	0.998687	2.008643	1.207384	1.245609	0.860584	0.765759
Gm51266	0.80345	0	0.803544	0	0	0	0	0	0.765759
Megf11	1.104411	0.884514	0.836772	0.898547	0.876524	0.773731	1.141463	0.430162	0.765528
Nsg1	0.229462	0.183081	0.711415	0.798619	0.284098	0.509279	0.426889	0.21847	0.765442
Casp12	0.719975	0.856146	1.024699	1.211594	1.028544	0.717026	1.316538	1.212784	0.765377
Gm41553	0.802914	0.758631	0.86112	0.619824	0.311912	0.258902	1.026396	0.442578	0.765249
Fam222a	0.494064	0.51328	0.247061	1.064475	1.117535	0.761491	2.042558	0.587996	0.765191
Adra1a	1.284457	0.777772	0.619364	1.756235	0.983011	1.442359	1.303844	0.742492	0.765126
Gm38426	0.661101	0.89482	0.519497	0.46957	0.674599	0.786042	0.488058	0.629433	0.765107
Zfp940	1.123202	1.180224	0.8425	1.894759	0.821397	0.649189	0.974315	0.725663	0.764651
Acer2	1.792891	1.882229	0.943736	2.205101	1.437919	1.308954	1.609221	1.302716	0.764456
Gm14439	0	0	0.057277	0	0.054544	0.052962	0	0	0.764177
Acsbg3	0.600722	0.85589	1.172975	1.22316	0.49039	0.793613	0.798269	0.898773	0.763389
Gm6767	1.1843	0.638458	1.344497	1.114009	0.94501	0.769603	0.760886	0.883771	0.762665
Ubash3a	0.064865	0.129385	0.043248	0.516014	0.329476	0.439892	0.067041	0.144102	0.762474
Myoc	2.078927	1.967065	0.959616	3.551485	1.116894	2.44013	0.853961	2.258481	0.762078
Gm18753	0	0	0	0	0	0.067192	0	0.27671	0.761745
Obsl1	0.559335	1.163507	0.927005	1.716279	1.050188	1.049289	0.644169	1.125945	0.761565
Gm32738	1.286163	1.017345	0.443556	0.529224	0.929255	1.148389	1.054283	2.153525	0.760859
Apom	0.362815	0.217109	0.725713	0.432939	0.345541	0.067104	0.824971	1.312653	0.760748
Dnal1	0.673142	0.776848	0.807864	0.411185	0.494558	0.613608	0.715604	0.659209	0.760712
LOC115488416	0	0	0.398881	0	0	0.36883	0.412216	2.27838	0.76025
Gm23582	0	0	0	1.983004	0	0	0	0	0.76025
Slc22a17	1.087495	1.518439	1.329315	2.210868	1.19683	1.16212	1.074025	1.656642	0.760085
Gm36518	0.187449	0.18695	0.281206	0	0.847991	0.650051	0.532779	0.490792	0.759286
Gm46967	1.039325	1.499702	0.707708	0.747641	0.926661	0.858887	0.617091	0.757945	0.758734
LOC118568757	0.318152	0.317305	0.795474	0	1.287771	0.073554	0.328826	0.075728	0.758069
H2ac8	1.390919	1.783564	2.285347	0.889154	2.838635	2.848188	1.745638	1.797253	0.757525
Nrarp	0.987665	0.556759	0.98778	2.540734	1.451859	1.350187	0.310679	1.349203	0.757159
LOC118567659	1.388431	0.989096	1.289407	0.197236	1.416778	0.275138	1.537514	0.094423	0.75617
6330409D20Rik	0.644053	0.197642	0.247741	0.443385	0.802124	0.549784	1.126502	0.801878	0.755495
Neo1	1.095706	0.84478	1.445567	0.996839	0.932524	0.847989	0.666629	0.665885	0.755455
Whrn	1.803308	1.471506	1.584909	0.869434	1.275077	1.238098	0.818946	1.092589	0.755192
Trgv2	0	0	0.158413	0	0.150853	0	0	0.603228	0.75482
LOC115487056	0	0	0	0	1.508532	0	3.274172	0	0.75482
Mir5623	4.751834	0.789864	4.752386	4.725216	0.754266	0	1.637086	0.754035	0.75482
Mir6912	0	0	1.584129	0	2.262797	0	1.637086	0.754035	0.75482
Gm23119	0.791972	3.159456	2.376193	2.362608	1.131399	2.929565	1.227814	1.508071	0.75482
Mir1949	3.959862	0	2.376193	8.662897	0	0	0.818543	0.754035	0.75482
Gm30604	0.723105	0.652496	0.757627	0.95874	1.016619	1.146351	0.427066	0.721251	0.75482
LOC115488404	0	0.789864	0	0	0	0	0	2.262106	0.75482
Tnfrsf25	0.61942	0.58345	0.825989	0.239536	1.671464	0.636467	1.529369	0.556984	0.754351
Ebf1	0.974005	1.131409	0.56155	2.176386	1.691563	1.038488	0.829033	1.112818	0.753572
Plekhg6	1.193599	1.080536	0.899894	0.401724	0.961882	1.409471	0.854061	0.576953	0.75257
Gm4833	0.970469	0.786407	1.516535	1.326921	0.982031	0.841368	0.87765	1.212726	0.751517

Rpusd2	1.230757	1.162877	1.014953	0.91253	0.760876	0.748793	0.848033	1.171802	0.751145
Adgrl1	0.98504	0.927402	1.229473	1.073557	1.11076	0.969234	1.058812	0.802804	0.751065
Gm13238	0.93113	0.142869	0.644704	0.641018	1.023229	0.066237	0.370142	0.818333	0.750919
Gm18959	1.477035	1.080276	2.658973	3.818781	0.656466	1.183794	2.340771	0.843769	0.750798
Gm30338	0.157494	0	0.315026	0	0.149996	0.145646	0	0.14995	0.750531
Rapsn	0.34221	0.443689	0.4107	0.884763	0.032592	0.379758	0.42443	0.358399	0.750161
Abra	0.786579	0.564829	0.314668	0.594451	0.149826	1.076557	0.162594	0.269604	0.749679
Gm24265	3.145422	0.392131	0	0.390975	2.246749	2.908788	1.625475	1.123031	0.749467
Gm51946	0	0	0	0	0	0	0	0	0.749467
Gm18720	0.550059	1.018818	0.668006	0.507908	0.280645	0.109002	1.05581	0.43019	0.748935
Arhgap32	0.956123	0.83438	0.640335	0.823432	0.528474	0.528937	0.979378	0.633975	0.748543
Pdzd2	0.8512	0.717203	0.535731	1.729348	1.257942	1.228246	0.940431	0.803439	0.748326
LOC118568773	0.47874	0.82124	0.283447	0.182806	0.605497	0.481683	0.49876	0.692827	0.748302
Zfp93	0.897177	0.149131	0.859895	1.226708	0.462833	0.967961	0.772731	0.462692	0.748203
Rsph1	0.391986	0.893583	0.224018	1.058003	0.213328	0.310711	0.347261	0.159947	0.747196
Gm41291	0.671524	1.22785	0.559669	0.296783	0.390838	0.793506	0.53982	0.639357	0.746693
Gm36856	0.704721	1.249502	1.566229	0.467182	1.118614	0.724116	1.699517	0.96917	0.746291
Gm36874	1.566047	1.40569	1.09636	0	0	0.724116	0	0.149103	0.746291
Lrrc63	0.65774	0.156188	0.626492	0.155727	0.626424	0.318611	0.323718	0.715694	0.746291
Zfp712	0.949569	0.765989	1.061406	1.027566	0.531976	0.723167	0.750503	1.050331	0.745313
LOC115486414	1.373519	1.110061	0.615787	0.73001	0.879601	0.350396	1.370649	0.338205	0.744824
LOC115490268	1.561636	5.451173	1.561817	1.552888	1.487285	2.166228	1.614028	7.434151	0.744189
Siglec15	1.75684	1.022095	1.415397	0.727916	0.046478	0.496427	0.958329	0.232317	0.744189
Mir6995	0	0	0	0	0	0	0	0	0.744189
n-TGgcc6	3.123271	0	0	0	0	0	0	0	0.744189
Epb41l3	0.589428	0.683187	0.557632	1.077205	0.910321	0.824992	0.954967	0.743201	0.743975
Acsbg1	0.379576	0.418414	1.0989	0.397315	0.38053	0.295596	0.371663	0.285311	0.74258
Gm7204	1.258594	1.434564	1.07892	0.476779	0.684955	1.71815	0.743325	1.540677	0.74258
Frs3	0.830922	1.424345	0.77908	0.413134	0.37095	0.960513	0.805124	0.29667	0.742446
Ttc39a	0.803814	0.576203	0.778786	0.599484	0.837314	1.022096	1.142325	0.478319	0.742166
C030034L19Rik	0.389104	0.498945	1.074793	0.865974	0.670571	0.599719	1.43628	0.58216	0.741701
Pla2g3	0.138943	0.194002	0.361293	1.381642	0.979222	2.36421	2.010456	1.428698	0.741578
LOC118567557	1.688565	1.021795	1.347214	4.641126	1.391339	1.614168	1.725607	1.535424	0.741387
Tspan12	0.376276	0.475348	0.351232	1.322063	1.146757	1.229489	0.674094	1.361358	0.741158
1700121C10Rik	0.647641	0.645917	0.259086	0	0.863529	0.718702	0.535495	0.123324	0.740711
Gm46379	0.777169	0.172244	0.259086	0.772816	0.657927	0.878413	1.070991	0	0.740711
Gm9465	0.476186	0.34994	0.200523	0.12461	0.811552	0.509893	1.346969	0.190895	0.740488
Zfp647	1.002044	1.374142	1.227646	1.145895	2.027963	0.741327	0.880313	1.025597	0.740153
Wdr35	1.438132	1.167751	1.400114	0.923854	0.715133	0.835625	0.762921	0.727031	0.739918
D130007C19Rik	1.692765	0.668269	1.939852	2.349583	0.873259	0.945772	1.129922	1.175182	0.739455
2410004P03Rik	2.326073	2.642085	3.231032	1.413526	1.1692	0.836531	0.868152	2.399203	0.738985
Gm46043	0.086084	0.17171	0.172188	0.085602	0.163971	0.079608	0	0.163921	0.738411
Particl	1.175872	0.972517	1.147326	1.511515	1.256459	0.928276	2.312077	1.856807	0.738029
4930577N17Rik	0.774181	0.77212	1.380221	1.907874	0.577034	0.466916	0.974101	1.121668	0.737863
Fam135a	0.698077	1.349593	1.138535	0.929116	0.675069	0.327746	0.987897	0.685088	0.73698
Gm14040	0.579492	0.337137	0.193186	0.672287	0.367935	0.401922	0.299467	0.183911	0.73641
2810408A11Rik	0.408969	0.36256	0.454463	0.271119	0.649163	0.420224	0.563587	0.216322	0.736259
Gm46170	0.154424	0	0	0.153559	0.588285	0.71403	1.596045	0.735132	0.735897
Gm39954	1.691081	0.733295	1.029474	1.242928	1.960691	1.121899	1.101885	0.665031	0.735799
4930568A12Rik	0.241245	0.433085	0.386037	1.007553	0.275711	0.401572	0.398942	0.643128	0.735769
Slc4a3	0.740434	1.304095	0.92959	1.347248	0.780201	0.990674	1.107208	0.374982	0.73573
Mir6966	3.079892	2.30377	1.540125	0.76566	2.199942	2.136141	2.387417	0	0.733853
Gm23123	1.539946	0.383962	0	0.38283	0	1.780118	1.989514	0.366545	0.733853
Gm22442	0	0.383962	0.385031	0.38283	0	2.136141	1.591611	0	0.733853

Mir7027	0.769973	1.535846	0.770063	0	0	2.848188	1.591611	2.932359	0.733853
n-TEttc4	0	0	1.540125	0	0	0	1.591611	0.73309	0.733853
n-TQttg6	0	0	0	0	0	0	1.591611	1.46618	0.733853
n-TPtgg5	0	0	0.770063	0	0	2.136141	0.795806	0.73309	0.733853
Rpl35a-ps5	0	0.383962	0.128344	0.63805	0.244438	0.474698	0.265269	0.488727	0.733853
LOC115488050	0	0	0	0.76566	0	0	0	0	0.733853
n-TDgtc13	0	0	0	0	0	0	0	0	0.733853
n-TDgtc15	0	0	0	0	0	0	0	0	0.733853
n-TDgtc8	0	0	0	0	0	0	0	1.46618	0.733853
n-TEttc6	0	0	0	0	0	0	0	0	0.733853
n-TMcat8	6.159785	0	0	0.76566	2.933256	2.848188	0	0.73309	0.733853
n-TWcca7	0	0.767923	3.08025	3.06264	2.933256	0.712047	0	2.19927	0.733853
Brsk1	1.539946	1.131676	1.580655	0.926852	1.389437	1.573999	1.884803	1.389012	0.733853
Fam171a2	0.876562	0.909911	0.697753	1.245217	0.902977	1.224197	0.721078	1.226311	0.733142
Gm8520	0.177496	0.413056	0.532551	0.529507	0.39444	0.328286	0.672655	0.225325	0.73307
Cdkl5	0.478209	0.681337	0.683235	0.577429	0.260252	0.284292	0.370689	0.146347	0.732496
Trabd2b	0.512261	0.652814	0.711557	2.179065	0.840224	0.921129	1.117723	0.75868	0.732346
1700110C19Rik	0.230352	0.382898	0.153586	1.145309	1.462565	1.491157	0.793601	0.146212	0.73182
Lrrc14b	0.666226	0.704722	0.787449	0.421587	0.692189	0.504086	0.751176	0.884193	0.73118
Slc5a3	0.54864	0.861301	0.863701	0.853711	0.83216	0.591926	1.029085	0.667459	0.731096
4933417D19Rik	1.082598	1.48461	0.270681	0.358845	0.472567	0.500577	1.11892	0.042947	0.730867
Kcnip3	1.229717	1.005281	1.008082	0.88204	0.806379	1.155846	0.854261	0.268711	0.730117
Zfp532	0.644746	0.974591	0.594444	1.202127	0.738777	0.642822	0.885032	0.767326	0.729719
Gm41914	0.633578	0.719654	0.774463	0.463771	0.435798	0.5615	0.763973	0.485934	0.729659
Polq	1.905059	2.074222	1.589121	1.108507	0.705143	0.753932	1.556263	0.712848	0.729448
Tshz3	0.476958	0.564188	0.621227	0.992693	0.390866	0.656485	0.401247	0.559718	0.729448
Slc12a2	1.496488	1.797788	1.326586	0.811694	1.352357	0.99075	0.905168	0.939075	0.729351
Uty	2.676957	4.484788	4.958881	1.232633	3.039311	5.670631	6.024196	5.963766	0.728965
Ptprv	0.628219	1.262174	1.156422	1.077381	1.127249	0.934584	1.317412	0.615463	0.728911
1810019D21Rik	0.241385	0.521608	0.241413	0.600082	0.842939	0.44645	0.291064	0.383037	0.728527
Il34	0.286584	0.698673	0.477695	1.171579	1.061431	0.883413	0.789863	0.818568	0.728373
AI429214	0.67918	0.5927	0.905679	1.435173	0.565988	0.680425	1.111447	0.296379	0.728234
Gm52551	0.916332	1.066207	0.458219	1.063065	0.290901	1.836022	1.578457	0.145406	0.727788
Dnah2	0.391466	0.657213	0.463288	0.506055	0.633807	0.717997	0.788969	0.72058	0.727548
Gm32448	0.416306	0.0692	1.249063	0.551965	0.660809	0.064164	0.932258	0.330303	0.727424
LOC118567916	0.864709	0.839712	0.768087	1.148372	0.666416	0.699699	1.187706	0.937031	0.726548
Stamos	0.069298	0.276452	0.554445	0.068909	0.461988	0.640842	0.501358	0.065978	0.726514
Gm16907	1.383946	1.380261	0.902678	0.897518	0.916908	0.797575	1.803519	0.859338	0.726419
Ildr1	0.417751	0.465656	0.466953	0.781951	0.468073	0.159074	0.78734	0.327551	0.726046
Grtp1	0.761744	1.519433	0.973453	1.052052	0.644868	0.704437	0.612345	1.087883	0.72601
Amigo2	0.29291	0.681637	0.371062	1.534017	1.301832	1.047379	0.201825	0.929596	0.72584
Gm2237	0.076151	0	0.07616	0	0.435153	0.070422	0.39353	0.072503	0.725788
Sdk2	0.741401	0.879318	0.828328	0.790383	0.407122	0.611503	1.014796	0.546903	0.725718
Magef1	1.940522	2.087149	2.473502	1.286435	1.485753	1.442665	2.202257	0.869444	0.72529
Rxrg	0.297698	0.362885	0.165407	1.085446	0.535547	1.009441	0.273499	0.283438	0.725096
Gm40191	0.855819	0.758703	0.47551	0.851025	0.543382	0.175874	1.179376	1.539111	0.725041
Zfp418	0.677576	1.392501	0.94461	0.755451	0.860422	0.645589	0.870081	0.997002	0.724068
Dusp9	2.546044	1.720148	1.60173	0.898374	0.449766	0.873444	0.827638	0.762413	0.724068
Gm17745	0.759426	0.504936	1.392442	0.566379	0.662996	0.819342	1.373582	1.144825	0.7238
Gm39673	0.645512	0.340832	0.835465	2.038963	1.15723	0.98321	1.098866	0.795352	0.7238
n-TRacg3	0	0	0	0	2.893074	4.213758	0.784904	5.78438	0.7238
Radx	0.835368	1.014921	1.336744	0.709861	0.810061	0.57588	0.643621	0.520594	0.7238
Gm32211	0	0	0	0.151034	1.15723	1.545044	0.156981	0.289219	0.7238
Mir7051	0.759426	0.757404	0	0	0	2.809172	0	0	0.7238

Mir7053	0	0	0	0	0	0	0	0	0.7238
n-TKctt21	0.759426	0	0	0	0.723269	0	0	0	0.7238
Xk	0.307806	0.394697	0.637672	0.961971	0.32456	0.254151	0.568094	0.261662	0.722939
Gm14018	1.011029	1.680561	2.612127	1.256706	1.283857	1.168709	1.741581	1.684547	0.7227
Gm5822	0.916071	0.535577	0.947769	1.256468	0.391101	0.642668	0.522375	0.421057	0.722563
H4f16	0.252565	1.259464	0.126297	0	2.285133	1.751733	1.044155	0.480934	0.722151
Gm36328	0.895273	0.755522	0.206625	0.342407	0.065588	0.191059	0.284711	0.262273	0.722002
Gm52255	0.15147	0	0.605951	0.451865	0	0	0	0.721072	0.721822
A330009N23Rik	0.799183	0.922906	0.757209	1.212973	0.640954	0.661264	0.739049	0.480569	0.721603
Prex2	1.062072	0.81803	0.578423	1.432563	0.831238	0.952998	0.271709	0.780924	0.721603
Efcab2	0.983213	0.724132	0.80179	0.857372	0.460997	0.531558	0.812959	0.633678	0.720838
Stx1a	0.5444	0.935082	0.937687	1.142851	0.748916	0.811104	1.031552	0.518322	0.720641
Enho	0.400114	0.886776	0.978171	1.149411	1.143194	1.850066	0.781128	1.312154	0.720317
Gm5492	0.849987	0.282575	0.283362	0.657398	0.629626	0.611366	0.390446	0.719352	0.720101
Acoxl	0.426149	0.115913	0.251844	0.077048	0.184481	0.268697	0.400405	0.110655	0.720006
Gm5388	0.965235	1.213795	0.629574	0.667707	0.279781	0.659762	0.433747	0.599347	0.719965
Ptpdc1	1.061689	1.058862	0.869918	1.208379	0.779675	0.449506	0.594926	0.828151	0.719291
Thrsp	0.352147	0.301037	0.150938	1.700849	0.622851	1.814363	0.623935	0.526867	0.719202
4933404O12Rik	1.466246	1.838373	1.941256	1.555235	0.66497	0.761908	0.865965	0.638176	0.718695
Prok2	0.041872	0.04176	0.167506	0.208186	0.438659	0	0.043276	0.039866	0.718333
Sdcbp2	1.150003	1.502888	0.515578	0.63093	0.490974	0.366719	0.327886	0.641847	0.718105
A930007A09Rik	0.69751	0.876522	1.018482	0.568754	0.744017	0.632134	0.634402	0.637534	0.717972
Prkn	0.22597	0.300492	0.527325	0.711565	0.322818	0.487597	1.128833	0.753011	0.717899
Gjc1	0.391469	1.231347	1.174544	1.706827	0.458869	0.501256	0.591343	0.946128	0.717509
Tead4	0.677221	0.337709	0.959508	0.91661	0.394153	0.713255	0.641613	0.913439	0.717169
Scpep1os	1.503869	1.840743	1.162215	1.087596	1.302062	0.88501	2.896693	1.757246	0.71666
Apol9a	0.037585	0.112455	0.639021	0.747492	0.322161	1.007969	0.194231	0.429417	0.716439
Klra4	0.115616	0	0.23126	0.804781	0.715727	0.801888	0.059748	0.330234	0.716253
Gm33977	0.450716	0.449516	0.751281	1.195177	1.0016	0.138936	0.931675	0.286084	0.715954
Bcl2l15	0.375597	2.846935	0.676153	0.373493	0	0.416808	0.310558	0.143042	0.715954
Gm52307	0.750431	1.403311	0.093815	0.093278	0.446689	0.260241	0.678657	0.08931	0.715227
Zscan30	0.689175	0.545829	0.871705	1.169066	0.945935	0.843522	0.817047	0.636863	0.714802
Gm33534	0.890383	1.028225	1.124825	0.698997	0.892622	0.823398	1.017124	0.758497	0.714622
Gm33858	0.89517	1.162699	1.061836	0.351922	0.674109	0.462042	0.796104	0.5748	0.714287
Fads6	0.749163	0.544234	0.8325	0.974895	0.845623	0.650037	0.650027	0.757306	0.714019
Mir191	1.498326	1.494337	0	0	0.713495	1.385605	0	0	0.714019
n-TIaat4	0	0	0	0	0	0	0	0	0.714019
LOC118568783	1.011548	0.982651	1.103635	0.600916	0.925852	0.643879	0.638153	0.637892	0.713681
Nutf2-ps2	0.49888	0.497552	0.436571	0.248043	0.475128	0.288343	0.386713	0.534356	0.713216
Rab30	0.489075	0.200848	0.402814	1.144318	0.958978	0.372467	0.148672	0.219128	0.712907
D830035M03Rik	0.514181	0.326335	0.046749	0.185928	0.133555	0.432271	0.531432	0.178018	0.712815
Atp1a2	0.961508	1.598247	0.9171	1.248271	1.305763	1.720714	0.699317	1.262982	0.712758
Gm15423	0.961354	0.248576	0.712197	0.778937	0.373015	0.230489	1.251209	0.339001	0.712643
LOC118568498	0.263781	0.701544	0.747468	0.524608	0.418704	0.406561	0.817894	0.627864	0.71232
Gm1976	1.19543	0.983604	0.717341	0.53493	0.711571	1.326595	1.019318	1.02435	0.712094
Tmem45a	0.37346	0.455236	0.083001	1.19663	0.592799	0.345364	0.514653	0.513602	0.711881
Sox7	1.052407	0.474015	0.543241	1.755439	1.050799	1.459849	0.982452	0.856543	0.711833
Nfatc4	0.322413	0.744653	0.492161	0.354355	0.193934	0.360927	0.368307	0.565469	0.711615
Samd12	0.949979	0.473725	0.542908	1.889315	1.034	0.627508	1.402644	2.067367	0.711397
Ggnbp2os	1.705787	3.296163	2.665601	3.604492	2.538394	1.084502	1.212073	2.740628	0.711273
Cadm4	0.720309	0.821019	0.643208	1.125574	0.808517	0.951599	0.638122	0.734791	0.711037
Ubap1l	0.706718	0.822309	0.431933	0.54659	0.261749	0.944017	0.649269	0.485958	0.710985
Gm6397	0.870104	0.909111	0.663014	0.57682	2.249268	0.766329	0.984943	0.788976	0.710817
Cd27	0.701748	0.481167	0.394779	1.046725	0.877192	1.662945	0.906614	0.876924	0.71063

1700028E10Rik	0.34398	0.743305	0.286683	0.627097	0.0546	0.424136	0.8888	0.272919	0.710327
Fam167a	0.337437	0.224359	0.520276	0.307584	0.160686	0.260043	0.18891	0.321273	0.710216
Angptl6	1.134283	1.378727	0.992613	2.150114	0.945244	1.081729	0.732711	1.012451	0.709454
B3galt1	0.24786	0.412001	0.661037	1.396674	0.472119	0.305618	0.170784	0.078662	0.708698
Gm15327	0	0	0.594579	0.147795	0.707756	0.137446	1.228912	0.424524	0.708276
Cnih2	0.412793	0.782218	0.123852	1.231441	1.415301	0.877997	1.493247	0.510925	0.708171
Sema4f	0.385284	0.256172	0.271157	0.610163	0.597976	0.369495	0.176982	0.027172	0.707219
Nrap	0.532958	0.708719	0.742047	1.153469	0.706635	0.763454	0.637245	0.92531	0.707155
Gm41252	0.049454	0.493225	0.642978	0.393417	0.612294	0.686004	0.664473	0.659192	0.707012
Cyp2d22	1.443073	1.819029	1.824096	1.733946	1.527075	1.204765	0.497163	1.965508	0.706791
Mir6939	1.478348	0.737206	0.73926	0.735034	3.519907	0	3.055894	2.111299	0.704499
Mir7081	0	0	0	0	0	0	0	2.111299	0.704499
1700025N23Rik	2.396989	1.716334	2.089926	2.566914	2.458472	0.341025	4.192537	0.81924	0.702937
Clcn2	0.753813	0.637401	0.508064	0.635523	0.312141	0.51525	0.982348	0.811318	0.702833
Tyw3	0.750269	0.897926	0.709428	0.623983	0.649589	0.618135	0.91643	0.896159	0.702072
Sult2b1	0.392829	0.636648	1.031297	0.097657	0.467658	1.362287	0.91352	0.280509	0.702003
Kdm5d	5.661949	7.469883	8.382442	2.305293	5.753286	10.82243	12.27979	8.362	0.701099
Gm8253	0.104996	1.047168	0.210017	0	1.49996	0.582584	1.193708	0.599801	0.700496
Gm2445	0.96454	0.778739	1.378074	1.141829	0.787386	1.401677	0.949428	0.918336	0.700413
Kif5c	0.532748	0.692338	0.912235	0.738458	0.891764	0.679285	0.825932	0.83001	0.700088
4933421O10Rik	0.777573	0.919114	0.61925	0.973681	0.575985	0.479383	0.788778	0.877423	0.699924
Ribc1	0.734279	0.878789	0.881237	0.511116	1.223809	0.509279	0.79686	0.943792	0.699833
LOC118567639	0.18357	0.732324	0	0	0	0	0	0	0.699833
Gm13410	0.733874	0.426953	0.611633	0.395288	0.378589	0.395887	0.537268	0.378473	0.699447
Ndnf	1.053371	1.074174	0.864101	0.776776	0.766504	0.952234	0.758428	1.036718	0.699385
E130112N10Rik	0.692976	0.691131	0.40768	0.648559	0.659983	0.716236	0.547702	1.047887	0.699319
Mall	2.029253	0.899489	1.747615	4.93261	2.227902	3.518605	0.233037	1.449036	0.69841
Cpxm1	1.076245	0.616622	0.801552	1.593939	0.436172	1.609385	1.13602	1.438927	0.698388
LOC100039029	0.610551	0.24357	0.122124	1.214263	0.348889	0.225848	0.757242	0.581305	0.698292
Gm34086	0.488287	0.278279	0.348817	0.312141	0.365388	0.419299	0.486646	0.481501	0.698072
Gm2253	0.609209	0.729105	0.731136	0.726956	0.116041	0.112676	2.140805	0.116005	0.696757
Cpeb1	1.198112	2.754397	1.299798	1.050048	0.599545	0.582157	0.923484	0.599361	0.696757
Gm18666	0.609209	0.243035	0	0.121159	0.232082	0.563378	0.314824	0.348016	0.696757
Gm46494	0.121842	0.121518	0	0	0	0	0	0	0.696757
D5Ertd605e	1.513845	1.457753	1.044153	0.363364	0.994324	1.689603	0.647435	1.49103	0.696538
Cd209b	0.417613	1.145377	0.991945	0.259546	2.137797	1.013762	0.323718	0.49701	0.696538
Gm10184	1.123934	0.448377	0.477728	0.391174	0.026761	0.337798	0.319452	0.802572	0.696286
Dpep1	1.904461	4.293142	1.5394	2.672064	0.720546	0.675523	0.269638	0.546454	0.69621
Lekr1	0.579316	0.40193	0.629765	0.350652	0.575723	0.535734	0.520654	0.815358	0.696177
Zfp469	1.412243	1.048871	1.51544	0.866503	0.605048	0.654984	0.984913	0.862338	0.695498
Itga3	0.598037	0.691876	0.933047	1.3321	1.013824	1.106092	0.568653	0.683268	0.695379
Gm46987	0	0	0	0	0.694718	0	3.015684	0	0.695229
9530077C05Rik	1.321038	1.135794	0.82005	0.86066	0.650763	0.42126	0.376651	0.650565	0.694658
Gm46842	0.504492	0.111811	0.280306	0.334444	0.480473	0.259188	0.289676	0	0.694526
Zfp507	0.44077	0.484453	0.395839	0.259403	0.539723	0.490796	0.316101	0.171288	0.694439
Gm16061	0.437094	0.43593	0.655717	2.462991	0.693806	0.741053	2.71055	1.04039	0.694315
Gm40453	1.911657	1.603938	1.183544	0.995735	0.520183	1.262743	1.129025	0.606695	0.694087
Gm32645	0.970469	0.120986	0.606614	0.442307	0.500643	0.074788	0.710479	0.731486	0.693708
Igf1os	0.121309	1.209857	0	0.482517	0.808731	1.794919	1.629921	1.03948	0.693708
Gm13807	0.342475	0.64043	0.428143	0.297987	0.448482	0.237532	0.176982	0.203793	0.693618
Catsper2	1.454643	0.805984	1.252755	1.084871	1.346903	1.494676	1.04406	1.61579	0.693202
Gm27252	1.388431	0.527518	2.049827	1.347781	0.31484	0.947698	1.571681	0.692435	0.693156
Gm4262	0.614737	0.529495	0.726591	0.583507	0.612081	0.516808	1.415122	0.957746	0.692426
Tmem181c-ps	0.465981	1.06617	0.795002	0.5724	0.482954	0.608365	0.920734	0.600246	0.692307

LOC108168764	0	0	0	0	0.230562	0	0	0	0.692193
Gm26536	0.508273	1.303508	1.198211	0.469324	0.795264	1.040792	1.425884	0.760455	0.692042
LOC115490443	0.314577	0.941217	0.701829	1.106882	0.94489	0.537066	0.525211	0.691172	0.691891
Tas1r3	0.867699	0.786717	0.220894	0.564767	0.781311	0.671115	0.750059	0.435598	0.691668
Gm3744	1.139848	0.723427	0.518173	1.442589	1.579024	0.766615	0.963892	0.394635	0.69133
Rslcan18	0.576511	0.983024	1.004362	0.462324	0.779315	0.550338	0.845727	0.672839	0.691264
Gm13217	1.359887	0.723342	1.40538	1.532572	1.295142	1.215662	1.077559	0.820006	0.69125
LOC102633548	0.56405	0.482184	0.40294	0.520827	0.805793	0.260808	0.374769	0.61375	0.691187
1700012B09Rik	1.1281	0.321456	0.40294	0.641018	0.460453	0	0.333128	0.767187	0.691187
Gm46142	0.24156	0.602293	0.966353	1.080932	0.575148	0.670162	0.249665	1.494928	0.690685
Ovgp1	1.053872	1.423319	0.61483	0.371156	0.773683	0.93398	0.521922	0.89887	0.690548
Gm5507	0.4073	0.857566	0.588391	0.675031	0.387908	0.46036	0.748382	0.818667	0.690121
Sh3d21	1.025514	0.842292	0.361988	1.139742	1.005414	1.450437	0.997571	0.97639	0.689933
Patj	0.54773	0.64382	0.626049	0.700279	0.596173	0.596974	0.62676	0.446993	0.689832
Ncr1	0.228454	0.341768	0.87584	2.688224	1.414248	2.007034	0.865767	0.398769	0.689499
Gm40085	0.065607	1.04692	0.328074	2.152909	0.749803	0	1.763016	0.312322	0.687824
Rab11b-ps1	1.246536	1.635813	1.64037	2.218149	1.124704	1.274101	1.288358	0.749574	0.687824
Flt4	0.835511	0.520804	0.87359	1.510602	0.696265	0.728754	0.245325	0.442942	0.687728
Fkbp14	0.927403	0.328865	0.556506	1.086156	0.785109	0.781399	0.830715	0.313948	0.687475
Dipk2b	1.153758	0.40274	0.548099	1.864354	0.576884	1.200329	0.17887	0.549245	0.687271
Pbld1	0.629978	1.97466	1.080088	1.252898	1.371392	1.997431	3.348585	0.942544	0.6862
Gm22888	1.43995	1.436116	0	2.863768	0	0	1.48826	0.685487	0.6862
Gm8013	0.959966	1.436116	0.480039	0.238647	2.171371	1.442589	0.496087	2.05646	0.6862
Gm40491	0.397228	0.173324	0.099318	0.271564	0.378315	0.091836	0.487533	0.3782	0.6862
Gm8712	1.047236	1.436116	0.785518	1.04137	0.62336	0.66581	0.473537	1.308656	0.6862
Pvrig	0.775358	0.994234	1.163171	1.321739	1.846105	0.460946	0.343445	1.212784	0.6862
Gm4986	0.629978	0.089757	0.090007	1.163406	0.685696	0.499358	0.186032	0.599801	0.6862
A630034I12Rik	1.097249	0.843171	0.989438	1.037442	0.582464	0.532302	0.390415	0.633664	0.685755
Ccr8	0	0.382633	0.19185	0.381505	0.137021	0.354792	0.049566	0.410936	0.685606
Tekt2	1.006657	1.07569	1.61803	1.251273	1.164172	0.930925	1.523488	1.266505	0.68531
LOC118568382	0	0.318219	0.079776	0	0	0.073766	0.082443	0.075946	0.684225
Tbx21	0.391021	0.519973	0.543148	0.734458	0.620673	0.944188	0.942992	0.310242	0.683242
Gm7993	0.6222	0.961843	0.560046	0.835266	0.592577	0.172617	0.932459	0.414677	0.681964
Gm30189	0.847596	1.373676	0.821204	0.895526	0.353168	0.612367	0.246384	0.605246	0.68161
1700120C14Rik	0.285763	1.425012	0.714491	0.710406	0.952552	2.246251	0.738376	0.136037	0.680894
Gimap5	0.285322	0.313019	0.313891	0.425586	0.733691	1.583141	0.383363	0.543309	0.679843
Eldr	0.463469	0.426679	0.320901	0.248162	0.407449	0.593449	0.847495	0.407325	0.679581
Gm14403	0.817013	1.022251	0.891391	0.679493	0.565901	1.085242	1.228253	0.707161	0.679581
Phf21b	1.357944	0.609448	1.052529	1.012753	1.034633	1.349968	0.771927	0.937349	0.679477
Art3	2.302941	1.252805	0.711901	2.040218	0.957074	2.400739	1.557952	0.757452	0.678426
LOC118567611	1.280982	1.135619	0.142348	0.636903	0.677774	0.72393	1.176852	0.474297	0.678272
Gm30984	0.853988	0.496834	0.711739	0.353835	0.474442	0.197435	0.735533	1.151864	0.678272
Gm9115	0.340301	0.617081	0.27846	0.061526	0.058927	0.400526	0.863307	0.412363	0.678159
Fignl2	1.317444	1.03801	1.620645	1.676883	1.066512	1.120865	1.320795	1.066186	0.678046
Mtmr7	1.185353	0.963946	1.021346	0.67096	0.712086	0.674571	1.413602	0.572968	0.677848
Mir5129	0.710744	0	0	4.240579	2.707621	0	1.46918	2.706793	0.677403
Mir677	1.421489	2.835409	1.421654	0	0.676905	0	1.46918	0	0.677403
Mir1960	0	0.708852	0	0	0	0	0.73459	0	0.677403
Kcnh6	0.495367	0.537009	0.430804	0.406924	0.943565	0.478018	0.601028	0.779228	0.677403
Mir1900	0	3.544261	0	1.413526	0	0	0	0.676698	0.677403
Csf1r-ps	0.448589	0.484677	0.373867	0.408903	0.356026	0.432126	0.347729	0.391509	0.676946
Wwc1	0.628271	0.544868	0.259533	0.746985	0.195117	0.581005	0.649349	0.533156	0.676902
Tpsab1	0.101442	0.50586	0.608723	0.201748	0.434755	0.656673	0.262113	0.241457	0.676783
E030018B13Rik	0.273094	0.054473	0.10925	0.054313	0.052018	0	0.508061	0.156007	0.676735

LOC118568522	0.371841	0.404564	0.388788	0.705901	0.33804	0.281345	0.524067	0.225291	0.676576
Tctn2	0.304048	0.44475	0.668983	0.564377	0.405401	0.431133	0.167599	0.424576	0.676164
Gm42199	0.23641	0.943121	0.236437	0.117543	0.225154	0.109312	0.122171	0	0.675958
Gm10777	0.155102	0.972334	0.975043	0.727102	1.034025	1.372868	0.687026	0.696172	0.675778
Zfp128	0.954582	1.370361	1.504364	1.423852	1.184628	1.070021	1.016505	1.225578	0.675459
Arhgef17	0.562494	0.447718	0.670743	0.984228	0.922044	0.700237	0.436024	0.448007	0.675288
Gm41958	0.326907	0.05434	0.490418	0.541794	0.207562	0.050386	0.22525	0.129687	0.675072
Gm44705	1.416301	0.706265	0.809409	0.804781	1.734261	0.748429	0.941026	1.733731	0.67493
Gm33315	0.809315	0.353133	0.961173	0.955678	0.385391	0.888759	0.209117	1.011343	0.67493
Gm17359	0.212406	0.494295	0.354052	0.915272	0.134862	0.916658	1.024485	1.14598	0.674807
Gm5182	1.16813	0.815514	0.913996	1.065926	0.497359	0.305012	0.511336	0.700015	0.674548
Gm35279	0.35386	0.999934	0.058984	0.46917	0.674025	0.327239	1.158151	0.056152	0.67452
9230114K14Rik	0.811416	0.913676	0.445022	0.208225	0.473642	0.484111	1.163274	0.27413	0.673565
4931422A03Rik	0.706217	0.469558	0.824016	1.287479	1.457286	0	0.851563	0.112065	0.673088
Gm36395	0	0	0	0.175565	0.168148	0	0	0.168097	0.673088
Dact3	0.823338	0.918901	0.431322	0.760245	1.138867	1.069581	0.871221	0.989205	0.672612
Gm6133	1.410189	0.351609	0.264441	0.613502	0.671524	1.385605	0.455469	1.594383	0.672018
Gm39170	1.101491	0.966732	1.002474	1.445824	0.702862	0.295401	0.990448	0.492902	0.671884
Zdhhc2	0.557904	0.67356	1.145305	0.963564	0.41948	0.62455	0.515925	0.363439	0.671662
Fam167b	0.352361	0.878556	0.352402	2.102321	0.615238	1.194791	0.424879	0.503223	0.671662
Gm31332	0.286217	0.834407	0.572501	1.072776	0.482275	0.468288	0.568884	0.586938	0.671484
Fxyd4	1.006135	0.200691	0.704377	2.000999	0.862409	0.093044	2.183772	0.766352	0.671256
Pou5f1-rs1	0.844234	0.140331	0.140722	0	0.268013	0	0.581706	0	0.670525
Zfp563	0.625339	0.710296	0.894687	0.647746	0.893349	0.971855	0.412926	0.793846	0.670504
Dok7	0.612701	0.633702	1.180153	1.241103	0.734815	0.545621	0.750526	0.34569	0.67047
Gm15736	0.610888	0.335094	0.458219	0.18224	0.349082	0.536683	0.631383	0.523463	0.669565
Kcnq3	0.614678	0.904965	1.200224	0.844086	0.808426	1.001528	0.665552	0.780311	0.669534
Mecom	0.482833	0.875542	0.658486	1.396739	0.334433	1.38012	0.181466	0.167165	0.669357
LOC115488429	0.701748	0.699879	2.105488	1.395634	0	0.648954	0.725291	4.008795	0.668828
Mir511	0	0	0	1.395634	0	0	0.725291	0.668133	0.668828
Slc25a47	0.350874	0.419928	0.210549	0.523363	0.033417	0.551611	0.290116	0.167033	0.668828
Gm12482	0.14035	0.069988	0.070183	0.139563	0.066834	0.259582	0	0.40088	0.668828
Gm52104	0	0	0	0	0	0	0	0	0.668828
Gm30101	0.501248	0.399931	0.501307	0.697817	0.954767	0.556246	0.621678	0.38179	0.668828
Gm7162	0.161784	1.07569	0.701146	0.589886	0	0.199484	0.445899	0.256724	0.668177
Gm6611	0.525203	0.291002	0.43772	0.464232	0.583564	0.161897	0.241255	0.138901	0.66742
LOC118567753	1.099565	1.23621	0.999721	1.312087	0.875851	1.312652	1.157118	0.932687	0.666898
Gata1	0.437209	0.02907	0.204054	0.869519	1.193659	1.374678	2.138885	0.888033	0.666718
Rasl10a	0.049944	0.846791	0.2997	0.645638	0.523229	0.230934	0.722678	0.52307	0.666418
LOC115488342	0.718462	1.000586	0.750912	0.978329	1.337688	0.688354	1.85307	0.671721	0.666251
4931413K12Rik	0.931732	1.219643	1.106561	1.216048	1.441979	1.077046	1.564861	1.164319	0.666018
4930556A17Rik	0	0	0	0	0	0	0	0	0.666018
Icos	0.306664	0.390805	0.255583	0.237181	0.519224	0.299349	0.369778	0.356858	0.665745
Gm5297	1.046824	0.696025	0.741587	0.52048	1.163148	0.927734	0.676216	1.370434	0.665144
Gm31161	0.791434	0.80812	0.942293	0.655834	0.394823	0.418225	1.110124	0.520289	0.664508
Epor	0.575901	0.846437	0.485026	0.482253	1.645446	1.261363	1.409738	0.519456	0.66444
Gm38800	1.293887	1.488971	1.244266	0.544347	0.568746	1.656756	1.388731	1.184526	0.664025
Gm38875	0	0.462682	0.115993	1.845273	0.883659	1.287047	0.719222	0.772965	0.663231
Chkb	0.632494	0.536188	0.506054	0.503161	0.662618	0.789629	0.980571	0.692525	0.663105
BC037704	0.445062	0.970982	0.445114	0.719175	0.768269	0.90033	0.316246	0.529679	0.662787
Gm14200	0.782042	0.519973	1.129747	1.728136	1.075834	1.205346	0.449044	0.496387	0.662538
Gm16476	0	0	0.138959	0	0	0	0	0	0.662123
Gm33803	0	0	0.115751	0	0	0	0	0	0.661846
Rwdd3	0.480677	0.319598	0.640977	0.531094	0.152597	0.296343	0.331202	0.661052	0.66174

Ttn	0.779008	1.15586	1.257493	0.907829	0.648203	0.616766	0.689316	0.710463	0.661705
Nmrk2	0.391682	0.240393	0.271196	0.389488	0.286949	1.170234	0.467103	0.258175	0.660467
Gm33044	0.692976	0.552905	0.554445	1.10255	0.923976	0.256337	0.143245	1.583474	0.660467
Gm38988	0.230992	0.287971	0.346528	0.057425	0.494987	0.213614	0.119371	0.494836	0.660467
E230016M11Rik	0.629263	0.753105	0.818137	0.782173	0.299652	0.640115	1.073118	1.168284	0.659718
Gas2	1.349616	0.931862	1.453601	0.584999	0.95578	1.024068	1.537961	0.626009	0.659643
Gm7931	0.437094	0.43593	0.29143	0.217323	0.485664	0.370526	0.639991	0.346797	0.6596
Ccdc141	0.499788	0.364258	0.422947	1.108667	0.384456	0.533295	0.456954	0.640564	0.659551
Gm6733	1.239645	0.948822	0.490149	0.487347	0.466758	0.613182	0.893885	0.521512	0.659437
LOC118568512	0.13825	0.275763	0.553062	1.512226	0.658337	0	1.000215	0.131627	0.65882
Gm51871	0.394577	0.688671	0.493279	2.648475	0.187895	0.638562	2.039075	0.751352	0.658117
Gfi1	0.06276	0.333829	0.209225	0.312043	0.4981	0.561039	0.043244	0.239015	0.657975
Adamdec1	0.32178	0.481385	0.666621	0.594244	1.094498	0.998991	0.498863	0.700265	0.657182
Gm13351	1.019137	0.687581	0.764442	1.028332	0.499581	0.374214	1.332152	1.041665	0.657075
Xrcc2	1.430326	2.079623	1.585606	1.353769	0.853443	0.988057	1.371447	1.148515	0.656977
Gm40151	2.238183	0.17171	0.688752	2.054442	0.163971	0	0.889721	0.327841	0.656365
Gm5510	0.476545	0.739319	0.847289	1.421627	0.453856	1.077252	0.820888	0.705783	0.656052
Gm40369	0.88465	0.392131	0.393223	0.977438	0.280844	0.0909	1.117514	0.561516	0.655783
Gm40582	0.211697	0.527833	0.476373	0.605219	0.378033	0.954381	1.367494	1.209336	0.655739
Etv4	0.638065	0.367135	0.711771	0.805316	0.514196	0.794315	0.431193	0.373847	0.654912
Aldh1l1	0.687099	0.522969	0.379757	0.467487	0.361634	0.969833	0.672775	0.482032	0.654867
Gm12447	1.099144	0.822163	1.099271	1.22961	0.719684	0.952925	1.278022	1.046493	0.654739
Trib2	0.392482	0.52844	0.255143	1.365992	0.971868	1.034422	0.730169	0.411049	0.654623
Zc3hav1l	0.814019	0.659629	0.822594	0.935937	0.629901	0.697889	0.955259	1.001075	0.654608
Gm39743	0.940921	1.014504	1.220796	1.213817	0.678147	0.776066	1.866128	0.799001	0.654408
Foxd2os	0.571919	0.912635	0.991442	0.739331	0.907816	0.669931	0.689625	0.562674	0.654108
Gm34235	0.137223	0	0.137239	0	0.392069	0	0.992787	0.391949	0.653928
Gm13015	0.274446	0.273715	0	0.409363	0	0.126899	0.42548	0	0.653928
Gm6505	0.4175	0.178452	0.357899	0.237235	0.227213	0.248201	0.431507	0.312322	0.653717
Gm6014	0.571526	1.596014	0.914549	0.795655	1.197494	1.162765	0.590701	0.870639	0.653659
Gm32364	0.718279	0.759783	0.892511	1.666596	0.652986	0.875591	1.63098	0.766765	0.653466
Gm19510	0.387262	0.297101	1.102336	0.059245	0.283711	0.440773	0.184733	0.510524	0.653015
Usf2-ps1	0.365325	0.728705	0.867748	0.635738	1.000303	0.506762	0.660768	0.999997	0.652851
Mir3473c	1.368841	2.730394	1.369	4.08352	1.955504	1.265861	1.414766	1.954906	0.652314
Cpm	0.286958	0.363246	0.253877	0.296326	0.725284	0.4695	0.365028	0.304737	0.652184
Zpbp	0.664049	0.802765	0.583626	0.34017	0.613269	0.48383	0.499148	0.191588	0.652077
Gm9875	0.28799	0.789864	0.288023	0.107391	0.754266	0.266324	0.409271	0.548389	0.65189
Gm6035	0.940353	0.980479	0.384734	0.892581	0.447791	0.434804	0.839369	0.284871	0.651811
Gm5920	0.918107	0.681877	1.074506	1.107212	0.558125	0.578068	0.343223	0.781136	0.651624
H3c15	1.537573	1.874253	1.196029	1.444018	1.627076	1.500894	1.854019	1.545249	0.651308
Ear-ps9	0	0.085193	0.170861	0	0	0	0.088287	0	0.651308
Ank3	0.491413	0.755924	0.61642	0.679155	0.325232	0.770243	0.430424	0.674055	0.650942
Cand2	0.556277	0.554796	0.417256	0.465158	0.33714	0.514428	0.548806	0.252778	0.650677
Gm8849	0.227516	0.453821	0.379237	0.301655	0	0.490933	0.627064	0.288823	0.650529
LOC118567808	0.25245	0.856046	0.605951	0.552279	0.192345	0.560299	0.182644	0.528786	0.64964
Ptprg	1.185746	1.094337	0.707991	2.305413	1.070299	1.342035	0.567035	0.985722	0.649398
Rarb	1.134758	1.707893	1.299964	0.984786	0.943183	0.648713	1.577987	0.785746	0.648915
Reep6	0.748703	0.712769	0.272287	0.947557	0.486175	0.597962	0.56278	0.518428	0.64871
Gm36445	1.034191	1.601442	1.388154	0.893082	0.647995	0.931219	1.265788	1.010563	0.648471
Rps19-ps4	0.680222	0.113068	0.680301	0.901882	1.079726	0.733889	0.468695	1.295275	0.648312
Gm4895	1.10099	0.419846	0.550559	2.608253	0.308403	0.50908	0.502027	1.079081	0.648122
Cacna1s	0.962528	1.338667	0.856661	0.860544	0.622347	1.249428	0.474593	0.353116	0.648053
Myzap	0.267113	0.266402	0.558574	0.772703	0.485664	0.965614	0.32627	0.878552	0.648028
Alkbh3os1	0.178832	0.677754	0.643872	1.315947	0.272509	0.23153	1.072027	0.95349	0.647684

Adam12	0.953462	0.655561	1.09083	0.847563	0.419637	0.794895	0.671898	0.942176	0.647129
Gm29917	0.4073	0.406216	0.769434	0.540025	0.215505	0.292956	0.374191	0.430877	0.646989
A730081D07Rik	0.493605	0.52306	0.308539	0.276098	0.440723	0.399412	0.095656	0.176235	0.646869
Gm39885	0.542182	0.675923	0.135561	0.134786	0.129092	0	0.140093	0.129052	0.645934
Filip1	0.809852	0.425876	0.441789	1.595989	0.378635	1.034879	0.304372	0.658905	0.645557
Prkg2	0.322502	0.192986	0.290285	0.481043	0.276432	0.507007	0.29999	0.245643	0.645483
Mylk2	0.827434	1.069049	1.109642	0.729299	0.680579	1.286902	0.388725	0.680371	0.645233
Nhlrc1	1.111661	0.819475	0.845928	0.696904	0.828574	0.603409	1.223889	0.43717	0.64492
Ptgis	0.644254	0.642539	0.515463	0.448452	0.552222	0.357472	0.066587	0.122679	0.644733
Gm46597	0.193164	1.252223	0.482966	1.344574	0.367935	0.44658	0.998223	1.103466	0.644359
1700123M08Rik	0.312034	0.155602	0.988223	0.568858	0.742945	0.480932	0.05375	0.396116	0.644359
Gm16116	0.686923	0.651401	0.563117	0.38073	0.428995	0.322829	0.80308	0.568245	0.643966
Gm16209	0.385992	0.769928	0.627309	1.439361	0.78118	1.02624	0.847751	0.321564	0.643798
Ncr3-ps	0.609637	0.905553	0.804296	1.018969	0.728853	0.659735	0.938432	0.802728	0.642851
Gm42281	0.325423	0.231826	0.209225	0.208028	0.154964	0.236453	0.33634	0.199179	0.642467
Prima1	2.327786	1.405172	0.79644	2.253833	0.700092	2.152664	0.063313	0.583232	0.642223
Naalad2	0.530302	0.200121	0.430025	0.855132	0.313952	0.530169	0.35552	0.395732	0.642026
Gm11604	0.067361	0.134363	0	0.267935	0	0.124587	0.069621	0	0.64201
Kcnj10	0.880792	0.568407	0.915439	0.824337	1.381646	1.261721	0.606895	0.937259	0.64195
Gm40443	0.458403	0.600052	0.65903	0.213673	0.586651	0.225206	0.518199	0.668305	0.641695
Gm34542	1.007965	0.670188	0.168014	0.334106	1.599958	0.155356	0.17363	0.319894	0.640453
Gm22042	0	0	0	0	0	0	0	1.279575	0.640453
Ckmt2	0.932673	2.15804	1.231271	2.522659	1.03039	1.449011	1.426666	1.491833	0.640022
Gm31852	0.582625	0.416858	0.50669	0.541577	0.759952	0.44509	0.811624	0.25324	0.639795
Rps12l1	0.670622	1.114727	0.558916	0.444577	0.425795	0.62017	0.693121	1.064163	0.639162
Vmn2r26	0.194292	0.473672	0.259086	0.429342	0.657927	0.878413	1.539549	0.308309	0.637835
Adarb1	0.886314	1.187272	0.869036	1.442993	0.802737	1.052669	0.529872	1.100324	0.637693
Dll1	0.603474	0.471734	0.505672	1.086656	0.776678	1.191563	0.522577	0.823028	0.637344
Gm34346	0.486299	0.272815	0.638341	1.027596	0.549985	0.534035	0.691094	0.694506	0.637293
2610020C07Rik	1.002822	0.615478	0.668626	0.562526	0.930588	0.856042	2.126086	0.73445	0.637186
Skap1	0.579337	0.222229	0.534834	0.620406	0.594197	0.741811	0.506654	0.466726	0.637107
Gm5525	1.139407	0.901262	1.100245	0.898606	1.010321	1.017354	0.690337	1.04742	0.636595
Mir1934	0.667928	2.664601	0	0	0	0	2.071012	0	0.636595
Mir3971	0	0	0	0	0	0	0.690337	0	0.636595
Gm20655	0.9351	0.71056	0.801607	0.575629	0.593719	0.123536	0.18409	0.975098	0.636595
n-TLcag10	0	0	0	0	0	0	0	0	0.636595
Gm19038	0.510407	0.861466	0.4712	0.976054	0.523499	0.689859	1.542014	0.523339	0.636144
Gm51983	0.175548	0.595274	0.351137	0.558607	0.300942	0.292214	0.798326	0.30085	0.635789
Slc39a2	1.072808	1.012116	1.478907	1.32629	1.049345	0.965285	0.86906	0.524512	0.635596
Gm5055	0.095091	0.094838	0.095102	0.094558	0.181127	0.087937	0	0.090536	0.634411
LOC118568330	0.083178	0.622173	0.249563	0.041356	0.316871	0.346141	0.558795	0.316774	0.634207
Gm39008	0.536992	0.331539	0.332462	0.101711	0.511426	0.236473	0.528579	0.462577	0.633659
Guca1b	0.697961	0.961285	0.797763	0.694052	0.981269	0.676189	0.721378	0.917681	0.633542
Gm15801	0.664397	0.389781	0.254064	0.660675	0.428046	0.289136	0.605901	0.29768	0.633229
Mterf2	0.70128	1.288393	0.922845	0.660649	0.738196	1.297044	0.572217	1.229951	0.633205
Gm9923	0.918107	0.584466	0.625167	0.505044	1.11625	0.578068	0.323033	0.743939	0.633006
Cacna1b	0.732404	1.275859	1.367312	0.786565	0.799838	0.487659	0.989115	0.650832	0.632895
Gm10290	0.995893	0.993242	0.71934	1.045332	0.579625	1.023301	1.315224	1.053542	0.632783
Enpp5	0.4031	0.614864	0.332003	1.768419	0.903312	1.184106	0.661696	0.609549	0.632783
Zfp976	0.365088	0.595826	0.343001	0.572068	0.389852	0.255774	0.20582	0.3476	0.632657
Gm36482	0.611449	1.067187	0.45864	1.013374	0.291169	0.141362	0.684627	1.115806	0.631329
Sardh	0.509744	0.78319	0.647587	0.575387	0.524837	0.815386	0.583802	0.314806	0.630267
Smim18	0	0.094192	0	0	0.26984	0	0	0	0.630088
LOC118567997	0.146856	0.732324	0.073436	1.606365	0.489523	0.950653	0.379457	0.699106	0.62985

Eml6	0.815721	0.741463	1.239214	0.985704	0.688378	0.515634	1.259297	0.678337	0.629837
Cd300ld2	0.247676	0.370524	0.660545	1.559826	1.140104	0.534433	1.109269	0.707434	0.629485
Zfp493	0.355589	0.52352	0.558848	0.353597	0.741825	0.360156	0.437523	0.306312	0.629401
Tspan6	0.817578	0.627232	1.320856	1.344574	1.257823	0.319876	0.552505	1.107743	0.629373
Gm35266	0.959207	1.05952	0.897427	1.405111	0.599203	0.190762	1.503073	0.40262	0.629134
Snord88c	3.299885	1.97466	1.320107	1.31256	0.628555	0	0.682119	1.256725	0.629017
Mir706	0	0	0	0	0	0	0	0	0.629017
Pln	0.047121	0.117489	0.235633	0.398286	0.134633	0.370398	0.194808	1.233759	0.628749
Rell2	0.345115	0.594521	0.5648	0.655166	0.119521	0.812386	0.713388	0.537682	0.627949
Ptchd1	1.166086	1.278392	0.845733	1.009078	0.864717	0.979579	0.588804	0.813603	0.627805
Slain1	0.677727	0.46349	0.271122	1.116799	1.180269	0.85953	0.560372	0.719007	0.627479
Plekhb1	0.814344	0.999602	0.501193	1.401547	0.894892	1.419267	0.938781	0.864798	0.626884
B4galnt2	0.826873	0.506048	0.469869	0.766179	0.429548	0.260682	1.165383	0.644125	0.626884
Gm41538	0.896089	0.357481	1.015686	0.415833	0.682741	0.607695	0.246974	0.341266	0.626305
Ms4a4d	1.386864	1.383172	1.861534	2.250103	0.729935	1.215027	0.377209	0.486474	0.626118
Olfr755-ps1	0.072945	0.363753	0.364766	0.290145	0.138944	0.067457	0.226176	0	0.625706
Ambp	1.13764	0.436389	0.481365	0	0.958459	0.283245	0.316564	0.374935	0.625541
Rpl32l	0.468227	0.373584	0.187313	0.558725	0.802681	0.866003	0.871085	1.694032	0.624767
Gm13039	0.327648	0.392131	0.589835	0.325813	0.374458	0.545398	0.135456	0.062391	0.624556
Gm32673	0.131059	0.235279	0.026215	0.15639	0.224675	0.07272	0.731464	0.324431	0.624556
Gm16279	0.982654	0.245009	0.982768	0.325717	0.233967	0.681546	0.423176	0.15593	0.624371
Rps12-ps10	0.65478	0	0.436571	0.542594	0.415737	1.110121	0.676748	0.623415	0.624064
Nrg2	0.429897	0.223697	0.747734	0.985084	0.907865	0.691401	0.695458	0.694038	0.623503
Tbx1	0.34221	0.341299	0.217795	1.175559	0.237031	0.834318	0.450153	0.592396	0.622663
Rps8-ps4	0.163293	0.244288	0.081656	0.243568	0.077759	0.679538	0.253158	0.777356	0.622532
Sectm1a	0.181407	0.434218	1.015999	0.61333	0.691081	0.671039	0.674976	0.725414	0.62243
Cltrn	0.054404	0	0.326464	0	0	0.050311	0	0	0.622226
Gm12227	0.63343	0.399675	0.607646	1.330465	0.744856	0.191274	1.242559	0.572318	0.622196
Cdkn2a	0	0.26019	0.326144	0.389135	0.31058	0.904719	0.337047	0.248388	0.621616
Gm15367	0.326106	0.813095	0.054357	1.459258	0.362343	0.201049	0.505571	0.41398	0.621616
Gm24524	0.652213	0.650476	0.326144	0	0.31058	0	0.337047	0	0.621616
Gm22192	0	0	1.304577	0	0	0	0	0	0.621616
Gm24920	0.652213	0.650476	0.652288	3.242796	1.24232	1.206291	0	1.24194	0.621616
Mir1948	1.956638	0	0	0	0	0	0	0.62097	0.621616
Pi15	0.551373	0.799862	0.501307	2.641735	1.002505	0.648954	0.466259	0.047724	0.621054
Gm39273	0	0.092769	0	0.554975	0.265765	0	0.480688	0	0.620573
Dtna	0.406736	1.663176	0.935601	1.456046	0.348633	1.918295	0.588534	0.774504	0.620248
Txn-ps1	0.520545	1.557478	0.650757	0.388222	0.867583	0.60173	0.538009	0.619513	0.620157
Dchs1	0.70267	0.664462	0.603846	1.24737	0.88733	1.034878	0.591755	0.644233	0.620099
Gm9575	0.75893	0.180217	0.361437	0.25156	0.275351	0.534732	0.709688	0.653759	0.619996
Zfp239	0.390147	0.704101	0.464515	0.849821	1.026246	0.309254	0.614456	0.459901	0.619742
Rps2-ps11	0.531922	0.471561	1.123076	1.116656	0.73175	1.202433	1.099535	1.125426	0.619628
Vpreb1	0.28874	0.071993	0.43316	0.143561	0.274993	0.60079	0.22382	0.893453	0.619188
Gimap8	0.719747	0.577766	0.702274	2.356623	1.036578	1.201326	0.671319	0.885836	0.619058
Efemp1	0.818586	1.351295	0.536378	1.515726	1.102211	0.887521	0.525134	0.295625	0.618768
Sorbs2	0.405545	0.525806	0.378553	1.277034	0.720976	1.212616	0.307378	1.390028	0.618433
Emid1	0.162218	0.202233	0.243356	0.806548	1.158711	0.900086	0.251491	1.312805	0.618433
Cuedc1	1.037958	1.423392	1.279061	1.916838	1.076802	1.422666	0.976997	0.882355	0.618291
Ankrd2	1.447078	1.293926	0.29943	1.488592	1.710846	1.707375	0.722027	0.855162	0.618259
B230206L02Rik	0	0	0.432316	0	0.514606	0.099936	0	0.411559	0.617981
Gm33272	0.324073	1.422124	1.404479	0.665999	0.390948	0.37961	5.024182	0.802228	0.61774
Nxpe3	0.698942	0.969113	0.954764	0.559412	1.071558	0.788244	0.792869	0.649231	0.617411
D630023F18Rik	1.471286	1.819538	1.883465	1.287479	0.560495	0.653088	1.520648	3.361941	0.616997
Gm9432	0.323443	0.064516	0	0.192979	0.123217	0.059822	0.267435	0.492718	0.616539

Adamts9	0.839449	0.507819	0.337195	1.108435	0.471826	0.744481	0.320021	0.884403	0.616447
Sema3g	0.782089	0.465004	0.78218	3.679157	1.389437	2.781735	0.170992	0.758945	0.616389
Kctd15	0.646578	1.026994	1.125655	1.095407	0.501758	0.974412	0.222757	0.661206	0.616246
Gm40595	0.574488	0.644578	0.502735	0.357044	0.615528	0.46486	0.519541	0.205113	0.61598
Gm12428	1.167117	0.582005	0.675778	0.427582	0.555775	0.852089	0.761857	0.731059	0.614729
Mir194-2	0.644629	0	0	0	0	0	1.332512	0	0.614388
Rps8-ps2	0.805786	0.642912	0.725292	1.041654	1.07439	0.894199	0.499692	0.997343	0.614388
Ppp1r2-ps2	0.608816	0.750065	0.608887	0.783466	0.477507	0.463659	0.370142	0.511458	0.614388
Gfi1b	1.833993	1.187317	1.737669	0.447931	2.42083	1.993566	1.52972	1.102826	0.613319
Dkk2	0.508743	0.910315	0.628521	0.342222	0.498772	0.650355	0.603137	0.427388	0.613228
Rps2-ps7	0.409353	0.408263	0.4094	0.465211	0	0.108159	0.181323	0.055678	0.613092
Nox4	0.392066	0.53179	0.596009	0.311895	0.328591	0.290056	0.194505	0.612187	0.612824
LOC118568401	0.24383	0.574791	0.19952	0.176338	0.295554	0.245985	0.504021	0.400986	0.612669
Gm32997	0.401725	0.400656	0.482126	0.95874	0	0.965907	0	0.229489	0.612608
Gm9497	0.275355	0.183081	0.275387	0	0	0.254639	0.094864	0.087388	0.612354
Gm39084	0.642388	0.961016	0.51397	0.383274	0.122361	0.415842	0.066394	0.428131	0.612253
Rpl7a-ps3	0.834138	0.511949	0.385031	1.14849	0.672204	0.712047	1.127391	1.038544	0.611544
Gm4995	0	0.127987	0.128344	0.12761	0	0	0.132634	0.244363	0.611544
D9Wsu149	0.4988	0.426405	0.712654	1.700592	0.203594	0.461275	0.736478	0.067844	0.61123
Lrrc73	0.56079	0.559297	0.640977	0.876305	0.762986	0.88903	0.124201	0.572064	0.610837
Ap1m2	0.576146	0.255383	0.224083	0.031829	0.182905	0.207201	0.099246	0.274274	0.610132
Chic1	0.128032	0.063846	0.640237	1.655099	0.548715	0.414401	0.066164	0.06095	0.610132
Catsperg1	1.555792	1.430966	0.968161	1.650216	1.53111	0.799304	1.715188	1.234389	0.609599
Gm11465	0.959136	1.46676	1.406896	0.826595	1.705145	0.354792	3.96526	0.974071	0.609428
Naaladl1	0.185549	0.185056	0.41238	0.307517	0.530146	0.972345	0.809715	0.392581	0.609133
3110070M22Rik	0.786356	0.490164	0.393223	0.488719	0.982953	0.499948	0.101592	0	0.608942
Gm11224	0.45628	1.183171	0.4107	0.453724	0.521468	0.295368	0.565906	0.434424	0.608826
Gm41442	0.651743	0.444393	0.572004	0.535668	0.342026	0.455109	0.625494	0.588864	0.608492
Cldn19	0.12759	0.445378	0.255211	1.078444	1.336674	0.117992	0.131871	0.85035	0.608025
Upb1	0.492054	0.308987	0.729053	0.743007	1.128175	0.758393	0.922946	0.763455	0.607925
Rmi2	0.563425	0.680225	0.696949	0.737196	0.917868	0.671865	0.505706	0.536436	0.607651
Unc5a	0.637386	0.736821	0.767849	1.022747	0.607039	0.669812	0.958211	0.84132	0.607485
1700022N22Rik	1.274438	1.429926	1.274586	0.396031	0.682741	0.14732	1.646494	1.516738	0.607326
Tgfbr3	1.310851	1.098546	0.773856	1.719906	1.109724	0.799738	0.809134	1.161388	0.607326
Gm13215	0	0.529602	1.168371	0.211217	0.404587	0.29464	0.219533	0	0.607326
Mir3098	0	0	0	0	0.606881	1.178561	0	2.42678	0.607326
n-TUtca2	0	0	0	0	0	0	0	0	0.607326
Fbxo17	0.268141	0.935993	0.536343	0.999895	0.606514	0.7749	0.623557	0.351032	0.606959
Gm41688	1.472511	1.428899	1.07466	1.662136	1.288695	0.441643	1.768711	1.098845	0.60689
Gm8172	1.193928	1.270133	0.796045	1.424688	1.137084	1.177715	1.275117	1.212519	0.60689
D830014E11Rik	0.293841	0.195373	0.097958	0.146098	0.513061	0.498181	1.316032	0.18651	0.60679
Pdzrn3	0.558883	0.882542	1.242106	0.957129	0.413991	0.732186	0.401134	0.827728	0.606646
Satb2	0.603994	0.462547	0.485409	0.975993	0.63687	0.488736	0.624258	0.431296	0.606499
Hyal3	0.30294	0.543841	0.545356	0.361492	0.519331	0.728389	0.720139	0.288429	0.606331
Gm34589	0.438592	0.809236	0.701829	0.06542	0.480367	0.365037	0.385311	0.208791	0.606125
Myo6	0.908076	0.633961	0.658432	1.151312	0.778358	0.62982	0.375417	0.497132	0.605834
Ppp1r3f	0.699133	0.618036	0.572084	0.790019	0.620448	0.602454	0.673321	0.635387	0.60576
Gm36358	0.181467	0	0	0.090225	0	0.083907	0.093777	0.431935	0.605338
Gm46911	0.26817	0.656483	0.658312	0.363638	0.162529	0.40581	0.730713	0.348169	0.604122
Kcnab3	0.415185	0.457666	0.721194	1.151659	0.312171	0.66686	0.474284	0.915423	0.603975
Krt18	0	0.355439	0.277223	0	0.075427	0.952109	0.040927	0.150807	0.603856
Gm525	0	0	0	0	0.402275	0.097652	0	0	0.603856
Tigit	0	0.057415	0	0.858684	0.109654	0.266186	0.594995	0.109621	0.603542
Gm36117	0.509314	0.277068	0.55568	0.491114	0.499764	0.228363	0.71782	0.617167	0.603099

Gm37201	0	0.045025	0	0.718274	0.085991	0	0.04666	0.042982	0.602381
Gm5605	0.03511	0.350161	0.105341	0.244391	0.735636	0.162341	0.326588	0.334278	0.602326
Gm41523	0.660319	0.544028	1.033662	0.256938	0.574195	0.265497	0.385745	0.410014	0.60198
Fgfbp3	0.789266	0.850138	0.789358	0.313938	0.841891	1.109431	1.533603	1.142217	0.601793
Tmsb15b1	0	0	0	0	0.24054	0.116782	0	0	0.601793
Pdcd1	0.544916	0.057207	0.45893	0.513345	0.327772	0.318266	0.14821	0.40959	0.601357
Prr18	0.375042	0.25503	0.170494	0.508557	0.34095	0.61483	0.669534	0.633	0.601164
Mir3109	3.779868	2.513203	1.890154	7.51739	2.399937	2.330336	1.302227	1.799402	0.600425
6330407A03Rik	0.419531	0.268981	0.86913	1.132349	0.913273	0.332545	0.712354	0.684746	0.599776
Arhgap27os3	0.811982	0.384665	0.629359	0.585389	0.869988	0.675806	0.650398	0.309235	0.599765
Gm40409	0.566337	0.564829	0.251734	1.188902	0.659233	0.640115	0.975562	0.119824	0.599743
AW046200	0.148032	0.664371	0.592198	0.404808	0.211476	0.205343	0.458996	0.281882	0.599623
Cldn23	1.227964	0.627282	0.868661	0.208478	0.142622	0.249274	0.154776	0.484766	0.599452
Gm32558	0.49378	0.313387	0	0.044638	0.342015	0.290584	0.510347	0.128217	0.598966
LOC118568513	1.15205	1.218619	1.187099	0.659586	0.631721	1.033096	1.010292	1.130103	0.598913
Gm26512	0.94229	1.253042	0.628266	0.624675	0.448713	0.290467	1.623173	0.598102	0.598724
LOC115490109	0.0523	0.260804	0.313837	0.312043	0.09962	0	0.270274	0.298769	0.598159
Gm46546	0	0	0.836898	0.416057	0.39848	0.773847	0.216219	0	0.598159
Gm35768	0.046469	0.32442	0.278849	0.046209	0.199157	0.107434	0.3362	0.110609	0.597909
Gm9054	0.705718	0.391022	1.176333	0.857713	0.672118	1.232738	0.243132	1.194511	0.597877
Gm10602	0.537364	0.714578	0.80614	0.178118	0.085297	0.745406	0.647958	0.085271	0.597515
Podnl1	0.164896	0.723611	1.18739	0.360739	0.502545	0.243985	0.136343	0.784986	0.59721
Gm15549	0.313209	0	0.078311	0	0	0.362058	0.404647	0.149103	0.597033
Prkag3	0.17618	0.117141	0.137045	0.603444	0.838961	1.086173	0.263021	0.61505	0.597033
Agbl3	0.459201	1.124129	0.417504	1.037792	0.318064	0.463259	0.474607	0.278221	0.596808
LOC108167751	1.351421	1.357733	0.934179	0.909076	0.842279	0.698391	1.37622	0.946092	0.596658
Gm33742	0.686165	0.5837	0.565142	0.662253	0.653496	0.597217	0.552747	0.701332	0.596272
Reck	0.425196	0.573734	0.76294	0.820757	0.702711	0.531987	0.297283	0.381015	0.595955
Gm35235	0.416828	0.207859	0.416876	0.207246	0.396982	0.770938	1.292436	0.992152	0.595911
Gm46901	0.625241	0.415718	0.949551	1.358615	0.396982	0.364054	0.335076	0.352765	0.595911
Hoxb6	0.351616	0.545501	0.039073	1.010089	0.22325	0.686455	0.242275	0.632348	0.595771
Gm32597	0.867304	0.345998	0.520443	0.758952	0.429526	0.449151	0.752978	0.495455	0.595165
Gm12924	0.71303	0.62224	0.623974	0	1.697704	1.153928	0.184238	0.848593	0.594633
Pcolce2	0.237677	1.24448	0.475408	1.624873	0.509311	1.126454	0.276357	0.113146	0.594633
Mir324	9.343494	9.93986	0.622972	8.052335	2.372971	2.88019	5.79418	2.372246	0.593679
Plcd3	0.759159	0.71831	0.895522	0.812976	0.48201	0.810053	0.643798	0.685727	0.593679
Slc35g1	0.958307	1.322129	0.846603	1.079998	0.425918	1.019144	0.990458	0.745128	0.593679
Aldh1a1	0.649982	1.053409	1.056344	1.238821	0.748002	0.626128	0.447859	0.850914	0.593679
Gm5511	0	0	0.069219	0.20647	0.197748	0.44803	0.357665	0.065896	0.593679
Mcmdc2	0.882441	0.15531	0.622972	0.464558	0.370777	0.240016	0.321899	0.27182	0.593679
Cav3	0.574984	0.238939	0.718814	1.048233	0.684511	0.620349	0.247615	0.593061	0.593679
Gm38424	0.398835	0.365951	0.454725	0.460057	0.524188	0.494232	0.453437	0.57719	0.592995
Gm39988	1.173658	0.963968	0.82856	0.137304	1.18353	0.957672	0.927614	0.854511	0.5922
Gm37108	0.698799	0.464626	0.155307	0.617675	0.29579	1.148849	1.12349	0.665325	0.592016
6530409C15Rik	0.451634	0.394128	0.846912	1.571864	1.021562	0.678692	1.050269	0.859999	0.591865
Gm10451	0.434565	0.309577	0.31044	0.432131	0.2365	0.459282	1.090779	0.531962	0.591684
Adam11	0.973452	0.242715	1.022243	0.544499	0.625796	0.528878	0.72943	0.706701	0.591463
Rasgef1a	0.551451	1.117153	0.310227	0.702589	0.377485	0.302792	0.623385	0.738331	0.59128
Gm40943	0	0.494769	0	0.493311	0	0.114692	0	0.472326	0.591022
Gm10171	0.413331	1.030577	0.878431	0.719278	0.344446	0.860031	0.533998	0.688681	0.590912
Myh3	0.259056	0.627462	0.656969	0.276006	0.308403	0.641698	0.219936	0.466868	0.590805
Slc22a23	0.826406	0.480787	0.533783	0.582092	0.639486	0.398039	0.854132	0.47537	0.590729
Gm8960	0.688672	0.480787	0.413251	0.821777	0.459118	0.955293	0.782954	0.917956	0.590729
LOC118568593	0.525835	0.412056	0.431986	0.410842	0.071543	0.121569	0.368788	0.250323	0.590662

Trim55	0.383552	0.500233	0.560642	0.381404	0.309092	0.982238	0.426914	0.252817	0.590519
Tmem262	0.30971	1.75035	0.825989	1.2319	1.278178	0.47735	1.067002	1.179497	0.590362
Otof	0.500301	0.784093	0.810105	0.781783	0.555894	0.528757	0.603266	0.95267	0.590362
P3h2	0.652016	1.117148	0.919616	1.147105	0.93942	1.205928	0.93308	1.098308	0.589561
Gm14154	0.618477	1.570115	1.124635	0.447282	1.23161	1.351878	0.75545	0.695915	0.589464
Gm11960	0.337351	0.280378	0.393622	0.223641	0.428386	0.415963	0.348669	0.160596	0.589464
Gm7107	1.120351	1.502668	0.656834	1.728738	1.250977	1.321877	1.317654	0.809209	0.589128
Gm5530	0.579492	0.19265	0.42501	0.653079	1.287771	0.357264	0.718721	0.809209	0.589128
4930445K14Rik	1.080965	1.2321	0.803096	0.644946	0.911842	0.742592	0.414972	0.352863	0.588718
Cdh11	0.260069	0.216147	0.834485	0.247837	0.175445	0.270567	0.279994	0.50554	0.588689
LOC118568792	0.508606	1.014504	0.326999	0.216753	0.311394	0.470343	1.351722	0.242121	0.588621
Fbxo44	1.472648	2.321536	2.660576	2.031263	1.990693	0.834687	2.651322	2.306689	0.588591
Sat2	0.823338	0.410573	0.617575	1.296316	0.914827	0.888296	0.070913	0.457274	0.588535
Tmem116	0.411414	0.871928	0.822924	1.380745	0.391826	0.332905	1.116195	0.391707	0.588171
Gm11415	0.339354	0.553828	0.617079	0.12271	0.205671	0.570589	0.286968	0.264353	0.588062
Gm9105	0.580503	0.868437	0.544285	0.61333	0.621973	0.33552	0.524982	0.794501	0.587851
Evc	0.486868	0.582686	0.600541	0.887592	0.293669	0.630337	0.721257	0.278128	0.58777
Cldn12	0.662729	1.014504	1.587652	0.842929	0.469712	0.541607	1.162845	0.630983	0.587572
Gm2623	0.461984	0.307169	0	0	0	0.142409	0.636644	0.293236	0.587082
Gm40080	0.403418	0.42352	0.360994	1.54129	0.424654	0.805041	1.272801	0.606463	0.586857
AW822252	0.205136	0.358033	0.5129	0.560965	0.781478	1.04337	1.696148	0.781239	0.586539
Peg10	0.257774	0.381485	0.390864	0.983977	0.530599	0.638247	0.996935	0.704611	0.586466
Map3k7cl	1.352148	0.735572	0.430279	2.566914	2.692612	2.500848	0.889326	1.63848	0.58578
Gm46752	0.122923	0.980762	0.122937	0.244468	0.11707	0.22735	0.254093	0.234069	0.58578
Gm9083	0.737535	0.674274	0.245874	0.305585	0.292675	0.682049	0.127047	0.351103	0.58578
Gm46239	0	0.204024	0	0	0	0.189179	0	0	0.584916
Gm14537	0.08758	0.78612	0	0.174179	0.417051	0.080991	0.633627	0.333539	0.5843
Gm31657	0.437899	0.611427	0.642327	0.406417	0.611674	0.755917	0.603454	0.500308	0.5843
Hoxc9	0.290252	0.611122	0.838602	0.737599	0.52215	0.954367	0.533315	0.706223	0.584008
Car7	0.880577	0.610944	0.995551	1.180216	1.057431	1.416226	1.068402	0.36452	0.583839
Gm41309	0.459431	0	0.153162	0.304572	0.58341	0	0.158282	0.291616	0.583839
Gm12992	0.290083	0.385747	0.87035	0.673069	0.58324	0.357679	0.966071	0.429625	0.583669
Gm46328	0.101908	0.101637	0	1.114711	1.067619	0.282725	0	0.776213	0.582765
Gm5711	1.069054	1.218523	1.756506	0.379666	1.890859	0.282465	1.10492	0.508922	0.58223
Gm5837	0.746412	0.473725	0.135727	0.641018	0.549312	0.81576	0.701322	0.355329	0.582052
St8sia2	0.269016	0.154789	0.569139	0.390975	0.108396	0.191368	0.224572	0.561516	0.581823
Gm39654	0.305164	0	0.203466	0	0	0.094069	0.420536	0	0.581696
Gm15545	0.457745	0.456527	0.966464	0.404606	0.581269	0.752549	0.262835	0.774789	0.581696
Gm33457	0.152554	0.060859	0.122057	0	0.232465	0.028215	0.157672	0.087148	0.581589
Tnfrsf9	0.332795	0.719136	0.637931	0.717016	1.082913	0.435991	0.68792	0.580898	0.581502
Gm46290	0.495664	0.437304	0.55292	0.568716	0.254189	0.493634	0.82755	0.254111	0.581429
Gm5551	0.457536	0.076053	0	0.303315	0.072625	0.493634	0.078814	0.145206	0.581429
Gm6104	0.394449	0.286109	0.681401	0.39224	0.341517	0.198968	0.481807	0.102424	0.581006
4930512H18Rik	0.064164	0.319968	0.336902	0.63805	0.534708	0.430195	0.298427	0.091636	0.580967
Gm3230	0.3445	0.211436	0.371044	0.184461	0.252383	0.122532	0.273891	0.580304	0.580908
Mir3057	0	1.215175	1.218561	5.452173	1.740613	2.816889	0.629648	0	0.580631
Gm30205	0	0	0.284331	0.040386	0.309442	0.037559	0.419766	0.154674	0.580631
Klrg1	0.081228	0.121518	0.324949	0.807729	0.657565	0.638495	0.293836	0.386685	0.580631
Selenok-ps5	0.609209	0.531639	0.30464	0.302898	0.290102	0.140844	0.236118	0.145007	0.580631
Zfp791	0.223213	1.275	1.197374	1.452848	1.082255	1.050869	1.740752	1.062605	0.580206
Ccdc62	1.779262	0.513678	0.796078	1.815856	0.802681	1.342305	1.403414	2.09525	0.58014
Chil5	0.050721	0	0	0.201748	0.338143	1.031914	0.052423	0.096583	0.5801
Gm14486	0.304326	0.151758	0.152181	0.050437	0.434755	0.046905	0.052423	1.448741	0.5801
Panct2	0.028306	0.112924	0.084929	0.014074	0.336984	0.615156	0.029256	0.202128	0.580038

Gm12185	0.975173	0.700674	0.639704	0.928003	0.878812	0.494541	0.953702	1.178046	0.579642
Gfod2	0.715765	0.659984	0.810395	0.940055	1.08041	0.512049	0.544366	0.385743	0.579216
Gm19240	0.758906	1.589459	1.024642	1.962102	0.795051	0.982537	2.000136	1.300595	0.578644
Timp4	1.93521	1.286705	0.815918	3.697808	1.282923	1.596623	0.647103	0.650297	0.578644
Pcbd1	0.269772	0.672633	0	0.201195	0.578087	0.810798	0.139411	0.128424	0.578512
Tmem182	0.303493	1.362083	0.758821	0.85508	0.963478	1.590412	0.836467	0.866865	0.578512
Fmo2	2.022303	1.102582	0.782048	2.399763	1.117091	1.570936	0.209015	1.437655	0.57823
Gm3534	0.746013	1.023036	0.652837	0.880928	0.799306	1.250424	0.433711	0.665885	0.577701
Gm13840	0.293391	0.483442	0.503925	0.780106	0.449505	0.418774	0.725124	0.461513	0.577491
Erp27	0.484704	0.18128	0.969521	0.301243	0.230814	0.448239	0.375725	0.807601	0.577458
Gm32311	0.106886	0.035534	0.071265	0.442863	0.576849	0.395378	0.202532	0.271375	0.577272
Gpm6b	0.567625	0.717078	0.529845	0.940743	0.66674	0.367445	0.801781	0.594478	0.577064
Itga10	0.758823	0.83358	0.604929	1.04984	0.76459	0.345783	0.988876	0.544473	0.576484
Gm8557	0.302389	0.301584	1.008082	0.501159	0.575985	1.118561	0.208356	0.767745	0.576408
Pth1r	0.719601	0.430611	0.518173	0.686947	0.38379	1.091362	0.892492	0.822157	0.576109
Gm14398	0.201227	0.200691	0.402501	0.250125	0.239558	0.139566	0.259973	0.47897	0.575362
Klra9	0.092861	0.370455	0.185744	0.738727	0.574859	1.459875	0.335918	0.61889	0.575282
Gm30708	0	0.150246	0	0	0.143474	0	0	0	0.57432
AA388235	0.444422	0.672007	0.573514	0.441933	1.133252	0.437503	0.459332	0.723421	0.573874
Bmp4	0.11466	0.800483	0.487361	0.883637	0.928207	1.03383	0.296267	0.382086	0.573726
Ankrd29	0.534987	0.266782	1.003218	0.930984	0.891653	0.742109	0.552936	0.700371	0.573627
Gm41049	0.859505	0.428608	1.89113	0.683752	0.736725	0.158969	0.799507	0.818333	0.573429
Efnb2	0.920036	0.799947	0.908346	1.759389	0.696492	1.38531	0.438878	0.797352	0.573342
Slc7a4	0.981485	1.326213	1.361573	1.038954	0.783988	0.936925	1.07986	1.356488	0.573336
Pcp2	0.120256	0.359808	0.48108	0.47833	1.145306	0.111209	0.248581	0	0.573074
Gm18583	0.150239	0.524435	0.525896	0.597588	0.071543	0.486276	0.621117	0.214563	0.572763
Slc13a2	0.085817	0.085589	0.257482	0.298679	0.694718	0.079361	0.133045	0.49024	0.572541
Garnl3	0.405605	0.825231	0.632817	0.580799	0.494456	0.450109	0.519824	0.386176	0.572135
Clmn	0.523603	0.789682	0.996239	0.736558	0.547325	0.602312	0.607166	1.179429	0.57207
LOC118567841	0.119996	0.119676	0.24002	0.357971	0	0.443873	0	0.114248	0.571833
Gm38483	0.234101	0.116739	0.117064	0.421931	0.320498	0.148836	0.120978	0.139304	0.571743
Gm13433	0.299665	0.149434	0.14985	0	0.142699	0.415681	0.154859	0.570621	0.571215
Rpsa-ps5	0.108916	0.162939	0.381249	0.216611	0.51865	0.100722	0.056285	0.207397	0.570935
Gm2401	0.374076	1.193856	0.897887	1.785507	0.783785	0.691868	1.082553	0.356157	0.570444
Gm12058	0.199417	0.397773	0.498602	0.396601	0.189923	0.184415	0.103054	1.13919	0.570188
Gm6665	0.747144	1.415794	0.149446	0.520071	0.640414	0.345468	1.776084	0.497948	0.569675
Mzf1	0.423381	0.397416	0.597784	0.643897	0.592976	0.575779	0.360365	0.142271	0.569675
Gm14037	0	0	0	0.592769	0	0	0.205369	0	0.568144
Pnpla1	0	0	0.137579	0.091195	0.131014	0.254429	0.023696	0	0.568144
Gm41062	0	0.891782	0.894266	0.148192	1.135454	1.102524	0	1.276995	0.568144
Fzd2	0.412577	0.441958	0.412624	0.866116	0.494805	0.833731	0.031587	0.407362	0.567987
Wdr89	0.855468	0.797548	0.743972	0.443831	0.407369	0.257971	0.326758	0.159357	0.567191
Gm31763	0.649031	0.431535	0.649106	0.968093	0.875684	0.150051	0.838507	0.463456	0.567035
Rpl7a-ps7	0.462534	0.593104	0.33042	0.657062	0.629304	1.038791	1.092691	0.754934	0.56679
Phkg1	0.594191	1.274111	0.623974	0.709036	1.216688	0.961607	0.184238	0.509156	0.566317
Gm7908	0.148429	0.592134	0.593783	0.295194	0.494766	0.137262	1.303971	0.565274	0.565862
Gm51503	0.074214	0.4441	0.074223	0.073799	0.706809	0.068631	0.536929	0	0.565862
Nlrp10	0.717245	0.505668	0.754431	0.651742	0.541766	0.59467	0.383435	0.518052	0.565736
Gm8444	0.118711	0.47358	0	0.708277	0.113059	0	0.858857	0.113025	0.565711
Sec31b	0.45221	0.408724	0.70666	0.674515	0.551808	0.457394	0.715677	0.538185	0.565682
Rbm3os	1.120627	1.183387	1.318538	1.179899	1.255615	1.158241	1.226354	1.317993	0.565442
Slc32a1	0.019764	0.078846	0.039533	0.491333	0.282345	0.182771	0.183844	0.376346	0.565106
Slc25a29	0.620997	0.42228	0.084691	0.224552	0.430131	0.313243	0.175045	0.3225	0.564962
Stk35os1	0.502189	0.983816	1.022432	0.713394	0.888233	0.829301	1.075149	0.58059	0.5641

Samd3	0.088748	0.118016	0.059172	0.470673	0.59166	0.601858	0.122301	0.253491	0.5639
Gm10259	0.478886	0.730464	0.535287	0.924394	0.241457	0.729414	0.84433	0.482766	0.563814
Gm41406	0.147835	0.147441	0	0.514524	0.211194	0.068357	0.840371	0.281507	0.563599
1700008O03Rik	0.073917	0.073721	0.295704	1.029047	0.070398	0	0.076397	0	0.563599
Tnfsf18	0.161157	0.160728	0.483528	0.614309	0.613937	0.173872	0.083282	0.076719	0.563189
Gm40346	0.63817	0.553965	0.661883	0.716858	0.326404	0.852453	0.671795	0.427571	0.563178
1700097N02Rik	0.787286	0.065433	0.721763	0.848116	0.249934	0.060671	0	0.187393	0.562765
Sele	0.932852	1.082265	0.72352	1.741666	1.559299	1.126756	0.550942	1.740081	0.562486
Gm23690	0.589767	0.588197	0	1.172926	1.123375	0	0.609553	1.123031	0.5621
Gm20939	0.401041	0.399974	0.754989	1.10255	0.62909	0.53449	0.536407	0.595207	0.5621
Eif1-ps2	0.1361	0.135738	0.090744	0.090225	0.302447	0.209768	0.140666	0.129581	0.5621
Gm51426	0	0	0	0	0	0	0	0	0.5621
Lin28a	0.175235	0.301873	0.047797	0.031682	0.30344	0.898652	0.246974	0.257845	0.561777
Prox2	0.884023	1.136374	1.060951	1.543258	0.486451	0.79935	0.812162	0.635933	0.561702
Unc5b	0.446545	0.360075	0.266058	1.180967	0.570062	0.817115	0.530264	0.379925	0.561426
Stk32c	0.883241	0.322993	0.353337	0.849017	1.233743	0.871246	0.608582	0.448497	0.561204
Gm11613	0.668312	0.603053	0.700218	1.091791	0.666802	0.75047	0.641396	0.696898	0.561132
Casc1	0.555688	0.391206	0.686518	1.105151	0.311313	0.544111	0.979742	0.311217	0.560774
Stox2	0.576153	0.641154	0.746053	0.862404	0.612258	0.650587	0.526532	0.334907	0.560686
Peg12	0.020999	0.041887	0.462038	0.271461	0.259993	0.213614	0.151927	0.159947	0.560397
Rgs20	0	0.130248	0.326528	0.064932	0.186568	0.120771	0.067489	0.310851	0.560114
Gm41410	1.09031	0.75282	0.838797	0.333601	0.878645	1.085845	0.693471	1.197787	0.559549
Gm18304	1.00644	0.083647	0.251639	0.667202	0	0	0.606787	0	0.559549
Gm10033	0.489131	0.800039	0.851186	0.690675	0.903735	0.759919	0.839198	0.633352	0.559422
Ptpn3	0.768421	1.379475	0.628781	0.236181	0.23951	0.439287	0.375441	0.332551	0.559267
Gm671	0.146662	0.65822	0.366696	0.3646	1.117431	0.203442	0.75791	1.256725	0.559126
Ccl19	0	0.097514	0	0	0	0	0	0	0.559126
Stard13	0.792266	1.108272	1.131941	1.002691	0.529162	0.846844	0.882653	1.24413	0.55897
Gnao1	0.790152	0.762627	0.917702	1.368683	0.801082	0.683565	0.079032	0.485356	0.55874
Gria3	1.330133	0.947566	0.934369	0.992012	0.889779	1.039698	0.883774	1.115653	0.558407
Gm16553	0.585613	0.584054	0.520606	1.811703	2.230927	1.504325	0.807014	0.681464	0.558142
Cage1	0.319374	0.026544	0.532352	0.264654	0.354863	0.295347	0.302582	0.152038	0.558052
Lyzl4	0.365686	1.385909	1.243478	0.29091	1.532413	1.285066	0.982683	0.90524	0.557651
Ndufs6b	0.467832	1.166466	0.116972	0.930422	0.556947	0.540795	0.483527	0.222711	0.557357
Gm8508	0.357246	0.453466	1.039381	0.678195	1.237226	0.780874	0.57063	1.020399	0.557161
Clec12b	0.22481	0.941687	0.224836	0.22355	0.385391	0.66527	0.232352	0.428082	0.557085
Gm9030	0.720734	0.466258	0.681854	0.348663	0.408141	0.414318	0.483188	0.426562	0.556965
Gm17819	0.219123	0.218539	0.511346	0	0.556507	0.810552	0.830406	0.069542	0.556916
Sfrp5	1.721498	3.433829	2.042692	0.696348	1.139338	1.160262	0.904705	0.833407	0.556183
Gm9449	0.437669	0.145501	0.486355	0.19343	0.416831	0.539657	0.552875	0.324103	0.556183
Limch1	0.17398	0.326619	0.409411	1.424747	0.643291	0.709812	0.222126	0.545656	0.555978
Vash2	0.640162	1.007345	1.351611	0.877061	0.718072	0.552535	0.705749	0.595953	0.555898
Abca8a	1.648532	1.77213	1.155094	1.894518	1.118774	0.985911	0.7754	1.06204	0.555094
Gm46649	0.232933	0.116156	0	1.158141	0.110921	0	0.120374	0.110888	0.555015
Gm6316	0.492584	0.40195	0.761354	0.445295	0.597076	0.703995	0.555393	0.511623	0.554835
LOC118567458	0.28685	0.597416	0.286884	0.268465	0.458	0.405708	0.52319	0.642611	0.554829
Gm9349	0.727534	0.725597	0.291047	0.434075	0.138579	1.749281	0.451165	1.24683	0.554723
Gm51579	0.430145	0.227117	0.430195	0.075483	0.481959	0.163794	0.33997	0.192725	0.55466
Gm9013	1.33796	1.682501	1.803546	1.330465	1.274258	1.882853	0.901857	2.215424	0.554432
Celf4	0.581688	0.338414	0.630235	0.433822	1.200318	0.896544	0.551103	0.523056	0.5544
9430069I07Rik	0.387679	0.096662	0.193862	0.096377	0.646137	0.717026	0	0.461385	0.554238
Echdc3	0.887254	0.671297	0.550773	0.821437	0.466213	0.16976	0.189728	0.407812	0.554035
Gm18017	0	0	0	0	0	0	0	0.069178	0.553996
Dipk1b	0.683577	0.306791	0.61529	0.577785	0.325515	0.347683	0.070651	0.260333	0.553783

Gm46732	0.532593	0.386309	0.532655	0.288878	0.138337	0.98505	0.400335	0.414884	0.553754
Gm2065	0.087121	0.086889	0.319481	0.115511	0.082973	0.134278	0.180088	0.110597	0.553561
Gm23444	0.580503	0.289479	0	0.288626	0	0	0.29999	0.276348	0.553271
Cabp4	0.309494	0.424421	0.386912	0.92328	1.363258	0.894407	0.439831	0.221001	0.553078
Cc2d2a	0.524474	0.734535	0.468734	0.754564	0.531387	0.464378	0.622804	0.5206	0.553048
Cntnap1	0.421903	0.894157	1.012685	0.744683	1.044721	0.487703	1.068342	0.743132	0.552903
Gm13690	0	0.231341	0.231985	0.634313	0.386601	0.214508	0.059935	0.828177	0.552692
D130037M23Rik	0.396737	0.520811	0.342522	0.630549	0.54578	0.470372	0.732478	0.493958	0.552645
Gm29797	0	0.052557	0	0	0	0.194933	0.054466	0	0.552482
Hsd17b1	0.703844	1.486525	0.745333	1.317461	0.591471	1.263498	0.855833	1.14316	0.552445
Galnt16	0.918785	0.888144	0.805797	1.714829	1.373141	0.862735	0.672032	0.538322	0.552354
Gm6570	0.578988	0.144362	0.434291	0.287872	0.275711	0	0.149603	0.137813	0.551827
Smt3h2-ps	0.530739	0.625567	1.351128	0.38383	0.689277	0.356953	0.099735	0.551253	0.551827
Brd8dc	0.05033	0.22588	0.176174	0.400381	0.2157	0.279259	0.312109	0.023959	0.551639
LOC118567335	0.518372	0.76164	0.61101	1.122985	0.678827	0.500775	1.100229	0.907763	0.5514
A830035O19Rik	0.24786	0.3296	0.192803	0.356015	0.131144	0.127341	0.56928	0.131104	0.55121
Kctd14	1.47204	0.707844	0.736105	1.411516	1.026431	1.969018	0.461862	0.675736	0.551172
Gm6192	1.088355	0.576649	0.544241	0.777873	0.647836	0.6605	0.843652	1.003838	0.551065
Gm5866	0.157644	0.104816	0.052554	0.313521	0.200184	0.145784	0.380176	0.250154	0.550911
Gm32620	0.288991	0.672517	0.481707	0.622639	0.688079	1.068998	1.144964	0.550295	0.550868
Bves	0.210142	0.366769	0.394062	0.274266	0.137594	0.352228	0.230767	0.287609	0.550781
Gm5814	0.469508	0.396218	0.288962	0.897842	0.447154	0.133596	0.223966	0.653333	0.550748
Gm6296	0.536231	0.370249	0.577547	0.205088	0.707124	0.305163	0.511589	0.392727	0.55039
Reep2	0.28874	0.518348	0.519792	1.263339	0.302492	0.293719	0.238742	0.164945	0.55039
LOC118567389	0.14437	0	0.144387	0	0	0.534035	0	0	0.55039
LOC118568354	0.672559	0.766592	0.672637	1.050958	0.274516	0.444258	0.59582	0.548864	0.549436
Gm34466	0.19216	0.095824	0.096091	0	0.183011	0.177703	0.099303	0	0.549436
Ifnb1	0	0	0	0.286377	0.137139	0.066581	1.116195	1.850814	0.54896
Zfp708	0.598832	1.263388	0.714075	1.236762	0.943224	0.745475	0.333266	0.986793	0.548789
Gm35578	0.095914	0.382633	0.19185	0.476882	0.913471	0.532187	0.495657	0.182638	0.548485
Star	0.712501	0.860924	0.863323	0.885637	0.756876	0.456161	0.962992	0.456596	0.548485
Rpl15-ps2	0.993701	1.616985	1.35996	1.456199	0.94639	1.934618	1.94597	1.294664	0.548313
Gm7153	0.395473	0.286851	0.86295	0.715013	0.479365	0.731441	0.780323	0.753057	0.548248
Gm32655	0.115017	0.344132	0.11503	0.91498	0.109541	0.638183	0.475502	1.314086	0.548106
Gm52397	0.814561	0.764605	0.239605	0.809998	0.821413	0.576038	0.792367	0.775542	0.548011
Lhx2	0.665015	0.572802	0.393009	0.751466	0.374254	0.587031	0.5936	1.179979	0.547389
Zcchc3	0.753771	0.948655	0.700012	1.14217	1.17938	0.66387	0.537922	0.324657	0.547361
Gm39265	0.382331	0.762627	0.95594	1.140569	0.364128	2.121409	3.754007	2.184102	0.546594
C430049B03Rik	0.83966	1.000825	0.389156	0.529485	0.760674	0.47347	0.317499	0.760442	0.546526
Gm52396	0.73699	0.326679	0.245692	1.221437	0.467934	0.075727	0.253905	0.077965	0.546325
LOC118568525	0	0.142869	0	0	0.409292	0	0	0.136389	0.546123
Gm42060	0.687249	0.856774	0.22911	0	0.436352	0	0.295961	0.599801	0.545841
Hcn2	0.798169	0.692212	0.88503	0.63841	0.512287	0.336969	0.412474	0.611252	0.545738
Sox10	1.001277	0.774433	0.388295	0.386076	0.836837	1.058228	0.654714	0.622572	0.545318
Rpl27-ps2	0.914442	0.570005	1.143186	1.022985	1.088631	2.114119	1.181402	0.870639	0.544715
Prkd1	0.85729	0.570005	0.571593	0.511492	0.381021	1.268471	0.590701	0.054415	0.544715
Galr2	0.057153	0.199502	0.257217	0.255746	0.244942	0.184985	0.383956	0.326489	0.544715
Nqo1	1.428816	0.926258	0.678767	0.994569	1.22471	0.891894	1.218321	0.748205	0.544715
Bace1	0.489639	0.461206	0.598518	0.933219	0.362699	0.377336	0.295206	0.090647	0.544448
9030607L20Rik	0.37441	0.227845	0.387147	0.624728	0.12692	0.164319	0.524707	0.447104	0.544341
Pard3	0.674808	0.845578	1.003677	1.393673	0.906343	0.880058	0.625915	0.691905	0.544205
Gm52494	0.214047	0	0.071357	0.354746	0.067952	0.197944	0.073743	0	0.544014
Gm46731	0.325388	0.324522	0.447461	0.56624	1.239586	0.376136	0.924839	0.96813	0.542717
Ky	0.334771	0.100164	0.368291	1.597899	0.765197	1.021633	0.207601	0.09562	0.542413

Gm32827	0.448891	0.447696	0.882922	1.160579	0.399018	0.553494	1.144414	0.455881	0.541922
LOC115489886	0.920358	0.566943	0.406088	0.430684	0.489831	0.801053	0.755394	0.798953	0.54179
Clip4	0.482811	0.481526	0.681695	0.621314	0.405727	0.446488	0.117414	0.297442	0.541367
Mtus2	1.242868	1.132437	1.258359	1.022293	0.83297	0.709485	0.713648	0.61358	0.541097
Tmem190	0.189208	0.094352	0.094615	0.094074	0.2703	0	0.782225	0.36029	0.540997
Klhl35	0.453945	0.424441	0.425623	0.310339	0.297229	0.472269	0.439852	0.567263	0.540813
S100b	0.600774	0.366162	0.166901	0.365083	0.095362	0.061731	0.206977	0.158888	0.54078
Gm36819	0.275583	0.183233	0.290927	0.13702	0.262462	0.1699	0.079119	0.437303	0.539902
Gm31888	0.754259	1.128377	0.440036	0.500023	0.957798	0.232505	0.974456	0.119688	0.539157
1700037F03Rik	0.282847	0	0	0	0.538761	0	0	0	0.539157
Ccdc92	0.226124	0.488631	0.452301	1.08681	0.71786	0.243964	0.155807	0.143528	0.538791
Zfp972	1.00454	1.127099	0.56512	0.374592	0.597946	0.580605	0.778682	1.374855	0.538547
1500015A07Rik	0.713412	0.474342	0.535121	0.620739	0.169861	0.659741	0.522287	0.283016	0.53829
1700018A23Rik	0	0.469359	0.5648	0.37438	0.627488	0	0.09728	0.358455	0.538242
Mpdz	0.831417	1.064656	0.564608	1.194209	0.645197	0.759376	0.583483	0.615681	0.538059
Gm34056	0	0	0	0	0	0	0.145796	0	0.537785
Rspo1	0.729885	0.661765	0.696789	0.593833	0.221179	0.767017	0.617214	0.947621	0.537544
Gm38882	0.16919	0.956193	0.902454	0.672971	0.751964	0.678002	0.466311	0.322172	0.537512
Gm38599	0.313209	0.624751	0.375895	0.872074	0.715913	0.173788	0.906409	0.178924	0.537329
H3c8	1.12679	0.449516	0.901537	0.672287	0.214628	0.729414	1.164594	0.429126	0.536965
4930451E10Rik	0.703529	0.982318	0.281444	0	0.670033	0.910842	0.436279	1.205691	0.53642
Calr3	0.374898	0.467375	0.60928	0.652397	0.223156	0.346694	0.145303	0.044617	0.535967
Zxda	0.300735	0.47358	0.459069	0.645319	0.376864	0.44644	0.703444	0.542518	0.53554
Gm19187	0.686733	0.560376	0.124875	0.062081	0.83241	0.519602	1.032396	0.772716	0.535514
Gm13778	0.473071	1.120554	0.916683	0.676231	0.535026	0.328111	0.488943	0.337808	0.535419
D030055H07Rik	0.070219	0.070032	0.316023	0.034913	0.066876	0.032468	0.254013	0.100283	0.535401
Ppifos	0.530359	0.497832	0.343213	0.682502	0.386259	0.115402	0.515908	0.267328	0.535213
Slc7a15	0.765719	0.814593	0.765808	1.218288	0.729262	0.519283	1.160733	0.437424	0.535185
Gm18381	0.431761	0.473672	0.669307	0.536678	0.452324	0.319423	0.334685	0.431632	0.534958
LOC118567966	0.673337	0.783468	1.122358	0.223188	0.534399	0.31134	0.695927	0.213694	0.534791
Gm8055	0.7479	0.512812	0.280495	0.557783	0.089036	0.259363	0.096624	0.445046	0.534611
Gm5621	0.911119	1.397989	0.911224	1.254482	0.934489	1.361081	0.651937	1.468033	0.534386
Klf12	1.119961	1.236656	0.680055	1.670531	0.838073	1.072694	0.78547	0.952065	0.533711
1700028J19Rik	0.933301	1.16352	0.793398	0.417633	0.666649	0.733624	0.192923	0.577586	0.533711
Snord8	0	0.55849	0.560046	0.556844	0	3.624967	0.578768	2.132625	0.533711
Gm24646	0	0	0	2.227375	0	0	0	0	0.533711
Gm30466	0.677691	0.499568	0.442024	0.380897	0.322713	0.354226	0.776561	0.561068	0.533569
Slc35g2	0.349767	0.383719	0.314827	0.243465	0.899408	0.517526	0.686853	0.899134	0.533374
Gm13921	0.372693	0.18585	0.838656	0.74121	0.443686	0.689309	0.481496	0.44355	0.532814
Gjb3	0.209596	0.03484	0.10481	0.312632	0.033269	0.452264	0.180523	0.033259	0.532702
Dnah17	0.711303	0.557393	0.653313	0.664013	0.68435	0.698063	0.607638	0.490646	0.532663
Prdm16	0.591655	0.622862	0.696919	0.614489	0.550964	0.431636	0.713422	0.438133	0.532572
Gm15372	0	0.061915	0.682967	0.308665	0	0	0	0	0.532516
Gm8337	0.558369	0.689474	0.372289	0.370161	0.354523	0.491773	0.604583	0.480991	0.532175
Cdkn2b	0.119393	0.158767	0.557231	0.277023	0	0.036804	0.28793	0.151565	0.531029
Gm46994	0	0	0	0	0	0	0	0	0.531029
Zfp575	0.303632	0.429	0.582028	0.83031	0.554253	0.397784	0.810697	0.578174	0.530544
Gm16206	0.638348	0.604	0.458354	0.683601	0.88855	0.30273	0.896603	0.311677	0.530402
Gm8545	0.71533	0.198174	0.119235	0.276626	0.378485	0.367508	0.246443	0.151348	0.530268
Ccdc146	0.458031	0.309979	0.130881	0.178933	0.24927	0.211786	0.101442	0.140172	0.530089
Gm30089	0.138943	0	0.138959	0.552657	0.529309	0.12849	0	0.529148	0.529698
Asic1	0.495519	0.509176	0.375437	0.731649	0.400423	0.694304	0.698378	0.500376	0.529519
Gm46294	0.786684	0.346142	0.161983	0.529187	0.484795	0.406544	0.502195	0.660882	0.529256
Ankrd61	0.999217	1.254924	0.740247	1.987241	0.493445	0.410687	1.682985	0.387588	0.529079

Bmp6	0.573625	0.645917	0.185062	1.45363	0.40533	0.650254	0.63112	0.528529	0.529079
Gm7561	0.493331	0.061502	0.370041	0.245284	0.293652	0.228108	0.254941	0.293562	0.528962
Cyp4f39	0.710175	0.929624	0.79904	0.286893	0.887727	0.615701	0.779877	0.781806	0.528797
Mettl27	0.771648	0.408846	1.27819	0.767325	0.298557	0.468297	0.074769	0.114794	0.528604
Gm13836	0.395986	0.315946	0.158413	0	0.678839	0.073239	0	0.150807	0.528374
Tafa5	0.38336	0.318616	0.319503	0.82596	0.283973	0.275737	0.814455	0.709714	0.527765
Dnm3	1.384064	1.405477	0.805367	2.652527	1.294201	0.628334	0.2861	1.365683	0.527654
Gm44180	0.645701	0.459987	0.369015	0.733811	0.351405	0.341214	0.476689	0.614771	0.527495
Vgll3	0.336863	0.823918	0.882363	0.677928	0.259715	0.726881	0.47251	0.572726	0.527457
Amh	0.71575	0.908529	0.45553	1.099962	1.239404	1.143287	0.504384	0.898293	0.527134
Csgalnact1	1.003733	0.681882	0.552845	1.027041	0.484899	0.457384	0.225524	0.429351	0.526849
Hmcn2	1.865512	1.439837	1.163807	2.151583	0.751545	0.837362	0.86445	0.772089	0.526814
Gm8546	0.294687	0.367379	0.368402	0.146518	0.280657	0.034065	0.456861	0	0.526619
Zfp975	1.033229	1.012711	1.068981	1.682878	1.204595	1.136713	0.883774	0.661477	0.526337
Gm40781	0.309093	0.462405	0.242887	0.439088	0.315404	0.204171	0.433557	0.210205	0.526059
Sspn	0.384987	0.716728	0.4107	0.701855	0.598873	0.676445	0.278532	0.452072	0.525928
Ybey	0.637501	0.562442	0.772447	0.63393	0.700556	0.691577	0.620876	0.315154	0.525803
2810403D21Rik	0.772272	0.990277	0.662024	0.767946	0.840575	0.153037	0.798181	0.735278	0.525745
Gm6992	0.578916	0.384916	0.523842	0.493434	0.315059	0.407896	0.427384	0.734913	0.525484
Gm51554	0.310489	0.379178	0.405583	0.693111	0.452611	0.269551	0.510829	0.555033	0.525415
Gm11545	0.847677	1.162453	0.86897	0.59005	1.009148	1.117065	0.657087	0.50442	0.525142
Gm7482	0.367139	0.549243	0.091796	0.638895	1.048979	1.018557	0.284593	0.611717	0.524875
Ppp1r32	0.293517	0.219552	0.550409	0	0.524142	0.237506	0.379206	0.209593	0.524527
Gm7785	0.259878	0.297302	0.29813	0.311627	0.334859	0.325148	0.450295	0.458472	0.524513
Spats2l	0.788148	1.408958	0.922092	1.316081	0.934739	0.976391	0.614785	1.203463	0.524406
Zfp383	0.888702	0.865233	0.782995	1.535996	0.523956	1.037088	1.115343	1.067737	0.524341
Chrna2	0.282927	0.282174	0.393	0.04689	0.239517	0.188964	0.422384	0.254409	0.524329
Gm42333	0.057899	0.461957	0.550102	0.460596	0.303282	0.508658	0.807857	0.41344	0.524235
Rpl10a-ps4	0.471145	0.234945	0.392666	0.858927	0.373928	0.145233	0.811586	0.971915	0.523884
Gm16287	0.620754	0.357174	0.525314	1.02088	0.227384	0.176632	0.690932	0.477361	0.523368
Gm20717	0.905671	0.739031	0.823433	1.118925	1.097793	0.761397	0.907691	0.679378	0.523143
Gm25390	1.646675	0.54743	0.548956	0	1.045517	0	0.567307	0	0.523143
Gm31005	0.109778	0.273715	0.054896	0.054582	0.365931	0.355319	0.170192	0.574859	0.523143
Mir7093	0	0	0	0	0	0	0	1.567796	0.523143
Gm33699	0.490858	0.259174	0.519792	0.315835	0.054999	0.347123	0.149214	0.192436	0.52287
LOC118567598	0.267858	0.245943	0.323168	0.596133	0.356337	0.283093	0.51414	0.396708	0.522741
Dnaaf4	0.137053	0.273377	0.274138	0.545142	0.358952	0.316857	0.424954	0.521953	0.522496
Prss53	0.876145	0.851967	0.810528	0.500961	0.438076	0.445627	1.064009	0.688195	0.521902
Gm1866	0.117288	0.428911	0.234603	0.349893	0.372346	0.650785	0.606114	0.186116	0.521667
Sh2d5	0.441386	0.088042	0.635669	0.561809	0.386741	0.195926	0.291964	0.235336	0.521643
Gm5950	0	0.167801	0.042067	1.463933	0.160239	0.116694	0.13042	0.080095	0.521158
Dact1	0.243016	1.030069	0.516469	0.438	0.231446	0.533743	0.251169	0.506133	0.521136
LOC118568241	0.546726	1.444968	1.284957	1.549442	0.833114	1.061751	0.875857	0.832859	0.521079
Hesx1	0.601399	0.599798	0.273395	1.467893	0.572766	0.606715	0.452055	0.364376	0.521079
Acot4	0.136682	0.272635	0.164037	0.163099	0.364487	0.02528	0.254281	0.156161	0.521079
Pmel	0.416436	0.648949	0.728848	0.207052	0.421397	0.505453	0.430408	0.223025	0.520932
Gm33596	0	0	0.136563	0	0.650229	0.126274	0	0	0.520566
Prrg4	0.331148	0.718815	0.526002	0.368033	0.148415	0.252194	0.281859	0.222554	0.519834
2310030A07Rik	0	0	0	0	0	0	0	0	0.519712
Gm15682	0.907333	0.090492	1.451902	1.172926	0.518481	1.174703	0.937774	0.950257	0.518862
Gm46069	0.090733	0.180984	0.635207	0	0.345654	0.335629	0.468887	0.086387	0.518862
Gm7497	0.499033	0.226229	0.453719	1.488714	0.777721	0.419537	0.093777	0.518322	0.518862
Twist1	0.238078	0.440967	0.442195	0.439667	0.388701	0.471786	0.246065	0.485728	0.518649
Sync	0.598448	0.922412	0.979393	0.676245	0.466327	0.301869	0.702871	0.673378	0.518522

Gm19951	0.627601	0.751116	2.008556	0.416057	0.19924	0.735155	0.389194	0.796717	0.518405
Gm7266	0.951143	0.542063	0.40768	0.675582	0.905858	1.005243	2.808726	1.811163	0.518014
Gm39874	0.232933	0.851814	0.46592	1.38977	0.147895	0.502621	1.043241	0.14785	0.518014
Zbtb10	0.364675	0.296901	0.387047	0.777069	0.262256	0.550596	0.761512	0.318863	0.517805
Gm32401	0.441332	0.406299	0.679051	0.742686	0.549649	0.533708	0.666664	0.872705	0.517696
Gm52523	0	0	0	0.067476	0.12925	0.376505	0.210397	0.064605	0.51738
Casq2	0.216894	0.540791	0.216919	0.905851	0.495762	0.681961	0.156919	0.722765	0.516798
Gm5815	0.497	0.090123	0.316309	0.629002	0.172123	0	0.233488	0.473192	0.516747
Pgm5	0.632109	0.533438	0.40293	0.725271	0.674785	0.886468	0.667675	0.634898	0.516391
Nrn1l	0.09029	0.09005	0.0903	0.089784	0.343965	0.166995	0.373277	0	0.516326
Gm7867	0.36116	0.270149	0.0903	0.359137	0.601938	0.333989	0.093319	0.257895	0.516326
Lbx2	0.06769	0.540078	0.270791	0.605797	0.45127	0	0.139922	0.451132	0.516116
Gm31719	0.486773	0.377593	0.108184	0.161349	0.61813	0.150051	0.111801	0.20598	0.515487
Ccl16-ps	0.254522	0.174518	0.206823	0.53783	0.212103	0.529591	1.233099	0.48466	0.515487
Ifnk	0.338037	0	0	0	0	0	0.139751	0.064369	0.515487
Gm16225	0	0	0	0.268915	0.515108	0.50017	0	0	0.515487
Sh2d1a	0.090143	0.359613	0	0.53783	0	0.083362	0	0	0.515487
Gm12003	0.077104	0.307597	0.11567	0.115009	0.036717	0.035652	0.039846	0.036705	0.514411
Pcx	1.065598	1.318362	1.214113	1.140107	0.46247	0.411636	1.143172	0.244007	0.514233
Podn	2.810121	2.578428	2.742997	2.70496	1.220406	2.598712	0.952643	2.375853	0.514233
Musk	0.507733	0.854518	0.983846	0.378666	0.151112	0.352151	0.327979	0.75533	0.514159
LOC102631992	0.471527	0.134363	0.741057	1.47364	1.539692	0.934399	0.278484	0.577208	0.513608
H2-Q6	0.404068	0.752251	0.83517	1.017904	0.718348	0.274024	0.501149	1.256725	0.513483
Aldh3b2	0.102616	0	0.051314	0	0.024432	0.023724	0	0.024425	0.513459
Ppp1r3c	0.497202	1.053742	0.621575	0.86523	0.335417	0.919594	0.513883	0.216968	0.513368
Gm16214	0.630276	0.261916	0.28891	0.457002	0.387673	0.485717	0.298568	0.35005	0.513106
Gm22215	0	1.610402	1.076592	0.535219	1.025216	0.497742	5.562913	0	0.512984
Gm33326	0	0	0	0.053522	0	0.348419	0.055629	0	0.512984
Gm6248	0	0	0	0	0	0	0	0	0.512984
Sema3c	0.637605	0.758626	0.324433	1.21245	0.49006	0.589637	0.439331	0.48991	0.511742
Efna1	1.26431	1.184523	1.494358	2.285868	1.53251	2.515539	0.514771	0.291817	0.511212
Gm42232	0.643382	0.962503	0.536214	0.213259	0.102125	1.090795	0.886623	0.408375	0.511
Gm15484	0.357377	0.757404	0.268064	0.222109	0.808359	0.537048	0.415538	0.425322	0.510918
Lmod2	0.535497	0.337308	0.310061	0.504472	0.885793	0.703721	0.174778	0.187838	0.510376
Apcdd1	0.5513	1.034978	0.940562	0.161239	0.277969	0.49483	0.201104	0.540329	0.509984
LOC118567910	0.516475	0.321938	0.258268	0.055027	0.228375	0.187636	0.581464	0.482954	0.509827
Ttc21a	0.664414	0.404052	0.437592	0.628463	0.339541	0.554485	0.334978	0.246863	0.509686
Gm32184	1.020332	1.550653	0.777487	1.497768	1.018027	1.123299	1.406086	0.832677	0.509388
Gm13772	0.267171	0	0.668006	0.664187	0.636128	0.370608	0.414202	0.38156	0.509276
Prr22	0.369587	0.860074	0.61605	0.245011	0.312881	0.303807	0.42443	0.234589	0.508805
Gm38880	0.388075	0.306408	0.566008	0.305505	0.130899	0.321496	0.384382	0.115464	0.508571
Prdm5	0.457326	0.177375	0.533609	0.202117	0.024197	0.305443	0.42015	0.556369	0.508518
Gm13770	0.421049	0.363937	0.449171	0.334952	0.949038	0.389373	0.812326	0.45433	0.508309
Mir1970	0.533058	2.126557	0	0	1.015358	0	2.754712	0	0.508052
E130215H24Rik	0	0.118142	0.118471	0.530072	0.282044	0.273864	0.795806	0.281958	0.508052
Gm22935	0	0	0.53312	2.120289	1.015358	0	0.550942	2.537619	0.508052
LOC115490295	0	0.531639	0	0	0	0	0.550942	0	0.508052
1600002D24Rik	0.399794	0.13291	0	0.132518	0.761518	0.369717	0	0	0.508052
LOC115490297	0	0	0	0	0	0	0	0	0.508052
Rgs7bp	0.998798	1.18588	0.799132	0.520249	0.353319	0.483821	1.11096	0.498119	0.507703
Cd3e	0.190247	0.531274	0.266377	0.227018	0.398617	0.844487	0.314608	0.688309	0.507703
Gm15232	0.325063	0.559978	0.325101	0.2057	0.956904	0.546561	0.549768	0.45017	0.506969
Gm6522	1.256661	0.626657	0.773419	0.096125	0.368255	0.357575	0.249773	0.322125	0.506723
Gm5644	1.594066	0.883234	1.062834	0.616442	0.843428	1.064658	1.647546	0.505902	0.506429

Gm6654	0.797033	0.883234	0.974264	0.968695	1.939885	0.818968	1.556016	0.674536	0.506429
Gm11769	0.250003	0.093501	0.531317	0.124301	0.386912	0	0.387585	0.26778	0.506334
Gm6478	0.265572	0.662161	0.199202	0.330105	0.126464	0.184194	0.411722	0.25285	0.506227
LOC100041034	0.303355	0.680731	0.227542	0.565604	0.866735	0.596132	0.235149	0.830367	0.505967
Gm36233	0.397881	0.529095	0.928497	0.263768	1.136812	0.122649	1.644919	0.378822	0.505621
Rpl35a-ps4	0.795762	0.264548	0.265285	0.527536	0	0.490597	0.274153	0.505095	0.505621
Myf6	0.353672	0.573187	0.265285	0.439613	0.294729	1.226492	0.228461	0.084183	0.505621
Gm46282	0	0	0.066321	0.131884	0.063156	0	0.205615	0	0.505621
Klrc1	0.353484	0.058757	0.206223	0.58584	0.3086	0.408614	0.121781	0.785286	0.505352
Gm42090	0.433675	0.192231	0.024096	0.910407	0.137676	0.245085	0.672336	0.711107	0.505182
Gm51708	1.720035	0	0.132326	1.184123	0.126011	0.122357	0.957246	0.377917	0.504414
Ptprk	0.426709	0.476642	0.256055	0.695883	0.552695	0.631372	0.123487	0.373767	0.504298
Acat3	0.250599	0.305472	0.167085	0.221507	0.132593	0.334744	0.172671	0.185574	0.504224
Asap3	0.663805	0.817202	0.819479	0.515692	0.770494	0.556316	0.707515	0.632007	0.504155
Zfp94	0.613393	1.223521	0.867312	0.462726	0.3626	0.625928	0.59025	0.483319	0.503981
Gm23205	1.055963	0.526576	0	0	0.502844	0	2.182781	0	0.503213
Gm26403	1.055963	0.526576	1.056086	5.25024	0.502844	2.441304	1.637086	0	0.503213
Rpl35a-ps6	0.527982	0.921508	0.792064	0	0.502844	0.976522	0.272848	0.50269	0.503213
Sox6os	0	0	0	0	0	0	0	0.377018	0.503213
Slco2a1	3.213801	2.428975	1.632198	3.245733	3.132526	2.136141	0.934207	3.131569	0.50253
Gm10247	0.526727	0.262663	0.131697	0.523777	1.128711	1.095977	0.408299	0.752244	0.502018
Gm13864	0.657628	0.393526	0.460393	0.261578	0.68895	0.304077	0.611722	0.187838	0.501423
Gm38906	0.047791	0.428978	0.238985	0.190095	0.500676	0.397764	0.197579	0.182009	0.501044
Gm30246	2.890136	1.834281	1.313851	1.045072	2.752534	0.72892	0.814664	0.250154	0.500828
Bace2	0.197055	0.52408	1.116773	1.143047	0.406624	1.123752	0.169722	0.625385	0.500828
Clec14a	0.598472	0.426342	0.146582	1.093077	0.290805	0.734166	0.214599	0.558175	0.500553
Tgfa	0.174883	0.058139	0	1.159359	0.277595	0.107818	0.06025	0.055502	0.500038
Ptpn14	0.295029	0.397773	0.464451	0.787769	0.218542	0.454722	0.372688	0.421344	0.499891
Gm45986	0.38736	0.454504	0.273462	0.430507	0.368917	0.295003	0.541658	0.238638	0.49949
Tcte3	0.960344	1.044859	0.436571	0.086815	0.498884	0.484416	1.353496	0.498732	0.499251
Gm31512	0.305564	0.261215	0.392914	0.217037	0.498884	0.322944	0.631632	0.374049	0.499251
Tespa1	0.556344	0.522224	1.505577	2.147826	2.493441	1.573733	1.420612	1.557924	0.499055
Sall2	0.720881	1.113231	1.081447	1.086826	0.686559	0.94621	0.552791	0.586718	0.498675
Mybl1	1.613219	2.217707	1.984054	1.474114	0.872018	1.612816	1.104051	0.664192	0.498662
1810034E14Rik	1.445943	1.196631	1.661489	1.00955	0.9669	1.877718	2.289376	2.284702	0.498466
Gm14017	1.569002	0.869347	1.1333	1.300177	0.664133	0.403045	1.621642	0.165983	0.498466
Gm23733	0	0	0	0	0	0	0	0	0.498466
Gm14046	0.223841	0.520906	0.373112	0.370979	0	0.138001	0.385585	0.07104	0.497795
Krt8	0.061427	0.183791	0.153586	0.39704	0	0.170418	0.031744	0.175454	0.497638
Gm5558	1.26673	1.486303	1.117833	1.778307	0.496761	1.584879	1.771309	0.993219	0.497126
Gm38641	0.086894	0	0	0.864068	0.165513	0.401782	1.706367	0.496387	0.496903
Gm26510	1.146276	0.98733	0.625314	0.155435	1.488682	0.289102	0.70007	0.595291	0.496592
Gm51785	0.651191	0.476269	0.086836	1.122408	0.289421	0.281027	0.807646	0.702664	0.496514
H1f1	1.116203	0.9648	0.967488	1.627927	2.551342	1.445121	1.461291	1.34613	0.496459
Ccdc3	0.104089	0.477537	0.291484	0.434727	0.594802	0.827825	0.150614	0.951393	0.496033
F630048H11Rik	0.571951	0.493344	0.628704	0.840312	0.294443	0.238253	0.916001	0.490589	0.496011
Gm6855	0.649919	0.648188	0.714994	0.646278	0.061898	0.300512	0.403034	0.247515	0.495544
Gm5692	0	0	0.324997	0	0	0	0.067172	0	0.495544
Gm7020	0.285897	0.388822	0.831798	0.801197	0.222779	0.36053	0.483527	0.866098	0.495428
LOC118568683	0.27224	0.493665	0.668304	0.49221	0.447845	0.274647	0.639487	0.659781	0.495351
Gm52117	0.593714	0	0.445337	0.664187	0.353404	0.274524	0.306817	0.211978	0.49513
Gm7434	0.207633	0.20708	0.103829	0.103235	0.098874	0.192013	0.429199	0.098844	0.494732
Gm10785	0.29195	0.355878	0.356869	0.741915	0.247156	0.839957	1.139925	1.2354	0.494674
Rasgrf1	1.685836	1.099343	1.880574	0.451336	1.420313	0.239847	1.139259	1.049476	0.494385

Gm46416	0.380186	0.172352	0.48393	1.340382	0.888755	0.607282	1.214546	0.329068	0.494115
Kif5a	1.062379	1.24045	1.153204	0.888946	1.11051	0.8267	1.057851	0.77712	0.493923
Gm23731	1.036225	3.617134	0.518173	4.121684	2.467224	2.874807	3.212972	1.973176	0.493807
Gnrh1	0.388585	2.196117	1.165889	1.288026	0.616806	2.156105	3.079098	0.739941	0.493807
Gm26243	3.108676	0	0	2.060842	0	0.479134	2.677477	0	0.493807
Gm22798	2.590564	2.583667	0.518173	0	1.480335	0.479134	2.141981	1.479882	0.493807
Gm22060	1.036225	0.516733	0	1.545632	0	0	1.606486	1.973176	0.493807
Gm22063	0.518113	3.617134	0.518173	1.030421	1.97378	1.437403	1.070991	0.493294	0.493807
Gm24967	1.036225	0.516733	3.109038	0	0.493445	1.916538	1.070991	0	0.493807
Mir6362	0	0.516733	0	0	1.480335	0.958269	1.070991	0	0.493807
Gm21378	0.872611	0.734305	0.927257	0.59656	0.779124	0.781746	0.704599	0.882737	0.493807
Gm22951	0	0	0	0	0	0	0.535495	1.973176	0.493807
Gm1848	0.633249	0.459319	0.057575	0.457965	0.767581	0.266186	0.416496	0.109621	0.493807
Gm6829	0.274295	0.638318	0.701058	0.848582	0.580523	0.507319	0.377997	0.551329	0.493807
Gm22092	0	0	0.518173	0.515211	0	0	0	0	0.493807
Gm22549	0	0	0	0	0.493445	0	0	0	0.493807
Gm22682	0	0	0	0	0	0	0	0	0.493807
Gm23966	0	1.033467	0.518173	0.515211	0.493445	2.874807	0	2.959765	0.493807
Gm24205	1.036225	2.066934	1.554519	0	0.493445	0	0	0	0.493807
Gm24445	0.518113	0	0	0	0	0	0	0	0.493807
Gm25245	0	0	0	0	0	0	0	0	0.493807
Gm25670	1.036225	0	0	0	0	0	0	0	0.493807
Gm25790	0	0	0.518173	0	0	0.479134	0	0	0.493807
Gm26127	0	0	0	0	0	0	0	0	0.493807
Hspa12a	0.46554	0.335328	0.465594	0.797272	0.480323	0.490311	0.561352	0.258557	0.493001
1700001L05Rik	0.637219	0.755259	0.535696	0.624466	0.536518	0.170806	1.030849	0.483598	0.492903
A630026N12Rik	0.786062	0.949016	0.693056	0.493679	0.866843	0.27738	0.823124	0.561306	0.492886
Lrrn1	0.177118	0.309132	0.339516	0.337575	0.238971	0.150144	0.198316	0.267004	0.492361
Gstp2	0.442325	1.029344	1.105941	0.586463	0.561687	0.136349	1.295301	0.982652	0.491837
Gm28364	0.562823	0.514548	0.797425	1.026062	0.491358	0.693975	1.114936	0.535863	0.491719
LOC118567766	0.322314	0.128582	0.838115	0.224356	0.184181	0.208646	0.366441	0.061375	0.491511
Snord3b3	0	0.257165	0.257881	0	1.227875	3.576794	0.266502	2.454999	0.491511
Gm12960	0.793509	0.710916	0.609773	0.945094	0.75146	0.509938	0.973037	0.550618	0.491372
AI182371	1.145415	0.685419	0.343664	0.05695	1.254512	0.847395	0.532729	0.981492	0.491257
Slc10a1	0.294492	0.146854	0.073631	0.475868	0.31553	0.476589	0.494604	0.806107	0.491184
Aanat	0.294492	0.146854	0.073631	0.073211	0.91153	0.068084	0.456558	0.56077	0.491184
Ehd3	0.77292	0.498793	0.40924	1.49197	0.375277	0.840908	0.031328	0.981194	0.491108
Atp6v1fnb	0.085817	0	0.600792	0.341347	0.245195	0.158723	0.177393	0.081707	0.49075
Gm37164	0.367626	0.219989	0	0	0	0.067994	0.151984	0.140007	0.490533
Papln	0.437432	0.230965	0.064336	0.89555	0.551389	0.797149	0.531891	0.391979	0.490484
Gm16380	0.617121	0.205159	0.92579	0.920497	0.29387	0.66581	0	0.88134	0.490143
Gm11725	0.933301	1.442765	0.746727	1.670531	2.57771	1.337785	1.591611	0.666445	0.489235
Gm23483	1.026631	0	0	0	0.488876	0	0	0	0.489235
Gm25781	0	0	0	0	0	0	0	0	0.489235
Gm26628	0.545082	0.159891	0.256539	1.17971	0.366445	0.444772	0.994182	0.244222	0.488952
Angptl8	0	0.06392	0.320488	0	0	0	0	0.06102	0.48867
Fabp5l2	1.11047	0.085193	0.512584	1.019307	0.976245	0.157989	1.500872	0.894618	0.488481
Sypl2	0.478062	0.953578	0.631798	0.611208	0.699212	0.726301	0.29999	0.325115	0.48818
Cx3cl1	0.246862	0.721027	0.476146	0.648765	0.369456	0.391354	0.291593	0.537226	0.487368
Dzip1	0.248339	0.582772	0.540566	0.377685	0.375642	0.243165	0.196278	0.333802	0.487301
Lpar3	0.555714	0.399049	1.244945	0.331561	0.190532	0.205563	0.298666	0.761896	0.487274
Adamts5	0.443958	0.970339	0.840784	0.732654	0.548767	0.576529	0.38075	0.467659	0.486151
Ptprn	0.302853	0.429224	0.988372	0.88762	0.091084	0.353771	0.263591	0.455283	0.486141
1700084E18Rik	0.764663	0.127105	0.509835	1.520759	0.485504	0	0.13172	0.728034	0.485861

Gm21178	0.823338	0.273715	0.470533	0.116961	0.672118	0.072514	0.567307	0.261299	0.485775
Zfp11	1.005133	0.974219	1.061884	0.5631	0.930312	0.903332	0.90717	0.727848	0.485737
Chrna9	0.611449	0.609821	0	0.506687	0	0	0.210654	0	0.485638
Gm3942	0.050954	0.050818	0	0.050669	0.048528	0	0.105327	0.339593	0.485638
Cdr2l	0.509431	0.732225	0.35964	0.67047	0.513716	0.401825	0.154859	0.470763	0.485533
LOC115490409	0.169795	0.084671	0.254722	0	0.323422	0.392553	1.66717	0.646646	0.485489
Gm10516	0.148571	0.148175	0.40331	0.105527	0.363849	0.176649	0.37292	0.080831	0.485489
Lrig1	0.662099	0.52065	0.547569	1.101538	0.37592	0.600514	0.88171	0.666751	0.485415
Krt86	0.113139	0.282094	0	0.196884	0.242443	0.209255	0.087701	0.323158	0.485241
Fibin	0.643071	0.374126	0.696741	0.58618	0.45934	0.545134	0.166161	0.943911	0.485215
Gm39065	0.312988	0.702349	0.19564	0.077809	0.745217	0.217081	1.213084	0.633241	0.484747
Snord3b4	0.508606	0.507252	0	0	0	0.705514	0	1.452729	0.484747
Hrh1	0.475371	0.73568	0.57379	0.58681	1.280155	0.939852	0.57603	0.436993	0.484317
Sox9	0.147086	0.38674	0.829127	0.983945	0.114613	0.284407	0.262581	0.292812	0.484279
Gm39538	1.422357	1.080816	0.948348	0.942927	0.483799	0.626358	0.630033	0.677112	0.484155
Gm7324	0.643447	0.472857	0.643522	0.841899	0.387039	0.720311	0.350018	0.806085	0.484155
Gm32468	0	0.168826	0	0	0	0	0	0.080584	0.484007
Gm4575	0.830879	0.552444	0.323157	0.826224	0.615471	0.640309	0.858754	0.922924	0.48394
Zfp763	0.556837	0.8657	0.671558	0.667719	0.748695	1.105619	0.575519	0.374233	0.483888
Nyap1	0.491182	0.473545	0.704109	0.879175	0.670508	0.45423	0.693803	0.140296	0.483745
4833418N02Rik	0.833383	1.011852	1.014671	1.873615	1.069776	1.340324	1.19839	1.24194	0.483479
Tgm3	0.126812	0.168633	0.084551	0.273221	0.120775	0.234544	0.021844	0	0.483453
Gm9530	0.777722	0.219206	0.456542	0.706117	0.128816	0.594133	0.384433	0.33804	0.483416
Gm46599	0.338037	0.505706	0.507114	0.336143	1.448742	0.52101	2.270958	0.321844	0.483269
Gm32934	0	0	0	0.315014	0.48273	0.058591	0.327417	0.060323	0.483085
Lrig3	0.438029	0.286691	0.31487	0.612528	0.365027	0.708882	0.509316	0.182458	0.482712
Zfp521	0.687323	0.559218	0.741672	1.016217	0.353139	0.677432	0.345844	0.576905	0.482691
Clec2f	0.084381	0.252468	0.168781	0	0.321453	0.858358	0.087212	0.964063	0.482533
BC034090	0.490467	0.397443	0.628483	0.640132	0.467115	0.510264	0.475239	0.55453	0.482066
Osbpl10	0.577856	1.008556	0.433442	1.328807	0.722326	0.701378	0.335949	0.412632	0.481905
Gm11716	0.33701	0.672225	0.589835	1.759389	0.401205	1.324537	1.82866	0	0.4818
Dcst2	0.216555	0.719928	0.252677	0.215342	0.206245	0.700921	0.559551	0.652908	0.481591
Gm10193	0.252565	0.125946	0.378892	0.25115	0.481081	0	0.130519	0	0.481434
Fam186b	0.156556	0.503114	0.330545	0.709203	0.115968	0.080432	0.485424	0.496854	0.480792
Gm30421	0.933216	0.628873	0.731524	0.852746	0.504445	0.769711	0.495297	0.288166	0.480777
0610040B10Rik	1.385952	1.130941	0.504041	0.25058	0.719981	0.582584	0.130223	0.599801	0.48034
Mir10a	0	0	1.008082	0.501159	0	0	0	0	0.48034
Mir199b	0	0	0	0	0	0	0	0	0.48034
Mir219a-1	0	0	0	1.002319	0	0.932134	0	0	0.48034
Mir6541	0	0.502641	1.008082	0	0	0	0	0	0.48034
Gm46401	1.655942	0.359029	1.72814	0.286377	0.205709	0.199743	1.48826	0.616938	0.48034
Gm17893	0	0.071806	0.072006	0.214783	0.137139	0.399486	0.595304	0.274195	0.48034
Hdac11	0.554381	0.904753	0.85687	0.576333	0.815978	0.932134	0.468802	0.959681	0.48034
Gm45974	0.167994	0	0	0.167053	0.319992	0	0	0	0.48034
Srpk3	0.335819	0.167462	0.615739	0.862672	0.719618	0.983423	0.896637	0.746042	0.480098
Gm15483	0.692582	0.313972	0.094454	0.375656	0.149911	0.43669	0.553132	0.419622	0.480067
Gm31224	0.50322	0.418234	0	1.167603	0.159754	0.07756	0.086684	0.399262	0.479613
LOC118568478	0.213191	0.255148	0.281444	0.729267	0.609121	0.331215	0.749166	0.243574	0.479527
Gm20072	0	0.752251	0.251449	0.87504	0.359174	0.930021	0.259855	0.119688	0.479251
Ret	0.950785	0.720308	0.484591	1.33637	0.748793	0.760895	0.500791	0.722451	0.479231
Zfp783	0.489713	0.250466	0.326514	0.511943	0.346808	0.313526	0.467209	0.514076	0.478708
Gm13073	0.23434	0.066776	0.267848	0	0.350715	0.278627	0.138401	0.09562	0.4786
Khdrbs3	0.84933	0.962578	0.849428	1.804313	0.955964	0.642627	0.279308	0.624862	0.478333
Gm32336	0.354237	0.441617	0.472371	0.763214	0.365486	0.272989	0.976324	0.365374	0.478294

Gm4468	0.501702	0.500366	0.25088	0.249446	0.716723	0.463958	0.777801	0.716504	0.478167
Gm40785	0	0	0	0.124723	0.477815	0	0.648167	0.119417	0.478167
Gm3076	0.668936	0	0.25088	0.498892	0	0	0.172845	0.238835	0.478167
LOC118568390	0.510044	0.571206	0.364766	0.790531	0.469478	0.569169	0.948291	0.556148	0.47797
Il2ra	0.525835	0.237245	0.626067	0.49799	0.834666	0.659946	0.414078	0.452966	0.477303
Ppp1r27	0.572264	0.356713	0.286165	1.493778	0.681272	0.661515	0.147866	1.430235	0.477241
4930597O21Rik	0.041714	0.124809	0.125157	0.497765	0	0.424335	0.388023	0.19858	0.477087
Gm30108	0.125142	0.124809	0.125157	2.364386	0.119184	0.347183	0.129341	0	0.477087
Gm4217	0.591218	0.408215	0.363869	0.587906	0.43313	0.883195	0.423037	0.346398	0.476794
LOC100040974	0.92873	0.783757	0.285796	0.852487	1.22471	0.52853	1.107565	0.544149	0.476626
Gm6257	0.277746	0.387809	0.555556	0.441904	0.529044	0.924662	0.114826	0.264441	0.47649
Aldh1l2	0.545069	0.702173	0.817699	0.67752	0.237929	0.693086	0.258205	0.475713	0.476208
Gm30470	0.249721	1.245281	0.124875	0	0.475663	0.692802	0.774297	0.118879	0.476013
Man2c1os	1.123745	0.435848	0.74925	0.062081	0.059458	0.577335	0.258099	0.416078	0.476013
Gm22378	1.997768	0	0.4995	0.496644	0.951326	0.461868	0	2.853106	0.476013
LOC115488669	0.743138	0.857627	1.008661	0.781202	1.051523	0.618507	1.76656	0.454847	0.475557
BC025920	0.205326	0.351051	0.264021	0.379184	0.307293	0.325507	0.545695	0.251345	0.475257
Gm39000	0.643663	0.496993	0.789098	0.743292	0.692117	0.537635	0.708178	0.533755	0.474943
A430093F15Rik	0.629084	0.261421	0.734017	0.15639	0.624097	0.193919	0.298004	0.573994	0.474662
LOC118568607	0.153144	0.152736	0.536066	0.304572	0.364631	0.389462	0.276993	0.218712	0.474369
Kif7	0.806185	1.16329	0.583265	0.90401	0.833146	0.475873	0.797775	0.097987	0.474098
Trp53cor1	0.319733	0.637763	0.42636	0.388595	0.406013	0.032853	0.348818	0.541185	0.47403
Gm6195	0.124301	0	0	0	0.118383	0.574746	0.128471	0.118346	0.473878
B4galnt4	0.240904	0.255279	0.466806	0.284471	0.043019	0.431637	0.062247	0.387052	0.473556
Gm51779	0.67348	0.777743	0.77991	0.599212	0.810209	0.622813	0.512898	1.349935	0.472969
Fn3k	0.055107	0.219843	0.110228	0.054799	0.472353	0	0.056956	0.104935	0.4727
Gm45915	0.234907	0.130156	0.156623	0.233591	0.323155	0.337921	0.809294	0.248505	0.472651
Pik3c2b	0.327941	0.239318	0.15999	0.540856	0.319945	0.369841	0.099203	0.152308	0.472647
Ddah1	0.611388	0.395521	0.694089	0.706552	0.75539	0.85573	1.144252	0.849554	0.472466
LOC118568654	0.49572	0.576801	1.404704	2.218246	0.393432	0.916853	0.341568	0.629299	0.472466
Ccna1	0.571526	0.418004	0.457274	0.189442	0.834617	0.458059	0.433181	0.362766	0.472087
LOC115487244	0.494983	0	0	0.49221	0	0	0.511589	0	0.471762
Kirrel3	0.530001	0.49335	0.388712	0.281084	0.824452	0.637166	0.693857	0.42051	0.471462
9530062K07Rik	0.635596	0.704337	0.07063	0.491583	0.067259	0.52247	0.218973	0.336194	0.471162
Gm13624	0.712853	0.273444	0.548413	0.163583	0.313345	0.304257	0.510071	0.365457	0.470363
Gm19244	0.164505	0.164067	0.658095	0.163583	0	0	0.170024	0	0.470363
Dhrs7c	0.098644	0.344336	0.246639	0.196183	0.751582	1.642016	0.305861	0.140878	0.470084
Zbtb11os1	0.205326	0.163824	0.16428	0.163341	0.19555	0.151903	0.297101	0.273687	0.469666
Rasl11a	0.54158	0.14731	0.09848	1.51772	1.031589	0.500836	0.915954	0.656265	0.469249
Gm29539	0.295407	0.098207	0.984805	1.077092	0.187562	0.364244	0.610636	0.093752	0.469249
Gm33345	0.492054	0.490744	0	0.08155	0.234313	0.151679	0.339041	0.156161	0.468971
4930447F24Rik	0.147572	0	0.295179	0.195661	0.234244	0	0.203365	0.187338	0.468832
Gna14	0.163872	0.065374	0.376951	0.293318	0.078035	0.333397	0.16937	0.436863	0.468555
BC051142	0.327551	0.204175	0.450435	0.773577	0.467934	0.151455	0.846352	0.467791	0.468278
S100a3	0.245663	0.408349	0.655179	0.081429	0	0.378637	0	0.077965	0.468278
Gm33971	0.061393	0.673527	0	0	0	0.283873	0	0.467619	0.468105
Camk2n1	0.322225	0.229548	0.567796	0.335678	0.438405	0.297984	0.190306	0.277572	0.467976
Zim1	0.807581	1.121286	0.934369	0.740072	0.346863	0.16108	0.523718	0.708591	0.467855
Mir496b	0.490602	0.489296	0.490659	0	0	0	3.042372	0	0.467588
Gm19791	0.070086	0.279598	0.140188	0.836321	1.001238	0.38888	0.651937	0.266915	0.467588
Gm20875	0.183773	0	0.490117	0.487315	0.466728	0.56649	0.316564	0.233293	0.467071
Zfp2	0.290347	0.542951	0.580761	0.830071	0.70859	0.201378	0.356354	0.138219	0.466975
Celf3	0.85091	1.054376	1.005738	1.743568	1.08053	1.049193	1.279211	0.6874	0.466935
Ttll7	0.636375	0.781146	1.02811	1.143927	0.279728	0.814846	0.53124	0.442767	0.466555

Gm35636	0	0	0	0	0	0.452692	0	0	0.466555
Lurap1l	0.03263	0.325429	0.228435	0.356917	0.310763	0.331925	0.202347	0.528135	0.466487
Clnk	0.419456	0.941263	0.349587	0.278071	0.83226	0.969749	0.108382	0.332802	0.466408
Gm6375	0.685025	0.488001	0.538296	0.291937	0.419406	0.723988	0.606863	0.372692	0.46635
Misp3	0.916837	1.280154	1.650498	2.309643	1.338884	1.243531	1.516154	1.105697	0.466041
Tmem82	0.461758	0.623068	0.706299	0.756282	0.77607	0.527494	0.617617	1.293054	0.465984
S1pr5	0.488833	0.110803	1.022223	0.817522	0.846471	0.883566	0.390407	1.375094	0.465901
Hoxa1	1.049686	0.706043	0.610353	0.388393	0.74397	0.203173	0.151382	0.185936	0.465323
Gm9168	0.487904	0.121651	0.24398	0.606463	0.174253	0.394798	0.315171	0.348399	0.465016
Gm15787	0.620906	0.353859	0.354845	0.44102	0.464628	0.73825	0.687576	0.21113	0.464969
Gm31348	0.04435	0.353859	0.266134	0.176408	0.295672	0.246083	0.183354	0.886745	0.464969
Gucy2c	0.086084	0.057237	0.200886	0.014267	0.136642	0.119412	0	0.163921	0.464925
Ikzf2	0.292678	0.316224	0.29881	0.654836	0.569101	0.507486	0.768847	0.487651	0.464913
Idi1-ps1	0.221664	0.442147	0.177351	0.154295	0.084444	0	0.2291	0.506509	0.464783
Gm11475	0.194861	0	0	0	0	0	0.100699	0	0.464301
Col26a1	0.024358	0.3401	0.584652	0.266434	0.672741	0.337878	0.075525	0.278291	0.464301
A430035B10Rik	0.200448	0.199914	0.315026	0.455599	0.27272	0.317773	0.384749	0.599801	0.463965
Gm16982	0.042319	0.147723	0.232782	0.547067	0.28213	0.215245	0.39365	0.161168	0.46384
Gm36591	0.637605	0.368157	0.385964	0.200221	0.511367	0.232751	0.641654	0.447309	0.463767
Dnhd1	0.611966	0.515564	0.46378	0.529164	0.57921	0.376113	0.569641	0.390847	0.463708
4921529L05Rik	0.460917	0.893841	0.614627	0.432872	0.097549	0.09472	0.793968	0.658257	0.4637
Cd96	0.054033	0.053889	0.243178	0.698497	0.488876	0.474698	0.167538	0.334392	0.463486
Mira	1.042069	0.762149	1.04219	1.657971	1.124782	0.963673	0.287208	0.396861	0.463486
Gm22056	0	0	0.486355	0	0	0.449714	0	0	0.463486
Capn3	0.27476	0.295107	0.507308	0.357289	0.946067	0.840449	0.677178	0.664057	0.463309
Gm9085	0.242936	0.24229	0.291557	0.628096	0.046274	0.314524	0.703043	0.04626	0.46308
Rpsa-ps11	0.269903	0.592206	0.269934	0.214713	0.205642	0.249598	0.167375	0.462553	0.463035
Icam4	0.546487	0.484473	0.607278	0.060381	0.578298	0.112305	0.502064	0.404685	0.462978
Mcts2	0.346922	0.276798	0.346962	0.344978	0.066081	0	0.573697	0.462425	0.462906
B3gnt4	0.441386	0.264126	0.794587	0.746153	0.924816	0.571452	1.414202	1.008582	0.462748
Gm20337	0.776715	0.145246	0.388403	0.772365	0.1387	0.269356	0.250867	0.69329	0.462674
LOC108167852	0	0	0	0	0	0	0	0	0.462472
Gm14051	2.586032	1.45077	1.131521	5.143093	2.770772	1.943079	4.00919	3.385464	0.462135
Gm22009	0.323254	2.579147	0.484937	1.446495	2.155045	0.747338	0.835248	1.692732	0.462135
Dnaja4	0.9883	0.911279	0.596779	0.648995	1.296434	1.414035	0.520365	0.869943	0.462083
LOC115489130	0.292906	0.516838	0.354908	0.957814	0.316513	0.333379	0.791763	0.418313	0.461696
Gm33548	3.026095	0.362165	0.363174	2.527681	0.230562	2.014875	1.376153	1.959175	0.461462
Gm40636	0.423653	0.241443	0.605289	0.180549	0.230562	0.251859	0.938286	0.979587	0.461462
4930570G19Rik	0	0	0	0.080244	0.461123	0	0	0.384152	0.461462
Insyn1	0.789426	0.253976	0.483898	0.405163	0.436553	1.08328	0.605353	0.169719	0.461144
Gm4035	1.1281	0.160728	0.161176	0	1.07439	0.447099	0.166564	0.460312	0.460791
Icam5	0.297454	0.25958	0.278896	0.110921	0.300998	0.240692	0.403507	0.194704	0.460688
B3gat2	0.228768	0.507019	0.368613	0.40442	0.22998	0.199804	0.302122	0.121005	0.460298
Il12rb1	0.578887	0.153959	0.559656	1.515863	0.937253	0.820849	0.558421	0.551157	0.459775
LOC118568224	1.042478	0.962113	0.435713	0.340389	0.637199	0.561164	0.948802	0.592562	0.459714
Neb	0.677559	0.615013	0.667486	0.502169	0.39153	0.389563	0.41965	0.490472	0.45954
Gm25364	0	0.961573	0.482126	0	1.377355	0	2.491218	0.917956	0.459456
Bmp2	0.96387	0.558176	0.342058	0.881175	1.140071	0.991994	1.189022	0.666072	0.459327
Rasd1	0.034412	0.446168	0	0.787047	0.098321	0.254587	0.071133	0.393166	0.45917
1810024B03Rik	0.613059	0.960814	0.832106	0.740259	1.209447	0.566938	0.950441	0.625385	0.459093
Gm36493	0.214047	0.213477	0.053518	0.638543	0.509639	0.346401	0	0.356638	0.459012
Ppef2	0.866822	0.576343	0.963247	0.670418	1.192463	1.175694	0.736632	0.843639	0.458977
Spock2	0.948506	0.663599	0.976933	0.534945	0.471898	0.117826	0.234108	0.539147	0.458752
Gm3336	0.14437	0.239976	0.770063	0.574245	0.549985	0.222515	0.149214	0.183272	0.458658

Mex3a	0.115774	0.364162	0.454244	0.469359	0.195079	0.189422	0.386589	0.330685	0.458349
Ccdc85c	0.480602	0.518186	0.428695	0.529576	0.222675	0.264265	0.228225	0.037101	0.458056
Gm45978	0.252911	0.176566	0.12647	0.201195	0.048174	0.327438	0.209117	0.216716	0.457988
Gm13375	1.080742	1.157707	1.401125	1.194098	1.410504	1.147501	1.323853	1.181413	0.457797
Srp54a	0.974639	0.609341	1.062044	0.96918	0.789693	0.322859	0.751745	0.623251	0.457527
Gm5643	0.287907	0.095713	0.415914	0.159052	0.152333	0.207081	0.330629	0.395945	0.457335
Gm29941	0.137078	0.332016	0.195848	0.165519	0.279752	0.226366	0.172036	0.438145	0.457265
Trgv1	0	0	0.319565	0	0	0	0.165124	0	0.456807
Fbxl22	0.563538	0.519885	0.408613	0.742505	0.509872	0.586285	0.742617	0.469476	0.456537
Gm41750	0	0	0.239501	0	0.456143	0	0.247508	0	0.456479
LOC105243223	0	0.119418	0.119751	0	0	0	0	0	0.456479
Gm5160	1.354091	0.317761	0.47797	0.554443	0.22758	0.22098	0.411624	0.606695	0.455495
Zfp964	0.31861	0.41309	0.414241	0.38019	0.394472	0.265176	0.09879	0.182009	0.455495
Gm25313	0.955829	4.76642	0.47797	2.851424	2.275802	3.093721	2.96369	0	0.455495
LOC118567680	0.584118	0.900324	0.371754	2.112166	0.657454	0.687494	1.317196	0.707811	0.455495
Gm24843	0	0.476642	1.91188	0	1.365481	1.325881	0.493948	0	0.455495
Gm39012	0.119479	0	0	0	0.11379	0	0.493948	0.341266	0.455495
Snora15	0	0.476642	1.91188	0.475237	0.45516	0.44196	0	0	0.455495
Gm33424	0.57251	0.095164	0.477147	0	0.181751	0.35296	0.295859	0.454238	0.454711
Gm13642	0.381673	0.237911	0.095429	0.094884	0.136313	0.4412	0.09862	0.317967	0.454711
Gm18190	0.667928	0.095164	0	0.094884	1.090505	0.08824	0.09862	0	0.454711
Gm36572	0.619136	0.708593	0.629359	0.777155	0.289996	0.234655	0.671379	0.570151	0.454661
Gm39193	0.476887	0	0.476942	0.948431	0.113545	0.330757	1.725101	0.113511	0.454515
Gm21014	0.043311	0.345565	0.64974	0.043068	0.247493	0.520684	0.313348	0.494836	0.454071
Hsp25-ps1	0	0.271364	0.952421	0.405847	0.518269	0.566143	0	0.388583	0.453818
Gm52062	0.222197	0.221605	0.190476	0.441904	0.256964	0.293544	0.426495	0.377773	0.4538
Gm26535	0.256319	0.474753	0.036621	0.036412	0.244115	0.338622	0.113536	0.174315	0.45369
Rbms3	0.487796	0.571635	0.46346	0.776102	0.476186	0.620261	0.415934	0.661813	0.45329
Gm39547	0.237762	0.513779	0.158526	0.512264	0.717065	0.25652	0.532433	0.603659	0.453216
Tex15	0.01978	0.085487	0.013189	0.163914	0.043957	0.164631	0.013629	0.037666	0.452461
Gm51584	0.079084	0.236621	0.474561	0.078641	0.376595	0.365673	0.163475	0.150592	0.452246
Gm46800	0.059292	0.295671	0.237196	0.05896	0.282345	0.054831	0.245125	0.225807	0.452085
Wnt8a	0.328252	0.472879	0.10943	0.290145	0.138944	0	0.263872	0.069451	0.451899
LOC118567474	0.189532	0.378055	0.189554	0.37694	0.541524	0.525819	1.46918	0.631585	0.451602
Gm8054	0.236915	0	0.592356	0.235588	0.112818	0.109546	0.244863	0	0.451602
Gm17511	0.236915	0	0.236942	0	0	0.109546	0	0.112783	0.451602
Gm46958	0	0	0	0	0	0.438183	0	0	0.451602
Sctr	0.315587	0.786866	0.368227	0.470728	0.150281	0.048641	0.21745	0	0.451173
Gm371	0	0.03628	0	0	0	0	0	0.034634	0.450713
Six4	0.472405	0.914579	0.778169	0.635555	0.238189	1.00222	0.086162	0.793721	0.450243
Cep112	1.06271	1.825351	1.476158	0.704505	0.955885	0.655174	0.671223	0.168634	0.450159
Armc3	0.636275	0.716463	0.780041	0.265331	1.036033	0.778216	0.381845	0.64488	0.44993
Pcdh18	0.332543	0.342356	0.557878	0.682694	0.561905	0.466247	0.288264	0.3064	0.449854
Ccdc40	0.453815	0.543128	0.562796	0.649833	0.62238	0.671479	0.712942	0.553058	0.449827
Gm15568	0.275224	0.117639	0.432546	0	0.149783	0.03636	0.243821	0.149738	0.44968
Gm4853	0.117953	0	0	0	0.44935	1.418034	0.243821	0.224606	0.44968
Gm5512	0.220117	0.250893	0.31449	0.469037	0.539067	0.319876	0.422504	0.149695	0.449552
Nrgn	0.471631	0.213807	0.171522	0.426354	0.204171	1.149849	0.265884	0.204109	0.449506
Igsf10	0.523865	0.653088	0.759693	0.911629	0.623655	0.581345	0.250417	0.511241	0.449361
Rgs4	0.226201	0.676796	0.490159	1.387092	0.359052	1.220237	0.175342	0.412784	0.449145
Plk-ps1	0.418796	0.652626	0.549733	1.275377	0.49857	0.459906	1.21738	0.747627	0.449043
Ccdc159	0.760418	0.650051	0.86915	0.612128	0.931131	0.301376	0.299402	0.620565	0.448652
Gm30908	0.235156	1.084702	0.499765	0.146149	1.063811	0.43493	0.334188	0.335838	0.448249
Gm35365	0.527303	0.61118	0.55587	0.892811	0.271458	0.065896	0.972151	0.32565	0.448235

2010308F09Rik	0	0	0	0	0	0	0	0	0.447775
Mamdc4	0.81108	0.879879	0.910793	0.919735	0.6776	0.802698	0.94124	0.677393	0.447545
Prx	0.276649	0.515836	0.61351	1.184123	0.778977	1.223565	0.261067	0.927616	0.447094
Pcsk6	1.067368	1.142419	0.729019	2.032172	0.89258	1.035218	0.497776	0.755983	0.446618
1700003F12Rik	0.390409	0.467243	0.156182	0.6988	0.446185	0.505453	0.56491	0.371708	0.446513
Edaradd	0.663228	0.972739	0.351162	0.737103	0.891743	1.190587	0.967735	1.002904	0.446199
Astl	0.443505	0.221162	0.24642	0.563526	0.23466	0.318997	0.356521	0.21113	0.446182
Nr3c2	0.121347	0.069156	0.19071	0.517144	0.462277	0.032062	0.107501	0.099029	0.446094
Eya2	0.285629	0.647429	0.207755	0.516417	0.32149	0.600321	0.10735	0.222502	0.445468
Gm16068	0.155725	0.15531	0.311486	0.696837	0.148311	0.072005	0.643798	0.074133	0.445259
Gm39959	0.31145	0	0.934458	0	0.148311	0	0	0.296531	0.445259
Tbx2	0.466912	0.217312	0.077828	1.176219	0.578087	0.719643	0.337804	0.755729	0.445009
Gm11210	0.254303	0.295897	0.211944	0.168586	0.363293	0.156781	0.087612	0.363182	0.444351
Gm15559	0.466072	0.220183	0.220797	0.317105	0.186898	0.317586	0.785946	0.373681	0.444208
Cd6	0.381309	0.211274	0.360167	0.589824	0.443855	0.783605	0.459785	0.201691	0.444181
n-R5s197	0.465866	1.393877	0.93184	1.853026	0	0	1.925983	0	0.444012
Hsd17b14	1.449361	1.755253	1.397761	0.977986	0.591581	1.292455	0.802493	0.492834	0.444012
1700071G01Rik	0.155289	0	0	0	0	0	0.481496	0	0.444012
n-R5s33	0	0.929252	0	0	0	0	0	0.44355	0.444012
Foxp3	0.075119	0.119871	0.405692	0.552769	0.772663	0.805829	0.512421	0.286084	0.443891
Chchd2-ps	0.697822	1.082611	1.240716	1.387826	0.664598	1.720863	0.721234	1.107325	0.443391
Ccn3	0.465084	0.487039	0.30234	1.45681	0.819441	0.602134	0.024034	0.487087	0.443267
Gm46758	0.348667	0.579565	1.278594	1.040142	0.110689	0.107479	0.840851	0.553275	0.443081
Gm15246	0.302178	0.811391	0.395202	0.600971	0.265653	0.429915	0.696705	0.86311	0.443081
1700034E13Rik	0	0	0	0	0	0	0	0.110655	0.443081
Enpp2	0.561349	0.501939	0.967956	0.712192	0.479317	0.232708	0.360113	0.589748	0.442771
Igdcc4	0.30971	0.617771	0.856356	0.61595	0.20821	0.530701	0.33893	0.433638	0.442771
C030034I22Rik	0.403626	0.281786	0.787163	0.642184	0.384409	0.24262	0.771761	0.307433	0.442395
Rcan2	0.231958	0.533864	0.14276	0.53229	0.59477	0.759028	0.295065	0.713506	0.442154
Gm30476	0	0	0	0	0	0	0	0.110424	0.442154
A930019D19Rik	0.289343	0.230858	0.347252	0.632988	0.055113	0.42812	0.47848	0.716255	0.441231
Col6a6	0.602892	0.643238	0.392627	1.422106	0.681014	1.387357	0.941925	0.61406	0.44098
Kiss1r	0.444097	0.332186	0.24058	0.386408	0.299592	0.444911	0.535495	0.405206	0.4409
S100g	0.115496	0.115188	0	0.459396	0	0	0.119371	0	0.440312
Gm19486	0	0.307169	1.84815	0.918792	0.293326	0.569638	0	0.293236	0.440312
Chst2	0.248681	0.372028	0.444125	0.715368	0.2453	0.62421	0.247845	0.372065	0.440171
Igsf23	0.057688	0.345206	0.057695	0.286824	0.054941	0.373436	0.119247	0.054925	0.439853
Gm43464	0.296636	0.394462	0.626305	0.5244	0.439465	0.36576	0.170327	0.596235	0.439788
Gm40260	0.345768	1.034541	0.576346	0.11461	0	0.63951	0.238245	0.21947	0.439396
Slc26a1	0.568517	0.413759	0.614684	0.993151	0.746322	0.369443	0.666995	0.336474	0.439335
LOC118567843	1.02426	0.91938	0.94755	0.58565	0.414585	0.781443	0.89983	0.487598	0.439295
Gm38927	0.460887	0.131331	0.592637	0.327361	0	0.547989	0.06805	0	0.439266
Guca2b	0.09209	0	0	0	0	0	0.095179	0	0.438849
LOC108167553	0.492101	0.485513	0.433946	0.678768	0.594658	0.357213	0.61252	0.357694	0.438757
Armcx1	1.073489	0.586298	0.639056	1.347053	0.292108	0.543638	0.581169	0.876058	0.438485
Gm36317	0.53491	0.565495	0.855955	0.872338	0.387176	0.168187	0.762941	0.315758	0.438442
Crlf1	0.752553	1.125824	2.383361	1.662972	0.39818	0.657274	0.432112	0.477669	0.438319
Gm12103	0	0.763681	0.612646	0	0	0	0	0.583232	0.437879
Gm11953	0.275583	0.687123	0.55123	0.182693	0.393693	0.3398	0.427243	0.349842	0.437758
Pla2g2d	0.357171	0.254443	0.408243	0.177585	0.194381	0.188743	0.421891	0.242901	0.437677
Gm10045	0.918229	0.457892	0.229584	0.342407	0.765197	0.636862	0.474518	0.655683	0.437577
Gm5445	0.114779	0.343419	0.229584	0.114136	0.109314	0.106144	0.118629	0.218561	0.437577
Dock3	0.293289	0.133537	0.28057	0.393089	0.425061	0.229951	0.757823	0.27924	0.437526
Gm38482	0.344158	0.286035	0.631029	0.741498	0.109257	0.159133	0.711408	0.300366	0.43735

LOC115486421	0.45841	0.53093	0.739457	0.426433	0.366168	0.464948	0.244537	0.225265	0.436906
Rpl31-ps12	0	1.370838	0.22911	0.911199	1.199968	0.317773	0.947074	0.981492	0.436673
Stpg4	0	0.195834	0.13092	0.065086	0.62336	0.726338	0.405889	0.436219	0.436673
Gm24726	0.458166	0	0	0	0	0.423697	0	0.436219	0.436673
Gm34255	0.025442	0.634356	0.254449	0.202396	0.290768	0.423503	1.209595	0.581358	0.436472
Hoxa3	0.261603	0.369618	0.087211	0.260138	0.145336	0.483845	0.045063	0.228316	0.436329
Tpd52l1	0	0	0.137352	0.045522	0	0.381013	0.047315	0.392273	0.436312
Cox4i2	0.305024	0.380265	0	0.303315	1.162005	2.256611	0.078814	0.435619	0.436072
Mfsd6l	0.242205	0.724681	0.565211	0.615501	0.410086	0.398193	1.251655	0.435583	0.436037
Gm12571	1.485969	0.228002	0.685911	0.90932	0.435452	0.105706	0.708841	0	0.435772
5830448L01Rik	0.32649	0.45587	0.391834	0.324662	0.031095	0.030193	0.371188	0.310851	0.435644
LOC100044633	0.228454	0.151897	0	0.227174	0	0.070422	0.550942	0.145007	0.435473
Gm35597	0.6229	0.621241	0.456846	0.825881	0.079099	0.691246	1.416355	0.15815	0.435364
Ncald	0.422933	0.269957	0.659852	0.807483	0.966712	0.891743	0.174849	0.676492	0.43534
Mup-ps22	0	0.455066	1.140833	0	0	0.210977	0	0.651635	0.434876
Ctla4	0.243283	0.060659	0.091242	0.66528	0.57925	0.309348	0.345737	0.173722	0.434756
Hrc	0.364874	0.545854	0.410531	0.680307	0.434378	0.84356	0.094279	0.151986	0.434697
Gm36279	0.179148	0.503527	0.472353	0.404873	0.449812	0.105426	0.218823	0.279108	0.43462
Gm36457	0.820629	0.727506	0.455958	0.544022	0.868398	0.674571	0.565441	0.694506	0.434518
LOC118567533	0.359538	0.440824	0.270509	0.396884	0.455512	0.283683	0.651152	0.508762	0.433841
Gm18030	0.834225	0.075637	0.075847	2.413243	0.794507	0.490933	1.175746	0.722058	0.433686
Gm7832	0.417113	0.453821	0.682627	0.527897	0.397254	0.315599	0.391915	0.505441	0.433686
Slc23a1	0.58447	0.172715	0.303094	0.279835	0.247397	0.100093	0.313226	0.432812	0.433263
H3c10	0.568013	0.453201	0.227232	0.90373	0.108194	0.420224	0.704484	0.648965	0.433093
Gm22303	0	0	0	0	0	0	0	0	0.433093
LOC118567475	0	0.906401	0	0	0.432775	0	0	1.730573	0.433093
Snora69	0	0.453201	0	0	0	0.420224	0	0	0.433093
Scara5	0.521414	1.476202	0.773801	1.58893	0.192228	1.026592	0.59106	0.224197	0.432831
Rps2-ps5	0.90789	0.679105	0.907996	0.451402	0.594457	0.839589	1.231585	0.4322	0.43265
Gm5842	0.259359	0	0.453932	0.19343	0.308764	0	0.402091	0.432137	0.432587
Gm35290	0.644906	0.559813	0.549429	0.807555	0.545957	0.331327	0.69123	0.170559	0.432534
Kbtbd8	0.760265	0.562566	0.625452	0.451165	0.385391	0.510292	0.798446	0.396948	0.432423
Gm45607	0.998067	1.085901	0.090744	0.721801	0	0.083907	0.468887	0.086387	0.432385
Gm52681	0.501378	0.642912	0.238779	0.261155	0.341076	0.110395	0.913017	0.204583	0.432347
Phf2os1	0.661101	0.320251	0.510052	0.976706	0.701583	0.698704	0.50758	0.323708	0.43206
Alox12e	0	0.166538	0.095429	0.355814	0.477096	0.683859	0.862922	0.59051	0.431975
LOC115487084	0.113293	0.30131	0.113306	0.375528	0.467563	0.034923	0.819658	0.323598	0.431913
Bnc2	0.377643	0.640285	0.73649	1.089031	0.413613	1.466778	0.72208	1.168549	0.431913
Gpc4	0.309976	0.665864	0.596177	1.114406	0.363345	0.242555	0.320376	0.794575	0.431789
Lsmem2	0.226047	0.394529	0.056518	1.123905	0.376748	0.313562	0.408854	0.484243	0.430886
Jpx	0.145813	0.261764	0.349992	0.521986	0.333289	0.323624	0.587749	0.374836	0.430815
Syne4	0.150511	0.45033	0.451584	0.898005	0.238908	0.510354	0.15556	0.382136	0.43035
Hmgb1-ps6	0.72232	1.350744	0.632103	0.448921	0.343965	0.584482	0.746554	0.34386	0.430272
Nppa	0.322314	0.321456	0.06447	0	0.245575	1.192265	0.199877	0	0.430072
Gm12258	0.34162	0.504251	0.546655	1.127825	0.481525	0.606565	0.423697	0.650511	0.429784
Rgl3	0.397881	1.190465	1.061139	0.764927	1.263125	1.152903	1.34335	1.010191	0.429778
Gm32611	0.708268	1.091682	0.70835	0.384164	0.674547	0.833616	0.332741	0.429126	0.429572
Gm41699	0.450716	0.224758	0.676153	0	0	0.729414	0	0.429126	0.429572
Nos2	0.232506	0.159422	0.348799	0.404606	0.345993	0.215014	0	0.12452	0.429347
Plet1	0.600629	1.078254	0.780909	0.388222	0.200211	0.305494	0.651819	0.428893	0.42934
Gm10654	0.160877	0.192538	0.032179	0.19197	0.030643	0.029755	0.266038	0.122536	0.429323
Gm40930	0.122832	0.530854	0.122846	0.855006	0.077989	1.060182	0	0.389826	0.429255
Gm14251	1.198661	0.747169	1.4985	1.34094	0.285398	0.692802	2.322892	1.997175	0.428411
Cd8b1	0.224597	0.037333	0.411809	0.409455	0.499109	1.176969	0.657708	0.178199	0.428122

Gm35714	0.128329	0.447955	0	0.063805	0.061109	0	0	0	0.428081
A530072M11Rik	0.299342	0.149272	0.209563	0.50603	0.313599	0.193775	0.123754	0.142501	0.427949
Clec4g	0.448891	0.447696	0.628521	0.446377	0.470271	1.452922	0.185581	0.213694	0.427833
9330159M07Rik	0.305837	0.366027	0.26509	0.364949	0.504878	0.452526	0.674342	0.349424	0.427518
LOC118567527	0.480399	0.276825	0.469778	0.626329	0.477861	0.592344	0.463415	0.172791	0.427339
Ctnnal1	0.215842	0.463652	0.182656	0.561346	0.537632	0.552748	0.257403	0.584891	0.427257
Clec1a	0.37924	0.636115	0.534446	0.891365	0.886544	0.717361	0.534496	0.525199	0.427168
Gm42005	0.271622	0.501163	0.176575	0.364636	0.452707	0.514935	0.814131	0.310333	0.427152
Cfap300	0.044817	0.044697	0.044822	0.534786	0.298779	0.16578	0	0.213349	0.427141
Neu3	0.630544	0.54612	0.86295	0.907517	0.853375	0.690521	0.377299	0.616138	0.427001
Shc2	1.035702	1.228367	0.741873	1.294335	0.399888	0.996614	0.477363	0.479719	0.426861
Gm35200	0.385737	0.784808	0.86415	1.611019	0.734743	0.54221	0.845197	0.719828	0.426464
Gm8482	0.178832	0.089178	0.26828	0	0.085159	0	0.369665	0.085133	0.426108
LOC100041708	0.357665	0.356713	0.089427	0.889154	0.255477	0.578825	0.092416	0.766197	0.426108
Gm23823	0	0	0	0	0.85159	0	0	0	0.426108
Hmgb1-ps4	0.536065	0.178213	0.402096	0.266531	0.340362	0.041311	0.323196	0.297725	0.425765
Pnma1	0.423012	0.679705	0.399558	0.116845	0.470017	0.56505	0.364337	0.469874	0.425566
Gm11868	0.297574	0.32646	0.35713	0.147954	0.481791	0.522856	0.21529	0.198324	0.425422
9630013D21Rik	0.167318	0.055624	0.111558	0.110921	0.531173	0	0.288219	0.424809	0.425251
Gm14279	0.111545	0.111248	0	0	0	0	0.076858	0	0.425251
Gm5940	0.981468	0.682232	0.565153	1.03512	0.226603	0.330048	0.338132	0.538019	0.425194
Gm5564	0.350471	0.381314	0.414241	0.316825	0.364128	0.29464	0.362229	0.515691	0.425128
Gm3695	0.535203	0.578259	0.401449	0.487854	0.424768	0.536183	0.368772	0.254783	0.42508
Olfr558	0.465377	0.270747	0.155144	0.82913	0.424753	0.430366	0.040083	0.49847	0.425065
Rab40b	0.059451	0.237171	0.29729	0.266031	0.254792	0.412338	0.122891	0.226413	0.424966
Plekhh2	0.287725	0.379526	0.464126	0.350719	0.114914	0.163081	0.306971	0.450679	0.42461
Gm5106	0.049498	0.049366	0.099008	0.049221	0.047142	0.091549	0.306954	0.047127	0.424586
Gm32853	0.856583	0.512582	0.925217	0.579214	0.522112	0.50697	1.876881	0.358842	0.424528
9130002K18Rik	0.38515	0.428548	0.474288	0.385598	0.364318	0.36829	0.582214	0.374185	0.424517
LOC118567919	0.667928	0.4441	0.222669	0.442791	0.424085	0	2.071012	0.635933	0.424397
Gm5523	0.267171	0.48851	0.534405	0.221396	0.254451	0.205893	0.644315	0.339164	0.424397
LOC118568732	0.394428	0.44702	0.316775	0.362505	0.352882	0.281856	0.478693	0.32717	0.42434
Serpinb12	0.582117	1.126983	0.753415	0.408604	0.163059	0.348327	0.460083	0.456427	0.424266
2310068J16Rik	0.197816	0	0.197839	0.049177	0	0.182934	0.051113	0.188341	0.424207
Gm51903	0.471145	0.495996	0.680622	0.208225	0.299142	0.508317	0.811586	0.772548	0.424096
Gm40095	0.837196	0.547947	0.28782	0.364221	0.523252	0.338718	0.703043	0.672547	0.423896
Gzmm	0.666145	1.624019	2.220741	2.208045	0.634429	0.958269	1.14749	1.268471	0.423264
Gm39210	0.384576	0.678592	0.473379	0.970762	0.19722	0.711287	0.244602	0.816804	0.422925
Gm52109	0.255947	0.187195	0.238911	0.407221	0.276262	0.189353	0.352712	0.11372	0.422829
4930539J05Rik	0.110876	0.331743	0.110889	0.110255	0	0	0.114596	0	0.422699
Gm36277	0	0	0	0.66153	0	0	0.114596	0	0.422699
Lipc	0.613705	0.578068	0.443283	0.745883	0.324715	0.472946	0.704773	0.162308	0.422439
Gm6625	0.166148	0.11047	0	0.22029	0.474713	0.153649	0.114482	0.738216	0.422277
F830045P16Rik	0.413496	0.206198	0.413545	0.910471	0.309422	0.300448	0.67158	0.337448	0.422249
Armh2	0.063285	0.568053	0.126586	0.503448	0.180817	1.111964	0.261635	0.421778	0.422217
Zfp960	0.258195	0.147147	0.368892	0.293427	0.316159	0.37521	0.457469	0.702362	0.421855
Ppp1r14c	0.708191	0.618017	0.841074	0.484154	0.611241	1.187029	0.160114	0.695338	0.421855
Vegfc	0.44209	0.852432	0.265285	0.674074	0.392972	0.899428	0.548306	0.140304	0.421351
Galnt18	0.927649	0.616786	0.706862	1.60331	0.67313	1.24594	0.182623	0.546751	0.421015
Gm17914	0.110434	0	0.331342	0.109816	0.105177	0	0.456558	0	0.421015
6430550D23Rik	0.252278	0.251606	0.441538	1.31704	0.480533	0.408273	0.651854	0.900725	0.420776
Zfp882	0.480705	0.410936	0.47095	0.731652	0.569937	0.48083	0.740179	0.49504	0.420754
Gm3086	1.059326	0.792379	0.264862	1.141175	0.588519	0.163272	0.547433	0.336194	0.42068
Gm5616	0.662079	0.308148	0.220719	0.087783	0.420371	0.285726	0.319336	0	0.42068

Lrrc30	0.409718	0.691524	0.378246	0.250722	0.540293	1.16583	0.39089	0.21005	0.420536
B230303A05Rik	0.0802	0.039993	0.020052	0.119626	0.114572	0.129791	0	0.152716	0.420406
Jmjd7	0.200427	0.519722	0.20045	0.279026	0.152707	0.222418	0.372872	0.457982	0.420254
Lta	0.200427	0.119936	0.36081	0.039861	0.190884	0.333627	0.165721	0.419817	0.420254
Gm46110	0.337896	0.355379	0.339472	0.574261	0.282314	0.228676	0.557185	0.543983	0.420123
Cited4	0.396616	1.494337	0.528882	0.964074	0.461673	1.507864	0.455469	0.545447	0.420011
Gfra1	0.195721	0.512401	0.526062	0.851484	0.291254	0.452492	0.227574	0.524098	0.419715
Gm40768	0.659977	0.438813	0.440036	2.1876	0.209518	0.610326	0.454746	0.104727	0.419344
Asb12	0.263991	0.087763	0.088007	0.21876	0.293326	0.935833	0.227373	0.293236	0.419344
Gm22300	0	0.438813	0	1.75008	0	0	0	0	0.419344
Gm31645	0	0.125233	0.125582	0.187296	0.538151	0.290302	0.06489	0.059776	0.41887
Gm28800	0.585871	0.67561	0.366212	0.764649	0.122058	0.30476	0.737986	0.679827	0.41879
Gm14277	0.329335	0.328458	0.219582	0.545817	0.313655	0.812157	0.226923	0.313559	0.418514
Nup210l	0.582152	0.809036	0.754022	0.332151	0.363564	0.43245	0.80882	0.481575	0.418406
Gm47792	0.329009	0.4922	1.041984	0	0.417793	0.304257	0.056675	0.522082	0.4181
Jph1	0.438519	0.920741	0.830975	1.101632	0.703395	1.259274	0.644066	1.076745	0.417948
Ninl	0.563618	0.452817	0.375789	0.264662	0.536783	0.419868	0.663434	0.760211	0.417805
Gm51752	0.062571	0.624046	0.438049	0.062221	0.417145	0.462911	0.905386	0.119148	0.417451
Naip3-ps1	0.518898	0.649139	0.575953	0.888817	0.505619	0.299566	0.914511	0.44835	0.417371
Gm40309	0.287594	0.402826	0.351075	0.691828	0.398769	0.324626	0.815233	0.493275	0.417201
Gm31684	0.823592	0.385031	0.514805	0.7422	0.759868	0.952041	0.851224	0.588106	0.417008
Zfp449	1.076794	0.805445	0.891824	1.104224	0.432644	0.606807	0.712965	0.448531	0.416926
Ccdc39	0.231528	0.744048	0.617479	0.920924	0.759516	0.30927	0.50518	0.710298	0.416815
Xirp1	0.408681	0.625608	0.712899	1.096313	0.787498	0.694347	0.432215	0.298615	0.416684
F830016B08Rik	0.437182	0.637257	0.470866	1.170436	0.608537	0.637538	0.399713	0.736425	0.416673
Gm14438	0.710044	0.435787	0.655502	0.325877	0.208073	0.151529	0.225805	0.364017	0.416452
Gimap7	0.305805	0.17428	0.524298	0.738509	0.291245	1.696793	0.316065	0	0.41637
Hectd2	0.940767	1.977053	0.974479	0.400927	0.479987	0.590352	0.729247	0.895703	0.416295
Zfp558	0.481939	0.690943	0.225935	0.269572	0.315558	0.598886	0.513674	0.358479	0.416269
Mroh6	0.454858	0.453647	0.491303	0.307571	0.398545	0.874927	1.03426	0.294487	0.416179
Gm23297	0	0	0.436571	0	0	0.80736	3.609323	0	0.416043
Mir8096	0	0	0	0.434075	0	0	2.255827	0	0.416043
LOC118568548	0.65478	1.088395	0.873142	1.953337	0.207869	0.20184	1.579079	0	0.416043
Gm39443	0.277686	0.375857	0.297555	0.335302	0.132233	0.421878	0.328003	0.207731	0.415894
4930565N06Rik	1.03258	0.984061	0.367182	0.821437	0.699319	0.04244	1.612692	0.568023	0.415526
Bbs1	0.871637	0.987859	0.643897	0.719012	0.528269	0.503789	0.665423	0.38665	0.415374
Gucy1a1	0.117703	0.269996	0.176575	0.901236	0.571705	0.566009	0.340625	0.437052	0.415071
Gm5073	0.16935	0.277478	0.278251	0.060143	0.783396	0.615253	0.675124	0.299442	0.415044
Elfn1	0.870755	1.302655	0.290285	0.144313	0.138216	0.402623	0.749974	0.552696	0.414953
2410022M11Rik	0.49317	0.289327	0.406187	0.490407	1.326182	0.268275	0.569682	0.911471	0.414736
A330069E16Rik	0.124301	0.557863	0.310788	0.494417	0.236765	0.172424	0	0.17752	0.414643
F730016J06Rik	0.014989	0.104646	0.052469	0.201223	0.171308	0.256441	0.209145	0.335376	0.414299
Gm46878	0.434653	0.354679	0.316148	1.414534	1.053714	0.511578	0.490076	0.263348	0.414263
Trim15	0.276598	0.157635	0.079037	0.196463	0.451592	0.036541	0.245038	0.263348	0.414263
A830005F24Rik	0.361867	0.433085	0	0.215904	0.413566	0.066929	0	0.20672	0.41387
Gnai1	0.156261	0.259742	0.27783	0.017265	0.165357	0.016056	0.251228	0.132245	0.413697
4933433H22Rik	0.278895	0.309058	0.309919	0.801183	0.472207	0.257913	0.480419	0.383551	0.413484
Tldc2	0.674312	0.480369	0.240854	0.862116	1.009183	0.222708	1.095183	1.008874	0.413151
Gm25346	0	0.431957	0	0	0	0	1.342922	0.412363	0.412792
Gm8339	0.129933	0.172783	0.476476	0.301479	0.412489	0.240316	0.850517	0.123709	0.412792
Gm26012	0	0	0	0	0	0	0	0.412363	0.412792
Gm18646	0.708222	0.353168	0.393503	0.234752	0.074945	0.291085	0.28466	0.149844	0.412499
Rpl26-ps6	0	0	0	0	0	0	0.893536	0.411559	0.411988
Gm17541	0.432266	0.215557	0	0.107461	0.102921	0	0.111692	0.10289	0.411988

Gm17881	0.486299	0.215557	0.702513	0.537305	0.308764	0.449714	0.111692	0.051445	0.411988
Gm36112	0.791972	1.148893	0.576047	0.787536	1.097114	0.532648	1.041782	0.274195	0.41172
4930473H19Rik	0.143995	0	0.144012	0.572754	0.548557	0	0.297652	0	0.41172
Tnr	0.667928	0.778883	0.441912	0.347421	0.137012	0.17105	0.286756	0.225023	0.411338
Gm12816	0.603994	0.258166	0.17259	0.55771	0.123265	0.438865	0.535079	0.205379	0.411186
Gm20369	0.344871	0.171977	0.603595	0	0.164226	0.318926	0	0.410439	0.410866
Slc14a1	0.338318	0.414103	0.092279	0.70343	0.585837	0.782165	0.874172	0.278188	0.410388
Gm7899	0.699704	0.536801	0.511381	0.615501	0.179413	0.522629	0.361589	0.614941	0.410388
Ell3	0.496757	0.495434	0.33121	0.691564	0.567727	0.520637	0.308054	0.220715	0.410326
Gm13328	0.191331	0.190821	0.765412	0.237824	0.318887	0.486576	0.19775	0.227707	0.410299
Cited1	0.286872	0.071527	0	0.285265	0	0.26529	0.296497	0	0.410122
Vps37d	0.344069	0.557623	0.172054	0.256606	0.491531	0.238638	0.355612	0.081897	0.40991
Samd15	0.10749	0.107204	0.026876	0.240498	0.307117	0.074553	0.249967	0.025585	0.409791
Map2	0.041935	0.156838	0.167759	0.510826	0.319507	0.504142	0.238381	0.299447	0.40967
Gm25492	2.578515	0.857217	0.429802	0	1.637166	0.397422	0.444171	0	0.409592
Gm32242	0.143251	0.285739	0	0.569794	0	0.264948	0.148057	0.545555	0.409592
Gm15382	0.107438	0.107152	0	0.213673	0	0.099355	0	0	0.409592
Gm24771	0	0.428608	0	0	0	0	0	0.409166	0.409592
Bdh1	1.431583	1.873951	1.539131	0.800755	0.443113	0.397165	0.943253	1.124481	0.409328
Stkld1	0.457929	0.313988	0.400735	0.626126	0.572416	0.370544	0.35497	0.354245	0.409169
Gm33058	0.429198	0.214028	0.214624	0	0.408763	0.396909	0.147866	0	0.409064
Osr2	0.943992	0.85589	0.386223	0.51202	1.389437	0.476168	0.221742	0.817066	0.408958
4930480K23Rik	0.375306	0.802086	0.857942	0.373204	1.327624	0.148745	1.662418	0.612563	0.4088
Gm46770	0.10723	0	0.214486	0.10663	0	0.099163	0.221656	0	0.4088
Gm12607	0	0	0	0	0.20425	0	0	0.408375	0.4088
Gm15910	0.708593	0.669511	0.522182	0.445025	0.83469	0.569063	0.82873	0.976467	0.408766
Ccdc15	0.822601	0.846594	0.927722	0.844099	0.65842	0.712159	1.076316	0.616559	0.408687
Zkscan4	0.473637	0.539858	0.180454	0.695262	0.644409	0.438005	0.209797	0.128842	0.408426
Gm8784	0.152975	0.030514	0.397781	0.638895	0.495351	0.509279	0.316214	0.116518	0.408236
Sema6a	0.650662	0.542144	0.939039	1.269465	0.69028	0.845443	0.229839	0.682228	0.408193
Gm6155	0.427763	0	0	0	0	0.079116	0.088423	0.325818	0.407696
1700001J03Rik	0.285029	0.852809	0.142531	0.779438	0.067865	0.988446	1.031069	0.407063	0.407486
Mdrl	0.213634	0.106533	0.053415	0	0.101731	0.049391	0.331202	0	0.407225
LOC118568759	0.085421	0.51116	0.64073	0	0.5288	0.236983	0.088287	0.935283	0.407068
LOC118568531	0.433579	0.71849	0.433629	0.577078	0.381171	0.407129	1.116866	0.336599	0.406882
Gm29773	1.066117	0.921508	0.924075	1.978937	1.556882	1.971822	1.028426	1.218057	0.406442
LOC115485642	1.27934	0.850623	1.279489	0	0	0.394364	0.881508	0	0.406442
Gm23370	0	0.425311	2.132481	2.544347	0	0	0	0.406019	0.406442
Gm24613	0	0	0	0	0	0	0	0	0.406442
Gm26800	0	0	0.426496	0	0	0.098591	0	0.406019	0.406442
Acot6	0.882384	1.092457	0.791195	0.726158	0.289784	0.590897	0.408822	0.724238	0.405995
Gm12230	0.557002	0.882295	0.753678	0.488719	0.312048	0.787797	1.151378	0.53032	0.405961
Ank2	0.62871	0.758512	0.598358	0.867197	0.289731	0.384482	0.482112	0.482737	0.405921
Tfpi2	0.774275	0.810824	0.077436	0.500459	0.848022	0.644422	0.16005	0.073719	0.405874
Gm46733	0.744849	0.212247	0.638516	0.952299	0.608045	0.787215	0.109977	0.20262	0.405661
Gm46850	0	0	0	0	0	0	0	0	0.405661
Zfp974	0.464305	0.571119	0.634624	0.538655	0.552749	0.508094	0.479883	0.714671	0.405647
Gm35021	0	0	0	0.422973	0.540139	0.327797	0	0.134994	0.405402
Spata25	1.132834	0.917977	0.495672	1.196894	0.404587	1.178561	1.463551	0.067411	0.404884
H2ac12	0.212406	0.10592	0.106216	0	0.202294	0.196427	0.219533	0.303348	0.404884
I730030J21Rik	0.424487	0.423357	0.254722	0.759797	0.565988	0.235532	0.263237	1.0508	0.404574
Kcna5	0.66415	0.220794	0.276761	1.027335	0.562248	0.818913	0.400419	0.702595	0.404413
Olfr920	0.650549	0.789864	0.226304	0.75941	0.808142	0.49698	0.584674	0.377018	0.404368
Gm11951	0	0.422872	0.106012	0.316219	0.201907	0.098026	0.109556	0.302768	0.40411

C1qtnf2	0.612315	0.375806	0.28264	1.40512	0.852314	0.174231	0.048681	0.627829	0.404024
Foxd2	0.423353	1.034453	0.381062	0.715669	0.584635	0.783007	0.590701	0.403073	0.403493
LOC118568621	0.341842	0.557396	0.439562	0.517984	0.253218	0.301071	0.902905	0.578608	0.403378
Gm25788	0.423191	0.422065	0.423241	1.683283	0	0.391354	1.312168	0	0.403339
1700028K03Rik	0.423191	0.351721	1.481342	0.070137	0.067174	0.587031	0.874779	0.335766	0.403339
Gm29887	0.282128	0	0.564321	0	0.268695	0	0.583186	0.134307	0.403339
Gm22247	0	2.532388	0	2.524925	0	0.782708	0.437389	0.805839	0.403339
Gm25635	0.423191	0	0	0	0	0	0.437389	0	0.403339
Gm5767	0	0	0	0.631231	0.100761	0.391354	0.218695	0.10073	0.403339
Gm25683	0	0	0	1.683283	0	0	0	0	0.403339
Gm9506	0	0	0	0	0.705325	0.097839	0	0	0.403339
Kctd4	0.404523	0.440123	0.110337	0.018284	0.210144	0.085021	0.13303	0.437666	0.403071
Tmem98	0.230672	1.45703	0.884344	1.032207	1.391364	0.426636	0.516556	0.219622	0.403059
Atp8b5	0.070442	0.084306	0.098631	0.336229	0.670884	0.403885	0.640689	0.093895	0.402826
H2al1m	0.105596	0	0	0	0.100569	0	0.109139	0	0.402571
Gm7335	0.324769	0.518247	0.357287	0.12918	0.309306	0.510571	0.537064	0.401975	0.402394
2310034G01Rik	0.140706	0.491159	1.125777	0.419753	0.134007	0.325301	0.363566	0.535863	0.402315
Otos	0.084381	0	0.253171	0	0.080363	0	0	0	0.402111
Gm32039	0.110033	0.201189	0.073364	0.346485	0.227053	0.220468	0.170586	0.384126	0.402005
C920006O11Rik	0.459749	0.343894	0.536436	0.495271	0.43786	0.212581	0.277185	0.328295	0.401666
Smkr-ps	0.33701	1.008337	0.421311	0	1.043134	0.701226	0.435395	1.684547	0.4015
Ncmap	0.306288	0.305472	0.650937	0.380715	0.401094	0.920547	0.197852	0.874848	0.401389
5830411N06Rik	0.076545	0.019085	0	0.418642	0.200478	0.035393	0	0.109318	0.401251
Gm39302	0.252374	0.083901	0.168269	0	0	0.077796	0.086947	0.240284	0.400891
Gm30042	0.70825	0.706364	0.926281	0.464362	0.385445	0.424649	0.35394	0.422378	0.400564
Gm5434	0.611309	0.609681	0.687802	0.341935	0.691367	0.353324	0.592329	0.800286	0.400559
Zfp820	0.270064	0.254381	0.225079	0.507263	0.271495	0.31912	0.077535	0.185703	0.400392
Gm34947	0.629978	0	0	1.670531	0	0	1.085189	0	0.400283
Gm25526	1.259956	0	0.840068	0	0.399989	0	0.434076	0.799734	0.400283
Gm23803	0	1.256602	0	0.417633	0	0	0	0.399867	0.400283
Serpini1	0.279814	0.122093	0.314827	0.260856	0.216524	0.161727	0.271126	0.366314	0.400031
Gm30151	0	0.94067	0.41924	2.084216	0.099808	0.193828	0.433255	0.199556	0.399527
Gm32340	0.104798	0	0	0.416843	0	0	0	0.598667	0.399527
Rpl39l	0.139642	0.417812	0.837952	1.041452	0.864461	0.83939	0.721637	0.465337	0.399275
Brdt	0.384017	0.626718	0.418976	0.659586	0.232739	0.613401	0.685555	0.631528	0.399275
Gm5587	0.152198	0.113844	0.266377	0.4162	0.181189	0.281496	0.432586	0.181134	0.39891
LOC118568493	0.4184	0.208643	0	0	0	0	0	1.195075	0.398773
Gm12219	0.22814	0.075844	0.228167	0.151241	0.072426	0.351628	0.078598	0	0.398636
1700067K01Rik	0.501702	0.708852	0.87808	0.374169	0.915813	0.579948	1.080279	0.437864	0.398472
AA986860	0.313564	0.708852	0.43904	0.519679	0.159272	0.521953	0.885829	0.497572	0.398472
Gm5857	0.1858	0.301121	0.60392	0.023095	0.265431	0.214777	0.072013	0.044225	0.398439
Gm42135	0.696458	0.694604	0.696539	0.138511	0.26532	0	0.431895	0	0.398272
Synm	0.33167	0.575815	0.479135	1.160451	0.315879	0.692956	0.380886	0.304087	0.398066
Adamts12	1.031787	1.184212	1.097424	1.400581	0.530326	0.863291	0.837888	0.943379	0.398037
C920021L13Rik	0.417552	0.096102	0.353354	1.149821	0.244721	0.148515	0.298773	0.244646	0.397964
Gm30427	0.113875	0.170357	0.341664	0.415202	0.216906	0.052654	0.156927	0.12649	0.397954
Tes3-ps	0.298161	1.189469	0.636151	0.731344	0.92762	0.974246	1.15048	2.214252	0.397843
Gm36462	0.128403	0.160077	0.35315	0.351131	0.458587	0.56403	0.132711	0.427883	0.397734
Rpl36-ps3	0.694713	0.970008	0.555835	1.519806	0.793964	1.541876	0.861624	1.587443	0.397274
Gm29767	0	0.103929	0	0.828985	0.595473	0.481836	0.538515	0	0.397274
Gm7729	0.577146	0.447696	0.73755	0.637681	0.73289	0.474423	0.497091	0.732666	0.397274
Gm22711	0	0	0	0	0.793964	0.770938	0	0	0.397274
Gm24607	0.416828	0	0.833752	0	0	0	0	0	0.397274
Gm35803	0	0	0	0.621739	0.099245	0	0	0	0.397274

Gm52274	0.416828	1.662871	0.416876	0	0.396982	0	0	0	0.397274
Sorcs2	0.445675	0.280221	0.66859	0.635864	0.258364	0.403186	0.360491	0.507346	0.397065
Zfp72	0.757797	0.75578	0.876769	1.462778	0.905685	0.879419	1.290011	0.763938	0.396528
Gm6981	0.415975	0.528013	0.416023	0.150416	0.432185	0.629477	0.234508	0.432053	0.396461
9930111J21Rik2	0.138422	0.046018	0.415315	1.674702	0.307607	0.213347	4.363518	0.219652	0.395786
Gata3	0.138422	0.189824	0.432619	0.326911	0.527327	0.816054	0.447082	0.313005	0.395786
Gm5149	0	0	0	0	0	0.384025	0	1.186123	0.395786
Gm52502	0.482148	0.61889	0.508993	0.661459	0.314632	0.301379	0.941278	0.505807	0.395706
Gdf10	0.770591	0.650302	0.592831	0.397873	0.23993	0.58928	0.413538	0.197529	0.395468
Gm18743	0	0.103347	0.207269	0	0	0.095827	0.749694	0	0.395046
Grin2d	0.782763	0.780679	0.782854	0.778379	0.745495	0.638713	1.261127	0.526071	0.394964
LOC118568691	0.180111	0.323336	0.270197	0.429844	0.222996	0.216529	0.521229	0.257225	0.394821
Gm18096	0.138077	0.13771	0.41428	1.304387	0	0.063845	0.14271	0.262926	0.3948
D7Ertd128e	0.225664	0.300084	0.45138	0.3366	0.788039	0.591279	0.388725	0.250663	0.394309
Gm34633	0.206858	1.237846	0.310324	1.234198	0.295515	1.052129	1.38969	0.590849	0.394309
Gm50432	0.24823	0.330092	0	0.16456	0.394019	0.306074	0.256558	0.236339	0.394309
Gm22672	0	0	0	0	0	0	0	0	0.394309
9430091E24Rik	0.587452	0.47739	0.2176	0.367805	0.745977	0.663981	0.427261	0.352159	0.393999
Gm35343	0.860839	0.446445	1.102003	0.821777	0.491912	0.700548	1.637086	0.95074	0.393819
Cysltr2	0.444775	0.506961	1.112067	0.726609	0.574885	0.470073	0.788053	0.635204	0.393631
Mnd1	2.063451	1.852162	2.132481	1.436325	1.113618	0.508857	0.710893	0.261948	0.393331
Stk36	0.369225	0.433226	0.412711	0.572333	0.537811	0.361533	0.819345	0.289502	0.393305
Gm51987	0.412639	0.720196	0.371418	0.51291	0.275095	0.400676	1.066208	0.707171	0.393282
Dbndd1	0.618728	0.575942	0.288773	0.533228	1.060686	0.152582	0.554222	0.746181	0.393135
Mttp	0.445087	0.213731	0.445139	0.819618	0.251198	0.259157	1.209681	0.219731	0.392785
Atxn7l1os2	0.274582	0.301236	0.082384	0.43687	0.156905	0.279317	0.312173	0.549	0.392551
Alpk3	0.295216	0.553172	0.250516	0.071167	0.460081	0.19855	0.221906	0.332179	0.392209
LOC118568707	0.308561	0.820638	1.131521	0	0.195913	0.66581	0.318913	0.29378	0.392114
Rpl23a-ps2	0.205707	1.641276	0.102866	0	0.391826	0.570694	0	0.88134	0.392114
LOC68395	0.180955	0.09844	0.246786	0.278091	0.094003	0.395535	0.272038	0.219274	0.391969
Gm6158	0.51395	0.307549	0.582544	0.272571	0.522112	0.348542	0.354129	0.293598	0.391872
Misp	0.296949	0.045563	0.571122	0.477	0.304565	0.16899	0.306911	0.413213	0.391872
Gm38530	0.221373	0.03154	0.094885	0.031448	0.150595	0.233964	0.130743	0.150549	0.391835
Sox6	0.602588	0.491714	0.93138	0.381317	0.495639	0.9372	0.990825	0.365096	0.391582
Gpr27	0.020533	0.020478	0.020535	0.367517	0.23466	0.588626	0.233436	0.039098	0.391388
4430402I18Rik	0.157763	0.377624	0.378676	0.627519	0.450756	0.350147	0.065223	0.210289	0.390943
Zfp442	0.456567	0.740911	0.650103	0.931104	0.655929	0.536719	1.007754	0.383122	0.390896
Oxt	0.204947	0	0	0	0.097594	0	0	0	0.390665
Gm42144	0.81957	0	0.058548	0.058213	0.111507	0.32482	0.12101	0.055737	0.390562
Slc1a4	0.409502	0.718241	0.522528	0.463375	0.34966	0.443987	0.233512	0.389886	0.390292
LOC118567347	0	0.08167	0.081897	0	0	0	0	0	0.390232
Olfr1417	0.233916	0	0.116972	0.639665	0	0.108159	0.060441	0.055678	0.39015
Comp	0.79438	1.104369	1.17967	1.148989	0.848262	0.4007	0.696632	0.710489	0.390029
Gm14026	0.649947	0.456152	0.577798	0.52662	0.733632	0.467484	0.572234	0.779246	0.390029
Gm32178	0.334412	0.37058	0.371612	0.88677	0.106163	0.137446	1.305719	0.212262	0.389552
Psmb6-ps2	0.068106	0	0	0.135448	0.259453	0.062982	0.211172	0	0.389465
Six5	0.427493	0.348836	0.971688	1.487844	0.166557	0.790664	0.682836	0.296011	0.388919
Lrrc24	1.019082	1.045408	0.32032	0.347442	0.693259	0.834711	0.842618	0.388106	0.38851
Gm44148	0	0	0.135893	0	0.258817	0.376966	0	0	0.38851
Gm52742	0.050954	0.203274	0.10192	0.658693	0.388225	0.094242	0.105327	0.14554	0.38851
Olfr1372-ps1	0.188138	0.187637	0.25088	0.654795	0.298635	0.231979	0.064817	0.20898	0.38851
Gm18712	0	0	0	0	0	0	0	0	0.38851
Gm23346	0	0	0	0	2.32935	0	0	0	0.38851
LOC118568748	0.256863	0.167841	0.132875	0.237808	0.244633	0.073719	0.228862	0.10963	0.388324

Sult5a1	0.407134	1.556525	0.407181	1.551938	0.064625	0.376505	0.701322	0.904473	0.388035
P4htm	0.677727	0.743515	0.135561	1.617433	0.580914	0.689415	1.050697	1.161472	0.38756
Gm42326	0.72232	0.450248	0.225751	1.122303	0.773921	0.793225	0.839873	0.472807	0.387245
Lmntd2	0.135413	0.081031	0.027086	0.053862	0.206345	0.200361	0.139956	0.154712	0.387182
Vangl2	0.314816	0.344363	0.142191	0.414037	0.444905	0.328697	0.314882	0.386756	0.387158
Sfrp1	0.48185	0.417334	0.596041	1.588762	0.265684	0.257979	0.380065	0.651934	0.386733
Cyp3a60-ps	0	0	0.067615	0	0	0	0.559005	0.064369	0.386615
Gm41358	0.247894	0.112379	0.202846	0.201686	0.107314	0.291766	0.37267	0.064369	0.386615
Krba1	0.578199	0.714369	0.742252	1.012616	0.846553	0.542681	0.383533	0.443688	0.386575
Fat1	0.401753	0.343983	0.485192	0.776391	0.270725	0.375033	0.364307	0.389725	0.386518
Smarca1	0.041935	0.041823	0.209699	0.1251	0.09319	0.025853	0.115578	0.066544	0.386355
Dlg5	0.311079	0.526486	0.848496	0.665542	0.368091	0.749702	0.428688	0.520555	0.38633
Gm38972	0.09007	0.17966	0.09008	0	0.171563	0.832939	0.093092	0	0.386301
Gm51817	0.380638	0.195935	0.245601	0.622703	0.198799	0.158969	0.824888	0.128595	0.386187
2310002L09Rik	0.289343	0.288572	0.463002	0.978255	0.606247	0.588665	0.11962	0.385676	0.386077
Tmem44	0.472388	0.740347	0.269967	2.013178	1.413961	1.716194	0.453362	0.514011	0.385909
A030001D20Rik	0.877291	0.134609	0.404951	0.369083	0.128542	0.218425	0.383614	0.449759	0.385909
Gm36809	0	0	0.809409	0.804781	0.770783	1.122643	0.139411	0	0.385674
Snord94	4.855889	0.40358	0.809409	0	0	1.496858	0.418234	3.467461	0.385674
Clstn3	0.239538	0.268763	0.299457	0.16376	0.356458	0.179983	0.216628	0.256572	0.385258
Tigd5	0.392604	0.218813	0.219422	0.734881	0.307928	0.363068	0.274496	0.35181	0.385192
LOC118568315	0.854917	0.599659	0.544955	1.008943	0.429475	0.556027	0.582593	0.366731	0.385021
LOC115489722	0.40392	0.402845	0.504959	0.90373	0.577034	0.466916	1.565519	0.288429	0.384972
Gm18072	0.706861	0.503556	0.100992	0	0.096172	0	1.252415	0.961429	0.384972
Diras2	0.533935	0.792276	0.833556	0.90649	0.880597	0.746671	0.47109	0.557954	0.384768
Gm19144	0.235477	1.140701	1.278453	1.103889	0.897064	0.591068	0.764901	0.608536	0.384738
Gm32515	0.370036	0.503251	0.201861	0.033451	0.64076	0.373306	0.069536	0.416367	0.384738
LOC118568381	0.30272	0.623955	0.464224	0.612082	0.557393	0.335935	0.615323	0.432328	0.384692
9530082P21Rik	0.192859	0.157373	0.350693	0.383556	0.450842	0.145922	0.344295	0.400626	0.384333
Gm39931	0.403186	0.402113	1.008082	0.701623	1.15197	0	2.500277	0.095968	0.384272
Gm52024	0.201593	0.301584	0.604849	0.100232	0.479987	0.466067	0.625069	0.479841	0.384272
Marco	0.28799	0.574446	0.460837	0.028638	0.274278	0.026632	0.148826	0.191936	0.384272
Rps8-ps5	0.483471	0.723277	0.644704	1.121781	0.690679	0.968715	0.333128	0.843906	0.383993
Echdc2	0.089488	0.044625	0.134248	0.711897	0.426139	0.537914	0.092491	0.51121	0.383807
Mkln1os	0.460067	0.458842	0.23006	0.228745	0.328622	0	0.237751	0	0.383674
4930594C11Rik	0.160982	0.120415	0.04025	0.82041	0.114988	0.223306	0.353563	0.459811	0.383575
Gm6394	0.201227	0.301037	0.503126	0.90045	0.191646	0.465221	0.311967	0.383176	0.383575
Cacng7	0.710137	1.298453	0.994308	1.341703	0.946858	1.247754	0.31805	0.788807	0.383534
Gm15530	0.13407	0.80228	0.402258	0.399958	0.383061	0.123984	0.138568	0.63824	0.383343
Flacc1	0.050261	0.300764	0.452403	0.499796	0	0.2324	0.259737	0.430682	0.383227
Iglon5	0.402016	0.721703	0.482476	0.279835	0.229726	0.316006	0.436279	0.440173	0.383157
Efcab11	0.455508	1.175824	1.1791	0.186512	0.357265	0.693807	0.886194	0.255111	0.383065
Efhc1	0.401725	0.400656	0.477104	0.474376	0.980409	0.534035	0.726605	0.525912	0.38288
Rbm11	0	0.701147	0	0.79895	0.669548	0.185751	0	0.191241	0.38288
LOC118568281	0.648579	0.24642	0.154442	0.337829	0.352971	0.485541	0.510734	0.147026	0.382666
Gm32059	0.234598	0.374357	0.271122	0.456199	0.30287	0.236233	0.366397	0.506283	0.382592
Gm39212	0.114541	0.742537	0.458219	0.683399	0.54544	0	1.006267	0.545273	0.382089
Gm12389	0.267171	0.53292	0.534405	0.398512	0.254451	0.123536	0.138067	0.508747	0.381957
Kcnip4	0.200318	0.099892	0.100171	0.697186	0.429257	0.277872	0.207039	0.524487	0.381842
Gm6813	0	0.798418	0.300241	0.298525	0.762435	0.09254	1.344538	1.238578	0.381497
Syt15	1.292837	0.368398	0.307854	1.867173	0.703591	1.679498	0.922622	0.967141	0.381392
D730005E14Rik	0.338131	0.306573	0.537998	0.183402	0.453772	0.12792	0.683065	0.278034	0.380863
Gm47922	0.079882	0.079669	0.279619	0.079434	0.190197	0.295489	0.288967	0	0.380673
Cxcr5	0.52538	0.54494	0.315264	0	0.200146	0.116605	0.173762	0.220094	0.380557

Pgam1-ps1	0.266049	0.398012	0.332601	0.198419	0.285055	0.461314	0.412463	0.411621	0.380353
Gm7756	0.066472	0.464069	0.06648	0.594901	0.063308	0.36883	0.618324	0.126577	0.380125
Gm36338	0.365599	0.331478	0	0.561851	0.506461	0.307358	0.583972	0	0.380125
Gm32422	0	0.056708	0.170598	0.565411	0	0.26291	0.058767	0.108272	0.379345
4930412M03Rik	1.093845	0.595054	0.298356	0.395534	0.236765	0.505777	0.719437	0.426047	0.379102
Brd3os	0.530191	0.991461	1.126786	1.186247	0.788981	1.103183	0.582227	0.662541	0.378989
Gm49400	0.198703	0.066058	0.132484	0.263453	0.063081	0.551262	0.068456	0.315307	0.378763
Gm15738	0.226973	0.198072	0.170249	0.141063	0.270208	0.052474	0.263911	0.054025	0.378569
Nfyc-ps	0.368077	0.423574	0.622972	0.16893	0.107862	0.104734	0.175581	0.323488	0.377795
Chodl	0.197993	0.526576	0.066005	0.809412	0.69141	0.895145	1.250552	0.460799	0.37741
Gm17930	0.079197	0.078986	0.396032	0.551275	0.150853	0.292956	0.245563	0.075404	0.37741
Zfp9	0.395885	0.451236	0.466633	0.32337	0.511693	0.523003	0.818334	0.390383	0.377314
Gm11998	0.461435	0.065744	0.197781	0	0.439465	0.06096	0	0	0.376961
Gm13216	0.236915	0.630091	0.315923	0.314117	0.150423	0.146061	0.163242	0.300755	0.376335
Gm5454	0.5574	1.575095	0.90588	0.415708	0.464504	0.493988	0.576101	0.685487	0.376303
Epdr1	0.255475	0.486426	0.255505	0.762132	0.420265	0.343644	0.168029	0.685487	0.376303
Hs3st1	0.460613	0.39376	0.427762	1.308665	1.629393	1.734268	0.442062	1.440946	0.37629
A830049F12Rik	0.112679	0.196663	0.253557	0.196084	0.134143	0.234455	0.349378	0.348665	0.375876
Ceacam2	0.222849	0.341932	0.360029	0.102277	0.195913	0.206084	0.212609	0.14689	0.375776
Gm33922	0.344642	0.049103	0.04924	0.293752	0.093781	0.136592	1.068613	0.140628	0.375399
Enpp3	0.472651	0.235697	0.45301	0.822507	0.600197	0.32782	0.142482	0.225005	0.375399
Spata48	0.551231	1.099526	0.86632	0.861368	0.524986	0.218469	0.732503	0.2999	0.375266
LOC118568718	0	0.31415	0.078756	1.096286	0.37499	0.801053	0.162778	0.14995	0.375266
Gm15446	0.285947	0.998152	0.321728	0.604235	0.544666	0.363598	0.591082	0.850781	0.374733
Cnn1	0.039318	0.548983	0.039322	0.31278	0.299567	0.327239	0	0.074869	0.374733
Rph3al	0.214322	0.285002	0.071449	0.568325	0.238138	0.693695	0.701458	0.136037	0.374492
Gm52051	0.462176	0.414851	0.392896	1.171948	0.220086	0.491517	0.764292	0.506043	0.374421
AA543186	0	0.391206	0.326913	0.390053	0.155656	0.151142	0.574331	0.311217	0.37385
Gm22146	0	0.180491	0	0.269939	0.028726	0.111572	0.031174	0.086152	0.373714
B930018H19Rik	0	0.026068	0.052281	0.051983	0.024893	0.024171	0.027015	0.024886	0.373673
Gm6051	0.587683	0.097686	0	0	0.74627	0.181157	0	0	0.373409
Fam189a2	0.717997	0.672686	0.23936	1.168323	0.994636	1.066394	0.044975	0.704319	0.373263
LOC118568000	0.567291	0.487742	0.352153	0.194522	0.316717	0.198991	0.242617	0.260746	0.372882
Gm13608	0	0.070931	0.355641	0.353608	0.237069	0.06577	0.47779	0.067713	0.37281
Arhgef33	0.408008	0.559517	0.306041	0.355007	0.501919	0.676019	0.614974	0.453207	0.372665
Zbtb7c	0.239392	0.301584	0.264622	0.325754	0.059998	0.186427	0.156267	0.11996	0.372263
Gm31253	0.583758	0.48769	0.424601	0.659646	0.613727	0.290953	0.854083	0.313988	0.37212
Gm11713	0	0	0.078091	0	0.074364	0.072208	0	0	0.372094
Gm52570	0.156164	0	0	0.465866	0.818007	0	0	0.074342	0.372094
Gm12958	0.638851	0.495561	0.177479	0.141172	1.352077	0.361038	0.623602	0.20275	0.372094
Gm36003	0.170729	0.145949	0.097571	0.315292	0.348429	0.112775	0.151249	0.023221	0.371931
Unc45b	0.452619	0.638206	0.374625	0.667366	0.445934	1.587672	0.27423	0.386358	0.371885
Afap1l2	0.423838	0.642519	0.661265	1.753291	0.88805	1.066111	0.420536	1.113759	0.371639
Gm41292	0.350874	0.388822	0.506877	0.581514	0.334168	0.180265	0.725291	0.111355	0.371571
Gm5050	0.389495	0.194229	0.129847	0.064552	0.12365	0.660353	0.536749	1.112511	0.371223
Rpl31-ps4	0.129832	0.129486	0	0	0	0	0.134187	0.123612	0.371223
Gm10865	0.077862	0.465931	0.155743	0.232279	0	0.576038	0.080475	0.074133	0.371049
Tigd3	0.596945	0.647126	0.415315	0.154853	0.469651	0.168011	0.751097	0.247109	0.371049
Kif26b	0.164682	0.328488	0.366835	0.052105	0.08555	0.02769	0.100577	0.192429	0.370989
LOC118567837	0.663792	1.118593	1.053034	0.477984	0.370593	0.80436	0.496804	0.479444	0.370865
Morc4	0.450549	0.796575	0.286747	1.181159	0.487612	0.340899	0.550332	0.506961	0.370857
Gm20302	0	0.582005	0.583626	0.19343	0.370517	0.719542	1.206274	0.555605	0.370789
Gm867	0	0	0	0	0	0	0	0	0.370789
Rragb	0.874954	0.605989	0.656292	0.555867	0.393501	0.696751	0.527513	0.416521	0.370626

LOC118567342	0.478715	0.308932	0.408364	0.669248	0.38083	0.291662	0.724702	0.332454	0.370374
Gm19293	0.3494	0.309751	0.11648	0.965118	0.184869	0	0.481496	0.184813	0.37001
Sox8	0.036971	0.129054	0.31429	0	0.105632	0.444465	0.324797	0.1584	0.369985
LOC115488213	1.163036	1.546587	2.326343	0	0.738442	2.151079	2.003427	0.738216	0.369492
Gm25776	0.775358	0.773293	0	1.542029	0	0	1.202056	0	0.369492
Gm17786	0.129226	0.644411	0	0.257005	0	0.358513	0.133562	0.246072	0.369492
Gm9199	0.043042	0.257564	0.215235	0.256805	0.081985	0.119412	0.355888	0.286861	0.369205
Gm51649	0	0	0	0	0	0	0.200168	0	0.36917
Gm8930	0.387227	0.064366	0.580909	0.128353	0.307326	0.835557	0.200109	0.122893	0.369062
Aipl1	0.527982	0.456366	0.316826	0.315014	0.167615	0.651014	0.363797	0.167563	0.369023
Gm18917	0.193501	0.385972	0.677333	0.769669	0.092144	0.089472	0.799972	0.276348	0.368847
Vipr2	0.548253	0.707615	0.451555	0.513113	0.245718	0.238592	0.199993	0.153527	0.368847
Pcdh12	0.337371	0.237511	0.109162	1.213654	0.491414	0.45881	0.34869	0.160605	0.368831
Gm13070	0.096582	0.096325	0.193186	0.192082	0.091984	0.178632	1.197868	0	0.368205
Gpr21	0.28962	0.072212	0.072413	0.575995	0.114929	0.334789	0.673507	0.160852	0.368045
Gm52734	0.220649	0.385108	0.055169	0	0.420287	0.204049	0.399091	0	0.368022
Zc2hc1c	0.295125	0.203773	0.068114	0.541794	0.713495	0.146958	0.39888	0.172916	0.367828
Gm33052	0.836073	0.384852	0.128642	1.279061	0.306256	1.189498	0.66471	0.551093	0.367778
Gm7418	0.128627	0.064142	0.257283	0.127906	0.061251	0.475799	0.066471	0.183698	0.367778
Gm1070	0.308632	0.327049	0.482294	0.421992	0.514391	0.285413	0.498417	0.330579	0.367692
Dbil5	0.096414	0.096157	0.096425	0.191748	0.642766	0	0	0.183591	0.367565
Gm14038	0.105129	0.838793	0.595801	0.243927	0.533993	0.518507	0.253531	0.53383	0.36739
Cd163l1	0.497562	0.080038	0.321045	0.01596	0.12229	0.341387	0.165889	0.229223	0.367139
Slc38a4	0.199674	0.369836	0.427923	0.340381	0.217334	0.052758	0.044223	0.52959	0.367021
Myorg	0.398357	0.476756	0.491364	0.40933	0.632319	0.33155	0.164689	0.493058	0.367014
Gm5845	0.834138	0.831917	0.449203	0.38283	0.672204	0.712047	0.397903	0.671999	0.366926
Pomc	0.219992	0.603368	0.055004	0.38283	0.471416	0.050861	0.284216	0.366545	0.366926
Gm6439	0.192493	0.054852	0.110009	0.27345	0.104759	0.228872	0.198951	0.026182	0.366926
Gm25848	0	0.383962	0.385031	0	0	1.424094	0	0.73309	0.366926
LOC115487655	0	0	0	0	0	0	0	0	0.366926
Gm49890	0	0.054797	0.05495	0	0	0.25405	0	0	0.366563
Gm17415	0.769083	0.639196	0.640977	1.657012	0.610388	0.651955	0.861126	0.244081	0.366502
Gm46040	0.12818	0.127839	0.256391	0	0.427272	0.474149	0.794886	0.12204	0.366502
Pwwp3b	0.066282	0.11899	0.344705	0.303188	0.101002	0.13485	0.082207	0.227184	0.3664
Gm15616	0.21934	0.328133	0.329048	0.436222	0.313345	0.202838	0.170024	0.783123	0.365838
Gm8663	0.047915	0.047788	0.479209	0.285882	0.136902	0.088621	0.148569	0.319341	0.365341
Gm4734	0.076642	0.331233	0.255505	0.050809	0.194649	0.283506	0.528092	0.121619	0.365235
Gm6266	0.447081	0.636987	0.511009	0.190533	0.243312	0.295319	0.330058	0.486474	0.365235
Gm8401	0.063869	0	0.031938	0	0	0.14766	0.132023	0.091214	0.365235
Gm10804	0.063869	0	0	0.444577	0.182484	0.059064	0.066012	0	0.365235
Tril	0.279363	0.474685	0.248352	1.286103	0.729208	0.334893	0.363593	0.295535	0.364872
Tmem252	0.202657	0.202118	0.630563	0.761306	1.265279	1.041174	0.325821	0.793238	0.36484
Abcb9	0.82258	0.457892	0.899204	1.084289	1.038482	0.725315	0.988578	0.892457	0.364647
Dmrt2	0.058851	0.440211	0.559154	0.058522	0.168148	0.163272	0	0.420243	0.364589
Gm5844	0.46299	0.240917	0.362382	0.22019	0.536805	0.372312	0.124832	0.574972	0.364528
Gm52204	0	0.127105	0.127459	0	0	0	0.526878	0	0.364396
LOC102636478	0.191166	0.190657	0.191188	0	0.364128	0.353568	0.395159	0.728034	0.364396
Gm19587	0.637219	0.190657	0.223053	0.348507	0.333784	0.176784	0.197579	0.303348	0.364396
3425401B19Rik	0.392592	0.2679	0.40297	0.462307	0.629726	0.305732	0.202881	0.344276	0.364328
Tbx3	0.692826	0.178702	0.943787	0.700824	0.477815	0.419772	0.283959	0.693758	0.364317
Gm34305	0.15288	0.285887	0.17201	0.399062	0.273002	0.176723	0.572783	0.363892	0.36427
Efhd1	0.636853	0.6034	0.509542	1.709872	1.061431	1.590143	0.394932	0.485077	0.364187
Efna4	0	0.034643	0.451616	0.069082	0.231573	0.12849	0.107703	0.297646	0.364168
Hs6st2	0.264481	0.234468	0.264511	0.438332	0.167926	0.217407	0.015186	0.209843	0.364106

Gm6440	0.127152	0.126813	0.254333	0.126439	0.605489	0.117586	0.394252	0.121061	0.36356
Mylk3	0.211838	0.08451	0.084745	0.568759	0.342979	0.705245	0.547363	0.44372	0.363421
Fam169b	0.134341	0.245636	0.268713	0	0.277214	0.269174	0.300838	0.213176	0.362777
F2rl1	0.080113	0.099874	0.040061	0.258908	0.228896	0.2593	0.207001	0.114413	0.362684
LOC118567764	0.103778	0.296704	0.276773	0.233912	0.230619	0.281513	0.607804	0.177852	0.362668
Gm35522	0.532489	0.488923	0.507192	0.588341	0.346141	0.203225	0.288281	0.289704	0.362507
A930024E05Rik	0.087718	0.583233	0.760315	0.639665	0.362016	0.784153	0.604409	0.97436	0.362282
Gm35833	0	0	0	0	0.045243	0	0	0	0.36221
Gm24336	0	0.378702	0	0	0.361634	0.351146	3.924521	1.084571	0.3619
Gm16148	0.189856	0.126234	0.253171	0.440517	0.120545	0	0.327043	0.120508	0.3619
Rpl17-ps7	0	0.302961	0.835465	0.075517	0.578615	0.070229	0	0	0.3619
Gm14830	0.270958	0	0.379386	0.161664	0.051612	0	0.168029	0.206383	0.361546
Gng7	0.362771	0.756503	0.247373	0.262356	0.376909	0.320231	0.340857	0.204096	0.361469
Perp	0.058325	0.17451	0.787481	0.550985	0.388838	0.296655	0.512397	0.277656	0.361329
Sema6c	0.722432	0.754819	0.636502	0.88943	0.786327	0.906683	0.586669	0.671449	0.360665
Gm40310	0.407419	0.232191	0.116419	0.636643	0.110863	0.215296	0.812098	0.83122	0.36057
Rtkn2	0.182132	0.391239	0.294247	0.292565	0.17346	0.310947	0.275123	0.160068	0.360528
Gm52609	0	0.075328	0	0	0	0.069847	0	0.719107	0.359928
Aqp11	0.796765	0.418234	0.251639	0.708902	0.399384	0.814384	0.563445	0.039926	0.35971
Hebp2	0.290252	0.434218	0.725713	0.375214	0.387005	0.724722	0.629978	0.359252	0.359626
LOC118568295	0.347054	0.556817	0.498005	0.390124	0.359272	0.460485	1.029305	0.100565	0.359536
Exoc3l2	1.173588	0.6131	0.475079	0.944726	0.638693	1.111138	0.12996	1.130673	0.359529
Cideb	0.047141	0.282094	0	0	0.224484	0.043595	0.097446	0.314181	0.359438
Gm1647	0	0.075225	0	0	0.071835	0	0	0.071813	0.359438
Gm22738	0	0	2.263041	0	1.077523	0	0	0	0.359438
Gm41519	0	0	0.094293	0	0.179587	0	0	0	0.359438
Tmem233	0.243927	0.199046	0.487912	0.308714	0.654703	0.492167	0.710495	0.253356	0.359294
Awat2	0.273937	0.136604	0.273969	0	0.032612	0.15833	0.106173	0.097806	0.358994
Gm34771	0.376617	0.863914	0.075332	0.524311	0.430423	0.20897	0.66173	0.286861	0.35895
B230216N24Rik	1.129468	0.516295	0.376533	0.748761	0.537847	0.348166	0.2432	0.537682	0.358828
Cdc34b	0.188245	0.046936	0.2824	0.046798	0.134462	0.304645	0.04864	0.448068	0.358828
Atf1-ps	0.860339	0.26814	1.021771	1.176339	0.409688	0.497259	1.000353	0.66554	0.358741
Arhgdig	0.376033	0.160728	0.429802	0.427345	0.665099	0.347744	0.277607	0.204583	0.358393
Itgb8	0.268595	0.857217	0.304443	0.124642	0.477507	0.149033	0.240592	0.136389	0.358393
Zscan18	0.823426	0.85694	0.751911	1.014617	0.647836	0.463509	0.777048	0.545379	0.358277
Bcl6b	0.483003	0.535242	0.447277	1.476471	1.005201	0.711358	0.443741	0.255481	0.358046
Tnfrsf13c	0.115556	0.144061	0.317816	0.660726	0.30265	0.480882	0.418016	0.05501	0.35794
Ldhc	0.234708	0.046817	0.046947	0.046679	0	0.217051	0.388132	0.178772	0.357916
Gm30605	0.311148	0.759749	0.665291	0.554796	0.756163	0.555636	0.698621	0.52098	0.357908
Gm39853	0.187714	0.686451	0.312892	0.373324	1.13225	0.520775	0.388023	0.297869	0.357815
Tescl	0.500569	0.062405	0.500628	1.057752	0.059592	0.173592	0.06467	0.119148	0.357815
Gm32643	0.351329	0.4579	0.271513	0.563741	0.220533	0.177217	0.746863	0.277482	0.357678
Gm18078	0.093804	0.280662	0.281444	0.652949	0.357351	0.433734	0.484755	0.08931	0.357614
Gm5115	0.034084	0.339935	0.238618	0.30504	0.211	0.283681	0.528417	0.340741	0.357339
Gm41667	0	0.186792	0.187313	0.186242	0.178374	0	0	0	0.357009
Gm46736	0	0.186792	0	0	0	0	0	0	0.357009
LOC100041877	0.936454	0.373584	0.374625	0	1.426989	0	0	0.356638	0.357009
Gm13140	0.280699	0.077764	0.171558	0.139563	0.326742	0.259582	0.161176	0.178169	0.356708
6330418K02Rik	0.822967	1.716168	1.122358	0.223188	1.496317	1.106988	2.087782	0.712314	0.356528
Crybb3	0.374076	0.447696	0.374119	1.413526	0.427519	1.660482	0.077325	0.284926	0.356528
Defb48	0	0	0	0	0	0	0	0	0.356207
Gm17399	0	0.27948	0.233549	0	0.044481	0.172763	0.193085	0	0.356107
Gm33470	1.270144	0.81967	1.046123	0.445775	0.284629	0.138187	1.003873	0.284542	0.356047
Gm13135	0.388201	0.387167	0.283718	0.341485	0.540357	0.414226	0.246908	0.341174	0.355759

Gm40616	0.390534	0.283269	0.284058	0.282434	0.253596	0.180577	0.311901	0.338024	0.355295
Fsd2	0.46571	1.133306	0.68933	0.685389	0.620951	0.84412	0.558347	0.141888	0.35509
Dnah8	0.197681	0.15924	0.364992	0.453631	0.108617	0.196871	0.220029	0.104964	0.355075
Adam23	0.319068	0.415452	0.381153	0.731508	0.818779	0.516362	0.632064	0.371292	0.354783
Gm23435	0	0	0	1.10995	0.354353	0.688153	0	0	0.354613
LOC115488470	0.412837	0.343869	0.276769	0.676688	0.401822	0.331434	0.675197	0.371464	0.354555
Apol7d	0.278817	0.092691	0.139425	0	0.088514	0.386761	0.144086	0.13273	0.354316
1500009L16Rik	0	0.324148	0.278616	0.092341	0.08844	0.386438	0.047988	0.044206	0.354019
Gm39430	0.092861	0.092614	0.464359	0.092341	0	0	0	0.795716	0.354019
Aloxe3	0.131059	0.08714	0.393223	0.282371	0.083213	0.0808	0.180608	0.187172	0.353915
Ttc9	0.243658	0.231438	0.556998	0.288445	0.32046	0.364816	0.40773	0.47502	0.353871
Gm6314	0	0	0	0	0.117854	0.114436	0.255795	0	0.353822
Gm13034	0.510309	0.462682	0.78875	0.369055	0.618561	0.171606	0.191792	0.309186	0.353723
Tex26	0	0.092459	0.092717	0	0	0	0	0	0.353427
Qprt	0.324199	0.415718	0.602154	0.046055	0.441091	0.556789	0.047868	0.529148	0.353132
9230116N13Rik	0.317242	0.395497	0.502358	0.736085	0.730167	0.464511	0.901685	0.377557	0.352753
Dnah12	0.108844	0.130266	0.145143	0.173175	0.221146	0.107366	0.247491	0.297074	0.35271
Chd5	0.646885	1.078344	1.192256	0.358389	0.510472	0.555489	0.936023	0.580704	0.352308
Gm42270	0	0.737206	0	0.367517	0	0	0.381987	0	0.352249
LOC118568163	0	0.184302	0	0.183758	0.879977	0	0.381987	0.175942	0.352249
1110002L01Rik	0.319189	0.519395	0.403233	0.200464	0.399989	0.326247	0.295172	0.303899	0.352249
Sncaip	0.305134	0.192203	0.160616	0.60685	0.260016	0.727724	0.199182	0.183485	0.352045
Sncb	0	0	0.246238	0	0	0	0	0.195346	0.351989
Sit1	0	0	0.046165	0.045901	0.087924	0.597621	0.190834	0	0.351956
Akap3	0.33407	0.333181	0.404448	0.104905	0.066982	0.04878	0.127208	0.033481	0.351914
Hoxa7	0.340808	0.396551	0.397655	1.609769	0.405727	1.129353	0.264181	0.621925	0.351888
Gm6476	0.073819	0.220867	0.147655	0.146811	0.281218	0.068265	0.152591	0.070283	0.35178
Gm32600	0.13825	0.137881	0.184354	0	0.175556	0.213081	1.238361	0.482633	0.351371
Usp46os1	0	0	0	0	0	0	0	0	0.351079
Gm5628	0.781465	0.045846	0.229869	0.319977	0.481579	0.297572	0.285065	0.612732	0.350497
4930459C07Rik	0.091861	0.595506	0.091872	0.045673	0.218718	0	0.047471	0.04373	0.350206
Gm30963	0	0.366162	0.458978	0.091271	1.573468	1.018557	0.189728	0.349553	0.349917
4930502E09Rik	0.073428	0	0	0	0	0	0	0	0.349917
Gm25131	1.101418	0.366162	0.367182	1.460332	0.34966	1.358076	0	0	0.349917
Gm52595	0	0	0	0	0	0	0	0	0.349917
Bbof1	0.200137	0.365942	0.433681	0.298525	0.254145	0.215928	0.310278	0.381101	0.349706
Atg4a-ps	0.209596	0.104519	0.131013	0	0.224569	0.169599	0.189549	0.124722	0.349586
LOC118567544	0.293323	0	0.440036	0.3646	0.069839	0.542512	0.151582	0.34909	0.349454
Gm39565	0.036641	0.109631	0.146582	0.036436	0	0.237192	0.03787	0.034886	0.349223
Slc25a53	0.323191	0.300842	0.560263	0.407082	0.184682	0.278952	0.6458	0.287196	0.349101
D530033B14Rik	0.137336	0.273941	0.366273	0.455223	0.217996	0.254009	0.283888	0.74096	0.34905
Gm36753	0.199657	0.199125	0.1664	0.452227	0.285227	0.410303	0.240748	0.200654	0.348866
LOC108168367	0.091482	0.091238	0	0	0.087126	0.338399	0	0	0.348762
6030442K20Rik	0.406437	0.324284	0.243891	0.242496	0.348378	0	0.042007	0.270878	0.348634
Tmtc1	0.666633	0.857882	0.881778	1.347182	0.532492	0.636368	0.311161	0.818968	0.348423
H4c1	0.243684	0	0.243712	1.332753	0.348123	0.112676	0.755578	0.928043	0.348378
Gm26787	0	0	0.091342	0	0	0.168921	0.094395	0.521738	0.348187
Rpl15-ps5	0	0.364352	0	0.09082	0	0	0	0.173913	0.348187
Tcrb	0.296024	0.281643	0.207291	0.192139	0.297522	0.231407	0.217914	0.319032	0.348117
Gm7334	0.688923	0.727506	1.01324	0.322383	0	0.262333	0.670152	0.308669	0.347614
Gm15953	0.056083	0.335602	0.364582	0.418267	0.347184	0.596434	0.695575	0.106793	0.347439
Hrct1	0.728649	0.363355	0.060728	0.664187	0	0.336916	0.251032	0.462497	0.347234
Fgfr3	0.405986	0.349056	0.322026	0.542923	0.466654	0.504906	0.086815	0.306565	0.346912
LOC118568612	0.303272	0.060493	0.303307	0.603146	0.693198	0.224365	0.062689	0.461991	0.346854

Fgf1	0.623483	1.088191	0.467667	3.771611	1.237081	2.018023	0.483301	1.978724	0.346637
Adhfe1	0.436377	0.725359	0.181845	0.560496	0.311701	0.571693	0.676526	0.276982	0.346588
Asic3	0.436234	0.616353	0.436285	0.036149	0.242354	0.235326	0.413297	0.380726	0.346475
LOC118568483	0.103865	0.051794	0.155816	0.335671	0.14838	0.120064	0.161025	0.271947	0.346475
Il17d	0.2722	0.090492	0.453719	0.631576	0.345654	0.293676	0.046889	0.043194	0.345908
Gm34157	0	0.040211	0.040323	0.240556	0.153596	0.074571	0	0.460647	0.345845
Fbxo47	0.791501	0.756503	0.626678	0.196767	0.502545	0.48797	0.613542	0.659388	0.345753
Gm30327	1.088206	0.422065	0.725555	1.683283	0.575775	0.279539	0.687326	0.34536	0.345719
Gm35060	0.120912	0	0.241852	0.060117	0	0	0	0	0.345719
Gm7428	0	0	0.060463	0	0	0.279539	0	0.05756	0.345719
1700088E04Rik	0.103623	0	0.051817	0.360647	0.049344	0.239567	0.107099	0.295976	0.345665
Gm36412	0.12078	0.361376	0	0	0.460119	0	0.124832	0	0.345342
LOC118568363	0	0	0	0	0	0	0	0	0.345342
Olfr1169-ps1	0	0	0	0	0	0	0	0	0.345342
Gm4890	0.085224	0.403735	0.298318	0.254239	0.202915	0.295546	0.176166	0.040571	0.34521
Trim43c	0.139292	0.777956	0.696539	0.637152	0.26532	0.437963	0.403102	0.636572	0.345169
9530027J09Rik	0.460887	0.492492	0.263394	0.130944	0.219472	0.152219	0.340249	0.344779	0.345137
Col22a1	0.126654	0.063158	0.171907	0.575744	0.801284	0.736216	1.000471	0.499573	0.344892
Zfp867	0.261121	0.400656	0.42186	0.119842	0.631288	0.520104	0.332162	0.248613	0.344592
Megf10	0.140181	0.286975	0.516518	0.11005	0.1054	0.16375	0.167761	0.273957	0.344561
1810006J02Rik	0.394343	0.58994	0.098597	0.130711	0.187784	0.243117	0.475502	0.125151	0.344524
Bspry	0.587363	1.126538	0.722993	0.224644	0.473337	0.208914	0.840558	0.258105	0.344498
Kcnc3	0.516183	0.463328	0.464619	0.372136	0.565348	0.632489	0.746901	0.65118	0.344378
Gm45805	0.060193	0.09005	0.0602	0.14964	0.028664	0.194827	0.186638	0.05731	0.344218
Nrcam	0.410837	0.248329	0.385982	0.383776	0.213424	0.333877	0.501824	0.331891	0.344102
Gm9790	0	0.359029	0.24002	0.119324	0	0.110968	0.124022	0.456991	0.3431
Gm32220	0.089997	0	0	0	0	0.166453	0	0	0.3431
Gm51821	0.399794	0.491766	0.586432	1.073397	0.431527	0.147887	0.826414	0.355267	0.342935
Rnf223	0.179877	0.071759	0.323816	0.214643	0.068525	0.432496	0.148729	0.17126	0.342877
Spdya	0.224627	0	0.179723	0.268043	0.470652	0.124637	0.232164	0.427735	0.342544
Gm5553	0	0.268835	0	0.089348	0	0.166183	0	0.171094	0.342544
Gm43221	0.153852	0.102295	0.71806	0	0.146527	0.047426	0.106009	0.19531	0.342148
Gm40802	0.538234	0.2684	0.807444	1.15964	0.256304	0.663655	1.019867	0.256226	0.34199
Gm6058	0.179411	0	0	1.694859	0.170869	0	0.092715	0.085409	0.34199
Gm5503	0.538234	1.133246	1.136403	0	0.455651	0.221218	0.309051	0.341634	0.34199
Gm39872	0.051237	0.919805	0.20497	0.560446	0.341581	0.047382	0.105911	0.195129	0.341832
Rplp0-ps1	0.153426	0.25503	0.25574	0.050856	0	0.520241	0.211432	0.048692	0.341201
Cd8a	0.232557	0.196255	0.071564	0.106733	0.085187	0.281234	0.184892	0.051096	0.340996
Gm52358	0.223541	0.445891	0.357706	0.044458	0.298057	0.289413	0.323456	0.340532	0.340886
Fgd1	0.561872	0.271698	0.459767	0.998932	0.681063	0.755785	0.439942	0.502536	0.340782
Gm16548	0.089344	0.133659	0.044677	0.222109	0.255271	0.413113	0	0.170129	0.340612
Trp53tg5	0.476374	0.534494	0.833752	1.243478	0.396982	0.495603	0.307723	0.453555	0.34052
Gm12749	0.758814	0.178069	0.401772	0.754564	0.297577	0.082556	0.230668	0.509976	0.340338
LOC115487419	0.357007	1.068169	0.459062	0.405722	0.340009	0.235821	0	0.437021	0.340259
Bcas1	0.225358	0.449516	0.37564	0.373493	1.108914	0.659946	0.271739	0.607928	0.340078
Gm6201	0.547008	0.118598	0.451929	0.165548	0.203856	0.17595	0.319551	0.18115	0.340009
BC049762	0.285395	0.142318	0.713572	0.212848	0.203856	0.065981	0.147485	0.067931	0.340009
Osr1	1.634027	0.711131	0.98053	1.12264	0.452721	0.439592	0.706246	0.961738	0.33979
Fbxo39	0.097203	0.064629	0.097214	0.257756	0.462875	0.209744	0.368368	0.154245	0.339691
LOC118568751	0.018748	0.149585	0.150002	0.130501	0.035711	0.017338	0.213148	0.0357	0.339503
Gm6365	0.273903	0.10927	0.246542	0.217895	0.521725	0.151978	0.141546	0.365096	0.339371
Gm14230	0.462725	0.230747	0.355984	0.371646	0.254247	0.164582	0.147154	0.355837	0.339245
Gm12933	0.237168	0.295671	0.474391	0.11792	0.282345	0.38382	0.428969	0.62097	0.339064
LOC118567807	0.160071	0.141907	0.035576	0.017686	0.084695	0.164477	0.091912	0.457211	0.339027

Gm5602	0.133371	0.399049	0.133387	0.265249	0.254043	0.082225	0.229743	0.465603	0.338973
Xkrx	0.131015	0.093333	0.037437	0.502513	0.124777	0.017308	0.096722	0.302938	0.33893
1700001P01Rik	0	0	0	0.088345	0.084613	0.246478	0.091824	0	0.338701
Gm24036	0	0	0	0	0	0	0	0	0.338701
LOC118567775	0	0	0	0.176691	0	0.164319	0	0	0.338701
Gm32362	0	0.118142	0	0.588969	0	0.438183	0	0	0.338701
LOC108168868	0.212996	0.153421	0.059172	0.223569	0.169046	0.010943	0.428053	0.135195	0.33834
Gm5527	0.23641	0.471561	0.650202	0.235085	0.337731	0.437248	0.366512	0.337628	0.337979
Gm31728	0.779819	0.141408	0.638108	0.775451	0.742691	0.262237	0.366356	0.539974	0.337835
Gm36079	0.809315	0.554923	0.809409	0.201195	0.578087	0.701652	0.62735	0.722388	0.337465
Gm13369	0.495615	0.847364	0.531078	0.668852	0.741743	0.556542	1.939205	0.775221	0.337404
Bcat1	0.261868	0.359992	0.21235	0.29559	0.114589	0.412338	0.204819	0.289754	0.337274
H2ac21	0.35386	0.705836	0.353901	0	1.011037	0.654477	0.975285	2.246063	0.33726
D830031N03Rik	0.320181	0.365898	0.486999	0.616876	0.463759	0.326937	0.372289	0.336599	0.336949
Gm24041	0	0	0	0.351131	1.345187	0	0	0	0.336544
Pamr1	0.852893	0.674632	0.529444	1.403778	0.504178	0.652741	0.820714	0.448021	0.336365
Rbfox1	0.149282	0.419585	0.285027	0.283397	0.43945	0.489456	0.19637	0.361789	0.336297
Gm16794	0.226792	0.452377	0.604849	0.676565	0.647983	0.48937	0.911559	0.335888	0.336238
5033406O09Rik	0.384764	0.38374	0.513078	0.605797	0.122148	0	0.430812	0.152639	0.336155
Arntl2	0.528989	0.386893	0.705401	0.631231	0.100761	0.652257	0.182246	0.470073	0.336116
Gm36371	0.352267	0.746575	0.836732	0.481654	0.96455	0.447928	0.091021	0.167696	0.335742
Pdzk1ip1	0	0.843057	0.422703	0	0.201265	0.390857	0.364028	0.33534	0.335689
Gm34861	0.322485	0.526297	0.645044	0.262373	0.781788	0.081334	0.454506	0.390774	0.335298
Gm9364	0	0.070166	0.070361	0.069959	0.134007	0	0	0.066983	0.335263
5730480H06Rik	0.255622	0.286809	0.447391	0.413059	0.21302	0.177294	0.528397	0.42591	0.334992
Macrod2	0.410002	0.40891	0.234314	0.262096	0.418372	0.541652	0.030268	0.195181	0.334944
LOC118567586	0	0	0	0	0	0	0	0	0.334944
B3gnt6	0.117106	0.303665	0.23424	0.535671	0.156143	0.086637	0.508347	0.445986	0.334838
Gm20036	0.397657	0.209964	0.49128	0.046521	0.089112	0.086527	0.169235	0.267253	0.334414
0610039K10Rik	0.408061	0.116279	0.524711	1.043423	2.054204	0.970361	1.205005	0.999037	0.333359
Gm11687	0.291472	0.232557	0.233205	0.28984	0.277595	0.646907	0.241001	0.610523	0.333359
Cdk3	0.893536	0.309968	0.621662	0.309054	0.702995	0.790387	0.200764	1.072664	0.333242
Yjefn3	0.069909	0.139446	0.27967	0.764695	0.732389	0	0.072255	0.06656	0.333149
Jakmip1	0.37238	0.580295	0.256041	0.486011	0.288154	0.753299	0.240546	0.332383	0.332729
Gm41668	0.174516	0.116035	0.058179	0	0.44322	0	0.420867	0	0.332659
Cpsf4l	0.548398	0.696103	0.698042	0.743627	0.474808	0.829868	0.669851	0.142399	0.33261
LOC102639428	0.149563	0.198887	0	0.1983	0.522288	0.276623	0.309162	0.047466	0.33261
Gm16576	0.23595	0.542264	0.277017	0.428452	0.33219	0.417425	0.40291	0.234415	0.332434
Rnf170-ps	0.479041	0.419854	0.304879	0.346442	0.456233	0.416153	0.465106	0.511378	0.33205
Gm30110	0.232201	0.521062	0.116114	0.057725	0.165859	0.107366	0.119996	0.110539	0.331963
Pacsin1	0.133941	0.173659	0.174143	0.332976	0.293396	0.322047	0.152278	0.331564	0.331909
Itga11	0.24704	0.671952	0.482907	0.859797	0.245973	0.695749	0.603503	0.673546	0.331772
Gm40884	0.031643	0.094675	0.031646	0.031465	0.060272	0.029262	0	0.060254	0.331742
Gm38427	0	0.416239	0.139133	0	0.463727	0.064325	0	0	0.331477
Bmp5	0.289721	0.086685	0.173853	0.576196	0.234538	0.428679	0.449161	0.303427	0.331356
Gm52883	1.042722	0.866622	0.347614	1.382508	1.324103	0.482138	0.898088	0.165462	0.331269
Abca5	0.595708	0.953071	0.930902	0.320868	0.200935	0.309877	0.29502	0.413563	0.331195
En1	0.217064	0.15154	0.19538	0.151093	0.020673	0.481761	0.134608	0.082666	0.33101
F420014N23Rik	0.20824	0.161533	0.185124	0.690244	0.594976	0.449339	1.076131	0.396529	0.330785
Ulk4	0.194325	0.484518	0.305403	0.317459	0.211512	0.192542	0.559495	0.502187	0.33073
Gm31439	0.277537	0.345998	0.242873	0.344978	0.75993	0.57748	0.860546	0.759698	0.330647
Gm4349	0.445883	0.197642	0.29729	0.39412	0.141551	0.137446	0.204819	0	0.330529
Gm41396	0.411615	0.302488	0.389997	0.323139	0.16506	0.420717	0.246299	0.268141	0.330363
Ablim2	0.692976	0.531639	1.466081	0.821612	0.71075	0.073943	1.267167	1.141928	0.330234

Ric3	0.202118	0.028797	0.115509	0.775231	0.054999	0.133509	0.32827	0.054982	0.330234
Mkx	0.034638	0.12091	0.190531	0.327217	0.56081	0.592594	1.145602	0.676064	0.330131
Gm35039	0.519371	0.36451	0.250097	0.286923	0.256482	0.160099	0.477152	0.293032	0.330005
Gm6392	0.588294	0.276107	0.173048	0.240882	0.16479	0.256017	0.357665	0.329479	0.329821
Gm4948	0.29646	0.394228	0.148247	0.442199	0.282345	0.137079	0.102135	0.235216	0.329645
Sycp3	0.04941	0.049278	0	0.049133	0.141173	0.045693	0	0	0.329645
Afp	0.451796	0.159033	0.318952	0.396411	0.227798	0.024577	0.192275	0.151819	0.329284
Rad9b	0.898062	0.93012	0.690897	1.064768	0.690823	0.574961	1.14239	0.756384	0.329205
Gm39520	0.561289	0.861222	0.38863	0.772816	0.904649	1.038125	0.713994	1.068804	0.329205
Spag11b	0	0	0	0	0	0	0	0	0.329205
Gm39637	0	0	0	0.274494	0.043816	0	0	0	0.328863
Gm13535	0.053076	0.052935	0.026541	0.184726	0.050549	0.024542	0	0.176868	0.328811
LOC118568158	0.172436	0.859883	0.776051	4.201009	0.821129	0.558121	1.158436	1.067142	0.328693
Kif26a	0.40068	0.327683	0.136247	0.828746	0.48082	0.689197	0.190496	0.213632	0.32842
Pet117	0	0.17171	0	0	0	0	0	0	0.328183
Gm34704	0.068867	0	0.068875	0	0	0.50949	0.071178	0	0.328183
1810010H24Rik	0.30971	0.274565	0.240913	0.376414	0.098321	0.09547	0.426801	0.032764	0.327979
Pde1a	0.068782	0.754585	0.412738	0.205189	0.622316	0.190822	0.248813	0.360178	0.327775
Gm16618	0.800963	0.342356	0.686619	0.227565	0.272439	0.687798	0.236524	0.599182	0.327167
Scrn1	0.280778	0.062229	0.811231	0.930684	0.207985	0.490459	0.290198	0.47525	0.327074
Adra2b	0.109697	0.21881	0.054855	0.054541	0.391778	0.304333	0.170066	0.104442	0.326721
Gm10231	0.257054	0.51274	0	0	0	0.316954	0.619917	0	0.326661
Gm30322	0	0.170913	0.085695	0	0.163211	0	0.08856	0	0.326661
4930524J08Rik	0.855526	0.853248	2.481313	1.53132	1.792545	1.186745	1.503188	0.814544	0.326157
Fam3b	0	0	0	0	0	0.105488	0.058949	0.271515	0.326157
Gm20082	0	0	0.570417	0	0.108639	0	0	0	0.326157
Gm26121	0	0	0	0	0	0.316465	0	0.325818	0.326157
Lefty1	1.060197	0.579851	0.752486	1.02025	0.097715	0.569286	0.212084	0.58611	0.325956
Gm39165	0.068358	0.068176	0.410194	0	0	0.063215	0.211953	0.065083	0.325755
Fndc3c1	0.105121	0.196577	0.433674	0.313596	0.175203	0.085061	0.203714	0.450384	0.325616
Gm38664	0.287616	0.107569	0.197759	0.26813	0.205442	0.199484	0.241529	0.376529	0.325522
Zfp108	0.255966	0.765855	0.831988	0.996919	0.711024	0.453694	0.220462	0.48741	0.325278
Adamts15	0.294823	0.192256	0.283517	0.823135	0.345583	0.387992	0.175797	0.194331	0.324222
Klra13-ps	0.136044	0.135682	0.27212	1.014617	0.712619	0.880667	0.632739	0.453346	0.324156
Gm21011	0.304113	0.231938	0.107347	0.44472	0.289634	0.46321	0.536187	0.357674	0.323947
Mirc35hg	0	0.338859	0	0.05631	0.053931	0.104734	0.058527	0.215659	0.323825
Flywch2	0.169795	1.608758	1.103796	1.181907	1.051121	1.727232	0.877458	0.080831	0.323659
B930059L03Rik	0.042449	0.211679	0.891527	0	0.646844	0.117766	0.350983	0.404154	0.323659
Gm28626	0	0.338686	0	0.084422	0.242566	0.078511	0.263237	0.080831	0.323659
Gm52920	0.322147	0.286866	0.512043	0.5606	0.345161	0.473467	0.457813	0.405303	0.323483
Wnt10a	0	0.056361	0.141296	0.11239	0	0	0.087612	0.161414	0.323165
Lmo7	0.561043	0.38469	0.385762	1.255276	0.656782	0.659353	0.22953	0.645453	0.323062
Gm42198	0.042351	0.084477	0.211782	0.673828	0	0.156661	0.218862	0.120968	0.322918
Klhl40	0.27098	0.85582	0.564608	0.291918	0.408625	0.751782	0.070018	0.43	0.322835
Gm18413	0.423191	0.084413	0.084648	0.252492	0.483651	0	0.262434	0.080584	0.322671
Inha	0.07517	0.14994	0.037589	0.149498	0.178978	0	0	0	0.322398
Trp53-ps	0.9127	0.775415	0.845191	0.806744	0.708274	0.906557	1.048134	0.482766	0.322179
Gm6974	0.253528	0.084284	0.169038	0.168072	0.160971	0.312606	0.611412	0.321844	0.322179
Gm13196	0.112679	0	0.112692	0.336143	0.429257	0	0.465837	0.107281	0.322179
Smtnl2	0.295782	0.484634	0.084519	0.168072	0.925585	0.312606	0.305706	0.301729	0.322179
Gm16867	0	0.449516	0.394422	0.112048	0.053657	0.156303	0.232919	0.268204	0.322179
Ccdc63	0.135215	0.033714	0.135231	0.302529	0.064389	0.125042	0.034938	0.160922	0.322179
Gm40128	0.464447	0.4211	0.042227	0	0	0.156184	0.087278	0.1608	0.321934
Cyp3a13	0.056282	0.056132	0.093815	0	0.017868	0	0.077561	0.107173	0.321852

Iqcg	0.099316	0.217913	0.019865	0.296278	0.056752	0.257164	0.123177	0.208028	0.321833
Rpl13a-ps1	0.168761	0.168312	0.253171	0.41954	0.401816	0.31213	0.261635	0.482032	0.321689
Anp32-ps	0.11245	0	0.337391	0.335462	0.428386	0.311972	0.116223	0.428255	0.321526
Itpka	0.061325	0	0.030666	0.182945	0.029203	0.085068	0	0.058388	0.321466
LOC118568760	0.042126	0.336112	0.631966	0.502683	0.641928	0.311656	0.348316	0.401083	0.3212
Acacb	0.350064	0.315562	0.148121	0.642652	0.269282	0.385984	0.250483	0.217924	0.32081
Gm52283	0.168198	0.0671	0.067287	0	0.032038	0.062218	0.139073	0.096085	0.320615
Gm8273	0.335988	0.111698	0	0	1.813285	0.10357	0.463014	0.426525	0.320227
Gm26110	0.335988	0.335094	1.680137	0.334106	0.959975	2.796403	0.347261	0.319894	0.320227
Marcksl1-ps3	0.251991	0.418867	0.294024	0.12529	0.279993	0.155356	0.086815	0.479841	0.320227
Myom2	0.402942	0.826064	0.638065	0.589897	0.330458	0.6728	0.173525	0.60743	0.320033
Map1a	0.269586	0.365255	0.254356	0.551326	0.295506	0.366901	0.383774	0.193715	0.319962
Chst3	1.088266	1.817992	1.32421	0.495994	0.190016	0.335465	0.496776	0.423089	0.319808
4930534H03Rik	0	0	0	0	0.319507	0	0.086684	0	0.319742
Gm39417	0	0	0	0	0	0	0	0	0.319742
Bmper	0.699166	0.406761	0.291353	0.521437	0.610388	0.188582	0.331202	0.540861	0.319301
LOC118567967	0	0.08352	0.083753	0	0	0	0	0	0.319259
Gm34939	0.121575	0.454691	0.273575	0.302234	0.521039	0.421607	0.376961	0.318315	0.318647
Gm17045	0.3343	0.166705	0.111446	0.110809	0.106128	0.051525	0.057586	0.159143	0.318617
Grip2	0.097014	0.193511	0.215612	0.20366	0.225854	0.129589	0.144833	0.256574	0.318483
Gm51963	0.222643	1.11025	0.556672	0.110698	0.742149	0.514733	2.531237	0.317967	0.318298
Gm51734	0	0	0	0	0	0	0	0	0.318298
Hmgb1-ps1	0.214599	0.118904	0.071541	0.260818	0.136254	0.352808	0.098577	0.249724	0.318161
Gper1	0.357511	0.213936	0.9058	0.308107	0.363189	0.683271	0.566575	0.839618	0.318024
Gm6129	0.466987	0.199605	0.667202	0.464371	0.190609	0.308468	0.275803	0.508134	0.317915
Gm6161	0.083366	0.374146	0	0.290145	0.357284	0.385469	0.301568	0.317489	0.317819
Gm33148	0	0.083144	0	0.082899	0.079396	0	0	0	0.317819
Gm52284	0.555492	0.44321	0.444445	0.441904	0.423235	0.10274	0.688954	0.211553	0.31766
Gm6781	0.199897	0.166137	0.46648	0.066259	0	0.061619	0.309905	0.03172	0.317532
Gm33325	0.832403	0.166037	0.1665	1.489933	0.317109	0.461868	1.204463	1.585059	0.317342
Gm36304	0	0.166037	0.08325	0.248322	0.475663	0.153956	0.516198	0.396265	0.317342
Gm9387	0.060522	0.120722	0.211851	0.481463	0.345842	0.251859	0.125105	0.403359	0.317255
Cobll1	0.585129	0.617898	0.562248	1.197928	0.633758	1.060997	0.379457	0.764647	0.317112
Abhd11os	0.554381	0.552905	1.219779	0.110255	1.055972	1.230417	1.14596	0.950084	0.317024
Tymp	0.546305	0.473783	0.261307	0.259813	0.429809	0.219655	0.736478	0.723667	0.316934
Platr7	0.124673	0.041447	0.041563	0.041325	0.514529	0.115294	0.171808	0.395671	0.316866
Gm13421	0.158304	0.347342	0.411638	0.409285	0.105537	0.54166	0.490845	0.211009	0.316843
Gm10356	0.166044	0.207003	0.145306	0.28895	0.019767	0.076776	0.150163	0.039523	0.31651
Wfdc1	0.521658	0.189189	0.094858	2.122103	1.038809	0.877115	0.392116	0.090304	0.316392
Gm46578	0.110655	0.11036	0	0.44014	0.526932	0	0	0	0.316392
Gm1123	0.082929	0.041354	0.290285	0	0	0.07669	0.214278	0.078957	0.316155
Vsig2	0.426447	0.330798	0.189554	0.141353	0.045127	0.569638	0.244863	0.045113	0.316121
4632427E13Rik	1.657341	0.826464	1.326027	1.73046	0.47353	1.149493	2.569417	0.946771	0.315919
Gm31015	0.343306	0.519492	0.331507	0.329612	0.304412	0.186108	0.39153	0.31559	0.315919
Fsip1	0.132548	0.165243	0.198845	0.329513	0.220915	0.214508	0.239741	0.126198	0.315824
Gm16044	0.110434	0	0.441789	0.878526	1.051765	0	1.826231	0.946299	0.315761
Fam174b	0.269117	0.43357	0.062111	0.802828	0.059147	0.995483	0.213958	0.394193	0.315683
Nova2	0.245008	0.105667	0.119207	0.869187	0.245956	0.189834	0.109504	0.138702	0.31556
Gm11814	0.110361	0.256824	0.257539	0.036581	0.175178	0.068039	0.30417	0.350249	0.315552
Dusp27	0.19302	0.481265	0.523972	0.315328	0.354529	0.331498	0.227995	0.367548	0.315369
1700086O06Rik	0.708927	0.282816	0.614474	0.516967	0.810209	0.524475	0.488474	0.359983	0.315313
Trgc2	0.110215	0.109921	0.220455	0.219195	0	0	0	0.314806	0.315134
Pdzd3	0.050837	0.278861	0.025422	0.176934	0.072625	0.211557	0.446614	0.169407	0.314941
Cacng6	0.256998	0.805557	0.330464	0.146033	0.384626	0.373471	0	0.174776	0.314925

Plp1	1.205894	0.494253	0.313899	1.264845	0.707967	0.886028	0.495126	0.393195	0.314883
Gm52912	0.256658	0.219407	0.183348	0.5469	0.244438	0.169535	0.682119	0.279272	0.314508
Gm46058	0.696182	0.877046	0.989426	1.712488	0.383863	0.508269	0.719539	0.558175	0.3143
Stk19-ps1	0.144263	0.164433	0.082445	0.143455	0.078511	0.019059	0.213004	0.11773	0.314274
Bcl2l14	0.506978	0.101126	0.304222	0.025207	0.627693	0.632931	0.183396	0.36202	0.314077
Tshr	0	0.041078	0.041192	0	0.235358	0.266621	0	0	0.314041
Gm35090	0	0	0	0.109163	0	0.10152	0	0	0.313886
Itgbl1	0.37633	0.797571	0.77627	0.584721	0.604821	0.413271	0.291717	0.358303	0.313841
Tspan11	0.210741	0.275861	0.131728	0.432219	0.137986	0.414134	0.149745	0.125404	0.313836
Gm11767	0.269407	0.507526	0.359252	0.178599	0.28509	0.276822	0.185631	0.256502	0.313829
Gm32051	0.054835	0.164067	0.054841	0.218111	0.052224	0	0	0	0.313575
4930522P08Rik	0.822523	0.820333	0.822619	1.145082	1.096707	0.912772	0.510071	2.662617	0.313575
Gm18649	0.657368	0.218539	1.424464	0.762634	0.313035	0.101319	0.905897	0.104313	0.313265
Tcaf2	0.149268	0.357289	0.432927	0.32655	0.056864	0.358899	0.015428	0.085271	0.312984
Gm12474	0.131059	0.108925	0.174766	0.26065	0.312048	0.201999	0.451521	0.145578	0.312278
Gm5456	0.917415	0.065355	0	0.130325	0.187229	0.181799	0.135456	0	0.312278
Gm29257	1.310205	0.408349	0.818974	0.325717	0.85788	1.13591	0.338541	0.623722	0.312186
Gm14769	0.10913	0	0	0	0.103934	0	0	0	0.312032
Gm32112	0	0	0	0.260343	0.187008	0	0.202945	0.186951	0.311909
Hus1b	0.233719	0.279716	0.420742	1.905758	0.623255	0	0.48312	0.222523	0.311856
Dnajb3	0.763159	0.326197	0.981319	0.162618	0.467244	0.201642	0	0.415201	0.311725
D830024N08Rik	0.7995	1.051081	0.963146	1.047983	0.969099	0.856977	0.619742	0.657402	0.311725
Ttc41	0.535059	0.830098	0.356747	0.443385	0.509584	0.632252	0.952407	0.481127	0.311641
Gm30054	0.518534	0.326032	0.473499	0.627723	0.40796	0.271035	0.233013	0.397103	0.311567
Gm20147	0.081647	0.162859	0	0.081189	0	0	0.421929	0.233207	0.311266
Gm39439	0.027216	0.027143	0	0.324757	0.077759	0	0.112514	0.181383	0.311266
Hoxa9	0.562363	0.180924	0.163286	0.559212	0.241878	0.469727	0.206243	0.276348	0.311215
Gm41844	0.424188	0.455601	0.261069	0.5516	0.24861	0.331925	0.404695	0.590269	0.310991
Ache	0.075289	0.375445	0.351391	0.274515	0.239016	0.394543	0.051877	0.119471	0.310949
BC065397	0.171688	0.256846	0.051512	0.239017	0.245271	0.285789	0.319407	0.310581	0.310904
Snca	0.232933	0.046463	0.093184	0.555908	0.93174	1.120128	0.337047	0	0.310808
Gm10190	0	0.032524	0	0	0	0	0	0	0.310808
LOC118568479	0.221526	0.277254	0.256305	0.786117	1.526497	0.96405	0.36364	0.260542	0.310492
Trim54	0.325681	0.505265	0.506673	0.107952	0.103392	0.368108	0.149603	0.275626	0.310402
Gm36839	0	0.185538	0.046514	0	0	0	0	0.044281	0.310287
Gm13743	0	0.108201	0.108502	0	0.103324	0.050164	0.056065	0.051646	0.3102
Itgad	0.310453	0.25065	0.458341	0.117605	0.253433	0.300769	0.244471	0.225205	0.309979
D130017N08Rik	0.26012	0.389141	1.073119	1.13165	0.123868	0.330757	0.705723	0.433404	0.309897
Gm7299	0.32515	0.243213	0.325188	0.727489	1.083842	0.526205	0.588103	0.541756	0.309897
Trgv5	0	0	0	0	0.619338	0	0	0.619149	0.309897
Fst	0.693518	1.015892	0.303449	0.237061	0.309609	0.541133	0.268794	0.28888	0.309836
R74862	0.574617	0.47342	0.549698	0.94405	0.404496	0.161727	0.464788	0.118933	0.309548
Zfp819	0.024983	0.049834	0.024986	0	0.023794	0.046208	0	0	0.309548
Gm5915	0.216434	0.215858	0.154614	0.199849	0.412259	0.271634	0.127826	0.294381	0.309421
9330175E14Rik	0.216273	0.539244	0.684945	0.896091	0.823905	0.70001	0.670588	0.823654	0.309192
LOC118568650	0.1621	0.40417	0.324237	0.040298	0.270168	0.374762	0.753921	0.077167	0.308991
Gm8523	0	0.080834	0	0	0	0	0.502614	0	0.308991
Avil	0.040525	0.424379	0.243178	0.201489	0.270168	0.243595	0.104711	0.289378	0.308991
Chdh	0.084015	0.155613	0.036011	0.250634	0.1486	0.355176	0.210882	0.091418	0.308857
Dpm1	0.299126	0.149165	0.648182	0.272663	0.094962	0.138311	0.386452	0.071199	0.308852
Chn1	0.051158	0.238101	0.204655	0.474799	0.178648	0.078849	0.123373	0.243536	0.308801
Abcc9	0.647893	0.581552	0.388781	1.47107	0.617046	0.419406	0.312494	0.729948	0.30875
Gm46302	0	0.565012	0.161882	0.563347	0.46247	0	0.418234	0	0.30854
Gm33786	0.215712	0.501989	0.503387	0.250255	0.102721	0.066495	0.557374	0	0.308389

LOC100038987	0.754993	0	0.215737	0.321756	0.410884	0.199484	0	0	0.308389
Gm32687	0.092397	0.253415	0.161713	0.298607	0.219994	0.064084	0.095497	0.175942	0.308218
Gm6012	0.193952	0.153137	0.072741	0.257155	0.346347	0.306409	0.250574	0.315464	0.30809
Slc43a1	0.645701	1.057971	1.060918	1.238305	0.922438	0.853035	0.667364	0.39521	0.307705
Igsf3	0.409311	0.259064	0.629783	0.446155	0.254885	0.291168	0.187115	0.27729	0.307587
Gm2383	0.161313	0.214512	0.21511	0.05347	0.307266	0.248629	0.277876	0.255977	0.307492
9330104G04Rik	0.570504	0.148431	0.347303	0.493311	0.519718	0.527584	0.051273	0.212547	0.307332
LOC118568082	0.492154	0.293378	0.384717	0.77066	0.145466	0.292956	0.748382	0.339316	0.30732
Sema3b	0.322314	0.711796	0.391427	0.686805	0.635864	0.276776	0.523487	0.263036	0.307194
Gm23887	0	0.321456	0.644704	1.282035	0.613937	0.298066	0.333128	0.920624	0.307194
Gm51917	0.483471	0.642912	0	0	0	0.447099	0.333128	0	0.307194
Gm19739	0	0	0	0	0	0	0	0	0.307194
Gm36347	0.080579	0	0.080588	0	0	0	0	0	0.307194
Gldc	0.629812	0.598919	0.19043	0.27673	0.376635	0.203173	0.257349	0.223123	0.307113
F730043M19Rik	0.500408	1.461579	0.750699	1.030753	0.748916	0.29749	0.812738	0.374344	0.3066
Plcxd1	0.880238	0.624656	0.744903	0.387155	0.628747	0.532242	0.75231	0.45127	0.306538
Efcab5	0.345446	0.451448	0.405052	0.130297	0.26093	0.187268	0.209297	0.351581	0.306534
Pitx3	0.160807	0.922176	0.241238	0.719576	0.344588	1.189671	0.290853	0.65069	0.306526
Neto2	0.462319	0.250591	0.422166	0.299823	0.143579	0.223063	0.249302	0.181811	0.306526
Gm34854	0.535806	0.694693	0.410832	1.065609	0.595345	0.743245	0.646079	0.646177	0.306402
Cyp2w1	0.16069	0.040066	0	0.079895	0.229559	0	0.04152	0.076496	0.306304
3830408C21Rik	0.730217	0.378702	0.379757	0.755172	0.083454	0.054023	0.332075	0.083429	0.306223
4930412C18Rik	0.083807	0.055722	0.083816	0.388907	0.106422	0.245422	0.707383	0.345766	0.306188
Ripor3	0.320946	0.341431	0.406579	0.510637	0.203777	0.474881	0.088457	0.346315	0.30589
Gm9176	0	0	0.040119	0.558455	0.114613	0	0.290221	0	0.30586
Lrrc69	0.229083	1.005281	0.595685	0.09112	0.567258	0.381328	0.947074	0.610706	0.305671
Gm12226	0.391914	0.213202	0.285062	0.106287	0.135729	0.36243	0.441887	0.407063	0.305615
Reep1	0.116589	0.377905	0.21863	0.3623	0.194317	0.350408	0.06025	0.152631	0.305579
Zfp30	0.11219	0.479536	0.192349	0.366561	0.290018	0.340893	0.198779	0.167854	0.305507
Gm10313	0.412009	0.228284	0.320488	0.091045	0.348793	0.465682	0.189258	0.305101	0.305418
1500035N22Rik	0	0	0.192293	0	0	0.059269	0	0.06102	0.305418
Rnft2	0.32021	0.249932	0.459485	0.443014	0.119334	0.514991	0.402899	0.291616	0.305188
Rhod	0.39941	0.796693	0.719021	1.429821	0.760787	0.627915	0.743058	0.646471	0.304538
Gpr33	0.559174	0.478016	0.559239	0.158869	0.190197	0.738723	0.206405	0.380277	0.304538
Gm6197	0.119823	0.239008	0.199728	0.476607	0	0.147745	0.123843	0.038028	0.304538
Gm34147	0	0.239008	0	0	0.456472	0.073872	0.041281	0	0.304538
Kif1a	0.679555	0.521864	0.577689	0.324359	0.686032	0.86095	0.442483	0.271741	0.304408
Gm36712	0.01878	0	0.037564	0	0.017886	0.034734	0.155279	0.01788	0.30428
4921517D16Rik	0.106339	0.035352	0.017725	0.176239	0.084397	0.049169	0.256449	0.168742	0.304052
Gm35781	0.063795	0.127251	0.127605	0.126876	0.121516	0.412971	0.263742	0.121479	0.304013
Gm52733	0	0.127251	1.020842	0	0.303789	0.058996	0	0.182218	0.304013
Gm13479	0.06564	0.243158	0.32824	0.503533	0.250061	0.095389	0.300446	0.294625	0.303869
Gm40194	0.677045	0.516362	0.358477	0.039603	0.22758	0.88392	0.535111	0.151674	0.303663
Gm34752	0.371711	0.476642	0.106216	0.052804	0.252867	0.049107	0.439065	0.455021	0.303663
Gm7525	0.531016	0	0.318647	0.63365	0.202294	0.29464	0.109766	0.101116	0.303663
Gm7871	0	0	0	0	0	0	0	0	0.303663
Cyp4f37	0.344995	0.238207	0.132706	0.131947	0.783512	0.269958	0.109714	0.252669	0.303518
Ankrd53	0.045478	0.090714	0	0.045224	0.086626	0.084114	0.235021	0	0.303414
Gm51528	0.045478	0.045357	0.045484	0	0	0	0.094008	0	0.303414
Gm51551	0.381893	0.317397	0.509249	0.443046	0.424329	0.235441	0.592057	0.424199	0.303315
Miox	0	0	0	0	0	0	0	0	0.302793
4921536K21Rik	0.317636	0.126716	0.423564	0.273742	0.342848	0.274157	0.700358	0.504034	0.302735
Gm12418	0.173244	0.60474	0.202141	0.229698	0.164996	0.026702	0.537169	0.192436	0.302714
Bloc1s6os	0	0.26379	0.21162	0.052603	0	0	0.054674	0.30219	0.302504

Gm31437	0.06343	0.44283	0.063438	0.12615	0.06041	0	0.327792	0.66431	0.302273
Gm6877	0.354416	0.39068	0.279834	0.389528	0.26648	0.293252	0.134955	0.142079	0.302233
Lax1	0.317068	0.243249	0.439068	0.60633	0.766544	0.586429	0.378121	0.557316	0.302194
AU021092	1.330514	0.821458	0.443556	3.71717	2.956722	2.343652	0.785801	1.689039	0.301928
Gm13562	0.428453	0.947518	0.987419	1.444875	0.709659	0.757986	0.943415	0.815858	0.301827
Gm7776	0	0.063117	0	0	0.301362	0.351146	0.261635	0.180762	0.301583
4930413G21Rik	0.158169	0	0.039547	0.039321	0.225957	0.219404	0.163475	0.150592	0.301497
Gm3436	0.143622	0.487015	0.37346	0.314198	0.191498	0.425015	0.445321	0.51962	0.301146
Spon2	0.660661	0.487015	0.287277	0.628397	0.382995	1.195354	0.445321	0.273484	0.301146
Chadl	0.315886	0.292542	0.180527	0.29168	0.537226	0.208658	0.583008	0.128895	0.301068
Gm14853	0.549702	0.664885	0.32752	0.523363	0.512392	0.324477	0.713203	0.478828	0.300973
Gm29776	0.315527	0.125875	0.031556	0.15688	0.03005	0.029179	0.326113	0.150206	0.300725
Gm12568	0.135215	0.134855	0	0	0.30048	0.208404	0.046584	0.17165	0.300701
Gm32802	0.473059	0.247133	0.270351	0.47041	0.25745	0.10416	0.488931	0.450399	0.300578
Tent5b	0.02423	0.048331	0.024233	0	0.023076	0	0.550942	0.069208	0.300212
Gm7290	0.236242	0.078538	0	0.234918	0.224994	0.072823	0.244168	0.374875	0.300212
Defb37	0.157494	0	0	0	0	0	0	0	0.300212
Actr3b	0.343447	0.342533	0.400735	0.256142	0.190805	0.344076	0.207066	0.299746	0.300058
Irs1	0.175456	0.241364	0.139171	0.439191	0.16134	0.251777	0.168836	0.190093	0.299852
Gm18821	0	0.078426	0.078645	0	0	0	0	0.149738	0.299787
Gm7818	0.044925	0.044806	0.044931	0	0	0	0.046433	0	0.299726
Gm40934	0.314394	0	0.20962	0.208422	0	0.193828	0.324941	0	0.299645
Gm48849	0.209596	0	0.62886	0.416843	0.299425	0.193828	0.108314	0.498889	0.299645
Gm5113	0.574207	0.929252	0.433414	0.430936	0.350821	0.160305	0.257544	0.371344	0.29945
Rassf9	0.104699	0.078315	0.104711	0.468506	0.398856	0.387289	0	0.249209	0.299362
Cd3d	0.125568	0.438316	0.627911	0.499457	1.315481	1.741814	0.973352	0.239105	0.299193
LOC118567543	0.1046	0.104322	0	0	0.29886	0	0	0	0.29908
Ccdc136	0.062748	0.156453	0.031378	0.436777	0.179282	0.203097	0.064853	0.47794	0.299023
Gm36040	0.235239	0.312817	0	0	0	0	0.081044	0.149314	0.298939
9330136K24Rik	0.179121	0.535933	0.179142	0.155853	0.298538	0.57976	0.462827	0.234494	0.298758
Gm17089	0.195756	0.50761	0.156623	0.038932	0.111861	0.144823	0.323718	0	0.298516
Gm34425	0.313209	1.40569	1.09636	0.830547	0.795459	0.579292	0.269765	1.391628	0.298516
Lnx1	0.120465	0.288347	0.337342	0.694784	0.11473	0.267366	0.249013	0.619351	0.298516
Gm4353	0.375639	0.1561	0.125228	0.249023	0.327942	0.231586	0.194121	0.298038	0.298348
Ndrg4	1.397423	1.331298	1.23071	1.431076	0.715105	0.327895	0.582034	0.476591	0.298179
Gm18760	0	0	0.312804	0	0	0	0	0	0.298095
Gm9574	0.142149	0.652144	0.227465	0.480599	0.379067	0.184037	0.381987	0.108272	0.298057
Gm7936	0.104207	0.207859	0	0.103623	0	0	0.107703	0	0.297955
Pglyrp4	0.416436	0.25958	0.260303	0	0.148728	0.192554	0.134502	0.396488	0.297675
Gm30679	0.166543	0.062287	0.333125	0.020701	0.456015	0.250273	0.279712	0.257669	0.29762
Gm39150	0.446002	0.622741	1.15974	0.177402	0.934489	0.16498	0.829738	0.169855	0.297556
Gpc6	0.29567	0.286691	0.24642	0.465521	0.320703	0.136713	0.135817	0.21113	0.297455
Gm16537	0	0.077764	0	0	0	0	0	0	0.297257
Gm32553	0.566562	0.113011	0.424971	0.309863	0.458649	0.157182	0.351342	0.350624	0.296991
Zfp773	0.384859	0.329001	0.476548	0.309808	0.488714	0.338958	0.359888	0.122141	0.296937
Gm34702	0.233587	0.465931	0.155743	0.851689	0.222466	0.036002	0.442611	0.074133	0.296839
Elovl7	0.377659	0.332341	0.266614	0.209862	0.232733	0.205439	0.321447	0.370144	0.296423
Gm38469	0.069082	0.137796	0.138179	0.103042	0.098689	0.319423	0.142799	0.032886	0.296284
Sim2	0.310498	0.197064	0.112922	0.505242	0.483898	0.104414	0.35009	0.456875	0.295932
Gm33104	0.558101	0.247385	0	0.801631	0.354353	0.057346	0	0.059041	0.295511
Zfp473	0.30971	0.138998	0.325233	0.12319	0.265468	0.272089	0.208065	0.28013	0.295181
LOC108168889	0	0.061777	0	0.061595	0.353957	0.057282	0.06402	0.235899	0.295181
Zfp78	0.285825	0.368207	0.393055	0.426335	0.567117	0.407496	0.418503	0.430877	0.295118
Barx2	0.061907	0.154356	0.154786	0.123121	0.05896	0.05725	0.191953	0.206297	0.295016

Gm5823	0.309278	0.231341	0.231985	0.230659	0.220915	0.143005	0.079914	0.147231	0.294769
Mrps36-ps2	0.309134	0.10277	0.206113	0.307403	0	0.285878	0.532509	0.098109	0.294632
Tmem89	0	0.616622	0.206113	0	0.098139	0	0.319506	0.882978	0.294632
Fzd6	0.308675	0.369424	0.308711	1.933761	0.705549	0.513816	0.127612	0.352667	0.294195
6430573P05Rik	0.308561	0.333384	0.257164	0.536956	0.097957	0.047558	0.318913	0.048963	0.294086
Mapk4	0.044057	0.131818	0.073436	0.08762	0.041959	0.230873	0.136605	0.013982	0.29393
Ly6h	0	0	0	0.122642	0	0.627298	0	0.352275	0.293868
Pih1d2	0.044033	0.263497	0.352308	0	0.461306	0.203604	0.546129	0.125772	0.293774
Lrrn4cl	0.660239	0.877975	0.176084	1.334966	0.649764	0.71233	0.181971	0.796242	0.293658
Gm41408	0.298656	0.242012	0.196016	0.102088	0.19555	0.39702	0.511245	0.408753	0.293541
Gnal	0.523097	0.327344	0.328256	0.397775	0.293054	0.341466	0.296826	0.283199	0.29327
Gm13246	0.153568	0.306318	0.307172	0	0.292513	0.28403	0.15872	0.292424	0.292728
1700112J16Rik	0	0.459478	0.153586	0	0	1.136119	0	0	0.292728
Gm42111	0.255829	0.255148	0.255858	0.356154	0.462932	0.544139	0.185088	0.09743	0.292593
Gm46869	0.442959	0.135932	0.102233	0.203298	0.032452	0.157552	0.387387	0.129766	0.292278
Fbxl13	0.087614	0	0.109531	0.23959	0	0.344348	0.135831	0.062563	0.292265
Gm8495	0.122651	0.428134	0.061332	0.060982	0.116811	0.056712	0.063383	0	0.292242
Mrgprh	0	0.091692	0.183896	0.121896	0	0.02834	0.031674	0.029178	0.292081
Ldhd	0.382992	0.439268	0.248974	0.628397	0.218854	0.442724	0.237505	0.200555	0.29202
Gng13	0	0.152736	0.153162	0	0.145852	0	0	0	0.291919
Gm32304	0	0	0.051054	0	0	0.141623	0	0	0.291919
LOC118568377	0.2105	0.515306	0.746405	0.4567	0.601434	0.265451	0.197784	0.58303	0.291819
Gm13067	0.1868	0.139727	0.20758	0.443745	0.158139	0.143956	0.278875	0.222315	0.291783
Gm40245	0.222643	0.416344	0.055667	0.608838	0.344569	0.102947	0.431461	0.211978	0.291773
Gm17619	0.114699	0.038131	0	0.076038	0.291303	0.035357	0.316127	0.145607	0.291517
Tox2	0.224281	0.142344	0.020392	0.486598	0.427205	0.527947	0.442537	0.427074	0.29149
Gm46508	0	0	0	0	0	0	0	0	0.291383
Gm39603	0.388844	0.554013	0.416667	0.386666	0.423235	0.308221	0.430596	0.211553	0.291188
Ak5	0.067856	0.338375	0.237522	0.067476	0	0.094126	0.035066	0.064605	0.291026
Rnase12	0.406885	0	0.406932	0.101151	0.484391	0	1.261607	0	0.290848
Fbxw10	0.588903	0.425311	0.507734	0.302898	0.348123	0.50704	0.566684	0.406019	0.290315
Ccdc116	0.257742	0.327163	0.187471	0.349498	0.044631	0.043337	0.121086	0.200779	0.290315
Gm38712	0.623388	0.202423	0.260984	0.187411	0.09665	0.134067	0.67427	0.60733	0.290164
Zfp937	0.288406	0.383518	0.28844	0.215093	0.373863	0.192623	0.289802	0.289846	0.290148
Auts2	0.235874	0.470493	0.745754	0.605298	0.311603	0.478477	0.251652	0.478128	0.290076
2700046G09Rik	0.43918	0.303238	0.439231	0.302345	0.225223	0.343657	0.209499	0.289483	0.289785
Gm36629	0.556404	0.302685	0.556469	0.352092	0.385391	0.327438	0.209117	0.144478	0.289256
Gm6218	0.606709	0.529457	1.061865	0.603311	0.505595	0.701332	0.156766	0.216618	0.289124
Gm12596	0.563043	0.561544	0.259896	0.387615	0.082498	0.400526	0.313348	0	0.288955
Gm6997	0.075735	0.302134	0.075744	0.075311	0.072129	0.070037	0.704484	0.072107	0.288729
Izumo1r	0	0	0	0.240995	0.057703	0.896479	0.062621	0.057686	0.288729
Gprin3	0.12454	0.177441	0.266902	0.566136	0.423609	0.493588	0.055165	0.186331	0.288266
Scn7a	0.551886	0.761535	0.574633	0.721702	0.381607	0.342575	0.242225	0.633418	0.288217
Gm4478	0.201593	0	0.100808	0.400927	0.095997	0	0.208356	0.095968	0.288204
Gm9828	0	0.301146	0	0	0.057515	0	0.374497	0.057497	0.287785
Gm15728	0.452248	0.150348	0.188459	0.037476	0.107679	0.104556	0.545324	0.035882	0.287355
Glyctk	0.424236	0.259323	0.136866	0.258559	0.377971	0.240454	0.240451	0.104236	0.286947
Cfap73	0.120387	0.120066	0.0602	0	0.22931	0.22266	0.124426	0	0.286848
Gm33667	0	0.150042	0	0.747999	0.07164	0.208687	0	0.071618	0.28677
Pde4c	0.175437	0.699879	0.175457	1.146413	0.525122	0.394008	0.414452	0.143171	0.28664
LOC118568680	0	0	0	0	0	0	0	0	0.28664
Myom3	0.109291	0.0545	0.054652	0.163019	0.065055	0.176871	0.02824	0.039021	0.286452
Gm46411	0.600955	1.348548	1.202049	2.390353	1.144685	0.555744	1.708071	0.858252	0.286382
Gm5841	0.38588	0.29933	0.364484	0.170541	0.183754	0.3172	0.177256	0.040822	0.286049

Gm46828	0.136413	0.244889	0.054571	0.434075	0.207869	0.15138	0.140989	0.363659	0.286029
A630072L19Rik	0.02995	0.059741	0	0.029783	0.228195	0.083091	0.030955	0	0.285453
Cdkl4	0.319298	0.298545	0.359252	0.218288	0.551173	0.406005	0.412513	0.304003	0.285299
Insyn2b	0.361261	0.298534	0.320011	0.769794	0.393212	0.295902	0.682754	0.265337	0.285289
Gm336	0.074815	0.410388	0.411531	0.520773	0.10688	0.069187	0.15465	0.071231	0.285222
Hhatl	0.598252	0.29833	0.365641	0.495751	0.284885	0.614717	0.412216	0.189865	0.285094
Halr1	0.099709	0.198887	0	0.594901	0	0.553245	0.103054	0.094932	0.285094
Maob	0.367748	0.298	0.114935	0.388544	0.394019	0.212551	0.023755	0.437666	0.284779
Sptb	0.21868	0.276612	0.245377	0.233366	0.243827	0.291012	0.25358	0.157423	0.284673
5830454E08Rik	0.671524	0	0.522357	0.074196	0.49743	0.690005	0.462703	1.207674	0.284454
Gm15441	0.298232	0.594877	0.662815	0.395416	0.915218	0.275796	0.068497	0.630992	0.284242
Gm35486	0.107323	0.166502	0.393562	0.189727	0.170355	0.209525	0.246496	0.22707	0.284133
Slc28a2b	0.099351	0.272489	0.34777	0.271686	0.094621	0.160785	0.66745	0.307425	0.284072
Hspb2	0.993514	0.396347	0.69554	0.592769	0.378485	0.735016	0.102685	1.040515	0.284072
Lrp3	0.344725	0.312552	0.235067	0.311631	0.253696	0.449205	0.210535	0.313293	0.283751
Gm9442	0.818549	0.816369	0.967488	0.44398	0.283482	1.238675	0.615281	0	0.283691
Zfp334	0.187254	0.27464	0.132194	0.087626	0.01049	0.08149	0.091076	0.262182	0.283451
Piwil4	0.033019	0.197584	0.066045	0.032834	0.251572	0.030534	0.136505	0.282932	0.283226
Evc2	0.108014	0.161589	0.310576	0.55047	0.295754	0.224747	0.181411	0.424214	0.283103
Gm5867	0	0	0	0.295194	0	0.068631	0.153408	0	0.282931
Frmd5	0.049454	0.246612	0.04946	0.147531	0.659394	0.320135	0.255566	0.235426	0.282805
Upk1a	0.254303	0.591794	0.296721	0.295025	0.403659	0.195976	0.175223	0.322829	0.282769
Car8	0.26352	0.361376	0.592989	2.194619	0.470576	0.974781	0.374497	0.376346	0.282553
Cd19	0.045572	0	0	0.045317	0.260412	0.927154	0.047101	0.151861	0.282321
LOC118567918	0.322653	0.284866	0.32269	0.299806	0.327441	0.386425	0.546685	0.483457	0.28231
Mansc4	0.296196	0	0.098744	0.294537	0.282094	0.273913	0.051022	0	0.282301
Prox1	0.122794	0.208915	0.187825	0.272944	0.089431	0.100197	0.074655	0.116912	0.282258
Pcsk5	1.882904	1.555967	1.452694	0.508211	0.486741	0.621875	0.194607	0.332932	0.282005
Enthd1	0.97571	0.265394	0.70969	0.705632	0.872937	0.73825	0.672297	0.816369	0.281799
Actg2	0.126475	0.084092	0.042163	0.20961	0.281057	0.467839	0.087145	0.561943	0.281264
LOC108167518	0.345725	0.451289	0.411868	0.56624	0.222738	0.35733	0.641082	0.193626	0.28105
Gm12602	0	0	0	0	0	0.034087	0	0	0.28105
Angpt1	0.139603	0.556927	0.310266	0.246794	0.251141	0.172134	0.144287	0.147685	0.280893
Fam169a	0.239433	0.183689	0.165781	0.293037	0.315739	0.153291	0.209395	0.12275	0.280863
Negr1	0.07359	0.097859	0.171731	0.048785	0.116811	0.181477	0.228178	0.256906	0.280553
1700029N11Rik	0.09812	0	0	0.097571	0	0	0	0	0.280553
Palm3	0.362192	0.270921	0.226397	0.202592	0.28027	0.167472	0.046793	0.237079	0.280476
Gm10790	0.359563	0.195603	0.196148	0.390053	0.4981	0.513883	0.945958	0.52907	0.280387
Gm29720	0.294101	0.146659	0.147068	0	0.280099	0.135988	0	0	0.280305
Fndc9	0.122488	0.268756	0.245004	0.341045	0.303306	0.317165	0.430431	0.046648	0.280181
Gm34065	0.065298	0.097686	0.293875	0.097398	0.310946	0	0.269955	0.031085	0.280057
Gng3	0.130519	0.260344	0.391603	0.129788	0.155381	0.18105	0.573317	0.466002	0.279892
Gm10847	0	0.877627	1.173429	0.14584	0	0.135628	0.303164	0.418908	0.279563
Gm15607	1.173292	0.731355	0.293357	1.60424	0	0.135628	0.303164	0.139636	0.279563
Gm40696	0.219925	0.09561	0.265069	0.325236	0.17186	0.135587	0.507062	0.144963	0.279478
Efnb3	0.172436	0.447139	0.448385	0.137176	0.377719	0.271087	0.053466	0.197011	0.279389
E130317F20Rik	0.570573	0.246077	0.400989	0.705387	0.543407	0.370779	0.669406	0.440466	0.279252
Gm16574	1.31821	0.730389	1.245121	1.238003	0.97646	0.474071	0.8326	0.418355	0.279194
Map7	0.219702	0.127818	0.183106	0.49156	0.156931	0.186242	0.283841	0.191746	0.279194
Gm52789	0.182964	0.583926	0.12809	0.309296	0.174253	0.10152	0.359294	0.243879	0.279009
Klhl23	0.204893	0.496271	0.204916	0.844086	0.139384	0.514298	0.090757	0.571299	0.278973
Nap1l5	0.234163	0.379502	0	0.378383	0.055754	0.027068	0.090757	0.390156	0.278973
Tmigd1	0	0	0.058548	0	0	0	0	0	0.278973
LOC115488927	0.251603	0.277819	0.292073	0.446775	0.338041	0.299153	0.673329	0.397826	0.278339

Sptbn2	0.21331	0.190348	0.224563	0.089312	0.203154	0.249173	0.319096	0.256537	0.278204
Gm17999	0.583559	0.679006	1.167253	1.160579	1.296808	1.708913	1.608365	0.185202	0.278092
Scand1	0.437669	0.363753	0.145907	0	0.138944	0.337285	0.150784	0.347253	0.278092
Gm18951	0.485873	0.290747	0.388743	0.434836	0.370192	0.269592	0.602608	0.416338	0.277848
Bmncr	0	0.193832	0	0	0	0	0	0	0.277848
Has1	0.3975	0.872173	0.477056	0.553383	0.656197	0.294077	0.30128	0.353229	0.277826
Gm51685	0.174639	0.7354	0.155252	0.501686	0.221765	0.394779	0.30083	0.314071	0.27741
Rnf225	0.188274	0.273124	0.102707	0.221259	0.195611	0.189938	0.22997	0.260735	0.277319
Gpr174	0.111883	0.033476	0.067138	0.011126	0.213112	0.082773	0	0.042609	0.27725
Tox	0.018176	0.072512	0.090893	0.162671	0.190421	0.218517	0.075145	0.103834	0.27718
Gm16073	0.872277	0.193323	0.290793	0	0.369221	0	1.001713	0.092277	0.277119
Cacnb2	0.18896	0.115974	0.10176	0.101178	0.221494	0.188187	0.165254	0.553565	0.277071
BC031361	0.092463	0.197607	0.409526	0.170755	0.352242	0.158798	0.204782	0.113186	0.276965
B230312C02Rik	0.207478	0.08277	0	0.041263	0.316159	0.268617	0.128663	0.197539	0.276843
LOC118567423	0.290442	0.205182	0.060516	0.108306	0.161358	0.358122	0.100062	0.103698	0.276817
Macroh2a2	0.319277	0.115792	0.464457	0.490664	0.497578	0.053683	0.509982	0.165809	0.276636
Celsr1	0.440059	0.280141	0.327742	0.651736	0.249681	0.199147	0.425792	0.178289	0.276636
Gm18490	0.435377	0	0.072571	0.072156	0.138216	0.268416	0.374987	0.138174	0.276636
Gm46424	0	0.289479	0	0	0	0	0	0	0.276636
Gm19412	0.434808	0.361376	0.724765	1.00887	1.035267	0.603146	0.224698	0.82796	0.276274
Gm7691	0.192828	0.480787	0.771402	0.575244	0.183647	0.089161	0.199297	0.550774	0.275673
Gm16193	0.086758	0.028842	0.433838	0.143786	0.082627	0.240692	0.328784	0.440542	0.275625
Gm6741	0.16524	0.659201	0.661037	0.492944	0.550805	0.764044	0.170784	0.078662	0.275605
LOC118567473	0.358411	0.449704	0.393141	0.298919	0.18719	0.181761	0.26289	0.165117	0.275482
Gm6475	0.192642	0.330889	0.117739	0.202205	0.356747	0.257327	0.387149	0.132466	0.275407
Gm18889	0	0	0	0.287336	0.039314	0	0.042664	0.235811	0.2754
Zmynd10	0.108348	0.396218	0.07224	0.502792	0.412758	0.267192	0.037328	0.034386	0.275374
Gm16793	0.417069	0.383962	0.834234	0.223318	0.519431	0.712047	0.729488	0.794181	0.275195
Olfr1444	0	0.115188	0.404283	0.172274	0.439988	0	0.059685	0.054982	0.275195
Gm19466	0.048082	0.047954	0.072131	0.262967	0.022896	0.066697	0.198779	0.022889	0.274956
Gm41979	0.360221	0.251484	0.540395	0.644766	0.411685	0.133249	0	0.480152	0.274658
Zmiz1os1	0.345768	0.574745	0.43226	0.286526	0.246979	0.026646	0.565833	0.274337	0.274623
Dgkeos	0	0.071806	0	0	0	0	0.148826	0	0.27448
Gm10105	0.215992	0.071806	0	0	0.137139	0	0	0	0.27448
LOC118567640	0.35707	0.482485	0.299513	0.240531	0.1097	0.159778	0.142858	0.219333	0.274451
Gm32404	0.760552	0.57402	0.801756	0.899374	1.076723	0.665316	0.594862	1.056824	0.274276
Gm8652	0.671395	0.621779	0.479624	0.476882	0.730777	0.399141	0.793052	0.365277	0.274243
Gm9619	0.287542	0.344132	0.46012	0.114372	0.109541	0.053182	0.237751	0.219014	0.274053
Nxf2	0.086263	0.143388	0.23006	0.028593	0.328622	0.106364	0.089157	0.054754	0.274053
Gm39747	0.143622	0.572958	0	0	0	0	0	0	0.273769
Zfp13	0.313209	0.20825	0.365453	0.493137	0.795459	1.086173	0.242788	0.223655	0.27364
Ldlrad1	0.035882	0.10736	0	0.107044	0.341739	0.298645	0.037086	0.034163	0.273592
Gm19150	0.236266	0.370286	0.151903	0.268505	0.176799	0.156065	0.627923	0.433829	0.273436
Selenot-ps	0.095583	0.285985	0.047797	0.332666	0.273096	0.265176	0.09879	0.136506	0.273297
Emx2	0.05733	0.228709	0.286683	0.114018	0.491404	0.159051	0.237013	0.382086	0.273203
Gm40543	0.04645	0.084932	0.092911	0.392613	0.302296	0.28637	0.480084	0.213754	0.273004
Gm5582	0.119273	0.071373	0.190859	0.118605	0.113594	0.13236	0.419133	0.272543	0.272826
Peli3	0.51517	1.255953	0.973213	1.166871	0.272579	0.423479	0.650778	0.272496	0.27278
Tex29	0.143066	0.142685	0	0.640191	0.136254	0	0.443597	0.544851	0.272709
Fbxo40	0.480379	0.547543	0.503313	0.216097	0.370364	0.750977	0.330997	0.163346	0.272526
Gm41061	0.071472	0.403927	0.214439	0.118452	0.045379	0.066095	0.196985	0.022683	0.272475
Sds	0.047627	0.285002	0.095265	0.284162	0.226798	0.176177	0.886052	0.362766	0.272358
Robo1	0.146507	0.409126	0.476201	0.473479	0.139531	0.250647	0.083282	0.202258	0.272286
LOC115489399	0.203854	0.162649	0.050969	0.101356	0.378589	0.216795	0.084277	0.184384	0.272007

2310075K07Rik	0.45628	0.142208	1.083792	0.397009	0.353077	0.36921	0.648434	0.705938	0.271797
Hoxc4	0.2444	0.365624	0.122214	0.243031	0.155176	0.67804	0.336799	0.504168	0.271757
Gm18675	0.284785	0.378702	0.094939	0.566379	0.63286	0.26336	0.686791	0.451905	0.271425
Rskr	0.48188	0.458751	0.372405	0.326714	0.438076	0.081023	0.543324	0.354525	0.271389
Smim6	0.568596	0.850623	0.056866	0.904657	1.407963	0.578401	1.527947	0.595495	0.270961
Gm29686	0.398017	1.24758	0.227465	0.226164	0.21661	0.052582	0.587672	0.595495	0.270961
Gm52482	0.689932	0.607143	0.649423	0.242141	0.115956	0.37531	0.209729	0.03864	0.270763
Gm5276	0.202921	0.202381	0.040589	0	0.154608	0.075062	0.167783	0.193201	0.270763
Gm39424	0.047342	0.047216	0	0	0	0	0.146792	0.045075	0.27073
Gm36174	0.141785	0	0	0	0	0.393356	0	0	0.270268
Gm13136	0.134124	0.505343	0.104331	0.444577	0.056773	0.137816	0.138624	0.113511	0.269868
Scml2	0.176892	0.352843	0.424591	0.175901	0.044925	0.076339	0.097508	0.26947	0.269751
Aqp3	0.471679	0.031362	0.660428	0.250153	0.479171	0.378035	0.0325	0	0.269731
Gm18303	0.188565	0	0.047147	0	0.089794	0	0	0.044883	0.269579
Gm39427	0	0	0	0.093754	0	0.087189	0	0	0.269579
4930486L24Rik	0.323019	0.563778	0.646112	0.923476	0.499914	0.298717	0.459052	0.73042	0.269382
Gm3365	0.847821	0.563709	0.329746	1.264608	0.628021	0.914711	0.584177	0.493294	0.26935
Cpxm2	0.164854	0.587197	0.094213	0.398117	0.919602	0.784038	0.413792	0.650251	0.26935
Slc18b1	0.259056	0.657661	0.847919	0.585466	0.403728	0.522692	0.316429	0.201802	0.26935
Gm38521	0.211933	0.352281	0.070652	0.33368	0.269123	0.310315	0.036507	0.622157	0.269321
Gm7384	0.188245	0.281615	0.376533	0.561571	0.448205	0.435207	0.09728	1.075364	0.269121
Gm9079	0.34492	0.625458	0.3136	0.031181	0.179181	0.202982	0.064817	0.268689	0.268969
Plxnb1	0.193839	0.298772	0.334852	0.499407	0.117479	0.268885	0.18213	0.260053	0.268722
Gm18537	1.12679	1.011411	0.112692	0.560239	0.697543	0.625212	0.524067	0.214563	0.268483
Gm5873	0.375533	0.046817	0.469471	0	0.044707	0.303871	0.048517	0.715088	0.268437
Gm42517	0.304991	0.210586	0.117318	0.769872	0.134063	0.47731	0.436464	0.424404	0.268324
Slc16a5	0.562252	0.336453	0.281159	0.223641	0.589031	0	0.464893	0.963574	0.267938
Nbea	0.15332	0.293082	0.319454	0.635256	0.340715	0.36628	0.356544	0.218964	0.267901
Snhg7os	0	0	0	0	0	0	0.072621	0.066898	0.26787
Prss50	0.160574	0.720658	0.20074	0.159674	0.152929	0.074247	0.08298	0.076441	0.267822
Il12a	0.140468	0.046698	0.046828	0.232802	0.401341	0.216501	0.096787	0.312059	0.267757
Ephb3	0.361116	0.280121	0.668812	0.319194	0.152855	0.284475	0.193528	0.165542	0.267692
Masp1	0.280801	0.280053	0.200595	0.718014	0.744988	0.482255	0.352412	0.725663	0.267628
Gm40916	0	0.046659	0.233943	0.046521	0.534669	0.346109	0.096705	0.267253	0.267531
Fam124a	0.389641	0.419692	0.218224	0.604434	0.519525	0.490045	0.515473	0.534205	0.267381
Zfp607b	0.353652	0.340548	0.451264	0.436558	0.150986	0.360879	0.390726	0.348322	0.267325
A930004D18Rik	0.37395	0.093239	0.373993	1.301493	0.356146	0.518726	0	0.089009	0.267306
A230103J11Rik	0.342595	0.093186	0.21804	0.278735	0.296621	0.172811	0.482848	0.029653	0.267155
Tceal3	0.560547	0.559054	0.280306	0.222963	0.373701	0.051838	1.042835	0.266848	0.267125
Gm17230	0.280273	0.15374	0.350382	0.292639	0.026693	0.038878	0.159322	0.133424	0.267125
Dclk2	0.215377	0.386647	0.387724	0.556844	0.779467	0.278844	0.823631	0.635686	0.266856
Rpl21-ps13	0.18666	0.465408	0	0.092807	0.088887	0.086309	0	0.177719	0.266856
Gm13427	0	0.349056	0.140011	0.278422	0.533319	0	0.144692	0.066645	0.266856
Gm8934	0	0.167378	0.111896	0	0	0.051733	0.115637	0.213047	0.266586
Klra14-ps	0.069909	0.20917	0.069917	0.139035	1.131874	0.32325	0.867057	0	0.266519
LOC118567856	0.136827	0.191628	0.230012	0.726619	0.302213	0.212683	0.463367	0.354784	0.266365
4732491K20Rik	0.349326	0.400656	0.471645	0.173685	0.182982	0.323046	0.23468	0.432371	0.266351
Gm6710	0.239473	0.19903	0.838254	0.119066	0.152048	0.479824	0.99003	0.380003	0.266279
Pla2g4c	0.246464	0.327744	0.098597	0.196067	0.125189	0.288702	0.067929	0.015644	0.266223
Gm8850	0.418927	0.139271	0	0.416581	0	0	0.721637	0.066477	0.266183
1700026J14Rik	0	0	0	0	0	0	0	0	0.266183
LOC118568447	0.139642	0.557083	0	0.277721	0	0.129137	0	0	0.266183
5033417F24Rik	0.062046	0.061881	0.403346	0.40104	0.295459	0.286891	0.128255	0.354443	0.266109
Ms4a1	0.037219	0.03712	0.241953	0.074022	0.24813	0.516288	0.28851	0.035436	0.266049

Cachd1	0.122799	0.178141	0.223296	0.22202	0.255168	0.3923	0.161533	0.148803	0.265996
Phf11c	0.348667	0.104322	0.104612	0.346714	0.132827	0.128975	0.036036	0.033197	0.265849
4921528I07Rik	0.167318	0.166873	0.055779	0.332762	0.663966	0.025788	0.115288	0.185854	0.265782
Gm41142	0	0.111248	0	0	0.053117	0.051577	0.115288	0.159303	0.265782
Gm32312	0.025349	0.151689	0.050704	0	0.24142	0.046884	0.026199	0.193077	0.265757
Gm38878	0.278817	0	0.046475	0	0.132771	0.042973	0	0.353948	0.265737
Adm	0.159076	0.237979	0.715926	0	0.037876	0.147109	0.24662	0.37864	0.265324
Gm17121	0.445286	0.055513	0.055667	0	0.265053	0.15442	0.057528	0	0.265248
Gm13363	0.139092	0.118904	0.238471	0.098795	0.246015	0.165379	0.246443	0.24594	0.265134
Gm18868	0.339921	0.246557	0.27815	0	0.058861	0.114309	0.383264	0.411903	0.265071
Slc22a1	0	0	0	0	0	0	0	0	0.264982
Necab2	0.19856	0.039606	0.11915	0	0	0.036724	0.041044	0.151239	0.264944
Gm9365	0.185102	0.276914	0.185124	0.552196	0.440723	0.171177	1.626154	0	0.264628
Mageh1	0.555174	0.118649	0.277619	0.74923	0.113302	0.733439	0.122957	0.302046	0.264565
Aicda	0.555074	0.461331	0.323831	0.16099	0.374458	0.171105	0.382465	0.616566	0.264518
Atrip-trex1	0.158583	0.237242	0.185035	0.302249	0.264308	0.403295	0.423418	0.176151	0.264502
Kcnrg	0.123333	0.430515	0	0.67453	0.79286	0.057027	0.54175	0.587124	0.264481
Gm36505	0	0	0.138611	0	0	0	0.143245	0	0.264187
Gm40454	0.13848	0.046037	0.230826	0.68852	0	0.042687	0.524794	0.395539	0.263967
Gm6610	0.519083	0.172567	0.657582	0.860292	0.527327	0.384025	0.608031	0.263583	0.263857
4833411C07Rik	0.184486	0.183995	0.876411	0.642084	0.527108	0.085303	0.333682	0.175649	0.263747
Gm7862	0.184486	0.321991	0.046127	0.321042	0.175703	0.341214	0.143007	0.263473	0.263747
Gm40150	0.760689	0.505776	0.922154	0.504285	0.307352	0.362389	1.191227	0.373099	0.263638
Gm8927	0.092166	0.091921	0.092177	0.137475	0.087778	0.298314	0.190517	0	0.263528
Steap2	0.15586	0.251103	0.311755	0.131142	0.057092	0.277181	0.08674	0.205468	0.262816
Plekha7	0.487601	0.148005	0.275632	0.611357	0.363432	0.646969	0.306758	0.726642	0.262672
Car14	0.367139	0.823865	0.275387	0.182541	0.611904	0.08488	0.948642	0.873882	0.262437
Adamtsl2	0.525361	0.723567	0.07506	0.447787	0	0.208216	0.129283	0.19055	0.26228
Gm18648	0.314319	0.078371	0.550123	0.586047	0.224516	0.327007	0.568513	0.037408	0.262127
Gm21992	0.068725	0.228473	0.137466	0.13668	0.218176	0.254218	0.260445	0.370786	0.262004
LOC118567848	0.045817	0.137084	0.183288	1.138998	0.392717	0.04237	0	0.218109	0.262004
D930048N14Rik	0.129339	0.306363	0.355724	0.401921	0.384942	0.313973	0.200518	0.307859	0.261953
Gm4827	0	0.068514	0.068704	0	0	0	0	0	0.261895
Mypn	0.45441	0.411042	0.454463	0.294238	0.382453	0.742722	0.349511	0.291783	0.26187
Gm20560	0.396858	0.57848	0.244249	0.516062	0.145371	0.254078	0	0.319718	0.261859
Gm46914	0.384605	0.252068	0.27475	0.262252	0.313966	0.223564	0.726873	0.324332	0.261831
Unc13b	0.354463	0.609821	0.540619	0.99575	0.413544	0.401551	0.485421	0.312173	0.261822
Nkd1	0.251679	0.547656	0.251708	1.069333	0.849833	0.846346	0.070943	0.914925	0.261679
Gm29718	0.137223	0.205286	0	0.136454	0	0	0.070913	0.13065	0.261571
Itga2b	0.500023	0.595213	0.354896	0.57742	0.706644	0.969561	1.06694	0.368571	0.261343
Ppp1r2-ps4	0.171317	0.102516	0.137069	0.681428	0.358952	0.190114	0.212477	0.260976	0.261248
Myo3b	0.20558	0.410065	0.274138	0.272571	0	0.316857	0	0	0.261248
Gm8825	0.182662	0.091088	0.228355	0.181639	0.130474	0.337841	0.047198	0.086956	0.26114
Apol7b	0	0.049655	0.024897	0	0	0.046042	0	0	0.260984
Gm34836	0.684421	0.13652	0.1369	0.340293	0.456284	0.506345	0.636644	0.651635	0.260925
Pard6g	0.318871	0.045432	0.296129	0.294436	0.130153	0.315945	0.211866	0.151798	0.260497
Pxt1	0.109238	0.544734	0.327751	0.054313	0.728257	0.505097	0.225805	0.104005	0.260283
Gm52594	0.273094	0.272367	0	0.271564	0	0	1.129025	0.780036	0.260283
Gm26448	0.273094	0.817101	0.273126	1.086257	1.040367	0.252549	0.564512	1.040049	0.260283
Gm52060	0	1.361834	0	1.086257	0.520183	0.252549	0.282256	0	0.260283
Gm15644	0.409641	0.136183	0.273126	0.543128	0.130046	0	0.141128	0.390018	0.260283
Gm29967	0.546188	0	0	0	0	0	0	0	0.260283
Nptx1	0.230817	0.062783	0.062957	0.020866	0.059953	0.145536	0.032531	0.059935	0.259987
Gm5979	0.136379	0.34004	0.068197	0.610268	0.259772	0	0.563818	0	0.259963

Fez1	0.077917	0.155419	0.155852	0.736067	0.371037	0.288221	0.281859	0.14837	0.259917
Mpv17l	0.326961	0.5616	0.290666	0.361255	0.380593	0.251969	0.619539	0.103766	0.259686
LOC118568370	0.262435	0.128399	0.163422	0.15264	0.226361	0.087003	0.087001	0.259292	0.259562
Gm51797	0.041887	0.20888	0.293246	0.249917	0.578451	0.348626	0.259757	0.358929	0.259496
Apol6	0.054431	0.162859	0.190531	0.108252	0.02592	0.251681	0	0.025912	0.259388
Gm11970	0.068022	0	0	0	0	0	0.070304	0.129528	0.259325
Pitx1	0.271876	0.246502	0.074157	0.344086	0.023539	0.342849	0.025545	0.094128	0.259122
Gm4544	0	0.045172	0.045298	0.270233	0.043136	0.335081	0	0.043123	0.259007
Gm45989	0.111835	0.350545	0.207717	0.095321	0.258668	0.162519	0.330248	0.167322	0.258858
Gm11185	0.316789	0.406216	0.407347	0.405019	0.517211	0.502211	0.327417	0.258526	0.258795
Gm17823	0.090511	0.18054	0.362087	0.180008	0.603413	0.627764	0.093548	0.473965	0.258795
Cstad	0.067856	0.13535	0	0	0.12925	0.251003	0	0.193816	0.25869
Fam83h	0.172551	0.307306	0.505386	0.232864	0.352147	0.113978	0.114647	0.164285	0.258431
4930595D18Rik	0.474409	0.135185	0.203342	0.539144	0.129092	0.188022	0.280186	0.064526	0.258374
Gm9237	0.271091	0	0.271122	0	0	0	0	0.51621	0.258374
Gm46496	0.054192	0.270237	0.108396	0.916098	0.258058	0.651492	0.280049	0	0.258247
Gm5122	0.033866	0.202653	0.101609	0.606167	0.19352	0.125272	0.350018	0.644868	0.258216
Kcnh4	0.367652	0.289479	0.32899	0.019242	0.239575	0.071578	0.479983	0.018423	0.258193
Mroh2a	0.334636	0.174819	0.143432	0.285224	0.16694	0.058945	0.329394	0.227576	0.258188
Gm9027	0.104168	0.270115	0.125016	0.248602	0.218258	0.115597	0.17226	0.17852	0.258131
Gm31623	0.433163	0.519761	0.32491	1.238367	0.438323	0.30669	1.210176	0.399525	0.258027
Gm31725	0.216502	0.364375	0.189461	0.228745	0.347953	0.187701	0.237751	0.077299	0.257932
Prickle1	0.202872	0.647461	0.229948	0.457267	0.437949	0.375219	0.223657	0.412061	0.257806
Gm19585	0.162258	0	0	0.215132	0.103022	0.600204	0.223602	0	0.257743
Gm36163	0	0.134855	0.067615	0	0	0	0.559005	0	0.257743
Gm7129	0.225358	0.089903	0.135231	0.313734	0.128777	0.208404	0.139751	0.17165	0.257743
Gm32605	0.207945	0.165913	0.27036	0.682374	0.613937	0.423062	0.601779	0.514758	0.257647
Tmeff1	0.40548	0.359467	0.157705	0.336007	0.36472	0.354143	0.256106	0.514742	0.257639
Gnb3	0.090094	0.029952	0.030035	0.238906	0.028602	0.111089	0.279351	0.057186	0.257604
Gm8565	0.54042	0.314406	0.450402	0.537393	0.343127	0.291529	0.418913	0.343022	0.257534
Gm35551	0.179994	0.31415	0.315026	0.044746	0.342848	0.249679	0.093016	0.428429	0.257325
LOC118568201	0.280504	0.307733	0.354177	0.212686	0.3139	0.155641	0.44212	0.310465	0.25732
P2rx6	0.171787	0.195805	0.441789	0.463667	0.210353	0.204253	0.355101	0.373847	0.257287
Colca2	0.485825	0.053837	0.053987	0	0	0.349437	0.390541	0	0.257241
Fbln7	0.925253	0.634418	0.308453	1.265096	0.771051	0.944774	0.617607	0.80752	0.257206
Gm30321	0.141227	0.140851	0.077042	0.229804	0.036683	0.154348	0.384818	0.122238	0.256967
4930533B01Rik	0.915886	0.322393	1.454812	1.285773	0.718348	0.049823	0.556832	0.359064	0.256741
Gm6039	0.345887	0.191648	0.4228	0.171975	0.439226	0.319866	0.178746	0.256137	0.256403
Gm8817	0.179121	0.535933	0.358284	0.089059	0	0.496937	0.462827	0.085271	0.256078
Gm10767	0.100674	0.133875	0.16781	0	0.127842	0.062067	0.17342	0.191704	0.255871
Gm19178	0.536497	0.356713	0.208662	0.414938	0.227091	0.35832	0.492886	0.340532	0.255665
Gm9316	0.156478	0.089178	0.20121	0.155602	0.04258	0.248068	0.277248	0.042567	0.255665
Gm51605	0.063869	0.178356	0.114977	0.139724	0.133821	0.118128	0.132023	0.364856	0.255665
Gm15283	0.142836	0.160262	0.696404	0.15979	0.323083	0.280691	0.442883	0.526975	0.255253
Cxcl5	0.703019	0.367268	0.033481	0.299606	0.350715	0.030959	0	1.115572	0.255253
Zkscan7	0.221147	0.406292	0.814847	0.462965	0.487747	0.387492	0.336835	0.387862	0.255146
Rgs9	0.0669	0.088963	0.178422	0.066526	0.084954	0	0.13829	0	0.255048
Gm32954	0.107023	0	0.374625	0.798178	0.407711	0.197944	0.940218	0.356638	0.255007
Fam166b	0	0.053369	0	0	0	0.197944	0.331842	0.254742	0.255007
9630015K15Rik	0.089129	0	0	0.088629	0	0	0.460595	0.933452	0.254843
Gm39011	0.133586	0	0	0	0	0	0.138067	0.127187	0.254638
Csdc2	0.15585	0.35528	0.155868	0.287814	0.127226	0.617679	0.18409	0.169582	0.254638
Ccdc73	0.182732	0.266359	0.421738	0.265574	0.120484	0.246978	0.145279	0.080298	0.254541
LOC118568335	0	0.443744	0.088996	0	0.508494	0.329165	0.183942	0.423615	0.254434

Dbx1	0.026691	0.02662	0.160167	0	0	0.098734	0	0.025413	0.254393
Gm35596	0.533486	0.532065	0.133387	0.530497	0.719789	0.123338	0.781128	0.846551	0.25423
Myrip	0.315127	0.096704	0.024243	0.626723	0.25395	0.201752	0.050108	0.069238	0.254137
Fndc4	0.506405	0.478475	0.079968	0.848116	0.96459	0.690138	0.19283	0.964295	0.254026
Cfap299	0.066552	0	0	0	0	0.123091	0	0	0.253721
Klra1	0	0.033168	0.03326	0.06614	0.126691	0.092263	0.137488	0.126653	0.253569
1810013A23Rik	0.53178	0.265182	0.13296	0	0.253231	0.245887	0.824432	0.37973	0.253417
Gm51445	0.398596	0.16564	0.132881	0.132121	0.06327	0.184305	0.411969	0.284627	0.253265
Gm19103	1.15127	1.059881	2.745654	2.377705	0.253028	0.327587	1.739077	0.758853	0.253214
Gm33481	0.531355	0.883234	0.531417	0.792568	0.506057	0.163794	1.281425	0.252951	0.253214
LOC118567454	1.326269	0.264548	1.591708	3.428985	0.252625	0.981194	3.289838	1.262738	0.252811
Fzd8	0.165685	0.231341	0.4474	0.37894	0.331372	0.980607	0.308238	0.347046	0.252659
Gm32048	0	0.158577	0	0.263516	0.15143	0	0.493004	0.504613	0.252569
Gm28729	0.066234	0	0.033121	0.032932	0.189242	0.030626	0.102685	0.031531	0.252508
Gm39839	0.088277	0.264126	0.706299	0.351131	0.084074	0	0.182478	0.168097	0.252408
Gm36377	0.044103	0.087972	0.088217	0	0.294026	0.203928	0.091166	0.167963	0.252207
Trgv4	0	0	0	0	0	0	0	0	0.252207
4930455G09Rik	0.352547	0.615315	0.705177	0.438216	0.083941	0.081506	0.72875	0.083915	0.252007
Hsbp1l1	0.132205	0.263707	0.352588	0.262929	0.335762	0.448284	0.501016	0.209787	0.252007
Hoxd8	0.132153	0.527204	0.16521	0.197119	0.534908	0.45829	0	0.2831	0.251907
C230072F16Rik	0.087997	0.043881	0.132011	0.43752	0.46094	0.081377	0.409271	0	0.251607
Tmem100	1.055963	0.677026	0.641195	1.08755	1.149357	0.767267	0.389782	0.466784	0.251607
Lrriq3	0.065998	0.164555	0.033003	0.196884	0.094283	0.061033	0.204636	0.157091	0.251607
Cfap99	0.150852	0.15045	0.301739	0.150007	0.14367	0.139503	0.194891	0.071813	0.251607
Ccdc85a	0.58078	0.21063	0.330027	1.010671	0.477702	0.793424	0.122781	0.251345	0.251607
Gm2178	0	0	0	0	0	0	0	0	0.251607
Gm14148	0.439636	0.219233	0.351749	0.174869	0.125611	0.121968	0.272631	0.376719	0.251407
LOC115486538	0.353109	0.579518	0.321858	0.533363	0.366095	0.351345	0.466589	0.276615	0.251343
Tnik	0.203772	0.20323	0.323676	0.774765	0.342477	0.421224	0.39644	0.262486	0.251335
Gm36287	0.246977	0.114949	0.098802	0.11461	0.047044	0.167491	0.238245	0.031353	0.251084
Gm17849	0.395046	0.328328	0.395091	1.571331	0.627062	0.304438	0.340249	0.125374	0.251009
Pjvk	0.065841	0	0.065849	0.392833	0.062706	0.608876	0	0.501496	0.251009
Adcy2	0.07899	0.1313	0.197499	0.248735	0.062691	0.243493	0.027213	0.200551	0.250949
6430590A07Rik	0.789716	0.21003	0.157962	0.052353	0.150423	0.438183	0.054414	0.050126	0.25089
Gm33504	0.350874	1.312274	0.087729	0	0.584795	1.13567	0.090661	0.334066	0.25081
Gm11764	0.065763	0	0.06577	0	0.125264	0.060815	0.203907	0	0.250711
Lrrc7	1.226764	0.582618	0.817938	0.697081	0.361634	1.053439	0.483018	0.02781	0.250546
Gm15345	0.367835	0.209632	0.367878	0.888311	0.400369	0.242973	0.651731	0.050031	0.250414
LOC115486431	0	0	0	0	0	0	0.271555	0	0.250414
Gm31172	0.087534	0.232802	0.262632	0.754377	0.333465	0.080949	0.57298	0.194462	0.250282
B230319C09Rik	0.459522	0.294621	0.032827	0.130557	0.250082	0.030354	0.407091	0.093752	0.250266
Baiap3	0.131285	0.202355	0.035809	0.261099	0.193235	0.353188	0.061677	0.181813	0.250252
Cacng8	0.130997	0.287427	0.078608	0.234474	0.374281	0.387655	0.406177	0.274389	0.249704
Nxpe2	0.065491	0	0	0.097686	0.124746	0	0	0.218238	0.249675
Lmod3	0.340432	0.652934	1.021415	0.468727	0.473866	0.96868	0.351854	0.498653	0.249586
Pgap4	0.494463	0.580173	0.669058	0.491694	0.415519	0.51106	0.360743	0.332314	0.249495
Gm3052	0.187926	0.301219	0.134248	0.113459	0.204547	0.235855	0.430082	0.332287	0.249474
Hcn3	0.174471	0.152255	0.109057	0	0.041541	0.221849	0.405729	0.062292	0.249429
Gm41884	0.130827	0.326197	0.392527	0.195142	0.467244	0.332709	0.709887	0.467101	0.24938
Gm40855	0.2615	0.65201	0.653827	2.730373	0.24905	0	0.135137	0.248974	0.249233
Gm42222	0.174334	0.086935	0.348708	0	0	0	0	0	0.249233
1700020N01Rik	0	0.104223	0	0.727413	0.149289	0.241599	0.054004	0.198991	0.248998
Gm39783	0.156605	0.416501	0.313246	0	0.149149	0.434469	0.37767	0.248505	0.248764
Gucy1a2	0.189775	0.212929	0.166073	0.990739	0.406664	0.197435	0.44132	0.271027	0.2487

Gm14472	0.173923	0.043365	0.130458	0	0	0.160839	0.404457	0.041398	0.248647
Gm19439	0.052152	0.208054	0	0.207441	0.049669	0.289374	0.431217	0.446888	0.24853
Gm39575	0	0	0	0.086407	0.082756	0	0.269426	0.330925	0.248452
Gm39092	0	0.086662	0.347614	0	0.082756	0.321426	0.179618	0.165462	0.248452
Gm2389	0	0.037133	0.148944	0.444278	0.035459	0.034431	0	0.035448	0.248396
E130201H02Rik	0.065145	0	0	0.06478	0.062043	0.120487	0.403981	0	0.248354
Gm39913	0.613911	0.426739	1.004699	0.536476	0.230329	0.36128	0.999831	0.407381	0.248229
Magix	0.100141	0.279648	0.340519	0.338572	0.228896	0.500079	0.124201	0.209757	0.248153
Gm34319	0	1.817058	2.342726	0.517629	2.478808	1.444152	2.152038	0.99122	0.248063
4931403G20Rik	0.097602	0.064895	0.227765	0.161759	0.185911	0.180519	0.269005	0.123903	0.248063
Gm30240	0.260217	0.283117	0.248418	0.45871	0.101384	0.065629	0.158923	0.38289	0.24801
Gm6409	0	0	0	0	0	0	0	0	0.247772
A430057M04Rik	0.27135	0.200029	0.188787	0.492734	0.168542	0.229116	0.353616	0.146025	0.247377
Gm13586	0.172974	0.25877	0.086497	0	0.247107	0.07998	0.446942	0.494064	0.247289
Gm7240	0.424271	0.129293	0.294667	0.421894	0.246932	0.130784	0.35324	0.190753	0.247114
Crybg2	0.235656	0.4113	0.412446	0.515539	0.168327	0.348684	0.572371	0.302896	0.247061
Dbx2	0.032382	0.129183	0	0.064401	0.03084	0	0.066937	0	0.246904
LOC118568076	0.051787	0.142035	0.064741	0.193114	0.184955	0.179591	0.347909	0.110939	0.246788
Ldhb-ps	0	0	0.129442	0.128702	0.123265	0.079794	0	0.205379	0.246712
Creb3l3	1.092659	0.774296	1.006513	0.45749	1.177562	0.611592	0.653815	0.76655	0.246648
Gm41461	0.279296	0.251154	0.212931	0.341474	0.109016	0.15243	0.307596	0.217965	0.246557
Gm6637	0.036909	0.110434	0.036914	0.550541	0.210913	0.477858	0.114443	0.281132	0.246246
Gm33864	0.258332	0.309173	0.051672	0.308262	0.492065	0.095559	0.480598	0.541106	0.246213
Gpr150	0.258332	0.669875	0.387543	0.770655	0.295239	0.167228	0.293699	0.196766	0.246213
Fscn2	0.258152	0.225281	0.129091	0.160441	0.184396	0.179048	0.200109	0.399402	0.246041
Aff3	0.349999	0.284766	0.386886	0.522058	0.543863	0.621784	0.314144	0.359542	0.245796
Il31ra	0.429752	0.400034	0.229228	0.398855	0.218289	0.079484	0.384948	0.2455	0.245755
Snord3b2	0	0	0.257881	0.256407	0.49115	1.669171	0.266502	0.981999	0.245755
Chrnb2	0.329866	0.544886	0.443309	0.574031	0.500693	0.71496	0.447474	0.255177	0.245618
Cdnf	0.257595	0.311961	0.809677	0.567193	0.490661	0.068062	0.399355	0.420438	0.245511
Rtn4r	0.486299	0.85589	0.257482	0.170673	0.217951	0.42326	0.206959	0.190649	0.245375
Gm18733	0.051427	0.359029	0.360029	0.051139	0.048978	0.618252	0.106304	0.685487	0.245071
Ap3m1-ps	0.231313	0.230697	0.334158	0.076672	0.048956	0.190143	0.239074	0.02447	0.244958
Ror1	0.488328	0.358863	0.668316	0.51115	0.6609	0.499126	0.053127	0.489406	0.244958
Gm30601	0	0	0	0	0	0	0	0	0.244844
Rnf43	0.135952	0.210917	0.287042	0.180253	0.230185	0.223509	0.359088	0.071911	0.244751
Olfr369-ps1	0.183327	0.255974	0.018335	0.1823	0.122219	0.169535	0.227373	0.069818	0.244618
Gm17826	0	0.255974	0	0	0.061109	0.356024	0.066317	0.183272	0.244618
Rnase10	0.036641	0	0	0	0	0.033885	0	0	0.244456
Gm30149	0.192271	0.958794	0.384586	2.166862	0.54935	0.414881	1.126088	1.586525	0.244335
Gm39286	0.17071	0.404357	0.234753	0.403165	0.406456	0.157867	0.264656	0.751713	0.244053
Mirg	0.554381	0.297718	0.170598	0.551275	0.406143	0.473237	0.132226	0.365417	0.243865
Gm15038	0.189467	0.396822	0.34108	0.367391	0.50525	0.175213	0.205615	0.19843	0.243782
Tmem25	0.255582	0.370766	0.697123	0.25415	0.221285	0.107434	0.1681	0.199096	0.243592
Gm39595	0.255475	1.273974	3.577065	0.254044	0.973246	0.236255	2.112369	1.216186	0.24349
Zfp385c	0.333846	0.352543	0.294604	0.371032	0.56109	0.181606	0.466828	0.317854	0.243318
Camsap3	0.254888	0.32684	0.309542	0.12673	0.104037	0.319895	0.338707	0.20801	0.242931
Taco1os	0.181883	0.03628	0.036381	0.25321	0.034645	0.23548	0.075194	0	0.242691
Nrn1	0.036377	0.362798	0.109143	0.578767	0.277158	0.23548	0.037597	0	0.242691
Tyro3	0.179653	0.179175	0.10481	0.208422	0.25665	0.443034	0.340415	0.256572	0.24257
Themis	0	0.18128	0.218142	0.036149	0.069244	0.134472	0.30058	0.034611	0.242532
Gm14052	0.101721	0.659428	0.101733	0.910363	0.242195	0.423309	1.156473	0.048424	0.242373
1810009A15Rik	0.084768	0	0.508665	0	0.322927	0.235171	0.876116	0.161414	0.242373
Pglyrp2	0.108987	0.036232	0.072666	0.036126	0.276795	0.436747	0.262835	0.345888	0.242373

4933423K11Rik	0	0.084542	0	0.084293	0.484391	0.313562	0	0	0.242373
Adamts20	0.084681	0.056304	0.056461	0.757863	0.67208	0.182725	0.262567	0.026875	0.242127
Kank4	0.173208	0.483691	0.08084	0.298545	0.351917	0.459175	0.226757	0.29684	0.242121
4930403D09Rik	0.635029	0.570005	0.762124	1.894417	0.241918	0.52853	0.721968	1.330142	0.242096
Col4a5	0.394047	0.436665	0.350305	0.618236	0.183473	0.396794	0.253411	0.250114	0.242029
Gm52456	0.296234	0.042206	0.042324	0.168328	0	0.117406	0.174956	0	0.242003
Yipf7	0.20307	0.101265	0.15232	0.454348	0.531854	0.046948	0.629648	0	0.241929
LOC118568481	0.126861	0.44283	0.190313	0	0.362462	0.293292	0.262233	0	0.241819
Foxs1	0.634303	0.463917	0.253751	0.71485	0.402735	0.547478	0	0.724702	0.241819
Gm41348	0.095091	0.316126	0.348708	0.126078	0.392442	0.058625	0.294844	0.030179	0.24168
Gm40116	0.084509	0.168569	0.253557	0.168072	0.080486	0	0.349378	0.160922	0.241634
Scube3	0.65877	0.101079	0.354764	1.360551	0.820454	0.093725	0.157124	0.482472	0.241487
4931428L18Rik	0	0.101079	0	0	0.048262	0.140587	0	0	0.241487
Olfr78	0.126619	0.231517	0.126634	0.33576	0.221083	0.429342	0.021811	0.381754	0.241359
Gm35177	0	0.084156	0	0.083908	0	0	0.174423	0.160677	0.241267
Lmod1	0.281269	0.21039	0.014065	0.447509	0.401816	0.572239	0.101747	0.187457	0.241267
Gm13252	0.168633	0.546598	0.084326	0.167688	0.080302	0.038987	0.174291	0.200694	0.241083
Gm32316	0.063213	0.063045	0.063221	0.062859	0	0	0	0	0.240992
Fam160a1	0.227412	0.151205	0.151626	0.577909	0.024065	0.245354	0.156695	0.096231	0.240827
Txk	0.075804	0.126004	0.151626	0.276391	0.33691	0.56081	0.052232	0.625499	0.240827
Gm6520	0.252565	0.125946	0.505189	0	0	0.116782	0.130519	0	0.240717
Ntf3	0.168377	0.083964	0.294694	0.25115	0.681531	0.272492	0.043506	0.681323	0.240717
Gm7531	0.252565	0	0	0	0.060135	0	0	0	0.240717
Gm38978	0.060102	0.035965	0.060109	0.358592	0.068689	0.011116	0.111813	0.034334	0.240587
Gm9333	0.839971	0.837734	1.176096	0.709976	0.599984	0.543745	0.737929	0.559814	0.24017
Gm46916	0.213149	0.096628	0.174415	0.192686	0.073818	0.197113	0.300409	0.147592	0.240086
Pde10a	0.135593	0.212507	0.135609	0.30819	0.064569	0.25974	0.170172	0.156761	0.240002
Gm8318	0.40282	0.451966	0.30215	0.250352	0.143865	0.512208	0.520418	0.767048	0.239952
Gm29897	0.125853	0.062759	0	0	0	0	0	0	0.239897
1700055D18Rik	0.287616	0.39442	0.647212	0.464759	1.02721	0.764689	0.966114	0.547678	0.239858
Dnmt3l-ps1	0.201227	0.150519	0.654064	0.450225	0.095823	0.046522	0.363962	0.095794	0.239734
Rpl30-ps11	0	0.752251	0.251449	0.250011	0.359174	0.581263	0.51971	0.119688	0.239625
Gm19249	0.12571	0	0.502898	0.500023	0.718348	0.930021	0.129927	1.196881	0.239625
Gm39459	0	0	0.062862	0.062503	0	0	0	0	0.239625
Gm9174	0	0	0	0.187509	0.299312	0.116253	0	0.239376	0.239625
Gm8162	0.50246	0.668163	0.167506	0.333097	0.717806	0.464659	1.557952	0.478391	0.239444
Bean1	0	0.02784	0.223341	0	0.026585	0.464659	0	0	0.239444
Smarca5-ps	0.251185	0.147363	0.177328	0.323242	0.070361	0.150304	0.183256	0.098475	0.239402
Gm5910	0.215293	0.250507	0.322978	0.214087	0.341739	0.199097	0.037086	0.10249	0.239393
Fbp2	0	0.083457	0.08369	0	0	0.038692	0	0	0.239264
Adam1a	0.251013	0.536453	0.197248	0.142633	0.170759	0.315032	0.35209	0.290201	0.239238
Gli3	0.167206	0.101909	0.269419	0.461859	0.336184	0.051542	0.153614	0.380303	0.239043
Svep1	0.668699	0.559034	0.536005	0.782297	0.435501	0.486529	0.457368	0.51027	0.238998
Ccdc149	0.033427	0.100013	0.016715	0.349014	0.175093	0.293663	0.034548	0.270516	0.238939
Gm4319	0.175437	0.324944	0.350915	0.049844	0.358038	0.023177	0.621678	0.143171	0.238867
Gm32778	0.116958	0.183302	0.083551	0.215991	0.127302	0.231769	0.207226	0.159079	0.238867
Gm10851	0.438493	0.749701	0.125298	0.560619	0.059659	0.057929	0	0.119282	0.238813
Sh3tc2	0.088418	0.088183	0.07369	0.219807	0.014035	0.258926	0.060923	0.126274	0.238765
H3c14	0.417455	0.749419	0.334003	1.079304	0.318064	0.30884	0.690337	0.317967	0.238723
5430400D12Rik	0.260226	0.48912	0.310305	0.049763	0.162047	0.222137	0.1862	0.114351	0.238479
D430001F17Rik	0.270938	0.498861	0.562783	0.393768	0.079396	0.096367	0.236947	0.396861	0.238364
Raver2	0.111154	0.166287	0.083375	0.082899	0.251422	0.334073	0.172325	0.277802	0.238364
Rps2-ps9	0.750008	0.187003	0.187524	0.683656	0.297625	0.346792	0.904365	0.535561	0.238275
Gm7466	0.125001	0.249337	0.312539	0.062151	0.178575	0.057799	0.064598	0.17852	0.238275

Slc4a8	0.27397	0.345146	0.274002	0.215081	0.247193	0.41782	0.407355	0.237965	0.238213
Ctxn3	0.499442	0.871697	0.624375	0.496644	1.070242	0.115467	0.645248	0.118879	0.238006
Gm43116	0.749163	0.249056	0.24975	0	0	0	0.516198	0	0.238006
Klrc3	0.08324	0.083019	0.08325	0.744967	0.951326	1.000715	0.086033	0.475518	0.238006
Gm11957	0	0.031097	0.062367	0.124021	0.059391	0.028834	0.128904	0	0.237739
Rpl29-ps2	0.249347	0.911837	0.831252	0.08265	0.47495	1.306665	0.343616	0.395671	0.237649
D6Ertd527e	0.274199	0.024861	0.249301	0.619689	0.308625	0.276623	0.231871	0.498396	0.237578
Tgfb2	0.162543	0.140496	0.596061	0.323265	0.433452	0.120252	0.167996	0.237294	0.237541
Gm4876	0.398656	0.472143	0.373783	0.668963	0.118649	0.207374	0.412031	0.237225	0.237471
4931403E22Rik	0.165982	0.413851	0.332003	0.66021	0.237119	0.613981	1.029305	0.790157	0.237294
Tubb1	0.055327	0.05518	0.083001	0.137544	0.184426	0.127913	0.171551	0.105354	0.237294
Arpp21	0.319835	0.921508	1.31503	0.247367	0.439988	1.117366	1.065155	1.894755	0.237091
Gm32234	0.235359	0.261305	0.253151	0.512239	0.198777	0.135519	0.523228	0.240997	0.237015
Cyp26a1	0	0.07084	0.355186	0	0.067647	0.032843	0.036706	0.169066	0.236939
Rnf32	0	0.206616	0.041438	0	0.236765	0	0	0	0.236939
Gm11346	0.198703	0.544978	0.546496	0.049397	0	0.275631	0.102685	0.283777	0.236727
Gm38728	0.139682	0.108352	0.031044	0.200632	0.103469	0.272699	0.288736	0.029553	0.236674
Pcdhgb7	0.307267	0.259303	0.271845	0.235035	0.236361	0.251364	0.305361	0.033756	0.236535
E330034G19Rik	0	0	0	0	0	0	0	0	0.236409
Gm16276	0.247491	0	0.24752	0.246105	0	0.228872	0.127897	0.471272	0.235881
Gm43013	0	0.061708	0	0	0	0.057218	0.063949	0	0.235881
Gm6860	0.247491	0	0.24752	0.246105	0	0.228872	0	0.706908	0.235881
Ifi202b	0.216434	0	0.092768	0.030746	0	0.343117	0.127826	0.058876	0.23575
Csrp3	1.050666	0.801311	1.17441	0.122915	2.001285	0.342926	0.766528	1.000337	0.235618
Gm7435	0.112359	0.067236	0.067423	0.11173	0.021402	0.207813	0.023226	0.021395	0.235594
Gm11663	0.494247	0.657242	0.329536	0.737218	0.31381	0.533242	0.340553	0.235286	0.235531
Slc25a2	0.041187	0.123233	0.20596	0.368609	0.627621	0.228532	0.212845	0.431357	0.235531
Frmd8os	0.282307	0.175972	0.035292	0.192999	0.050412	0.35897	0.127653	0.33598	0.235431
Gm19205	0.12347	0.123141	0.123484	0.368335	0.058796	0	0.063806	0.293889	0.235356
Gm11349	0.041157	0.041047	0	0	0.078394	0.03806	0	0.117556	0.235356
Gm41806	0.12347	0	0.123484	0	0.117592	0	0	0	0.235356
LOC118567978	0.061735	0.061571	0.185227	0.675281	0.352775	0.399634	0	0.235111	0.235356
Tktl1	0.112177	0.044751	0.201943	0.156169	0.021367	0	0.046376	0.021361	0.235213
Apln	0.528149	0.175581	0.281712	1.225445	0.93894	0.960551	0.272934	0.469326	0.234907
Crabp2	0.184794	0.061434	0.12321	0.061253	0.703981	0.056964	0.509316	0.058647	0.234833
9230105E05Rik	0.073917	0	0.221778	0.122506	0.140796	0.091142	0.127329	0	0.234833
Gm29793	0	0	0.061537	0	0	0	0.063594	0.585821	0.234572
Myh2	0.136704	0.154519	0.13672	0.335315	0.27775	0.160132	0.15071	0.112802	0.234524
LOC118568146	0.122923	0.061298	0	0.213909	0.058535	0.142094	0.031762	0.175551	0.234312
Gm13748	0.406305	0.405223	0.235785	0.319233	0.238864	0.22266	0.43549	0.305653	0.234259
Gm15707	0.947959	0.45521	1.509888	1.18704	0.869388	0.584429	0.762038	1.236828	0.234238
2010110K18Rik	0.429752	0.918446	0.859605	0.183148	0.526232	0.62452	0.888341	0.175357	0.234053
H3c7	0.18418	0.367379	0.368402	0.671542	0.584702	1.078716	0.190359	1.402856	0.234053
Ffar1	0	0	0.184201	0	0.292351	0.454196	0.126906	0.409166	0.234053
Fam13a	0.18079	0.16743	0.05166	0.269666	0.172183	0.191073	0.186856	0.19672	0.233848
Abhd1	0.286185	0.081549	0.24533	0.406545	0.038937	0.037808	0.507062	0.194626	0.233794
Gm31092	0.122651	0	0	0	0	0.226847	0	0.233551	0.233794
LOC118567736	0.479308	0.380758	0.387392	0.551442	0.22559	0.226778	0.812207	0.222868	0.233723
Zfp977	0.030646	0.152821	0.122597	0	0	0.02834	0	0	0.233665
Gm34186	0.24512	0.448191	0.531156	0.89374	0.155633	0.642259	0.126672	0.116689	0.233622
Gm5578	0.506508	0.228136	0.245113	0.194969	0.311221	0.151098	0.607936	0.171119	0.233587
Gm36028	0	0.048886	0	0	0.046683	0.181317	0.202645	0.280013	0.233587
Pdzd7	0.030629	0.488756	0.582014	0.578687	0.262534	0.226596	0.379876	0.17497	0.233535
Gm5648	0.244991	0.139622	0.105009	0.38283	0.033332	0.097097	0.072346	0.066645	0.233499

Atad3aos	0.081647	0.570005	0.326624	0.324757	0.233278	0.226513	0.084386	0.466414	0.233449
Gm10384	0.733955	0.390401	0.685104	0.486562	0.559209	0.203622	0.556291	0.349399	0.233175
St8sia5	0	0	0	0.040535	0	0	0	0	0.233106
Gm10369	0.3666	0.121875	0.040738	0	0.271558	0.037669	0.336799	0.54295	0.232935
Neu2	0	0.034818	0.349147	0.034715	0.132994	0	0	0	0.23291
1700095J03Rik	0	0	0	0.030373	0	0	0	0	0.232892
Slc23a3	0.298638	0.189536	0.298673	0.377956	0.310276	0.150639	0.785673	0.49112	0.232878
Gm6822	0.610551	0.608926	0.122124	0	0.116296	0.451695	1.514485	0.116261	0.232764
Morn4	1.892709	1.339637	1.221245	1.214263	0.639631	0.790466	1.514485	1.104479	0.232764
Gm31879	0	0	0	0	0	0	0	0	0.232764
Plekhg4	0.012851	0.166615	0.257045	0.30669	0.134628	0.249563	0.119537	0.281409	0.23271
LOC118568325	0.360745	0.412694	0.328953	0.516985	0.373885	0.30417	0.372848	0.47479	0.232586
Gm26590	0.696896	0.625536	0.453035	1.212736	0.232301	0.483351	0.93636	1.028445	0.232471
Gm51771	0.162575	0	0.081297	0	0.232252	0	0.084015	0	0.232423
Rpgrip1	0.258985	0.835663	0.716101	0.196938	0.391746	0.619886	0.456621	0.304598	0.232316
Gm34891	0.243643	0.074767	0.09372	0.186368	0.356989	0.086659	0.135594	0.08922	0.232213
Ccdc120	0.152261	0.212597	0.411151	0.317956	0.39153	0.267531	0.267527	0.275437	0.232189
Unc93a2	0.208806	0.034708	0	0	0.165721	0	0.035969	0.033134	0.232179
Gm12741	0	0.080952	0.081178	0.564997	0.309216	0.075062	0.335567	0.618243	0.232082
Rps2-ps4	0	0	0	0	0	0	0	0	0.232082
Tbx4	0.112337	0.112037	0.187249	0.465447	0.124819	0.086571	0.193509	0.463473	0.231978
Plcd4	0.22305	0.384242	0.141957	0.241964	0.289678	0.281277	0.167661	0.212365	0.231913
LOC118567944	0.034757	0.034665	0.069523	0.725817	0.066205	0.321426	0.287388	0.13237	0.231888
Susd5	0.042931	0.014272	0.100184	0.327293	0.286208	0.145571	0.029581	0.245246	0.231863
Aldoart1	0	0.026945	0.02702	0.026865	0	0.074952	0	0	0.231743
Gm12508	0	0.161668	0	0	0	0	0	0.154335	0.231743
Islr2	0.185698	0.413147	0.157148	0.511361	0.095231	0.277407	0.014764	0.040801	0.231444
Adprhl1	0.080814	0.161197	0.323292	0.401804	0.038483	0.261568	0.16705	0.230827	0.231067
Klhl33	0.373626	0.463272	0.313075	0.301243	0.692441	0.261473	0.542713	0.480715	0.230983
Gm52500	0.07269	0.120827	0.024233	0.240942	0.138458	0.112035	0.300514	0.230693	0.230933
Slc6a4	0.201813	0.181148	0.161469	0.381297	0.672716	0.522565	0.292017	0.153717	0.230815
Gm32236	0.026899	0.026827	0	0.053496	0.025618	0.04975	0	0.02561	0.230731
Gm51692	0	0	0.484231	0	0.230562	0	0.750629	0	0.230731
Gm12796	0.258227	0.563367	0.274398	0.208634	0.292045	0.104475	0.433697	0.384152	0.230731
Gm46773	0.242044	0.211224	0.423625	0.200573	0.182495	0.149223	0.354399	0.432092	0.230689
Klk1	0.060456	0	0.060463	0.901759	0.23031	0.335446	0.374905	0.11512	0.230479
Gm32946	0.241824	0.301475	0.181389	0.601173	0.345465	0.223631	0.312421	0.34536	0.230479
Slc2a4	0.386328	0.481624	0.265631	0.552236	0.528906	0.44658	0.149733	0.321844	0.230128
Rbm6-ps1	0.297978	0.127365	0.227057	0.05644	0.067569	0.301804	0.381302	0.310724	0.229904
LOC118568428	0.241035	1.121836	0.803544	0.15979	0.153039	0.148601	0.498244	0.305985	0.229728
Gm20522	0.16069	0.240393	0.160709	0.079895	0.229559	0.074301	0.166081	0.076496	0.229728
Armcx4	0.077982	0.120197	0.198523	0.211487	0.11478	0.216346	0.146542	0.20924	0.229728
Magi2	0.160574	0.160146	0.20074	0.309369	0.143371	0.083528	0.155588	0.257988	0.229561
Gm12216	0.481233	0.143986	0.336902	0.38283	0.274993	0.623041	0.795806	1.053817	0.229329
Nlrp6	0.012651	0.050471	0.012653	0.113224	0.012049	0.035099	0.117682	0.012045	0.229099
Ugt1a1	0.13735	0.102739	0.034342	0.136581	0.130811	0	0.141959	0.098078	0.229088
Gm12020	0.048061	0.047933	0.096133	0.047792	0.091545	0.17778	0.049673	0.045759	0.229031
Gm12279	0.400275	0.379249	0.420337	0.179115	0.114365	0.203589	0.186167	0.228661	0.228898
Lrrc23	0.040027	0.319367	0.160129	0.039803	0.304974	0	0.124111	0	0.228898
LOC118568679	0	0	0.080064	0.039803	0	0	0	0	0.228898
Pcdhga12	0.250999	0.097956	0.294685	0.249593	0.239049	0.080736	0.315816	0.083122	0.228823
Gm13311	0	0.047871	0.048004	0.095459	0.137139	0.177549	0.198435	0.228496	0.228733
Gucy1b1	0.274276	0.478705	0.308597	0.937543	0.408152	0.36461	0.088587	0.179532	0.228733
Gm13031	0	0	0.300024	0.059662	0.114283	0.721294	0	0.114248	0.228733

Gm35165	0.719975	0.119676	0.840068	0.954589	0	0	0	0.114248	0.228733
Col7a1	0.221752	0.227139	0.14985	0.244349	0.136991	0.099764	0.130082	0.182599	0.228486
Gm13986	0.095886	0.143446	0.303674	0.444961	0.197862	0.295574	0.264274	0.197801	0.22847
Tmprss4	0.026615	0.50433	0.4525	0.476378	0.405558	0.221511	0.247567	0.253396	0.228294
Rpl31-ps15	0.119737	0.358254	0.718503	0.119066	0	0.553643	0.247508	0.114001	0.228239
Gm39760	0.119737	0	0	0	0	0.110729	0	0	0.228239
Gm7609	0.1026	0.136435	0.786689	0.510125	0.423431	0.853929	0.318126	0.58611	0.228169
Tbx10	0.0342	0.068218	0	0.136033	0.065143	0	0.035347	0	0.228169
Gm2788	0.079767	0.079555	0.319105	0.07932	0.037985	0.258181	0.741989	0.227838	0.228075
LOC108168673	0.119479	0	0	0	0	0.66294	0.740922	0	0.227747
Klhl30	0.220284	0.421086	0.220309	0.52937	0.524489	0.71299	0.474321	0.471896	0.227446
H2bu1-ps	0	0.118904	0.357706	0.355661	0.340636	0.44101	0.862551	0.113511	0.227258
Pou5f2	0.158962	0.079269	0.198726	0.355661	0.264939	0.514511	0.246443	0.075674	0.227258
Gm39221	0.164994	0.182839	0.091674	0.16407	0.279358	0.067814	0.208425	0.157091	0.227145
Gm6937	0	0.158425	0.079433	0	0.151285	0.146898	0.082089	0.529337	0.227095
Smim41	0.059547	0.415718	0.119107	0	0	0	0.307723	0	0.227014
Eqtn	0.238136	0.190002	0.047633	0.236802	0.725754	0.308309	0.295351	0.136037	0.226965
Tbc1d30	0.162353	0.539735	0.270619	0.182969	0.154623	0.170157	0.458653	0.123661	0.226947
Amn	0.158621	0.474596	0.15864	0	0.151069	0.220032	0.204928	0.037756	0.22677
Eef1akmt3	0.158621	0.079099	0	0.788663	0.075534	0	0.081971	0.604091	0.22677
Gm10120	0.594829	0.474596	1.189796	1.182994	0.566509	0.880127	0.983657	0.226534	0.22677
Gm14239	0.047586	0.118649	0.190367	0.354898	0.02266	0	0	0.090614	0.22677
Boc	0.713634	1.023898	0.613546	0.622488	0.238476	0.590478	0.414078	0.369525	0.226719
Cdh4	0	0	0	0	0.03774	0.219874	0	0.226372	0.226608
Gm18817	0.047546	0.047419	0.095102	0.141838	0.271691	0	0.442266	0.045268	0.226575
Gdf11	0.199588	0.236973	0.418234	0.207921	0.190086	0.386725	0.363454	0.416251	0.226459
Parm1	0.263991	0.368603	0.105609	0.708782	0.301706	0.5615	0.05457	0.377018	0.226446
Gm42043	0	0	0	0	0.301706	0	0	0	0.226446
LOC118568176	0.205469	0.300979	0.276131	0.140469	0.250723	0.100943	0.37827	0.22008	0.226429
Mark1	0.152722	0.118467	0.118798	0.455599	0.290901	0.094155	0.017538	0.274656	0.226423
4933411K16Rik	0.316563	0	0.039575	0.078697	0	0.036593	0.408979	0	0.226284
Gm18943	0.03957	0.670905	0.633199	0.59023	0.150745	0.219561	0.081796	0.226049	0.226284
Havcr1	0.296778	0.177593	0.593624	0.147558	0.141324	0.301896	0	0.056512	0.226284
Zfp551	0.166124	0.21302	0.569635	0.117996	0.361634	0.46088	0.196226	0.248548	0.226187
Arhgap28	0.503982	0.635693	0.548515	0.560119	0.409401	0.411236	0.245125	0.310485	0.226042
Gm4977	0.338656	0.337755	0.406435	0.572491	0.258026	0.093954	0.070004	0.580381	0.225939
Gm16701	0.296143	0.413497	0.296178	0.471175	0.338453	0.438183	0.918237	0.620307	0.225801
Gm15473	0.236915	0.425311	0.236942	0.141353	0.090254	0.394364	0.73459	0.406019	0.225801
Gm4600	0.47383	0.630091	0.552865	1.099409	0.526482	0.438183	0.326484	0.150377	0.225801
Gzmc	0	0	0.236942	0.058897	0	0	0	0	0.225801
Olfr1371	0	0	0	0	0	0	0	0	0.225801
Vstm4	0.107605	0.279028	0.322852	0.256805	0.45092	0.537352	0.689533	0.655683	0.225626
Dcst1	0.142027	0.259691	0.071022	0.094155	0.225442	0.175123	0	0.045075	0.225608
4930578L24Rik	0.050706	0.252853	0.016904	0.11765	0.016097	0.171933	0.034938	0	0.225525
Gm4117	0.363334	0.252814	0.464784	0.168046	0.040237	0.117209	0.026199	0.273526	0.225491
LOC118567814	0.177421	0.212338	0.035488	0.19995	0.394272	0.18595	0.52567	0.259014	0.225464
Optc	0	0.188705	0.18923	0	0	0	0.048889	0	0.225416
Gm15928	0.551623	0.510857	0.433468	0.626894	0.112577	0.291499	0.488682	0.600227	0.225319
1700086P04Rik	0.047262	0.23568	0.330871	0.234985	0.315081	0.087412	0.439627	0.269987	0.225223
Gm16938	0.163588	0.217536	0.236321	0.198821	0.138488	0.117663	0.093931	0.103834	0.225209
Hoxa5	0.029535	0.265111	0.118156	0.08811	0.112517	0.573583	0.518948	0.253086	0.225199
Pik3r3	0.436706	0.388458	0.30691	0.645521	0.640732	0.600321	0.329369	0.280937	0.224984
Camk2b	0.177024	0.470808	0.206552	0.352065	0.309092	0.300128	0.289692	0.266862	0.22496
Pspn	0.157271	0.941114	0.550513	0.703756	0.074892	0.07272	0.406369	0.374344	0.22484

Gm14869	0.37713	0.047016	0.14144	0.187509	0	0.043595	0.389782	0.044883	0.224649
Rims3	0.383715	0.25223	0.331429	0.381565	0.265779	0.282265	0.369548	0.307213	0.224416
Rpl30-ps9	0.117703	0.234779	0.588583	0.702261	0.112099	0.217696	0.364955	0.448259	0.224363
Gm35330	0.235406	0	0.35315	0	0.336297	0	0	0.112065	0.224363
Gm51491	0.159715	0.402417	0.22699	0.284206	0.176129	0.155473	0.538662	0.192082	0.224329
Bik	0.117578	0.351795	0.293979	0.233839	0.27995	0.706761	0.243046	0	0.224125
Has2	0.235128	0.117251	0.300477	0.27278	0.087085	0.314079	0.081006	0.236302	0.224098
Dlk2	0.078358	0	0.156734	0.272716	0	0.144925	0	0.335719	0.224046
Gm35598	0.117454	0.117141	0.939737	0.467182	0.78303	0	0.728364	0.111827	0.223887
Gm10108	0.234907	0.292852	0.117467	0.350387	0.055931	0.543087	0.121394	0.223655	0.223887
Pllp	0.352174	0.292697	0.117405	0.612852	0.111802	0.597079	0.030332	0.530898	0.223769
Gm39214	0.12875	0.2266	0.12119	0.180746	0.100981	0.063034	0.172207	0.165847	0.223765
D830015G05Rik	0.046942	0.14045	0.093894	0	0.178827	0	0	0	0.223698
Gm52568	0.335176	0.133713	0.033521	0.166649	0.031922	0.30996	0.138568	0	0.223617
Gm52873	0.208512	0.025995	0.052134	0.155508	0.223407	0.096413	0.242446	0	0.223572
Gm14391	0.058603	0.292233	0.293047	0.233097	0.502312	0.270969	0.060569	0.055795	0.223414
LOC118568505	0.234287	0.292079	0.175735	0.196572	0.181295	0.10156	0.151342	0.174269	0.223296
Gm15433	0.102446	0.087577	0.168324	0.072766	0.048784	0.196245	0.09832	0.139341	0.223178
Efna2	1.092632	1.16756	0.832578	0.517386	0.619411	0.866084	0.618421	0.470609	0.223152
LOC118567707	0.146275	0.029177	0.409616	0.232728	0.306483	0.27054	0.030236	0.139268	0.22306
Gm6087	0.191459	0.148516	0.127654	0.317311	0.202604	0.157383	0.043974	0.081017	0.223028
Spata9	0.292395	0.583233	0.292429	0.058151	0.222779	0.108159	0.483527	0.445422	0.222943
Sla2	0.116958	0	0.467886	0.697817	0.148519	0.612901	0.040294	0.111355	0.222943
Dnajb7	0.37395	0.139858	0.046749	0.232409	0.178073	0.172909	0.531432	0.044505	0.222755
Slamf1	0.327206	0.512812	0.046749	0.371855	0.044518	0.259363	0	0	0.222755
Slc12a5	0.136188	0.097018	0.068102	0.319215	0.185291	0.179917	0.402162	0.213019	0.222512
Ankrd35	0.016668	0.016624	0.06668	0.31492	0.095247	0.015414	0.051682	0.01587	0.222406
LOC118568122	0.233088	0.103998	0.153365	0.274479	0.356353	0.181518	0.209209	0.087601	0.222153
Slit1	0.433937	0.285002	0.508083	0.294687	0.262078	0.264265	0.711029	0.110845	0.221921
Gm7061	0.284298	0.257764	0.025848	0.051401	0.049229	0.454117	0.347261	0.024607	0.221695
Ccdc78	0.271185	0.579565	0.309962	0.231143	0.332067	0.214958	1.161175	0.7377	0.22154
Gm4742	0	0.057956	0.058118	0	0	0	0	0	0.22154
Olfr455	0	0	0	0	0	0	0	0	0.22154
Gm47655	0.696604	0.231583	0.4064	0.288626	0	0.107366	0.119996	0.939583	0.221308
Tsg101-ps	0.735048	0.385837	0.812515	0.3847	0.368448	0.28621	0.319877	0.478836	0.221231
Gm40476	0.282112	0.30637	0.225716	0.44885	0.292563	0.301471	0.408207	0.286504	0.221077
Gm18726	0.185567	0.254475	0.231985	0.023066	0.132549	0.064352	0.239741	0.287101	0.221077
Gm4247	0.371133	0.185073	0.301581	0.184527	0.353463	0.193057	0.11987	0.265017	0.221077
Olfr222	0.057929	0	0	0.230418	0.110342	0	0.059873	0	0.220846
Gm34853	0.115858	0	0	0	0	0	0	0	0.220846
Gm10608	0.386059	0.269522	0.501935	0.307117	0.147071	0.249911	0.35911	0.073513	0.220769
Agtr2	0.173727	0.462037	0.386104	0.268727	0.165455	0.124955	0.119703	0.312431	0.220769
Csf2	0.077212	0	0.077221	0	0.073536	0.142806	0.079802	0.073513	0.220769
Gsta4	0	0.057714	1.157505	0	0.055113	0	0	0	0.220615
AI661453	0.517804	0.318645	0.385643	0.14242	0.293792	0.173201	0.204961	0.241255	0.220506
Cyp4b1	1.07001	0.634528	0.375993	1.754188	0.468219	1.069742	0.717346	0.192737	0.2205
Ttc16	0.084061	0.104796	0.063053	0.041795	0.26019	0.155473	0.152042	0.100043	0.220323
Mfsd2b	0.105036	0.460929	0.483222	0.45957	0.280099	0.271976	0.738208	0.260012	0.220239
Ptger3	0.09232	0.046037	0.184661	0.229507	0.219811	0.256123	0.811046	0.219744	0.219973
Kcnk5	0.148318	0.180795	0.280189	0.802987	0.251123	0.28956	0.051098	0.517783	0.219894
Gm34222	0.046122	0.045999	0	0	0.219628	0.042652	0	0	0.21979
Ebf2	0.28548	0.317573	0.219626	0.2948	0.230059	0.294465	0.181574	0.313621	0.219763
Gm5446	0.128092	0.229951	0.230592	0.254748	0.048797	0.094764	0.158867	0.121956	0.219749
Adora1	0.072785	0.08469	0.012132	0.265384	0.03466	0.100964	0.025076	0.0231	0.219674

Axdnd1	0.098761	0.213413	0.378629	0.278257	0.125412	0.289216	0.238175	0.078359	0.219633
4931408C20Rik	0.499211	0.178727	0.627287	0.661887	0.097527	0	0.51596	0	0.219597
Gm13768	0.176123	0.14863	0.284539	0.215552	0.025806	0.18793	0.280049	0.128989	0.21951
Gm45972	0.345289	0.420896	0.230219	0.419656	0.730777	0.390271	0.555136	0.657498	0.219394
Slc17a8	0.086307	0.315618	0	0.042912	0.150697	0.026605	0.089203	0.109564	0.219356
Gm40365	0	0	0	0	0.438163	0.425455	1.188755	0.438029	0.219242
Gm45914	0	0	0	0	0	0	0	0.073005	0.219242
LOC664787	0	0	0	0	0	0	0	0	0.219242
Cpz	0.255122	0.025444	0.153091	0.405909	0.024298	0.401079	0.158209	0.121451	0.218839
Gm41018	0.016382	0.098033	0.049153	0.048872	0.031205	0.151499	0.203184	0.015598	0.218594
Gm15413	0.573003	0.171443	0.171921	0.683752	0.081858	0.741854	0.355336	0.681944	0.218449
Gm32990	0.114541	0.114237	0.057277	0	0	0.105924	0.414345	0	0.218336
Gm2805	0	0.171355	0.057277	0.05695	0.109088	0.052962	0.355153	0.163582	0.218336
Gm39393	0.503982	0.34271	0.274931	0.29614	0.436352	0.677916	0.260445	0.23992	0.218336
Igsf9b	0.166418	0.041494	0.208047	0.041372	0.21793	0.038475	0.150501	0.039612	0.218091
1700034H15Rik	0.165221	0.063377	0.050843	0.012638	0.108938	0.387855	0.157629	0.399317	0.218036
D830039M14Rik	0.076204	0.038	0	0.075777	0	0	0	0.253936	0.217886
LOC115490435	0.394208	0.558699	0.083001	0.26821	0.15808	0.460485	0.192995	0.217293	0.217519
Gm30967	0.354723	0.252699	0.532146	0.529103	0.361965	0.515486	0.680872	0.53072	0.217338
Gm2213	0.032572	0	0	0	0	0.030122	0	0	0.21731
Gm13423	0.056976	0.454598	0.056983	0.793202	0	0.10538	0.058888	0.054247	0.217214
Gm8174	0.227578	0.045394	0	0.090521	0.520183	0.29464	0	0.606695	0.216902
Gm9081	0.181913	0.090714	0.136451	0.045224	0.043313	0.210285	0.047004	0.173199	0.216724
Gm30745	0.530145	0.2266	0.075744	0.301243	0.721292	0.28015	0.078276	0.50475	0.216547
Gm34983	0.227205	0.13596	0.159062	0.06778	0.649163	0.357191	0.070448	0.086529	0.216547
Slc26a9	0.283861	0.141553	0.255505	0.141135	0.189242	0.157503	0.117354	0.432422	0.216436
D330050G23Rik	0.226939	0.80834	0.713321	0.934911	0.277887	0.029981	0.737167	0.061734	0.216293
Gm34882	0.340111	0	0	0.169103	0	0.366944	0.410108	0.05397	0.216104
Gm3940	0.11337	0.113068	0	0	0	0.104841	0.117174	0	0.216104
Card14	0.104633	0.226101	0.052323	0.138729	0.215911	0.193523	0.180239	0.132828	0.21607
Gm41253	0.075563	0.251206	0.42824	0.125233	0.167919	0.25622	0.052065	0.071943	0.216055
Gm30502	0.032363	0.096831	0.194201	0.257455	0.092467	0.029928	0.133796	0.369755	0.215915
Ddx43	0.045293	0	0.135893	0.495427	0.388225	0.041885	0.046812	0.172492	0.215839
Syngr3	0.028299	0.169343	0.22642	0.056281	0.026952	0.287872	0	0.026944	0.215773
Gm46344	0.056569	0.056419	0.113152	0.168758	0.161628	0	0.058467	0.05386	0.215663
Gm5830	0.419741	0.450825	0.516664	0.674245	0.338255	0.268728	0.500565	0.522599	0.215412
Gm12728	0.150647	0	0.451993	0.149803	0.143474	0.139314	0.077851	0.430292	0.21537
Gm11557	0.090364	0.180246	0.135561	0.134786	0.301215	0.083565	0	0	0.215311
Gm7293	0.135545	0.045062	0.090374	0.134786	0.043031	0.167131	0	0	0.215311
Gm6263	0.161345	0.257464	0.258182	0.096265	0.245861	0.089524	0.300164	0.245786	0.215286
Caly	0.112908	0.394128	0.282304	0	0.322598	0.104414	0.233393	0.05375	0.215224
Rep15	0.056454	0.056304	0.056461	0	0.107533	0	0.058348	0	0.215224
Gm7042	0.09029	0.045025	0	0.224461	0.042996	0.041749	0	0.042982	0.215136
Gm36132	0.13538	0.157523	0.225659	0.448739	0.042978	0.062598	0.069961	0.021482	0.215048
Ces1d	0	0.056247	0	0	0	0.156462	0	0.134238	0.215005
Gm32604	0.173064	0.219676	0.262248	0.140804	0.174813	0.092146	0.254754	0.109849	0.214928
Gm36787	0.169104	0.056218	0.197311	0.336314	0.214738	0	0.174778	0.107336	0.214895
Gm15795	0.112679	0.449516	0.676153	0.112048	0.107314	0	0	0.107281	0.214786
Gm34885	0	2.24758	0	0	0.214628	0.833616	0	0.429126	0.214786
Napb	0.330257	0.404236	0.435389	0.403044	0.128672	0.208235	0.372367	0	0.214612
Gm12907	0	0	0	0	0	0	0	0	0.214612
Gm41604	0.429098	0.666488	0.401007	0.19586	0.241181	0.065052	0.886989	0.160738	0.214541
Pcdhgb6	0.319562	0.283299	0.295925	0.52962	0.180354	0.262685	0.415912	0.315523	0.214328
LOC118567594	0.214694	0.180666	0.11407	0.35026	0.146965	0.145805	0.506203	0.207604	0.214214

4930479D17Rik	0.224749	0.074717	0	0.148993	0.570796	0.277121	0.07743	0.142655	0.214206
Gm32438	0.112365	0.098058	0.224756	0.488843	0.133769	0.194834	0.290337	0.240711	0.214188
Cspg4b	0.101123	0.033618	0.089898	0.078211	0.010701	0.010391	0.023226	0.032093	0.214177
4930592I03Rik	0.112337	0	0	0	0.053494	0.051943	0.232211	0.106955	0.214133
Gm33682	0.190895	0.13439	0.348143	0.133994	0.31014	0.238839	0.127664	0.449031	0.214047
LOC105246046	0.224446	0.335772	0	0	0	0.10378	0.347964	0.106847	0.213917
Gm18258	0.056111	0	0.112236	0	0	0	0	0.053424	0.213917
Gm46767	0.224446	0.447696	0.224472	0	0.21376	0	0	0	0.213917
Gm6043	0.074714	0.223546	0	0.222888	0.071157	0.069094	0.154442	0.071135	0.213628
A330023F24Rik	0.341486	0.255432	0.202781	0.244068	0.172777	0.286189	0.275736	0.162564	0.213587
Gm12856	0.037307	0.074415	0.335801	0.259686	0.106592	0	0	0	0.213341
D330050I16Rik	0.639424	0.191317	0.255799	0.286129	0.487184	1.034809	0.462614	0.456596	0.2133
Gm7045	0.571783	0.371909	0.298356	0.914672	0.260442	0.229899	0.693743	0.260362	0.213245
Erfe	0.391393	0.083647	0.279599	0.417001	0.106502	0.439509	0.433419	0.079852	0.213161
Gm9626	0.307523	0.167293	0.587158	0.278001	0.053251	0.180974	0.057789	0.186322	0.213161
Tal2	0.27952	0.204435	0.335463	0.148242	0.106485	0.120629	0.03852	0.070968	0.213126
Gm47708	0.223541	0.111473	0	0	0.532244	0.206723	0.462081	0.212833	0.213054
H3c2	0.223541	0	0.223567	0	0.532244	0.413447	0.23104	0.106416	0.213054
Gm5864	0.267817	0.178069	0.401772	0.044386	0.042511	0.701727	0.461337	0.594971	0.212711
Gm17833	0.111545	0.166873	0.446233	0.166381	0.424938	0.051577	0.807014	0	0.212625
Gm17046	0.390409	0.166873	0.055779	0.221841	0.159352	0.103154	0.115288	0.053101	0.212625
Gm42354	0	0	0.111558	0	0	0.051577	0	0	0.212625
Mme	0.47474	0.318872	0.436037	0.481716	0.119955	0.215033	0.120164	0.221387	0.212384
Hipk4	0	0.166621	0.027848	0.110753	0.026519	0	0.057557	0.026511	0.212305
Gm34325	0	0	0	0	0	0.102947	0	0.211978	0.212198
Ror2	0.055647	0.083248	0.097393	0.193673	0.013249	0.205841	0.057514	0.278151	0.212145
LOC115486328	0.074148	0.098601	0.181272	0.311316	0.10985	0.099045	0.400209	0.243165	0.212009
Npm2	0.611654	0.720939	0.166834	0.387054	0.741405	0.257108	0.517234	0.317648	0.211986
Gm41598	0.324949	0.204685	0.213807	0.357143	0.179172	0.142344	0.477262	0.219825	0.211904
Dzip1l	0.18305	0.065201	0.209225	0.247034	0.14943	0.169279	0.229733	0.211628	0.211848
Gm46093	0.294213	0.239534	0.114096	0.203004	0.102932	0.072184	0.062058	0.085751	0.211739
Syt5	0.158666	0.031649	0	0	0.12089	0.029346	0.295181	0	0.211713
Kcna7	0.253866	0.126595	0.031737	0.063111	0.045334	0.044019	0	0	0.211713
Triqk	0.111024	0.221457	0.037012	0.147203	0	0.239567	0.152999	0.739941	0.211632
Gm41308	0.204482	0.262206	0.303838	0.197527	0.422878	0.486254	0.446839	0.333749	0.211595
Gm40304	0.332628	0.221162	1.10889	0.66153	0.422389	0.410139	0.114596	1.689039	0.21135
H2bc12	0.221752	0.552905	0	0.992295	0.422389	0.307604	0	0.105565	0.21135
Wdr31	0	0.132697	0	0	0.168956	0.615209	0	0	0.21135
LOC115490344	0.398676	0.530152	0.199361	0.352393	0.084377	0.040965	0.343376	0.168701	0.211096
Gm41331	0.331964	0	0.110668	0	0	0.40932	0.343102	0.210708	0.210928
9230117E06Rik	0.331964	0	0.110668	0	0	0	0	0	0.210928
Npy1r	0.073745	0	0.073754	0.384994	0.175586	0.187543	0	0.38617	0.210858
Ypel4	0.147442	1.102868	0.073729	0	0.070211	0.068175	0.076194	0.350947	0.210787
Actl7a	0	0.073525	0	0	0.140422	0.034087	0.038097	0	0.210787
Gm38983	0.269519	0.37908	0.283374	0.60474	0.38174	0.466532	0.349988	0.111855	0.21077
Gm8789	0.252565	0.314866	0.284169	0.439513	0.150338	0.204369	0.293669	0.210408	0.210627
1110002E22Rik	0.158589	0.152518	0.266233	0.304136	0.070125	0.183322	0.064393	0.21031	0.210529
Gm29886	0.031553	0.220281	0.315563	0.031376	0.420706	0	0.065223	0.180247	0.210508
LOC118568157	0	0.044056	0	0.351411	0	0	0.045656	0	0.210508
Olfr433	0.496955	0.495632	0.883578	0.329447	0.420706	0.561694	0.171209	0.052572	0.210508
Cerkl	0.472752	0.565792	0.724971	0.877527	0.420228	0.845227	1.0098	0.780184	0.210268
Jrk	0.350937	0.220002	0.310866	0.378883	0.305581	0.41726	0.341985	0.448684	0.210241
Gm41414	0.048995	0.024432	0	0.097441	0.046662	0.135928	0.025319	0	0.210135
Gm18230	0.110215	0.109921	0	0.43839	0	0.101923	0	0	0.210089

Gm5472	0.16524	0.2472	0	0.410786	0.131144	0.28015	0.256176	0.262208	0.209985
Adgrg7	0.19278	0.274667	0.110173	0.273858	0.393432	0.305618	0	0.026221	0.209985
Spata46	0.293712	0.366162	0	0.511116	0.279728	0.611134	0.075891	0	0.20995
Smt3h2-ps4	0.550527	0.549061	0.385414	0.437955	0.681611	0.610932	0.341398	0.576571	0.20988
Chrdl2	0.330316	0.054906	0.110118	0.328466	0.104863	0.101822	0.113799	0.052416	0.20988
Crtac1	0	0.036592	0	0.145937	0.069886	0	0	0	0.209811
Gm33767	0.628549	0.752251	0.534329	0.343766	0.478899	0.348758	0.844528	0.089766	0.209672
Gm18362	0.109996	0.767923	0.550045	0.10938	0.314277	0.508605	0.454746	0	0.209672
Gm10353	0.379576	0.358641	0.31968	0.357584	0.133186	0.166273	0.516198	0.26629	0.209446
Gm9655	0.137257	0.123202	0.151	0.013649	0.039217	0.057119	0.063838	0.169887	0.209309
Tmem30b	0.016892	0.016847	0.016894	0.050391	0.096524	0.156208	0.034917	0	0.209289
Gm52918	0.109778	0.109486	0.219582	0	0	0.10152	0.340384	0.10452	0.209257
Adam30	0.015678	0	0	0.01559	0.164249	0.072493	0.032408	0.074636	0.209198
Adamtsl1	0.226567	0.169473	0.453186	0.436514	0.182064	0.327378	0.175626	0.411204	0.20919
Rtn4rl2	0.21947	0	0.395091	0.480129	0.041804	0.405918	0.362933	0.459705	0.209174
Cnih3	0	0	0	0.087296	0.020902	0	0.022683	0.041791	0.209174
Gm47692	0.146275	0.29177	0.292583	0.072728	0.13931	0.338175	0.226773	0	0.209119
Cpa6	0	0	0.043864	0	0	0.324477	0	0	0.209009
Ppp4r4	0.018254	0.018206	0.036513	0	0.017385	0.067524	0.037733	0	0.208775
Adamts7	0.180328	0.565237	0.476637	0.256169	0.122673	0.476463	0.359444	0.539598	0.208698
Gm46594	0.277166	0.363721	0.408503	0.594745	0.426057	0.269805	0.547801	0.328704	0.20855
Gm39859	0.109376	0.187003	0.234405	0.26414	0.11905	0.144497	0.274539	0.282657	0.20849
Sptbn5	0.160953	0.395138	0.297179	0.578648	0.212248	0.286239	0.469201	0.282911	0.20847
Gm42141	0.349767	0.087209	0.655889	0.391284	0.166557	0.525612	0.135563	0	0.208349
Rundc3b	0.109222	0.09337	0.18726	0.062063	0.163463	0.158723	0.064507	0.059423	0.208197
Gm52481	0.136413	1.170025	0.682142	0.813891	0.207869	0.20184	2.199431	0.519512	0.208021
Gm33730	0	0	0	0	1.039343	0	0.902331	0	0.208021
Dusp26	0.036377	0.03628	0.072762	0.108519	0.06929	0.63916	0.037597	0.277073	0.208021
St6galnac2	0.790804	0.571126	0.409084	1.382933	0.908977	0.983486	0.986439	0.882737	0.207919
Gm41947	0.19628	0.261009	0.174491	0.173493	0.062311	0.060504	0.405729	0.103821	0.207858
Gm10355	0	0.289858	0.435999	0.433506	0.484391	0.537535	0.300383	0.415065	0.207749
Gm35196	0.254137	0.108626	0.145238	0.288815	0.138307	0	0.150093	0.276529	0.207613
Gm35149	0.087133	0.282427	0.065357	0.303256	0.082984	0.181299	0.315195	0.124438	0.207613
Gm13612	0.108916	0	0	0	0	0.100722	0	0.103698	0.207613
Gm36035	0.098961	0.078958	0.178151	0.118088	0	0.128123	0.265931	0.037688	0.207501
Kcnj13	0.199548	0.162832	0.108857	0.541173	0.190047	0.117432	0.337488	0.259076	0.207477
Ugt1a6b	0.120885	0.048225	0.07254	0.120208	0.184208	0.178866	0.149929	0.184152	0.207386
Cfd	0.434808	1.011852	0.072476	0.288248	0.207053	1.608389	0.898792	0.551974	0.207205
Gm5735	0.217404	0	0	0.648559	0	0.402097	0.224698	1.03495	0.207205
Gm46619	0	0.216825	0	0.216186	0	0.201049	0	0	0.207205
Adgrl3	0.194479	0.068457	0.240267	0.364028	0.18522	0.042317	0.082766	0.206947	0.207163
Sgip1	0.148566	0.330535	0.137154	0.46593	0.359174	0.285347	0.401594	0.337303	0.206949
Gm35243	0.303889	0.519566	0.434178	0.259017	0.49615	0.040147	0.673038	0.413332	0.206881
Gm14036	0.207938	0.099184	0.054251	0.01798	0.137765	0.050164	0.196226	0.068862	0.2068
Gm8724	0	0	0.325506	0	0	0.300983	0	0.206585	0.2068
C4a	0.278895	0.381171	0.289258	0.318419	0.295129	0.420303	0.533799	0.393385	0.206742
Slc27a2	1.084186	1.105329	0.28915	0.023958	0.298297	0.044561	0.174309	0.068817	0.206665
Gm13142	0.406437	0.054047	0.189693	0.107776	0.438698	0.275631	0.364064	0.283777	0.206598
Gcgr	0.043345	0	0.04335	0.129306	0	0	0	0.082537	0.206557
Gm15860	0.027083	0.02701	0	0.026931	0.025793	0.025045	0	0	0.206497
8030431J09Rik	0.230948	0.242707	0.131713	0.14045	0.168146	0.12532	0.167679	0.20353	0.206471
Gm31113	0.162416	0	0.21658	0.646026	0.360928	0.250329	0.279775	0.206182	0.206396
Gm14198	0	0.143986	0.288773	0	0.068748	0.133509	0.074607	0.48109	0.206396
Gm13477	0.118037	0.274687	0.275452	0.058688	0.074945	0.272892	0.223661	0.262227	0.20625

Gm39326	0.288364	0.407429	0.720995	0.955831	0.137318	0.122224	0.732679	0.160155	0.206128
Tbxa2r	0.185308	0.184815	0.154442	0.307117	0.029414	0.057122	0.191525	0.352863	0.206051
Gm33100	0	0.143705	0	0	0.102921	0	0.037231	0	0.205994
Gm46891	0.185257	0.076985	0.247038	0.199571	0.176436	0.114213	0.23934	0.20578	0.205994
A930035F09Rik	0.215922	0.215348	0.053987	0.429426	0.359874	0.099839	0.278958	0.205579	0.205793
Rpl27a-ps1	0.107856	0.215138	0.539343	0.107252	0.924489	0.199484	0.222949	0.10269	0.205593
Gm18120	0	0.107569	0.107869	0	0.410884	0	0.111475	0.308069	0.205593
Gm10015	0.15408	0.061468	0.123279	0.153217	0.088047	0.085493	0	0.146699	0.205593
Mcpt1	0	0.430276	0	0.429008	0	0	0	0	0.205593
Ces2g	0.107647	0.483121	0.05383	0.29437	0.153782	0.223984	0.222517	0.128113	0.205194
Gm33800	0.080657	0.214512	0.107555	0.133675	0.025606	0.223766	0.277876	0.102391	0.204995
Liph	0.394322	0.429024	0.358516	0.499053	0.119492	0.331506	0.277876	0.324238	0.204995
Gm34121	0.107438	0.392891	0.179084	0.427345	0.341076	0.033118	0.185071	0.170486	0.204796
Ppm1e	0.053667	0.12489	0.089455	0.08005	0.204448	0.190247	0.092446	0.195869	0.204598
LOC118568094	0.204605	0.218992	0.199638	0.302708	0.299425	0.203057	0.118629	0.280328	0.20452
Gm13641	0	0.106945	0	0.10663	0.306375	0	0.110828	0.102094	0.2044
8030442B05Rik	0	0	0.053621	0.319889	0.051062	0.049582	0	0	0.2044
Gm38930	0.085751	0.384852	0.171522	0	0.040834	0	0.044314	0.040822	0.204321
Ccdc54	0	0.042662	0	0	0.162959	0	0.442114	0.366545	0.203848
Gm36199	0.74772	0.852262	1.602442	1.062187	0	0.592687	0.441603	0.203401	0.203612
Gm32006	0.165302	0.206077	0.289312	0.547919	0.085275	0.229299	0.291865	0.216402	0.203498
Pcnx2	0.047444	0.082806	0.094899	0.011795	0.135555	0.087749	0.098071	0.090342	0.203482
Arhgef19	0.05334	0.319188	0.444552	0.123763	0.135468	0.263078	0.165389	0.355495	0.203351
Tubb4b-ps2	0.14224	0.070931	0.177821	0.106082	0.067734	0.098654	0	0.169283	0.203351
Msi1	0.021331	0.127642	0.127998	0.063633	0.101575	0.039452	0.022046	0.16247	0.203299
Gm38594	0.106612	0.744295	0.213248	0	0.101536	0	0.550942	0.101505	0.203221
Bnipl	0.142149	0.106328	0.213248	0.035338	0.135381	0.065727	0.367295	0.033835	0.203221
Gm46195	0	0.042531	0	0.084812	0.203072	0.039436	0.044075	0.121806	0.203221
Cpa2	0.053204	0.053062	0	0.158716	0.202682	0.049201	0	0	0.202831
Lincmd1	0	0	1.170613	0	0.101341	0	0	0.20262	0.202831
Gm40528	0.402606	0.40911	0.205125	0.626964	0.260448	0.337193	0.620244	0.202509	0.20272
Pcdhga3	0.059077	0	0.082718	0.140991	0	0	0.048847	0.011249	0.202701
Gm32998	0	0.035329	0	0	0.033737	0	0	0	0.202571
H2bc9	0.31861	0.847364	0.212431	0.211217	0.101147	1.178561	0.219533	0.202232	0.202442
Gm9028	0	0	0	0	0.202294	0.29464	0.054883	0.050558	0.202442
LOC118567951	0	0	0	0	0	0	0	0	0.202442
Omp	0.053076	0.397012	0.265412	0.105558	0.252746	0.294499	0.164571	0.48007	0.202345
Serpinc1	0.035378	0.105853	0.106148	0	0.101082	0.294452	0.073131	0.033684	0.202313
Hoxaas2	0.035378	0.176421	0.14153	0.070361	0.033694	0.032717	0.036565	0.033684	0.202313
LOC118568184	0.247491	0.035262	0.14144	0.597684	0.505089	0.523137	0.146168	0.100987	0.202184
LOC118568693	0.070622	0.422602	0.42378	0.351131	0.269037	0.065309	0.145982	0.134478	0.201926
Gm46130	0.211798	0.580893	0.158867	0.789793	0.705999	0.097932	1.094518	0.151239	0.201862
Krt10	0.291222	0.237638	0.2383	0.342243	0.756427	0.220347	0.136815	0.252065	0.201862
Gm32267	0.592468	0.253239	0.507889	0.504985	0.241826	0.234812	0.437389	0.241752	0.201669
Gm8543	0.338553	0.464271	0.592537	0.21041	0.120913	0.547896	0.39365	0.282044	0.201669
Ggt1	0.079235	0	0.158488	0.236373	0.100617	0.195397	0.027298	0.050293	0.201381
Myo15b	0.063346	0.084236	0.010559	0.020997	0.04022	0.078107	0.076383	0.050259	0.201247
Jchain	0.237366	0.026304	0.342901	0.288488	0.251183	0	0.054518	0.326438	0.201094
Gm30794	0.126571	0.126234	0	0	0.040182	0.546228	0.218029	0.321354	0.201056
Gm9419	0	0	0	0.034957	0	0.032509	0.036334	0	0.20103
Gm38410	0.193225	0.052557	0.105408	0.576428	0.317862	0.2924	0.38126	0.317765	0.200903
LOC118568394	0.16859	0.336283	0.16861	0.153675	0.187324	0.168899	0.479177	0.147138	0.200852
Gm14488	0.178257	0.161621	0.226899	0.257831	0.077168	0.164847	0.251234	0.046287	0.200785
Gm33610	0.084252	0.042014	0.105328	0.146616	0.220663	0.097392	0.239467	0.040108	0.20075

Plin5	0.14035	0.17497	0.035091	0.034891	0.066834	0.064895	0.036265	0.133627	0.200648
P2rx3	0.105139	0.059919	0.060086	0	0.157352	0.194458	0.139713	0.085802	0.200413
Gm29998	0.280108	0.302643	0.560281	0.208904	0.289003	0.410139	0.289506	0.155569	0.200226
LOC118568369	0.015004	0.044891	0.075026	0.014919	0.042868	0.013875	0.031014	0.142848	0.200196
1700048O20Rik	0.059998	0.149595	0.060005	0	0.028571	0.027742	0.155027	0.228496	0.200142
Snhg10	0.523989	2.299415	0.83848	0.729476	0.399233	0.484569	0.433255	0.199556	0.199763
Gm19911	0.104732	0.139271	0.139659	0.243005	0.132994	0.129137	0.396901	0.299145	0.199638
Gm46183	0	0	0	0.416581	0.398982	0	0	0	0.199638
Tmem238l	0	0	0	0	0	0	0	0	0.199575
Ano1	0.153529	0.222721	0.209383	1.110323	0.531708	0.141979	0.15868	0.358793	0.199537
Gm42274	0.023254	0.115962	0	0	0.132882	0	0.096138	0.02214	0.19947
Adam3	0.18305	0.02608	0.183071	0.390053	0.323765	0.193462	0.243246	0.199179	0.199386
Higd1b	0	1.043216	0.104612	1.040142	0	0.483655	0.216219	0	0.199386
Kcp	0.229947	0.396123	0.648107	0.415743	0.238908	0.560616	0.453717	0.13932	0.199236
Lrrc15	0.062713	0.114667	0.167253	0.218265	0.159272	0.028997	0	0.218932	0.199236
Arhgap20	0.104518	0.173733	0.087108	0.268492	0.165903	0.10471	0.018004	0.03317	0.19923
4931406B18Rik	0.069558	0.231244	0.115944	0.276675	0.331233	0.021442	0.047928	0.44151	0.198886
Fbxl21	0.347356	0.485004	0.069479	0	0.198491	0.385469	0.287208	1.256725	0.198637
Foxc1	0.263991	0.124715	0.097271	0.77372	0.489611	0.269828	0.201046	0.291031	0.198637
Gm39397	0.208414	0.103929	0.208438	0	0.595473	0.385469	0	0.099215	0.198637
Slc24a1	0.439893	0.230906	0.162085	0.09209	0.330749	0.128463	0.502509	0.220432	0.198595
4921522P10Rik	0.036771	0.232262	0.1471	0.121883	0.163427	0.249366	0.164686	0.151707	0.198593
4930471E19Rik	0.069428	0	0.104154	0.034519	0.165306	0.032102	0.179393	0.033051	0.198512
LOC118568085	0.109681	0.194221	0.199239	0.247069	0.185468	0.111779	0.277618	0.14705	0.198404
Ehf	0.143016	0.168569	0.351079	0.206858	0.433384	0.312606	0.524067	0.210437	0.198264
Gm15464	0.208023	0.103734	0.31207	0.103429	0.297178	0.480932	0	0.396116	0.198264
Gm8801	0.178162	0.236917	0.089091	0.354328	0.08484	0.137299	0.153449	0.028271	0.198105
Gm35455	0	0.082832	0.166126	0.165176	0.118649	0.230415	0	0	0.197893
Gm33958	0	0	0	0	0	0	0	0	0.197893
Platr6	0.103623	0.034449	0.034545	0.137389	0.36186	0.223596	0.178498	0.26309	0.197523
Gm12248	0.155289	0	0	0	0.098597	0	0.0535	0.246417	0.197339
D630010B17Rik	0	0	0	0	0	0	0	0	0.197339
Adamtsl3	0.121735	0.291386	0.097399	0.61737	0.115939	0.258926	0.050328	0.046361	0.197241
Gm18752	0	0.020638	0.041392	0.082311	0.078833	0.076547	0.19249	0.137916	0.197228
Tnfsf4	0.034455	0.412359	0.241213	0	0.525033	0.44608	0.142444	0.098414	0.197032
Gm14379	0.02952	0.235529	0.324755	0.264189	0.1968	0.081897	0.03051	0.112423	0.196945
E330033B04Rik	0.082595	0.131801	0.239555	0.221759	0.180925	0.305527	0.324393	0.204461	0.196802
Gm35658	0.206396	0.192124	0.371557	0.492577	0.458662	0.343564	0.597299	0.20961	0.196714
Gm42113	0.03437	0	0	0	0	0	0	0.065446	0.196543
Gm31365	0.13745	0.068542	0.091644	0.04556	0.196358	0.084739	0.047354	0.043622	0.196503
Gm2164	0.085897	0.119936	0.085907	0.102499	0.196338	0.238305	0.177558	0.098139	0.196482
Dusp15	0	0.10277	0	0.40987	1.373941	1.334096	0	0	0.196422
Gm5134	0.288311	0.575087	0	0.204783	0.156905	0.609419	0.425691	0.470572	0.196276
1700029J07Rik	0.123562	0.657242	0.20596	0.081913	0.235358	0.30471	0.297984	0.039214	0.196276
Gm14730	0.164749	0.123233	0.20596	0.040957	0.039226	0	0.212845	0.078429	0.196276
Prss21	0	0.359362	0	0.307117	0.098048	0	0.212806	0.196035	0.196239
Gm38649	1.893208	0.820942	1.152521	0.654818	0.548761	0.91345	2.0418	0.97963	0.19613
Gm42814	0.308561	0.512899	0.720059	0.204555	0.097957	1.141389	0.318913	0.489633	0.196057
Npas1	0.411305	0.263707	0.264441	0.146072	0.223841	0.760724	0.121458	0.615376	0.196005
Elapor2	0.239843	0.205033	0.102802	0.136286	0.16316	0.063371	0.318716	0.16311	0.195936
Gnat2	0.171317	0	0.068535	0	0.065264	0.031686	0.141651	0.293598	0.195936
Slc4a5	0.324579	0.280553	0.367899	0.333519	0.164867	0.230123	0.011182	0.19572	0.195923
Arhgef5	0.400757	0.286957	0.452189	0.960517	0.626341	0.427624	0.55227	0.24459	0.195875
Ccr1l1	0.359654	0.409939	0.077078	0.791915	0.856326	0.332597	0.292066	0.171213	0.195875

4632428C04Rik	0.15983	0.387124	0.319696	0.249754	0.565389	0.211151	0.589971	0.152174	0.195855
Tbc1d22bos	0.082191	0.163945	0.369904	0.490386	0	0	0.38227	0.039127	0.195839
Tpbg	0.14223	0.220658	0.45835	0.220007	0.090305	0.13153	0.212336	0.165509	0.195806
Fbxo24	0.33381	0.307312	0.282487	0.357474	0.317917	0.474918	0.371548	0.195581	0.195785
Asphd1	0.615978	0.409559	0.8214	0.102088	0.097775	0.949396	0.954967	0.195491	0.195694
Gm41603	0.102663	0.20478	0.102675	0.408352	0.879977	0.189879	0.636644	0.195491	0.195694
AI463170	0	0.10239	0	0	0.097775	0	0.318322	0	0.195694
Gm36885	0.0342	0	0.171019	0.170042	0.162858	0.031627	0	0	0.195573
Chrdl1	0.040964	0.285985	0.327751	0.366612	0.351124	0.252549	0.310482	0.455021	0.195212
Gm41810	0.021559	0.118257	0.107806	0.50379	0.225854	0.169462	0.144833	0.164208	0.195199
Dcbld1	0.368631	0.224675	0.143373	0.203648	0.312072	0.246205	0.021167	0.194985	0.195188
Gm38529	0.531878	0.306036	0.040918	0.589926	0.214312	0.208096	0.296004	0.175292	0.194972
Hspe1-ps5	0.102284	0.714084	0.613777	0.915402	0.584486	0.378357	0.634295	0.097385	0.194972
Npnt	0.217304	0.216725	0.434658	0.584705	0.24348	0.330986	0.224594	0.352938	0.194927
Plce1	0.348429	0.317284	0.333319	0.459459	0.281342	0.217146	0.203545	0.223563	0.194919
LOC118568637	0.2989	0.219656	0.314668	0.344156	0.299652	0.290961	0.113816	0.329516	0.194917
Zfp248	0.188618	0.391909	0.36156	0.375124	0.613763	0.130821	0.422384	0.508819	0.194751
Bbox1	0	0	0	0	0	0	0	0	0.194732
Pla2g4f	0.09077	0.045264	0.204257	0.338483	0.086449	0.104927	0.164178	0.453717	0.194653
Gm13547	0.612575	0.814593	0.306323	0.203048	0.58341	0.094415	0.211042	0.194411	0.194613
Tmem198	0.612012	0.534085	0.102014	0.329649	0.097146	0.448059	0.342628	0.121395	0.194434
Kcnq1ot1	0.248497	0.259101	0.194036	0.313176	0.191104	0.191092	0.303531	0.206861	0.194411
Kcna1	0.15451	0.17259	0.123622	0.3626	0.206015	0.377218	0.159693	0.141224	0.194385
Gm35715	0.254888	0.101684	0.229426	0.050692	0.169927	0.117856	0.079032	0.072803	0.194344
Gm25395	0.815266	0	0	0	0	0	1.263927	0	0.194255
Gm2396	0.713357	0.66064	0.40768	0.456018	0.727922	0.047121	0.105327	0.388106	0.194255
Gm16299	0.040763	0.08131	0.326144	0.24321	0	0.150786	0.084262	0.077621	0.194255
Gm18676	0	0	0	0	0	0	0	0	0.194255
Gm33444	0.25991	0.175147	0.16861	0.405144	0.200704	0.298822	0.392054	0.347781	0.194157
Epb42	0	0	0	0	0	0	0	0	0.194145
A430088P11Rik	0.063634	0.012693	0	0.265766	0.048484	0.058847	0.118384	0.109055	0.194077
Gvin-ps4	0.139963	0.02538	0.063627	0.037958	0.072709	0.152967	0.118357	0.133258	0.194032
Gm8407	0.040674	0.101413	0.040678	0	0.13558	0.112841	0.147134	0.251714	0.193828
Gm34407	0.28157	0.187214	0.219025	0.295548	0.283062	0.202524	0.323352	0.059574	0.193817
Gm7370	0	0.067592	0.203342	0.067393	0	0	0.070046	0	0.19378
LOC118568357	0.084677	0.084452	0.055046	0.063152	0.076613	0.11746	0.148781	0.044341	0.193691
Gm39318	0.06769	0.135019	0	0.201932	0	0.25039	0.629648	0.064447	0.193544
Gm29010	0.304605	0.101265	0.101547	0	0	0.093896	0	0.096671	0.193544
Wnt4	0.11601	0.115701	0.464092	0.273979	0.621485	0.120692	0.149877	0.138066	0.193493
Gm19653	0.217994	0.232407	0.112768	0.209298	0.128864	0.187691	0.512769	0.114511	0.193439
Kazn	0.347847	0.101185	0.376878	0.691796	0.289875	0.562936	0.074899	0.524375	0.193392
Gm38921	0	1.415113	0.405444	0.100782	0	0.187449	0.733247	0	0.19319
Gm4199	0.040525	0.282919	0	0.161192	0.038595	0.112428	0.041885	0.231502	0.193119
Itgb6	0.011917	0.011885	0.023837	0.071102	0	0.044082	0.061584	0.011346	0.193086
Tdrp	0.292609	0.044897	0.112555	0.044765	0.021437	0.12489	0	0	0.193072
Kirrel3os	0.178717	0.095062	0.166822	0.082934	0.158861	0.044073	0.110828	0.045375	0.193044
5330438D12Rik	0.202476	0.242325	0.081	0.402685	0.115702	0.149795	0.125562	0.038555	0.192978
LOC118568238	0	0	0	0	0	0	0	0	0.192837
Serpinf2	0.379366	0.554923	0.177058	0.528138	0.216783	0.327438	0.209117	0.505671	0.192837
Gm6153	0.101072	0.201606	0.101084	0	0.09626	0.093468	0.104463	0.144346	0.192661
Hmgcs2	0.38709	0.083926	0.286143	0.317979	0.160287	0.264586	0.017395	0.304453	0.192486
Syngr4	0.740071	0.3355	0.807444	0.267609	0.32038	0.186653	0.486755	0.064056	0.192369
Gm6190	0.20174	0	0.040353	0.280853	0.115281	0.14925	0.25021	0.07683	0.192276
Ccnd3-ps	0.14407	0.028737	0.230538	0.573051	0.164653	0.106585	0.238245	0.411506	0.192236

Tmem217	0.067198	0.067019	0.403233	0	0	0	0	0.063979	0.192136
Gm38630	0	0	0	0	0	0	0	0	0.192136
E530011L22Rik	0.201349	0.016734	0.134248	0.083425	0.111861	0.155168	0.26013	0.271581	0.191903
Efcc1	0.744811	1.084127	1.207941	0.540466	0.153373	0.130309	0.728188	0.191657	0.191857
Gpld1	0.241473	0.140484	0.140875	0.04002	0.076659	0.428004	0.083191	0.440652	0.191787
Gm33666	0.301841	0.100346	0.100625	0	0.095823	0	0.103989	0.287382	0.191787
Spry4	0.248389	0.389286	0.283906	0.517519	0.383007	0.601601	0.403421	0.236491	0.191644
Gm31024	0.080403	0.962271	0.321651	0.359788	0.114863	0.446127	0.373954	0.114828	0.191579
Pm20d2	0.301476	0.133633	0.167506	0	0.063805	0.185864	0.553938	0	0.191556
Gm15050	0.703019	0.901475	1.205315	0.599212	0.191299	0.185751	1.038007	1.529927	0.19144
Fat3	0.317984	0.200298	0.2427	0.232992	0.255027	0.162508	0.311355	0.342588	0.191411
Cyp26b1	0.401041	0.458793	0.247731	0.304961	0.112337	0.207251	0.10972	0.303218	0.191114
Gm19557	0.501248	0.419928	0.360941	0.83738	0.477383	0.72312	0.600956	0.305432	0.191094
E030024N20Rik	0.156876	0.147766	0.252773	0.181996	0.107905	0.128954	0.252216	0.157659	0.191049
Gm34312	0.066793	0	0.066801	0	0	0.123536	0.069034	0	0.190979
2310034O05Rik	0	0	0	0.099598	0	0.046312	0.155279	0.238403	0.190921
Gm4879	0.120169	0.159799	0.480733	0.039832	0.190746	0.296343	0.496804	0.076275	0.190887
Gm34254	0.300387	0.199725	0.06676	0.132757	0.158936	0.030865	0.034496	0.222443	0.190864
Gm39098	0	0.399209	0.400321	0	0	0	0	0	0.190749
Btf3-ps18	0.150035	0.399029	0.300106	0.745975	0.238153	0.046249	0.284293	0.214273	0.190663
Gm35558	0.172545	0.168175	0.258848	0.350957	0.224087	0.101541	0.255342	0.272556	0.190614
Cd209f	0.099978	0.598274	0.39996	0.099418	1.380667	0.739656	1.653324	1.094677	0.190577
Gm30992	0.173161	0.1727	0.093251	0.052982	0.038058	0.098544	0.110136	0.152184	0.190428
Gm8983	0.59933	0.39849	0.4995	0.943624	0.951326	1.847473	0.671058	1.759416	0.190405
Gm32497	0.162319	0.112075	0.106144	0.279362	0.047566	0.028867	0.316171	0.053496	0.190405
Myadml2	0.049944	0.39849	0.0999	0.347651	0.237832	0.13856	0.154859	0.095104	0.190405
Vegfd	0.279587	0.278842	0.63913	0.278021	0.190197	0.627915	0.454091	0.038028	0.190336
4930519G04Rik	0.399314	0.2655	0.06656	0	1.014139	0.123091	0	0.190093	0.190291
Gm20590	0.131965	0.206701	0.22589	0.266658	0.156297	0.201831	0.215955	0.212628	0.190274
Gm12743	0.349295	0.199066	0.748576	1.290113	0.047523	0.276872	0.46416	1.092706	0.190234
C030005K06Rik	0.365818	0.099503	0.19956	0.396839	0.158364	0.184526	0.515579	0.253305	0.190177
LOC118567762	0.398835	0.198887	2.193847	1.189803	0.189923	0.73766	0.412216	1.89865	0.190063
Fam171b	0.125876	0.251083	0.157364	0.385945	0.099903	0.029102	0.271041	0.089885	0.189955
Gm14137	0.489159	0.180688	0.380502	0.090078	0.258817	0.268065	0.131074	0.086246	0.189938
Omg	0.04981	0.124192	0.174354	0.495306	0.189752	0.437592	0.205923	0.403101	0.189892
Gm40061	0	0.397059	0	0	0.189582	0	0.205738	0.094762	0.189721
Tshb	0	0.397059	0	0	0	0	0	0	0.189721
Zfp982	0.199008	0.170125	0.071083	0.113082	0.203072	0.328637	0.117534	0.257145	0.189673
Epb41l5	0.119307	0.178484	0.517058	0.197731	0.113627	0.110332	0.041103	0.18932	0.189517
Paqr8	0.491713	0.646916	1.07771	0.624203	0.787147	0.59017	0.594714	0.517963	0.189453
Gm8731	0.099351	0.396347	0.099363	0.098795	0.094621	0	0	0.094592	0.189381
Gm30363	0.024827	0.222846	0.173807	0	0.047289	0.091836	0.051319	0.023637	0.189297
Gm29868	0.167202	0.229291	0.156771	0.155874	0.139337	0.057984	0.334823	0.099496	0.189239
Clec11a	0.481824	0.819746	0.34015	0.732779	0.674829	0.995992	0.146467	0.593668	0.189091
Smco1	0.039655	0.197748	0	0.039433	0.188836	0	0.040986	0	0.188975
Gm36660	0.759878	0.362452	0.297378	0.624209	0.346117	0.274974	0.136586	0.503289	0.18893
Dll4	0.28049	0.51012	0.280523	0.738315	1.005688	0.564552	0.102318	0.361309	0.188705
Ccdc154	0.079197	0.236959	0.039603	0.236261	0.263993	0.073239	0.081854	0.075404	0.188705
Gm51547	0	0.592398	0	0	0	1.64788	0	0.377018	0.188705
Gm10603	0.149897	0.179398	0.29983	0.172907	0.239838	0.249517	0.452385	0.102756	0.188583
Ankrd42	0.254173	0.394328	0.282448	0.477416	0.242072	0.182818	0.525402	0.215109	0.188417
Gm7582	0.065893	0.109529	0.021967	0.043683	0.14643	0.121872	0.022701	0.167298	0.188406
Kcnk7	0.29646	0.295671	0.296495	0.687866	0.847036	0.685393	0.561745	0.470432	0.188369
Trim63	0.157943	0.472568	0.434394	0.471175	0.150423	0.511213	0.204053	0.150377	0.188167

Gm45857	0.032881	0.59029	0.131541	0.032697	0.344475	0.182446	0.475784	0.219144	0.188033
Gm42004	0.263051	0	0	0	0.062632	0.364892	0.203907	0.125225	0.188033
Gvin-ps6	0.053806	0	0.197311	0.160514	0.102488	0.116102	0.018537	0.051229	0.188033
Gm34818	0.197288	0	0	0	0	0	0	0	0.188033
Gm46946	0.023206	0.219866	0.081229	0.092302	0.022101	0.053649	0.095936	0.077329	0.187994
Kank1	0.34505	0.393293	0.279359	1.274436	1.017163	0.668573	0.339644	0.594468	0.187922
Thsd4	0.007291	0.123611	0.102081	0.297242	0.131927	0.074164	0.037676	0.180477	0.187613
LOC118567790	0.138885	0.057714	0.057875	0.057544	0.099204	0.053515	0.17943	0.16529	0.187523
Gm16183	0.538234	0.43357	0.289852	0.504341	0.305593	0.248871	0.66327	0.384338	0.187437
Gm42377	0.294883	0.098033	0.294918	0	0.093615	0.727197	0	0.561516	0.187367
Nat8f4	0.343726	0.48973	1.129516	0.244143	0.935316	0.862781	1.573284	0.374012	0.187201
Gm30494	0	0.048973	0	0	0.046766	0	0.050751	0.093503	0.187201
Gm32296	0.056111	0	0.028059	0	0.02672	0.05189	0	0.026712	0.187177
A830052D11Rik	0.245301	0.097859	0.294396	0.487854	0.093449	0.090739	0.304237	0	0.187035
Selenbp2	0.196241	0.097859	0.196264	0.162618	0.467244	0.24197	0.135217	0.12456	0.187035
Gm51810	0.353109	0.469558	0.255053	0.487682	0.242881	0.145131	0.425781	0.541646	0.186969
Gm38729	0.431577	0.11739	0.35315	0.312116	0.186832	0.253979	0.243304	0.597678	0.186969
Gm26511	0.140066	0.223509	0.168099	0.027856	0.160077	0.207246	0.086859	0.400069	0.186893
6330549D23Rik	0.11762	0.019551	0.17645	0.019493	0.242709	0.543855	0.283653	0.298628	0.186837
Sgca	0.274446	0.938452	0.745011	0.935686	1.194876	0.398827	0.081044	0.522599	0.186837
Ppfia3	0.235156	0.371339	0.548761	0.350758	0.186633	0.217465	0.405076	0.335838	0.186771
Gm46922	0.065298	0.195373	0.261223	0	0.062189	0.181157	0	0	0.186705
Sidt1	0.026082	0.026013	0.130427	0.181554	0.173884	0.096481	0.080872	0.148998	0.186441
Mia	0	0.195028	0	0.388907	0.465596	0	0	0	0.186375
Gm38956	0.039096	0.038992	0	0	0	0	0.040408	0.148893	0.18631
Ssu2	0.048844	0.048714	0.146549	0.777128	0.186074	0.361356	0	0.046504	0.186211
Gm2446	0	0.064933	0.097671	0	0.062007	0.090312	0	0.030994	0.186156
Gm8655	0	0	0.097614	0.048528	0.092955	0.270778	0.050438	0.046463	0.186047
Gm16090	0	0	0	0	0	0	0	0	0.186047
Arhgef28	0.169098	0.038919	0.052036	0.375105	0.272541	0.264637	0.053776	0.074307	0.18596
Actl7b	0.156054	0.03891	0	0.038795	0.371559	0.180392	0.040322	0	0.185916
A930018O16Rik	0.097516	0	0	0.19394	0.046437	0	0	0.092845	0.185884
Itga2	0.241781	0.108901	0.132605	0.411052	0.185701	0.425545	0.169282	0.14109	0.185838
Gm35478	0.129984	0.064819	0.259998	0	0.309488	0.661127	0.470206	0.061879	0.185829
Gm31834	0.103577	0.103301	0.115776	0.236287	0.110251	0.05071	0.04408	0.046407	0.185822
Slc7a3	0.024347	0.121411	0.267848	0.290527	0.092751	0	0.301966	0.046361	0.185639
Atp6v0e2	0.194747	0.420829	0.097385	0.322761	0.309125	0.180096	0.201281	0.092709	0.185611
LOC115487771	0.218638	0.19649	0.242693	0.461108	0.201364	0.153309	0.444498	0.333979	0.185483
Gm17825	1.167117	1.228677	2.010269	1.805346	2.161346	1.61897	1.474335	1.296412	0.185394
Gm18614	1.361637	0.291002	0.778169	0.483575	0.463146	0.089943	1.105751	0.185202	0.185394
Gm8130	0	0	0.097271	0	0	0	0	0	0.185394
Stpg3	0	0	0.038772	0	0	0.143405	0.040069	0.110732	0.184746
Gm33394	0	0	0.193862	0	0.184611	0	0	0.184554	0.184746
Lrrc3	0.425851	0.077221	0.309746	0.487627	0.368705	0.131271	0.1067	0.073719	0.184488
Gm9354	0.289746	0.288975	0.482966	0.192082	0.643885	0.44658	0.199645	0.643689	0.184102
Zfp462	0.371469	0.125963	0.326931	0.140368	0.155665	0.371005	0.184287	0.113176	0.184102
Cyp2j9	0.225358	0.770599	0.676153	0.96041	0.306612	0.327492	0.133096	0.275867	0.184102
Gm7105	0.578484	0.224367	0.353559	0.255664	0.336687	0.386363	0.26573	0.21419	0.183782
Cbx3-ps7	0.032138	0.160262	0.160709	0	0.061216	0	0.033216	0.122394	0.183782
LOC108168645	0	0.307597	0.11567	0.191681	0	0.071304	0.278919	0.036705	0.183718
Tafa3	0.218311	0.217729	0.462359	0.229858	0.085613	0.142508	0.066363	0.305666	0.183591
Phf20-ps	0.122573	0.034928	0.35025	0.139299	0.166768	0.161931	0.271469	0.233403	0.183579
LOC108167736	0.384987	0.09599	0.096258	0.861368	0.549985	0.178012	1.591611	1.191271	0.183463
4930598A11Rik	0	0.191981	0.192516	0	0	0	0.198951	0	0.183463

Gm5586	0.064164	0.191981	0.192516	0.063805	0	0.593373	0.132634	0.488727	0.183463
Gm35391	0	0	0	0	0	0.178012	0	0	0.183463
LOC118567379	0.222846	0.161639	0.172219	0.120872	0.057883	0.103041	0.324546	0.212171	0.18343
4933433G15Rik	0.048082	0	0.240436	0.286874	0	0.133393	0.099389	0.27467	0.183304
Thnsl2	0.329661	0.493176	0.357175	0.355133	0.130819	0.330266	0.255541	0.104623	0.183281
Hyal5	0	0	0.027475	0	0	0	0.056787	0	0.183281
Gm5566	0.09608	0.47912	0.096091	0.57325	0.274516	0.355406	0.198607	0.731819	0.183145
Gm807	0.576479	0.543003	0.704667	0.286625	0.061004	0.296172	0.198607	1.067237	0.183145
Dmtn	0.554807	0.468203	0.362801	0.827549	0.812912	0.374935	0.99246	0.081266	0.183039
Podxl2	0.137087	0.218756	0.383889	0.436222	0.339457	0.304257	0.028337	0.052208	0.182919
Gm46178	0	0.382633	0.19185	0	0.182694	0.177396	0.396526	0.182638	0.182828
LOC118567952	0	0	0.19185	0	0.182694	0	0.198263	0	0.182828
4930566D17Rik	0.038365	0.076527	0.07674	0.381505	0	0.141917	0.198263	0.036528	0.182828
Gm40687	0	0	0	0	0	0	0	0	0.182828
Gm41576	0	0	0	0.381505	0.182694	0	0	0	0.182828
Gm5782	0.156802	0.017376	0	0.069299	0.199115	0.064447	0.144055	0.033176	0.182656
Pcdhgb2	0.143715	0.214999	0.239553	0.059546	0.11406	0.143978	0.160915	0.068415	0.182631
Scn3b	0.462915	0.286562	0.303324	0.30159	0.228039	0.398566	0.478446	0.182375	0.182565
Gm5469	0.446053	0.254209	0.573564	0.50692	0.364128	0.88392	0.526878	0.303348	0.182198
Dlec1	0.044068	0.175804	0.190985	0.116857	0.05596	0.135844	0.075912	0.125872	0.182005
Gm39101	0	0	0	0	0	0	0.032892	0.0303	0.181989
Tagln3	0.286009	0.38033	0	0.331808	0.272392	0.132246	0.640476	0.045385	0.181728
Slc22a13b	0.076204	0.038	0.076212	0.151553	0.036288	0.317118	0.275661	0.362766	0.181572
Gm39272	0.282582	0.373856	0.294151	0.407163	0.247159	0.234658	0.476838	0.362389	0.181383
Gm32679	0.063358	0	0	0.063003	0	0.117183	0.261934	0	0.181157
Gm33258	0.047505	0.047378	0.04751	0.047239	0	0	0.196394	0.045229	0.181105
Myo15	0.119996	0.009973	0.050004	0.009944	0.123806	0.083226	0.062011	0.114248	0.181081
Nhsl1	0.164069	0.284203	0.25045	0.240432	0.279619	0.27151	0.160649	0.139767	0.181063
Apol7c	0.488202	0.568053	0.406882	1.132757	1.239889	0.627047	0.504581	0.748871	0.18095
Gm10693	0.306691	0.509791	0.759514	0.551856	0.111272	0.378158	0.407546	0.347619	0.18095
Gm40746	0.379713	0.568053	0.569635	0.943964	0.904086	0	0.392452	0.723048	0.18095
Vmn1r-ps145	0.759426	0.094675	0.094939	0.094396	0.090409	0	0.098113	0.180762	0.18095
Exoc3l	0.403272	0.165611	0.284696	0.212301	0.135555	0.109686	0.318731	0.519468	0.180873
Gm34415	0.320873	0.261833	0.233389	0.130531	0.152798	0.148367	0.286415	0.291616	0.180712
Slc35f1	0.111478	0.222363	0.011149	0.077598	0.244192	0.061855	0.011522	0.010614	0.180623
Gm31559	0	0.047216	0	0	0	0.175123	0	0	0.180486
Gm52129	0.086652	0.12163	0.073823	0.201056	0.181864	0.123168	0.174143	0.123752	0.180468
Tmem51os1	0.094604	0	0.157692	0.313581	0	0.058325	0.097778	0.030024	0.180332
Gm9529	0	0	0	0.188149	0	0	0	0.045036	0.180332
LOC115486422	0	0	0.567691	0	1.0812	0.174974	0	0.72058	0.180332
Axin2	0.067525	0.296319	0.337664	0.362593	0.668825	0.961654	0.739779	0.565756	0.180201
Slc16a14	0.33041	0.870901	0.495672	0.093874	0.157339	0.152776	0.512243	0.337053	0.179949
Mir133a-1hg	0.247807	0.317761	0.34225	0.281622	0.202294	0.054563	0.256121	0.337053	0.179949
Gm2011	0.282847	0.470157	0.28288	0	0.179587	0.087189	0	0.718129	0.179719
BC106179	0.10464	0.271339	0.669771	0.083243	0.079726	0.212888	0.15141	0.099627	0.179516
Tmem74	0.376617	0.037561	0.150664	0.411958	0.107606	0.20897	0.077851	0.071715	0.179475
Rem2	0.037611	0.375105	0.037615	0	0.21492	0.31303	0.194362	0.035809	0.179231
Gm6724	0.281888	0.421707	0.375895	0.373746	0.58168	0.738598	0.437019	0.805156	0.17911
2210418O10Rik	0	0.187425	0.072287	0.158123	0.137676	0.173788	0.059763	0.013763	0.17911
1110020A21Rik	0.187608	0.374216	0.750518	0	0.089338	1.127709	0	0.357242	0.178807
Gm8000	0.600141	0.336681	0.150053	0.037299	0.142892	0.173435	0.038767	0.249985	0.178746
4930432K21Rik	0.140646	0.187029	0.140663	0.279717	0.200925	0.281808	0.314957	0.468682	0.178731
Gm36090	0	0	0.062508	0	0	0	0	0	0.178706
LOC115488002	0.093677	0.224226	0.018738	0.298087	0.178434	0.138607	0.348552	0.249731	0.178565

Gm19945	0	0.186792	0.093656	0.186242	0.089187	0.346401	0.580723	0	0.178505
Gm46762	0.156076	0.217924	0.530719	0	0.178374	0.0866	0.12905	0.08916	0.178505
Gm41541	0	0	0	0	0	0	0	0.178319	0.178505
Gm9525	0.046823	0	0.046828	0	0	0.0866	0	0.08916	0.178505
Rnf17	0.093593	0.077786	0.109204	0.946196	0.193129	0.158678	0.112855	0.074258	0.178404
LOC118568677	0.093487	0.65267	0.186997	0	0.089036	0.60518	0	0.712074	0.178204
Gm44040	0.242931	0.130461	0.21181	0.148658	0.100851	0.063364	0.173825	0.106751	0.178104
D330041H03Rik	0.410929	0.48435	0.672507	0.260035	0.533679	0.483655	0.540547	0.46238	0.178024
Gm6189	0.026666	0.053189	0.400033	0.053033	0	0.221937	0.137802	0.025388	0.177904
Gm31813	0.06222	0.103424	0.103712	0.020624	0.039505	0.172617	0.042872	0.177719	0.177904
Gm32117	0.103623	0.082677	0.352358	0	0.157902	0.038331	0.04284	0.019732	0.177771
Gm8170	0	0.139505	0.046631	0	0	0.172472	0	0.044392	0.177754
Gm43380	0.23148	0.266843	0.447982	0.538092	0.569759	0.236306	0.546849	0.26905	0.177643
Ankrd7	0.232933	0.092925	0.23296	0.370605	0.354949	0.258491	0.096299	0.26613	0.177605
Fam13c	0.304605	0.421936	0.338489	0.673108	0.483504	0.547728	0.367295	0.612251	0.177415
Gramd1c	0.13538	0.084387	0.15232	0.117794	0.177285	0.203442	0.157412	0.338349	0.177415
Zfp433	0.209288	0.3015	0.139542	0.23124	0.442941	0.193543	0	0.177122	0.177307
Fat4	0.234018	0.19564	0.196185	0.615988	0.170434	0.162309	0.170731	0.101574	0.17712
Wasf3	0.206473	0.061777	0.061949	0.287443	0.078657	0.229128	0.25608	0.098291	0.177109
Usp29	0.046411	0.092575	0.092833	0.046151	0.132604	0.04292	0.023984	0.198846	0.176936
Gm34464	0.092706	0.462295	0	0	0	0	0.095816	0.088265	0.176714
1700108I11Rik	0	0	0	0	0.176584	0	0	0	0.176714
Gm8885	0	0.092305	0.092562	0.092033	0.088145	0.171177	0.478281	0	0.176419
Gm40816	0.092551	0.184609	0.092562	0	0.352578	1.369413	0.191312	0	0.176419
Tmem255a	0.03085	0.153841	0	0.736261	0.205671	0.085588	0	0	0.176419
C130036L24Rik	0.030816	0.399542	0.061639	0	0.322837	0.256479	0.222949	0.11736	0.176223
Scin	0.061621	0.061457	0.349224	0.04085	0.156498	0.15196	0.042459	0.019556	0.17619
Nebl	0	0.020486	0.061628	0.081701	0.117374	0.07598	0	0.019556	0.17619
Cycl	0	0	0	0	0.527986	0.170891	0.57298	0.351883	0.176125
Gm10126	0.739174	0.737206	0.73926	0.735034	1.407963	0.170891	0.190993	0.175942	0.176125
Gm29770	0.092397	0.092151	0.277223	0.275638	0.175995	0.085446	0	0	0.176125
Actl10	0.147736	0	0	0.146909	0.070351	0.034155	0.229039	0	0.176007
Enpep	0.382059	0.144533	0.144936	0.235812	0.777926	0.243666	0.313178	0.012543	0.17579
Chl1	0.13275	0.205951	0.236028	0.132007	0.168573	0.156864	0.053357	0.105326	0.175726
Ptch2	0.086724	0.162174	0.151784	0.247934	0.23746	0.280697	0.033612	0.330277	0.175643
Asb14	0.276422	0.275687	0.21502	0.152708	0.409518	0.142015	0.444417	0.263181	0.175637
Nctc1	0.263239	0.341299	0	0	0.025071	0.07303	0	0.17544	0.175623
Ccnjl	0.143251	0.449018	0.102334	0.203498	0.03898	0.075699	0.21151	0.058452	0.17554
Tex14	0.198246	0.3107	0.184107	0.25346	0.337156	0.248807	0.526878	0.606695	0.17545
Gm15428	0.858554	0.428134	0.551992	0.060982	0.233622	0.226847	0.253531	0.233551	0.175345
Gm11843	0	0.228946	0.13775	0.022827	0	0.106144	0.142355	0.021856	0.175031
Gm12984	0	0.018308	0	0	0.017483	0.016976	0.018973	0.069911	0.174958
Gm7693	0	0.18293	0.229299	0.045598	0.087343	0.08481	0.094786	0.21829	0.174814
Gm14111	0.275127	0.137197	0.04586	0.227988	0.480384	0.381643	0.047393	0.087316	0.174814
Tg	0.814187	1.302157	0.447488	0.145879	0.272446	0.142447	0.250177	0.174591	0.174773
Gm16010	0.076403	0.06096	0.068771	0.068378	0.065489	0.091852	0.102656	0.087292	0.174765
Gm7653	0.091633	0	0.458219	0.911199	0.349082	0.338958	0.37883	0.174488	0.174669
Gm39355	0.137336	0.182627	0.274704	0.728357	0.043599	0.08467	0.283888	0.871717	0.174525
Gm32663	0	0.912384	0	0.09097	0.087126	0.0846	0.094551	0.348399	0.174381
Gm51809	0	0	0	0	0	0	0	0	0.174381
Gm7251	0.091407	0.045582	0.091417	0.136342	0.174109	0.042265	0.188946	0.21757	0.174237
Gm32838	0.045703	0	0	0.090895	0.087055	0.042265	0	0	0.174237
1500026H17Rik	0.018266	0.018218	0.054805	0.145311	0.034793	0.168921	0.151033	0	0.174094
Gm52344	0	0.273264	0	0	0.434914	0	0.094395	0	0.174094

4933427E11Rik	0	0.182176	0	0	0	0.168921	0	0	0.174094
Syt7	0.324377	0.545929	0.456208	0.68544	0.617866	0.318722	0.188584	0.096512	0.173903
Gm12017	0.045591	0.090938	0	0.09067	0.04342	0	0.04712	0.043407	0.173807
Tnfsf15	0.220716	0.066996	0.134364	0.143139	0.127952	0.257357	0.049591	0.13705	0.173777
Plaat1	0.020255	0.040402	0	0.281982	0	0.093656	0.020935	0.096424	0.173744
Hes5	0	0	0	0	0	0.042126	0.047081	0.043371	0.173664
LOC118567441	0.252119	0.474956	0.182107	0.417844	0.280134	0.194293	0.434295	0.253377	0.173544
E030007A22Rik	0.016534	0.082449	0.198429	0.197295	0	0	0.11962	0.110193	0.173341
Lmtk3	0.170474	0.17002	0.113662	0.113013	0.194829	0.210198	0.070477	0.238051	0.173308
Gm3809	0.090808	0.271698	0	0.135448	0.129726	0.41988	0	0.216144	0.173095
Gm17925	0	0.090492	0.181488	0.18045	0.086413	0.419537	0.468887	0.518322	0.172954
Gm11694	0.151222	0.241311	0.15124	0.2406	0.432067	0.251722	0.218814	0.172774	0.172954
Gm4107	0.181407	0.07237	0	0.036078	0.034554	0	0.037499	0	0.172897
Gm51905	0.11337	0.361819	0.317474	0.541129	0.107973	0.146778	0.234348	0.086352	0.172883
Gm51667	0.317307	0.542507	0.09067	0.090151	0.172686	0.167677	0.093701	0.47474	0.172812
Gm5966	0.04533	0.090418	0	0	0	0.167677	0.093701	0.086316	0.172812
Gm39860	0.802887	0.477867	0.518052	1.017303	0.38233	0.311365	0.762903	0.382214	0.172792
LOC118568217	0.077644	0.051625	0.155307	0.154419	0.098597	0.047869	0.534995	0.073925	0.172671
Olfm2	0.36234	0.210802	0	0	0.316332	0.223387	0.093624	0.086246	0.172671
Gm30474	0.241166	0.27059	0.331642	0.014988	0.014355	0.153328	0.249257	0	0.17239
Tbrg3	0.041688	0.152449	0.187618	0.248727	0.178664	0.019276	0.12926	0.099228	0.172174
Cisd3b	0.36116	0.540298	0	0.269353	0.68793	0.250492	0.279958	0	0.172109
Atp5k-ps1	0.18058	0.180099	0	0.718274	0.171982	0.166995	0	0	0.172109
Rapgef4	0.105338	0.105058	0.180601	0.404029	0.057327	0.417487	0.264404	0.17193	0.172109
Gm34402	0.156435	0.624073	0.361045	0.358981	0.458421	0.278204	0.547235	0.34371	0.172034
Lamc2	0.496191	0.472376	0.518805	0.056069	0.075181	0.365004	0.27973	0.182527	0.171969
Pde1c	0.072138	0.197851	0.270549	0.143468	0.103055	0.033355	0.205035	0.085853	0.171885
Dmc1	0	0.029968	0.120205	0.119518	0.085851	0.138936	0	0.200259	0.171829
Cldnd2	0.179994	0.089757	0	0.357971	0.257136	0.083226	0.372065	0.085686	0.17155
Gm35019	0	0.269272	0.900073	0.089493	0.085712	0.66581	0.372065	0.171372	0.17155
1700011L22Rik	0	0.359029	0	0	0	0.083226	0.093016	0	0.17155
Pde3a	0.10272	0.092202	0.087322	0.33197	0.122287	0.156737	0.037158	0.220049	0.171327
Il22ra2	0.157239	0.112015	0.044931	0	0.06418	0.14541	0.162515	0.085547	0.171272
Gm41043	0	0	0.044931	0	0	0	0	0	0.171272
Tex12	0.808659	0.448059	0.089861	0.089348	0	0	0	0	0.171272
Gm52135	0.233537	0.322498	0.143732	0.107182	0.034218	0.149516	0.334207	0.393388	0.171216
Hoxa10	0.025654	0.255856	0.153941	0.153061	0.048865	0.142344	0	0	0.171153
Ptpru	0.146791	0.244	0.130496	0.194625	0.093201	0.19608	0.168573	0.015529	0.170995
Gm7618	0	0	0.179432	0.535219	0.170869	0.165914	0	0.683268	0.170995
Prrt3	0.1046	0.238449	0.19428	0.104014	0.09962	0.373105	0.247107	0.142271	0.170903
Gm52727	0.071718	0.071527	0.179316	0	0.102455	0.099484	0.481807	0.477978	0.170884
Gm5298	0.143436	0.178818	0.215179	0.641847	0.409821	0.132645	0.037062	0.580402	0.170884
Gm5131	0	0.044697	0	0.222828	0.042683	0	0.185281	0	0.170857
Gm41501	0.143343	0.03574	0.03584	0.07127	0.170648	0.331399	0.037038	0	0.170774
Gm51544	0.051189	0.051053	0.153586	0	0.12188	0.189353	0.026453	0.073106	0.170758
Pp2d1	0.04478	0.200975	0.044786	0.04453	0.106621	0	0.16199	0.042635	0.170719
Zfp423	0.217454	0.153089	0.127929	0.470632	0.560391	0.165608	0.145426	0.255753	0.170679
Tmem236	0.025559	0.025491	0.281185	0.025416	0.219081	0.023636	0.237751	0.04867	0.170522
Lix1	0.089359	0.071297	0.303854	0.088858	0.017021	0.11569	0.166242	0.102094	0.170333
Gm38908	0.279676	0.054237	0.334097	0.162231	0.059191	0.064659	0.305118	0.125743	0.1703
Gm8225	0.119094	0.35633	0.833752	0.118426	0.34027	0.330402	0.307723	0	0.17026
Gvin-ps2	0.076501	0	0.063759	0.21554	0.170005	0.129701	0.105424	0.194232	0.17013
Igfbp2	0	0.59261	2.139338	0	0	0.054949	0.245651	0.339437	0.169895
Gm42151	0.089129	0.088891	0	1.063554	0	0.329694	0	0	0.169895

Mn1	0.072902	0.177731	0.121518	0.354414	0.162006	0.149817	0.075348	0.215942	0.169846
Magi1	0.427635	0.189554	0.237602	0.838666	0.49778	0.560239	0.356041	0.237504	0.169822
Cd1d2	0.044529	0	0.044534	0	0	0	0.046022	0	0.169759
Unc93a	0.029678	0.059198	0	0.029512	0	0.10978	0.184041	0.113025	0.169713
Gm16897	0	0.022196	0.022258	0.265568	0.339132	0.123486	0.023002	0.021189	0.169691
Rnf186	0.044457	0	0.088925	0.442081	0.042341	0.16445	0.367589	0.084655	0.169486
Kcnn1	0.710744	0.630091	0.454139	0.667499	0.282044	0.419925	0.938643	0.432335	0.169351
Gm7252	0	0.088607	0.26656	0.441727	0.253839	0.164319	0.091824	1.015047	0.169351
Gm30804	0	0	0	0	0	0.164319	0	0	0.169351
Gm4432	0.355372	0.177213	0.059236	0.176691	0.394861	0.328637	0.244863	0.225566	0.169351
Gm51955	0.166447	0.265607	0.022196	0.264824	0.285341	0.020523	0.194969	0.327513	0.169215
[truncated: 575,983 more chars]
